# A long-read RNA-seq approach to identify novel transcripts of very large genes

**DOI:** 10.1101/gr.259903.119

**Published:** 2020-06

**Authors:** Prech Uapinyoying, Jeremy Goecks, Susan M. Knoblach, Karuna Panchapakesan, Carsten G. Bonnemann, Terence A. Partridge, Jyoti K. Jaiswal, Eric P. Hoffman

**Affiliations:** 1 Center for Genetic Medicine Research, Children's Research Institute, Children's National Health System, Washington, D.C. 20010, USA;; 2 Department of Genomics and Precision Medicine, The George Washington University School of Medicine and Health Sciences, Washington, D.C. 20052, USA;; 3 Neuromuscular and Neurogenetic Disorders of Childhood Section, National Institute of Neurological Disorders and Stroke, National Institutes of Health, Bethesda, Maryland 20892, USA;; 4 Computational Biology Program, Oregon Health and Science University, Portland, Oregon 97239, USA;; 5 Department of Pharmaceutical Sciences, School of Pharmacy and Pharmaceutical Sciences, Binghamton University, Binghamton, New York 13902, USA

## Abstract

RNA-seq is widely used for studying gene expression, but commonly used sequencing platforms produce short reads that only span up to two exon junctions per read. This makes it difficult to accurately determine the composition and phasing of exons within transcripts. Although long-read sequencing improves this issue, it is not amenable to precise quantitation, which limits its utility for differential expression studies. We used long-read isoform sequencing combined with a novel analysis approach to compare alternative splicing of large, repetitive structural genes in muscles. Analysis of muscle structural genes that produce medium (*Nrap*: 5 kb), large (*Neb*: 22 kb), and very large (*Ttn*: 106 kb) transcripts in cardiac muscle, and fast and slow skeletal muscles identified unannotated exons for each of these ubiquitous muscle genes. This also identified differential exon usage and phasing for these genes between the different muscle types. By mapping the in-phase transcript structures to known annotations, we also identified and quantified previously unannotated transcripts. Results were confirmed by endpoint PCR and Sanger sequencing, which revealed muscle-type-specific differential expression of these novel transcripts. The improved transcript identification and quantification shown by our approach removes previous impediments to studies aimed at quantitative differential expression of ultralong transcripts.

Alternative splicing occurs in >90% of genes (Wang et al. 2008; Nilsen and Graveley 2010). Through this process, a single gene may create protein isoforms with multiple different functions and interactions (Yang et al. 2016). Skeletal muscle has one of the highest rates of tissue-specific alternative splicing (Castle et al. 2008), which made muscle an ideal model system for early mechanistic studies (Nadal-Ginard et al. 1991). Many muscle proteins involved in myofiber type specialization and sarcomere formation are large, and their transcripts contain numerous exons, repetitive unit structures, and extensive alternative splicing.

The largest protein encoded by the mammalian genome, titin (Ttn; 3.3–3.7 MDa), spans half the sarcomere in length, and is coded by transcripts up to ∼106 kilobases long, with 363 exons (Bang et al. 2001). Titin primarily functions as a molecular spring that helps maintain the sarcomere as muscle cells contract (Linke 2018). The elasticity of muscle cells depends on differential splicing of the titin transcript coding a highly repetitive PEVK region and repeating units of immunoglobulin (Ig) and fibronectin-type-3 domains (Linke 2018).

Nebulin (Neb) is a large (∼0.9 MDa) structural protein that binds to titin in the Z-disk and extends along the actin thin filament within the sarcomere (Wang and Wright 1988; Politou et al. 2002). It plays roles in assembling, stabilizing, and determining the length of the actin thin filaments (McElhinny et al. 2005) and helps define the width of the Z-disk (Witt et al. 2006). The human nebulin gene has 183 exons that produce transcripts (up to ∼22 kb) that code for multiple conserved 35 amino acid simple repeats, called nebulin domains (Labeit et al. 1991). In the central region of the protein are 22 super repeats composed of seven nebulin domains each, one for each turn of the actin molecule (Labeit and Kolmerer 1995). The super-repeat region is alternatively spliced during development and in different muscle tissues (Donner et al. 2006; Buck et al. 2010).

A paralog of nebulin is the nebulin-related anchoring protein (Nrap) (Luo et al. 1997). Nrap is the second largest (∼196 kDa) member of the nebulin family of proteins (Luo et al. 1997; Pappas et al. 2011). It is a scaffolding protein, with roles in myofibrillar assembly and organization during muscle development (Luo et al. 1997; Carroll et al. 2001, 2004; Dhume et al. 2006). Its structure is also highly repetitive, containing nebulin domains and super repeats (Luo et al. 1997). It is primarily expressed in intercalated disks of cardiac muscle and myotendinous junctions of skeletal muscle (Carroll and Horowits 2000; Herrera et al. 2000). Alternative splicing of the *Nrap* gene produces tissue-specific isoforms (*Nrap-c* and *Nrap-s* for cardiac and skeletal muscle, respectively, up to ∼5.5 kb) (Mohiddin et al. 2003; Lu et al. 2008).

Recently, Savarese et al. (2018) reported the most comprehensive study on alternative splicing of titin by RNA sequencing (RNA-seq) of 12 human muscle types from 11 non-neuromuscular disease patients. Short-read RNA-seq has been indispensable for studying alternative splicing in whole transcriptomes (Trapnell et al. 2010; Li et al. 2015), but limited read lengths make it challenging to determine the full-length and complete splicing patterns of transcripts (Supplemental Fig. S1). Further, mapping short-read sequences to highly repetitive regions in the reference genome can be computationally difficult and create ambiguities (Treangen and Salzberg 2011), and reconstructing whole transcripts from short-read data is prone to false positives and artifacts (Steijger et al. 2013). Also, accurate predictions of the complete transcript of a large gene with a vast number of potential exon combinations are nearly impossible: The 363 coding exons in titin could theoretically produce more than a million alternative splice isoforms (Guo et al. 2010).

Long-read sequencing technologies such as Pacific Biosciences (PacBio) isoform sequencing (Iso-Seq) are able to sequence full-length transcripts up to 10 kb in length (Wang et al. 2016). Although single pass reads (subread) have ∼15% error rate, circular consensus sequencing (CCS, or HiFi) provide PacBio with a ∼2% error rate compared to Oxford Nanopore at ∼14% (Weirather et al. 2017). HiFi long reads combined with the Iso-Seq bioinformatics pipeline have been systematically shown to have ≤0.12% error rate and identify mRNA isoforms missed by short-read sequencing (Gordon et al. 2015). However, the entire length of longer transcripts such as titin and nebulin still cannot be captured within maximum Iso-Seq read lengths. In addition, Iso-Seq is a transcript discovery tool because the bioinformatics pipeline does not support transcript quantification.

Here, we have addressed deficits of the PacBio long-read Iso-Seq technology and used it to compare alternative splicing of transcripts between different murine muscle types: fast skeletal, slow skeletal, and heart. To our knowledge, this is the first study to apply an exon phasing approach that results in accurate quantification of very long and difficult transcripts.

## Results

### Comparison of long-read versus short-read RNA-seq on repetitive DNA sequences

**Figure 1.**
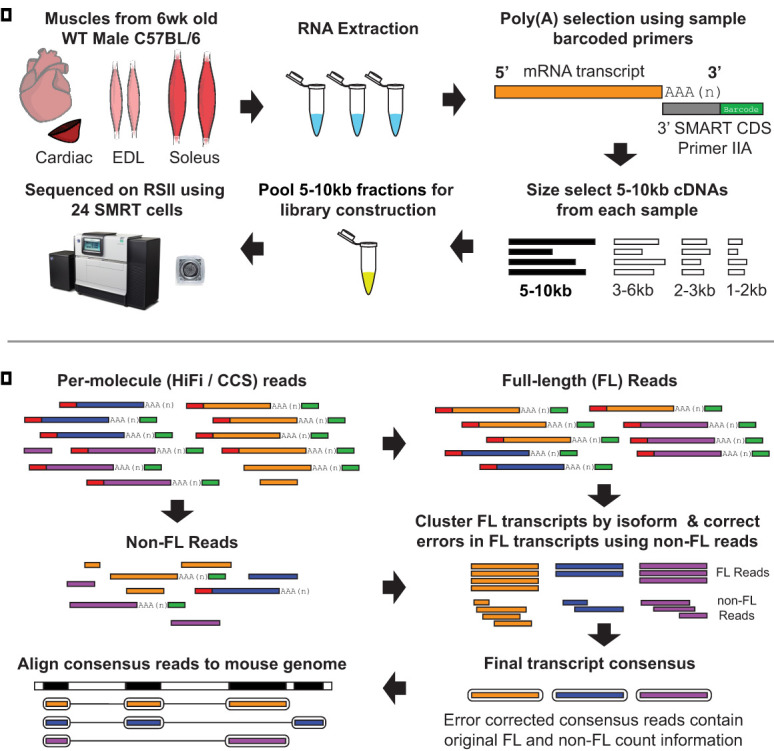
Experimental design. (*A*) Total RNA from cardiac apex, extensor digitorum longus (EDL), and soleus muscles were extracted, and cDNA was generated using barcoded oligo(dT) primers. Next, 5–10 kb cDNAs from all muscles were size selected, pooled for library construction, and sequenced on the PacBio RSII. (*B*) PacBio's Iso-Seq bioinformatics pipeline. Raw reads are converted into circular consensus (CCS/HiFi) reads. HiFi reads are separated into full-length (FL) and non-FL reads. FL reads must have 5′ and 3′ primers and a poly(A) tail. FL reads are grouped by similarity (isoform), polished using non-FL reads to generate high quality transcript consensus reads, and aligned to the mouse genome. (Reprinted, with permission, from Pacific Biosciences.)

The mRNAs were isolated from three mouse muscle tissue types: soleus, extensor digitorum longus (EDL), and heart (Fig. 1A). The 5–10 kb mRNAs were subjected to long-read sequencing using PacBio methods (Supplemental Table S1). We aligned the long reads to the mouse GENCODE reference, the most comprehensive annotation database (Frankish et al. 2019). Although single-molecule reads are prone to random errors, (∼15%), PacBio's HiFi reads and Iso-Seq method produce consensus reads with ≤0.12% error rate (Fig. 1B; Gordon et al. 2015). For our purposes of exon and splicing patterns, this was sufficient error reduction. Consensus reads retain the number of full-length (FL) reads used to generate them, a key component of our downstream analysis.

For comparison of long-read versus short-read sequences, we accessed published short-read RNA-seq data of WT EDL and soleus muscles (Singh et al. 2018). We compared mapping of *Myh2*, *Myh1*, and *Myh4* on Chromosome 11 (Supplemental Fig. S2) using EDL samples. The short-read data had substantial reads that were mismapped and split across two genes. In contrast, almost all long reads aligned to the correct myosin heavy chain gene.

A similar comparative analysis of short-read versus long-read sequencing was carried out for nebulin (Supplemental Fig. S3). The short reads provided many more novel splice junctions compared to long reads, but are likely false positives caused by mismapping and poor specificity (Gordon et al. 2015). These results suggest that long reads perform better than short reads at resolving intergenic and intragenic regions of the genome.

### Oligo(dT) internal priming provides coverage across large transcripts (>10 kb)

The aligned data were surveyed in the Integrative Genomics Viewer (IGV) for read coverage (Thorvaldsdóttir et al. 2013). We observed that genes with transcripts close to the 6-kb average read length were often fully covered by a single long read (e.g., *Nrap*, Myomesins, Myosins). For very large genes such as titin (106 kb transcript) and nebulin (22 kb transcript), the middle and 5′ end of the gene were also covered (Supplemental Fig. S4A). This was unexpected given that Iso-Seq uses a poly(A) capture method.

On close inspection, reads that aligned upstream of the poly(A) region (last 3′ exon) of the gene consistently started on specific exons in all samples. Focusing in on these exons (e.g., exon 137 of nebulin), the 3′ ends of these reads aligned within 20 bases upstream of multiple stretches of “A” bases in the genomic sequence (Supplemental Fig. S4B). These reads were likely generated from cDNAs synthesized with internally primed oligo(dT) primers (Nam et al. 2002). These observations suggest internal priming in long-read sequencing may be beneficial for covering very large genes if they contain internal priming sites. In contrast, *Obscn* (27-kb transcript) showed no internal priming sites, leading to loss of 5′ coverage (Supplemental Fig. S5).

### Observing splice transcripts across samples

**Figure 2.**
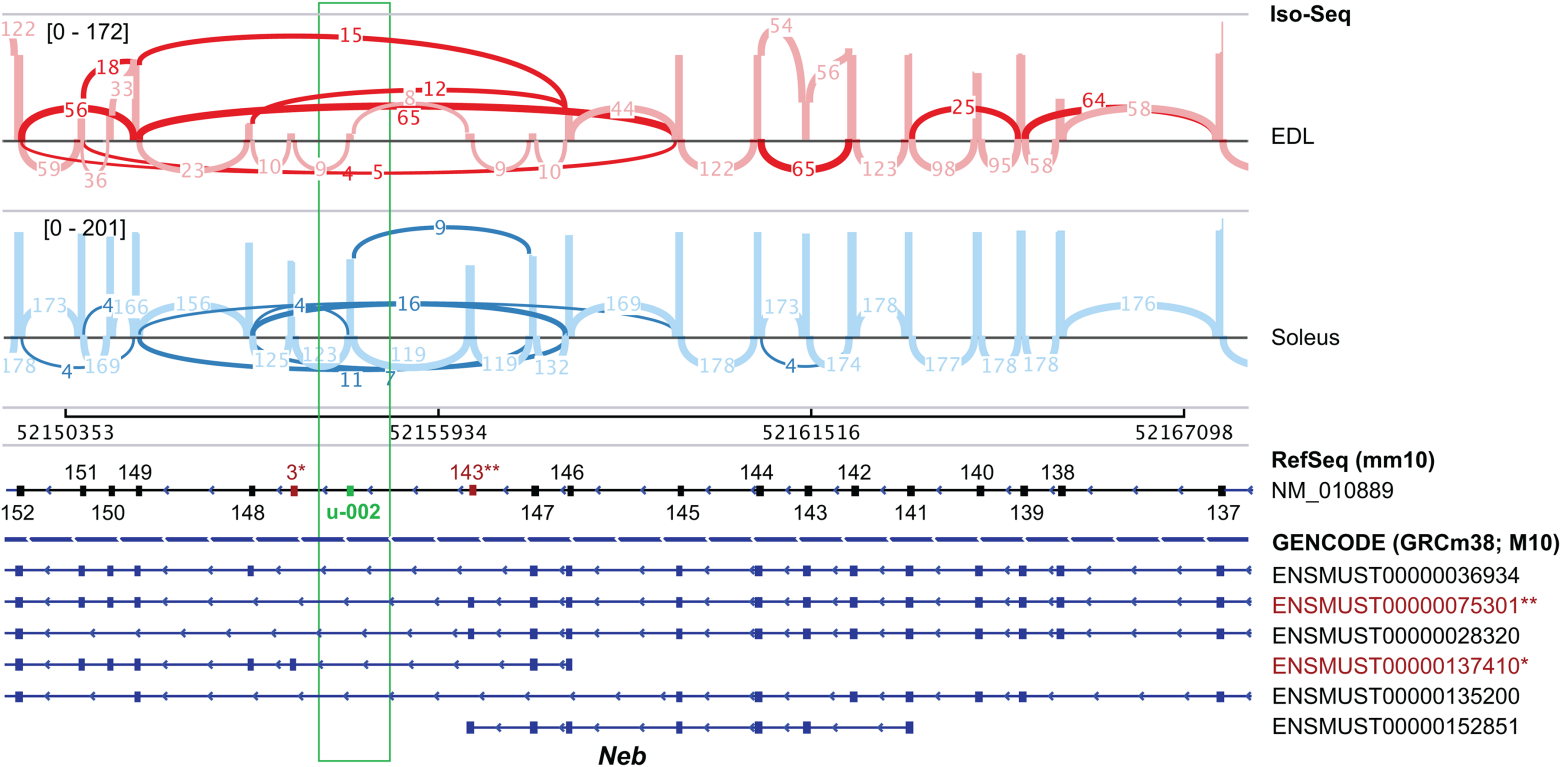
Sashimi plot showing extensive alternative splicing of EDL and soleus transcripts between exons 152 and 137 of nebulin. Splice junctions that skip known exons are highlighted. Three exons between exons 147 and 148 are not annotated in RefSeq. Two of these exons (red) are in GENCODE. Exon u-002 (green) is not annotated in either database. The plot shows consensus reads (not full-length reads). Minimum splice junction coverage set to 5 for visual clarity.

To survey splice differences between tissues, the 3′ region of nebulin was studied. Our long-read data confirm reports that splicing between exons 137 and 152 are highly variable (Fig. 2; Supplemental Fig. S3; Donner et al. 2004). In addition, three novel exons were detected between 147 and 148 (NM_010889.1) that were not in RefSeq but show a high degree of inter-species conservation. Two of these exons were annotated in the GENCODE mouse database as part of two short nebulin transcripts. The third exon was unannotated (u-002). Four other exons between 85 and 86 of NM_010889.1 (exons 2–5 of ENSMUST00000028320.13) were expressed in all murine soleus and EDL transcripts and conserved in human (Supplemental Fig. S6).

### Identifying unannotated exons and differential exon usage

To identify unannotated exons and differential exon usage between tissues, we first selected for genes with at least 10 cluster (consensus) reads produced from the Iso-Seq method (Table 1). This threshold resulted in 686 of 48,440 genes that were adequately covered for further analysis. To reduce the complexity of the splice analysis, we collapsed the GENCODE annotation file using a script included in the *DEXSeq* R package, “dexseq_prepare_annotation.py.” This produced a collapsed annotation file containing one metatranscript per gene (Supplemental Fig. S7A). Different components of the transcript were renamed as unique exonic parts. See *DEXSeq* manual for details (https://bioconductor.riken.jp/packages/3.0/bioc/html/DEXSeq.html).

**Table 1.**
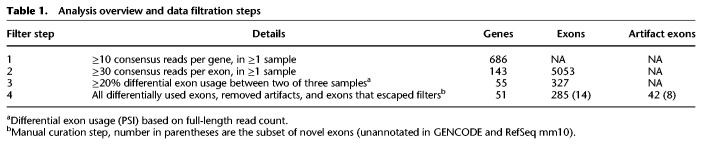
Analysis overview and data filtration steps

Next, we developed the exCOVator pipeline to perform a preliminary exon-based analysis (exon usage independent from their neighbors) to identify unannotated exons and differential exon usage for the 686 genes. To find unannotated exons, exCOVator screens for exons in the sample reads that are missing from the collapsed annotation file and combines them with existing annotations to form a new annotation file (Supplemental Fig. S7B). This resulted in 1218 additional unannotated exons/exonic parts.

Using this new collapsed annotation file, the percent spliced-in (PSI) for each exonic part was calculated by exCOVator for given samples. Briefly, all consensus reads that overlapped an exonic part (total reads) and the subset that contained the matching exonic part were counted. For each exonic part, the FL read counts were extracted from the total and matching consensus reads. The PSI calculation was performed by dividing the exonic part's matching FL reads by the total FL reads (Supplemental Fig. S7C). PSI normalizes the read coverage data and allows differential usage analysis of the same exonic part across multiple samples (Supplemental Methods; Supplemental Fig. S7D).

Additional filter criteria were used to reduce artifacts. We selected for exonic parts with ≥30× consensus read coverage and a 20% difference in PSI (based on FL reads) between two of three samples (Table 1). This yielded 327 exons across 55 genes. On manual inspection in IGV, we found 285 (87%) exonic parts across 51 genes were differentially spliced, and 42 (13%) were artifacts and/or exons that escaped filters.

For the remainder of the study, we focused on three large muscle structural genes: (1) *Nrap*, that produces ∼6-kb transcripts which fall within average read lengths; (2) *Neb*, that produces ∼22-kb transcripts that are more than twice as long as the maximum read length; and (3) *Ttn*, that produces 106-kb transcripts that are more than 10 times the read length.

### Novel muscle-type-specific splicing patterns in nebulin-related anchoring protein

**Figure 3.**
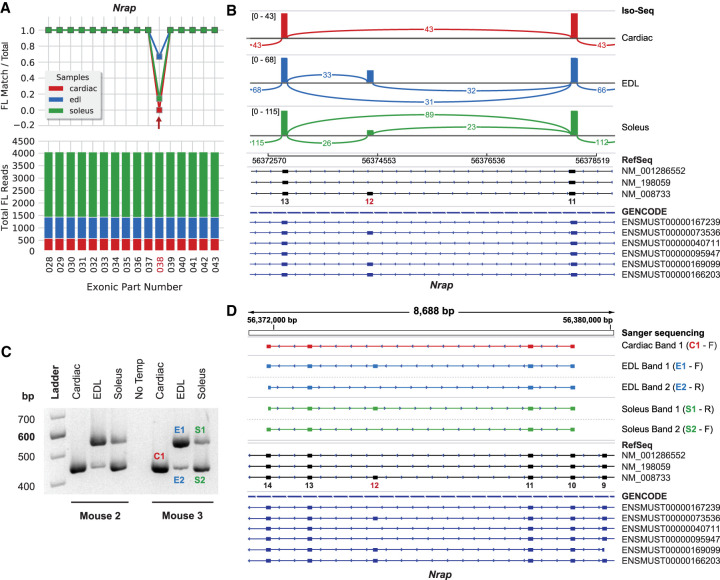
Differential usage of *Nrap* exon 12 (exonic part 038). (*A*) *Nrap* exon coverage graph (cropped) produced by exCOVator. (*Bottom*) Stacked bar graph showing total full-length (FL) read coverage of all exonic parts (EP) for the gene. (*Top*) Line graph displaying the ratio of [FL reads matching the EP/total FL reads] that overlap the EP coordinates. (*B*) Sashimi plot from the Integrative Genomics Viewer (IGV) displaying differential splicing of exon 12. The plot displays consensus reads from the BAM file (not FL reads). Minimum splice junction coverage = 5. (*C*) Agarose gel showing RT-PCR products pertaining to exon 12 from soleus, EDL, and heart. Primers target exons 9–14 (549 bp includes and 444 bp excludes exon 12). (*D*) Sanger sequencing of products excised from the gel in *C*. The *top* half shows sequences aligned using the IGV BLAT tool. Cardiac band 1 and EDL and soleus band 2 are missing exon 12.

Differential usage of exon 12 of *Nrap* was observed between all three muscle tissues. Lu et al. (2008) described the expression and alternate splicing of *Nrap* in skeletal and cardiac muscle during development and identified two splice variants: (1) *Nrap-s* that includes exon 12 (NM_008733.4) and is exclusively expressed in skeletal muscle; and (2) *Nrap-c* that is missing exon 12 and is exclusively expressed in cardiac muscle. The present analysis confirms their findings that only *Nrap-c* is expressed in cardiac tissue (0/567 reads) but is not a heart exclusive isoform. *Nrap-c* is also expressed in skeletal muscle, suggesting that *Nrap-s* and *Nrap-c* are misnomers. Additionally, *Nrap-s* or exon 12 is used 53% more among transcripts in the predominantly fast-twitch EDL (576/862 reads; 67% PSI) compared to the slow-twitch soleus muscle (377/2621 reads; 14% PSI; shown as exonic part 38) (Fig. 3A,B). This shows that alternative splicing of *Nrap* transcripts also occur between fast and slow skeletal muscle types.

To validate the findings, we performed RT-PCR and Sanger sequencing using the same primers in the publication (Supplemental Table S2; Lu et al. 2008) and total RNA extracted from muscles of two independent mice. Our RT-PCR results confirm the Iso-Seq analysis (Fig. 3C), showing (1) low usage of exon 12 in soleus transcripts (444 bp > 549 bp product); (2) high usage of exon 12 in EDL transcripts (549 bp > 444 bp product); and (3) no usage of exon 12 in cardiac transcripts (single 444 bp product). Sanger sequencing of the excised bands further confirmed the splice products (Fig. 3D). By corroborating and extending the findings of the prior study, the present results establish that PacBio long-read Iso-Seq can be used as a semiquantitative approach for differential exon usage between samples.

### Nebulin exon 138 is differentially used between slow and fast skeletal muscle

**Figure 4.**
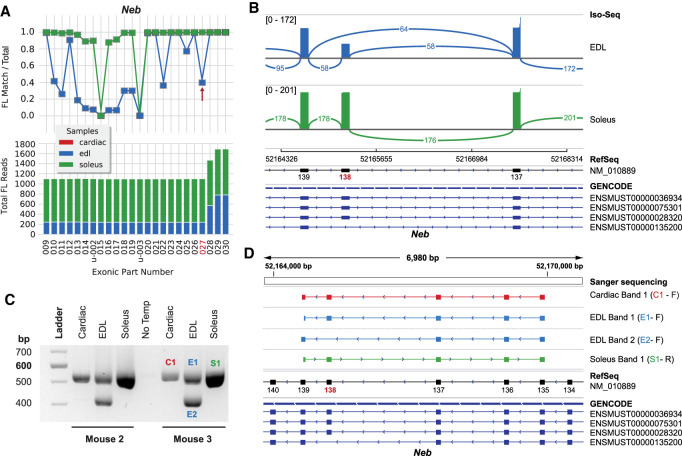
Differential usage of exon 138 of mouse nebulin. (*A*) Nebulin exon coverage graph (cropped) produced by exCOVator. (*Bottom*) Stacked bar graph displays full-length (FL) read coverage of exonic parts (EP). (*Top*) Line graph displays the ratio of [FL reads matching the EP/total FL reads] that overlap the EP coordinates. (*B*) Sashimi plot from the Integrative Genomics Viewer (IGV) displaying differential splicing of exon 138. The plot displays consensus reads from the BAM file (not FL reads). Minimum splice junction coverage = 5. (*C*) Agarose gel showing RT-PCR products pertaining to exon 138 from soleus, EDL, and heart. Primers target exon 135 and 139. Cardiac shows similar banding pattern as soleus; however, few reads were detected during sequencing. (*D*) Sanger sequencing of products cut from the gel seen in *C*. The *top* portion shows sequences aligned in IGV using the BLAT tool. Only the EDL band 2 is missing exon 138.

The initial survey and the exCOVator pipeline found multiple differentially used exons between 137 and 152 of nebulin (NM_010889.1) (Figs. 2, 4A,B). We used exon 138 to validate this region and found a single RT-PCR product at ∼500 bp in soleus muscles, suggesting that exon 138 is constitutively expressed with all exons between 135 and 139 in the majority of transcripts in the soleus (Fig. 4C). The EDL muscles had two products: a ∼500 bp and a smaller ∼400 bp product missing exon 138. These results suggest differential exon usage of 138 in skeletal muscles, which is excluded from a subset of EDL transcripts, but not in the soleus.

Cardiac muscle has low expression of nebulin (Bang and Chen 2015). However, we identified a prominent single ∼500-bp product similar to the soleus (Fig. 4C). This product could be the result of the higher sensitivity of RT-PCR compared to Iso-Seq; we may have lost some sensitivity by selecting for cDNAs >5 kb. Sanger sequencing of the amplicon products show that the cardiac product is identical to what is found in the EDL and soleus (Fig. 4D), which rules out possible mispriming to nebulette, a related gene highly expressed in heart.

### Titin, cardiac-specific unannotated cassette exon 191

**Figure 5.**
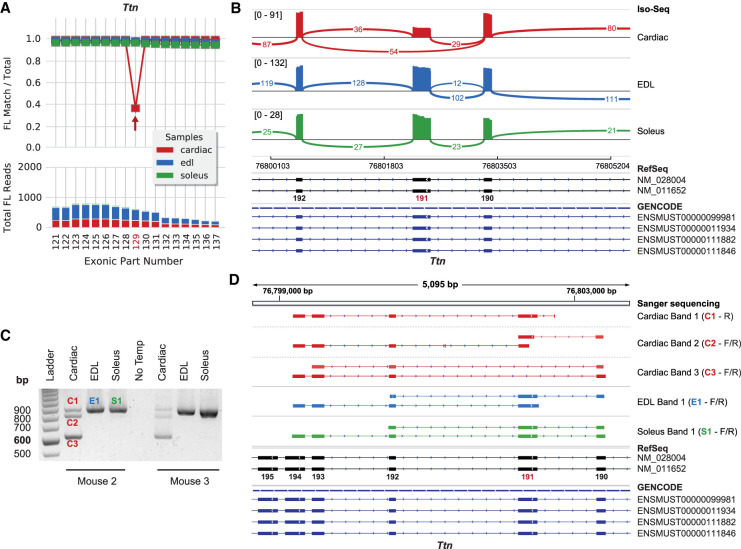
Titin exon 191, an undocumented cassette exon removed from a subset of cardiac transcripts. (*A*) Titin exon coverage graph (cropped) produced by exCOVator. (*Top*) Line graph; the red arrow points to exonic part (EP) 129 or exon 191 of NM_011652 (N2-A). (*Bottom*) Stacked bar graph displays full-length (FL) read coverage of EP. (*B*) Sashimi plot showing the same data in the Integrative Genomics Viewer (IGV). The plot shows consensus reads from the BAM file (not FL reads). Minimum splice junction coverage = 5. (*C*) Agarose gel of RT-PCR products from soleus, EDL, and heart. Primers target exons 189 and 194 with predicted product sizes of 882 bp (+exon 191, C1) and 615 bp (no exon 191, C3). All soleus and EDL transcripts include exon 191. Cardiac has both predicted bands and one unknown band in the middle (C2) that is likely a heteroduplex. (*D*) Sanger sequencing data of products from *C* displayed using the IGV BLAT tool. Exon 191 is missing only from cardiac band 3 (C3) and is present in all other tissues.

The Iso-Seq analysis results show that titin exon 191 (NM_011652.3) is almost always used in transcripts of soleus (34/35 FL reads; 97% PSI) and EDL (357/361 FL reads; 99% PSI) muscles, but is missing from 64% of the transcripts in cardiac muscle (84/232 FL reads; 36% PSI) (Fig. 5A,B). This is corroborated by RT-PCR data showing exon 191 is retained in all soleus and EDL transcripts by the presence of a single 852-bp product and by the cardiac samples having multiple RT-PCR products (Fig. 5C). Exon 191 is constitutively expressed according to GENCODE and RefSeq. Therefore, the present data suggest titin exon 191 may be an unannotated cassette exon removed from a subset of cardiac transcripts. The cardiac 615-bp product is predicted to only be missing exon 191, and the 882-bp product contains all exons between 190 and 194. The ∼780-bp product in the middle is unknown, and Sanger sequencing only resulted in truncated sequences (cardiac band 2) (Fig. 5D). The product is likely a heteroduplex between the 615- and 882-bp splice products. Overall, these data confirm that titin exon 191 is an unannotated cassette exon that is only removed from a subset of mouse cardiac transcripts.

### Annotation and quantification of transcript structures

One of the strengths of long-read sequencing is the ability to phase multiple neighboring exons to determine transcript splice patterns within the same read. However, the exon-based approach used here to identify and quantify differentially used exons does not account for phasing. To address this, we developed the exPhaser pipeline to quantify and annotate splicing patterns of larger transcript structures. The pipeline takes in mapped Iso-Seq BAM files and a BED file of exon coordinates to determine the splicing pattern of the given exons within each consensus read. Then it outputs a table of extracted FL read counts for each splicing pattern and all known transcript annotations that match the pattern. Similar to our normalization approach for calculating PSI, percentages of each transcript isoform expressed in the sample is calculated by dividing the FL read counts for the exact matching splicing pattern by the total FL reads of all other patterns overlapping the input exons. As exPhaser input, cassette exons were selected that defined the isoform according to known annotations and exCOVator output (e.g., novel and differentially used exons).

### Phasing of full-length *Nrap* transcripts

**Figure 6.**
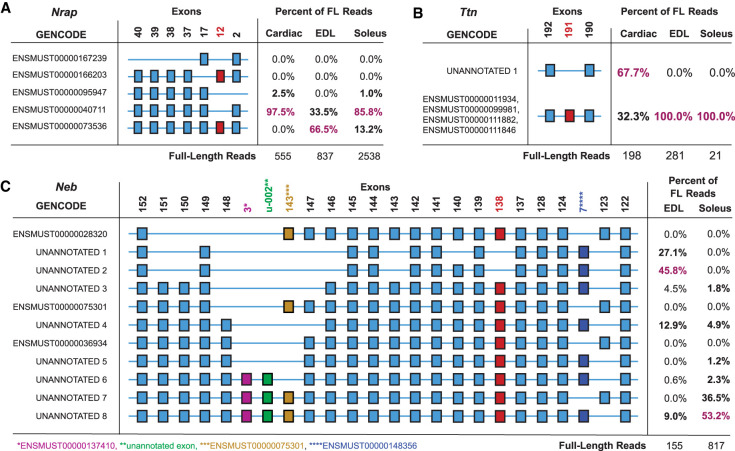
Identifying and quantifying *Nrap*, titin, and nebulin transcript isoforms using exPhaser. Exons highlighted as red squares were validated using RT-PCR and Sanger sequencing. (*A*) *Nrap* transcript structures. Exon numbering based on NM_008733. (*B*) Titin transcript structures. Exon numbering based on NM_011652. The *bottom* splice pattern could not be uniquely mapped to a single annotation. (*C*) Nebulin transcript structures. Exon numbering based on NM_010889. Some neighboring constitutively expressed exons were also included for clarity.

Most *Nrap* reads span whole transcripts; therefore, we phased all known cassette exons: 2, 12, 17, and 37–40 (NM_008733) (Fig. 6A). We found nearly all (97.5%) *Nrap* transcripts expressed in cardiac tissue excludes exon 12 (ENSMUST00000040711; 541/555 FL reads). The soleus also primarily (85.6%) expressed the exon 12 excluding transcript, but to a lesser extent (ENSMUST00000040711; 2177/2544 FL reads). In contrast, the primary transcript (66.5%) expressed in EDL muscle includes all exons (ENSMUST00000073536; 557/837 FL reads). These data closely reflect the results of the exCOVator analysis (Fig. 3). However, we were able to identify a rare transcript that excludes both exon 2 and 12 (ENSMUST00000095947) exclusively expressed in cardiac (2.5%; 14/555 FL reads) and soleus muscles (1.0%; 26/2544 FL reads).

### Phasing nebulin exons in the Z-disk and 3′ end of the super repeat region

The exCOVator analysis suggested that differential splicing of nebulin in EDL and soleus only occurred in the 3′ half of the transcript. This includes 14 exons between 122 and 152 of NM_010889 that code for nebulin peptides found within the Z-disk and 3′ end of the super repeat region (Figs. 2, 6C). This includes two mutually exclusive exons between 122 and 124, and 12 cassette exons between 137 and 152. After phasing the 14 differentially spliced exons and additional flanking exons, we did not find any of the transcript structures in the RefSeq or GENCODE databases. This was because most reads contained exons from other annotated transcripts or the unannotated exon. Corroborating our exon-based analysis, the transcripts expressed in the EDL have more exons spliced out between exons 137 and 152 (Fig. 4A). This includes differential splicing of exons 138, 140, 143, 146, 147, 143*** (of ENSMUST00000075301), u-002, 3* (of ENSMUST00000137410), 148, 150, and 151. Only 9% (14/155 FL reads) of EDL reads retain all exons between 137 and 152. In contrast, the soleus retained all of the listed exons in 89.6% (733/817 FL reads) of transcripts. It is known that the width of the sarcomeric Z-disk in fast-twitch muscle (e.g., EDL) is narrower compared to slow-twitch muscle (e.g., soleus). Alternative splicing of nebulin transcripts in the EDL and soleus correlate with these Z-disk width observations.

In addition, we analyzed the splicing pattern of two known mutually exclusive nebulin exons 127^♦^ and 128^♦^ (Donner et al. 2006). Translated to our exon numbering using NM_010889, exon 127^♦^ = 123 and exon 128^♦^ is not annotated in RefSeq, but instead annotated as exon 7**** of ENSMUST00000148356 in GENCODE. Our phasing results show that 100% of transcript structures in the EDL include exon 7**** (155/155 FL reads) (Fig. 6C). On the other hand, 63% (514/817 FL reads) of soleus transcripts contain exon 7**** and only 37% (303/817 FL reads) contain exon 123, which agrees with previous studies (Donner et al. 2006).

Based on RefSeq annotation NM_001271208, alternative splicing of human nebulin occurs in exons 63–66 (66–69 of NM_010889 in mouse), 82–105 (83–87), 143–144 (124–7**** of ENSMUST00000148356), and 169–173 (four exons between 148 and 149 and 149 itself) (Donner et al. 2004, 2006; Lam et al. 2018). We found alternative splicing of all of these exons to be conserved in mouse except for exons 63–66 and 82–105 (Supplemental Data; Supplemental Fig. S6) that are constitutively expressed.

### Phasing multiple transcript structures of titin

**Figure 7.**
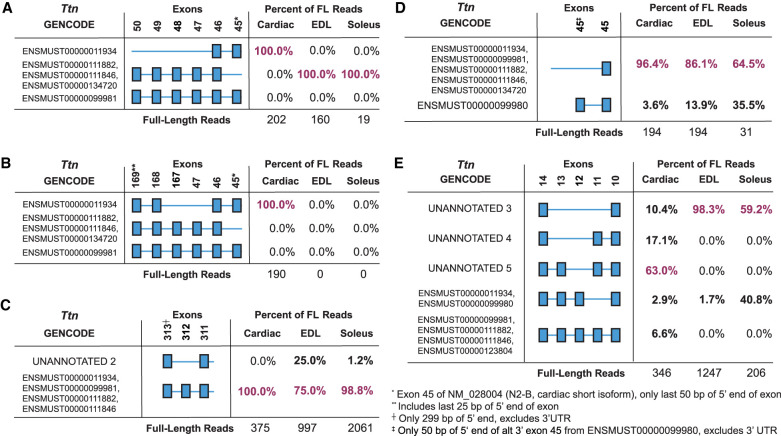
Phasing and quantifying additional titin transcript structures using exPhaser. Exons are numbered by NM_011652 (N2-A) unless noted. (*A*) Focused phasing of skeletal muscle N2-A isoform defining exons (47, 48, 49, 50) and one cardiac N2-B exon (45*). (*B*) Focused phasing of cardiac N2-B isoform defining exons (45, 46, 168, 169) and two N2-A defining exons (47 and 167). (*C*) Phasing of exon 312, a cassette exon only spliced out of skeletal muscle transcripts. (*D*) Phasing of exon 45^‡^ of ENSMUST00000099980, an alternate 3′ terminal exon. (*E*) Phasing of exons 11–13. The most abundant isoforms are unannotated. Transcript structures listed with multiple accession numbers cannot be uniquely assigned using the exons provided.

For titin, we could not phase all differentially spliced exons at once because they are too far apart to be contained within the length of a single read. To illustrate this, we compared the expression of titin N2-A (NM_011652, principle skeletal isoform) versus N2-B (*NM_028004, principle cardiac isoform) transcripts between the three muscles. All subsequent *Ttn* exon numbering is based on NM_011652 (N2-A) by default, unless noted. The N2-B transcript excludes exons 47–167 of N2-A but includes an alternate exon 45*. Initially, we sought to phase transcripts using exon 45* (N2-B specific), 46, 47 (N2-A specific), and 168. However, the distance between exon 45* and 168 in N2-A transcripts is much greater than the 10-kb maximum read length. Therefore, we phased the skeletal and cardiac-specific exons using two groups of exons (Fig. 7A,B). In the skeletal muscle isoform analysis, we phased exons 45*, 46, 47, 48, 49, and 50. Results confirmed that N2-A is the primary isoform expressed in skeletal muscle (soleus: 19/19 FL reads; and EDL: 160/160 FL reads) and that cardiac muscle did not express exons 47–50 (202/202 FL reads) (Fig. 7A). In the parallel cardiac isoform analysis, we phased exons 45*, 46, 47, 167, 168, and 169. Those data showed all transcripts expressed in cardiac muscle were missing exons 47 and 167, but included exons 45*, 46, 168, and 169 aligning with titin isoform N2-B (cardiac: 190/190; EDL: 0/0; and soleus: 0/0 FL reads).

Next, we phased four other sets of differentially used exons in titin including exon 191, exon 312, exon 45^‡^ of ENSMUST00000099980 and exons 11–13. First, we phased exon 191 and found cardiac data similar to the RT-PCR results with 32.3% (64/198 FL reads) of transcripts having exon 191 spliced in (Fig. 5, 6B). In both skeletal muscles, exon 191 is present in all transcripts (EDL: 281/281; soleus: 21/21 FL reads), corroborating that it is a cassette exon that is only removed from cardiac muscle transcripts. Further, mouse exon 191 is in-frame and conserved in human as exon 191/312 (NM_133378) or exon 243 based on the Locus Reference Genomic (LRG) coordinates used for clinical reporting.

To determine if alternative splicing of exon 191 is also conserved in humans, we consulted the CardioDB website for titin expression in human heart, and the comprehensive short-read RNA-seq study by Savarese et al. on 11 different human skeletal muscle types (Roberts et al. 2015; Savarese et al. 2018). In heart, 59% of exon 191 is spliced in in left ventricle tissue of dilated cardiomyopathy (DCM) patients (84 end-stage patients) and 69% in the GTEx data set (left ventricle tissue, 105 samples). The two exons flanking 191 are almost constitutively expressed with a spliced-in rate of 96%–100% in DCM patients and GTEx. Savarese et al. (2018) show that exon 191 (LRG exon 243) is constitutively expressed across 11 adult skeletal muscle types. These data corroborate our findings that exon 191 is a cassette exon that is only spliced out from a subset of cardiac and not from skeletal muscle transcripts. However, it also suggests there are slight differences in splicing of exon 191 in mouse compared to human. These discrepancies could result from differences in tissue sampling locations: left ventricle of human heart and apex of mouse heart that includes the lower portion of both ventricles.

Second, we phased the penultimate exon of titin, 312, along with adjacent neighbors. The results show that exon 312 is 100% included in mouse cardiac transcripts (375/375 FL reads) (Fig. 7C). In contrast, Kolmerer et al. (1996) found transcripts in heart that exclude exon 312 also known as M-line exon 5 (Mex5) in mouse, rabbit, and human. However, they showed that a majority of heart transcripts included Mex5. The Iso-Seq data may have missed Mex5 negative transcripts because of differences in heart regions sampled (i.e., whole vs. apex). In our results, EDL and soleus muscles splice out exon 312 in 25% (249/997 FL reads) and 1.2% (25/2061 FL reads) of transcripts, respectively. In contrast, Kolmerer et al. (1996) showed that most transcripts had exon 312 spliced out (Mex5 negative) in rabbit fast-twitch muscles, but this discrepancy could result from species differences.

Similar to the Iso-Seq results, CardioDB and GTEx report that nearly all (99%) titin transcripts expressed in human left ventricle have exon 312 (363 LRG) spliced in. Across different human skeletal muscles, data from Savarese et al. (2018) show that exon 312 is mostly (91%), but not exclusively, spliced in. These data support that exon 312 is conserved and is a cassette exon that is mostly removed from fast-twitch skeletal muscle transcripts compared to heart.

Third, we phased the alternative 3′ exon 45^‡^ of ENSMUST00000099980 (Fig. 7D). After manually inspecting the reads in IGV, we noted numerous partial reads of the large exon 45^‡^. Therefore, we phased the exon (only 50 bp of 5′ end) along with its upstream neighbor, exon 45 of NM_011652. The phasing results show that exon 45^‡^ is expressed in 3.6% (7/27 FL reads) of all cardiac muscle transcripts, 13.9% (27/194 FL reads) of EDL transcripts, and 35.5% (97/118 FL reads) of soleus transcripts. Our data suggest that ENSMUST00000099980 is a minor isoform but is expressed higher in skeletal compared to cardiac muscle.

In humans, the paralog of ENSMUST00000099980 in mouse is Novex-3 (NM_133379), the minor small cardiac isoform of titin. The alternative 3′ exon is conserved in humans as exon 46 of NM_133379 (48 LRG) and has a 22% sequence difference compared to exon 45^‡^ in the mouse paralog. According to CardioDB, exon 46 is expressed in 9.4% and 7.3% of human left ventricle transcripts in the DCM and GTEx, respectively. The GTEx data were calculated by taking the ratio of the alternative 3′ exon versus the primary end exon (364 LRG). Their result is close to our 18% expression of exon 45^‡^ found in mouse cardiac tissue. On the other hand, Savarese et al. (2018) described exon 46 as only 2% spliced in in adult skeletal muscles compared to our 13.9% and 35.5% in mouse EDL and soleus transcripts, respectively. However, this discrepancy may result from differences in species, sampling location, and unreliability of PSI calculations on 3′ exons using short-read data.

Last, we phased exons 11–13 of titin (and neighbors 10 and 14) and observed that the highest expressed transcripts of each muscle are unannotated (Fig. 7E). Our results show that there are substantial differences in how these exons are used in each muscle. Exon 11 is not used in any titin transcripts expressed in the two skeletal muscles and is likely that the N2-A annotation is incorrect. In contrast, cardiac muscle makes extensive use of exon 11, which appears in 86.7% of expressed transcripts. Exons 12 and 13 seem to be coexpressed within skeletal muscle, but only in less than half of soleus (40.8%) and very rarely in EDL (1.7%) transcripts. Exon 12 is rarely used in heart (9.5%), but exon 13 is included in more than half (69.6%) of expressed transcripts. This suggests that peptides coded by all three exons are dispensable for titin's function in fast skeletal muscle because 98.3% of transcripts in EDL do not express them. These data also suggest similarities between slow soleus and cardiac muscle based on shared expression of exon 13. However, soleus includes exon 12 in more transcripts than cardiac muscle with the inverse pattern seen for exon 11.

Exons 11–13 are conserved in human and retain the same numbering. Savarese et al. (2018) show that exon 11 is constitutively spliced out in human, which agrees with our mouse data. They also show exon 12 to have an average of 54% inclusion and exon 13 with a 79% inclusion in skeletal muscle transcripts. Our soleus muscle data agree with theirs, showing a similar exon inclusion rate of 40.8%. However, in the EDL muscle, exon 12 is only found in 1.7% of transcripts, which is a large discrepancy. Our mouse exon 13 results also conflict with their human data. We found exon 13 to be coexpressed in transcripts with exon 12, sharing the same inclusion rate of 1.7% in EDL, which is vastly lower than their average of 79% inclusion in skeletal muscle. These discrepancies are likely because Savarese et al. (2018) averaged their data across multiple skeletal muscles.

For the heart, exon 11 is expressed in left ventricle tissue at 59% in DCM and 69% in GTEx according to CardioDB. This agrees with our analysis that most (86.7%) cardiac transcripts include exon 11, the highest inclusion rate of our three muscles. The database reports that exon 12 is included in 79% of left ventricle transcripts in the DCM study and 75% in GTEx. This is much higher than our study in which the exon is included in only 9.5% of mouse cardiac transcripts. Exon 13 is expressed in 96% of DCM and 95% of GTEx transcripts, but only 72.5% in mouse cardiac transcripts. We see some agreement between species such as exclusive expression of exon 11 in heart, but exons 12 and 13 seem to be included at higher rates in humans. Again, this could result from differences in species and/or sampling location.

## Discussion

The approach presented here differs from previous usage of Iso-Seq by extending its ability to identify novel exons and isoforms of ultralong transcripts and compare their usage between samples. The exon-based (exCOVator) approach enables differential exon usage (PSI) analysis and identification of novel exons. We illustrated this by finding 285 differentially used exons: 14 novel across 51 genes between the EDL, soleus, and heart muscle with a fairly low number of artifacts. We also showed how our approach can work on very large and repetitive genes by focusing on three that are important to muscle: titin, nebulin, and *Nrap*, without resorting to additional short-read data. This method reduces cost and is useful for transcripts with highly complex and repetitive sequences, which are difficult to resolve using short-read sequencing and other molecular assays. Combined with our exon phasing (exPhaser) approach, were able to determine splicing patterns between cassette exons to generate transcript structures. The exon pattern of these structures was mapped to known annotations for identification and used to quantify their relative expression across samples.

*Nrap* transcript sizes (∼5 kb) are within our 6-kb average read length making them an ideal test sample for our study. There are also fewer known splice isoforms, with one described as unique to cardiac (*Nrap-c*) and another to skeletal muscle (*Nrap-s*) (Mohiddin et al. 2003; Lu et al. 2008). We confirmed that *Nrap-c* (exon 12 excluded) was indeed the only isoform expressed in heart (Fig. 3). However, *Nrap-c* is not exclusively expressed in cardiac muscle but is differentially expressed alongside *Nrap-s* (+exon 12) between fast and slow skeletal muscles. This skeletal muscle-type difference went undetected in previous studies using a mixture of lower limb muscles (Lu et al. 2008). By phasing every known cassette exon in *Nrap*, we quantified the relative expression of all known *Nrap* isoforms in the three muscles. Doing so increased our sensitivity for rare isoforms, leading to the detection of an isoform exclusively expressed in heart and soleus muscles (Fig. 6A). This rare transcript lacks both exon 12 and exon 2, the latter being one of two exons that code for the N-terminal LIM domain (not part of a repeat region) known to interact with proteins that link the actin cytoskeleton to the cell membrane (Zhang et al. 2001). The LIM domain is also responsible for Nrap’s role in myofibrillogenesis during development (Carroll et al. 2001). Alternative splicing of exon 2 may modulate the binding affinity of the Nrap LIM domain to these myofibrillar and linker proteins.

At 22 kb, nebulin is more than twice the length of Iso-Seq's maximum read length. This resulted in reads of truncated nebulin transcripts. However, internal priming enabled coverage across the entire length of the transcripts. We only detected alternative splicing in exons of the 3′ half of the gene encoding the super repeat and Z-disk regions of the protein. Using our exon-based analysis, we detected differential splicing of multiple nebulin exons between 122 and 152 (NM_010889.1). Exons 13–132 (13–137 of ENSMUST00000238749.1) code for the super repeat region and exons 134–157 (139–165) code for the Z-disk region of nebulin in mouse. As all these exons are close in proximity, they fit within a single read, and were phased (exPhaser) together.

The majority of differential splicing of nebulin was in the Z-disk region including the unannotated exon u-002 (Figs. 2, 6C). Most of the exons of the Z-disk region are removed from fast-twitch EDL but are included in the slow-twitch soleus. The Z-disk plays a key role in contractile force transmission within and across sarcomeres. Differential splicing of the nebulin Z-disk region correlates with Z-disk width being thinner in white muscles and thicker in red muscles (Rowe 1973). Independent observations also found that the slow soleus and fast tibialis cranialis muscles corroborate our splicing data, suggesting that nebulin may have a functional role regulating the Z-disk width of different muscles (Buck et al. 2010).

We also detected alternative splicing of exons 127 and 128 (ENSMUST00000238749.1), which are located in the super repeat region bordering the Z-disk anchorage point. These exons are expressed in a mutually exclusive manner, are conserved in humans (NM_001271208), and code for peptides with different charge and hydrophobicity, suggesting distinctive functional roles (Donner et al. 2006; Lam et al. 2018). In humans, these exons show developmentally regulated inclusion (Lam et al. 2018). The peptides in the super repeat region of nebulin are binding sites for kelch like family member 40 (*KLHL40*), loss of which causes a nemaline-like myopathy (Garg et al. 2014). In turn, *KLHL40* is suggested to stabilize nebulin and Leimodin 3 (*LMOD3*, another protein implicated in nemaline myopathy) by acting like a chaperone and maintaining proper folding of these two proteins during muscle contraction (Garg et al. 2014).

Using exPhaser, we identified and quantified multiple novel nebulin transcripts in both the Z-disk and 3′ end of the super repeat region (Fig. 6C). Thus, our phasing data present a catalog of more rigorous splice combinations. None of the current nebulin transcripts annotated^7^ in RefSeq and GENCODE release M10 are expressed in the EDL and soleus muscles.

Titin transcripts (∼106 kb) are more than 10-fold longer than maximum Iso-Seq read lengths. This did not thwart the ability to determine differential exon usage through our exon-based exCOVator analysis. However, the read length puts limitations on exPhaser and required the exons to be analyzed in groups based on proximity. We then determined if our mouse findings are conserved in human by using CardioDB, TITINdb, and other resources (Roberts et al. 2015; Laddach et al. 2017; Savarese et al. 2018).

Titin exon 191 (NM_133378, N2-A) or 243 (LRG) in human is a cassette exon that is only removed in a subset of cardiac transcripts. The exon codes for one of multiple repetitive immunoglobulin-like (Ig-like) domains in the highly extensible I-band region of titin (Linke 2018). Ig-like domains can quickly unfold and refold, a property important for muscle elasticity and passive stiffness (Rief et al. 1997). This is especially relevant in heart where stiffness can affect the ejection fraction. It is possible that this cassette exon is spliced to adjust the length of the I-band region of titin and stiffness of sarcomeres in the heart (Opitz et al. 2004).

Exon 312 or 363 (LRG) in human is the penultimate exon and codes for part of the C-terminal region of titin that integrates into the M-band of the sarcomere, functions as a scaffold, and may play a role in mechanosensitivity (Zacharchenko et al. 2015; Linke 2018). This exon codes for the M-line intervening sequence 7 (M-is7) situated between the final Ig domains M9 and M10 of titin (Kolmerer et al. 1996). M-is7 binds calpain 3, a muscle-specific cysteine protease important for calcium regulated functions (Kinbara et al. 1997). Therefore, alternative splicing of exon 363 may alter the number of calpain 3 binding sites in the M-line region of titin in different muscle types. We found exon 363 to be excluded more often in skeletal muscle transcripts than in heart, but its physiological role is unclear. It is speculated that the availability of M-is7 allows cleavage of C-terminal titin by calpain 3 and thereby facilitates local remodeling of the sarcomere or the production of cleaved titin fragments for cellular signaling (Charton et al. 2015).

Alternative 3′ exon 45 (48 LRG) is part of the smallest titin isoform conserved in humans as Novex-3. The isoform is also known to interact with obscurin in the I-band and a variety of proteins in the Z-disk (Bang et al. 2001). It is speculated to play a role in myofibrillar signaling during muscle development and cardiac disease (Bang et al. 2001). We found it expressed more in skeletal muscle compared to heart even though it is often labeled as a cardiac isoform.

Exons 11–13 of titin are conserved in humans with the same numbering (LRG). They code for Z-repeat domains 4–6, respectively, in the N-terminal region of titin imbedded in the Z-disk. These exons are variably spliced in human and may be involved in assembling Z-disks of variable width (Gautel et al. 1996). Titin's Z-repeats interact with actinin alpha in the Z-disk, and proposed to be important for Z-disk organization, assembly, and force transmission between adjacent sarcomeres (Joseph et al. 2001). Alternative splicing of these exons seem to correlate with Z-disk width much like the Z-disk region of nebulin; the fast EDL excludes more exons compared to soleus. In addition, exon 11 of titin is always spliced out in skeletal muscle in a similar manner to humans (Savarese et al. 2018).

Iso-Seq is generally recommended for transcript isoform discovery rather than for quantitative measures. Yet based on our validations, we found FL reads extracted from consensus reads were accurate for relative quantification of the same exon/isoform across multiple samples. However, there are some caveats. This is not an absolute quantitative approach. PSI and percentages of isoform values cannot be used for comparisons across genes. Although the percentage of a specific exon/isoform may differ across samples, the absolute quantity of RNA for that isoform could be the same. Lastly, some genes do not support internal priming of oligo(dT) primers, but randomized primers could help. Despite these limitations, our approach opens the door to differential expression analysis of difficult transcripts.

The data shown have implications for clinical sequencing, as we have identified new exons and splicing patterns that could alter the interpretation of pathogenicity.

## Methods

### Animals and tissue harvesting

Six-week-old C57BL/6 (WT) male mice (Jackson Laboratory) were euthanized using CO_2_ followed by cervical dislocation. EDL, soleus, and cardiac (only 10 mg apex) muscles were excised. The excised muscles were flash frozen in liquid nitrogen for further processing.

### RNA isolation and purification

RNA was isolated from the cardiac apex, EDL, and soleus muscles using the *mir*Vana RNA Isolation kit (AM1560). DNA was removed using the Turbo DNA-free kit (AM1907) and assessed for purity using a NanoDrop 2000. All samples had 260/280 ratios >1.8 and 260/230 >2.0. RNA was quantified using a Qubit Fluorometer (v2.0) and RNA broad range assay kit (Q10210). Quality of total RNA was assessed on an Agilent Bioanalyzer 2100 using the RNA 6000 Nano kit (5067-1511). All samples had a RIN >7.6.

### Iso-Seq library generation and sequencing

Sequencing libraries were generated from the total RNAs from one mouse, following PacBio's Iso-Seq protocol (see “Data access”) with a few modifications. Briefly, poly(A)-selected cDNAs were generated for each muscle using barcoded primers following the Iso-Seq barcoding protocol. The amplified cDNA libraries were pooled and size selected for 5–10 kb using the BluePippin system (Sage Science). SMRTbell adapters were added and a second size selection step was performed to remove small library fragments. Sequencing was performed according to PacBio RS II protocol for the P6 chemistry on 24 SmrtCells.

### Iso-Seq data processing and alignment

Raw data were processed using SmrtAnalysis (v2.3) to produce FASTQ files (Supplemental Methods; Supplemental Code). Alignments performed using STAR v2.5.0a (Dobin et al. 2013; Thorvaldsdóttir et al. 2013) and GENCODE primary mouse sequence and annotation (GRCm38) version M10 (Ensembl 85) released 2016-07-19.

### Short-read RNA-seq data processing and alignment

Data were obtained from NCBI Sequence Read Archive (SRA; https://www.ncbi.nlm.nih.gov/sra) under accession numbers SRR7415846 and SRR7415850 (Singh et al. 2018). QC was performed using FastQC (http://www.bioinformatics.babraham.ac.uk/projects/fastqc) and Trimmomatic (Bolger et al. 2014). Data were aligned using STAR 2.5.2a (Dobin et al. 2013) and the same GENCODE reference above.

### Reverse-transcription PCR and Sanger sequencing

Primers were designed using NCBI Primer-Blast (Supplemental Table S2). RT-PCR reactions were performed using SuperScript III (12574030) following the manufacturer's protocol. Samples used in validation (mouse 2 and 3) are independent from the sequenced sample (mouse1). Amplification products were run on a 2% agarose gel, excised, gel purified (Qiagen 28704), and Sanger sequenced.

### exCOVator and exPhaser analysis pipeline

Exon-based analysis was performed using the exCOVator pipeline, which includes scripts for identifying novel exons, and differential exon usage or PSI analysis. The identification and relative quantification of transcript structures were performed using the exPhaser pipeline. Both pipelines were created using Python, MyGene (Xin et al. 2016), Biopython (Cock et al. 2009), and HTSeq (Anders et al. 2015), tools from the *DEXSeq* R library (Anders et al. 2012). For full details of the analysis approach, see Supplemental Methods and Supplemental Code.

## Data access

All raw and processed sequencing data generated in this study have been submitted to the NCBI Gene Expression Omnibus (GEO; https://www.ncbi.nlm.nih.gov/geo/) under accession number GSE138362. Sanger sequencing data can be found in the Supplemental Data. All scripts, detailed notes, links, and archived versions of PacBio protocols used in this project are archived in the Supplemental Code and made available in our GitHub repository (https://github.com/puapinyoying/isoseq_manuscript_resources).

## Competing interest statement

The authors declare no competing interests.

## Supplementary Material

Supplemental Material

## Footnotes

7 Our analysis was based on GENCODE release M10 (Ensembl 85). At the time of writing, we checked the latest GENCODE annotation (release M22, Ensembl 97) to ensure our findings were up to date. We found no changes for *Nrap* or titin. However, alternative splicing of titin exon 312 is well established (Kolmerer et al. 1996; Kinbara et al. 1997; Charton et al. 2015). Yet, it remains unannotated in the latest GENCODE and RefSeq databases. Without a formal accession number, we decided to keep the label as “UNANNOTATED 2” (Fig. 7C). For nebulin, two new inferred transcripts (ENSMUST00000238749.1 and ENSMUST00000238288.1) were added to release M22 that contain the three missing exons between 147 and 148. The exons labeled as 3*, u-002, and 143** in Figures 2 and 6C are 153, 154, and 155 of ENSMUST00000238749.1, respectively. The two mutually exclusive exons labeled as 7*** and 123 in Figure 6C are 127 and 128 of ENSMUST00000238749.1, respectively. The four exons between 85 and 86 of NM_010889.1 are exons 71–74 of ENSMUST00000238749.1. The “UNANNOTATED 7” (Fig. 6C) transcript matches ENSMUST00000238288.1 (exon 128 spliced out). ENSMUST00000238749.1 contains all exons and is not expressed in any muscle, further supporting that exons 127 and 128 are mutually exclusive and the annotation is likely incorrect but may serve as an inferred complete transcript similar to titin NM_001267550.1. Thus, we used the RefSeq annotation (NM_010889.1) that contains 157 exons as a reference for the analyses.
