# Supplementary material for: A long-read RNA-seq approach to identify novel transcripts of very large genes
Source: Genome Res. 2020 Jun;30(6):885–97. doi: 10.1101/gr.259903.119 (PMC7370890; doi:10.1101/gr.259903.119)
Supplement: Supplemental Material [file supp_30_6_885__index.html]

A long-read RNA-seq approach to identify novel transcripts of very large genes — Supplemental Material 

# A long-read RNA-seq approach to identify novel transcripts of very large genes

## Supplemental Material

- Supplemental\_Material.pdf
- Supplemental\_Methods.pdf
- Supplemental\_Data.zip
- Supplemental\_Code.zip
