# Supplementary material for: A long-read RNA-seq approach to identify novel transcripts of very large genes
Source: Genome Res. 2020 Jun;30(6):885–97. doi: 10.1101/gr.259903.119 (PMC7370890; doi:10.1101/gr.259903.119)
Supplement: Supplemental Material [file supp_gr.259903.119_Supplemental_Material.pdf]

SUPPLEMENTAL MATERIAL

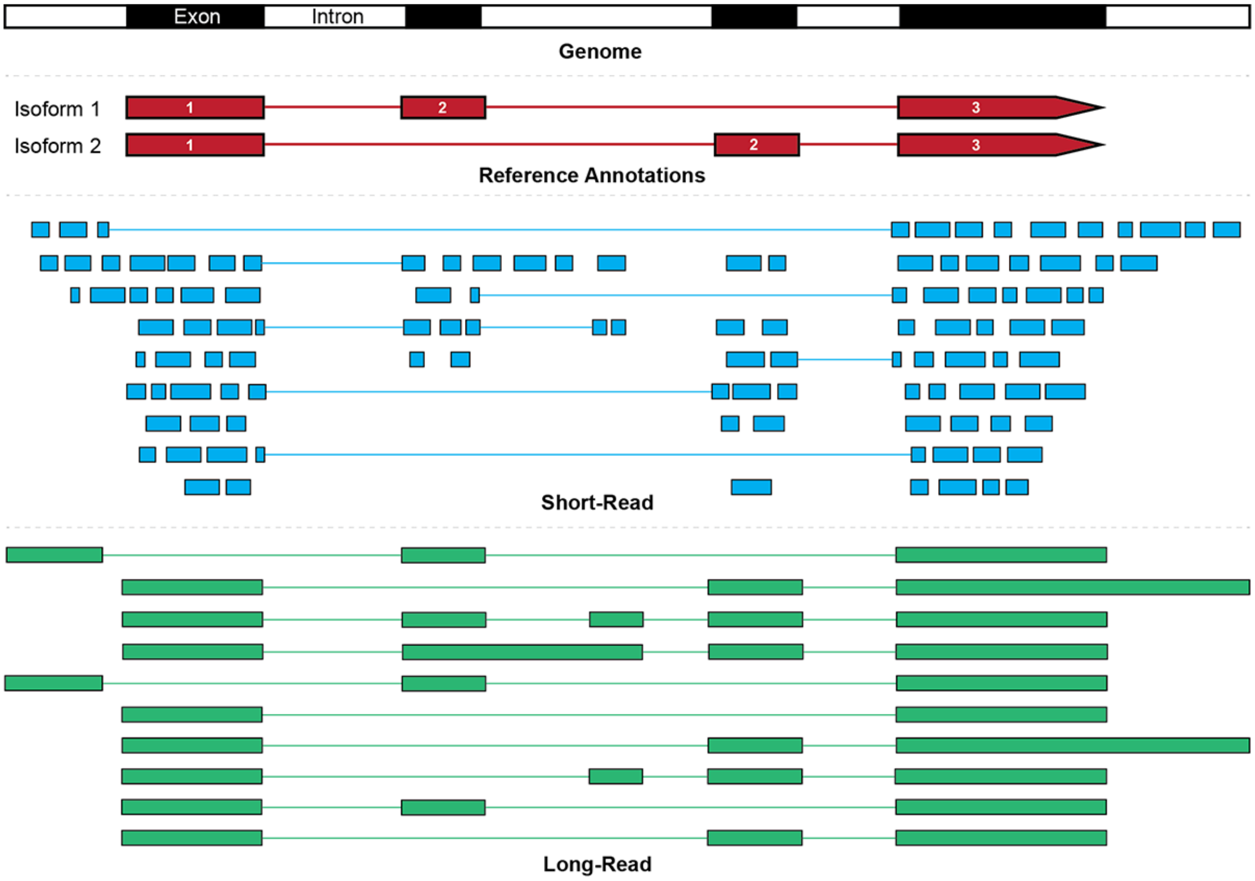

Supplemental Figure S1. Comparison between short-read and long-read RNA-seq. Short-reads (<=300bp) are too short to span more than two exon junctions. Algorithms are used to assemble transcripts from the data. However, predictions based on data may incorrectly predict novel isoforms from mis-mapped reads and artifacts. Long-read sequencing (<= 10kb+, 6kb average) may span entire transcripts or large portions of them which allows for phasing of exons and isoform identification.

Supplemental Table S1. Sequencing statistics from classify output (24 SMRT cells)

Multiplexed (pooled)	Number of reads
Reads of Insert (ROI)	1,955,502
Five prime (5’)	711,096
Three prime (3’)	1,020,887
Poly(A)	935,179
Filtered short reads	26,521
Non-full-length	1,415,922
Full-length*	513,059
Full-length non-chimeric**	509,095

Demultiplexed data	Reads of insert (ROI)
Soleus (barcode 0)	163,368
EDL (barcode 1)	167,632
Cardiac (barcode 2)	178,095
Total identified by barcode	509,095

Average read length	Base pairs
Full-length non-chimeric	6,009

*Full-length - determined by presence of cDNA library primers and poly(A) sequence
**Chimeric reads (artificial chimeras) - sequencing artifacts formed by insufficient SMRTbell adapters, random fusion of ligated transcripts during PCR or true biological fusion events of two separate transcripts that cannot be distiguished

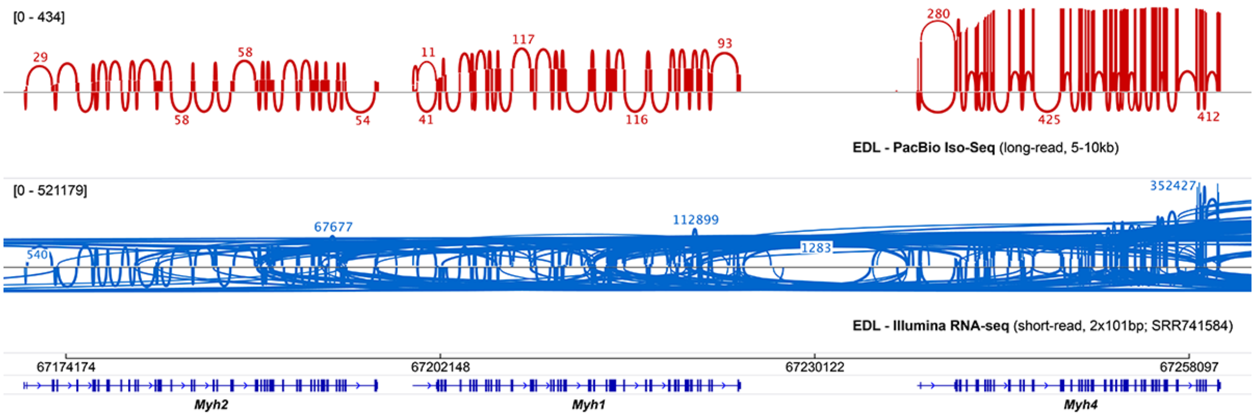

Supplemental Figure S2. Sashimi plot showing the splice junctions of long-read and short-read RNA-seq data mapped to three members of the myosin heavy chain gene family (*Myh2, Myh1* and *Myh4* on Chromosome 11). Sashimi plot for Iso-Seq data show consensus reads counts (prior to FL read extraction using exCOVator and exPhaser). Minimum splice junction coverage was set to 5 and most junction values were removed for visual clarity.

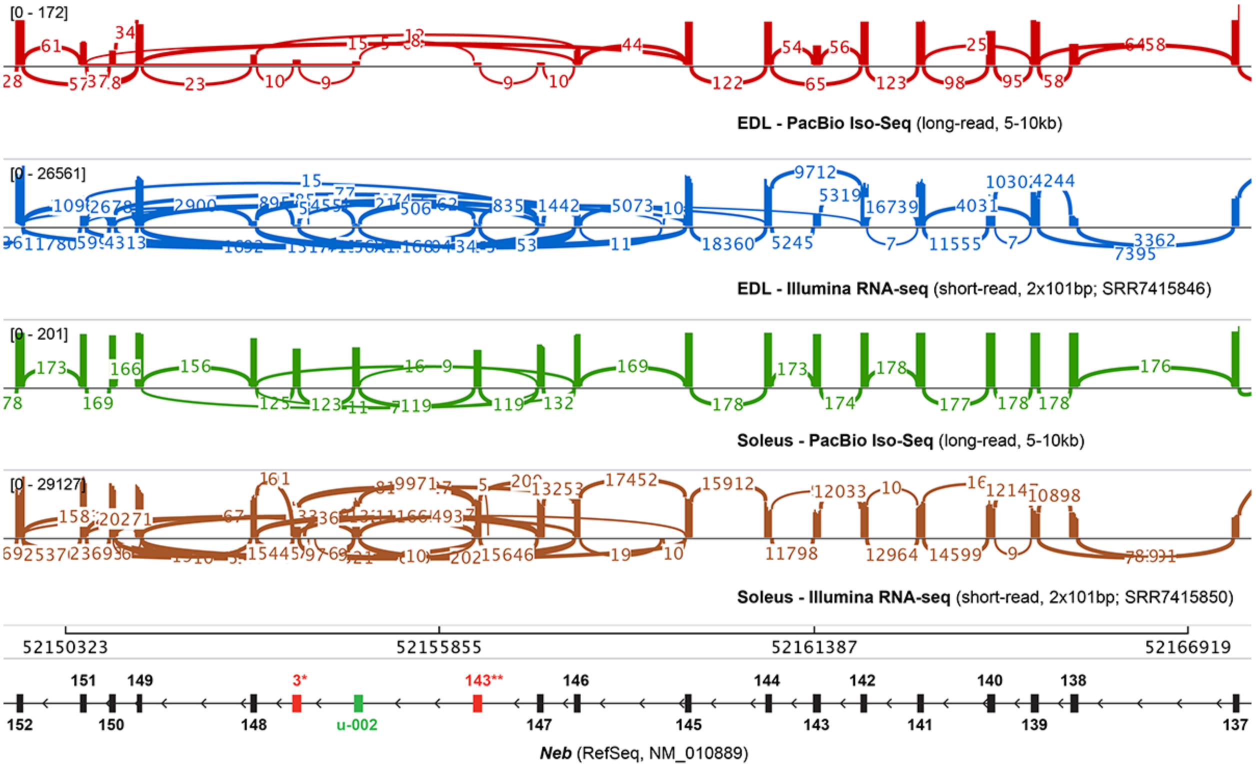

Supplemental Figure S3. Sashimi plot of nebulin exons 137-152 comparing long-read vs short-read RNA-seq of EDL and soleus muscles. This region of nebulin contains highly repetitive nebulin domains making this intragenic region difficult to map unambiguously with short reads, leading to increased splice artifacts. Long-reads can span the entire region, greatly reducing spice artifacts. Sashimi plots for Iso-Seq data show consensus reads in the BAM file prior to full-length read extraction using exCOVator and exPhaser. Consensus reads may be composed of one or more full-length reads and therefore splice junctions are underrepresented when compared to Illumina short-reads. Minimum splice junction coverage was set to 5 for visual clarity.

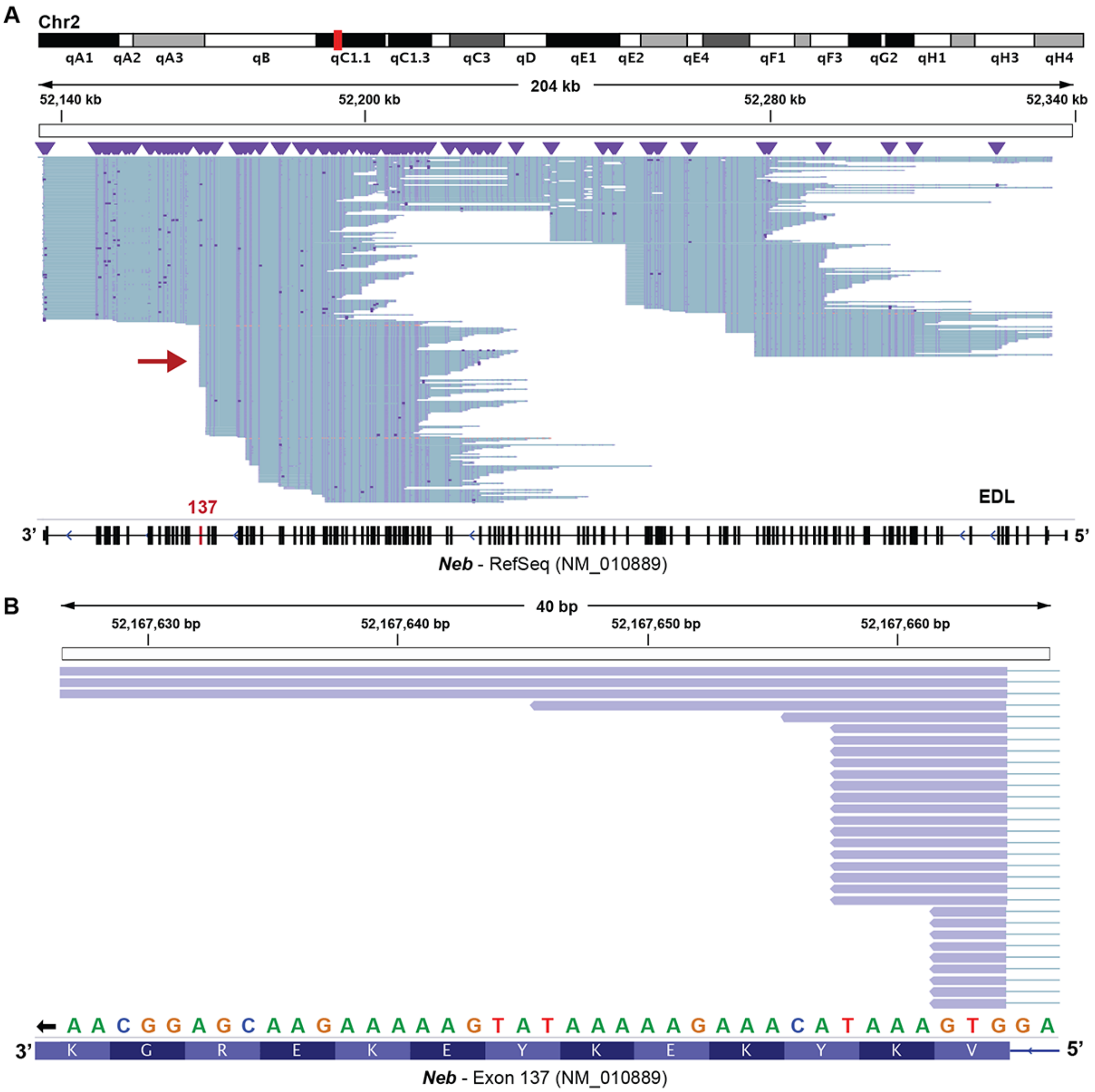

Supplemental Figure S4. IGV screenshots of long reads tiled across mouse nebulin due to internal oligo(dT) priming. A) Screenshot of full-length reads (blue/purple lines) tiled across the entire nebulin gene. Nebulin is on the anti-sense DNA strand and is oriented in the right (5') to left (3') direction. The mouse nebulin transcript (NM_010889.1) can be up to 22.4kb, or 2x the maximum read length (10kb). Red arrow, the first location upstream of the 3'-end to map several reads due to internal priming. B) Zoomed image of exon 137 showing reads produced from cDNAs generated by internal priming of oligo(dT)s. Reference sequence shows consecutive 'A' bases followed by non-A bases downstream of

where the reads align. Purple lines are coding/exon regions of a reads. Thin connecting blue lines signify intronic sequences that were spliced out. The black lines, dots, and arrowheads are indel errors. All reads shown are consensus reads. Each consensus read can be made up of one or more full-length reads.

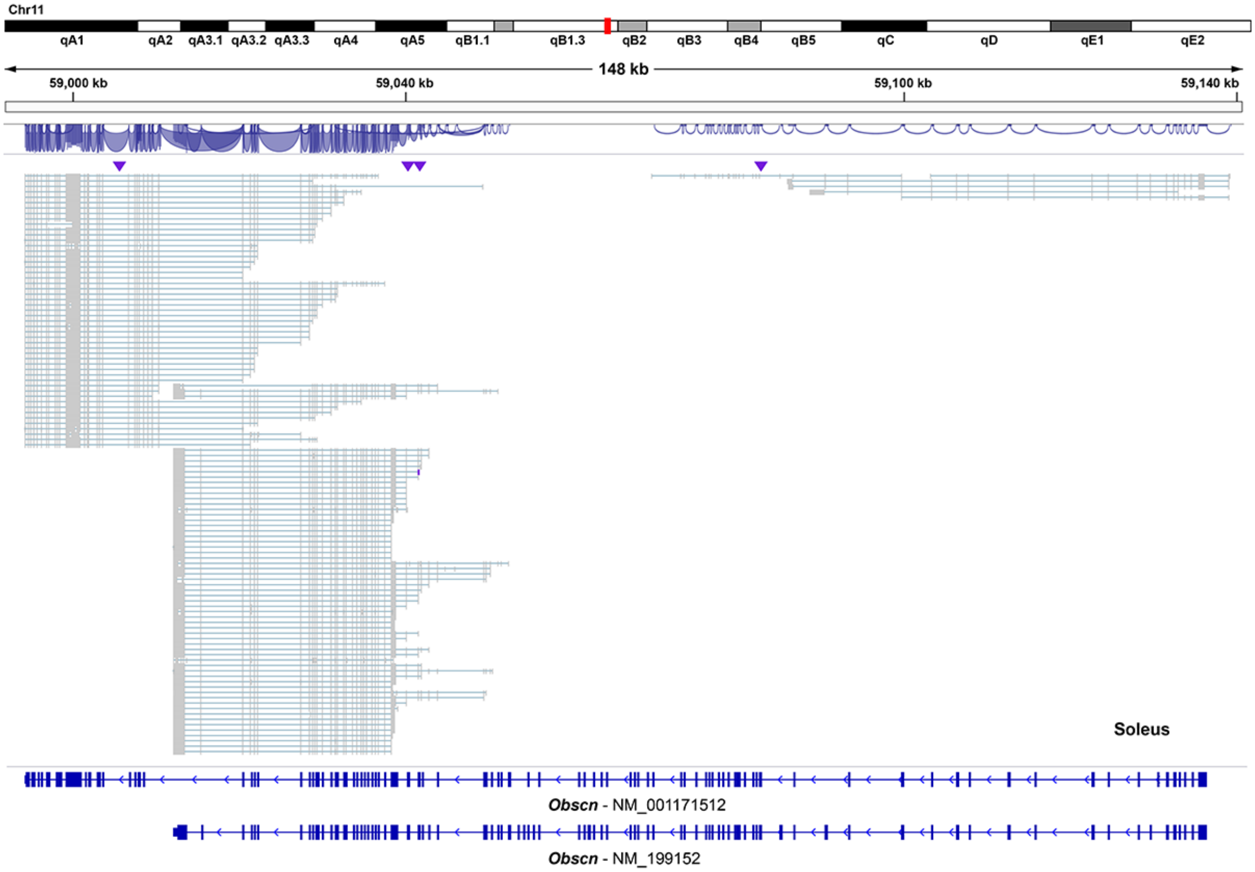

Supplemental Figure S5. IGV screenshot of long reads failing to cover the middle of the *Obscn* gene due to insufficient internal priming. All reads shown are consensus reads. Each consensus read can be made up of one or more full-length reads.

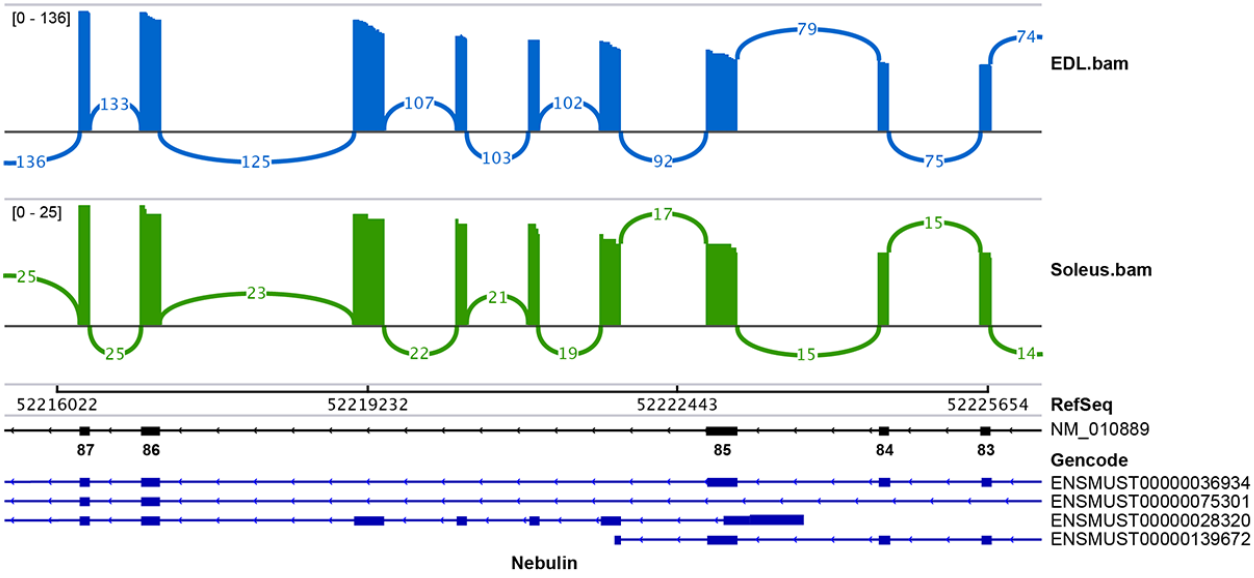

Supplemental Figure S6. Sashimi plot showing the splicing pattern of Nebulin exons 83-87 in EDL and soleus muscles. The data shows 4 exons between 85 and 86 that are not annotated in the RefSeq database and are constitutively expressed in both EDL and soleus skeletal muscles. These 4 unannotated exons are represented in GENCODE M10 in ENSMUST00000028320 and GENCODE M22 in two additional transcripts ENSMUST00000238749 and ENSMUST00000238288 (not shown). Sashimi plot showing consensus reads. Minimum splice junction coverage was set to 5 for visual clarity.

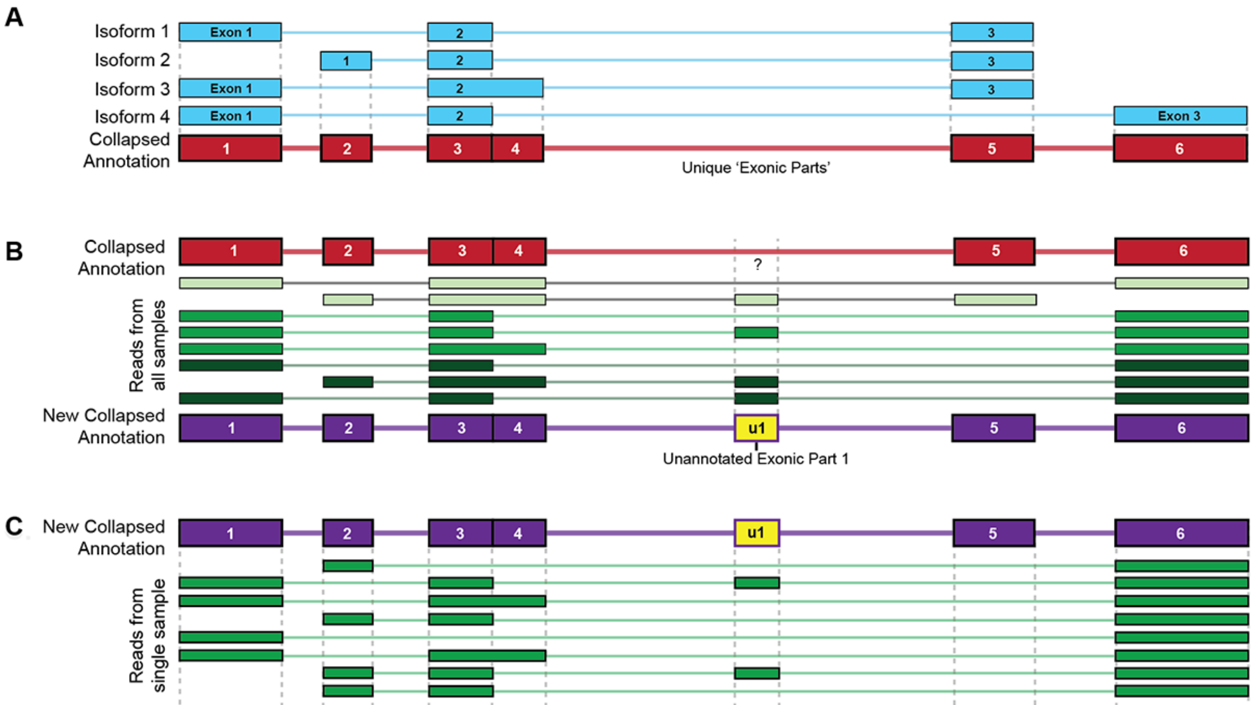

	1	2	3	4	u1	5	6
Match Exon	4	4	6	2	2	0	8
Total Reads	4	8	8	8	8	8	8
PSI (Match/ Total * 100)	100%	50%	75%	25%	25%	0%	100%

PSI	1	2	3	4	u1	5	6
Sample A	100%	75%	75%	75%	50%	0%	100%
Sample B	100%	50%	75%	25%	25%	0%	100%
Sample C	100%	50%	25%	0%	25%	25%	100%

Supplemental Figure S7. Custom exon-based Iso-Seq analysis pipeline (exCOVator). A) Transcript isoforms often share multiple exons complicating analysis. Using the 'dexseq_prepare_annotation.py' script, annotations were collapsed into unique exonic parts (EP) for analysis. B) Read data from all samples are used to find unannotated sequences (potential novel exons) not present in the collapsed annotation file. Unannotated exons (e.g. u1) are added to the list of annotations for differential usage analysis. C) Count match and total reads for each all EPs for individual samples and calculate a ratio (match/total) that is multiplied by 100 to obtain the percent spliced-in (PSI). In this illustration, EP6 is constitutively expressed; EP1 and EP2 are alternate 5' exons; EP5 is an alternate 3' exon that is not expressed in this sample; EP3, EP4 and unannotated exonic part 1 (u1) are cassette exons. D) Observing differential exon usage across samples. The ratio is a normalized value that can be used to compare exon usage across multiple samples. The ratios for each sample are used by 'filterDiffUsedExons.py' for further filtering exons with less than

defined % difference across samples. EP1 and EP6 have 0% change across samples and would be filtered out. EP2-5 and u1 are differentially used and will be retained for further analysis.

Supplemental Table S2. Primers used for RT-PCR and Sanger sequencing validation

Gene	Exon	Exon Location	RefSeq (mm10)	Primer Dir	Primed Exons	Primer sequence 5'-->3'
Neb	138	Chr2:52165167-52165277	NM_010889.1	Fwd	134-135	ACCTTGCAAGCGAGGTCAA
Neb	138	Chr2:52165167-52165277	NM_010889.1	Rev	139	GCTCTCAGCATGTCAGGAGT
Nrap	12	Chr19:56374364-56374468	NM_008733.4	Fwd	9	GACCGATGTGGCCAGGTTTACTCAGAAG
Nrap	12	Chr19:56374364-56374468	NM_008733.4	Rev	14	CAGGGGAACCAGCCTCATCGTTGTTTG
Ttn	191	Chr2:76802231-76802497	NM_011652.3	Fwd	189-190	TGGAGCCTAACGATAAGGTGG
Ttn	191	Chr2:76802231-76802497	NM_011652.3	Rev	194	CATCCTCAAGCTGCGCATTC
