# Supplementary material for: A long-read RNA-seq approach to identify novel transcripts of very large genes
Source: Genome Res. 2020 Jun;30(6):885–97. doi: 10.1101/gr.259903.119 (PMC7370890; doi:10.1101/gr.259903.119)
Supplement: Supplemental Material [file supp_gr.259903.119_Supplemental_Methods.pdf]

SUPPLEMENTAL METHODS

Detailed version of laboratory methods

Animals and tissue harvesting

Six-week old C57BL/6 (WT) male mice (JaxLabs) were euthanized using CO_2 followed by cervical dislocation. EDL, soleus and cardiac muscles from the same animal were excised. For cardiac muscle, only 10 mg of the apex was acquired. The excised muscles were flash frozen in 2-methybutane (Sigma) in liquid nitrogen and stored at -80°C for further processing.

RNA isolation and purification

The cardiac apex was processed individually, while the left and right EDL, and soleus from the same mouse were pooled by muscle type for processing. The muscles were thawed to -20°C, cut into 2 mm cubes, placed into pre-chilled T-prep devices (COVARIS, Cat# 520097) and crushed into a fine powder using the impactor (COVARIS, Cat# 500305) following manufacturer's protocol. Afterwards, 600µl of lysis buffer from the MirVana RNA Isolation kit (Thermo Fisher: AM1560) was added to each T-prep device, the homogenate transferred into fresh tubes and total RNA was extracted following the MirVana protocol.

DNA was removed using the Turbo DNA-free kit (ThermoFisher, #AM1907). Purification by ethanol precipitation was performed by mixing 0.1x volume of 3M sodium acetate (ThermoFisher, #AM9740) and 3x volume of 100% molecular grade ethanol into the RNA samples. The mixture incubated overnight at -20°C and centrifuged at 10,000g at 4°C for 20 minutes. The supernatant discarded, and RNA pellet rinsed with 400µl of fresh 70% ethanol and centrifuged again. This wash step was repeated twice before the RNA pellet was left to dry at

room temperature for 5 minutes. The pellets were resuspended in nuclease-free water and assessed for purity using a NanoDrop 2000. All samples had 260/280 ratios > 1.8 and 260/230 > 2.0

Assessing RNA quantity and quality. RNA was quantified using a Qubit Fluorometer (v2.0) and RNA broad range assay kit (ThermoFisher, #Q10210). Quality of total RNA was assessed on an Agilent Bioanalyzer 2100 using the RNA 6000 Nano kit (Agilent, # 5067-1511). All samples had a RIN > 7.6. The purified total RNA samples were stored in - 80°C.

Iso-Seq library generation and sequencing

Total RNAs from one mouse was reverse transcribed following PacBio's Iso-Seq procedure and checklist using Clonetech SMARTer PCR cDNA Synthesis Kit and BluePippin Size-Selection System (P/N100-377-100-04). Three starting reactions of 600ng-1μg total RNA were used per muscle sample for first stand cDNA synthesis. Custom 3' primers containing barcodes were added during this step: soleus (dT_BC1), EDL (dT_BC2), and Cardiac (dT_BC3) following the Iso-Seq Barcoding protocol. After first strand synthesis, the number of cycles chosen for large scale PCR were: 12 for soleus and EDL and 10 for cardiac samples.

After amplification, cDNA libraries were pooled in equimolar amounts and the subsequent steps were performed according to protocol with modifications. During the BluePippin size selection step, 500ng of cDNA was loaded per lane of the gel cassette and only the 5-10kb size fraction were collected for further library generation. SMRTbell adapters were added using the SMRTbell Template Prep Kit 1.0 (#100-259-100). A second BluePippin size selection step was performed after SMRTbell templates were generated to remove small library fragments.

Single-molecule sequencing. Sequencing was performed according to PacBio RS II protocol. Briefly, DNA polymerase was bound using DNA/Polymerase Binding Kit P6 V2 (#100-

372-700). DNA P6 Control Complex (#100-356-500) was added and DNA Sequencing Reagent 4.0 (#100-356-200) was loaded onto the system. During sequencing loading optimization (first 8 SMRTcells), two concentrations were used on four cells each: 1.0nM and 1.2nM. The 1.2nM concentration resulted in a higher yield of total reads of interest and the concentration was used for the remaining SMRTcells. The pooled Iso-Seq libraries were sequenced using a total of 24 SmrtCells (#100-171-800) on the PacificBiosciences RSII machine (output statistics in Table S1).

Iso-Seq data processing and alignment

Raw data (bax.h5) was processed according to the official Iso-Seq protocol using SmrtAnalysis (v2.3) with a few modifications to help process the large amount of data. Briefly, the fasta files produced from individual SmrtCells after the circular consensus step (CCS) were merged into two fasta files composed of eight SmrtCells. The two merged files were demultiplexed into the three muscle samples based on their barcodes yielding six total files. Barcodes numbers were converted to zero-based according to protocol (dT_BC1→BC0, BC1→dT_BC2, and dT_BC3→BC2). The six files were processed through classify and cluster steps in the Iso-Seq method to produce FASTQ files. FASTQ files belonging to each muscle were concatenated producing three large FASTQ files (e.g. soleus.fastq, edl.fastq, cardiac.fastq) ready for alignment. To allow for this type of merging, cluster numbers (c1, c2, c3 etc) from each FASTQ file's read names were renumbered for each consecutive FASTQ file to avoid overwriting redundant cluster numbers. See mergeFastq.py script in github repository. Finally, the alignment steps were performed using STAR v2.5.0a and GENCODE primary mouse sequence and annotation (GRCm38) version M10 (Ensembl 85) released 2016-07-19. All data processing steps were performed on the Colonial One George Washington University HPC using the Slurm

workload manager. See settings and scripts (https://github.com/puapinyoying/isoseq_manuscript_resources/s1_isoseq_data_processing)

Short-read RNA-seq data processing and alignment

Data was obtained from GEO (SRR7415846, SRR7415850). QC using FastQC.
FASTQ files trimmed using Trimmomatic (v0.36). Samples aligned using STAR 2.5.2a and the same GENCODE annotation and reference genome. See settings and scripts (https://github.com/puapinyoying/isoseq_manuscript_resources/s1b_shortread_data_processing)

Reverse-transcription PCR and Sanger sequencing

Primers were designed using NCBI Primer-Blast (Table S2). RT-PCR reactions were performed using SuperScript III One-Step RT-PCR system (ThermoFisher, #12574030) following manufacturing protocol. Samples used in validation (mouse 2 and 3) are independent from the sequenced sample (mouse1). The amplification products were loaded into a 2% agarose gel made with 1x TBE buffer and 5µl ethidium bromide for electrophoresis and imaging.

The RT-PCR products were excised from the agarose gel and purified using Qiagen gel purification kit and sent along with primers to Quintarabio for Sanger sequencing. Alignments of the sequences were visualized in IGV after using the BLAT tool.

exCOVator exon-based analysis pipeline

Identifying novel exons and differential usage analysis. The goal of this analysis pipeline was to compare splice differences between the three different tissues and identify unannotated / novel exons. We decided to compare differential exon usage (inspired by *DEXSeq* for short reads) between samples. This approach can measure relative exon usage across conditions

regardless if the full-length reads cover entire transcripts or if the read was derived from internal priming. Therefore, analysis through differential exon usage would open up opportunities to analyze and compare the splicing behavior of extremely long transcripts such as ones from structural genes that are far beyond technical sequencing limitations.

Counting number of cluster-reads per gene. During the preliminary analysis, determining which muscle structural genes to observe consisted of randomly choosing familiar gene names into the Integrated Genomics Viewer (IGV). We need a systematic way to narrow down the scope of the analysis. According to the GENCODE release archive for version M10, there are 48,440 total functional elements in the genome. These functional elements are discrete units or genes located on the genome. We needed to determine which of these genes were transcribed and were sequenced. In other words, how many of these genes contain sequence coverage or cluster reads mapped to them. In order to determine which genes had mapped reads, the number of cluster-reads per gene in each alignment (BAM) file were counted using HTSeq-count software. We found 3219 genes that had at least one cluster read in one of the three samples. This step reduced the scope of the analysis to 6.65% of the total genes annotated in the GENCODE file. Since we sequenced a specific tissue type (muscle), not all of the genes in the genome should be expressed (contain no RNA transcripts or mapped reads). By reducing the number of target genes to a fraction of the total, we were able to dramatically reduce noise and computational load for downstream analyses.

Obtaining a list of genes with coverage above a minimum threshold. The next step was to generate a list of genes that had enough cluster reads to be confident in the data and be able to compare differential exon usage between samples. To do this, the cluster read count data was imported into Excel. We chose a minimum cut-off filter of 10 reads per gene in at least one of the three muscle samples. This method yielded 1520 genes that met the minimum threshold

minus the Riken genes from the data set (see ‘excovator_exon-based_counts.xlsx’ in Supplemental Data). The final result was a list of 686 unique genes with >= 10 cluster reads present in at least one of the three samples. These criteria were selected because some genes may be uniquely expressed in one of the muscle types or poorly expressed in some tissues (e.g. Nebulin in heart). This method ensured that those genes would not be filtered out of the analysis.

Reducing the number of annotations to analyze from the (GENCODE) GTF file. Once We obtained the list of 686 genes to analyze, their transcript annotations were selected from the original GENCODE annotation file and placed into a separate file. To do this, a custom script called ‘selectGencodeAnnot.py’ was used to automatically search for and place all of the annotations for the 686 genes into a new abbreviated annotation (GTF) file. The original GENCODE annotation file is extremely large (765 Mb GTF file) and only 1.4% of the exon annotations pertained to the list of genes needed for analysis. This step further scaled down the complexity of subsequent analyses by reducing the number of annotated exons to analyze by 91.9% from 1,617,660 to 130,820.

Collapsing redundant exons in the abbreviated GTF file. Most genes express more than one splice isoform and the resulting transcripts often share multiple exons (e.g. many exons are constitutively expressed). This redundancy of exon annotations can make differential exon usage analysis difficult and inefficient. In addition, having multiple splice isoforms / different transcripts can lead to confusion in exon numbering (e.g. exon 4 of transcript-A is may have the same genomic location as exon 7 of transcript-B). In some cases, an exon of one transcript could contain shortened or extended nucleotide sequences 5’ or 3’ of an exon of an alternate isoform making analysis even more complex.

In order to reduce analysis complexity, we used the ‘dexseq_prepare_annotation.py’ script included in the *DEXSeq* software package (v1.28.1). This *DEXSeq* script takes the transcripts/splice isoforms belonging each gene and creates a single collapsed (‘meta’) annotation (Supplementary Figure S7A, red). This annotation has an independent and consistent numbering system that is ordered by chromosomal location. The new numbering system can also account for the existence of shorter and/or longer exons by dividing each coding region into discrete units referred to as exonic parts (for more detailed explanation see *DEXSeq* documentation). After collapsing the GENCODE annotations (GTF) file for the 686 genes, the number of exon annotations to analyze was reduced by 80% from 130,820 exons to 26,513 unique exonic parts in the collapsed annotation (GFF) file. With the target gene and exonic part annotations ready, we could then determine exon usage.

Turning qualitative cluster reads into quantitative full-length reads. PacBio’s real-time sequencing technology is known to produce many random insertion and deletion errors. Their Iso-Seq method (bioinformatics processing pipeline) corrects for these errors by collapsing similar full-length reads into consensus (cluster) reads which are further improved through additional polishing using non-full length (NFL) reads. Although this approach greatly improves sequence quality and is useful for determining unique splice transcripts, the method reduces the quantitative nature of the data (e.g. a single cluster read could be produced from 1 full-length read or >5000 full-length reads). Fortunately, the Iso-Seq method notes the number of full-length and NFL reads used to form each cluster read in the read names of the FASTQ file. PacBio states that this information should not be directly used for precise quantitative expression analysis (e.g. like Illumina RNA-seq data). However, we sought to test if the data could be used quantitatively by validating our findings through RT-PCR. Therefore, in subsequent steps the ‘exCOVator.py’ script, we extract and analyze only the full-length reads

counts instead of cluster read counts. Its important to note that this can be muddled with collapsed reads, so do not use the ToFU pipeline.

Finding unannotated exons from the data set. Determining read coverage based on annotated exons alone neglects potentially novel/unannotated exons that may exist in our data. Long read technology has the opportunity to differentiate potential unannotated exons from sequencing and alignment artifacts seen in short-read data because assembly of transcripts is not required. In order to find unannotated exons, the 'exCOVator.py' script uses the python HT-Seq library to: 1) sort all of the reads belonging to each input sample BAM file by the genes they map to, 2) iterate through each sample read and split into its constitutive exons using information provided in their CIGAR strings for every gene, 3) iterate through each exon of the read, 4) check to see if the exon's genomic coordinates intersect with any annotated exons belonging to the same gene, 5) skip to the next read exon if there is a match, and 6) if the exon does not intersect with any annotated exons belonging to the gene, add the unannotated exon coordinates into the collapsed annotation file (Supplementary Figure S7B). Unannotated exons are first numbered, prefixed with a 'u-' (for differentiation), and sorted by starting coordinates before they are added alongside the list of annotated exons. Using this method, 1,218 unannotated exons were found in the three muscle samples, not present in the abbreviated GENCODE annotation (GFF) file. This addition brings the total exonic parts to 27,731. Unannotated exons found depend on the sample BAM and the annotation GTF files given (e.g. the more comprehensive GENCODE reference file will contain fewer unannotated exons than a RefSeq annotation file and samples from different tissues may express different transcript isoforms/exons).

Normalizing exon coverage for cross sample comparisons. Recalling the primary observations of the data (Supplemental Figure S4), there was uneven exon coverage of genes within samples, especially 3' end vs 5' ends of very large genes due to internal priming of oligo(dT)

primers. In addition, there is sample-to-sample coverage variation for each gene from sequencing and library preparations. This variation makes it difficult to compare differential exon usage between samples. In the 'exCOVator.py' script, this variation is accounted for by normalizing read coverage for each exonic part within each sample.

To normalize read coverage, a fraction (ratio similar to percent spliced in or PSI) is calculated from the total population of reads mapping to each exonic part (Supplementary Figure S7C). The denominator for the fraction is a total count of reads that have genomic coordinates intersecting the exonic part's coordinates. This value will be referred to as the 'total reads' for the exonic part. Reads that have intersecting coordinates with the exonic part (total reads) may or may not have a sequence match with the exonic part. The numerator of the fraction is the subpopulation of total reads that contain an exact sequence match to the annotated exonic part in question. To check if any of the reads in the population of total reads have a sequence match to the annotated exonic part, a check is performed on each read one by one. The script breaks each read into its constituent exons using the exon boundary information contained in its CIGAR string. Next, each exon of that read is checked to see if its coordinates intersect that of the annotated exonic part. If it does, it is counted as a 'match read'. This allows for the analysis to tolerate reads containing some inaccurate base calls since we are analyzing the data at the exon level. However, the analysis can produce artifacts if the read contains indels at an exon junction, is mismapped or if the sequencing data is contaminated with DNA (reads containing introns). Therefore, some manual inspection of reads in IGV is required.

Using this method, the total count, and match count for all 27,731 annotated and unannotated exonic parts were calculated. Finally, the population of match reads is divided by the total reads to generate a normalized ratio or PSI for each exonic part (PSI = match / total = ratio * 100). This ratio can be used to compare differential exon usage within and across different samples. However, this will only determine coverage for all annotated (known) exonic parts. After the match reads, total reads, and ratios were calculated for each exon in all three of

the samples, the data was output into a CSV file. However, this large sum of exonic parts and the resulting data table was going to be challenging to analyze manually.

Visualizing differential exon usage between tissues. The CSV data file with all of the ratios contained too many exons to compare between samples by eye. Therefore, an intuitive way to visualize differential exon usage between samples would be useful for analysis. The `exCOVator.py` script generates a PDF file containing coverage plots for each gene. Each gene plot is composed of two types of graphs that share a common x-axis (Figure 3A, 4A and 5A). This x-axis displays all of the exonic parts (annotated and unannotated) for the gene ordered by chromosomal location. The top graph represents each sample as a colored line. Its y-axis displays the coverage ratio (full-length match/total read counts) for each exonic part. A ratio of 1 represents 100% of the total reads of the exon are all sequence matches. However, a ratio of zero is more ambiguous; a zero can represent no match reads or no total reads. In addition, a low denominator (total reads) can make ratios unreliable and/or provide less confidence in the data (e.g. a 0.5 ratio could be match/total of 300/600 or 2/4). To remove this ambiguity, we added a stacked bar graph below the line graph that shows the total reads for each sample. This way the user can assess both the total read count and differential exon usage in one glance to determine if the exon is worth analyzing further or rule out as an artifact. Once it was possible to visualize the data and find differentially used exonic parts, it would still be useful to filter and and obtain additional information about the exonic parts from the original CSV data file.

Filter for exon coverage and differentially used exons between samples. Most exons of a gene are used in all splice isoforms across multiple tissues (constitutively expressed). However, some exons are not used at all in splice isoforms of specific tissues (e.g. muscle vs brain). Both cases are examples of exons that will not be differentially used between samples; the ratios (PSI) remain constant in all samples and become additional noise in the analysis (Supplementary

Figure S7D, black percentages). To narrow down our candidates by selecting for exons that were adequately covered and differentially used to for further analysis, we developed the script `s6_filterDiffUsedExons.py` that takes the exCOVator output (686_genes_exon_ratios.csv) allows the user to provide variety of filter settings such as a minimum coverage threshold, choice to filter by total consensus reads per exon or FL reads (in at least one sample), and a minimum ratio difference between at least two samples. The default settings used in this analysis are more conservative to capture the most robust changes and reduce the number of artifacts. We chose a minimum of 30x total consensus ('ctotal') read coverage per exon in at least one of three samples and a 0.20 (20% PSI) ratio difference between at least 2 samples (Table 1). Filtering by 30x total consensus reads reduced the number of genes from 686 to 143. By selecting for exons with a 0.20 ratio difference further reduced the 143 genes and 5,053 exonic parts to 55 genes and 327 exonic parts respectively.

Obtaining full gene names and summaries. Many genes found to have differential exon usage may have little to do with the focus of the study. Therefore, additional information about the genes would help determine their relevance to the study (muscle) such as their full names and function rather than just gene symbols. The NCBI gene database contains comprehensive summaries for each gene and an application-programming interface (API) for users to automate queries and obtain data. In order to obtain additional gene information from NCBI, we created a script called `selectGeneSummaries.py` that uses two python libraries: MyGene and BioPython. The goal was to convert the 55 gene symbols into NCBI gene ids. These ids could then be used in a separate query to obtain the gene summaries using NCBI's batch Entrez. The BioPython library makes automating the queries with NCBI's databases much simpler. We had 55 genes to query (gene list output by `s6_filterDiffUsedExons.py`), but other studies may have much larger number of genes to query for gene ids and summaries that could reach NCBI's user limit. Going over the limit can result in a permanent IP address ban. Therefore, the MyGene library was

used to rapidly query for NCBI gene ids which has no limitations on the number of queries. Then BioPython was used to do an NCBI Batch query with the gene ids to download all the gene information for the 55 genes containing differentially used exons into a CSV file. The automated the retrieval of the full gene names and their functions is a time saver and could help reduce the number of genes of interest by searching through the gene information.

Manual verification of differentially used exonic parts. The `exCOVator.py` script finds unannotated exons by looking for sequences in the samples that are not part of the annotation (GFF) file. This method can lead to potential novel discoveries, but also lead to false positives due to sequencing indels, mapping errors, or PCR artifacts during cDNA generation. In addition, even though we have used automated filtering using `s6_filterDiffUsedExons.py' some exons may slip past the filters. In order to differentiate likely novel candidates from potential false positives, we manually cross checked the exonic part data using a combination of observations from the IGV browser, the coverage graphs, and the coverage ratios calculated from the `exCOVator.py` script. Using these tools, exon candidates were selected using the following guidelines: 1) each candidate should have a reasonable amount of reads in at least one of the samples (e.g. >= 30 total consensus read coverage in at least one of the samples), 2) there should be a marked difference in coverage ratios between at least two of the three samples (e.g. >= 20%), 3) multiple reads containing the sequence matched exon should have the same genomic interval (start and end coordinates), and 4) a majority of the reads should not contain insertion or deletion errors at the splice sites/exon boundaries and 5) if it is an unannotated exon, it should be conserved in other species or is in-frame. Using this manual verification step, we found 51/55 genes and 87% (285/327) of exons to be differentially used while 13% (42/327) of exons were artifacts and/or missed by the automated filters (Table 1, supplementary data).

Scripts for this analysis pipeline can be found in the Supplemental Code and Github repository.

www.github.com/puapinyoying/isoseq_manuscript_resources/s2_exCOVator_analysis_pipeline

exPhaser transcript structure analysis pipeline

The exCOVator analysis pipeline is exon-based so it is helpful for identifying unannotated exons and quantifying differential usage of individual exons between tissues. However, it cannot determine exon phasing. The goal of the exPhaser analysis pipeline is to determine exon phasing pattern of multiple key (ideally isoform defining) exons within a transcript and identify and quantify the total transcripts in the given samples. (See ‘exPhaser implementation’ PDF slides in s3_exPhaser_analysis_pipeline of Supplemental Code and Figures 6 and 7).
Generate Boolean reference table to count all possible splice patterns. First, the script takes the user list (BED file) of key exons to phase and calculates the total possible splice patterns using this formula: 2^number of exons. Next, the total possible patterns starting from zero (e.g. 1-8 patterns would be 0-7 patterns), were converted to binary numbers (e.g. 0 = 000, 1=001, 7=111, etc) and split into a table and converted into a Boolean (True/False) table containing presence and absence of all exon combinations.

Calculate interval range to select reads and annotations for analysis. In order to normalize the data for comparisons between each sample, we need to calculate a denominator of total reads (that span the range of exons, matching the sequence or not). Not all the reads that map to a gene of interest will span the list of key exons provided for phasing analysis. In order to filter out reads that do not cover the exons of interest, we calculate an interval range variable which is the start coordinate of the first exon and the end coordinate of the last exon given by the user (pre-sorted by genomic coordinates). The reads that *contain* the interval range are selected for further analysis. The same is done for the given GENCODE annotation (GTF) file to select annotations that contain the interval range.

Annotate and count each splice pattern in the Boolean reference table. Using HT-Seq, each annotation and read that contained the interval range was checked for its splicing pattern. If the splice pattern of the user provided exons matched one or more annotations, then they are annotated in the pattern's row in Boolean table. For each sample read, if the splice pattern found in the cigar string matched the Boolean table, then we would start a running count for it.

Filter and normalize the count data. After the table is filled with count and annotation data, it is filtered to remove splice patterns that have a zero read count or if it is not annotated. Then the data is normalized by dividing the number of reads for each transcript structure/splice pattern by the total full-length read count of all reads that contain the interval range. Finally, the data table is exported for manual analysis.

Scripts for this analysis pipeline can be found in Supplemental Code and Github repository.
www.github.com/puapinyoying/isoseq_manuscript_resources/s3_exPhaser_analysis_pipeline
