# Supplementary material for: A long-read RNA-seq approach to identify novel transcripts of very large genes
Source: Genome Res. 2020 Jun;30(6):885–97. doi: 10.1101/gr.259903.119 (PMC7370890; doi:10.1101/gr.259903.119)
Supplement: Supplemental Material [file supp_gr.259903.119_Supplemental_Code.zip › isoseq_manuscript_resources-master/s0_isoseq_notes/bench_protocols/IsoSeq_bench_protocol.pdf]

Procedure and Checklist - Isoform Sequencing (Iso-Seq™) Using the Clontech® SMARTer® PCR cDNA Synthesis Kit and BluePippin™ Size-Selection System

Before You Begin

The long read lengths of the PacBio® System are well-suited for characterizing full-length transcripts produced from high-quality RNA samples. This document describes methods for generating full-length cDNA libraries (using the BluePippin System) for cDNA sequencing.

Once double-stranded cDNA is prepared, the PacBio Template Prep Kit can be used to generate SMRTbell™ libraries. The SMRTbell templates are then sequenced on the PacBio System.

To perform this procedure, you must have reviewed the *User Bulletin - Guidelines for Preparing cDNA Libraries for Isoform Sequencing (Iso-Seq™)*. Below are the three available procedures for specific project requirements. This procedure is for BluePippin size-selection followed by Iso-Seq cDNA sequencing.

Procedure	Size Selection	Target Size	Required Size Selection Equipment
Procedure & Checklist - Isoform Sequencing Using the Clontech SMARTer PCR cDNA Synthesis Kit with No Size Selection	No	1.0 kb - 1.5 kb<br>Full Length Transcripts	None
Procedure & Checklist - Isoform Sequencing Using the Clontech SMARTer PCR cDNA Synthesis Kit and Manual Agarose-gel Size Selection	Yes	1.0 kb - 6 kb<br>Full Length Transcripts	Agarose Gel Electrophoresis Chamber and Agarose
Procedure & Checklist - Isoform Sequencing Using the Clontech SMARTer PCR cDNA Synthesis Kit and the BluePippin Size-Selection System	Yes	1.0 kb - 10 kb<br>Full Length Transcripts	BluePippin System
Procedure & Checklist - Isoform Sequencing (Iso-Seq™) using the Clontech® SMARTer® PCR cDNA Synthesis Kit and SageELF™ Size Selection System	Yes	1.0 kb - 10 kb<br>Full Length Transcripts	SageELF System

The procedure described in this document is for Iso-Seq cDNA sequencing using BluePippin size selection.

P/N100-377-100-05

Materials and Kits Needed

Item	Vendor
SMARTer PCR cDNA Synthesis Kit	Clontech (634925 or 634926)
KAPA™ HiFi PCR Kit	Kapa Biosystems (KK2101 or KK2102)
Additional 5’ PCR Primer IIA	Any Oligo Synthesis Vendor
1.2% FlashGel® system or<br>0.80% Agarose Gels	Lonza<br>Any MLS
FlashGel DNA Marker (100 bp - 4 kb)	Lonza
Qubit® dsDNA BR Assay Kit	Invitrogen
DNA 7500 Kit	Agilent
BluePippin system with Software v5.90 or later<br>PacBio SMRTbell cassette definition set “0.75% DF 2 – 6kb Marker S1”<br>0.75% Dye-Free Agarose Gel Cassettes<br>Loading Solution<br>S1 Marker<br>Electrophoresis Buffer	Sage Science
Template Prep Kit<br>DNA/Polymerase Binding Kit<br>DNA Sequencing Kit<br>AMPure® PB Beads	Pacific Biosciences

Preparing cDNA from RNA Samples

First-Strand Synthesis

First strand cDNA synthesis employs the Clontech SMARTer PCR cDNA Synthesis Kit. The CDS Primer IIA is first annealed to the polyA+ tail of transcripts, followed by first-strand synthesis with SMARTScribe™ Reverse Transcriptase. The first-strand product is diluted with Elution Buffer (EB) to an appropriate volume and subsequently used for large-scale PCR.

– Before proceeding with the first-strand synthesis, determine if one primer annealing and first-strand reaction is enough to proceed to the Test Amplification and Large-Scale PCR steps (see dilution table on step 7).
– Perform additional annealing and first-strand synthesis reactions, if necessary. If starting with total RNA, we recommend setting up three separate reactions of first-strand synthesis to ensure there is enough diluted first-strand product for the Test Amplification and Large-Scale PCR steps. If starting with polyA+ RNA, one first-strand reaction is sufficient.

1. For each sample and Control Mouse Liver Total RNA, combine the reagents below in separate PCR tubes. For polyA+ RNA, the minimum is 1 ng; total RNA requires 2 ng.

   Do not change the size (volumes) of any of the reactions. All components have been optimized for the volumes specified. If using > 1 µg RNA, split the sample into multiple reactions.

Reagent	Volume	✓	Notes
RNA (1 ng - 1 µg)	1 - 3.5 µL		
3' SMART® CDS Primer II A (12 µM)	1 µL		
Nuclease-Free Water	X		
Total Volume	4.5 µL		

2. Mix contents and spin the tubes briefly in a microcentrifuge.
3. Incubate the tubes at 72°C in a hot-lid thermal cycler for 3 min; slow ramp to 42°C at 0.1°C/sec let sit for 2 minutes.

   During this incubations step, prepare a Master Mix for all reaction tubes, at room temperature, by combining the following reagents in the order shown. It is important to go immediately into step 4 after step 3. However, add the reverse transcriptase to the master mix just prior to use. Mix well by pipetting and spin the tube briefly in a microcentrifuge.

Reagent	Volume	✓	Notes
5X First-Strand Buffer	2 µL		
DTT (100 mM)	0.25 µL		
dNTP (10 mM)	1 µL		
SMARTer II A Oligonucleotide (12 µM)	1 µL		
RNase Inhibitor	0.25 µL		
SMARTScribe Reverse Transcriptase (100 U) - add before use	1 µL		
Total Volume added per reaction	5.5 µL		

Place the master mix at 42°C for 1 min to bring it up to temperature and proceed immediately to step 4.

4. Aliquot 5.5 µL of the Master Mix into each reaction tube. Mix the contents of the tubes by gently pipetting, and spin the tubes briefly to collect the contents at the bottom.
5. Incubate the tubes at 42°C for 90 minutes.
6. Terminate the reaction by heating the tubes at 70°C for 10 min.
7. Dilute the first-strand reaction product by adding the appropriate volume of PacBio Elution Buffer (EB):

Input Sample	Volume of EB added
Total RNA (2 ng - 1 µg)	40 µL
PolyA+ RNA, > 0.2 µg	190 µL
PolyA+ RNA, < 0.2 µg	90 µL

8. If multiple reactions were performed with the same RNA samples, pool the diluted first-strand reactions together before the amplification steps.

Large-Scale PCR

It is highly recommended to perform cycle optimization to determine the optimal number of cycles (while minimizing artifacts during large-scale amplification) for large-scale PCR.

PCR Cycle Optimization

Test Amplification

In this section, perform test amplifications to determine the best number of cycles required for the sample. Collect a total of 5 x 5 µl aliquots from each recommended cycle below.

1. Add the following reagents to an appropriately sized PCR tube:

Reagent	Volume	✓	Notes
KAPA HiFi Fidelity Buffer (5X)	10 µL		
Diluted first-strand cDNA from step 7 above	10 µL		
KAPA dNTP Mix (10 mM)	1.5 µL		
5’ PCR Primer II A (12 µM)	3.2 µL		
Nuclease-free water	24.3 µL		
KAPA HiFi Enzyme (1 U/µL)	1 µL		
Total Volume	50 µL		

2. Cycle the reaction with the following conditions (using a heated lid):
   - Initial denaturation:
     - 95°C for 2 minutes
   - 10 cycles at the following temperatures and times:
     - 98°C for 20 seconds
     - 65°C for 15 seconds
     - 72°C for 4 minutes
   - Final extension:
     - 72°C for 5 minutes
3. After the initial **10** cycles, remove 5 µL of the reaction and transfer it to a tube labeled “10.”
4. Return the remaining 45 µL PCR reaction to the thermocycler and run two cycles of the above amplification conditions.
   - 2 cycles at the following temperatures and times:
     - 98°C for 20 seconds
     - 65°C for 15 seconds
     - 72°C, for 4 minutes
   - Final extension:
     - 72°C for 5 minutes
5. Remove 5 µL again and transfer to a tube labeled “12.”
6. Repeat steps 4-5 for 14, 16, and 18 cycles.

Note that the number of cycles is dependent on the sample, and may be changed for particular samples. Therefore, it may be necessary to adjust the cycle PCR optimization starts.

7. Load the 5 aliquots on an Agarose gel or Bioanalyzer® instrument to view distribution of the ds cDNA. See *UB - Guidelines for Preparing cDNA Libraries for Isoform Sequencing (Iso-Seq™)*.

Large-Scale PCR for Size Selection on the BluePippin™ System

Use the cycle number (as determined in the PCR Cycle Optimization step) to generate double-stranded cDNA for size selection on the BluePippin System.

1. Set up 8 X 50 µL PCR reactions.
2. Make a master mix by adding the following reagents:

Reagent	Volume	✓	Notes
KAPA HiFi Fidelity Buffer (5X)	80 µL		
Diluted first-strand cDNA Synthesis	80 µL		
KAPA dNTP Mix (10 mM)	12 µL		
5' PCR Primer II A (12 µM)	25.6 µL		
Nuclease-free water	194.4 µL		
KAPA HiFi Enzyme (1U/µL)	8 µL		
Total Volume	400 µL		

3. Transfer 50 µL aliquots into 8 PCR tubes and perform PCR using the cycle number determined during the optimization step. Cycle the reaction with the following conditions (using a heated lid):
   - Initial denaturation:
     - 95°C for 2 minutes
   - ***n*** cycles (optimal cycle determined in the optimization step) at the following temperatures and times:
     - 98°C for 20 seconds
     - 65°C for 15 seconds
     - 72°C for 4 minutes
   - Final extension:
     - 72°C for 5 minutes

4. Pool the eight PCR reactions together and perform a 1X AMPure PB bead purification step.

Purifying the Large-Scale PCR Products

STEP		Purify the Pooled PCR Products	Notes
1		Add **1X** volume of AMPure PB magnetic beads.	
2		Mix the bead/DNA solution thoroughly.	
3		Quickly spin down the tube (for 1 second) to collect the beads. Do not pellet beads.	
4		Allow the DNA to bind to beads by shaking in a VWR® vortex mixer at 2000 rpm for 10 minutes at room temperature.	
5		Spin down the tube (for 1 second) to collect beads.	
6		Place the tube in a magnetic bead rack until the beads collect to the side of the tube.	
7		With the tube still on the magnetic bead rack, slowly pipette off cleared supernatant and save in another tube. Avoid disturbing the bead pellet.	
8		Wash beads with freshly prepared 70% ethanol.	
9		Repeat step 8 above for a total of 2 ethanol washes.	
10		Remove residual 70% ethanol and dry the bead pellet.<br>– Remove tube from magnetic bead rack and spin to pellet beads. Both the beads and any residual 70% ethanol will be at the bottom of the tube.<br>– Place the tube back on magnetic bead rack.<br>– Pipette off any remaining 70% ethanol.	
11		Check for any remaining droplets in the tube. If droplets are present, repeat step 10.	
12		Remove the tube from the magnetic bead rack and allow beads to air-dry (with the tube caps open) for 30 to 60 seconds.	
13		Elute the DNA off the beads in **40 µL** of Elution Buffer. Vortex for 10 minutes at 2000 rpm.	
14		Determine concentration using a Qubit system or another double-strand-specific quantitation system.	
15		Prepare 4 tubes of PCR product with **30 µL** of **500 ng** for size selection with the BluePippin System.	

Size Selection using the BluePippin™ System

STEP		Running the BluePippin System	Notes
**1**		Follow the BluePippin Manual and instructions to calibrate your instrument.<br>– A new calibration s recommended before each BluePippin run.	
**2**		Inspect the gel cassette (using Sage Sciences' BluePippin manual).<br>– Ensure that the buffer wells are full.<br>– Ensure that there is no separation of the gel from the cassette.	
**3**		Prepare the gel cassette:<br>– Remove all bubbles from the elution buffer chamber by tilting the cassette and tapping it until all air bubbles move into the buffer chamber.<br>– Place the gel cassette in the BluePippin System and carefully remove the plastic seals on the cassette.<br>– Remove the buffer from the elution well and fill with **40 µL** of fresh Electrophoresis Buffer.<br>• Keep the pipette down the center of the well and avoid creating a vacuum in the well.<br>• The bottom of the well is okay to touch.<br>• If the well "bubbles" over when adding the buffer to the well, remove buffer and try again. If the well continues to "bubble" over, then use this well for the S1 marker. The elution well may be damaged and should not be used for sample collection.<br>– Cover the elution wells with a clear adhesive tape<br>– Remove the buffer from the sample well and fill with **70 µL** of fresh Electrophoresis Buffer<br>– Close the lid and perform a Continuity Test.	
**4**		Prepare samples for loading<br>– For fractions 1 kb - 2 kb, 2 kb - 3 kb, and 3 kb - 6 kb, prepare aliquots of **500 ng** in **30 µL**.<br>– For fractions 5 - 10 kb, >**1.0 µg** (up to **5 µg**) is required.<br>– Use Elution Buffer to dilute sample to **30 µL**.<br>– Add **10 µL** of the Loading Solution and vortex to mix well.	
**5**		Load samples:<br>– Remove **40 µL** of buffer from each well.<br>– Load all **40 µL** of the sample prepared in step 4 into each lane.<br>– Load **40 µL** of S1 Marker in one of the lanes.	

STEP		Running the BluePippin System	Notes
**6**		Set up the run Protocol:<br>– Click on the “New” button to create a new protocol.<br>– Select the “0.75% DF 2 – 6kb Marker S1” cassette definition file.<br>– Click on the box below “End Run when Elution is Complete.”<br>– Set the lane where the S1 Marker is loaded as the reference “Ref” lane.<br>– Click on the “Range” button and enter the following:<br><br>Library Size / BP Start / BP End<br>1 kb - 2 kb / 500 / 2000<br>2 kb - 3 kb / 1500 / 3000<br>3 kb - 6 kb / 2500 / 6000<br>5 kb - 10 kb / 4500 / 10000	
**7**		Start the run.	
**8**		After the run, collect approximately **40 µL** the respective fractions from each lane.	
**9**		The samples can be stored in - 20°C or used directly in the next step, large-scale PCR. Note that quantification is not necessary at this point. If desired, perform 1X AMPure PB bead purification.	

Large-Scale PCR for SMRTbell™ Library Preparation

After size selection, the double-stranded cDNA is not sufficient for SMRTbell library construction. Perform large-scale PCR using the eluted DNA from the previous step to generate more double-stranded cDNA..

PCR for Each Fraction

1. Set up 8 X 50 µL PCR reactions for each size fraction: 1 kb – 2 kb, 2 kb – 3 kb, 3 kb – 6 kb, and 5 kb – 10 kb.
2. Add the following reagents to an appropriately sized PCR tube:

Reagent	Volume	✓	Notes
KAPA HiFi Fidelity Buffer (5X)	80 µL		
Eluted DNA from the BluePippin System	20 µL		
KAPA dNTP Mix (10 mM)	12 µL		
5' PCR Primer II A (12 µM)	25.6 µL		
Nuclease-free water	254.4 µL		
KAPA HiFi Enzyme (1U/µL)	8 µL		
Total Volume	400 µL		

3. Aliquot 50 µL into 8 PCR tubes and perform PCR using the cycle number and extension parameters below.

Size Desired	Extension Time	Number of Cycles
1 kb - 2 kb	1 min	8 -12 cycles
2 kb - 3 kb	1 min 45 sec	10 - 12 cycles
3 kb - 6 kb	3 min 00 sec	12 - 15 cycles
5 kb - 10 kb	4 min 30 sec	15 cycles

4. Cycle the reaction with the following conditions (using a heated lid):
   - Initial denaturation:
     – 95°C for 2 minutes
   - ***n*** cycles at the following temperatures and times:
     – 98°C for 20 seconds
     – 65°C for 15 seconds
     – 72°C for 4 minutes (for this step, see extension times in table above)
   - Final extension:
     – 72°C for 5 minutes

Purifying the Large-Scale PCR Products

After PCR, pool the reactions for each size fraction into a 1.5 mL tube and purify with 1X AMPure PB beads. Note that it is critical to use the AMPure PB beads since these are specially formulated for compatibility with PacBio template preparation and sequencing chemistry.

STEP		Pooling and Purification	Notes
1		Add 1X volume of AMPure PB magnetic beads to the amplified cDNA sample.	
2		Mix the bead/DNA solution thoroughly.	
3		Quickly spin down the tube (for 1 second) to collect the beads. Do not pellet beads.	
4		Allow the DNA to bind to beads by shaking in a VWR vortex mixer at 2000 rpm for 10 minutes at room temperature.	
5		Spin down the tube (for 1 second) to collect beads.	
6		Place the tube in a magnetic bead rack until the beads collect to the side of the tube.	
7		With the tube still on the magnetic bead rack, slowly pipette off cleared supernatant and save in another tube. Avoid disturbing the bead pellet.	
8		Wash beads with freshly prepared 70% ethanol.	
9		Repeat step 8 above for a total of 2 ethanol washes.	
10		Remove residual 70% ethanol and dry the bead pellet.<br>– Remove tube from magnetic bead rack and spin to pellet beads. Both the beads and any residual 70% ethanol will be at the bottom of the tube.<br>– Place the tube back on magnetic bead rack.<br>– Pipette off any remaining 70% ethanol.	
11		Check for any remaining droplets in the tube. If droplets are present, repeat step 10.	
12		Remove the tube from the magnetic bead rack and allow beads to air-dry (with the tube caps open) for 30 to 60 seconds.	
13		Elute the DNA off the beads in Elution Buffer. We recommend eluting in volume, 1/10th of the initial PCR Master Mix volume (e.g., for 400 µL of PCR, 40 µL of Elution Buffer). Mix sample until homogeneous, then vortex for 10 minutes at 2000 rpm.	
14		Spin down the tube (for 1 second) to collect beads.	
15		Place the tube in a magnetic bead rack to collect the beads to the side of the tube. Carefully pipette eluted DNA into a LoBind microcentrifuge tube.	
16		Check concentration of samples.	

cDNA SMRTbell™ Template Preparation and Sequencing

Repair DNA Damage

In general, 1 – 1.5 µg of cDNA is recommended for each sample going into SMRTbell template preparation. For size-selected samples, the input amount depends on the size:

If preparing larger amounts of DNA than what the table below indicates, scale the reaction volumes accordingly.

Fraction	Input Requirement
1 kb - 2 kb	Up to 500 ng
2 kb - 3 kb	Up to 1 µg
3 kb - 6 kb	Up to 1.5 µg
5 kb - 10 kb	Up to 1.5 µg

1. In a LoBind microcentrifuge tube, add the following reagents:
   Note that short insert SMRTbell templates may be present in the 3 kb – 6 kb and 5 kb -10 kb fractions, and will load preferentially during sequencing. Their removal may be necessary if long full-length transcripts are desired. This can be achieved by running the SMRTbell libraries on a BluePippin system again. If this is desired, we recommend preparing enough SMRTbell templates for both fractions by doubling the reaction volumes and input mass (3 µg).

Reagent	Tube Cap Color	Stock Conc.	Volume	Final Conc.	✓	Notes
Amplified ds cDNA	–		__ µL for 0.5 to 1.5 µg	–		
DNA Damage Repair Buffer	🔴	10 X	5.0 µL	1 X		
NAD+	🔴	100 X	0.5 µL	1 X		
ATP high	🔴	10 mM	5.0 µL	1 mM		
dNTP	🔴	10 mM	0.5 µL	0.1 mM		
DNA Damage Repair Mix	🔴		2.0 µL			
H_2O	–		__ µL to adjust to 50.0 µL	–		
Total Volume			50.0 µL	–		

2. Mix the reaction well by pipetting or flicking the tube.
3. Spin down contents of tube with a quick spin in a microfuge.
4. Incubate at 37ºC for 20 minutes, then return the reaction to 4ºC for 1 minute.

Repair Ends

Use the following table to prepare your reaction then purify the DNA.

Reagent	Tube Cap Color	Stock Conc.	Volume	Final Conc.	✓	Notes
DNA (Damage Repaired)	–		50.0 µL	–		
End Repair Mix	🔴	20 X	2.5 µL	1X		
Total Volume			52.5 µL	–		

1. Mix the reaction well by pipetting or flicking the tube.
2. Spin down contents of tube with a quick spin in a microfuge.
3. Incubate at 25ºC for 5 minutes, return the reaction to 4ºC.

STEP	✓	Purify DNA	Notes
1		For all insert sizes, add **1X** volume of AMPure PB beads.	
2		Mix the bead/DNA solution thoroughly.	
3		Quickly spin down the tube (for 1 second) to collect the beads. Do not pellet beads.	
4		Allow the DNA to bind to beads by shaking in a VWR vortex mixer at 2000 rpm for 10 minutes at room temperature.	
5		Spin down the tube (for 1 second) to collect beads.	
6		Place the tube in a magnetic bead rack to collect the beads to the side of the tube.	
7		Slowly pipette off cleared supernatant and save (in another tube). Avoid disturbing the bead pellet.	
8		Wash beads with freshly prepared 70% ethanol.	
9		Repeat step 8 above.	
10		Remove residual 70% ethanol and dry the bead pellet.<br>– Remove tube from magnetic bead rack and spin to pellet beads. Both the beads and any residual 70% ethanol will be at the bottom of the tube.<br>– Place the tube back on magnetic bead rack.<br>– Pipette off any remaining 70% ethanol.	
11		Check for any remaining droplets in the tube. If droplets are present, repeat step 10.	
12		Remove the tube from the magnetic bead rack and allow beads to air-dry (with tube caps open) for 30 to 60 seconds.	
13		Elute the DNA off the beads in **30 µL** Elution Buffer. Mix until homogenous, then vortex for 10 minutes at 2000 rpm.	
14		Optional: Verify your DNA amount and concentration using a Nanodrop or Qubit quantitation platform, as appropriate.	
15		Optional: Perform qualitative and quantitative analysis using a Bioanalyzer instrument with the DNA 7500 Kit. Note that typical yield at this point of the process (following End-Repair and one **1X** AMPure PB bead purification) is approximately between **80-100%** of the total starting material.	
16		The End-Repaired DNA can be stored overnight at 4ºC or (or -20ºC for longer).	
17		Actual recovery per µL and total available sample material: ___________________	

Prepare Blunt Ligation Reaction

Note: It is important to maintain an optimal ratio of adapter to insert molecules. If a larger amount of library is being prepared than what is indicated in the table below, scale the ligation reaction volumes appropriately.

Fraction	Input Requirement
1 kb - 2 kb	Up to 500 ng
2 kb - 3 kb	Up to 1 µg
3 kb - 6 kb	Up to 1.5 µg
5 kb - 10 kb	Up to 1.5 µg

Use the following table to prepare your blunt ligation reaction:

1. In a LoBind microcentrifuge tube (on ice), add the following reagents in the order shown. If preparing a Master Mix, ensure that the adapter is NOT mixed with the ligase prior to introduction of the inserts. Add the adapter to the well with the DNA. All other components, including the ligase, should be added to the Master Mix.

Reagent	Tube Cap Color	Stock Conc.	Volume	Final Conc.	✓	Notes
ds cDNA (End Repaired)	–		29.0 µL to 30.0 µL			
**Annealed** Blunt Adapter (20 µM)		20 µM	2.0 µL	1 µM		
Mix before proceeding						
Template Prep Buffer		10 X	4.0 µL	1X		
ATP low		1 mM	2.0 µL	0.05 mM		
Mix before proceeding						
Ligase		30 U/µL	1.0 µL	0.75 U/µL		
H_2O	–	–	__ µL to adjust to 40.0 µL	–		
Total Volume	–	–	40.0 µL	–		

2. Mix the reaction well by pipetting or flicking the tube.
3. Spin down contents of tube with a quick spin in a microfuge.
4. Incubate at 25ºC for 15 minutes. At this point, the ligation can be extended up to 24 hours or cooled to 4ºC (for storage up to 24 hours).
5. Incubate at 65ºC for 10 minutes to inactivate the ligase, then return the reaction to 4ºC. You must proceed with adding exonuclease after this step.

Add exonuclease to remove failed ligation products:

Reagent	Tube Cap Color	Stock Conc.	✓	Volume
Ligated DNA				40 µL
Mix reaction well by pipetting				
ExoIII	● (green)	100.0 U/µL		1.0 µL
ExoVII	● (green)	10.0 U/µL		1.0 µL
Total Volume				42 µL

1. Mix the reaction well by pipetting or flicking the tube.
2. Spin down contents of tube with a quick spin in a microfuge.
3. Incubate at 37ºC for 1 hour, then return the reaction to 4ºC. You must proceed with purification after this step.

Purify SMRTbell™ Templates

STEP	✓	Purify SMRTbell Templates - First Purification	Notes
1		For all insert sizes, add **1X** volume of AMPure PB beads.	
2		Mix the bead/DNA solution thoroughly.	
3		Quickly spin down the tube (for 1 second) to collect the beads. Do not pellet beads.	
4		Allow the DNA to bind to beads by shaking in a VWR vortex mixer at 2000 rpm for 10 minutes at room temperature.	
5		Spin down the tube (for 1 second) to collect beads.	
6		Place the tube in a magnetic bead rack to collect the beads to the side of the tube.	
7		Slowly pipette off cleared supernatant and save (in another tube). Avoid disturbing the bead pellet.	
8		Wash beads with freshly prepared 70% ethanol.	
9		Repeat step 8 above.	
10		Remove residual 70% ethanol and dry the bead pellet.<br>– Remove tube from magnetic bead rack and spin to pellet beads. Both the beads and any residual 70% ethanol will be at the bottom of the tube.<br>– Place the tube back on magnetic bead rack.<br>– Pipette off any remaining 70% ethanol.	
11		Check for any remaining droplets in the tube. If droplets are present, repeat step 10.	
12		Remove the tube from the magnetic bead rack and allow beads to air-dry (with tube caps open) for 30 to 60 seconds.	
13		Elute the DNA off the beads in **50 µL** of Elution Buffer. Vortex for 10 minute at 2000 rpm.	
14		The eluted DNA in **50 µL** Elution Buffer should be taken into the second and final AMPure bead purification step.	

STEP	✓	Purify SMRTbell Templates - Second Purification	Notes
**1**		For all insert sizes, add **1X** volume of AMPure PB beads.	
**2**		Mix the bead/DNA solution thoroughly.	
**3**		Quickly spin down the tube (for 1 second) to collect the beads. Do not pellet beads.	
**4**		Allow the DNA to bind to beads by shaking in a VWR vortex mixer at 2000 rpm for 10 minutes at room temperature.	
**5**		Spin down the tube (for 1 second) to collect beads.	
**6**		Place the tube in a magnetic bead rack to collect the beads to the side of the tube.	
**7**		Slowly pipette off cleared supernatant and save (in another tube). Avoid disturbing the bead pellet.	
**8**		Wash beads with freshly prepared 70% ethanol.	
**9**		Repeat step 8 above.	
**10**		Remove residual 70% ethanol and dry the bead pellet.<br>– Remove tube from magnetic bead rack and spin to pellet beads. Both the beads and any residual 70% ethanol will be at the bottom of the tube.<br>– Place the tube back on magnetic bead rack.<br>– Pipette off any remaining 70% ethanol.	
**11**		Check for any remaining droplets in the tube. If droplets are present, repeat step 10.	
**12**		Remove the tube from the magnetic bead rack and allow beads to air-dry (with tube caps open) for 30 to 60 seconds.	
**13**		Elute the DNA off the beads in **10 µL** of Elution Buffer. Vortex for 10 minute at 2000 rpm.	
**14**		Verify your DNA amount and concentration with either a Nanodrop or Qubit quantitation platform reading. For general library yield expect 20% total yield from the Damage Repair input. If your yield concentration is below 12 ng/µL, use the Qubit system for quantitation.<br><br>To estimate your final concentration:<br>(____ng of DNA going into Damage Repair X 0.2) / of Elution Buffer = ng/µL	
**15**		Perform qualitative and quantitative analysis using a Bioanalyzer instrument. Note that typical DNA yield, at this point of the process, following blunt ligation, exonuclease treatment and two AMPure PB bead purifications is between approximately **15-25%** of the total starting material going into the ligation reaction.	

Optional Second BluePippin™ Size Selection for the 3 kb - 6 kb and 5 kb - 10 kb SMRTbell™ Library to Remove Contaminating Short SMRTbell Templates (< 2 kb)

This is an optional BluePippin size selection for the 3 kb – 6 kb and 5 kb - 10 kb SMRTbell libraries. This additional step further removes short contaminating SMRTbell templates that preferentially load during sequencing.

After size selection, one round of 1X AMPure PB bead purification is sufficient.

STEP		Running the BluePippin System	Notes
**1**		Follow the BluePippin Manual and instructions to calibrate your instrument.<br>– A new calibration s recommended before each BluePippin run.	
**2**		Inspect the gel cassette (using Sage Sciences' BluePippin manual).<br>– Ensure that the buffer wells are full.<br>– Ensure that there is no separation of the gel from the cassette.	
**3**		Prepare the gel cassette:<br>– Remove all bubbles from the elution buffer chamber by tilting the cassette and tapping it until all the air bubbles move into the buffer chamber.<br>– Place the gel cassette in the BluePippin System and carefully remove the plastic seals on the cassette.<br>– Remove the buffer from the elution well and fill with **40 µL** of fresh Electrophoresis Buffer.<br>• Keep the pipette down the center of the well and avoid creating a vacuum in the well.<br>• The bottom of the well is okay to touch.<br>• If the well "bubbles" over when adding the buffer to the well, remove buffer and try again. If the well continues to "bubble" over, then use this well for the S1 marker. The elution well may be damaged and should not be used for sample collection.<br>– Cover the elution wells with a clear adhesive tape<br>– Remove the buffer from the sample well and fill with **70 µL** of fresh Electrophoresis Buffer<br>– Close the lid and perform a Continuity Test.	
**4**		Prepare samples for loading<br>– Using Elution Buffer , bring the 3 kb - 6 kb (and 5 kb - 10 kb) SMRTbell templates up to **30 µL**.<br>– Add **10 µL** of the Loading Solution and vortex to mix well.	
**5**		Load samples:<br>– Remove **40 µL** of buffer from each well.<br>– Load all **40 µL** of the sample prepared in step 4 into each lane.<br>– Load **40 µL** of S1 Marker in one of the lanes.	

STEP		Running the BluePippin System	Notes
**6**		Setting up the run Protocol:<br>– Click on the “New” button to create a new protocol.<br>– Select the “0.75% DF 2 – 6 kb Marker S1” cassette definition file.<br>– Click on the box below “End Run when Elution is Complete.”<br>– Set the lane where the S1 Marker is loaded as the reference “Ref” lane.<br>– Click on the “Range” button and enter the following:<br><br>Library Size / BP Start / BP End<br>3 kb - 6 kb / 2500 / 6000<br>5 kb - 10 kb / 4500 / 10000	
**7**		Start the run (takes approximately 2.5 hours)	
**8**		Collect samples from the elution wells and perform a final **1X** AMPure PB bead purification.	

STEP	✓	Purify SMRTbell Templates - Final Purification	Notes
1		For all insert sizes, add **1X** volume of AMPure PB beads.	
2		Mix the bead/DNA solution thoroughly.	
3		Quickly spin down the tube (for 1 second) to collect the beads. Do not pellet beads.	
4		Allow the DNA to bind to beads by shaking in a VWR vortex mixer at 2000 rpm for 10 minutes at room temperature.	
5		Spin down the tube (for 1 second) to collect beads.	
6		Place the tube in a magnetic bead rack to collect the beads to the side of the tube.	
7		Slowly pipette off cleared supernatant and save (in another tube). Avoid disturbing the bead pellet.	
8		Wash beads with freshly prepared 70% ethanol.	
9		Repeat step 8 above for a total of 2 ethanol washes.	
10		Remove residual 70% ethanol and dry the bead pellet.<br>– Remove tube from magnetic bead rack and spin to pellet beads. Both the beads and any residual 70% ethanol will be at the bottom of the tube.<br>– Place the tube back on magnetic bead rack.<br>– Pipette off any remaining 70% ethanol.	
11		Check for any remaining droplets in the tube. If droplets are present, repeat step 10.	
12		Remove the tube from the magnetic bead rack and allow beads to air-dry (with tube caps open) for 30 to 60 seconds.	
13		Elute the DNA off the beads in **10 µL** of Elution Buffer. Vortex for 10 minute at 2000 rpm.	
14		Verify your DNA amount and concentration with either a Nanodrop or Qubit quantitation platform reading. For general library yield expect 20% total yield from the Damage Repair input. If your yield concentration is below 12 ng/µL, use the Qubit system for quantitation.<br>To estimate your final concentration:<br>(____ng of DNA going into Damage Repair X 0.2) / of Elution Buffer = ng/µL	
15		Perform qualitative analysis on all three SMRTbell libraries (3 fractions) using a Bioanalyzer instrument. Run 20 ng on a 7500 DNA Chip. Use the average size as determined by the region function for the Binding Calculator. Typical yield is approximately **15-30%**.	

Anneal and Bind BluePippin™ Size-Selected SMRTbell™ Templates

To anneal sequencing primer and bind polymerase to SMRTbell templates, see the Calculator. For more information about using the calculator, see the *Pacific Biosciences Template Preparation and Sequencing Guide*.

Sequence

MagBead loading is suggested for all three fractions, or for non-size selected libraries. We recommend performing loading titrations to determine an appropriate loading concentration.

The Calculator provides recommended sample concentrations for binding polymerase/template complexes to MagBeads, and for loading complexes on PacBio systems. For information on how to prepare and sequence using MagBeads, see the *Pacific Biosciences Procedure & Checklist - Preparing MagBeads for Sequencing.*

For Research Use Only. Not for use in diagnostic procedures. © Copyright 2014-2015, Pacific Biosciences of California, Inc. All rights reserved. Information in this document is subject to change without notice. Pacific Biosciences assumes no responsibility for any errors or omissions in this document. Certain notices, terms, conditions and/or use restric- tions may pertain to your use of Pacific Biosciences products and/or third party products. Please refer to the applicable Pacific Biosciences Terms and Conditions of Sale and to the applicable license terms at http://www.pacificbiosciences.com/licenses.html. Pacific Biosciences, the Pacific Biosciences logo, PacBio, SMRT, SMRTbell and Iso-Seq are trademarks of Pacific Biosciences. BluePippin and SageELF are trademarks of Sage Science, Inc. NGS-go and NGSengine are trademarks of GenDx. All other trademarks are the sole property of their respective owners.
