# Supplementary material for: A long-read RNA-seq approach to identify novel transcripts of very large genes
Source: Genome Res. 2020 Jun;30(6):885–97. doi: 10.1101/gr.259903.119 (PMC7370890; doi:10.1101/gr.259903.119)
Supplement: Supplemental Material [file supp_gr.259903.119_Supplemental_Code.zip › isoseq_manuscript_resources-master/s2_exCOVator_analysis_pipeline/exCOVator_implementation.pdf]

exCOVator.py (See manuscript supplementary data for more detailed implementation notes)

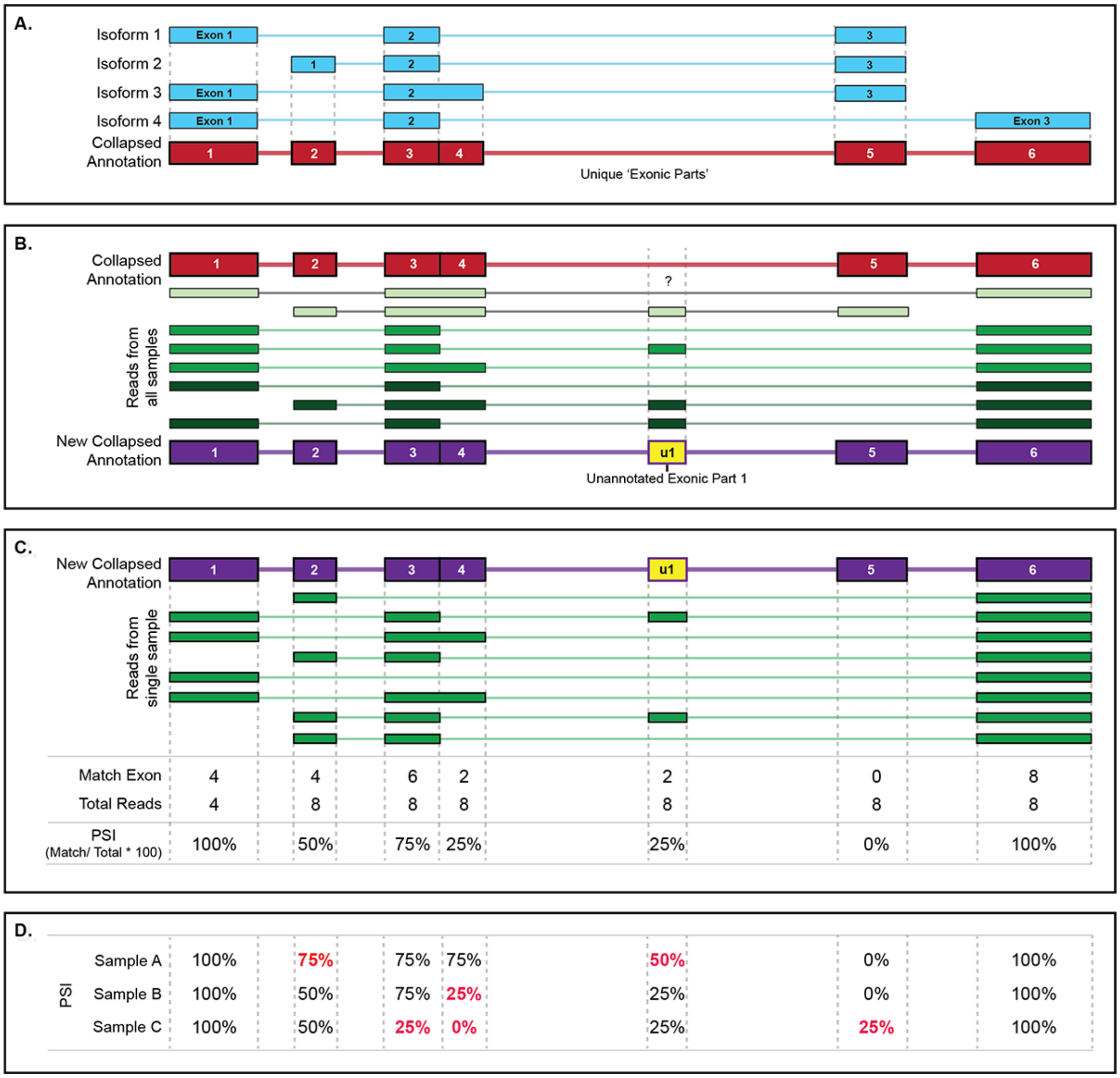

Custom exon-based IsoSeq analysis pipeline (exCOVater). A) Transcript isoforms often share multiple exons complicating analysis. Using the 'dexseq_prepare_annotation.py' script, annotations were collapsed into unique exonic parts (EP) for analysis. B) Read data from all samples are used to find unannotated sequences (potential novel exons) not present in the collapsed annotation file. Unannotated exons (e.g. u1) are added to the list of annotations for differential usage analysis. C) Count match and total reads for each all EPs for individual samples and calculate a ratio (match/total) that is multiplied by 100 to obtain the percent spliced-in (PSI). In this illustration, EP6 is constitutively expressed; EP1 and EP2 are alternate 5' exons; EP5 is an alternate 3' exon that is not expressed in this sample; EP3, EP4 and unannotated exonic part 1 (u1) are cassette exons. D) Observing differential exon usage across samples. The ratio is a normalized value that can be used to compare exon usage across multiple samples. The ratios for each sample are used by 'filterDiffUsedExons.py' for further filtering exons with less than defined % difference across samples. EP1 and EP6 have 0% change across samples and would be filtered out. EP2-5 and u1 are differentially used and will be retained for further analysis.
