# Supplementary material for: A long-read RNA-seq approach to identify novel transcripts of very large genes
Source: Genome Res. 2020 Jun;30(6):885–97. doi: 10.1101/gr.259903.119 (PMC7370890; doi:10.1101/gr.259903.119)
Supplement: Supplemental Material [file 686_genes_exon_ratios.pdf › Supplemental_Data/exCOVator_results/686_genes_exon_ratios.pdf]

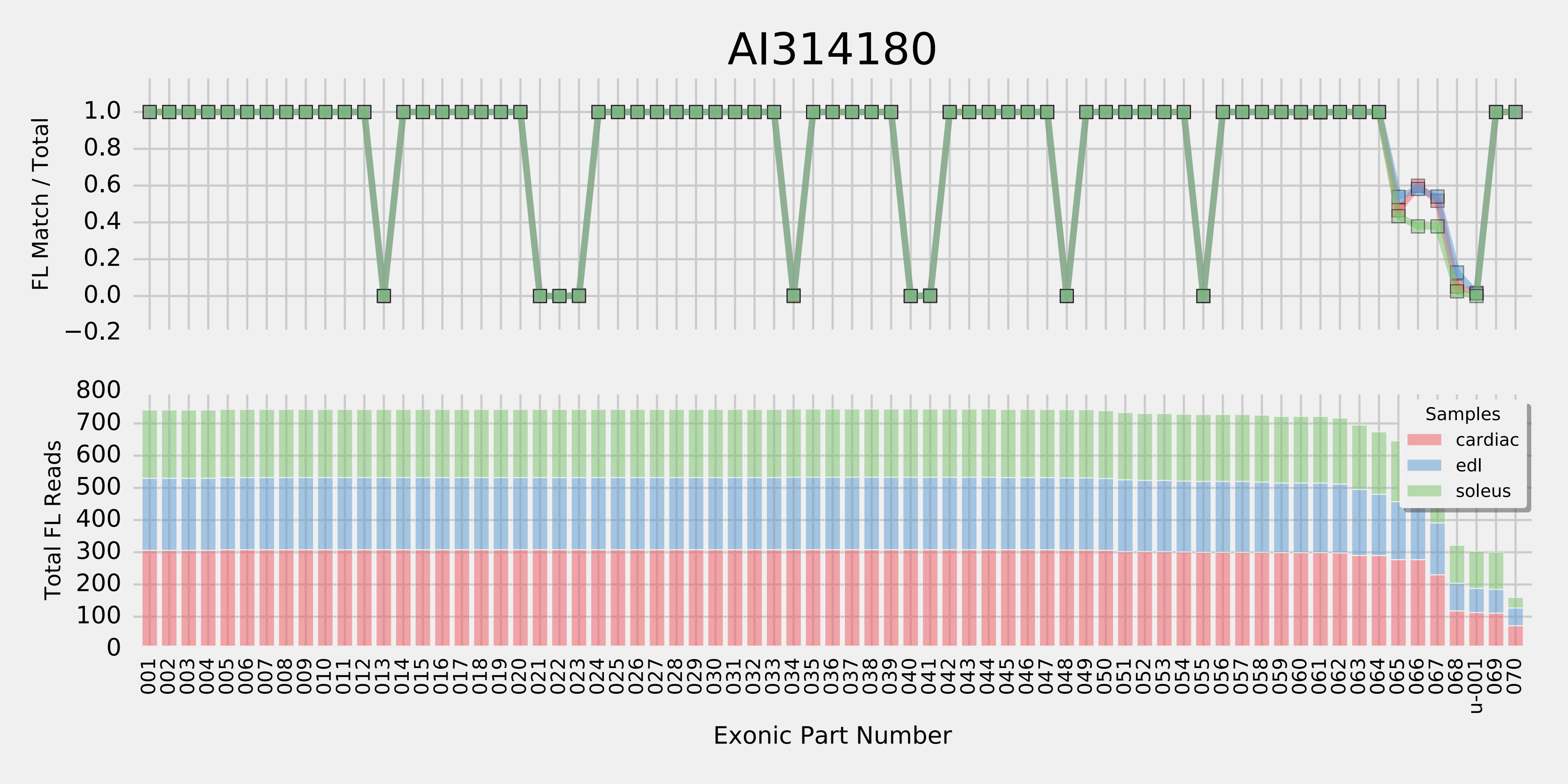

AI314180
FL Match / Total
Total FL Reads
Exonic Part Number
Samples
cardiac
edl
soleus

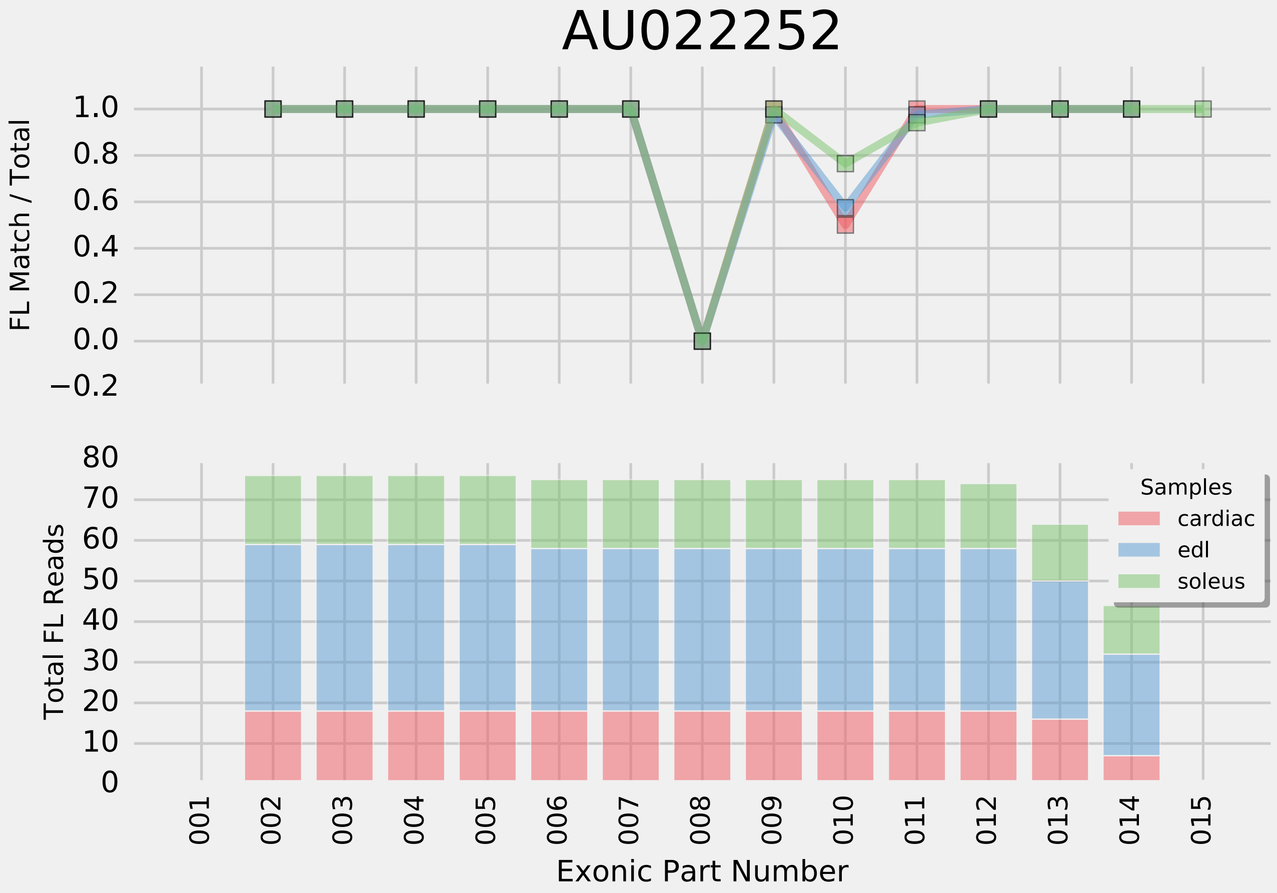
AU022252
FL Match / Total
1.0
0.8
0.6
0.4
0.2
0.0
−0.2
Total FL Reads
80
70
60
50
40
30
20
10
0
Samples
cardiac
edl
soleus
001
002
003
004
005
006
007
008
009
010
011
012
013
014
015
Exonic Part Number

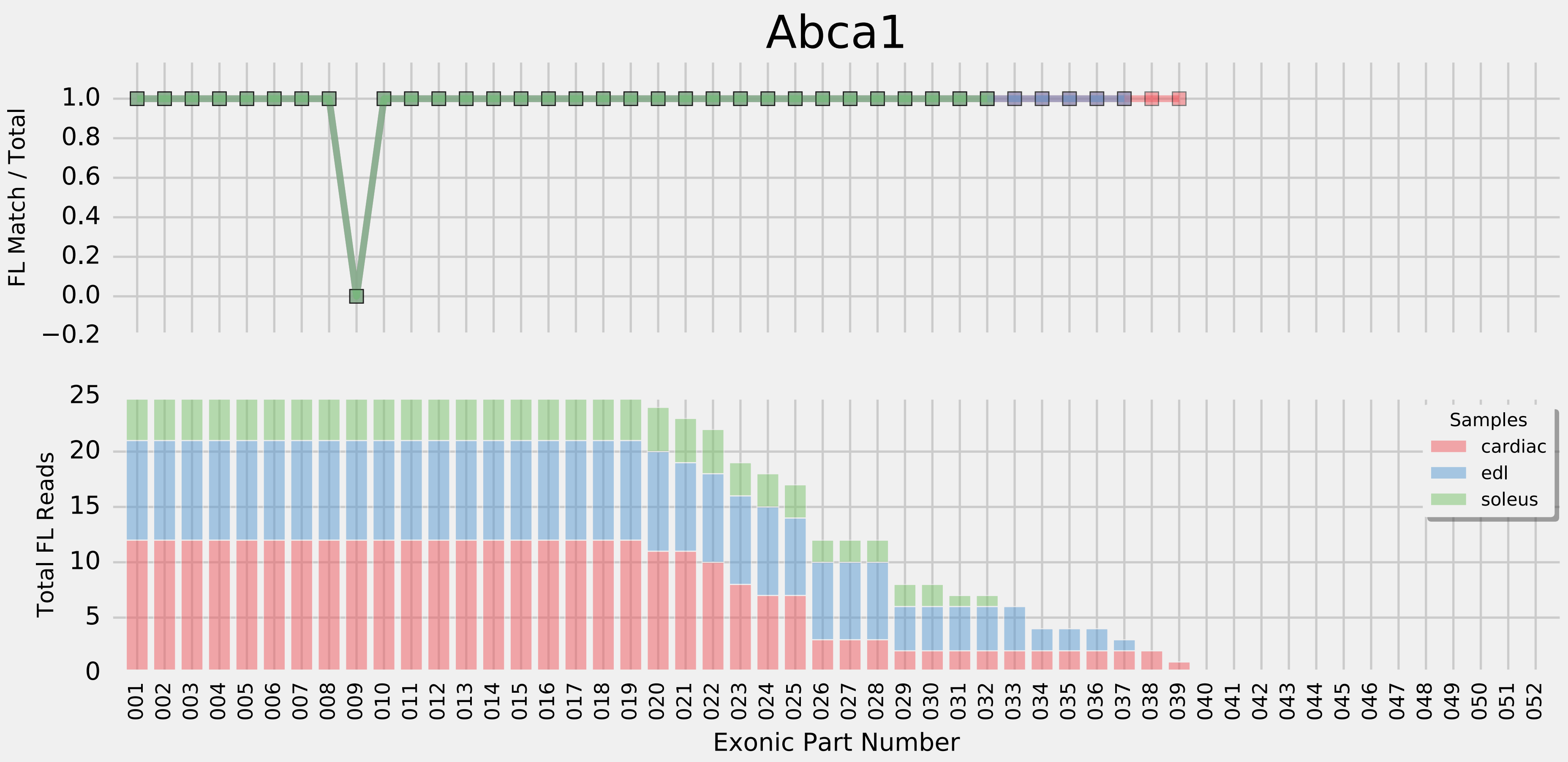

Abca1
FL Match / Total
1.0
0.8
0.6
0.4
0.2
0.0
−0.2
Total FL Reads
25
20
15
10
5
0
Samples
cardiac
edl
soleus
001 002 003 004 005 006 007 008 009 010 011 012 013 014 015 016 017 018 019 020 021 022 023 024 025 026 027 028 029 030 031 032 033 034 035 036 037 038 039 040 041 042 043 044 045 046 047 048 049 050 051 052
Exonic Part Number

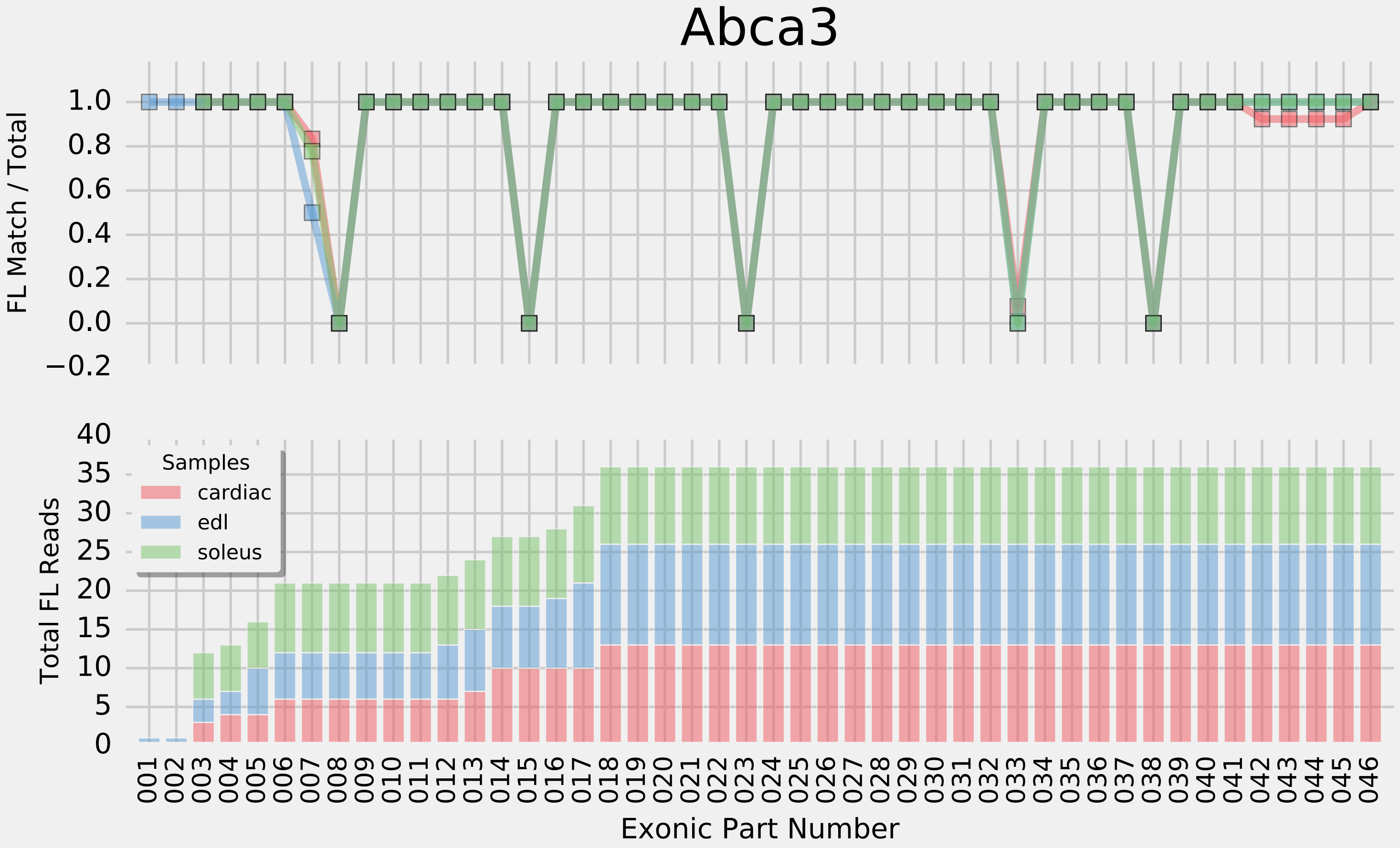

Abca3
FL Match / Total
1.0
0.8
0.6
0.4
0.2
0.0
−0.2
Total FL Reads
40
35
30
25
20
15
10
5
0
Samples
cardiac
edl
soleus
001 002 003 004 005 006 007 008 009 010 011 012 013 014 015 016 017 018 019 020 021 022 023 024 025 026 027 028 029 030 031 032 033 034 035 036 037 038 039 040 041 042 043 044 045 046
Exonic Part Number

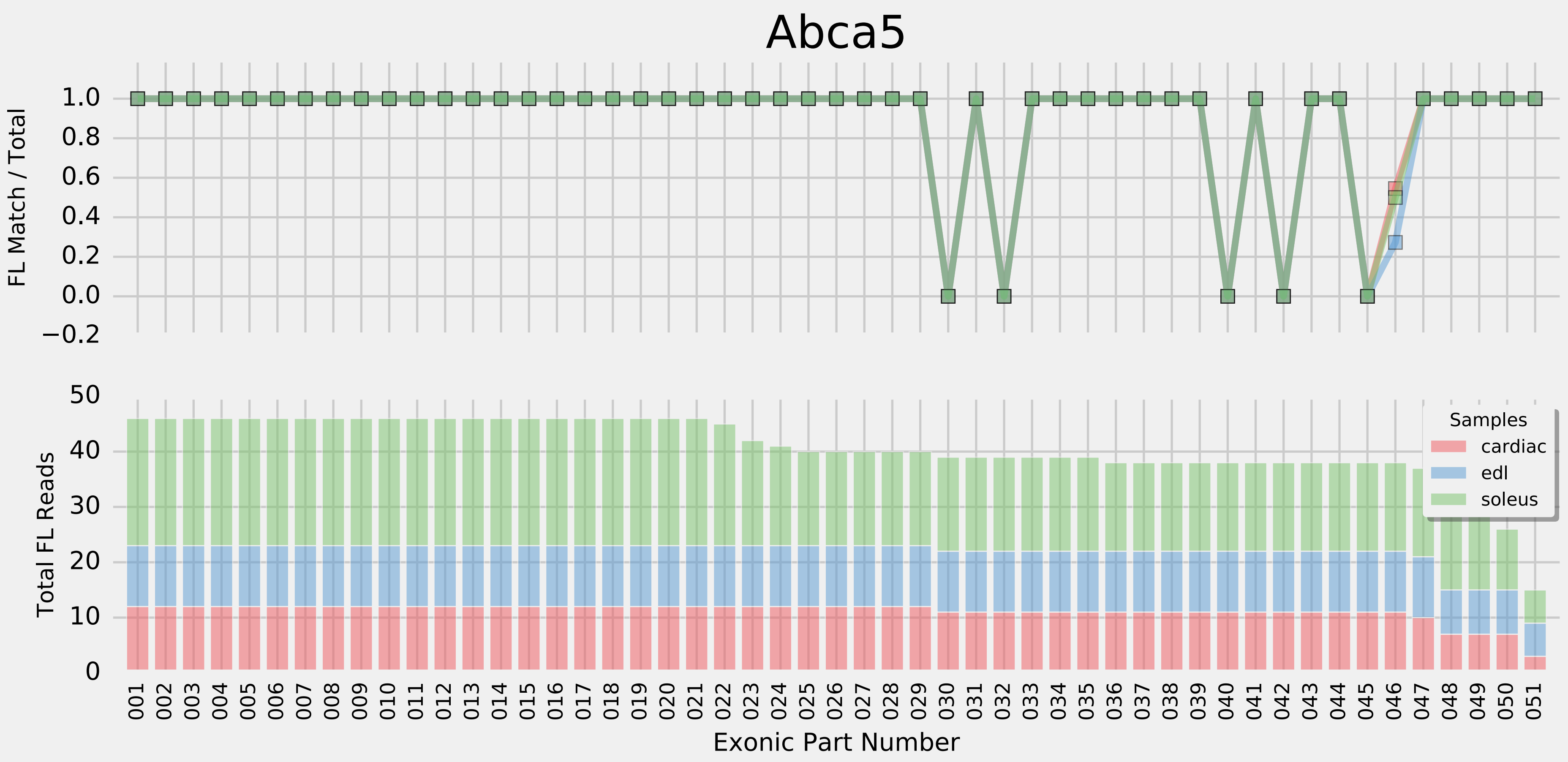
Abca5
FL Match / Total
Total FL Reads
Exonic Part Number
Samples
cardiac
edl
soleus

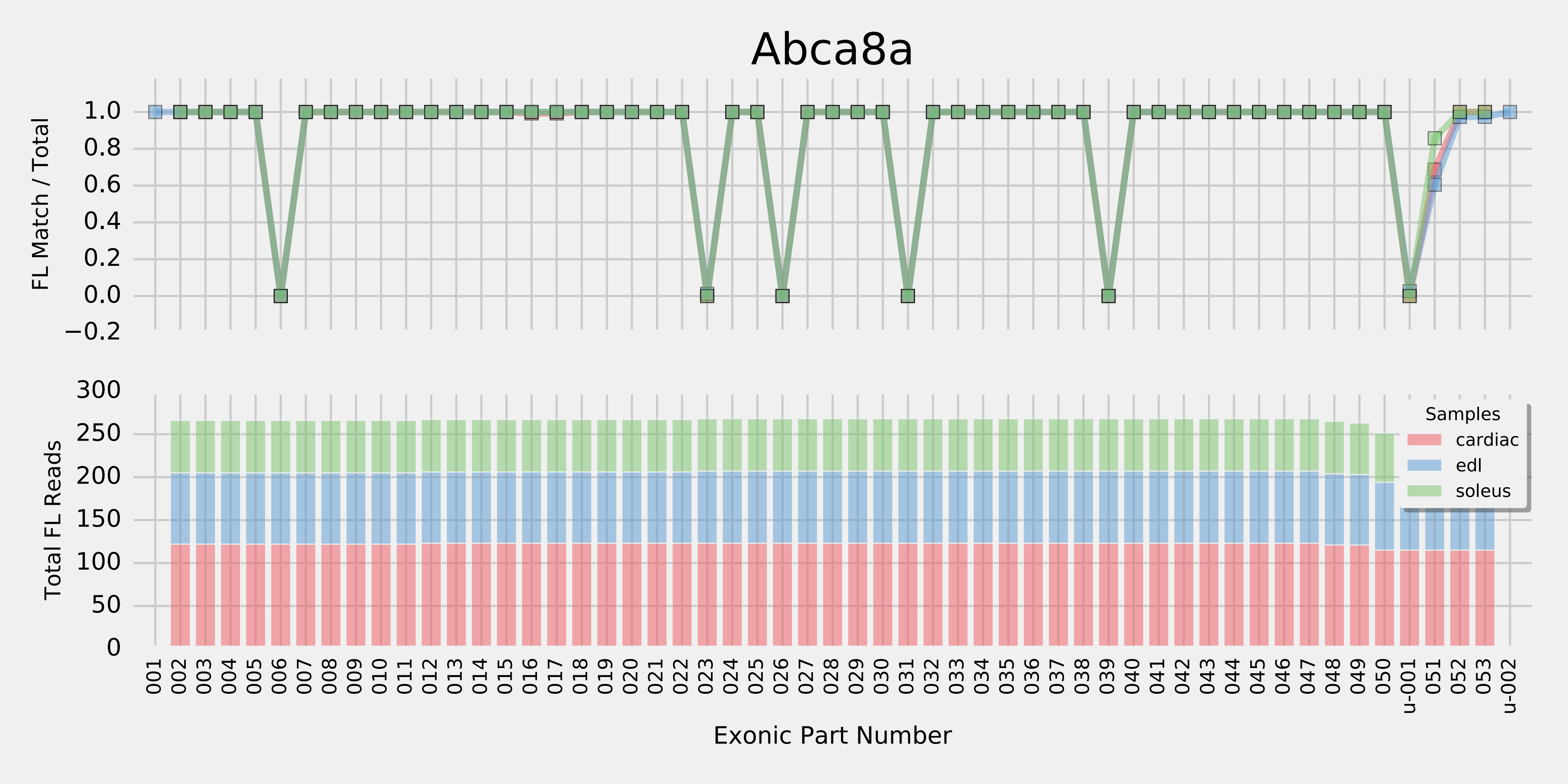

Abca8a
FL Match / Total
Total FL Reads
Samples
cardiac
edl
soleus
Exonic Part Number

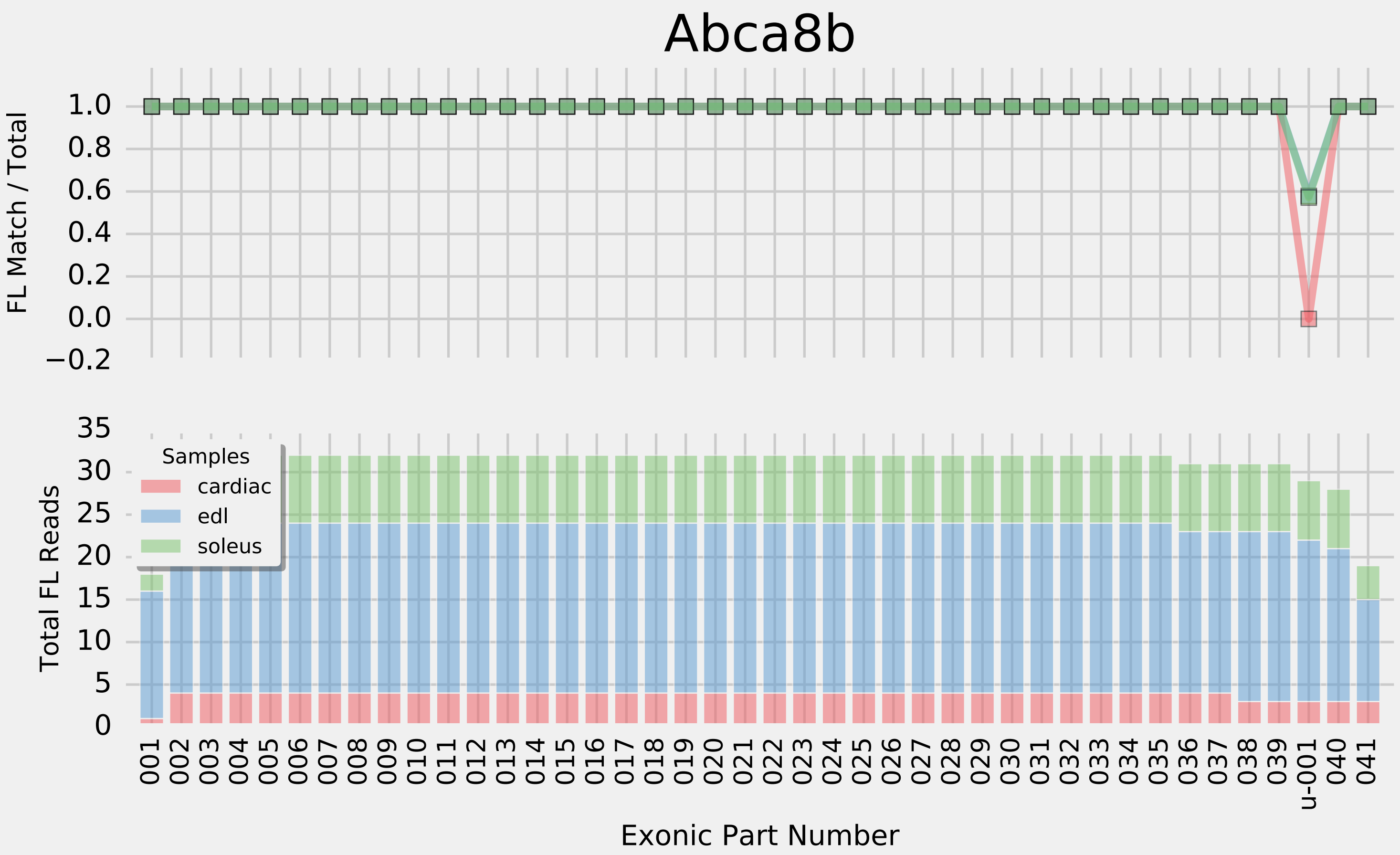

Abca8b
FL Match / Total
1.0
0.8
0.6
0.4
0.2
0.0
−0.2
Total FL Reads
35
30
25
20
15
10
5
0
Samples
cardiac
edl
soleus
001
002
003
004
005
006
007
008
009
010
011
012
013
014
015
016
017
018
019
020
021
022
023
024
025
026
027
028
029
030
031
032
033
034
035
036
037
038
039
u-001
040
041
Exonic Part Number

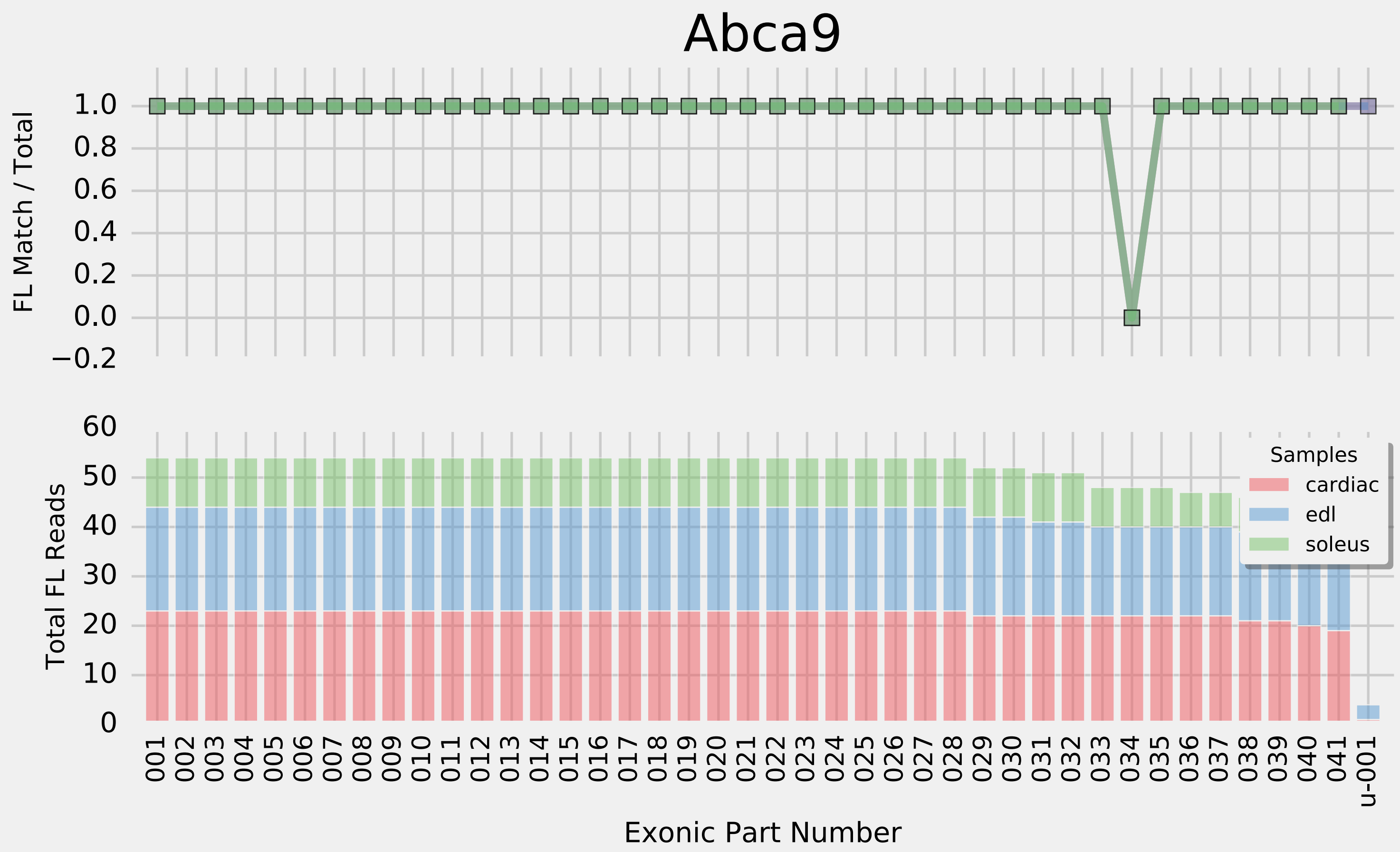
Abca9
FL Match / Total
1.0
0.8
0.6
0.4
0.2
0.0
−0.2
Total FL Reads
60
50
40
30
20
10
0
Samples
cardiac
edl
soleus
001
002
003
004
005
006
007
008
009
010
011
012
013
014
015
016
017
018
019
020
021
022
023
024
025
026
027
028
029
030
031
032
033
034
035
036
037
038
039
040
041
u-001
Exonic Part Number

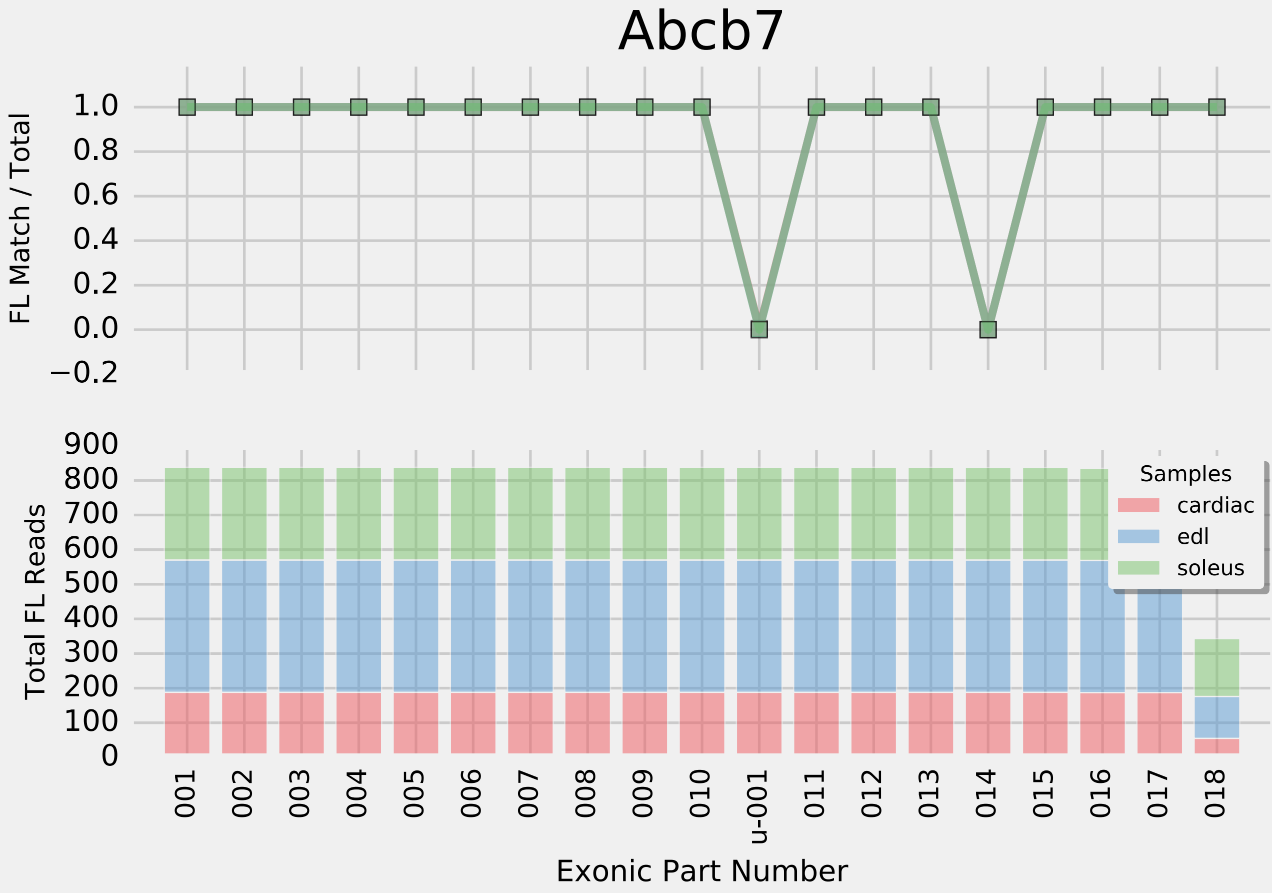
Abcb7
FL Match / Total
1.0
0.8
0.6
0.4
0.2
0.0
−0.2
Total FL Reads
900
800
700
600
500
400
300
200
100
0
Samples
cardiac
edl
soleus
001
002
003
004
005
006
007
008
009
010
u-001
011
012
013
014
015
016
017
018
Exonic Part Number

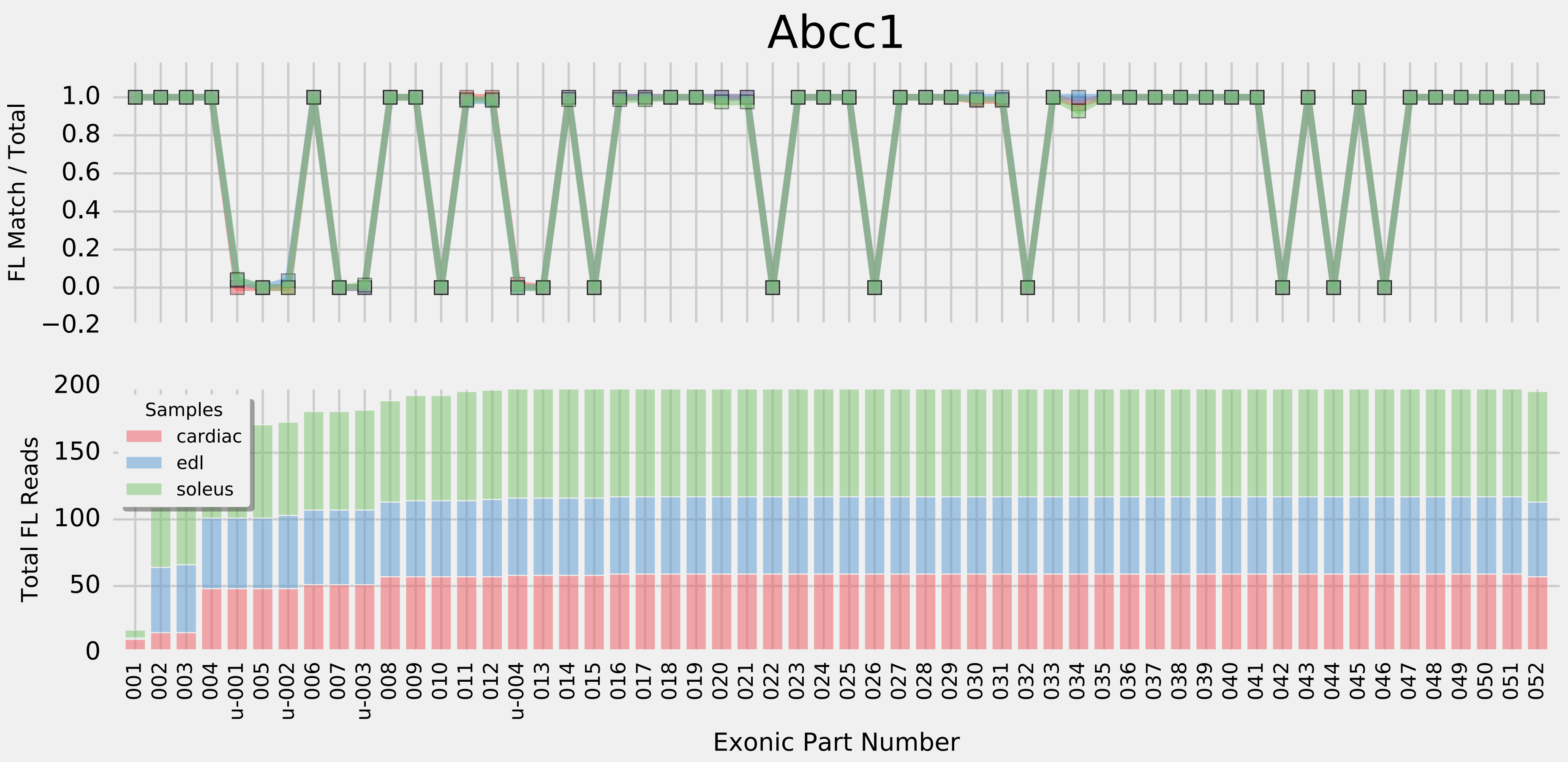
Abcc1
FL Match / Total
1.0
0.8
0.6
0.4
0.2
0.0
−0.2
Total FL Reads
200
150
100
50
0
Samples
cardiac
edl
soleus
001 002 003 004 u-001 005 u-002 006 007 u-003 008 009 010 011 012 u-004 013 014 015 016 017 018 019 020 021 022 023 024 025 026 027 028 029 030 031 032 033 034 035 036 037 038 039 040 041 042 043 044 045 046 047 048 049 050 051 052
Exonic Part Number

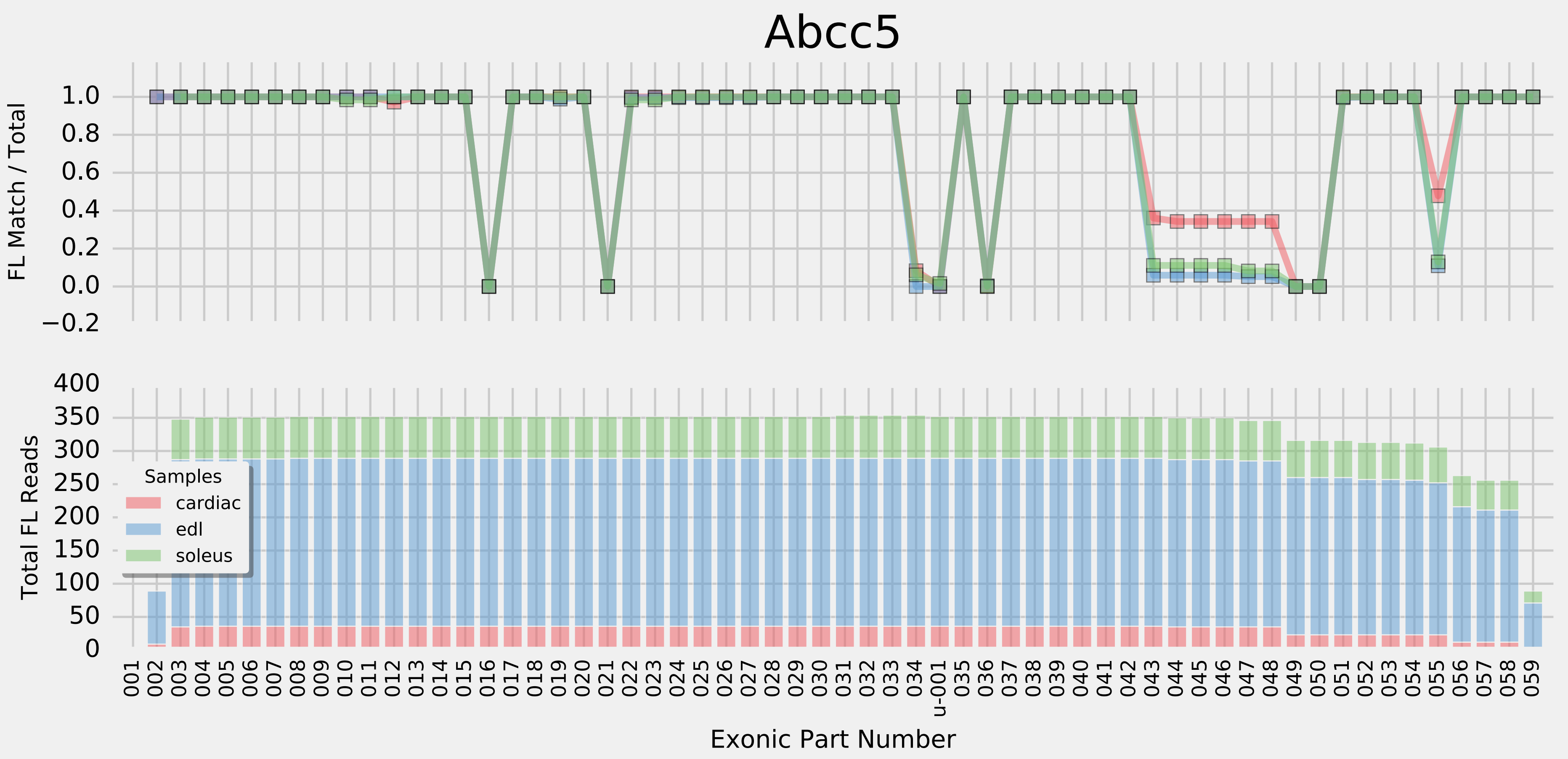

Abcc5
FL Match / Total
Total FL Reads
Samples
cardiac
edl
soleus
Exonic Part Number

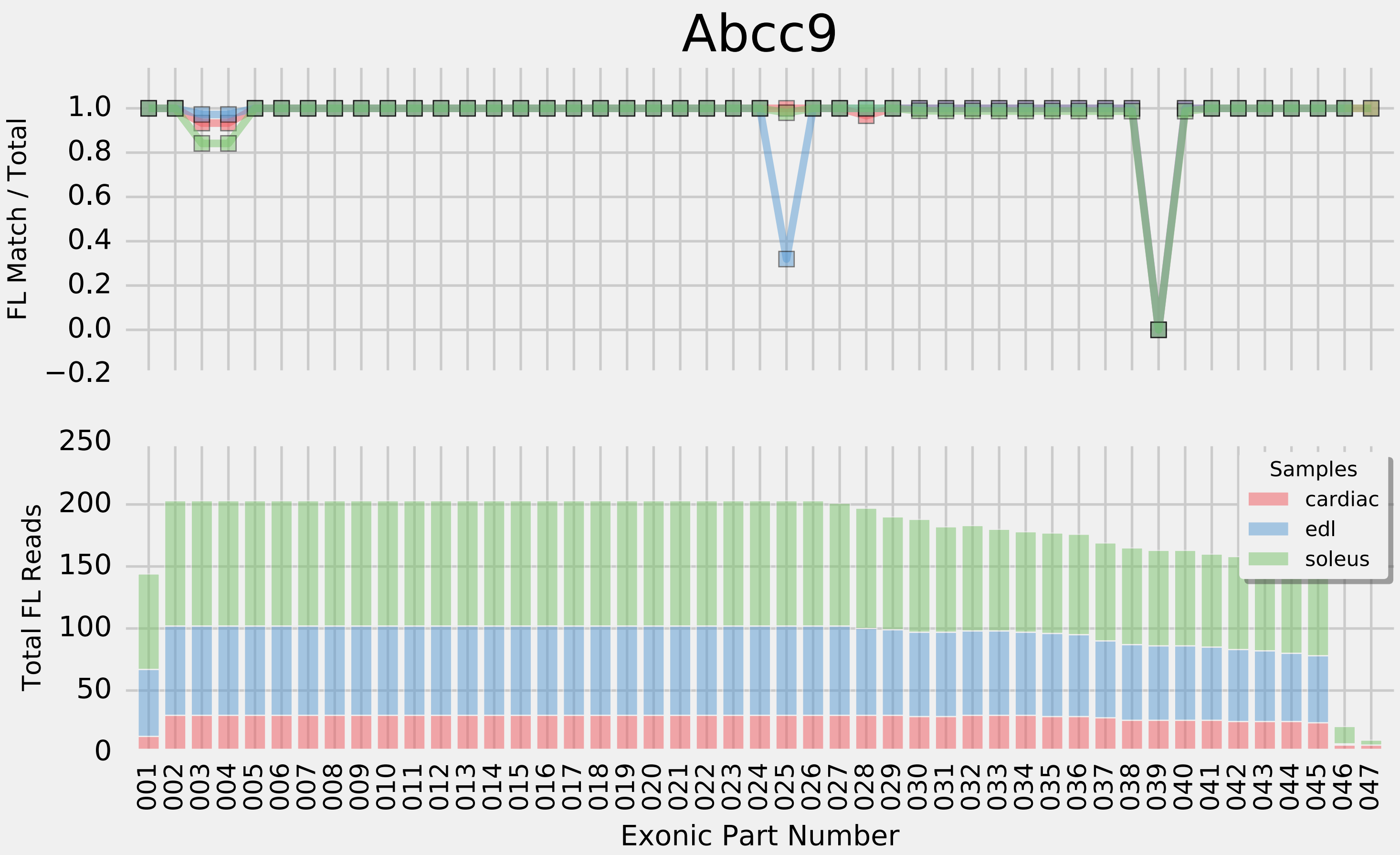

Abcc9
FL Match / Total
1.0
0.8
0.6
0.4
0.2
0.0
−0.2
Total FL Reads
250
200
150
100
50
0
Samples
cardiac
edl
soleus
001 002 003 004 005 006 007 008 009 010 011 012 013 014 015 016 017 018 019 020 021 022 023 024 025 026 027 028 029 030 031 032 033 034 035 036 037 038 039 040 041 042 043 044 045 046 047
Exonic Part Number

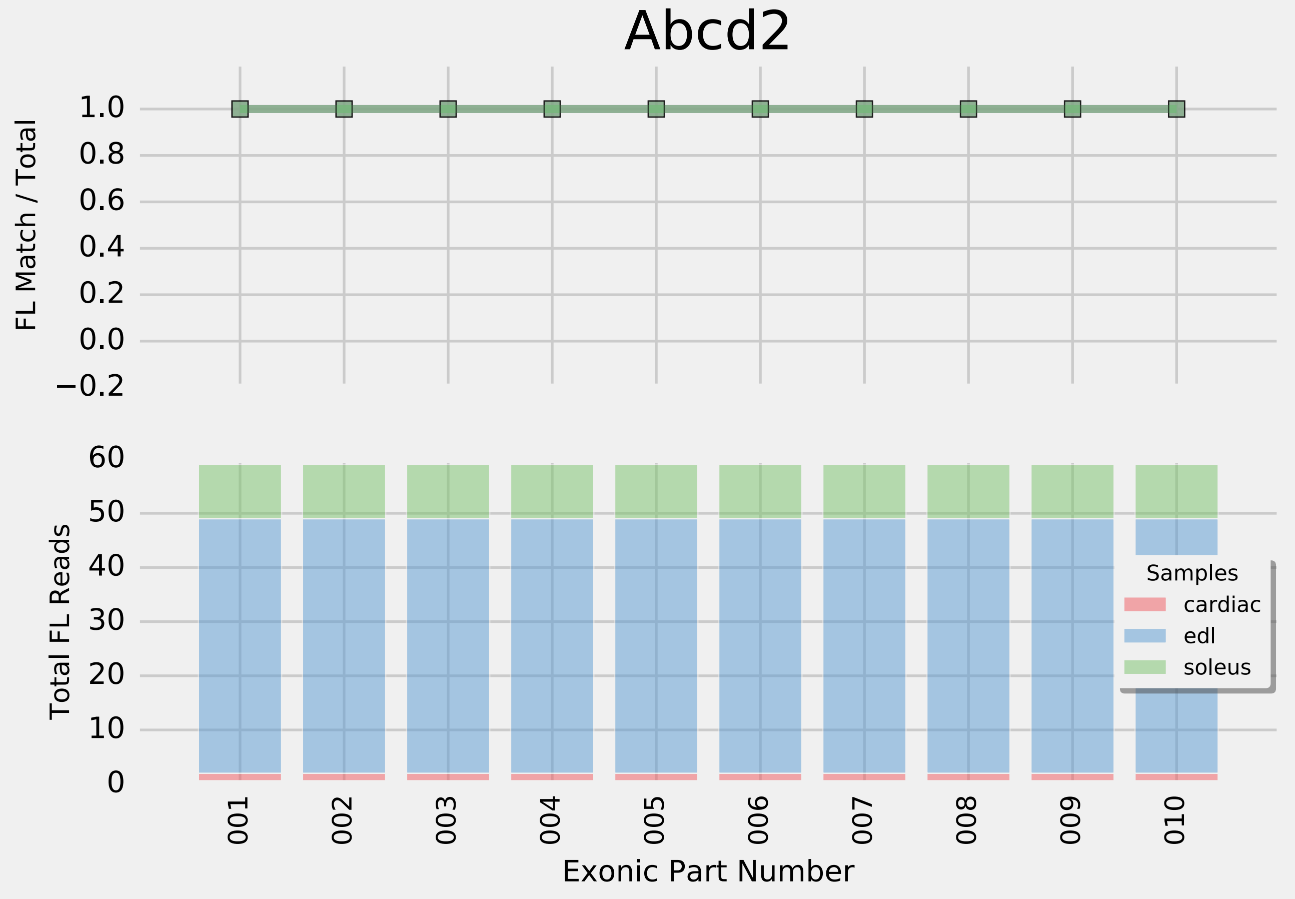
Abcd2
FL Match / Total
1.0
0.8
0.6
0.4
0.2
0.0
−0.2
Total FL Reads
60
50
40
30
20
10
0
Samples
cardiac
edl
soleus
001
002
003
004
005
006
007
008
009
010
Exonic Part Number

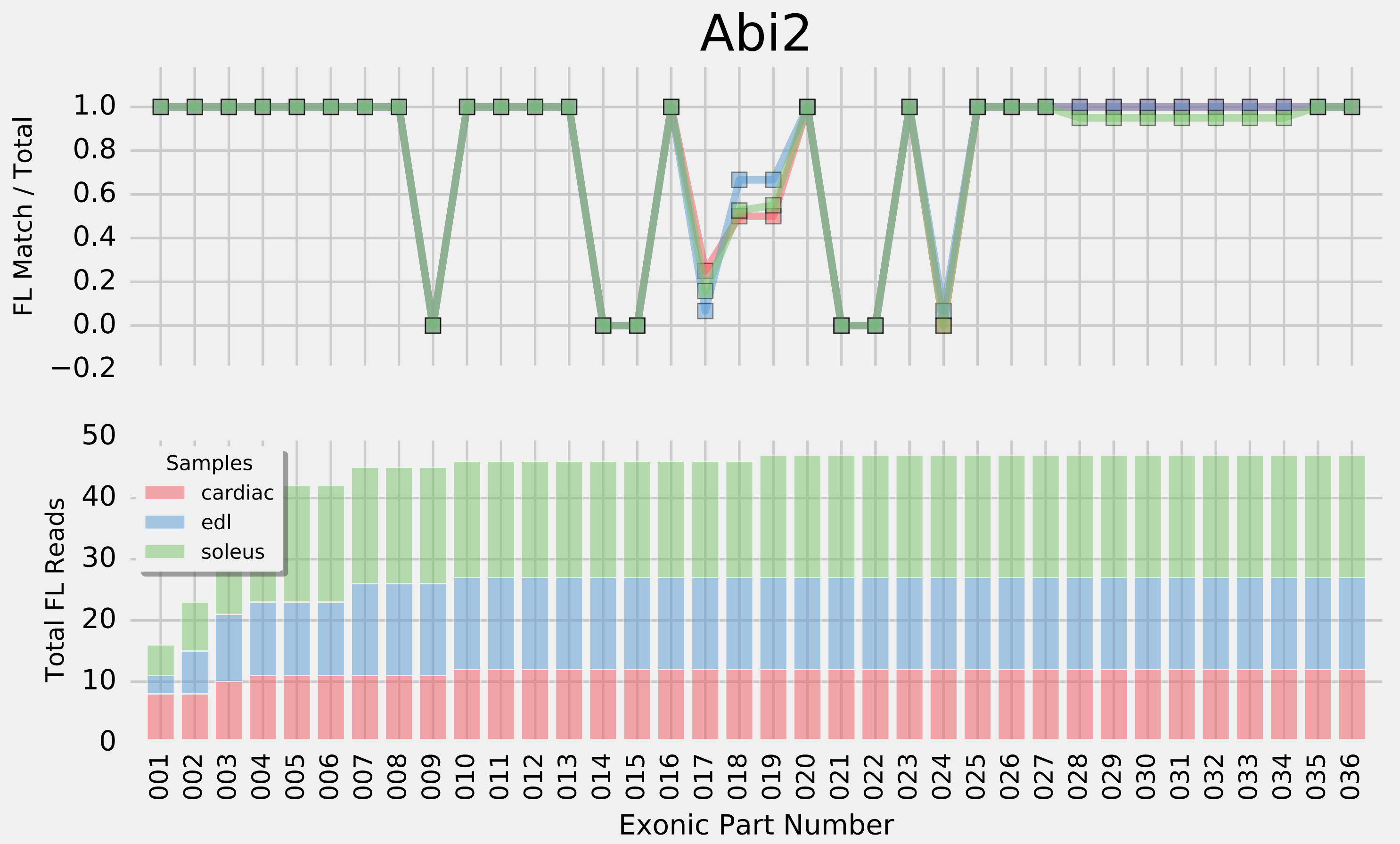

Abi2
FL Match / Total
1.0
0.8
0.6
0.4
0.2
0.0
−0.2
Total FL Reads
50
40
30
20
10
0
Samples
cardiac
edl
soleus
001 002 003 004 005 006 007 008 009 010 011 012 013 014 015 016 017 018 019 020 021 022 023 024 025 026 027 028 029 030 031 032 033 034 035 036
Exonic Part Number

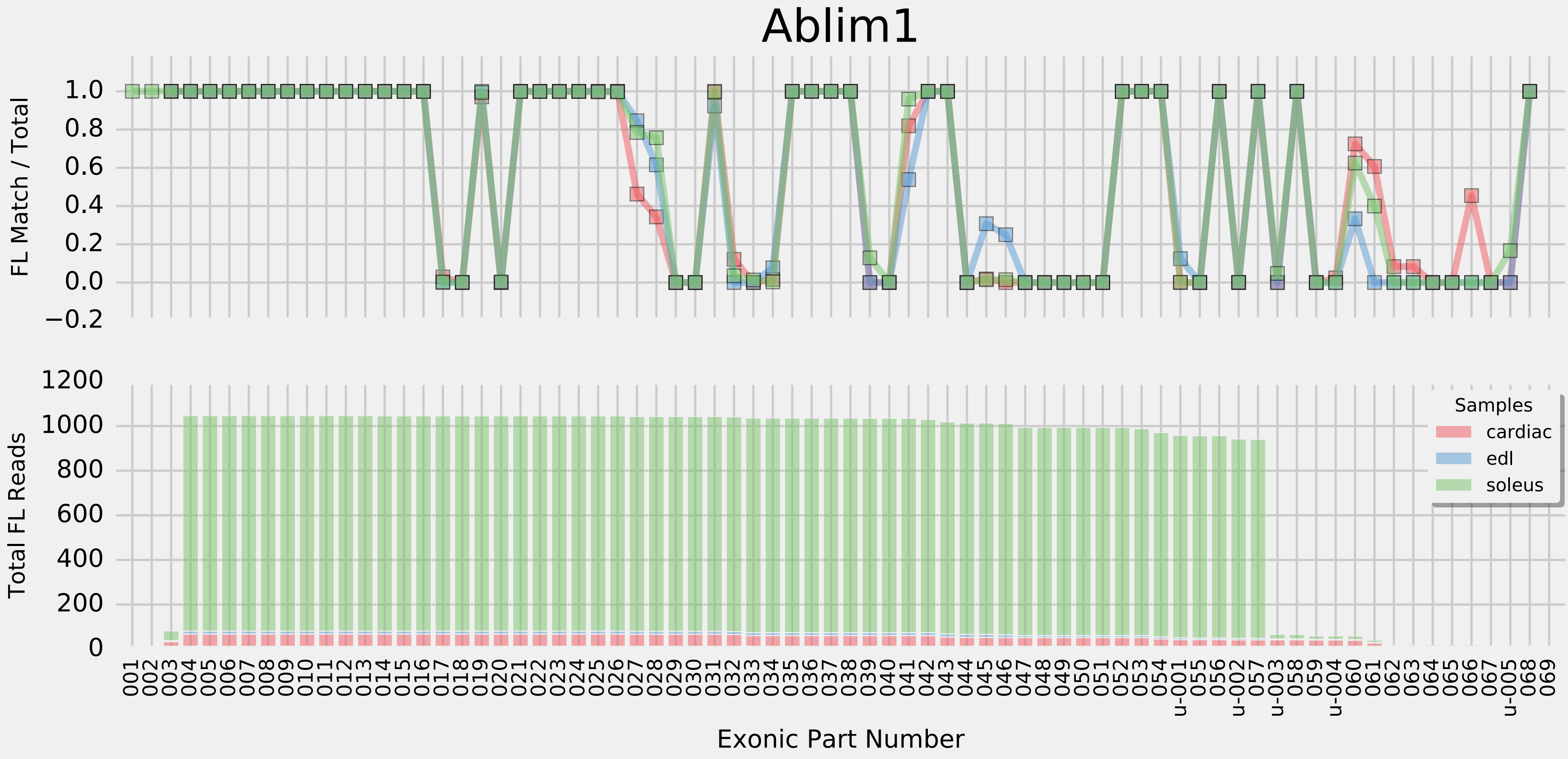

Ablim1
FL Match / Total
1.0
0.8
0.6
0.4
0.2
0.0
−0.2
Total FL Reads
1200
1000
800
600
400
200
0
Samples
cardiac
edl
soleus
Exonic Part Number

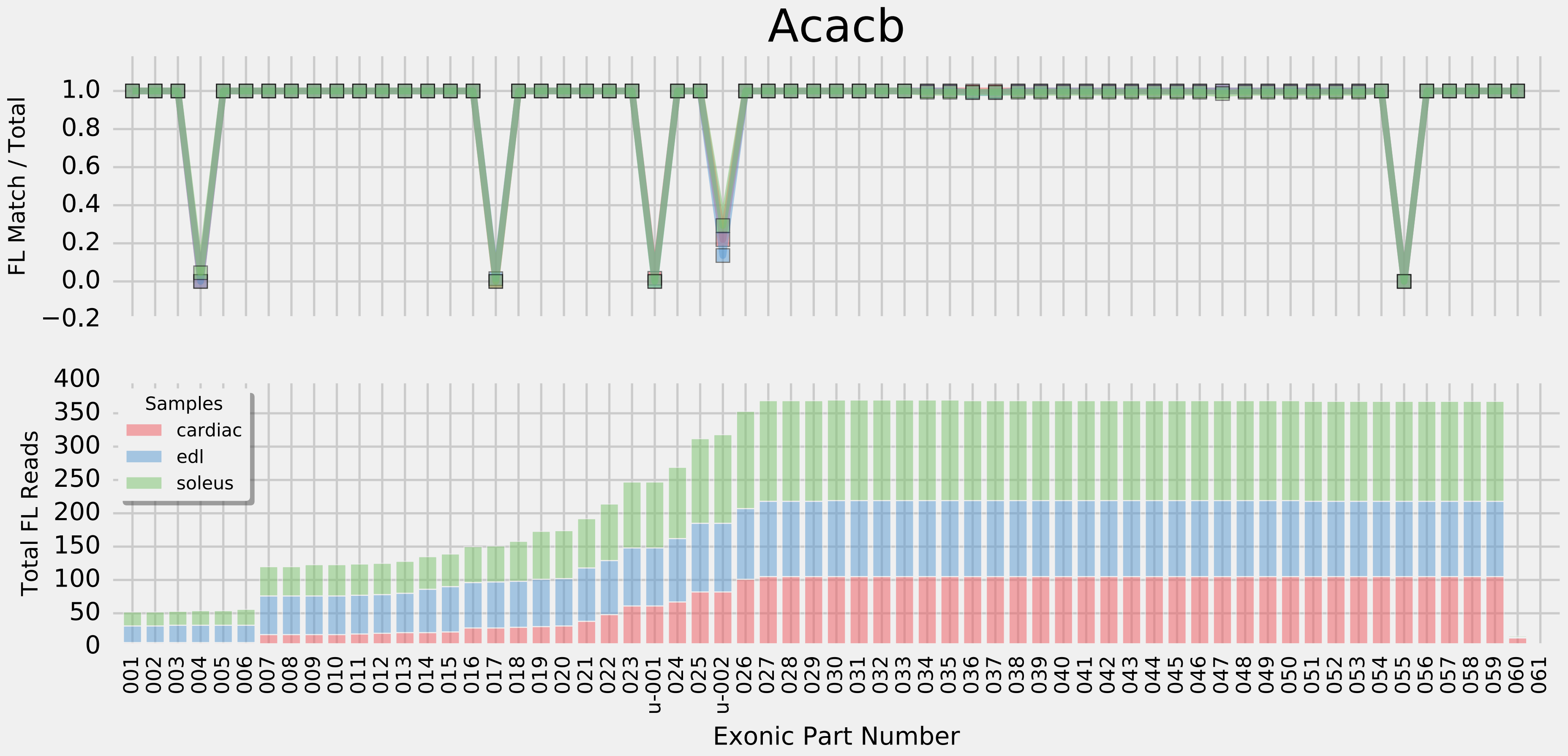

Acacb
FL Match / Total
1.0
0.8
0.6
0.4
0.2
0.0
−0.2
Total FL Reads
400
350
300
250
200
150
100
50
0
Samples
cardiac
edl
soleus
001 002 003 004 005 006 007 008 009 010 011 012 013 014 015 016 017 018 019 020 021 022 023 u-001 024 025 u-002 026 027 028 029 030 031 032 033 034 035 036 037 038 039 040 041 042 043 044 045 046 047 048 049 050 051 052 053 054 055 056 057 058 059 060 061
Exonic Part Number

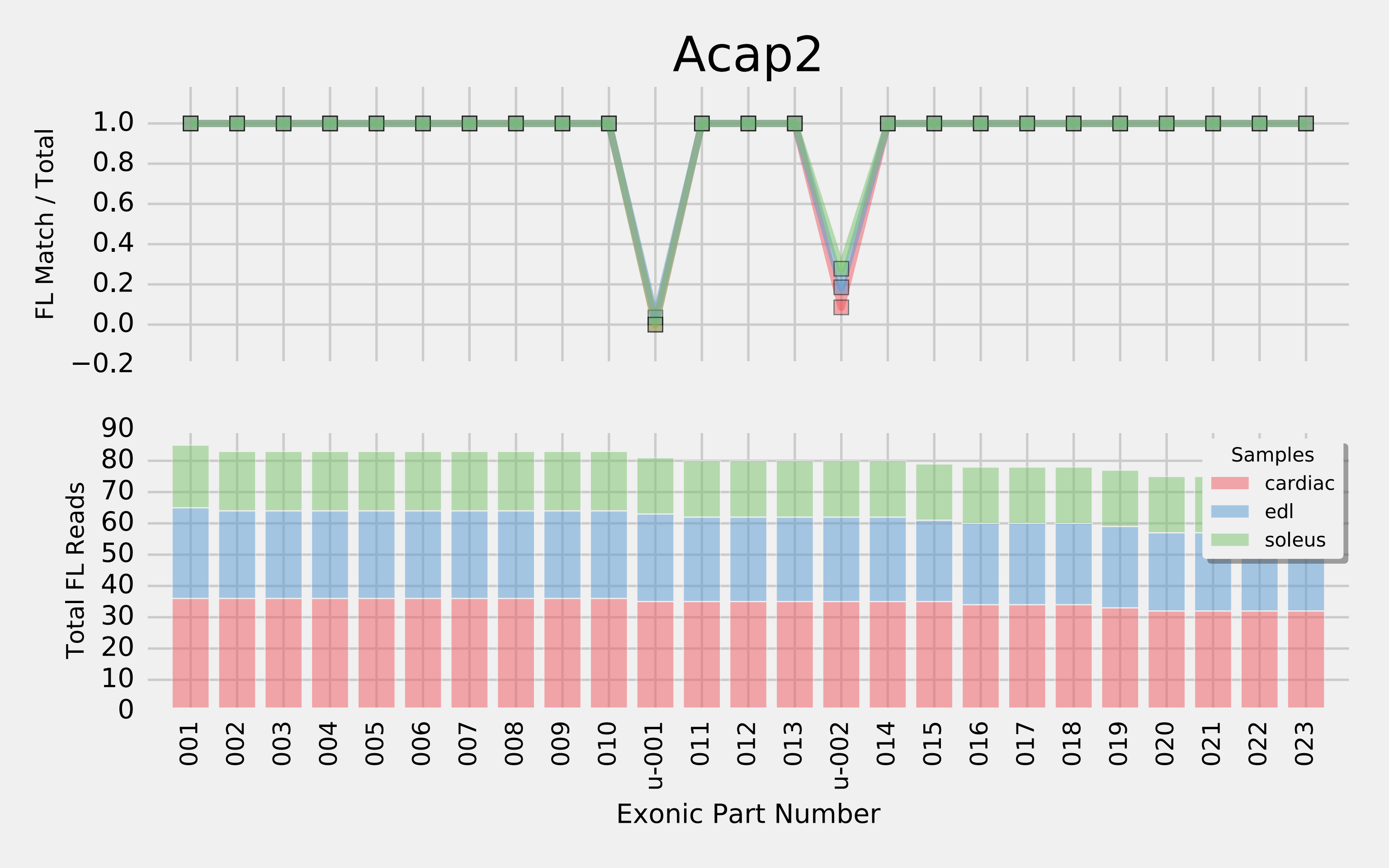

Acap2
FL Match / Total
1.0
0.8
0.6
0.4
0.2
0.0
−0.2
Total FL Reads
90
80
70
60
50
40
30
20
10
0
Samples
cardiac
edl
soleus
001
002
003
004
005
006
007
008
009
010
u-001
011
012
013
u-002
014
015
016
017
018
019
020
021
022
023
Exonic Part Number

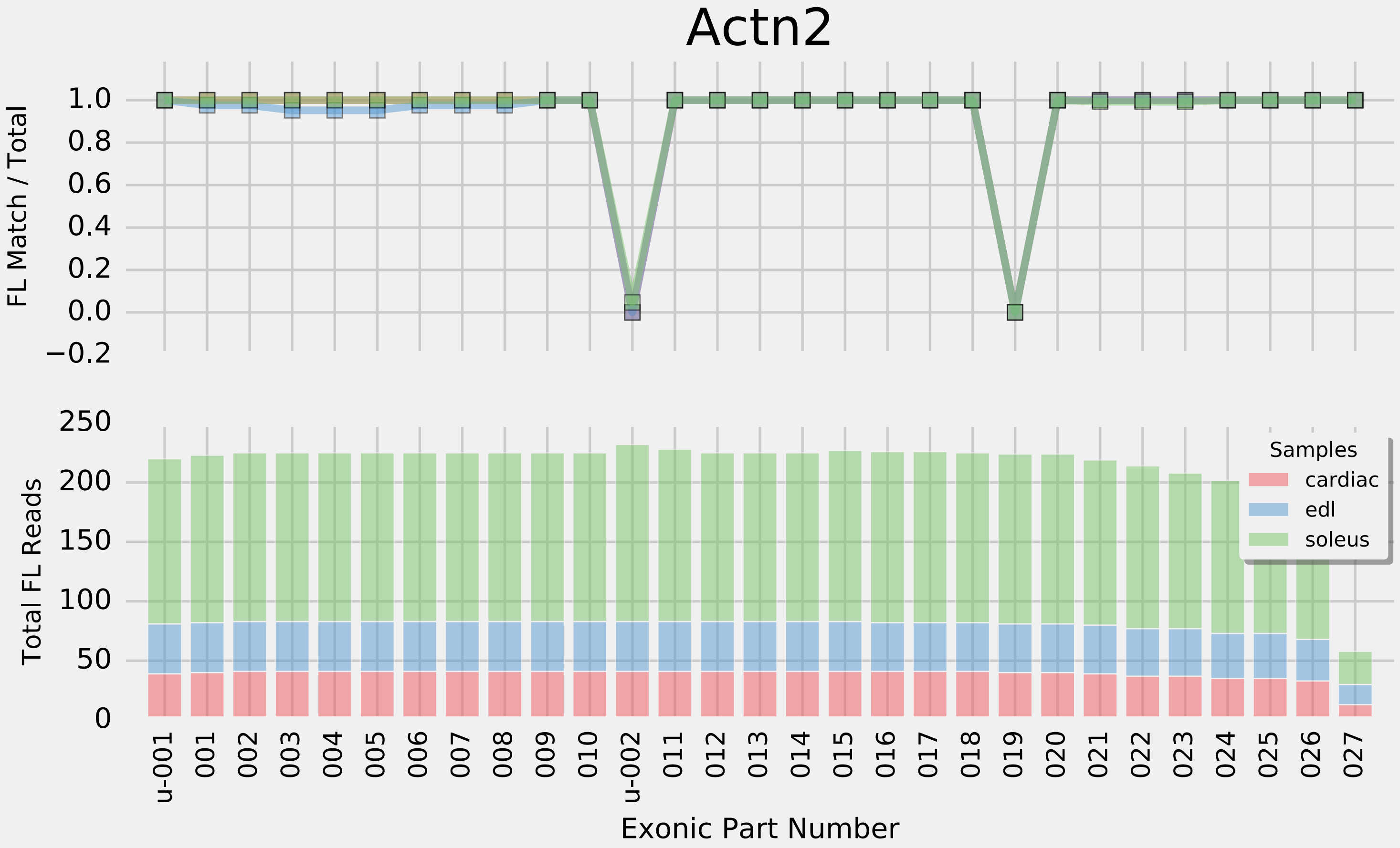

Actn2
FL Match / Total
1.0
0.8
0.6
0.4
0.2
0.0
−0.2
Total FL Reads
250
200
150
100
50
0
Samples
cardiac
edl
soleus
u-001
001
002
003
004
005
006
007
008
009
010
u-002
011
012
013
014
015
016
017
018
019
020
021
022
023
024
025
026
027
Exonic Part Number

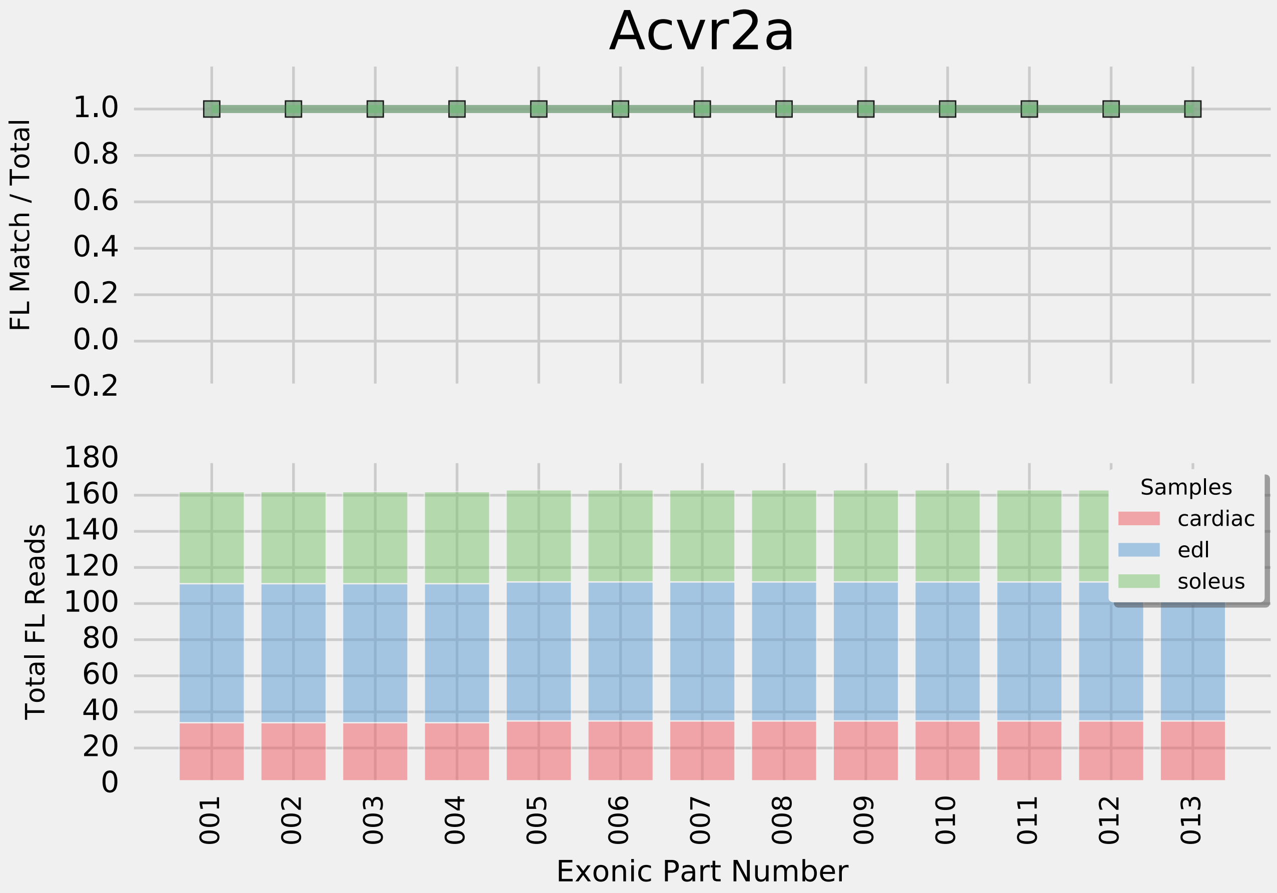
Acvr2a
FL Match / Total
1.0
0.8
0.6
0.4
0.2
0.0
−0.2
Total FL Reads
180
160
140
120
100
80
60
40
20
0
Samples
cardiac
edl
soleus
001
002
003
004
005
006
007
008
009
010
011
012
013
Exonic Part Number

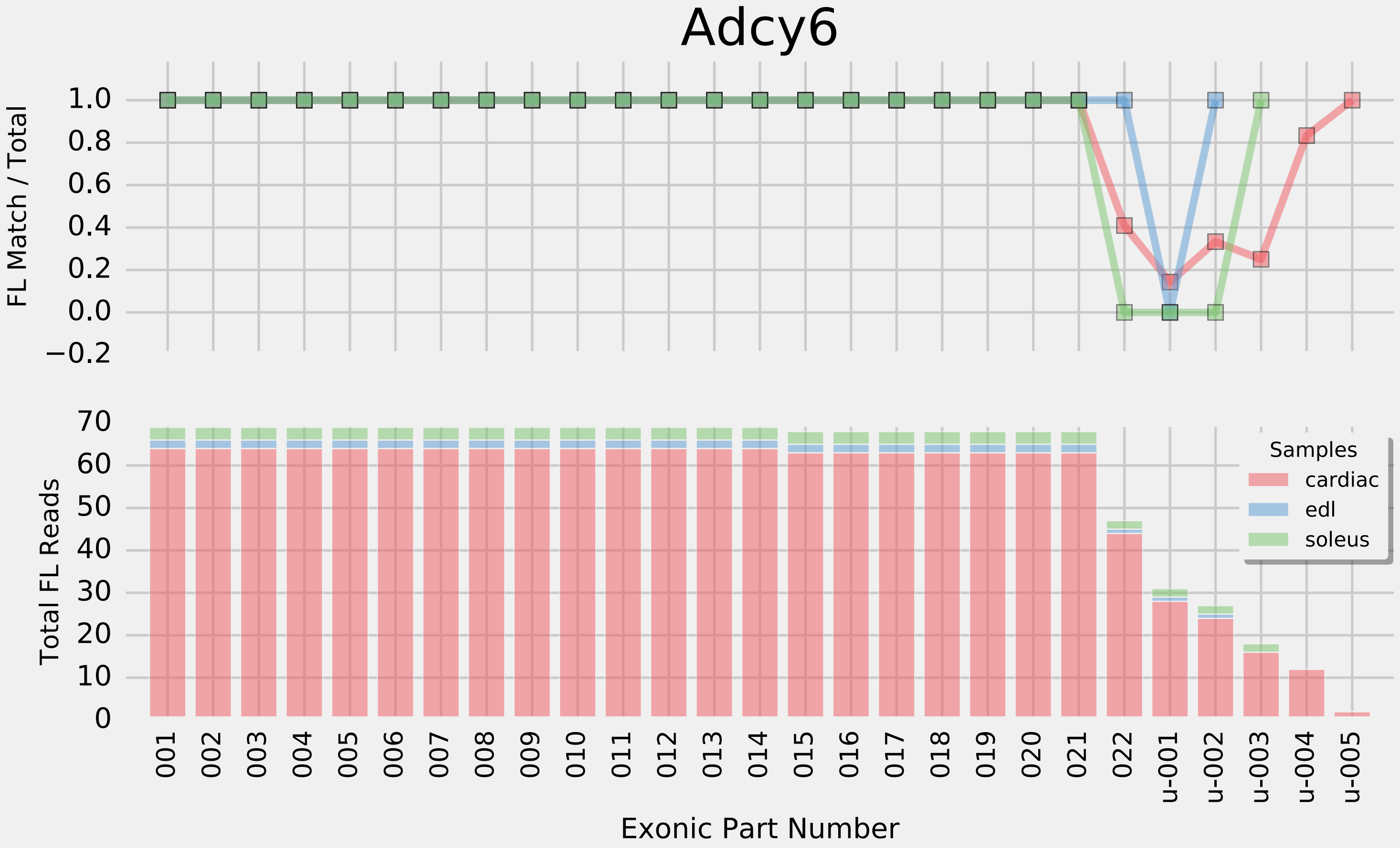

Adcy6
FL Match / Total
1.0
0.8
0.6
0.4
0.2
0.0
−0.2
Total FL Reads
70
60
50
40
30
20
10
0
Samples
cardiac
edl
soleus
001 002 003 004 005 006 007 008 009 010 011 012 013 014 015 016 017 018 019 020 021 022 u-001 u-002 u-003 u-004 u-005
Exonic Part Number

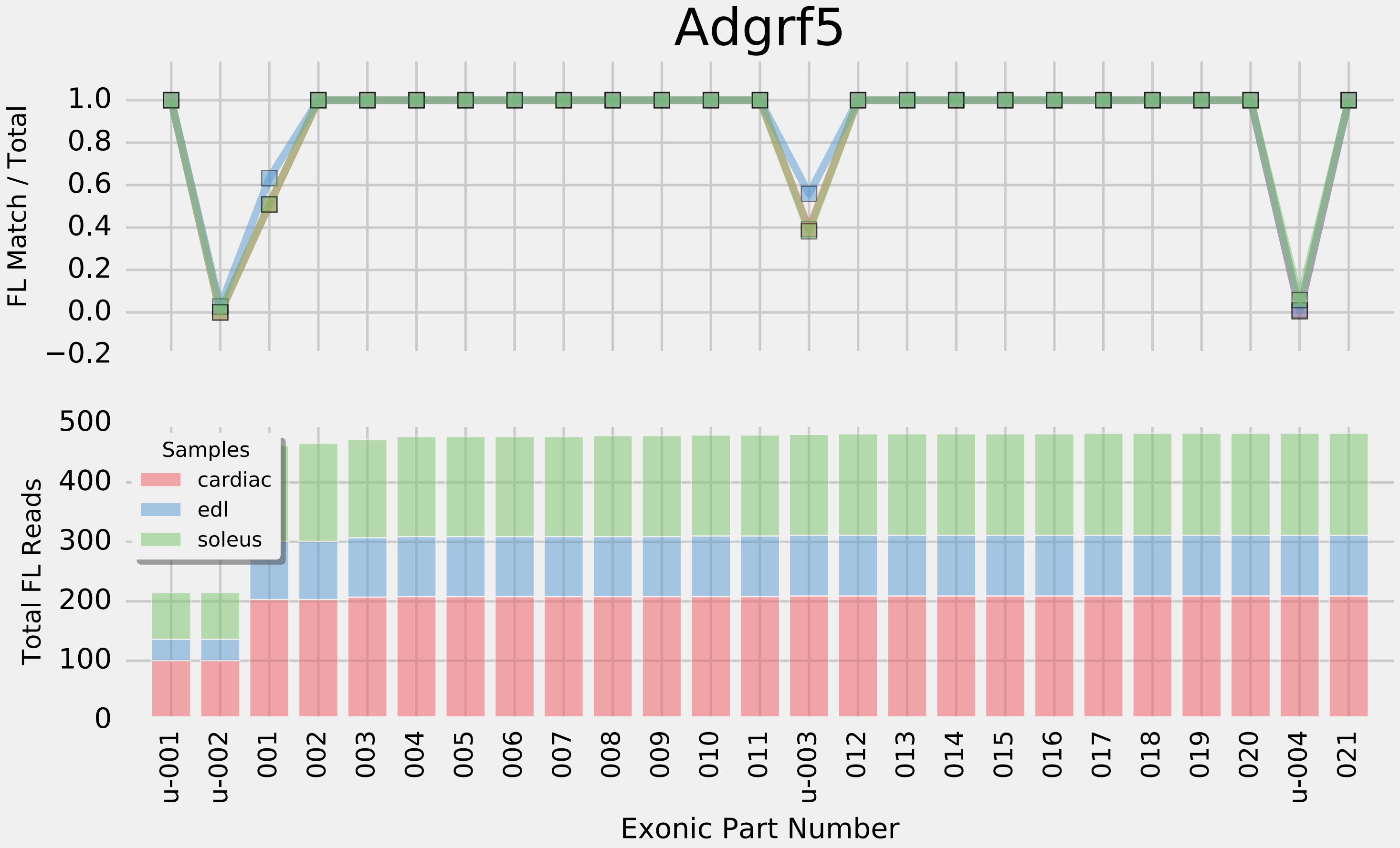

Adgrf5
FL Match / Total
1.0
0.8
0.6
0.4
0.2
0.0
−0.2
Total FL Reads
500
400
300
200
100
0
Samples
cardiac
edl
soleus
u-001
u-002
001
002
003
004
005
006
007
008
009
010
011
u-003
012
013
014
015
016
017
018
019
020
u-004
021
Exonic Part Number

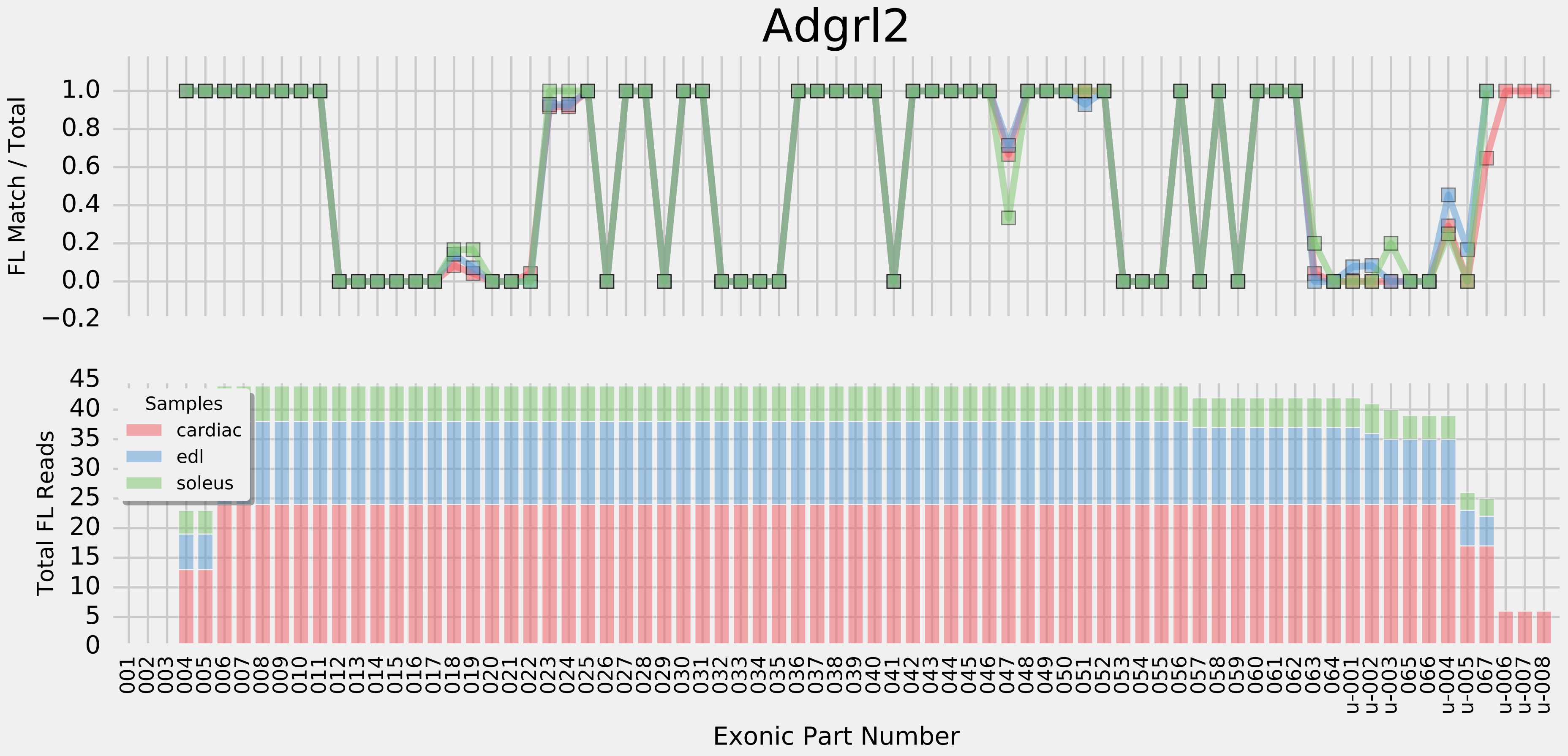
Adgrl2
FL Match / Total
Total FL Reads
Samples
cardiac
edl
soleus
Exonic Part Number

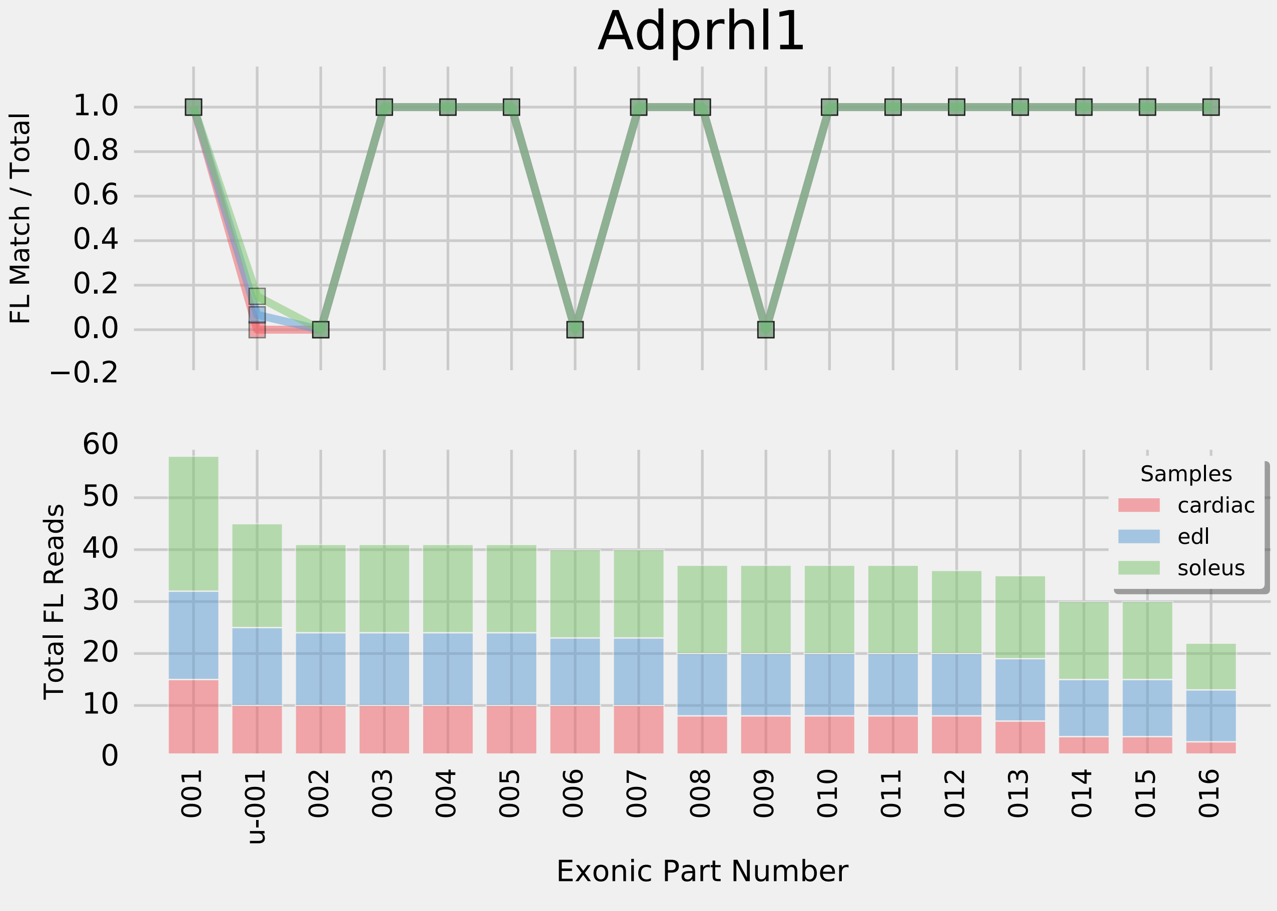

Adprhl1
FL Match / Total
1.0
0.8
0.6
0.4
0.2
0.0
−0.2
Total FL Reads
60
50
40
30
20
10
0
Samples
cardiac
edl
soleus
001
u-001
002
003
004
005
006
007
008
009
010
011
012
013
014
015
016
Exonic Part Number

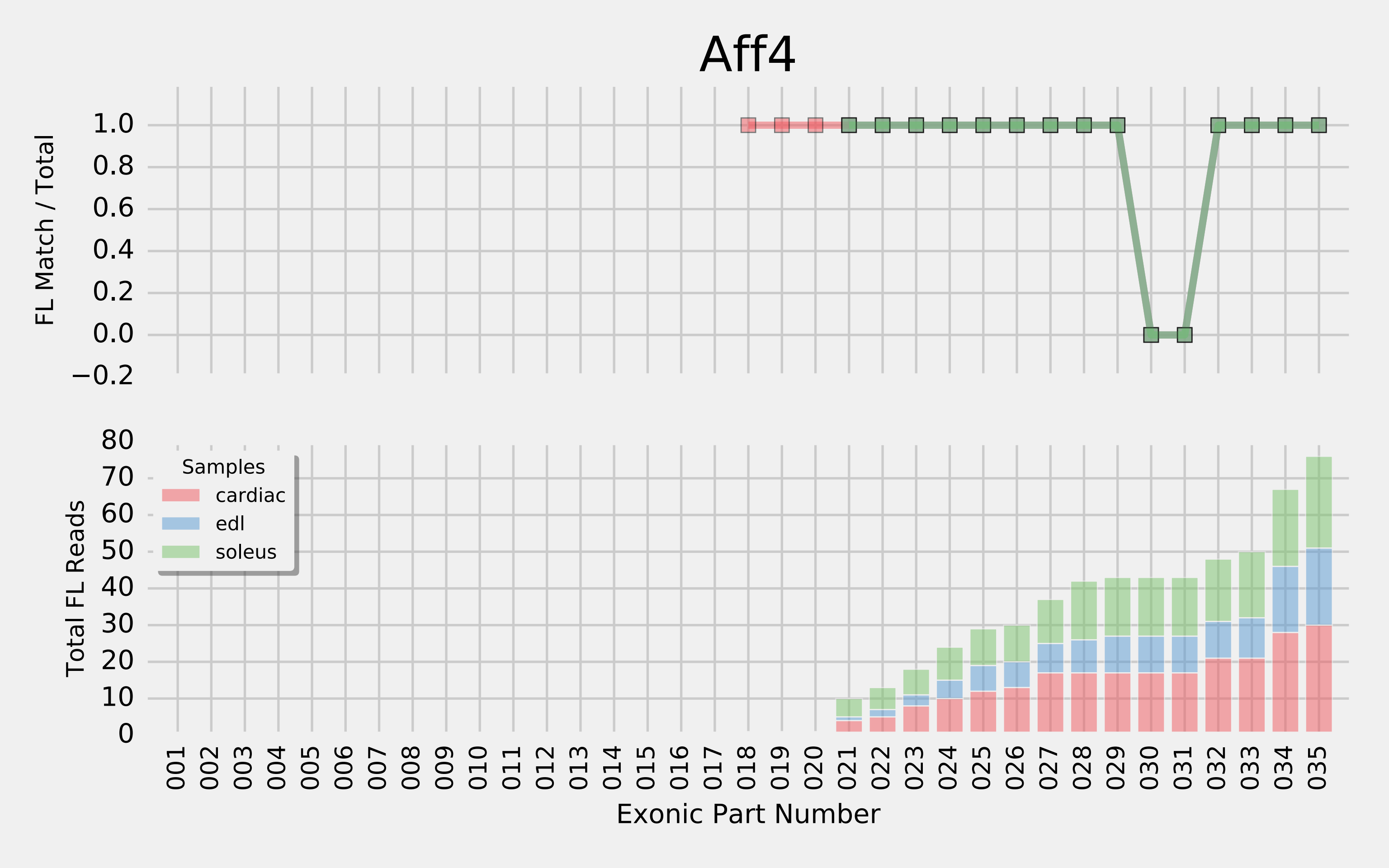

Aff4
FL Match / Total
1.0
0.8
0.6
0.4
0.2
0.0
−0.2
Total FL Reads
80
70
60
50
40
30
20
10
0
Samples
cardiac
edl
soleus
001
002
003
004
005
006
007
008
009
010
011
012
013
014
015
016
017
018
019
020
021
022
023
024
025
026
027
028
029
030
031
032
033
034
035
Exonic Part Number

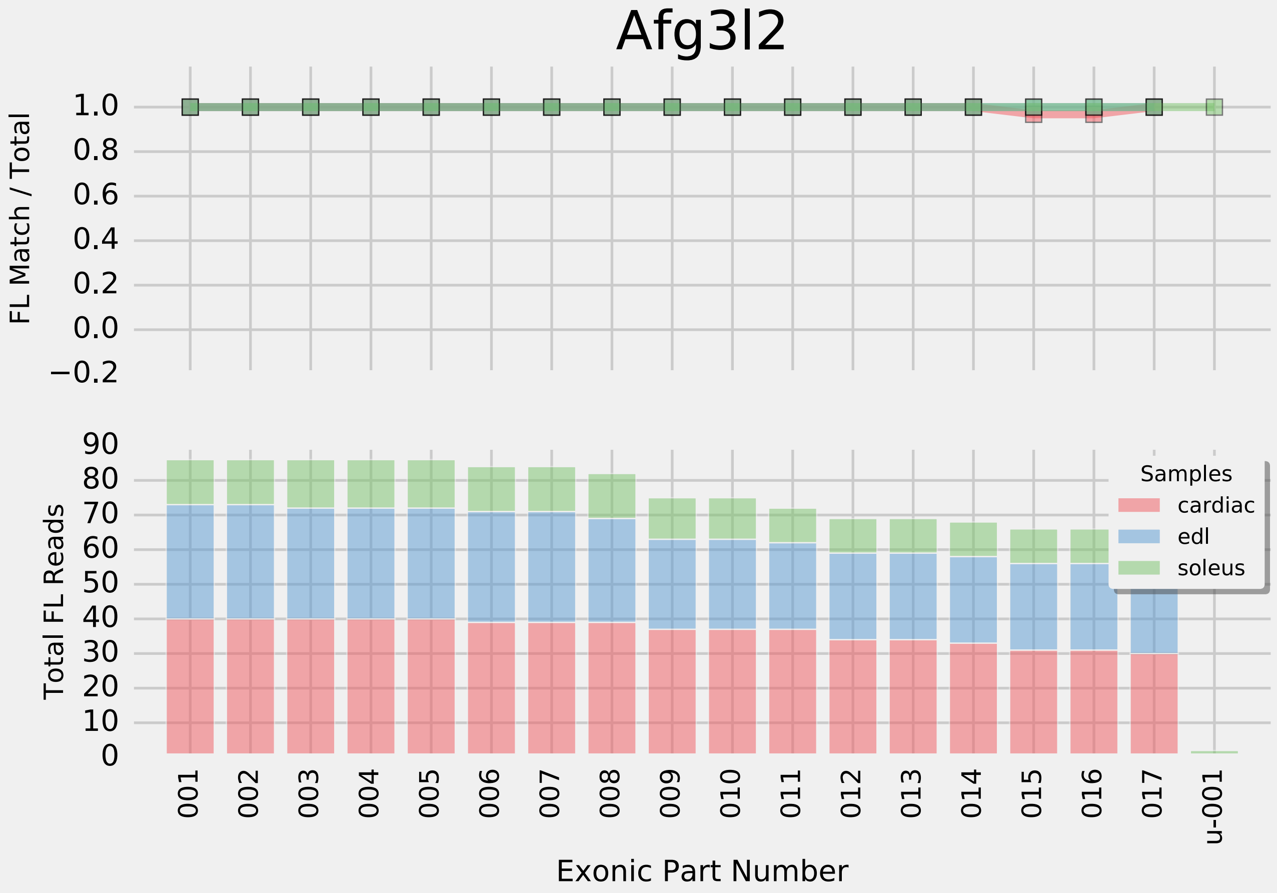

Afg3l2
FL Match / Total
1.0
0.8
0.6
0.4
0.2
0.0
−0.2
Total FL Reads
90
80
70
60
50
40
30
20
10
0
Samples
cardiac
edl
soleus
001
002
003
004
005
006
007
008
009
010
011
012
013
014
015
016
017
u-001
Exonic Part Number

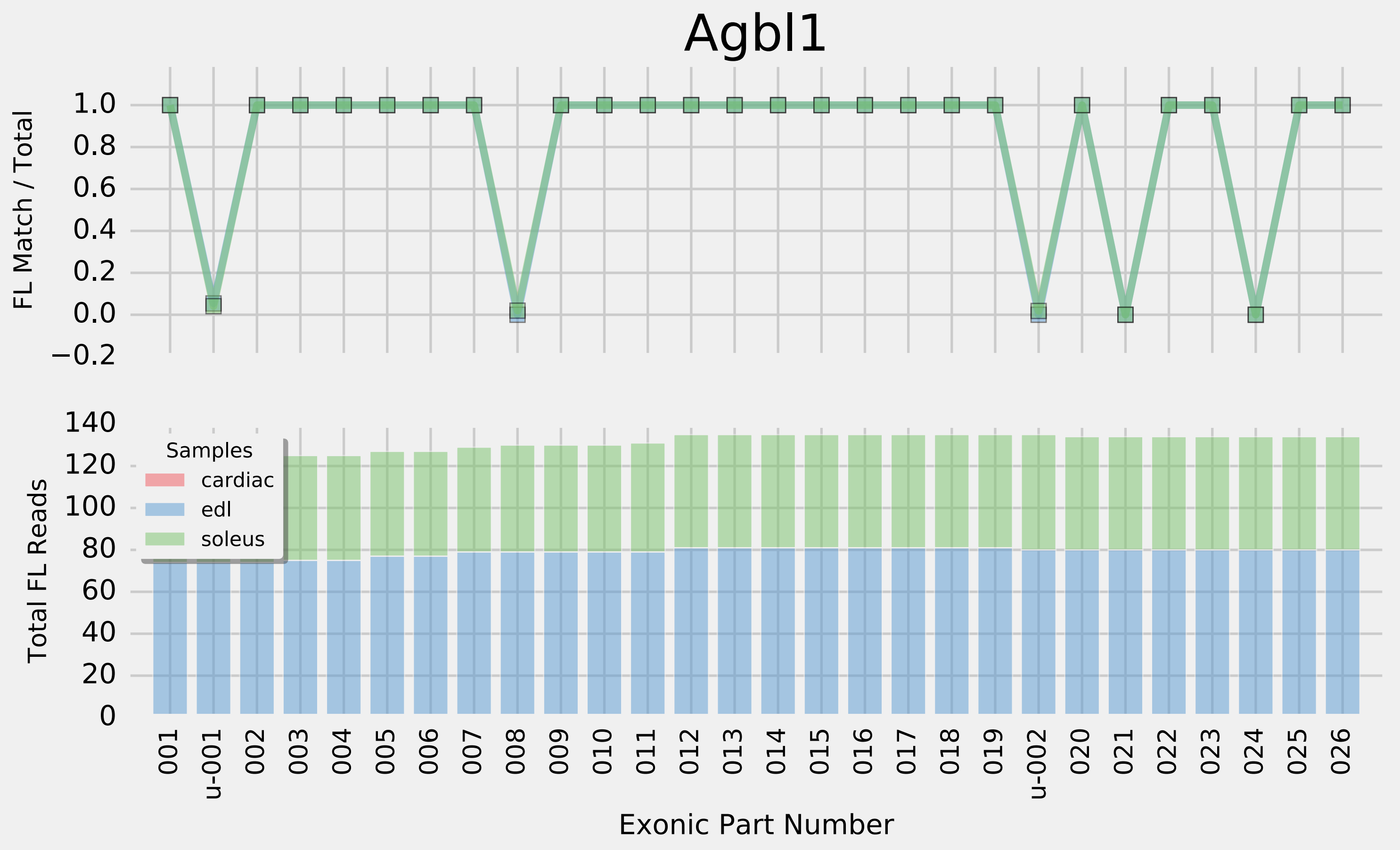

Agbl1
FL Match / Total
1.0
0.8
0.6
0.4
0.2
0.0
−0.2
Total FL Reads
140
120
100
80
60
40
20
0
Samples
cardiac
edl
soleus
001
u-001
002
003
004
005
006
007
008
009
010
011
012
013
014
015
016
017
018
019
u-002
020
021
022
023
024
025
026
Exonic Part Number

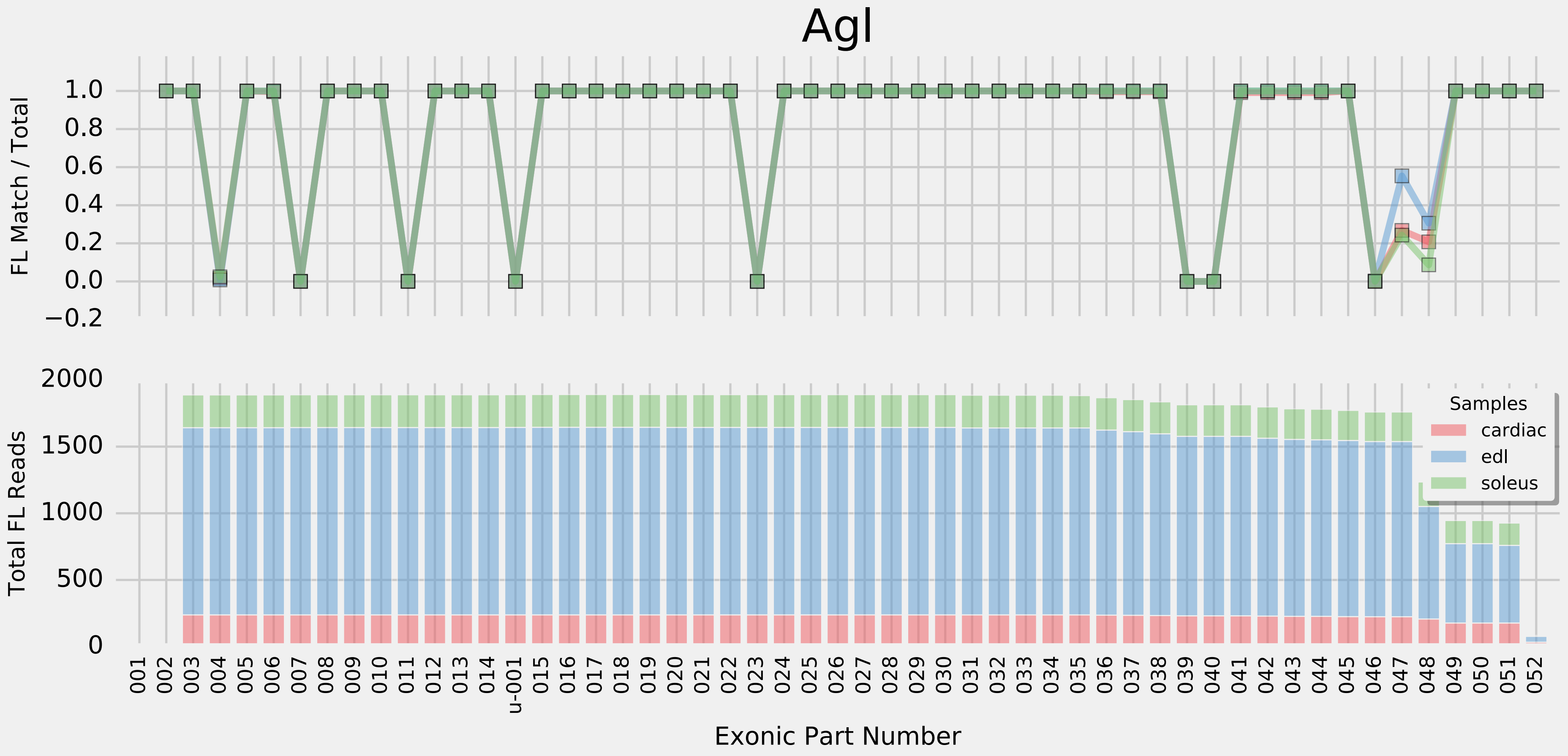

Agl
FL Match / Total
1.0
0.8
0.6
0.4
0.2
0.0
−0.2
2000
1500
1000
500
0
Total FL Reads
Samples
cardiac
edl
soleus
001 002 003 004 005 006 007 008 009 010 011 012 013 014 u-001 015 016 017 018 019 020 021 022 023 024 025 026 027 028 029 030 031 032 033 034 035 036 037 038 039 040 041 042 043 044 045 046 047 048 049 050 051 052
Exonic Part Number

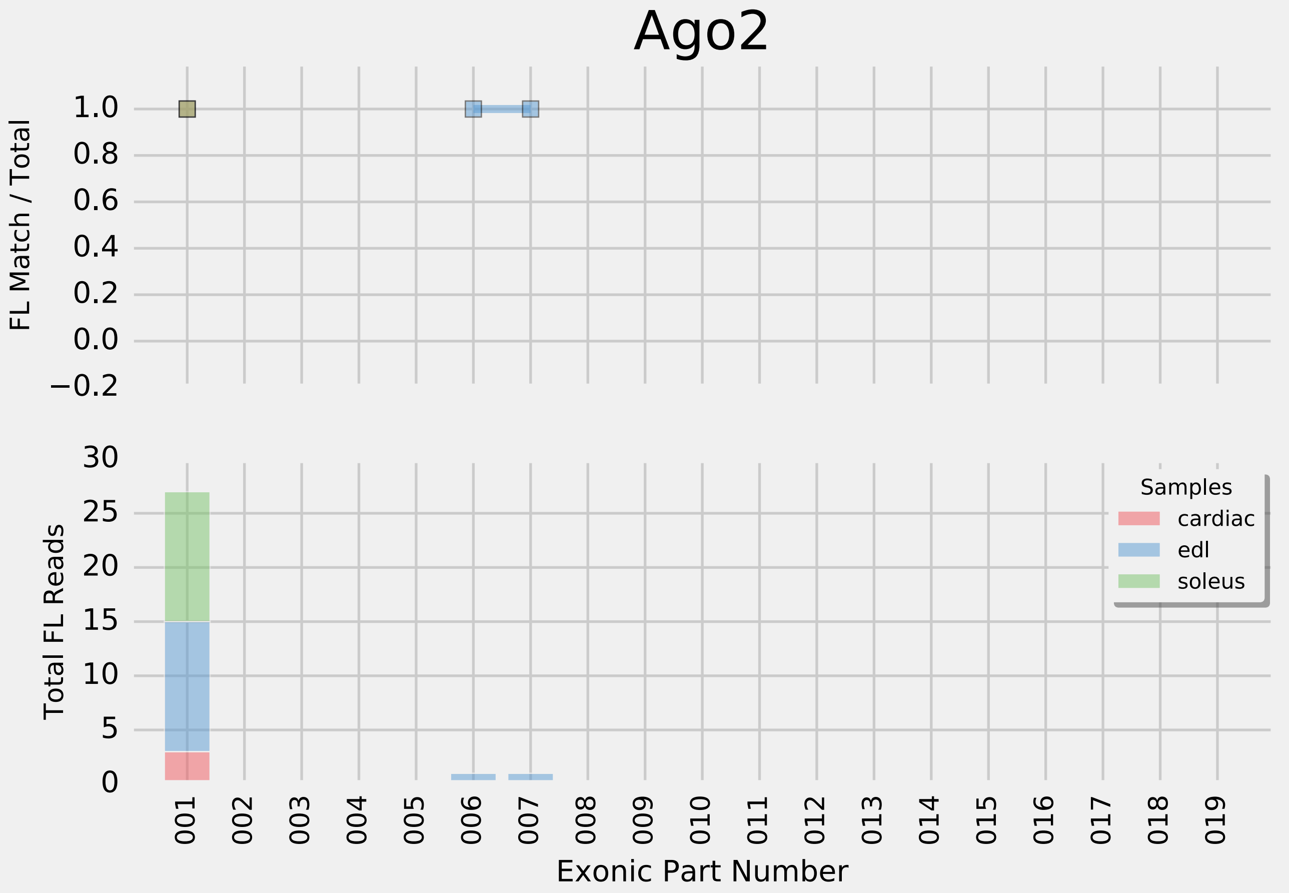
Ago2
FL Match / Total
1.0
0.8
0.6
0.4
0.2
0.0
−0.2
Total FL Reads
30
25
20
15
10
5
0
Samples
cardiac
edl
soleus
001
002
003
004
005
006
007
008
009
010
011
012
013
014
015
016
017
018
019
Exonic Part Number

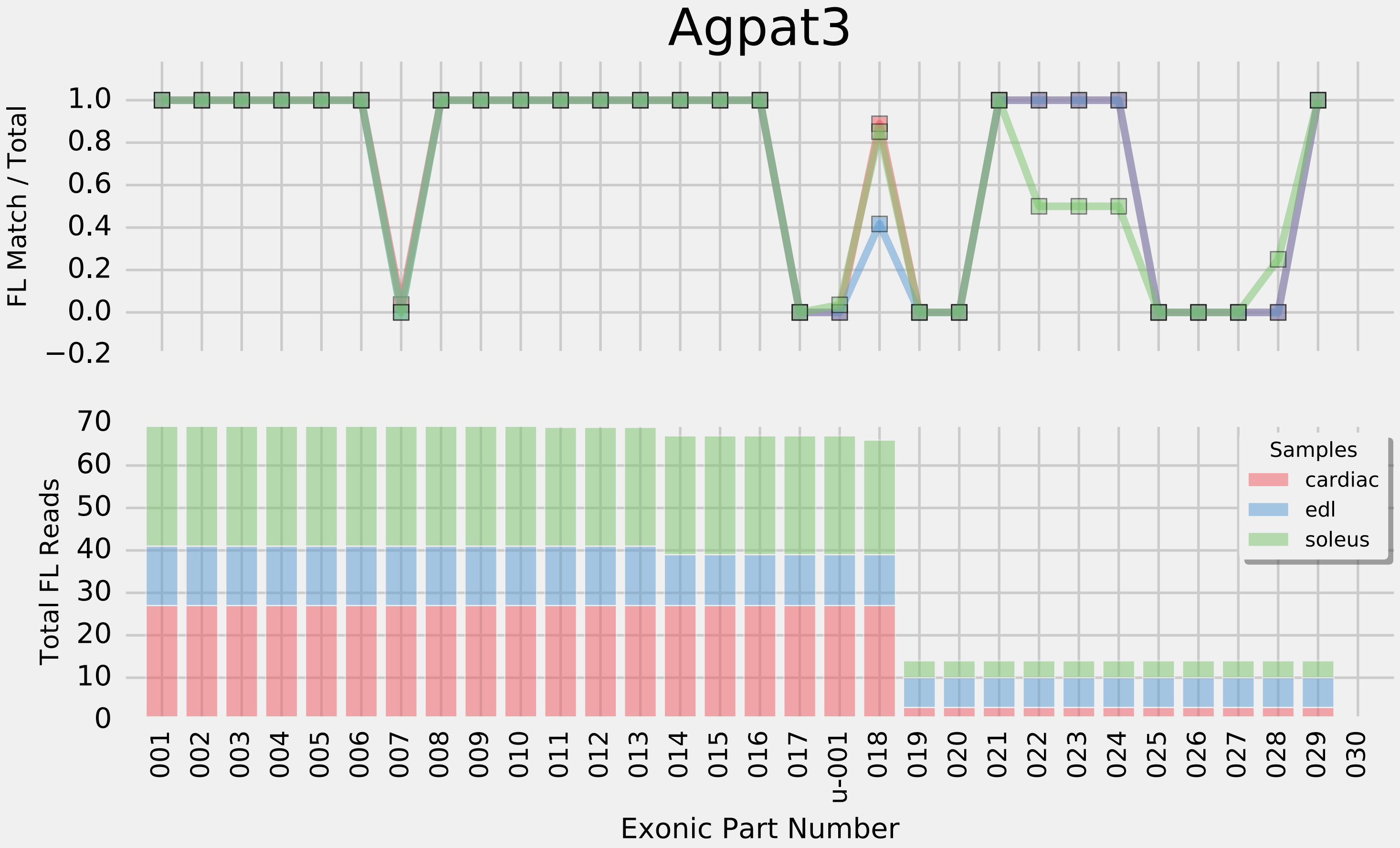

Agpat3
FL Match / Total
1.0
0.8
0.6
0.4
0.2
0.0
−0.2
Total FL Reads
70
60
50
40
30
20
10
0
Samples
cardiac
edl
soleus
001
002
003
004
005
006
007
008
009
010
011
012
013
014
015
016
017
u-001
018
019
020
021
022
023
024
025
026
027
028
029
030
Exonic Part Number

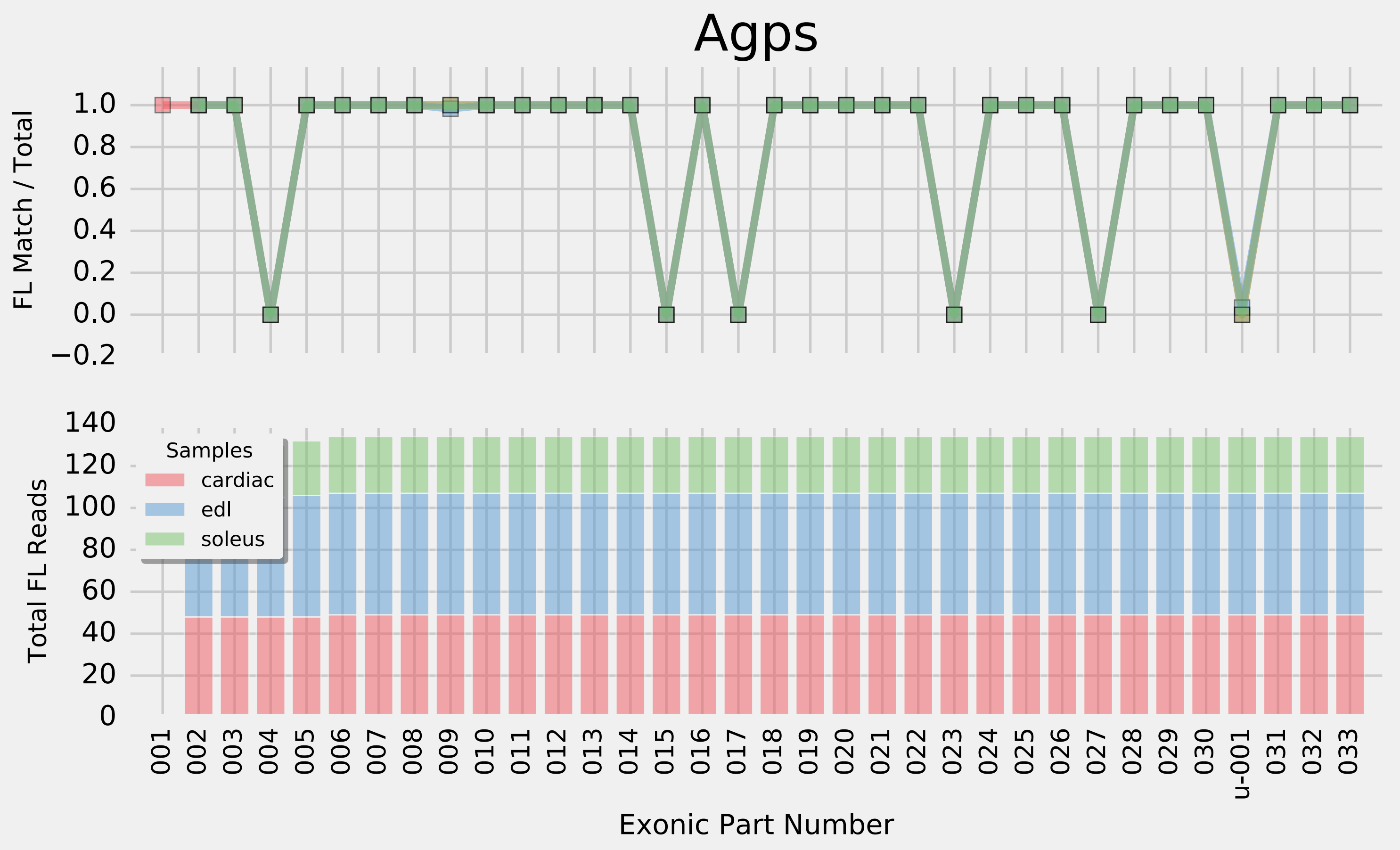
Agps
FL Match / Total
1.0
0.8
0.6
0.4
0.2
0.0
−0.2
Total FL Reads
140
120
100
80
60
40
20
0
Samples
cardiac
edl
soleus
001
002
003
004
005
006
007
008
009
010
011
012
013
014
015
016
017
018
019
020
021
022
023
024
025
026
027
028
029
030
u-001
031
032
033
Exonic Part Number

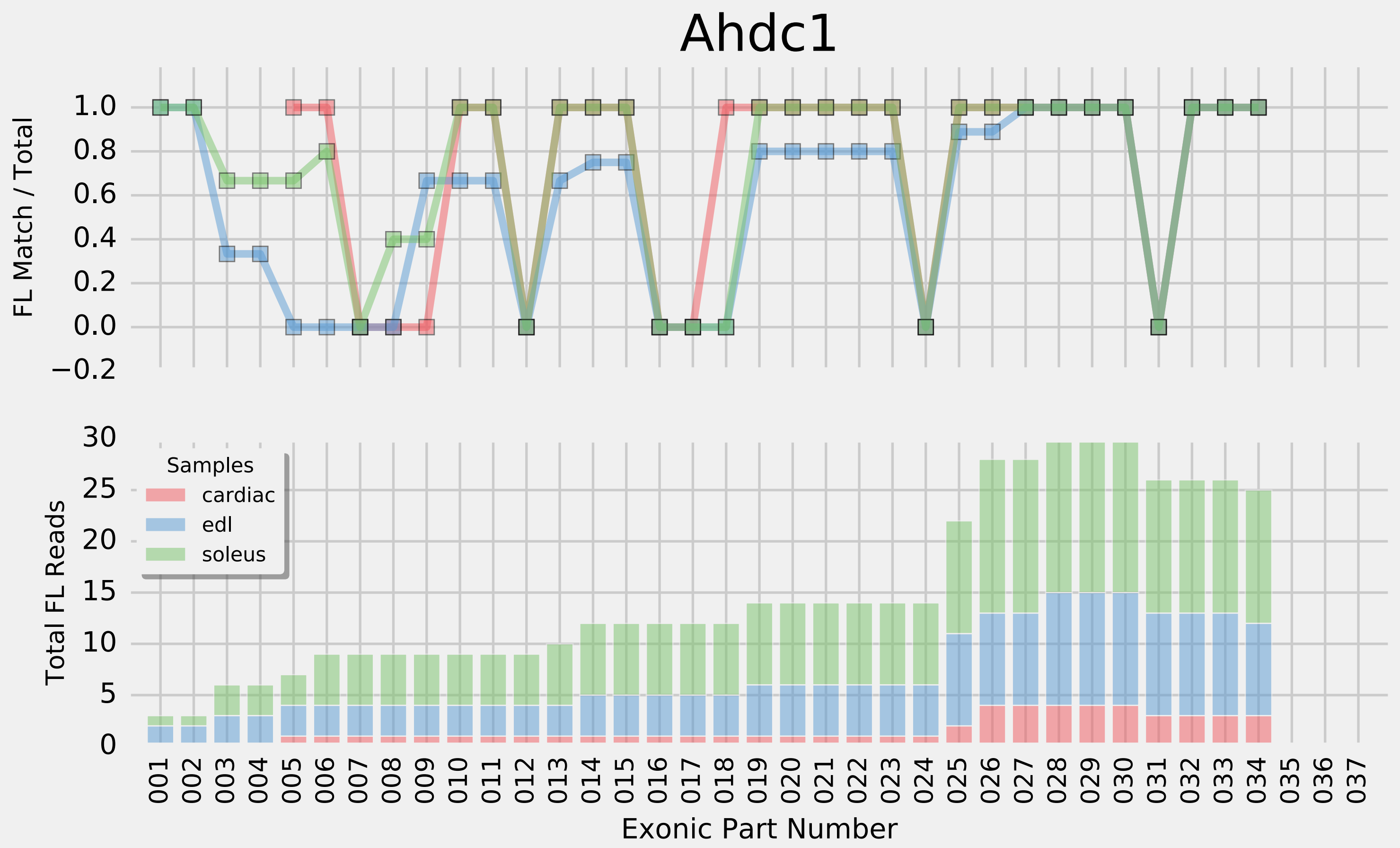
Ahdc1
FL Match / Total
1.0
0.8
0.6
0.4
0.2
0.0
−0.2
Total FL Reads
30
25
20
15
10
5
0
Samples
cardiac
edl
soleus
001 002 003 004 005 006 007 008 009 010 011 012 013 014 015 016 017 018 019 020 021 022 023 024 025 026 027 028 029 030 031 032 033 034 035 036 037
Exonic Part Number

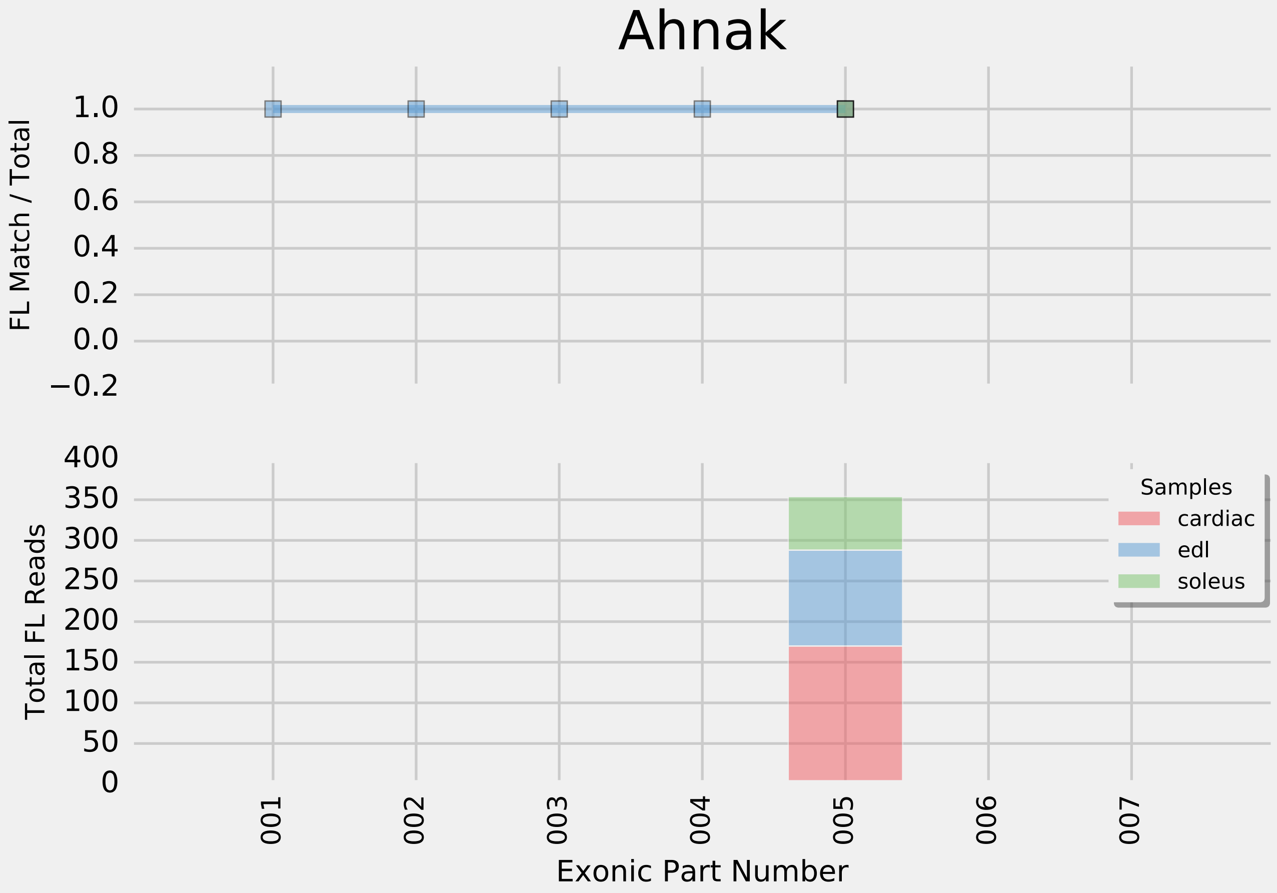
Ahnak
FL Match / Total
1.0
0.8
0.6
0.4
0.2
0.0
−0.2
Total FL Reads
400
350
300
250
200
150
100
50
0
Samples
cardiac
edl
soleus
001
002
003
004
005
006
007
Exonic Part Number

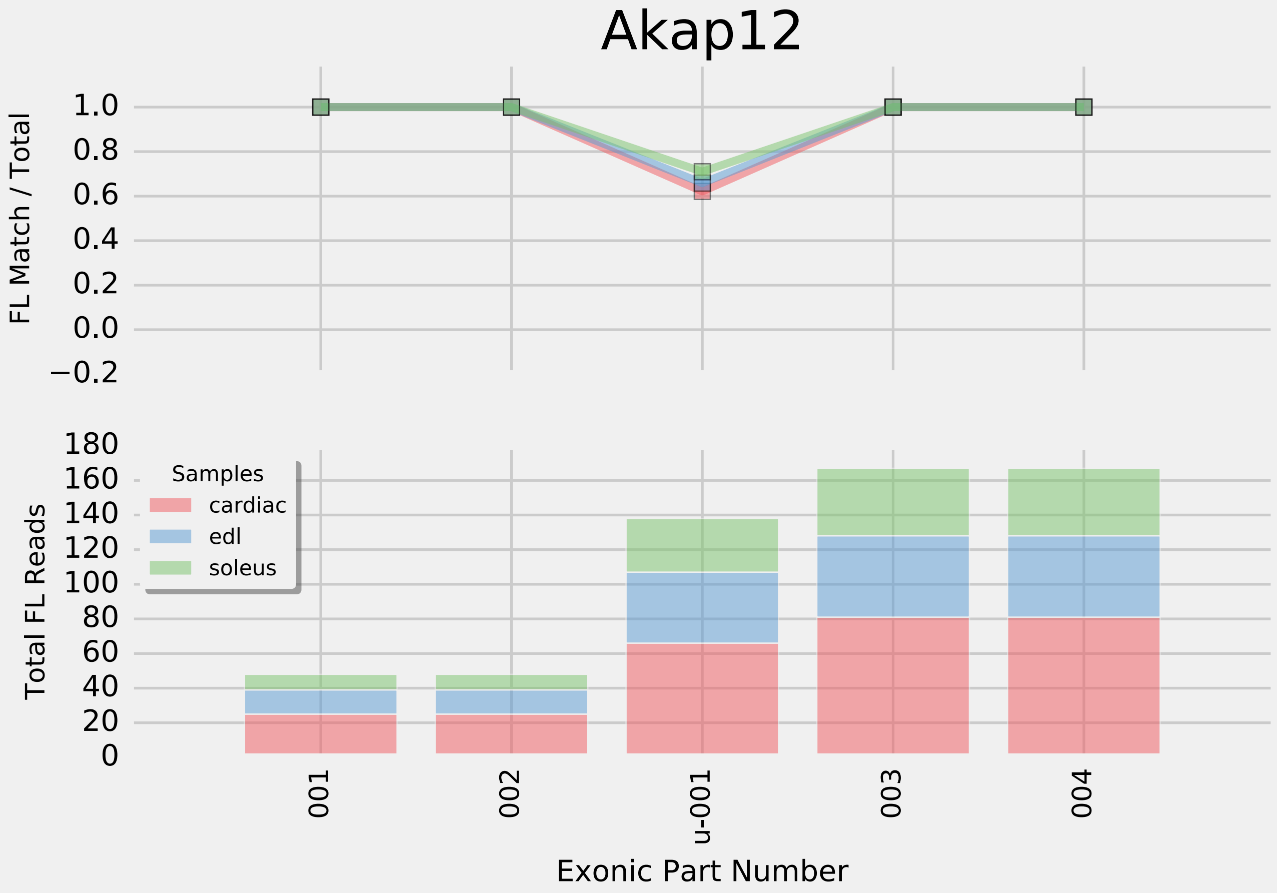
Akap12
FL Match / Total
1.0
0.8
0.6
0.4
0.2
0.0
−0.2
Total FL Reads
180
160
140
120
100
80
60
40
20
0
Samples
cardiac
edl
soleus
001
002
u-001
003
004
Exonic Part Number

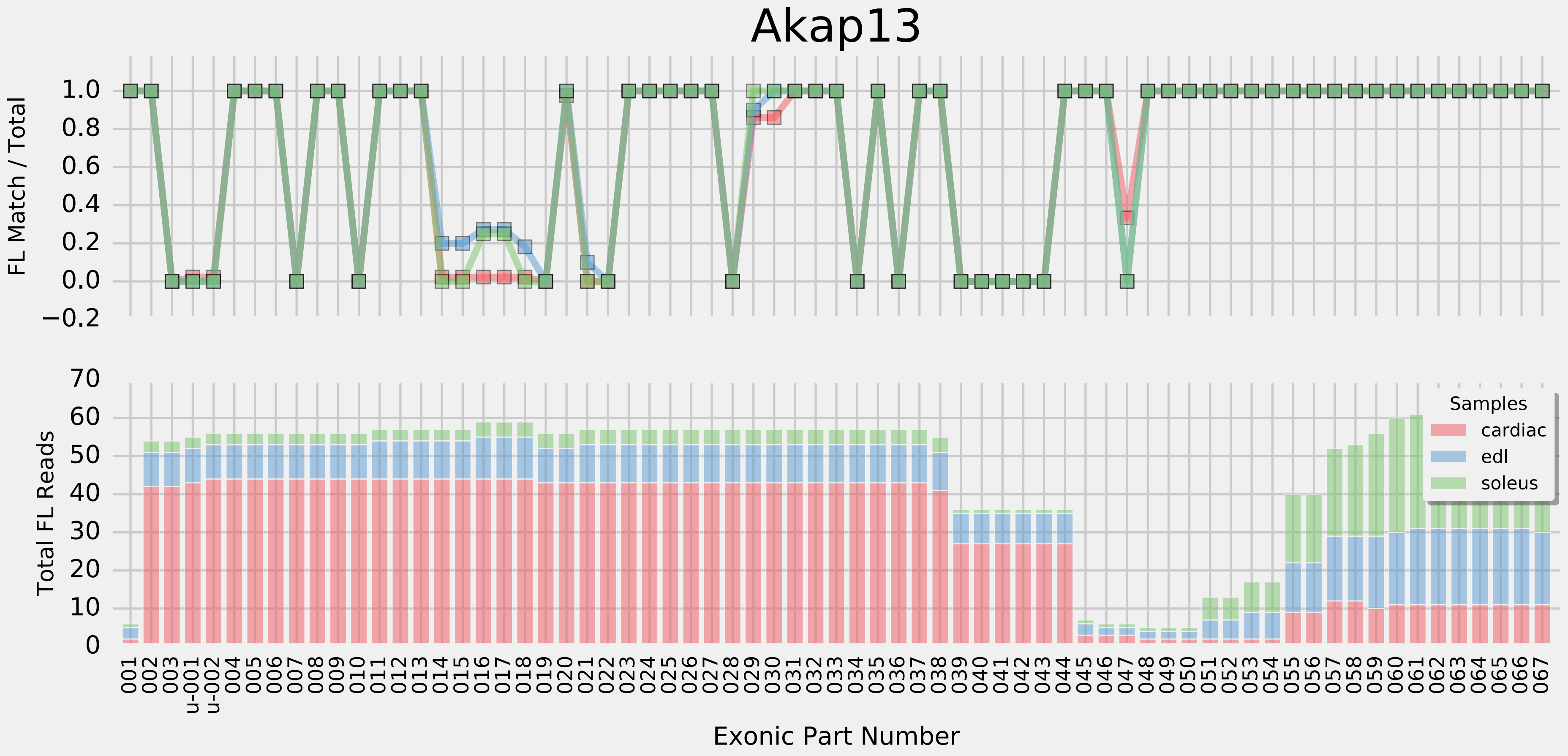

Akap13
FL Match / Total
1.0
0.8
0.6
0.4
0.2
0.0
−0.2
Total FL Reads
70
60
50
40
30
20
10
0
Samples
cardiac
edl
soleus
001 002 003 u-001 u-002 004 005 006 007 008 009 010 011 012 013 014 015 016 017 018 019 020 021 022 023 024 025 026 027 028 029 030 031 032 033 034 035 036 037 038 039 040 041 042 043 044 045 046 047 048 049 050 051 052 053 054 055 056 057 058 059 060 061 062 063 064 065 066 067
Exonic Part Number

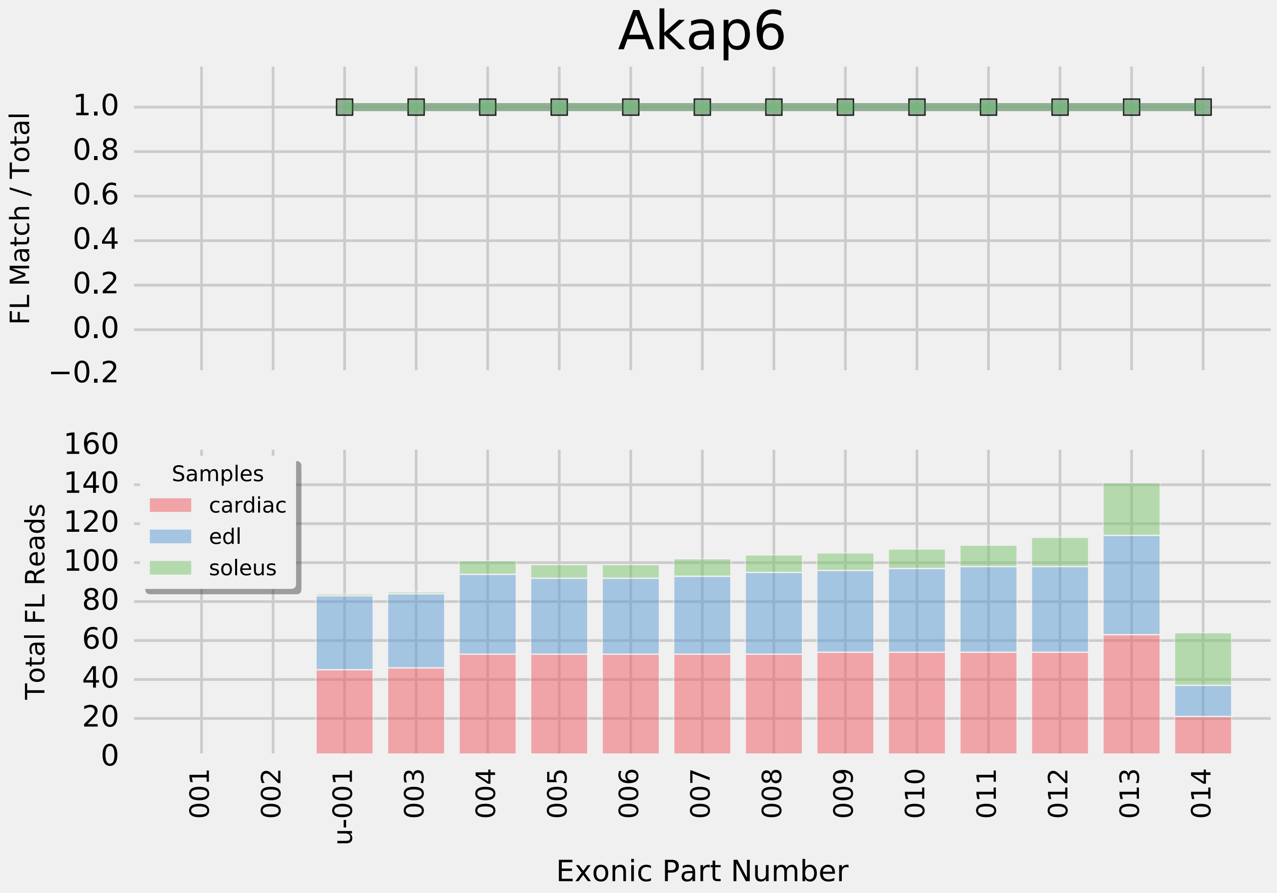
Akap6
FL Match / Total
1.0
0.8
0.6
0.4
0.2
0.0
−0.2
Total FL Reads
160
140
120
100
80
60
40
20
0
Samples
cardiac
edl
soleus
001
002
u-001
003
004
005
006
007
008
009
010
011
012
013
014
Exonic Part Number

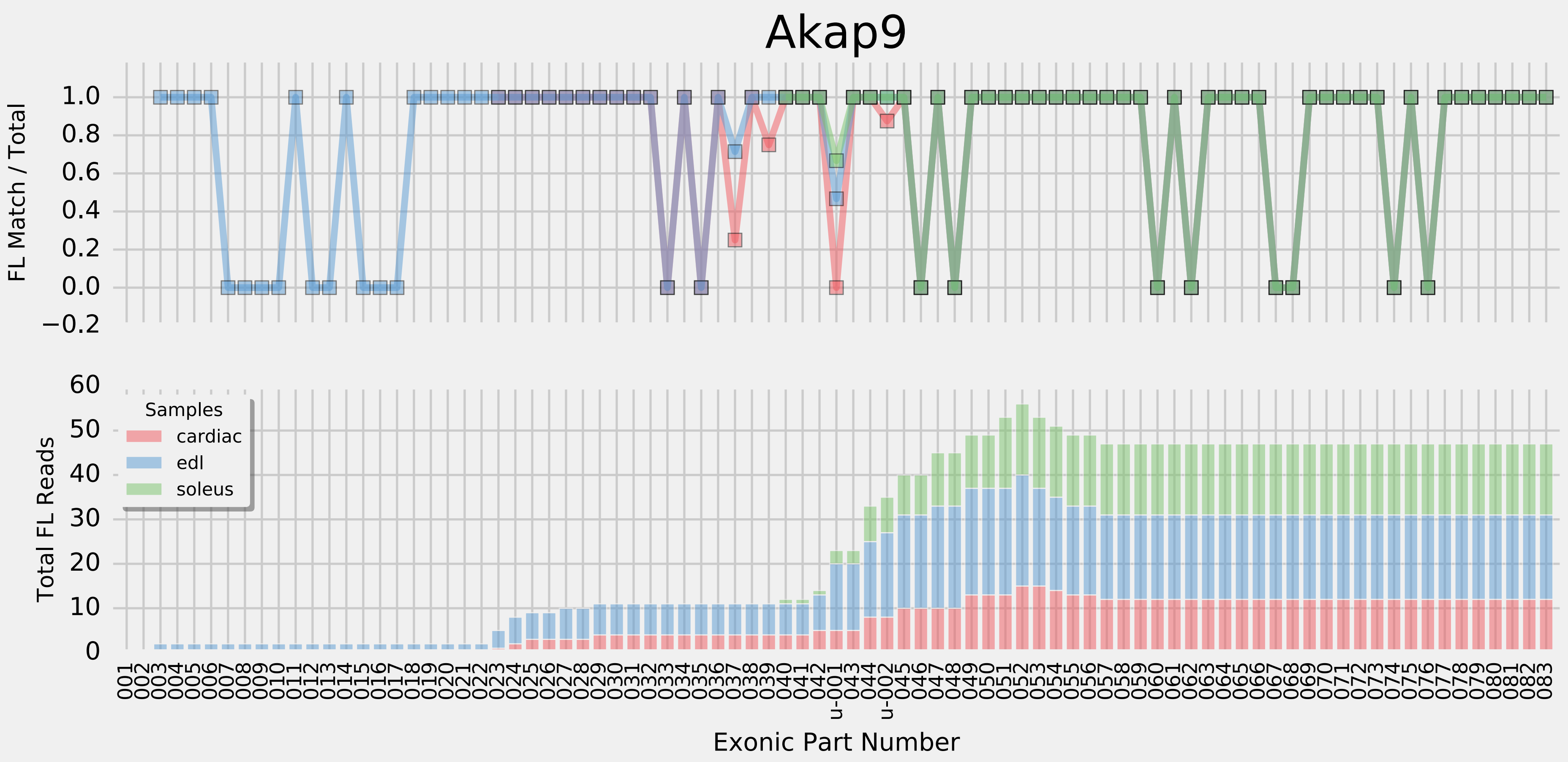

Akap9
FL Match / Total
Total FL Reads
Samples
cardiac
edl
soleus
Exonic Part Number

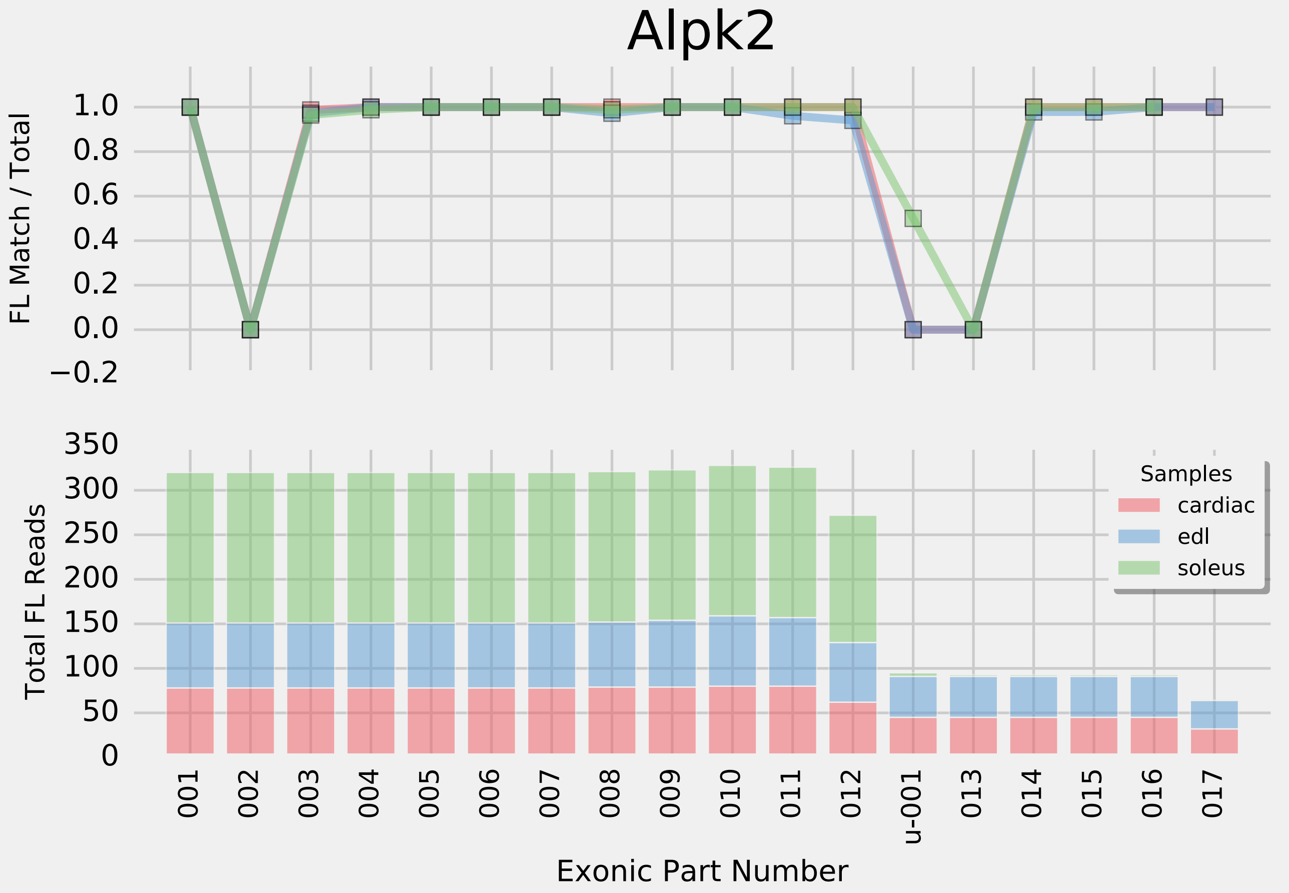

Alpk2
FL Match / Total
1.0
0.8
0.6
0.4
0.2
0.0
−0.2
Total FL Reads
350
300
250
200
150
100
50
0
Samples
cardiac
edl
soleus
001
002
003
004
005
006
007
008
009
010
011
012
u-001
013
014
015
016
017
Exonic Part Number

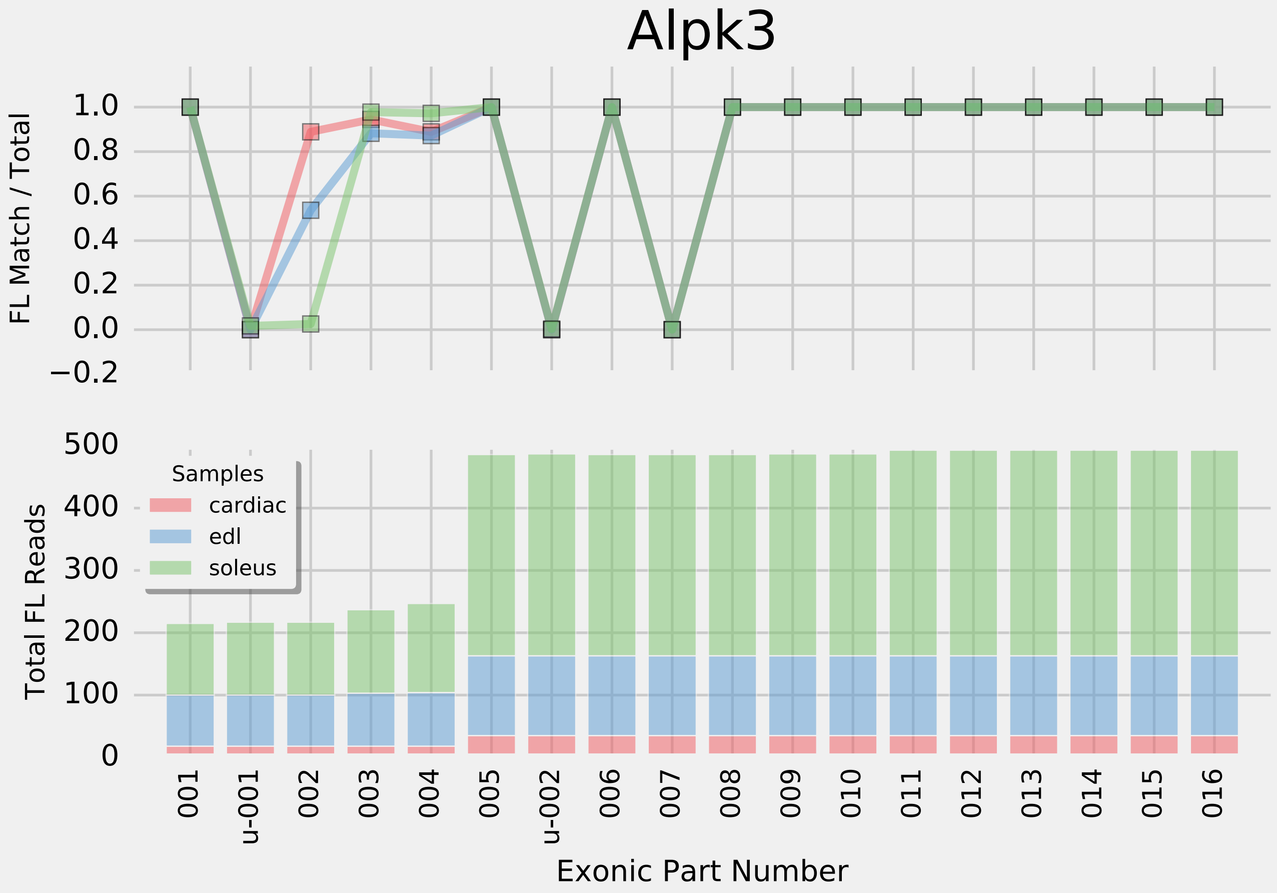

Alpk3
FL Match / Total
1.0
0.8
0.6
0.4
0.2
0.0
−0.2
Total FL Reads
500
400
300
200
100
0
Samples
cardiac
edl
soleus
001
u-001
002
003
004
005
u-002
006
007
008
009
010
011
012
013
014
015
016
Exonic Part Number

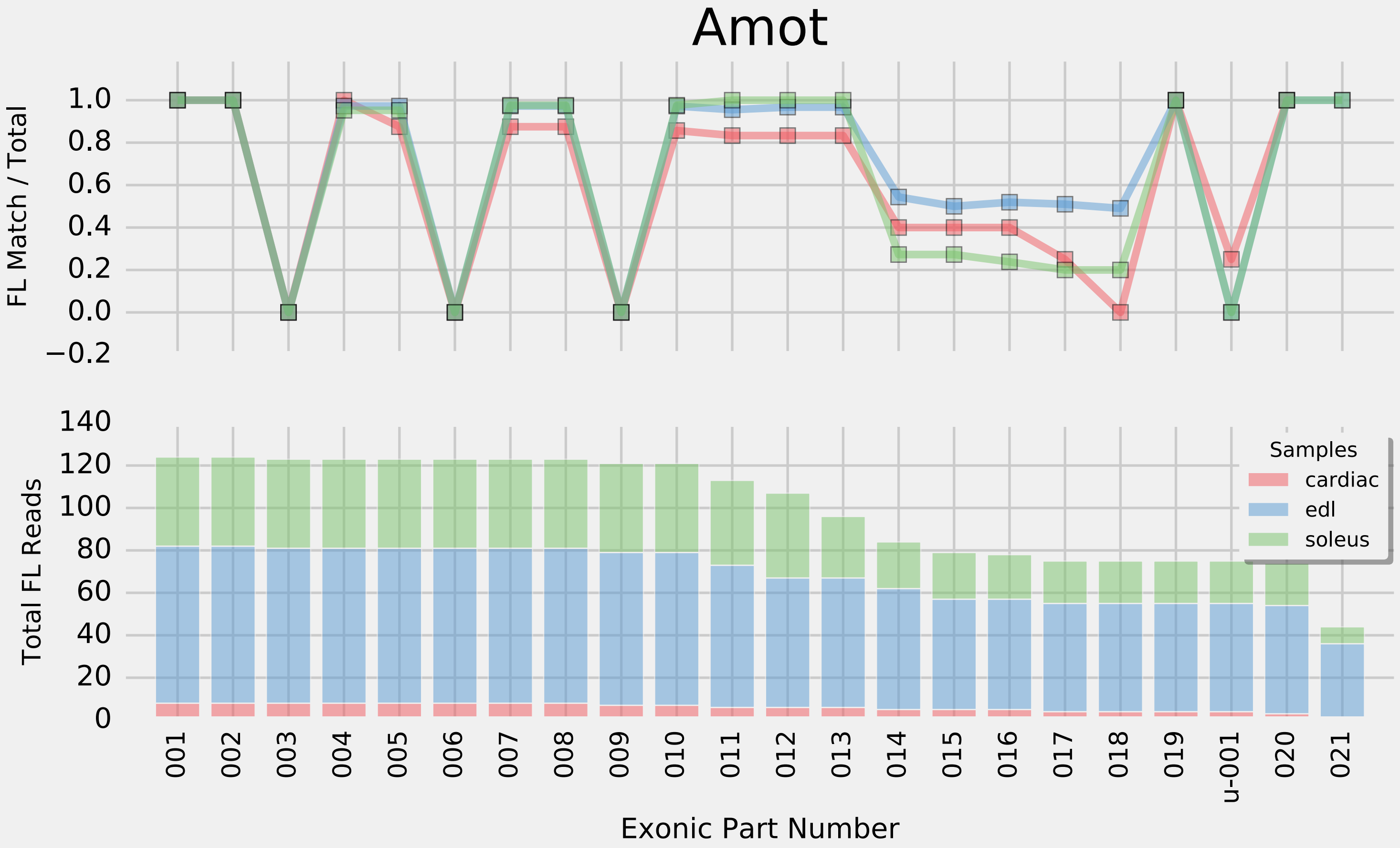

Amot
FL Match / Total
1.0
0.8
0.6
0.4
0.2
0.0
−0.2
Total FL Reads
140
120
100
80
60
40
20
0
Samples
cardiac
edl
soleus
001
002
003
004
005
006
007
008
009
010
011
012
013
014
015
016
017
018
019
u-001
020
021
Exonic Part Number

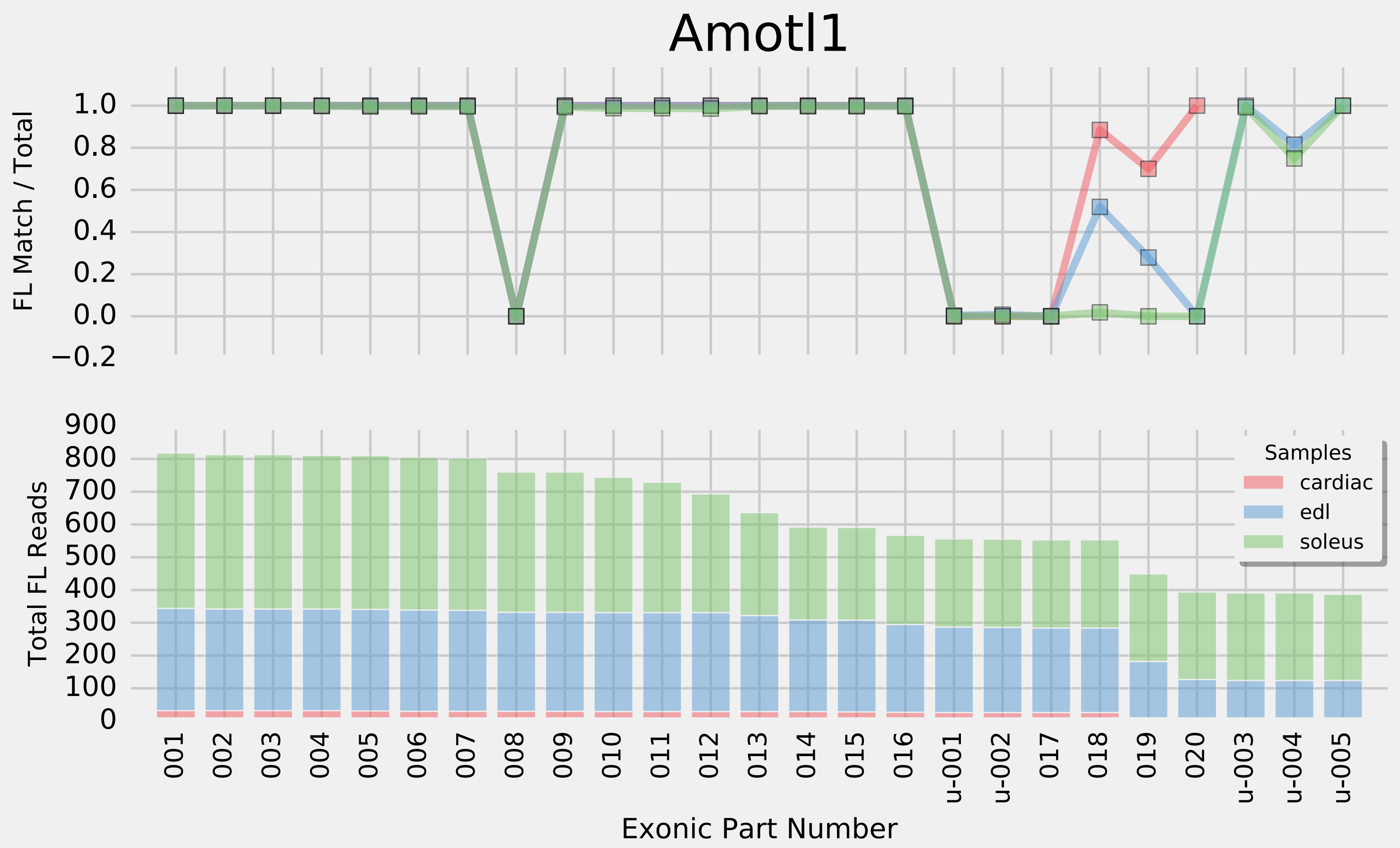

Amotl1
FL Match / Total
1.0
0.8
0.6
0.4
0.2
0.0
−0.2
Total FL Reads
900
800
700
600
500
400
300
200
100
0
Samples
cardiac
edl
soleus
001
002
003
004
005
006
007
008
009
010
011
012
013
014
015
016
u-001
u-002
017
018
019
020
u-003
u-004
u-005
Exonic Part Number

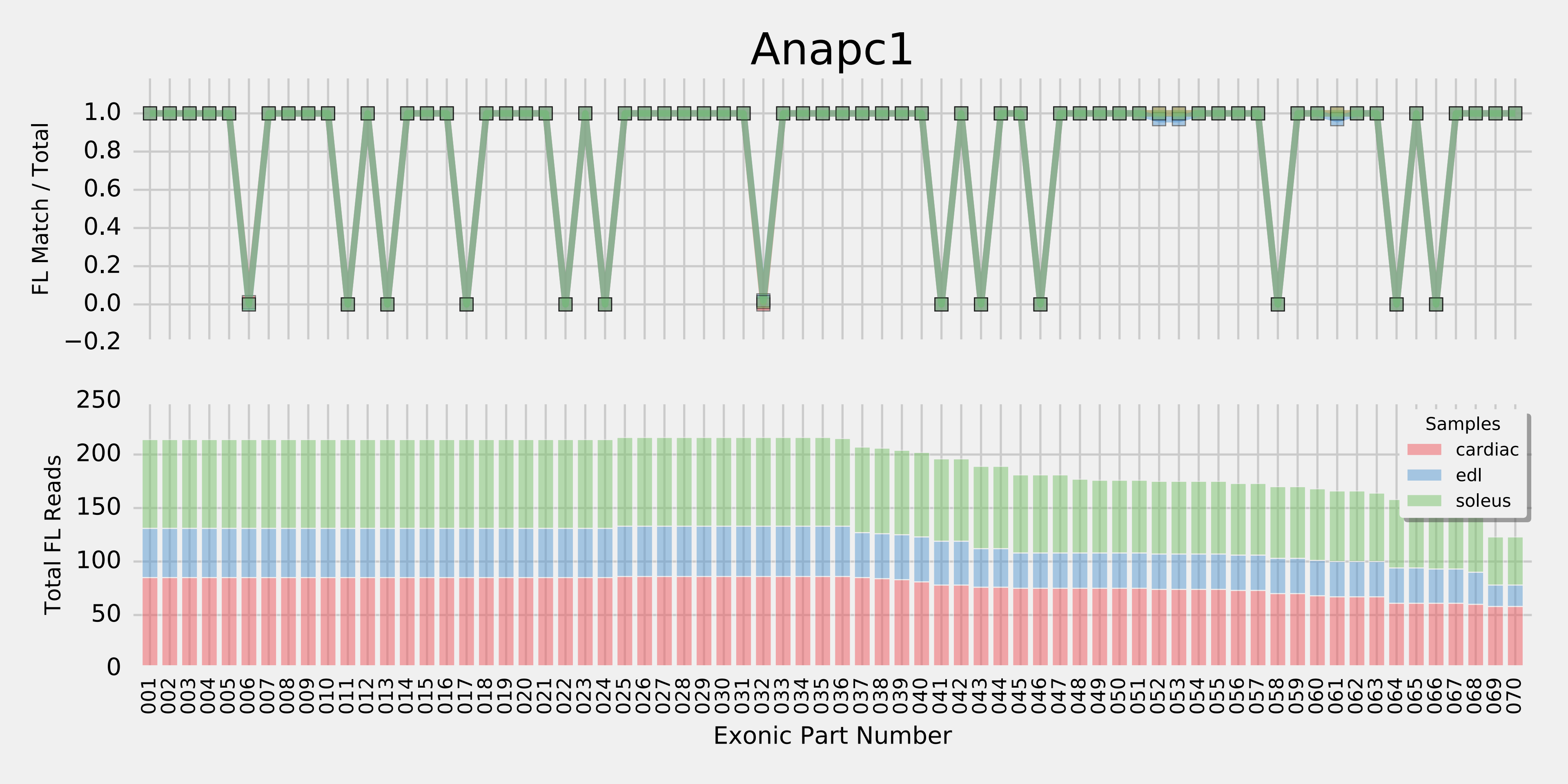

Anapc1
FL Match / Total
Total FL Reads
Samples
cardiac
edl
soleus
Exonic Part Number

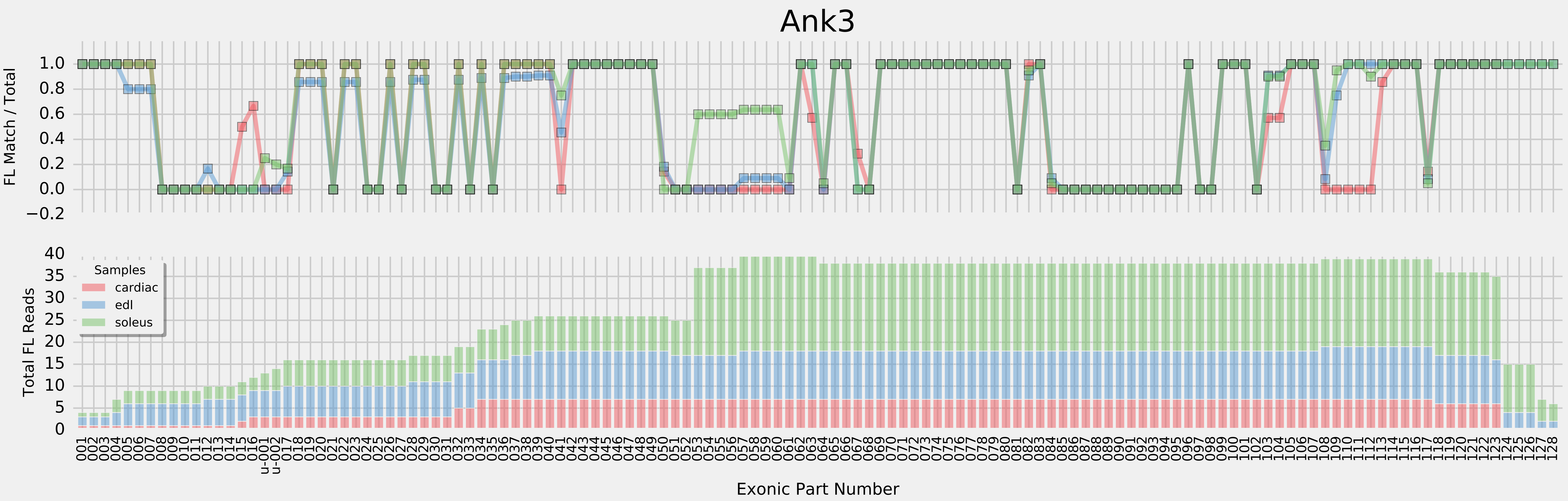
Ank3
FL Match / Total
Total FL Reads
Samples
cardiac
edl
soleus
Exonic Part Number

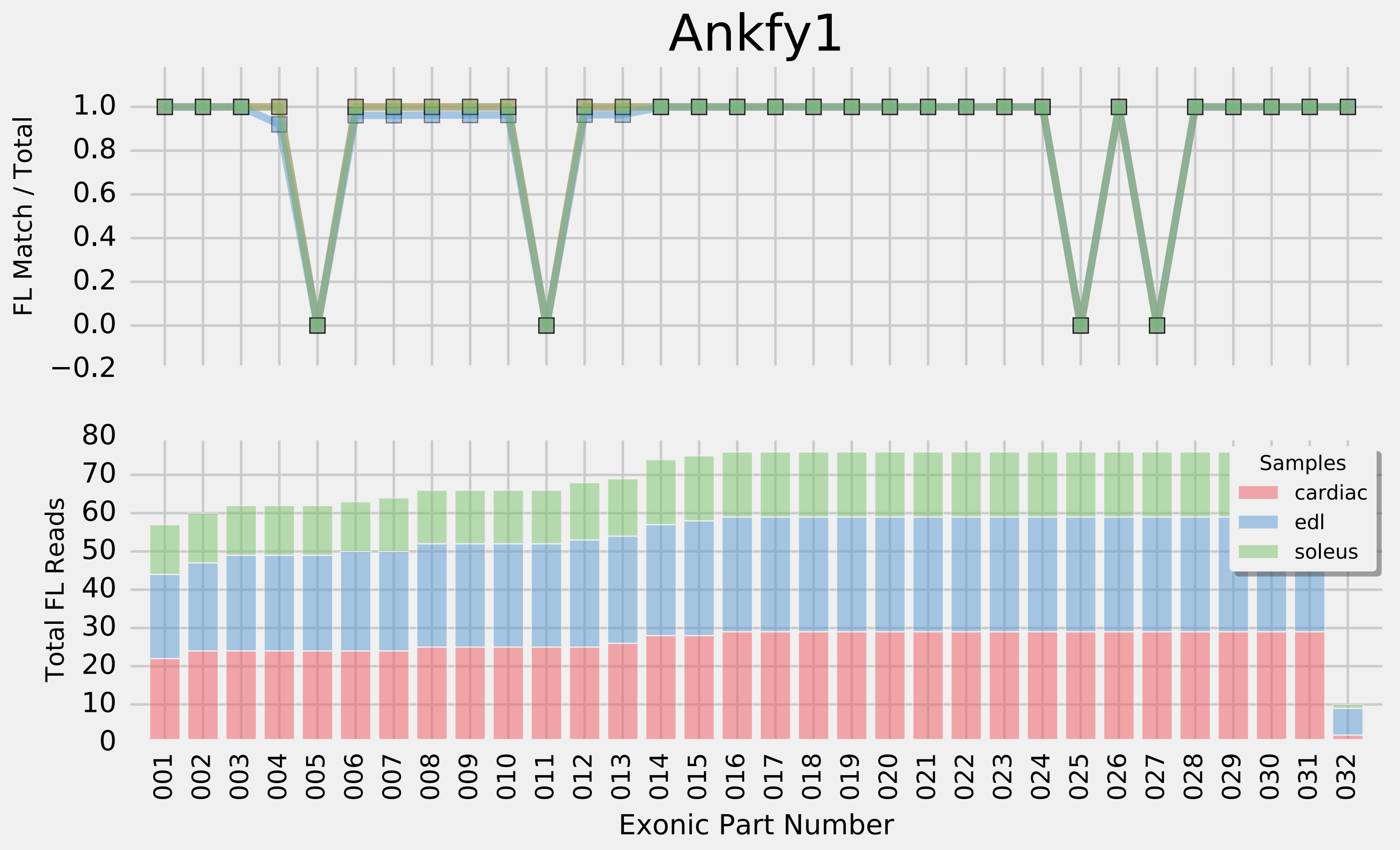

Ankfy1
FL Match / Total
1.0
0.8
0.6
0.4
0.2
0.0
−0.2
Total FL Reads
80
70
60
50
40
30
20
10
0
Samples
cardiac
edl
soleus
001 002 003 004 005 006 007 008 009 010 011 012 013 014 015 016 017 018 019 020 021 022 023 024 025 026 027 028 029 030 031 032
Exonic Part Number

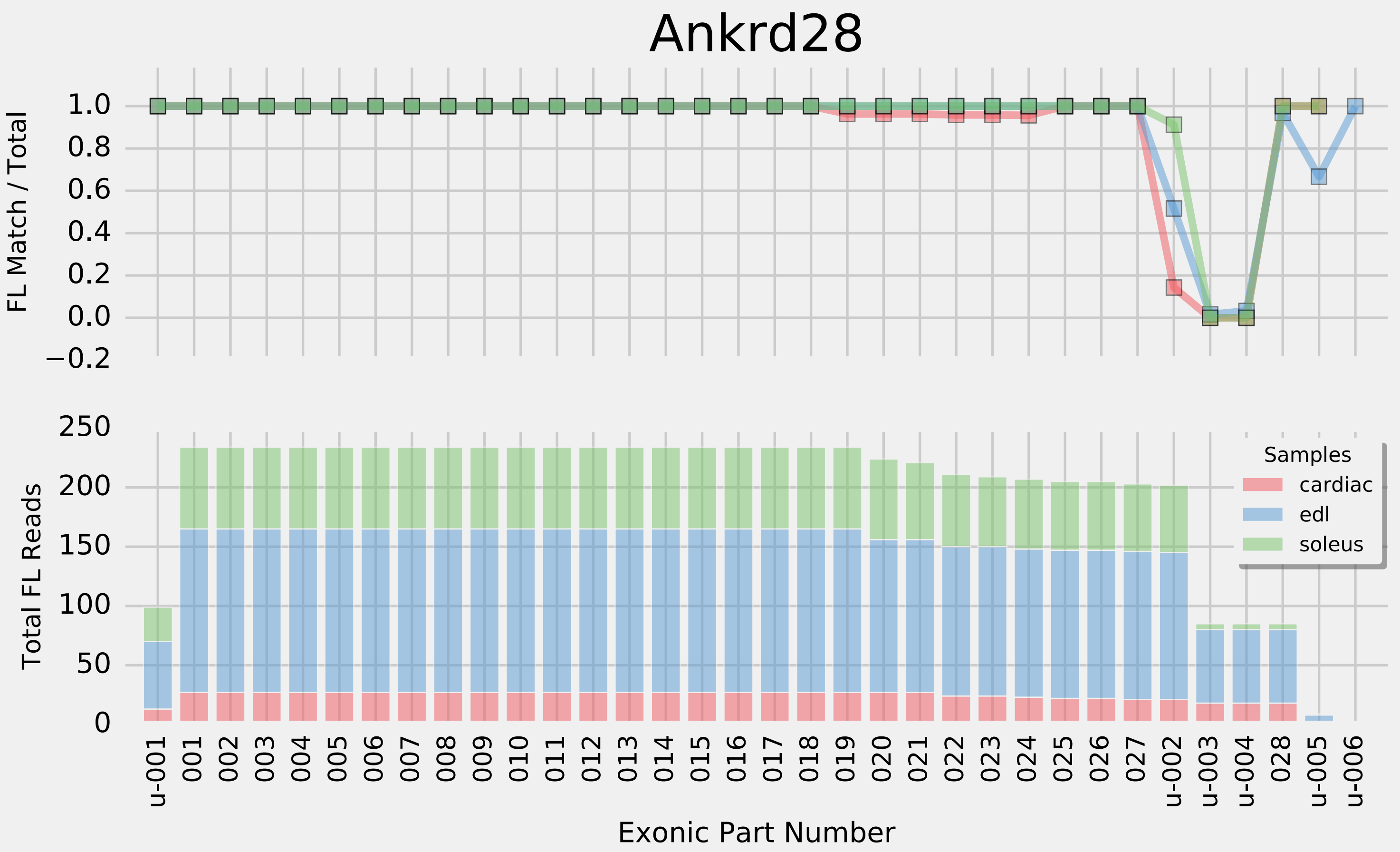

Ankrd28
FL Match / Total
1.0
0.8
0.6
0.4
0.2
0.0
−0.2
Total FL Reads
250
200
150
100
50
0
Samples
cardiac
edl
soleus
u-001
001
002
003
004
005
006
007
008
009
010
011
012
013
014
015
016
017
018
019
020
021
022
023
024
025
026
027
u-002
u-003
u-004
028
u-005
u-006
Exonic Part Number

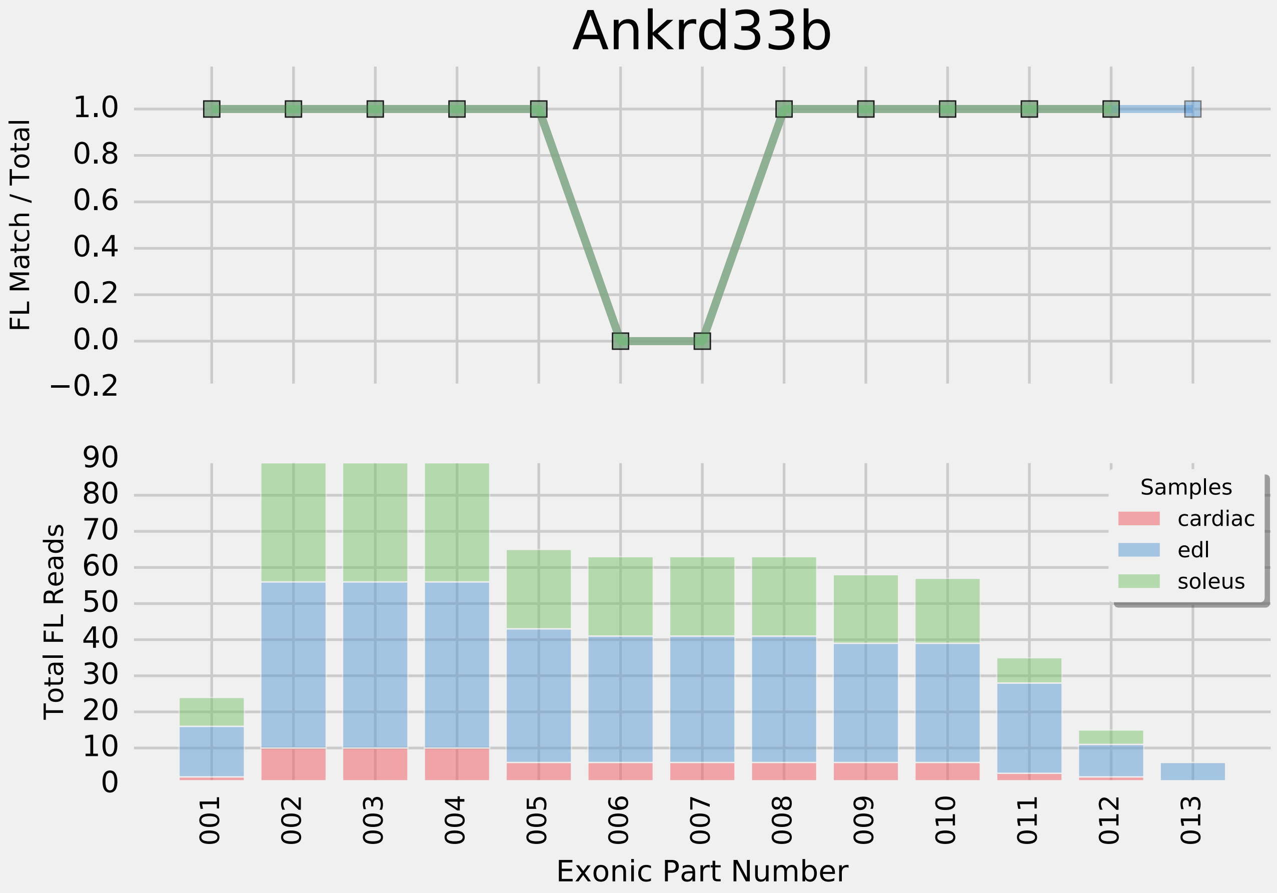

Ankrd33b
FL Match / Total
1.0
0.8
0.6
0.4
0.2
0.0
−0.2
Total FL Reads
90
80
70
60
50
40
30
20
10
0
Samples
cardiac
edl
soleus
001
002
003
004
005
006
007
008
009
010
011
012
013
Exonic Part Number

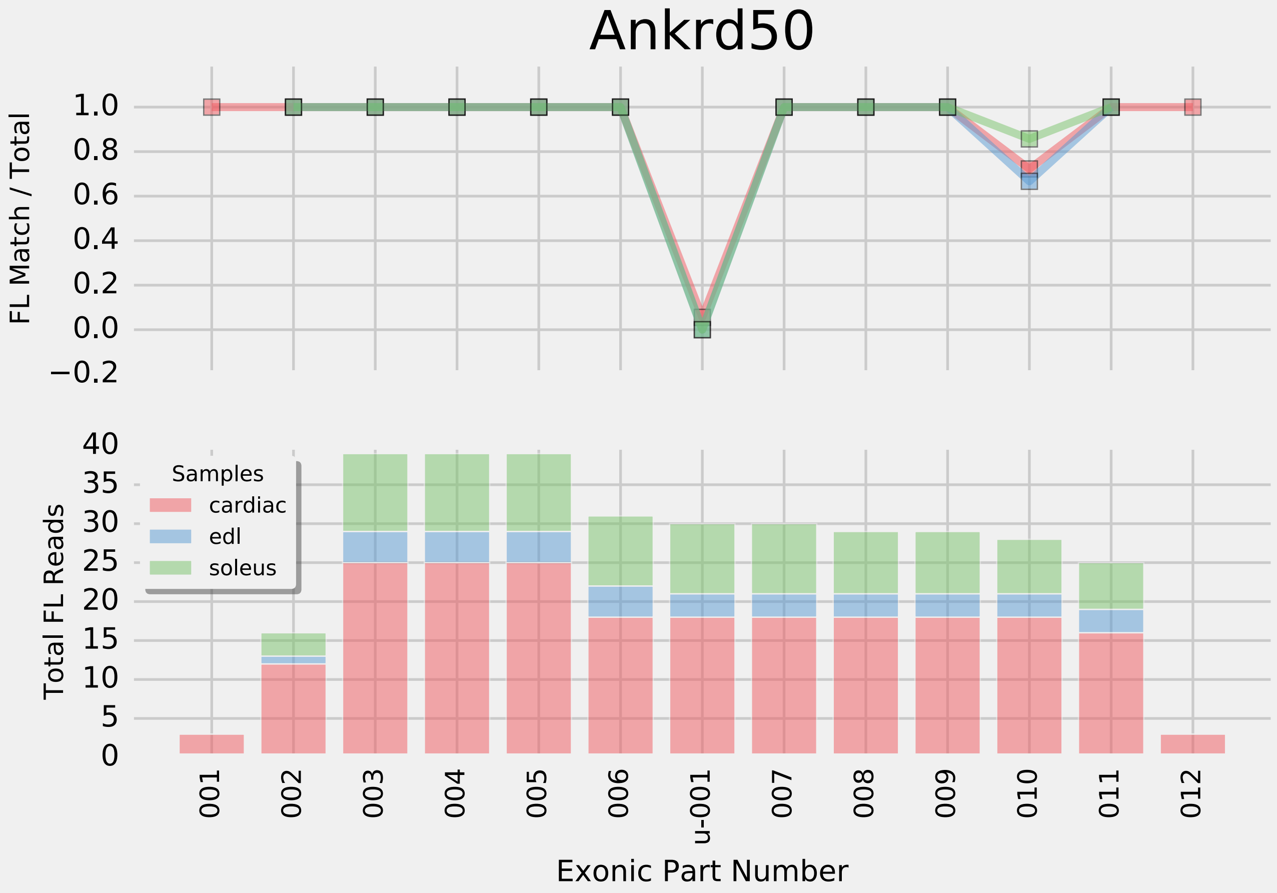

Ankrd50
FL Match / Total
1.0
0.8
0.6
0.4
0.2
0.0
−0.2
40
35
30
25
20
15
10
5
0
Total FL Reads
Samples
cardiac
edl
soleus
001
002
003
004
005
006
u-001
007
008
009
010
011
012
Exonic Part Number

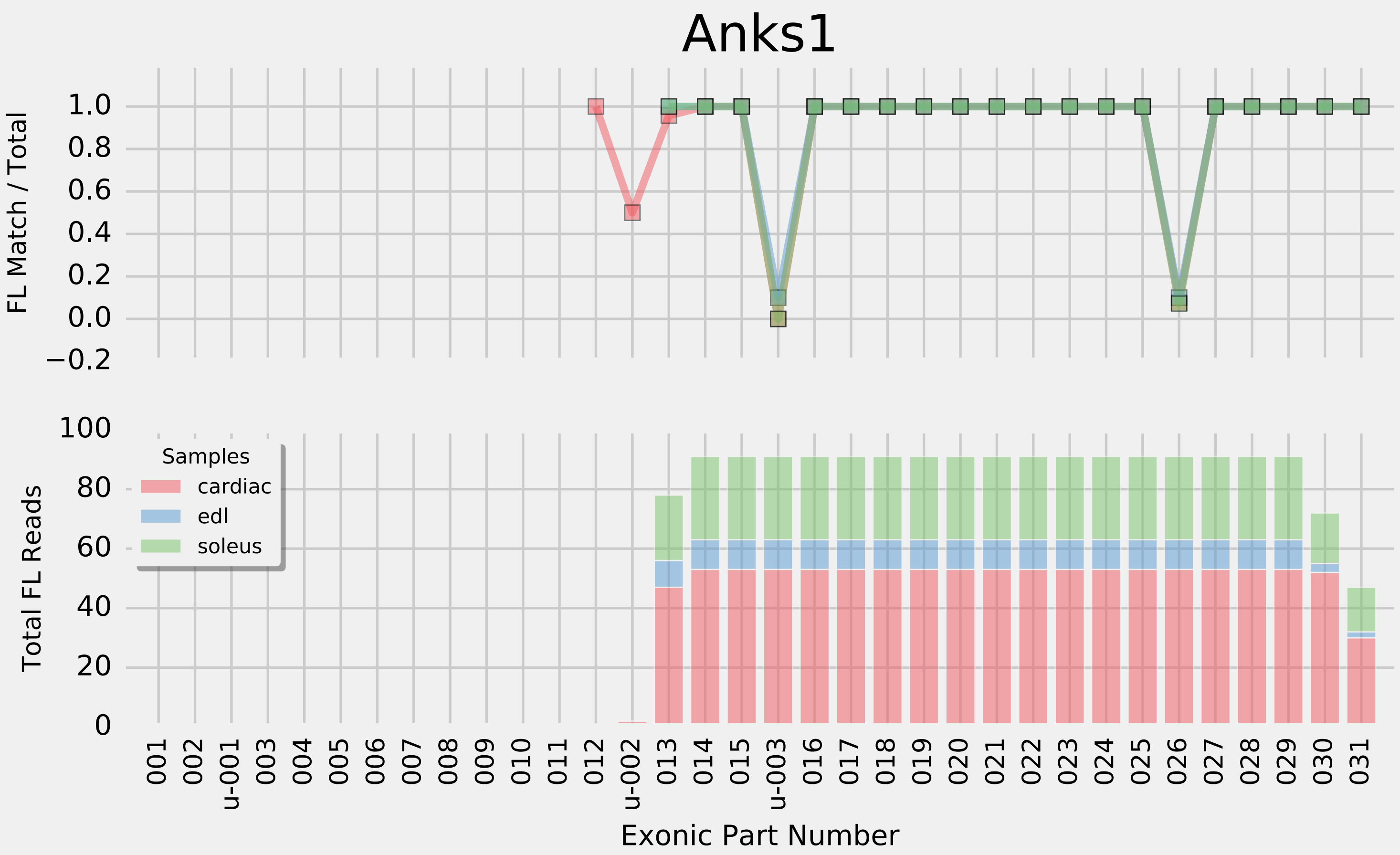
Anks1
FL Match / Total
1.0
0.8
0.6
0.4
0.2
0.0
−0.2
Total FL Reads
100
80
60
40
20
0
Samples
cardiac
edl
soleus
001
002
u-001
003
004
005
006
007
008
009
010
011
012
u-002
013
014
015
u-003
016
017
018
019
020
021
022
023
024
025
026
027
028
029
030
031
Exonic Part Number

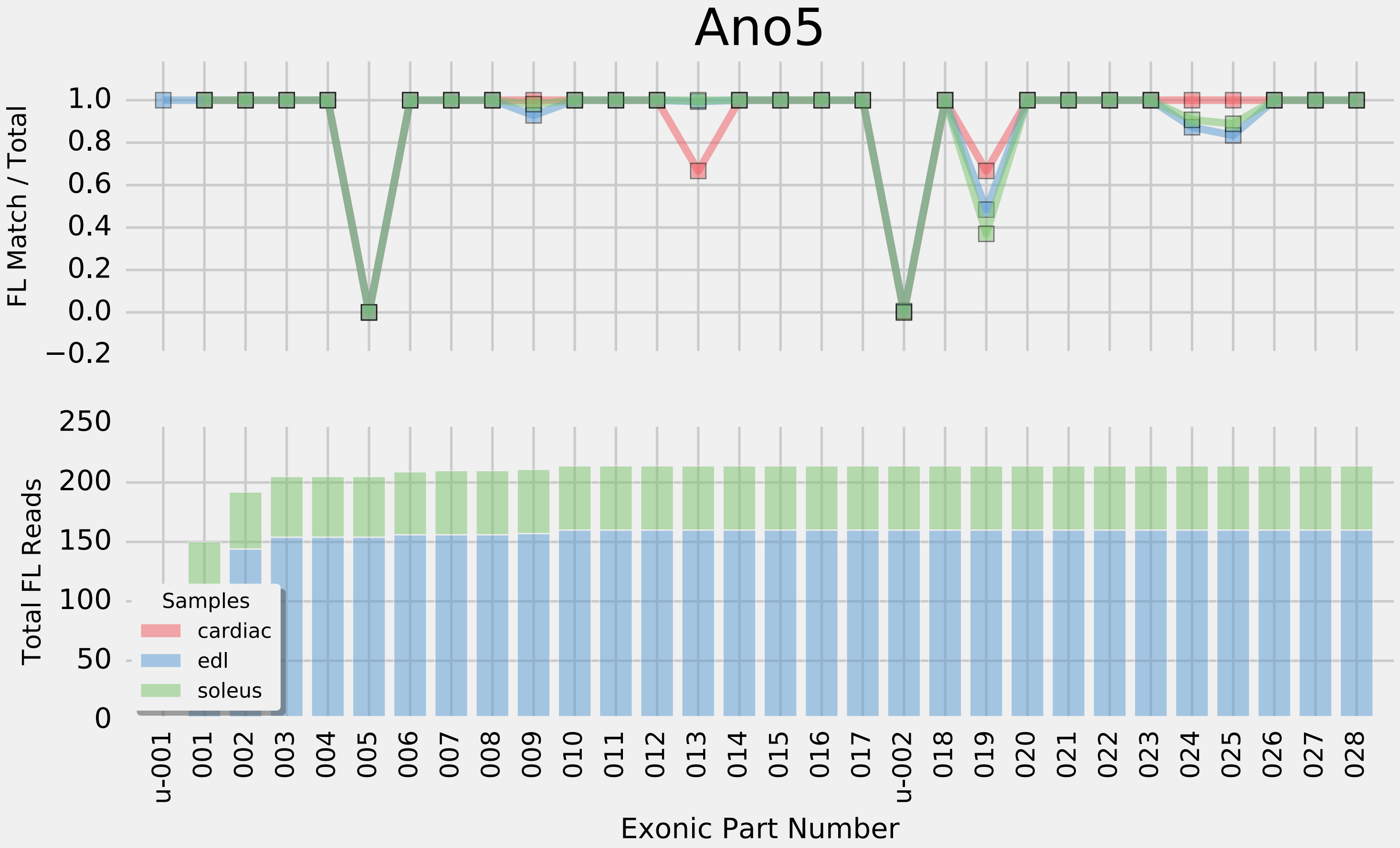

Ano5
FL Match / Total
1.0
0.8
0.6
0.4
0.2
0.0
−0.2
250
200
150
100
50
0
Total FL Reads
Samples
cardiac
edl
soleus
u-001
001
002
003
004
005
006
007
008
009
010
011
012
013
014
015
016
017
u-002
018
019
020
021
022
023
024
025
026
027
028
Exonic Part Number

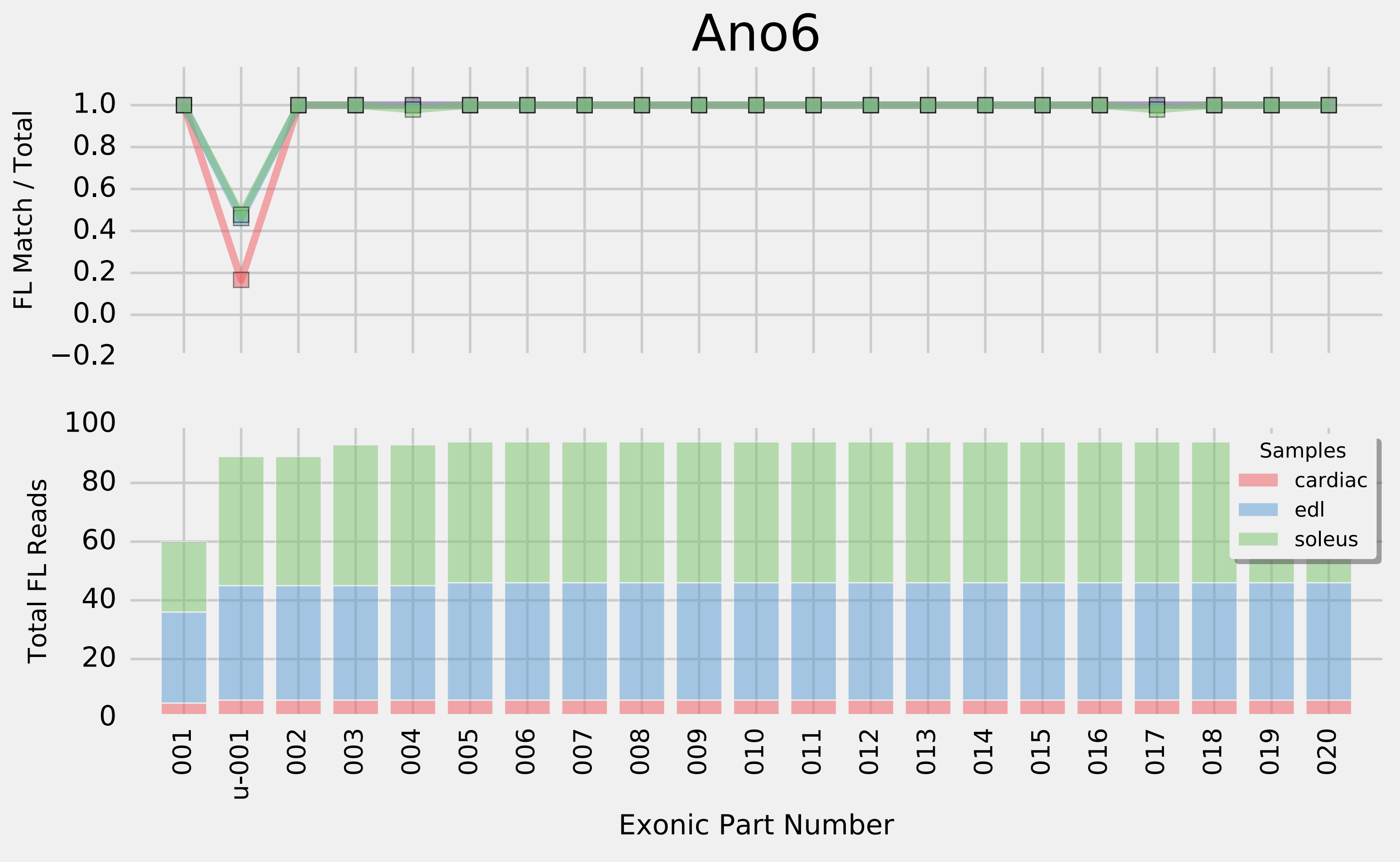

Ano6
FL Match / Total
1.0
0.8
0.6
0.4
0.2
0.0
−0.2
Total FL Reads
100
80
60
40
20
0
Samples
cardiac
edl
soleus
001
u-001
002
003
004
005
006
007
008
009
010
011
012
013
014
015
016
017
018
019
020
Exonic Part Number

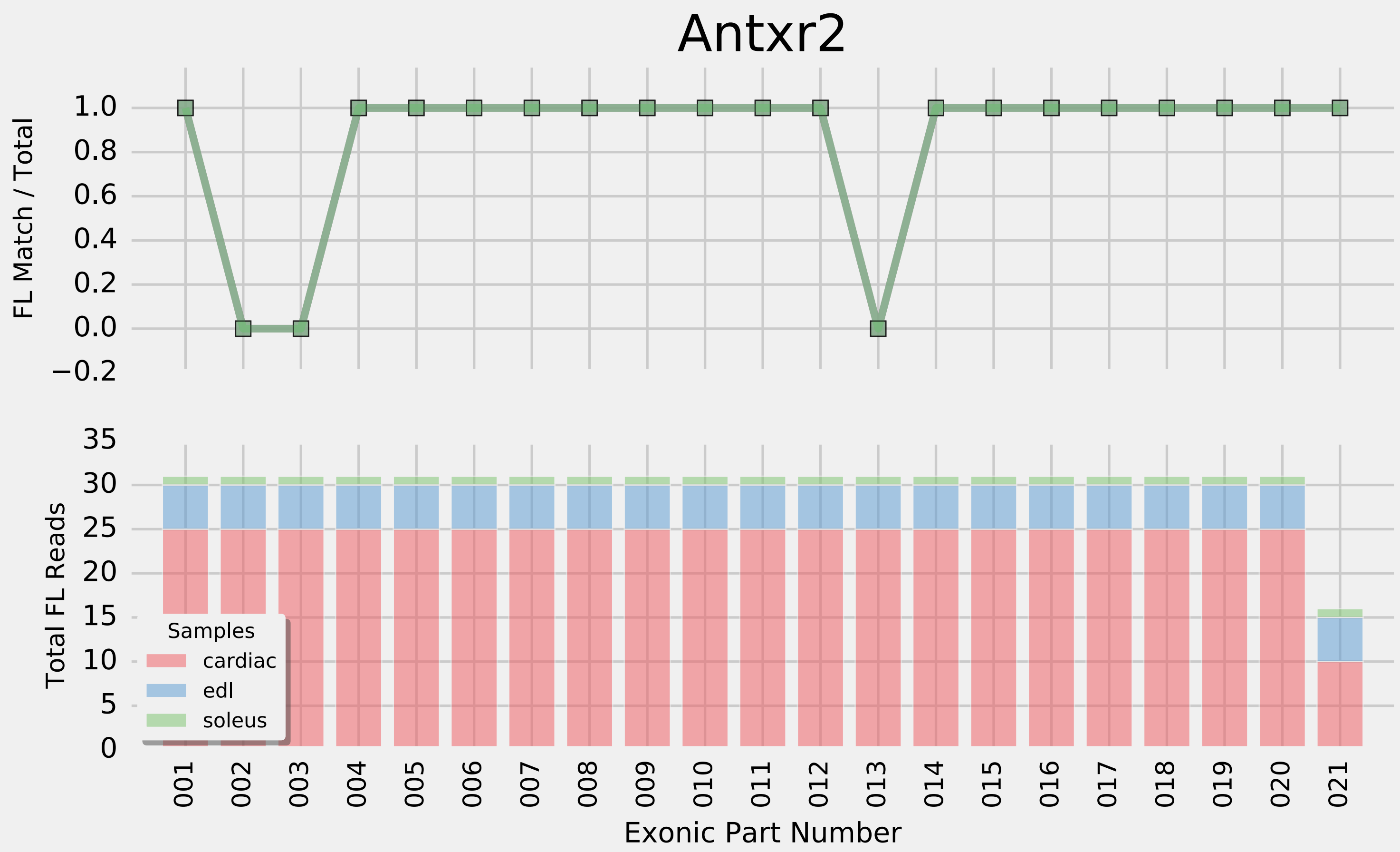
Antxr2
FL Match / Total
1.0
0.8
0.6
0.4
0.2
0.0
−0.2
Total FL Reads
35
30
25
20
15
10
5
0
Samples
cardiac
edl
soleus
001
002
003
004
005
006
007
008
009
010
011
012
013
014
015
016
017
018
019
020
021
Exonic Part Number

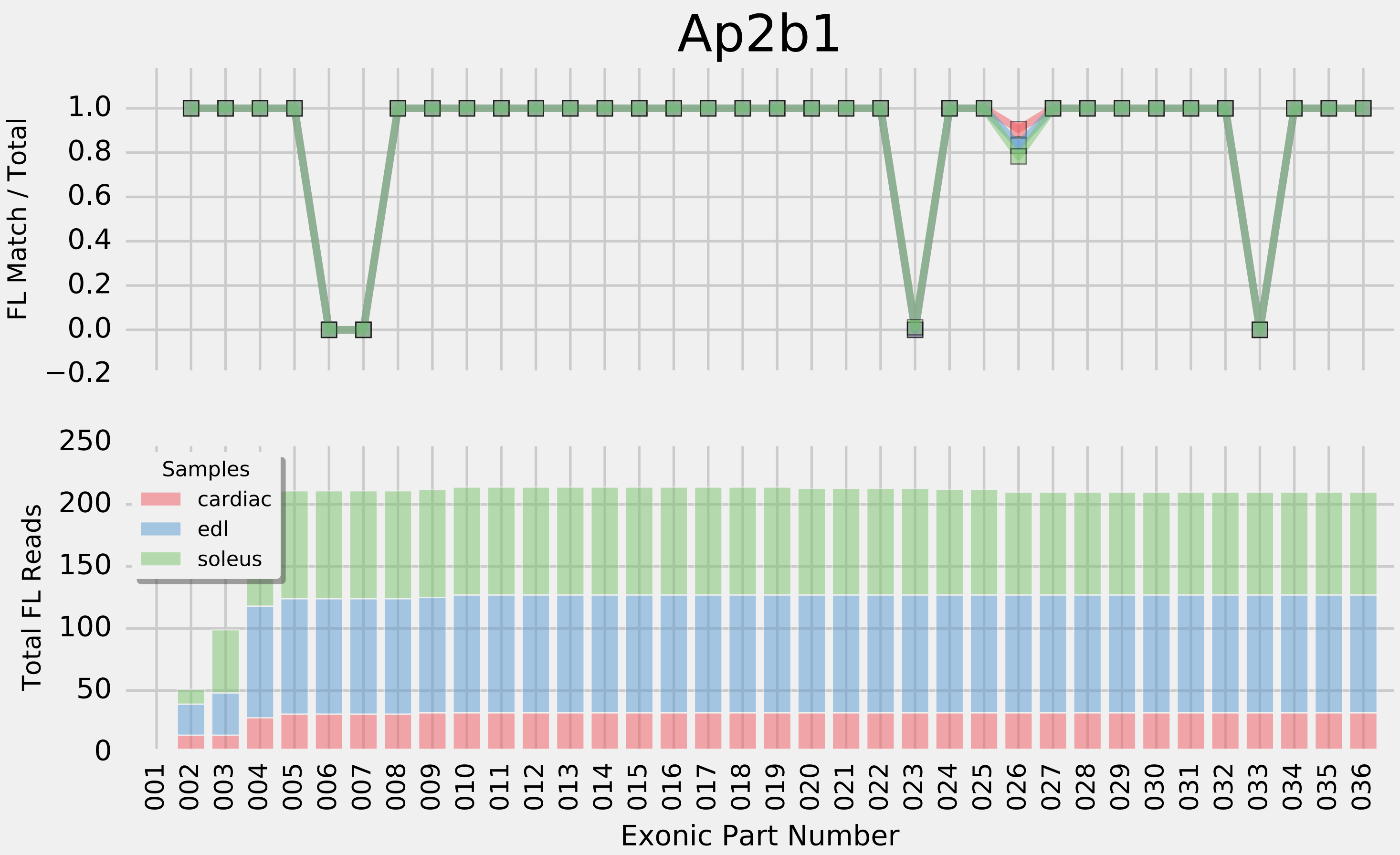

Ap2b1
FL Match / Total
1.0
0.8
0.6
0.4
0.2
0.0
−0.2
Total FL Reads
250
200
150
100
50
0
Samples
cardiac
edl
soleus
001
002
003
004
005
006
007
008
009
010
011
012
013
014
015
016
017
018
019
020
021
022
023
024
025
026
027
028
029
030
031
032
033
034
035
036
Exonic Part Number

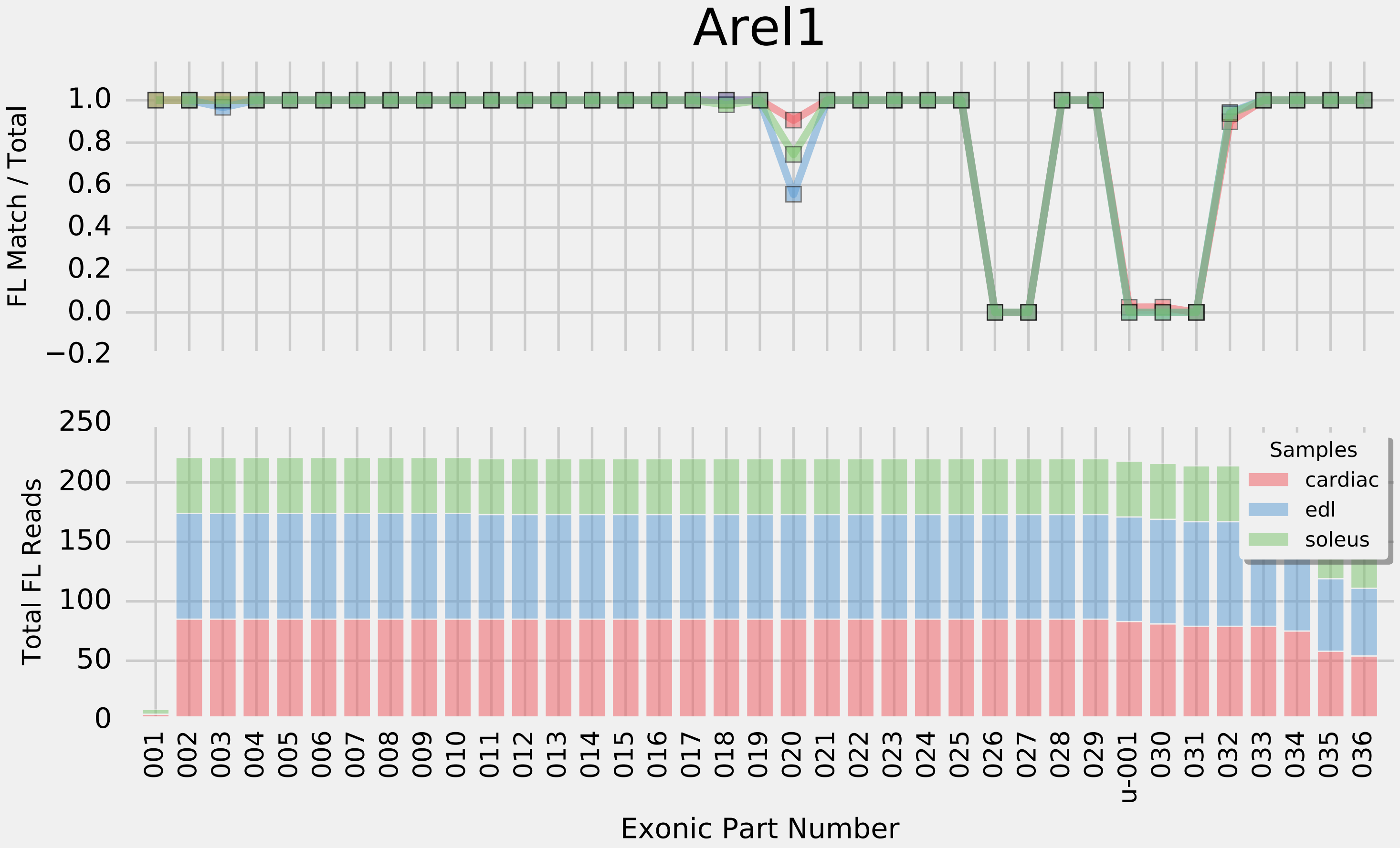

Arel1
FL Match / Total
1.0
0.8
0.6
0.4
0.2
0.0
−0.2
Total FL Reads
250
200
150
100
50
0
Samples
cardiac
edl
soleus
001 002 003 004 005 006 007 008 009 010 011 012 013 014 015 016 017 018 019 020 021 022 023 024 025 026 027 028 029 u-001 030 031 032 033 034 035 036
Exonic Part Number

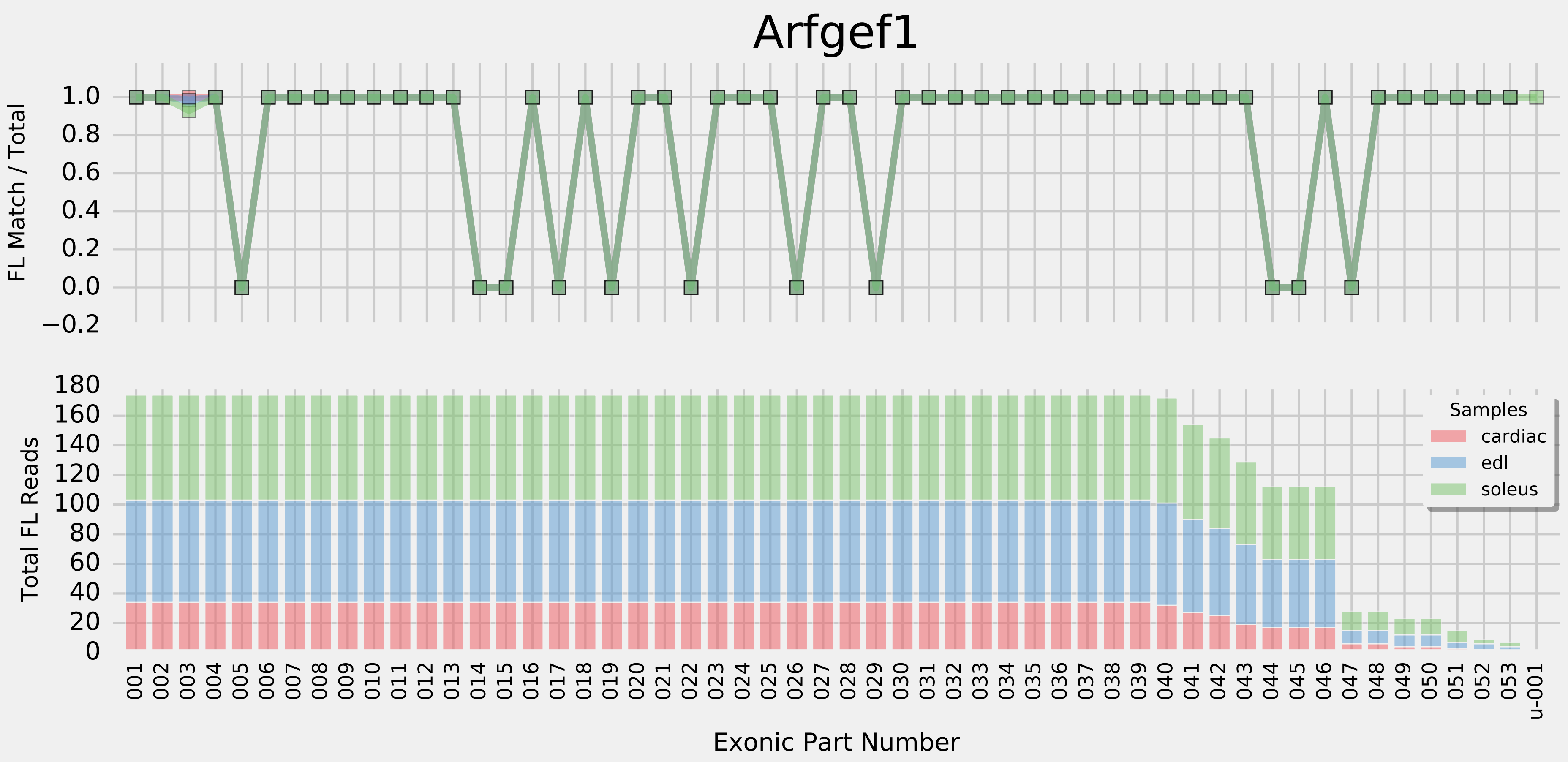

Arfgef1
FL Match / Total
Total FL Reads
Samples
cardiac
edl
soleus
Exonic Part Number

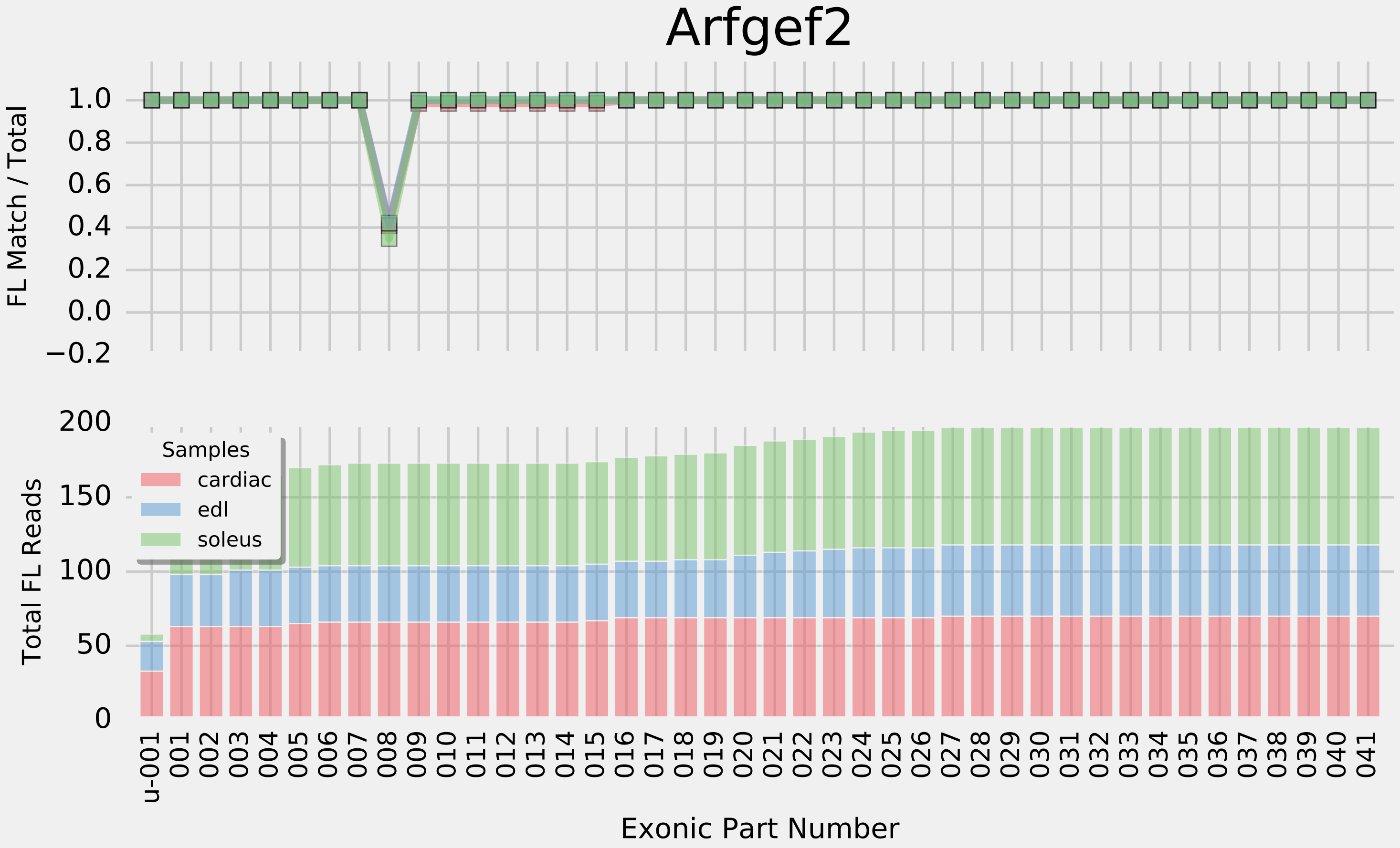
Arfgef2
FL Match / Total
1.0
0.8
0.6
0.4
0.2
0.0
−0.2
Total FL Reads
200
150
100
50
0
Samples
cardiac
edl
soleus
u-001
001
002
003
004
005
006
007
008
009
010
011
012
013
014
015
016
017
018
019
020
021
022
023
024
025
026
027
028
029
030
031
032
033
034
035
036
037
038
039
040
041
Exonic Part Number

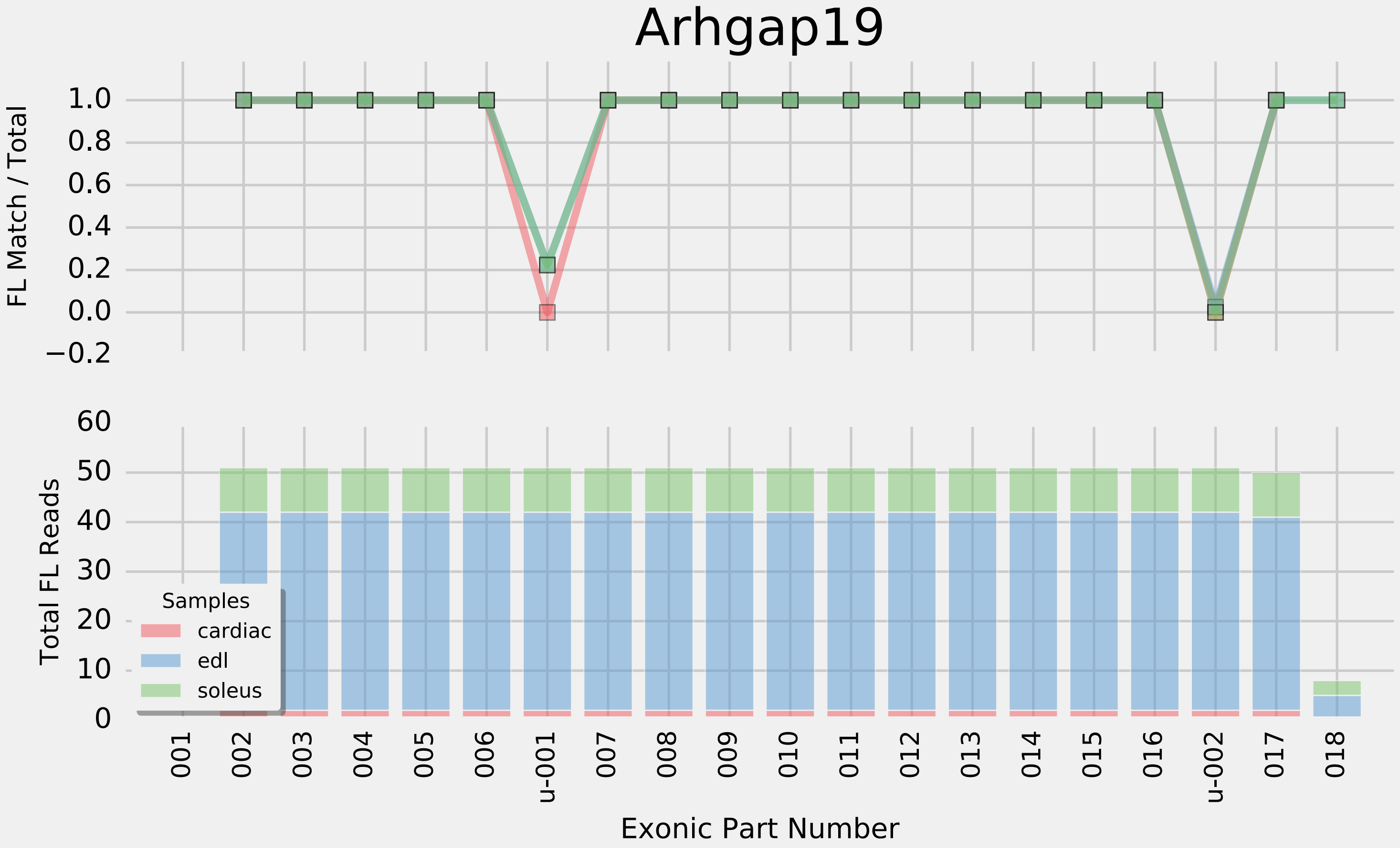

Arhgap19
FL Match / Total
1.0
0.8
0.6
0.4
0.2
0.0
−0.2
Total FL Reads
60
50
40
30
20
10
0
Samples
cardiac
edl
soleus
001
002
003
004
005
006
u-001
007
008
009
010
011
012
013
014
015
016
u-002
017
018
Exonic Part Number

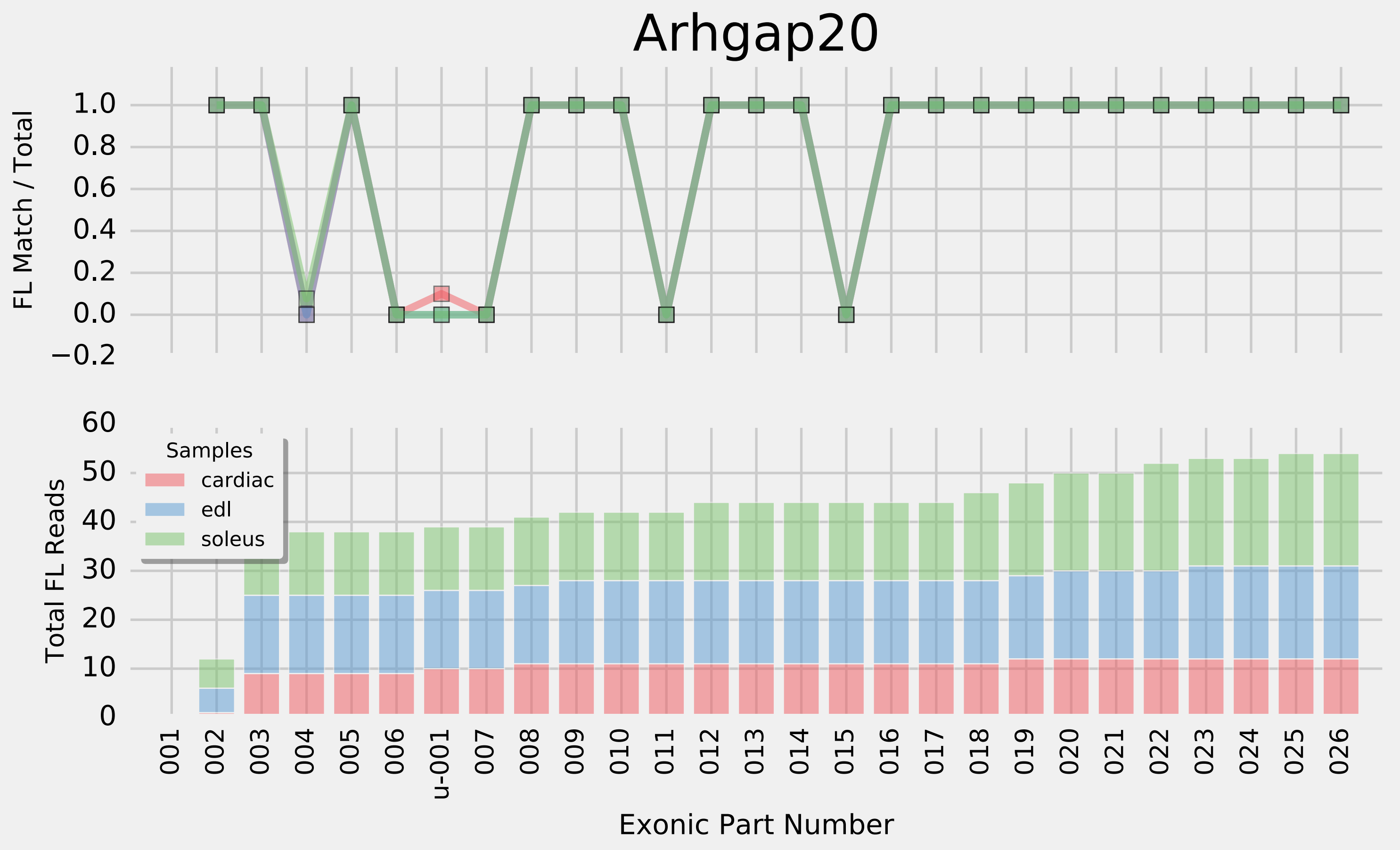

Arhgap20
FL Match / Total
1.0
0.8
0.6
0.4
0.2
0.0
−0.2
Total FL Reads
60
50
40
30
20
10
0
Samples
cardiac
edl
soleus
001
002
003
004
005
006
u-001
007
008
009
010
011
012
013
014
015
016
017
018
019
020
021
022
023
024
025
026
Exonic Part Number

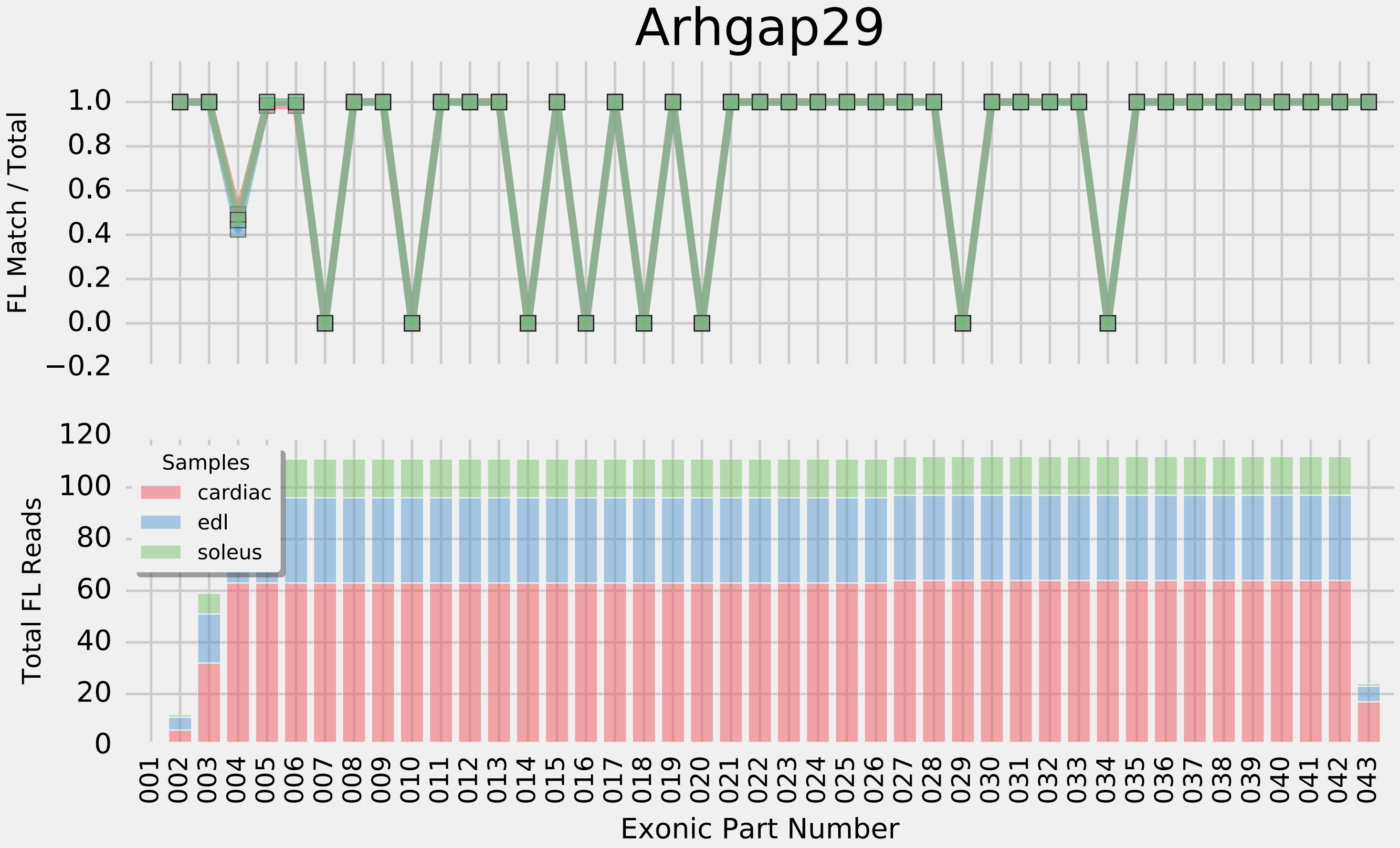

Arhgap29
FL Match / Total
Total FL Reads
Samples
cardiac
edl
soleus
Exonic Part Number

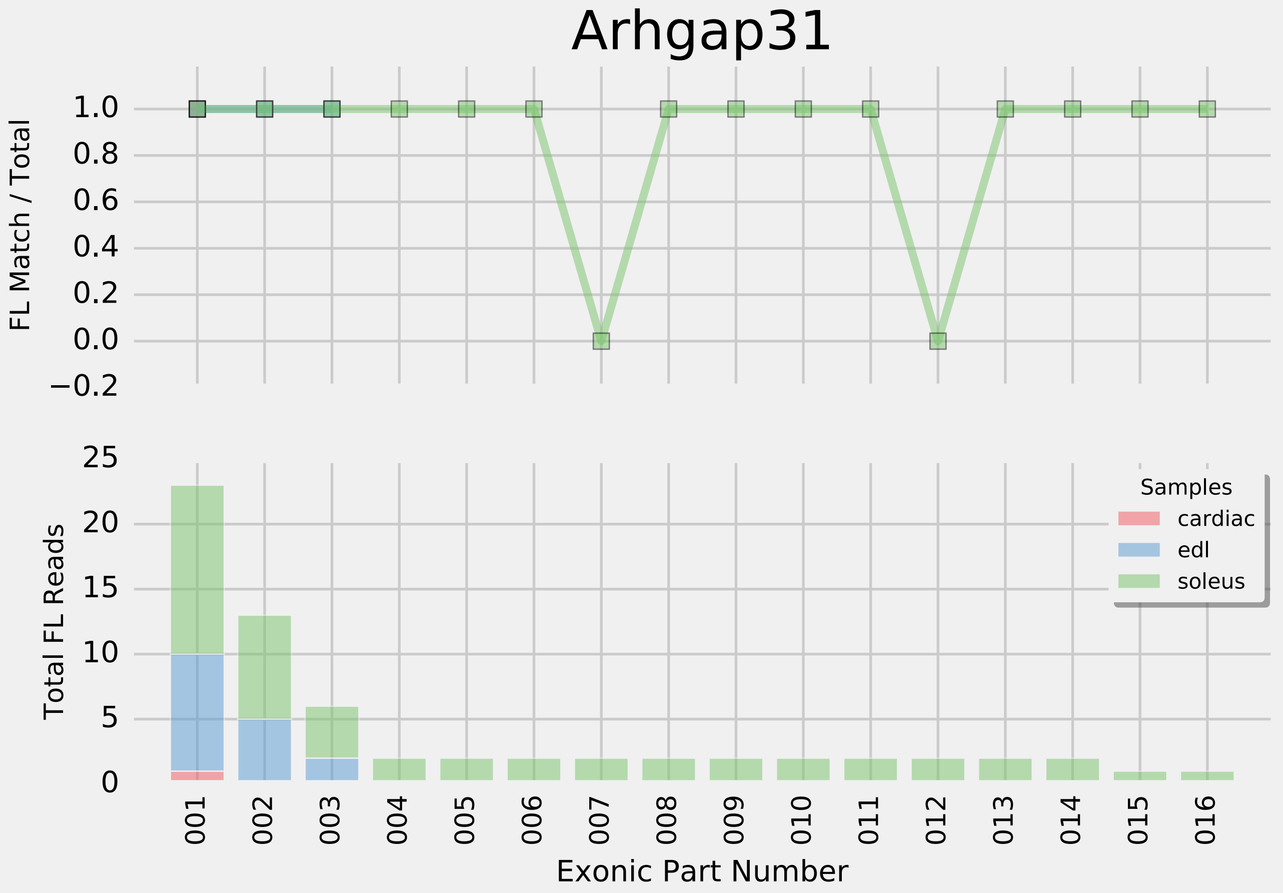
Arhgap31
FL Match / Total
1.0
0.8
0.6
0.4
0.2
0.0
−0.2
Total FL Reads
25
20
15
10
5
0
Samples
cardiac
edl
soleus
001
002
003
004
005
006
007
008
009
010
011
012
013
014
015
016
Exonic Part Number

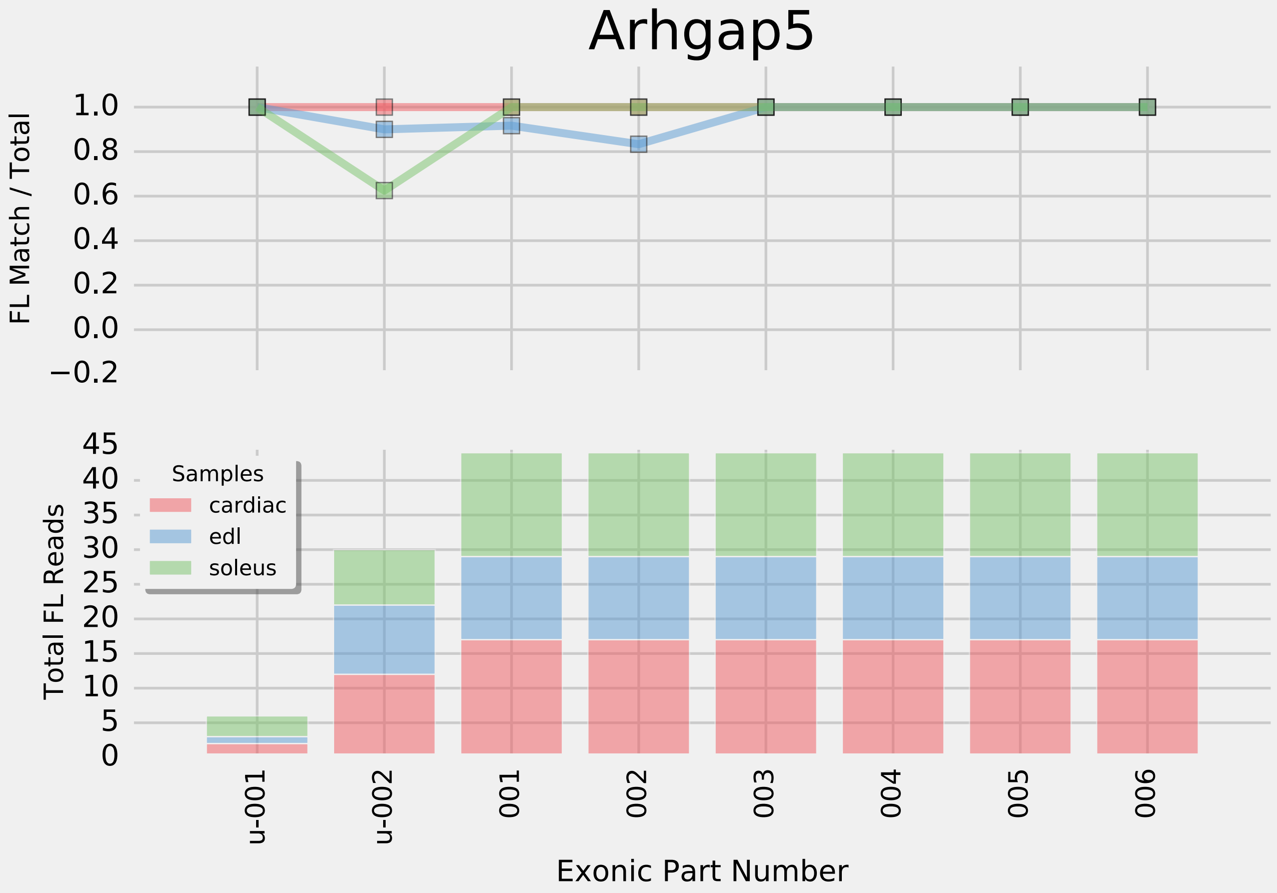

Arhgap5
FL Match / Total
1.0
0.8
0.6
0.4
0.2
0.0
−0.2
Samples
cardiac
edl
soleus
Total FL Reads
45
40
35
30
25
20
15
10
5
0
u-001
u-002
001
002
003
004
005
006
Exonic Part Number

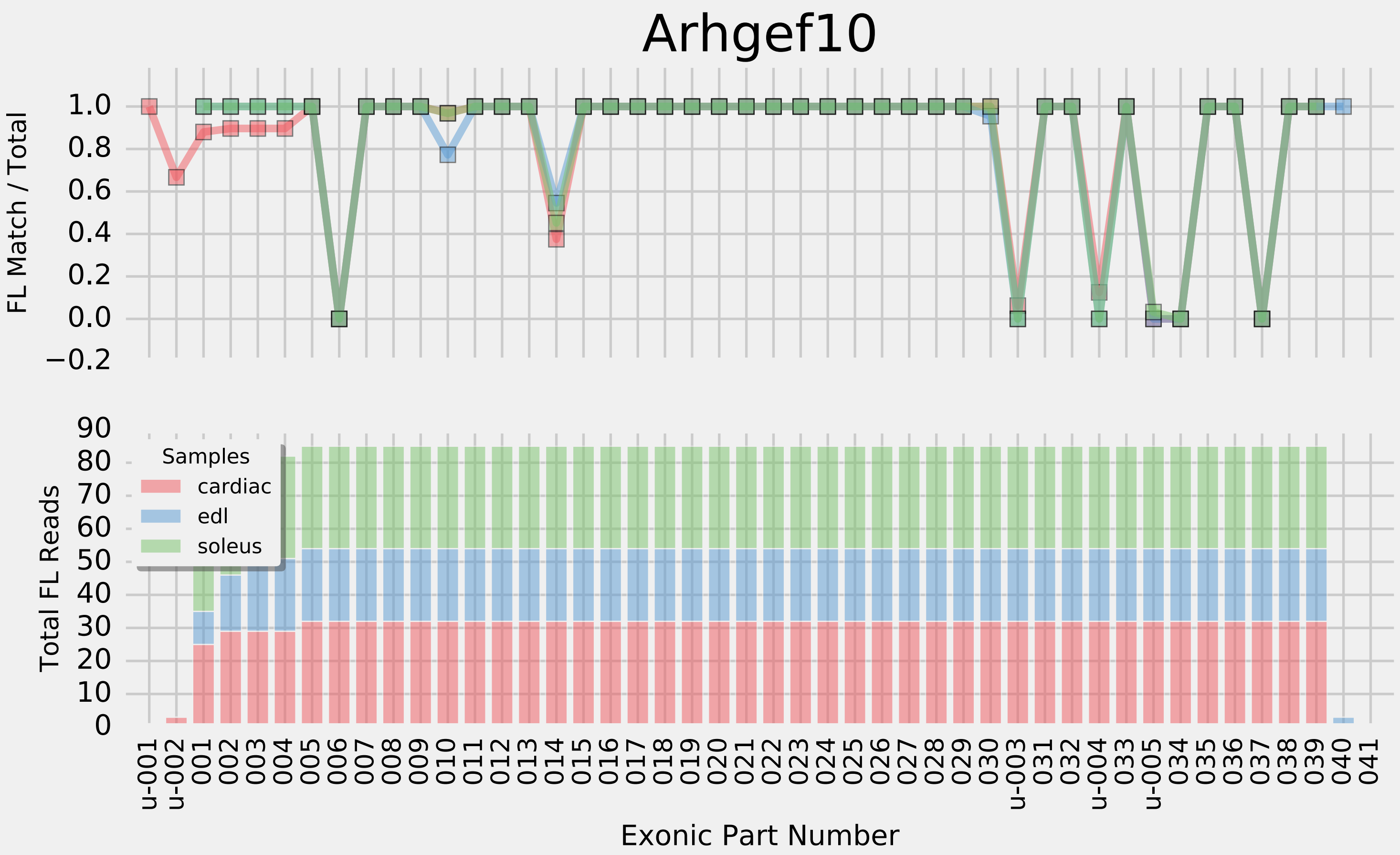
Arhgef10
FL Match / Total
1.0
0.8
0.6
0.4
0.2
0.0
−0.2
Total FL Reads
90
80
70
60
50
40
30
20
10
0
Samples
cardiac
edl
soleus
u-001
u-002
001
002
003
004
005
006
007
008
009
010
011
012
013
014
015
016
017
018
019
020
021
022
023
024
025
026
027
028
029
030
u-003
031
032
u-004
033
u-005
034
035
036
037
038
039
040
041
Exonic Part Number

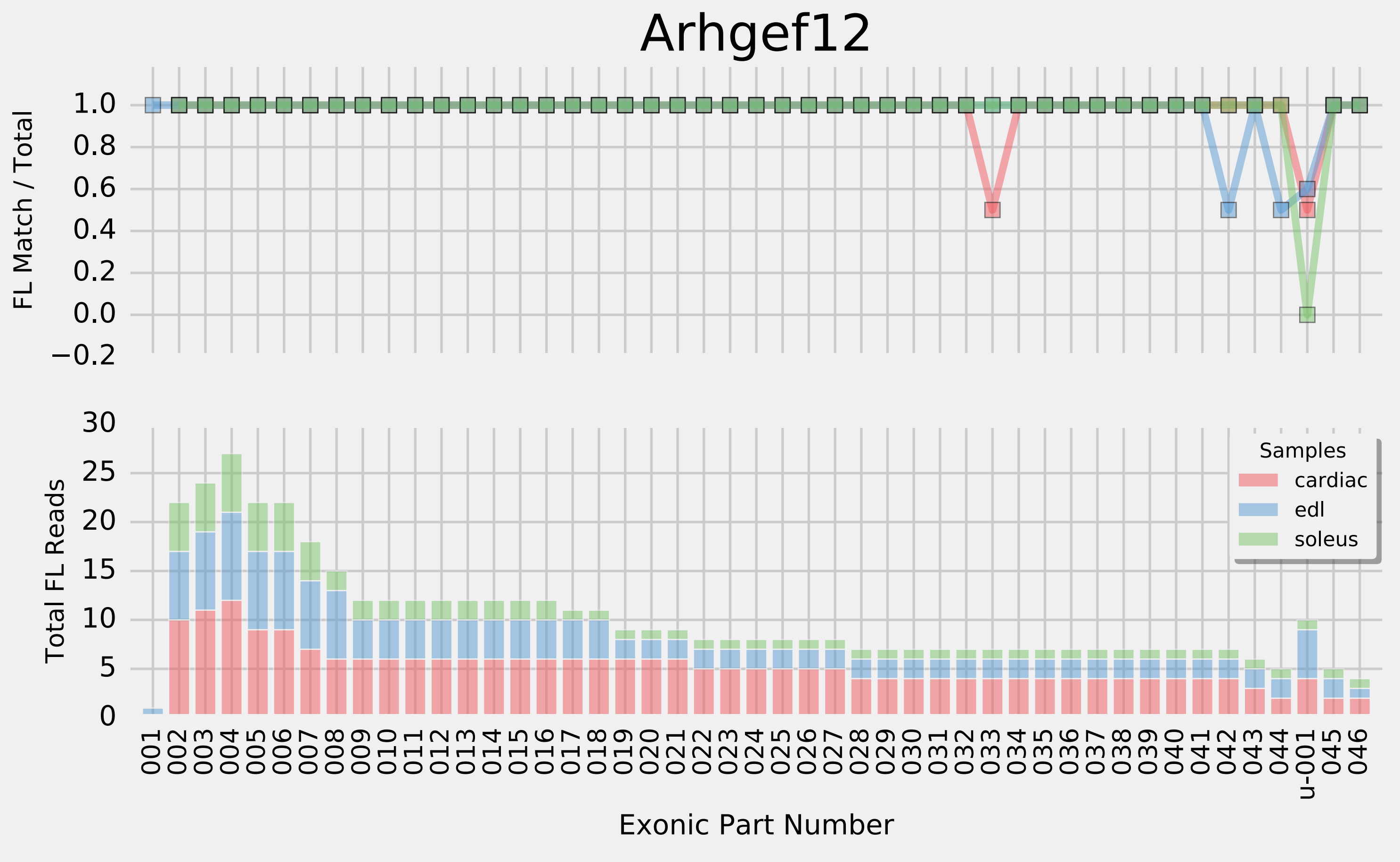

Arhgef12
FL Match / Total
1.0
0.8
0.6
0.4
0.2
0.0
−0.2
Total FL Reads
30
25
20
15
10
5
0
Samples
cardiac
edl
soleus
001 002 003 004 005 006 007 008 009 010 011 012 013 014 015 016 017 018 019 020 021 022 023 024 025 026 027 028 029 030 031 032 033 034 035 036 037 038 039 040 041 042 043 044 u-001 045 046
Exonic Part Number

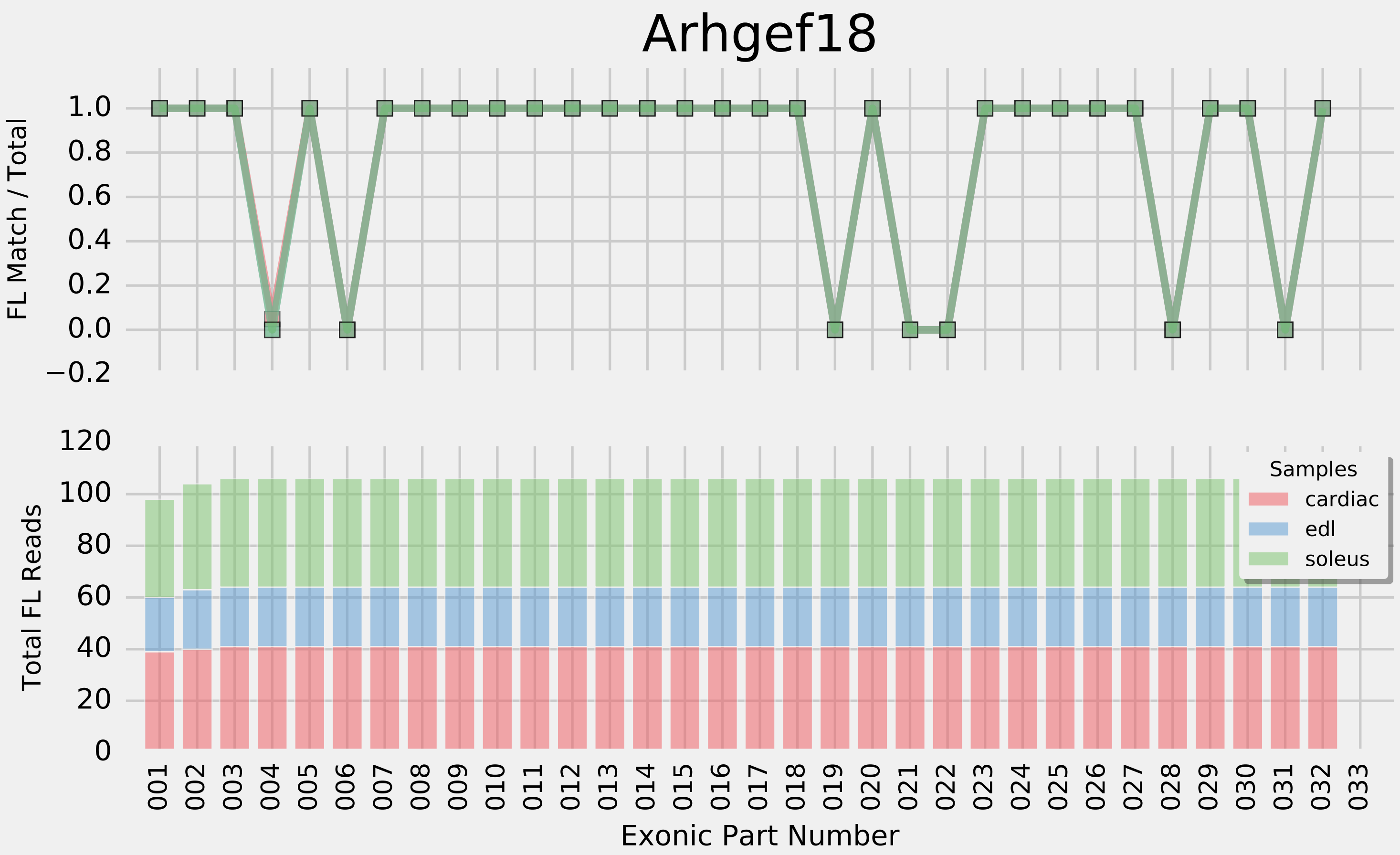

Arhgef18
FL Match / Total
1.0
0.8
0.6
0.4
0.2
0.0
−0.2
Total FL Reads
120
100
80
60
40
20
0
Samples
cardiac
edl
soleus
001 002 003 004 005 006 007 008 009 010 011 012 013 014 015 016 017 018 019 020 021 022 023 024 025 026 027 028 029 030 031 032 033
Exonic Part Number

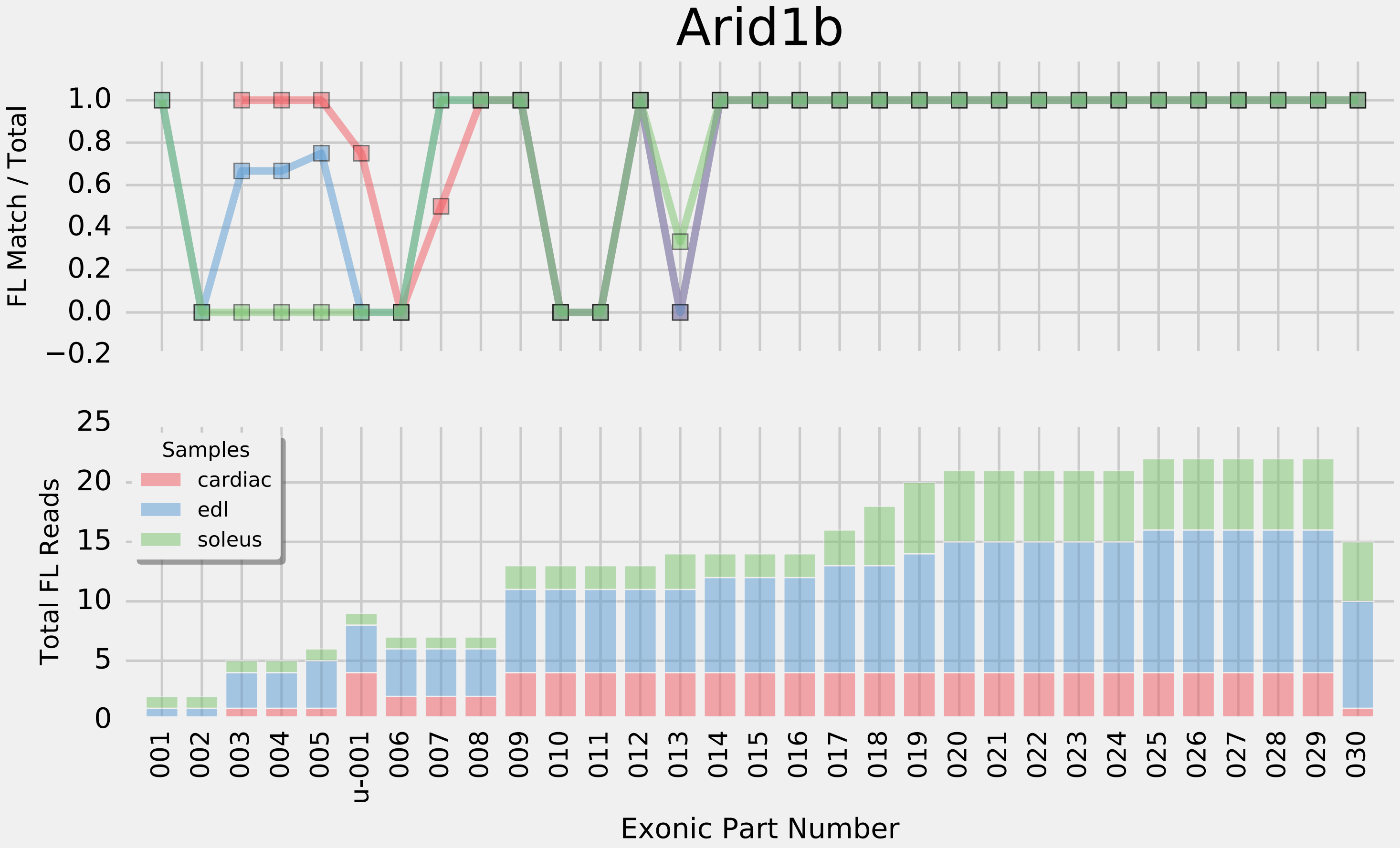
Arid1b
FL Match / Total
1.0
0.8
0.6
0.4
0.2
0.0
−0.2
Total FL Reads
25
20
15
10
5
0
Samples
cardiac
edl
soleus
001
002
003
004
005
u-001
006
007
008
009
010
011
012
013
014
015
016
017
018
019
020
021
022
023
024
025
026
027
028
029
030
Exonic Part Number

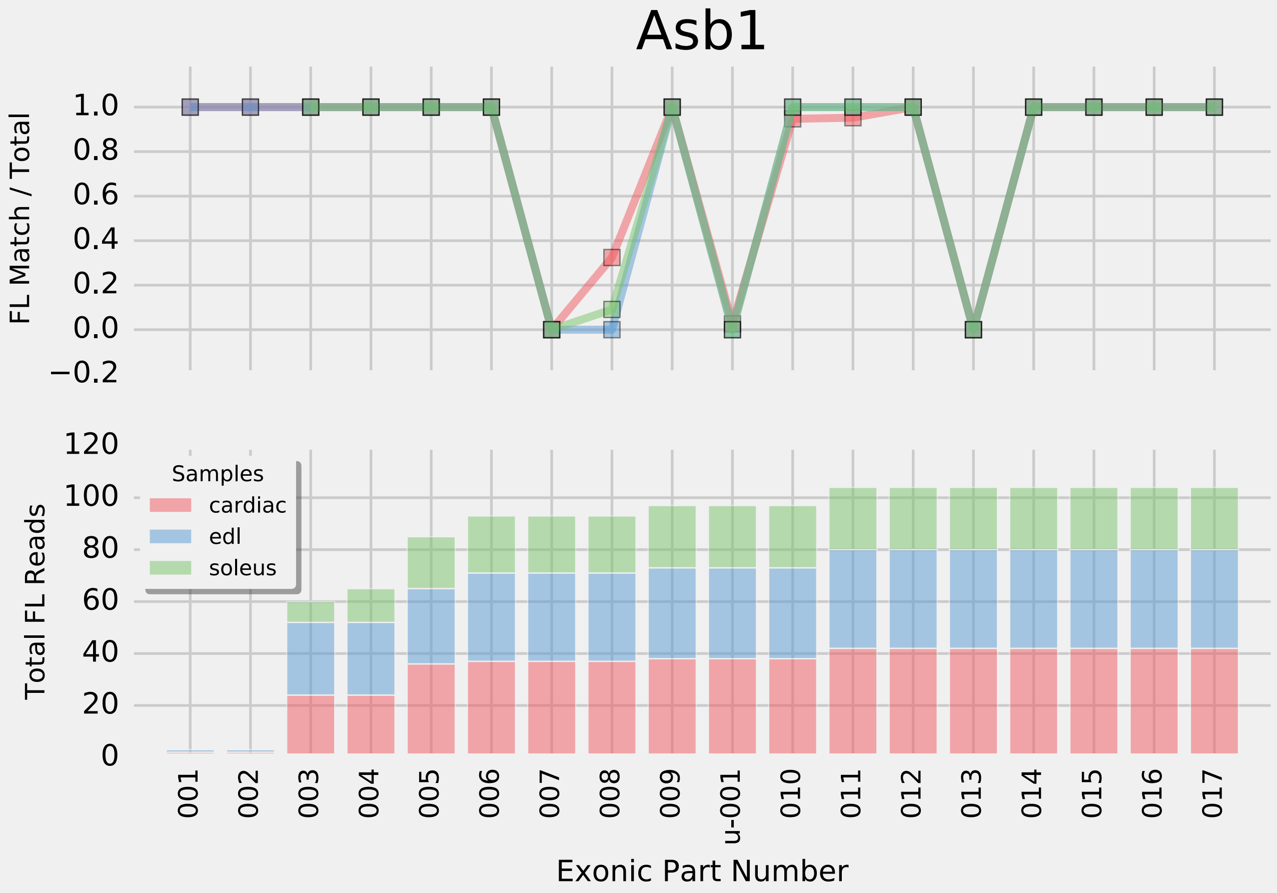

Asb1
FL Match / Total
1.0
0.8
0.6
0.4
0.2
0.0
−0.2
120
100
80
60
40
20
0
Total FL Reads
Samples
cardiac
edl
soleus
001
002
003
004
005
006
007
008
009
u-001
010
011
012
013
014
015
016
017
Exonic Part Number

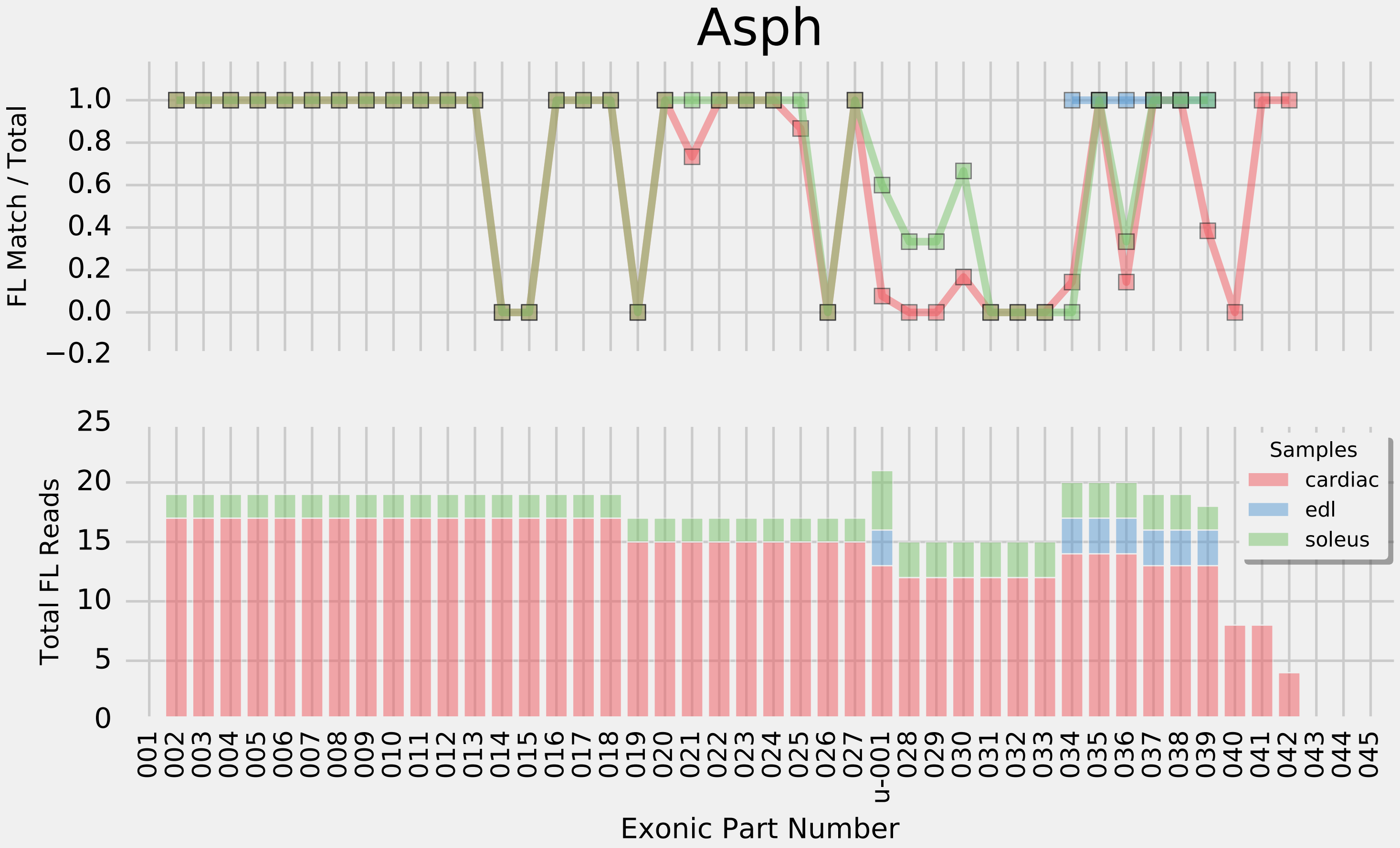

Asph
FL Match / Total
1.0
0.8
0.6
0.4
0.2
0.0
−0.2
Total FL Reads
25
20
15
10
5
0
Samples
cardiac
edl
soleus
001 002 003 004 005 006 007 008 009 010 011 012 013 014 015 016 017 018 019 020 021 022 023 024 025 026 027 u-001 028 029 030 031 032 033 034 035 036 037 038 039 040 041 042 043 044 045
Exonic Part Number

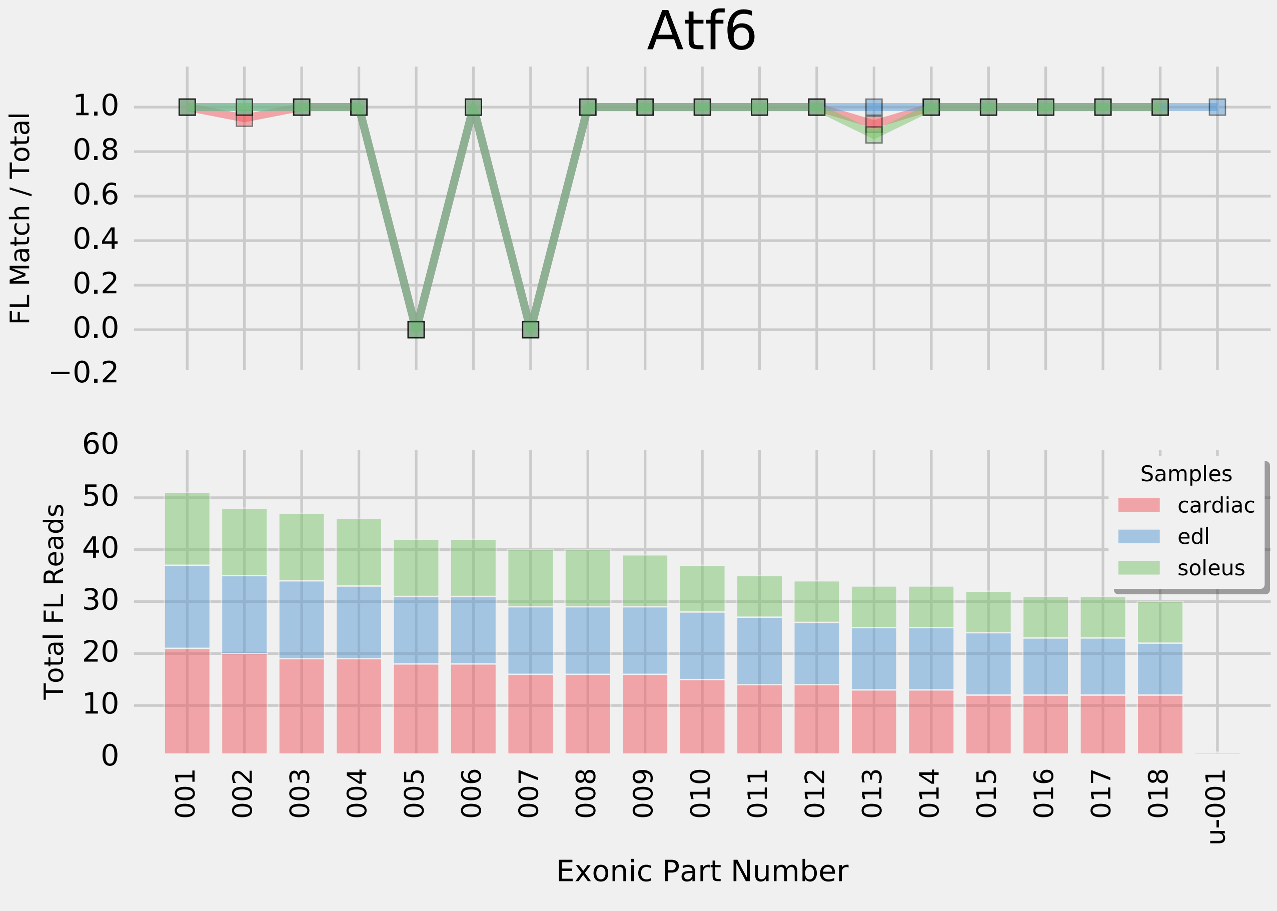
Atf6
FL Match / Total
1.0
0.8
0.6
0.4
0.2
0.0
−0.2
Total FL Reads
60
50
40
30
20
10
0
Samples
cardiac
edl
soleus
001
002
003
004
005
006
007
008
009
010
011
012
013
014
015
016
017
018
u-001
Exonic Part Number

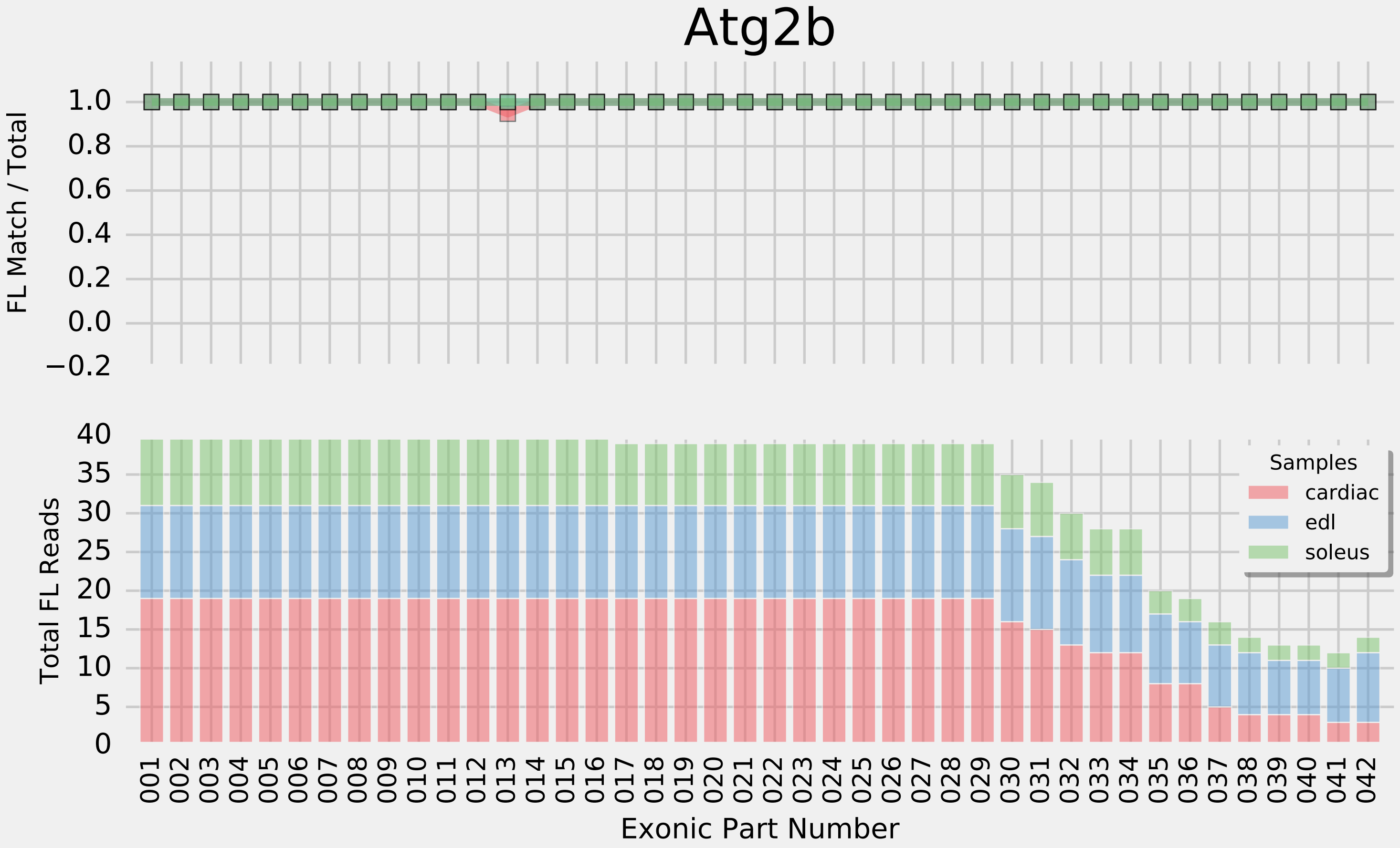

Atg2b
FL Match / Total
1.0
0.8
0.6
0.4
0.2
0.0
−0.2
Total FL Reads
40
35
30
25
20
15
10
5
0
Samples
cardiac
edl
soleus
001 002 003 004 005 006 007 008 009 010 011 012 013 014 015 016 017 018 019 020 021 022 023 024 025 026 027 028 029 030 031 032 033 034 035 036 037 038 039 040 041 042
Exonic Part Number

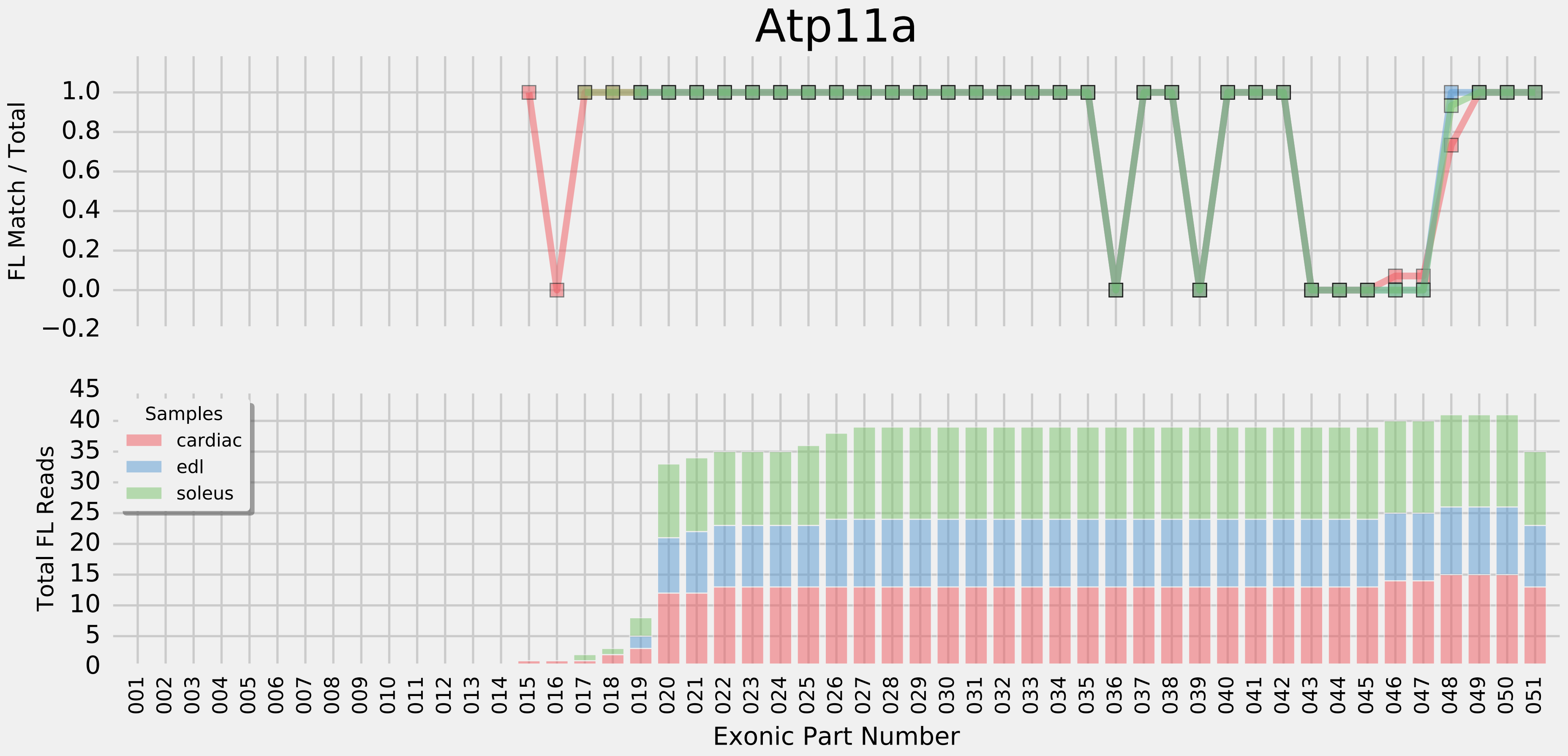

Atp11a
FL Match / Total
Total FL Reads
Samples
cardiac
edl
soleus
Exonic Part Number

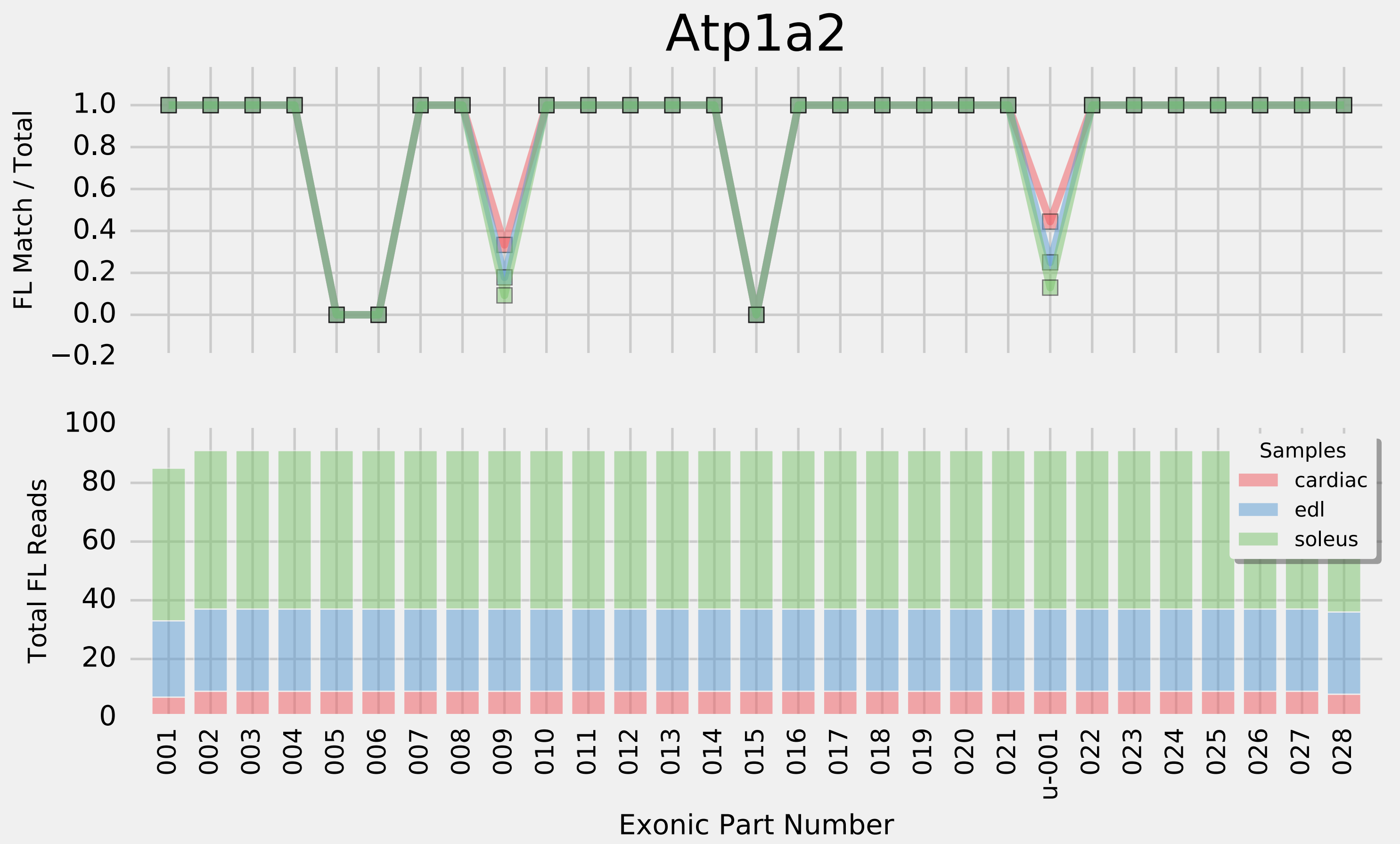

Atp1a2
FL Match / Total
1.0
0.8
0.6
0.4
0.2
0.0
−0.2
Total FL Reads
100
80
60
40
20
0
Samples
cardiac
edl
soleus
001 002 003 004 005 006 007 008 009 010 011 012 013 014 015 016 017 018 019 020 021 u-001 022 023 024 025 026 027 028
Exonic Part Number

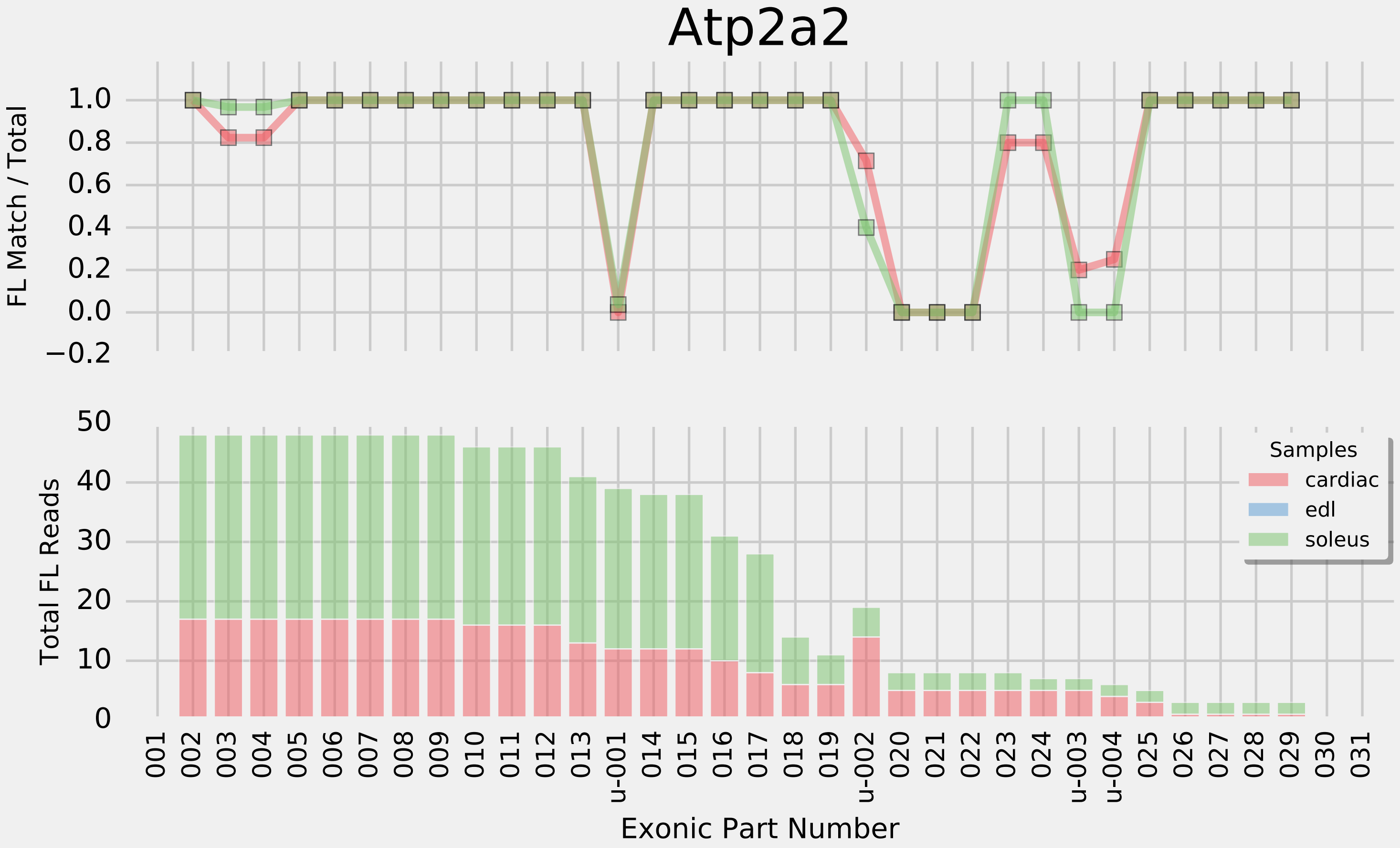

Atp2a2
FL Match / Total
1.0
0.8
0.6
0.4
0.2
0.0
−0.2
Total FL Reads
50
40
30
20
10
0
Samples
cardiac
edl
soleus
001
002
003
004
005
006
007
008
009
010
011
012
013
u-001
014
015
016
017
018
019
u-002
020
021
022
023
024
u-003
u-004
025
026
027
028
029
030
031
Exonic Part Number

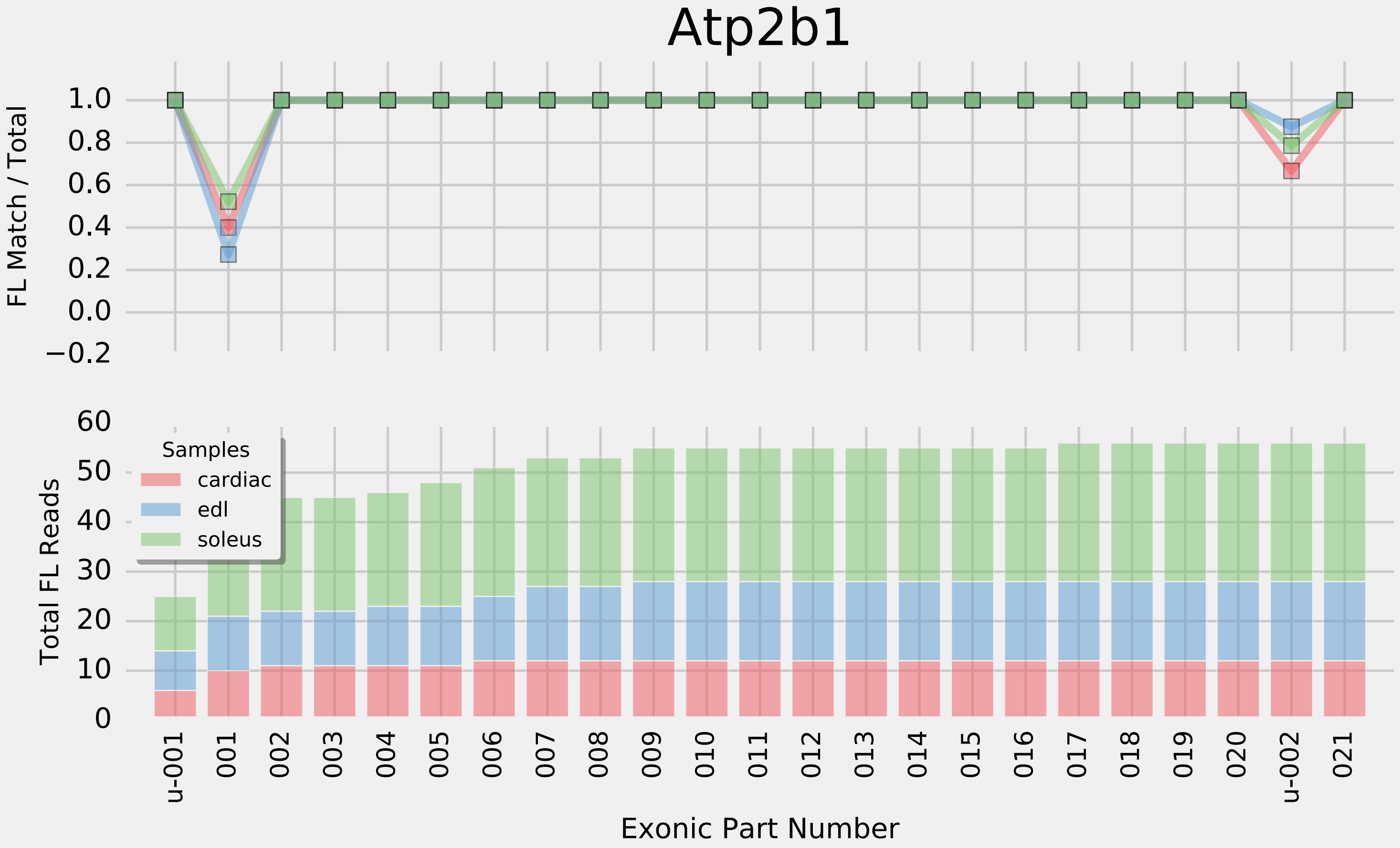

Atp2b1
FL Match / Total
1.0
0.8
0.6
0.4
0.2
0.0
−0.2
Total FL Reads
60
50
40
30
20
10
0
Samples
cardiac
edl
soleus
u-001
001
002
003
004
005
006
007
008
009
010
011
012
013
014
015
016
017
018
019
020
u-002
021
Exonic Part Number

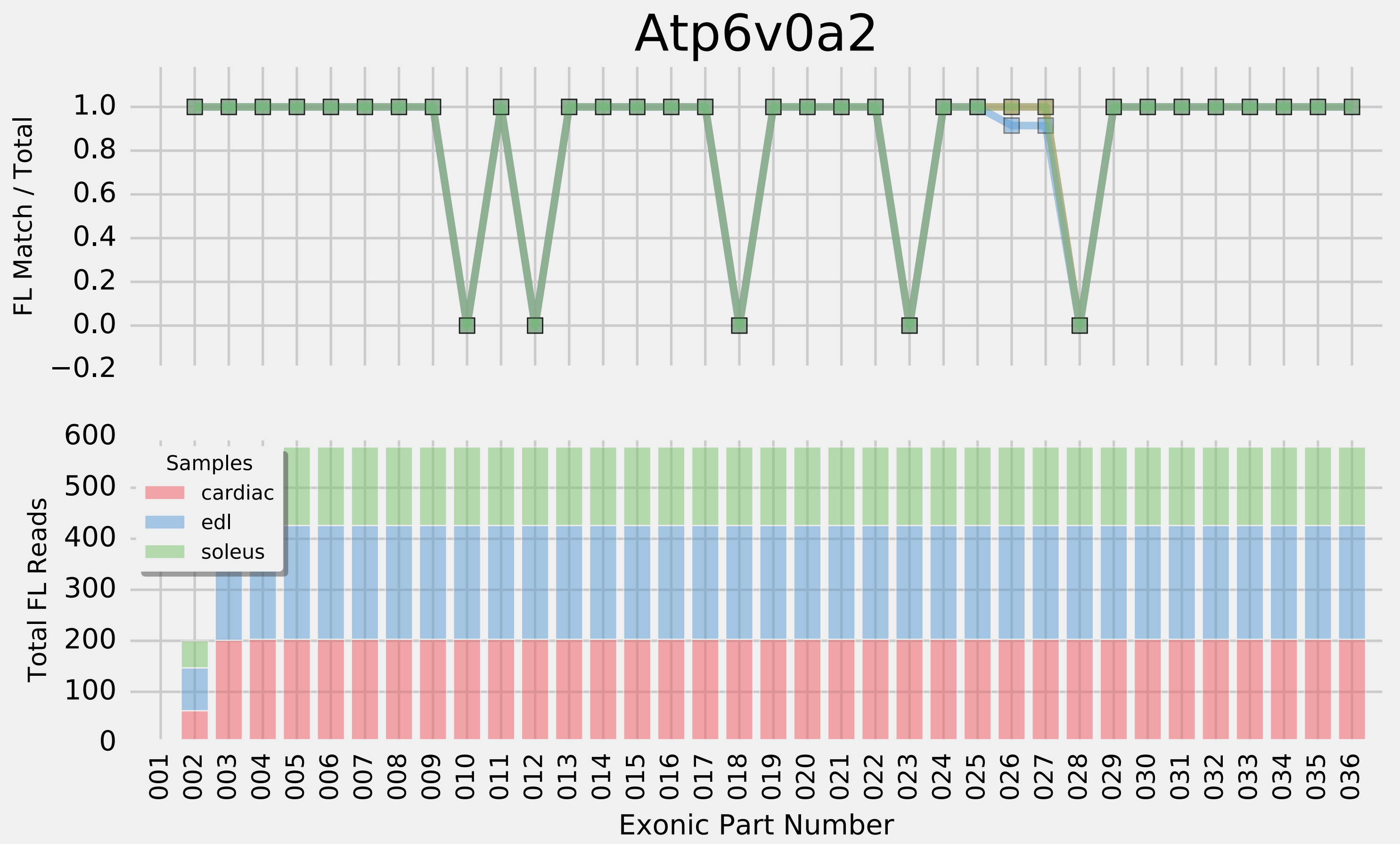

Atp6v0a2
FL Match / Total
1.0
0.8
0.6
0.4
0.2
0.0
−0.2
600
500
400
300
200
100
0
Samples
cardiac
edl
soleus
Total FL Reads
001
002
003
004
005
006
007
008
009
010
011
012
013
014
015
016
017
018
019
020
021
022
023
024
025
026
027
028
029
030
031
032
033
034
035
036
Exonic Part Number

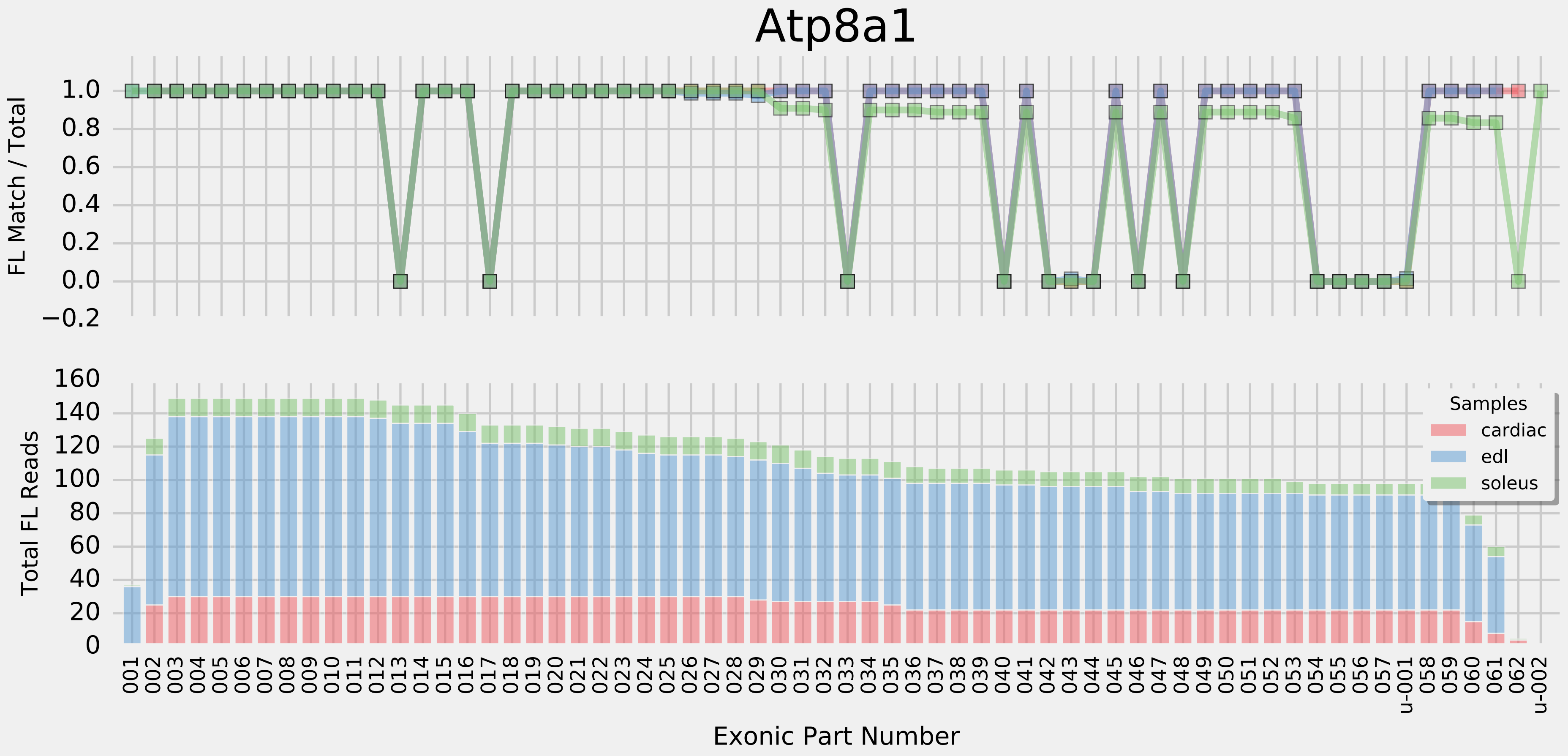

Atp8a1
FL Match / Total
Total FL Reads
Exonic Part Number
Samples
cardiac
edl
soleus

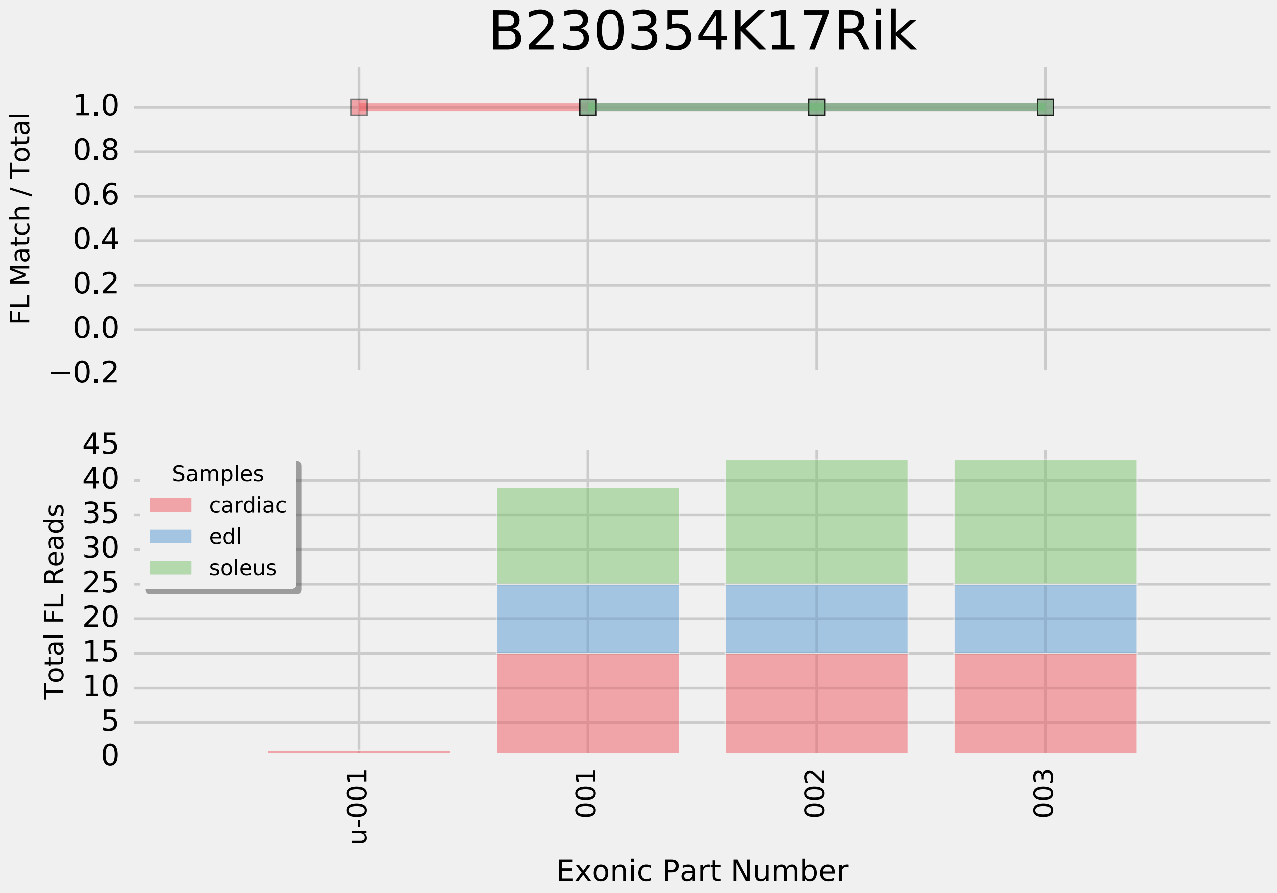
B230354K17Rik
FL Match / Total
1.0
0.8
0.6
0.4
0.2
0.0
−0.2
Total FL Reads
45
40
35
30
25
20
15
10
5
0
Samples
cardiac
edl
soleus
u-001
001
002
003
Exonic Part Number

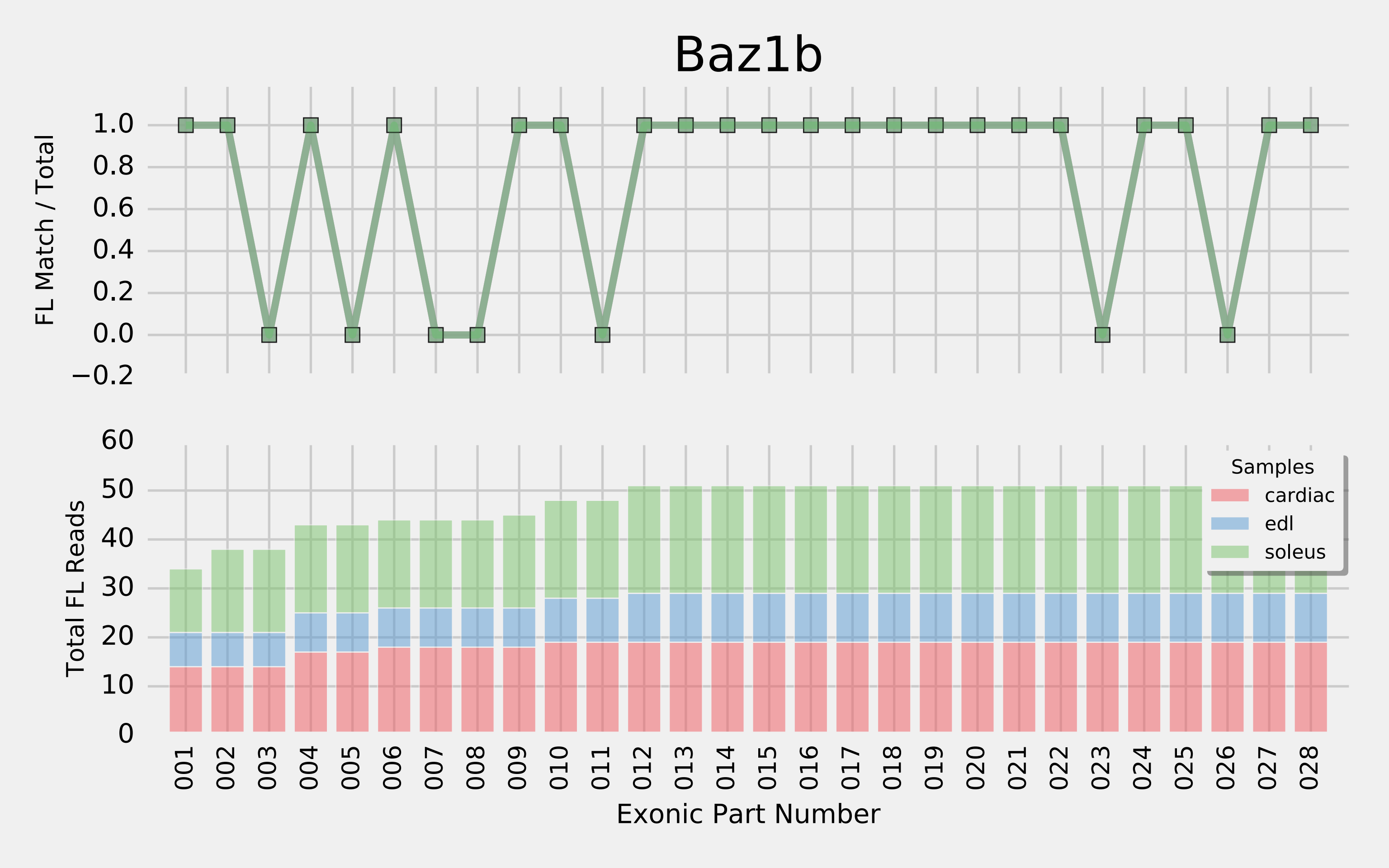

Baz1b
FL Match / Total
1.0
0.8
0.6
0.4
0.2
0.0
−0.2
Total FL Reads
60
50
40
30
20
10
0
Samples
cardiac
edl
soleus
001 002 003 004 005 006 007 008 009 010 011 012 013 014 015 016 017 018 019 020 021 022 023 024 025 026 027 028
Exonic Part Number

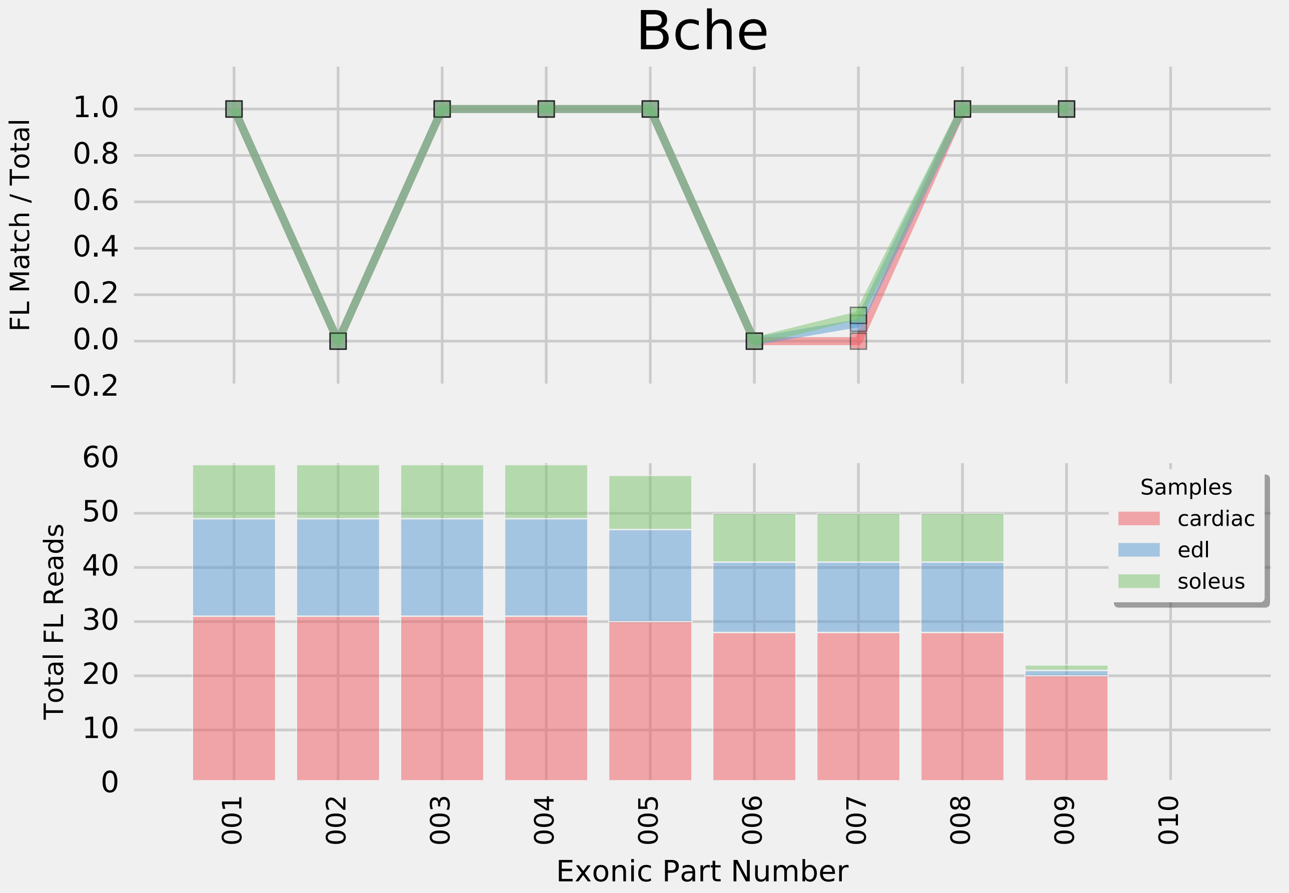
Bche
FL Match / Total
1.0
0.8
0.6
0.4
0.2
0.0
−0.2
Total FL Reads
60
50
40
30
20
10
0
Samples
cardiac
edl
soleus
001
002
003
004
005
006
007
008
009
010
Exonic Part Number

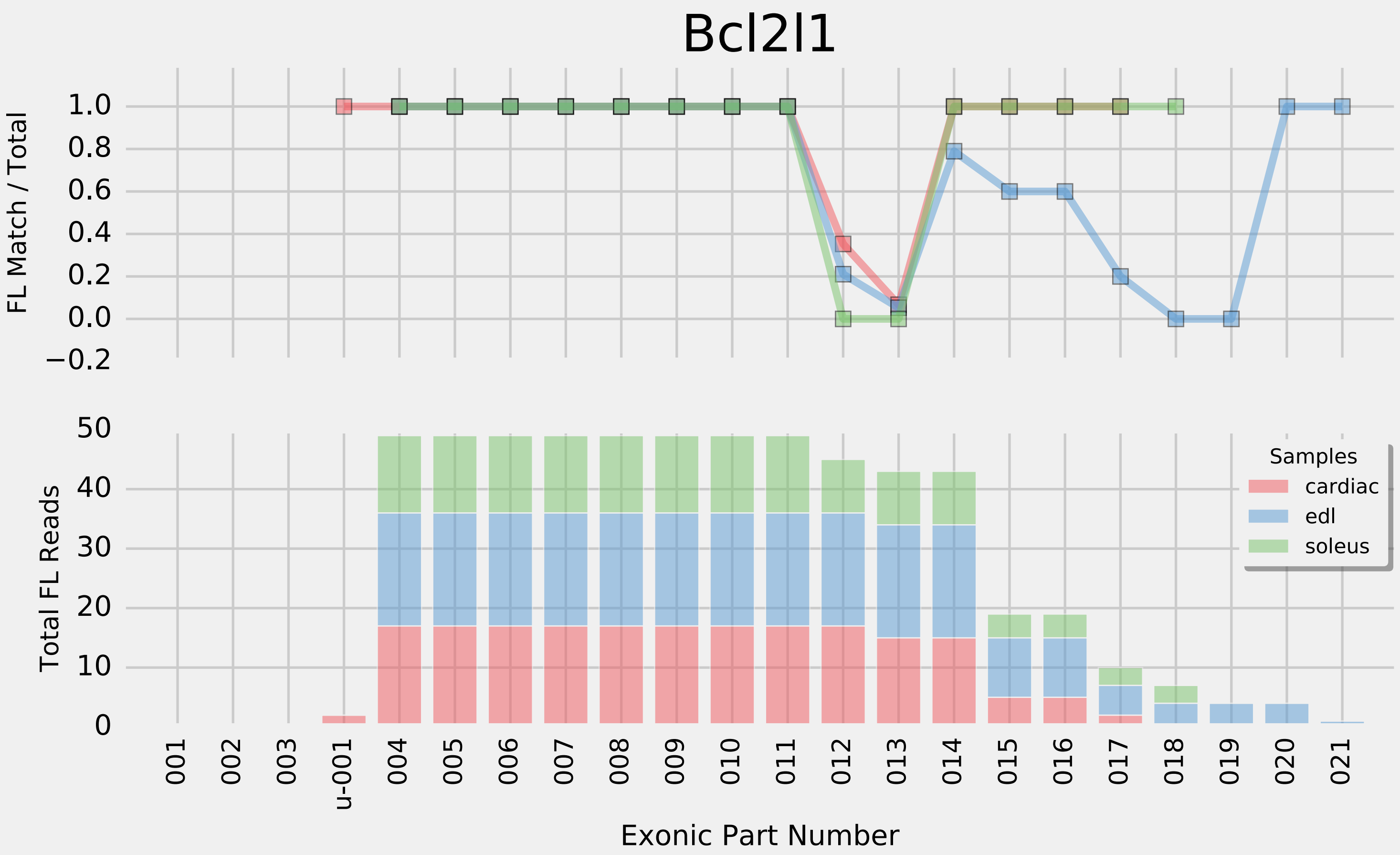
Bcl2l1
FL Match / Total
1.0
0.8
0.6
0.4
0.2
0.0
−0.2
Total FL Reads
50
40
30
20
10
0
Samples
cardiac
edl
soleus
001
002
003
u-001
004
005
006
007
008
009
010
011
012
013
014
015
016
017
018
019
020
021
Exonic Part Number

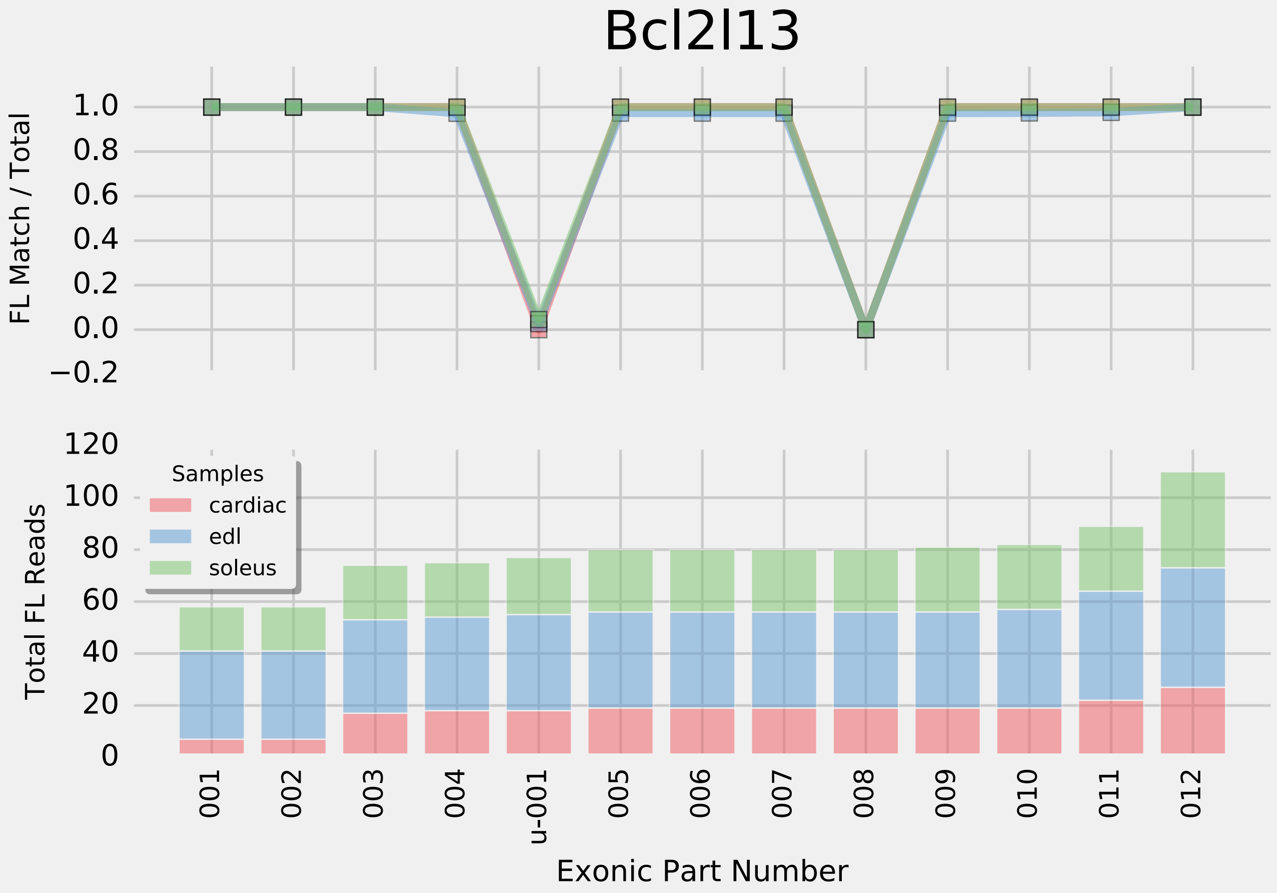

Bcl2l13
FL Match / Total
1.0
0.8
0.6
0.4
0.2
0.0
−0.2
Total FL Reads
120
100
80
60
40
20
0
Samples
cardiac
edl
soleus
001
002
003
004
u-001
005
006
007
008
009
010
011
012
Exonic Part Number

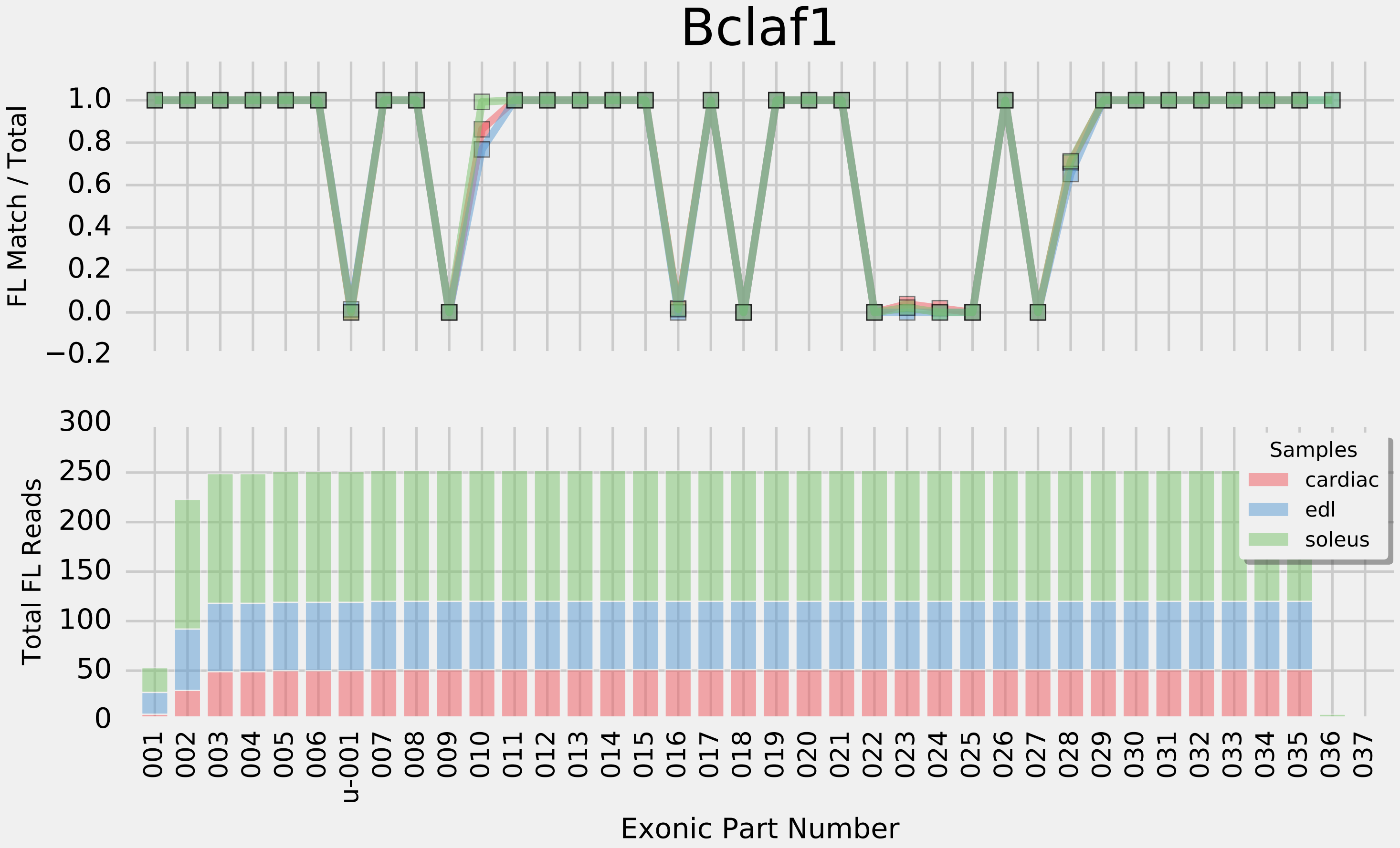

Bclaf1
FL Match / Total
1.0
0.8
0.6
0.4
0.2
0.0
−0.2
300
250
200
150
100
50
0
Total FL Reads
Samples
cardiac
edl
soleus
001 002 003 004 005 006 u-001 007 008 009 010 011 012 013 014 015 016 017 018 019 020 021 022 023 024 025 026 027 028 029 030 031 032 033 034 035 036 037
Exonic Part Number

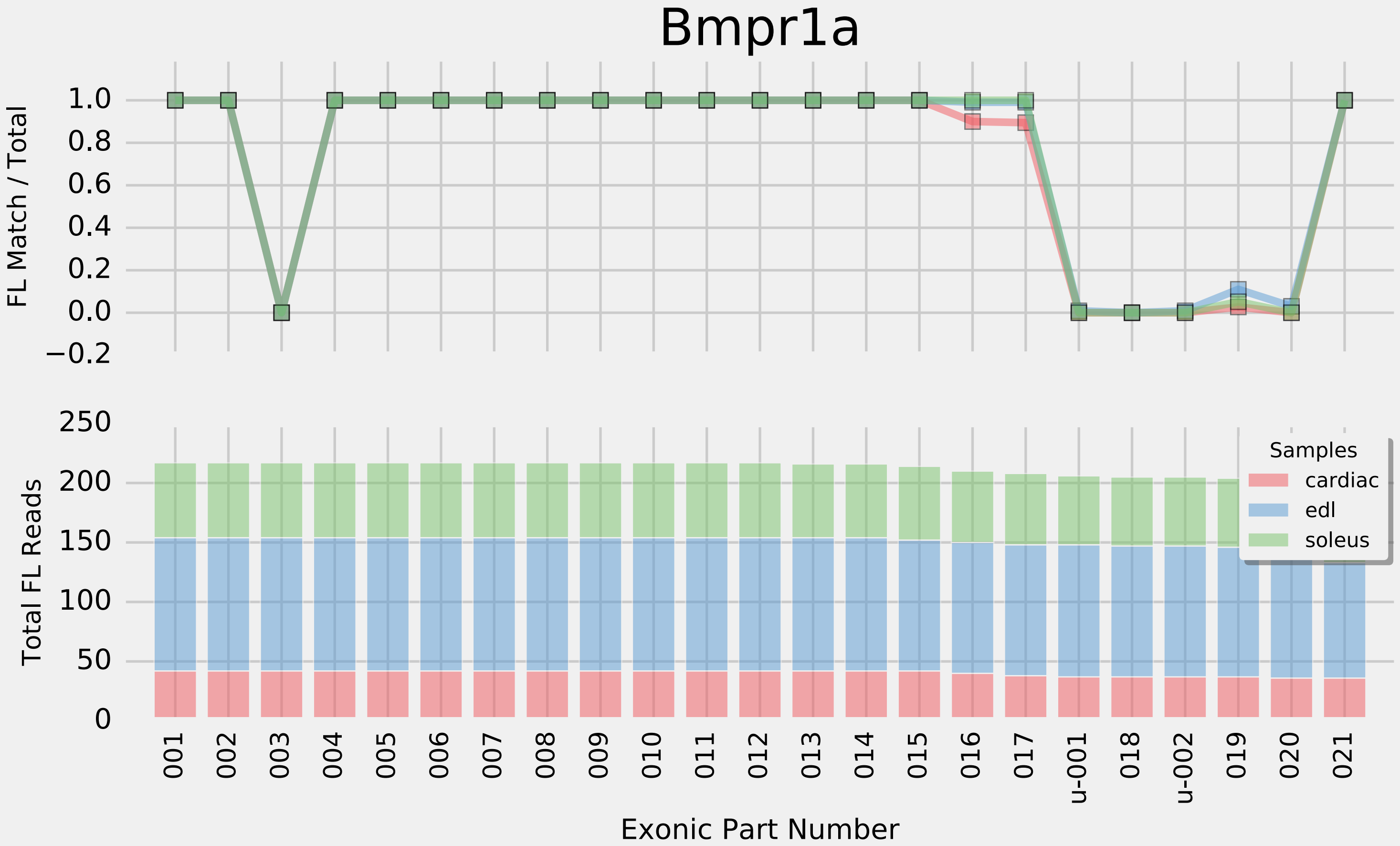

Bmpr1a
FL Match / Total
1.0
0.8
0.6
0.4
0.2
0.0
−0.2
Total FL Reads
250
200
150
100
50
0
Samples
cardiac
edl
soleus
001
002
003
004
005
006
007
008
009
010
011
012
013
014
015
016
017
u-001
018
u-002
019
020
021
Exonic Part Number

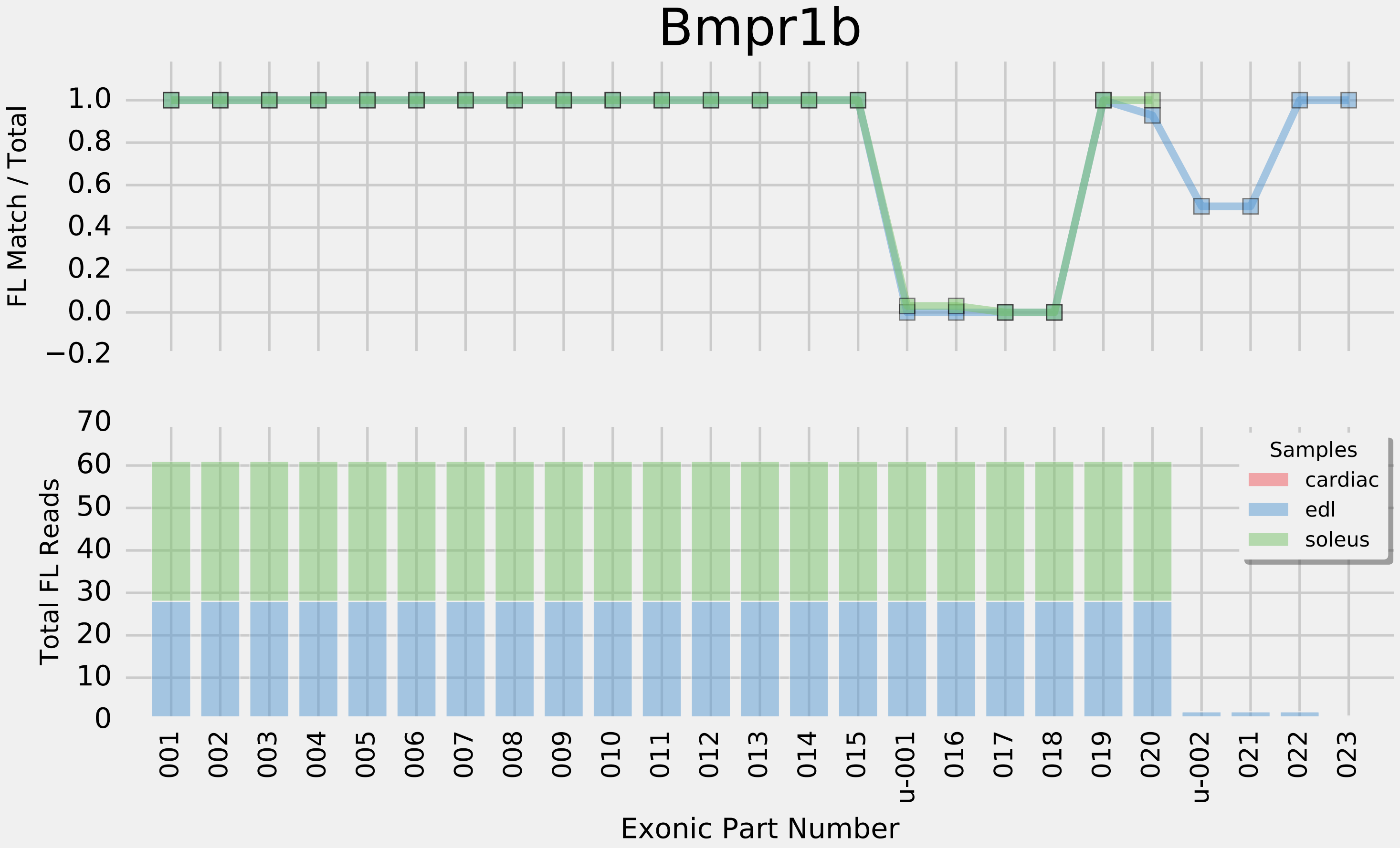
Bmpr1b
FL Match / Total
1.0
0.8
0.6
0.4
0.2
0.0
−0.2
Total FL Reads
70
60
50
40
30
20
10
0
Samples
cardiac
edl
soleus
001
002
003
004
005
006
007
008
009
010
011
012
013
014
015
u-001
016
017
018
019
020
u-002
021
022
023
Exonic Part Number

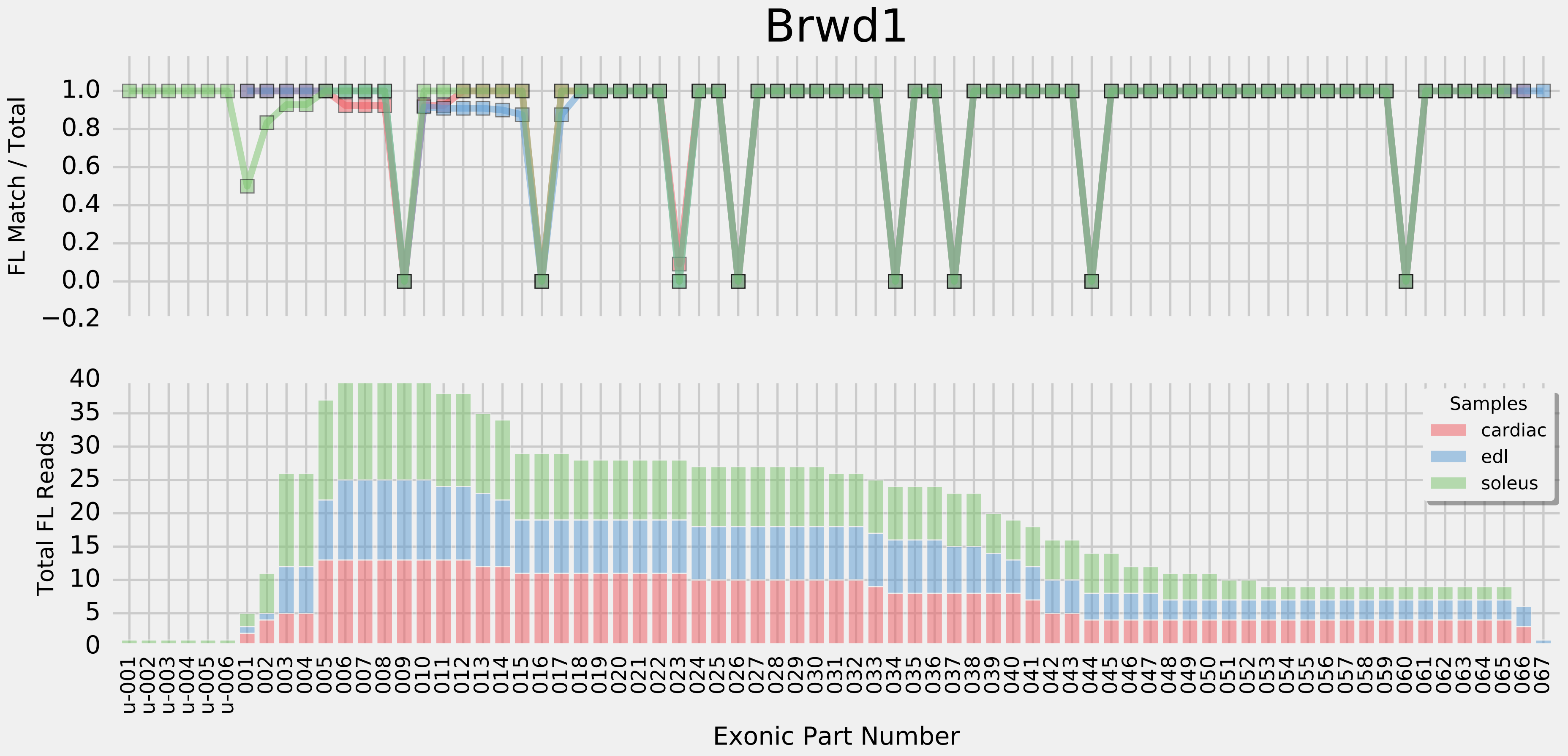

Brwd1
FL Match / Total
Total FL Reads
Exonic Part Number
Samples
cardiac
edl
soleus

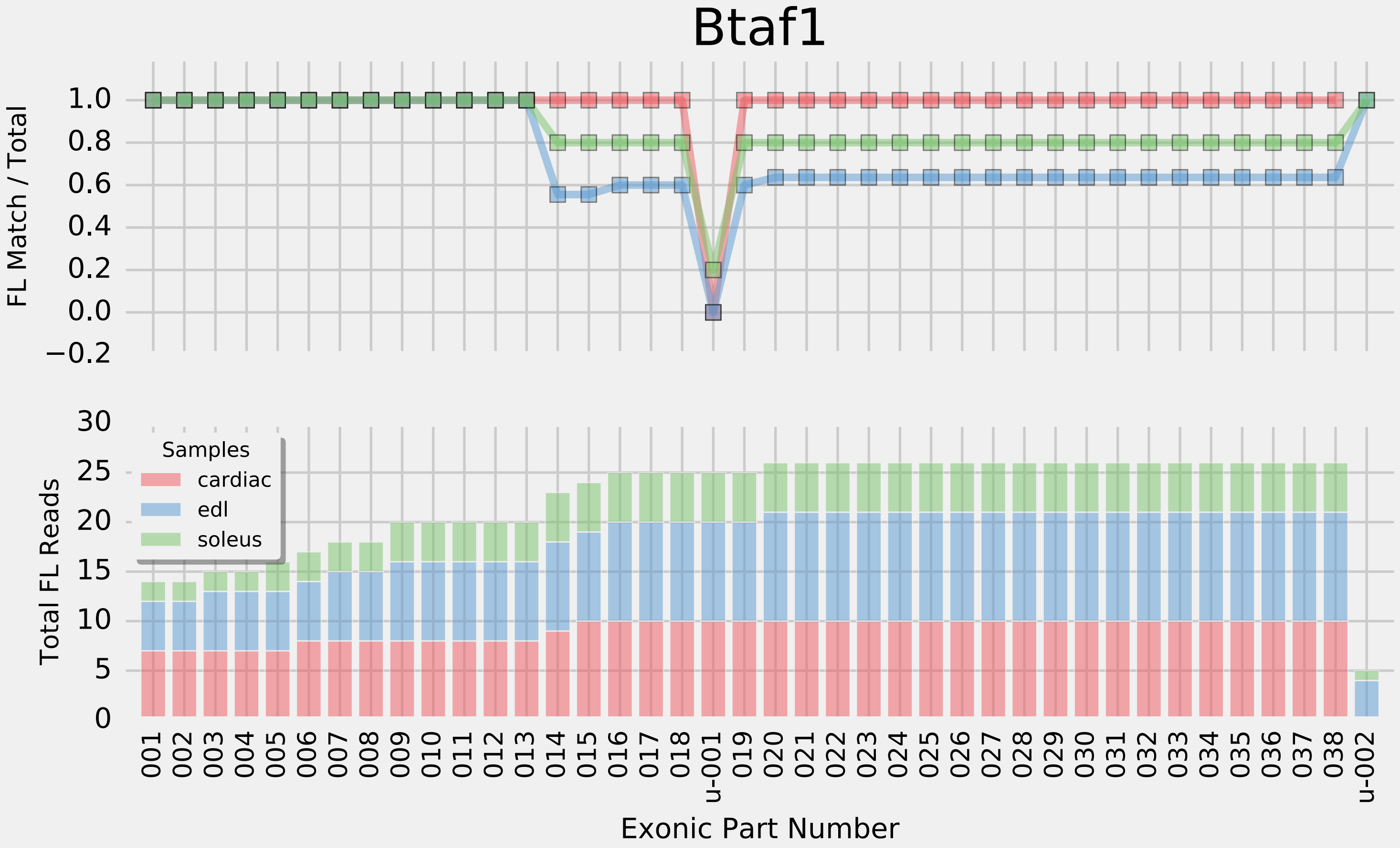

Btaf1
FL Match / Total
1.0
0.8
0.6
0.4
0.2
0.0
−0.2
Total FL Reads
30
25
20
15
10
5
0
Samples
cardiac
edl
soleus
001 002 003 004 005 006 007 008 009 010 011 012 013 014 015 016 017 018 u-001 019 020 021 022 023 024 025 026 027 028 029 030 031 032 033 034 035 036 037 038 u-002
Exonic Part Number

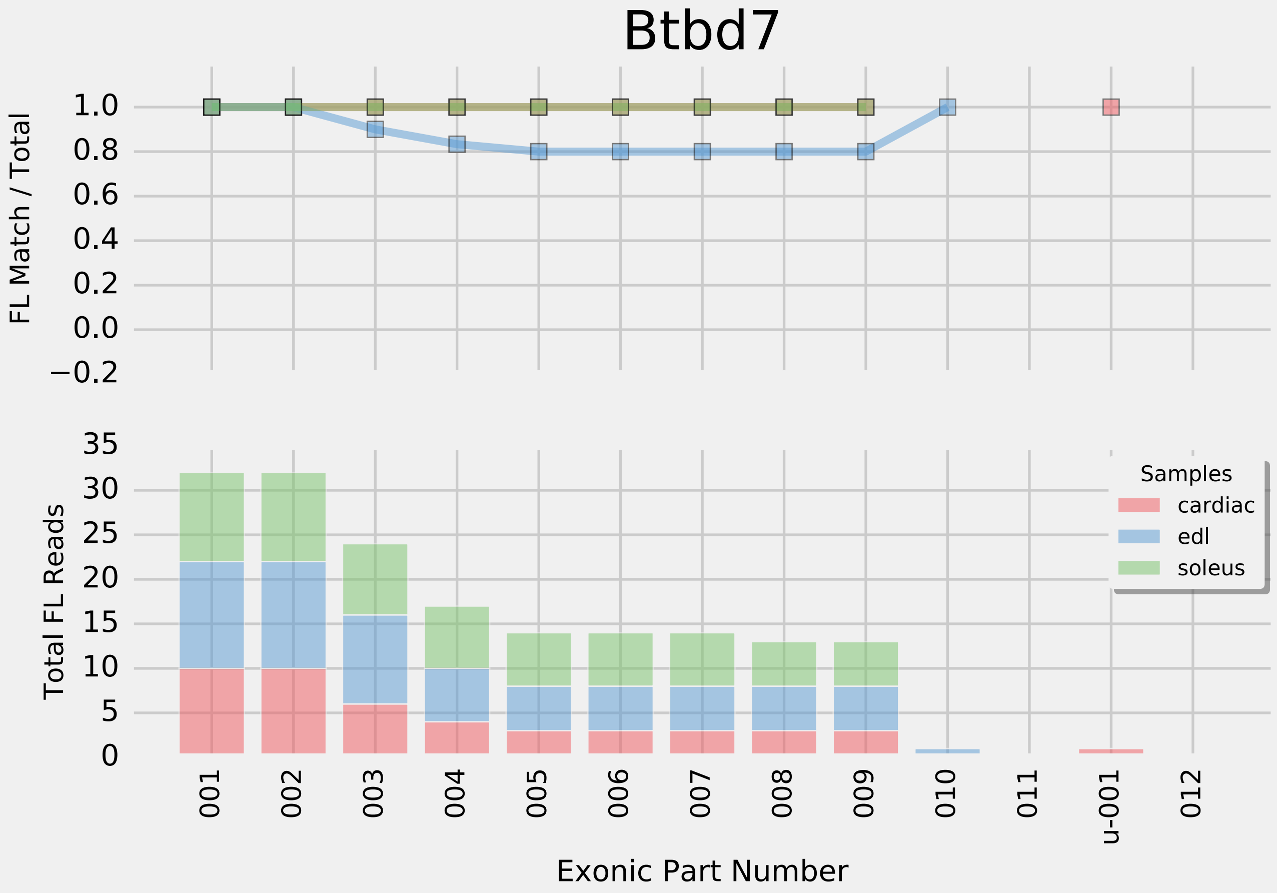

Btbd7
FL Match / Total
1.0
0.8
0.6
0.4
0.2
0.0
−0.2
Total FL Reads
35
30
25
20
15
10
5
0
Samples
cardiac
edl
soleus
001
002
003
004
005
006
007
008
009
010
011
u-001
012
Exonic Part Number

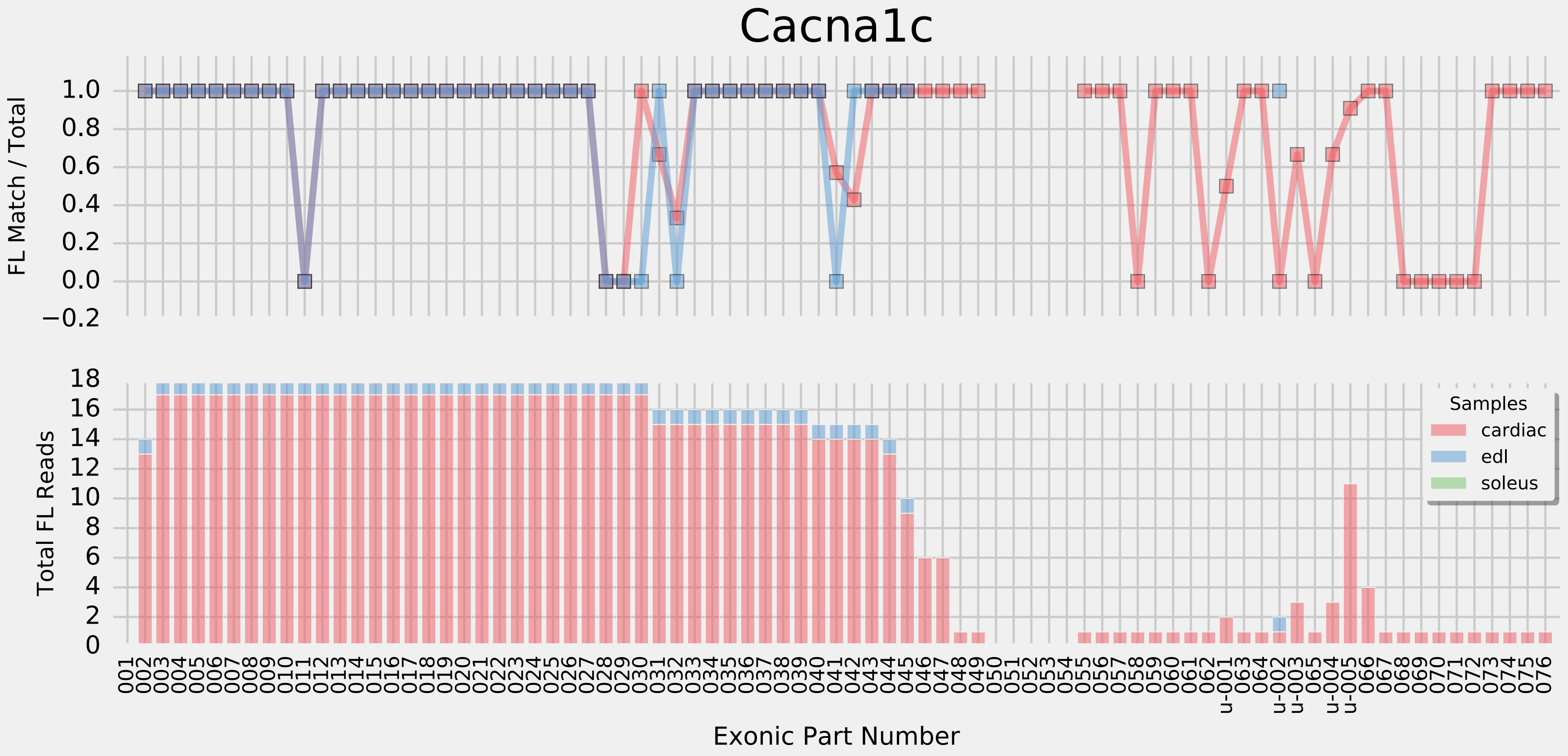

Cacna1c
FL Match / Total
Total FL Reads
Samples
cardiac
edl
soleus
Exonic Part Number

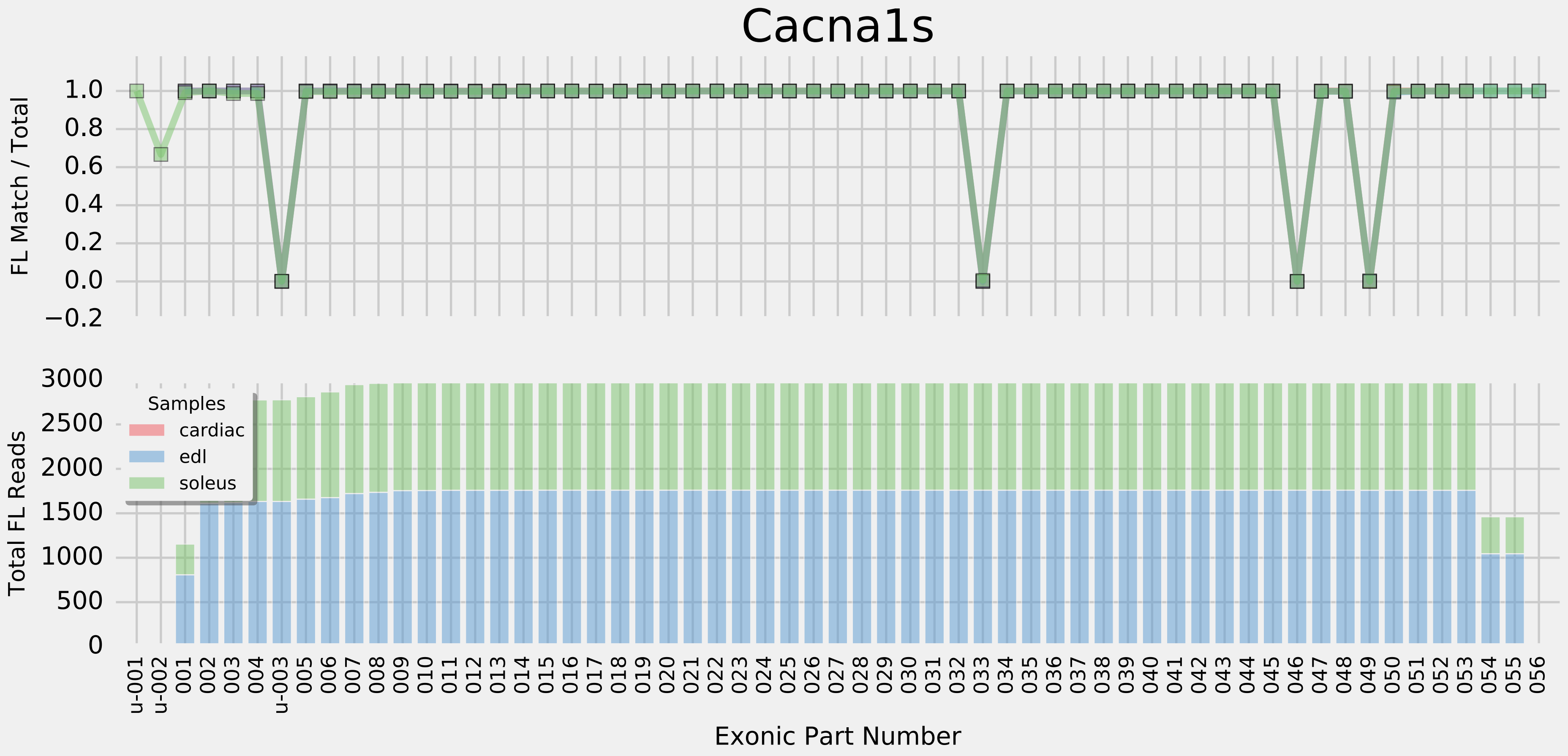

Cacna1s
FL Match / Total
Total FL Reads
Samples
cardiac
edl
soleus
Exonic Part Number

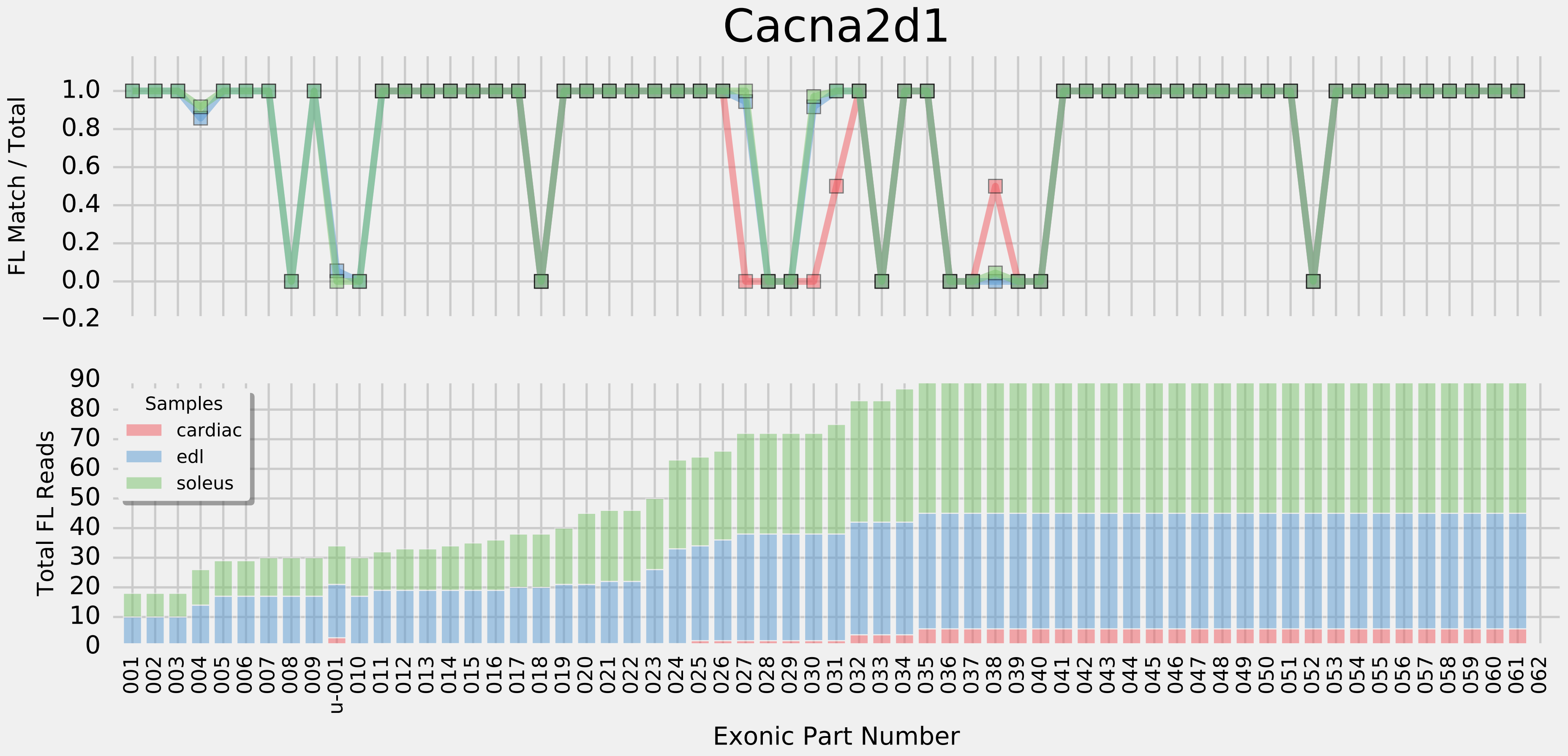

Cacna2d1
FL Match / Total
Total FL Reads
Samples
cardiac
edl
soleus
Exonic Part Number

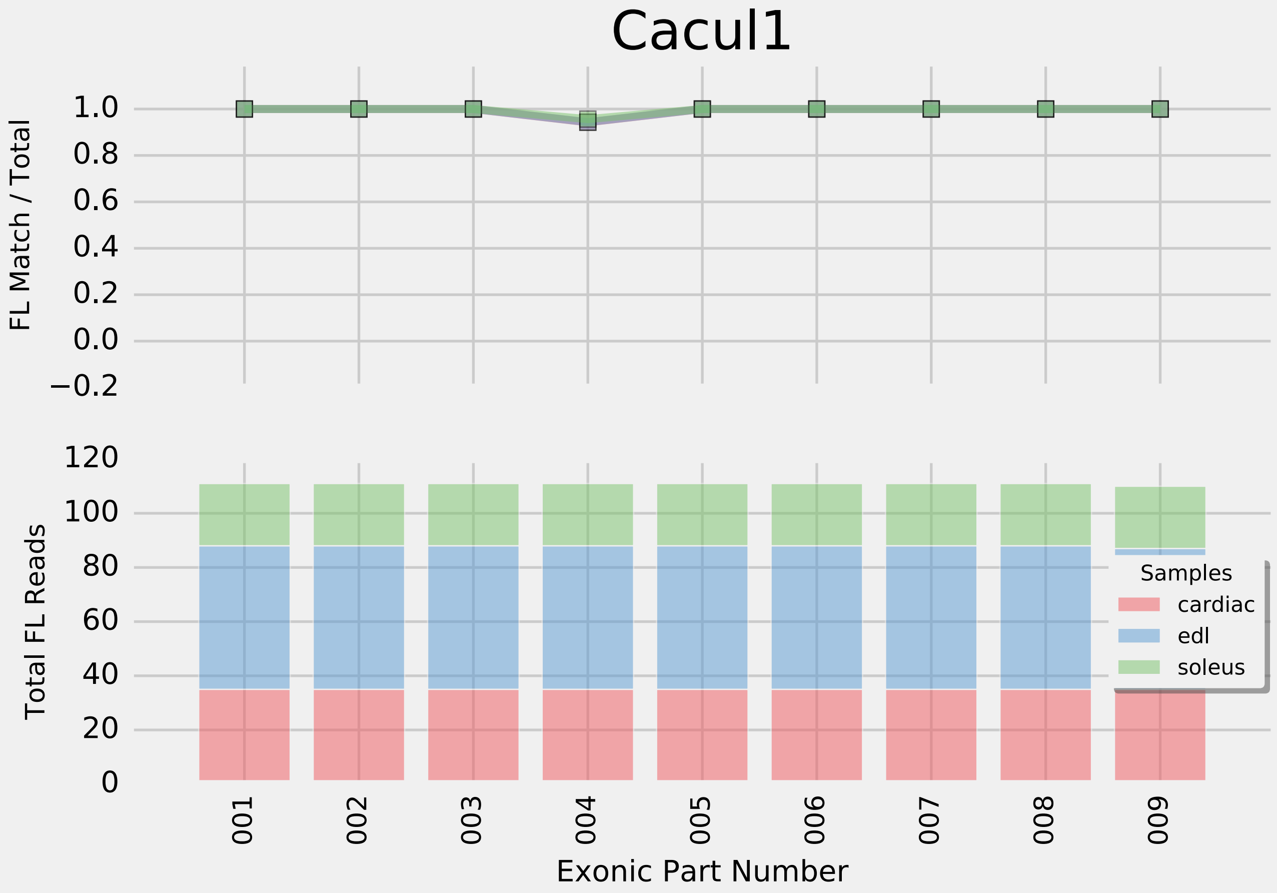
Cacul1
FL Match / Total
1.0
0.8
0.6
0.4
0.2
0.0
−0.2
Total FL Reads
120
100
80
60
40
20
0
001
002
003
004
005
006
007
008
009
Exonic Part Number
Samples
cardiac
edl
soleus

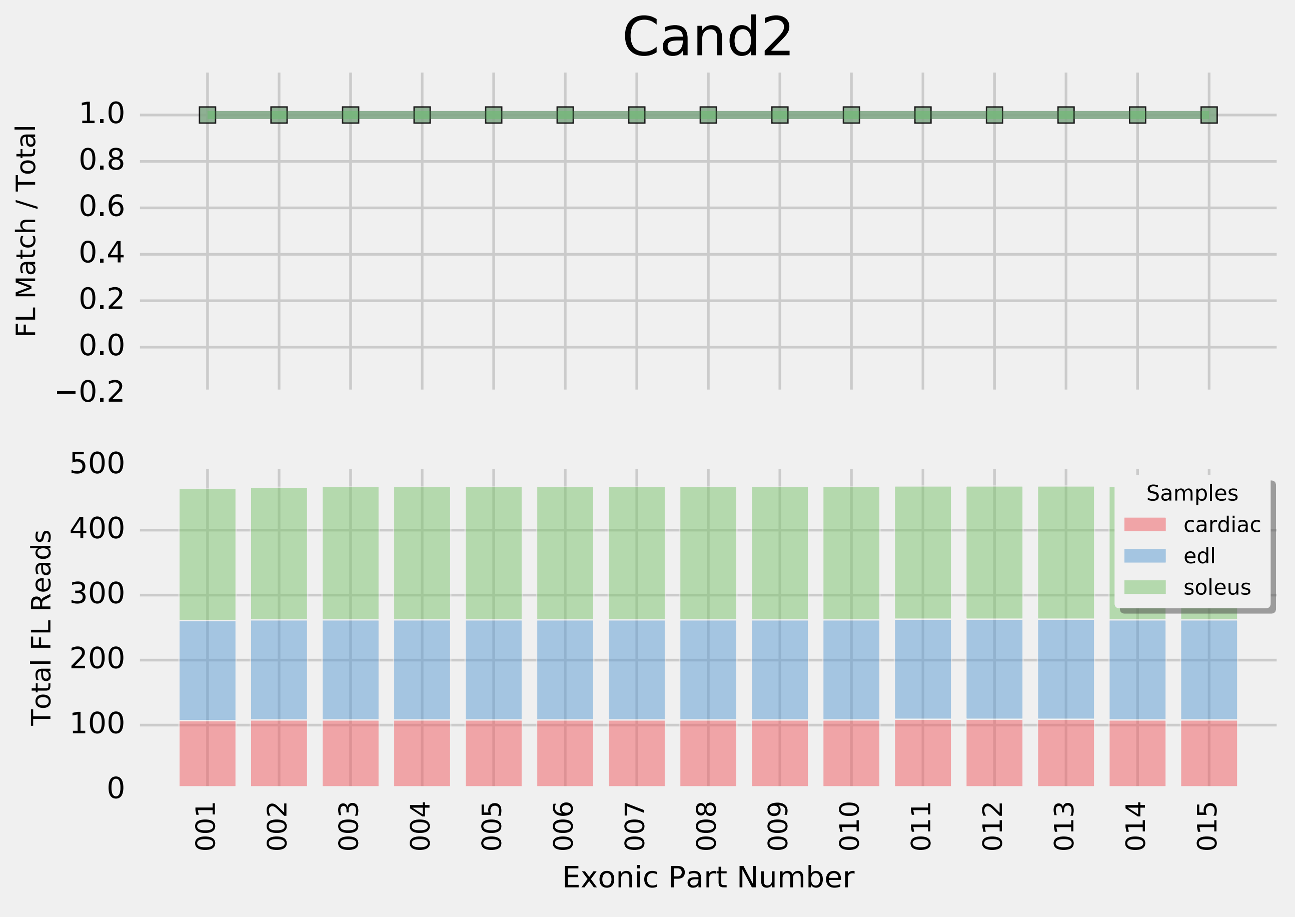

Cand2
FL Match / Total
1.0
0.8
0.6
0.4
0.2
0.0
−0.2
Total FL Reads
500
400
300
200
100
0
Samples
cardiac
edl
soleus
001
002
003
004
005
006
007
008
009
010
011
012
013
014
015
Exonic Part Number

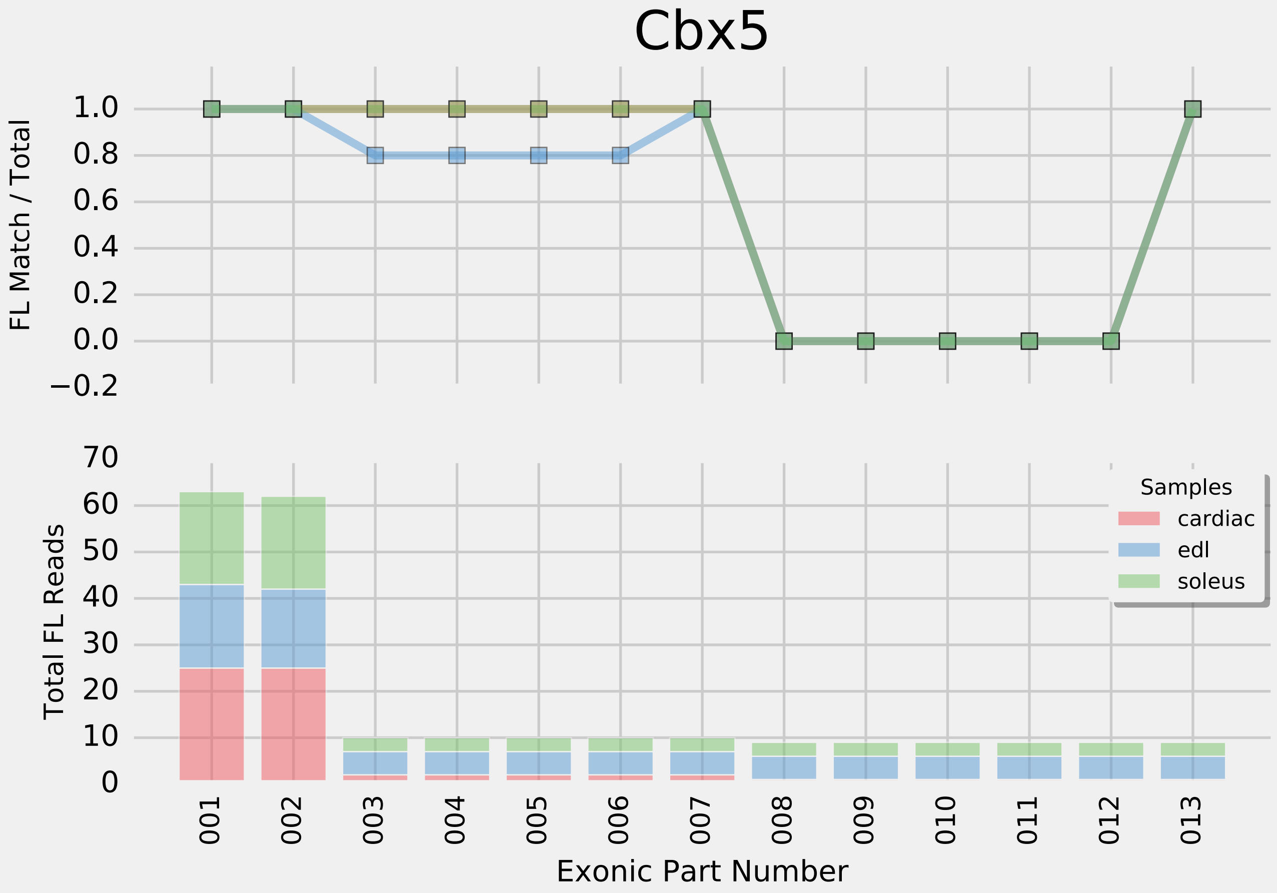
Cbx5
FL Match / Total
1.0
0.8
0.6
0.4
0.2
0.0
−0.2
Total FL Reads
70
60
50
40
30
20
10
0
Samples
cardiac
edl
soleus
001
002
003
004
005
006
007
008
009
010
011
012
013
Exonic Part Number

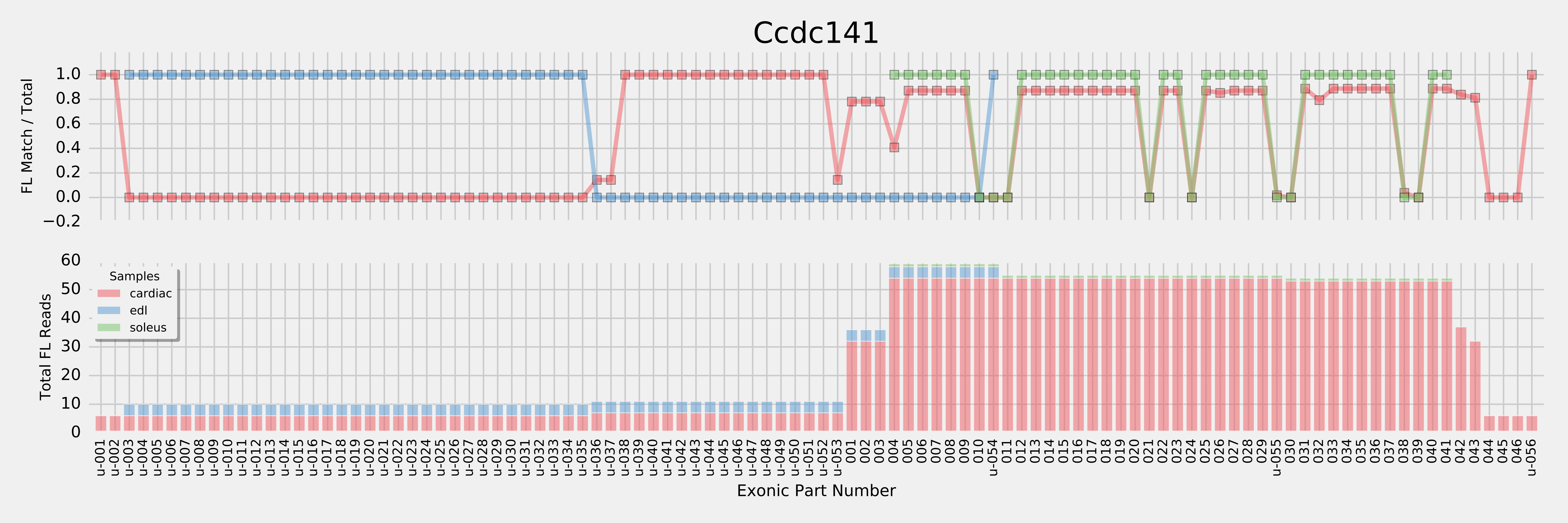
Ccdc141
FL Match / Total
Total FL Reads
Samples
cardiac
edl
soleus
Exonic Part Number

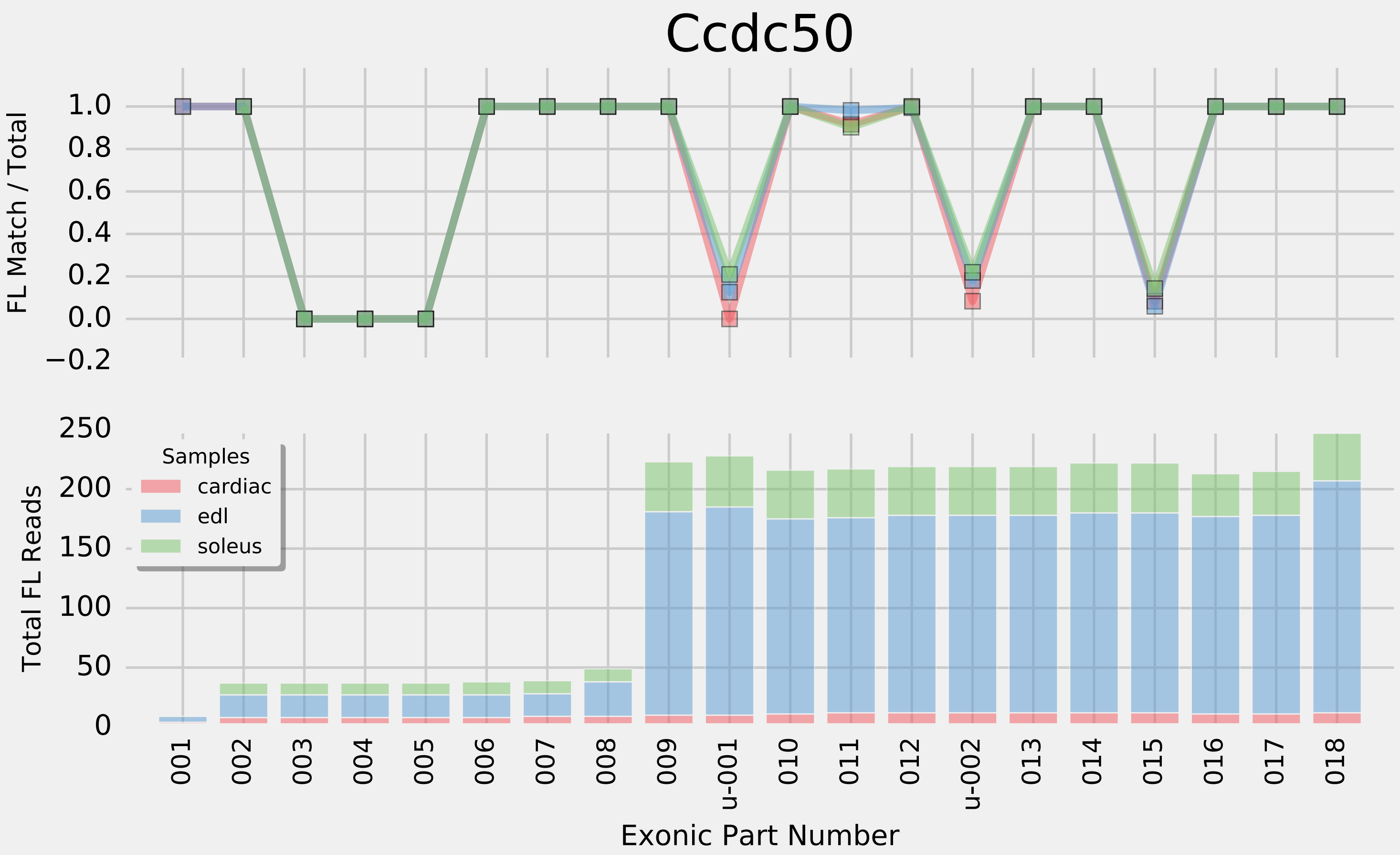
Ccdc50
FL Match / Total
1.0
0.8
0.6
0.4
0.2
0.0
−0.2
Total FL Reads
250
200
150
100
50
0
Samples
cardiac
edl
soleus
001
002
003
004
005
006
007
008
009
u-001
010
011
012
u-002
013
014
015
016
017
018
Exonic Part Number

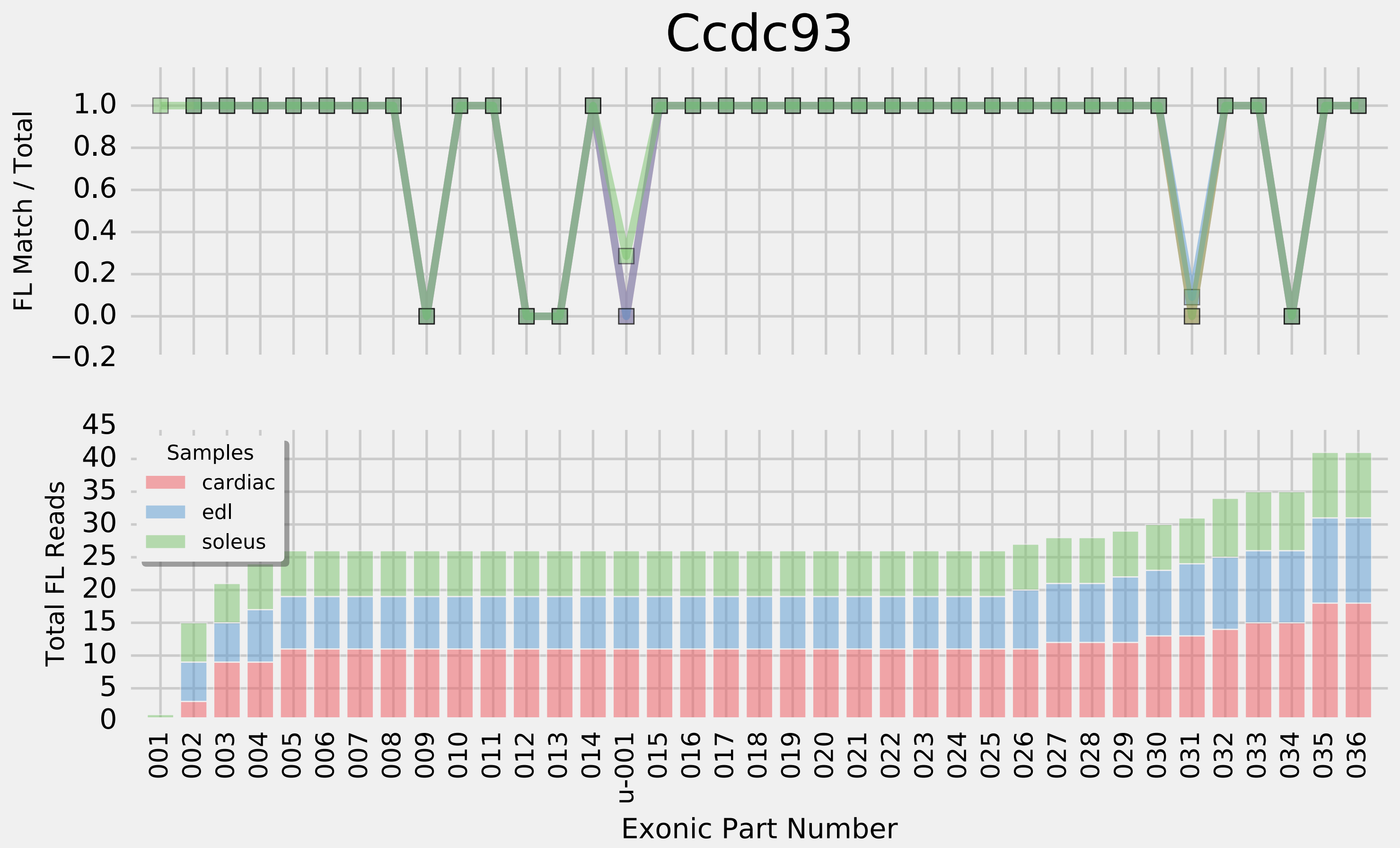

Ccdc93
FL Match / Total
1.0
0.8
0.6
0.4
0.2
0.0
−0.2
Total FL Reads
45
40
35
30
25
20
15
10
5
0
Samples
cardiac
edl
soleus
001
002
003
004
005
006
007
008
009
010
011
012
013
014
u-001
015
016
017
018
019
020
021
022
023
024
025
026
027
028
029
030
031
032
033
034
035
036
Exonic Part Number

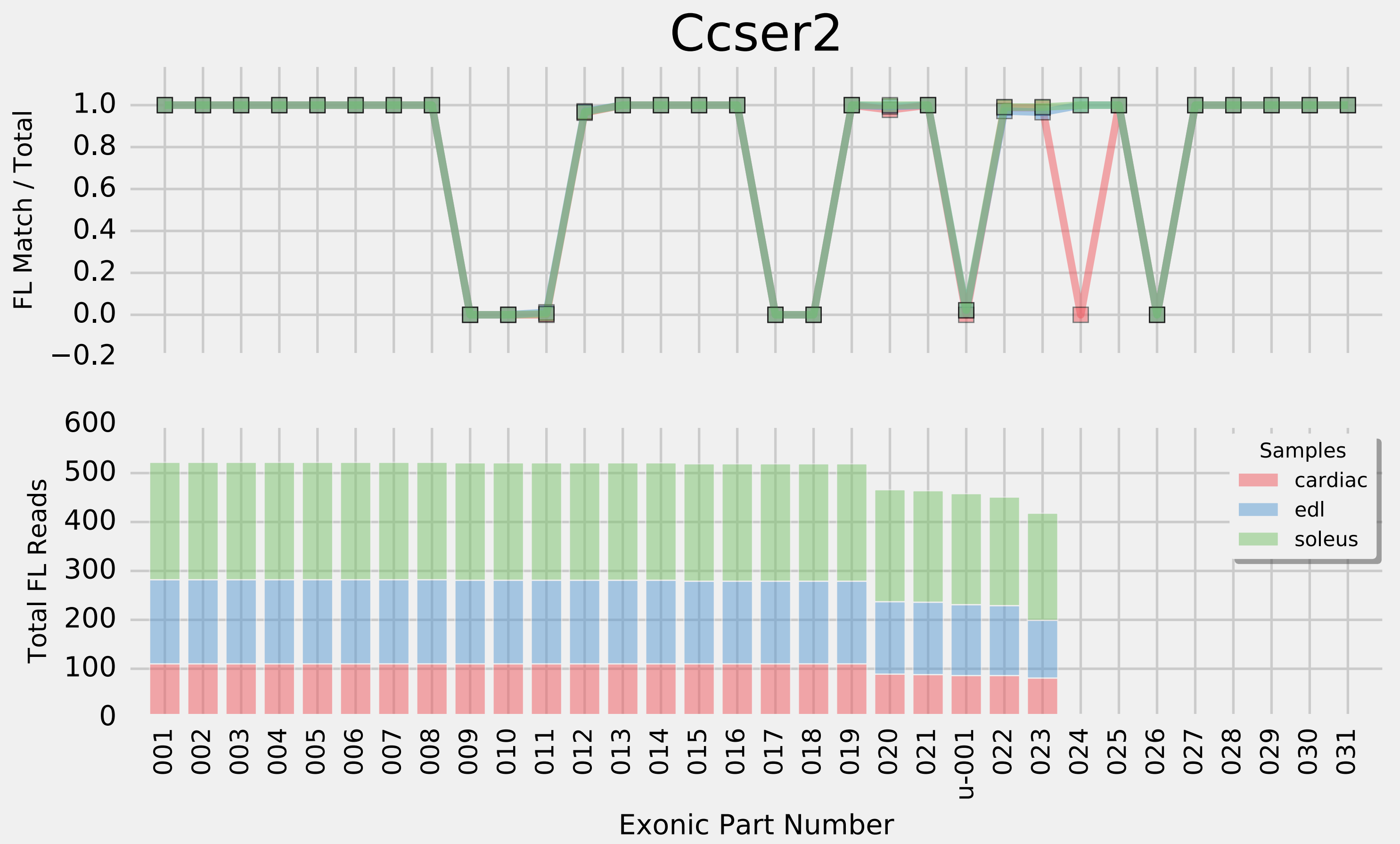

Ccser2
FL Match / Total
1.0
0.8
0.6
0.4
0.2
0.0
−0.2
Total FL Reads
600
500
400
300
200
100
0
Samples
cardiac
edl
soleus
001 002 003 004 005 006 007 008 009 010 011 012 013 014 015 016 017 018 019 020 021 u-001 022 023 024 025 026 027 028 029 030 031
Exonic Part Number

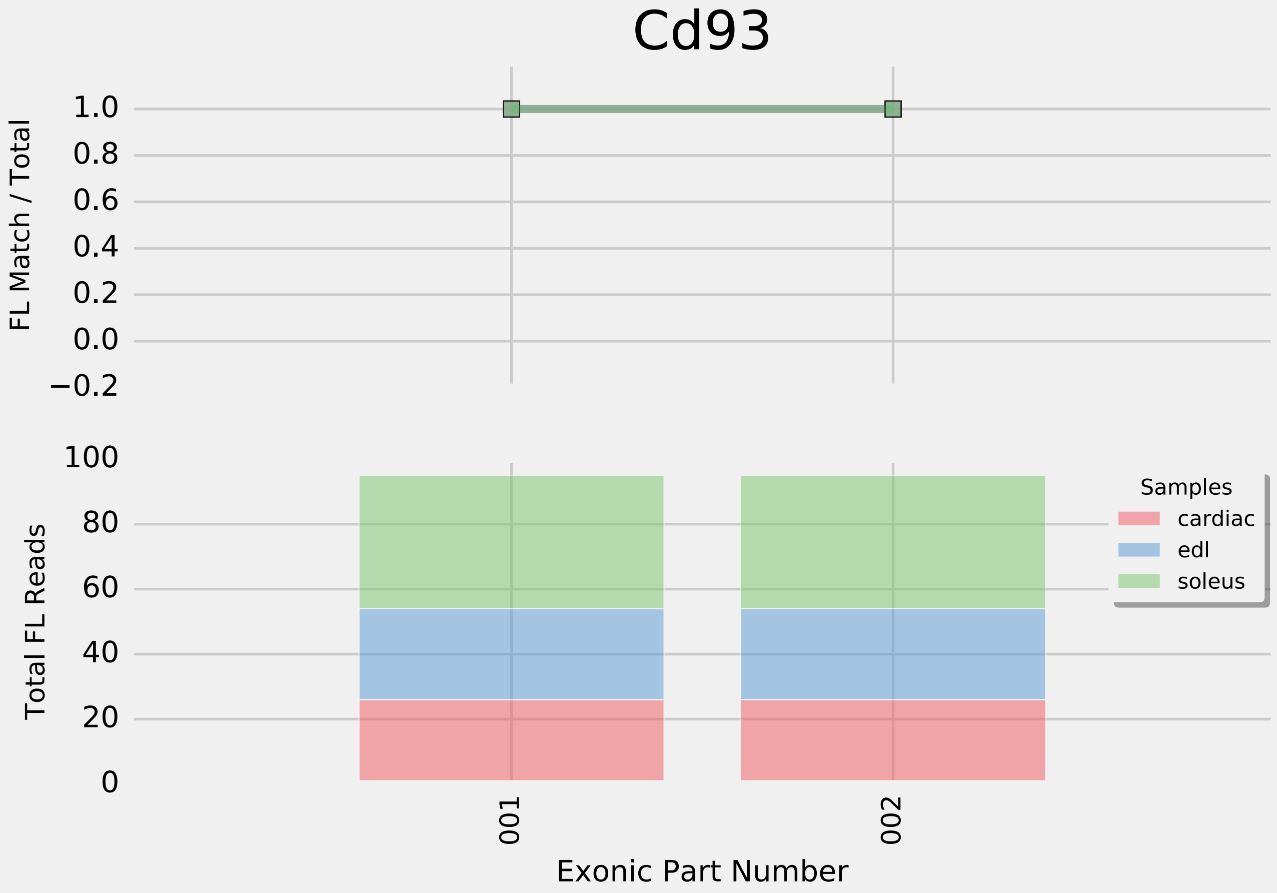
Cd93
FL Match / Total
1.0
0.8
0.6
0.4
0.2
0.0
−0.2
Total FL Reads
100
80
60
40
20
0
Samples
cardiac
edl
soleus
001
002
Exonic Part Number

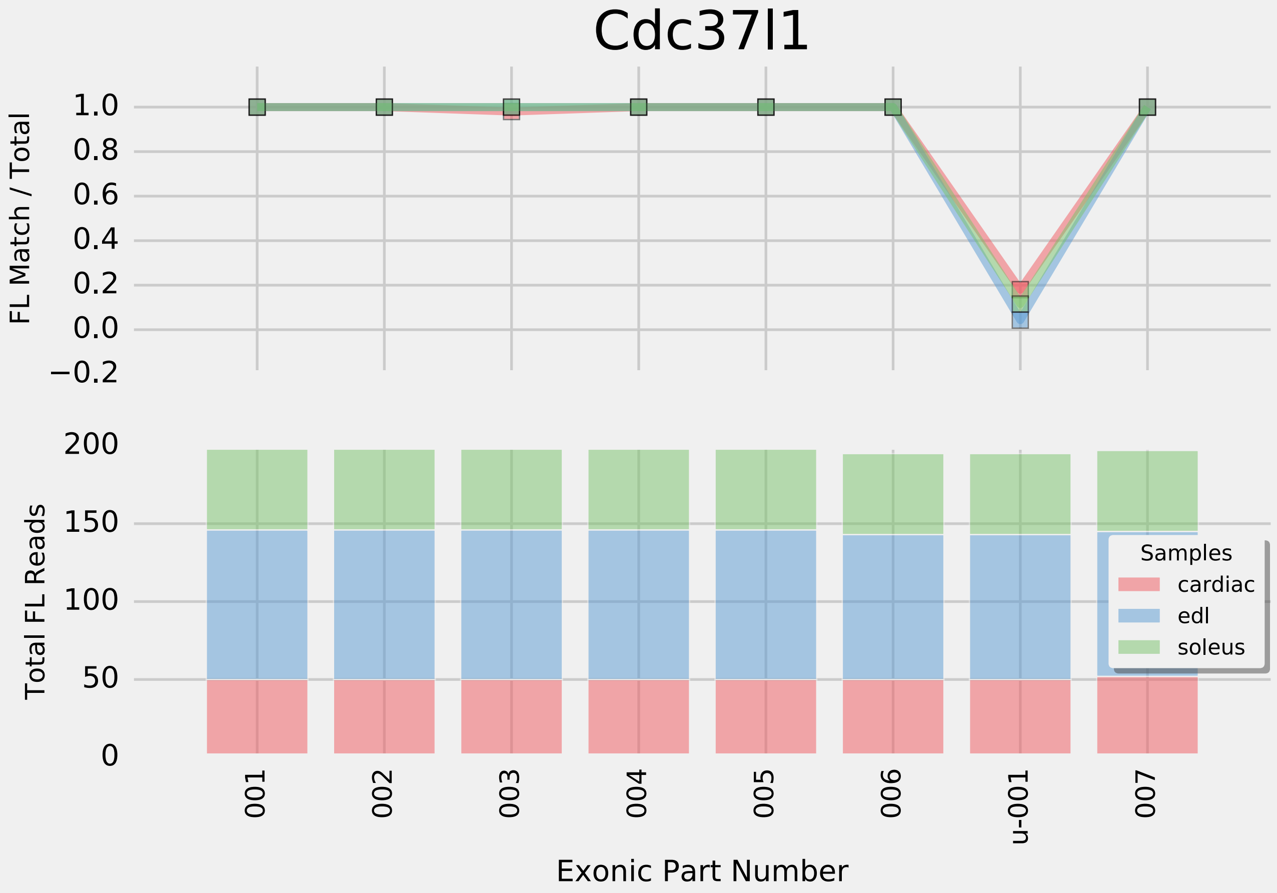

Cdc37l1
FL Match / Total
1.0
0.8
0.6
0.4
0.2
0.0
−0.2
Total FL Reads
200
150
100
50
0
001
002
003
004
005
006
u-001
007
Exonic Part Number
Samples
cardiac
edl
soleus

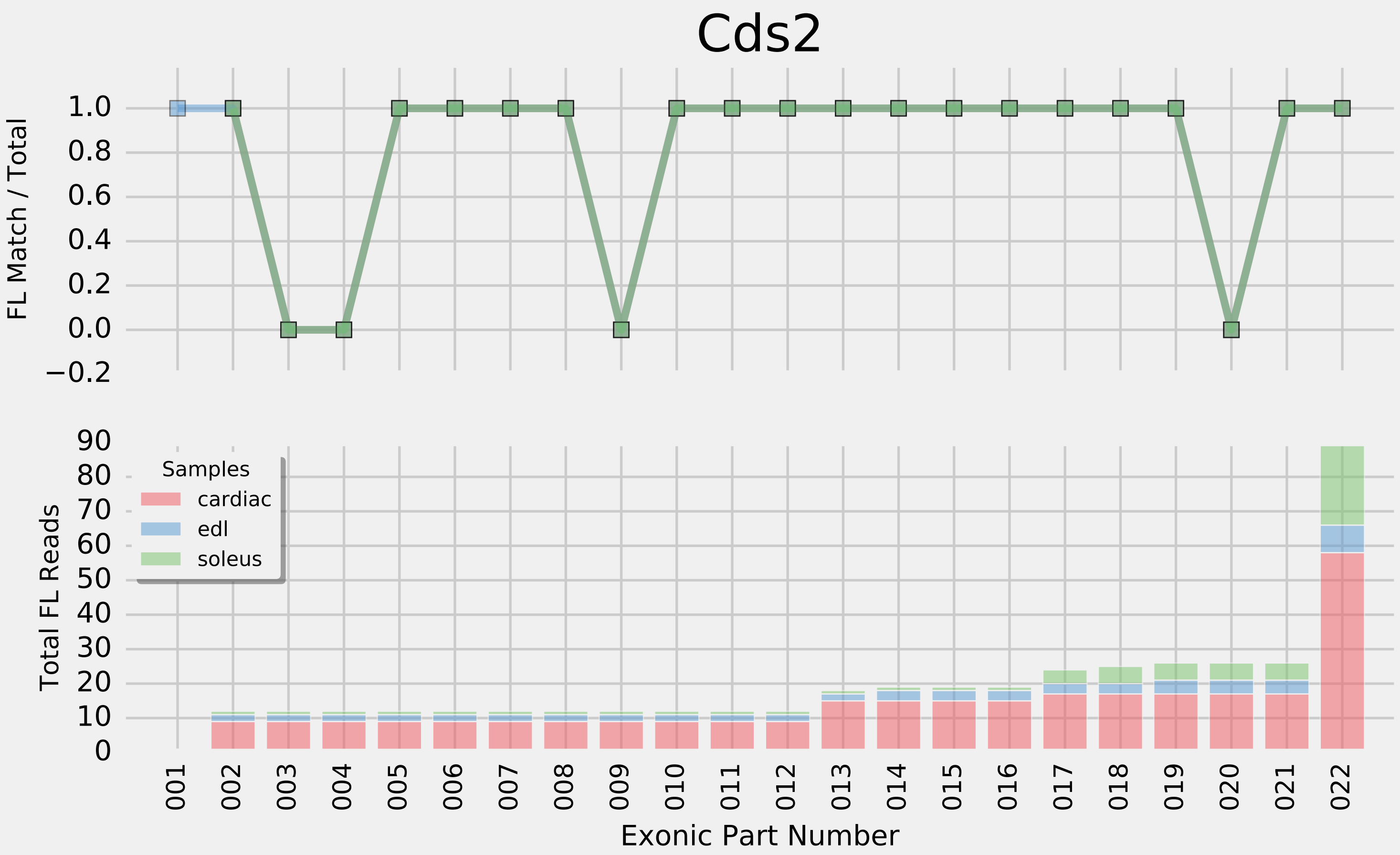

Cds2
FL Match / Total
1.0
0.8
0.6
0.4
0.2
0.0
−0.2
Total FL Reads
90
80
70
60
50
40
30
20
10
0
Samples
cardiac
edl
soleus
001
002
003
004
005
006
007
008
009
010
011
012
013
014
015
016
017
018
019
020
021
022
Exonic Part Number

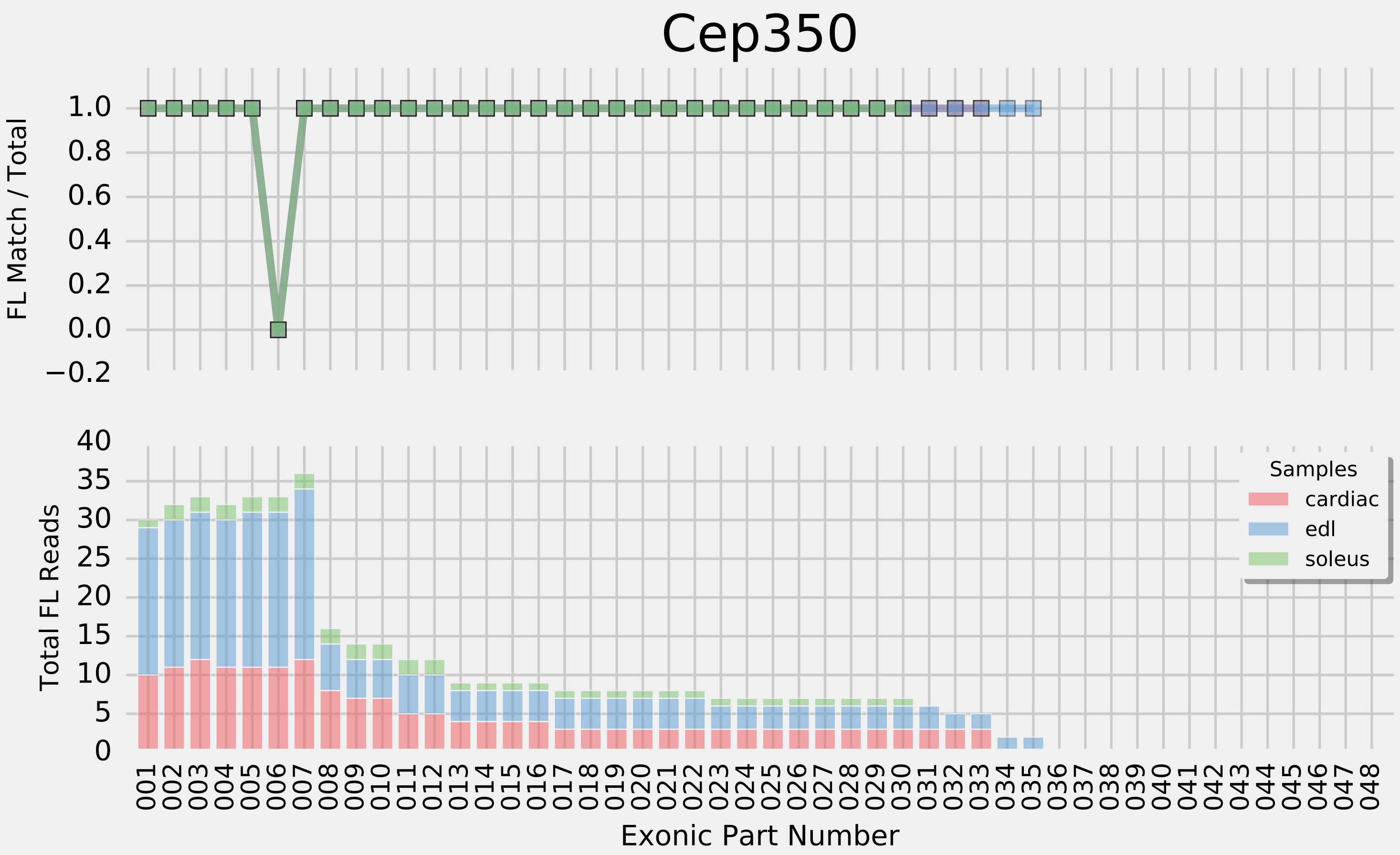

Cep350
FL Match / Total
1.0
0.8
0.6
0.4
0.2
0.0
−0.2
Total FL Reads
40
35
30
25
20
15
10
5
0
Samples
cardiac
edl
soleus
001 002 003 004 005 006 007 008 009 010 011 012 013 014 015 016 017 018 019 020 021 022 023 024 025 026 027 028 029 030 031 032 033 034 035 036 037 038 039 040 041 042 043 044 045 046 047 048
Exonic Part Number

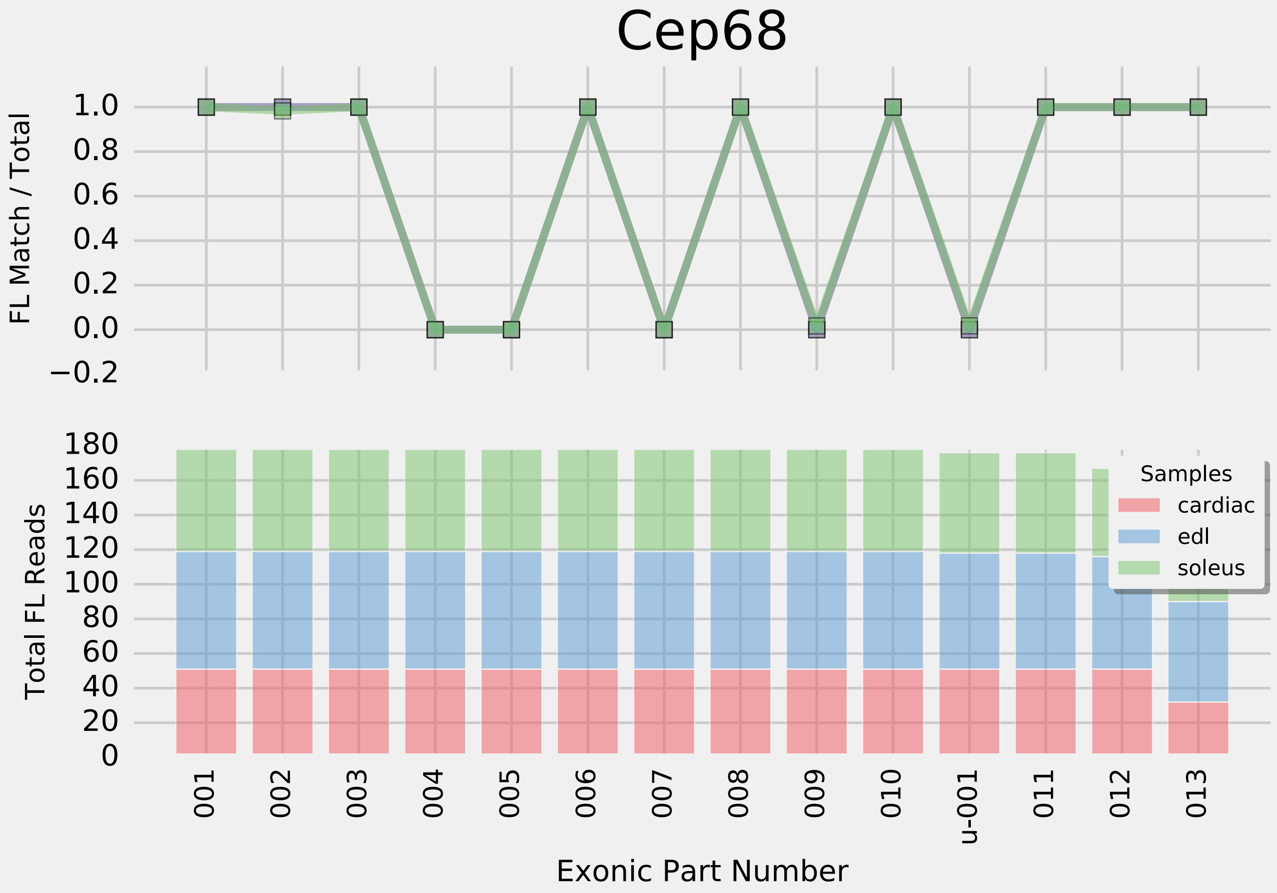

Cep68
FL Match / Total
1.0
0.8
0.6
0.4
0.2
0.0
−0.2
Total FL Reads
180
160
140
120
100
80
60
40
20
0
Samples
cardiac
edl
soleus
001
002
003
004
005
006
007
008
009
010
u-001
011
012
013
Exonic Part Number

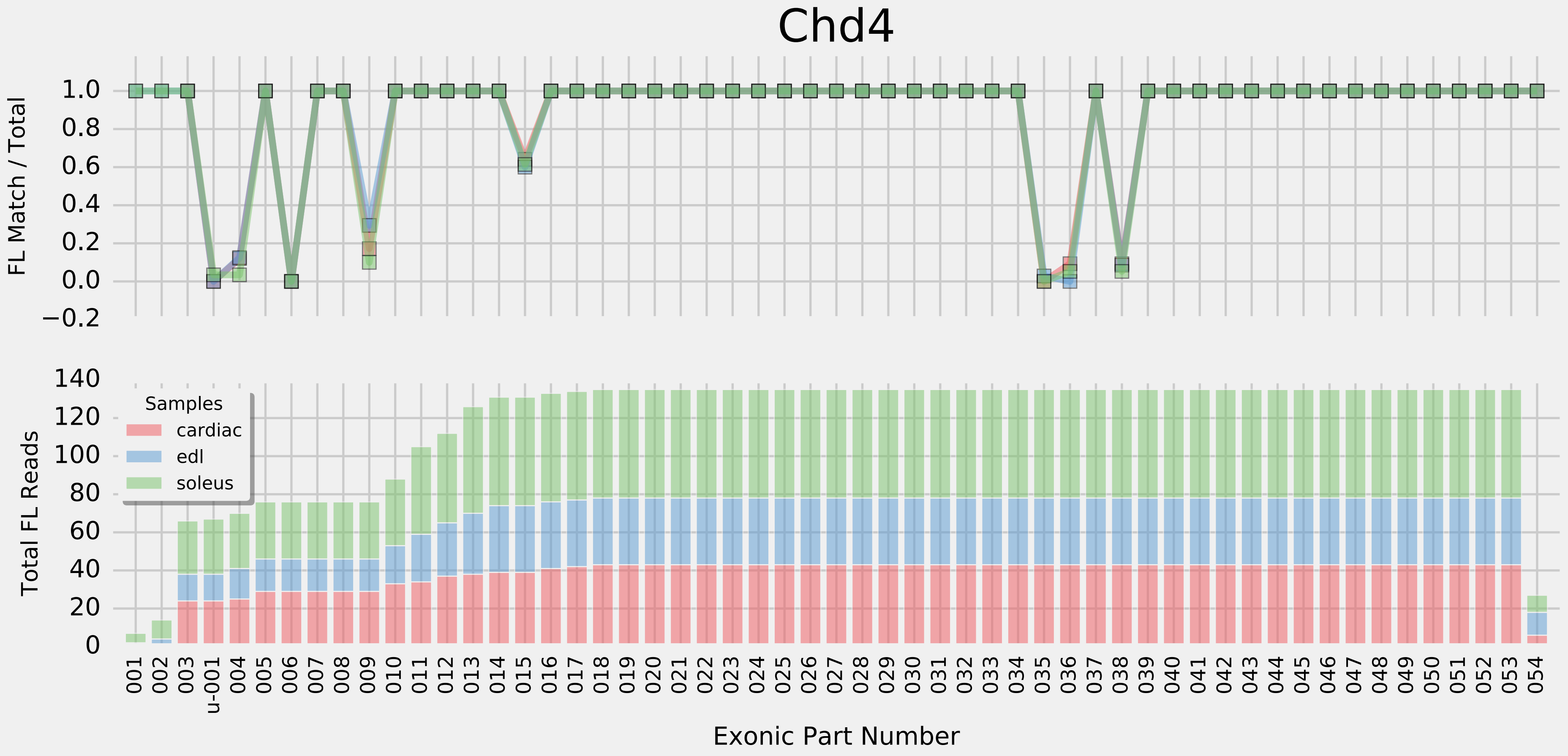

Chd4
FL Match / Total
Total FL Reads
Samples
cardiac
edl
soleus
Exonic Part Number

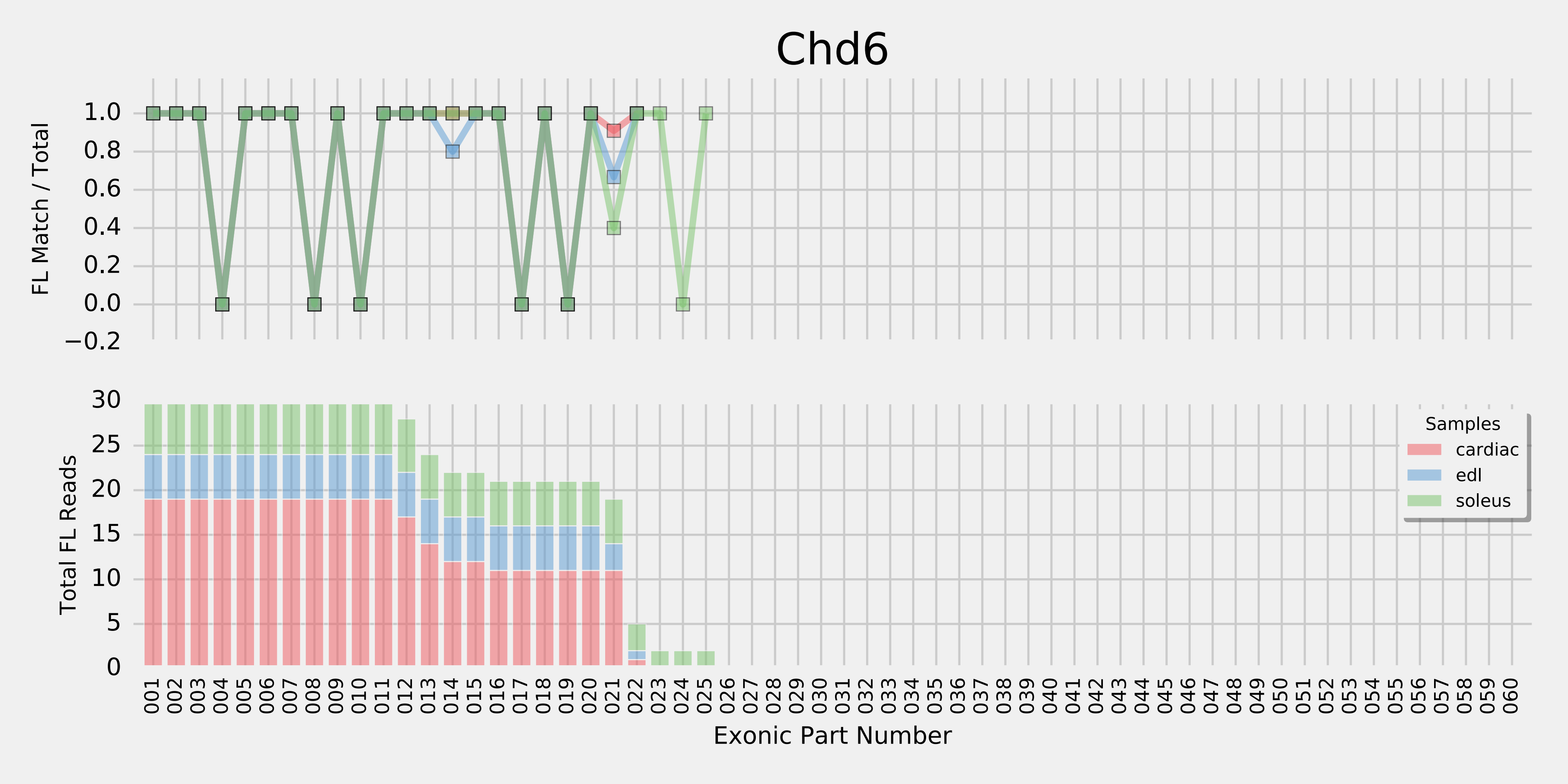
Chd6
FL Match / Total
1.0
0.8
0.6
0.4
0.2
0.0
−0.2
Total FL Reads
30
25
20
15
10
5
0
Samples
cardiac
edl
soleus
001 002 003 004 005 006 007 008 009 010 011 012 013 014 015 016 017 018 019 020 021 022 023 024 025 026 027 028 029 030 031 032 033 034 035 036 037 038 039 040 041 042 043 044 045 046 047 048 049 050 051 052 053 054 055 056 057 058 059 060
Exonic Part Number

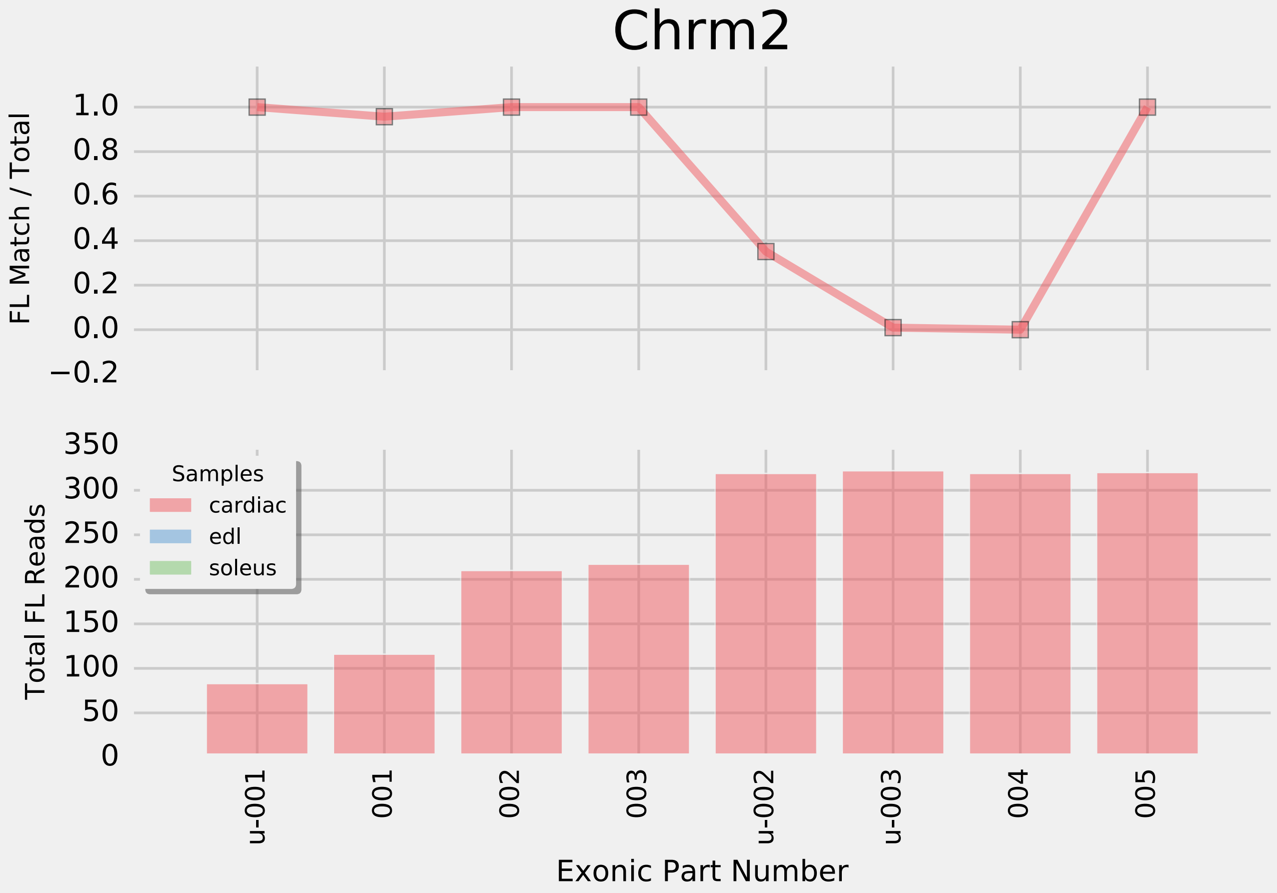

Chrm2
FL Match / Total
1.0
0.8
0.6
0.4
0.2
0.0
−0.2
Total FL Reads
350
300
250
200
150
100
50
0
Samples
cardiac
edl
soleus
u-001
001
002
003
u-002
u-003
004
005
Exonic Part Number

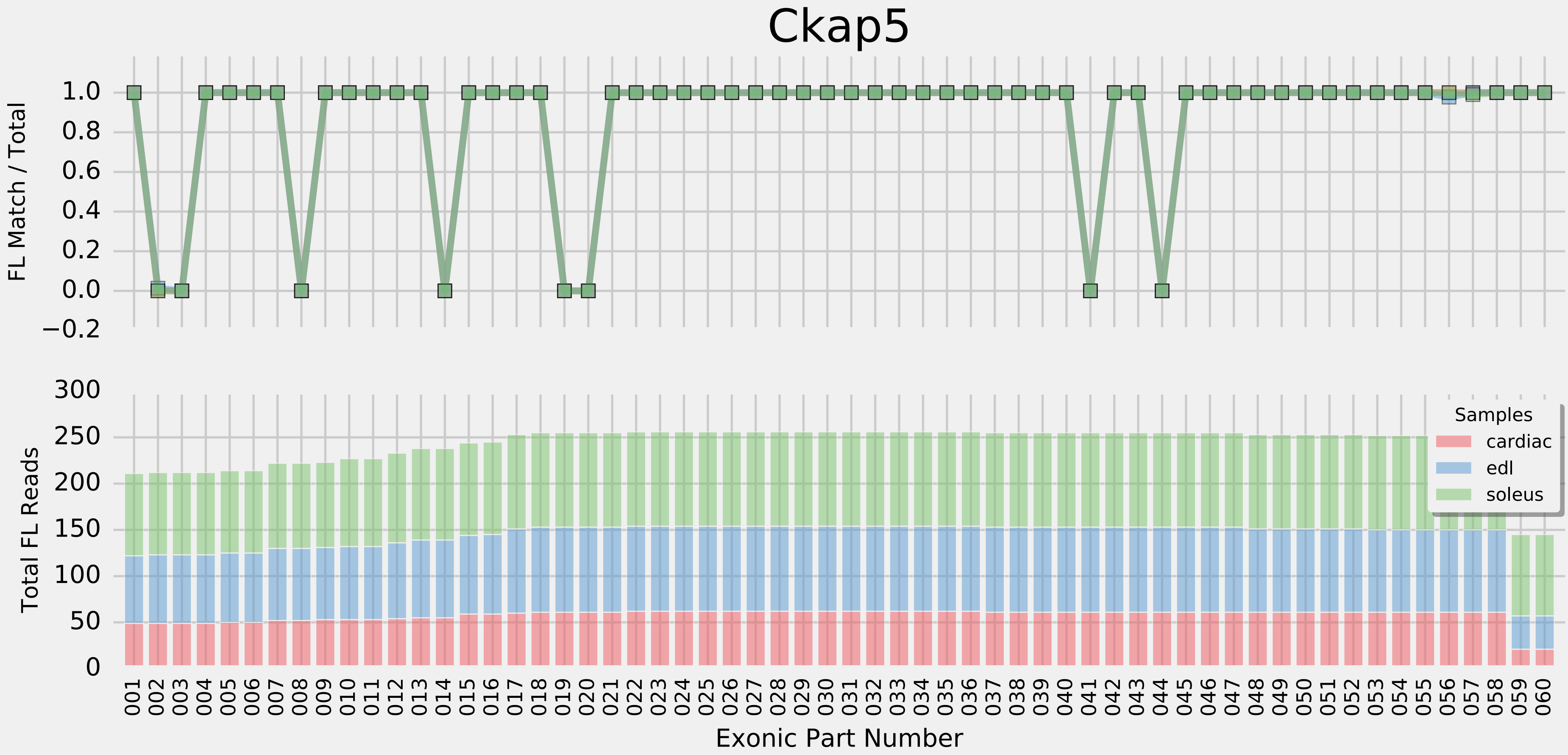

Ckap5
FL Match / Total
1.0
0.8
0.6
0.4
0.2
0.0
−0.2
Total FL Reads
300
250
200
150
100
50
0
Samples
cardiac
edl
soleus
001 002 003 004 005 006 007 008 009 010 011 012 013 014 015 016 017 018 019 020 021 022 023 024 025 026 027 028 029 030 031 032 033 034 035 036 037 038 039 040 041 042 043 044 045 046 047 048 049 050 051 052 053 054 055 056 057 058 059 060
Exonic Part Number

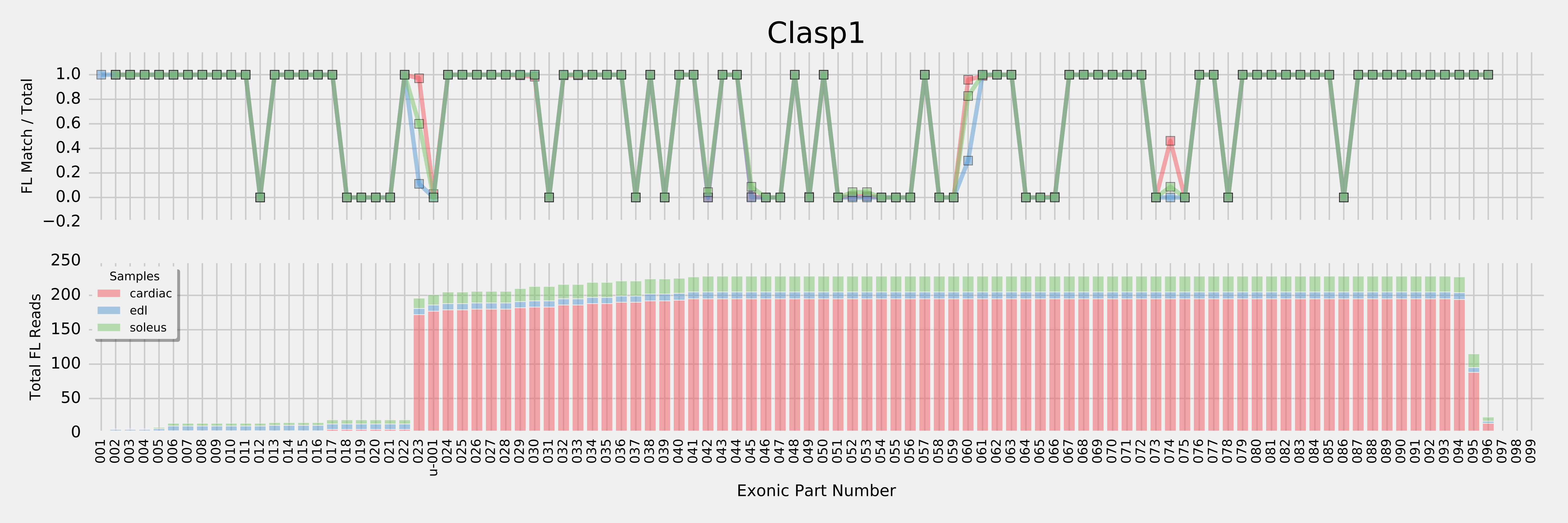

Clasp1
FL Match / Total
Total FL Reads
Samples
cardiac
edl
soleus
Exonic Part Number
u-001

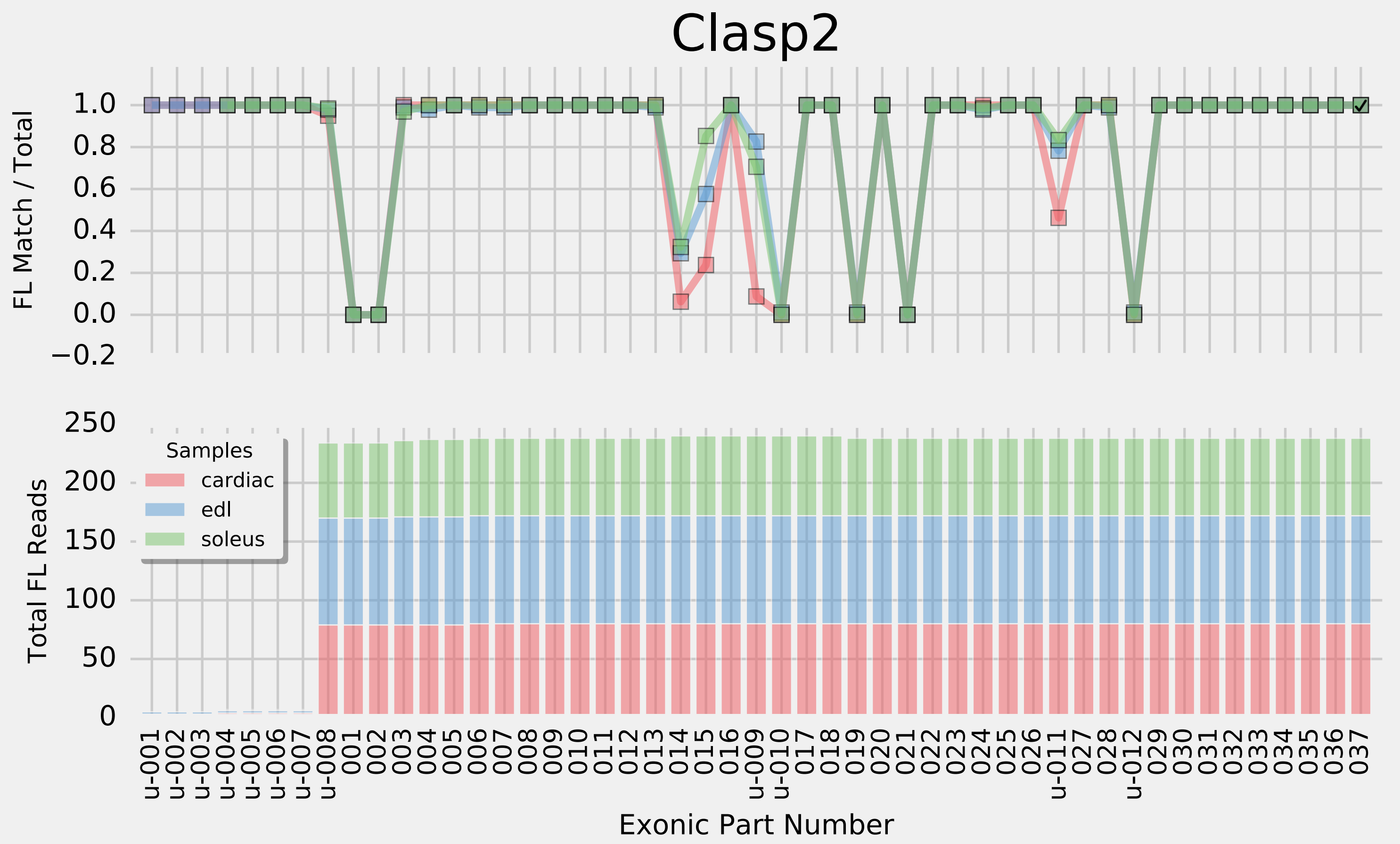

Clasp2
FL Match / Total
1.0
0.8
0.6
0.4
0.2
0.0
−0.2
Total FL Reads
250
200
150
100
50
0
Samples
cardiac
edl
soleus
u-001
u-002
u-003
u-004
u-005
u-006
u-007
u-008
001
002
003
004
005
006
007
008
009
010
011
012
013
014
015
016
u-009
u-010
017
018
019
020
021
022
023
024
025
026
u-011
027
028
u-012
029
030
031
032
033
034
035
036
037
Exonic Part Number

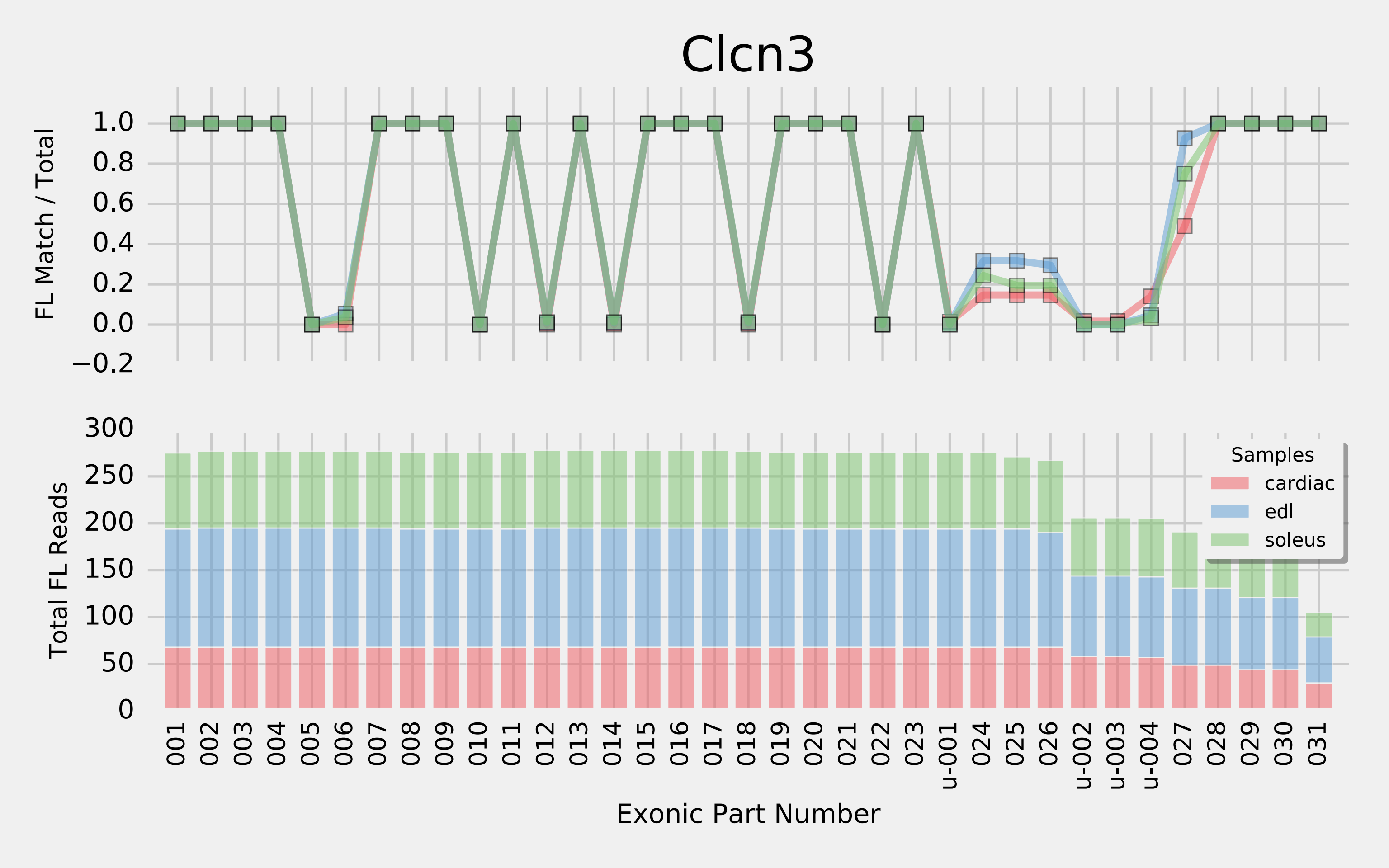

Clcn3
FL Match / Total
1.0
0.8
0.6
0.4
0.2
0.0
−0.2
Total FL Reads
300
250
200
150
100
50
0
Samples
cardiac
edl
soleus
001 002 003 004 005 006 007 008 009 010 011 012 013 014 015 016 017 018 019 020 021 022 023 u-001 024 025 026 u-002 u-003 u-004 027 028 029 030 031
Exonic Part Number

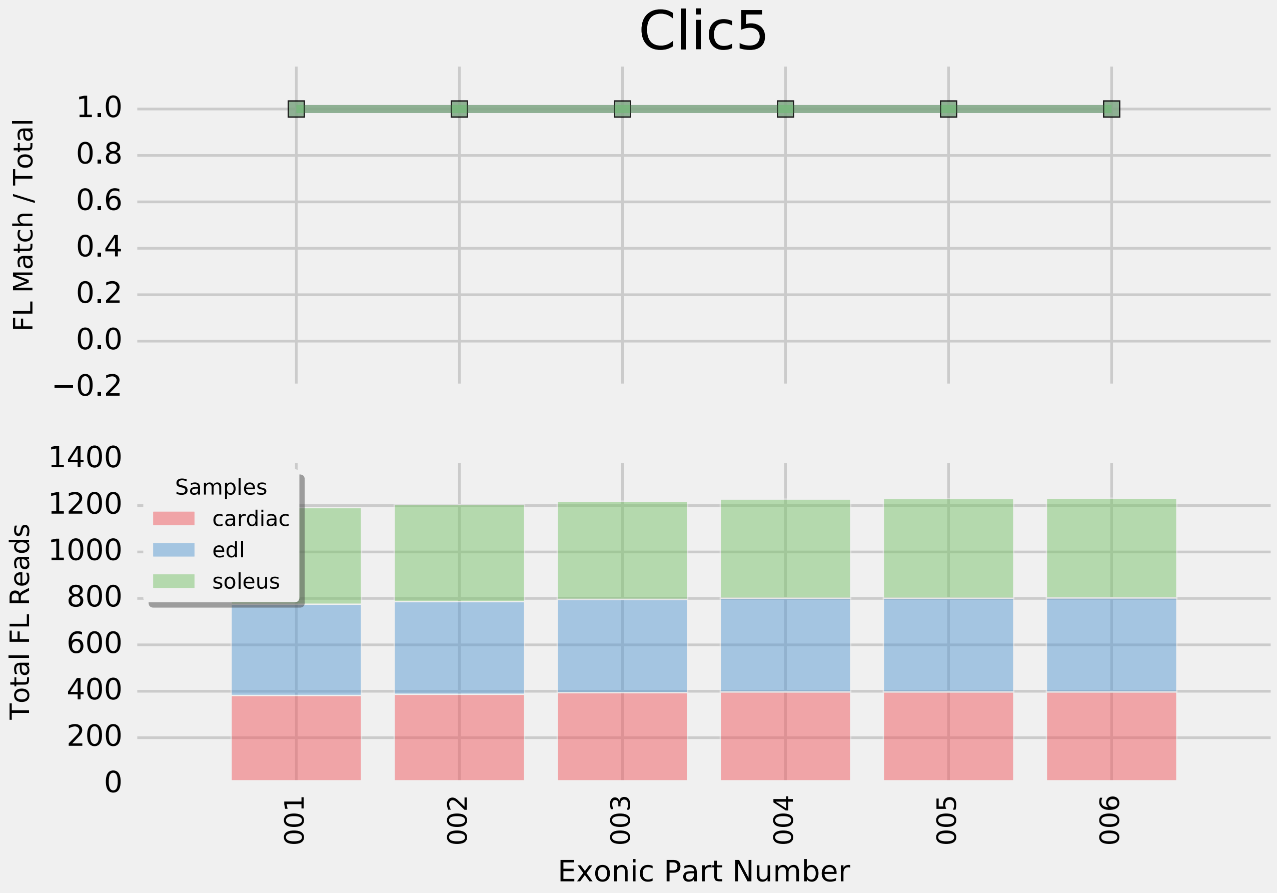

Clic5
FL Match / Total
1.0
0.8
0.6
0.4
0.2
0.0
−0.2
Total FL Reads
1400
1200
1000
800
600
400
200
0
Samples
cardiac
edl
soleus
001
002
003
004
005
006
Exonic Part Number

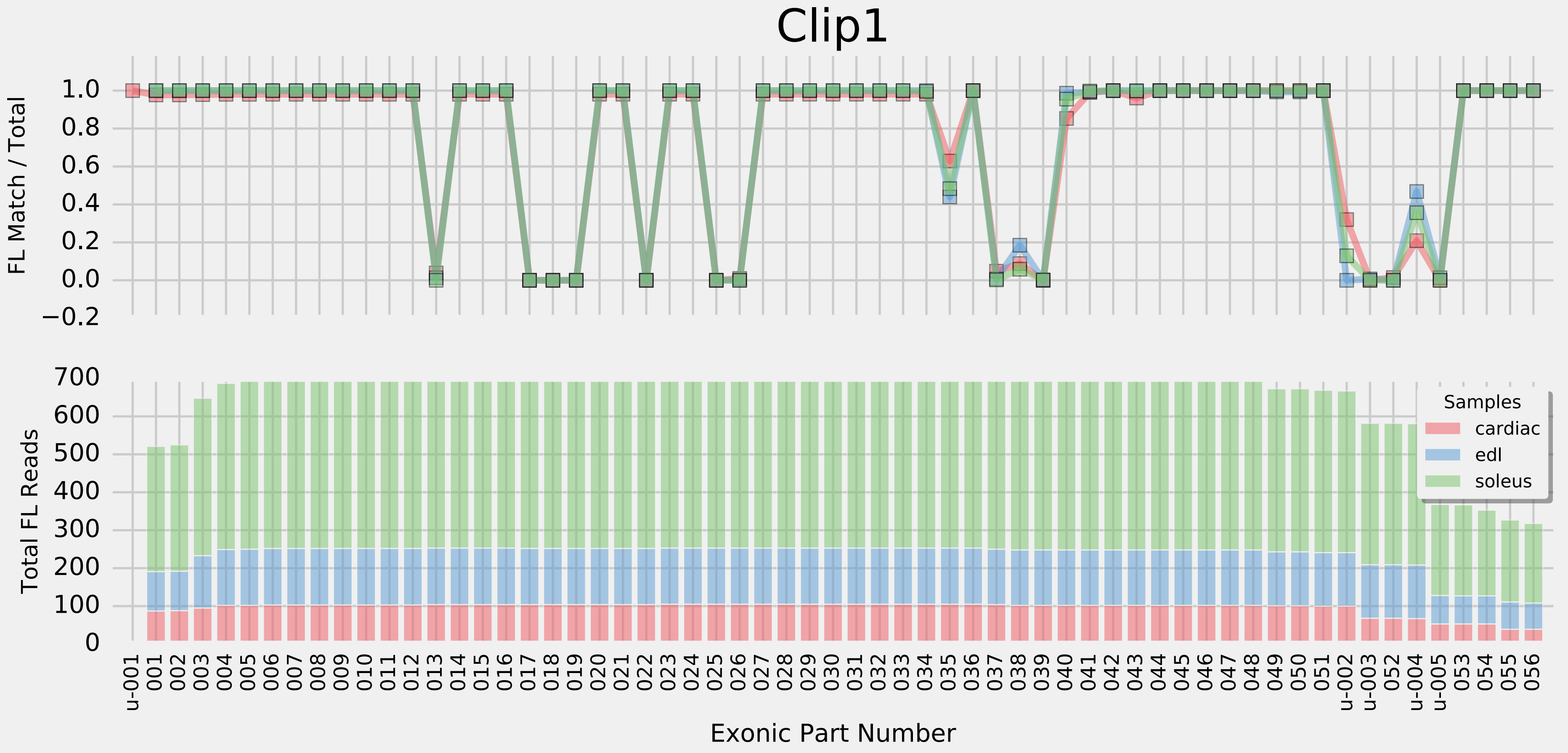

Clip1
FL Match / Total
1.0
0.8
0.6
0.4
0.2
0.0
−0.2
Total FL Reads
700
600
500
400
300
200
100
0
Samples
cardiac
edl
soleus
u-001 001 002 003 004 005 006 007 008 009 010 011 012 013 014 015 016 017 018 019 020 021 022 023 024 025 026 027 028 029 030 031 032 033 034 035 036 037 038 039 040 041 042 043 044 045 046 047 048 049 050 051 u-002 u-003 052 u-004 u-005 053 054 055 056
Exonic Part Number

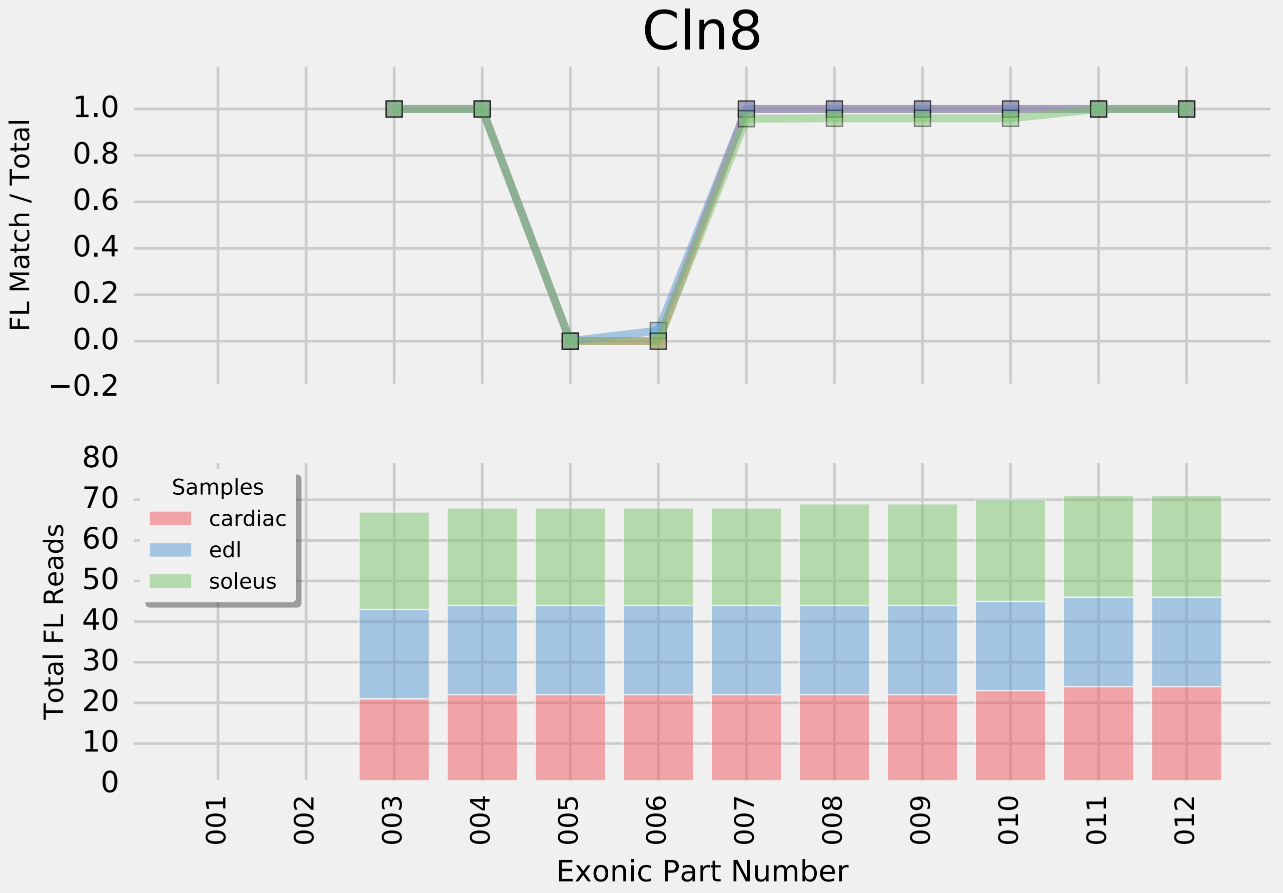

Cln8
FL Match / Total
1.0
0.8
0.6
0.4
0.2
0.0
−0.2
Total FL Reads
80
70
60
50
40
30
20
10
0
Samples
cardiac
edl
soleus
001
002
003
004
005
006
007
008
009
010
011
012
Exonic Part Number

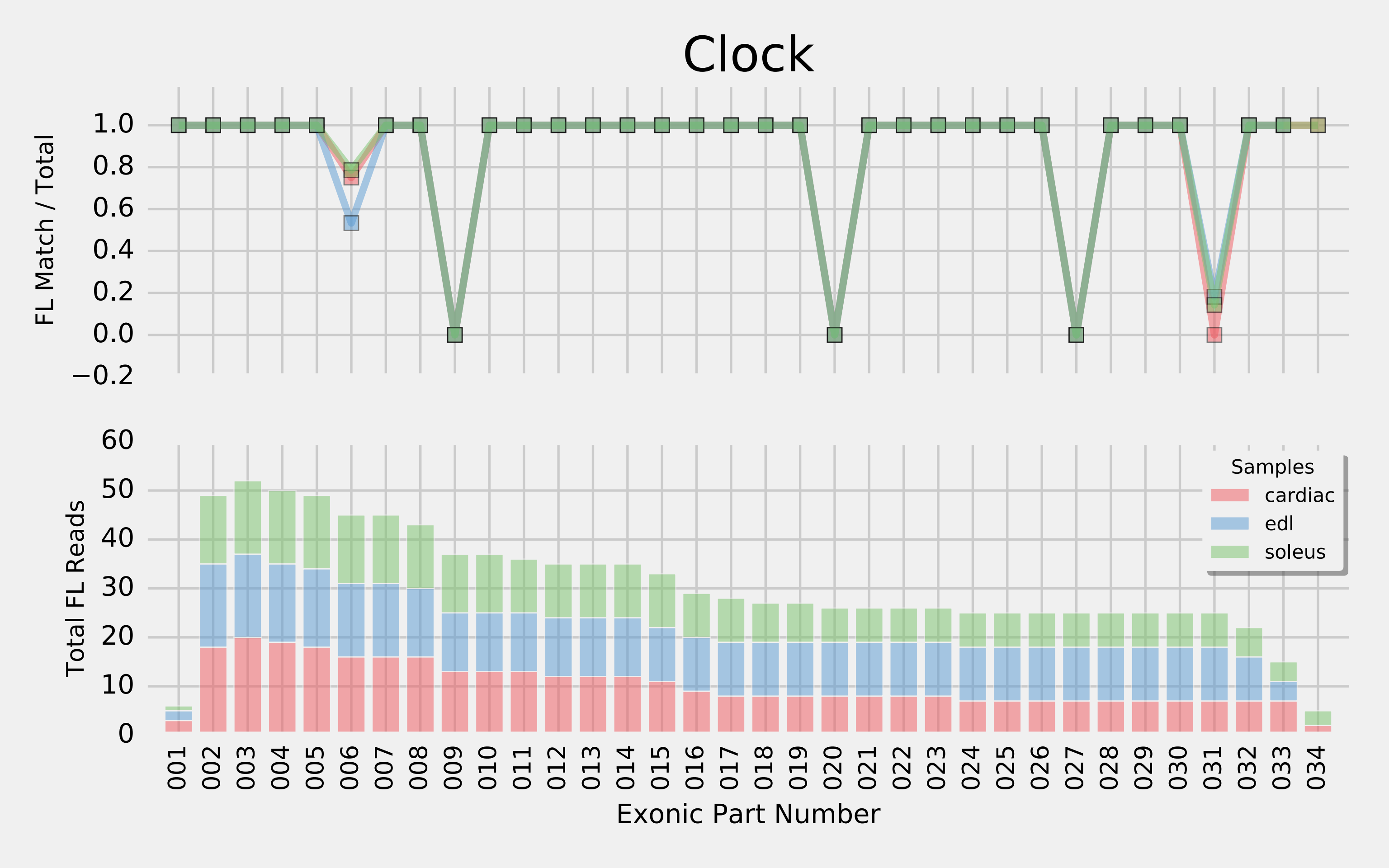
Clock
FL Match / Total
1.0
0.8
0.6
0.4
0.2
0.0
−0.2
Total FL Reads
60
50
40
30
20
10
0
Samples
cardiac
edl
soleus
001 002 003 004 005 006 007 008 009 010 011 012 013 014 015 016 017 018 019 020 021 022 023 024 025 026 027 028 029 030 031 032 033 034
Exonic Part Number

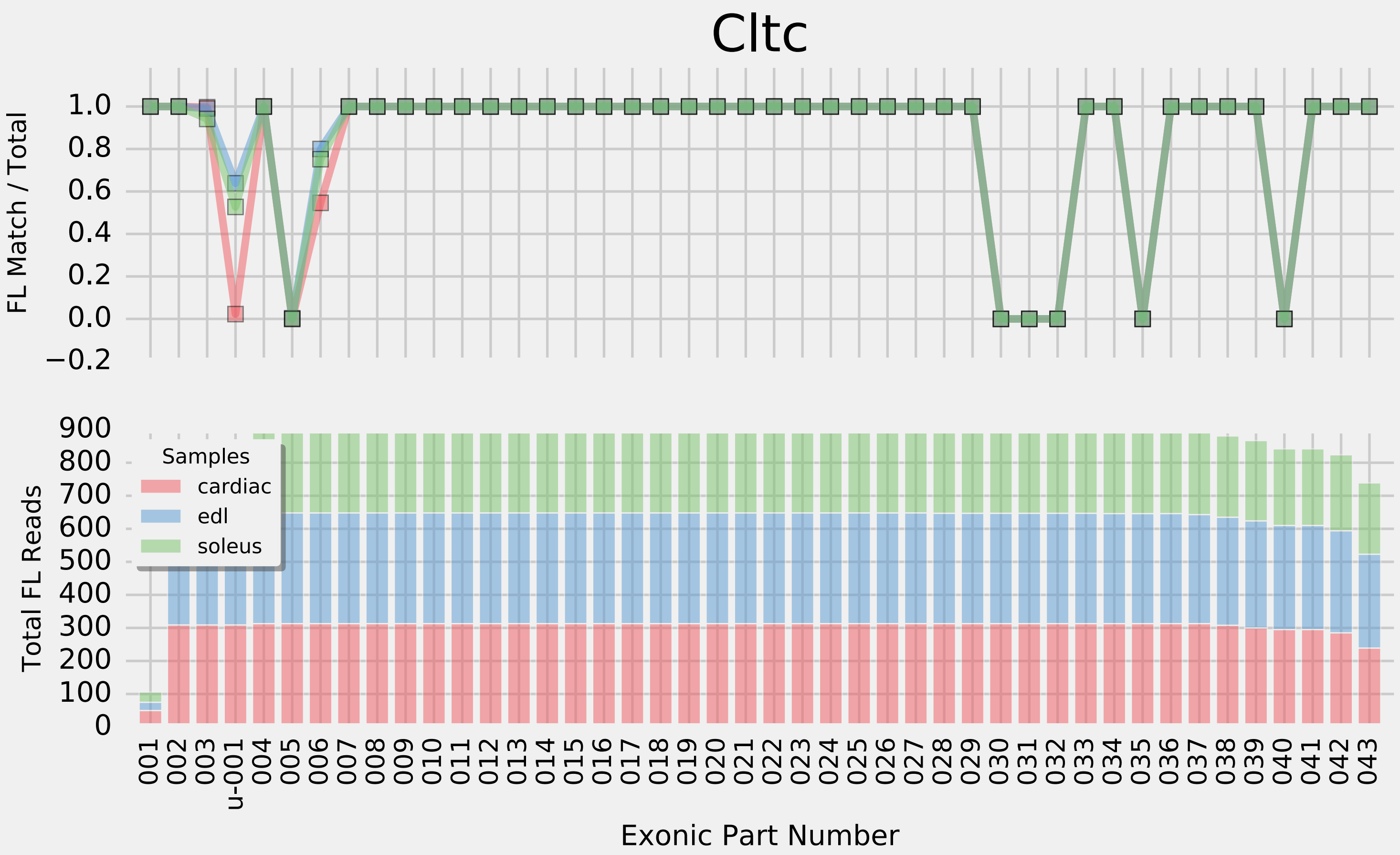

Cltc
FL Match / Total
1.0
0.8
0.6
0.4
0.2
0.0
−0.2
900
800
700
600
500
400
300
200
100
0
Total FL Reads
Samples
cardiac
edl
soleus
001
002
003
u-001
004
005
006
007
008
009
010
011
012
013
014
015
016
017
018
019
020
021
022
023
024
025
026
027
028
029
030
031
032
033
034
035
036
037
038
039
040
041
042
043
Exonic Part Number

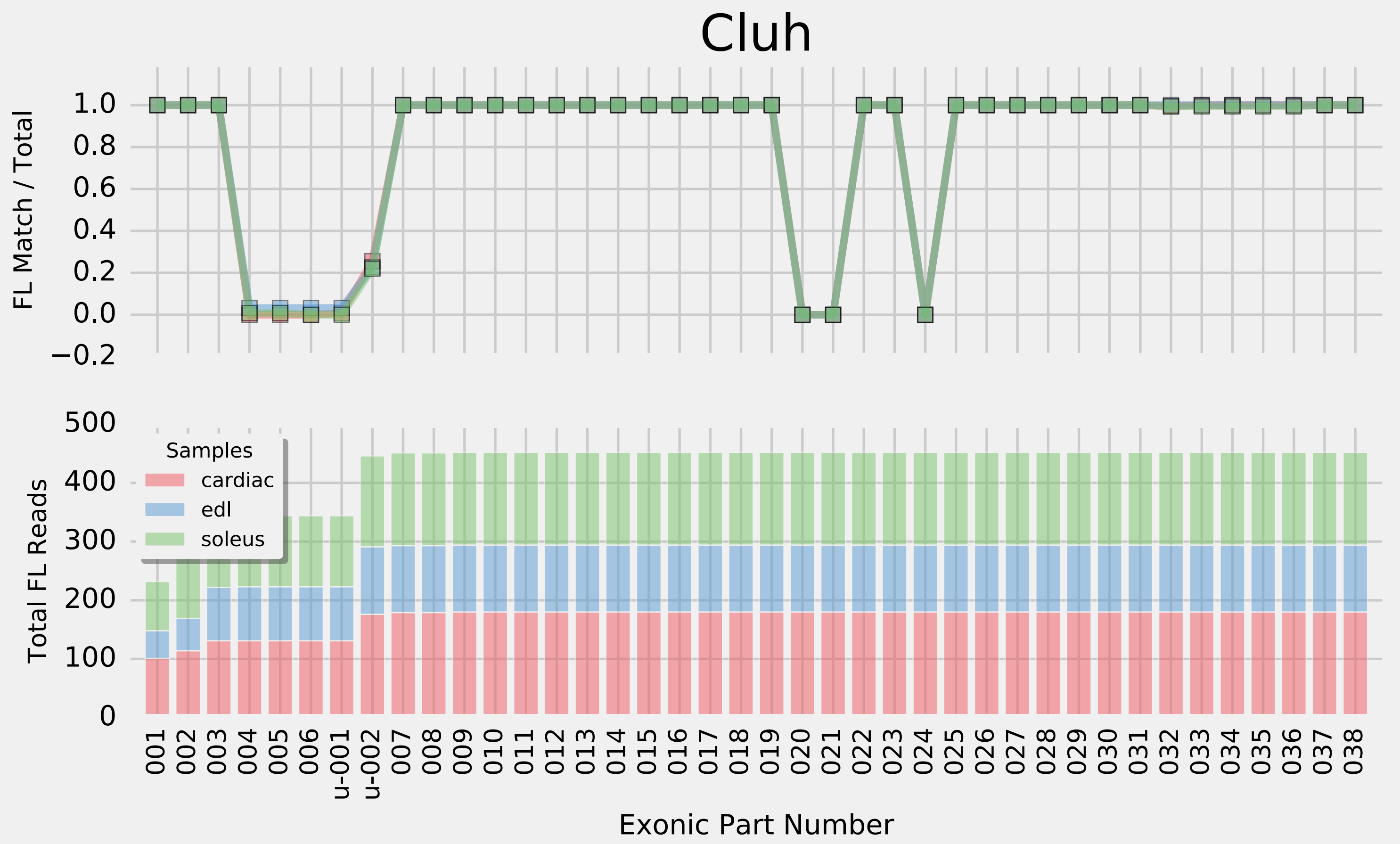

Cluh
FL Match / Total
1.0
0.8
0.6
0.4
0.2
0.0
−0.2
Total FL Reads
500
400
300
200
100
0
Samples
cardiac
edl
soleus
001
002
003
004
005
006
u-001
u-002
007
008
009
010
011
012
013
014
015
016
017
018
019
020
021
022
023
024
025
026
027
028
029
030
031
032
033
034
035
036
037
038
Exonic Part Number

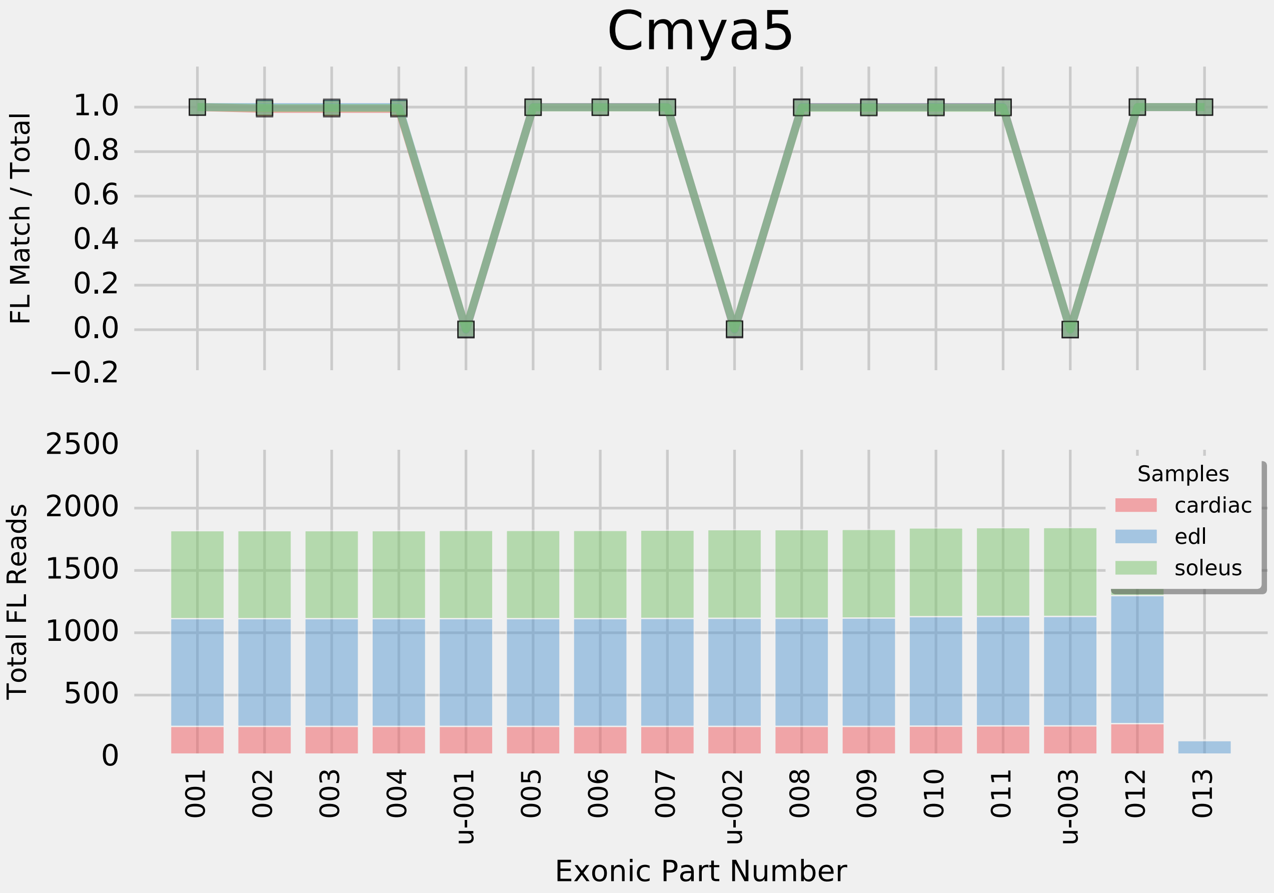

Cmya5
FL Match / Total
1.0
0.8
0.6
0.4
0.2
0.0
−0.2
Total FL Reads
2500
2000
1500
1000
500
0
Samples
cardiac
edl
soleus
001
002
003
004
u-001
005
006
007
u-002
008
009
010
011
u-003
012
013
Exonic Part Number

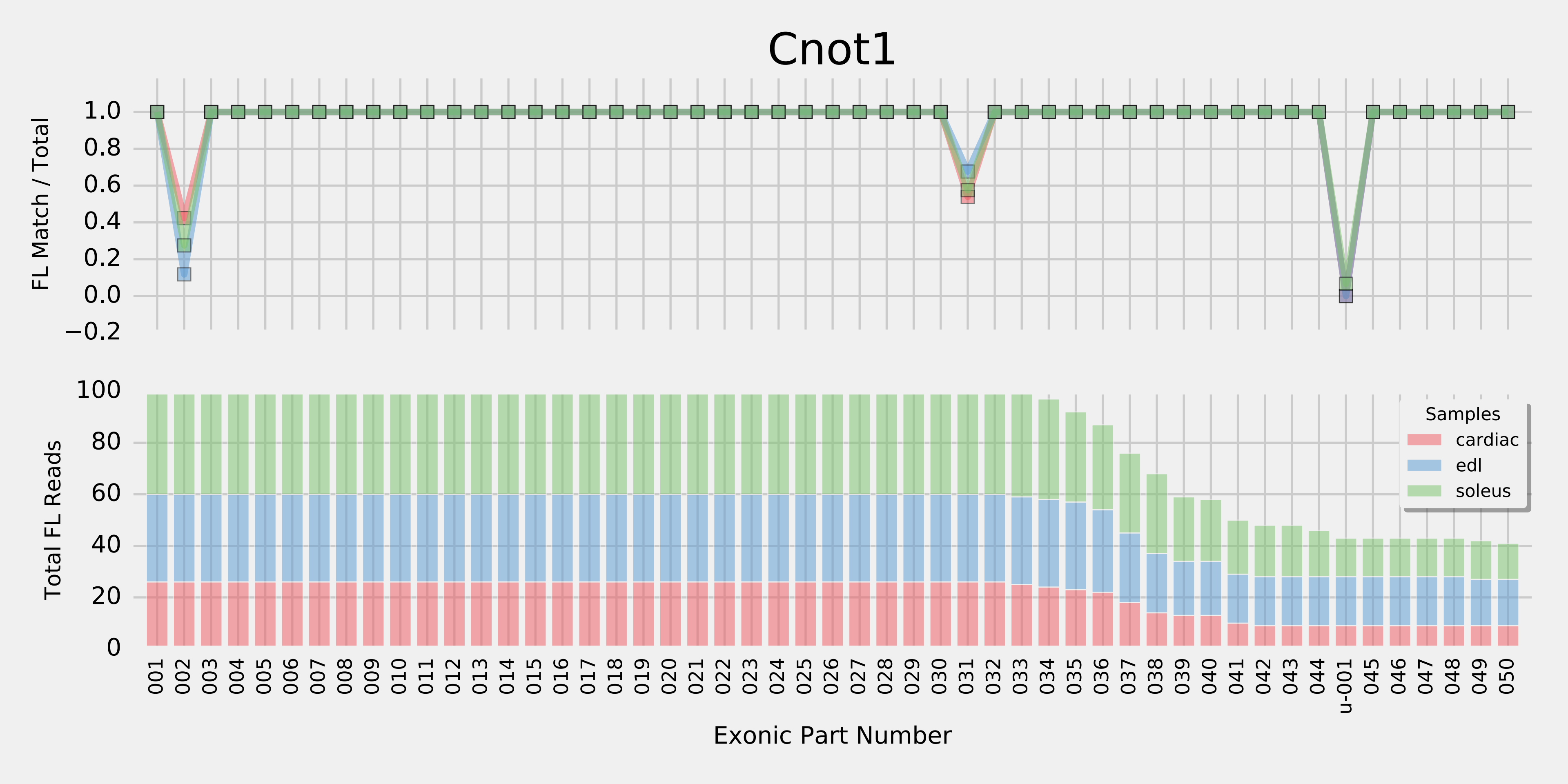

Cnot1
FL Match / Total
1.0
0.8
0.6
0.4
0.2
0.0
−0.2
Total FL Reads
100
80
60
40
20
0
Samples
cardiac
edl
soleus
001
002
003
004
005
006
007
008
009
010
011
012
013
014
015
016
017
018
019
020
021
022
023
024
025
026
027
028
029
030
031
032
033
034
035
036
037
038
039
040
041
042
043
044
u-001
045
046
047
048
049
050
Exonic Part Number

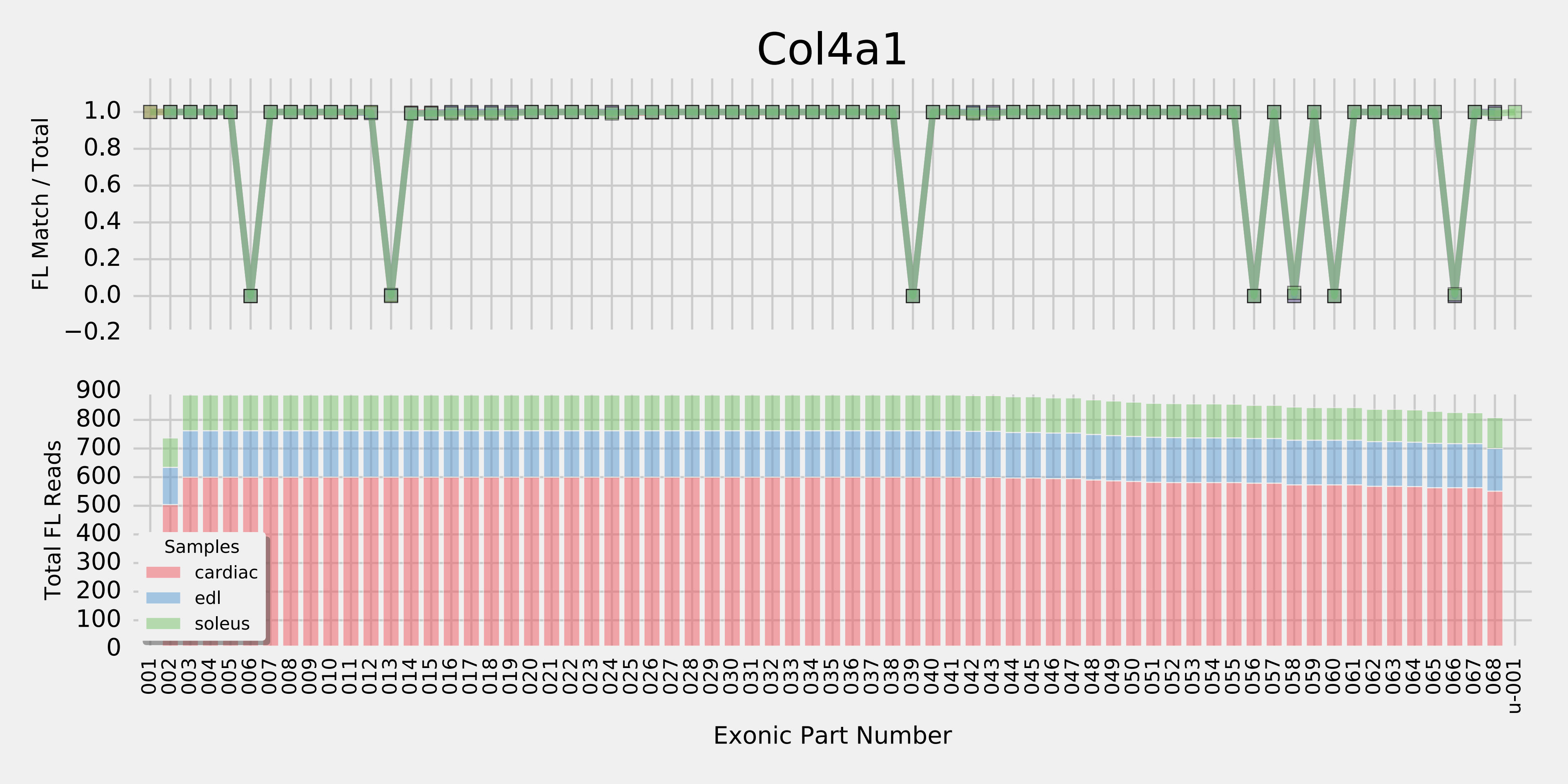

Col4a1
FL Match / Total
1.0
0.8
0.6
0.4
0.2
0.0
−0.2
Total FL Reads
900
800
700
600
500
400
300
200
100
0
Samples
cardiac
edl
soleus
001 002 003 004 005 006 007 008 009 010 011 012 013 014 015 016 017 018 019 020 021 022 023 024 025 026 027 028 029 030 031 032 033 034 035 036 037 038 039 040 041 042 043 044 045 046 047 048 049 050 051 052 053 054 055 056 057 058 059 060 061 062 063 064 065 066 067 068 u-001
Exonic Part Number

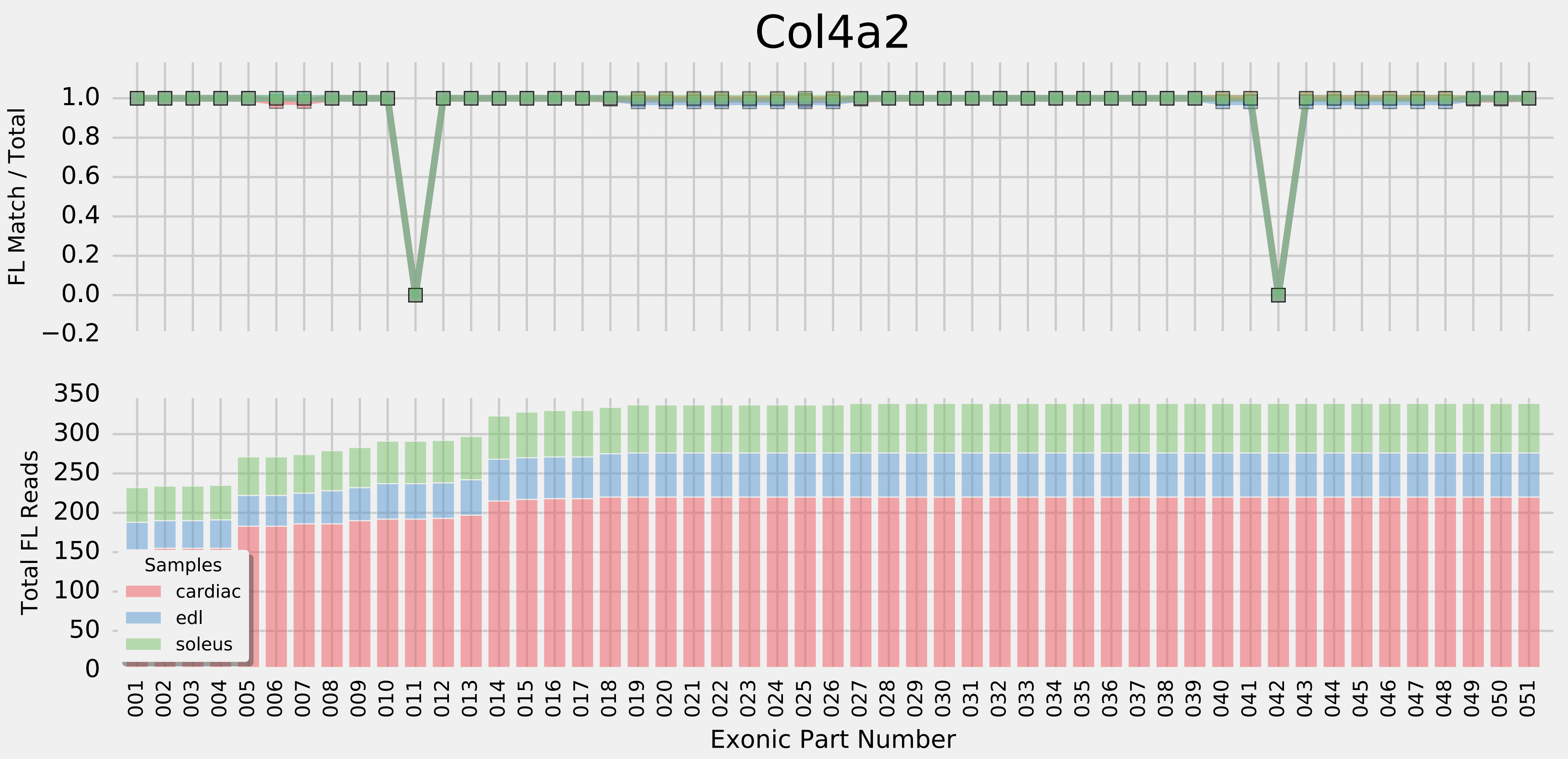

Col4a2
FL Match / Total
1.0
0.8
0.6
0.4
0.2
0.0
−0.2
Total FL Reads
350
300
250
200
150
100
50
0
Samples
cardiac
edl
soleus
001 002 003 004 005 006 007 008 009 010 011 012 013 014 015 016 017 018 019 020 021 022 023 024 025 026 027 028 029 030 031 032 033 034 035 036 037 038 039 040 041 042 043 044 045 046 047 048 049 050 051
Exonic Part Number

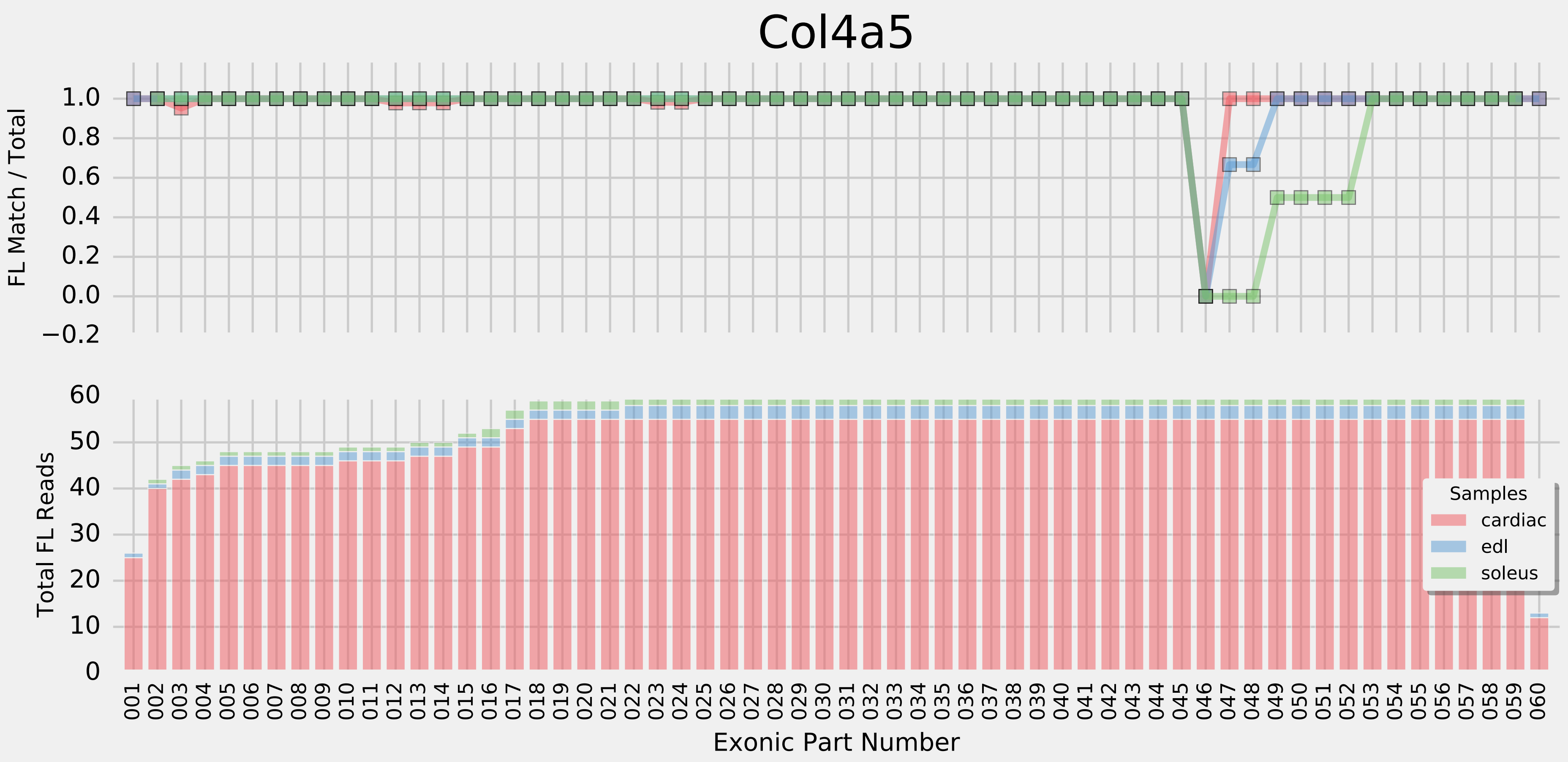
Col4a5
FL Match / Total
Total FL Reads
Exonic Part Number
Samples
cardiac
edl
soleus

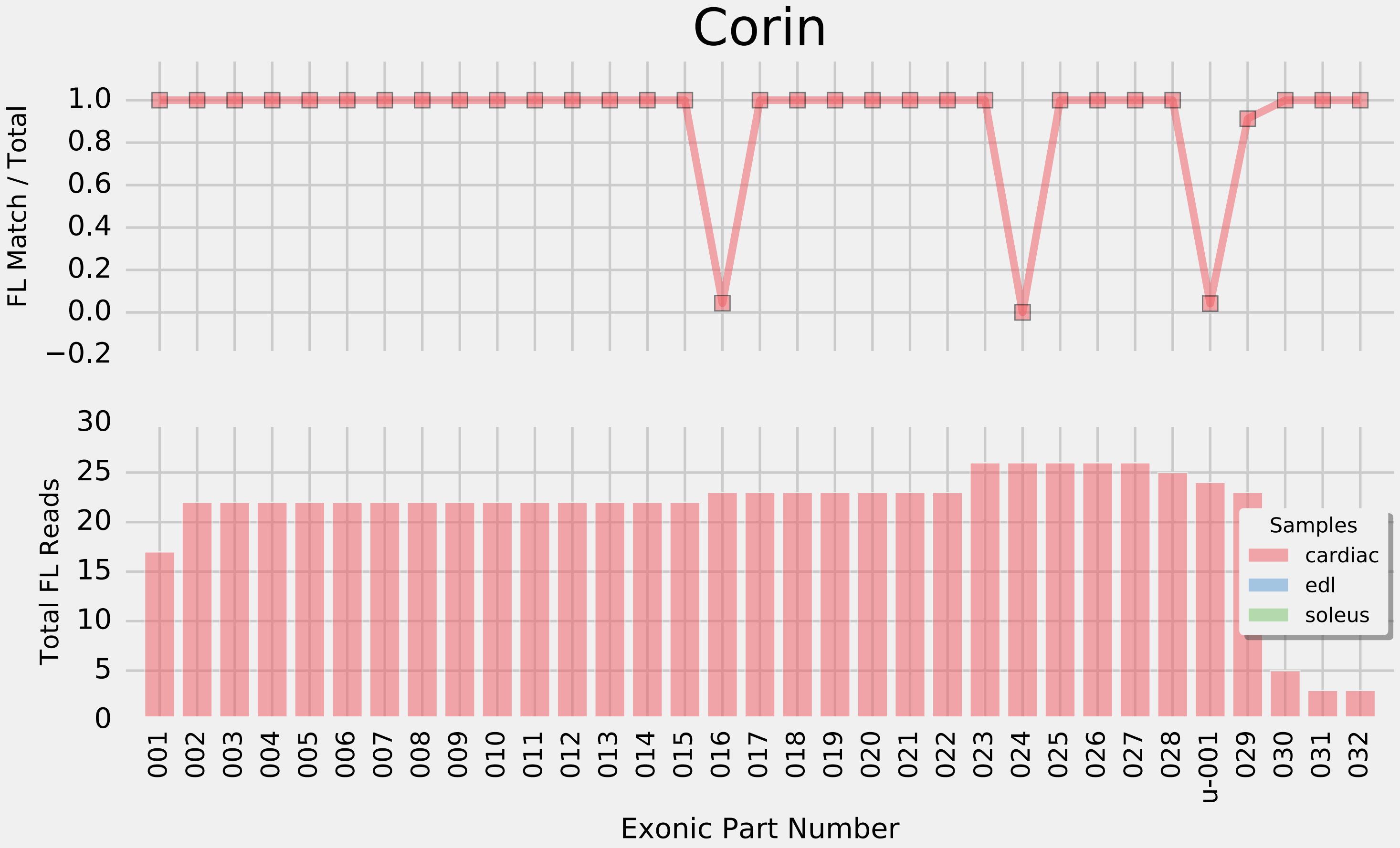

Corin
FL Match / Total
1.0
0.8
0.6
0.4
0.2
0.0
−0.2
Total FL Reads
30
25
20
15
10
5
0
Samples
cardiac
edl
soleus
001
002
003
004
005
006
007
008
009
010
011
012
013
014
015
016
017
018
019
020
021
022
023
024
025
026
027
028
u-001
029
030
031
032
Exonic Part Number

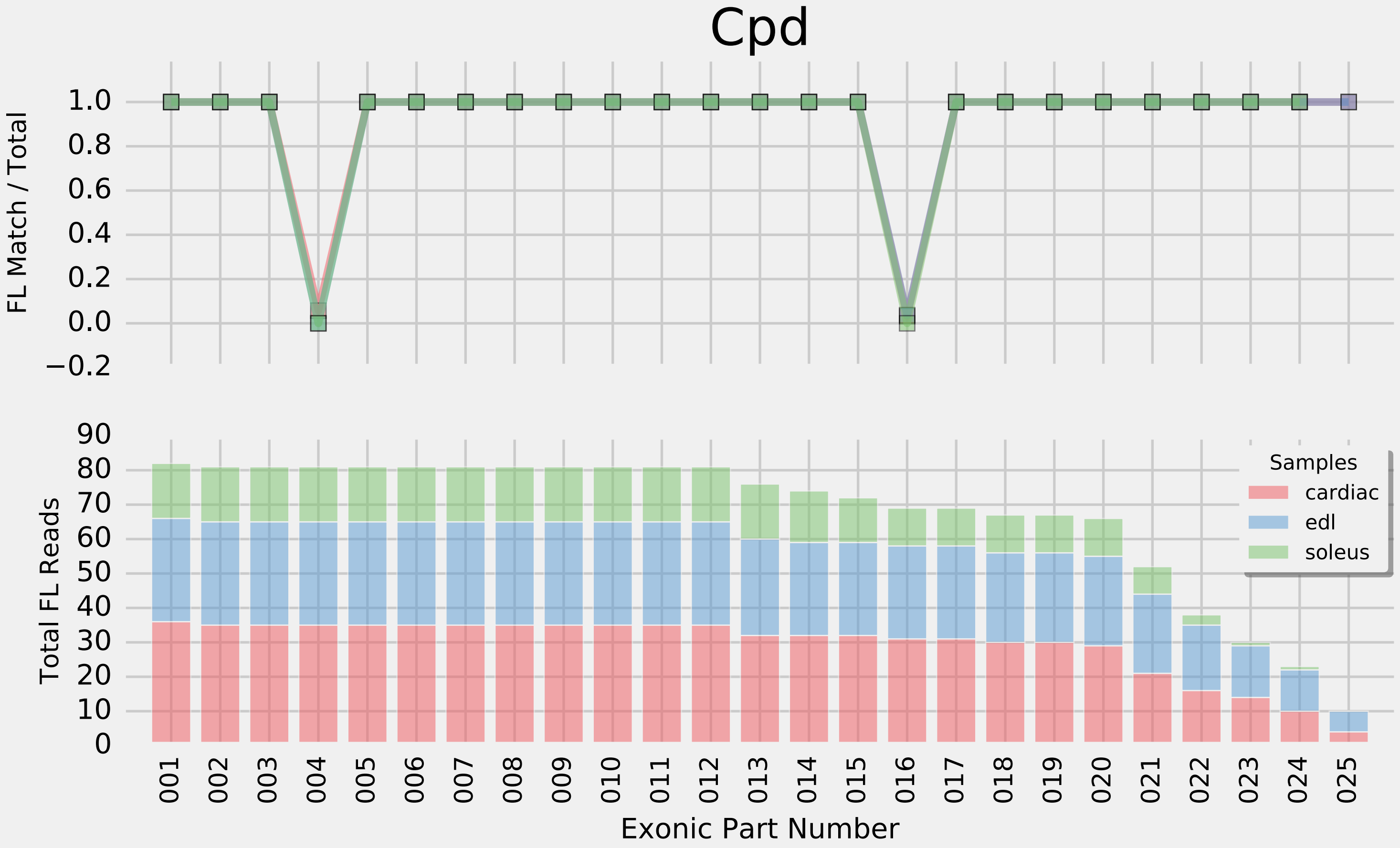

Cpd
FL Match / Total
1.0
0.8
0.6
0.4
0.2
0.0
−0.2
Total FL Reads
90
80
70
60
50
40
30
20
10
0
Samples
cardiac
edl
soleus
001
002
003
004
005
006
007
008
009
010
011
012
013
014
015
016
017
018
019
020
021
022
023
024
025
Exonic Part Number

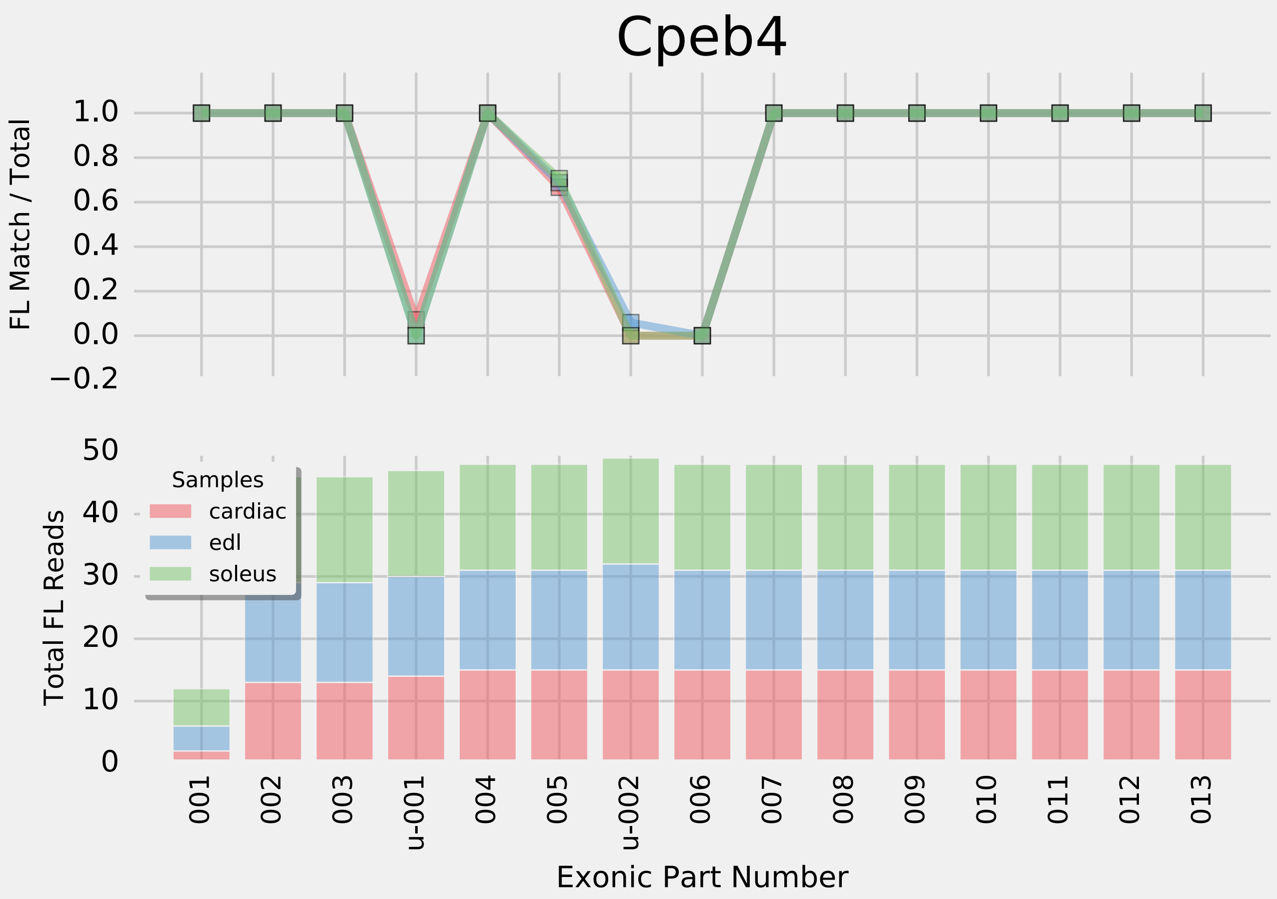
Cpeb4
FL Match / Total
1.0
0.8
0.6
0.4
0.2
0.0
−0.2
Total FL Reads
50
40
30
20
10
0
Samples
cardiac
edl
soleus
001
002
003
u-001
004
005
u-002
006
007
008
009
010
011
012
013
Exonic Part Number

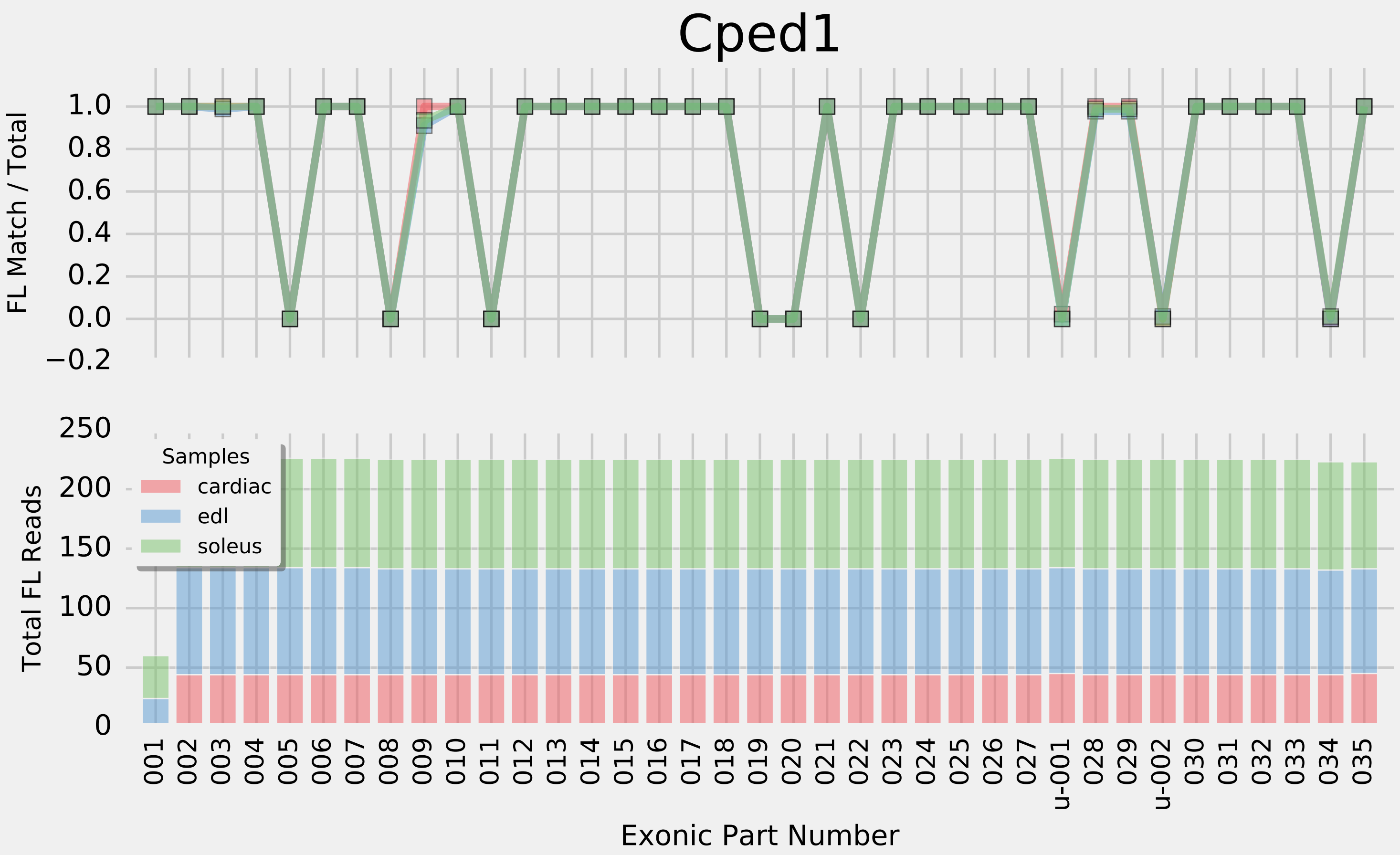
Cped1
FL Match / Total
1.0
0.8
0.6
0.4
0.2
0.0
−0.2
250
200
150
100
50
0
Total FL Reads
Samples
cardiac
edl
soleus
001
002
003
004
005
006
007
008
009
010
011
012
013
014
015
016
017
018
019
020
021
022
023
024
025
026
027
u-001
028
029
u-002
030
031
032
033
034
035
Exonic Part Number

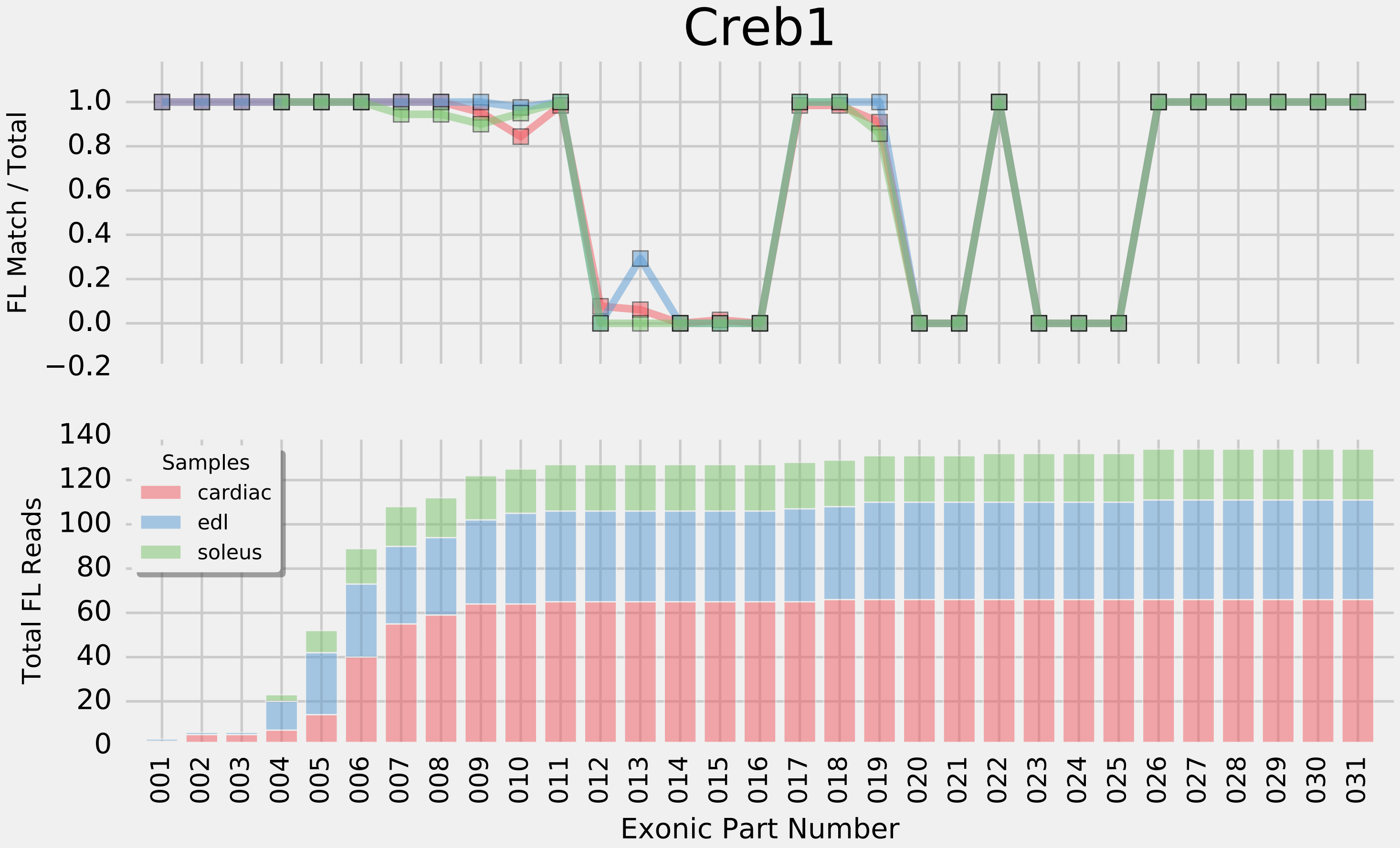
Creb1
FL Match / Total
1.0
0.8
0.6
0.4
0.2
0.0
−0.2
Total FL Reads
140
120
100
80
60
40
20
0
Samples
cardiac
edl
soleus
001
002
003
004
005
006
007
008
009
010
011
012
013
014
015
016
017
018
019
020
021
022
023
024
025
026
027
028
029
030
031
Exonic Part Number

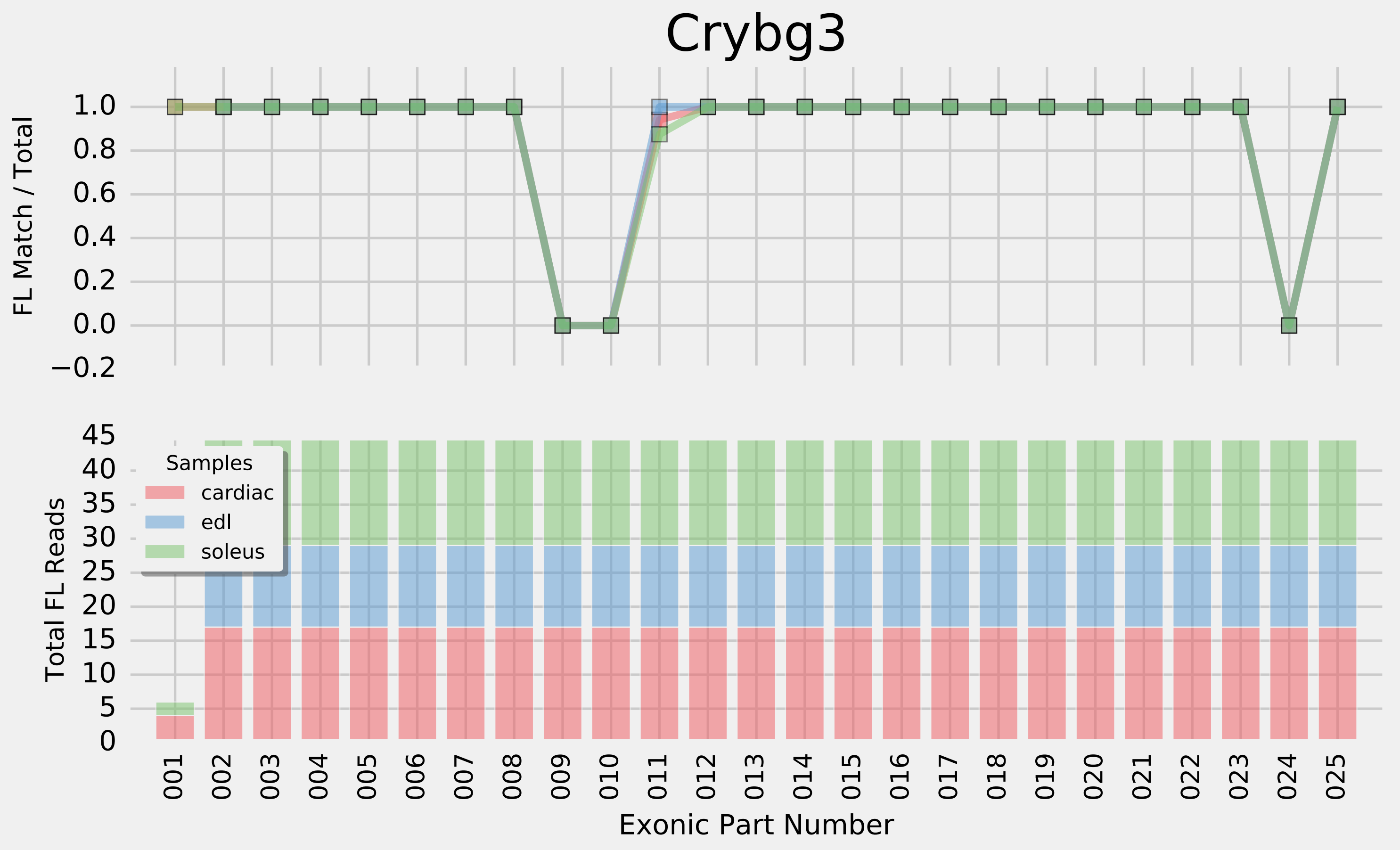
Crybg3
FL Match / Total
1.0
0.8
0.6
0.4
0.2
0.0
−0.2
Total FL Reads
45
40
35
30
25
20
15
10
5
0
Samples
cardiac
edl
soleus
001
002
003
004
005
006
007
008
009
010
011
012
013
014
015
016
017
018
019
020
021
022
023
024
025
Exonic Part Number

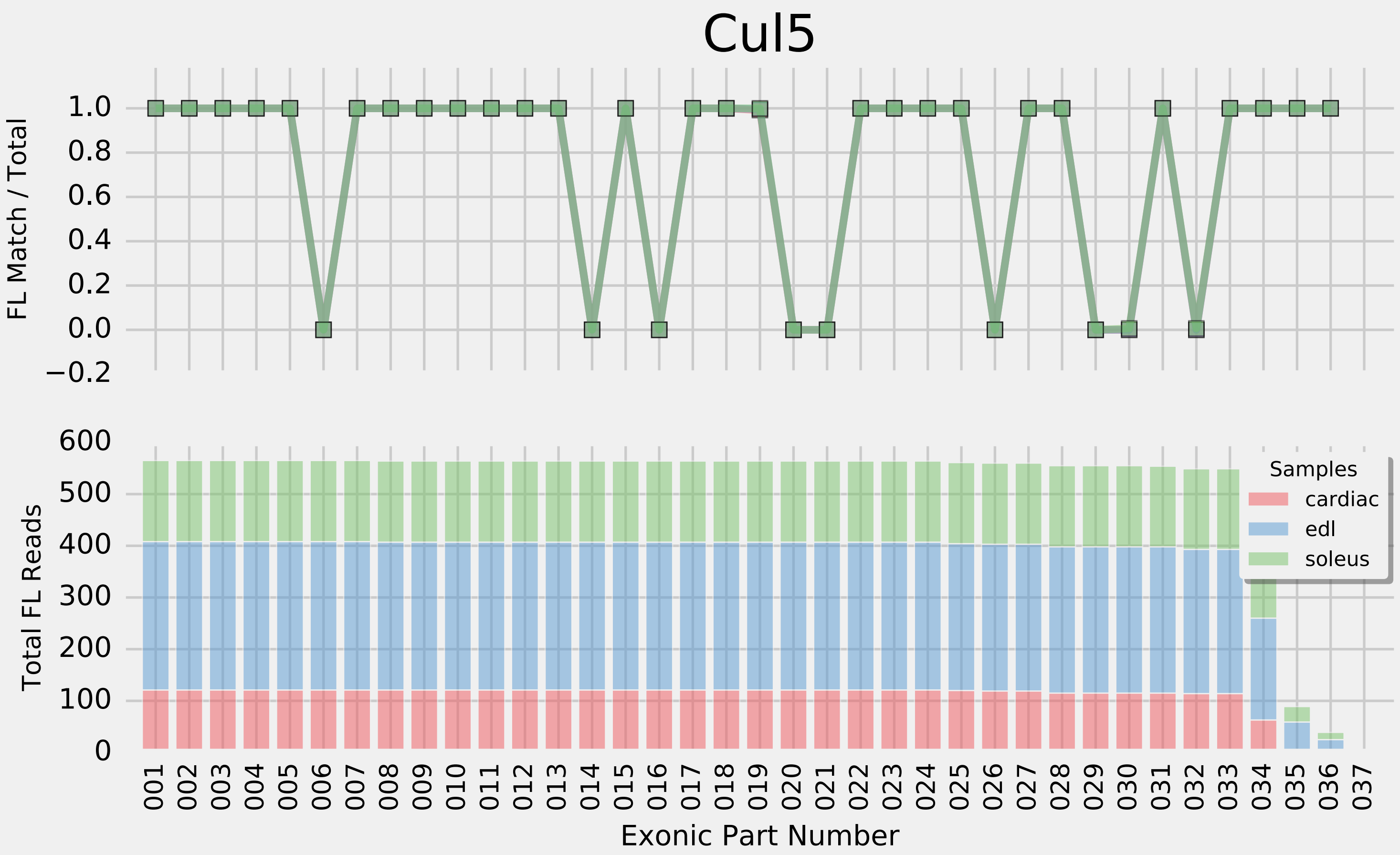

Cul5
FL Match / Total
1.0
0.8
0.6
0.4
0.2
0.0
−0.2
Total FL Reads
600
500
400
300
200
100
0
Samples
cardiac
edl
soleus
001 002 003 004 005 006 007 008 009 010 011 012 013 014 015 016 017 018 019 020 021 022 023 024 025 026 027 028 029 030 031 032 033 034 035 036 037
Exonic Part Number

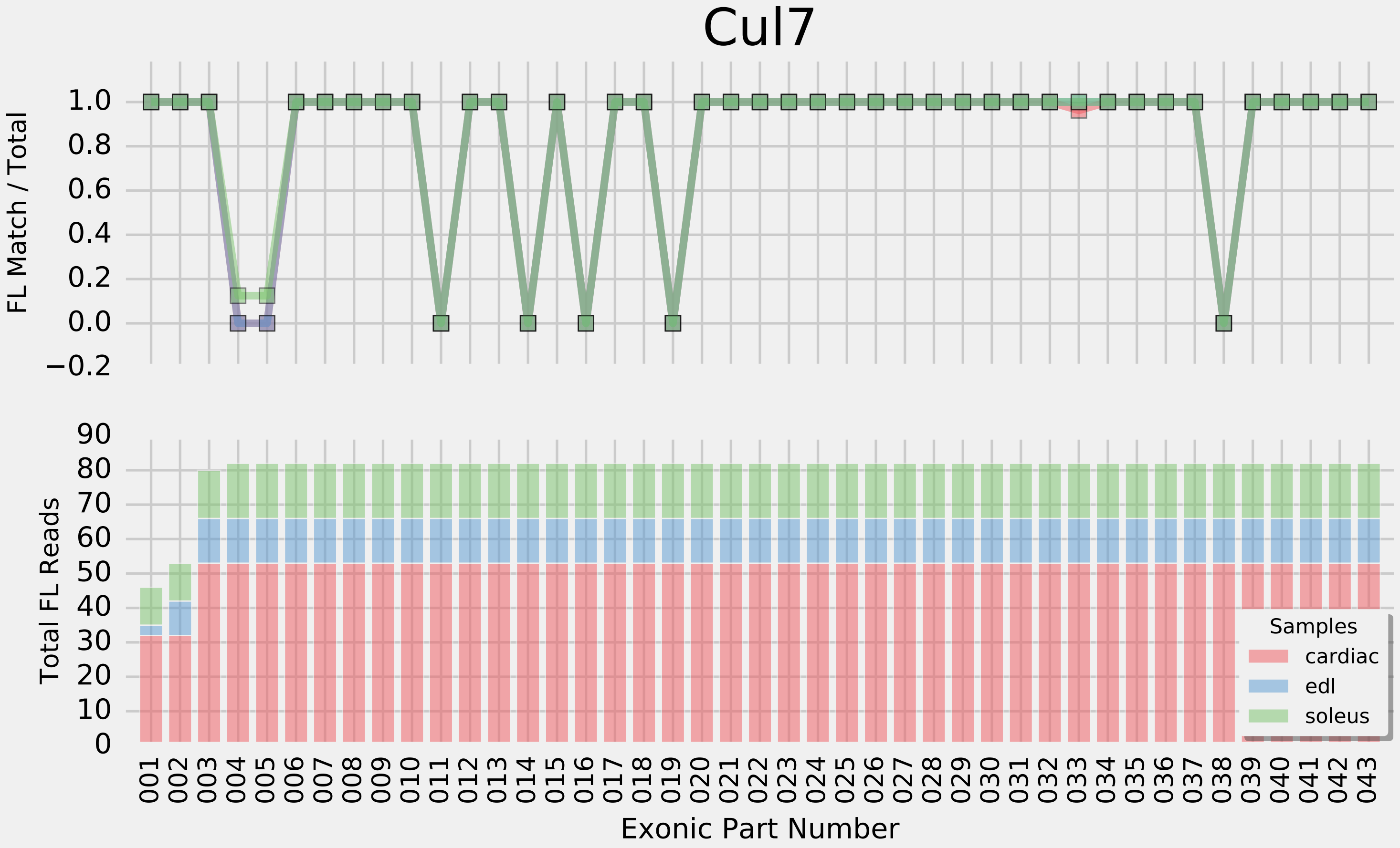

Cul7
FL Match / Total
1.0
0.8
0.6
0.4
0.2
0.0
−0.2
Total FL Reads
90
80
70
60
50
40
30
20
10
0
Samples
cardiac
edl
soleus
001 002 003 004 005 006 007 008 009 010 011 012 013 014 015 016 017 018 019 020 021 022 023 024 025 026 027 028 029 030 031 032 033 034 035 036 037 038 039 040 041 042 043
Exonic Part Number

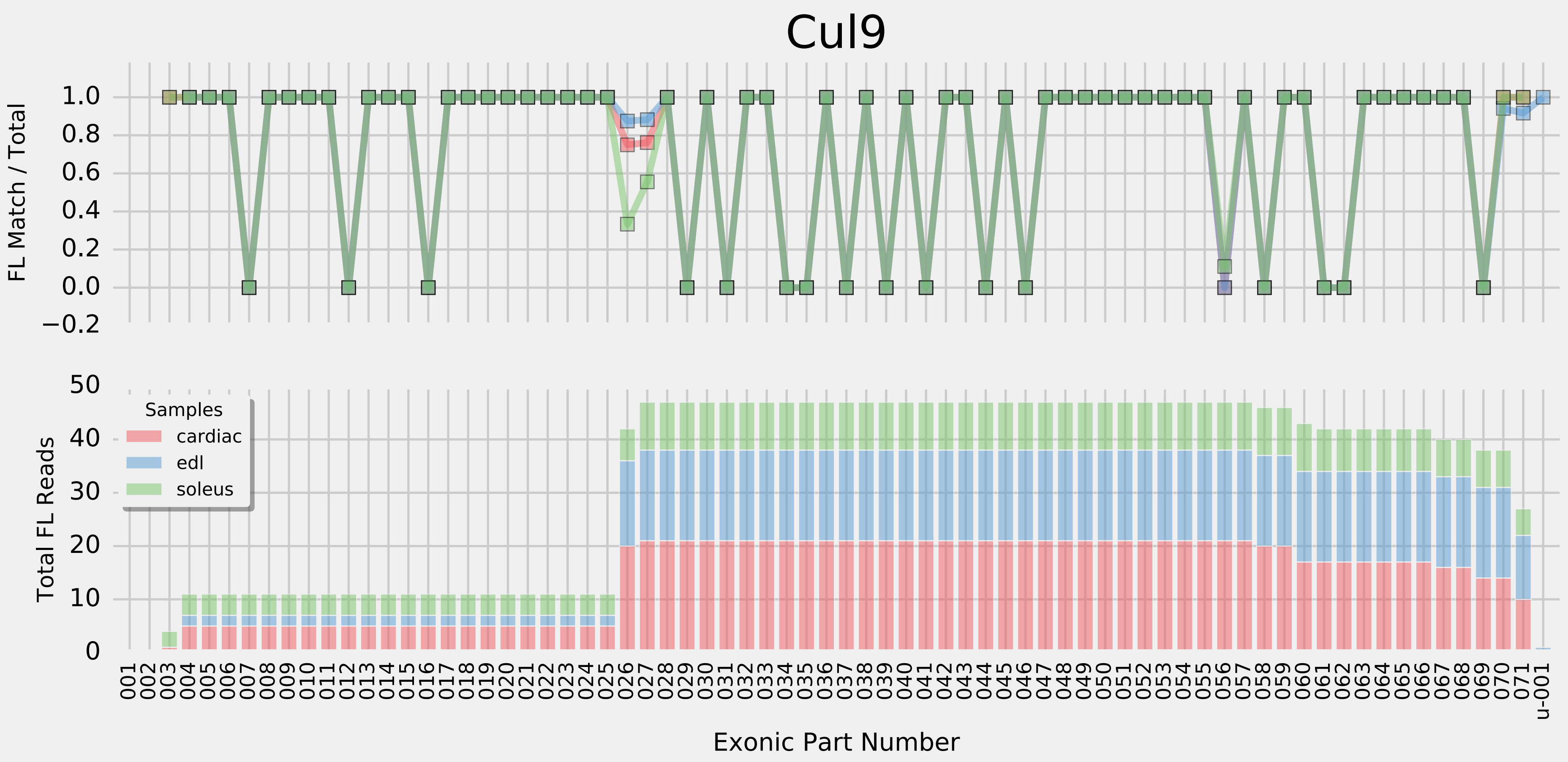

Cul9
FL Match / Total
Total FL Reads
Samples
cardiac
edl
soleus
Exonic Part Number

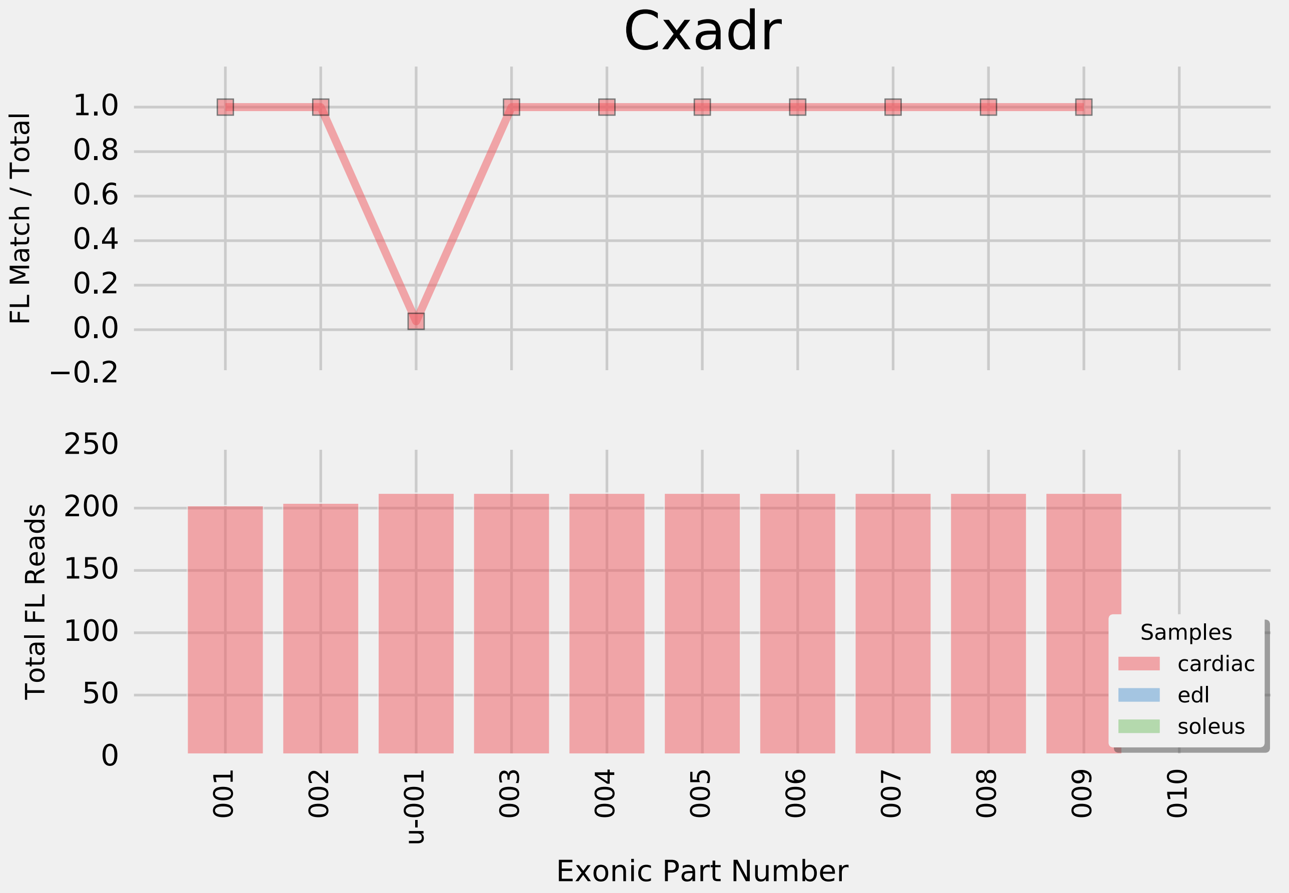
Cxadr
FL Match / Total
1.0
0.8
0.6
0.4
0.2
0.0
−0.2
250
200
150
100
50
0
Total FL Reads
Samples
cardiac
edl
soleus
001
002
u-001
003
004
005
006
007
008
009
010
Exonic Part Number

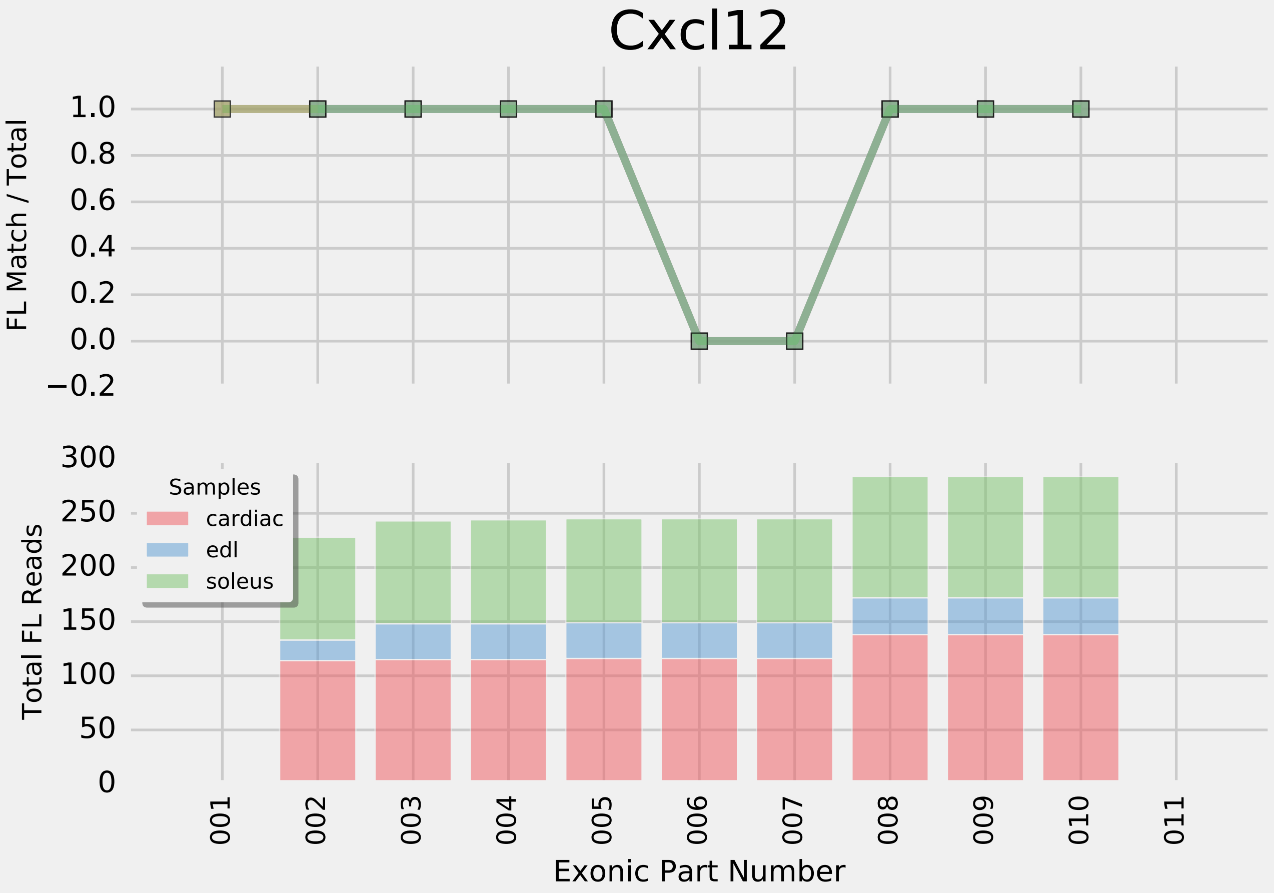

Cxcl12
FL Match / Total
1.0
0.8
0.6
0.4
0.2
0.0
−0.2
Total FL Reads
300
250
200
150
100
50
0
Samples
cardiac
edl
soleus
001
002
003
004
005
006
007
008
009
010
011
Exonic Part Number

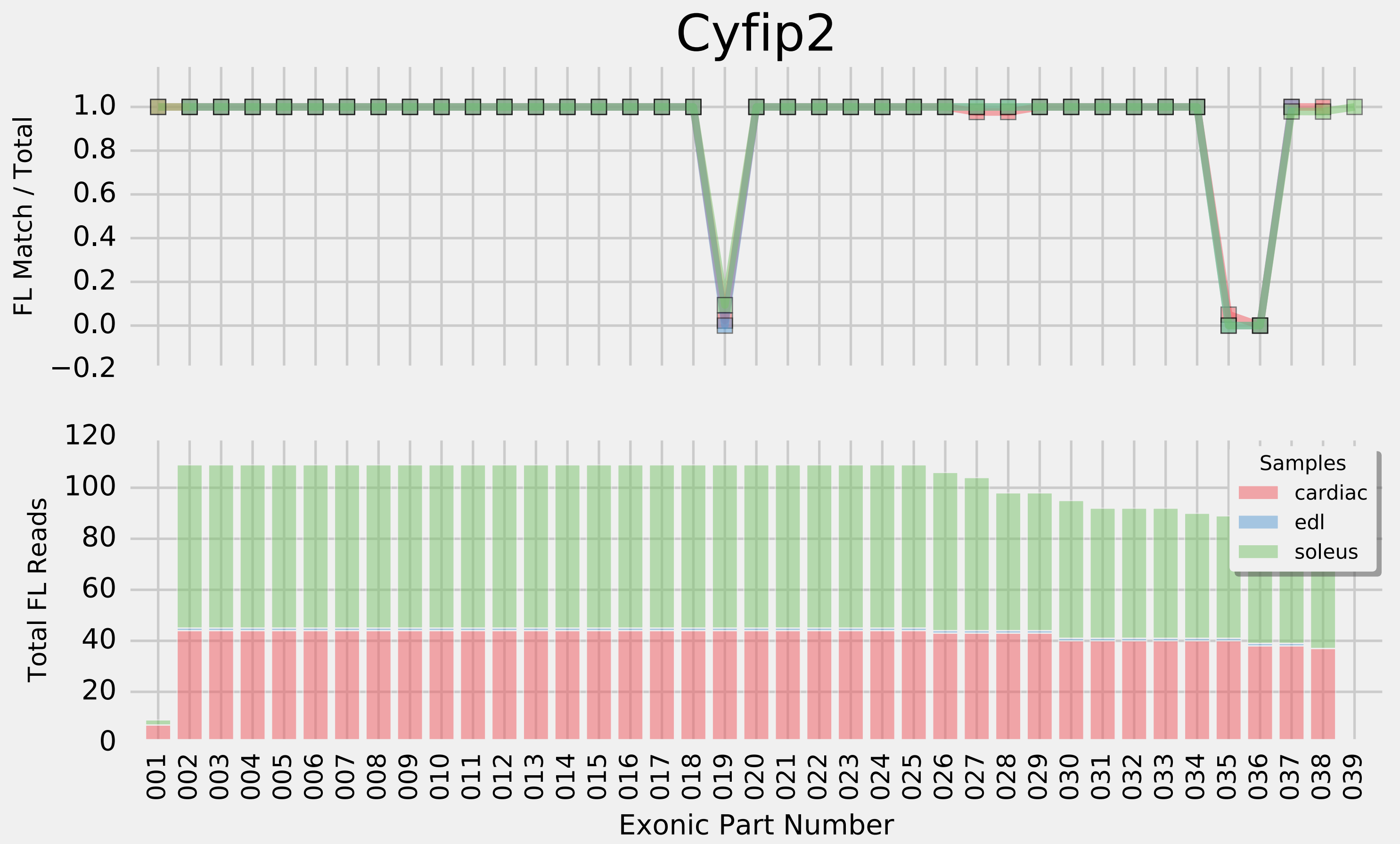
Cyfip2
FL Match / Total
1.0
0.8
0.6
0.4
0.2
0.0
−0.2
Total FL Reads
120
100
80
60
40
20
0
Samples
cardiac
edl
soleus
001 002 003 004 005 006 007 008 009 010 011 012 013 014 015 016 017 018 019 020 021 022 023 024 025 026 027 028 029 030 031 032 033 034 035 036 037 038 039
Exonic Part Number

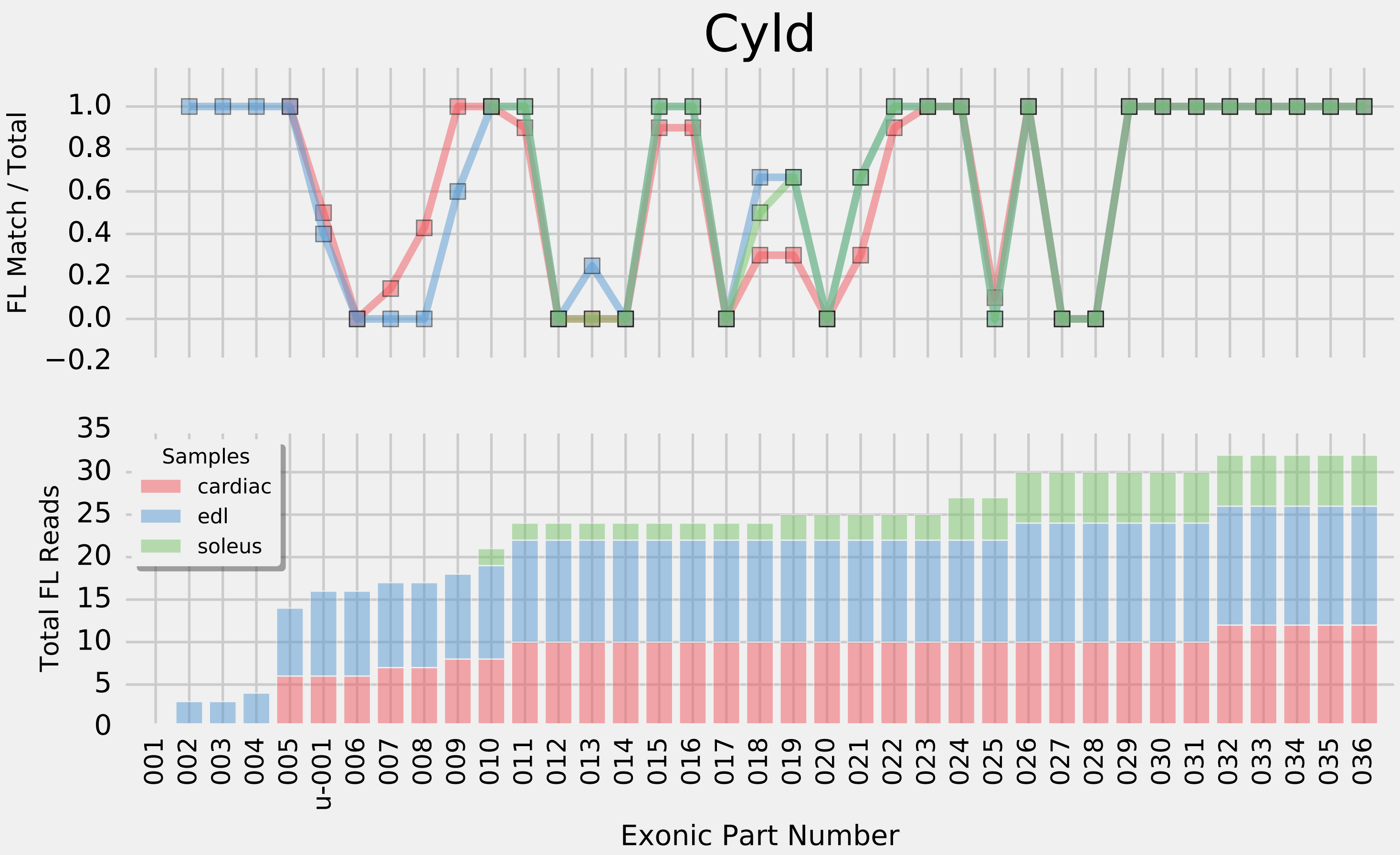
Cyld
FL Match / Total
1.0
0.8
0.6
0.4
0.2
0.0
−0.2
Total FL Reads
35
30
25
20
15
10
5
0
Samples
cardiac
edl
soleus
001
002
003
004
005
u-001
006
007
008
009
010
011
012
013
014
015
016
017
018
019
020
021
022
023
024
025
026
027
028
029
030
031
032
033
034
035
036
Exonic Part Number

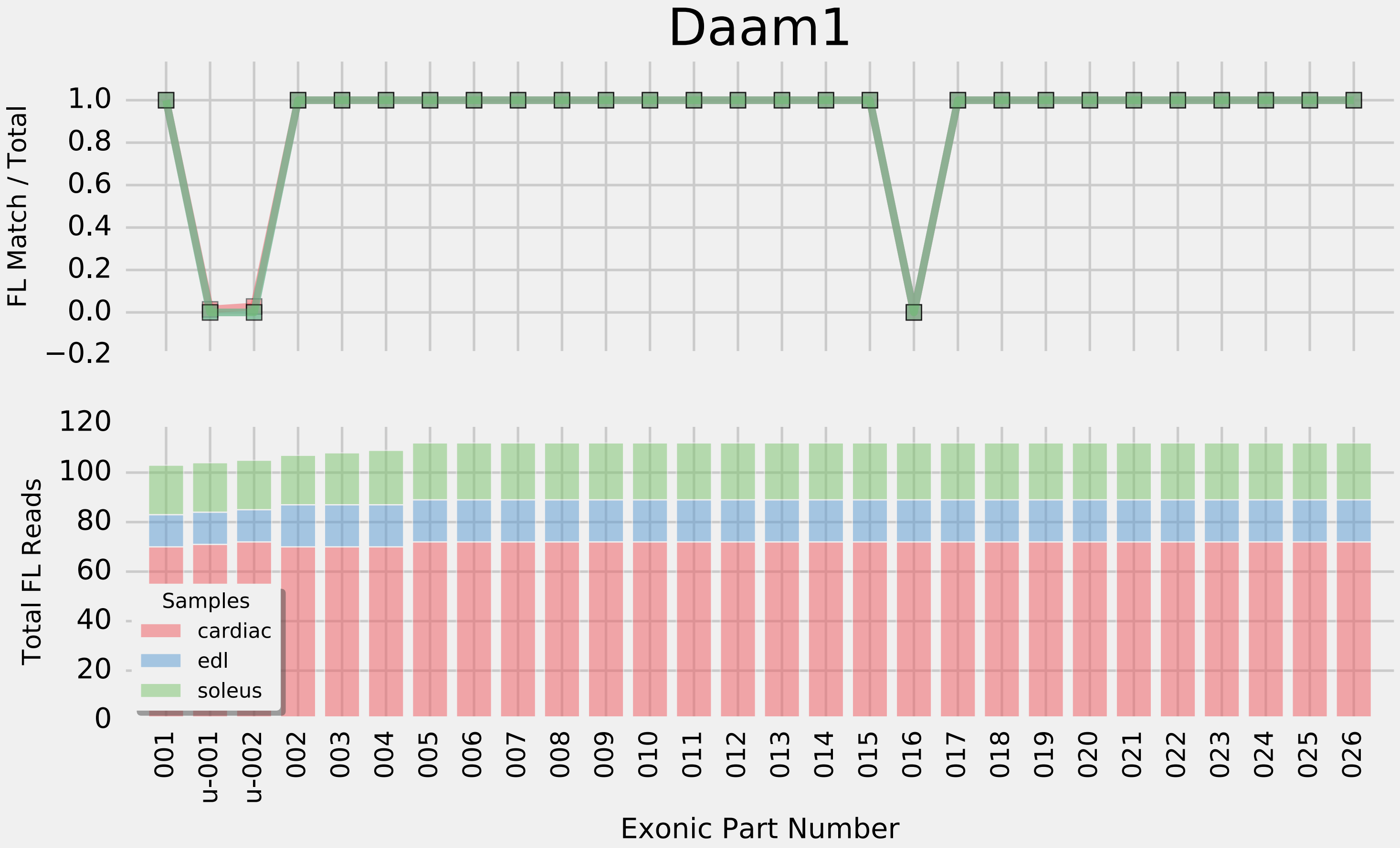

Daam1
FL Match / Total
1.0
0.8
0.6
0.4
0.2
0.0
−0.2
Total FL Reads
120
100
80
60
40
20
0
Samples
cardiac
edl
soleus
001
u-001
u-002
002
003
004
005
006
007
008
009
010
011
012
013
014
015
016
017
018
019
020
021
022
023
024
025
026
Exonic Part Number

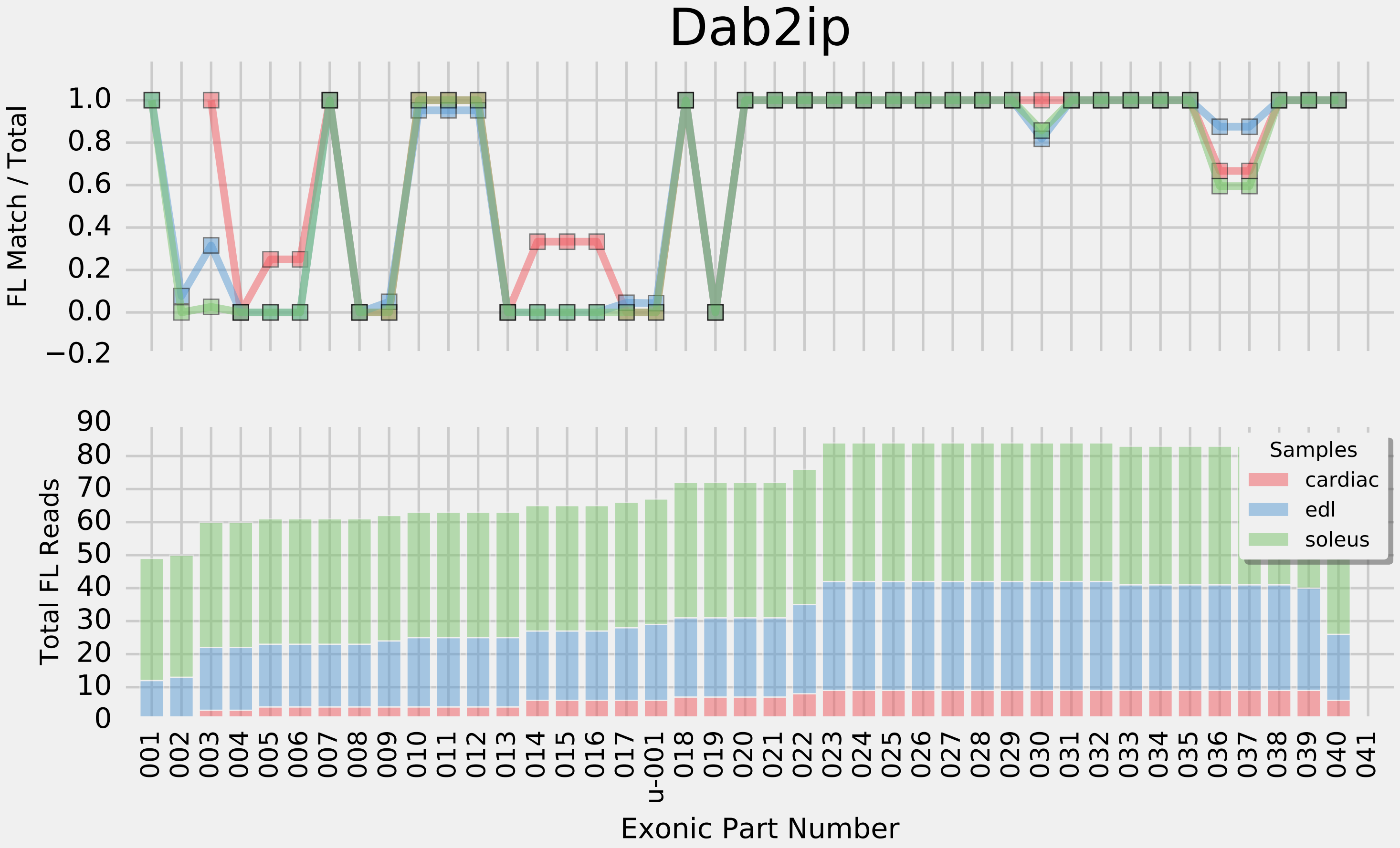

Dab2ip
FL Match / Total
1.0
0.8
0.6
0.4
0.2
0.0
−0.2
Total FL Reads
90
80
70
60
50
40
30
20
10
0
Samples
cardiac
edl
soleus
001 002 003 004 005 006 007 008 009 010 011 012 013 014 015 016 017 u-001 018 019 020 021 022 023 024 025 026 027 028 029 030 031 032 033 034 035 036 037 038 039 040 041
Exonic Part Number

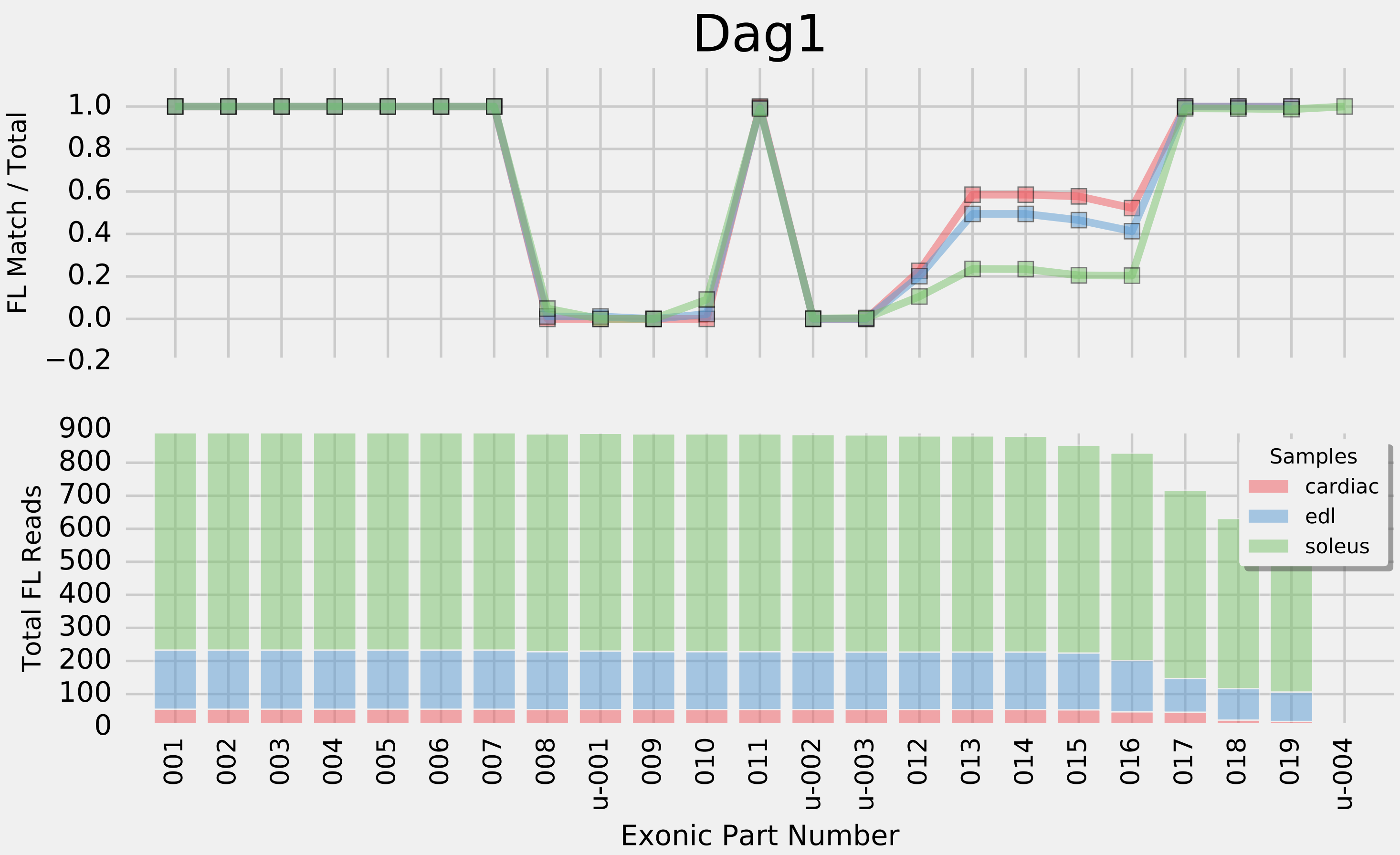

Dag1
FL Match / Total
1.0
0.8
0.6
0.4
0.2
0.0
−0.2
Total FL Reads
900
800
700
600
500
400
300
200
100
0
Samples
cardiac
edl
soleus
001
002
003
004
005
006
007
008
u-001
009
010
011
u-002
u-003
012
013
014
015
016
017
018
019
u-004
Exonic Part Number

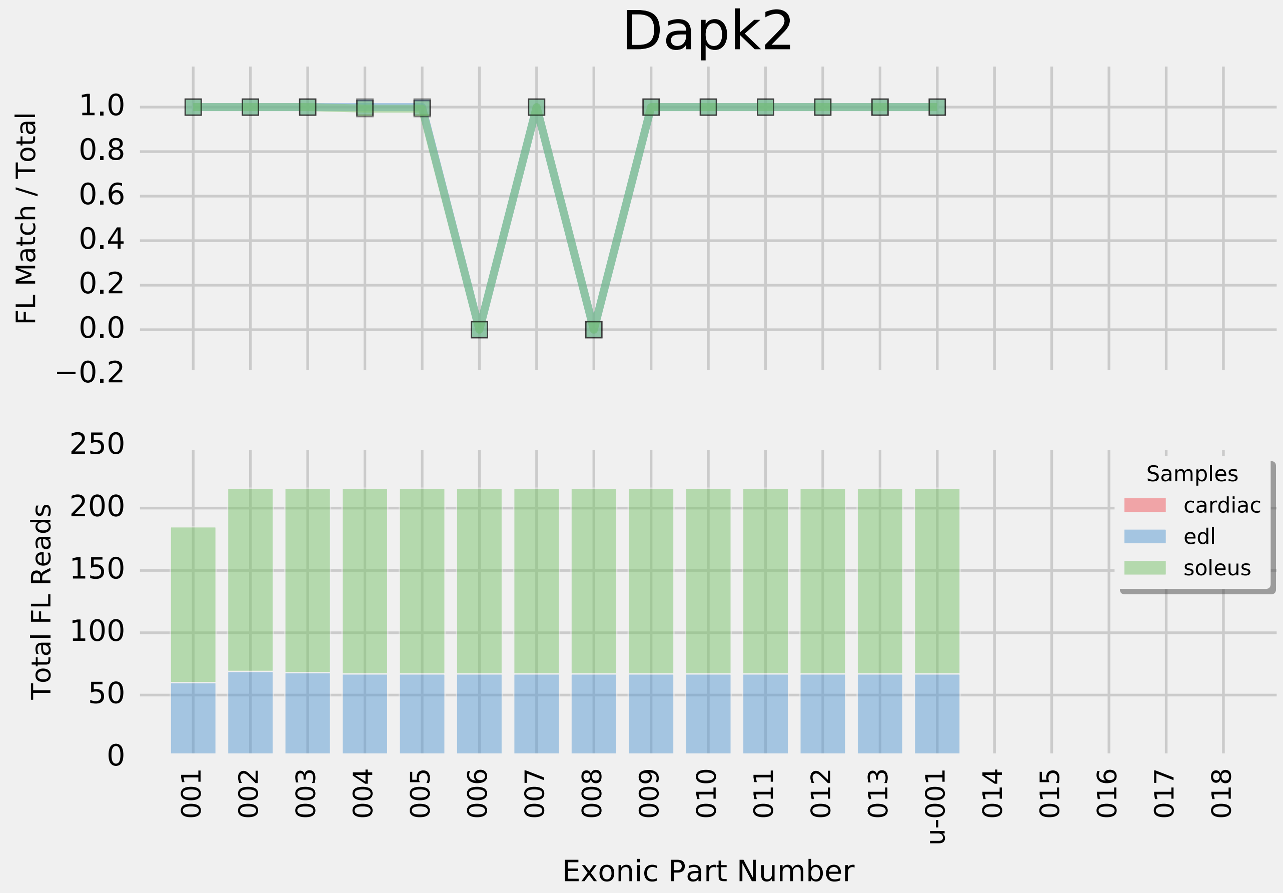
Dapk2
FL Match / Total
1.0
0.8
0.6
0.4
0.2
0.0
−0.2
Total FL Reads
250
200
150
100
50
0
Samples
cardiac
edl
soleus
001
002
003
004
005
006
007
008
009
010
011
012
013
u-001
014
015
016
017
018
Exonic Part Number

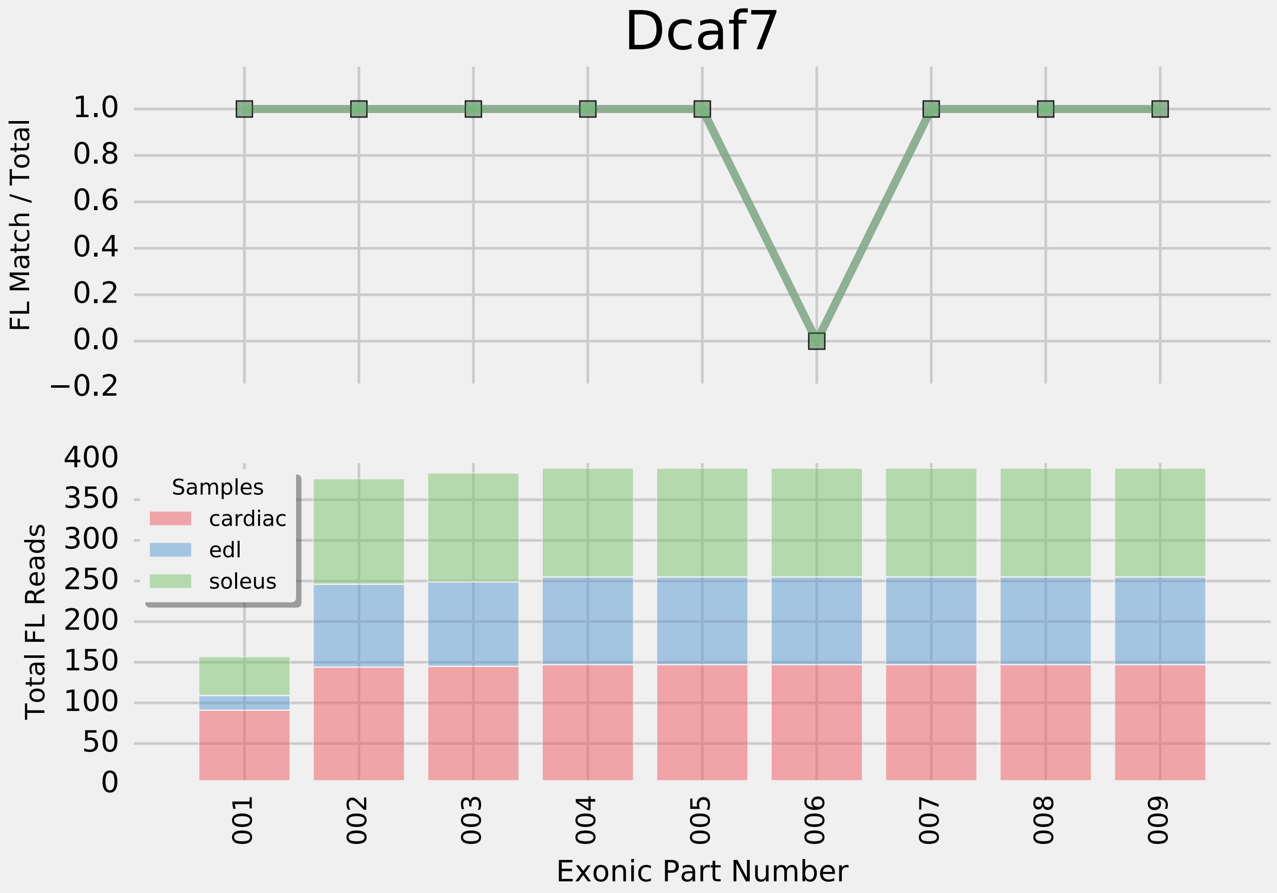

Dcaf7
FL Match / Total
1.0
0.8
0.6
0.4
0.2
0.0
−0.2
400
350
300
250
200
150
100
50
0
Total FL Reads
Samples
cardiac
edl
soleus
001
002
003
004
005
006
007
008
009
Exonic Part Number

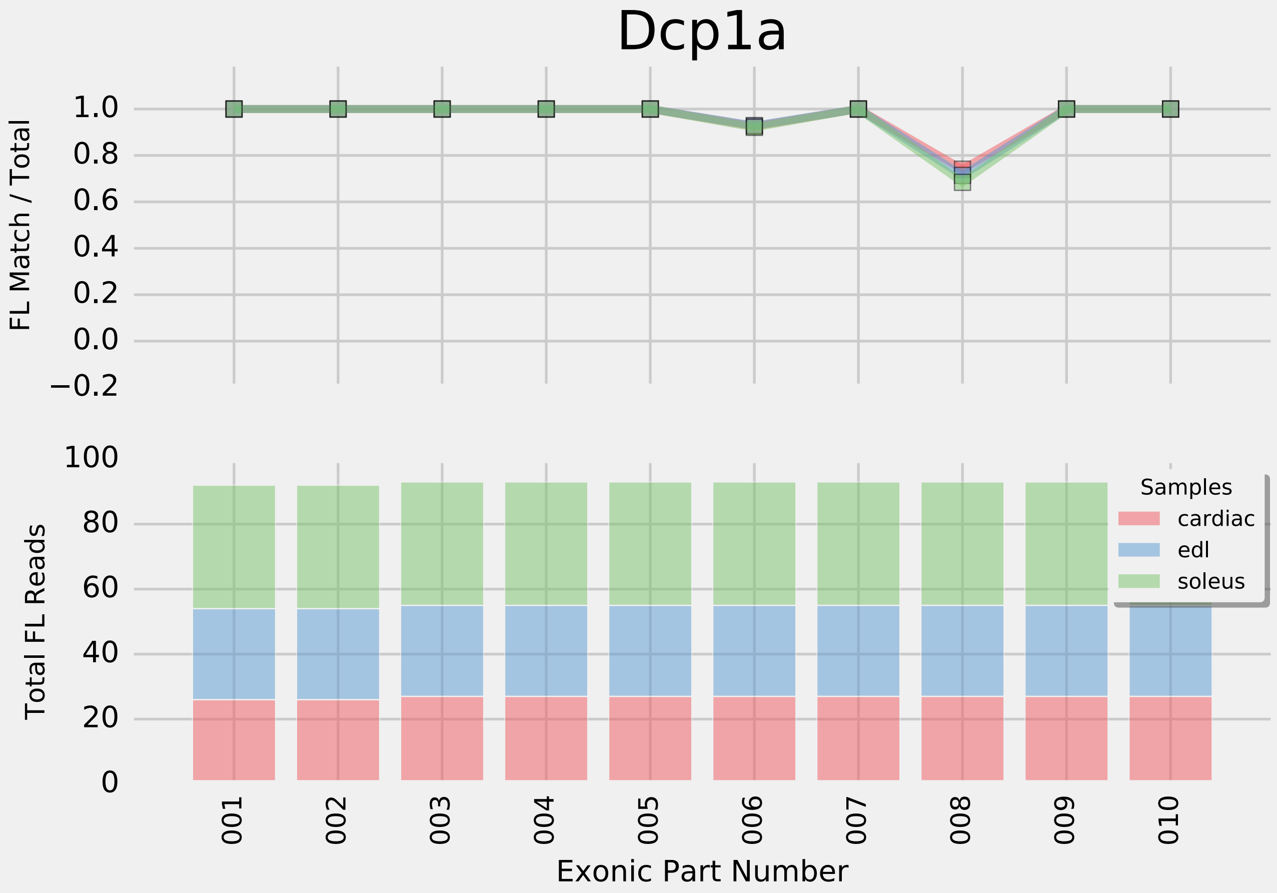

Dcp1a
FL Match / Total
1.0
0.8
0.6
0.4
0.2
0.0
−0.2
Total FL Reads
100
80
60
40
20
0
Samples
cardiac
edl
soleus
001
002
003
004
005
006
007
008
009
010
Exonic Part Number

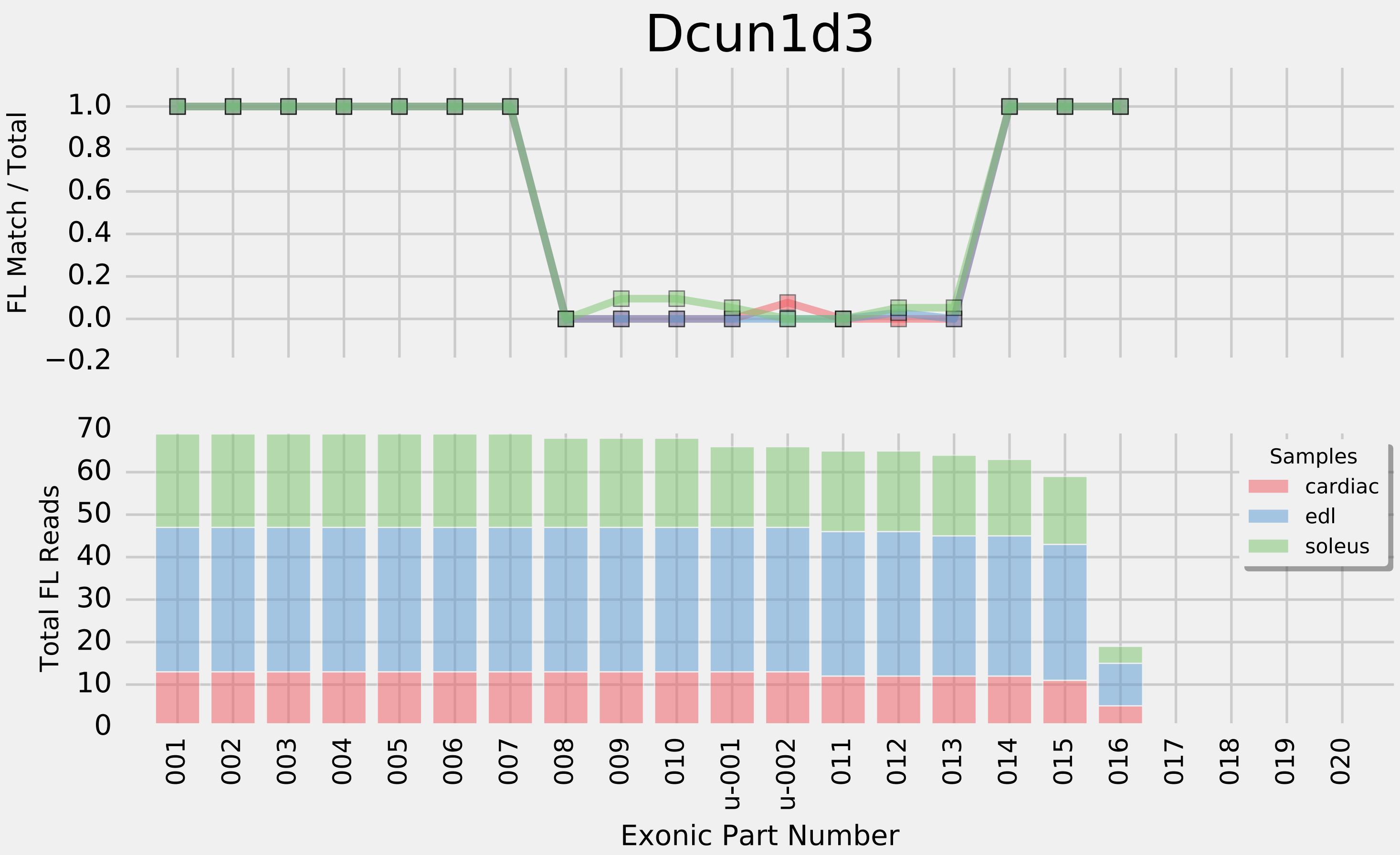

Dcun1d3
FL Match / Total
1.0
0.8
0.6
0.4
0.2
0.0
−0.2
Total FL Reads
70
60
50
40
30
20
10
0
Samples
cardiac
edl
soleus
001
002
003
004
005
006
007
008
009
010
u-001
u-002
011
012
013
014
015
016
017
018
019
020
Exonic Part Number

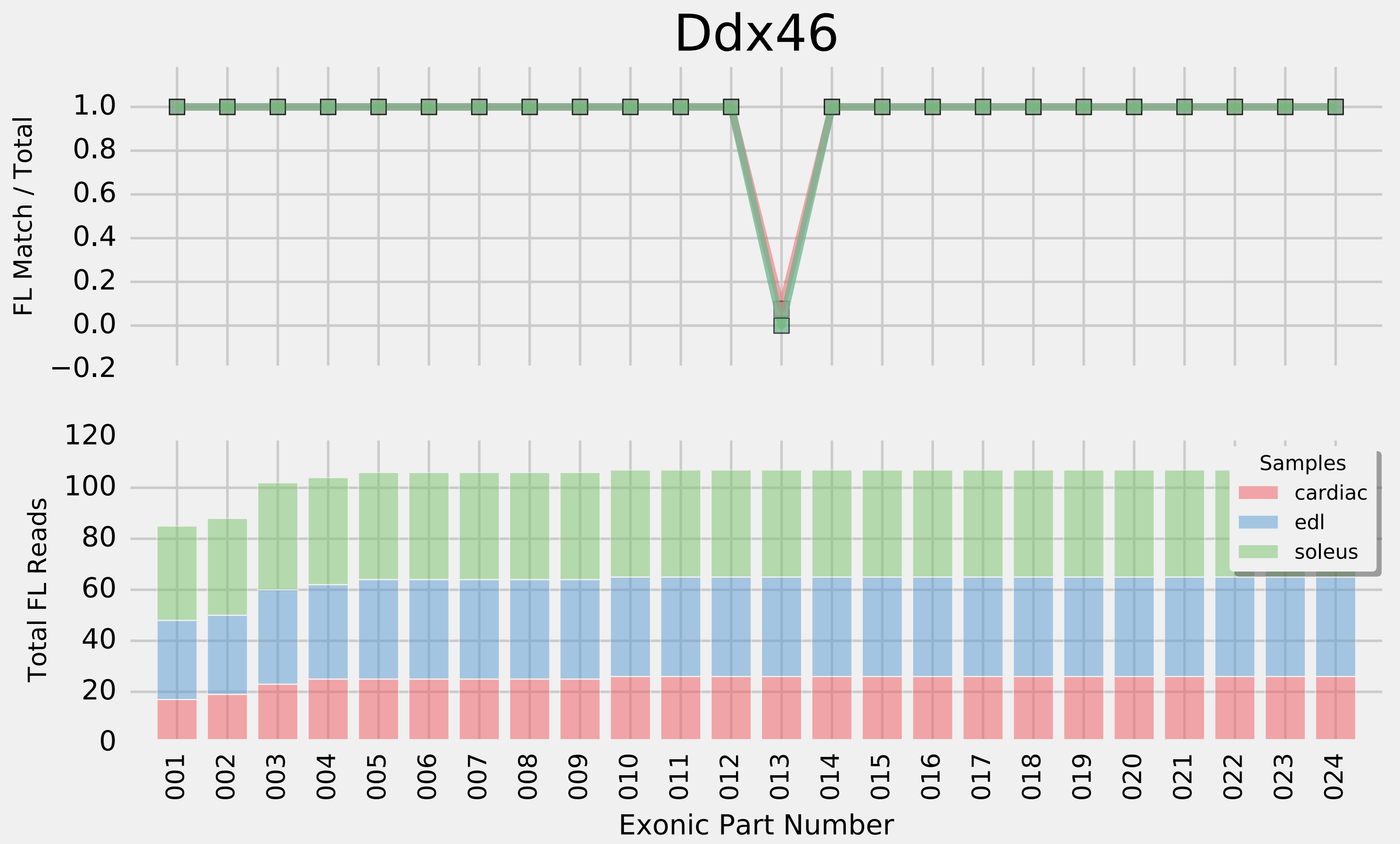

Ddx46
FL Match / Total
1.0
0.8
0.6
0.4
0.2
0.0
−0.2
Total FL Reads
120
100
80
60
40
20
0
Samples
cardiac
edl
soleus
001
002
003
004
005
006
007
008
009
010
011
012
013
014
015
016
017
018
019
020
021
022
023
024
Exonic Part Number

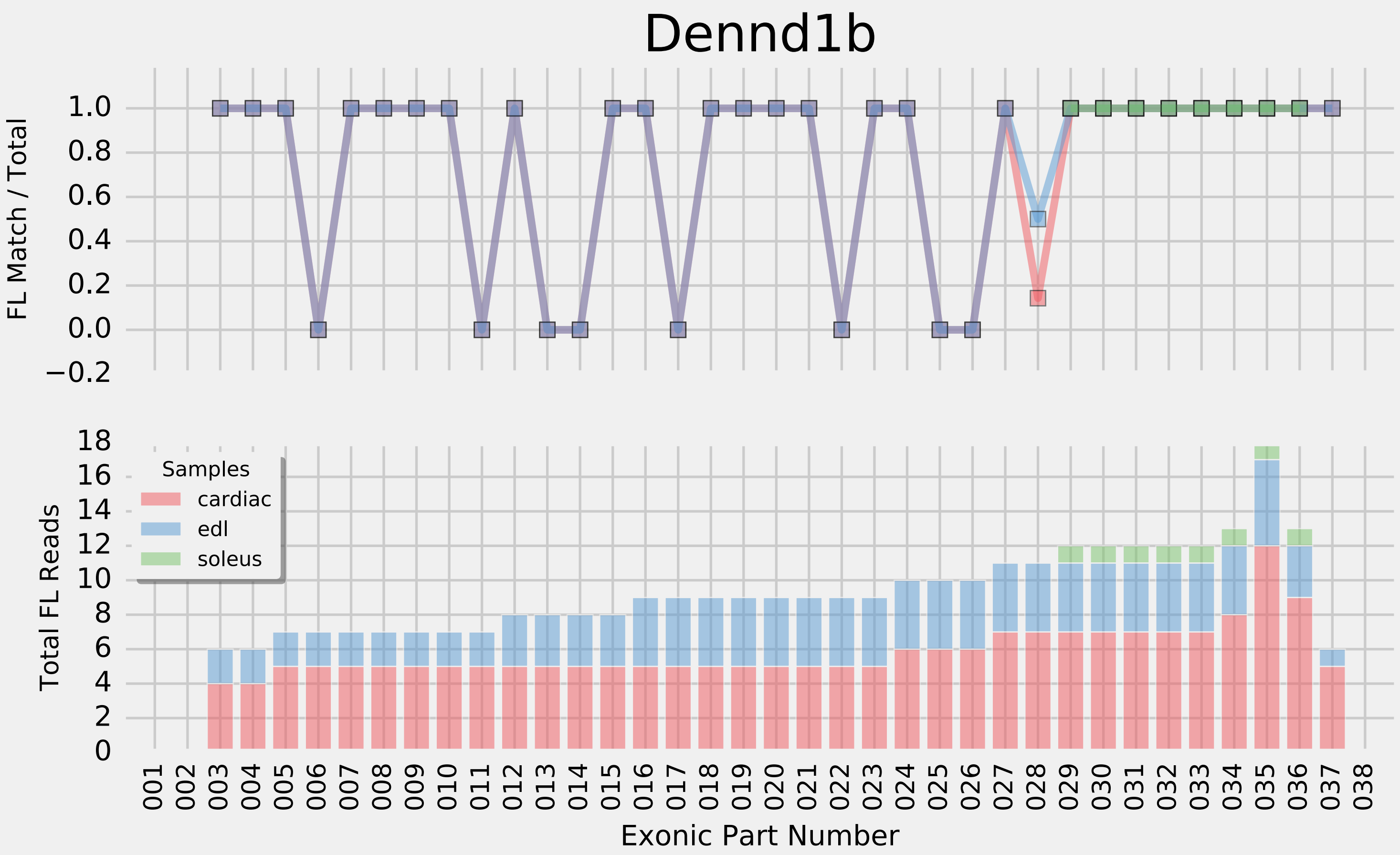

Dennd1b
FL Match / Total
1.0
0.8
0.6
0.4
0.2
0.0
−0.2
Total FL Reads
18
16
14
12
10
8
6
4
2
0
Samples
cardiac
edl
soleus
001 002 003 004 005 006 007 008 009 010 011 012 013 014 015 016 017 018 019 020 021 022 023 024 025 026 027 028 029 030 031 032 033 034 035 036 037 038
Exonic Part Number

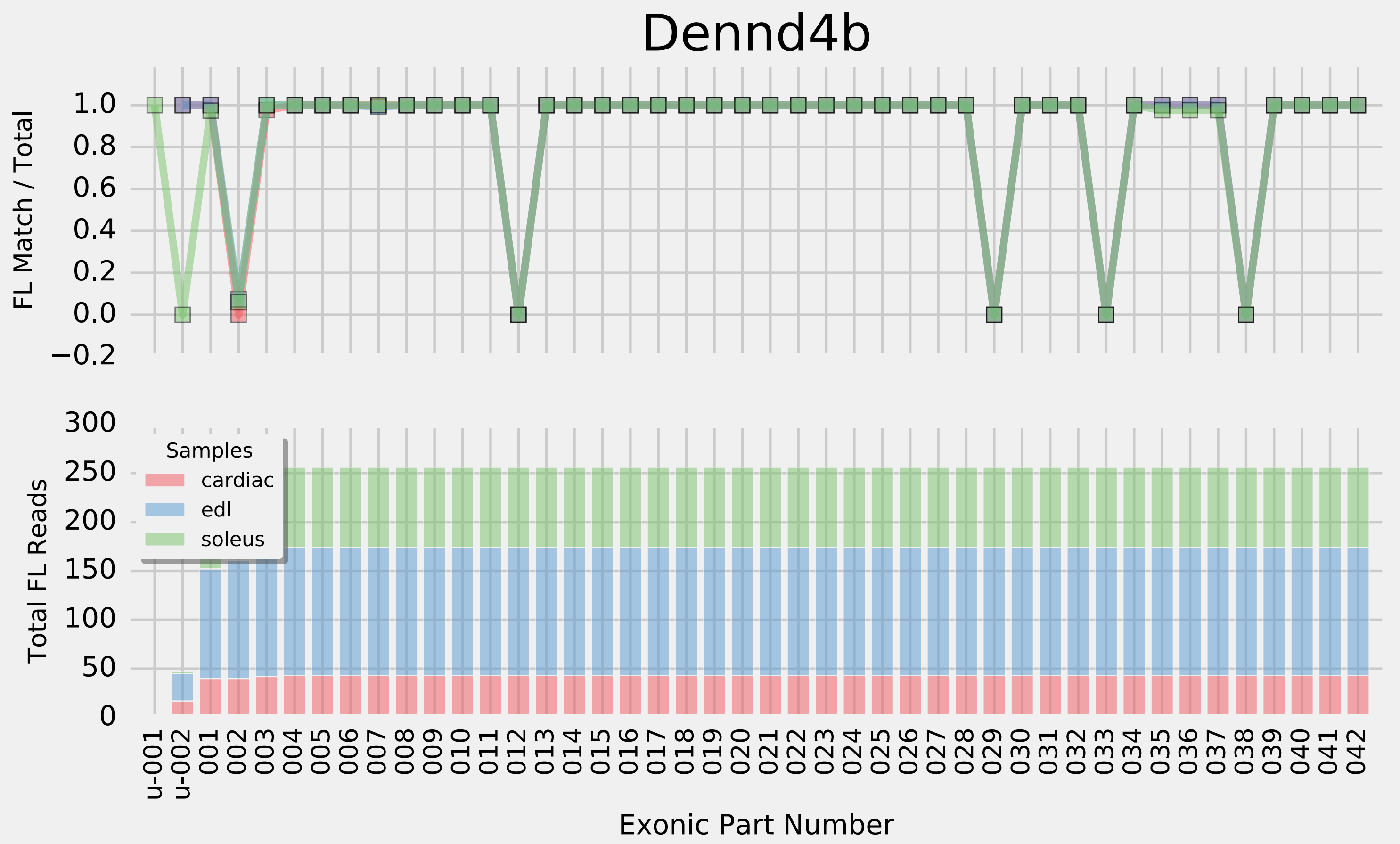

Dennd4b
FL Match / Total
1.0
0.8
0.6
0.4
0.2
0.0
−0.2
300
250
200
150
100
50
0
Total FL Reads
Samples
cardiac
edl
soleus
u-001
u-002
001
002
003
004
005
006
007
008
009
010
011
012
013
014
015
016
017
018
019
020
021
022
023
024
025
026
027
028
029
030
031
032
033
034
035
036
037
038
039
040
041
042
Exonic Part Number

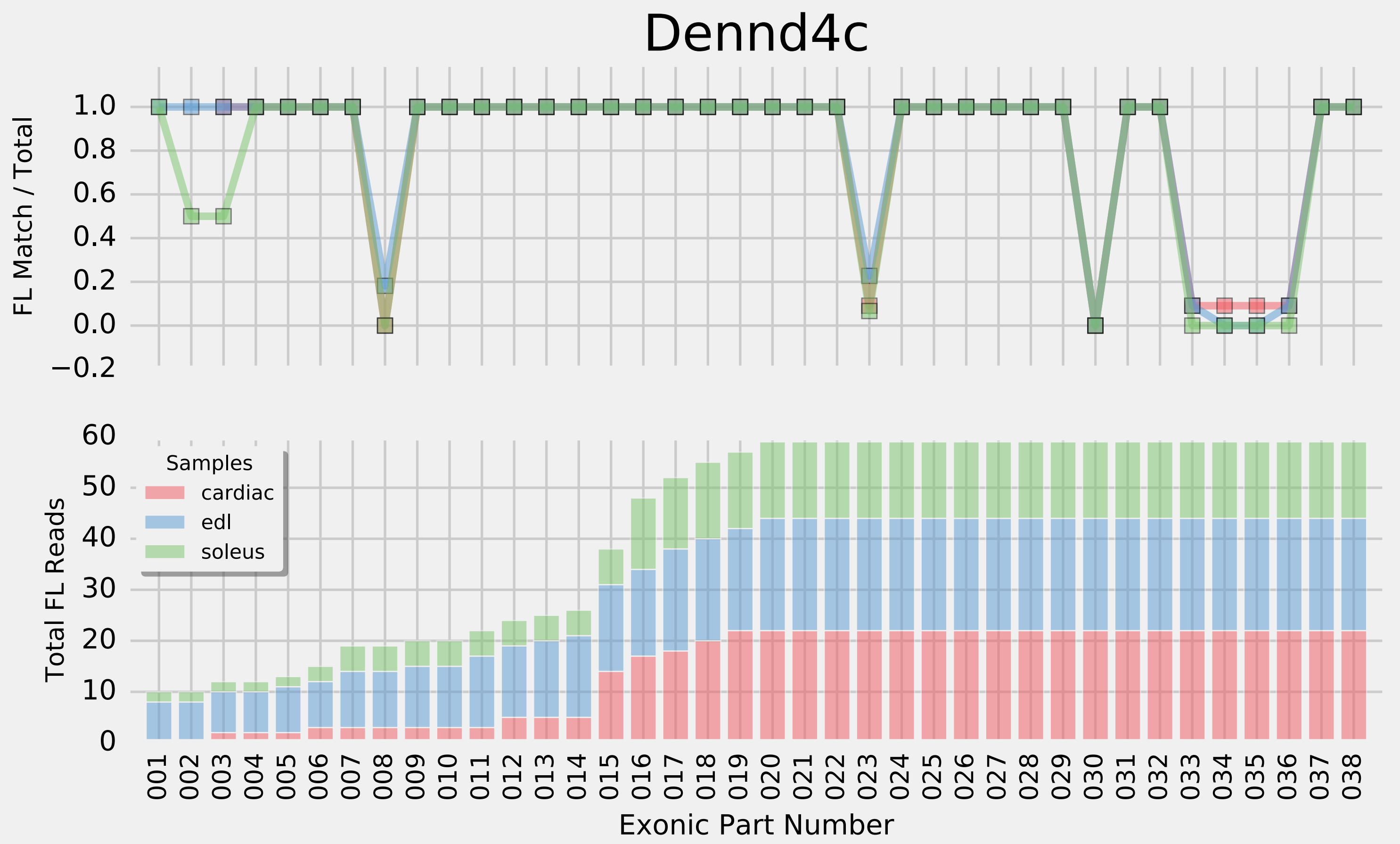
Dennd4c
FL Match / Total
1.0
0.8
0.6
0.4
0.2
0.0
−0.2
Total FL Reads
60
50
40
30
20
10
0
Samples
cardiac
edl
soleus
001 002 003 004 005 006 007 008 009 010 011 012 013 014 015 016 017 018 019 020 021 022 023 024 025 026 027 028 029 030 031 032 033 034 035 036 037 038
Exonic Part Number

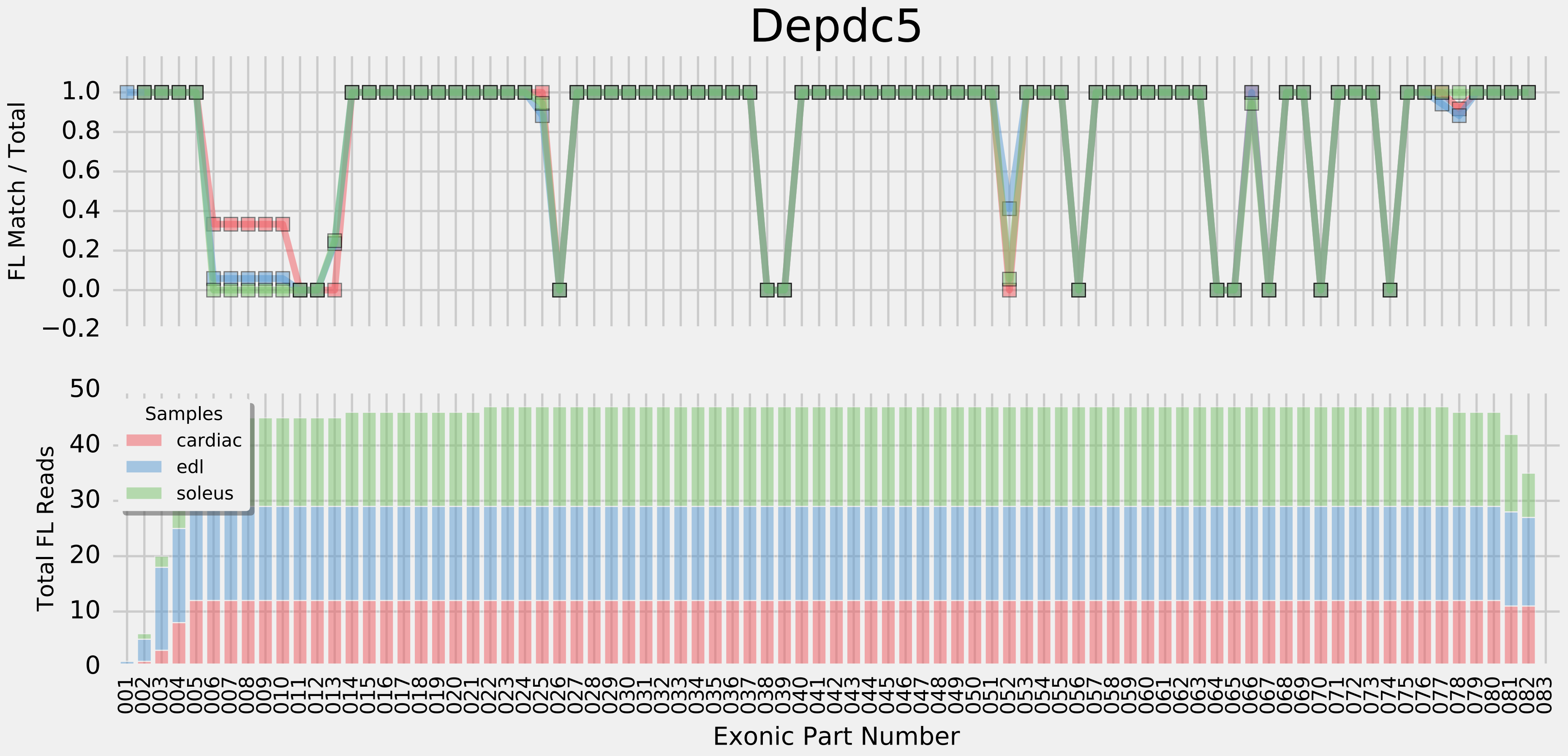

Depdc5
FL Match / Total
Total FL Reads
Samples
cardiac
edl
soleus
Exonic Part Number

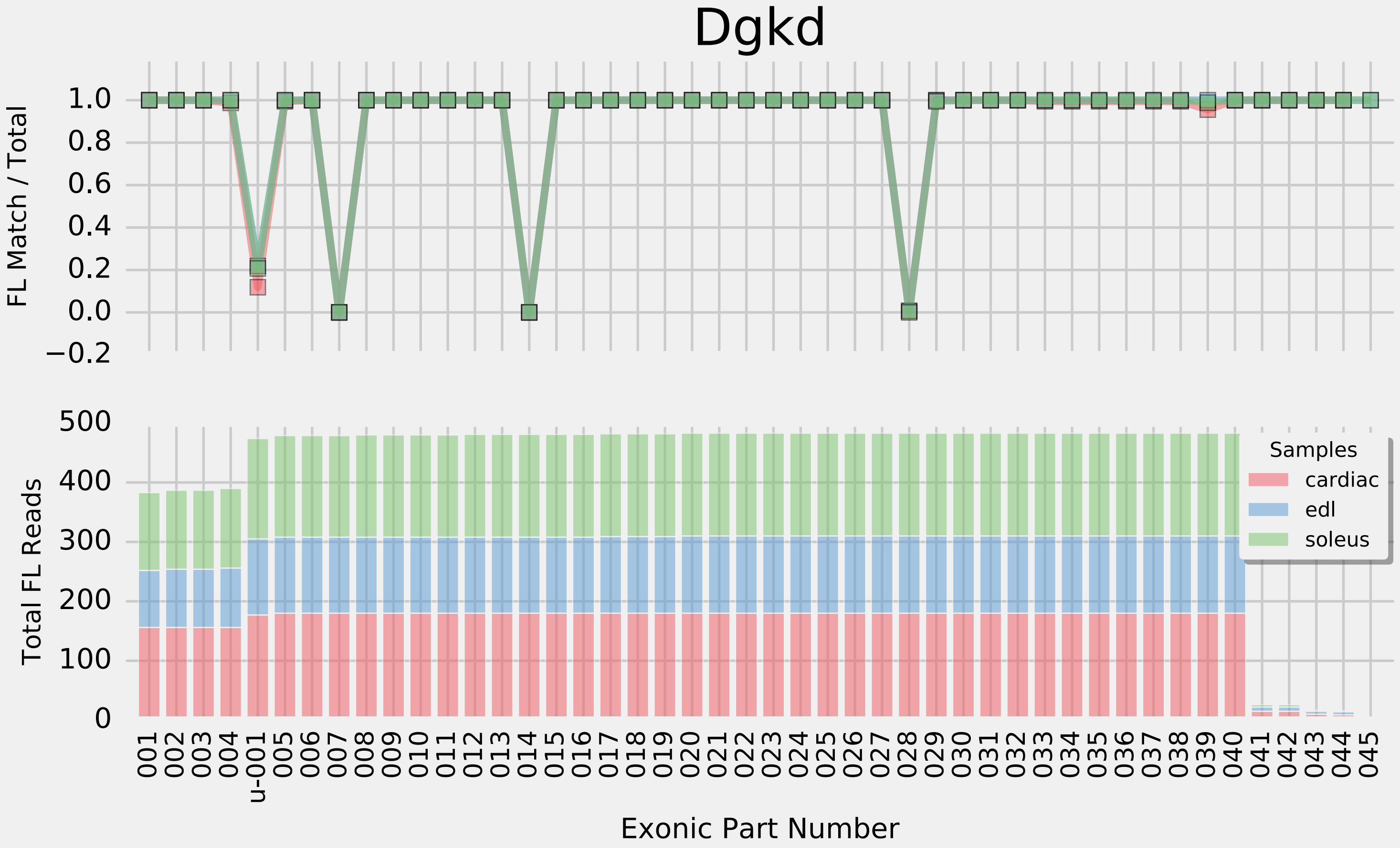

Dgkd
FL Match / Total
1.0
0.8
0.6
0.4
0.2
0.0
−0.2
Total FL Reads
500
400
300
200
100
0
Samples
cardiac
edl
soleus
001 002 003 004 u-001 005 006 007 008 009 010 011 012 013 014 015 016 017 018 019 020 021 022 023 024 025 026 027 028 029 030 031 032 033 034 035 036 037 038 039 040 041 042 043 044 045
Exonic Part Number

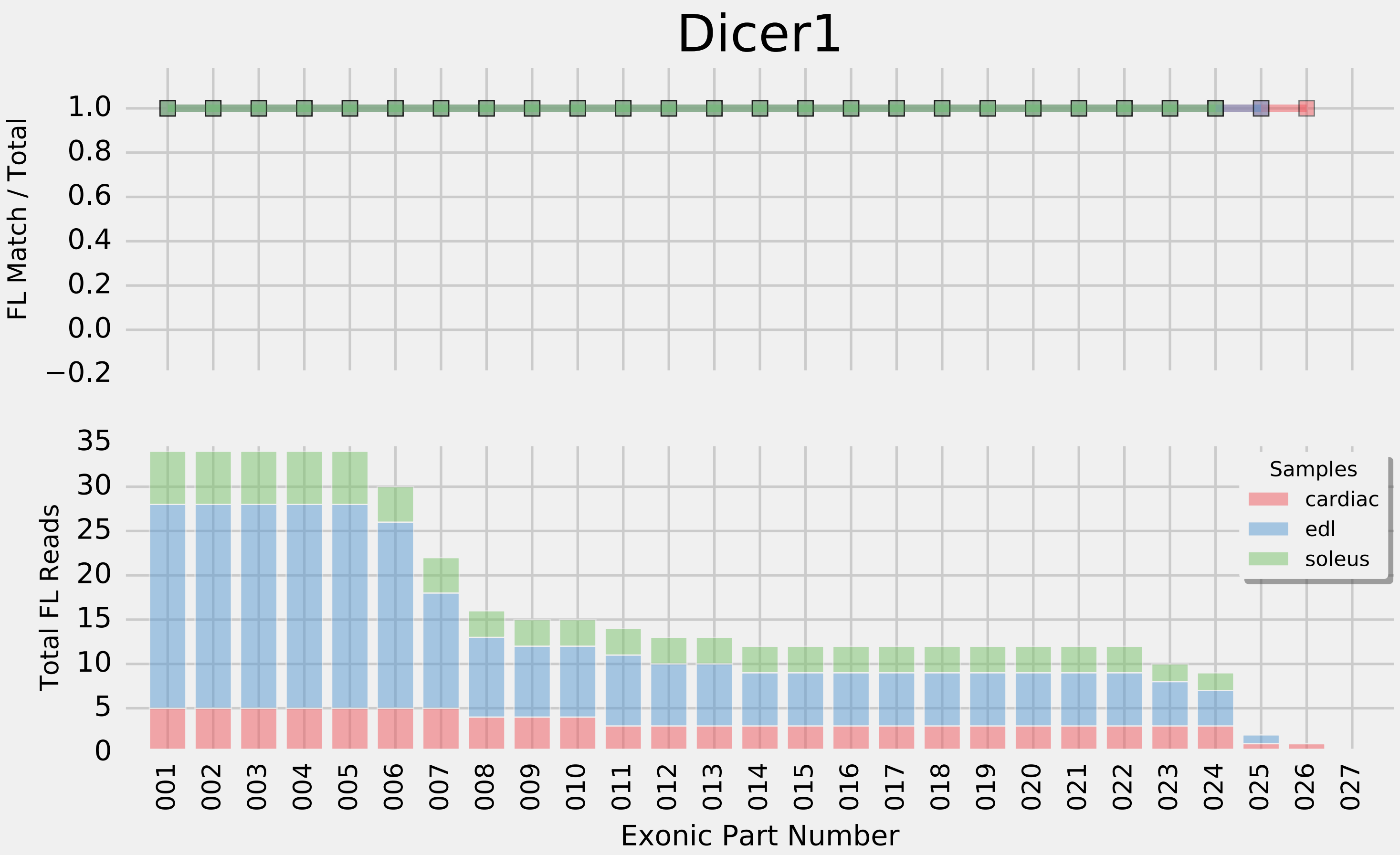
Dicer1
FL Match / Total
1.0
0.8
0.6
0.4
0.2
0.0
−0.2
Total FL Reads
35
30
25
20
15
10
5
0
Samples
cardiac
edl
soleus
001
002
003
004
005
006
007
008
009
010
011
012
013
014
015
016
017
018
019
020
021
022
023
024
025
026
027
Exonic Part Number

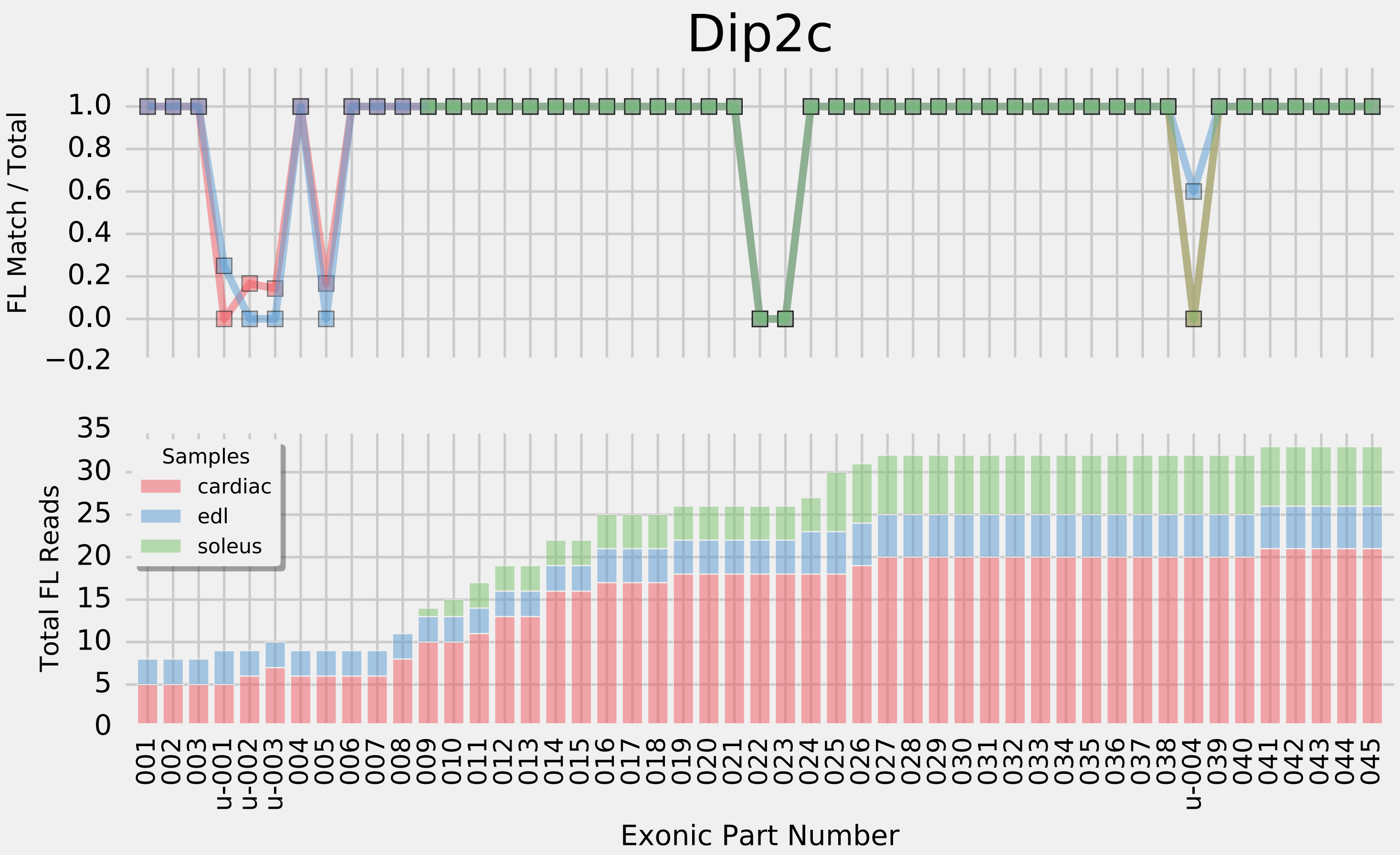

Dip2c
FL Match / Total
1.0
0.8
0.6
0.4
0.2
0.0
−0.2
Total FL Reads
35
30
25
20
15
10
5
0
Samples
cardiac
edl
soleus
001
002
003
u-001
u-002
u-003
004
005
006
007
008
009
010
011
012
013
014
015
016
017
018
019
020
021
022
023
024
025
026
027
028
029
030
031
032
033
034
035
036
037
038
u-004
039
040
041
042
043
044
045
Exonic Part Number

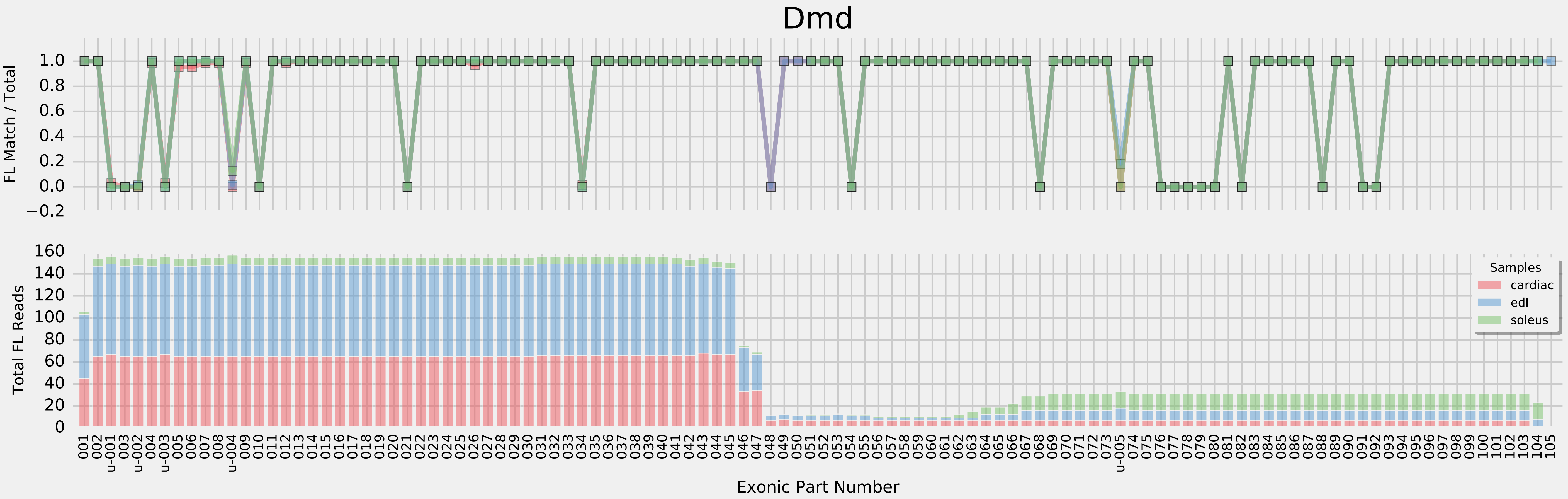
Dmd
FL Match / Total
1.0 0.8 0.6 0.4 0.2 0.0 −0.2
Total FL Reads
160 140 120 100 80 60 40 20 0
Samples
cardiac
edl
soleus
001 002 u-001 003 u-002 004 u-003 005 006 007 008 u-004 009 010 011 012 013 014 015 016 017 018 019 020 021 022 023 024 025 026 027 028 029 030 031 032 033 034 035 036 037 038 039 040 041 042 043 044 045 046 047 048 049 050 051 052 053 054 055 056 057 058 059 060 061 062 063 064 065 066 067 068 069 070 071 072 073 u-005 074 075 076 077 078 079 080 081 082 083 084 085 086 087 088 089 090 091 092 093 094 095 096 097 098 099 100 101 102 103 104 105
Exonic Part Number

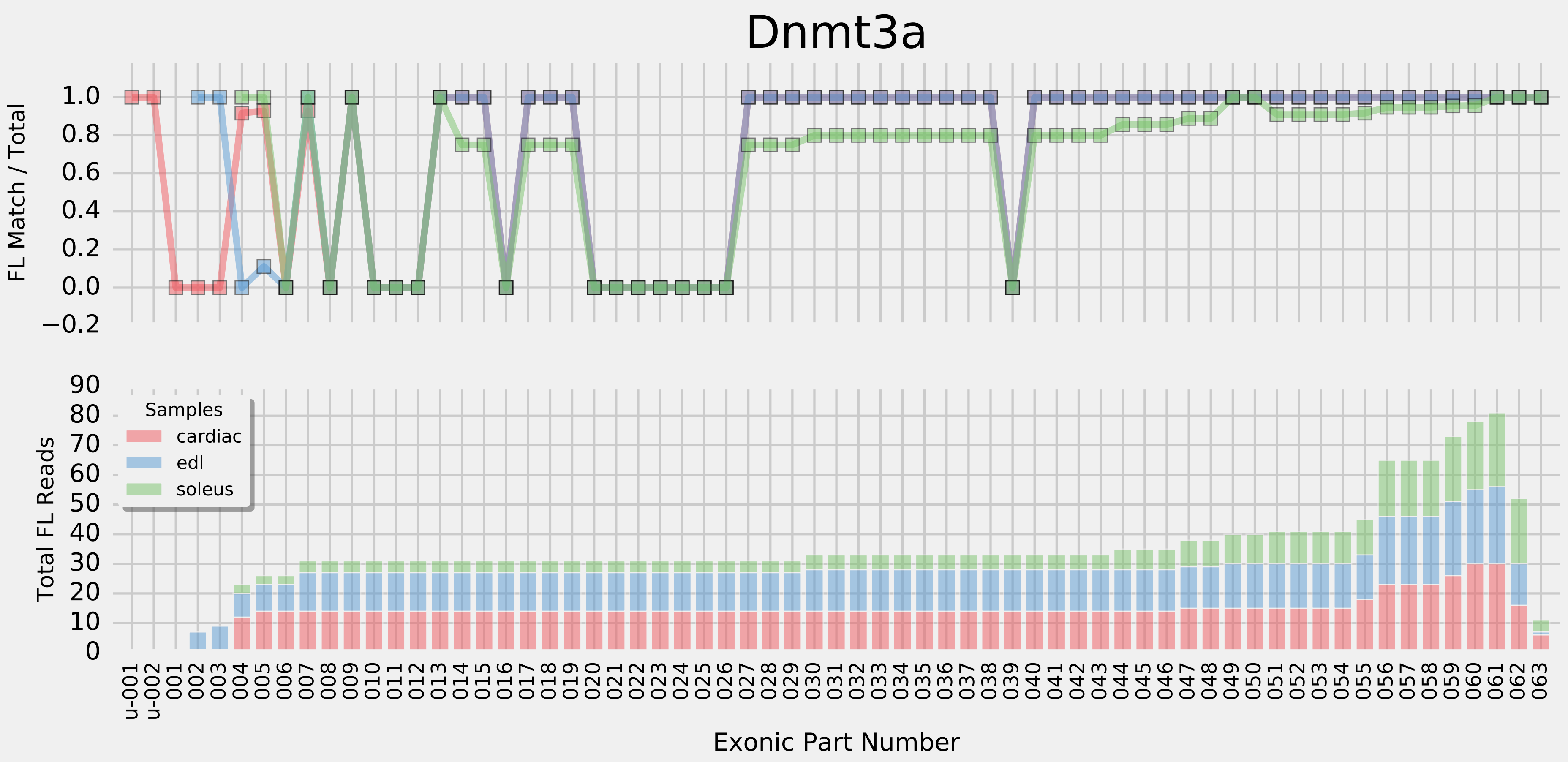

Dnmt3a
FL Match / Total
Total FL Reads
Samples
cardiac
edl
soleus
Exonic Part Number

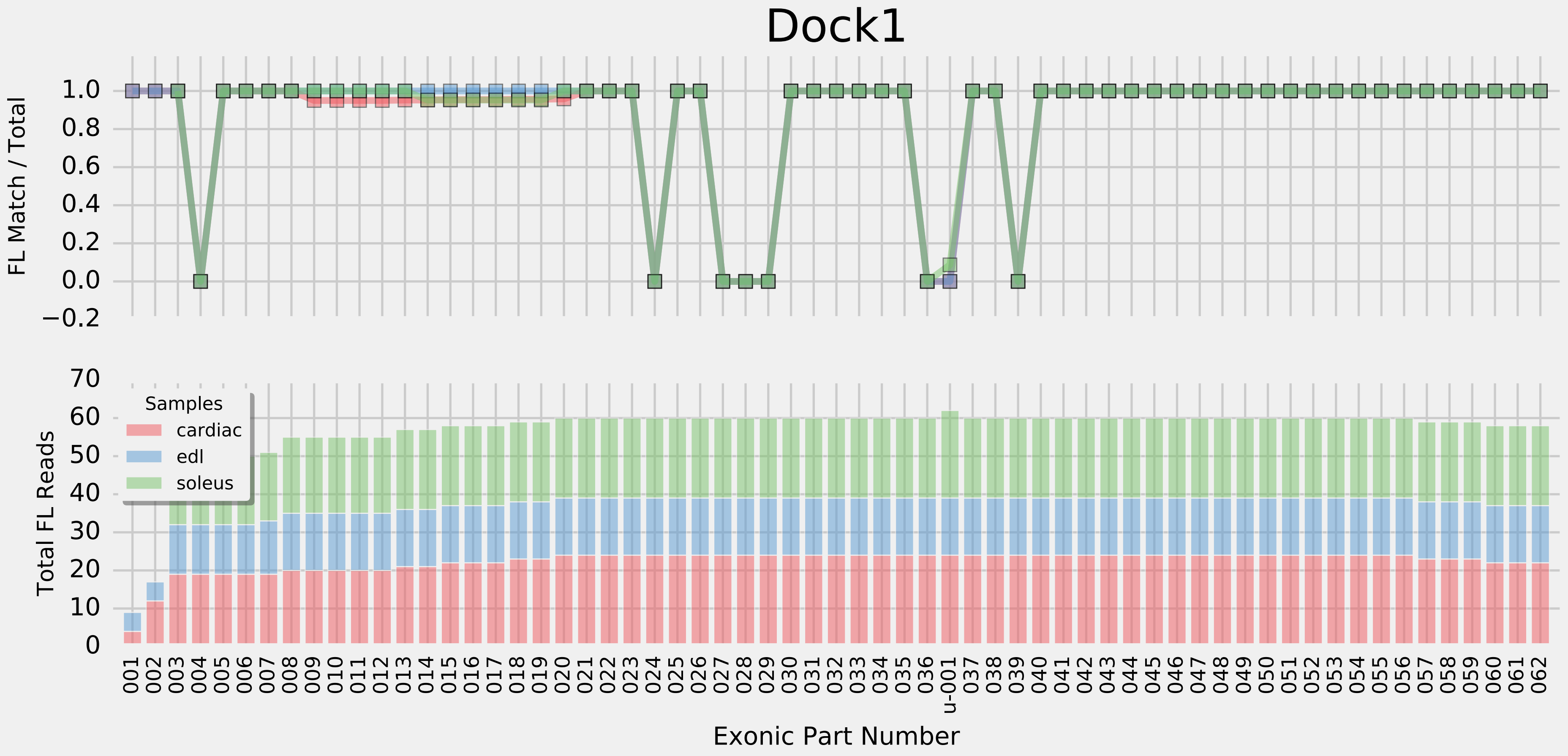

Dock1
FL Match / Total
Total FL Reads
Samples
cardiac
edl
soleus
Exonic Part Number
001 002 003 004 005 006 007 008 009 010 011 012 013 014 015 016 017 018 019 020 021 022 023 024 025 026 027 028 029 030 031 032 033 034 035 036 u-001 037 038 039 040 041 042 043 044 045 046 047 048 049 050 051 052 053 054 055 056 057 058 059 060 061 062

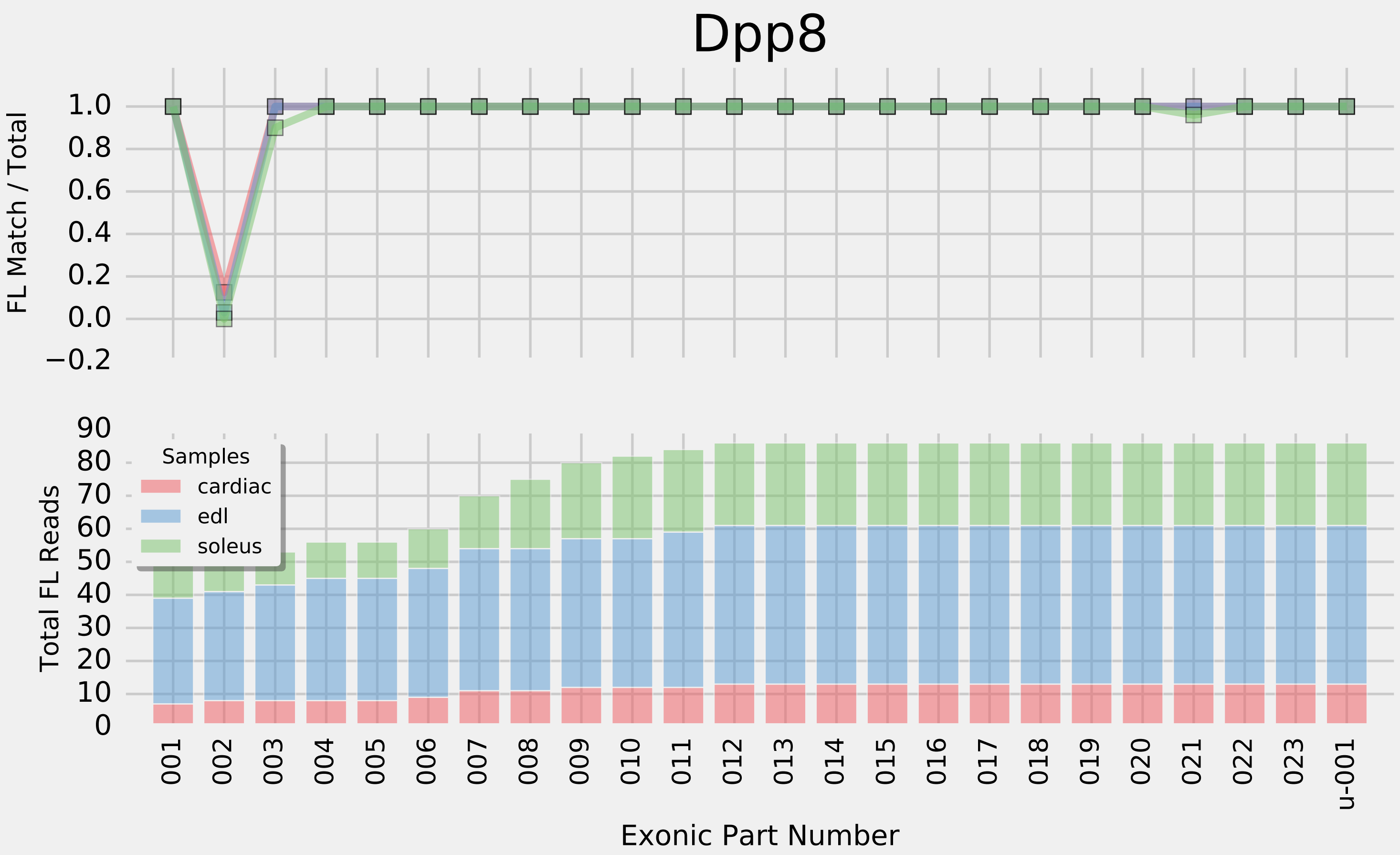

Dpp8
FL Match / Total
1.0
0.8
0.6
0.4
0.2
0.0
−0.2
Total FL Reads
90
80
70
60
50
40
30
20
10
0
Samples
cardiac
edl
soleus
001
002
003
004
005
006
007
008
009
010
011
012
013
014
015
016
017
018
019
020
021
022
023
u-001
Exonic Part Number

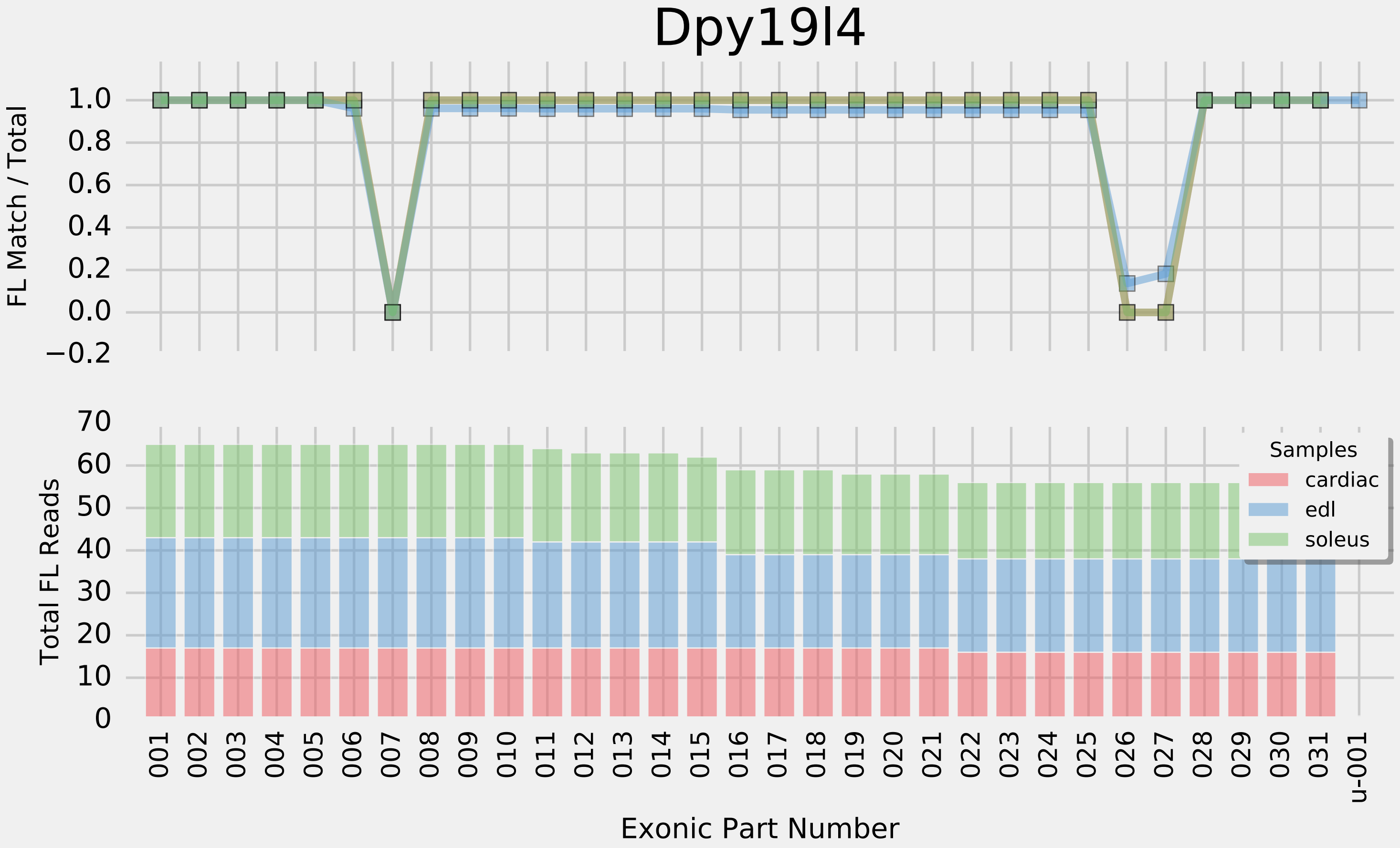

Dpy19l4
FL Match / Total
1.0
0.8
0.6
0.4
0.2
0.0
−0.2
Total FL Reads
70
60
50
40
30
20
10
0
Samples
cardiac
edl
soleus
001
002
003
004
005
006
007
008
009
010
011
012
013
014
015
016
017
018
019
020
021
022
023
024
025
026
027
028
029
030
031
u-001
Exonic Part Number

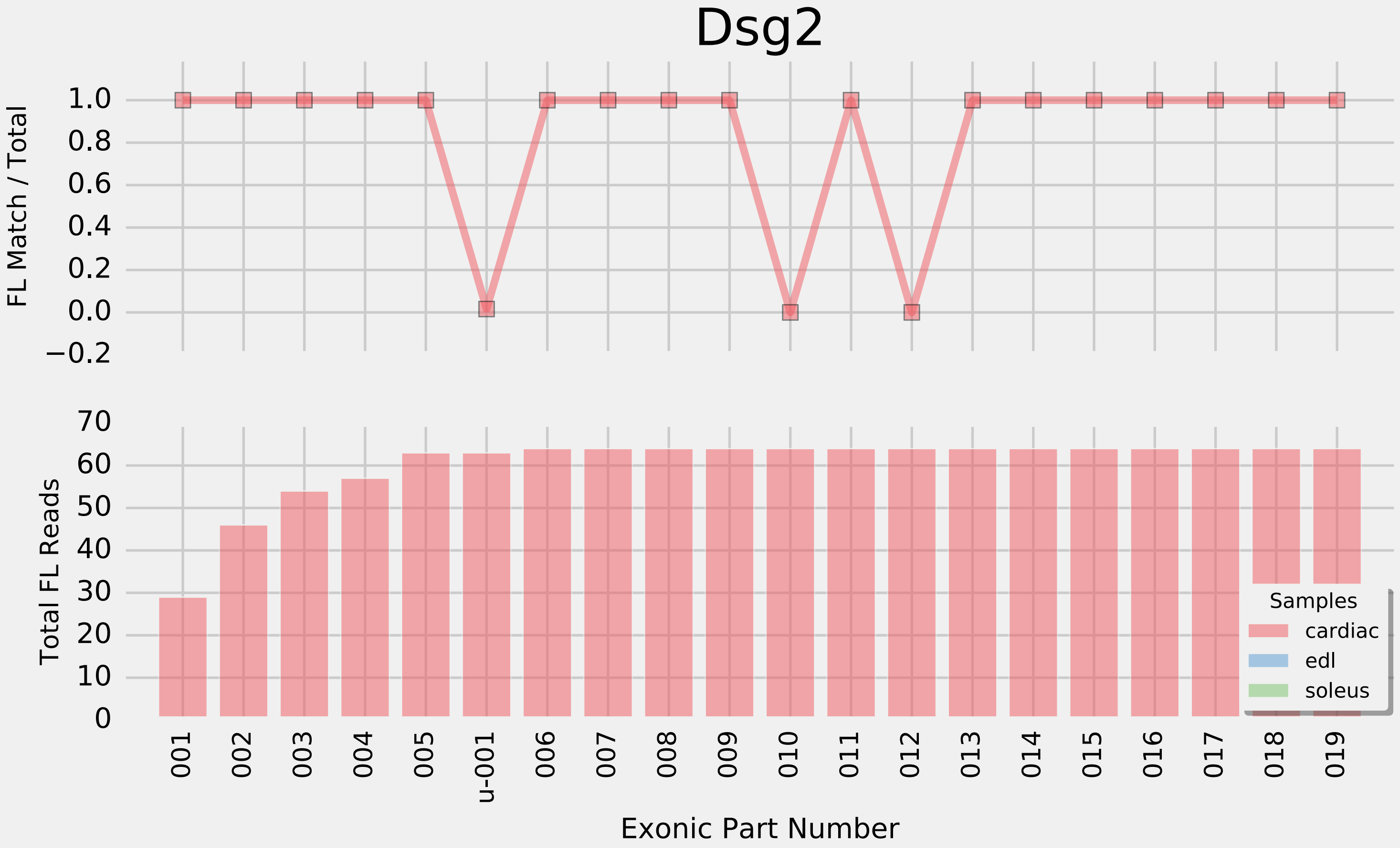

Dsg2
FL Match / Total
1.0
0.8
0.6
0.4
0.2
0.0
−0.2
Total FL Reads
70
60
50
40
30
20
10
0
001
002
003
004
005
u-001
006
007
008
009
010
011
012
013
014
015
016
017
018
019
Exonic Part Number
Samples
cardiac
edl
soleus

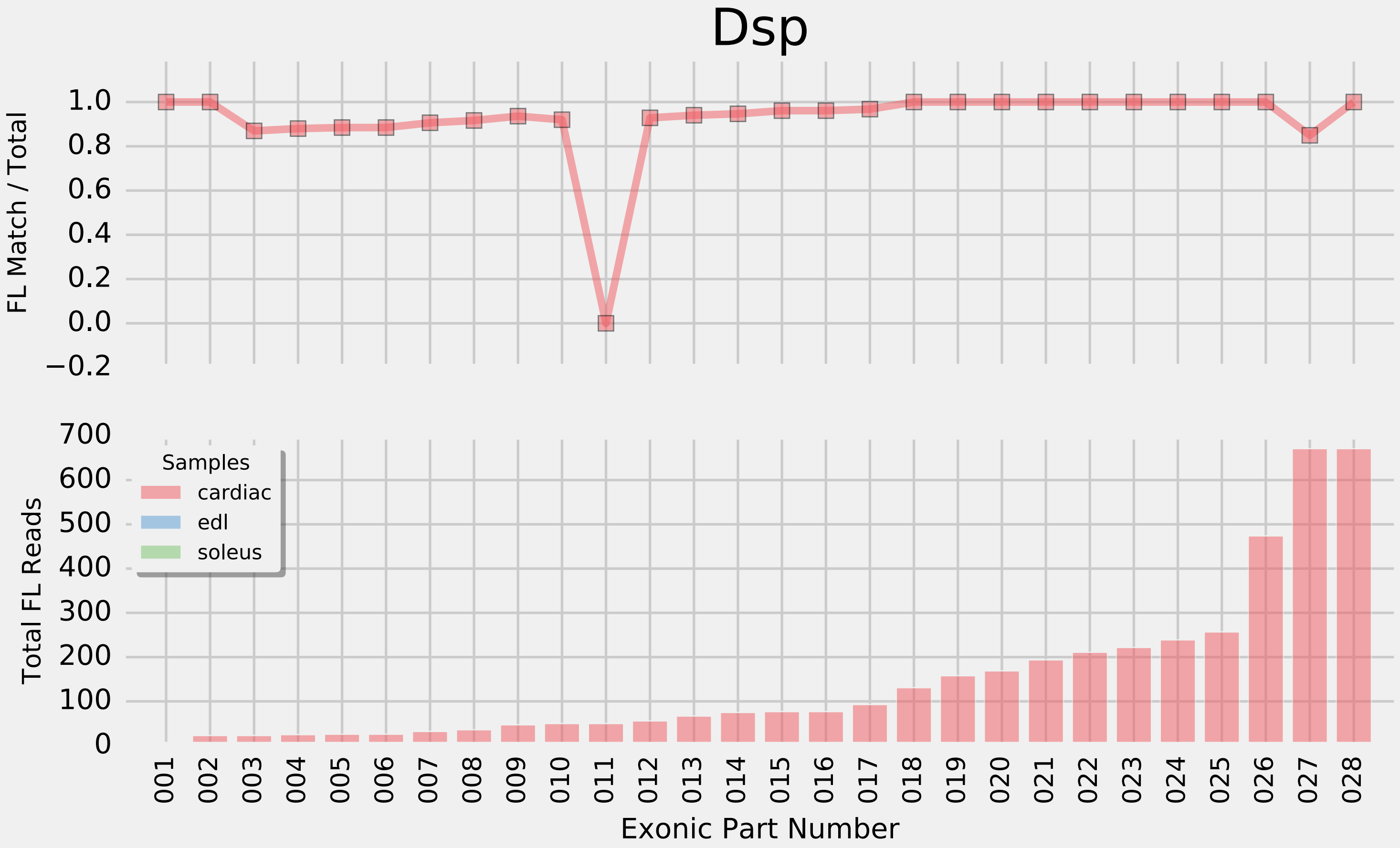

Dsp
FL Match / Total
1.0
0.8
0.6
0.4
0.2
0.0
−0.2
Total FL Reads
700
600
500
400
300
200
100
0
Samples
cardiac
edl
soleus
001
002
003
004
005
006
007
008
009
010
011
012
013
014
015
016
017
018
019
020
021
022
023
024
025
026
027
028
Exonic Part Number

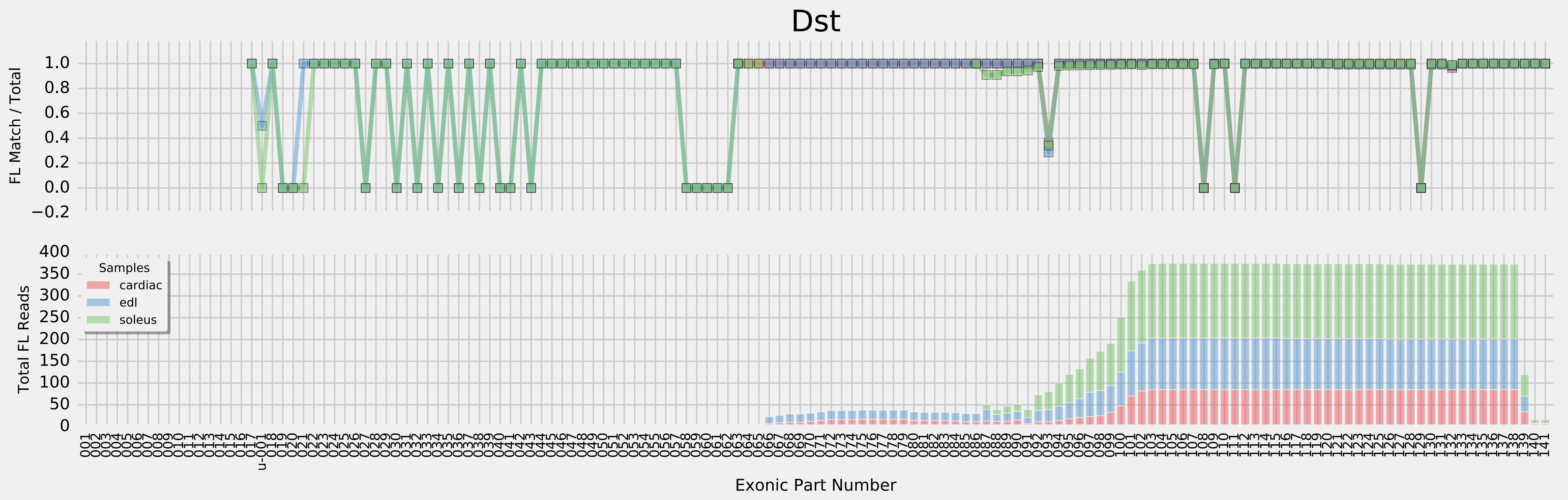
Dst
FL Match / Total
Total FL Reads
Samples
cardiac
edl
soleus
Exonic Part Number

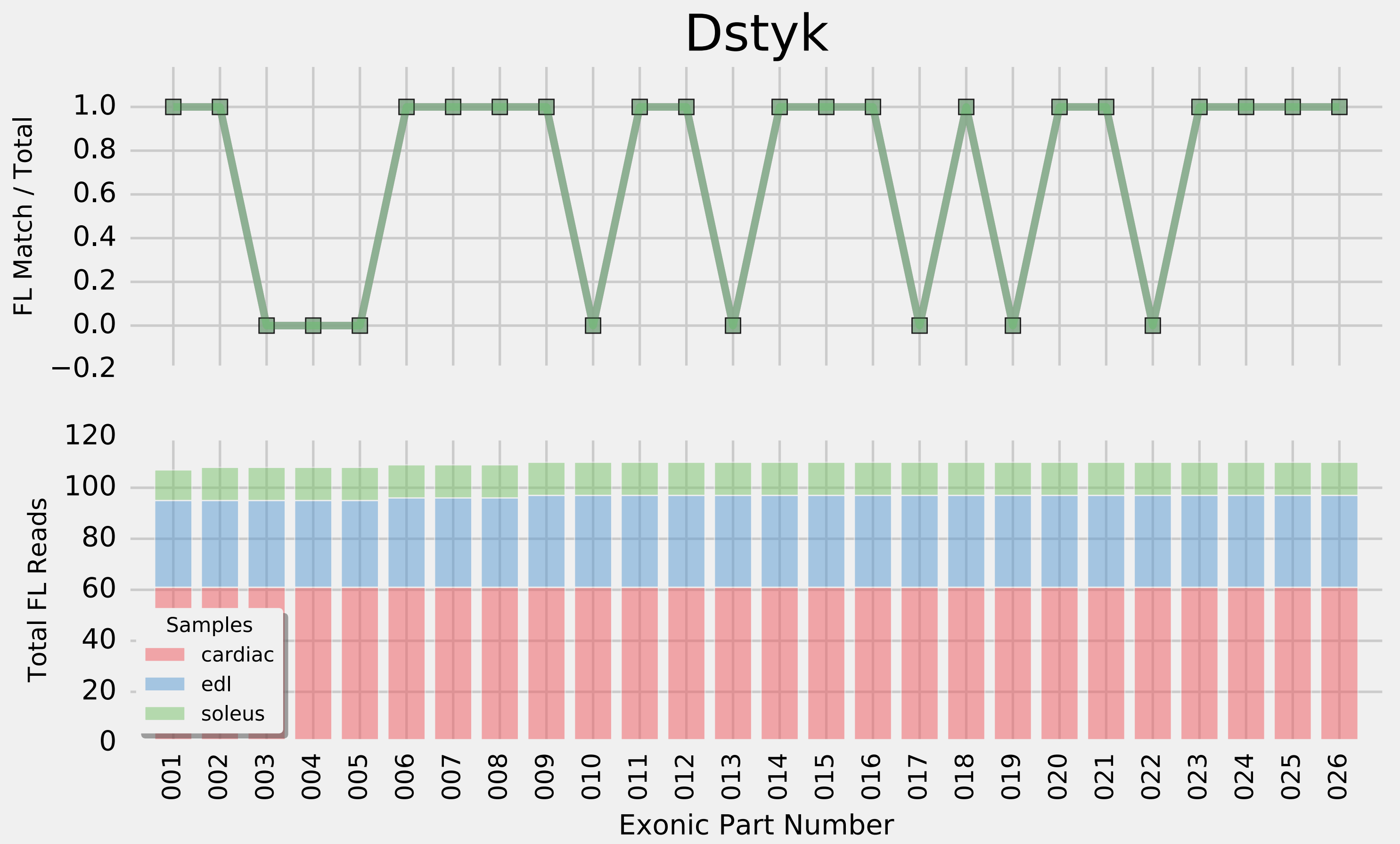
Dstyk
FL Match / Total
1.0
0.8
0.6
0.4
0.2
0.0
−0.2
Total FL Reads
120
100
80
60
40
20
0
Samples
cardiac
edl
soleus
001
002
003
004
005
006
007
008
009
010
011
012
013
014
015
016
017
018
019
020
021
022
023
024
025
026
Exonic Part Number

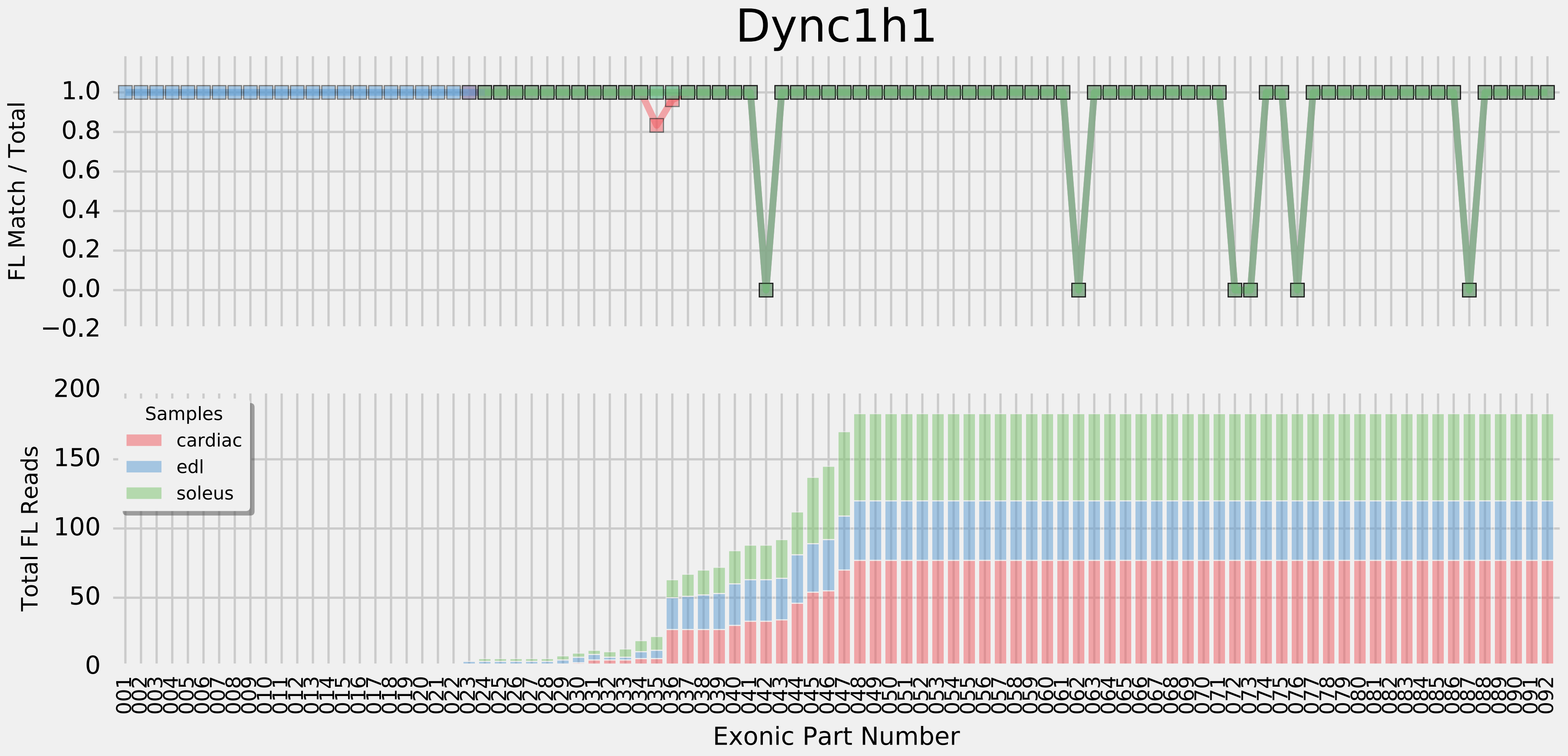

Dync1h1
FL Match / Total
Total FL Reads
Samples
cardiac
edl
soleus
Exonic Part Number

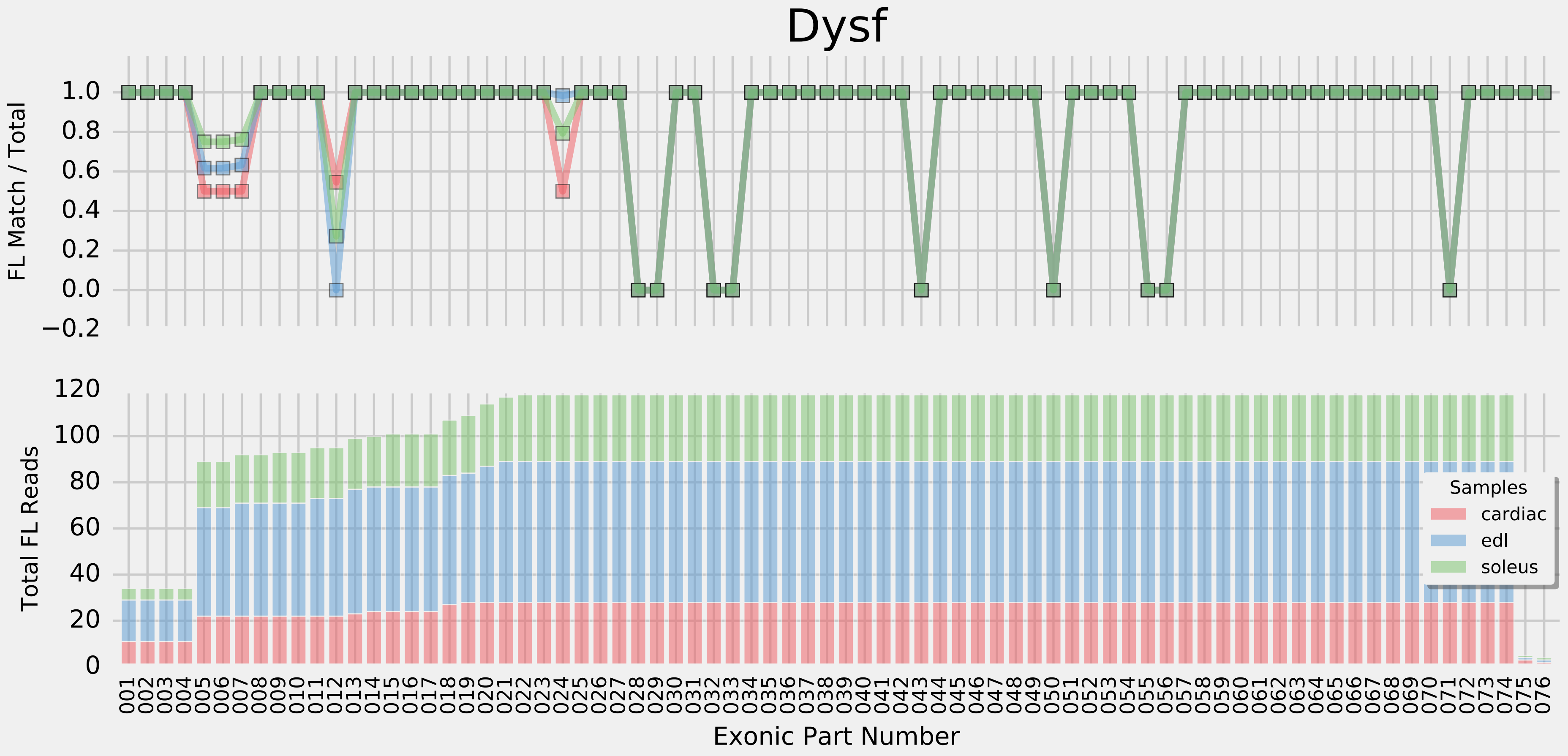

Dysf
FL Match / Total
Total FL Reads
Exonic Part Number
Samples
cardiac
edl
soleus

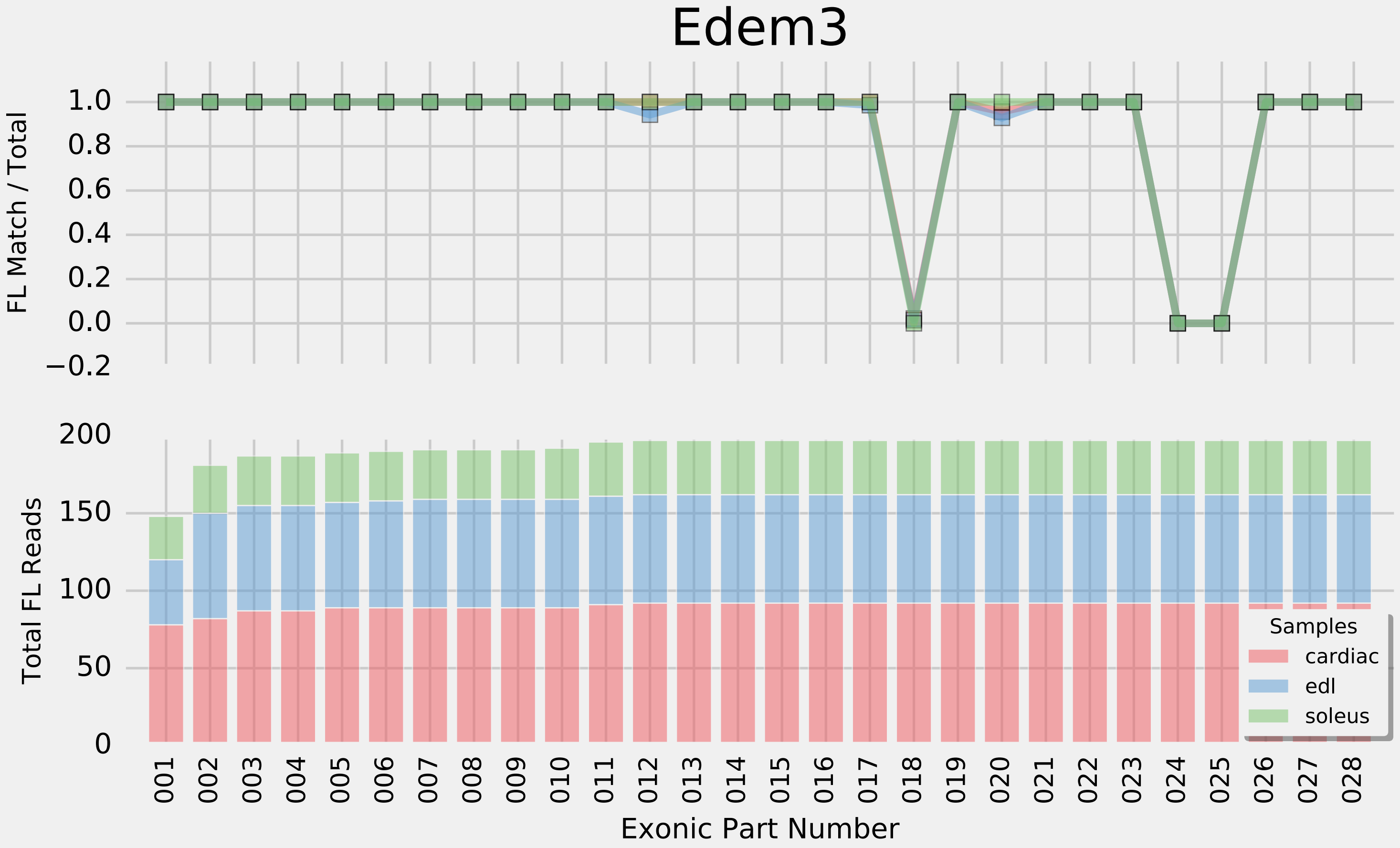

Edem3
FL Match / Total
1.0
0.8
0.6
0.4
0.2
0.0
−0.2
Total FL Reads
200
150
100
50
0
001
002
003
004
005
006
007
008
009
010
011
012
013
014
015
016
017
018
019
020
021
022
023
024
025
026
027
028
Exonic Part Number
Samples
cardiac
edl
soleus

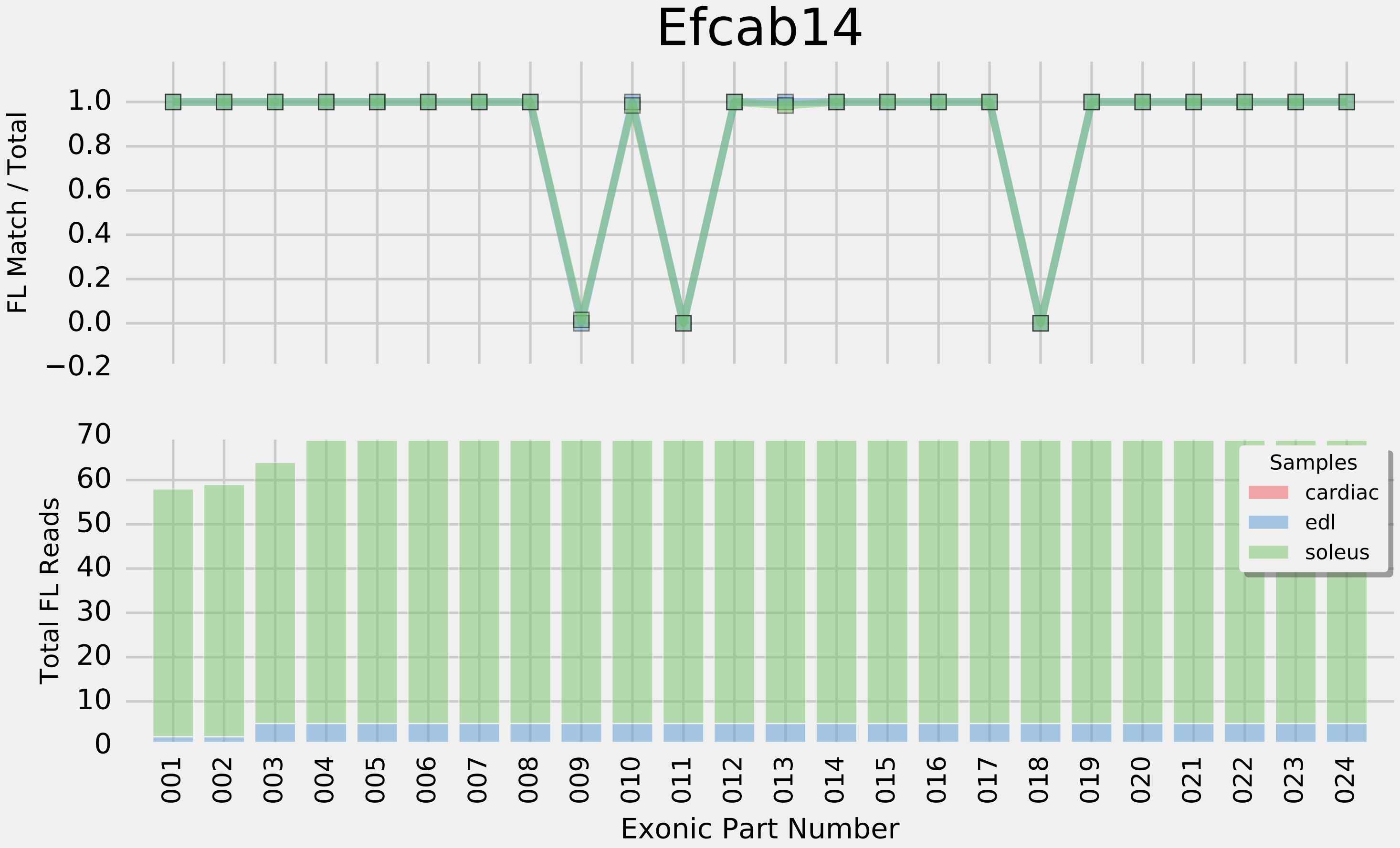

Efcab14
FL Match / Total
1.0
0.8
0.6
0.4
0.2
0.0
−0.2
Total FL Reads
70
60
50
40
30
20
10
0
Samples
cardiac
edl
soleus
001
002
003
004
005
006
007
008
009
010
011
012
013
014
015
016
017
018
019
020
021
022
023
024
Exonic Part Number

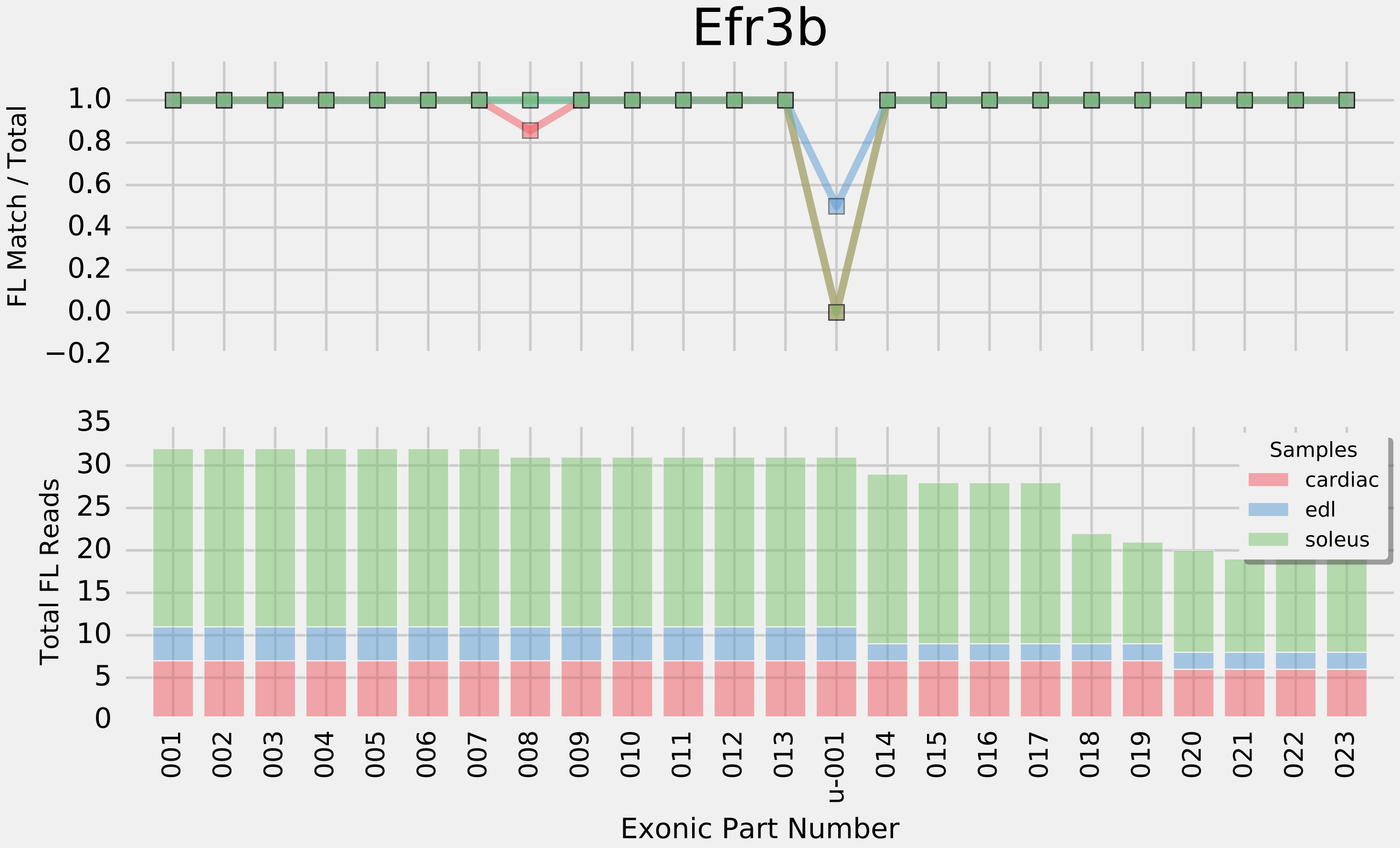
Efr3b
FL Match / Total
1.0
0.8
0.6
0.4
0.2
0.0
−0.2
Total FL Reads
35
30
25
20
15
10
5
0
Samples
cardiac
edl
soleus
001
002
003
004
005
006
007
008
009
010
011
012
013
u-001
014
015
016
017
018
019
020
021
022
023
Exonic Part Number

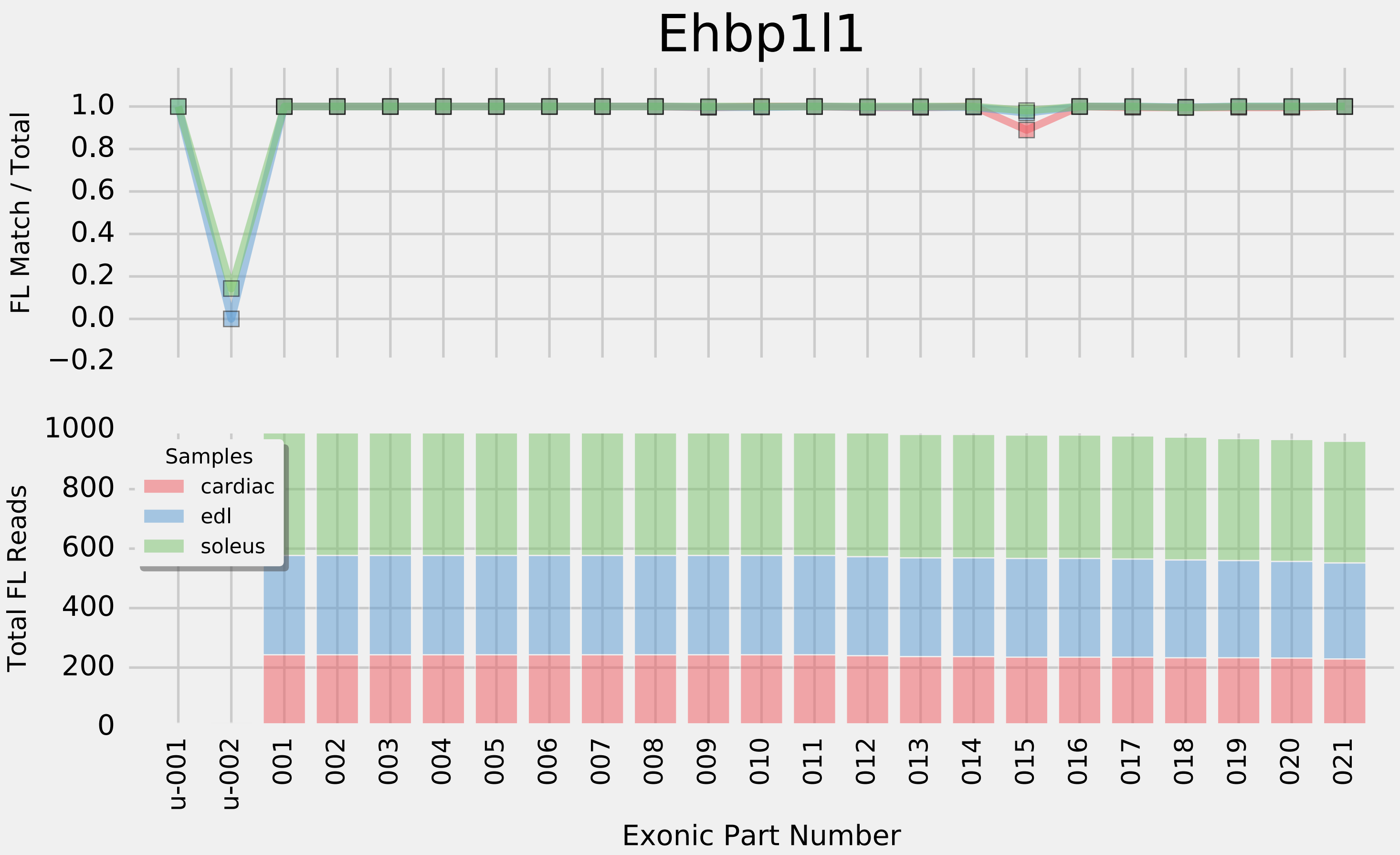
Ehbp1l1
FL Match / Total
1.0
0.8
0.6
0.4
0.2
0.0
−0.2
Total FL Reads
1000
800
600
400
200
0
Samples
cardiac
edl
soleus
u-001
u-002
001
002
003
004
005
006
007
008
009
010
011
012
013
014
015
016
017
018
019
020
021
Exonic Part Number

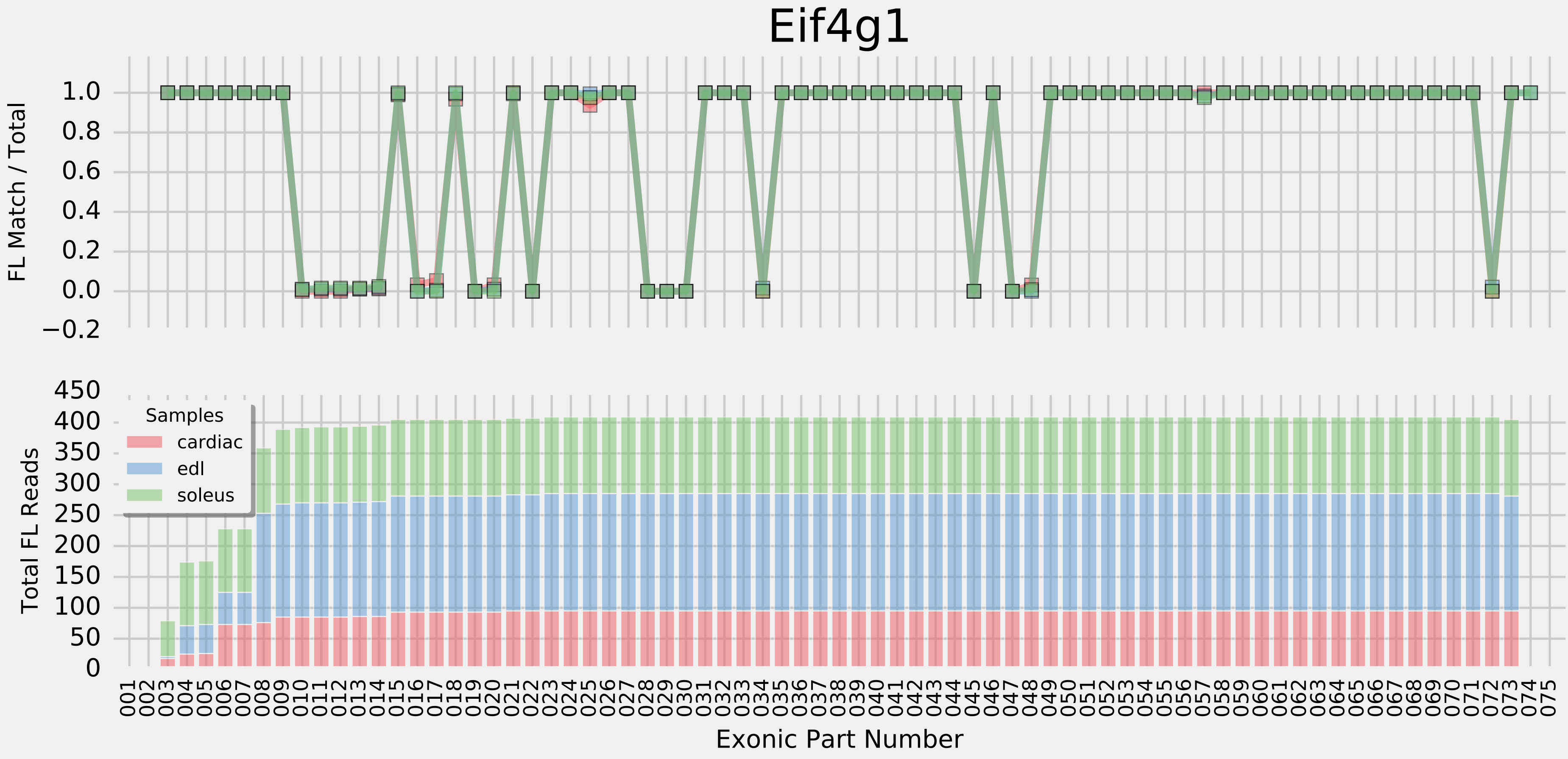

Eif4g1
FL Match / Total
1.0
0.8
0.6
0.4
0.2
0.0
−0.2
Total FL Reads
450
400
350
300
250
200
150
100
50
0
Samples
cardiac
edl
soleus
001 002 003 004 005 006 007 008 009 010 011 012 013 014 015 016 017 018 019 020 021 022 023 024 025 026 027 028 029 030 031 032 033 034 035 036 037 038 039 040 041 042 043 044 045 046 047 048 049 050 051 052 053 054 055 056 057 058 059 060 061 062 063 064 065 066 067 068 069 070 071 072 073 074 075
Exonic Part Number

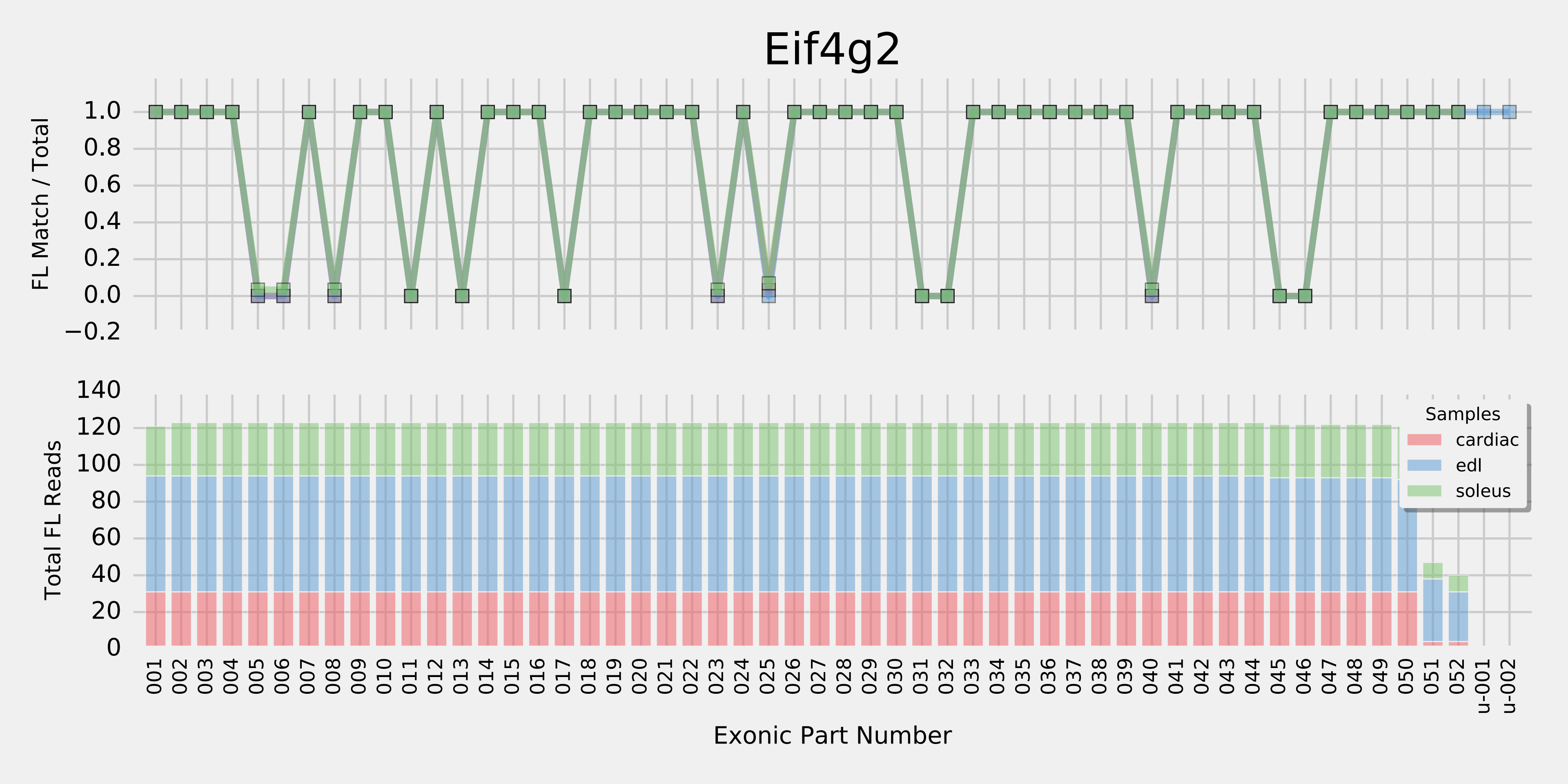

Eif4g2
FL Match / Total
1.0
0.8
0.6
0.4
0.2
0.0
−0.2
140
120
100
80
60
40
20
0
Total FL Reads
Samples
cardiac
edl
soleus
001 002 003 004 005 006 007 008 009 010 011 012 013 014 015 016 017 018 019 020 021 022 023 024 025 026 027 028 029 030 031 032 033 034 035 036 037 038 039 040 041 042 043 044 045 046 047 048 049 050 051 052 u-001 u-002
Exonic Part Number

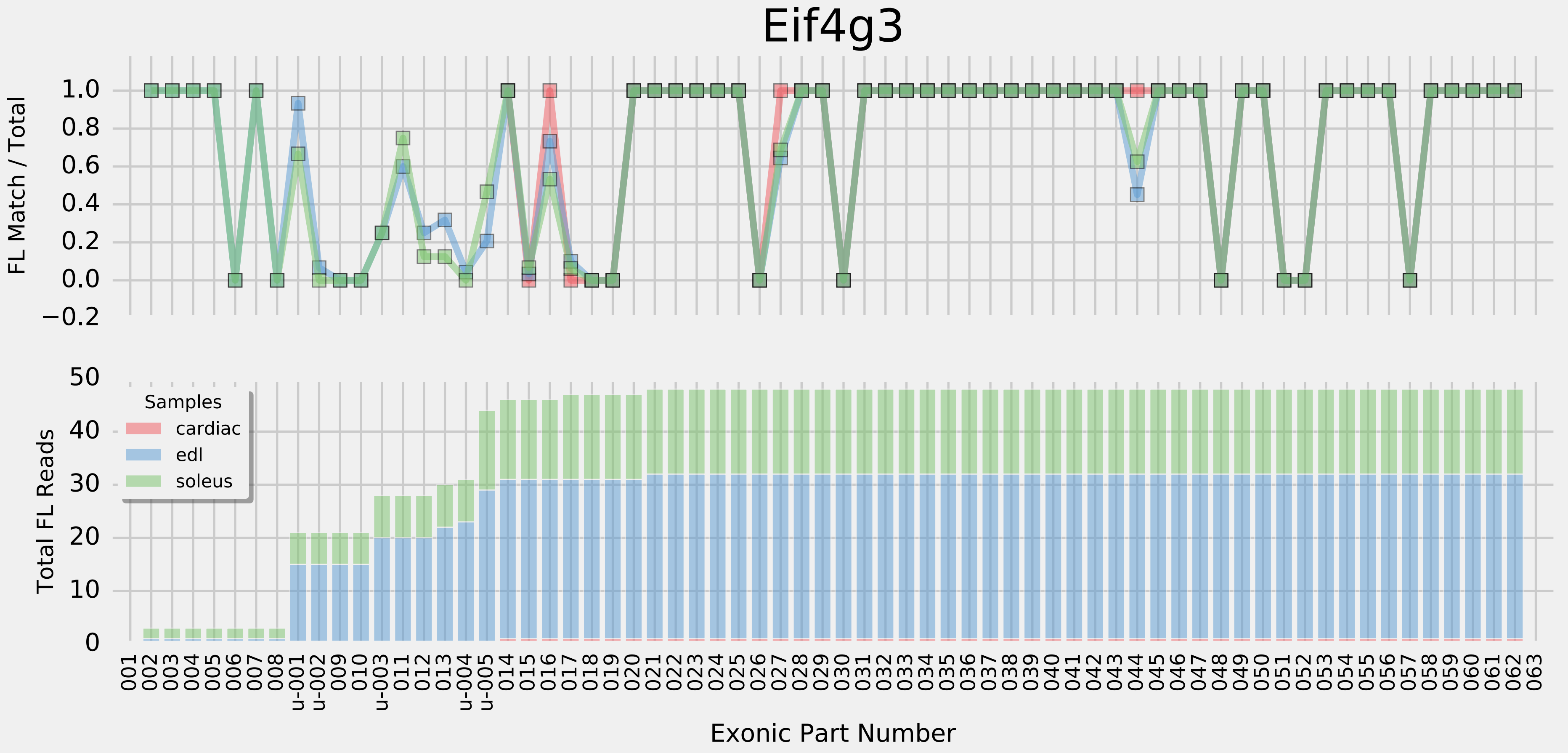

Eif4g3
FL Match / Total
1.0
0.8
0.6
0.4
0.2
0.0
−0.2
Total FL Reads
50
40
30
20
10
0
Samples
cardiac
edl
soleus
001 002 003 004 005 006 007 008 u-001 u-002 009 010 u-003 011 012 013 u-004 u-005 014 015 016 017 018 019 020 021 022 023 024 025 026 027 028 029 030 031 032 033 034 035 036 037 038 039 040 041 042 043 044 045 046 047 048 049 050 051 052 053 054 055 056 057 058 059 060 061 062 063
Exonic Part Number

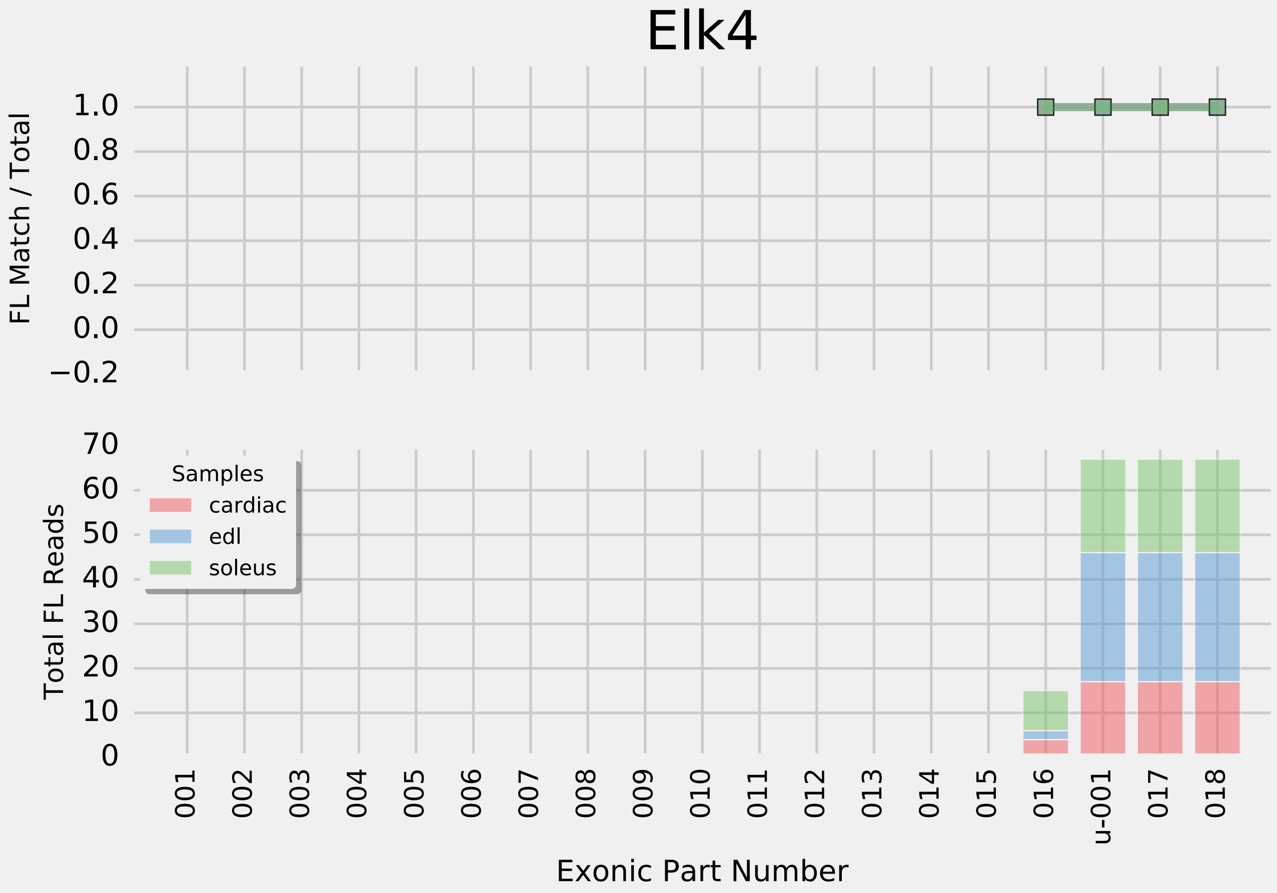
Elk4
FL Match / Total
1.0
0.8
0.6
0.4
0.2
0.0
−0.2
Total FL Reads
70
60
50
40
30
20
10
0
Samples
cardiac
edl
soleus
001
002
003
004
005
006
007
008
009
010
011
012
013
014
015
016
u-001
017
018
Exonic Part Number

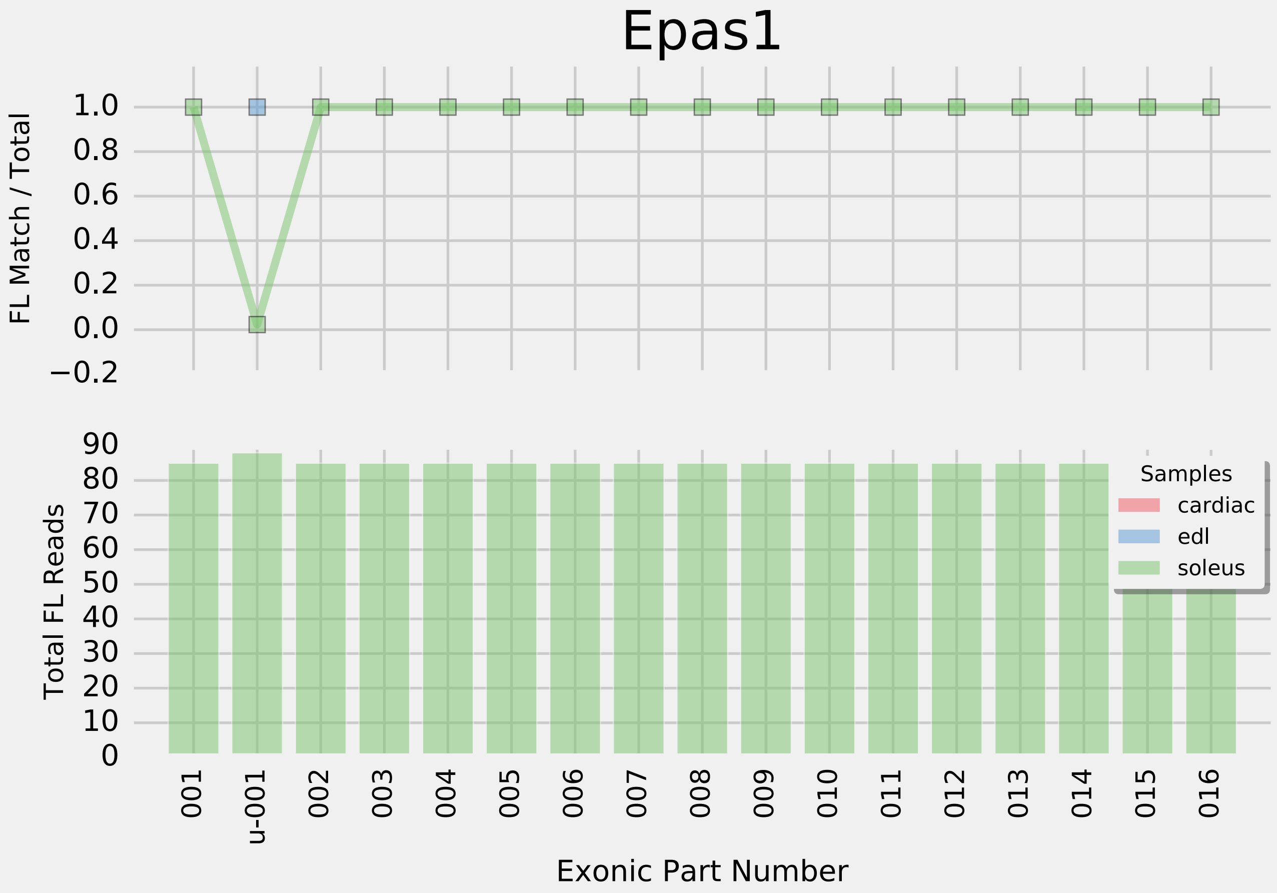

Epas1
FL Match / Total
1.0
0.8
0.6
0.4
0.2
0.0
−0.2
Total FL Reads
90
80
70
60
50
40
30
20
10
0
Samples
cardiac
edl
soleus
001
u-001
002
003
004
005
006
007
008
009
010
011
012
013
014
015
016
Exonic Part Number

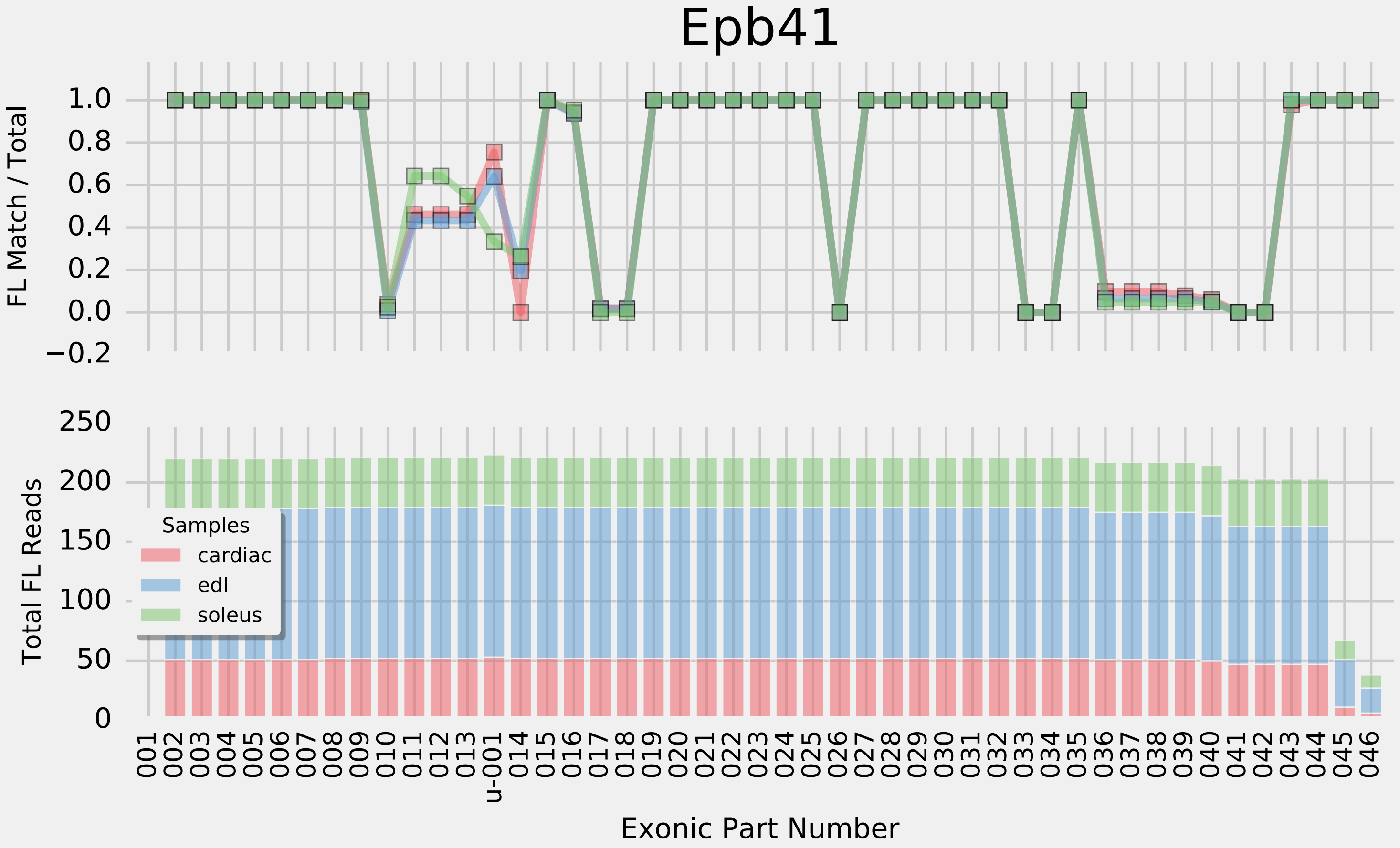

Epb41
FL Match / Total
1.0
0.8
0.6
0.4
0.2
0.0
−0.2
250
200
150
100
50
0
Total FL Reads
Samples
cardiac
edl
soleus
001
002
003
004
005
006
007
008
009
010
011
012
013
u-001
014
015
016
017
018
019
020
021
022
023
024
025
026
027
028
029
030
031
032
033
034
035
036
037
038
039
040
041
042
043
044
045
046
Exonic Part Number

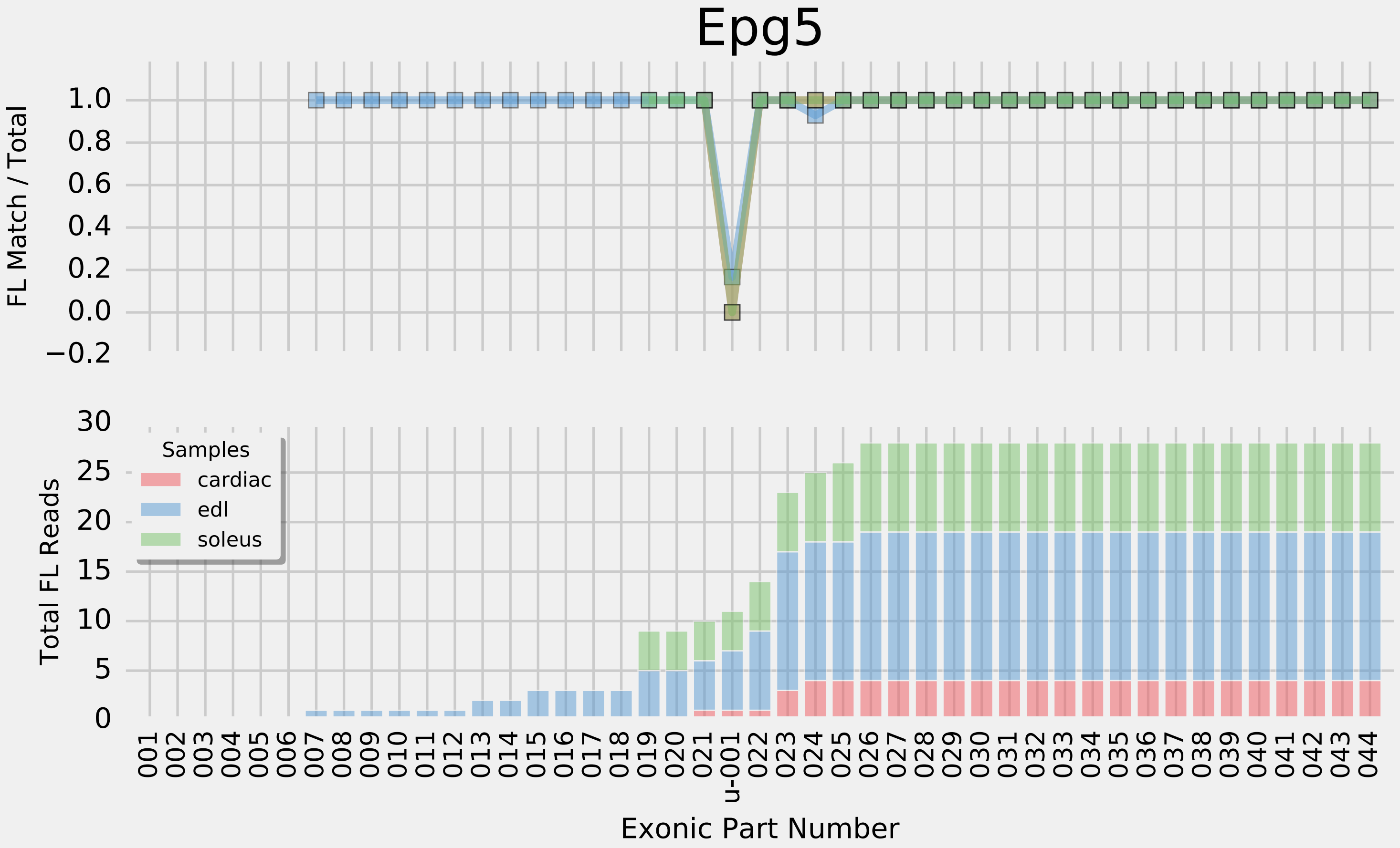

Epg5
FL Match / Total
1.0
0.8
0.6
0.4
0.2
0.0
−0.2
Total FL Reads
30
25
20
15
10
5
0
Samples
cardiac
edl
soleus
001
002
003
004
005
006
007
008
009
010
011
012
013
014
015
016
017
018
019
020
021
u-001
022
023
024
025
026
027
028
029
030
031
032
033
034
035
036
037
038
039
040
041
042
043
044
Exonic Part Number

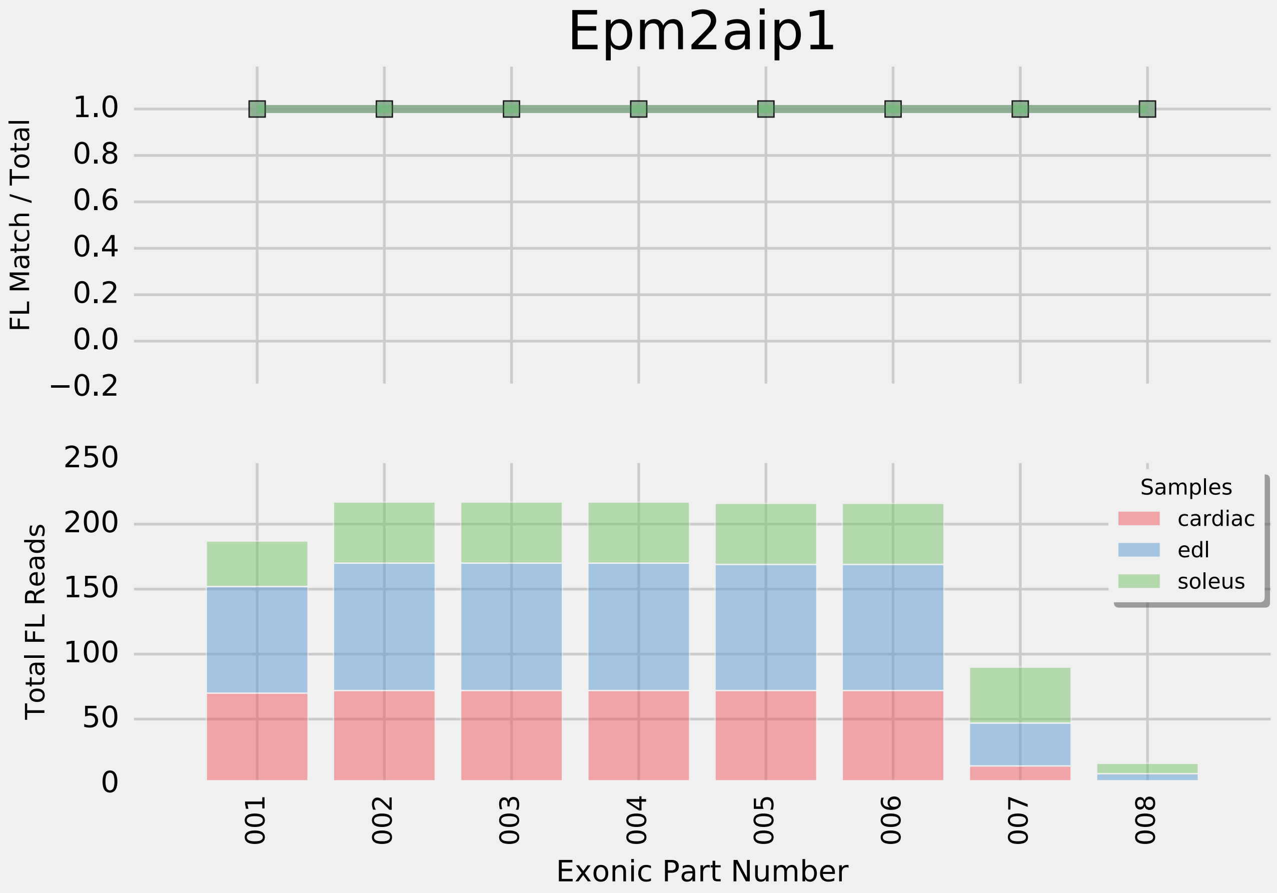
Epm2aip1
FL Match / Total
1.0
0.8
0.6
0.4
0.2
0.0
−0.2
Total FL Reads
250
200
150
100
50
0
Samples
cardiac
edl
soleus
001
002
003
004
005
006
007
008
Exonic Part Number

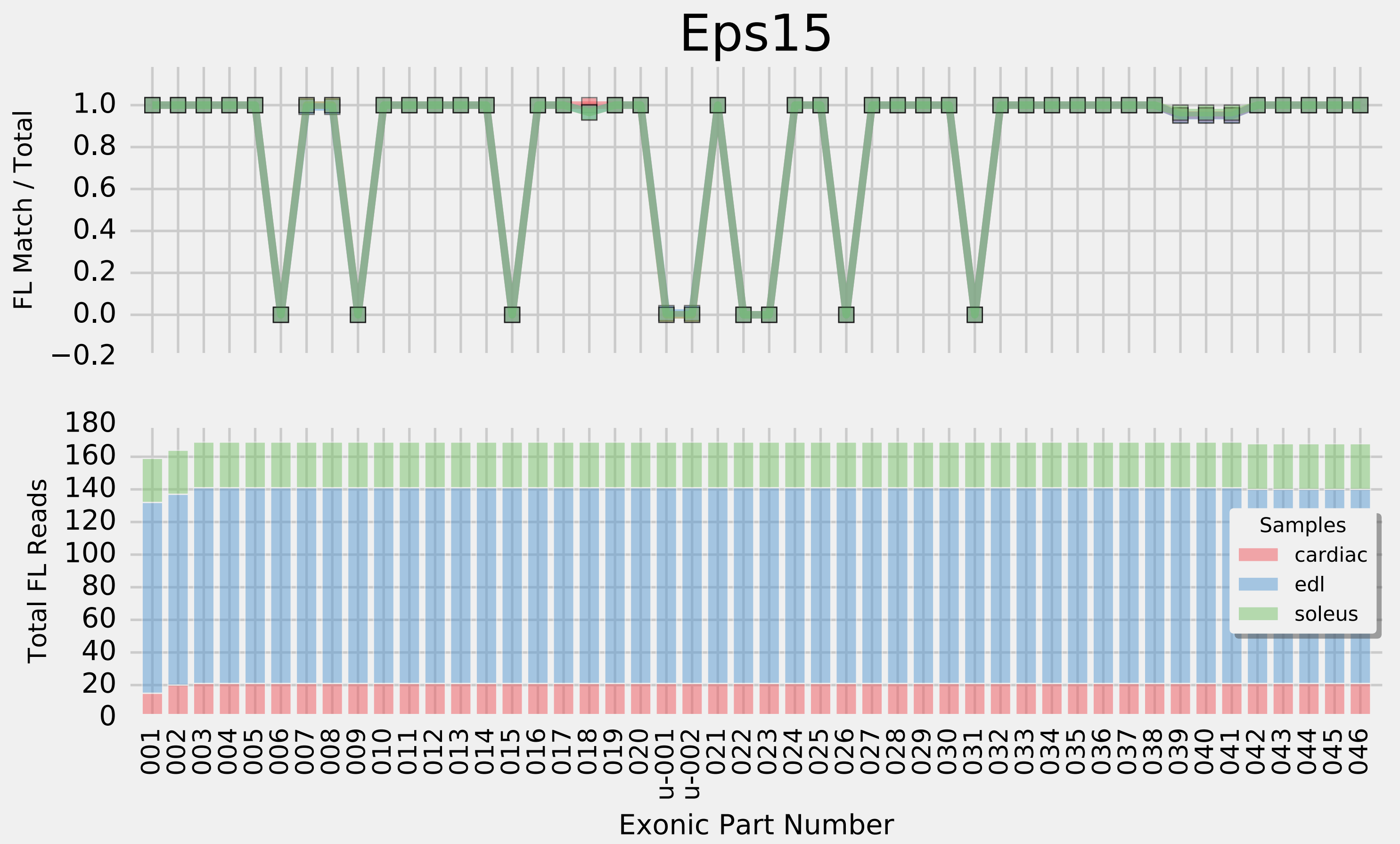
Eps15
FL Match / Total
1.0
0.8
0.6
0.4
0.2
0.0
−0.2
Total FL Reads
180
160
140
120
100
80
60
40
20
0
Samples
cardiac
edl
soleus
001 002 003 004 005 006 007 008 009 010 011 012 013 014 015 016 017 018 019 020 u-001 u-002 021 022 023 024 025 026 027 028 029 030 031 032 033 034 035 036 037 038 039 040 041 042 043 044 045 046
Exonic Part Number

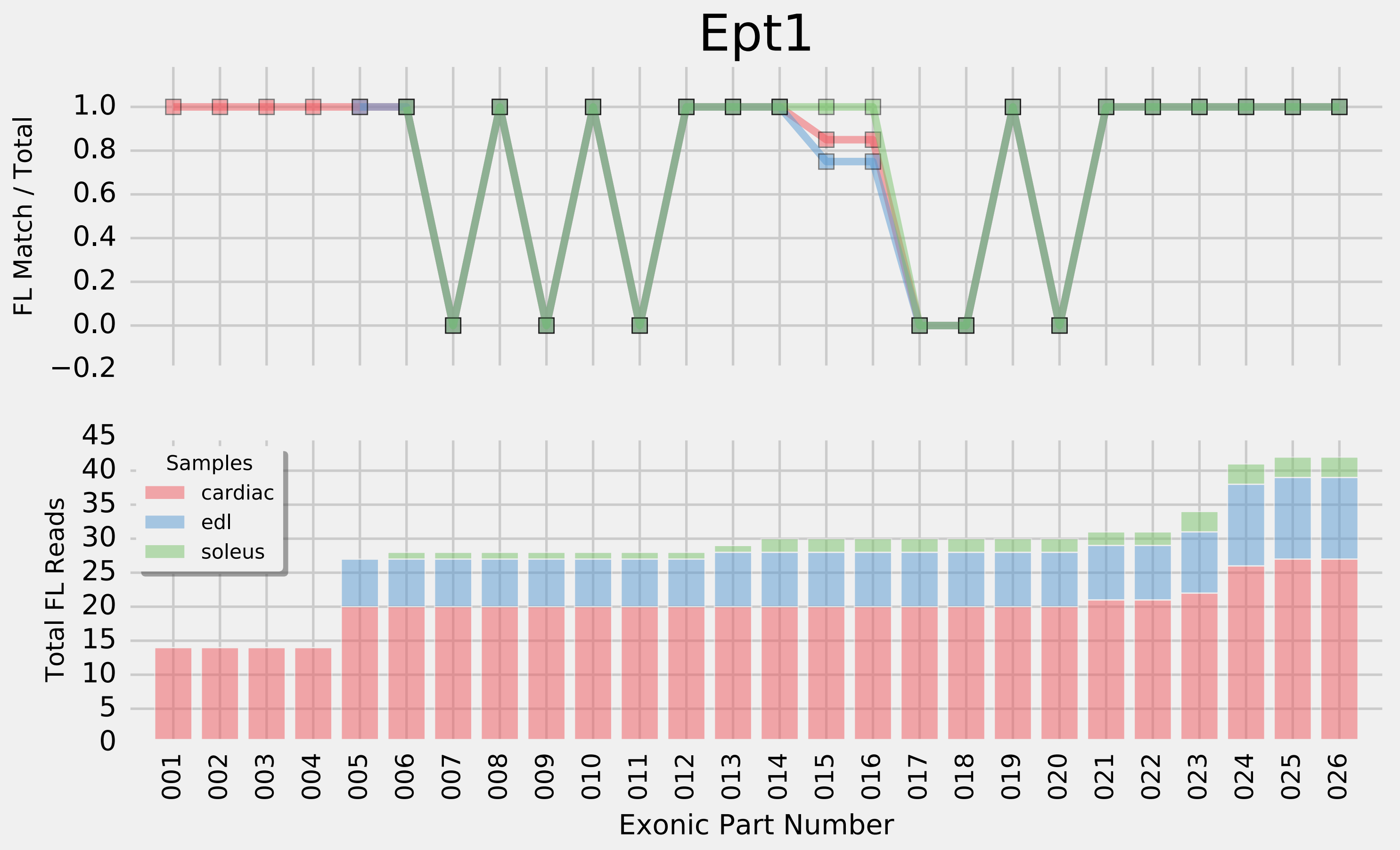

Ept1
FL Match / Total
1.0
0.8
0.6
0.4
0.2
0.0
−0.2
Total FL Reads
45
40
35
30
25
20
15
10
5
0
Samples
cardiac
edl
soleus
001
002
003
004
005
006
007
008
009
010
011
012
013
014
015
016
017
018
019
020
021
022
023
024
025
026
Exonic Part Number

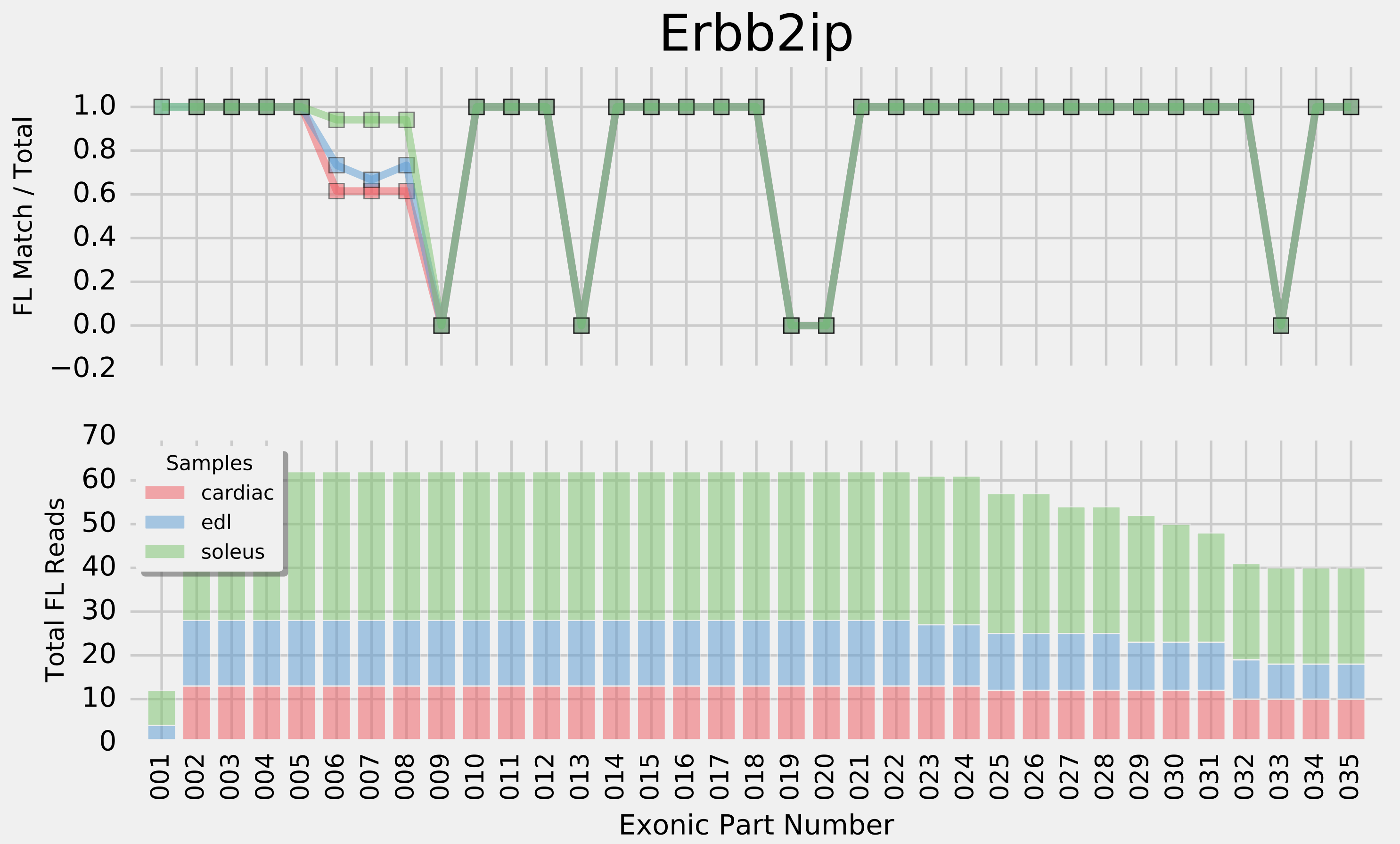

Erbb2ip
FL Match / Total
1.0
0.8
0.6
0.4
0.2
0.0
−0.2
Total FL Reads
70
60
50
40
30
20
10
0
Samples
cardiac
edl
soleus
001 002 003 004 005 006 007 008 009 010 011 012 013 014 015 016 017 018 019 020 021 022 023 024 025 026 027 028 029 030 031 032 033 034 035
Exonic Part Number

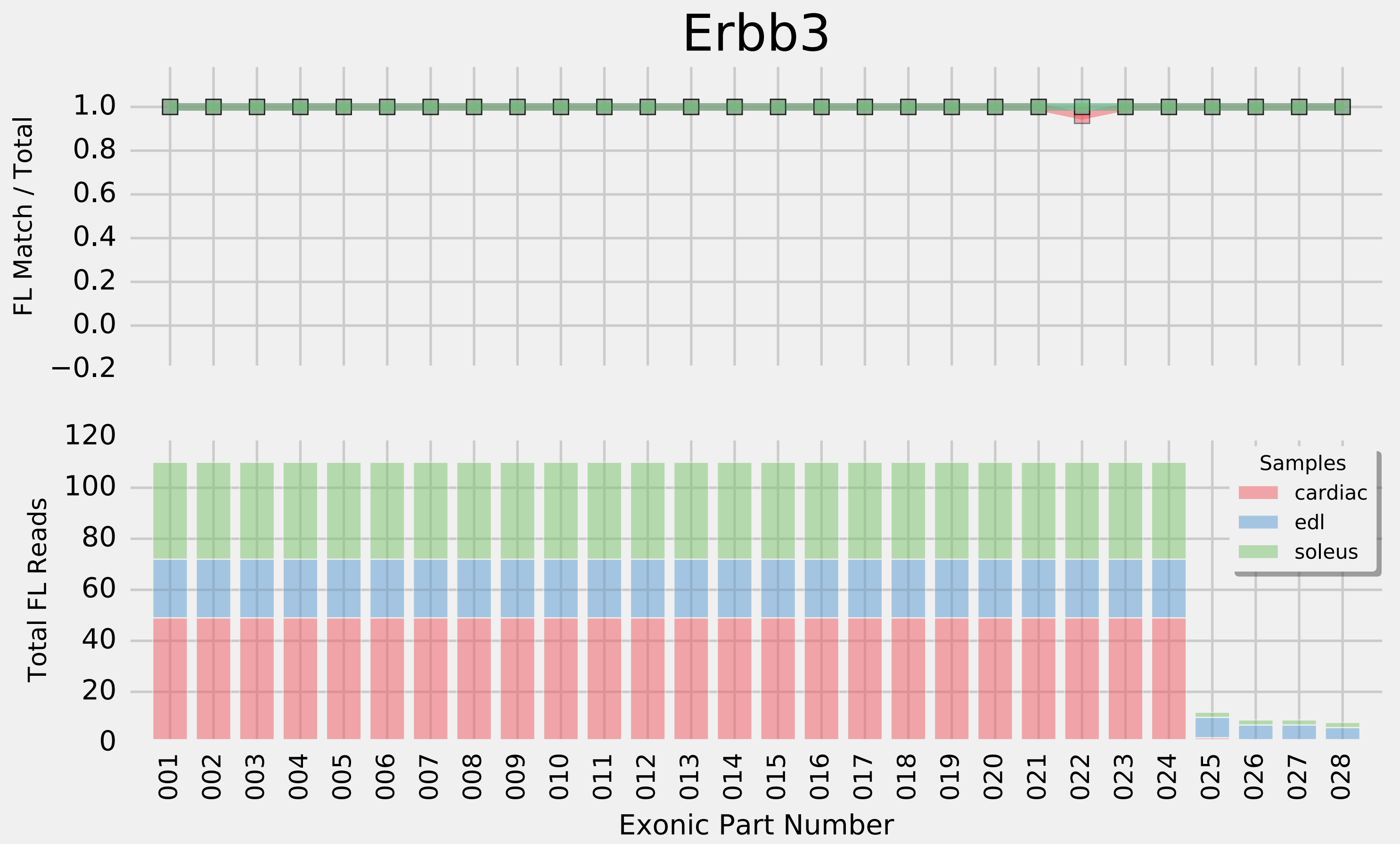
Erbb3
FL Match / Total
1.0
0.8
0.6
0.4
0.2
0.0
−0.2
Total FL Reads
120
100
80
60
40
20
0
Samples
cardiac
edl
soleus
001
002
003
004
005
006
007
008
009
010
011
012
013
014
015
016
017
018
019
020
021
022
023
024
025
026
027
028
Exonic Part Number

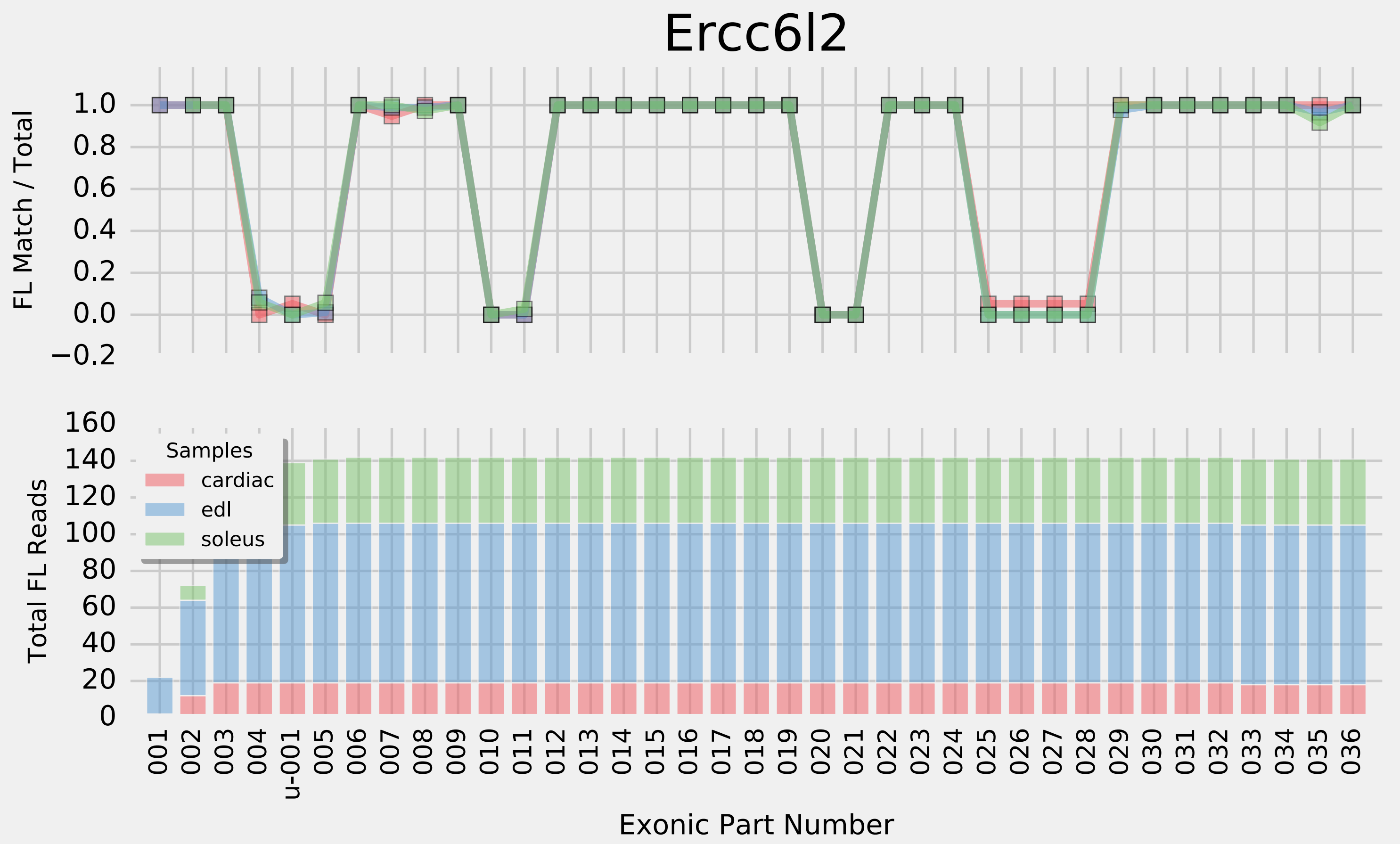

Ercc6l2
FL Match / Total
1.0
0.8
0.6
0.4
0.2
0.0
−0.2
Total FL Reads
160
140
120
100
80
60
40
20
0
Samples
cardiac
edl
soleus
001
002
003
004
u-001
005
006
007
008
009
010
011
012
013
014
015
016
017
018
019
020
021
022
023
024
025
026
027
028
029
030
031
032
033
034
035
036
Exonic Part Number

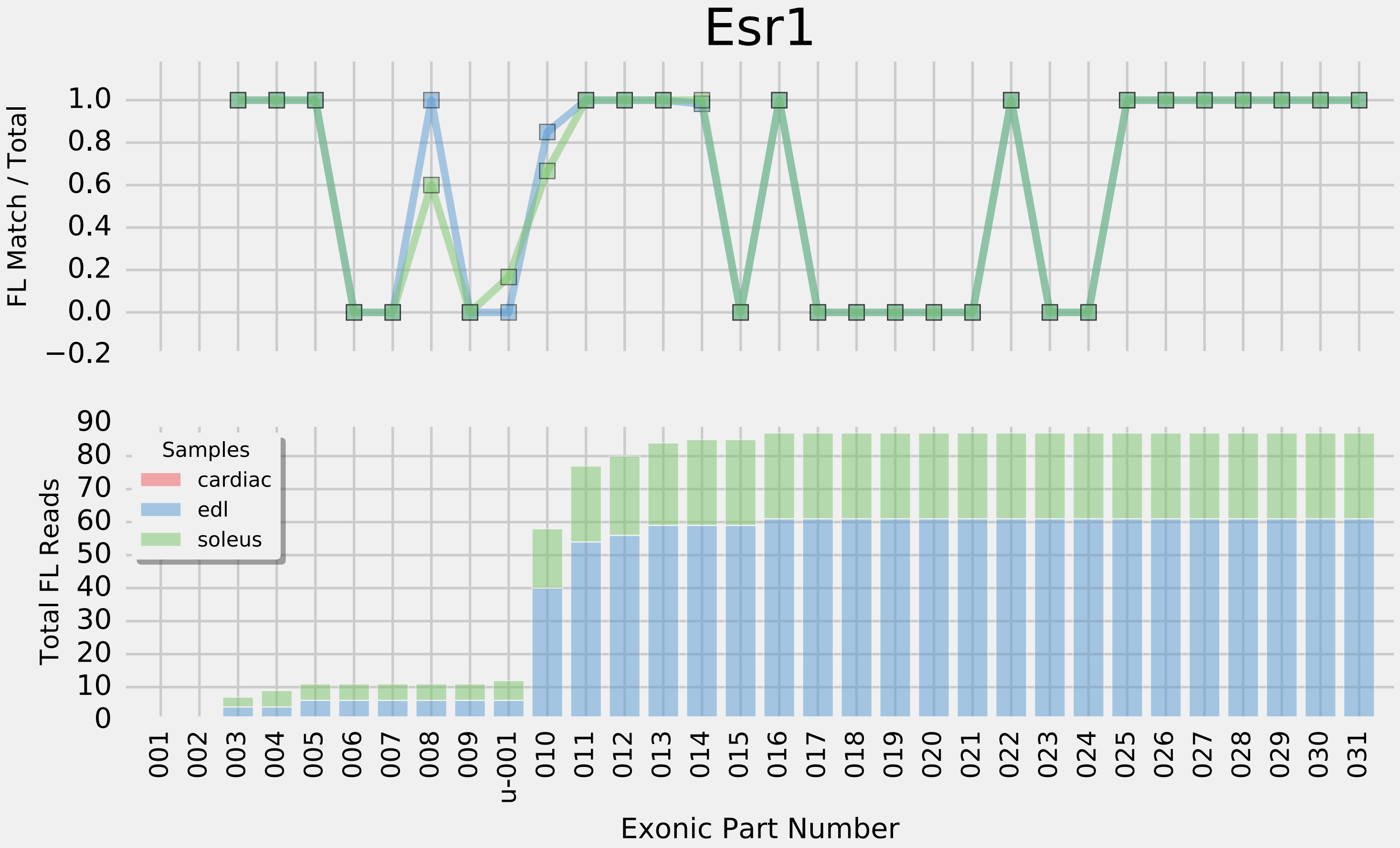

Esr1
FL Match / Total
1.0
0.8
0.6
0.4
0.2
0.0
−0.2
Total FL Reads
90
80
70
60
50
40
30
20
10
0
Samples
cardiac
edl
soleus
001
002
003
004
005
006
007
008
009
u-001
010
011
012
013
014
015
016
017
018
019
020
021
022
023
024
025
026
027
028
029
030
031
Exonic Part Number

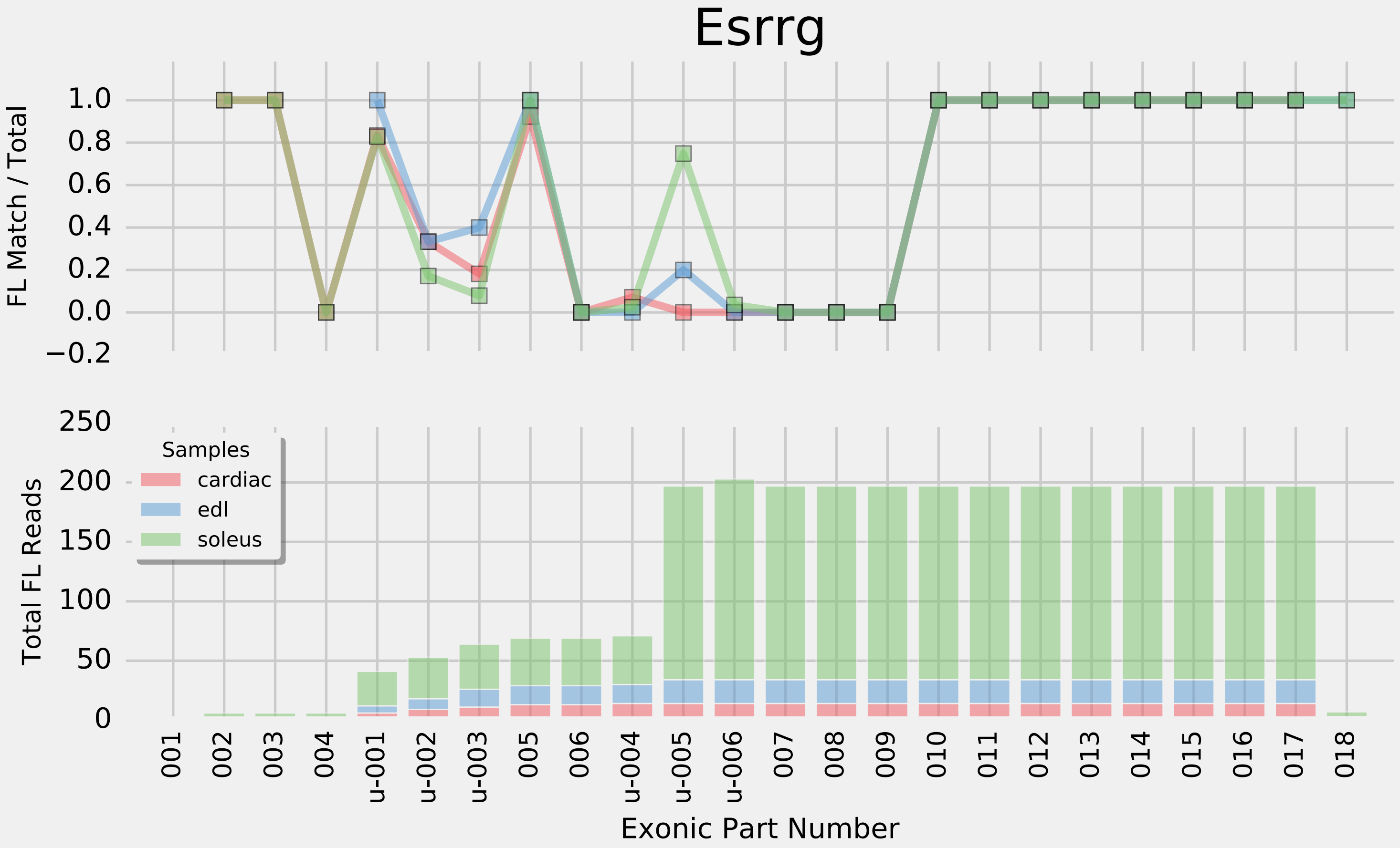

Esrrg
FL Match / Total
1.0
0.8
0.6
0.4
0.2
0.0
−0.2
Total FL Reads
250
200
150
100
50
0
Samples
cardiac
edl
soleus
001
002
003
004
u-001
u-002
u-003
005
006
u-004
u-005
u-006
007
008
009
010
011
012
013
014
015
016
017
018
Exonic Part Number

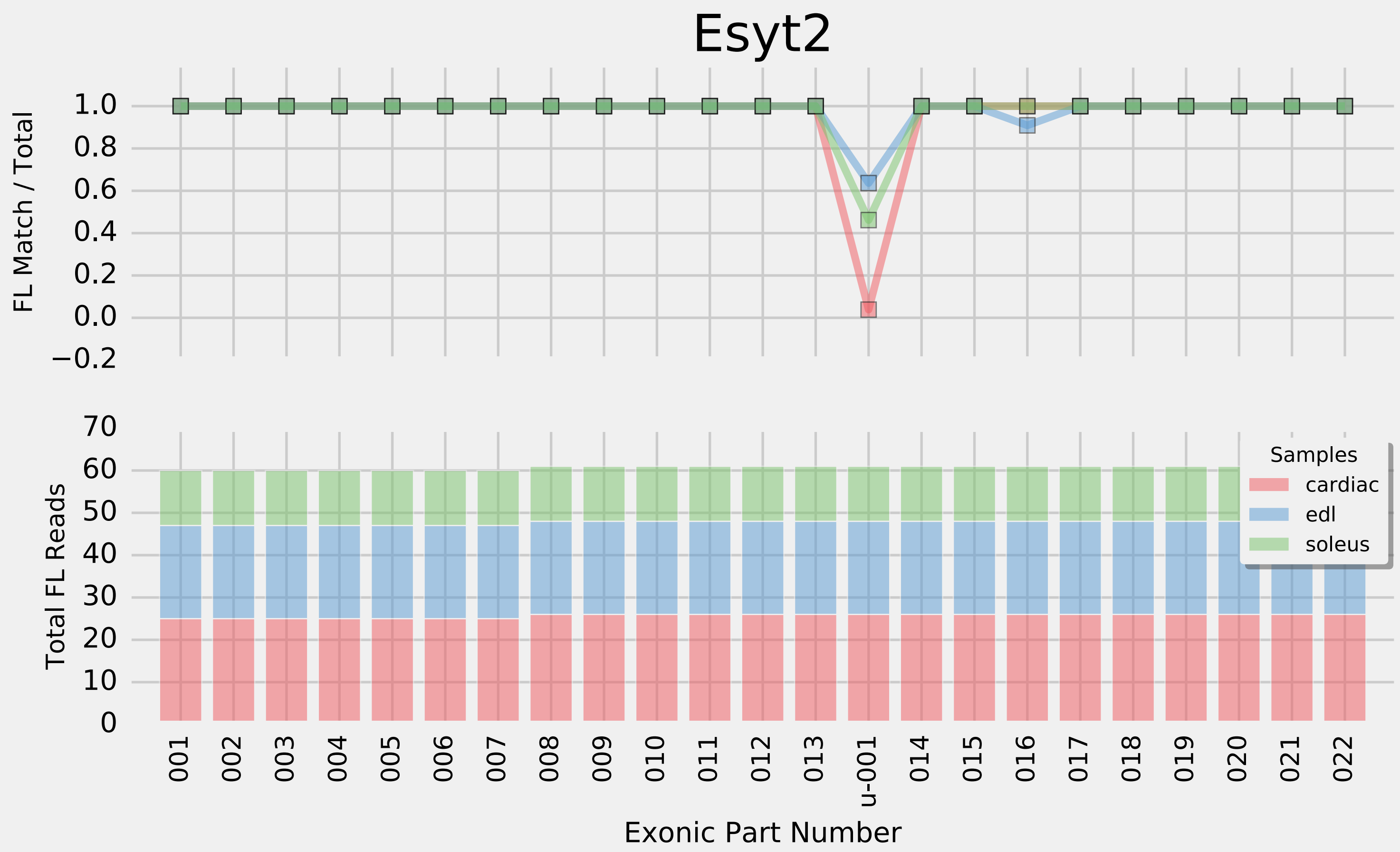

Esyt2
FL Match / Total
1.0
0.8
0.6
0.4
0.2
0.0
−0.2
Total FL Reads
70
60
50
40
30
20
10
0
Samples
cardiac
edl
soleus
001
002
003
004
005
006
007
008
009
010
011
012
013
u-001
014
015
016
017
018
019
020
021
022
Exonic Part Number

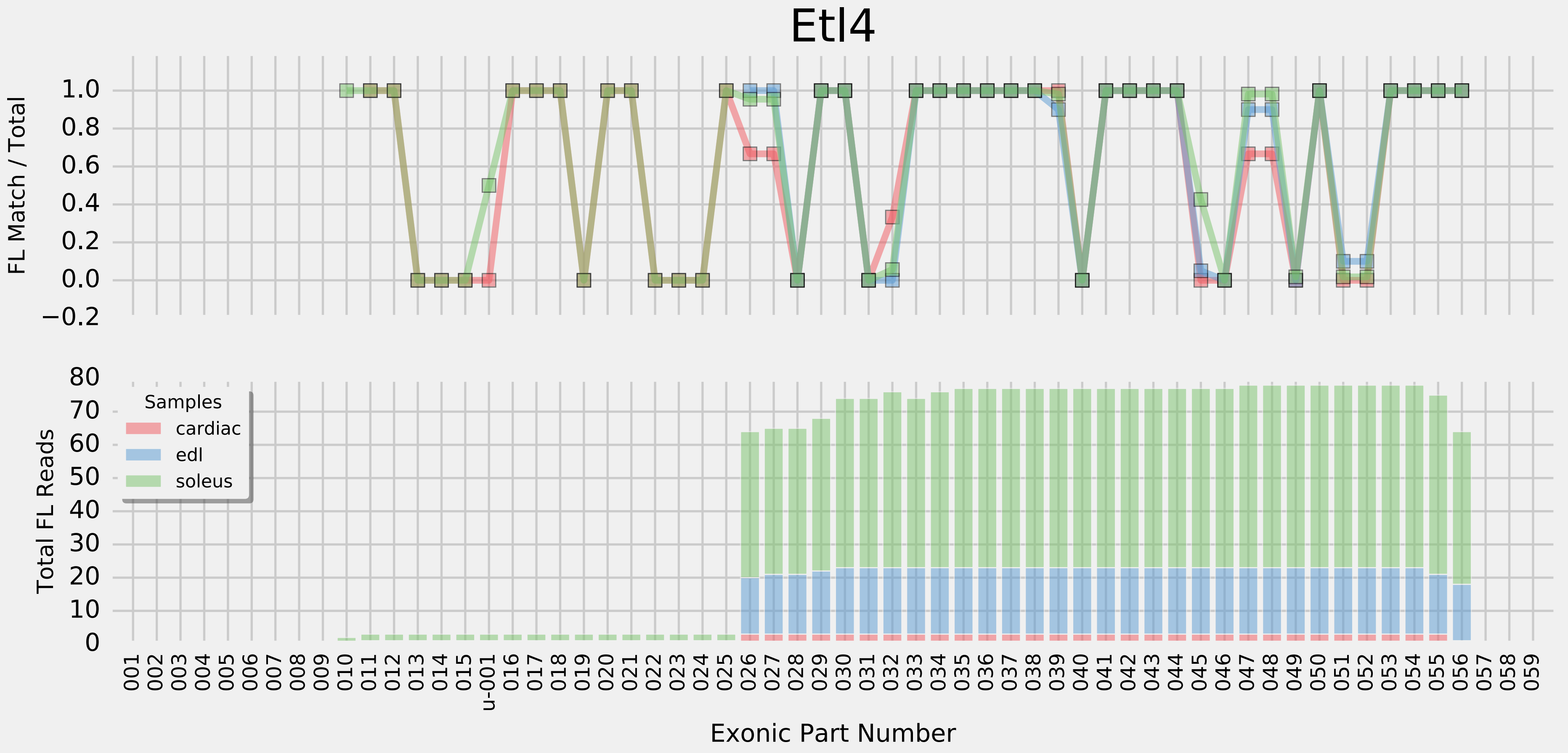

Etl4
FL Match / Total
1.0
0.8
0.6
0.4
0.2
0.0
−0.2
Total FL Reads
80
70
60
50
40
30
20
10
0
Samples
cardiac
edl
soleus
001 002 003 004 005 006 007 008 009 010 011 012 013 014 015 u-001 016 017 018 019 020 021 022 023 024 025 026 027 028 029 030 031 032 033 034 035 036 037 038 039 040 041 042 043 044 045 046 047 048 049 050 051 052 053 054 055 056 057 058 059
Exonic Part Number

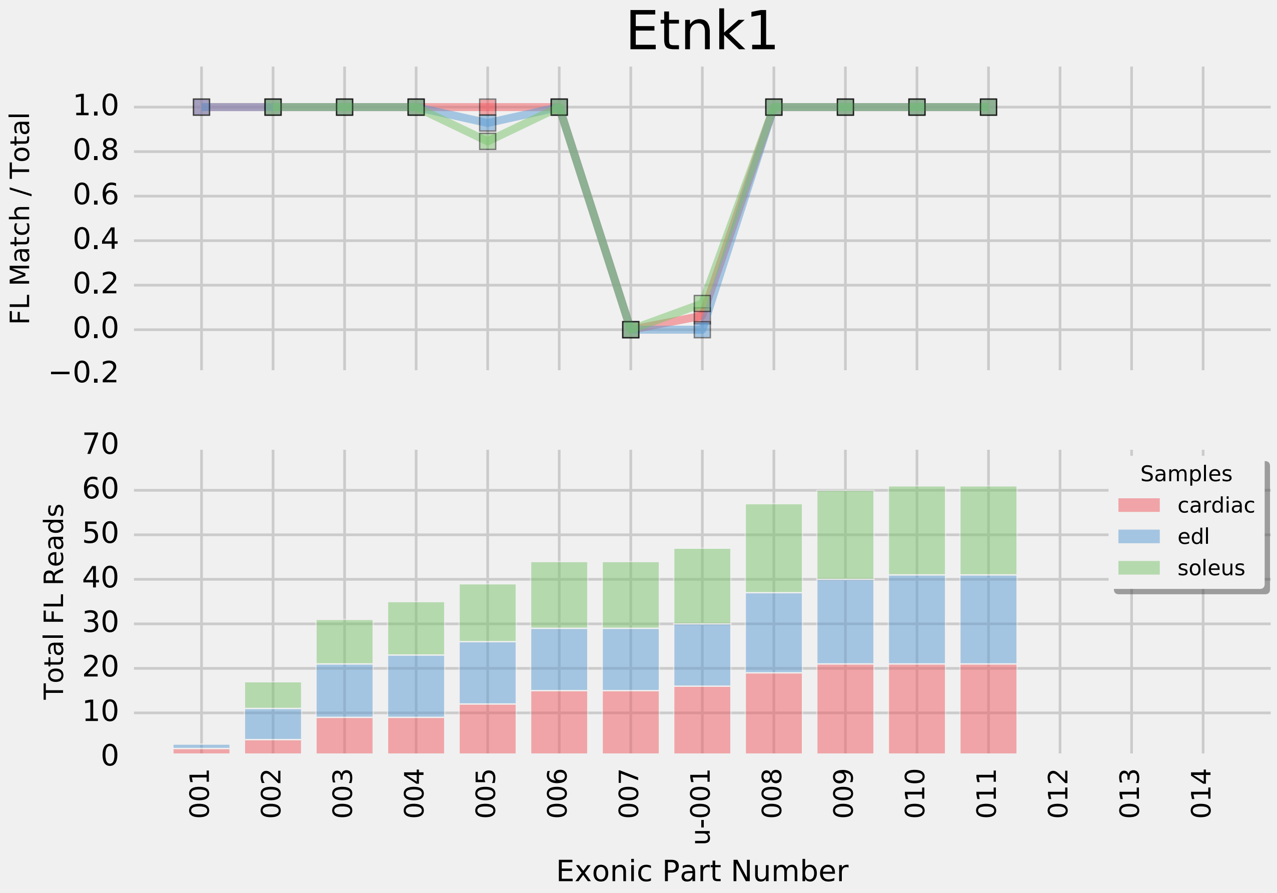

Etnk1
FL Match / Total
1.0
0.8
0.6
0.4
0.2
0.0
−0.2
70
60
50
40
30
20
10
0
Total FL Reads
Samples
cardiac
edl
soleus
001
002
003
004
005
006
007
u-001
008
009
010
011
012
013
014
Exonic Part Number

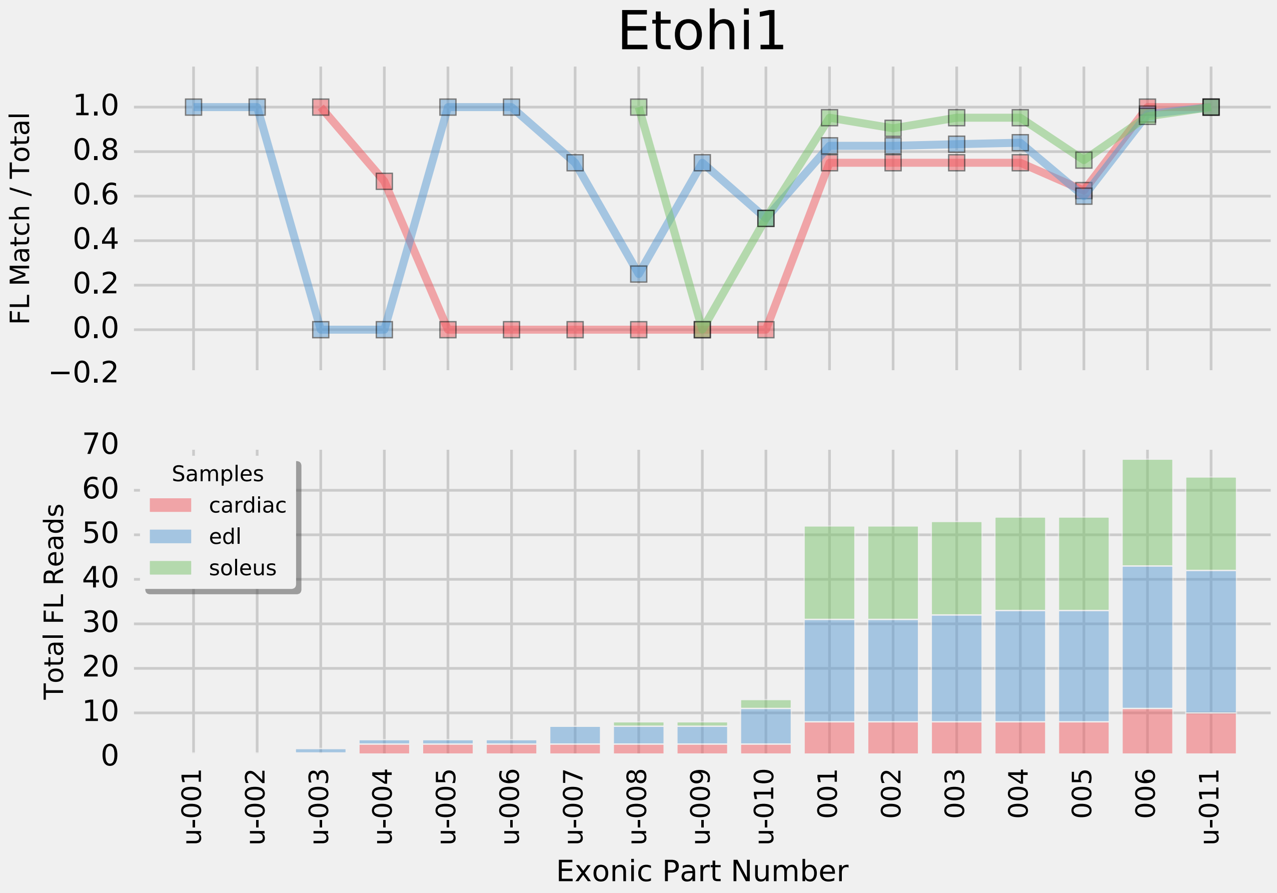

Etohi1
FL Match / Total
1.0
0.8
0.6
0.4
0.2
0.0
−0.2
Total FL Reads
70
60
50
40
30
20
10
0
Samples
cardiac
edl
soleus
u-001
u-002
u-003
u-004
u-005
u-006
u-007
u-008
u-009
u-010
001
002
003
004
005
006
u-011
Exonic Part Number

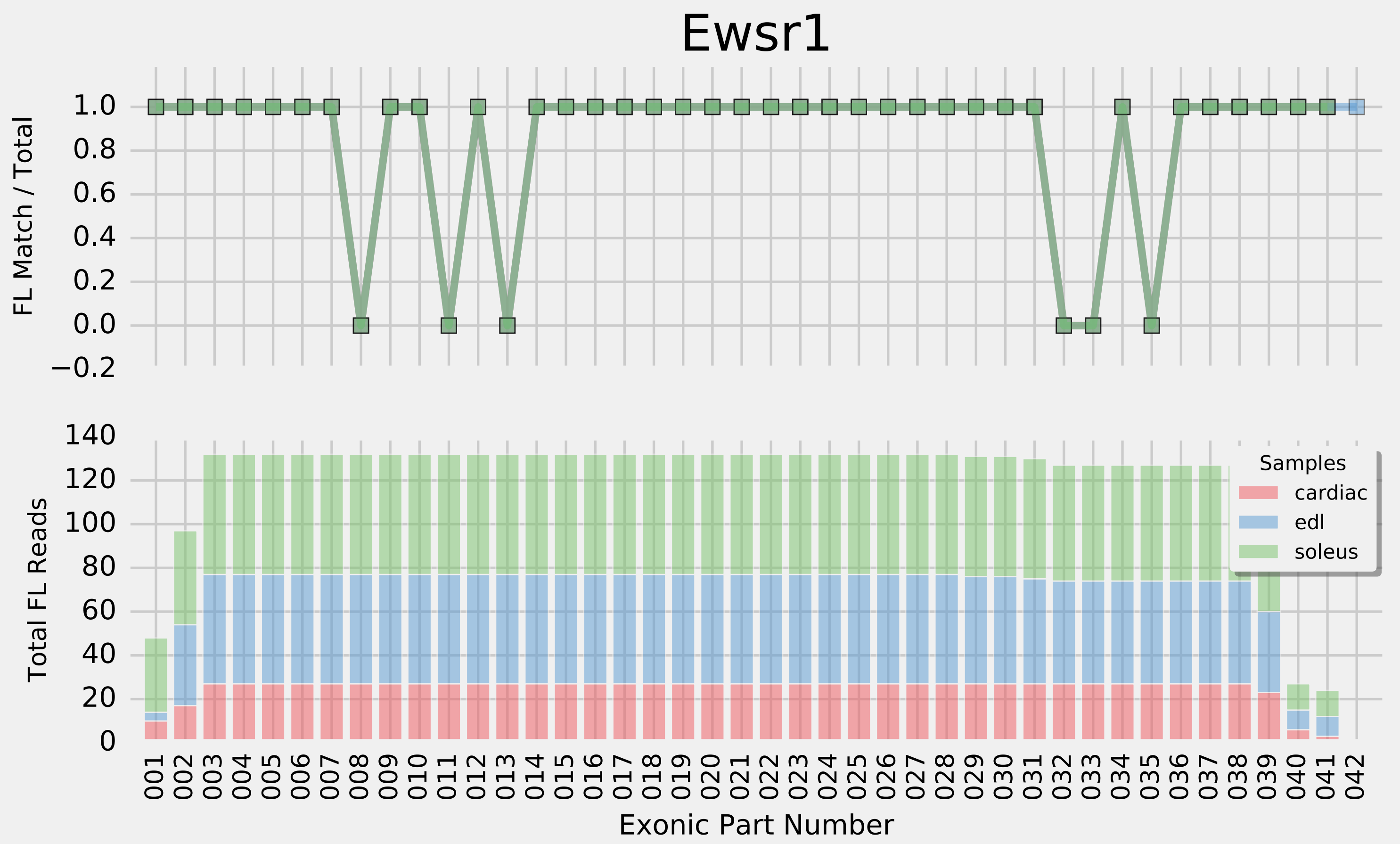

Ewsr1
FL Match / Total
1.0
0.8
0.6
0.4
0.2
0.0
−0.2
Total FL Reads
140
120
100
80
60
40
20
0
Samples
cardiac
edl
soleus
001
002
003
004
005
006
007
008
009
010
011
012
013
014
015
016
017
018
019
020
021
022
023
024
025
026
027
028
029
030
031
032
033
034
035
036
037
038
039
040
041
042
Exonic Part Number

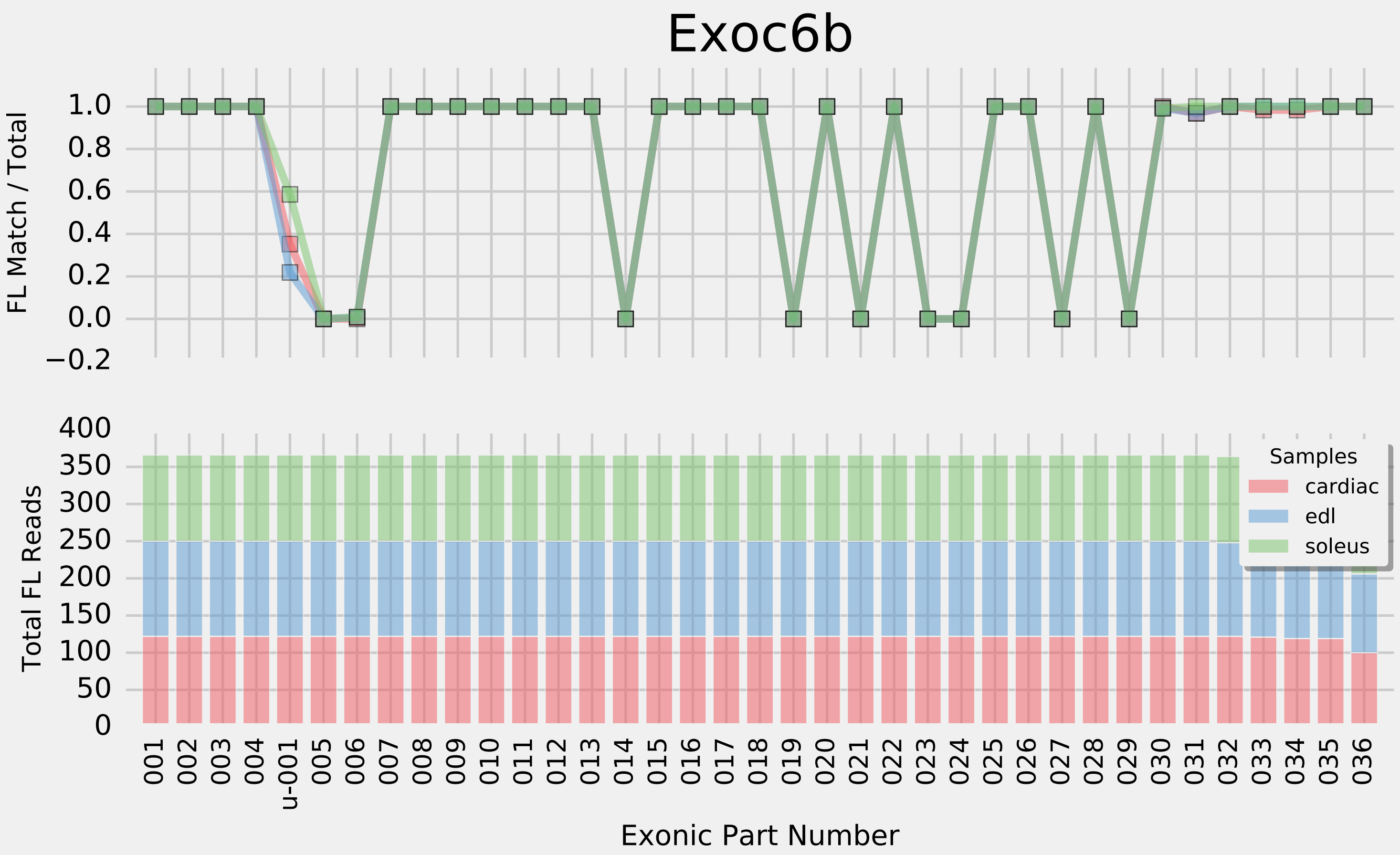

Exoc6b
FL Match / Total
1.0
0.8
0.6
0.4
0.2
0.0
−0.2
Total FL Reads
400
350
300
250
200
150
100
50
0
001
002
003
004
u-001
005
006
007
008
009
010
011
012
013
014
015
016
017
018
019
020
021
022
023
024
025
026
027
028
029
030
031
032
033
034
035
036
Exonic Part Number
Samples
cardiac
edl
soleus

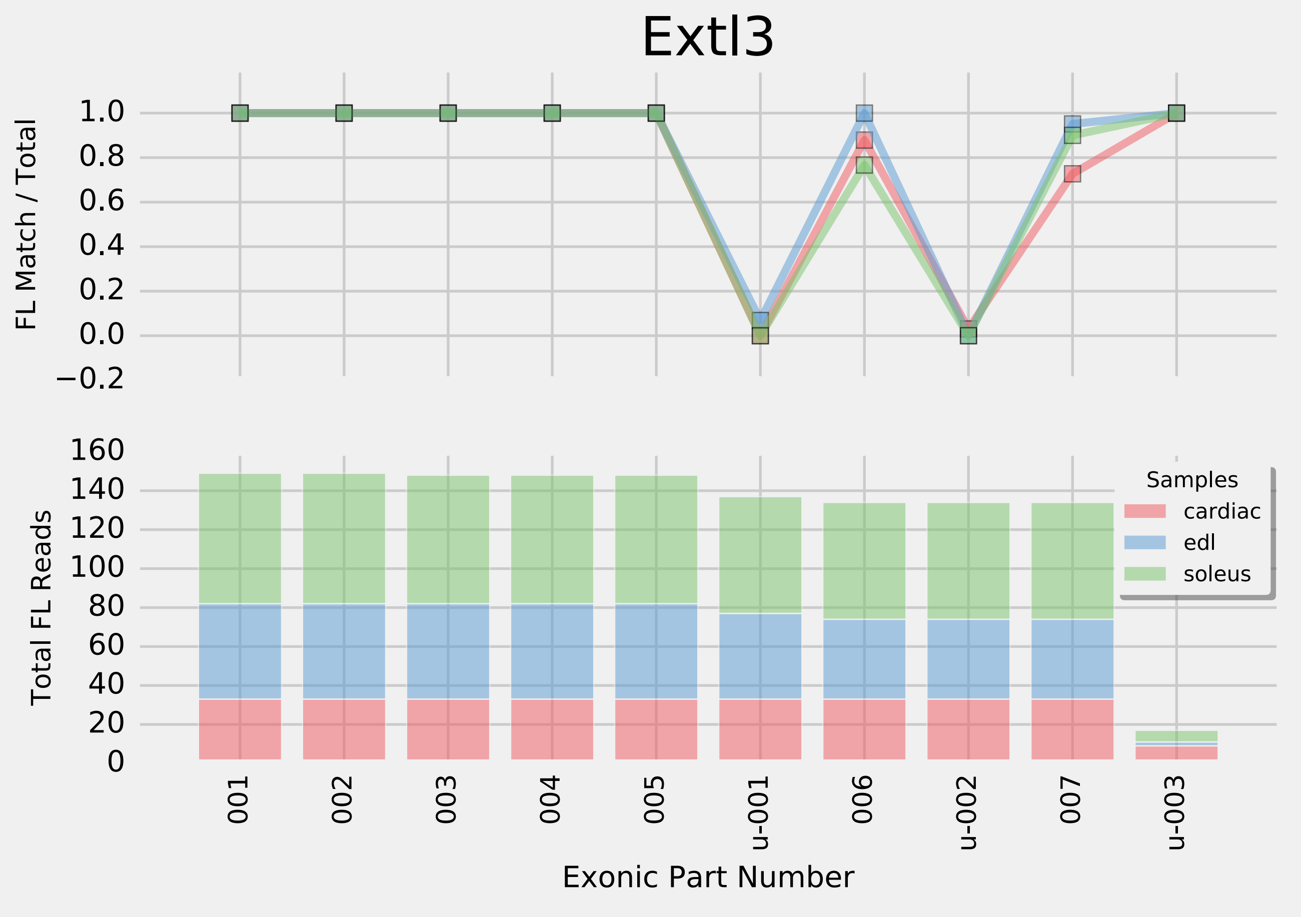

Extl3
FL Match / Total
1.0
0.8
0.6
0.4
0.2
0.0
−0.2
Total FL Reads
160
140
120
100
80
60
40
20
0
Samples
cardiac
edl
soleus
001
002
003
004
005
u-001
006
u-002
007
u-003
Exonic Part Number

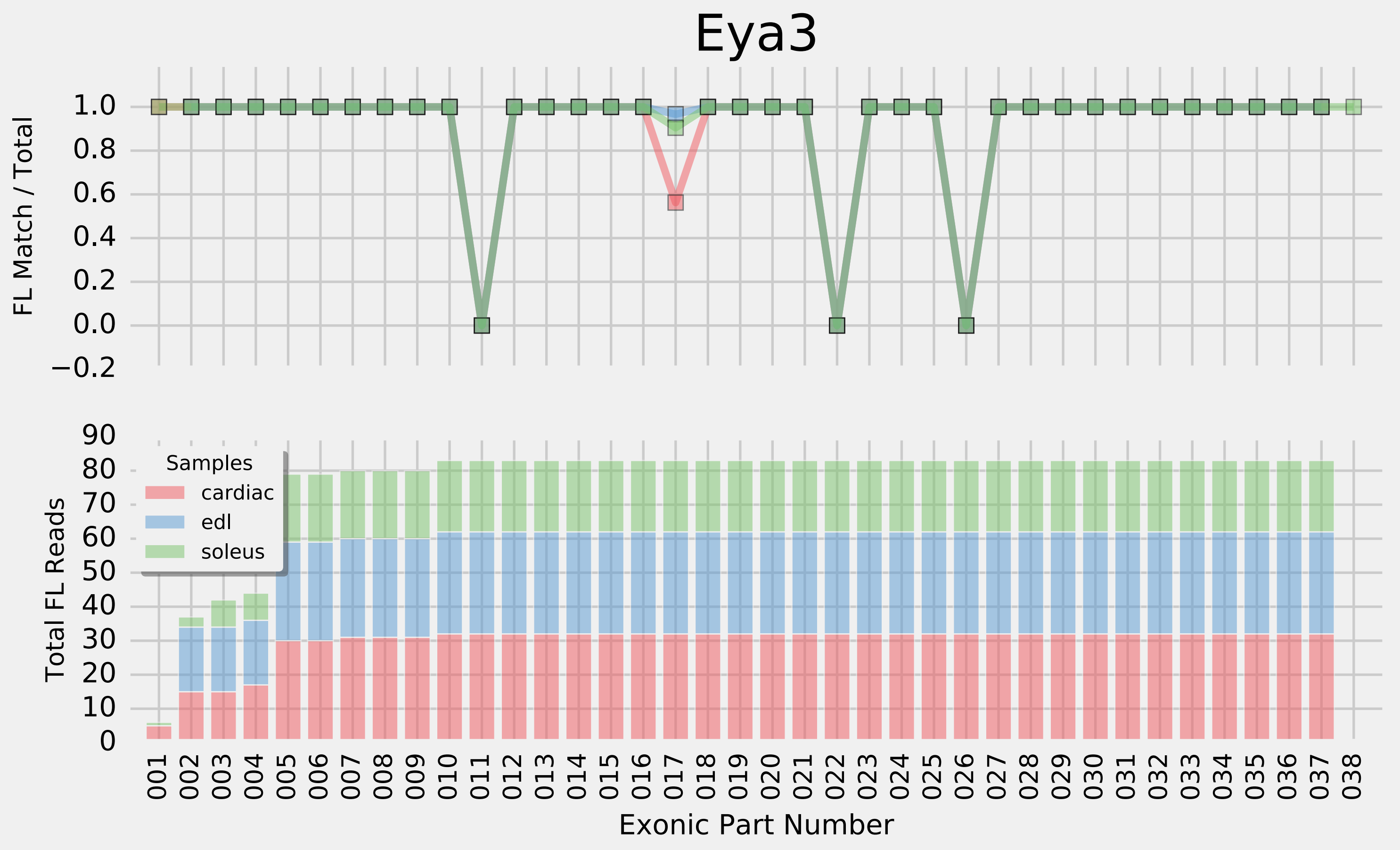

Eya3
FL Match / Total
1.0
0.8
0.6
0.4
0.2
0.0
−0.2
Total FL Reads
90
80
70
60
50
40
30
20
10
0
Samples
cardiac
edl
soleus
001 002 003 004 005 006 007 008 009 010 011 012 013 014 015 016 017 018 019 020 021 022 023 024 025 026 027 028 029 030 031 032 033 034 035 036 037 038
Exonic Part Number

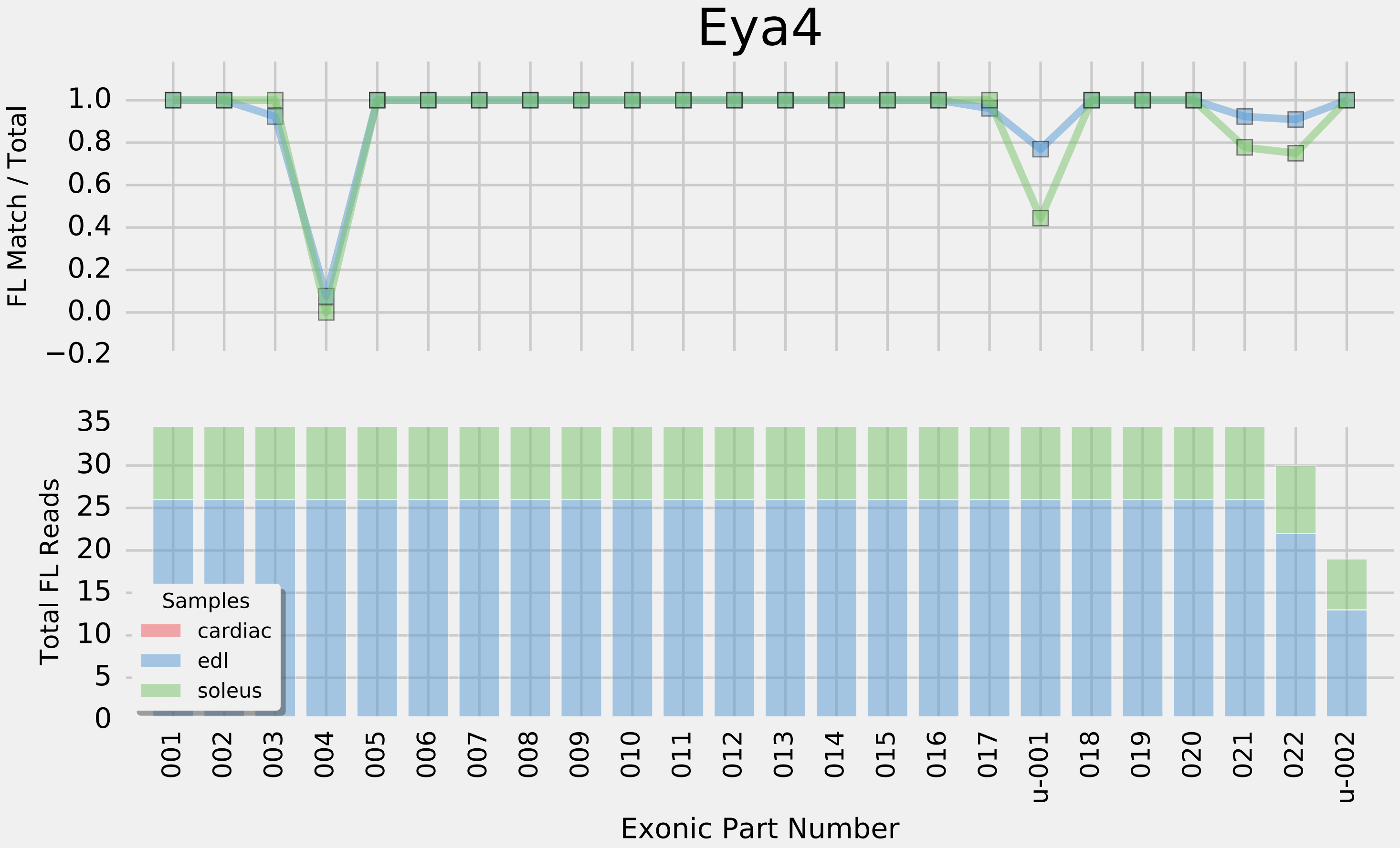

Eya4
FL Match / Total
1.0
0.8
0.6
0.4
0.2
0.0
−0.2
Total FL Reads
35
30
25
20
15
10
5
0
Samples
cardiac
edl
soleus
001
002
003
004
005
006
007
008
009
010
011
012
013
014
015
016
017
u-001
018
019
020
021
022
u-002
Exonic Part Number

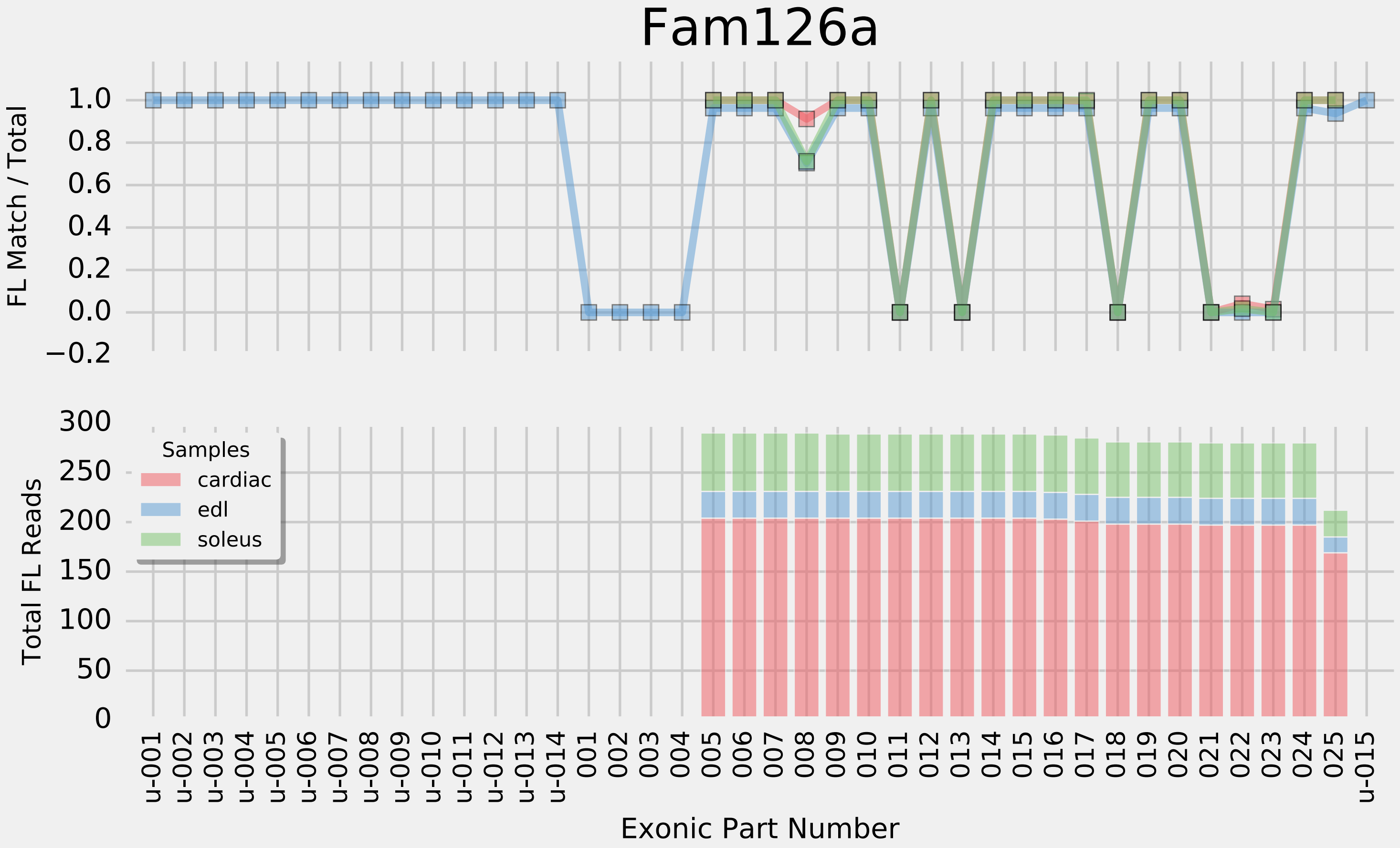

Fam126a
FL Match / Total
1.0
0.8
0.6
0.4
0.2
0.0
−0.2
Total FL Reads
300
250
200
150
100
50
0
Samples
cardiac
edl
soleus
u-001
u-002
u-003
u-004
u-005
u-006
u-007
u-008
u-009
u-010
u-011
u-012
u-013
u-014
001
002
003
004
005
006
007
008
009
010
011
012
013
014
015
016
017
018
019
020
021
022
023
024
025
u-015
Exonic Part Number

Fam129a

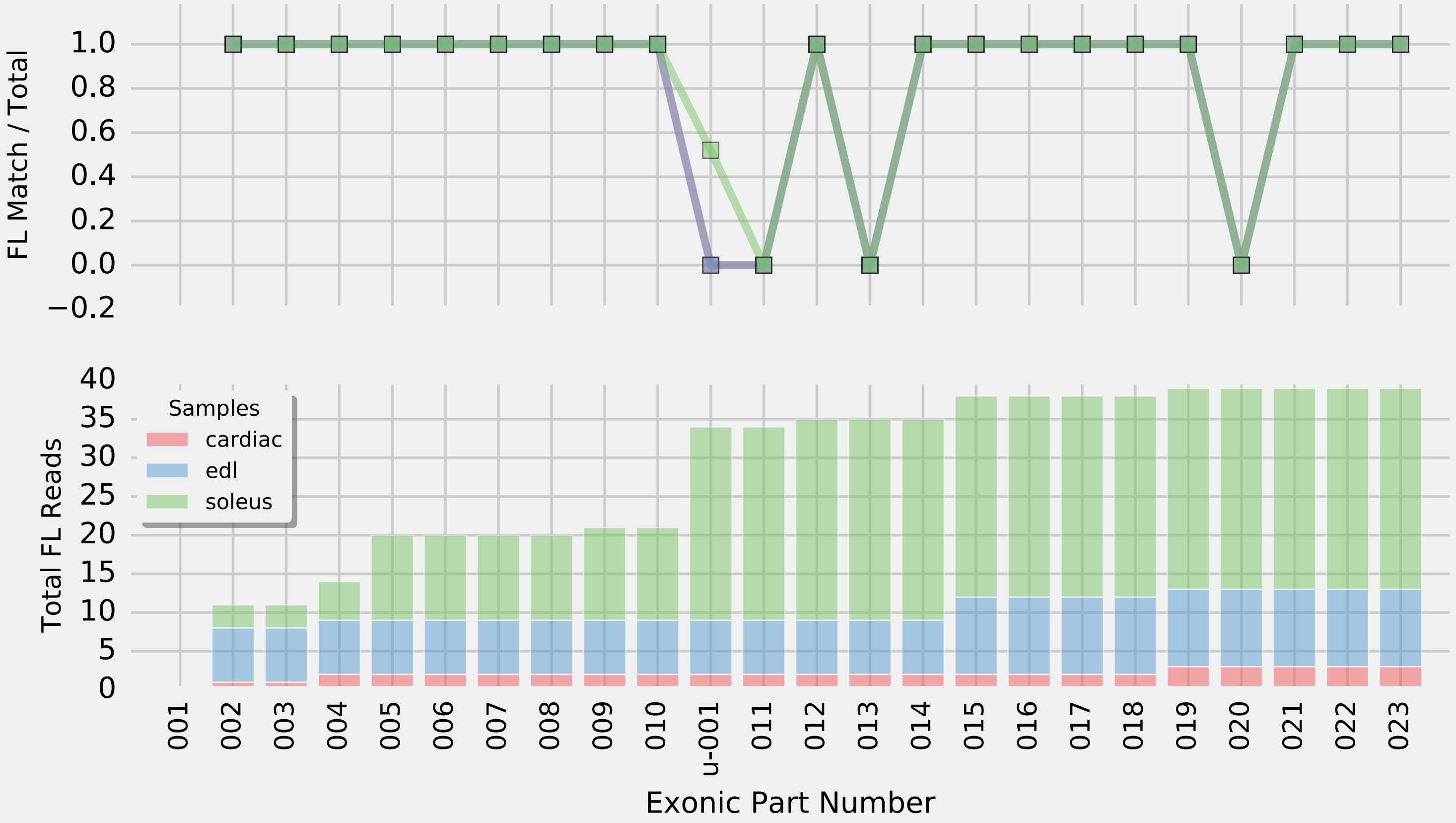

FL Match / Total
1.0
0.8
0.6
0.4
0.2
0.0
−0.2
Total FL Reads
40
35
30
25
20
15
10
5
0
Samples
cardiac
edl
soleus
001
002
003
004
005
006
007
008
009
010
u-001
011
012
013
014
015
016
017
018
019
020
021
022
023
Exonic Part Number

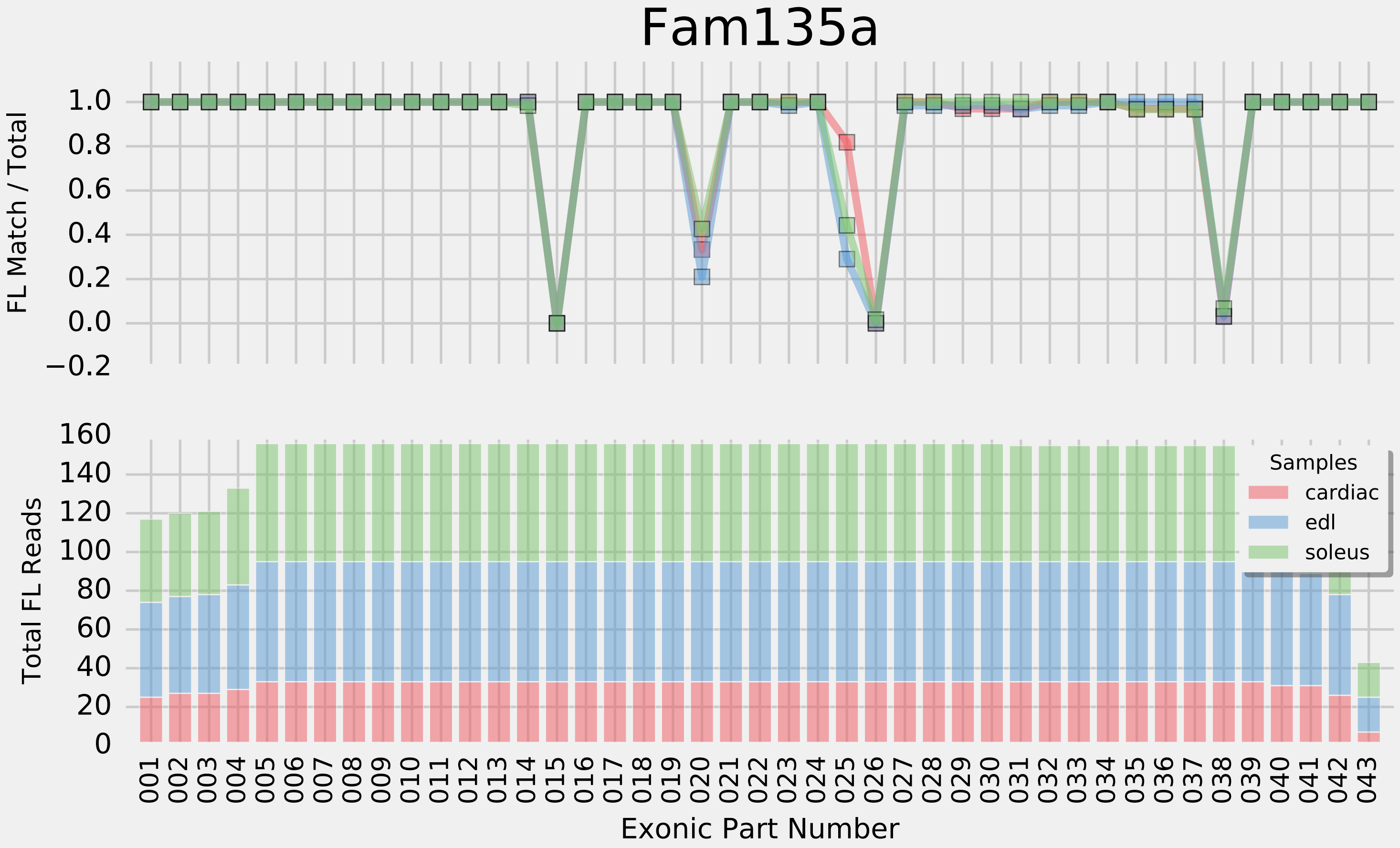

Fam135a
FL Match / Total
1.0
0.8
0.6
0.4
0.2
0.0
−0.2
Total FL Reads
160
140
120
100
80
60
40
20
0
Samples
cardiac
edl
soleus
001 002 003 004 005 006 007 008 009 010 011 012 013 014 015 016 017 018 019 020 021 022 023 024 025 026 027 028 029 030 031 032 033 034 035 036 037 038 039 040 041 042 043
Exonic Part Number

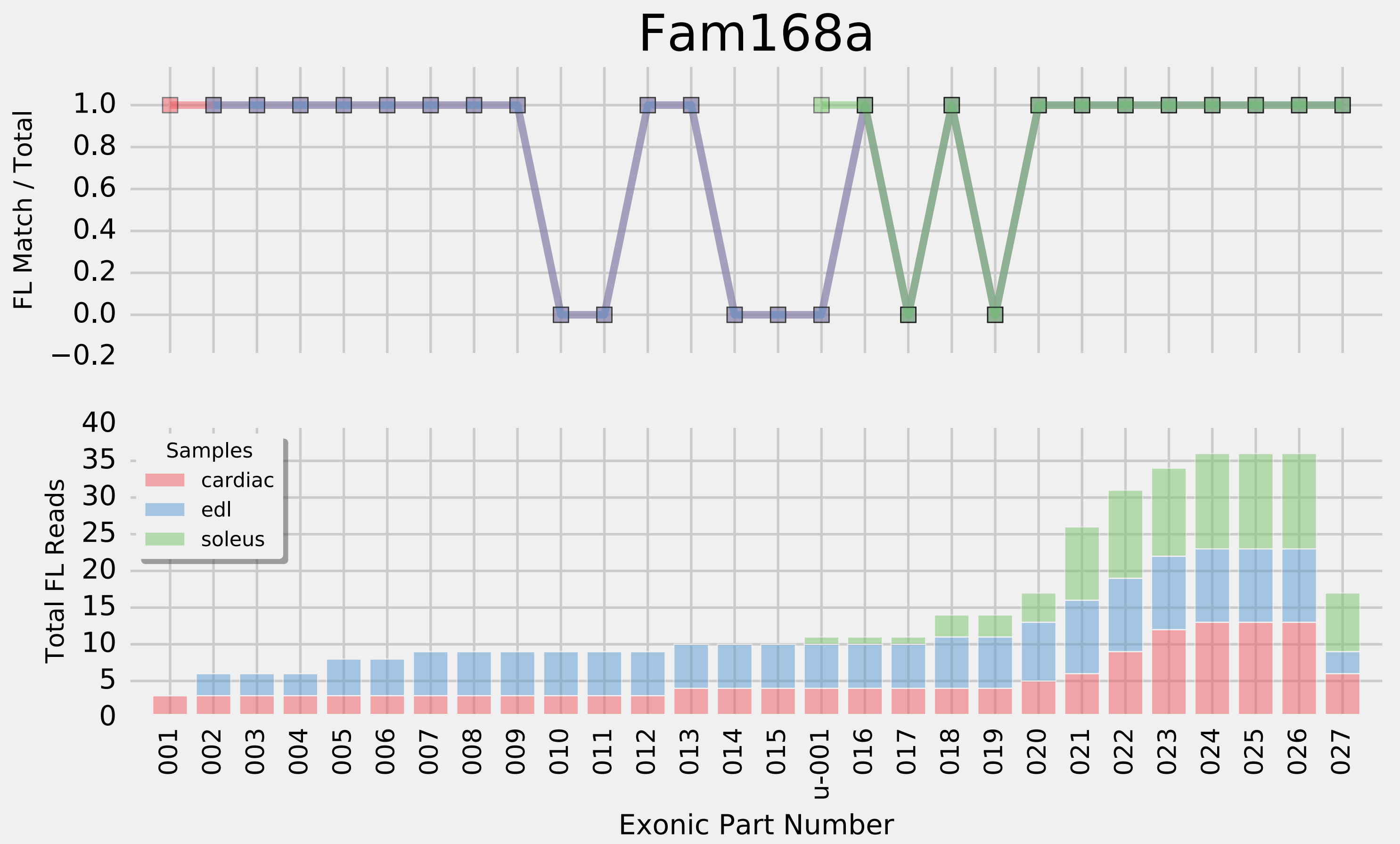

Fam168a
FL Match / Total
1.0
0.8
0.6
0.4
0.2
0.0
−0.2
Total FL Reads
40
35
30
25
20
15
10
5
0
Samples
cardiac
edl
soleus
001
002
003
004
005
006
007
008
009
010
011
012
013
014
015
u-001
016
017
018
019
020
021
022
023
024
025
026
027
Exonic Part Number

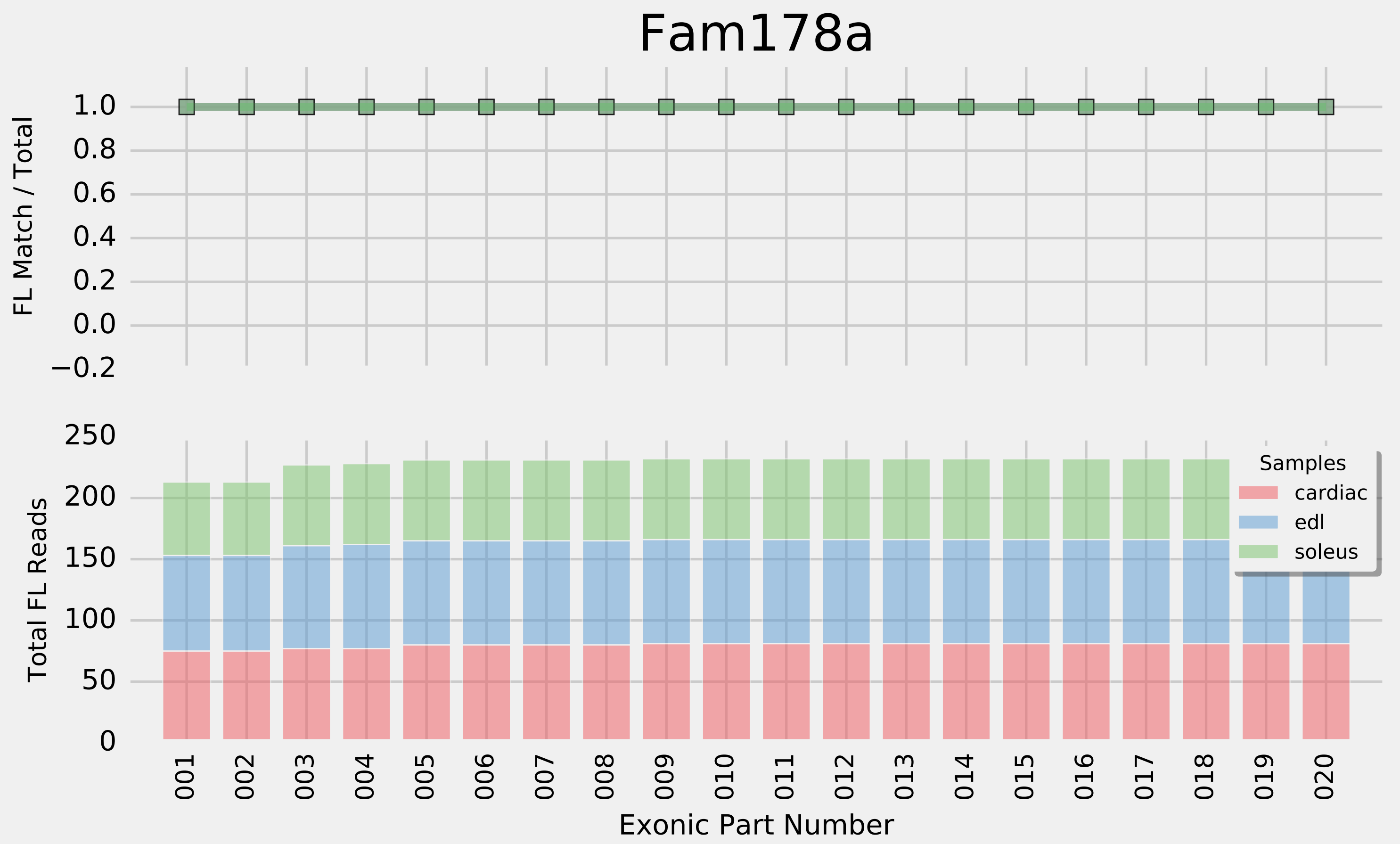

Fam178a
FL Match / Total
1.0
0.8
0.6
0.4
0.2
0.0
−0.2
Total FL Reads
250
200
150
100
50
0
Samples
cardiac
edl
soleus
001
002
003
004
005
006
007
008
009
010
011
012
013
014
015
016
017
018
019
020
Exonic Part Number

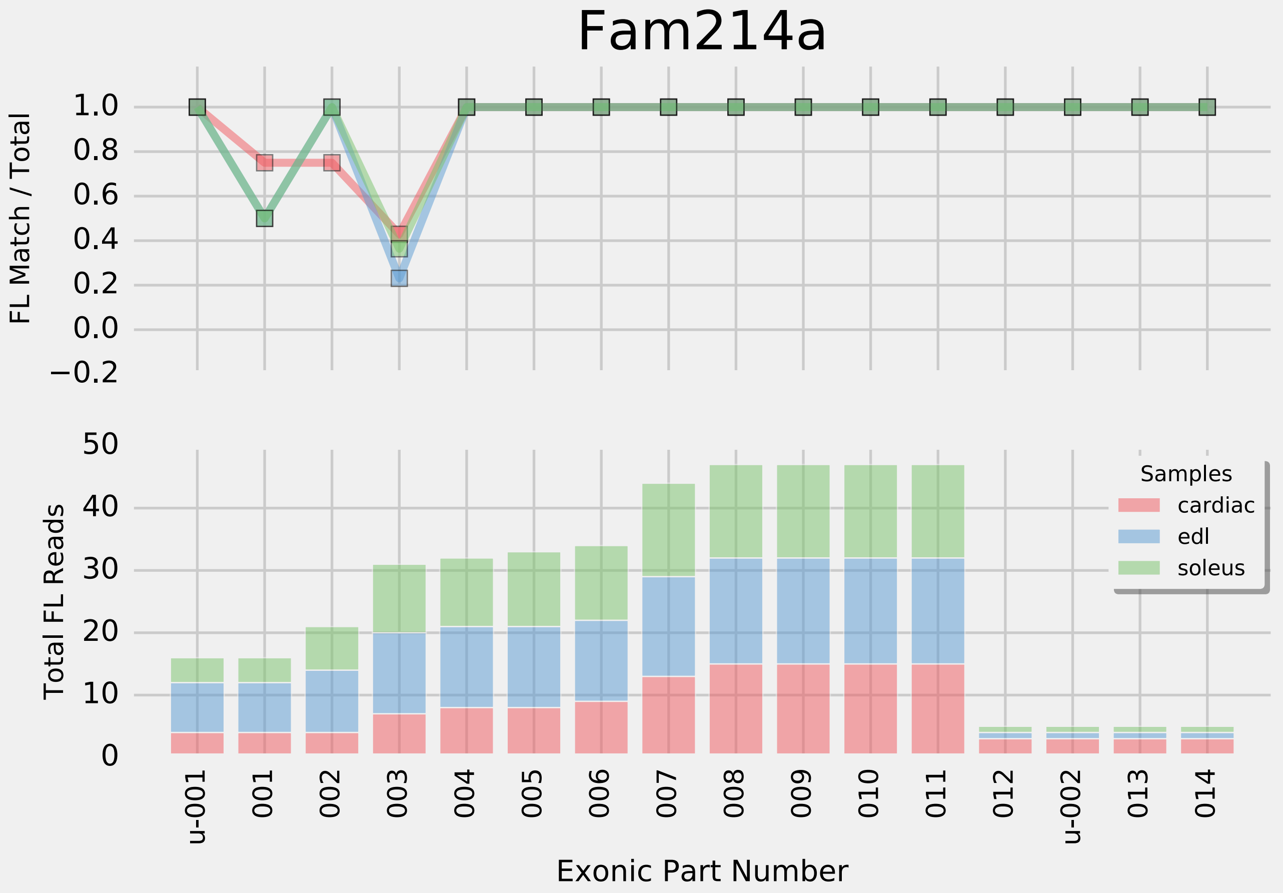
Fam214a
FL Match / Total
1.0
0.8
0.6
0.4
0.2
0.0
−0.2
50
40
30
20
10
0
Total FL Reads
Samples
cardiac
edl
soleus
u-001
001
002
003
004
005
006
007
008
009
010
011
012
u-002
013
014
Exonic Part Number

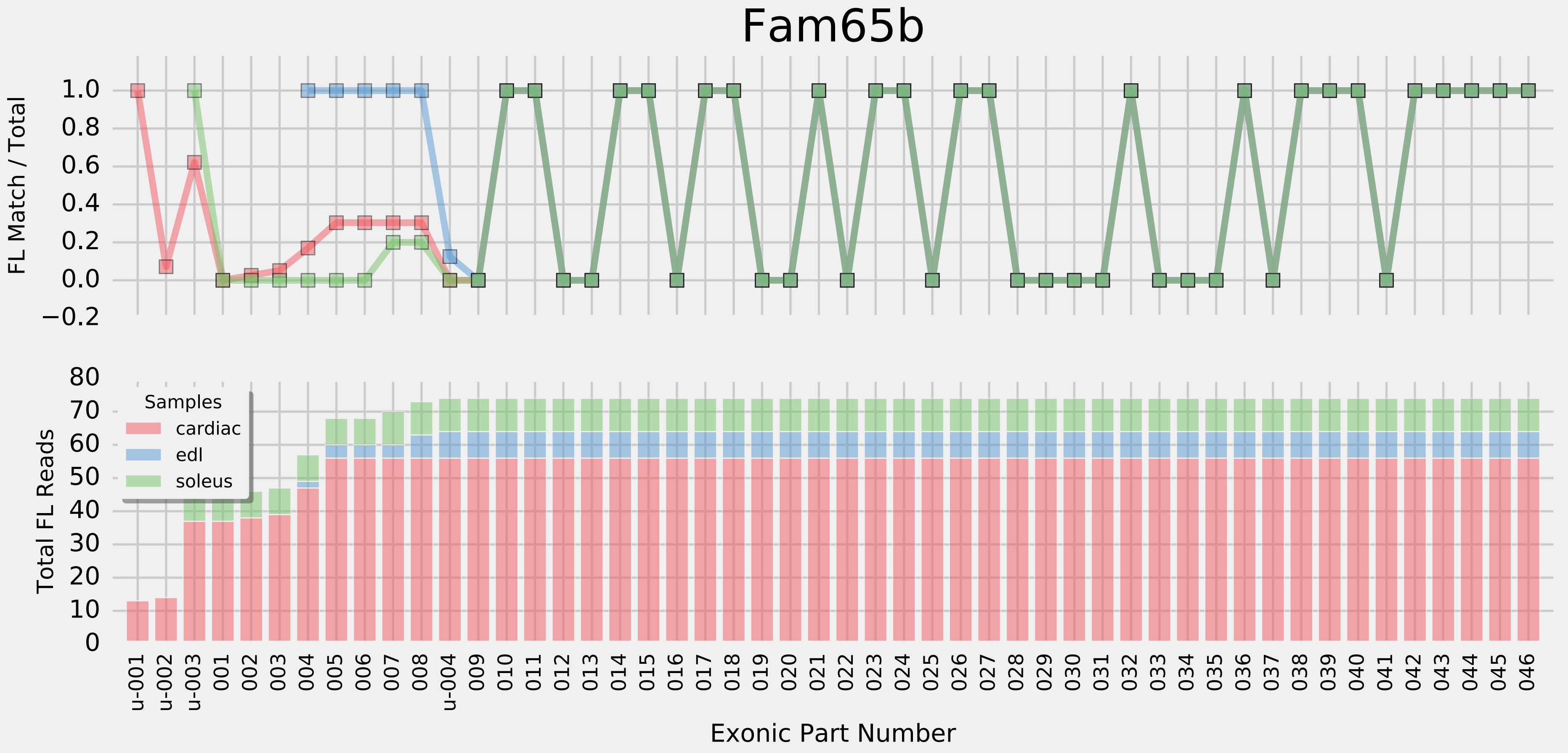

Fam65b
FL Match / Total
1.0
0.8
0.6
0.4
0.2
0.0
−0.2
Total FL Reads
80
70
60
50
40
30
20
10
0
Samples
cardiac
edl
soleus
u-001
u-002
u-003
001
002
003
004
005
006
007
008
u-004
009
010
011
012
013
014
015
016
017
018
019
020
021
022
023
024
025
026
027
028
029
030
031
032
033
034
035
036
037
038
039
040
041
042
043
044
045
046
Exonic Part Number

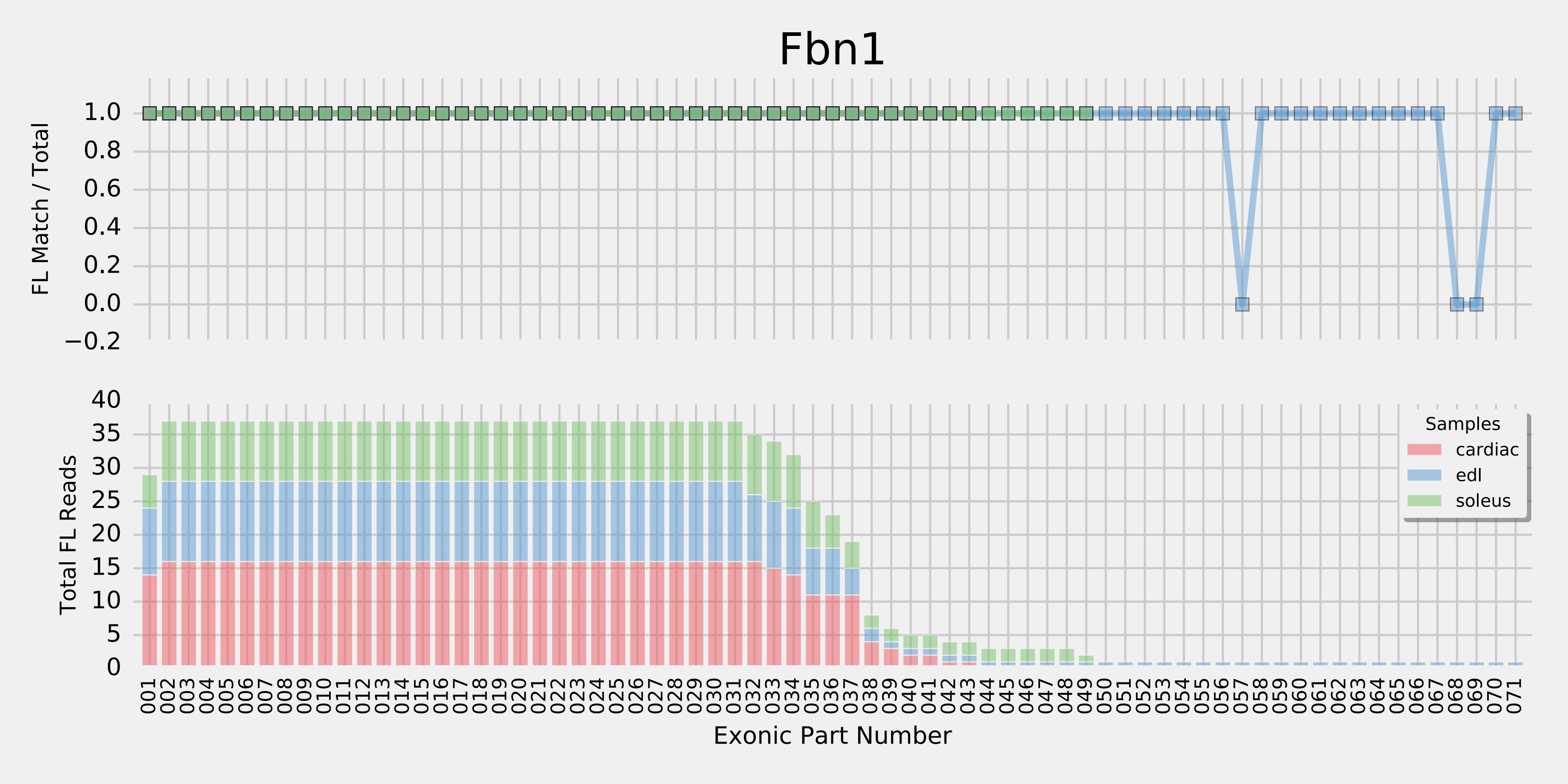

Fbn1
FL Match / Total
1.0
0.8
0.6
0.4
0.2
0.0
−0.2
Total FL Reads
40
35
30
25
20
15
10
5
0
Samples
cardiac
edl
soleus
Exonic Part Number
001 002 003 004 005 006 007 008 009 010 011 012 013 014 015 016 017 018 019 020 021 022 023 024 025 026 027 028 029 030 031 032 033 034 035 036 037 038 039 040 041 042 043 044 045 046 047 048 049 050 051 052 053 054 055 056 057 058 059 060 061 062 063 064 065 066 067 068 069 070 071

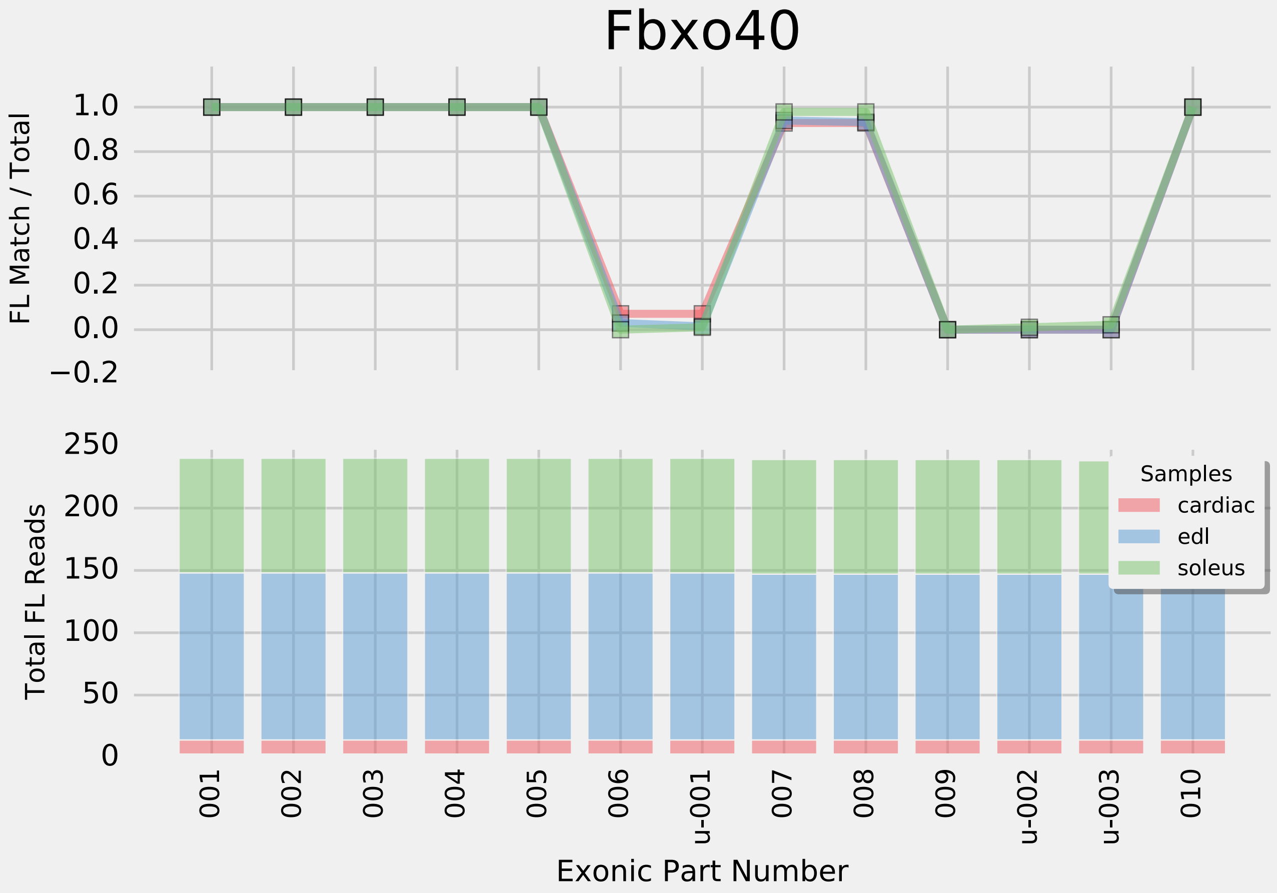
Fbxo40
FL Match / Total
1.0
0.8
0.6
0.4
0.2
0.0
−0.2
Total FL Reads
250
200
150
100
50
0
Samples
cardiac
edl
soleus
001
002
003
004
005
006
u-001
007
008
009
u-002
u-003
010
Exonic Part Number

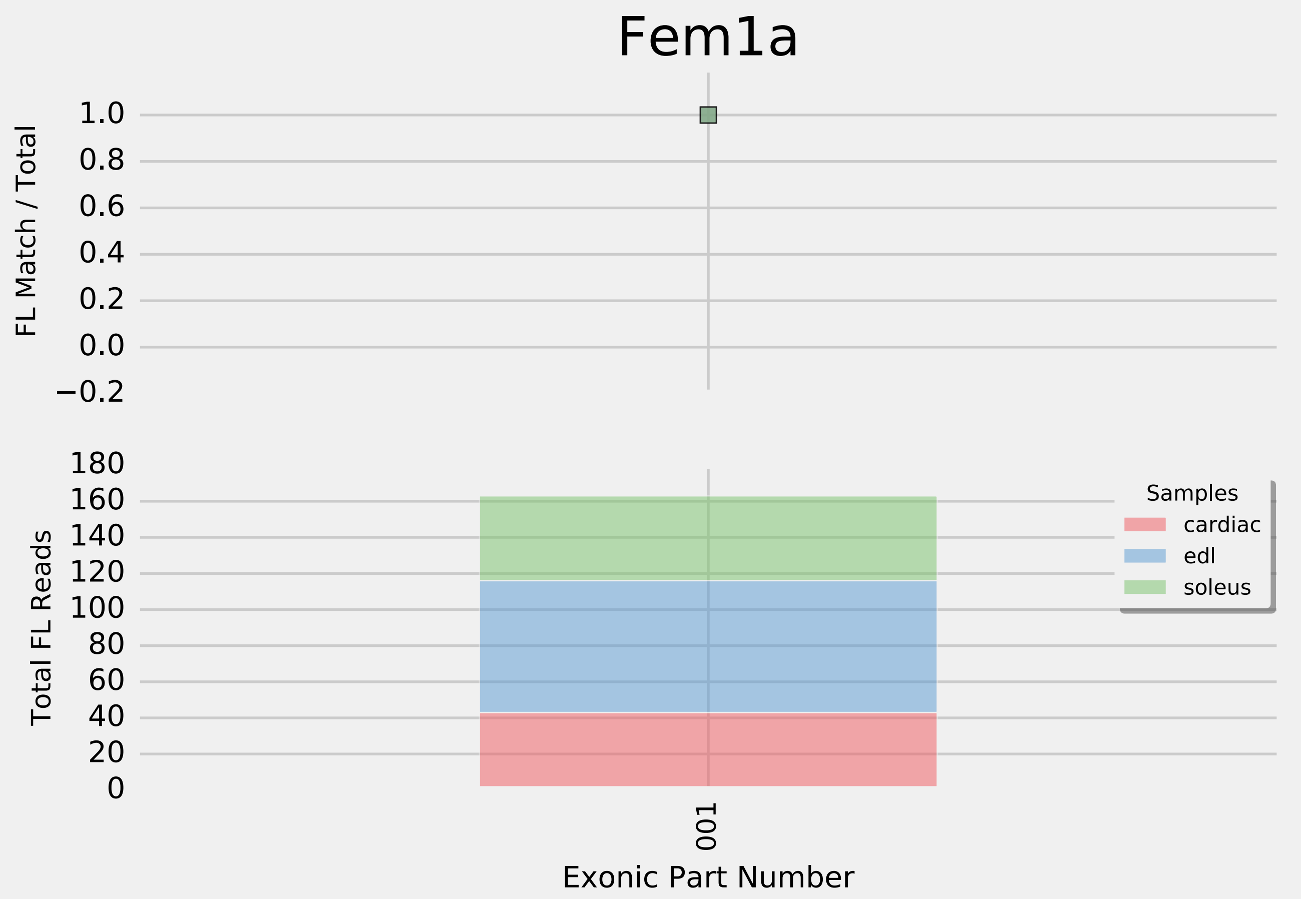
Fem1a
FL Match / Total
1.0
0.8
0.6
0.4
0.2
0.0
−0.2
Total FL Reads
180
160
140
120
100
80
60
40
20
0
Samples
cardiac
edl
soleus
001
Exonic Part Number

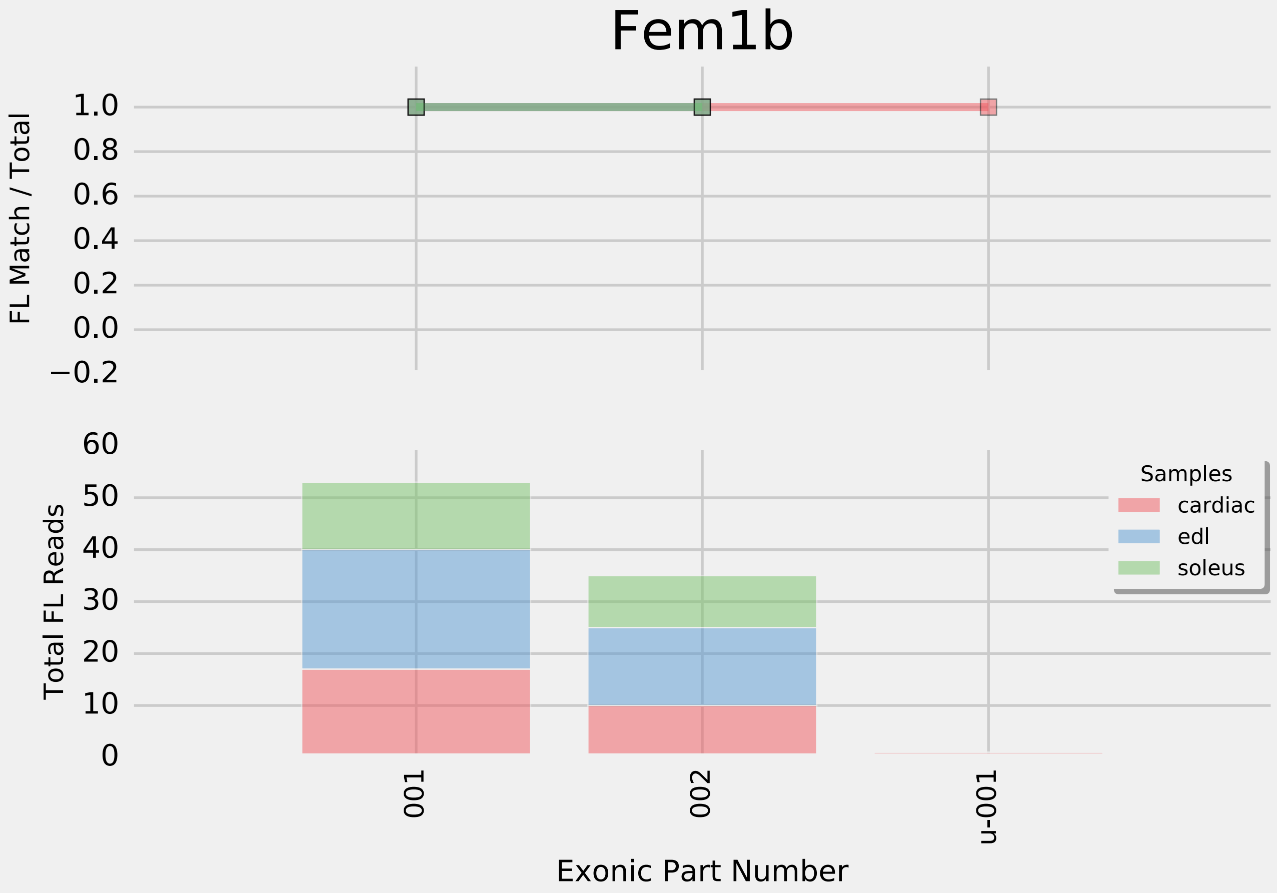
Fem1b
FL Match / Total
1.0
0.8
0.6
0.4
0.2
0.0
−0.2
Total FL Reads
60
50
40
30
20
10
0
Samples
cardiac
edl
soleus
001
002
u-001
Exonic Part Number

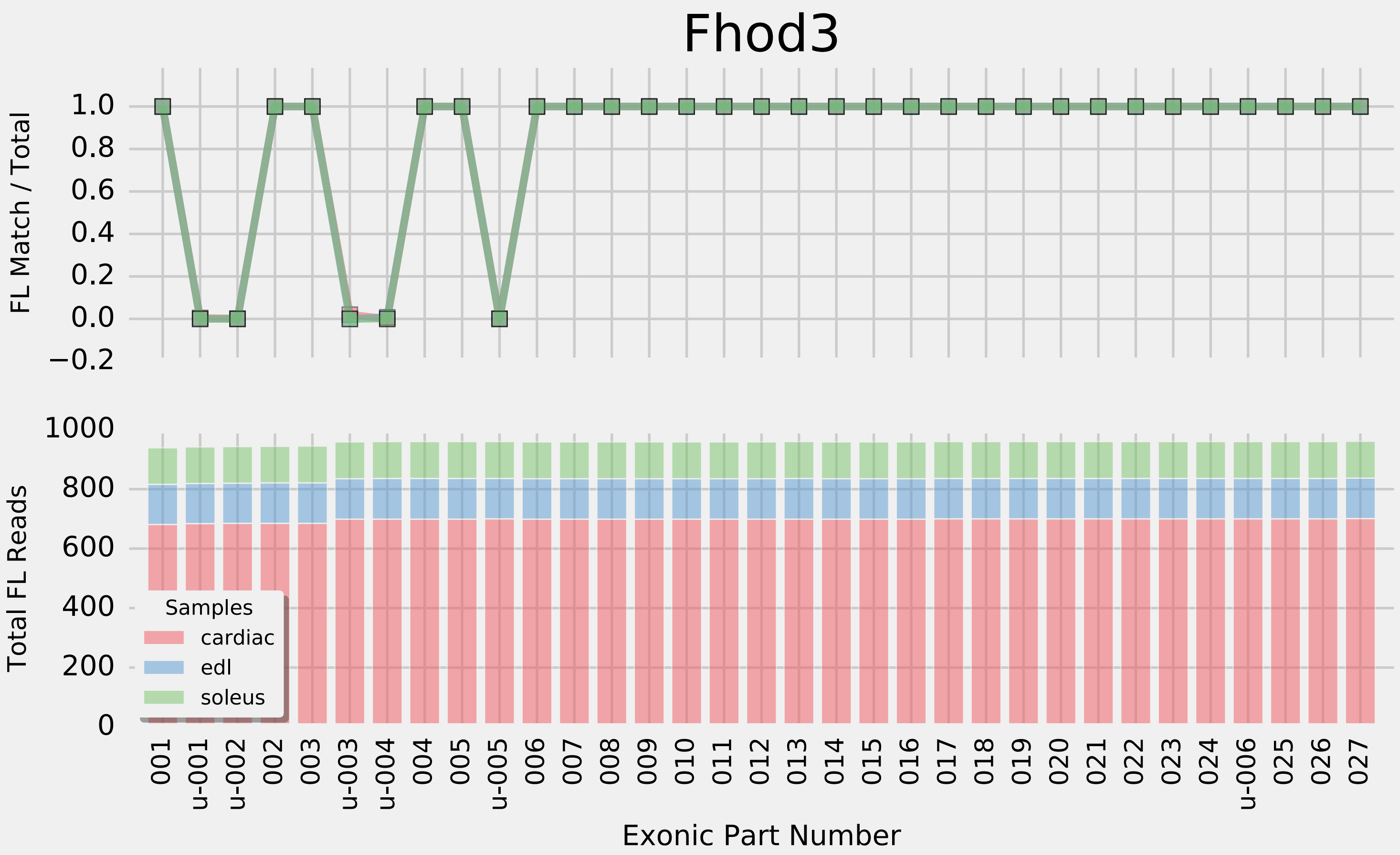

Fhod3
FL Match / Total
1.0
0.8
0.6
0.4
0.2
0.0
−0.2
Total FL Reads
1000
800
600
400
200
0
Samples
cardiac
edl
soleus
001
u-001
u-002
002
003
u-003
u-004
004
005
u-005
006
007
008
009
010
011
012
013
014
015
016
017
018
019
020
021
022
023
024
u-006
025
026
027
Exonic Part Number

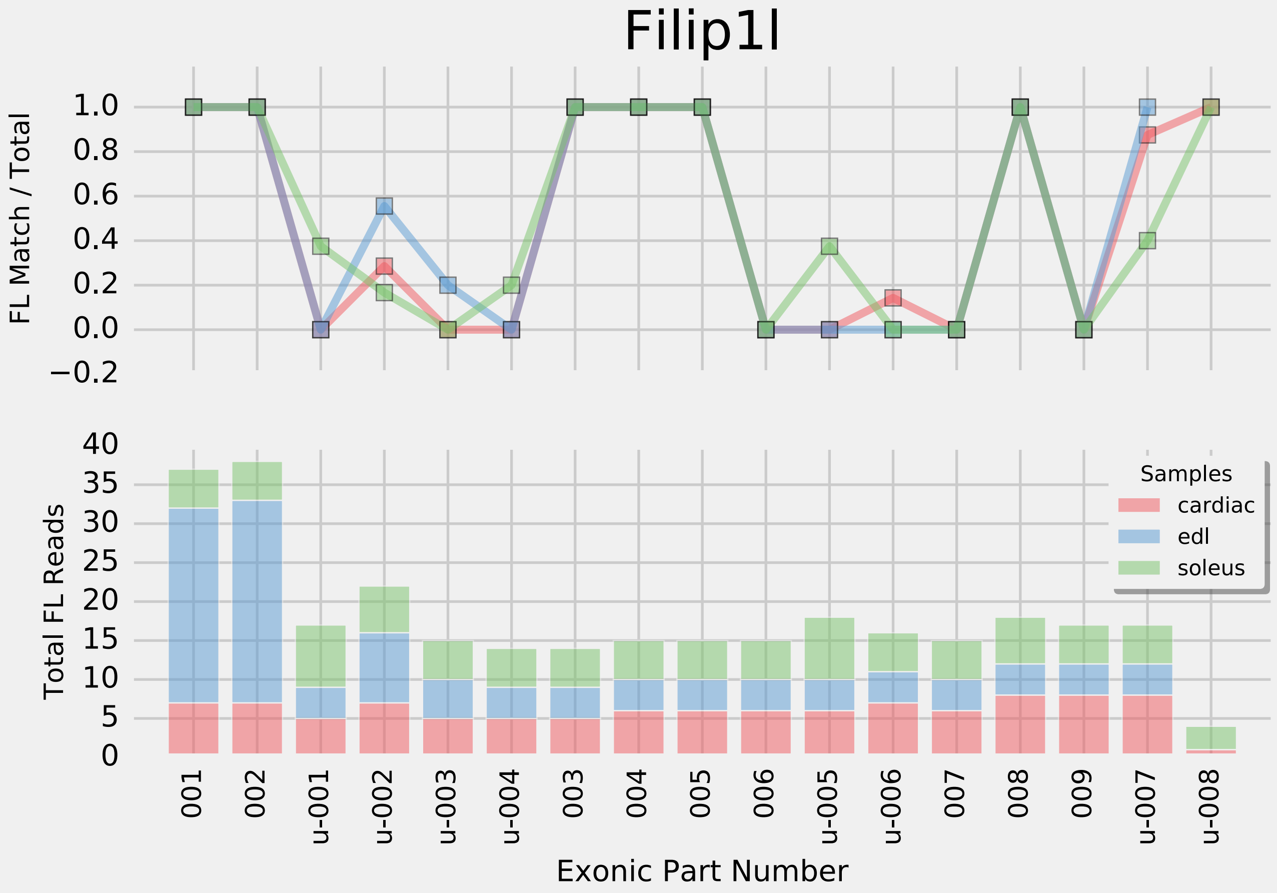
Filip1l
FL Match / Total
1.0
0.8
0.6
0.4
0.2
0.0
−0.2
Total FL Reads
40
35
30
25
20
15
10
5
0
Samples
cardiac
edl
soleus
001
002
u-001
u-002
u-003
u-004
003
004
005
006
u-005
u-006
007
008
009
u-007
u-008
Exonic Part Number

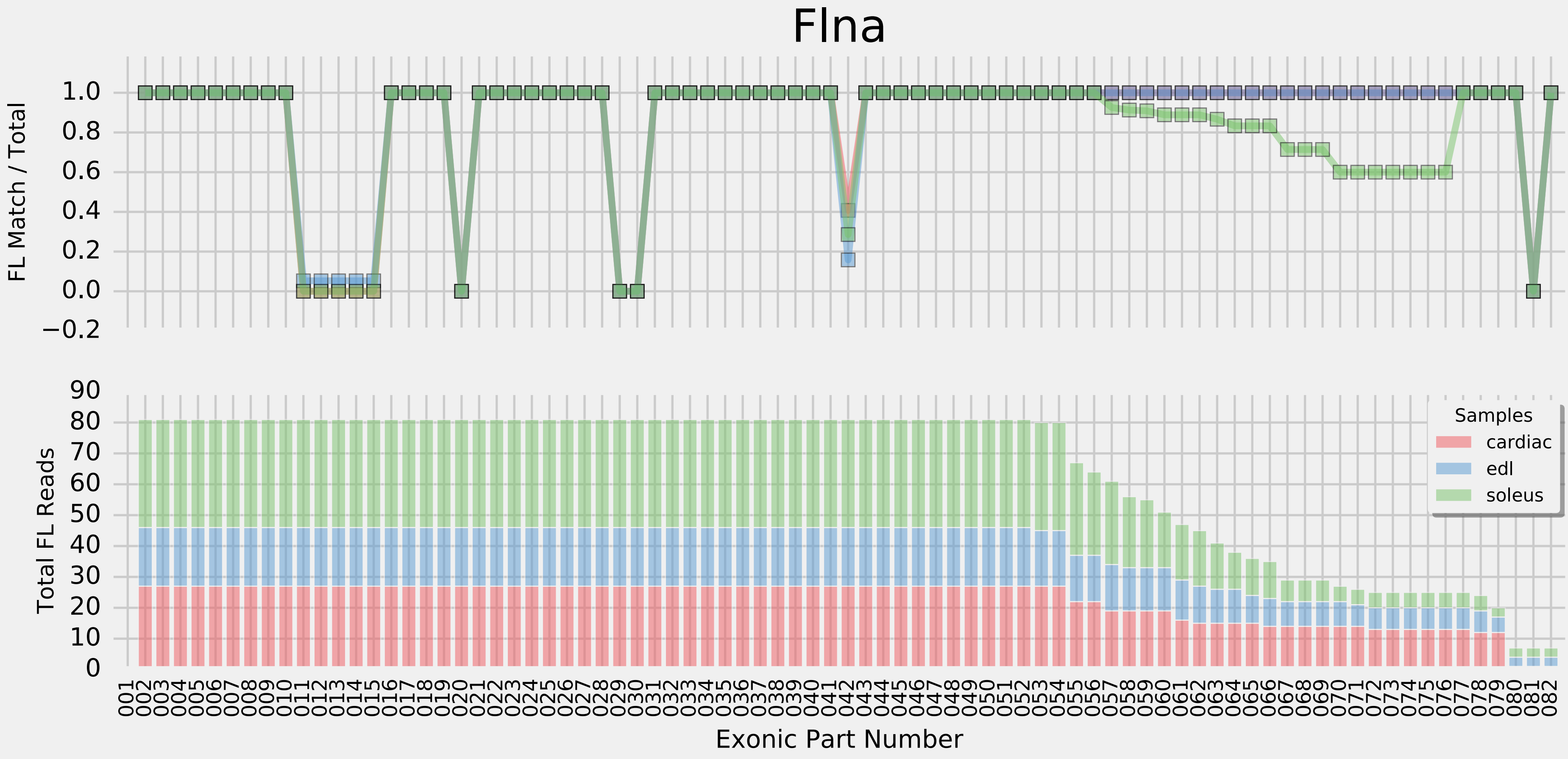

Flna
FL Match / Total
Total FL Reads
Exonic Part Number
Samples
cardiac
edl
soleus

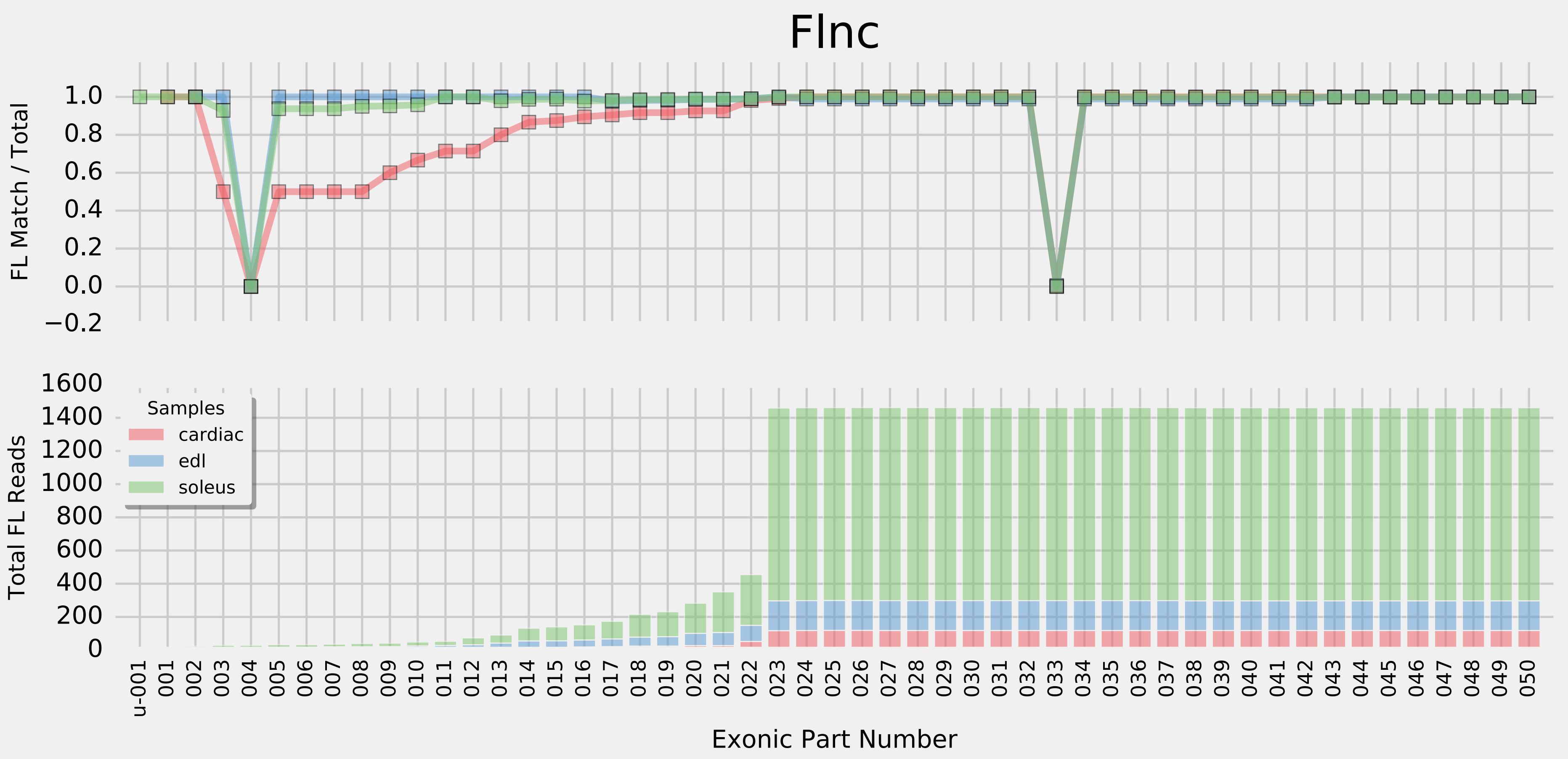

Flnc
FL Match / Total
Total FL Reads
Samples
cardiac
edl
soleus
Exonic Part Number

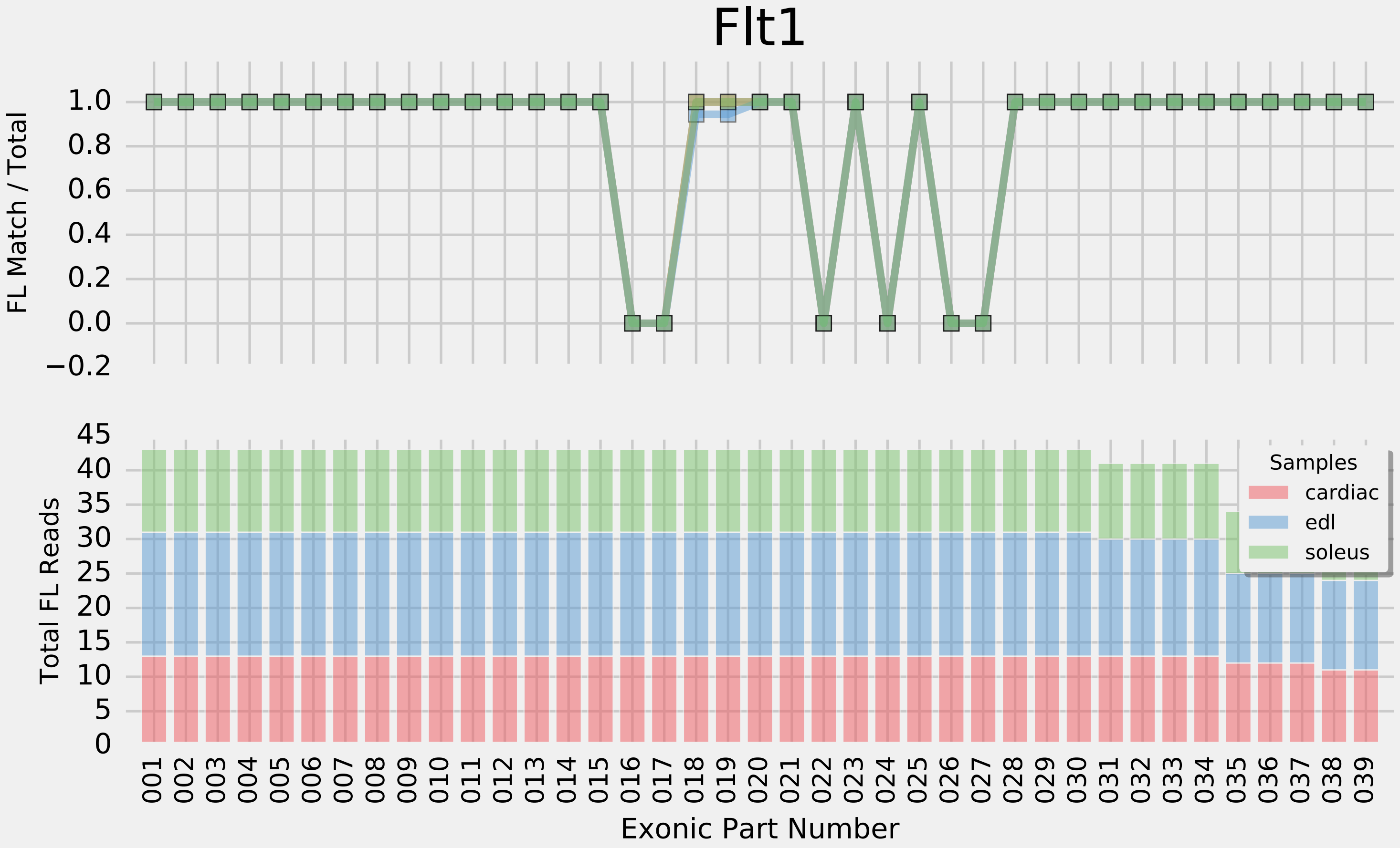
Flt1
FL Match / Total
1.0
0.8
0.6
0.4
0.2
0.0
−0.2
Total FL Reads
45
40
35
30
25
20
15
10
5
0
Samples
cardiac
edl
soleus
001 002 003 004 005 006 007 008 009 010 011 012 013 014 015 016 017 018 019 020 021 022 023 024 025 026 027 028 029 030 031 032 033 034 035 036 037 038 039
Exonic Part Number

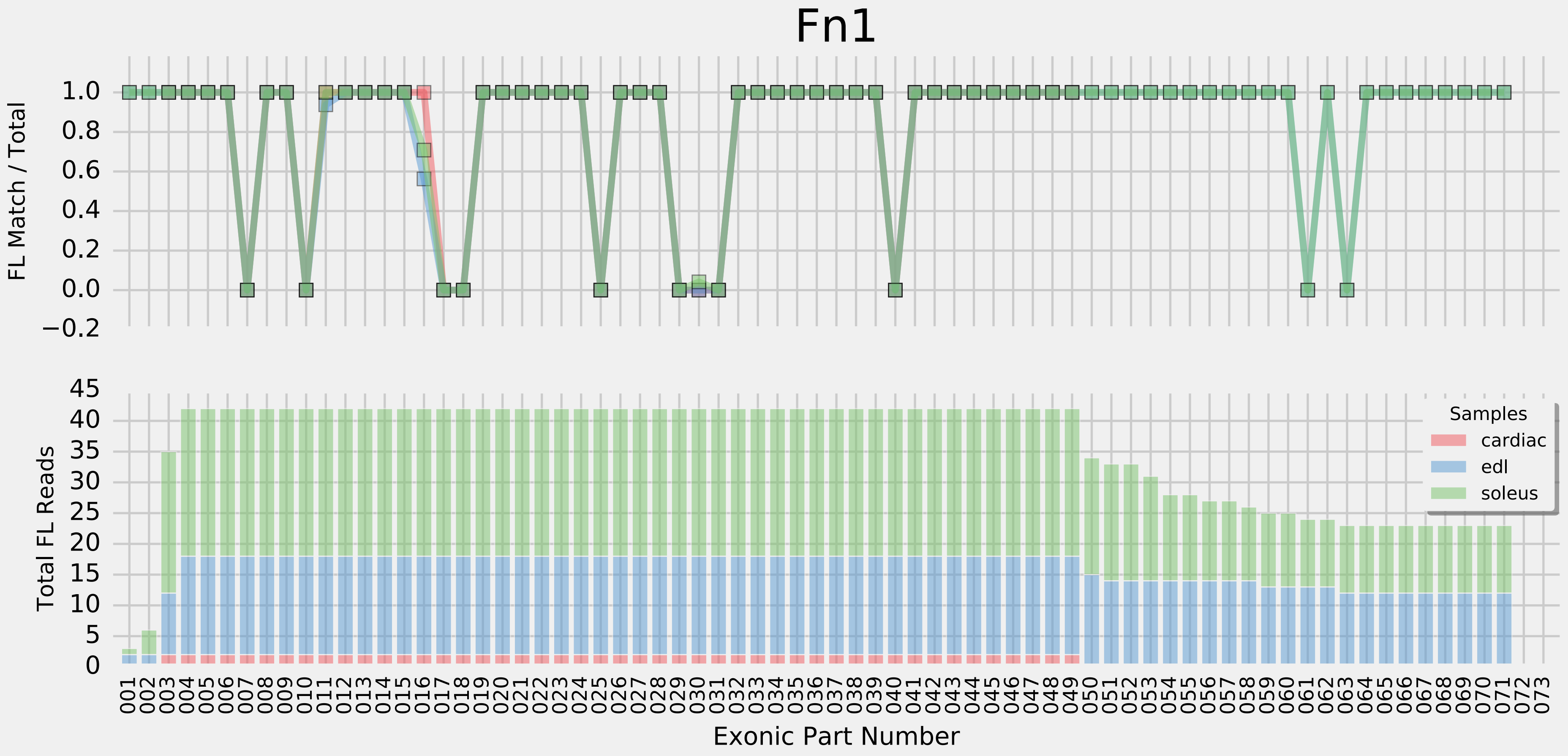

Fn1
FL Match / Total
1.0
0.8
0.6
0.4
0.2
0.0
−0.2
Total FL Reads
45
40
35
30
25
20
15
10
5
0
Samples
cardiac
edl
soleus
001 002 003 004 005 006 007 008 009 010 011 012 013 014 015 016 017 018 019 020 021 022 023 024 025 026 027 028 029 030 031 032 033 034 035 036 037 038 039 040 041 042 043 044 045 046 047 048 049 050 051 052 053 054 055 056 057 058 059 060 061 062 063 064 065 066 067 068 069 070 071 072 073
Exonic Part Number

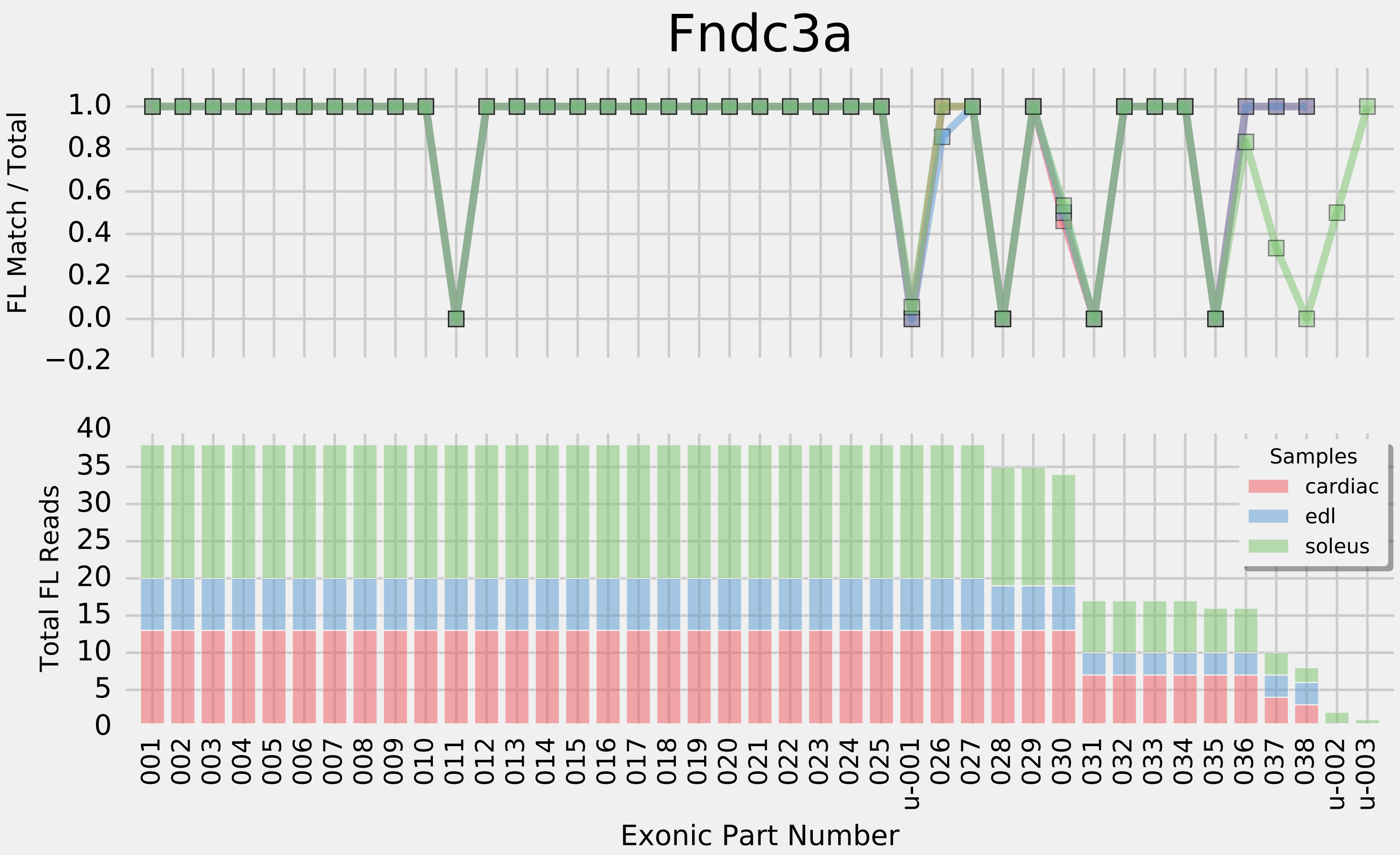

Fndc3a
FL Match / Total
1.0
0.8
0.6
0.4
0.2
0.0
−0.2
Total FL Reads
40
35
30
25
20
15
10
5
0
Samples
cardiac
edl
soleus
001
002
003
004
005
006
007
008
009
010
011
012
013
014
015
016
017
018
019
020
021
022
023
024
025
u-001
026
027
028
029
030
031
032
033
034
035
036
037
038
u-002
u-003
Exonic Part Number

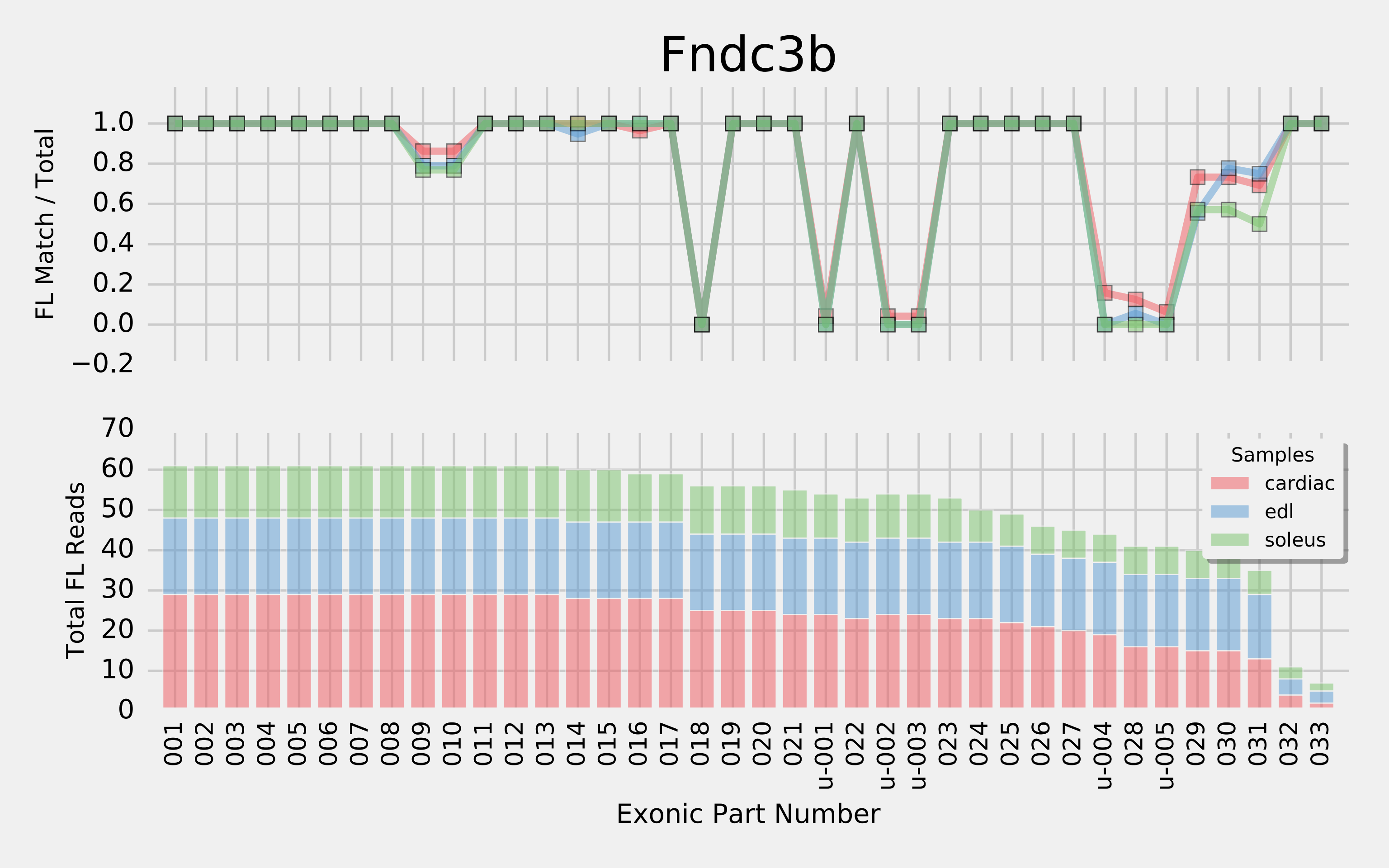

Fndc3b
FL Match / Total
1.0
0.8
0.6
0.4
0.2
0.0
−0.2
Total FL Reads
70
60
50
40
30
20
10
0
Samples
cardiac
edl
soleus
001 002 003 004 005 006 007 008 009 010 011 012 013 014 015 016 017 018 019 020 021 u-001 022 u-002 u-003 023 024 025 026 027 u-004 028 u-005 029 030 031 032 033
Exonic Part Number

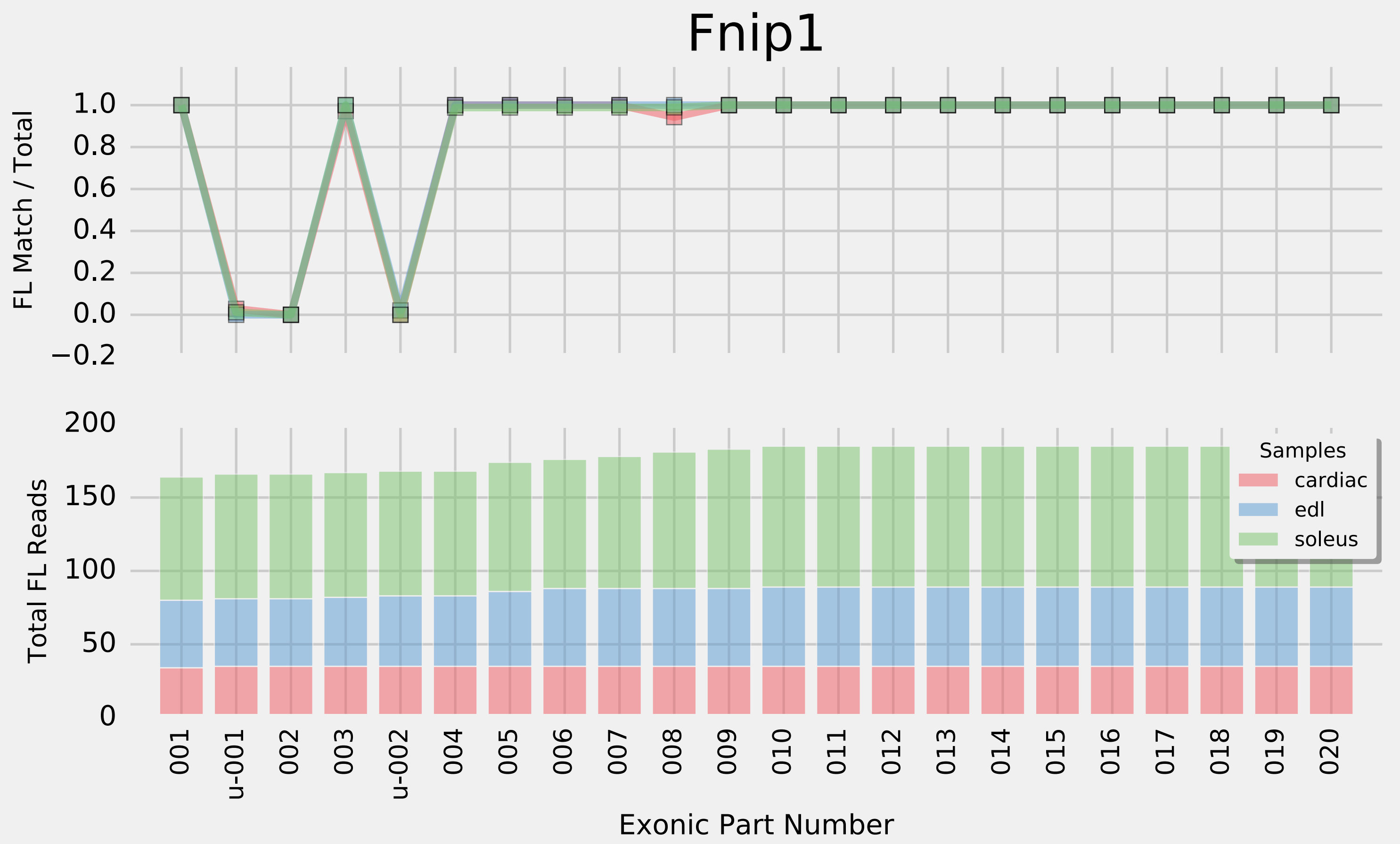
Fnip1
FL Match / Total
1.0
0.8
0.6
0.4
0.2
0.0
−0.2
Total FL Reads
200
150
100
50
0
Samples
cardiac
edl
soleus
001
u-001
002
003
u-002
004
005
006
007
008
009
010
011
012
013
014
015
016
017
018
019
020
Exonic Part Number

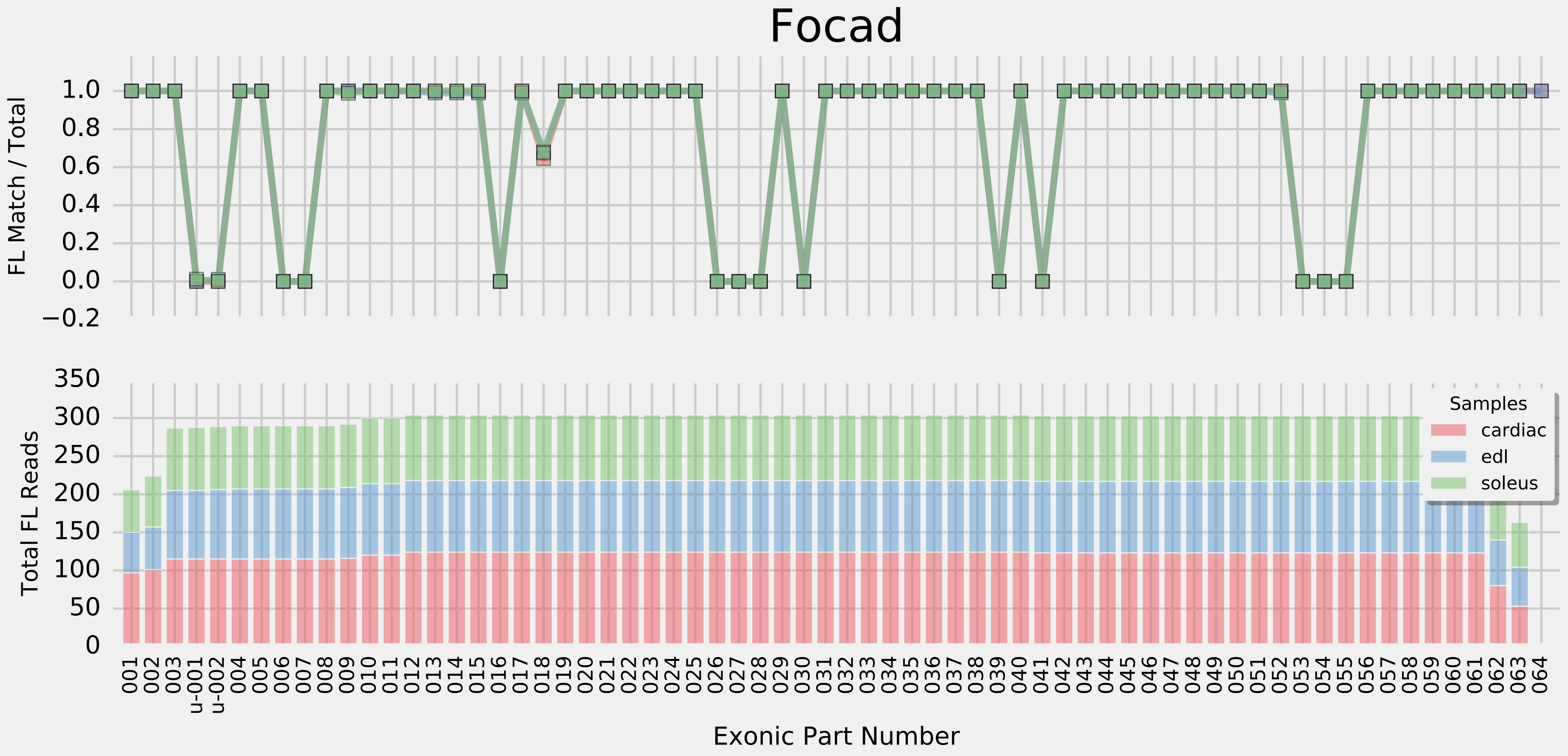

Focad
FL Match / Total
Total FL Reads
Samples
cardiac
edl
soleus
Exonic Part Number
001
002
003
u-001
u-002
004
005
006
007
008
009
010
011
012
013
014
015
016
017
018
019
020
021
022
023
024
025
026
027
028
029
030
031
032
033
034
035
036
037
038
039
040
041
042
043
044
045
046
047
048
049
050
051
052
053
054
055
056
057
058
059
060
061
062
063
064

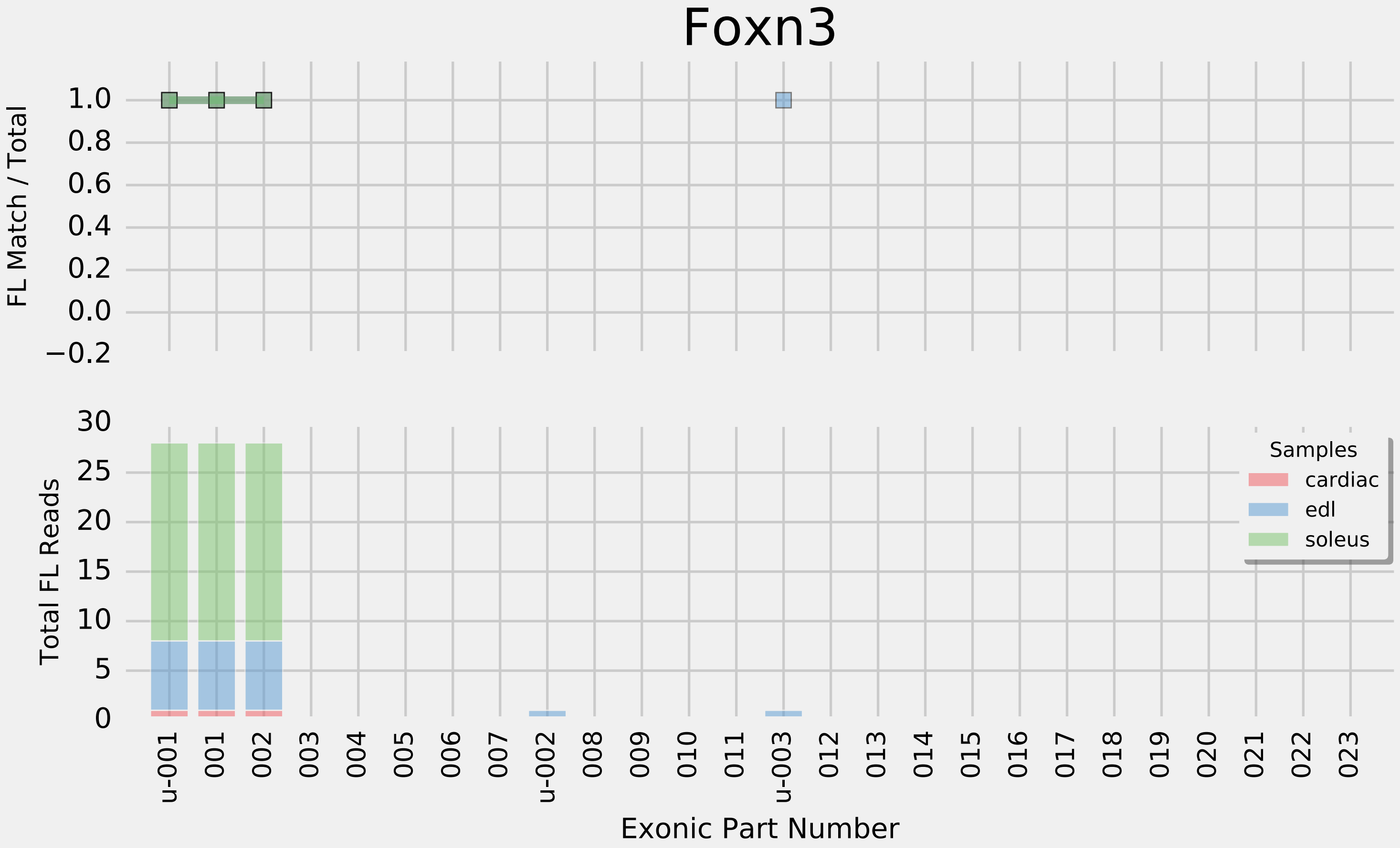

Foxn3
FL Match / Total
1.0
0.8
0.6
0.4
0.2
0.0
−0.2
Total FL Reads
30
25
20
15
10
5
0
Samples
cardiac
edl
soleus
u-001
001
002
003
004
005
006
007
u-002
008
009
010
011
u-003
012
013
014
015
016
017
018
019
020
021
022
023
Exonic Part Number

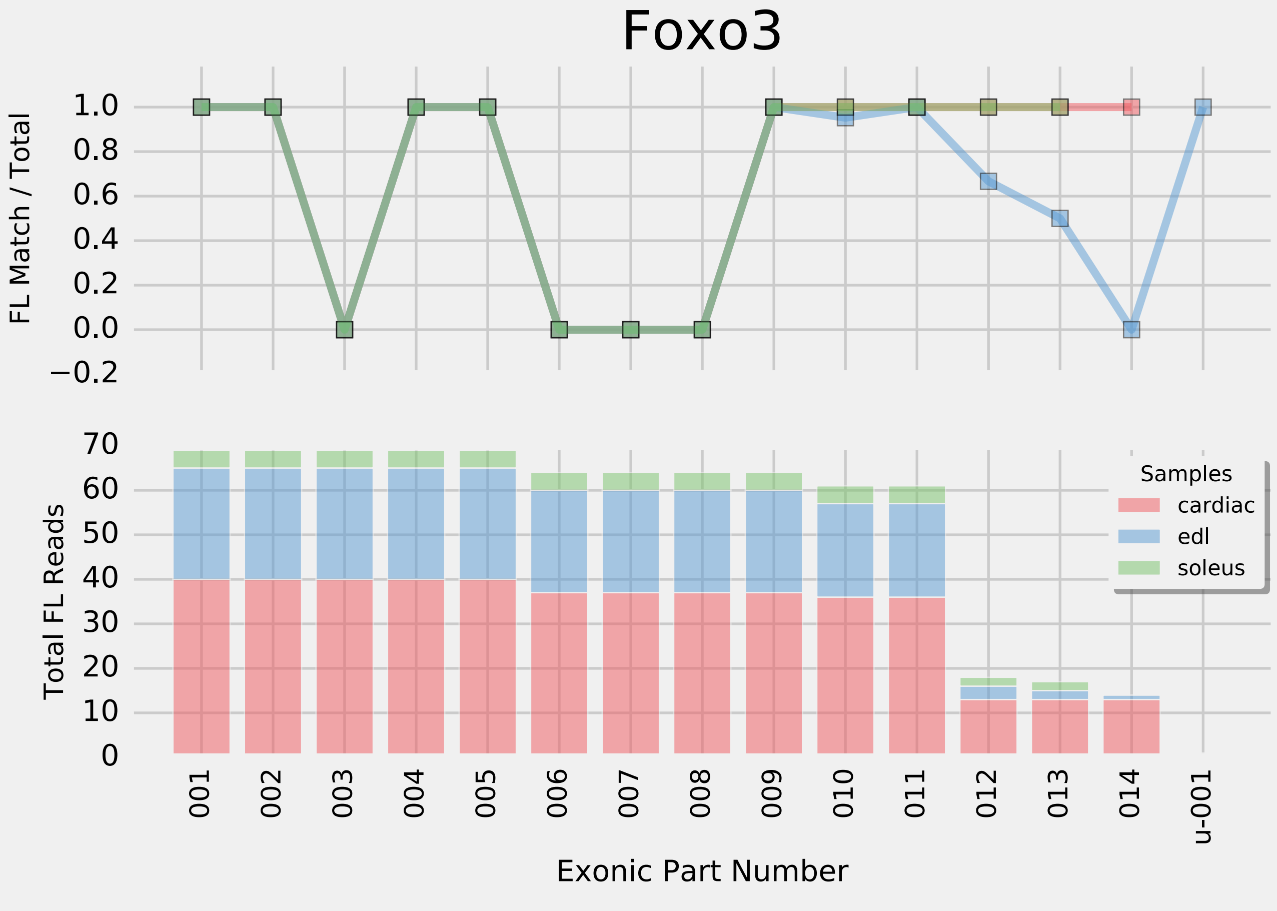
Foxo3
FL Match / Total
1.0
0.8
0.6
0.4
0.2
0.0
−0.2
Total FL Reads
70
60
50
40
30
20
10
0
Samples
cardiac
edl
soleus
001
002
003
004
005
006
007
008
009
010
011
012
013
014
u-001
Exonic Part Number

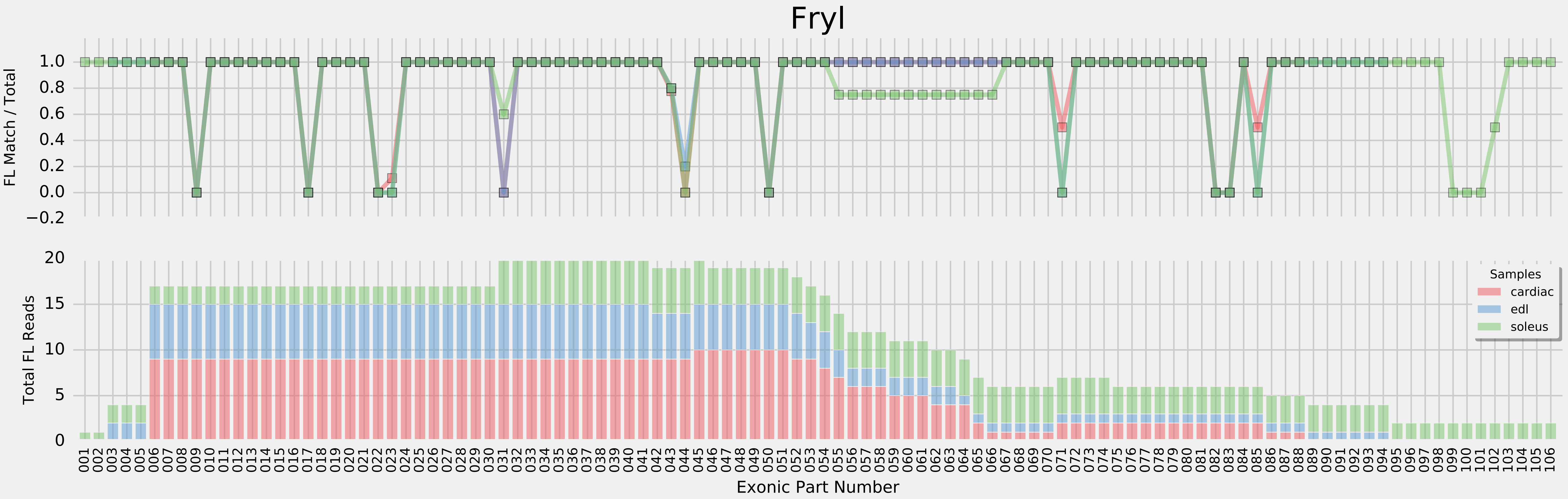
Fryl
FL Match / Total
Total FL Reads
Exonic Part Number
Samples
cardiac
edl
soleus

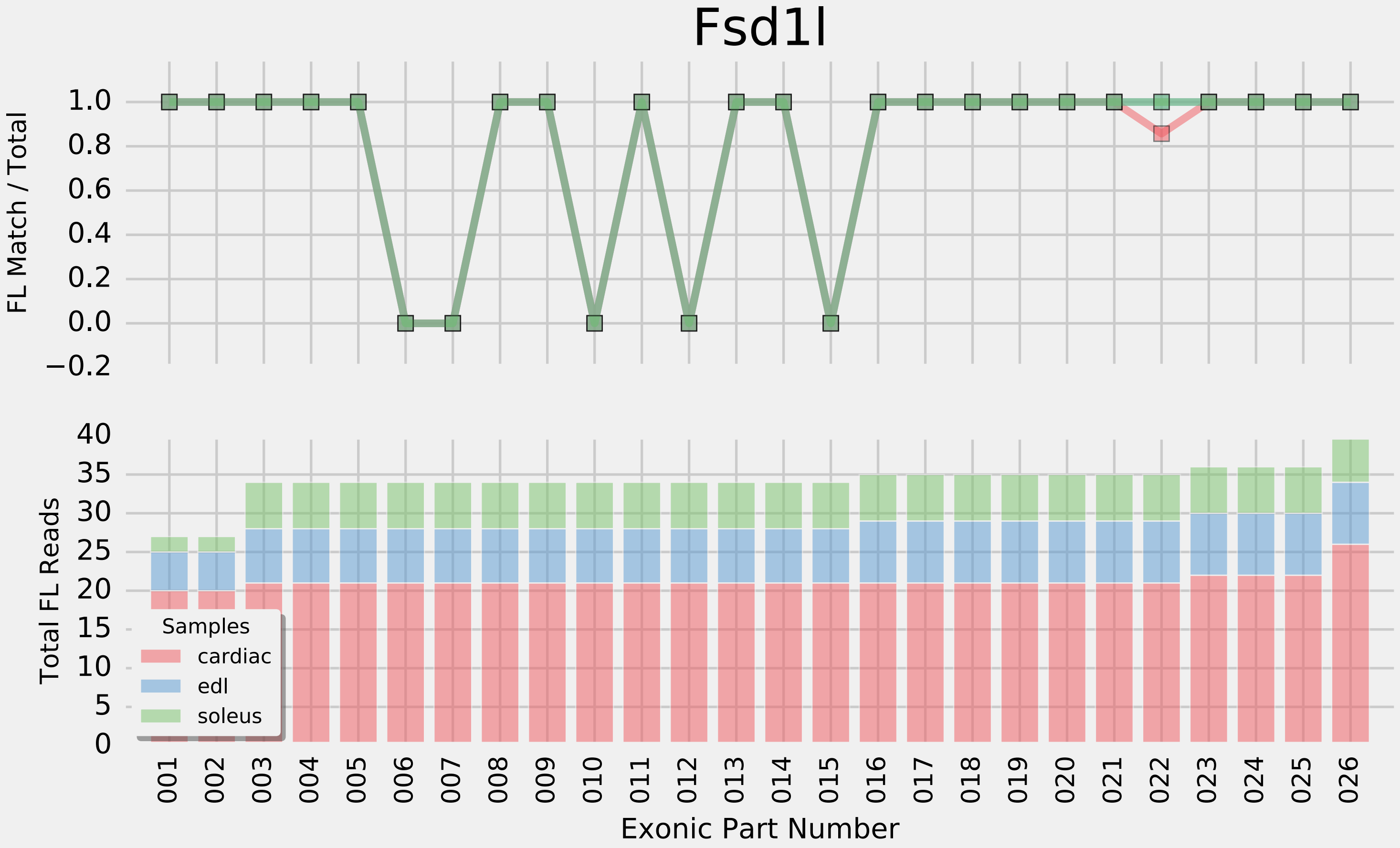

Fsd1l
FL Match / Total
1.0
0.8
0.6
0.4
0.2
0.0
−0.2
Total FL Reads
40
35
30
25
20
15
10
5
0
Samples
cardiac
edl
soleus
001
002
003
004
005
006
007
008
009
010
011
012
013
014
015
016
017
018
019
020
021
022
023
024
025
026
Exonic Part Number

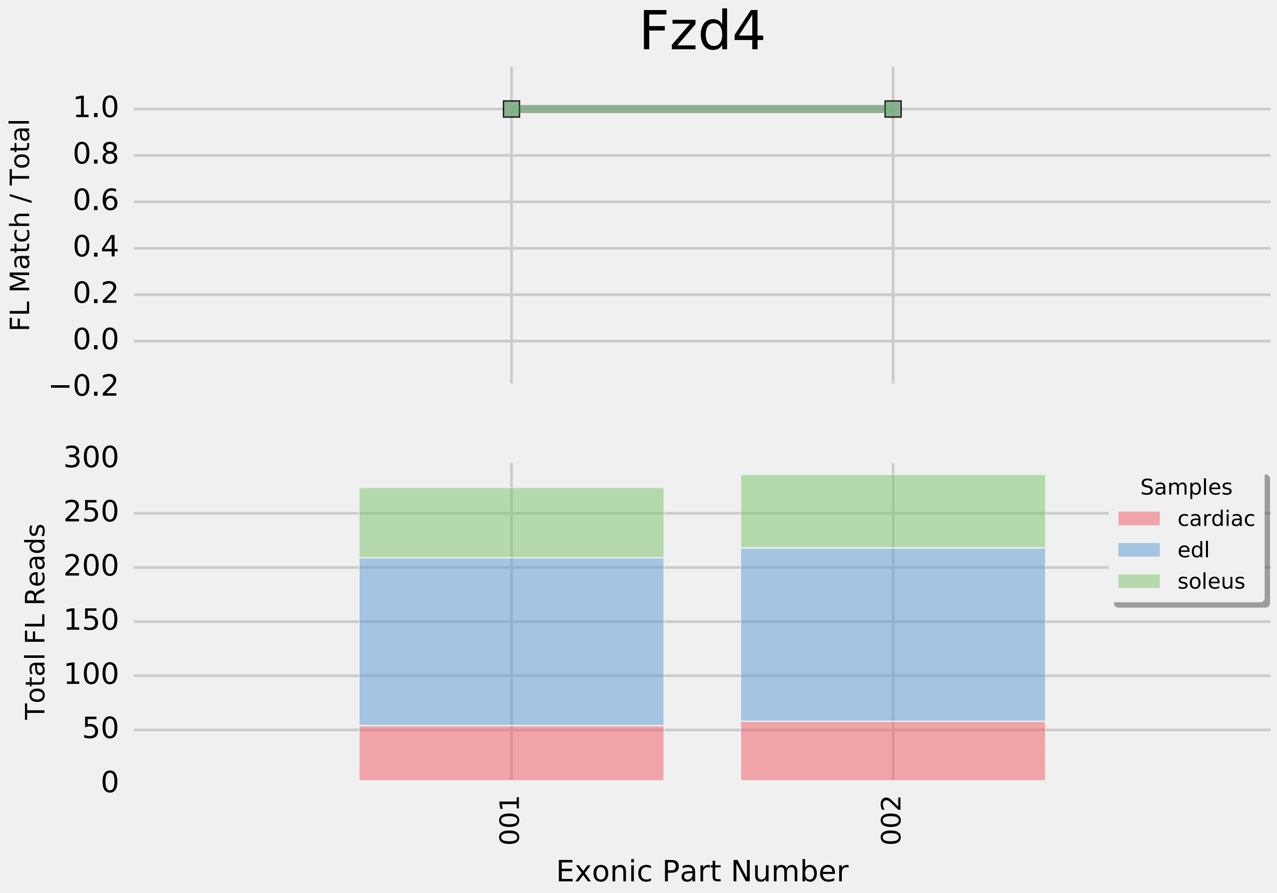
Fzd4
FL Match / Total
1.0
0.8
0.6
0.4
0.2
0.0
−0.2
Total FL Reads
300
250
200
150
100
50
0
Samples
cardiac
edl
soleus
001
002
Exonic Part Number

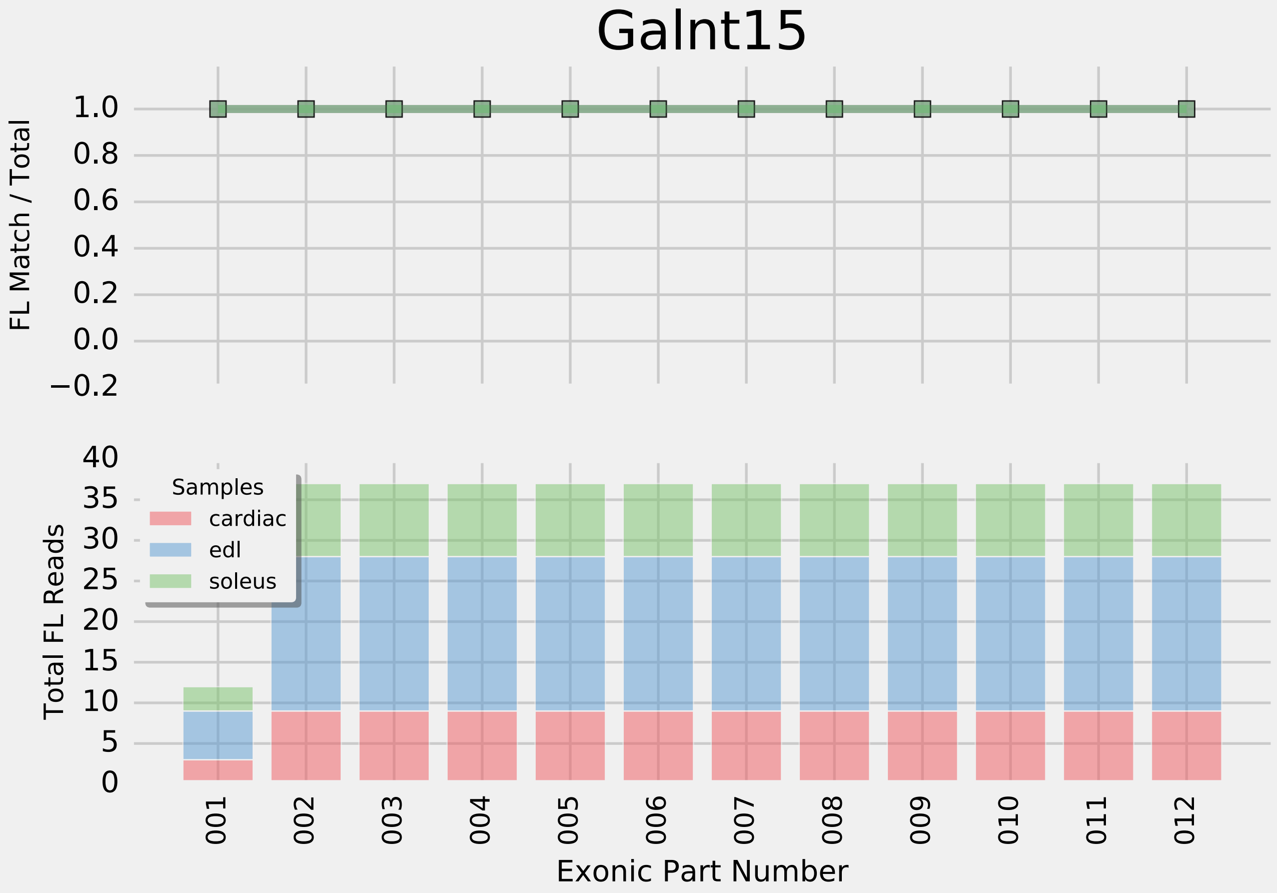
Galnt15
FL Match / Total
1.0
0.8
0.6
0.4
0.2
0.0
−0.2
Total FL Reads
40
35
30
25
20
15
10
5
0
Samples
cardiac
edl
soleus
001
002
003
004
005
006
007
008
009
010
011
012
Exonic Part Number

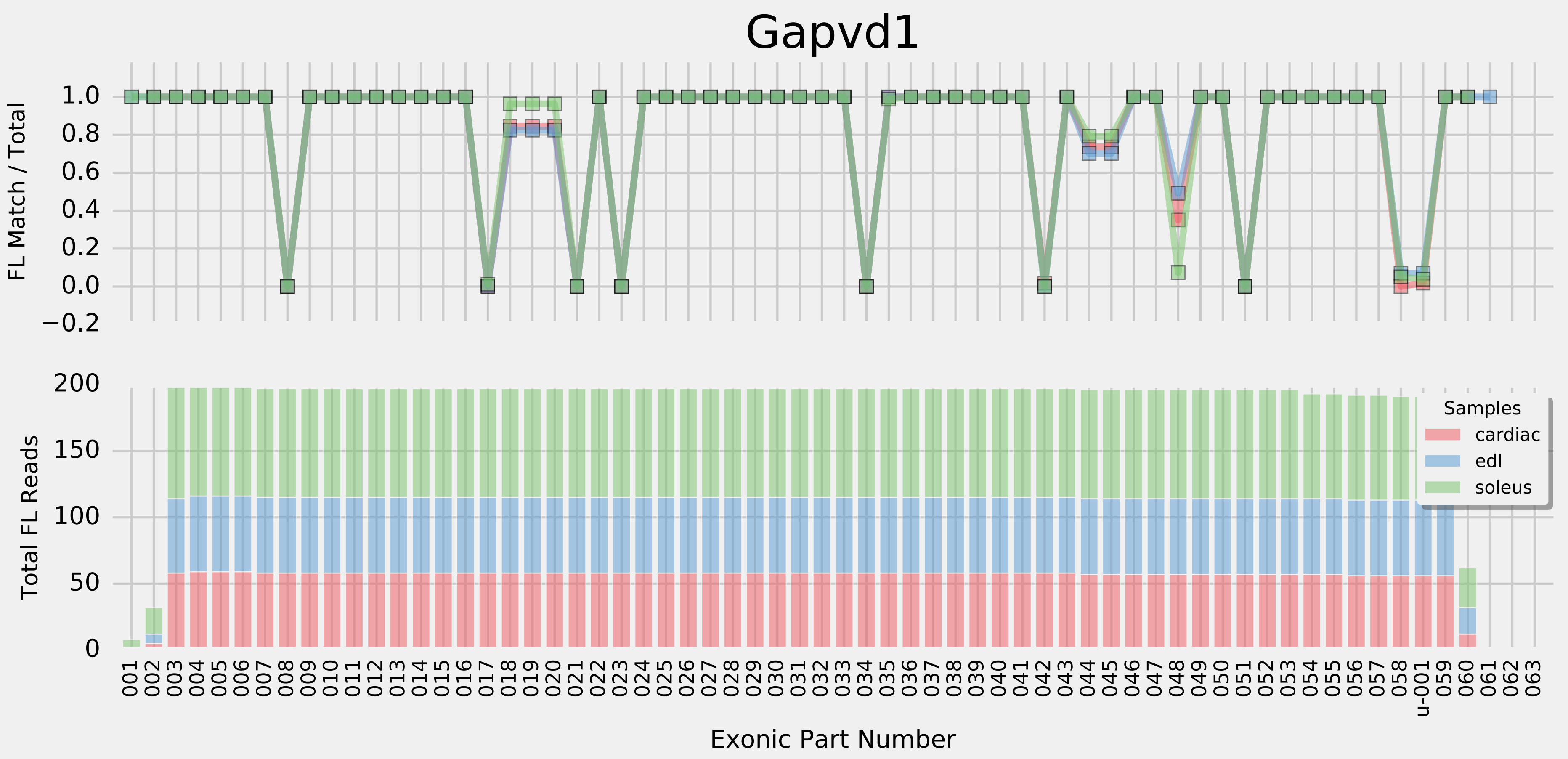

Gapvd1
FL Match / Total
1.0
0.8
0.6
0.4
0.2
0.0
−0.2
200
150
100
50
0
Total FL Reads
Samples
cardiac
edl
soleus
001 002 003 004 005 006 007 008 009 010 011 012 013 014 015 016 017 018 019 020 021 022 023 024 025 026 027 028 029 030 031 032 033 034 035 036 037 038 039 040 041 042 043 044 045 046 047 048 049 050 051 052 053 054 055 056 057 058 u-001 059 060 061 062 063
Exonic Part Number

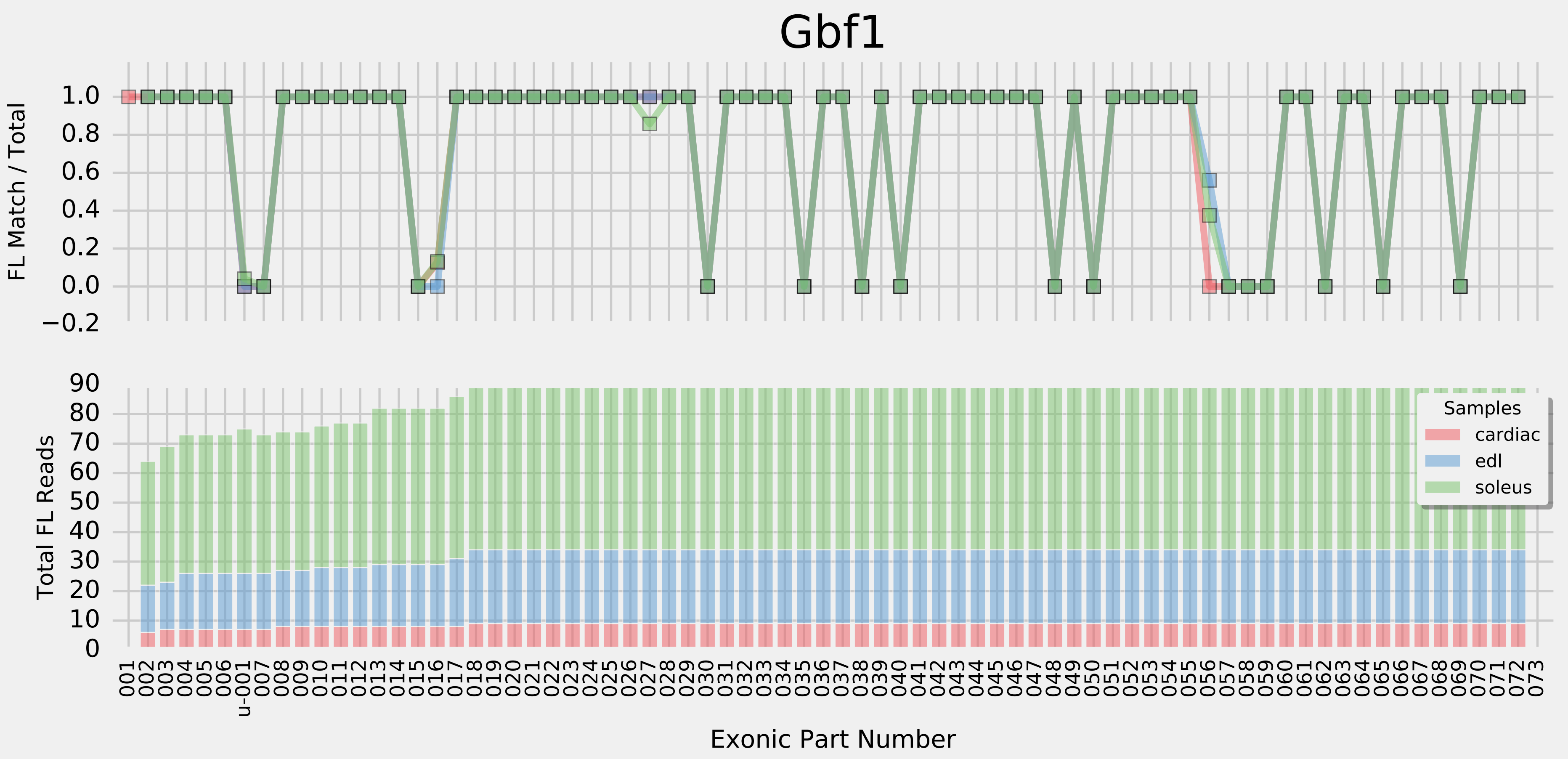

Gbf1
FL Match / Total
Total FL Reads
Exonic Part Number
Samples
cardiac
edl
soleus

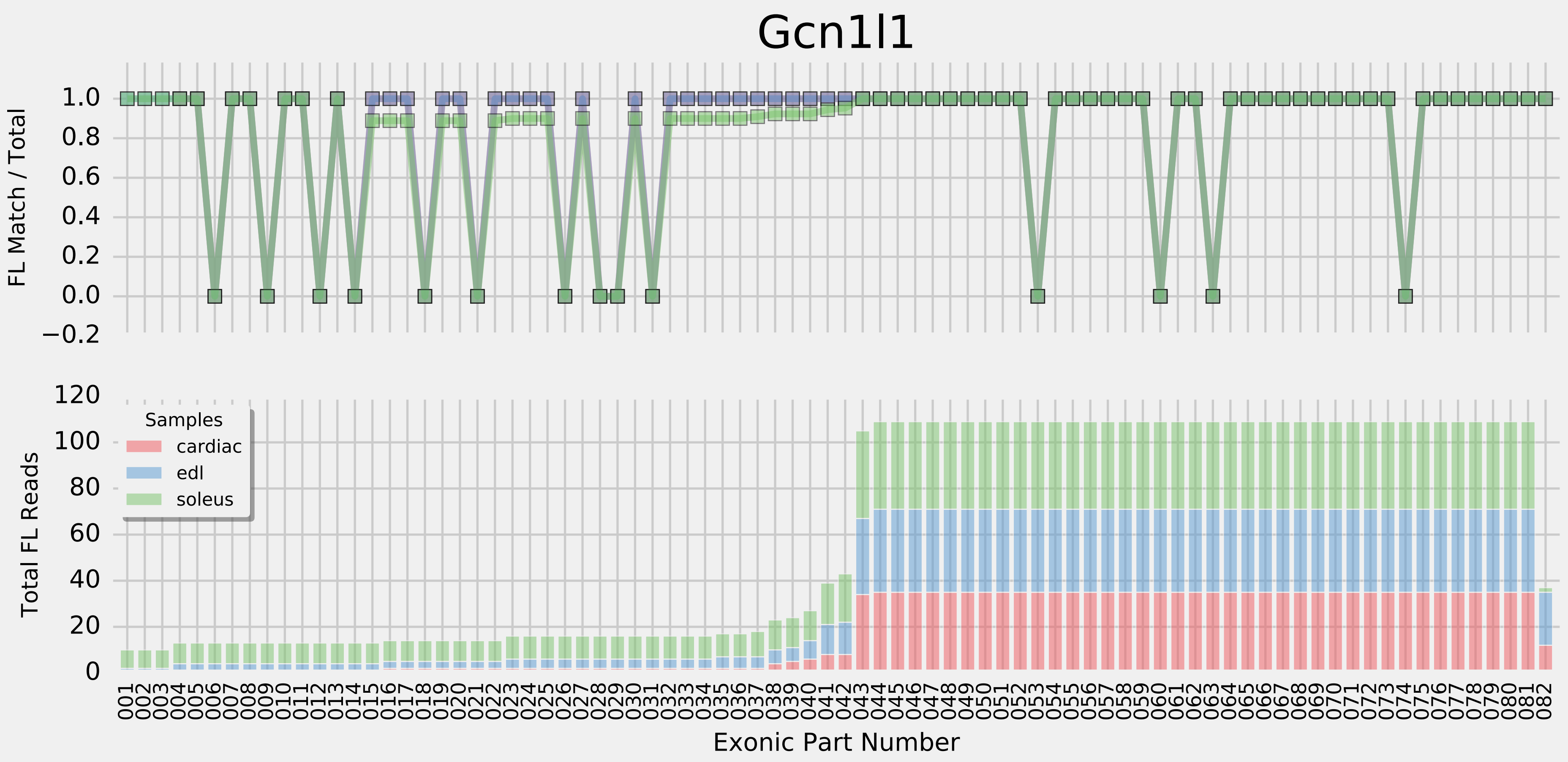

Gcn1l1
FL Match / Total
Total FL Reads
Samples
cardiac
edl
soleus
Exonic Part Number

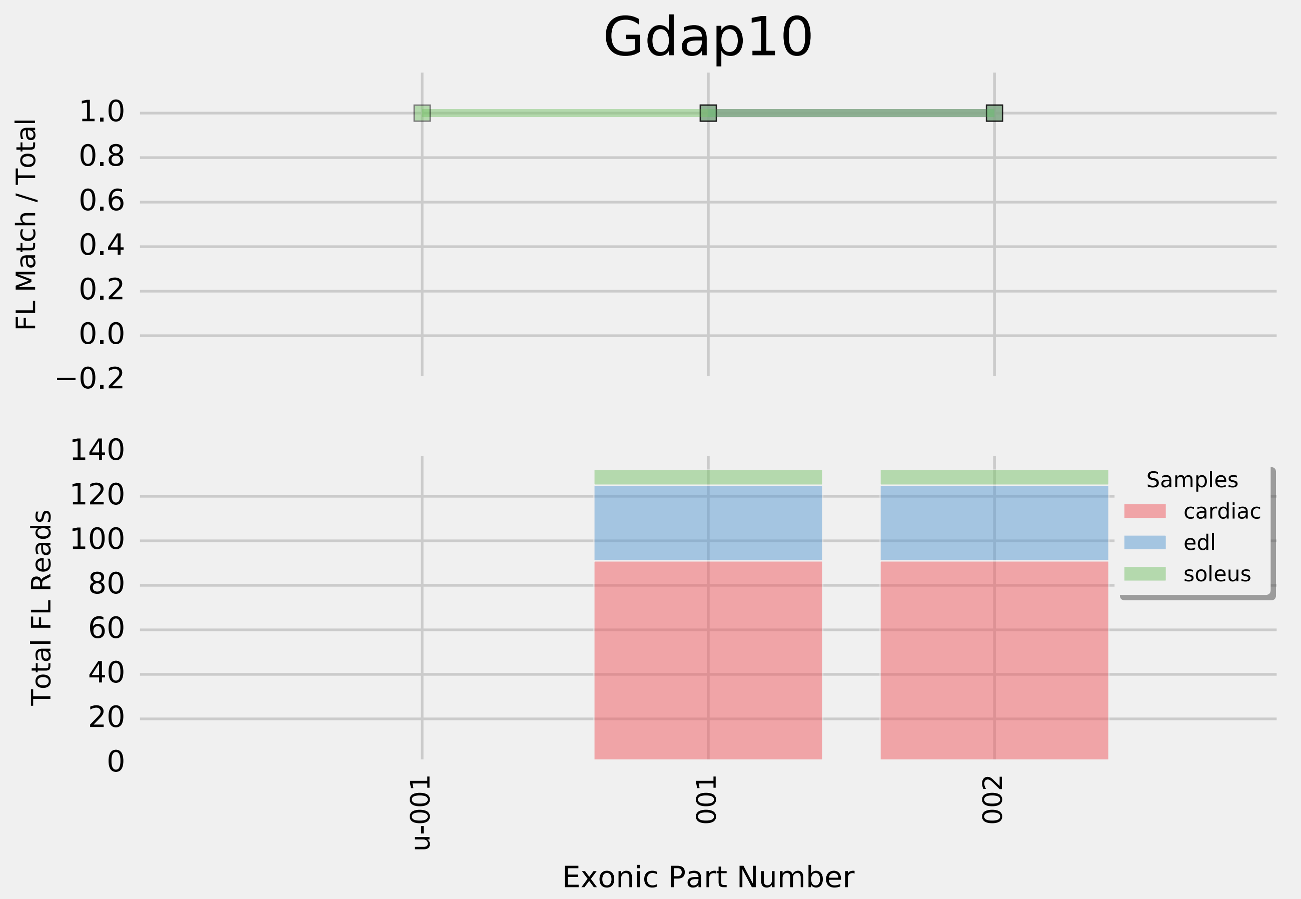
Gdap10
FL Match / Total
1.0
0.8
0.6
0.4
0.2
0.0
−0.2
Total FL Reads
140
120
100
80
60
40
20
0
u-001
001
002
Exonic Part Number
Samples
cardiac
edl
soleus

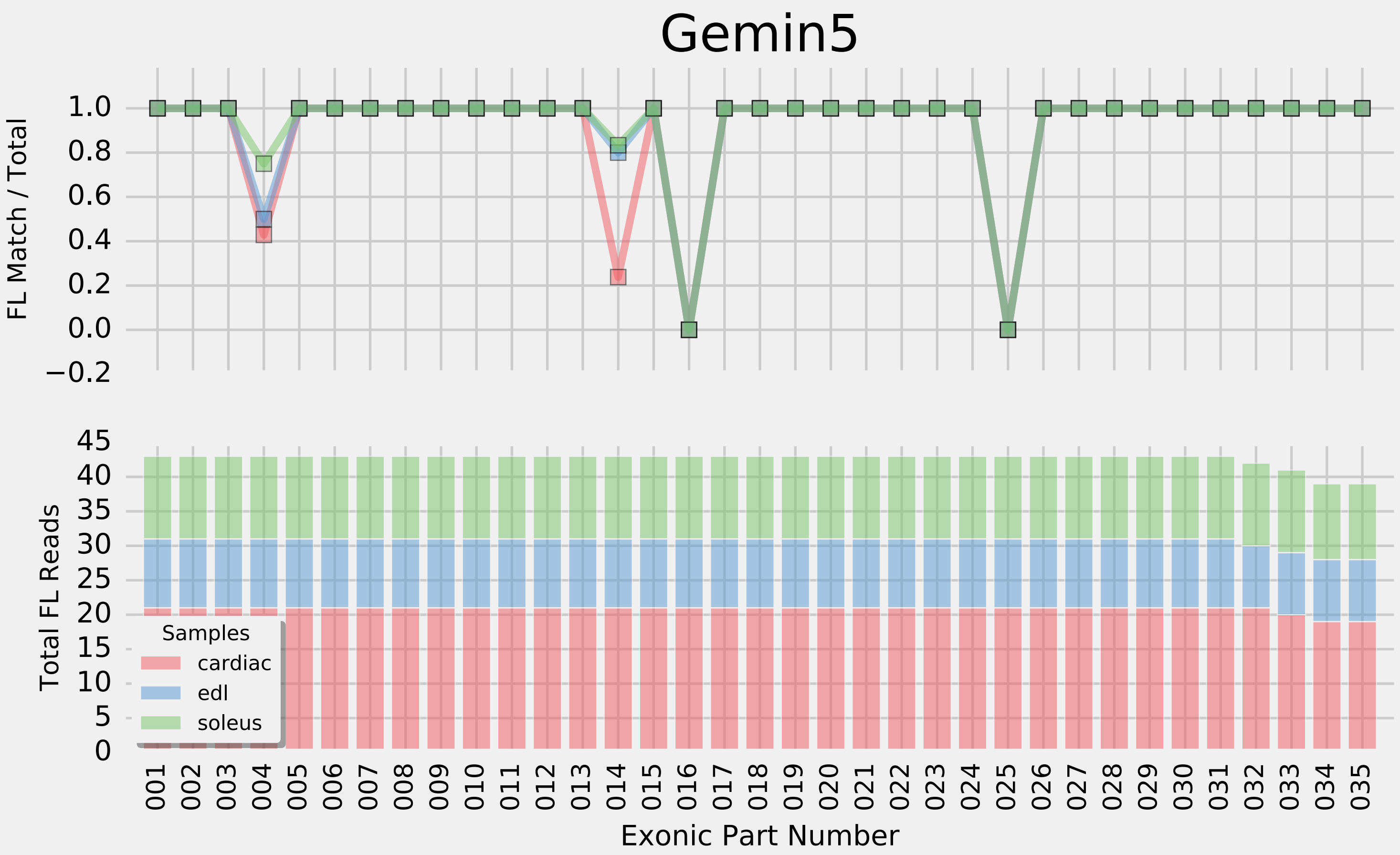

Gemin5
FL Match / Total
1.0
0.8
0.6
0.4
0.2
0.0
−0.2
Total FL Reads
45
40
35
30
25
20
15
10
5
0
Samples
cardiac
edl
soleus
001 002 003 004 005 006 007 008 009 010 011 012 013 014 015 016 017 018 019 020 021 022 023 024 025 026 027 028 029 030 031 032 033 034 035
Exonic Part Number

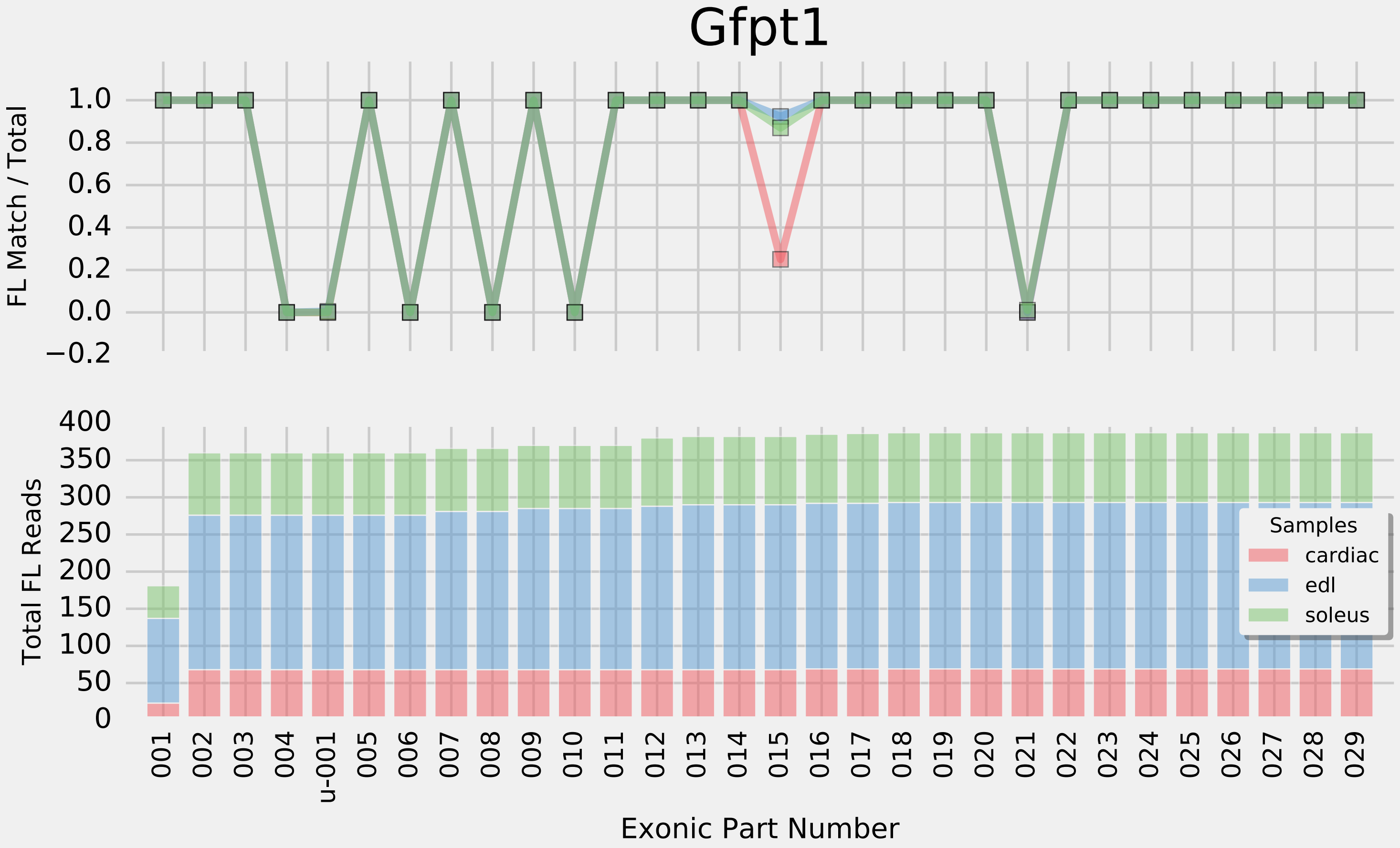

Gfpt1
FL Match / Total
1.0
0.8
0.6
0.4
0.2
0.0
−0.2
Total FL Reads
400
350
300
250
200
150
100
50
0
001
002
003
004
u-001
005
006
007
008
009
010
011
012
013
014
015
016
017
018
019
020
021
022
023
024
025
026
027
028
029
Exonic Part Number
Samples
cardiac
edl
soleus

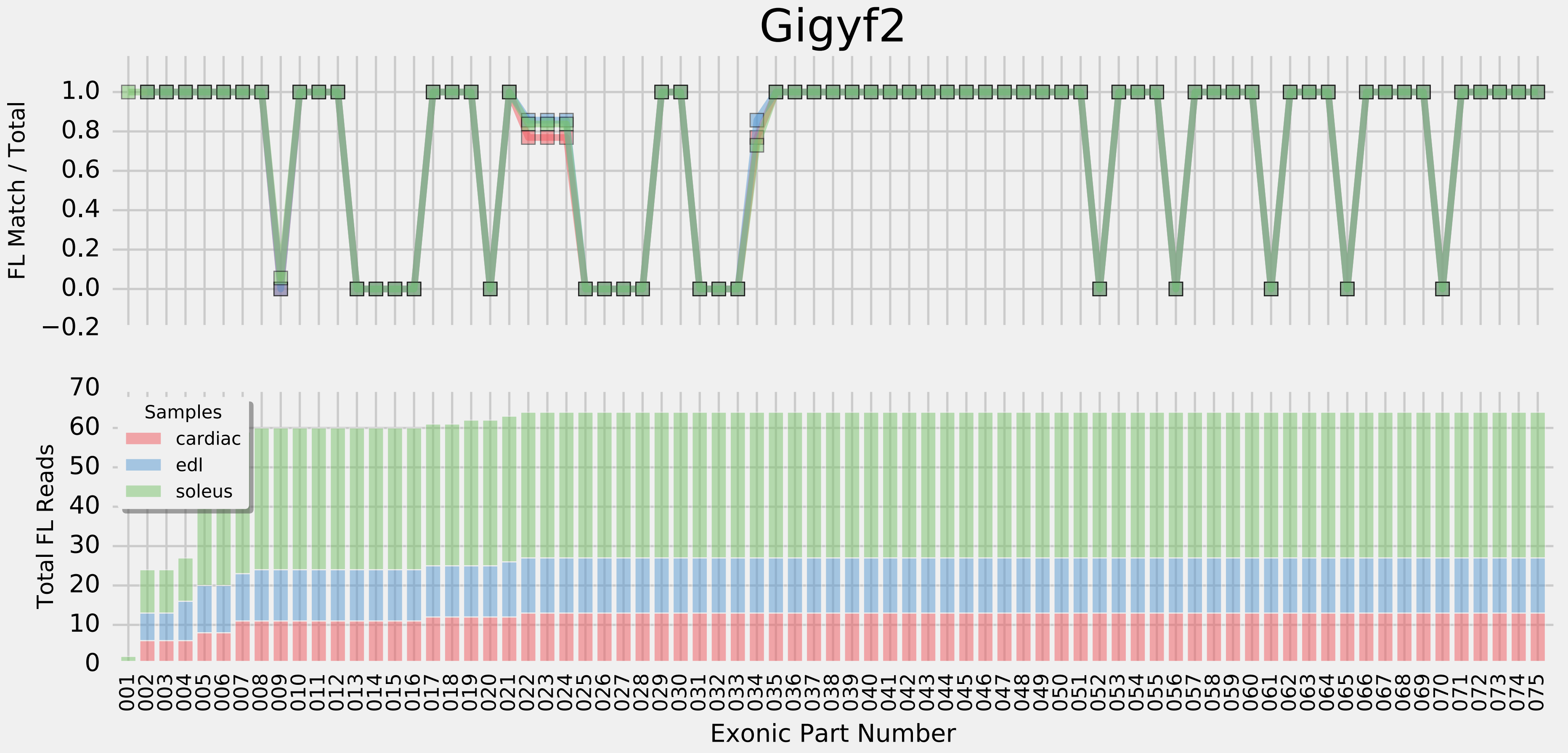

Gigyf2
FL Match / Total
1.0
0.8
0.6
0.4
0.2
0.0
−0.2
Total FL Reads
70
60
50
40
30
20
10
0
Samples
cardiac
edl
soleus
Exonic Part Number
001 002 003 004 005 006 007 008 009 010 011 012 013 014 015 016 017 018 019 020 021 022 023 024 025 026 027 028 029 030 031 032 033 034 035 036 037 038 039 040 041 042 043 044 045 046 047 048 049 050 051 052 053 054 055 056 057 058 059 060 061 062 063 064 065 066 067 068 069 070 071 072 073 074 075

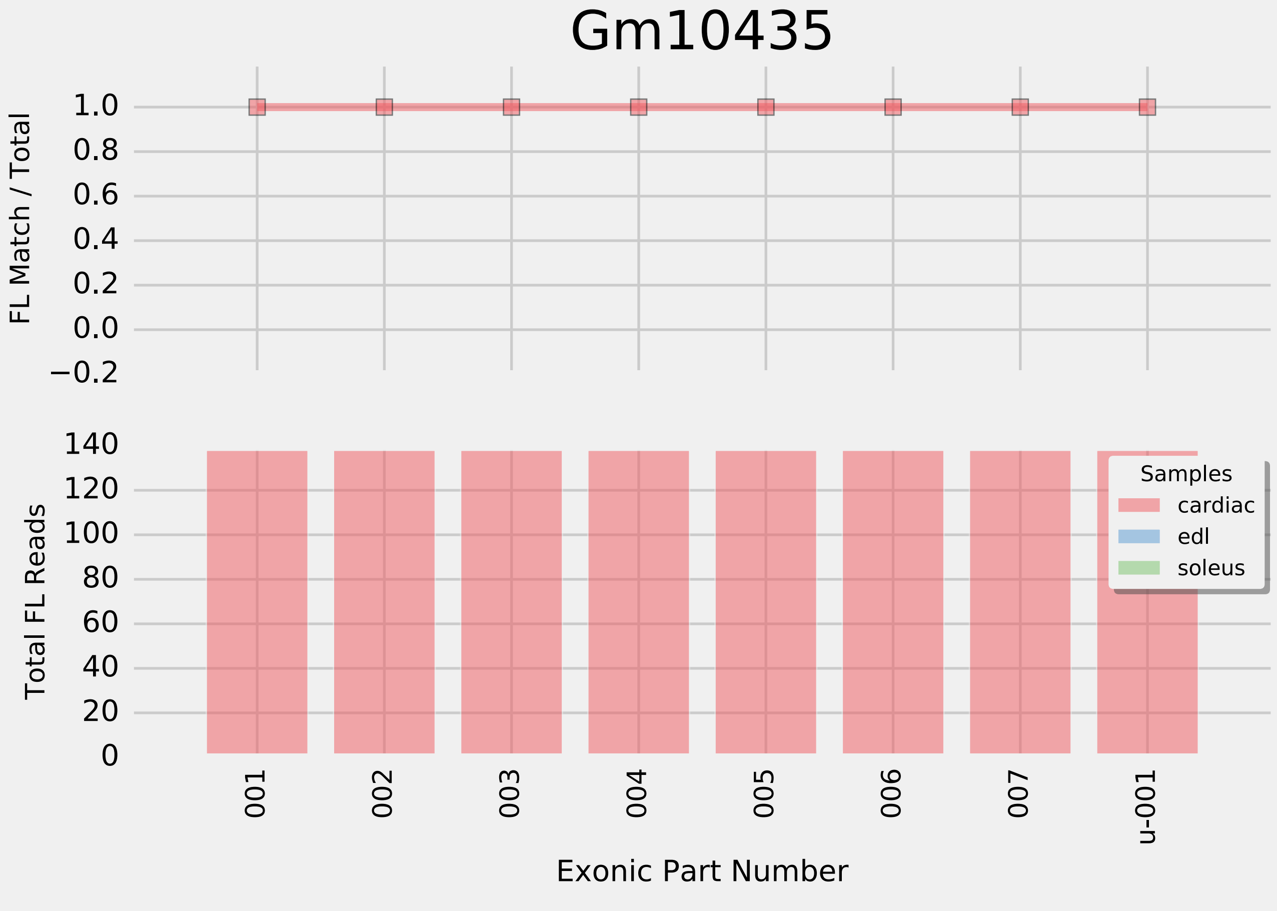
Gm10435
FL Match / Total
1.0
0.8
0.6
0.4
0.2
0.0
−0.2
Total FL Reads
140
120
100
80
60
40
20
0
Samples
cardiac
edl
soleus
001
002
003
004
005
006
007
u-001
Exonic Part Number

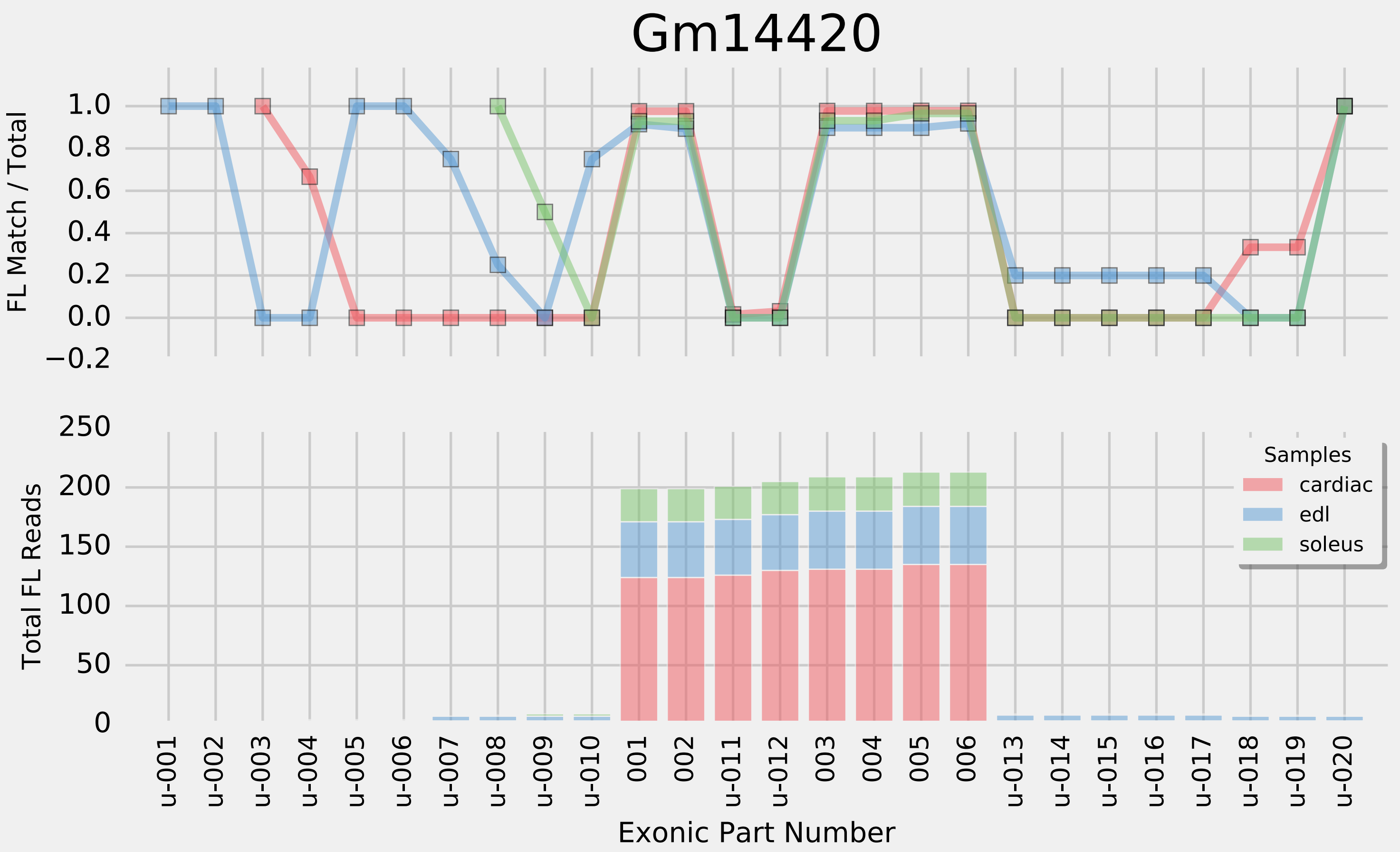

Gm14420
FL Match / Total
1.0
0.8
0.6
0.4
0.2
0.0
−0.2
Total FL Reads
250
200
150
100
50
0
Samples
cardiac
edl
soleus
u-001
u-002
u-003
u-004
u-005
u-006
u-007
u-008
u-009
u-010
001
002
u-011
u-012
003
004
005
006
u-013
u-014
u-015
u-016
u-017
u-018
u-019
u-020
Exonic Part Number

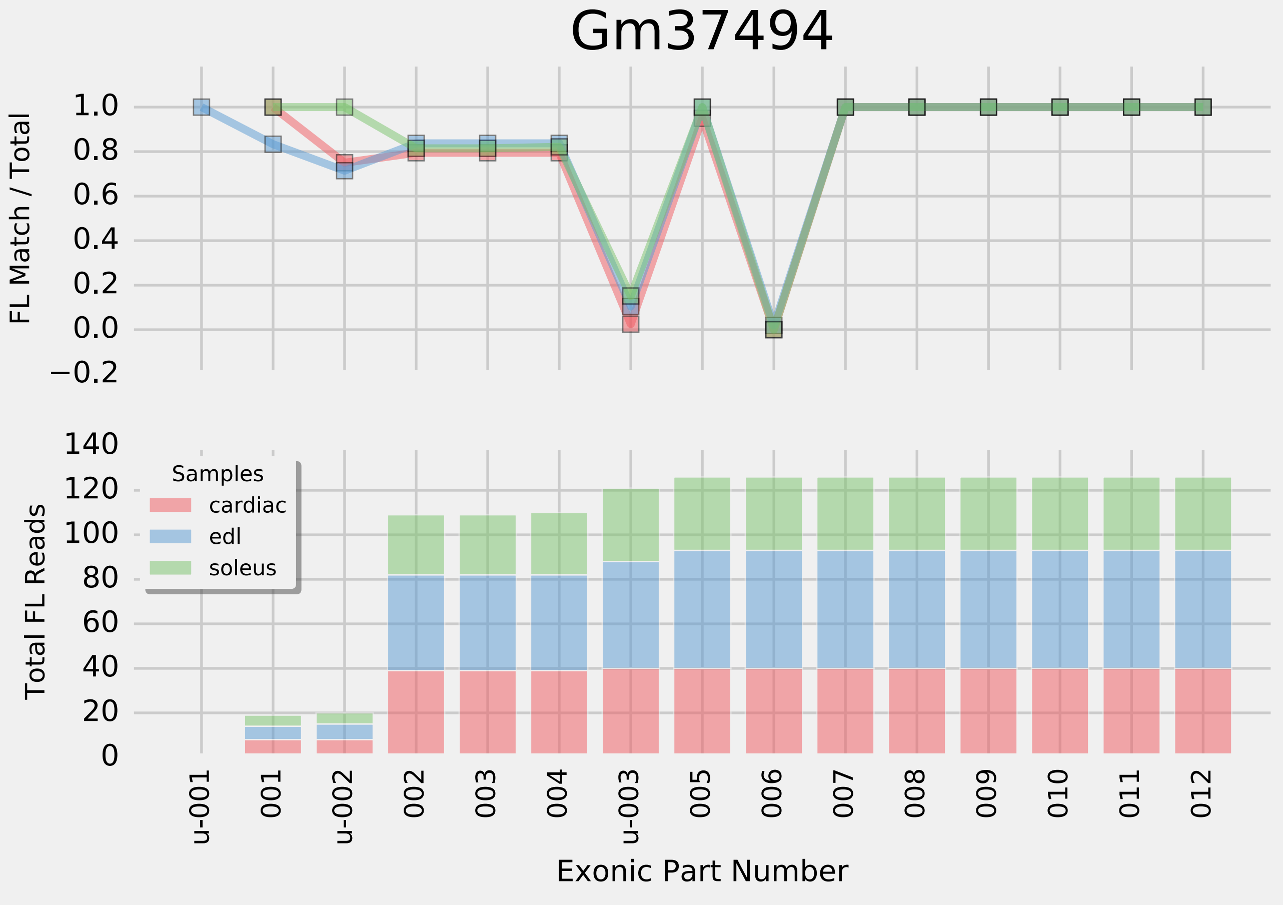

Gm37494
FL Match / Total
1.0
0.8
0.6
0.4
0.2
0.0
−0.2
Total FL Reads
140
120
100
80
60
40
20
0
Samples
cardiac
edl
soleus
u-001
001
u-002
002
003
004
u-003
005
006
007
008
009
010
011
012
Exonic Part Number

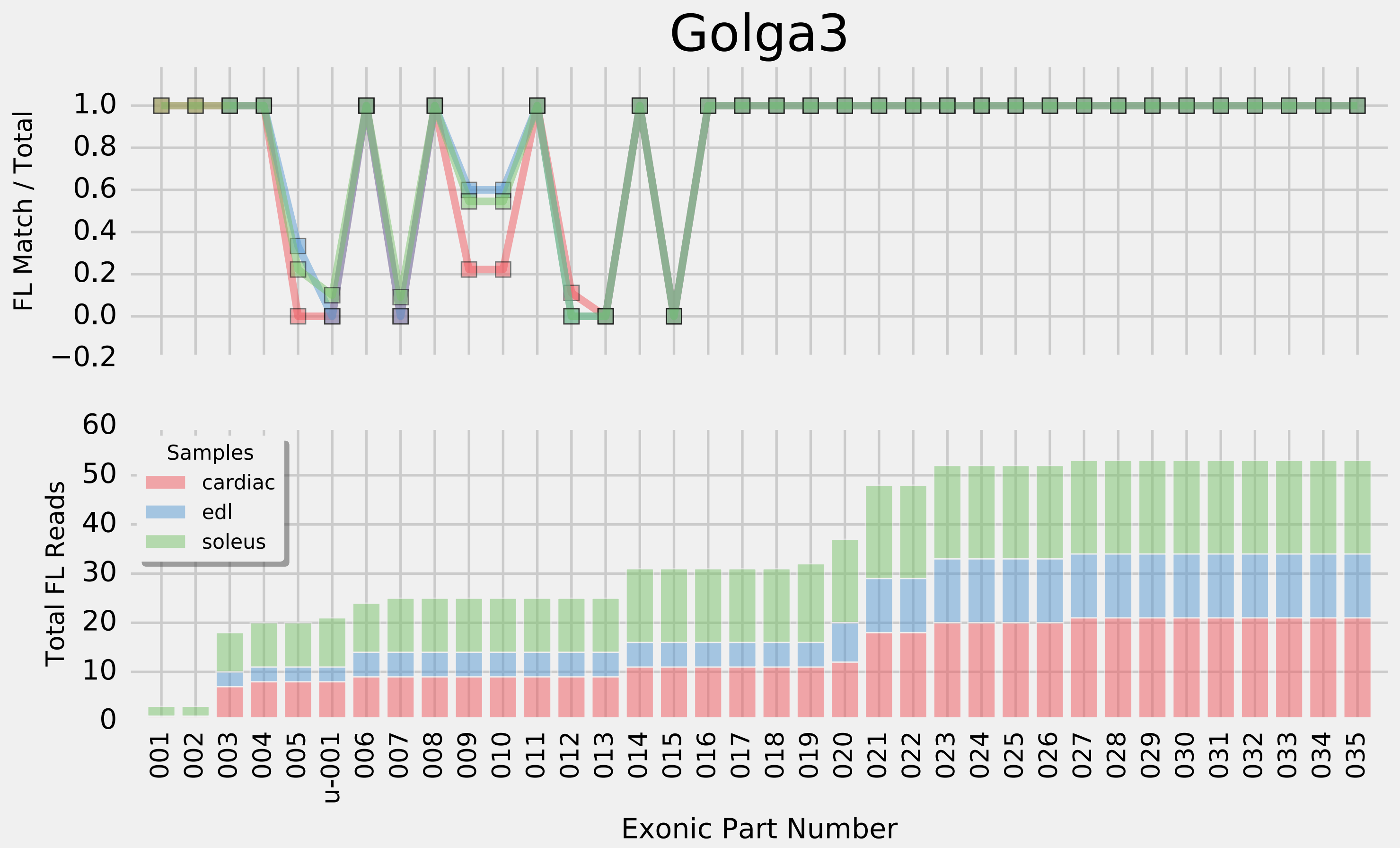

Golga3
FL Match / Total
1.0
0.8
0.6
0.4
0.2
0.0
−0.2
Total FL Reads
60
50
40
30
20
10
0
Samples
cardiac
edl
soleus
001
002
003
004
005
u-001
006
007
008
009
010
011
012
013
014
015
016
017
018
019
020
021
022
023
024
025
026
027
028
029
030
031
032
033
034
035
Exonic Part Number

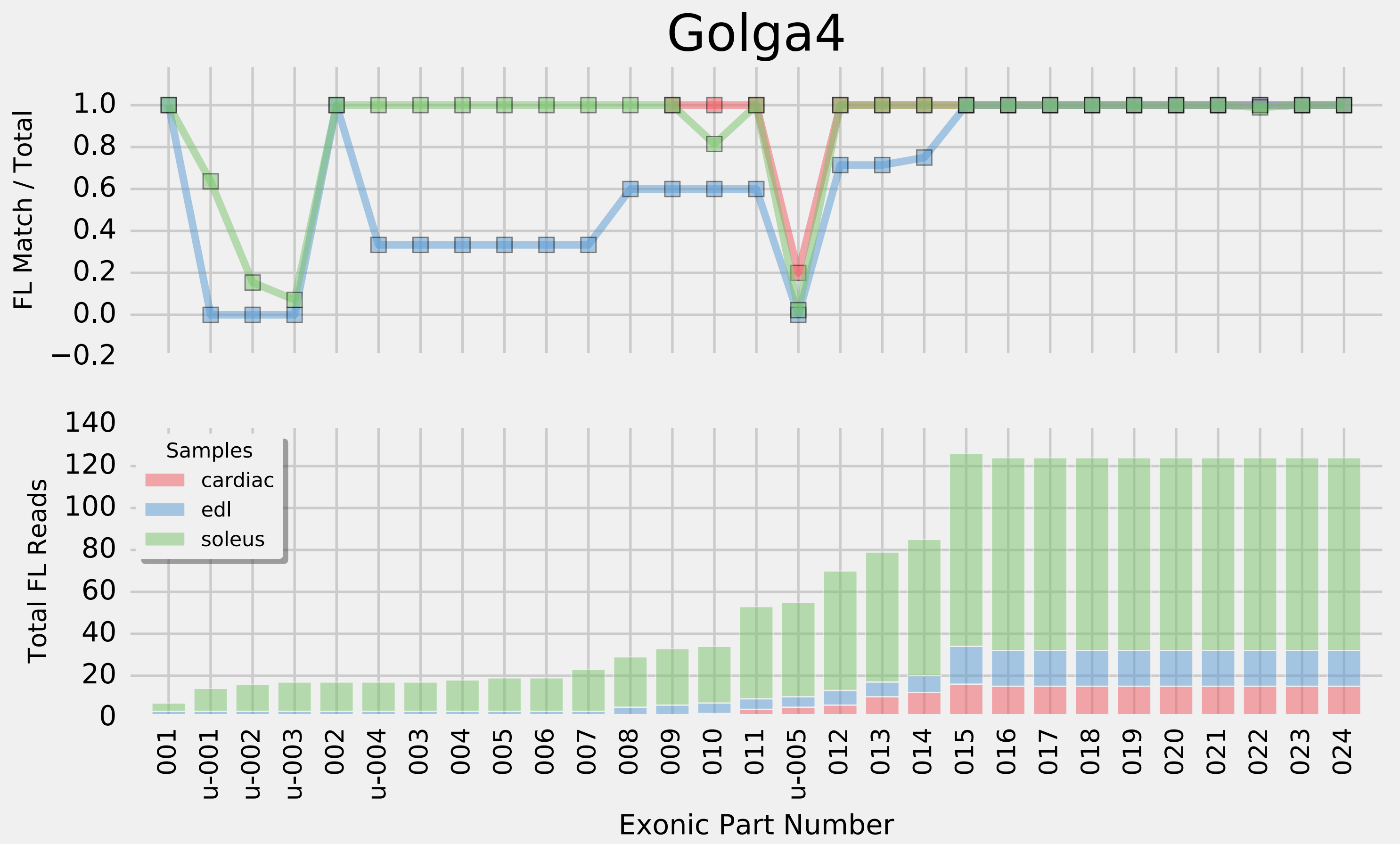

Golga4
FL Match / Total
1.0
0.8
0.6
0.4
0.2
0.0
−0.2
Total FL Reads
140
120
100
80
60
40
20
0
Samples
cardiac
edl
soleus
001
u-001
u-002
u-003
002
u-004
003
004
005
006
007
008
009
010
011
u-005
012
013
014
015
016
017
018
019
020
021
022
023
024
Exonic Part Number

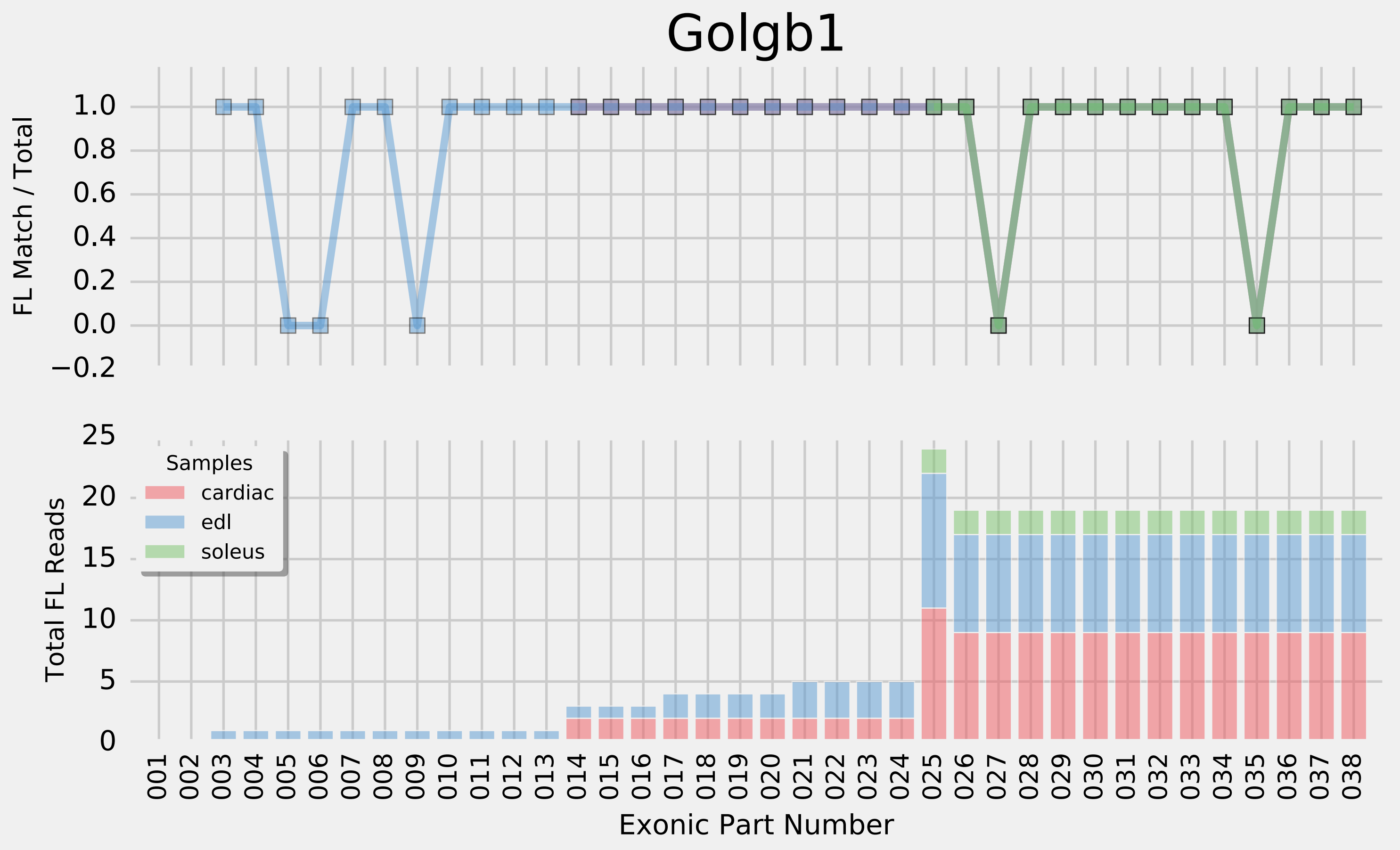
Golgb1
FL Match / Total
1.0
0.8
0.6
0.4
0.2
0.0
−0.2
Total FL Reads
25
20
15
10
5
0
Samples
cardiac
edl
soleus
001
002
003
004
005
006
007
008
009
010
011
012
013
014
015
016
017
018
019
020
021
022
023
024
025
026
027
028
029
030
031
032
033
034
035
036
037
038
Exonic Part Number

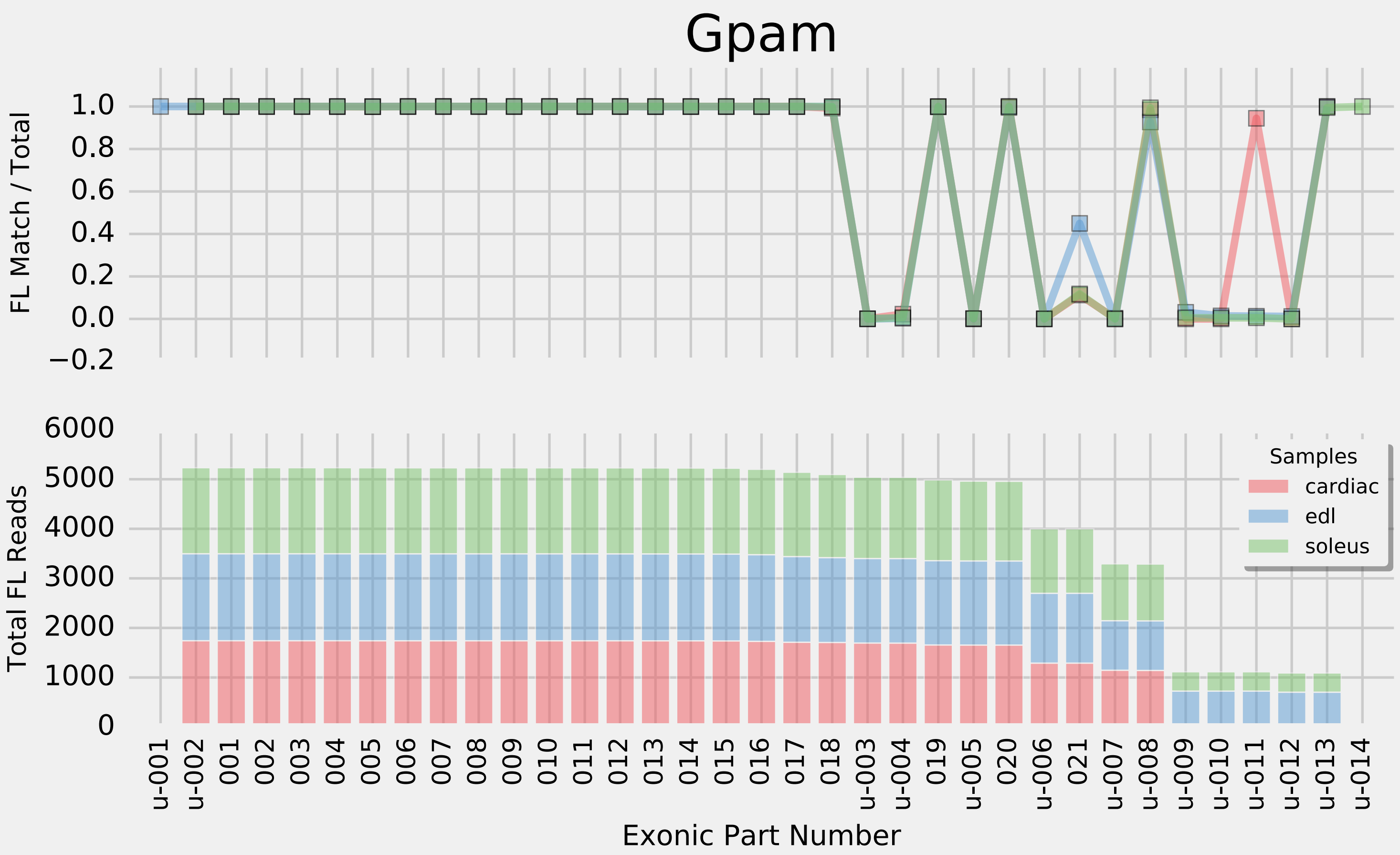

Gpam
FL Match / Total
1.0
0.8
0.6
0.4
0.2
0.0
−0.2
6000
5000
4000
3000
2000
1000
0
Total FL Reads
Samples
cardiac
edl
soleus
u-001
u-002
001
002
003
004
005
006
007
008
009
010
011
012
013
014
015
016
017
018
u-003
u-004
019
u-005
020
u-006
021
u-007
u-008
u-009
u-010
u-011
u-012
u-013
u-014
Exonic Part Number

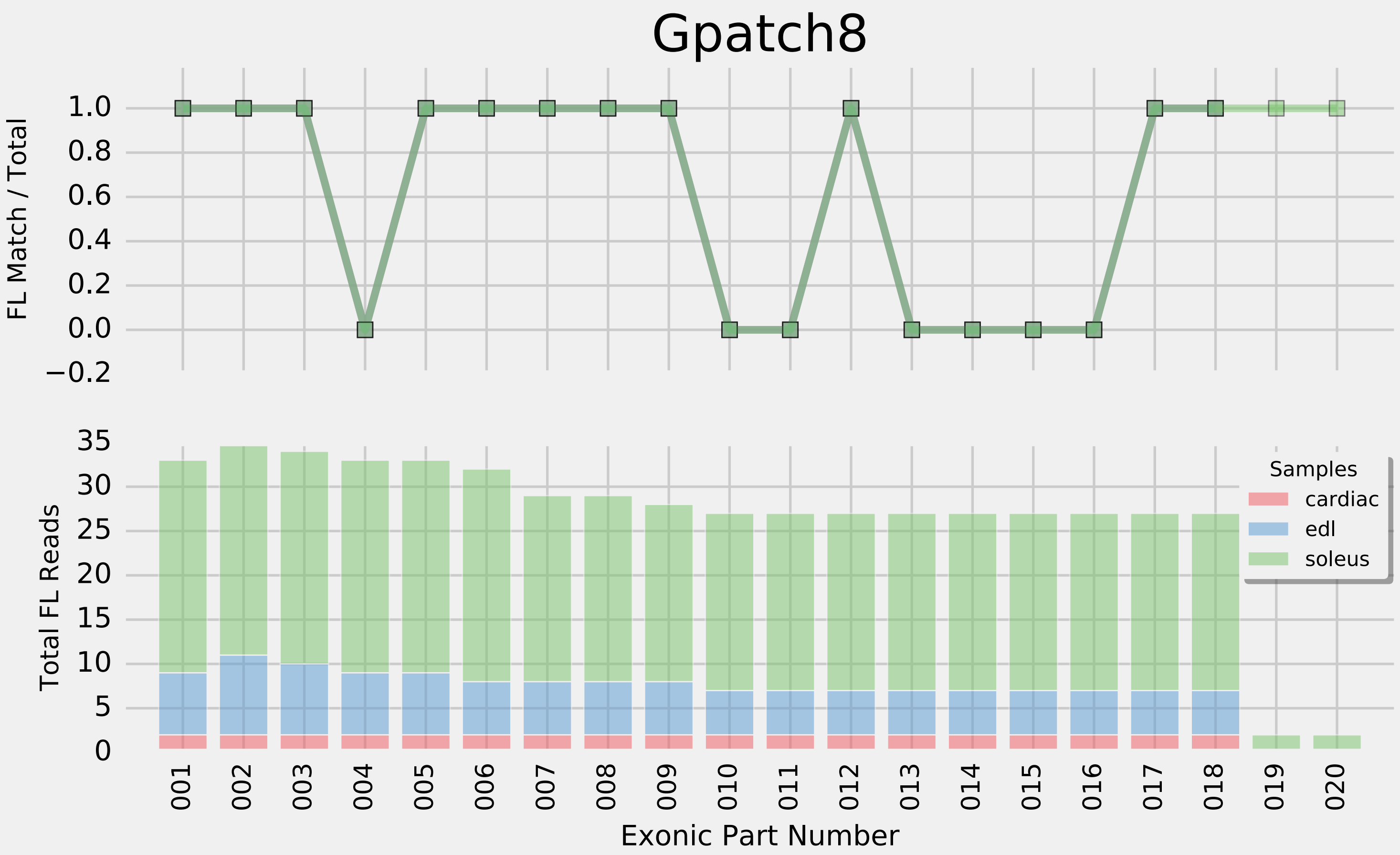
Gpatch8
FL Match / Total
1.0
0.8
0.6
0.4
0.2
0.0
−0.2
Total FL Reads
35
30
25
20
15
10
5
0
Samples
cardiac
edl
soleus
001
002
003
004
005
006
007
008
009
010
011
012
013
014
015
016
017
018
019
020
Exonic Part Number

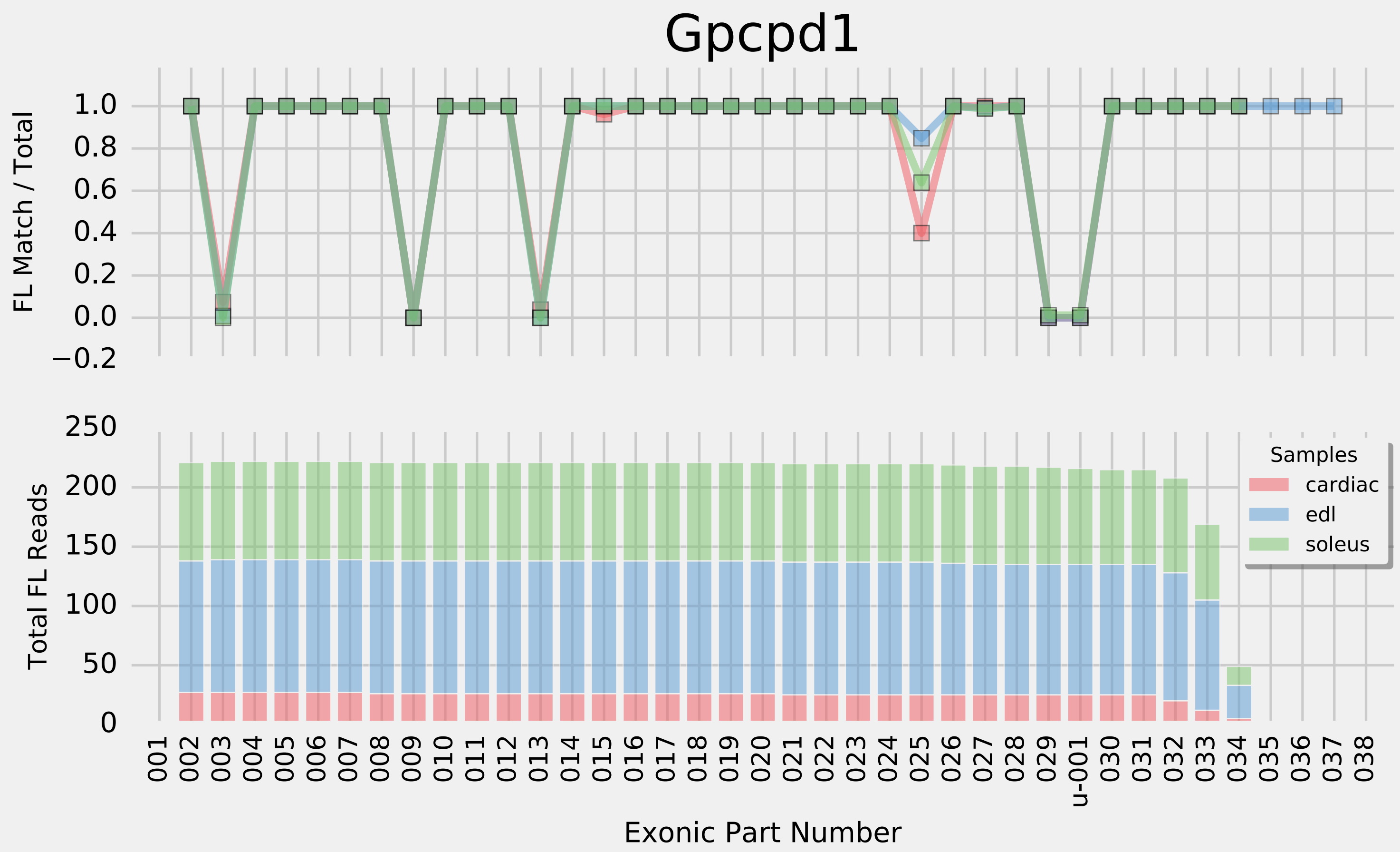

Gpcpd1
FL Match / Total
1.0
0.8
0.6
0.4
0.2
0.0
−0.2
250
200
150
100
50
0
Total FL Reads
Samples
cardiac
edl
soleus
001
002
003
004
005
006
007
008
009
010
011
012
013
014
015
016
017
018
019
020
021
022
023
024
025
026
027
028
029
u-001
030
031
032
033
034
035
036
037
038
Exonic Part Number

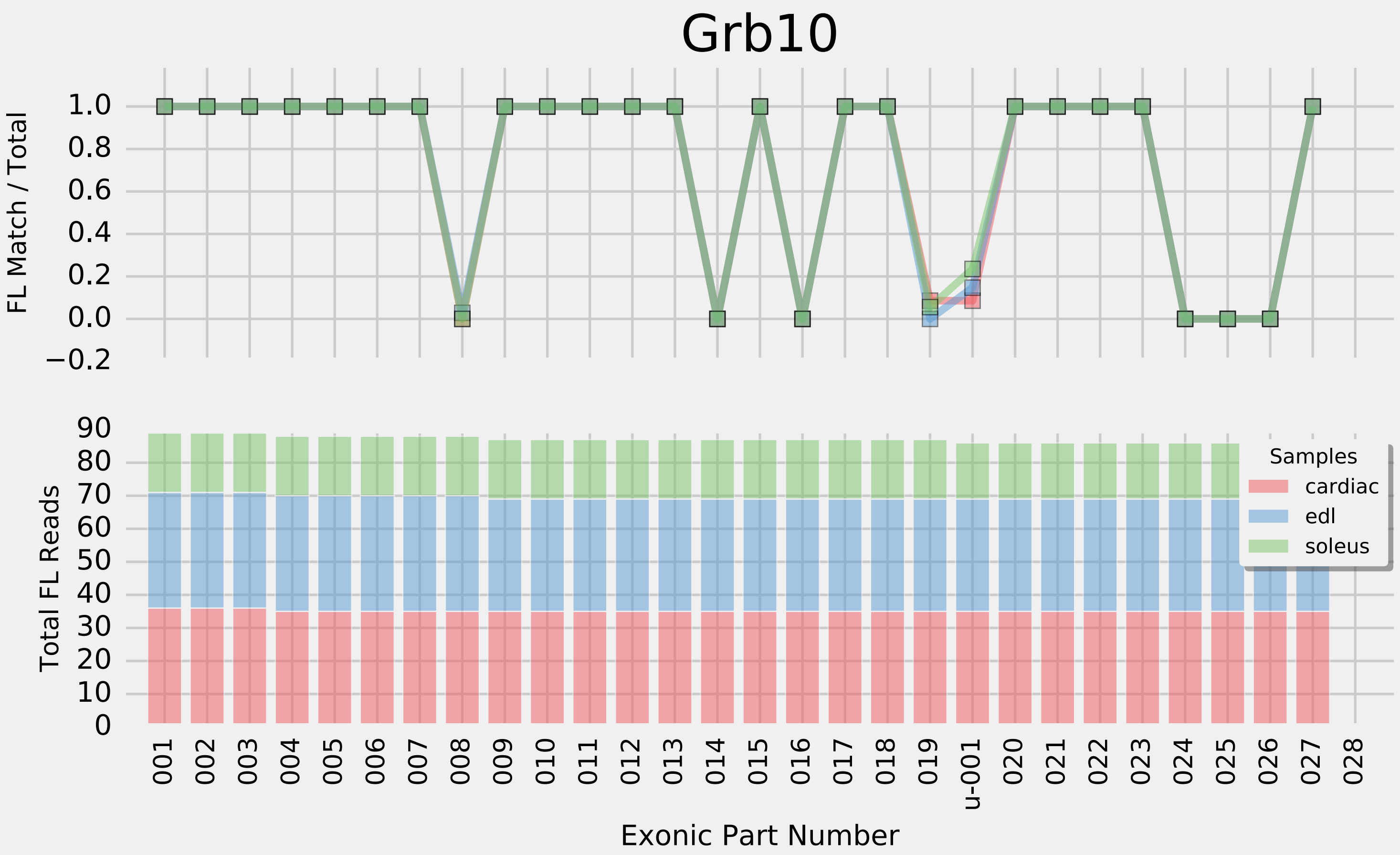

Grb10
FL Match / Total
1.0
0.8
0.6
0.4
0.2
0.0
−0.2
Total FL Reads
90
80
70
60
50
40
30
20
10
0
Samples
cardiac
edl
soleus
001
002
003
004
005
006
007
008
009
010
011
012
013
014
015
016
017
018
019
u-001
020
021
022
023
024
025
026
027
028
Exonic Part Number

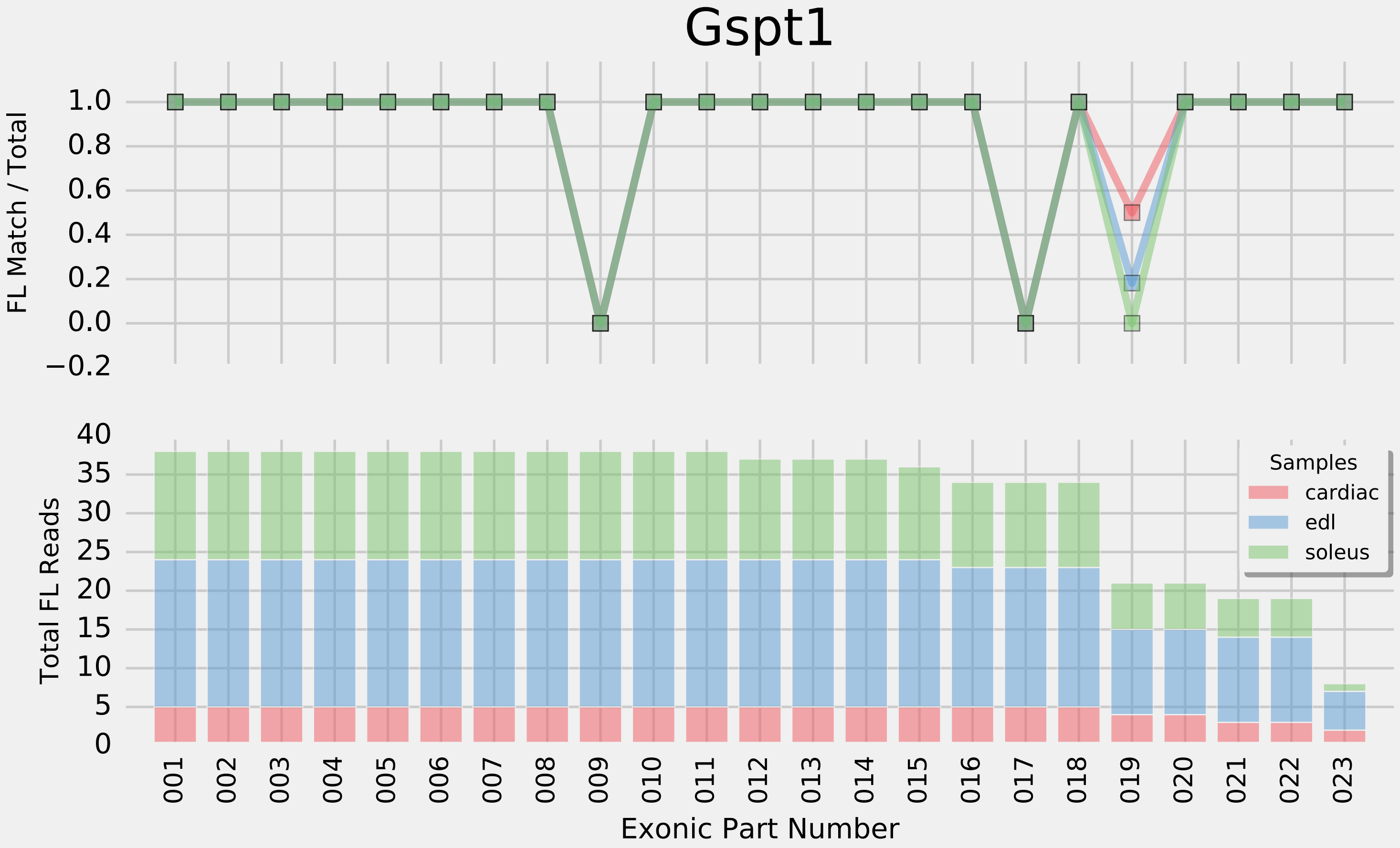
Gspt1
FL Match / Total
1.0
0.8
0.6
0.4
0.2
0.0
−0.2
Total FL Reads
40
35
30
25
20
15
10
5
0
Samples
cardiac
edl
soleus
001 002 003 004 005 006 007 008 009 010 011 012 013 014 015 016 017 018 019 020 021 022 023
Exonic Part Number

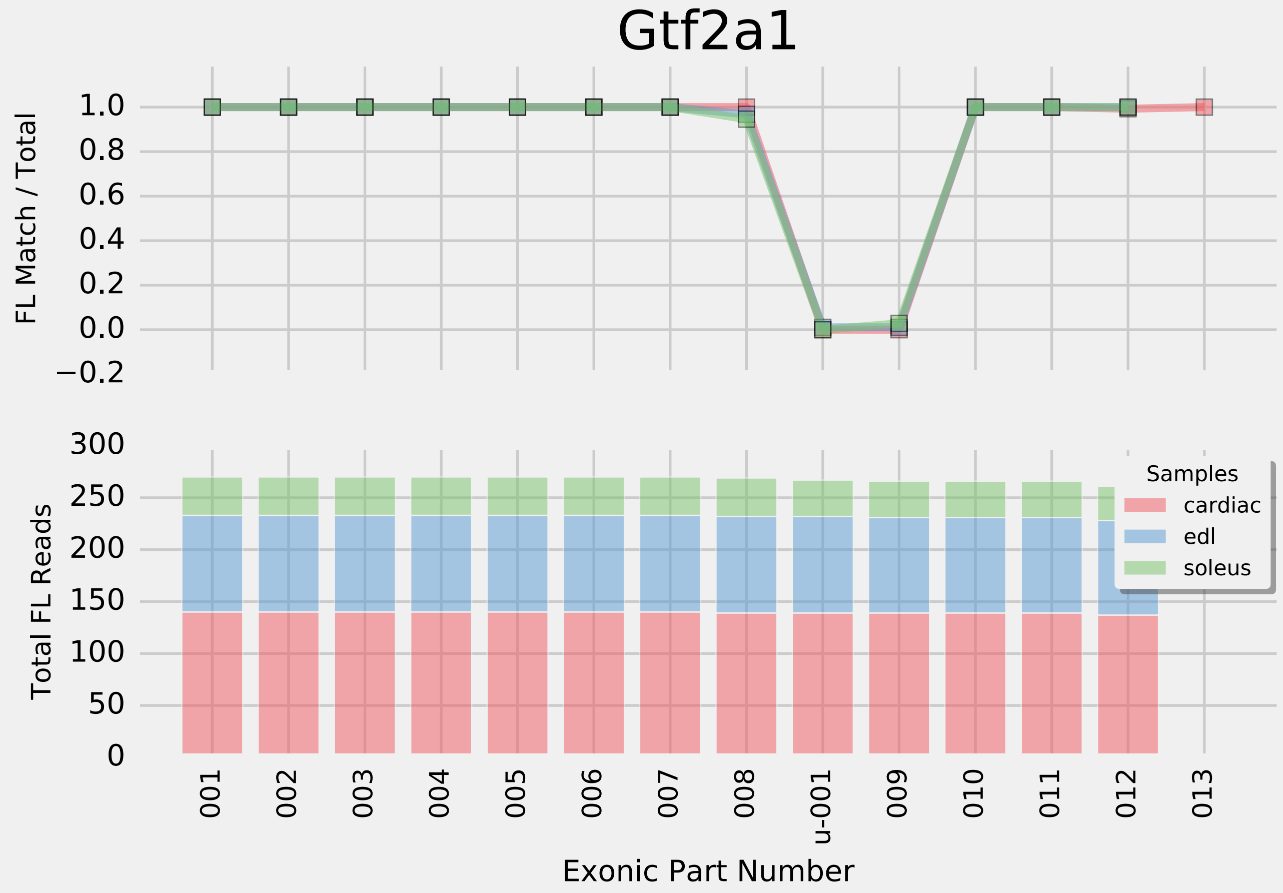
Gtf2a1
FL Match / Total
1.0
0.8
0.6
0.4
0.2
0.0
−0.2
Total FL Reads
300
250
200
150
100
50
0
Samples
cardiac
edl
soleus
001
002
003
004
005
006
007
008
u-001
009
010
011
012
013
Exonic Part Number

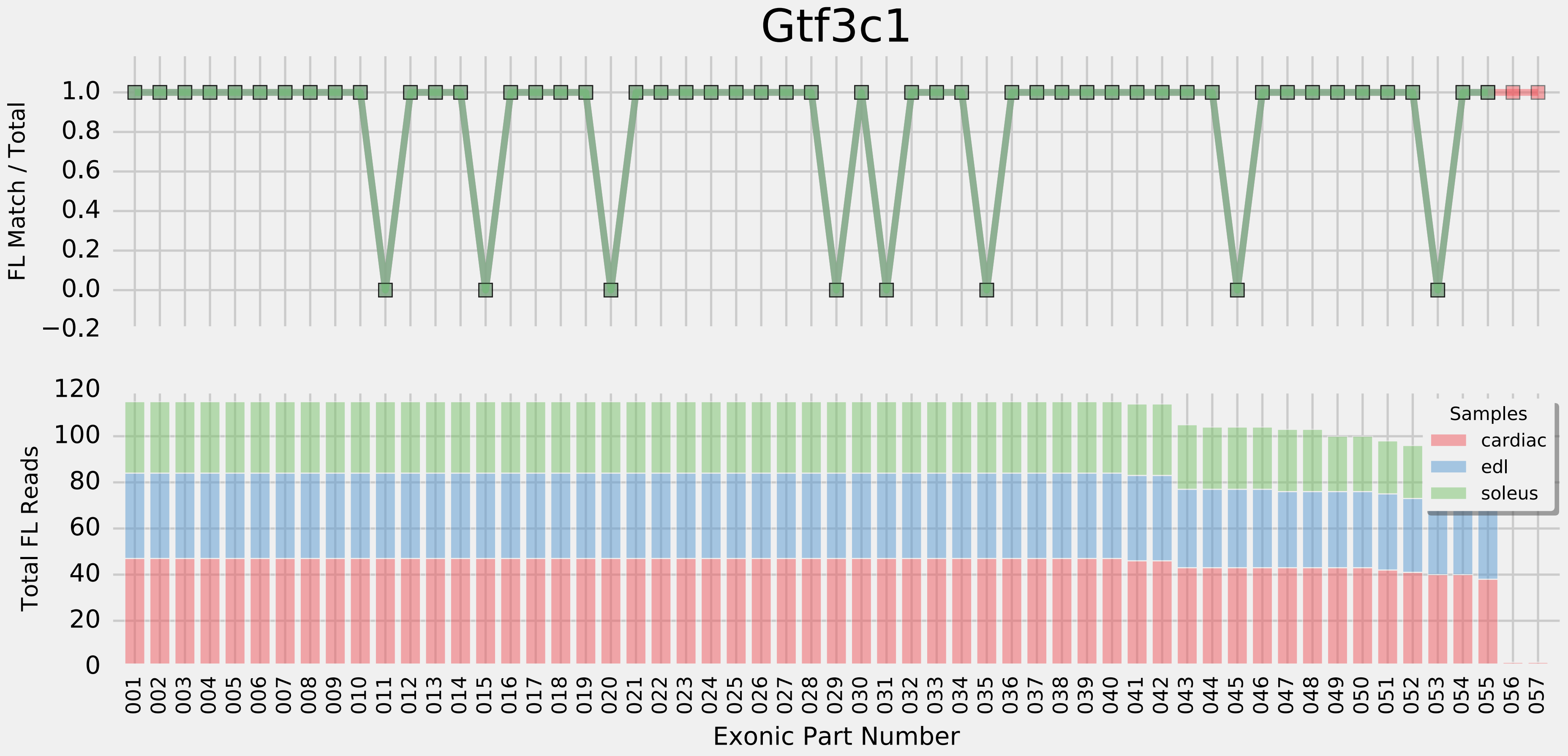

Gtf3c1
FL Match / Total
1.0
0.8
0.6
0.4
0.2
0.0
−0.2
Total FL Reads
120
100
80
60
40
20
0
Samples
cardiac
edl
soleus
001 002 003 004 005 006 007 008 009 010 011 012 013 014 015 016 017 018 019 020 021 022 023 024 025 026 027 028 029 030 031 032 033 034 035 036 037 038 039 040 041 042 043 044 045 046 047 048 049 050 051 052 053 054 055 056 057
Exonic Part Number

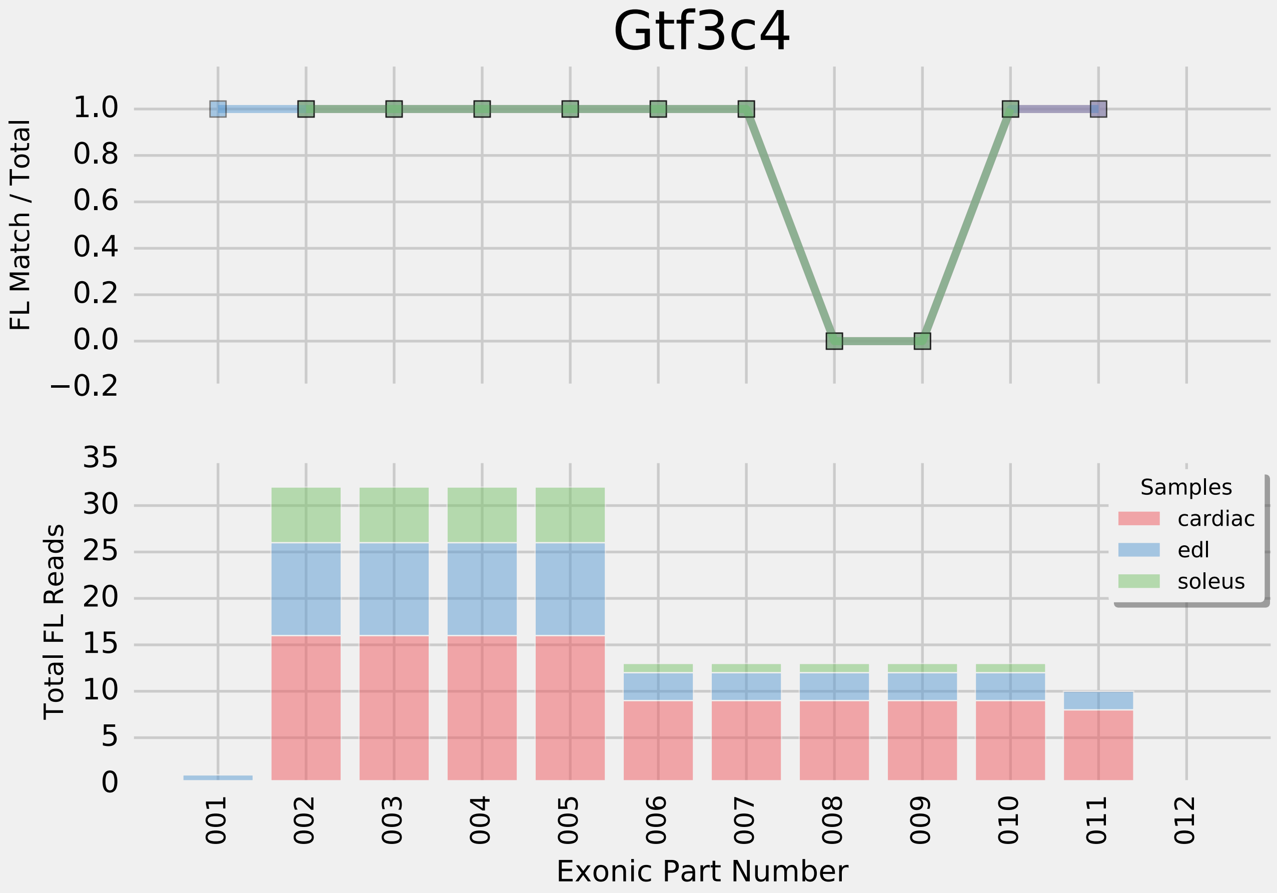

Gtf3c4
FL Match / Total
1.0
0.8
0.6
0.4
0.2
0.0
−0.2
Total FL Reads
35
30
25
20
15
10
5
0
Samples
cardiac
edl
soleus
001
002
003
004
005
006
007
008
009
010
011
012
Exonic Part Number

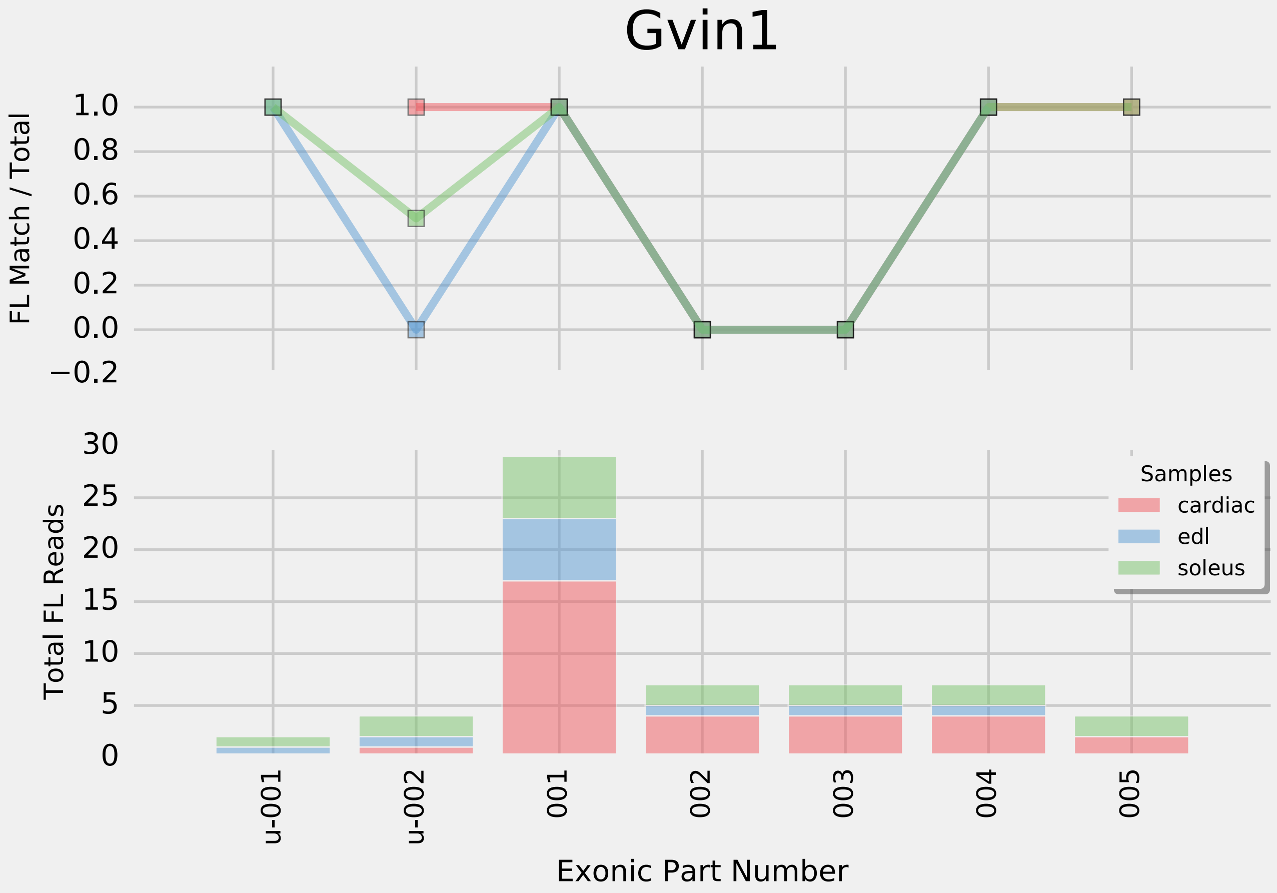
Gvin1
FL Match / Total
1.0
0.8
0.6
0.4
0.2
0.0
−0.2
Total FL Reads
30
25
20
15
10
5
0
Samples
cardiac
edl
soleus
u-001
u-002
001
002
003
004
005
Exonic Part Number

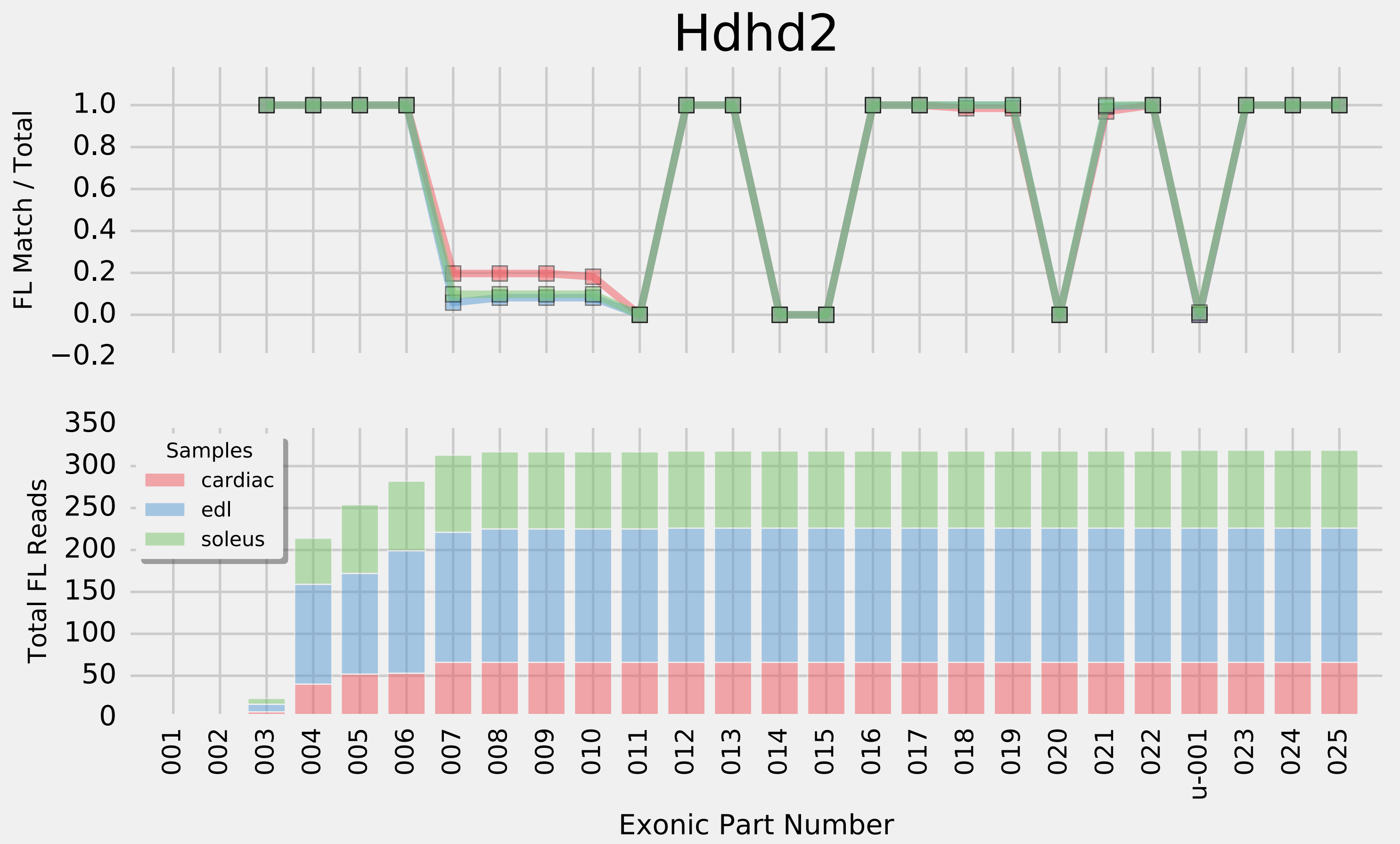

Hdhd2
FL Match / Total
1.0
0.8
0.6
0.4
0.2
0.0
−0.2
Total FL Reads
350
300
250
200
150
100
50
0
Samples
cardiac
edl
soleus
001
002
003
004
005
006
007
008
009
010
011
012
013
014
015
016
017
018
019
020
021
022
u-001
023
024
025
Exonic Part Number

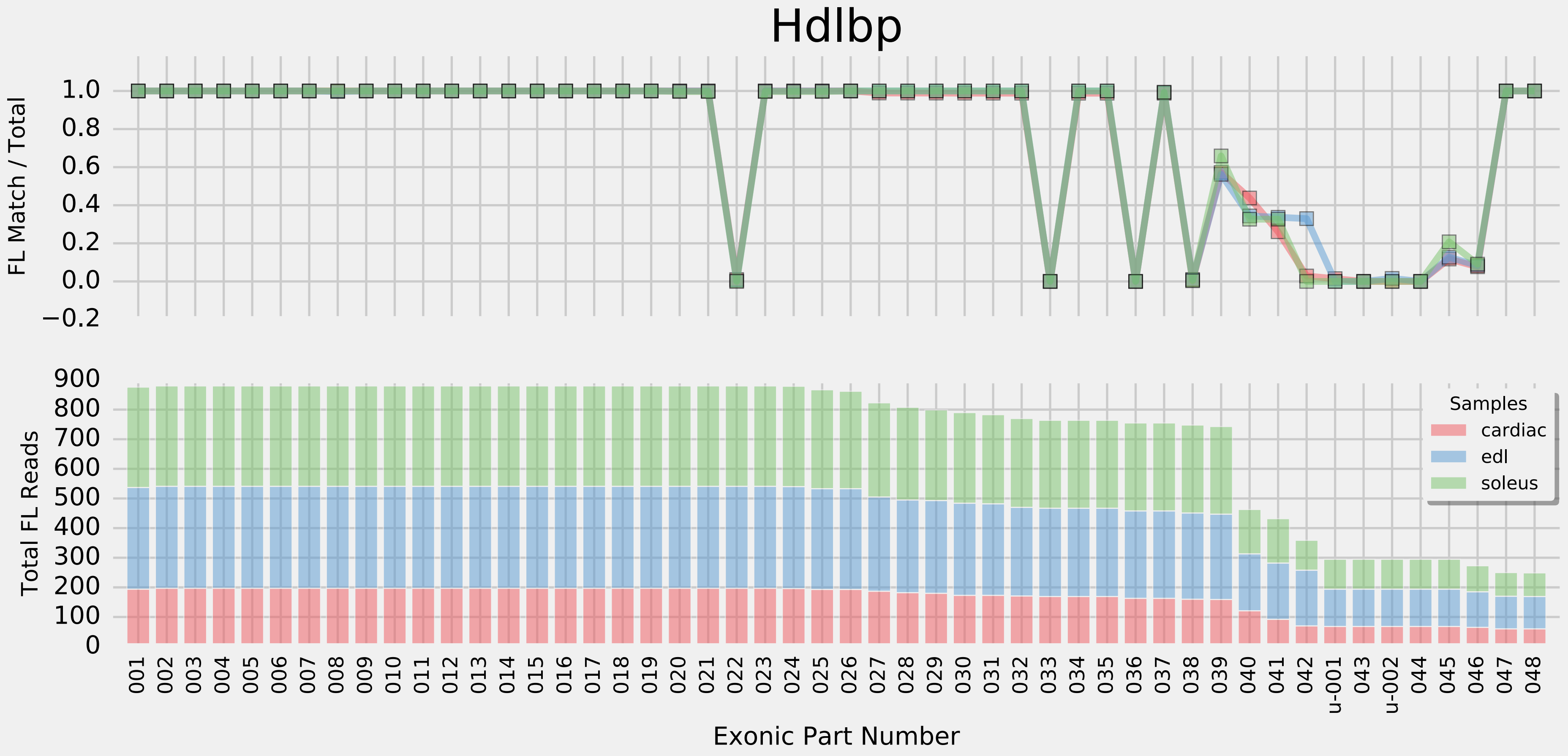
Hdlbp
FL Match / Total
1.0
0.8
0.6
0.4
0.2
0.0
−0.2
Total FL Reads
900
800
700
600
500
400
300
200
100
0
Samples
cardiac
edl
soleus
001 002 003 004 005 006 007 008 009 010 011 012 013 014 015 016 017 018 019 020 021 022 023 024 025 026 027 028 029 030 031 032 033 034 035 036 037 038 039 040 041 042 u-001 043 u-002 044 045 046 047 048
Exonic Part Number

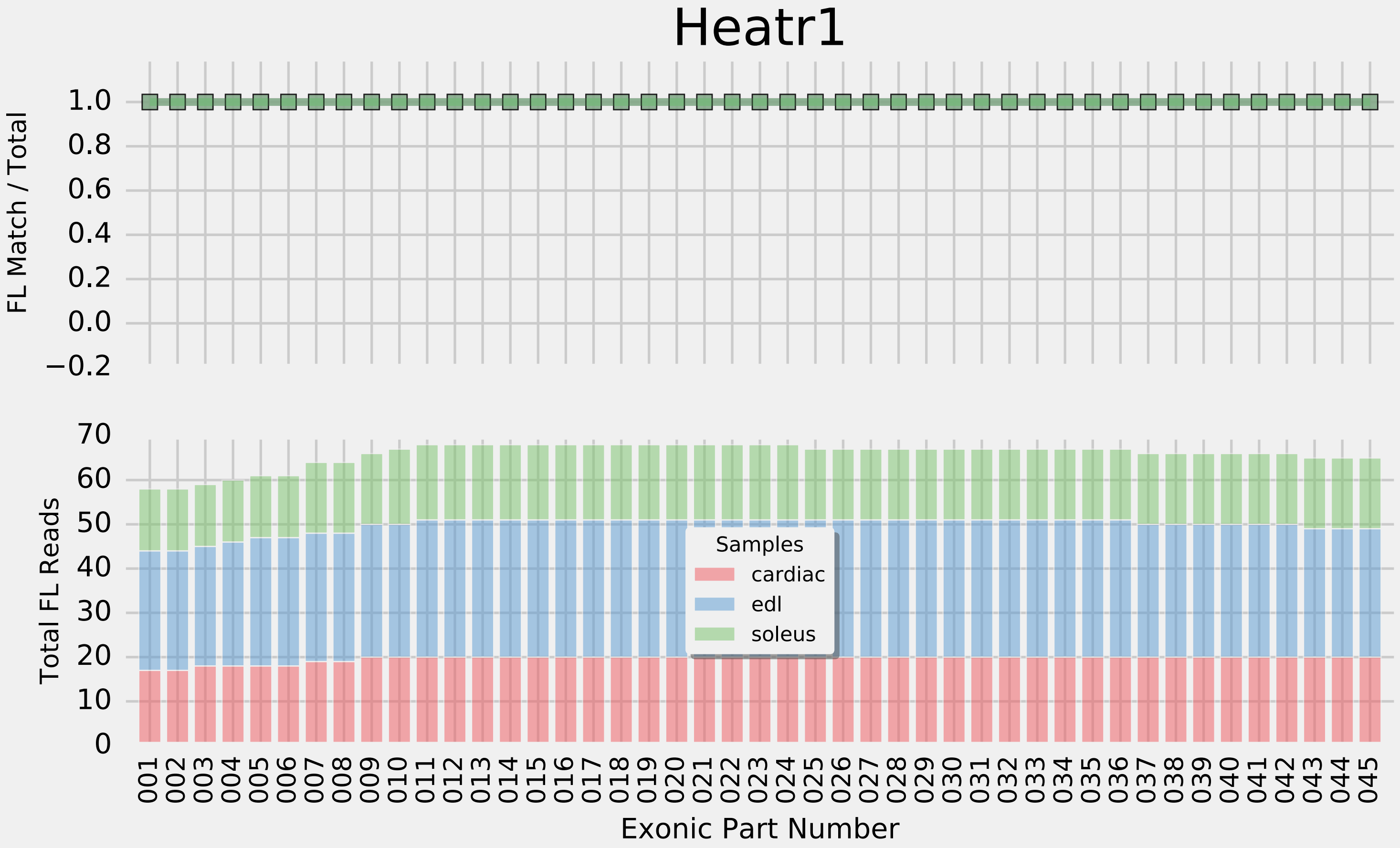
Heatr1
FL Match / Total
1.0
0.8
0.6
0.4
0.2
0.0
−0.2
Total FL Reads
70
60
50
40
30
20
10
0
Samples
cardiac
edl
soleus
001 002 003 004 005 006 007 008 009 010 011 012 013 014 015 016 017 018 019 020 021 022 023 024 025 026 027 028 029 030 031 032 033 034 035 036 037 038 039 040 041 042 043 044 045
Exonic Part Number

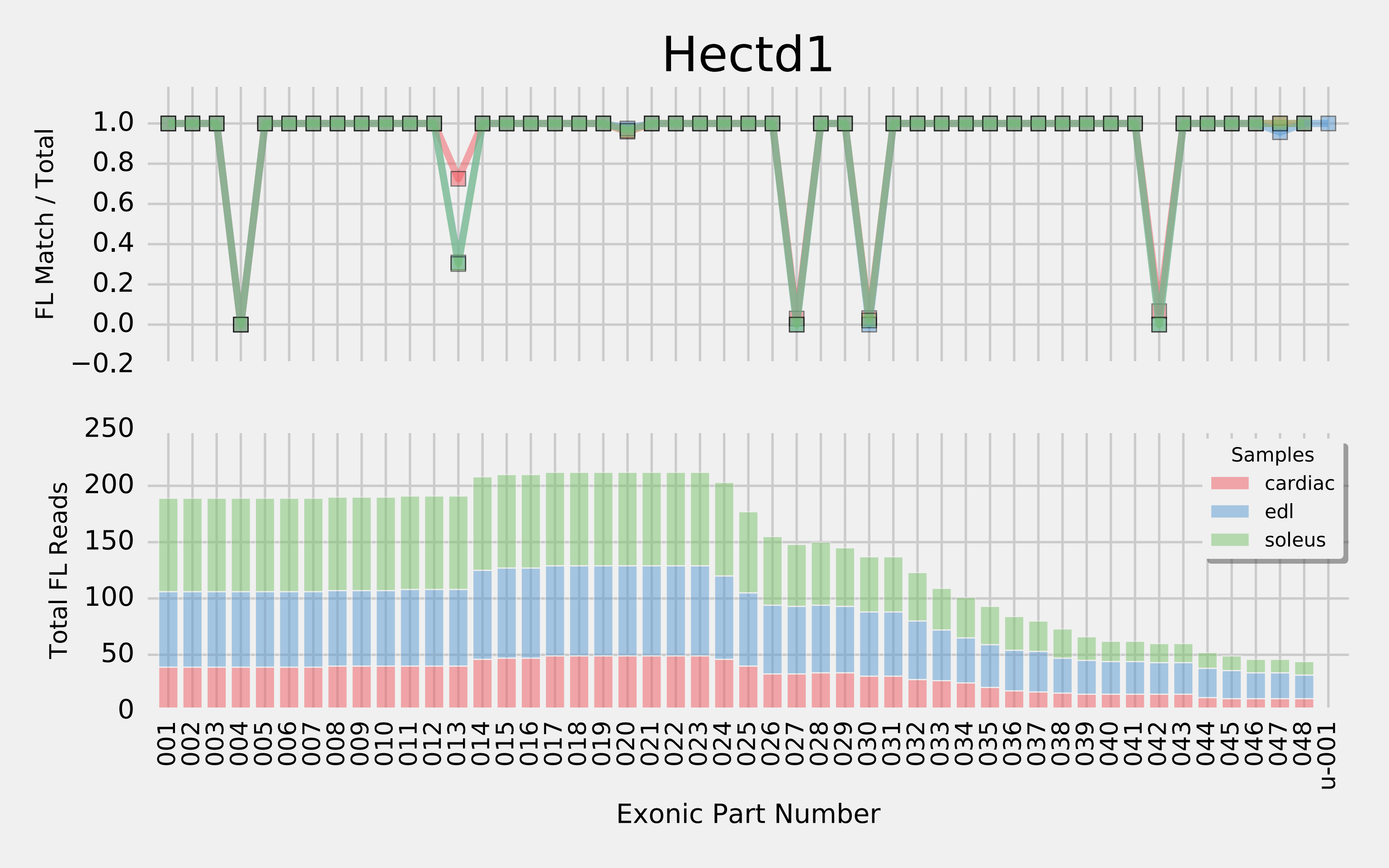
Hectd1
FL Match / Total
1.0
0.8
0.6
0.4
0.2
0.0
−0.2
Total FL Reads
250
200
150
100
50
0
Samples
cardiac
edl
soleus
001
002
003
004
005
006
007
008
009
010
011
012
013
014
015
016
017
018
019
020
021
022
023
024
025
026
027
028
029
030
031
032
033
034
035
036
037
038
039
040
041
042
043
044
045
046
047
048
u-001
Exonic Part Number

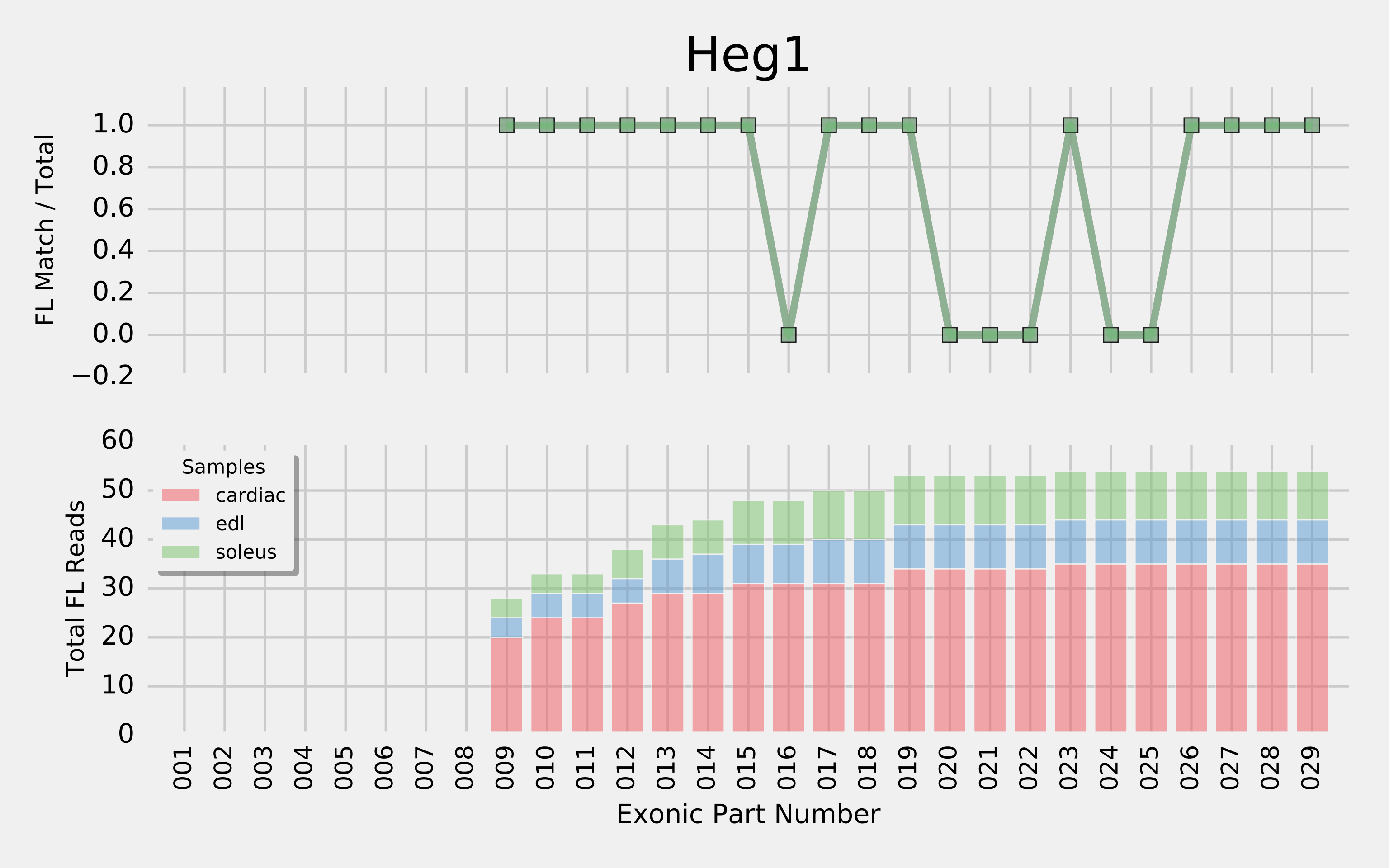
Heg1
FL Match / Total
1.0
0.8
0.6
0.4
0.2
0.0
−0.2
Total FL Reads
60
50
40
30
20
10
0
Samples
cardiac
edl
soleus
001
002
003
004
005
006
007
008
009
010
011
012
013
014
015
016
017
018
019
020
021
022
023
024
025
026
027
028
029
Exonic Part Number

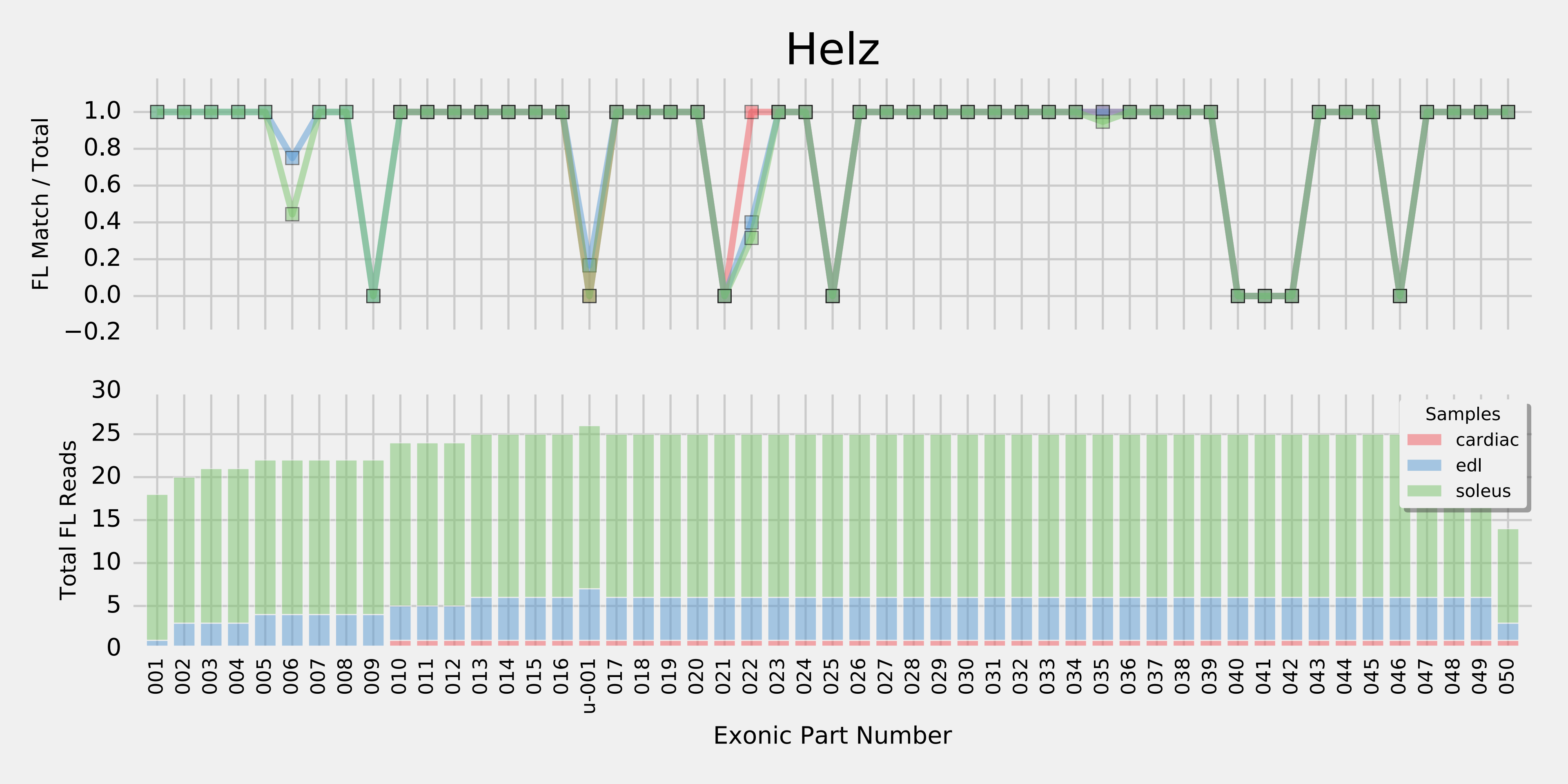
Helz
FL Match / Total
1.0
0.8
0.6
0.4
0.2
0.0
−0.2
Total FL Reads
30
25
20
15
10
5
0
Samples
cardiac
edl
soleus
001 002 003 004 005 006 007 008 009 010 011 012 013 014 015 016 u-001 017 018 019 020 021 022 023 024 025 026 027 028 029 030 031 032 033 034 035 036 037 038 039 040 041 042 043 044 045 046 047 048 049 050
Exonic Part Number

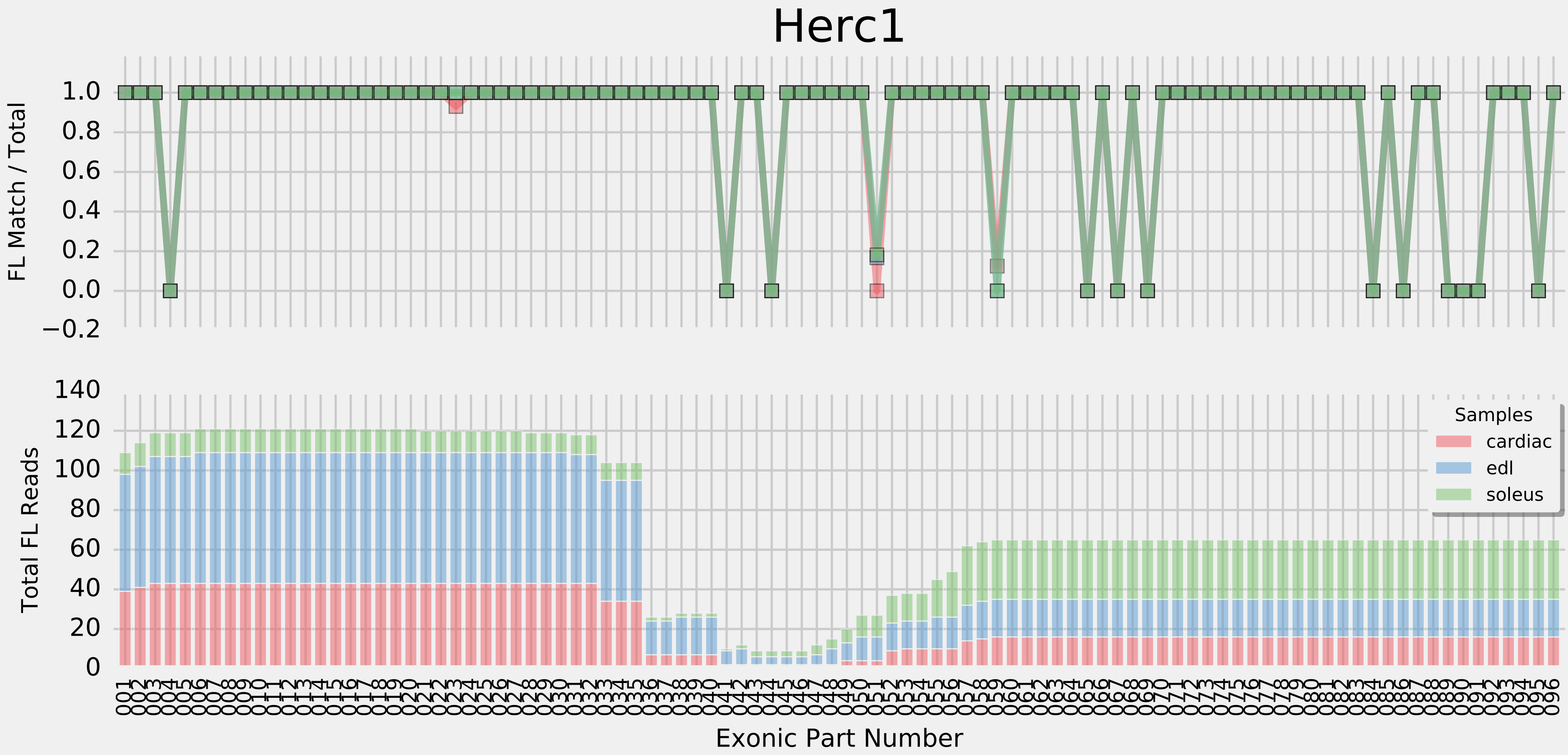

Herc1
FL Match / Total
Total FL Reads
Samples
cardiac
edl
soleus
Exonic Part Number

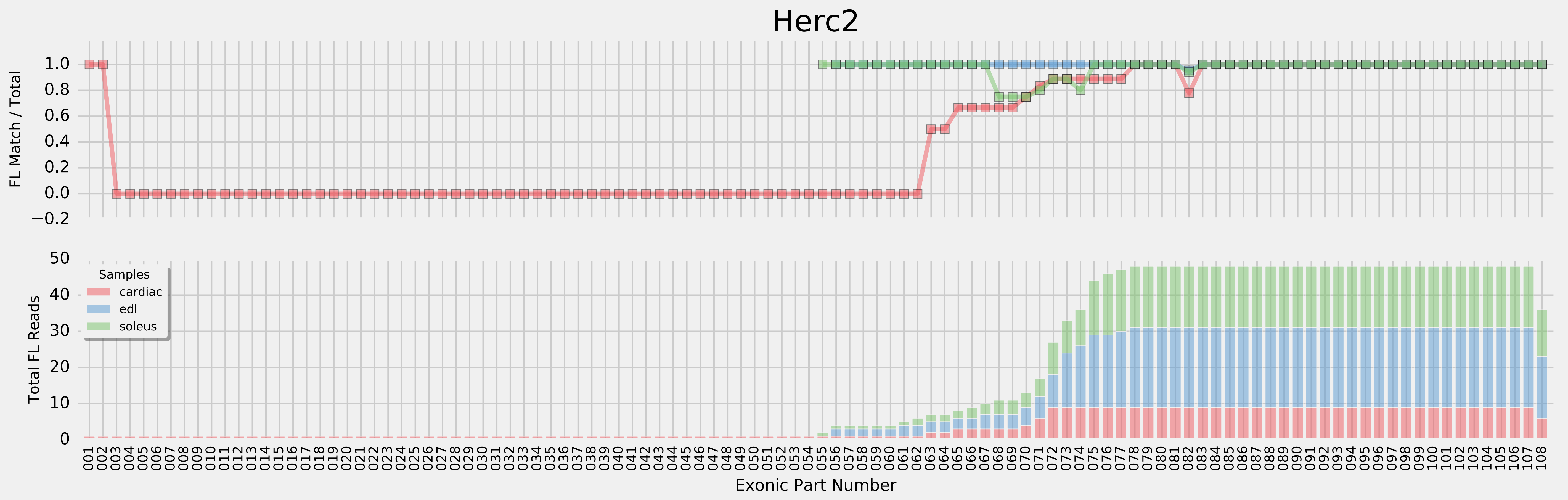
Herc2
FL Match / Total
Total FL Reads
Samples
cardiac
edl
soleus
Exonic Part Number

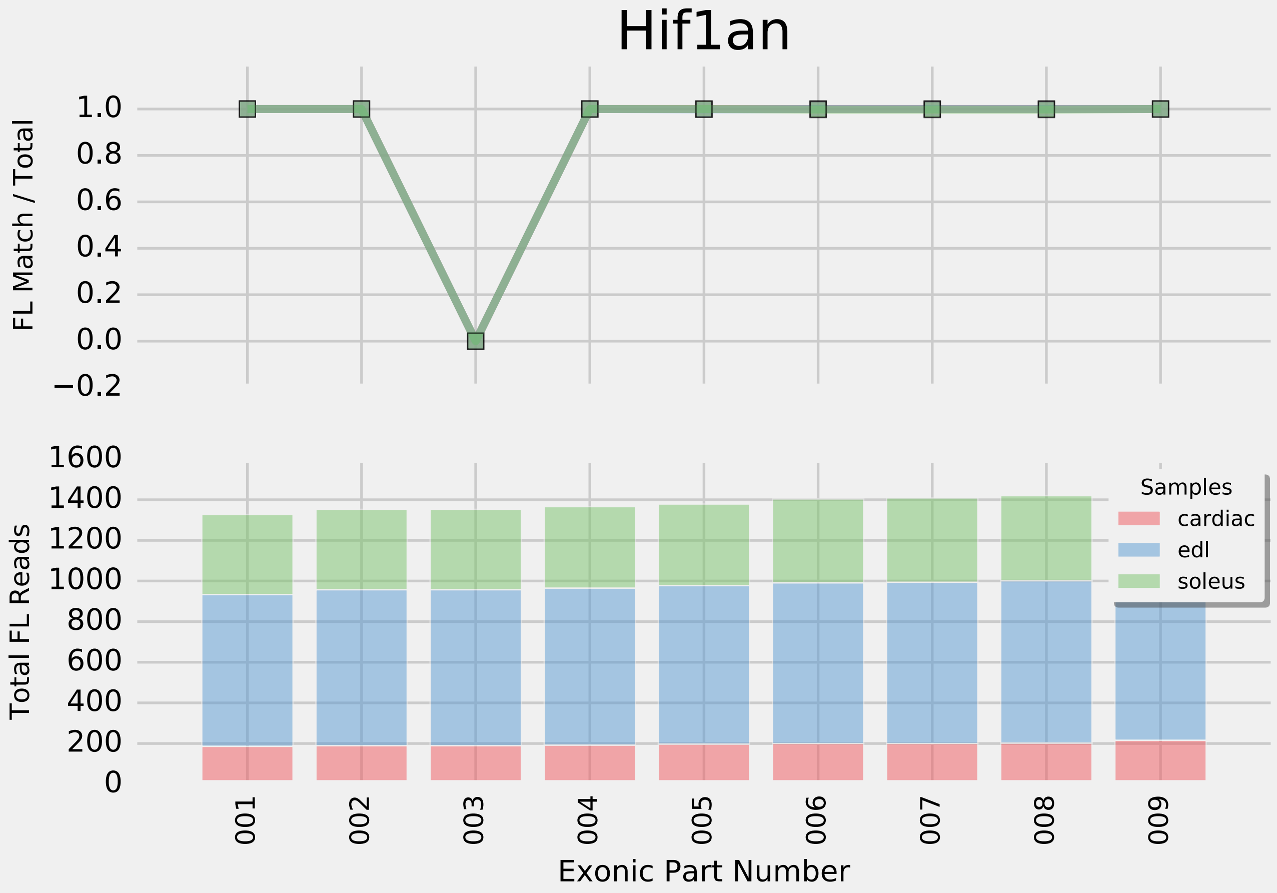

Hif1an
FL Match / Total
1.0
0.8
0.6
0.4
0.2
0.0
−0.2
Total FL Reads
1600
1400
1200
1000
800
600
400
200
0
Samples
cardiac
edl
soleus
001
002
003
004
005
006
007
008
009
Exonic Part Number

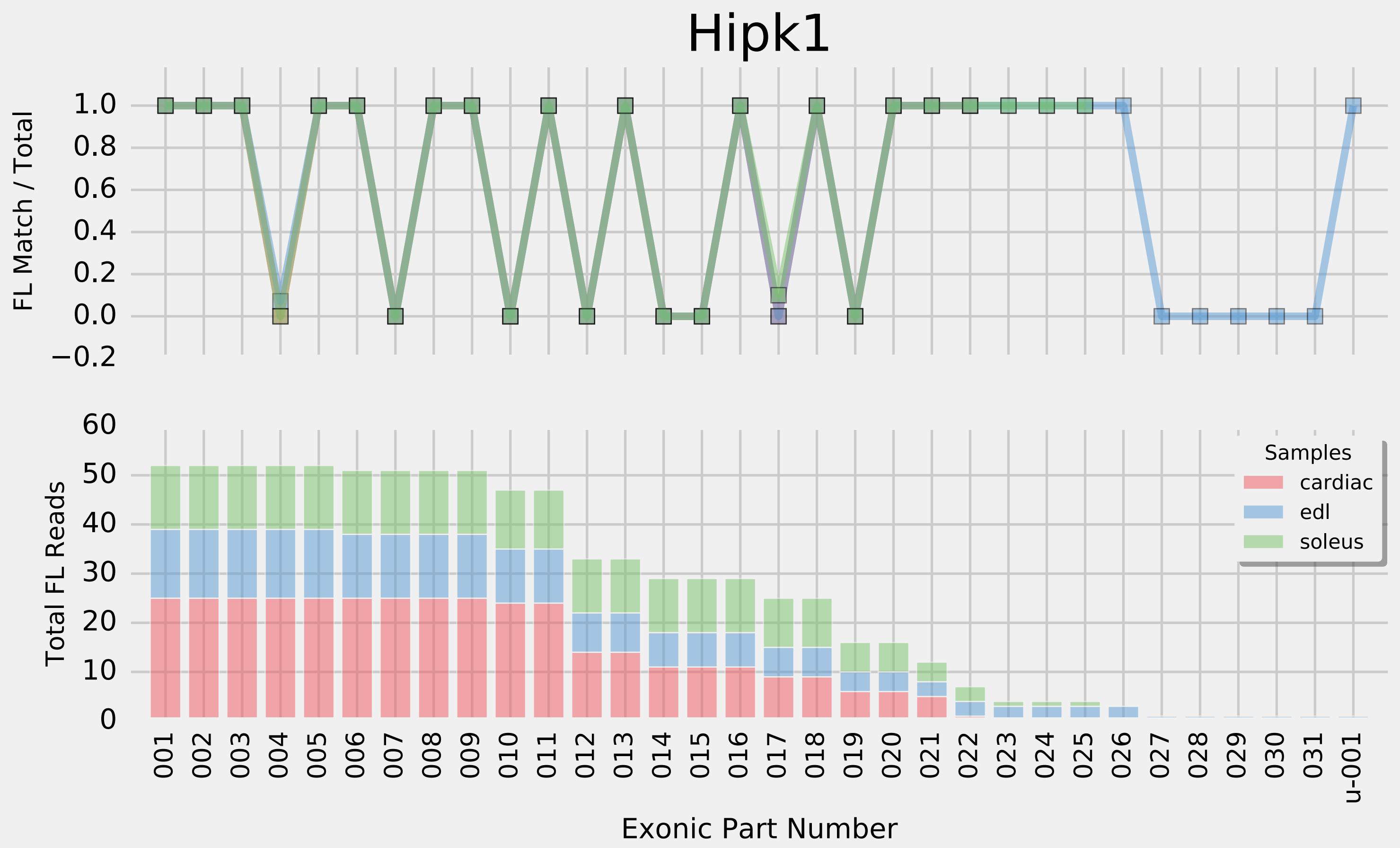
Hipk1
FL Match / Total
1.0
0.8
0.6
0.4
0.2
0.0
−0.2
Total FL Reads
60
50
40
30
20
10
0
Samples
cardiac
edl
soleus
001
002
003
004
005
006
007
008
009
010
011
012
013
014
015
016
017
018
019
020
021
022
023
024
025
026
027
028
029
030
031
u-001
Exonic Part Number

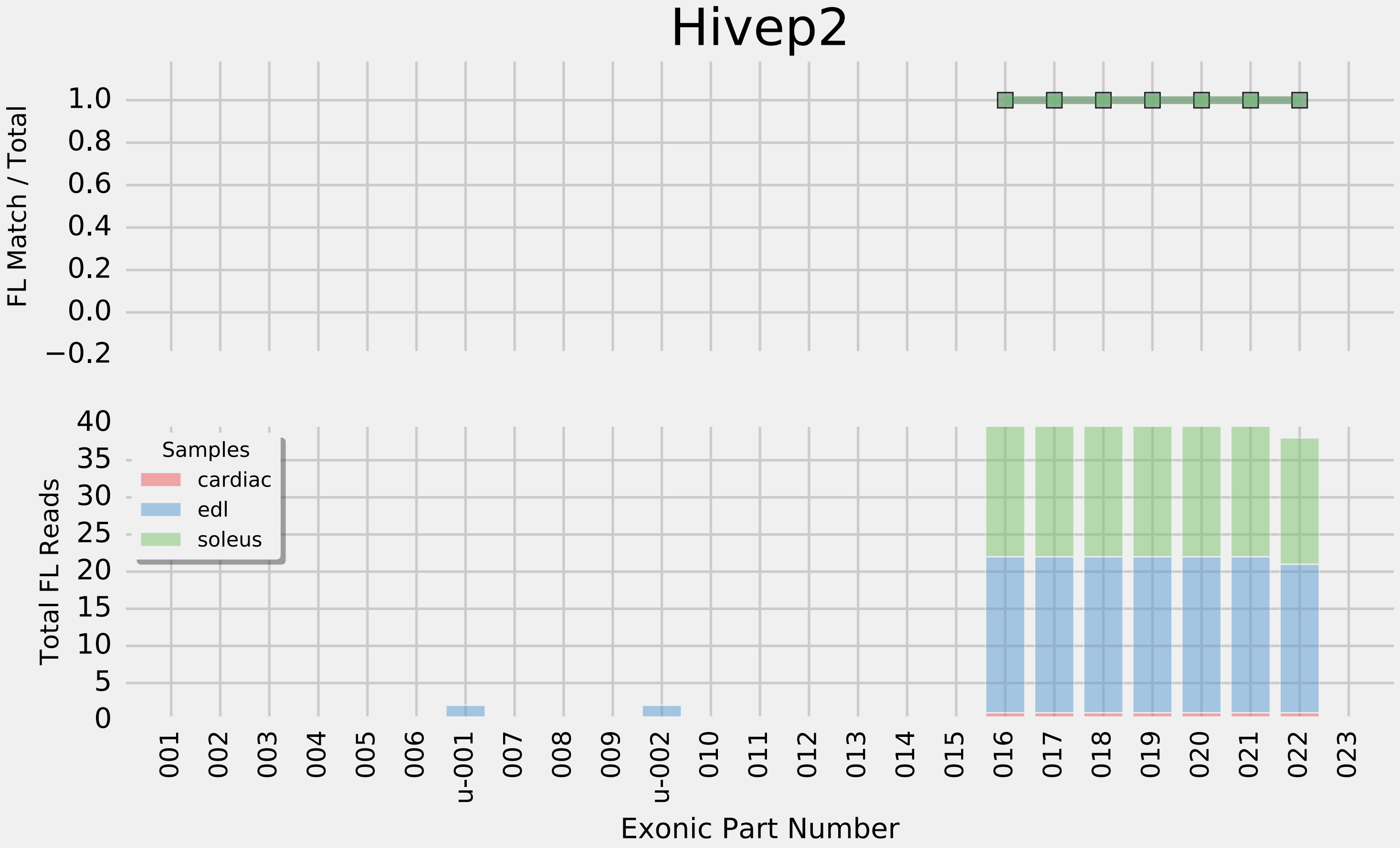
Hivep2
FL Match / Total
1.0
0.8
0.6
0.4
0.2
0.0
−0.2
Total FL Reads
40
35
30
25
20
15
10
5
0
Samples
cardiac
edl
soleus
001
002
003
004
005
006
u-001
007
008
009
u-002
010
011
012
013
014
015
016
017
018
019
020
021
022
023
Exonic Part Number

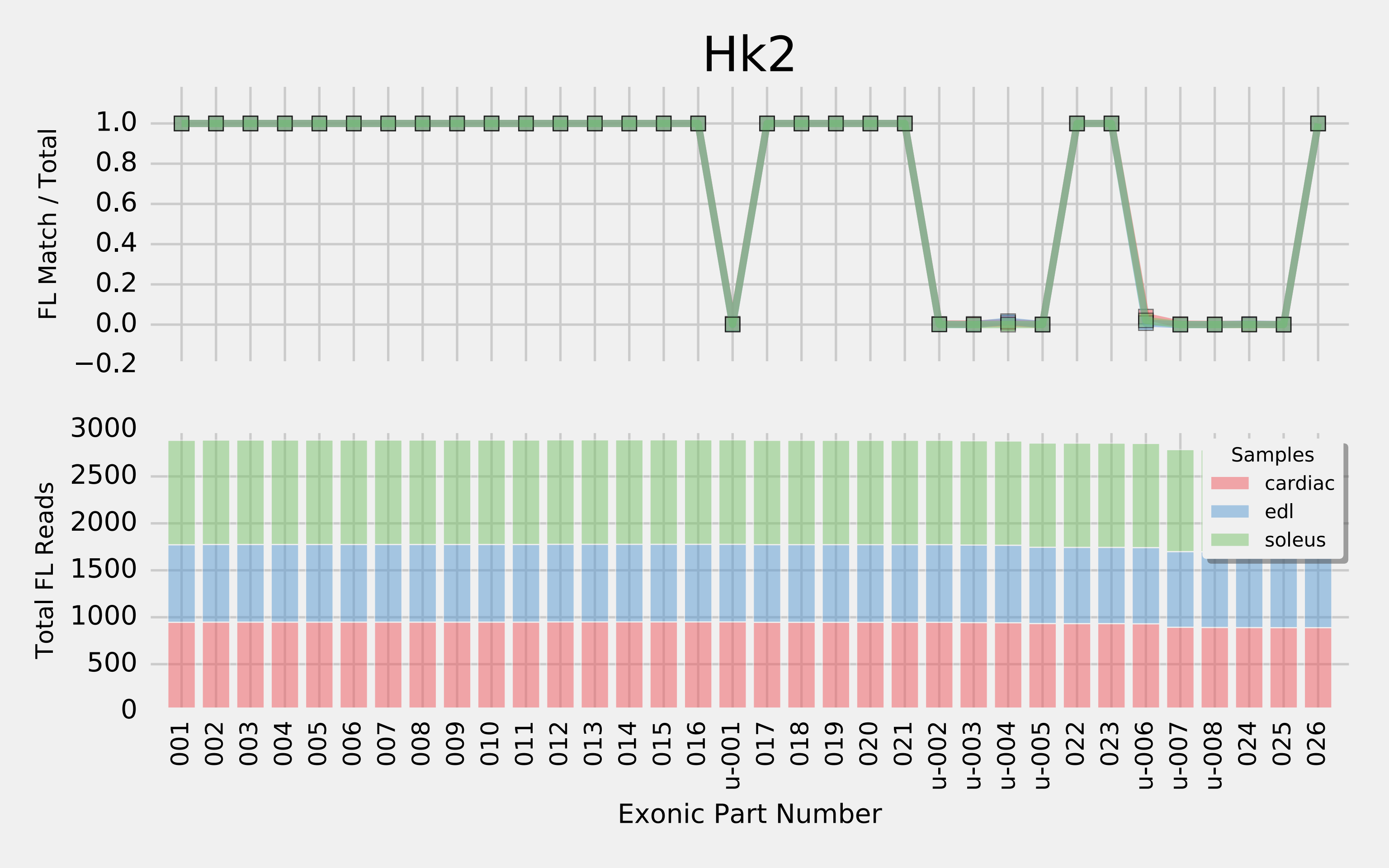
Hk2
FL Match / Total
1.0
0.8
0.6
0.4
0.2
0.0
−0.2
Total FL Reads
3000
2500
2000
1500
1000
500
0
Samples
cardiac
edl
soleus
001
002
003
004
005
006
007
008
009
010
011
012
013
014
015
016
u-001
017
018
019
020
021
u-002
u-003
u-004
u-005
022
023
u-006
u-007
u-008
024
025
026
Exonic Part Number

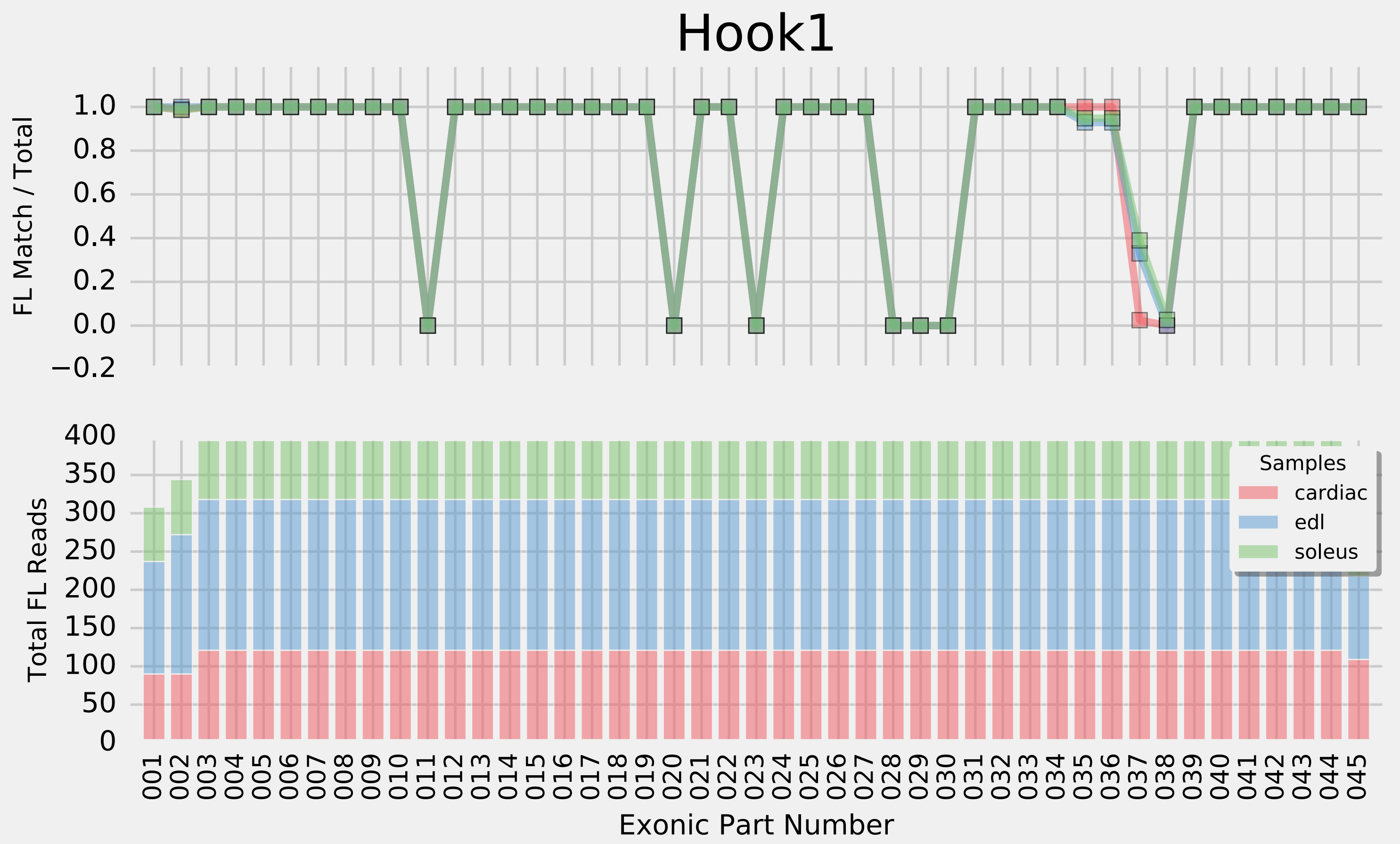

Hook1
FL Match / Total
1.0
0.8
0.6
0.4
0.2
0.0
−0.2
Total FL Reads
400
350
300
250
200
150
100
50
0
Samples
cardiac
edl
soleus
001 002 003 004 005 006 007 008 009 010 011 012 013 014 015 016 017 018 019 020 021 022 023 024 025 026 027 028 029 030 031 032 033 034 035 036 037 038 039 040 041 042 043 044 045
Exonic Part Number

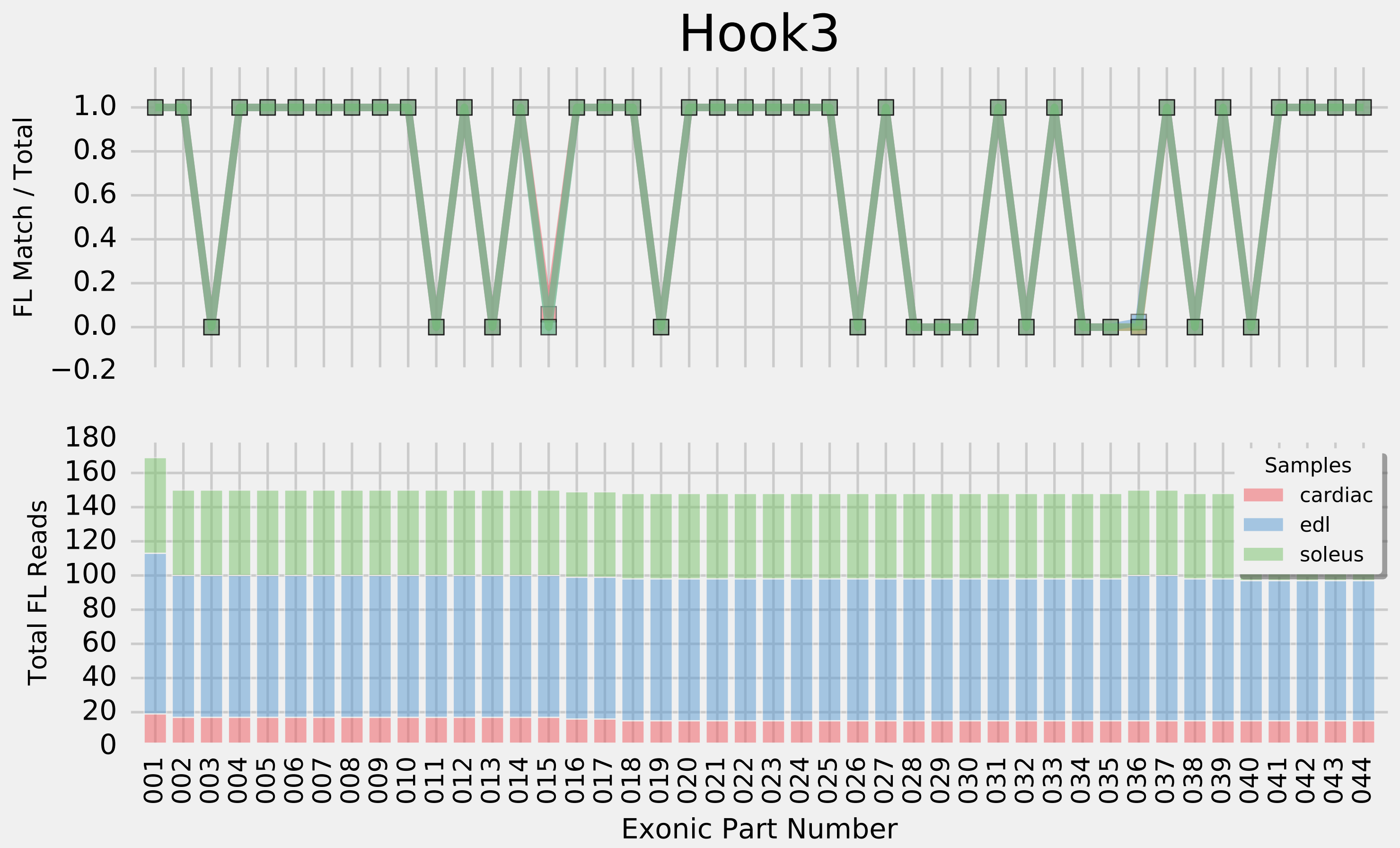
Hook3
FL Match / Total
Total FL Reads
Samples
cardiac
edl
soleus
Exonic Part Number

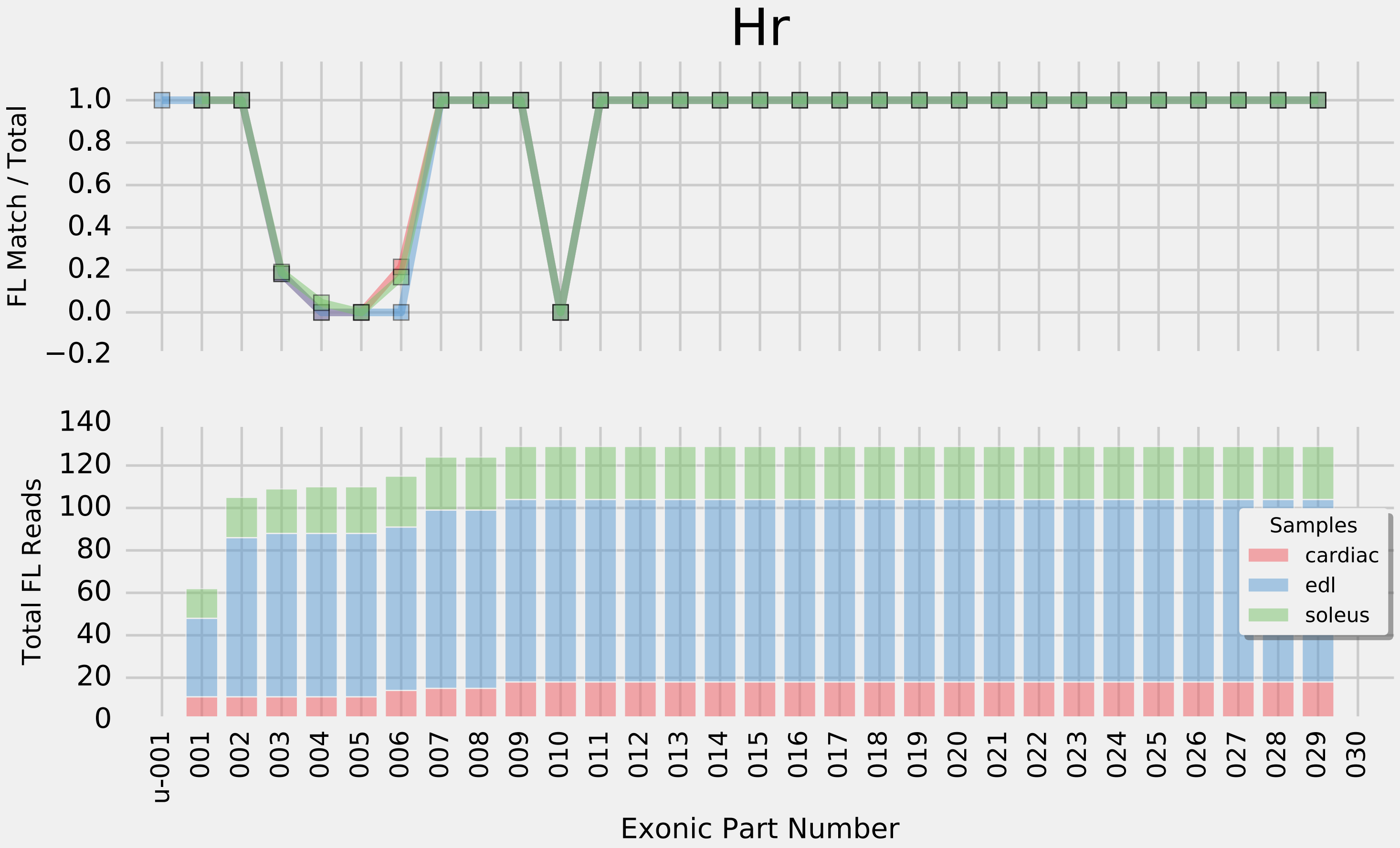

Hr
FL Match / Total
1.0
0.8
0.6
0.4
0.2
0.0
−0.2
140
120
100
80
60
40
20
0
Total FL Reads
Samples
cardiac
edl
soleus
u-001
001
002
003
004
005
006
007
008
009
010
011
012
013
014
015
016
017
018
019
020
021
022
023
024
025
026
027
028
029
030
Exonic Part Number

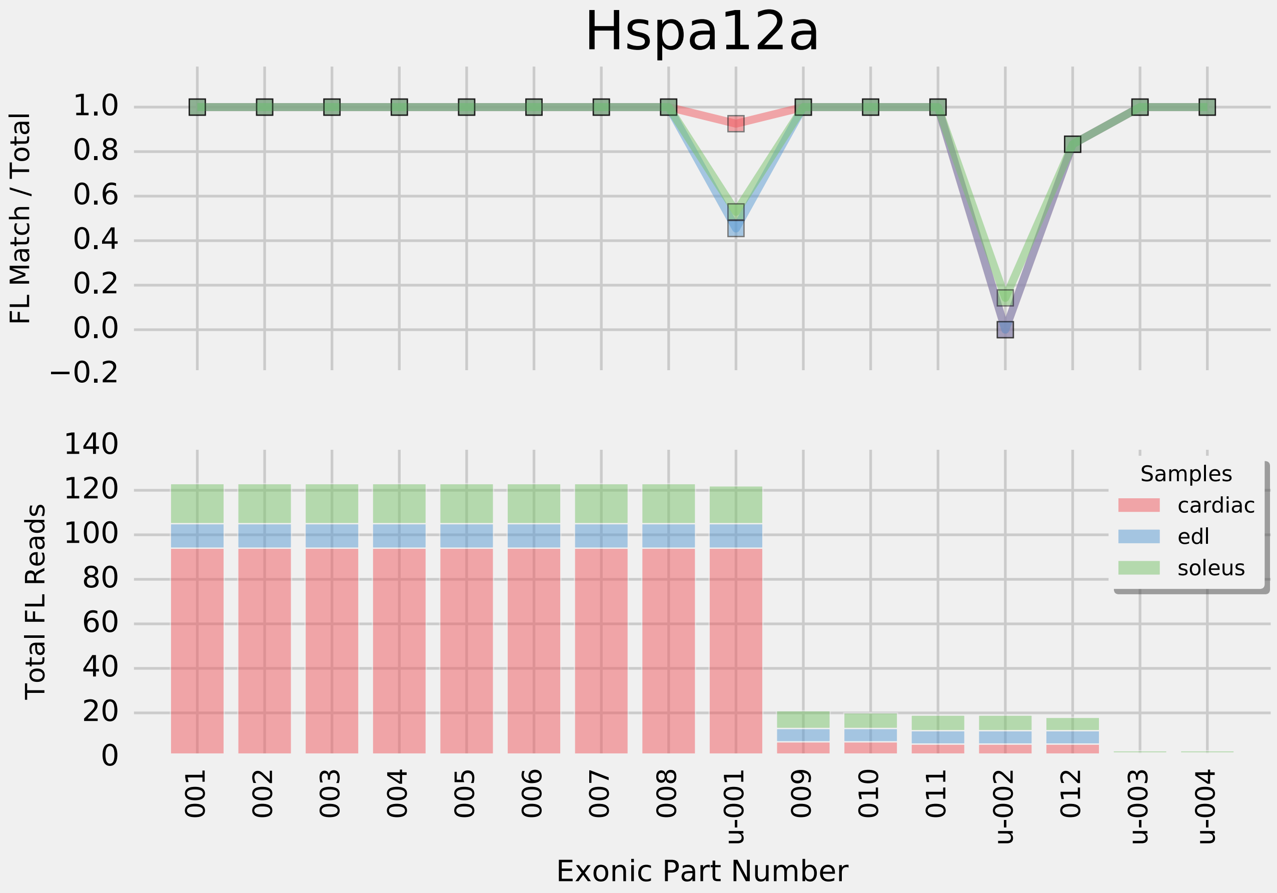

Hspa12a
FL Match / Total
1.0
0.8
0.6
0.4
0.2
0.0
−0.2
Total FL Reads
140
120
100
80
60
40
20
0
Samples
cardiac
edl
soleus
001
002
003
004
005
006
007
008
u-001
009
010
011
u-002
012
u-003
u-004
Exonic Part Number

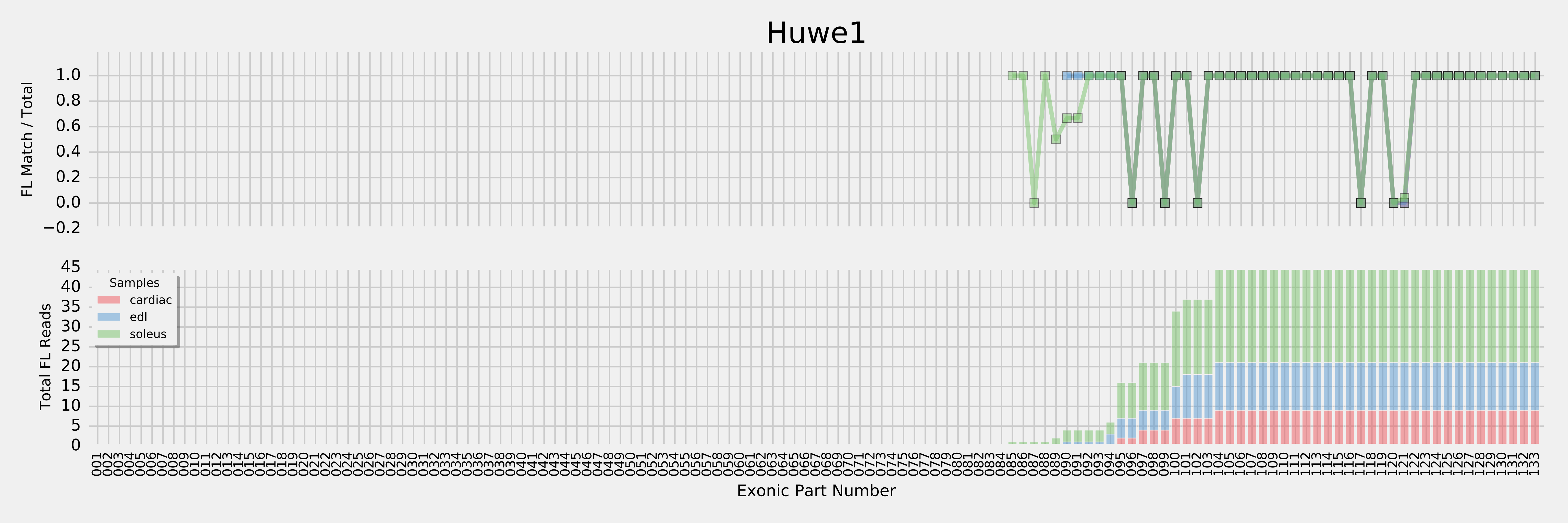

Huwe1
FL Match / Total
Total FL Reads
Samples
cardiac
edl
soleus
Exonic Part Number

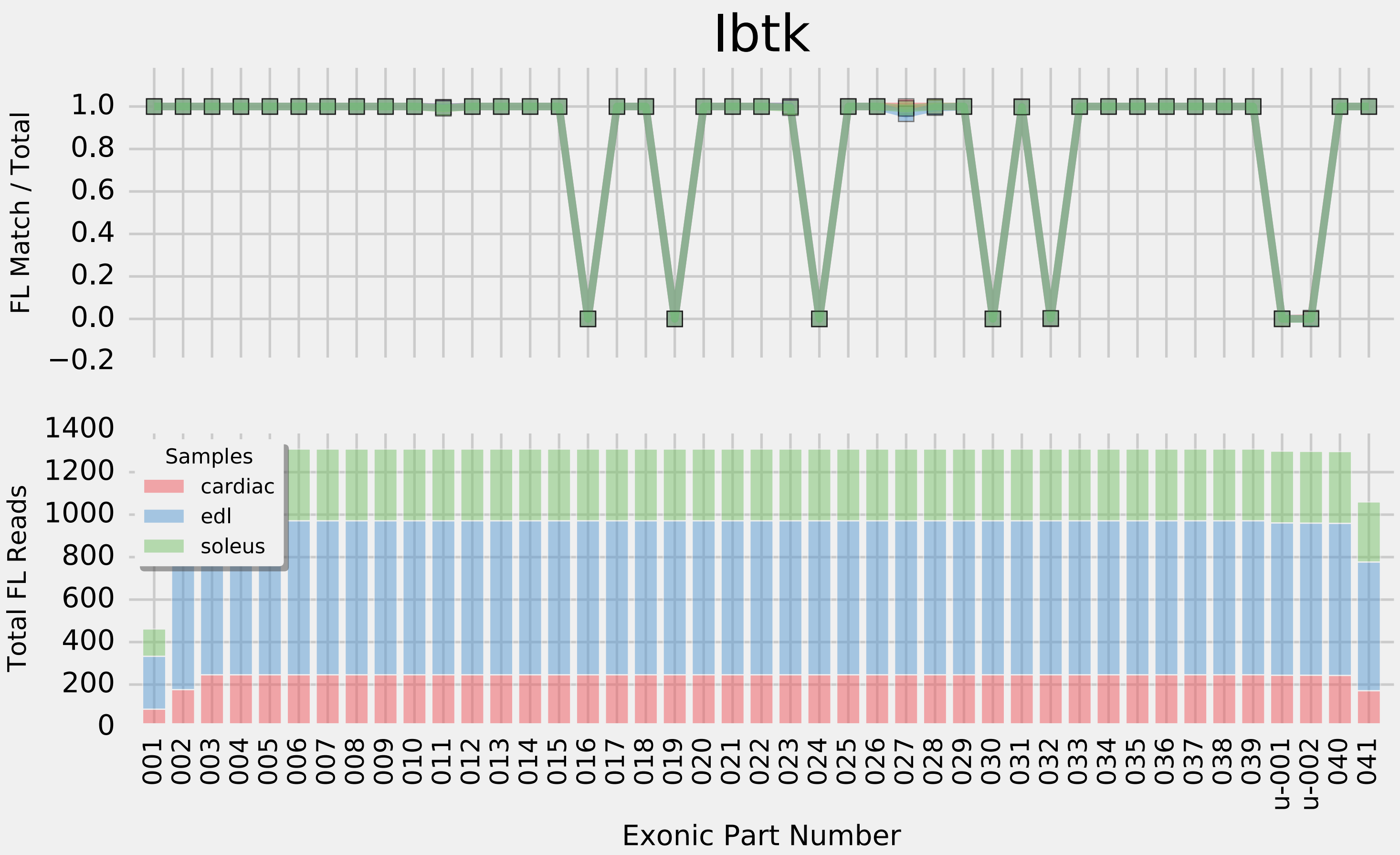
Ibtk
FL Match / Total
1.0
0.8
0.6
0.4
0.2
0.0
−0.2
Total FL Reads
1400
1200
1000
800
600
400
200
0
Samples
cardiac
edl
soleus
001 002 003 004 005 006 007 008 009 010 011 012 013 014 015 016 017 018 019 020 021 022 023 024 025 026 027 028 029 030 031 032 033 034 035 036 037 038 039 u-001 u-002 040 041
Exonic Part Number

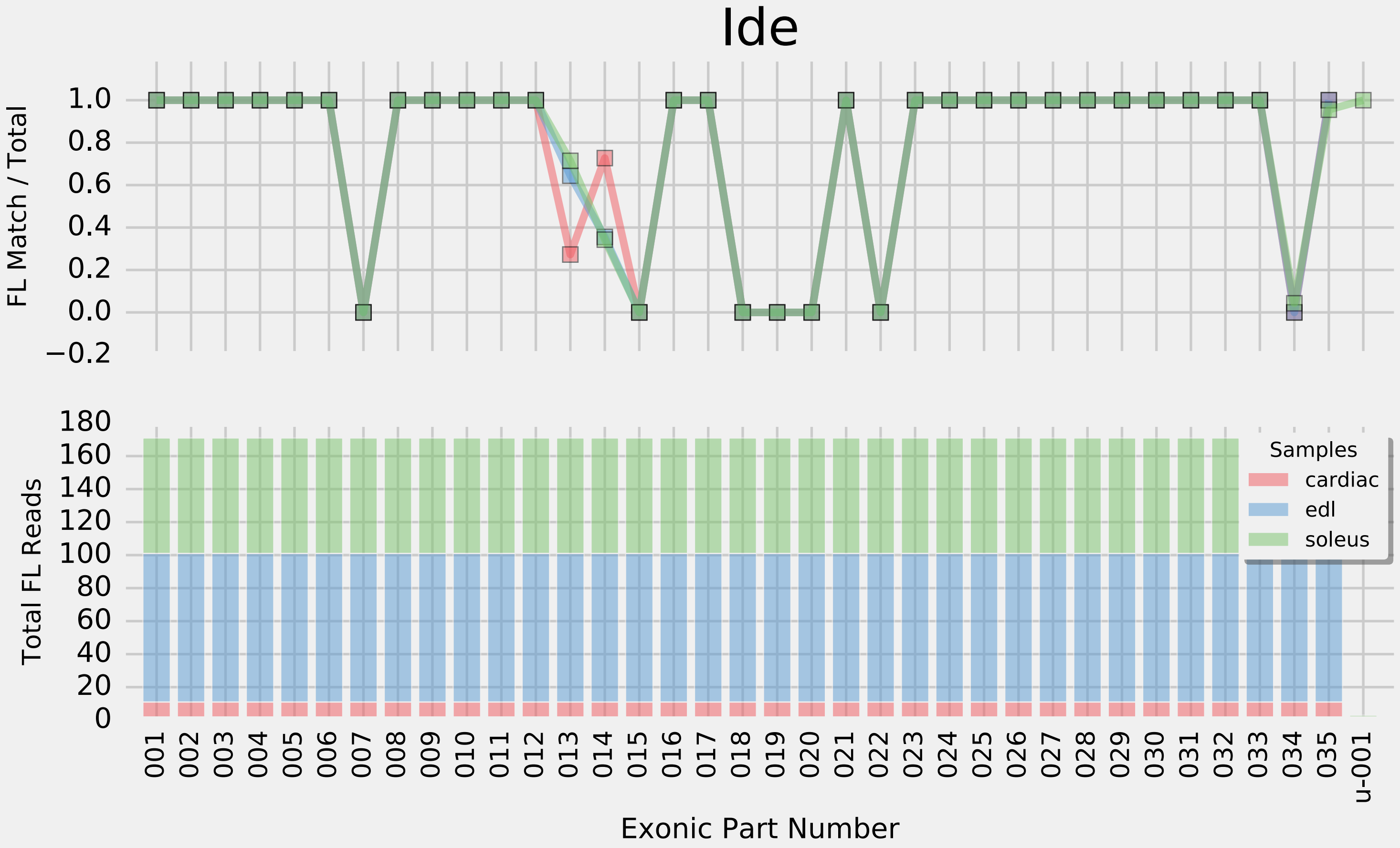
Ide
FL Match / Total
1.0
0.8
0.6
0.4
0.2
0.0
−0.2
Total FL Reads
180
160
140
120
100
80
60
40
20
0
Samples
cardiac
edl
soleus
001
002
003
004
005
006
007
008
009
010
011
012
013
014
015
016
017
018
019
020
021
022
023
024
025
026
027
028
029
030
031
032
033
034
035
u-001
Exonic Part Number

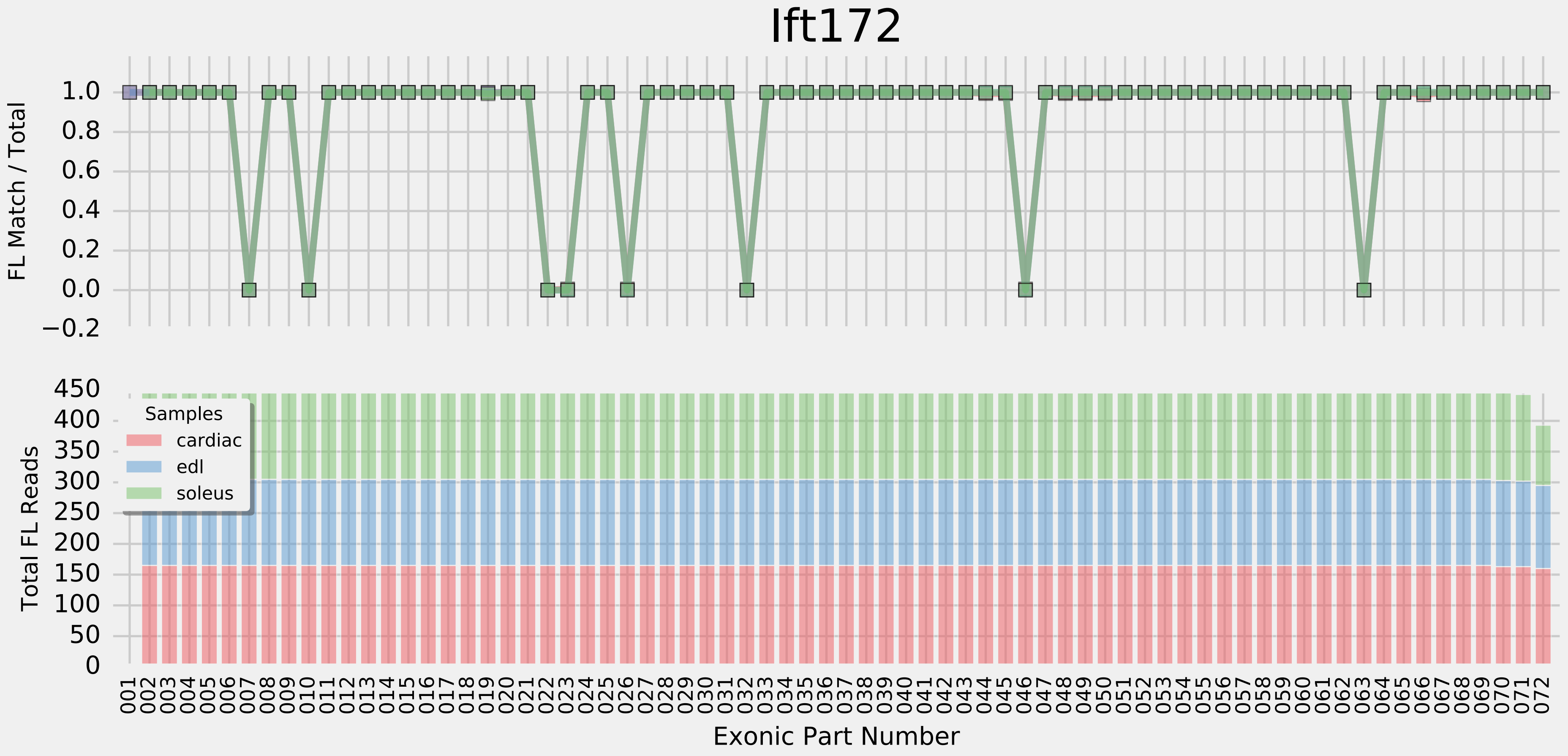
Ift172
FL Match / Total
Total FL Reads
Samples
cardiac
edl
soleus
Exonic Part Number

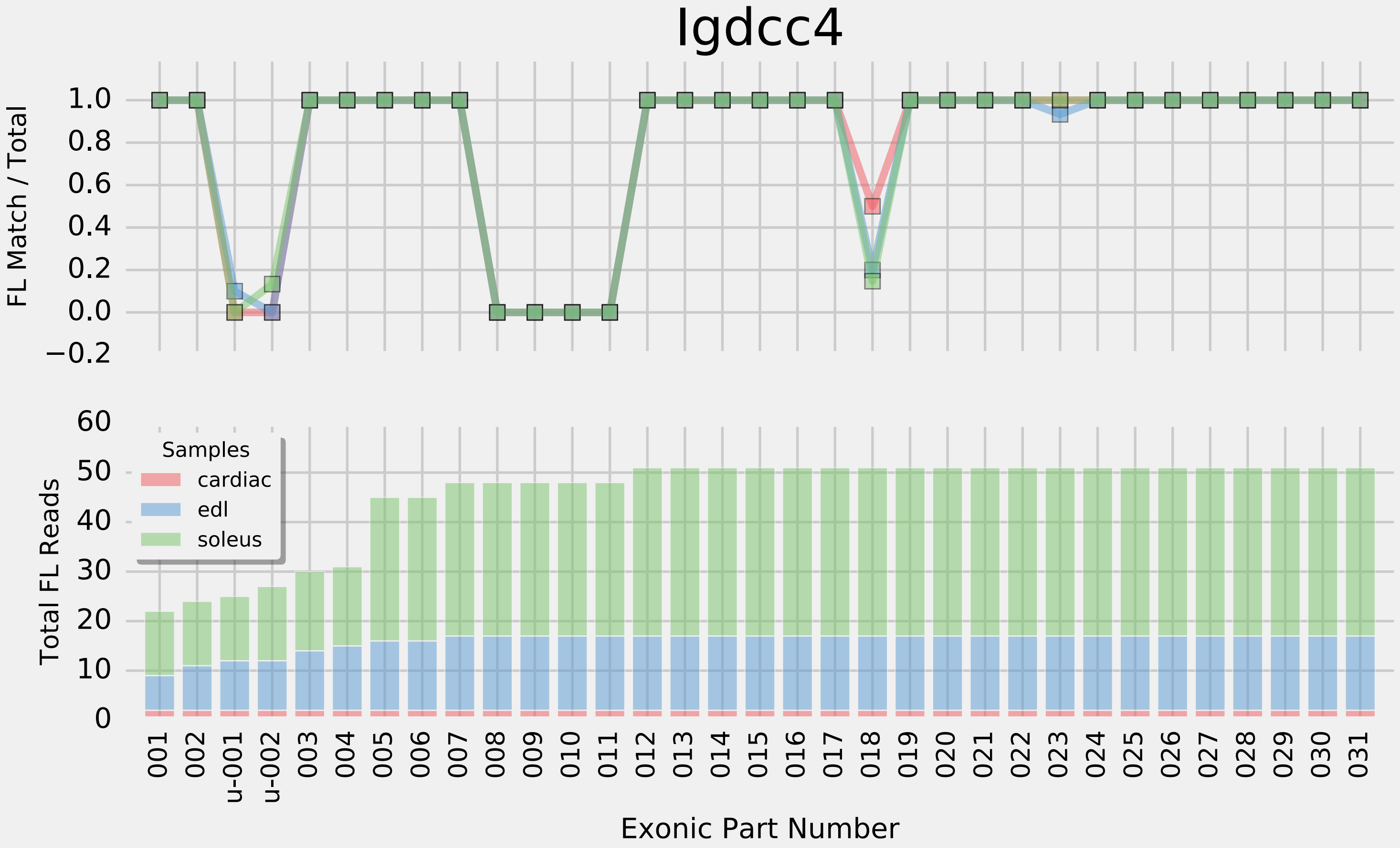

Igdcc4
FL Match / Total
1.0
0.8
0.6
0.4
0.2
0.0
−0.2
Total FL Reads
60
50
40
30
20
10
0
Samples
cardiac
edl
soleus
001
002
u-001
u-002
003
004
005
006
007
008
009
010
011
012
013
014
015
016
017
018
019
020
021
022
023
024
025
026
027
028
029
030
031
Exonic Part Number

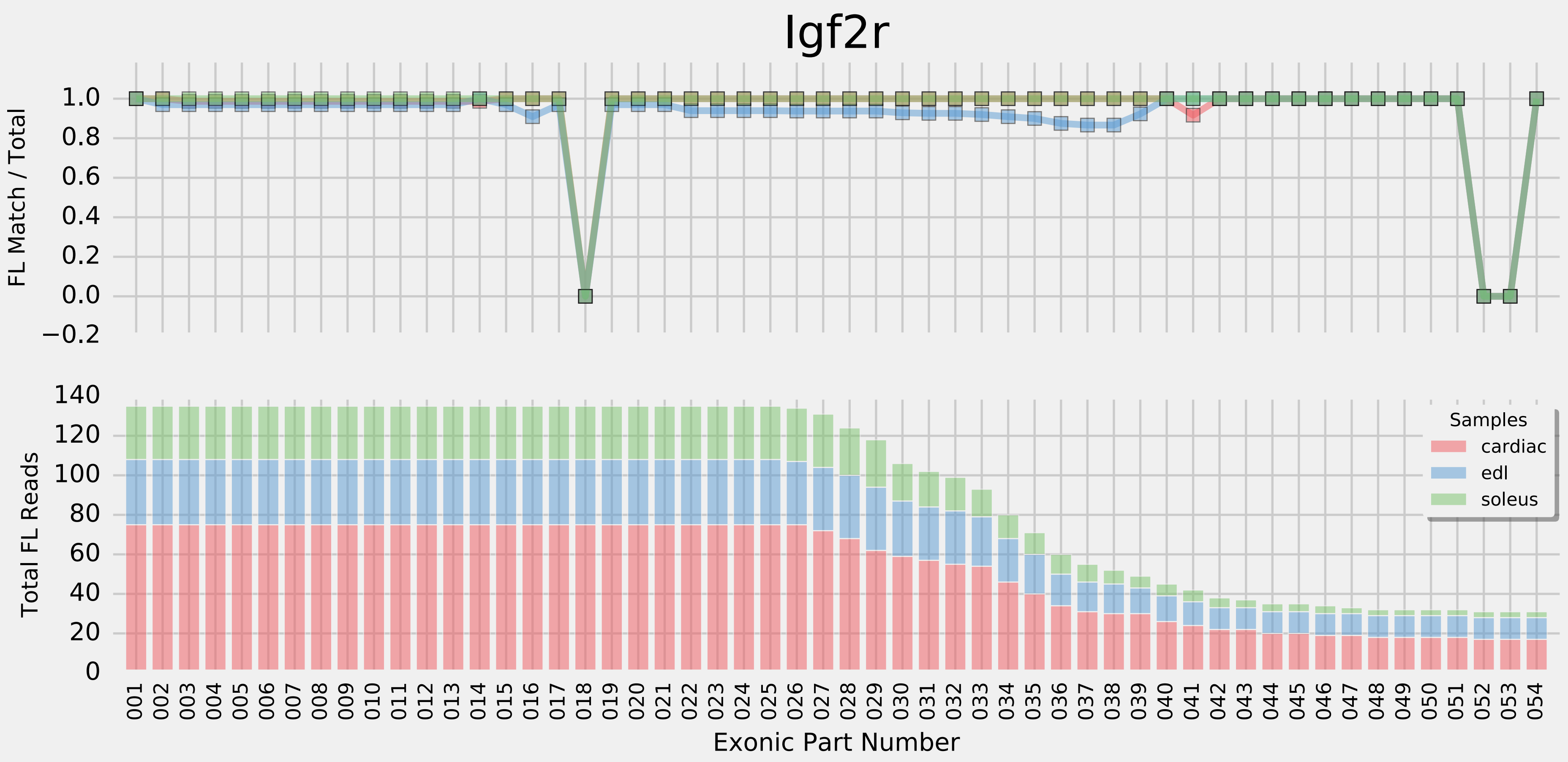

Igf2r
FL Match / Total
1.0
0.8
0.6
0.4
0.2
0.0
−0.2
Total FL Reads
140
120
100
80
60
40
20
0
Samples
cardiac
edl
soleus
001 002 003 004 005 006 007 008 009 010 011 012 013 014 015 016 017 018 019 020 021 022 023 024 025 026 027 028 029 030 031 032 033 034 035 036 037 038 039 040 041 042 043 044 045 046 047 048 049 050 051 052 053 054
Exonic Part Number

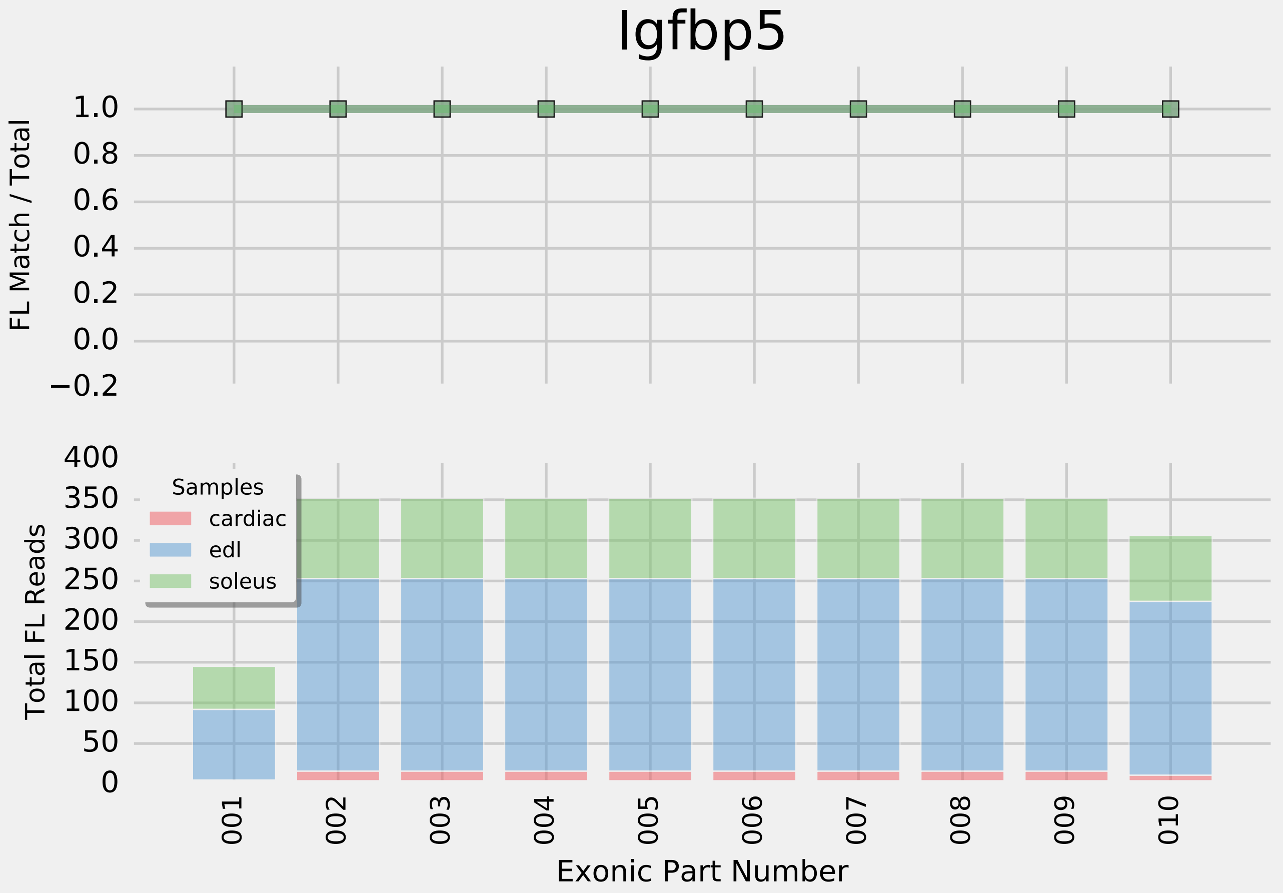
Igfbp5
FL Match / Total
1.0
0.8
0.6
0.4
0.2
0.0
−0.2
Total FL Reads
400
350
300
250
200
150
100
50
0
Samples
cardiac
edl
soleus
001
002
003
004
005
006
007
008
009
010
Exonic Part Number

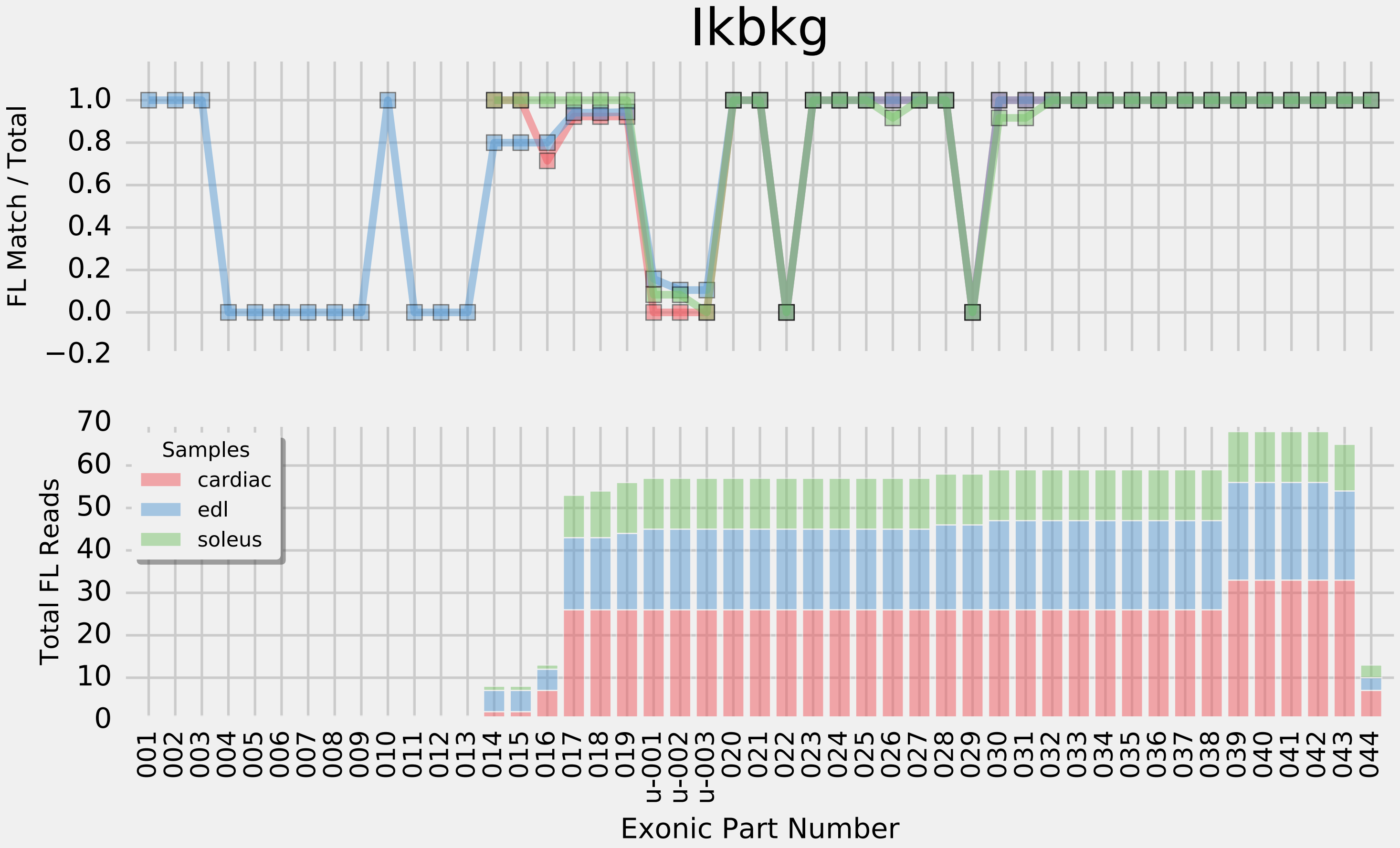
Ikbkg
FL Match / Total
1.0
0.8
0.6
0.4
0.2
0.0
−0.2
Total FL Reads
70
60
50
40
30
20
10
0
Samples
cardiac
edl
soleus
001
002
003
004
005
006
007
008
009
010
011
012
013
014
015
016
017
018
019
u-001
u-002
u-003
020
021
022
023
024
025
026
027
028
029
030
031
032
033
034
035
036
037
038
039
040
041
042
043
044
Exonic Part Number

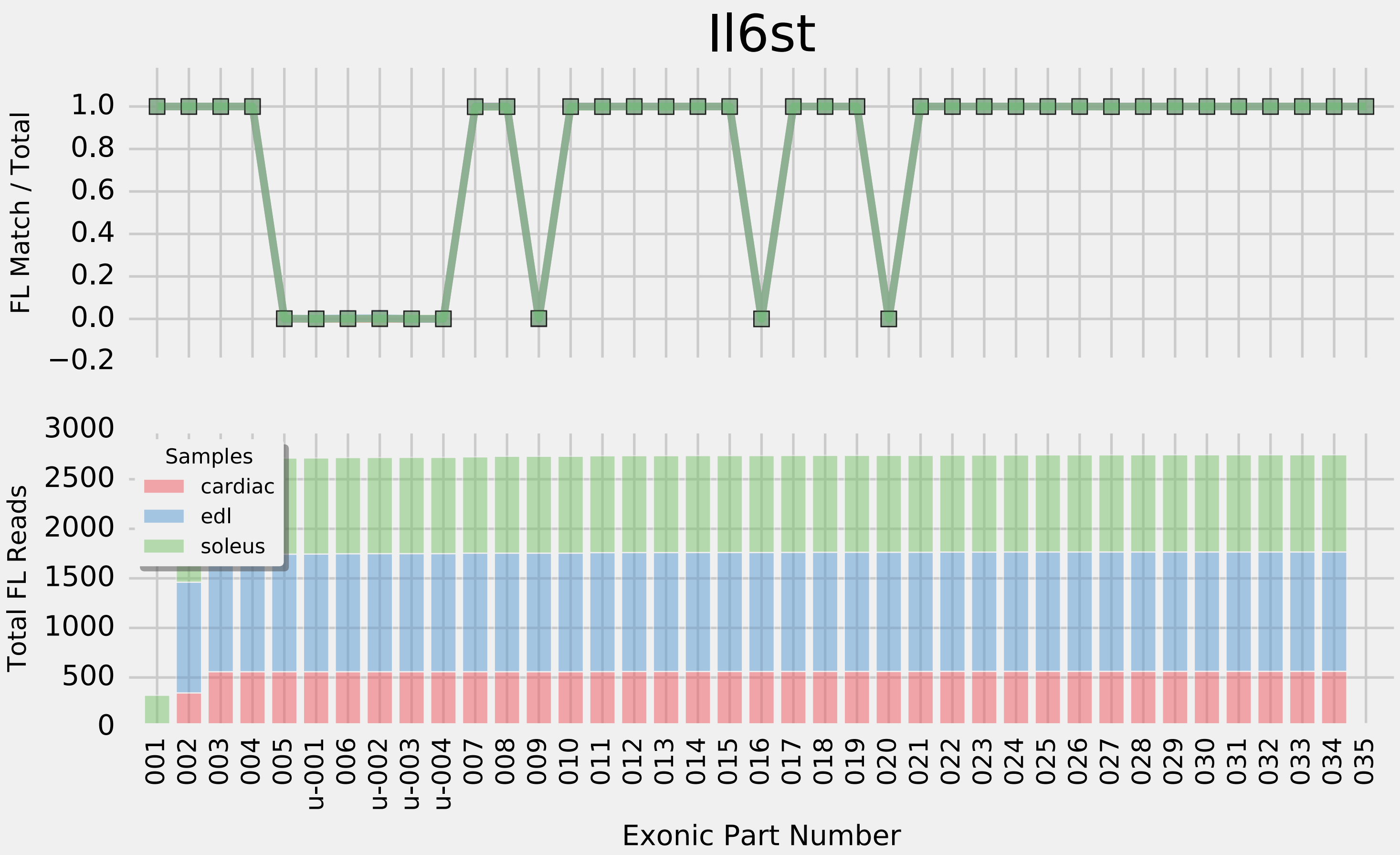

Il6st
FL Match / Total
1.0
0.8
0.6
0.4
0.2
0.0
−0.2
3000
2500
2000
1500
1000
500
0
Total FL Reads
Samples
cardiac
edl
soleus
001 002 003 004 005 u-001 006 u-002 u-003 u-004 007 008 009 010 011 012 013 014 015 016 017 018 019 020 021 022 023 024 025 026 027 028 029 030 031 032 033 034 035
Exonic Part Number

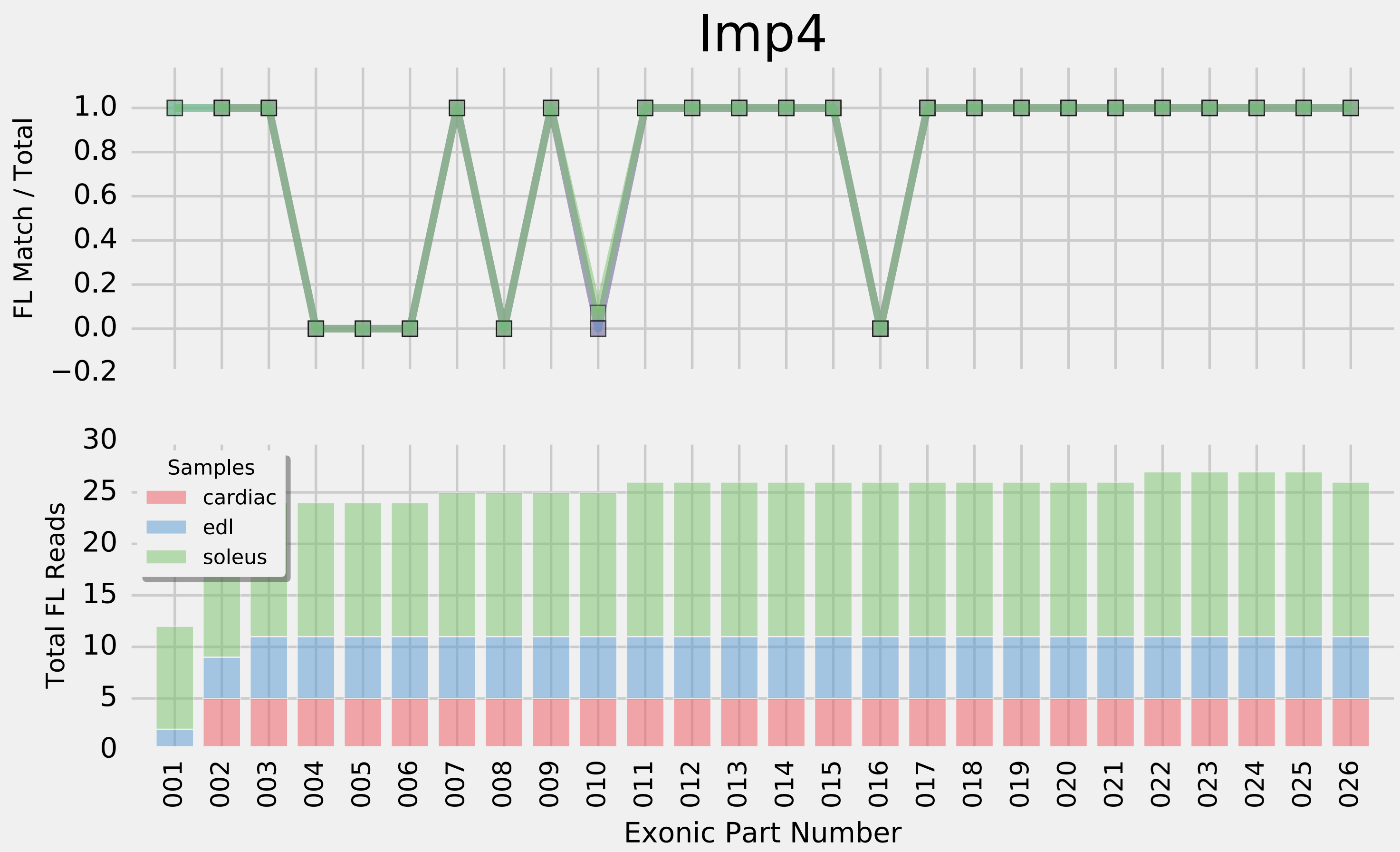
Imp4
FL Match / Total
1.0
0.8
0.6
0.4
0.2
0.0
−0.2
Total FL Reads
30
25
20
15
10
5
0
Samples
cardiac
edl
soleus
001
002
003
004
005
006
007
008
009
010
011
012
013
014
015
016
017
018
019
020
021
022
023
024
025
026
Exonic Part Number

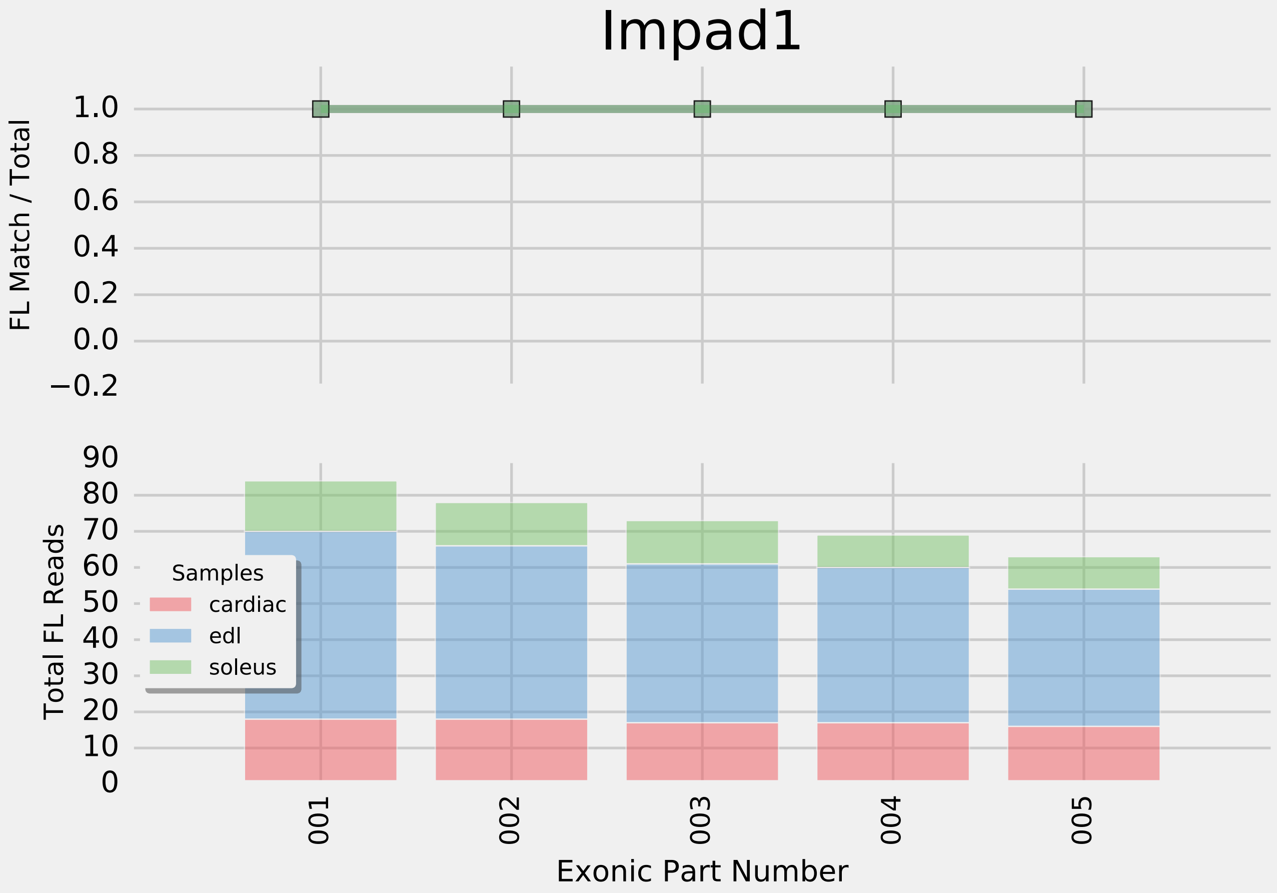
Impad1
FL Match / Total
1.0
0.8
0.6
0.4
0.2
0.0
−0.2
Total FL Reads
90
80
70
60
50
40
30
20
10
0
Samples
cardiac
edl
soleus
001
002
003
004
005
Exonic Part Number

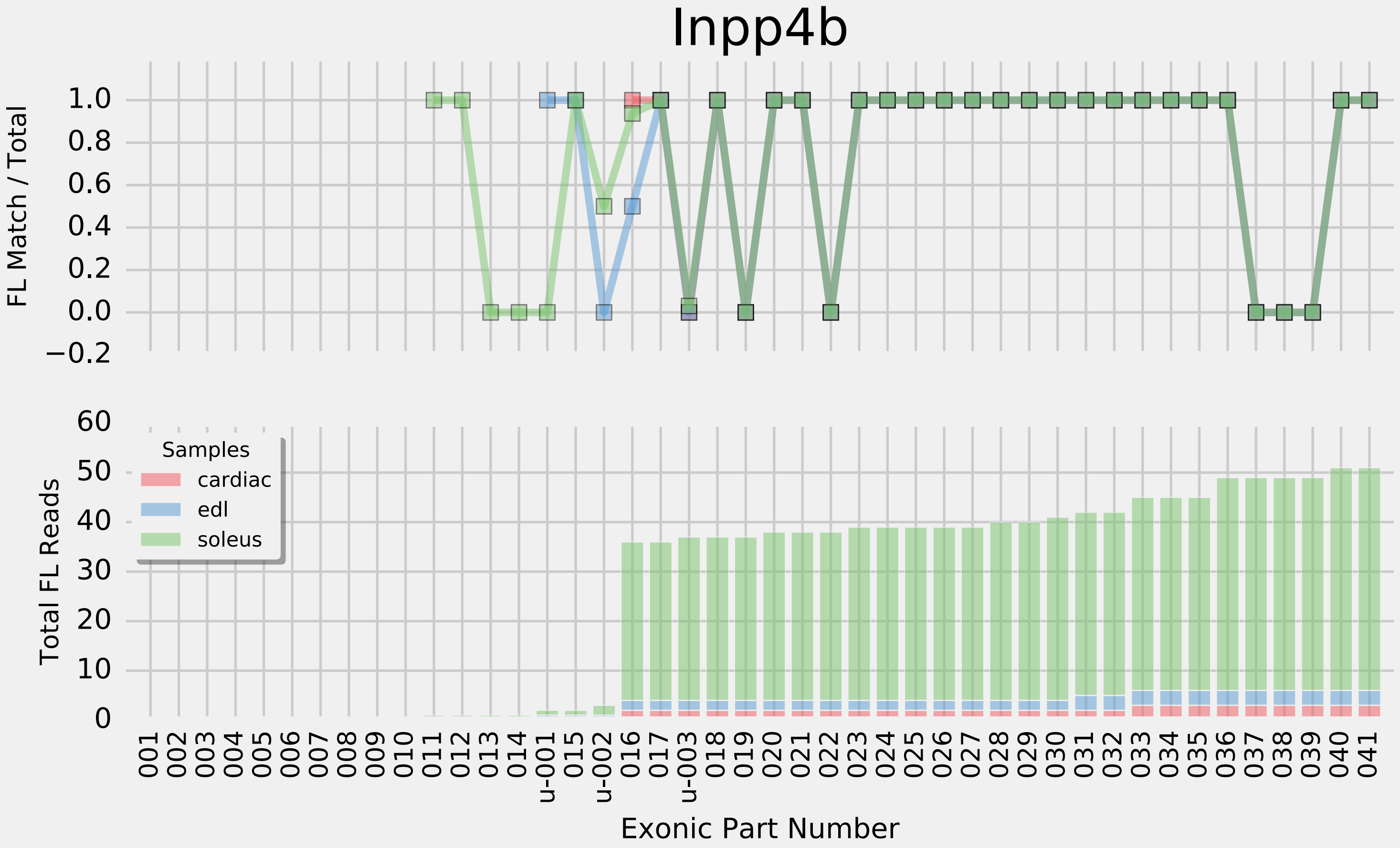
Inpp4b
FL Match / Total
1.0
0.8
0.6
0.4
0.2
0.0
−0.2
Total FL Reads
60
50
40
30
20
10
0
Samples
cardiac
edl
soleus
001
002
003
004
005
006
007
008
009
010
011
012
013
014
u-001
015
u-002
016
017
u-003
018
019
020
021
022
023
024
025
026
027
028
029
030
031
032
033
034
035
036
037
038
039
040
041
Exonic Part Number

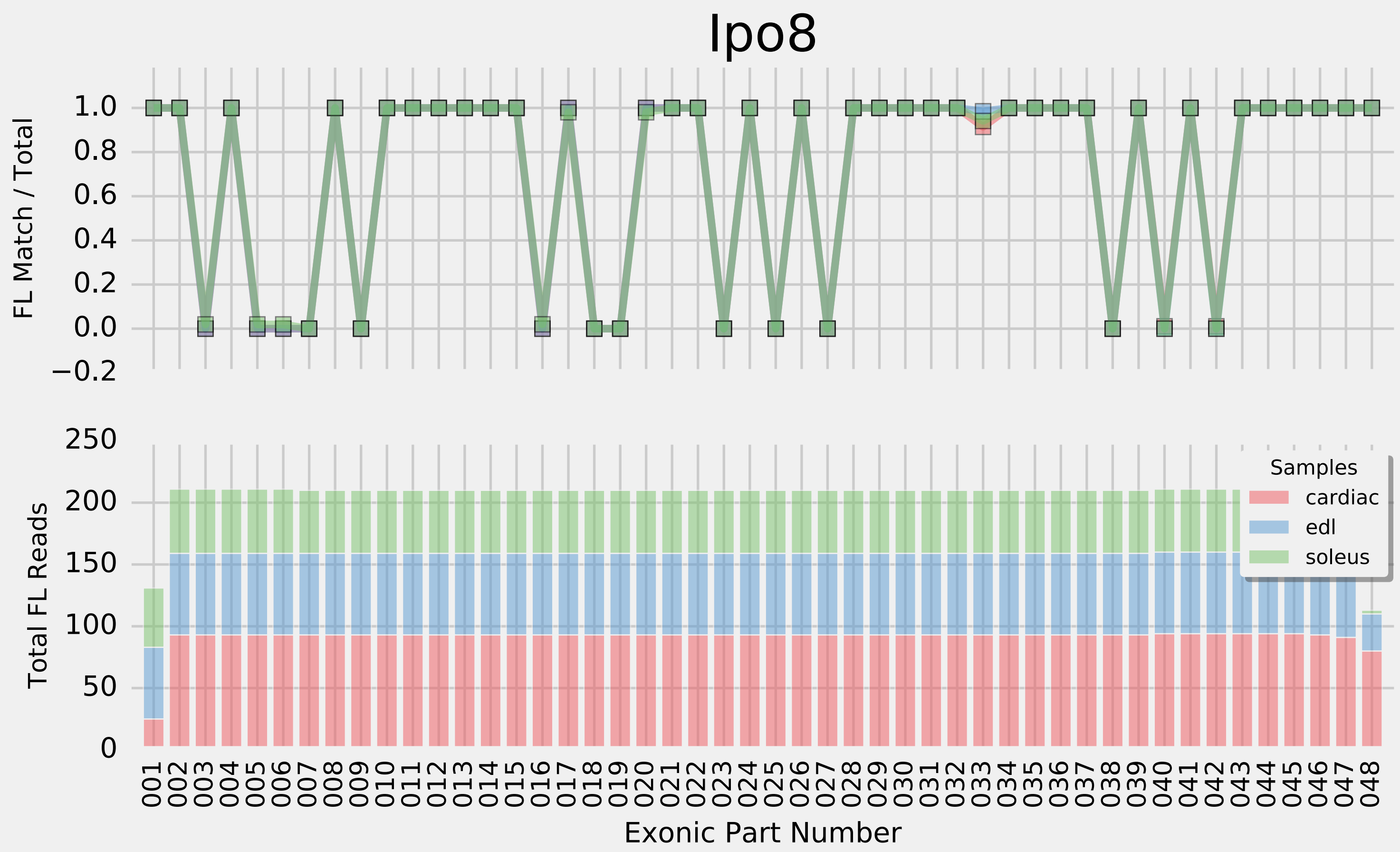
Ipo8
FL Match / Total
1.0
0.8
0.6
0.4
0.2
0.0
−0.2
Total FL Reads
250
200
150
100
50
0
Samples
cardiac
edl
soleus
001 002 003 004 005 006 007 008 009 010 011 012 013 014 015 016 017 018 019 020 021 022 023 024 025 026 027 028 029 030 031 032 033 034 035 036 037 038 039 040 041 042 043 044 045 046 047 048
Exonic Part Number

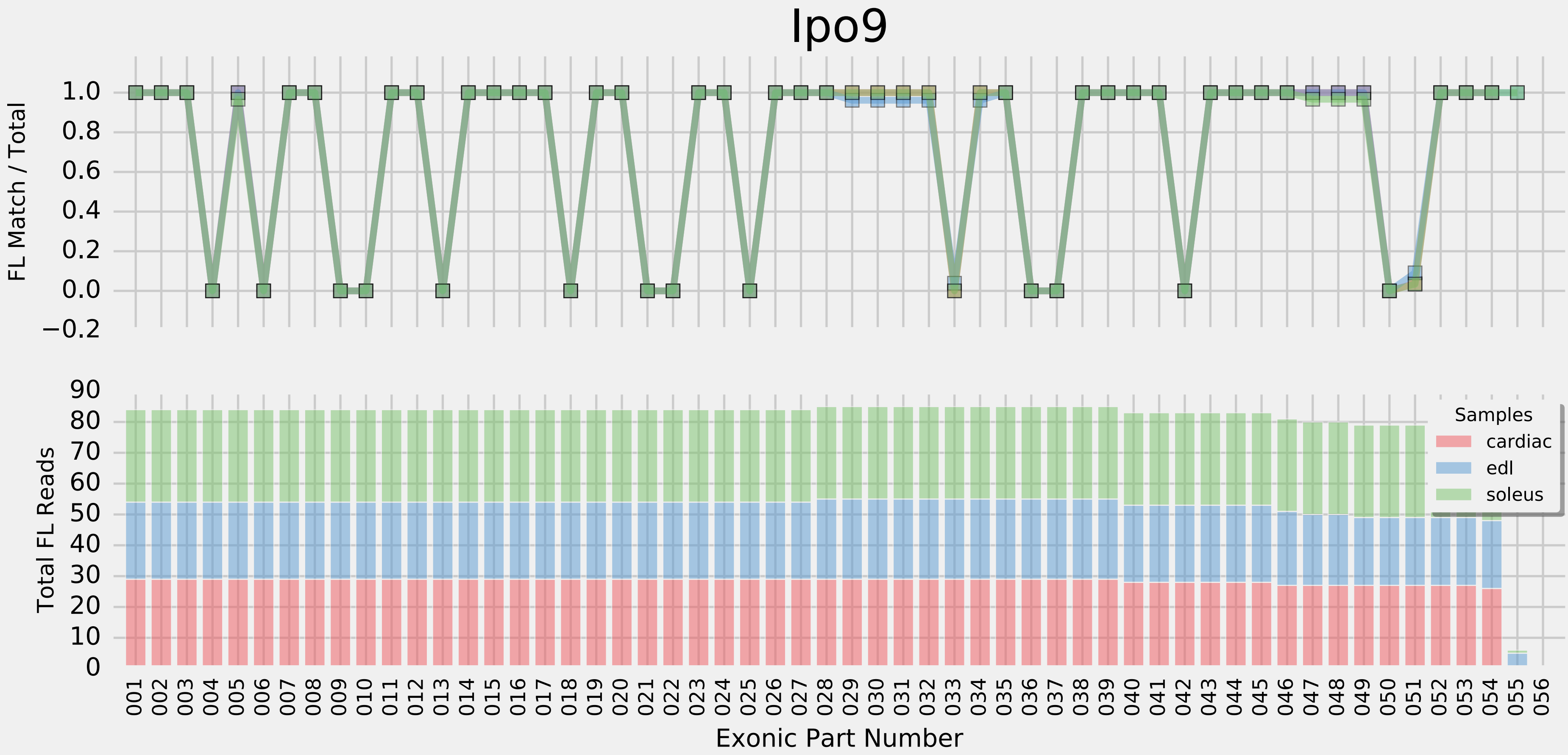

Ipo9
FL Match / Total
Total FL Reads
Exonic Part Number
Samples
cardiac
edl
soleus

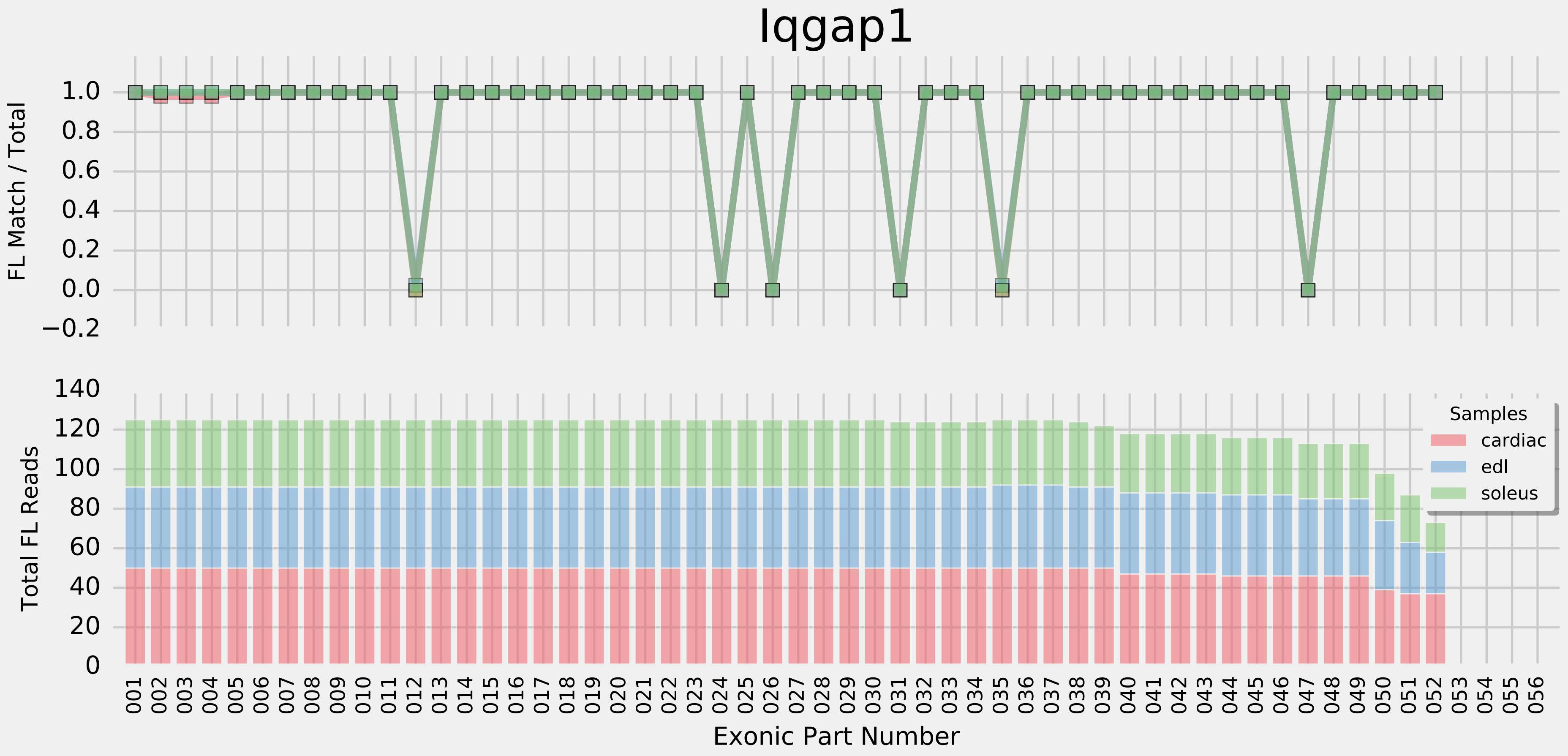

Iqgap1
FL Match / Total
Total FL Reads
Samples
cardiac
edl
soleus
Exonic Part Number

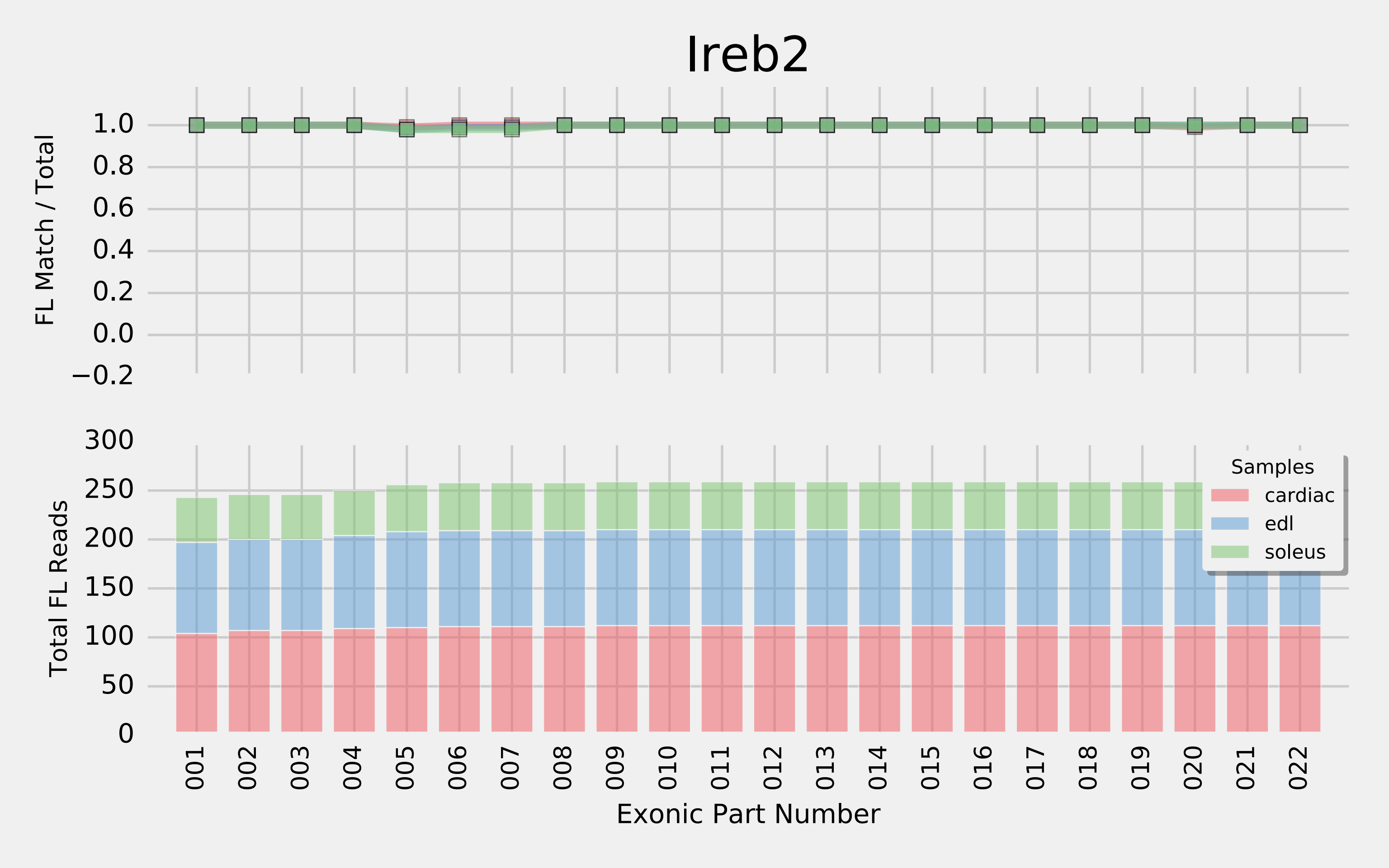

Ireb2
FL Match / Total
1.0
0.8
0.6
0.4
0.2
0.0
−0.2
Total FL Reads
300
250
200
150
100
50
0
Samples
cardiac
edl
soleus
001
002
003
004
005
006
007
008
009
010
011
012
013
014
015
016
017
018
019
020
021
022
Exonic Part Number

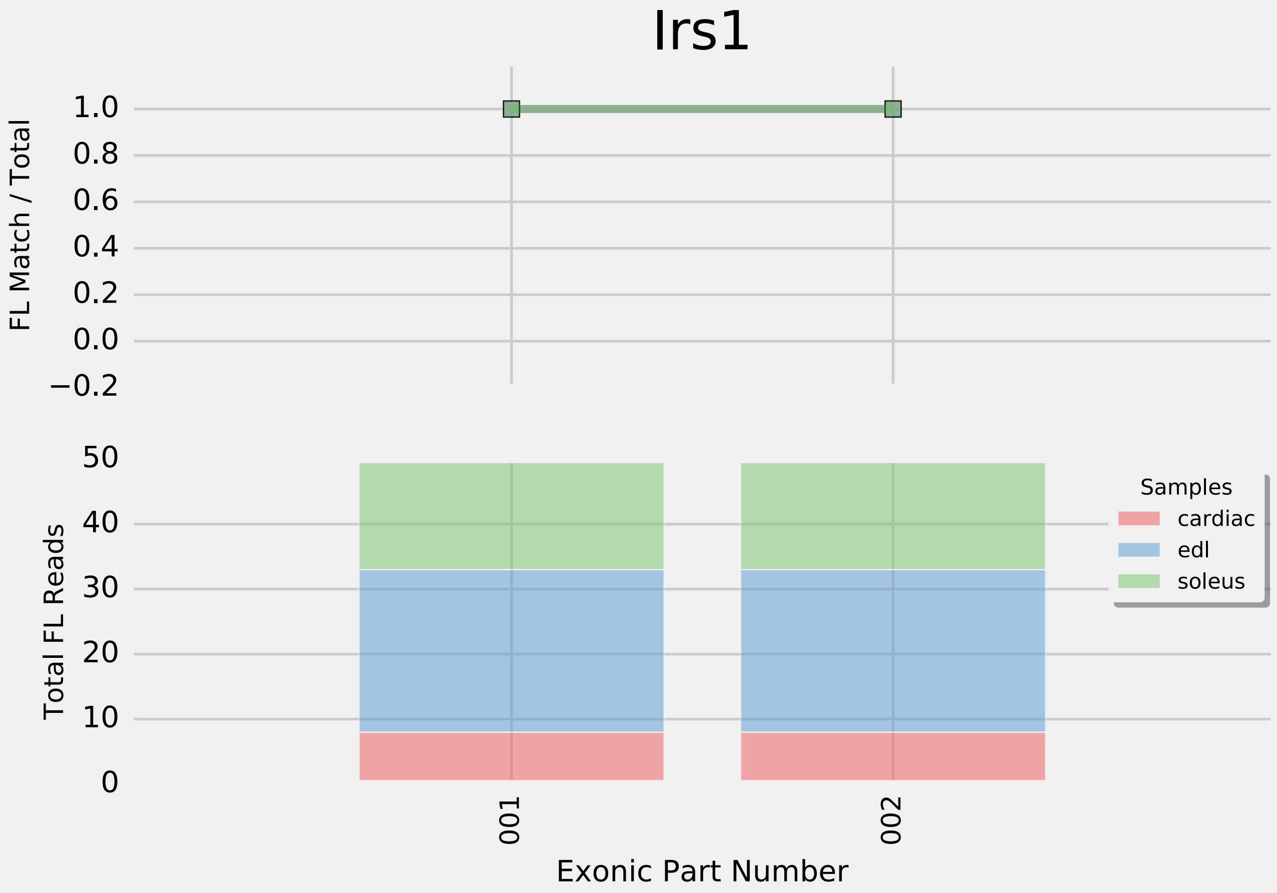

Irs1
FL Match / Total
1.0
0.8
0.6
0.4
0.2
0.0
−0.2
Total FL Reads
50
40
30
20
10
0
Samples
cardiac
edl
soleus
001
002
Exonic Part Number

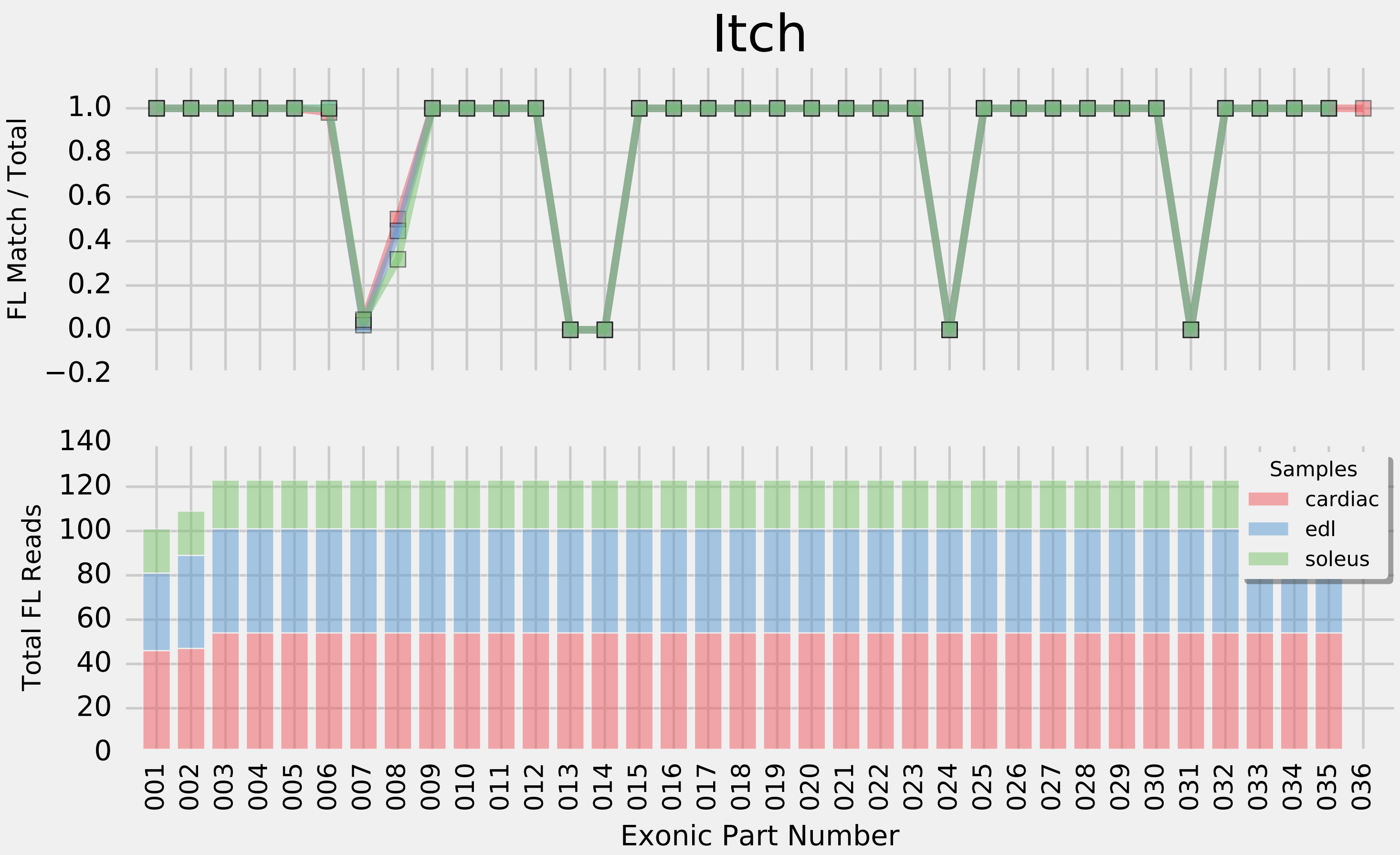

Itch
FL Match / Total
1.0
0.8
0.6
0.4
0.2
0.0
−0.2
Total FL Reads
140
120
100
80
60
40
20
0
Samples
cardiac
edl
soleus
001 002 003 004 005 006 007 008 009 010 011 012 013 014 015 016 017 018 019 020 021 022 023 024 025 026 027 028 029 030 031 032 033 034 035 036
Exonic Part Number

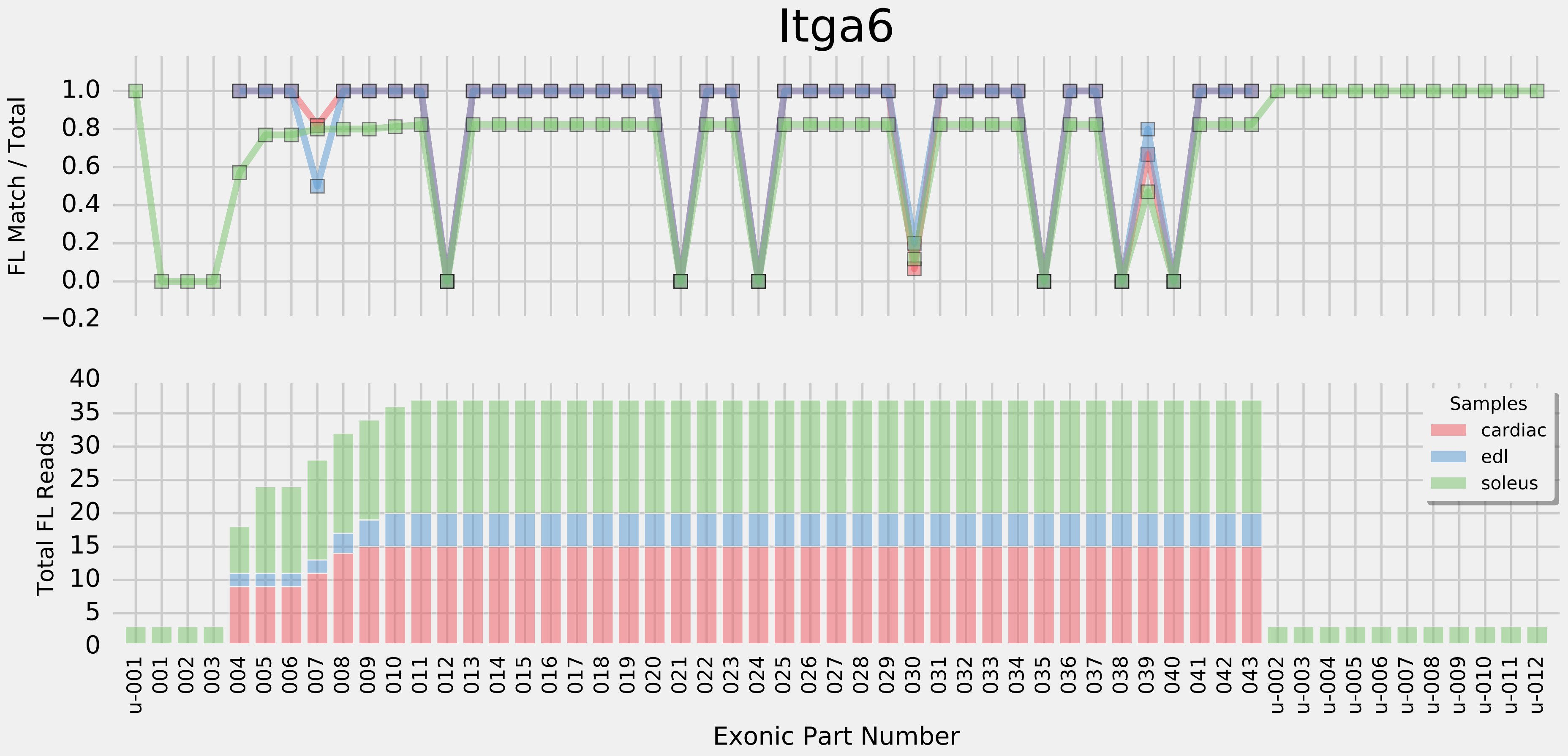

Itga6
FL Match / Total
Total FL Reads
Exonic Part Number
Samples
cardiac
edl
soleus
u-001
001
002
003
004
005
006
007
008
009
010
011
012
013
014
015
016
017
018
019
020
021
022
023
024
025
026
027
028
029
030
031
032
033
034
035
036
037
038
039
040
041
042
043
u-002
u-003
u-004
u-005
u-006
u-007
u-008
u-009
u-010
u-011
u-012

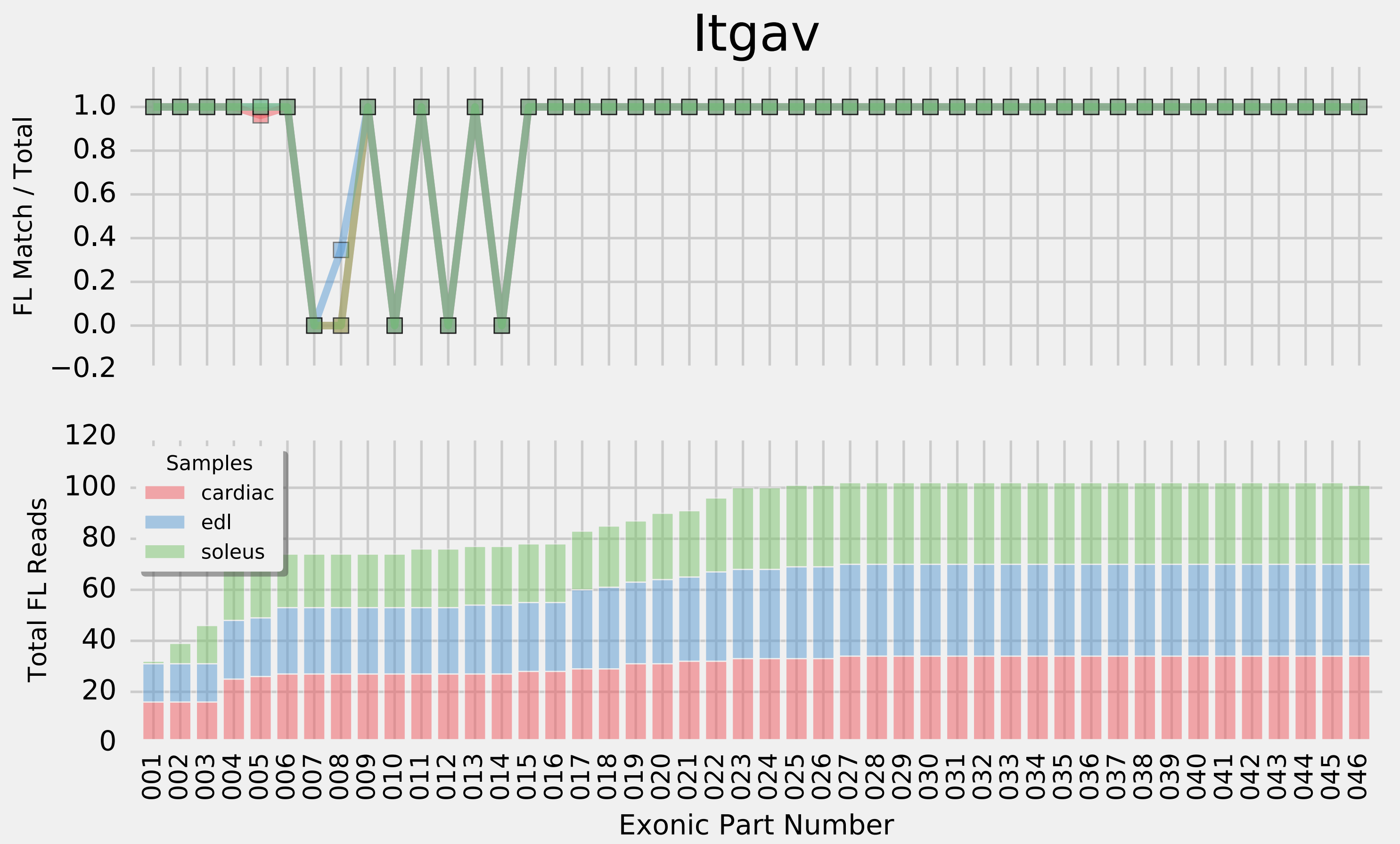
Itgav
FL Match / Total
1.0
0.8
0.6
0.4
0.2
0.0
−0.2
Total FL Reads
120
100
80
60
40
20
0
Samples
cardiac
edl
soleus
001 002 003 004 005 006 007 008 009 010 011 012 013 014 015 016 017 018 019 020 021 022 023 024 025 026 027 028 029 030 031 032 033 034 035 036 037 038 039 040 041 042 043 044 045 046
Exonic Part Number

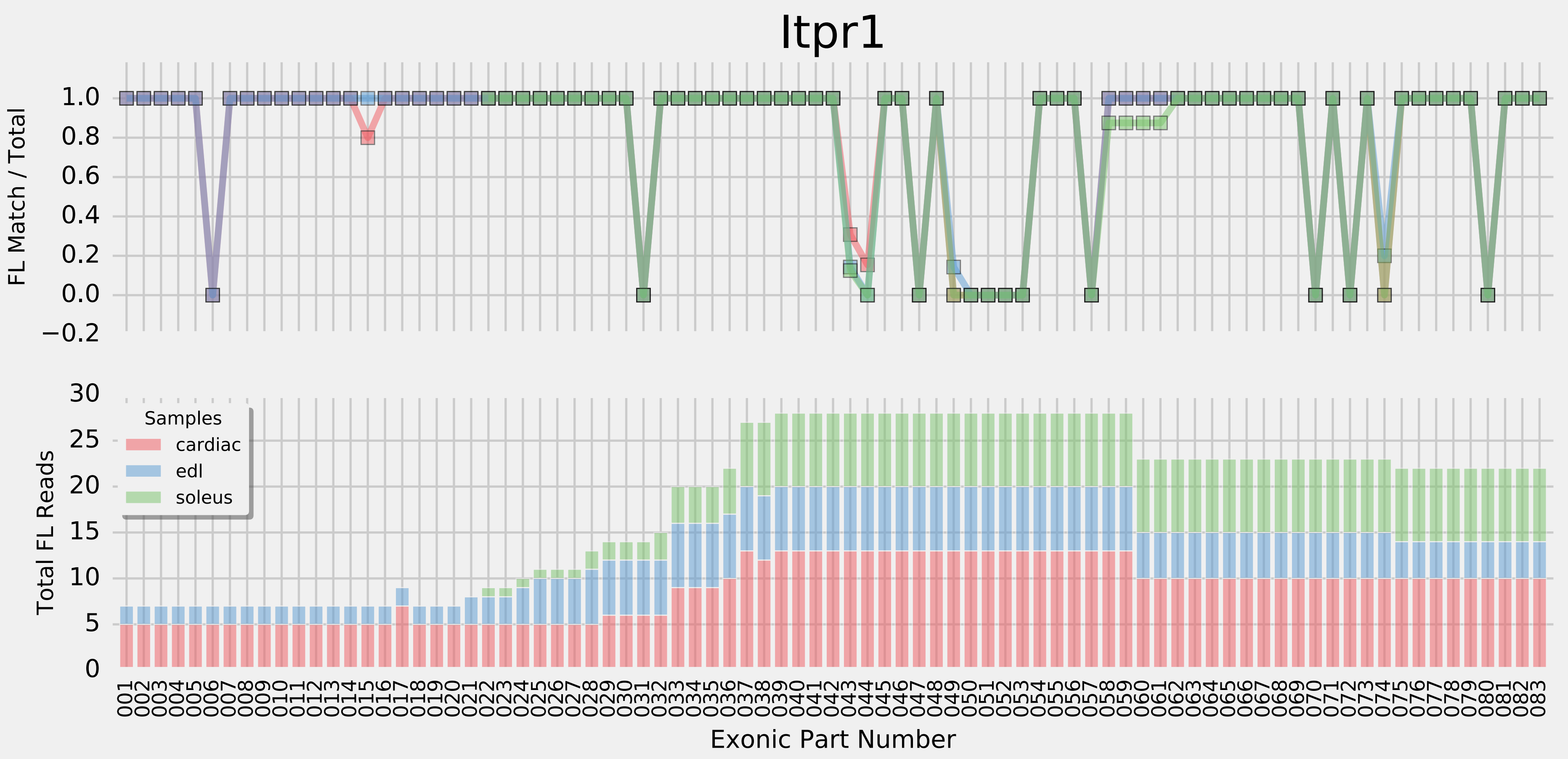
Itpr1
FL Match / Total
Total FL Reads
Samples
cardiac
edl
soleus
Exonic Part Number

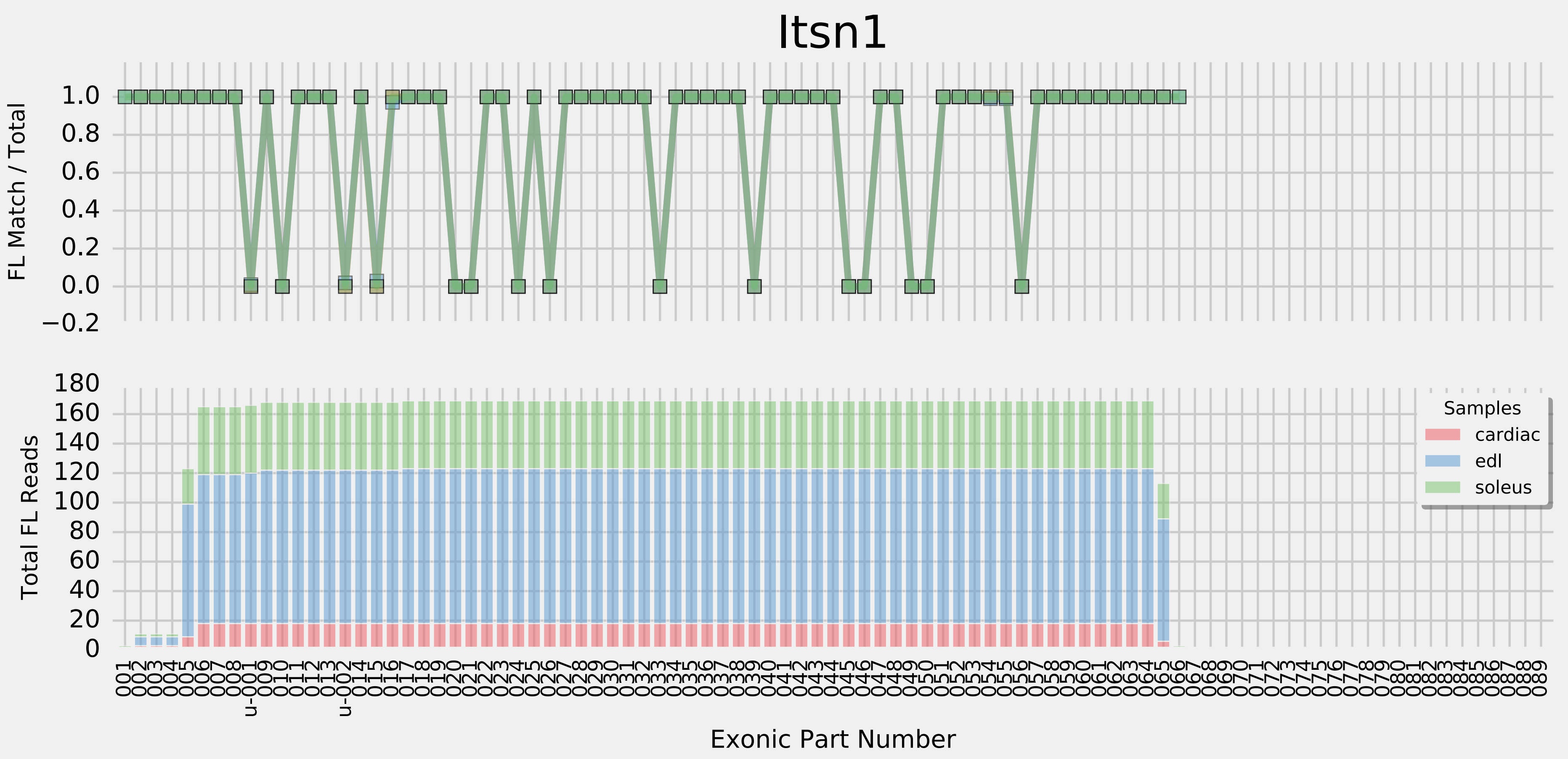

Itsn1
FL Match / Total
Total FL Reads
Exonic Part Number
Samples
cardiac
edl
soleus

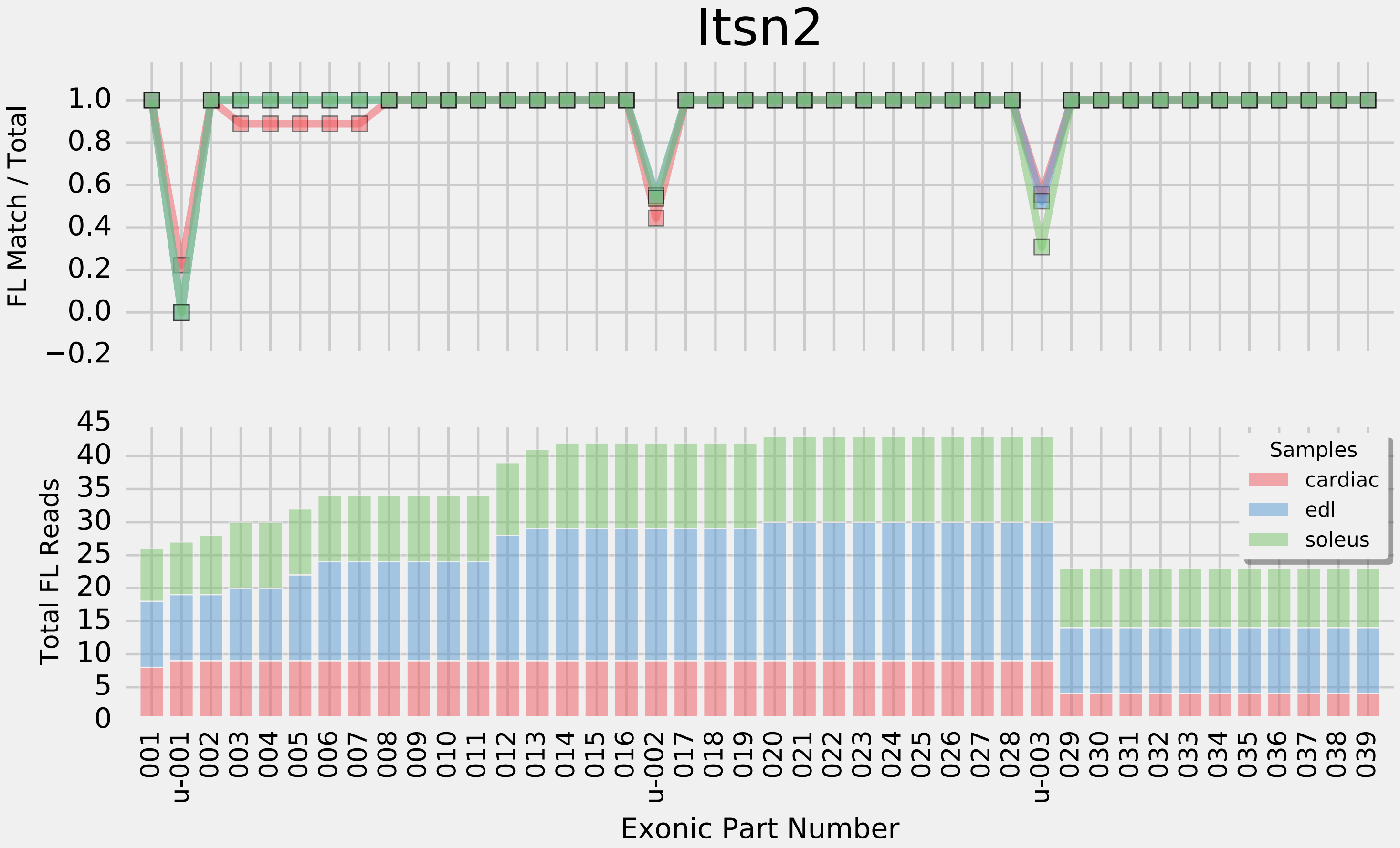
Itsn2
FL Match / Total
1.0
0.8
0.6
0.4
0.2
0.0
−0.2
Total FL Reads
45
40
35
30
25
20
15
10
5
0
Samples
cardiac
edl
soleus
001
u-001
002
003
004
005
006
007
008
009
010
011
012
013
014
015
016
u-002
017
018
019
020
021
022
023
024
025
026
027
028
u-003
029
030
031
032
033
034
035
036
037
038
039
Exonic Part Number

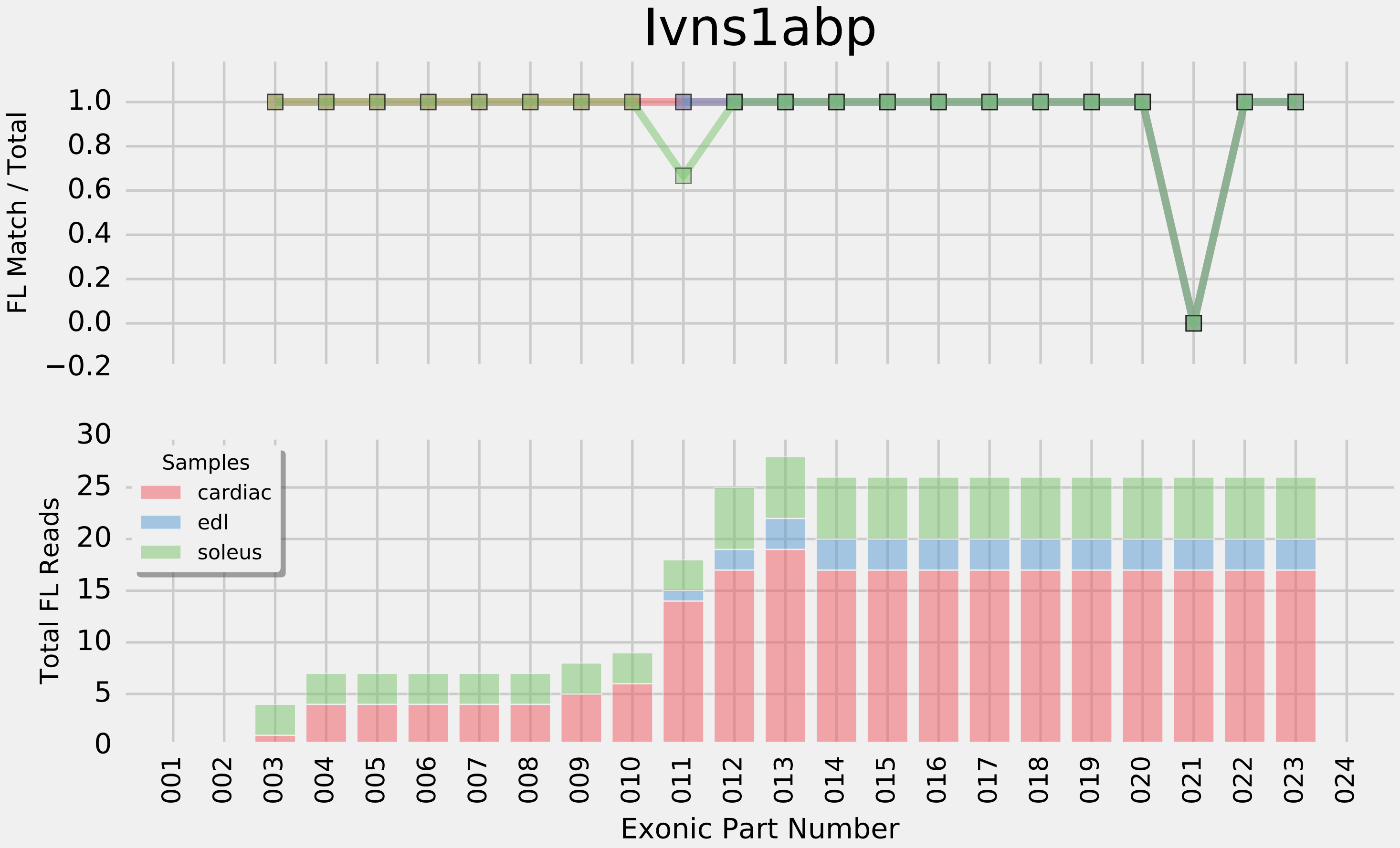
Ivns1abp
FL Match / Total
1.0
0.8
0.6
0.4
0.2
0.0
−0.2
Total FL Reads
30
25
20
15
10
5
0
Samples
cardiac
edl
soleus
001
002
003
004
005
006
007
008
009
010
011
012
013
014
015
016
017
018
019
020
021
022
023
024
Exonic Part Number

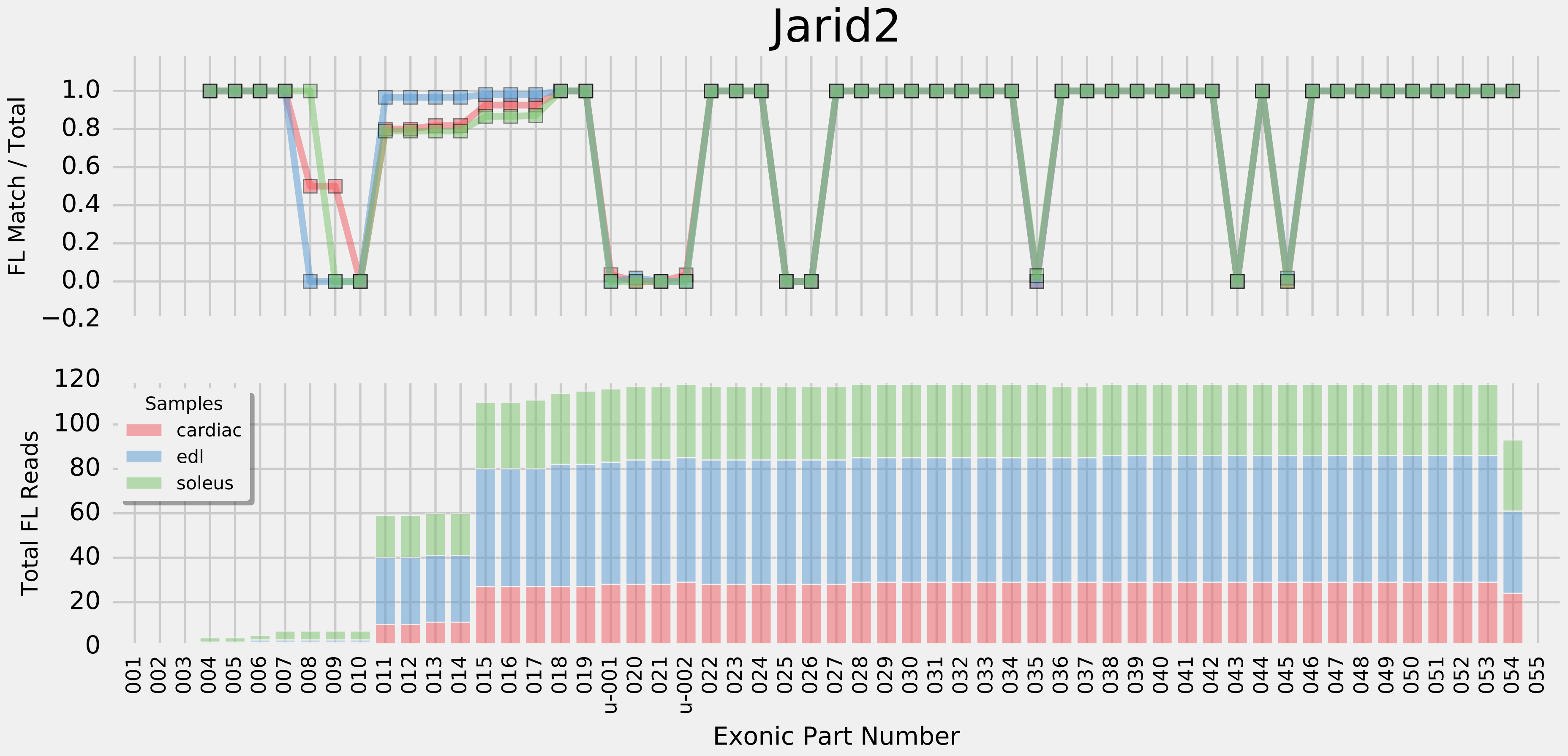

Jarid2
FL Match / Total
1.0
0.8
0.6
0.4
0.2
0.0
−0.2
Total FL Reads
120
100
80
60
40
20
0
Samples
cardiac
edl
soleus
001 002 003 004 005 006 007 008 009 010 011 012 013 014 015 016 017 018 019 u-001 020 021 u-002 022 023 024 025 026 027 028 029 030 031 032 033 034 035 036 037 038 039 040 041 042 043 044 045 046 047 048 049 050 051 052 053 054 055
Exonic Part Number

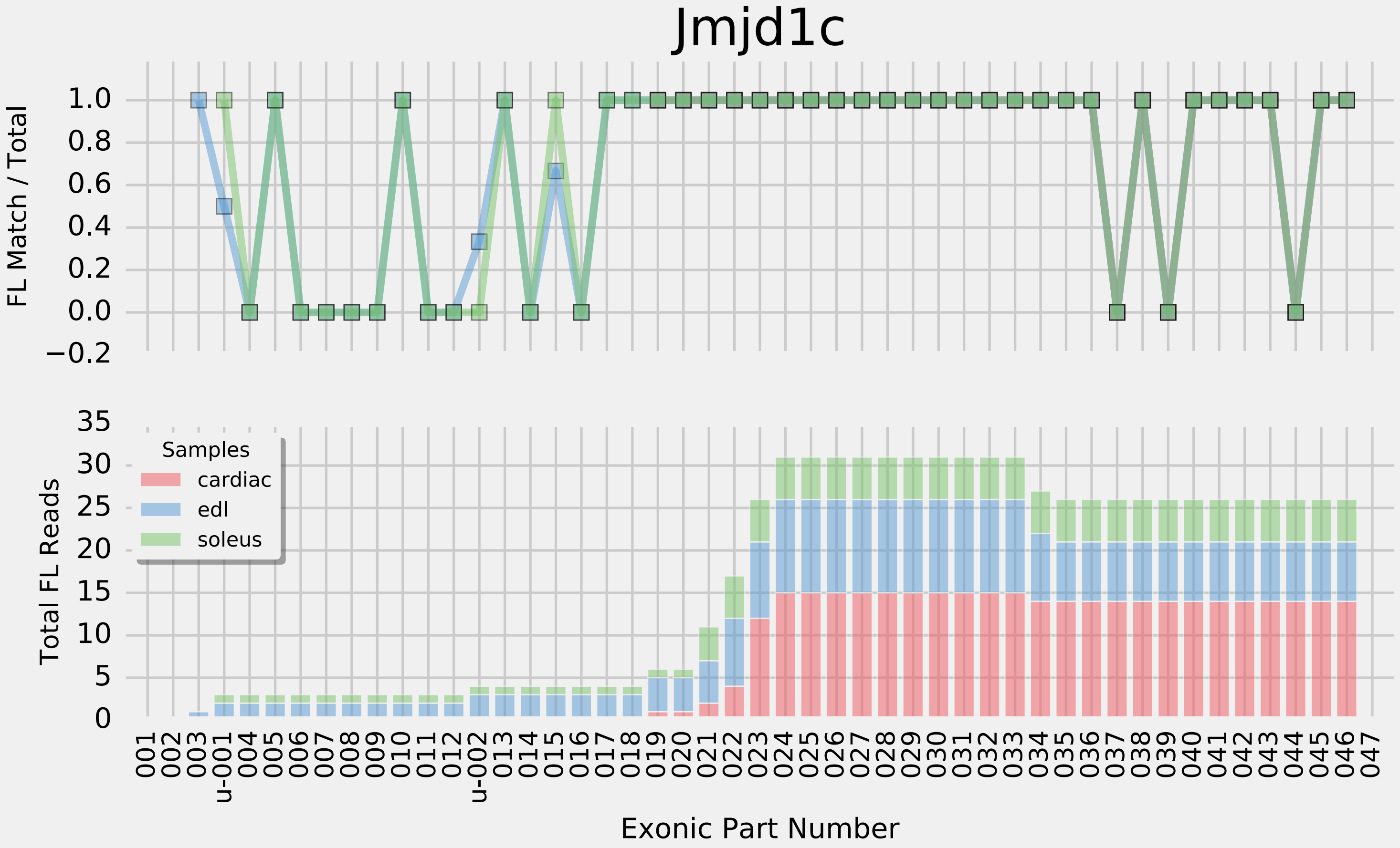

Jmjd1c
FL Match / Total
1.0
0.8
0.6
0.4
0.2
0.0
−0.2
Total FL Reads
35
30
25
20
15
10
5
0
Samples
cardiac
edl
soleus
001
002
003
u-001
004
005
006
007
008
009
010
011
012
u-002
013
014
015
016
017
018
019
020
021
022
023
024
025
026
027
028
029
030
031
032
033
034
035
036
037
038
039
040
041
042
043
044
045
046
047
Exonic Part Number

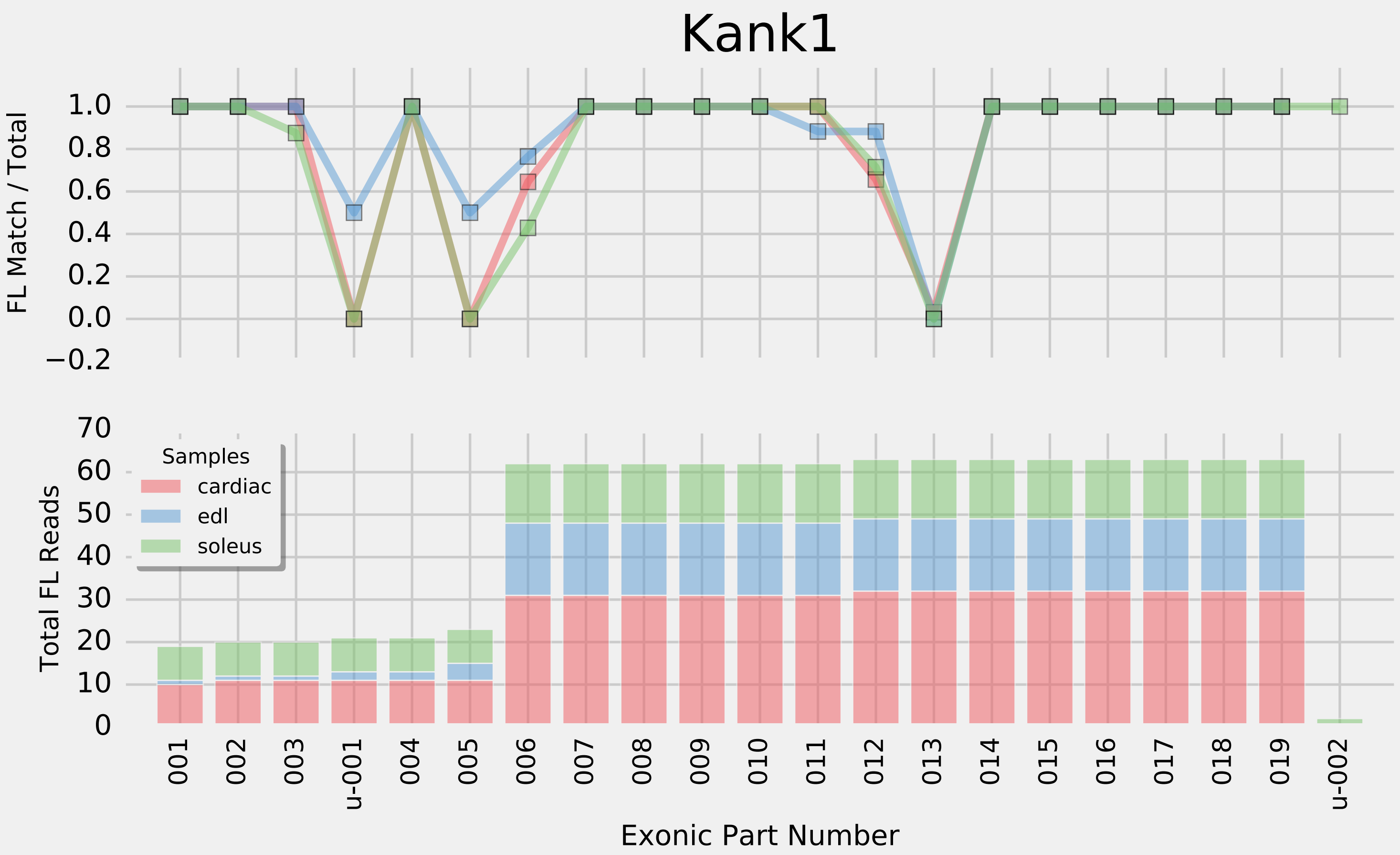

Kank1
FL Match / Total
1.0
0.8
0.6
0.4
0.2
0.0
−0.2
Total FL Reads
70
60
50
40
30
20
10
0
Samples
cardiac
edl
soleus
001
002
003
u-001
004
005
006
007
008
009
010
011
012
013
014
015
016
017
018
019
u-002
Exonic Part Number

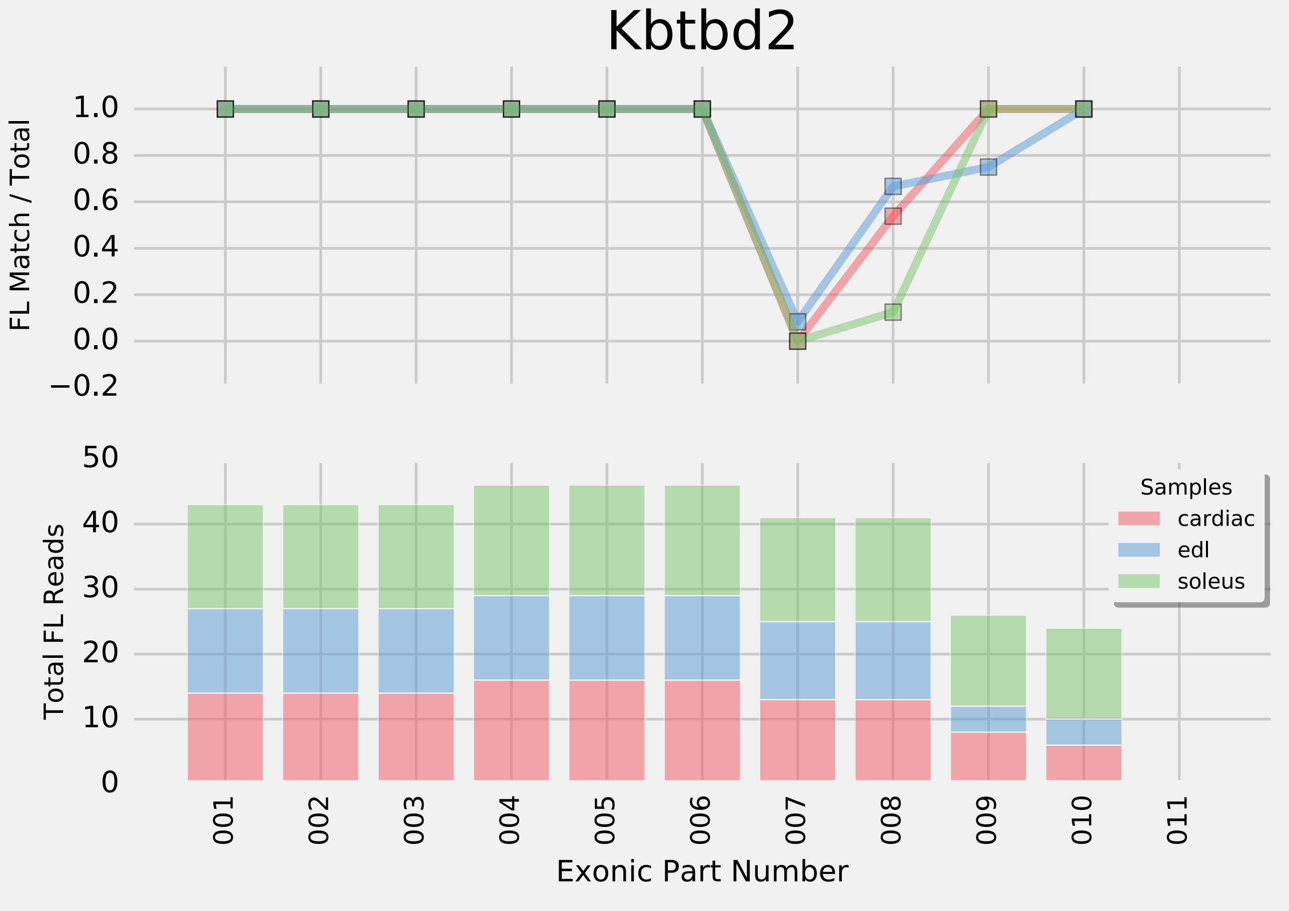

Kbtbd2
FL Match / Total
1.0
0.8
0.6
0.4
0.2
0.0
−0.2
Total FL Reads
50
40
30
20
10
0
Samples
cardiac
edl
soleus
001
002
003
004
005
006
007
008
009
010
011
Exonic Part Number

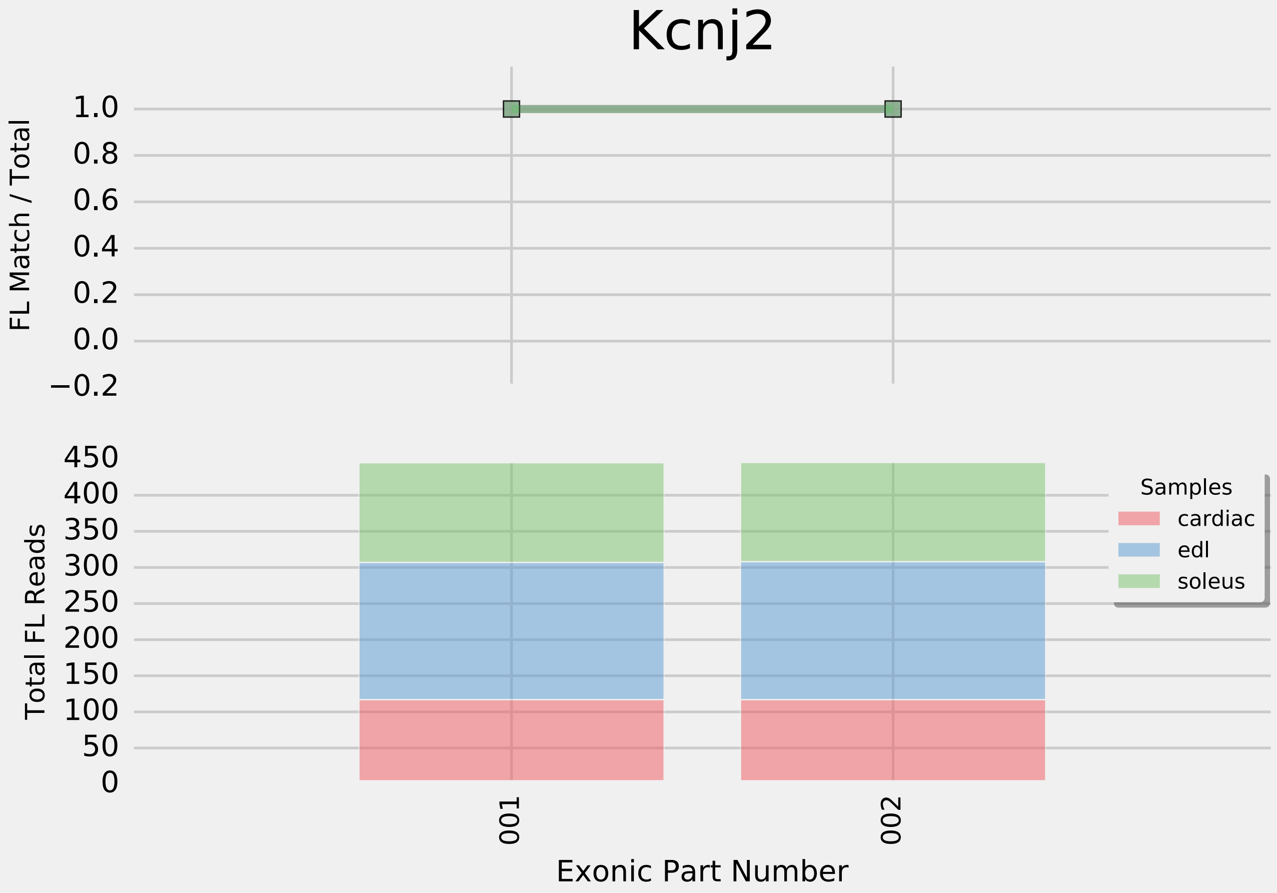

Kcnj2
FL Match / Total
1.0
0.8
0.6
0.4
0.2
0.0
−0.2
Total FL Reads
450
400
350
300
250
200
150
100
50
0
Samples
cardiac
edl
soleus
001
002
Exonic Part Number

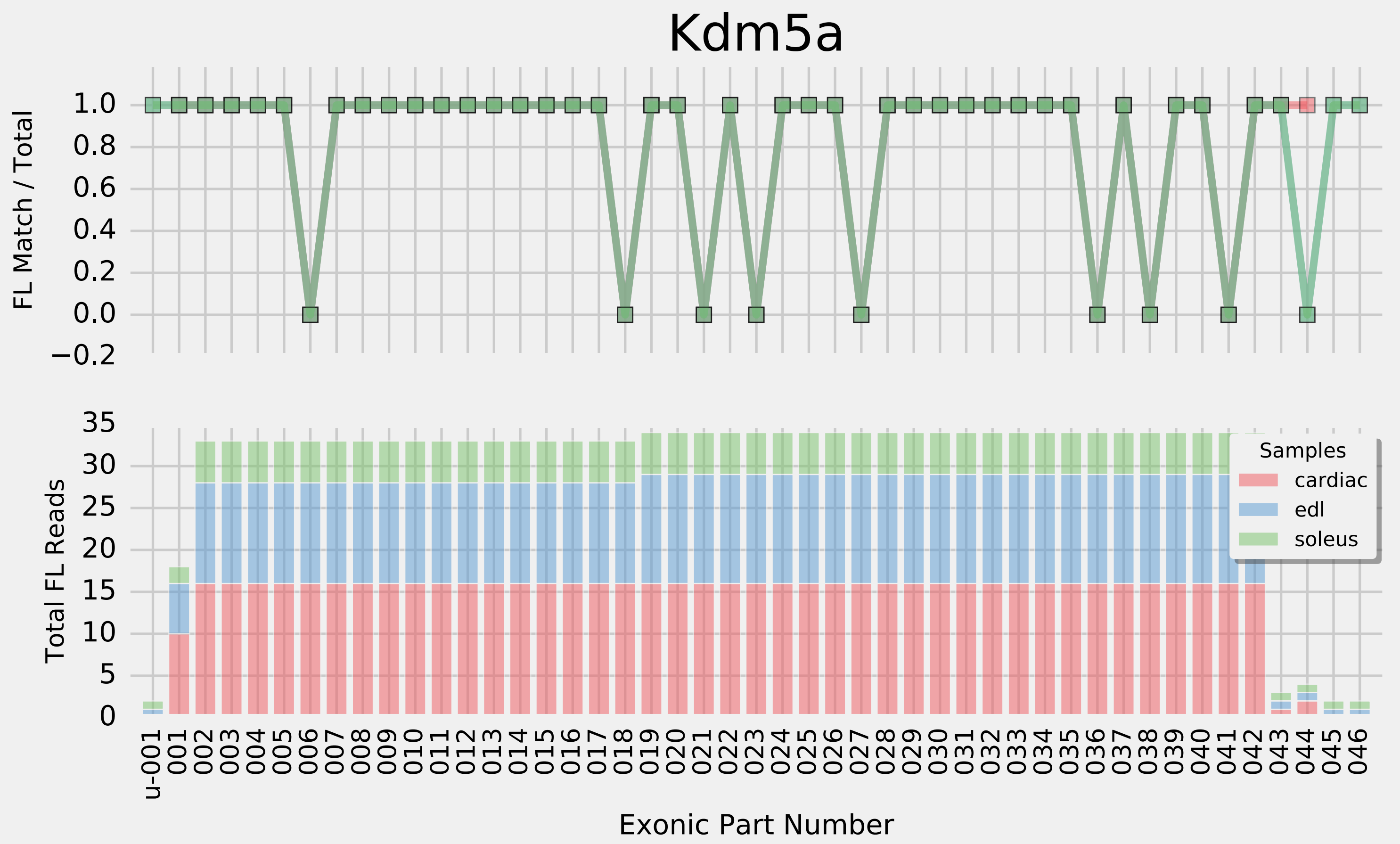
Kdm5a
FL Match / Total
1.0
0.8
0.6
0.4
0.2
0.0
−0.2
Total FL Reads
35
30
25
20
15
10
5
0
Samples
cardiac
edl
soleus
u-001
001
002
003
004
005
006
007
008
009
010
011
012
013
014
015
016
017
018
019
020
021
022
023
024
025
026
027
028
029
030
031
032
033
034
035
036
037
038
039
040
041
042
043
044
045
046
Exonic Part Number

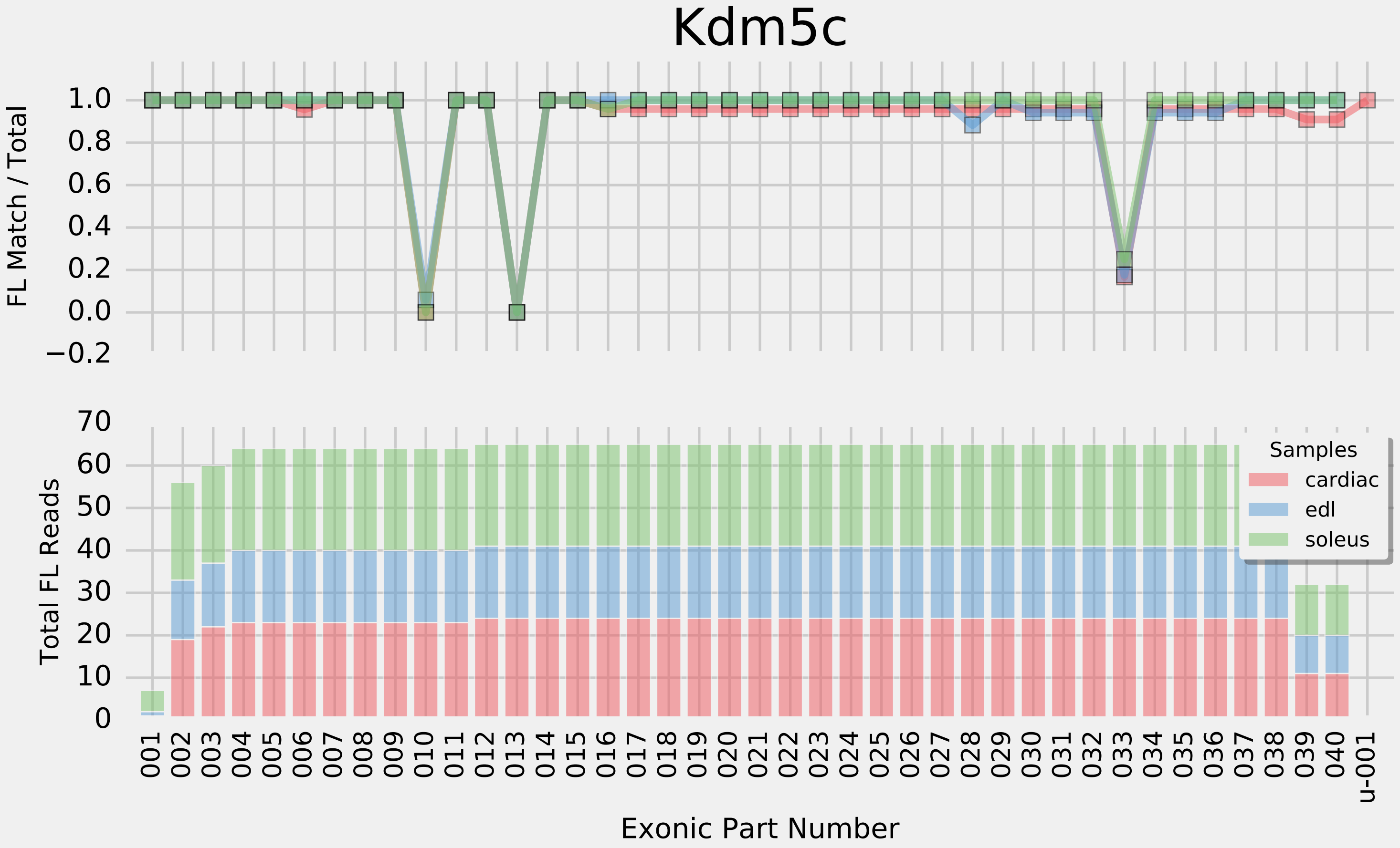

Kdm5c
FL Match / Total
1.0
0.8
0.6
0.4
0.2
0.0
−0.2
70
60
50
40
30
20
10
0
Total FL Reads
Samples
cardiac
edl
soleus
001
002
003
004
005
006
007
008
009
010
011
012
013
014
015
016
017
018
019
020
021
022
023
024
025
026
027
028
029
030
031
032
033
034
035
036
037
038
039
040
u-001
Exonic Part Number

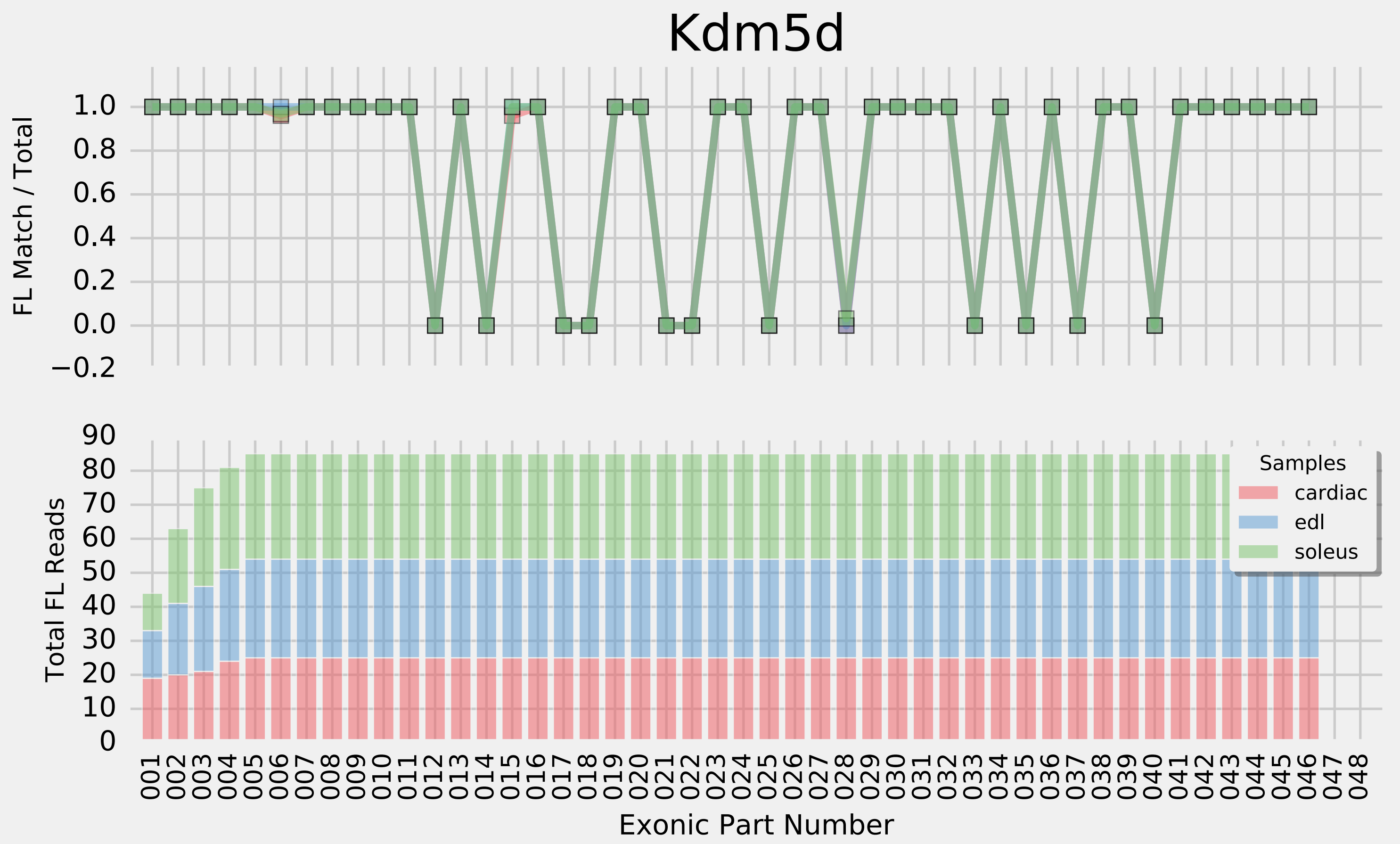

Kdm5d
FL Match / Total
1.0
0.8
0.6
0.4
0.2
0.0
−0.2
Total FL Reads
90
80
70
60
50
40
30
20
10
0
Samples
cardiac
edl
soleus
001 002 003 004 005 006 007 008 009 010 011 012 013 014 015 016 017 018 019 020 021 022 023 024 025 026 027 028 029 030 031 032 033 034 035 036 037 038 039 040 041 042 043 044 045 046 047 048
Exonic Part Number

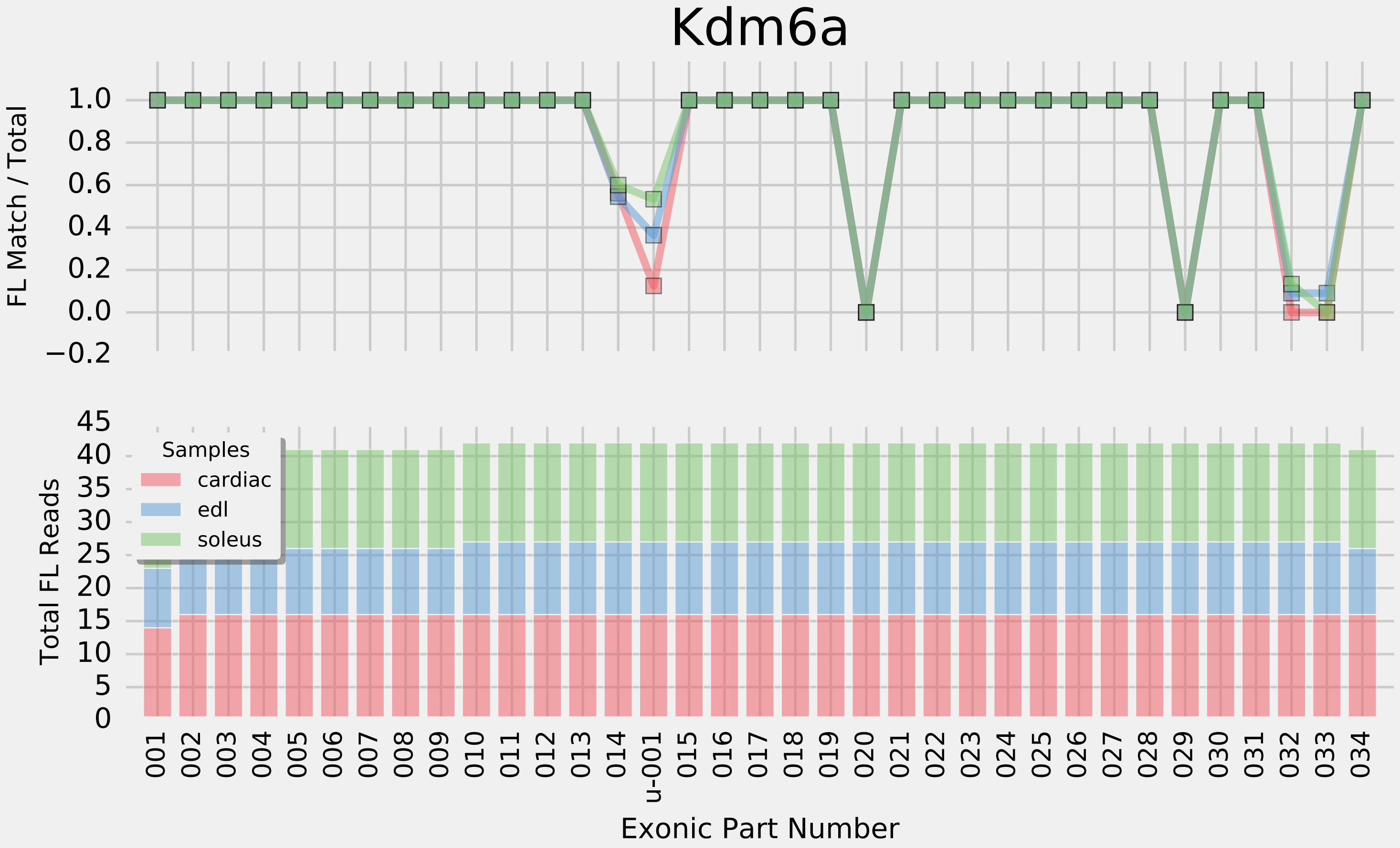
Kdm6a
FL Match / Total
1.0
0.8
0.6
0.4
0.2
0.0
−0.2
Total FL Reads
45
40
35
30
25
20
15
10
5
0
Samples
cardiac
edl
soleus
001
002
003
004
005
006
007
008
009
010
011
012
013
014
u-001
015
016
017
018
019
020
021
022
023
024
025
026
027
028
029
030
031
032
033
034
Exonic Part Number

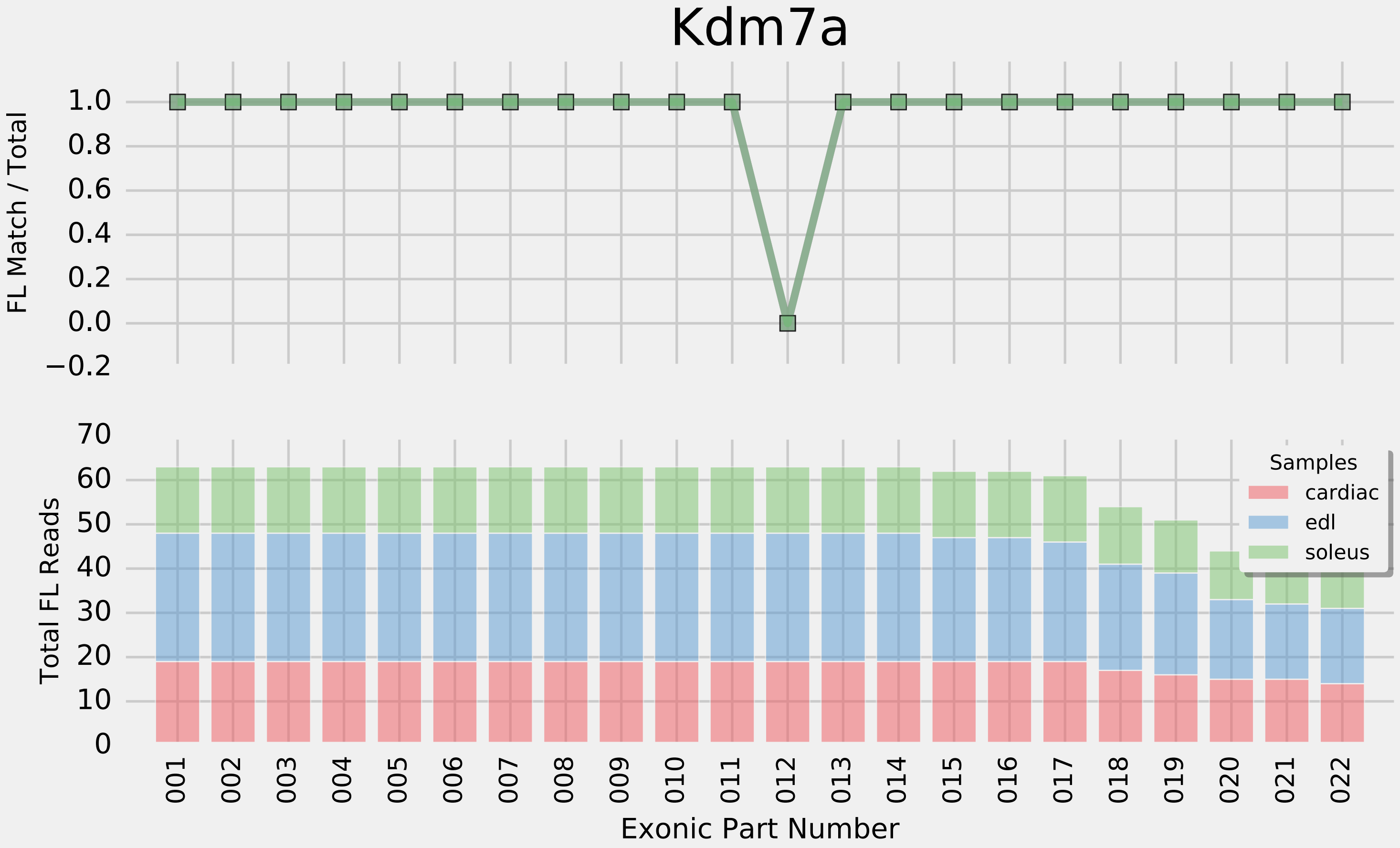
Kdm7a
FL Match / Total
1.0
0.8
0.6
0.4
0.2
0.0
−0.2
Total FL Reads
70
60
50
40
30
20
10
0
Samples
cardiac
edl
soleus
001
002
003
004
005
006
007
008
009
010
011
012
013
014
015
016
017
018
019
020
021
022
Exonic Part Number

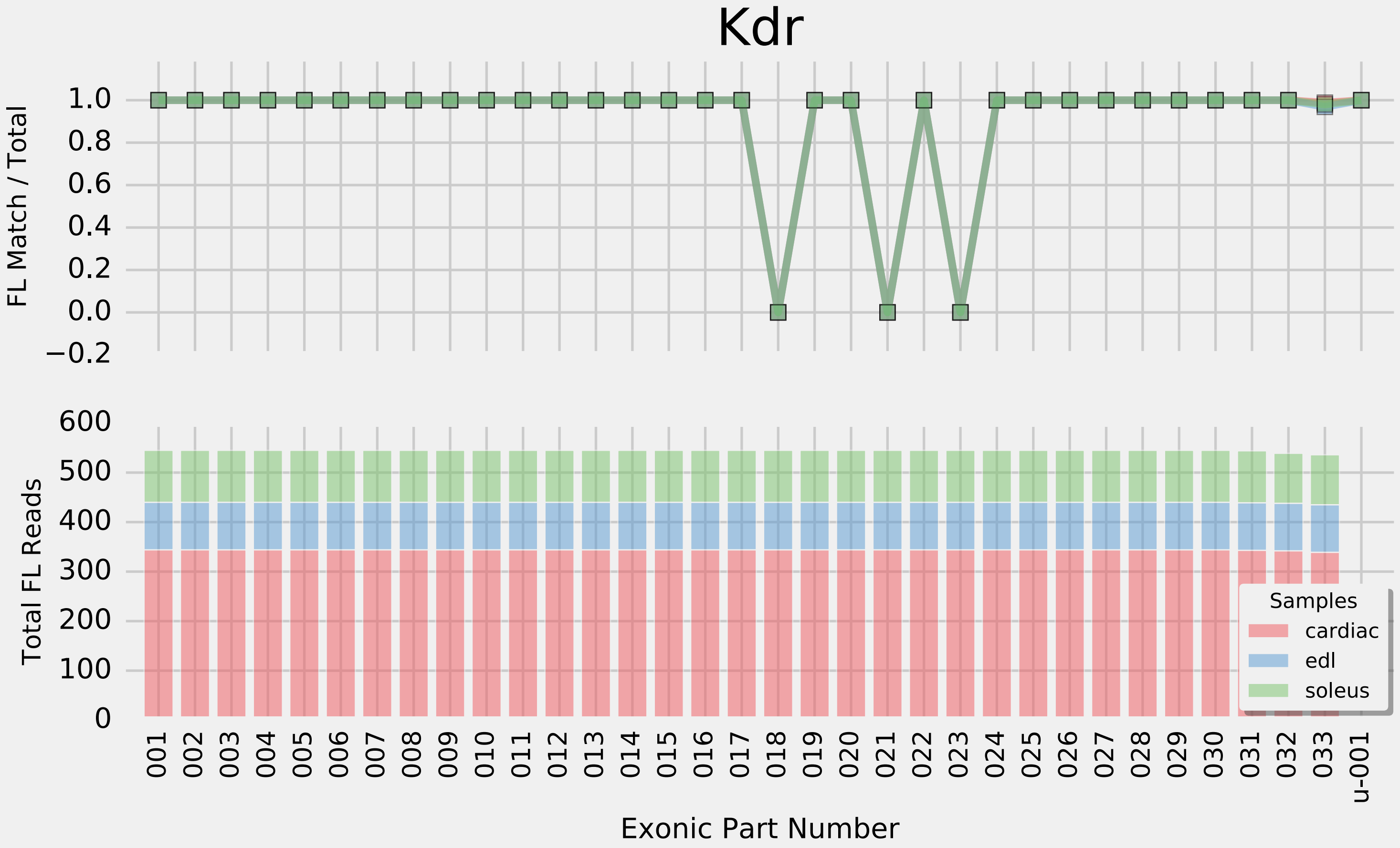

Kdr
FL Match / Total
1.0
0.8
0.6
0.4
0.2
0.0
−0.2
Total FL Reads
600
500
400
300
200
100
0
Samples
cardiac
edl
soleus
001
002
003
004
005
006
007
008
009
010
011
012
013
014
015
016
017
018
019
020
021
022
023
024
025
026
027
028
029
030
031
032
033
u-001
Exonic Part Number

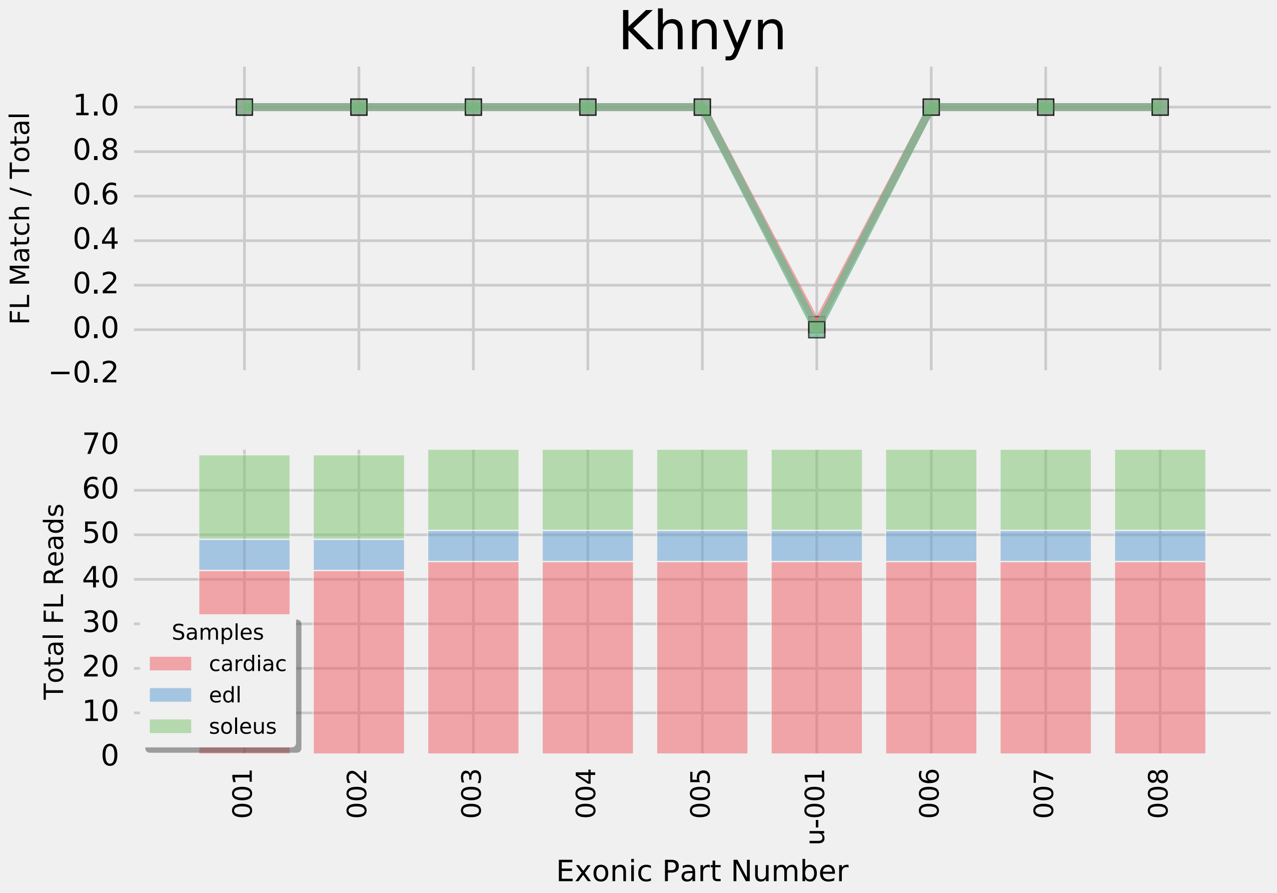
Khnyn
FL Match / Total
1.0
0.8
0.6
0.4
0.2
0.0
−0.2
Total FL Reads
70
60
50
40
30
20
10
0
Samples
cardiac
edl
soleus
001
002
003
004
005
u-001
006
007
008
Exonic Part Number

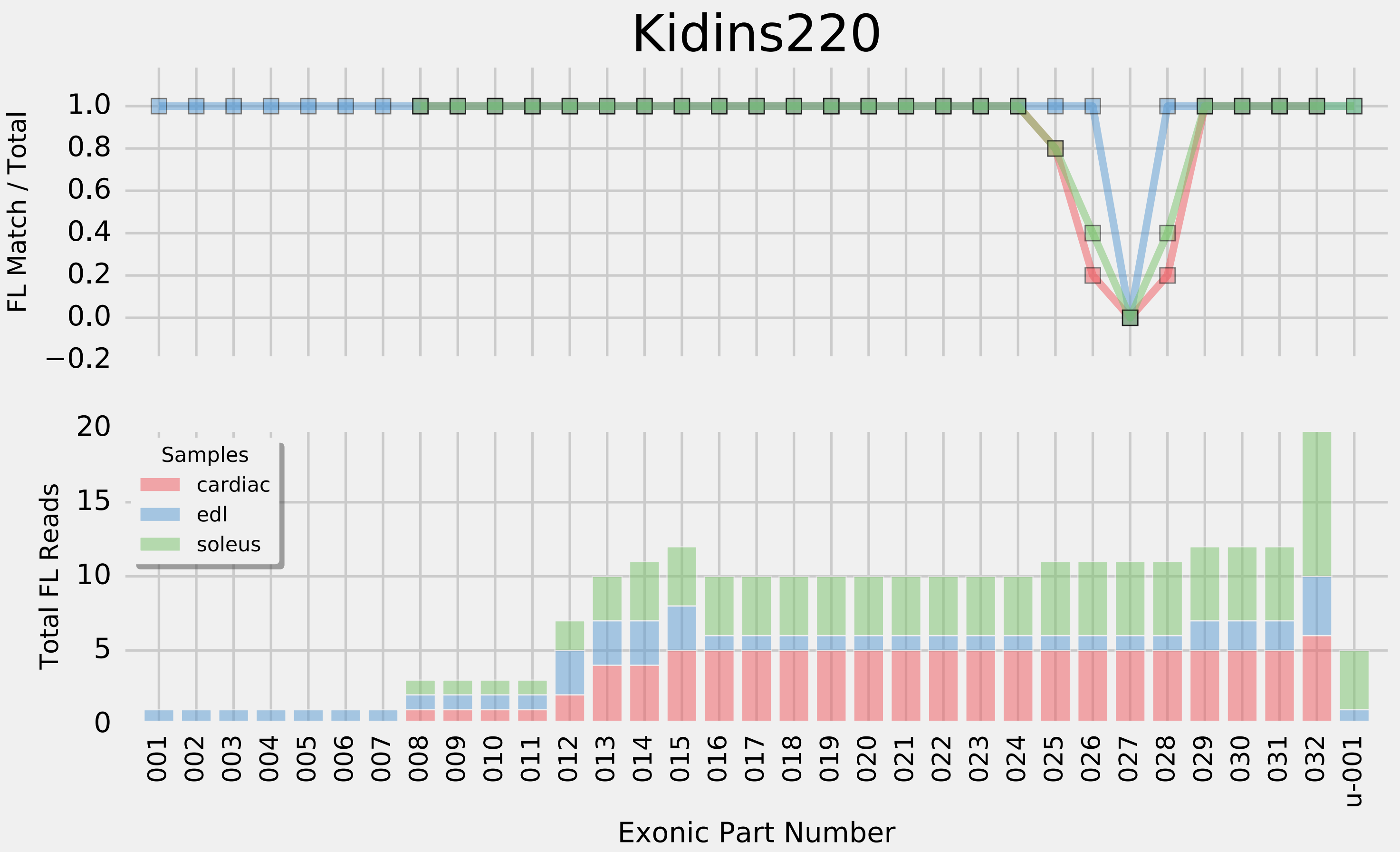

Kidins220
FL Match / Total
1.0
0.8
0.6
0.4
0.2
0.0
−0.2
Total FL Reads
20
15
10
5
0
Samples
cardiac
edl
soleus
001
002
003
004
005
006
007
008
009
010
011
012
013
014
015
016
017
018
019
020
021
022
023
024
025
026
027
028
029
030
031
032
u-001
Exonic Part Number

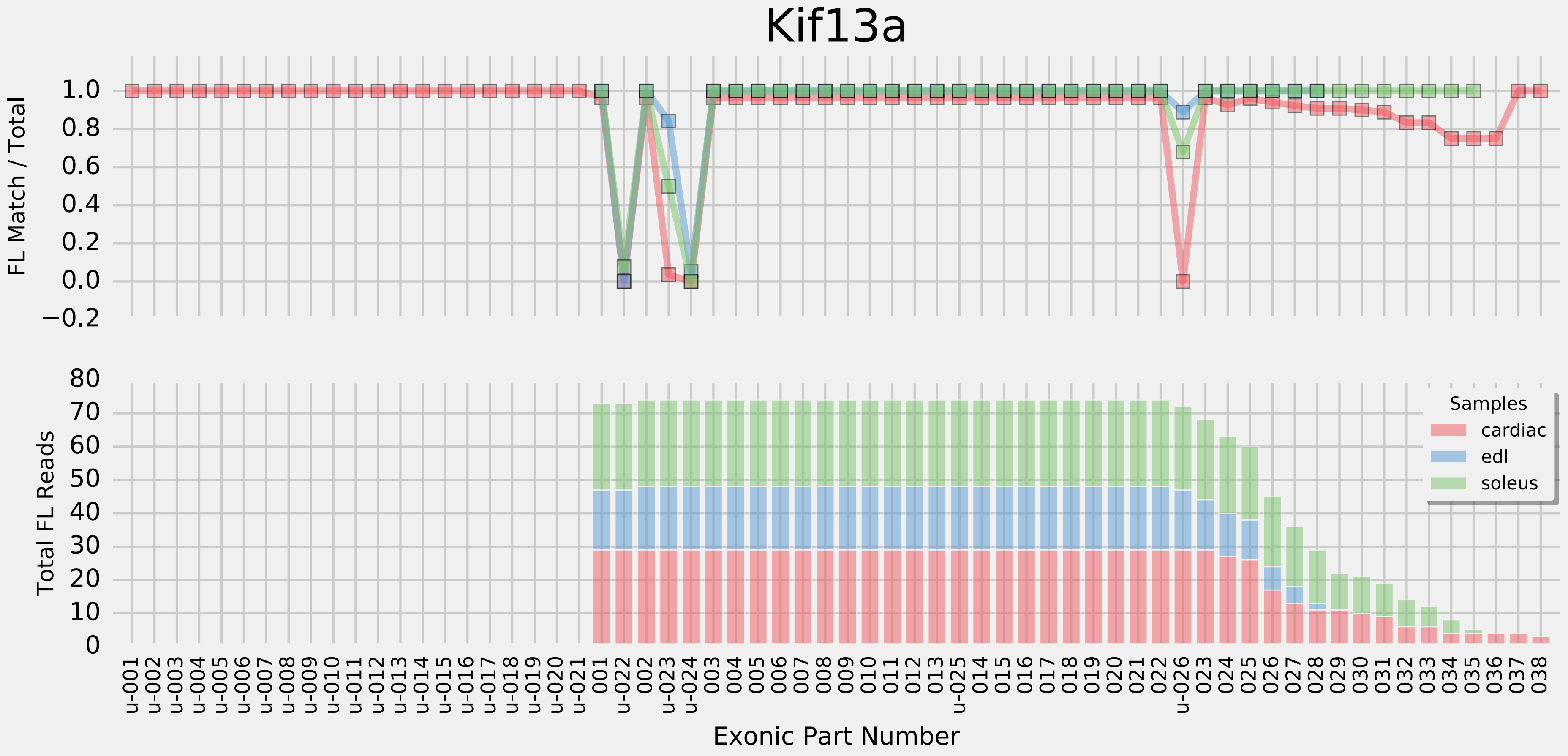

Kif13a
FL Match / Total
Total FL Reads
Samples
cardiac
edl
soleus
Exonic Part Number

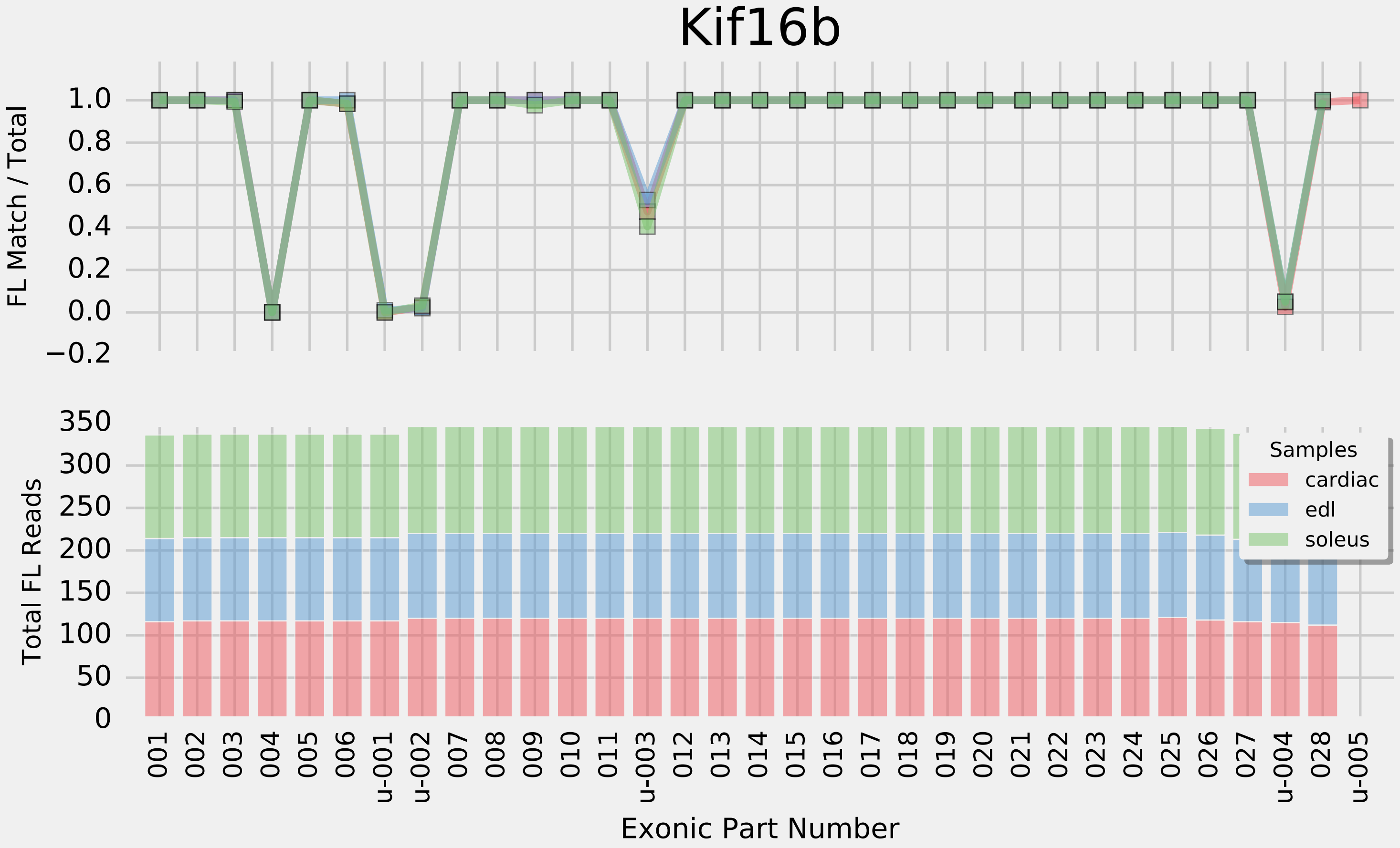

Kif16b
FL Match / Total
1.0
0.8
0.6
0.4
0.2
0.0
−0.2
Total FL Reads
350
300
250
200
150
100
50
0
Samples
cardiac
edl
soleus
001
002
003
004
005
006
u-001
u-002
007
008
009
010
011
u-003
012
013
014
015
016
017
018
019
020
021
022
023
024
025
026
027
u-004
028
u-005
Exonic Part Number

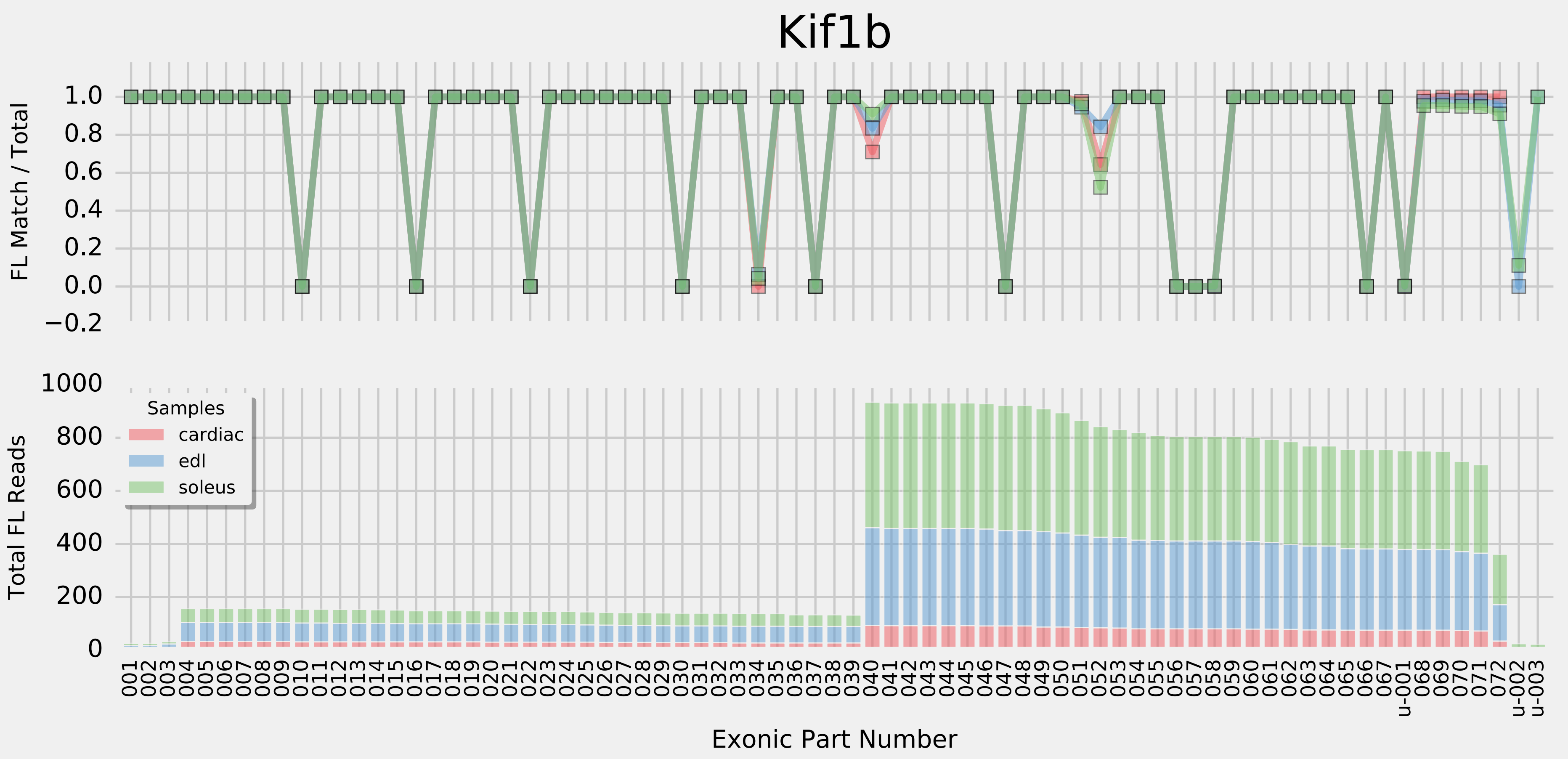
Kif1b
FL Match / Total
Total FL Reads
Samples
cardiac
edl
soleus
Exonic Part Number

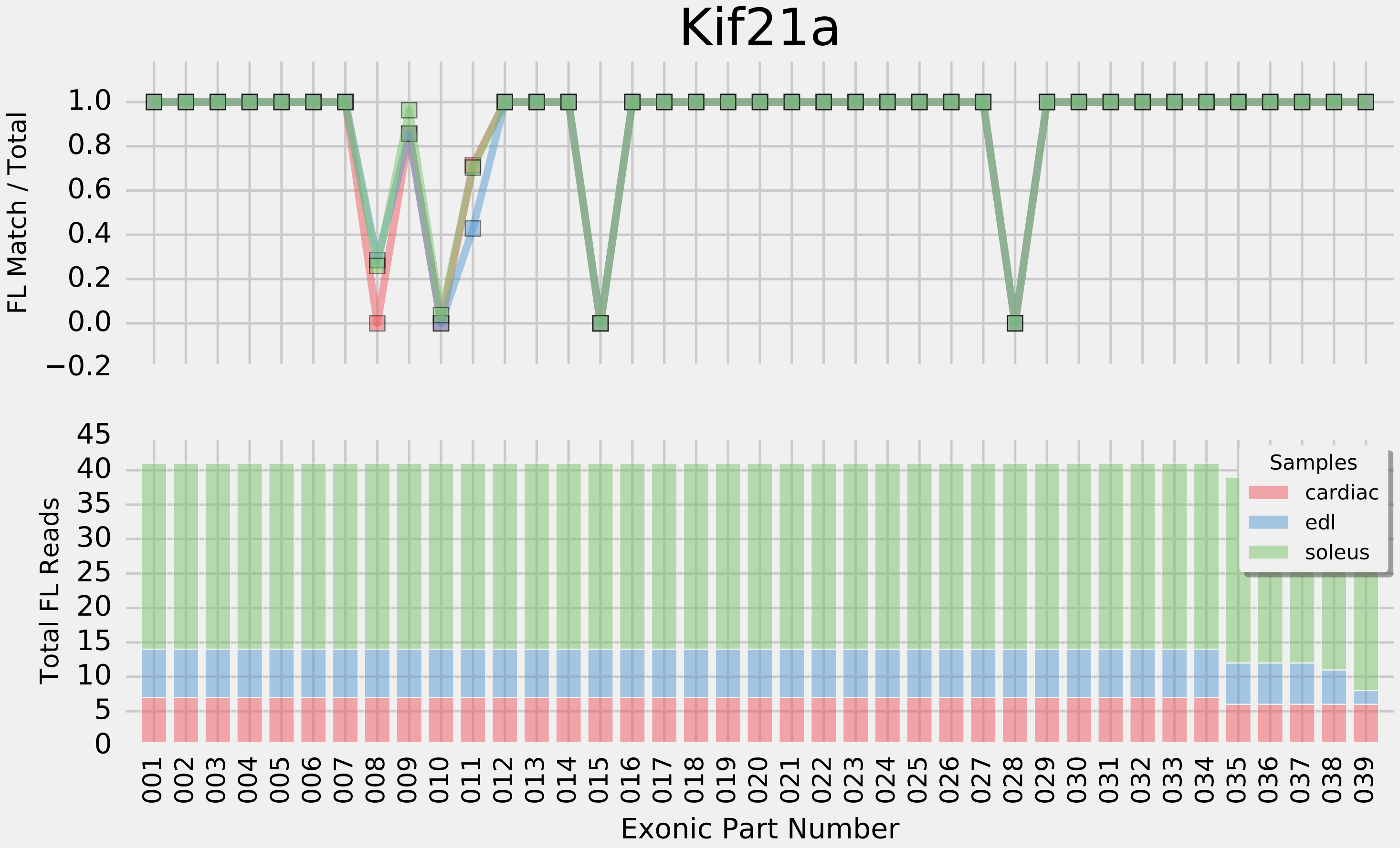

Kif21a
FL Match / Total
1.0
0.8
0.6
0.4
0.2
0.0
−0.2
Total FL Reads
45
40
35
30
25
20
15
10
5
0
Samples
cardiac
edl
soleus
001 002 003 004 005 006 007 008 009 010 011 012 013 014 015 016 017 018 019 020 021 022 023 024 025 026 027 028 029 030 031 032 033 034 035 036 037 038 039
Exonic Part Number

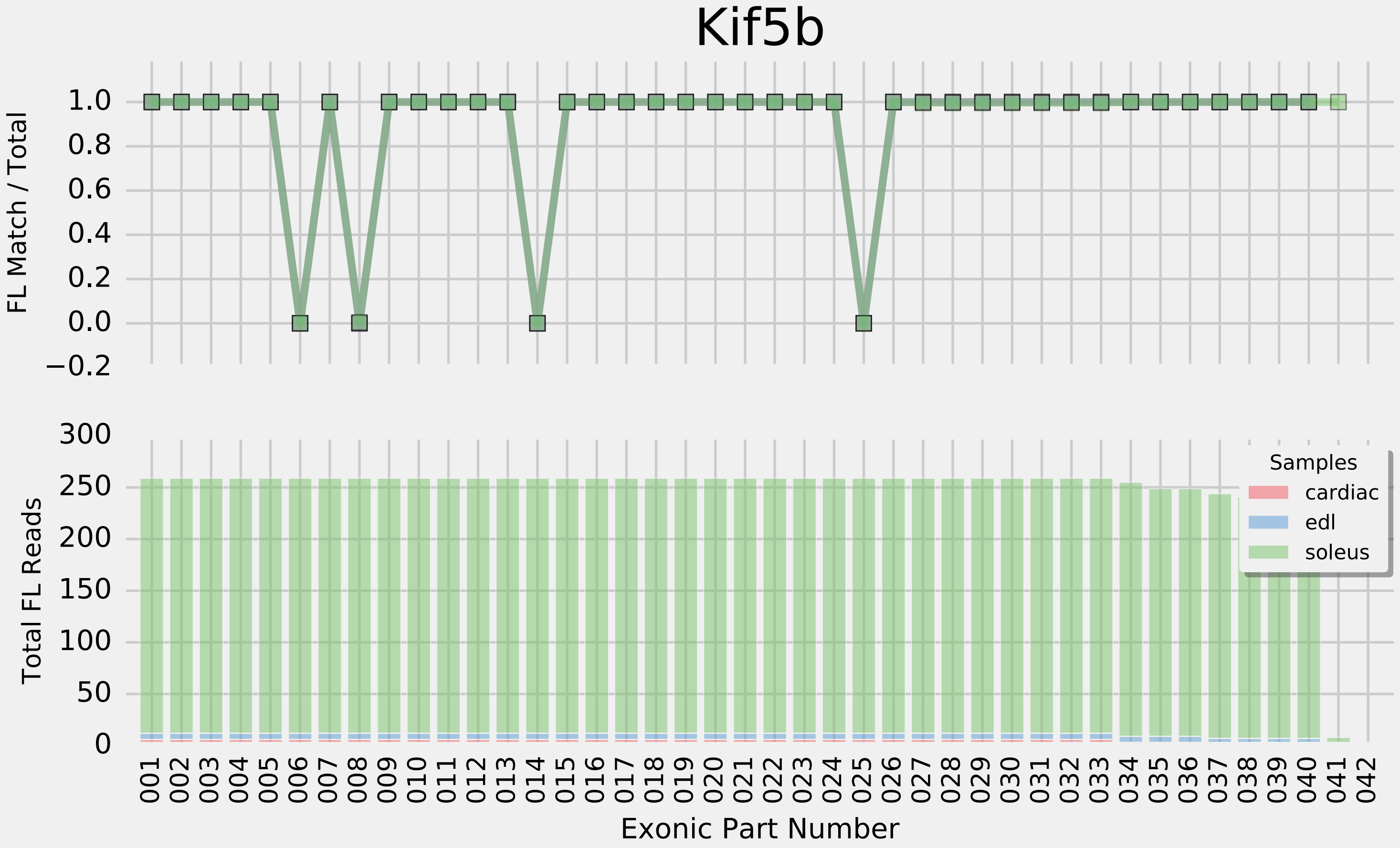

Kif5b
FL Match / Total
1.0
0.8
0.6
0.4
0.2
0.0
−0.2
Total FL Reads
300
250
200
150
100
50
0
Samples
cardiac
edl
soleus
001 002 003 004 005 006 007 008 009 010 011 012 013 014 015 016 017 018 019 020 021 022 023 024 025 026 027 028 029 030 031 032 033 034 035 036 037 038 039 040 041 042
Exonic Part Number

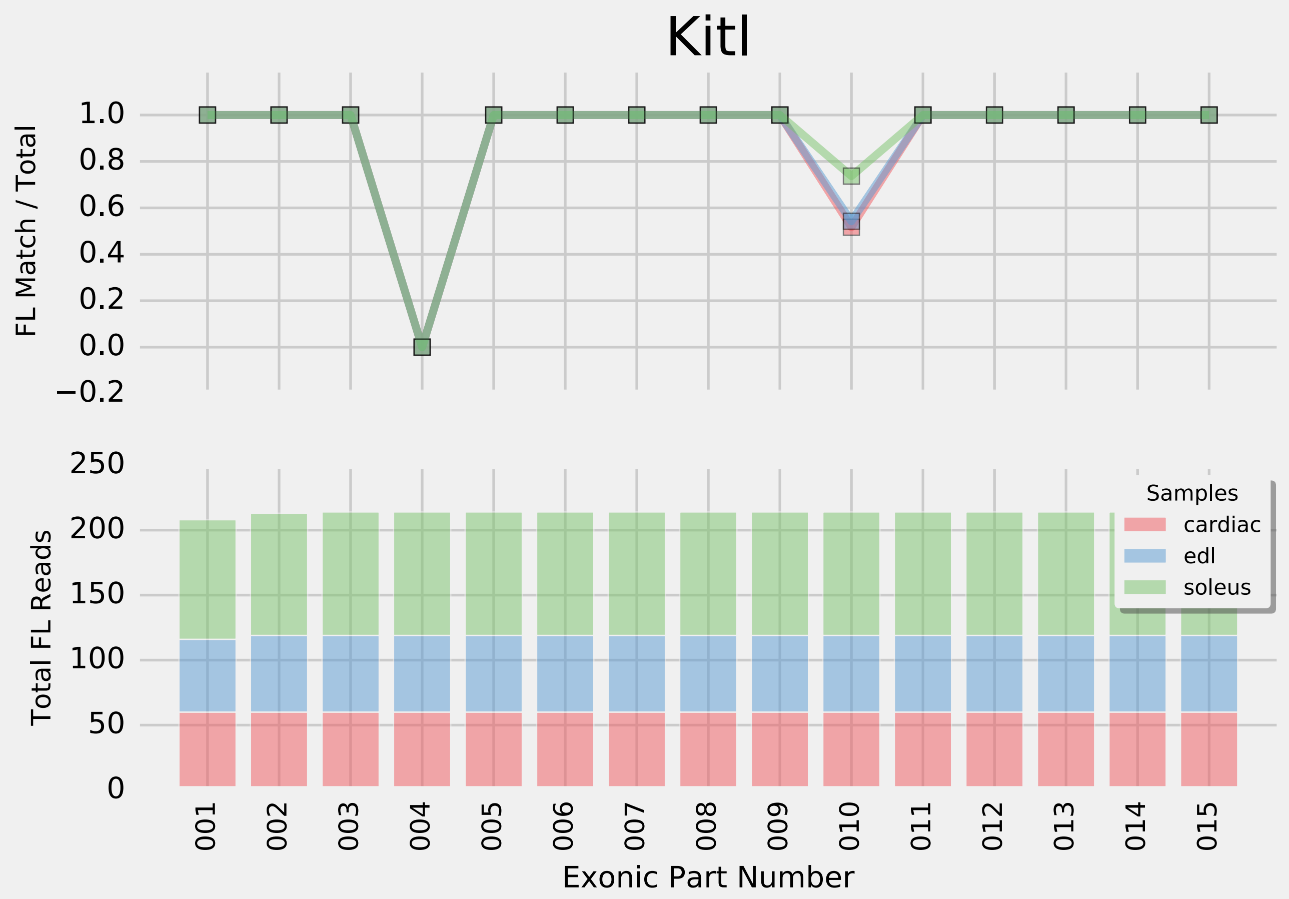

Kitl
FL Match / Total
1.0
0.8
0.6
0.4
0.2
0.0
−0.2
Total FL Reads
250
200
150
100
50
0
Samples
cardiac
edl
soleus
001
002
003
004
005
006
007
008
009
010
011
012
013
014
015
Exonic Part Number

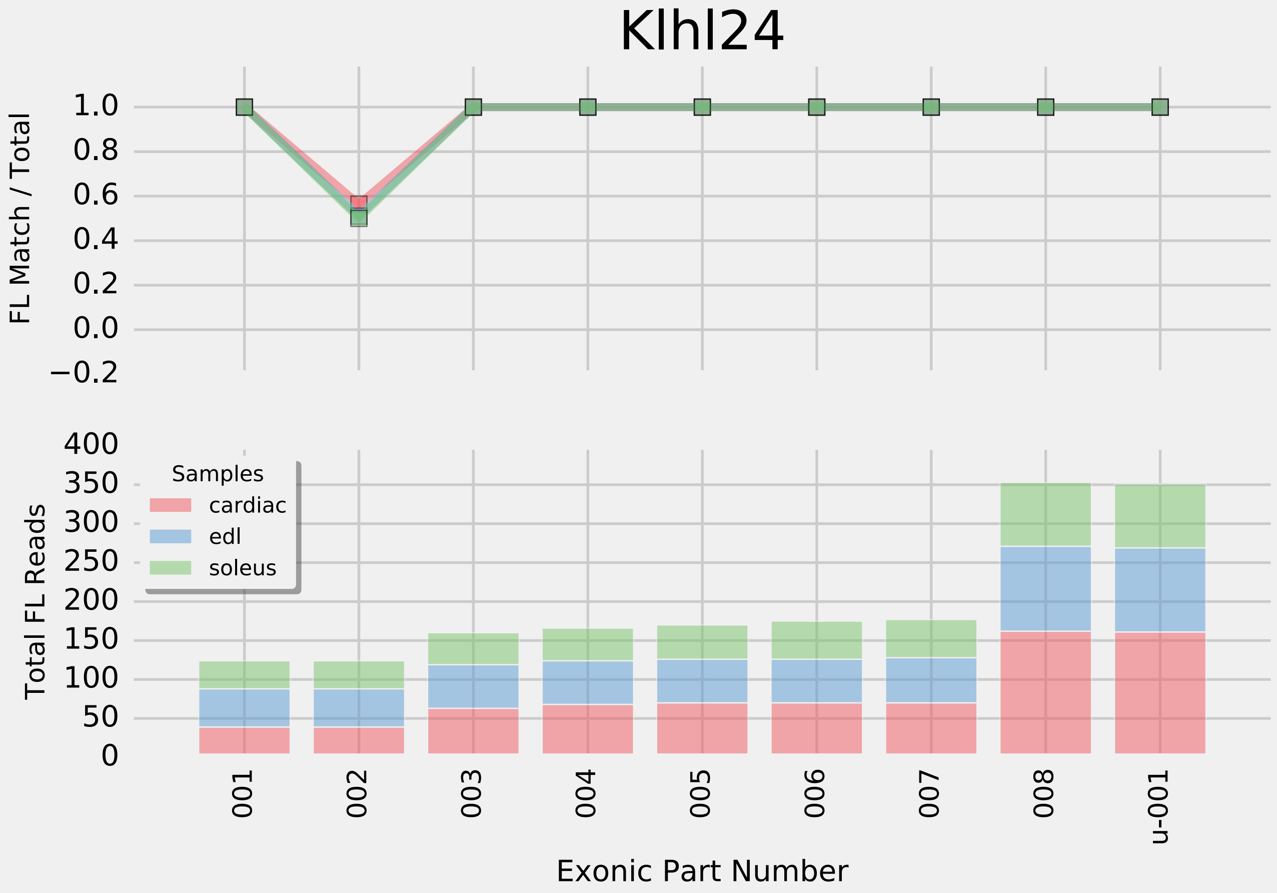

Klhl24
FL Match / Total
1.0
0.8
0.6
0.4
0.2
0.0
−0.2
Total FL Reads
400
350
300
250
200
150
100
50
0
Samples
cardiac
edl
soleus
001
002
003
004
005
006
007
008
u-001
Exonic Part Number

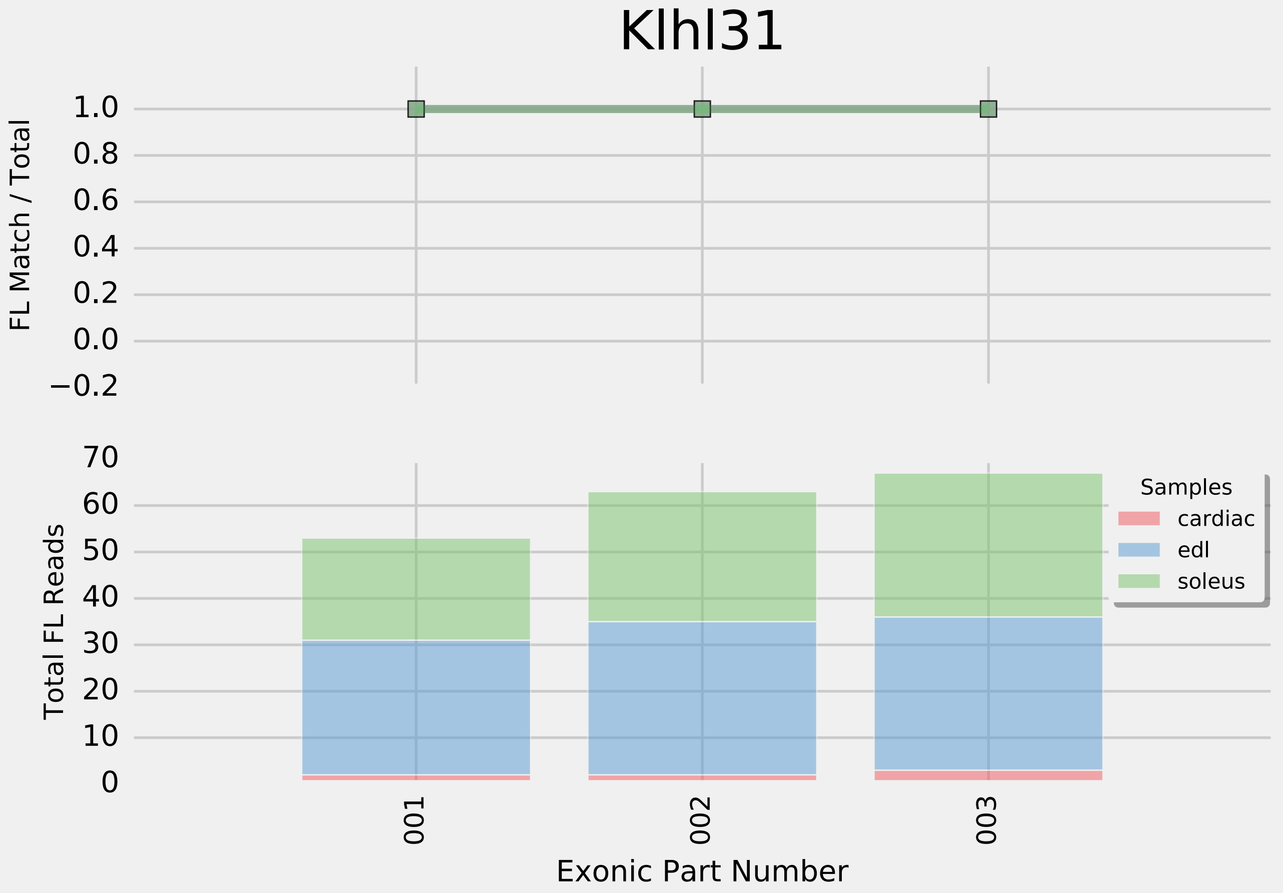

Klhl31
FL Match / Total
1.0
0.8
0.6
0.4
0.2
0.0
−0.2
Total FL Reads
70
60
50
40
30
20
10
0
Samples
cardiac
edl
soleus
001
002
003
Exonic Part Number

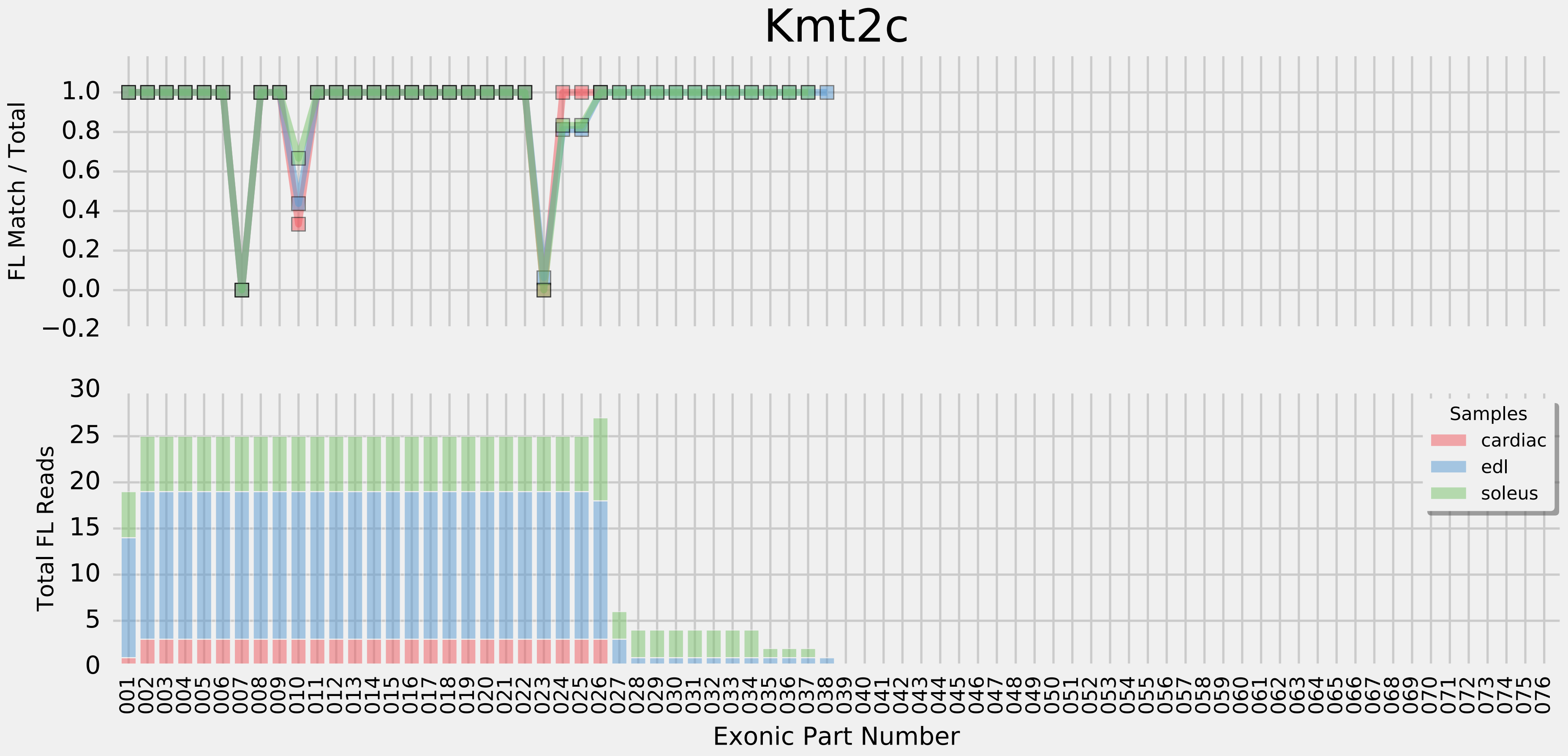

Kmt2c
FL Match / Total
1.0
0.8
0.6
0.4
0.2
0.0
−0.2
Total FL Reads
30
25
20
15
10
5
0
Samples
cardiac
edl
soleus
Exonic Part Number
001 002 003 004 005 006 007 008 009 010 011 012 013 014 015 016 017 018 019 020 021 022 023 024 025 026 027 028 029 030 031 032 033 034 035 036 037 038 039 040 041 042 043 044 045 046 047 048 049 050 051 052 053 054 055 056 057 058 059 060 061 062 063 064 065 066 067 068 069 070 071 072 073 074 075 076

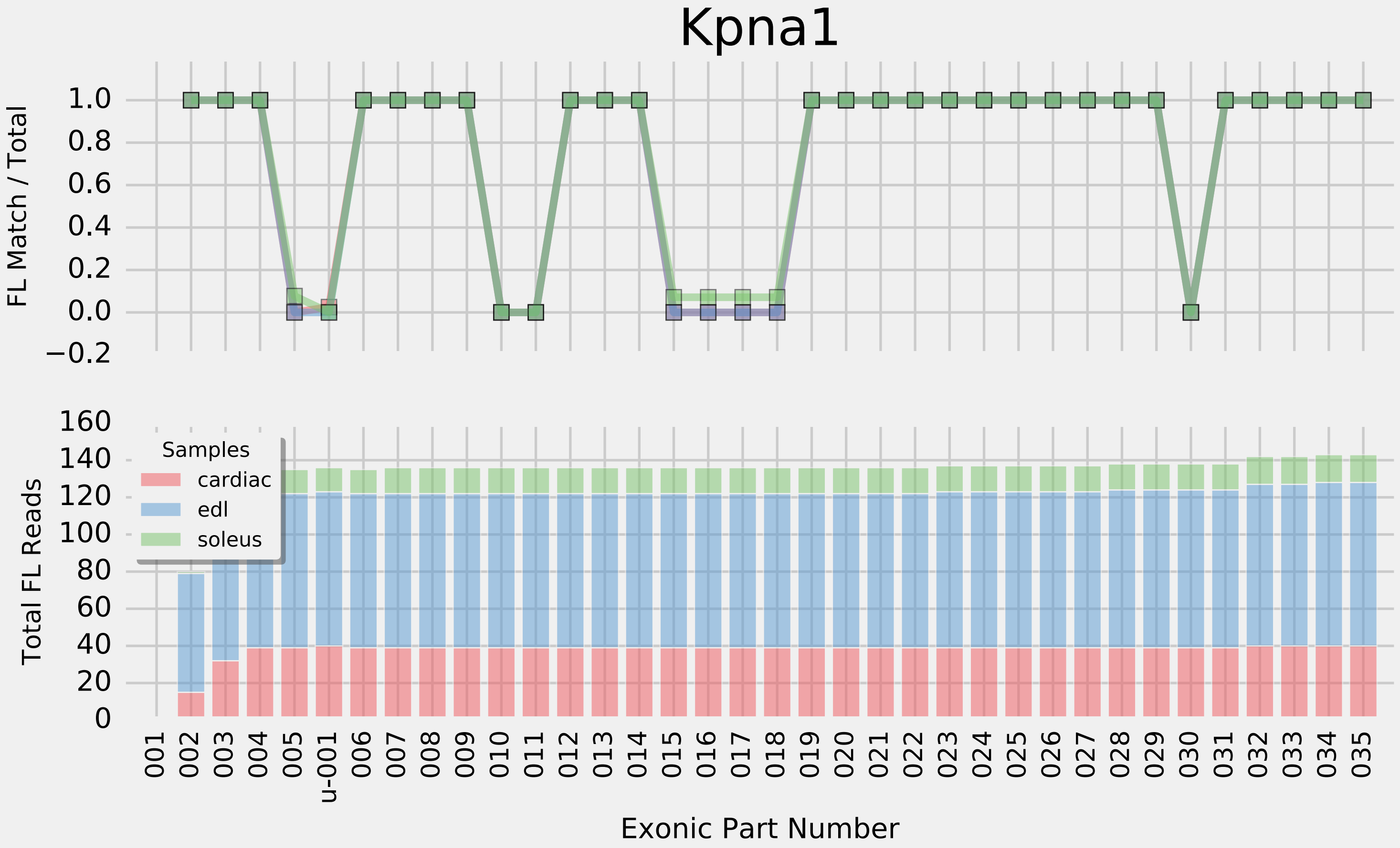

Kpna1
FL Match / Total
1.0
0.8
0.6
0.4
0.2
0.0
−0.2
Total FL Reads
160
140
120
100
80
60
40
20
0
Samples
cardiac
edl
soleus
001 002 003 004 005 u-001 006 007 008 009 010 011 012 013 014 015 016 017 018 019 020 021 022 023 024 025 026 027 028 029 030 031 032 033 034 035
Exonic Part Number

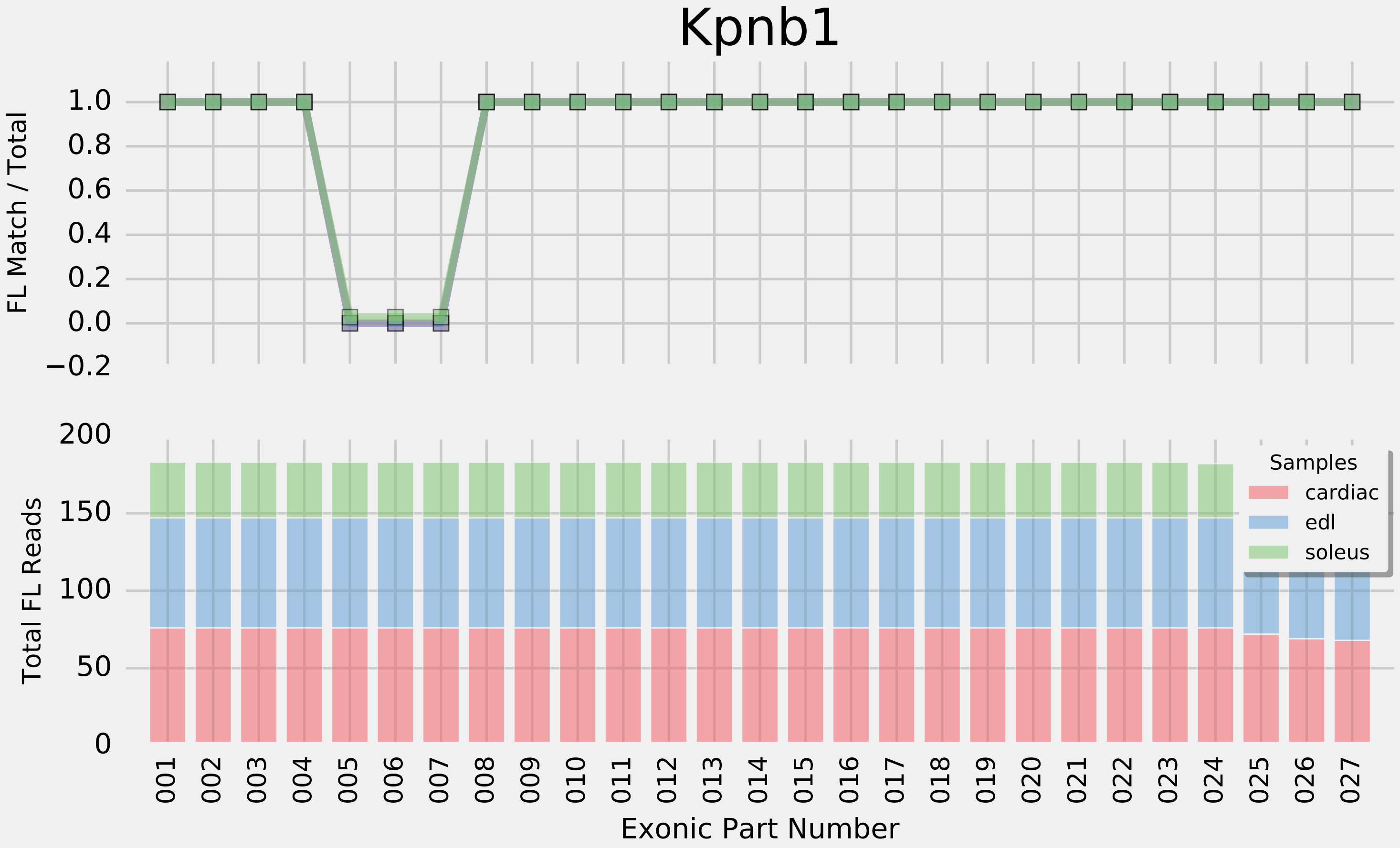

Kpnb1
FL Match / Total
1.0
0.8
0.6
0.4
0.2
0.0
−0.2
200
150
100
50
0
Total FL Reads
Samples
cardiac
edl
soleus
001
002
003
004
005
006
007
008
009
010
011
012
013
014
015
016
017
018
019
020
021
022
023
024
025
026
027
Exonic Part Number

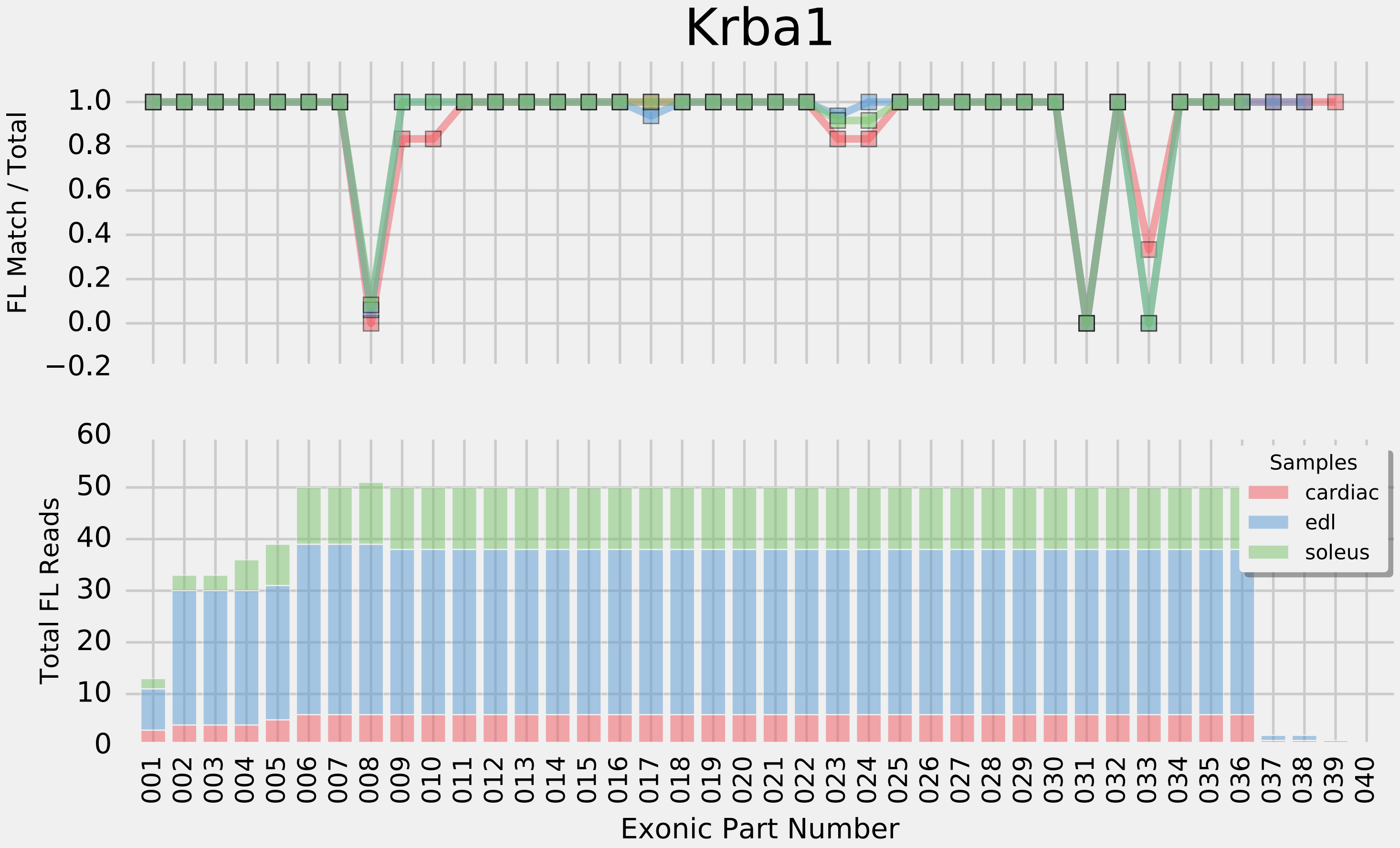

Krba1
FL Match / Total
1.0
0.8
0.6
0.4
0.2
0.0
−0.2
Total FL Reads
60
50
40
30
20
10
0
Samples
cardiac
edl
soleus
001 002 003 004 005 006 007 008 009 010 011 012 013 014 015 016 017 018 019 020 021 022 023 024 025 026 027 028 029 030 031 032 033 034 035 036 037 038 039 040
Exonic Part Number

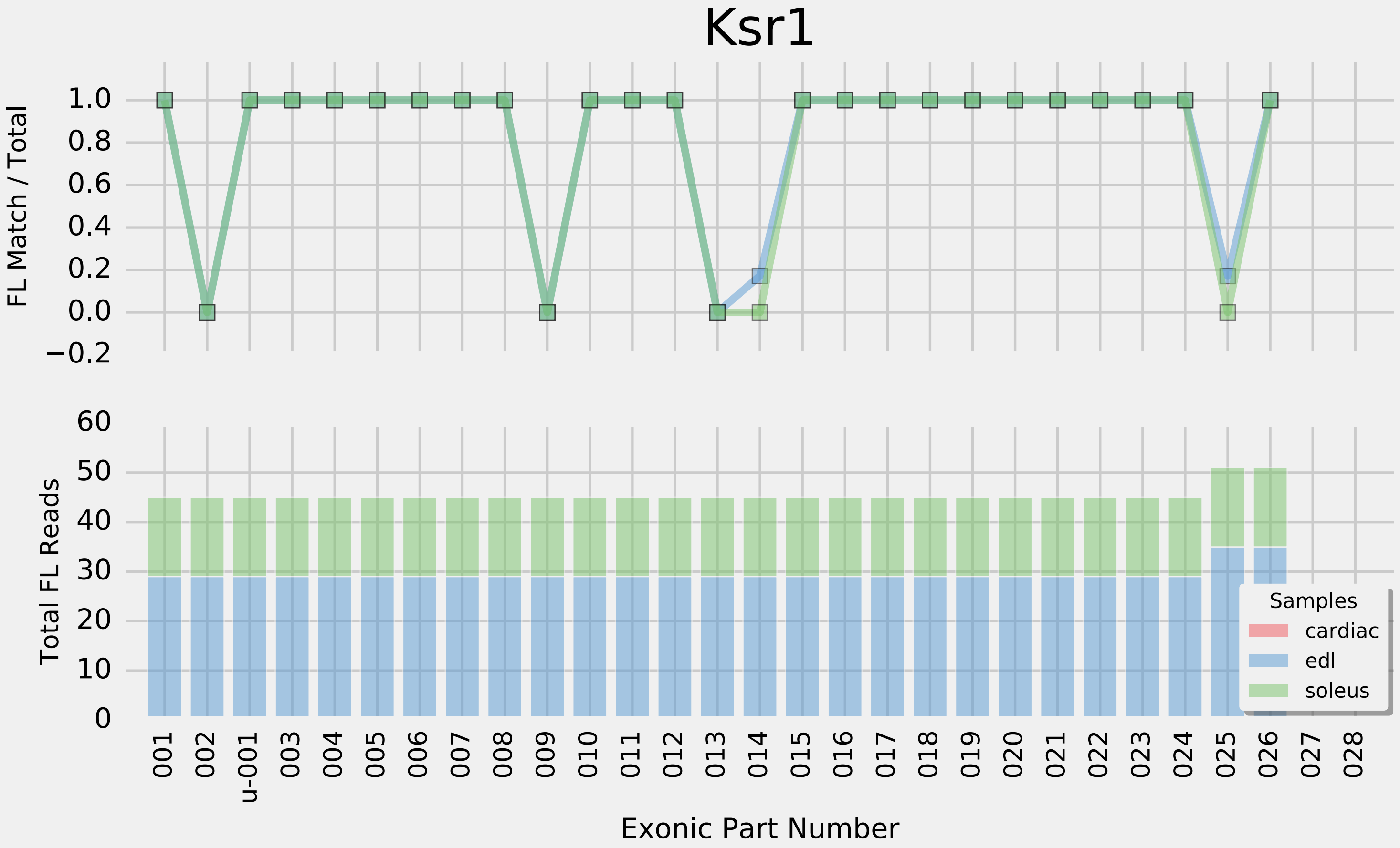

Ksr1
FL Match / Total
1.0
0.8
0.6
0.4
0.2
0.0
−0.2
Total FL Reads
60
50
40
30
20
10
0
Samples
cardiac
edl
soleus
001
002
u-001
003
004
005
006
007
008
009
010
011
012
013
014
015
016
017
018
019
020
021
022
023
024
025
026
027
028
Exonic Part Number

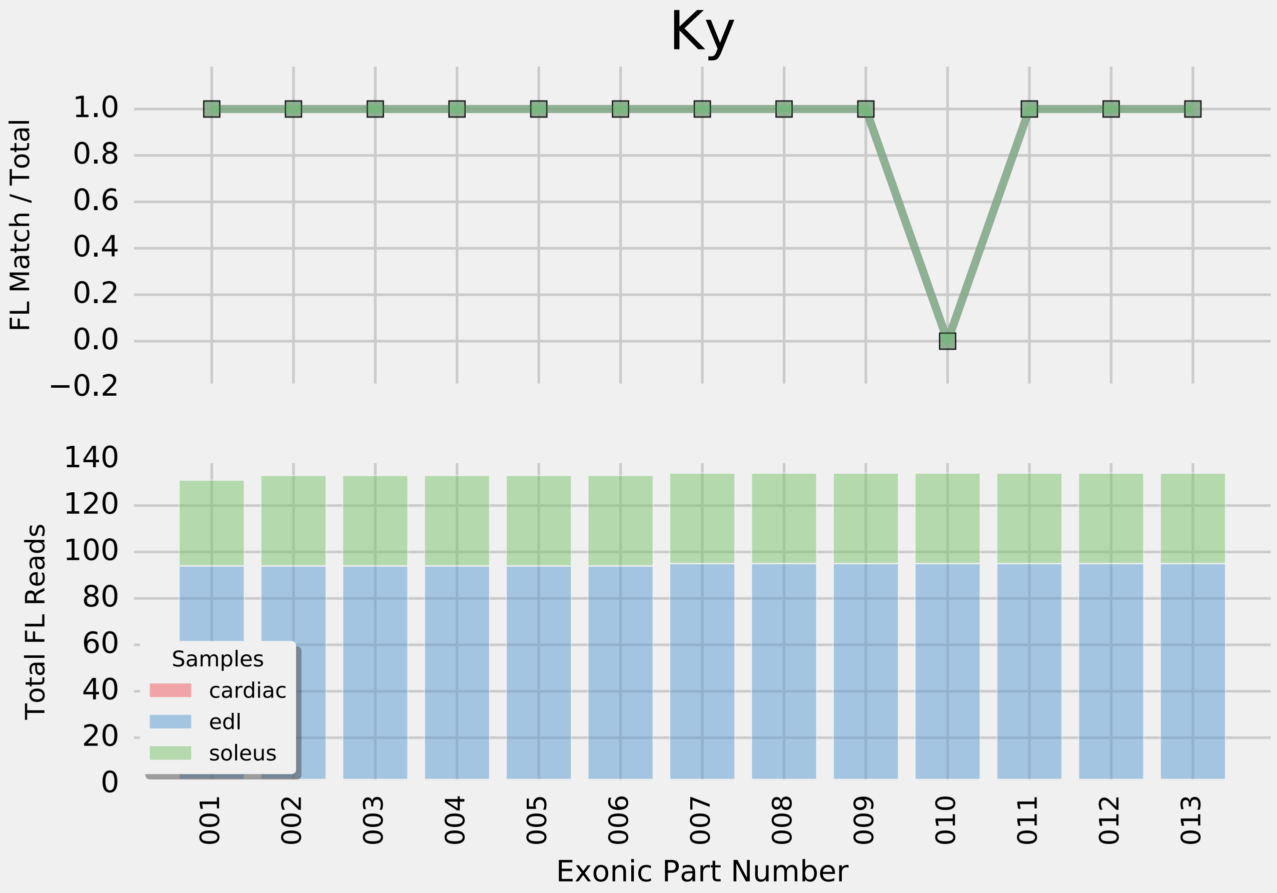

Ky
FL Match / Total
1.0
0.8
0.6
0.4
0.2
0.0
−0.2
Total FL Reads
140
120
100
80
60
40
20
0
Samples
cardiac
edl
soleus
001
002
003
004
005
006
007
008
009
010
011
012
013
Exonic Part Number

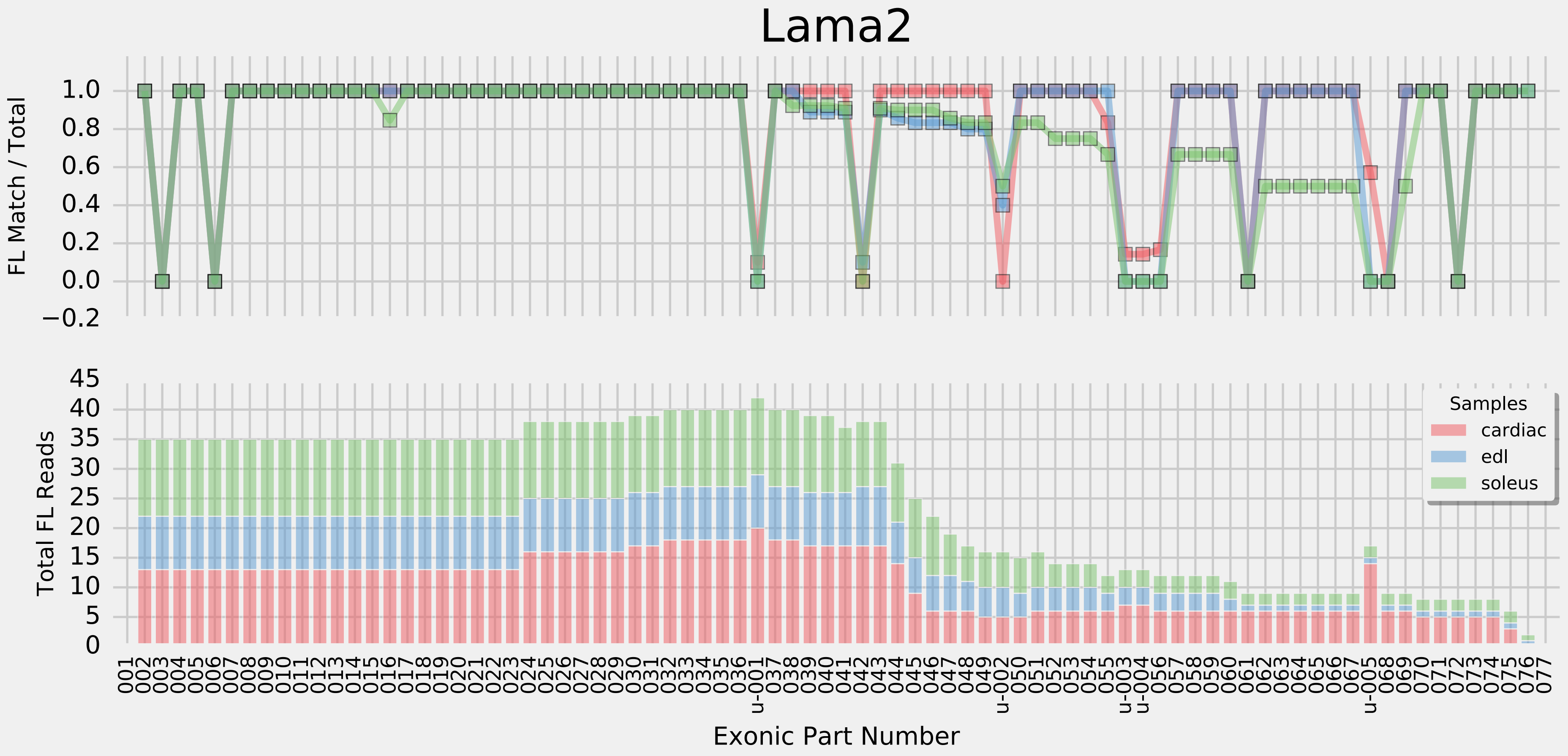

Lama2
FL Match / Total
Total FL Reads
Exonic Part Number
Samples
cardiac
edl
soleus

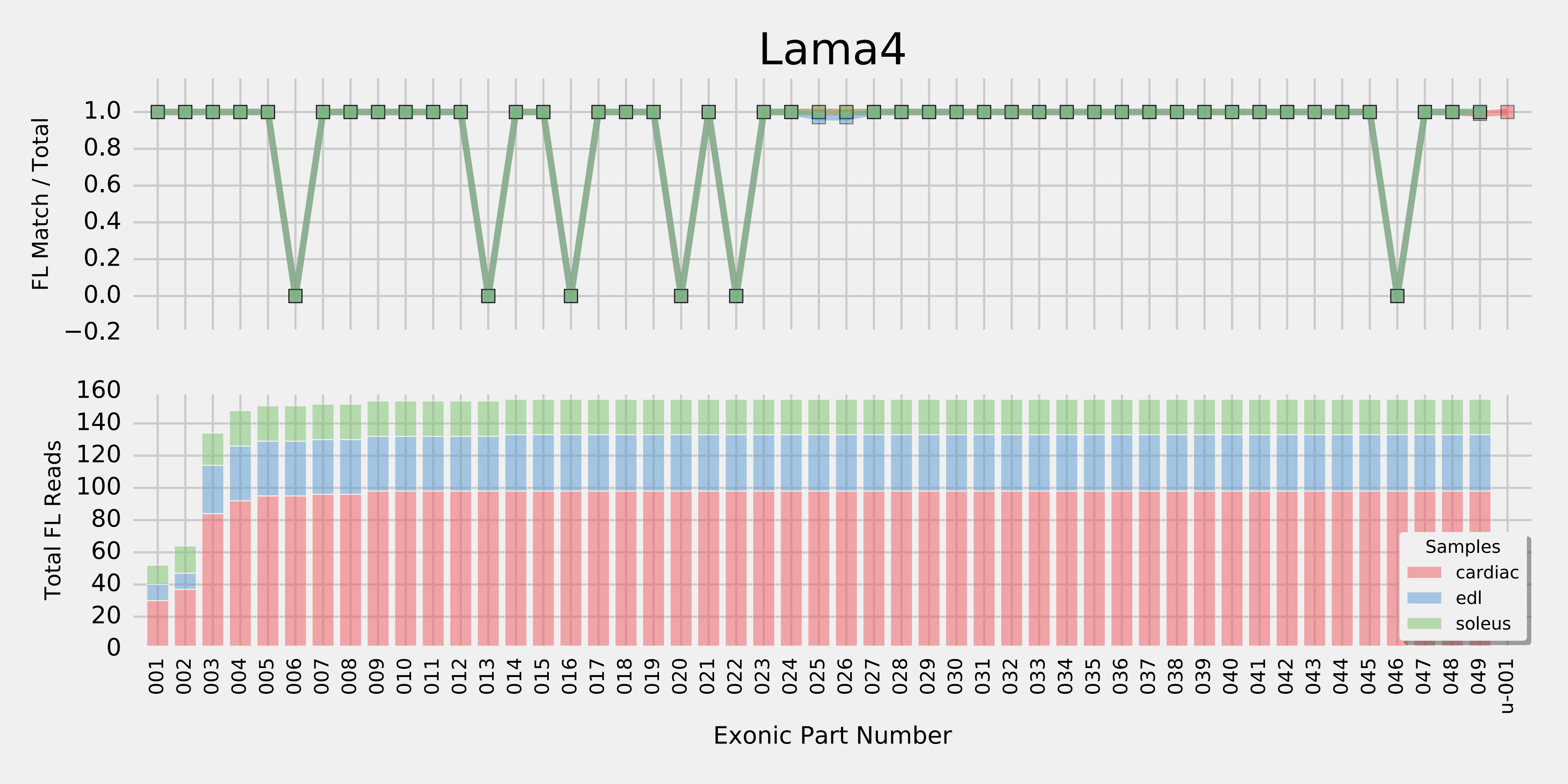

Lama4
FL Match / Total
Total FL Reads
Exonic Part Number
Samples
cardiac
edl
soleus

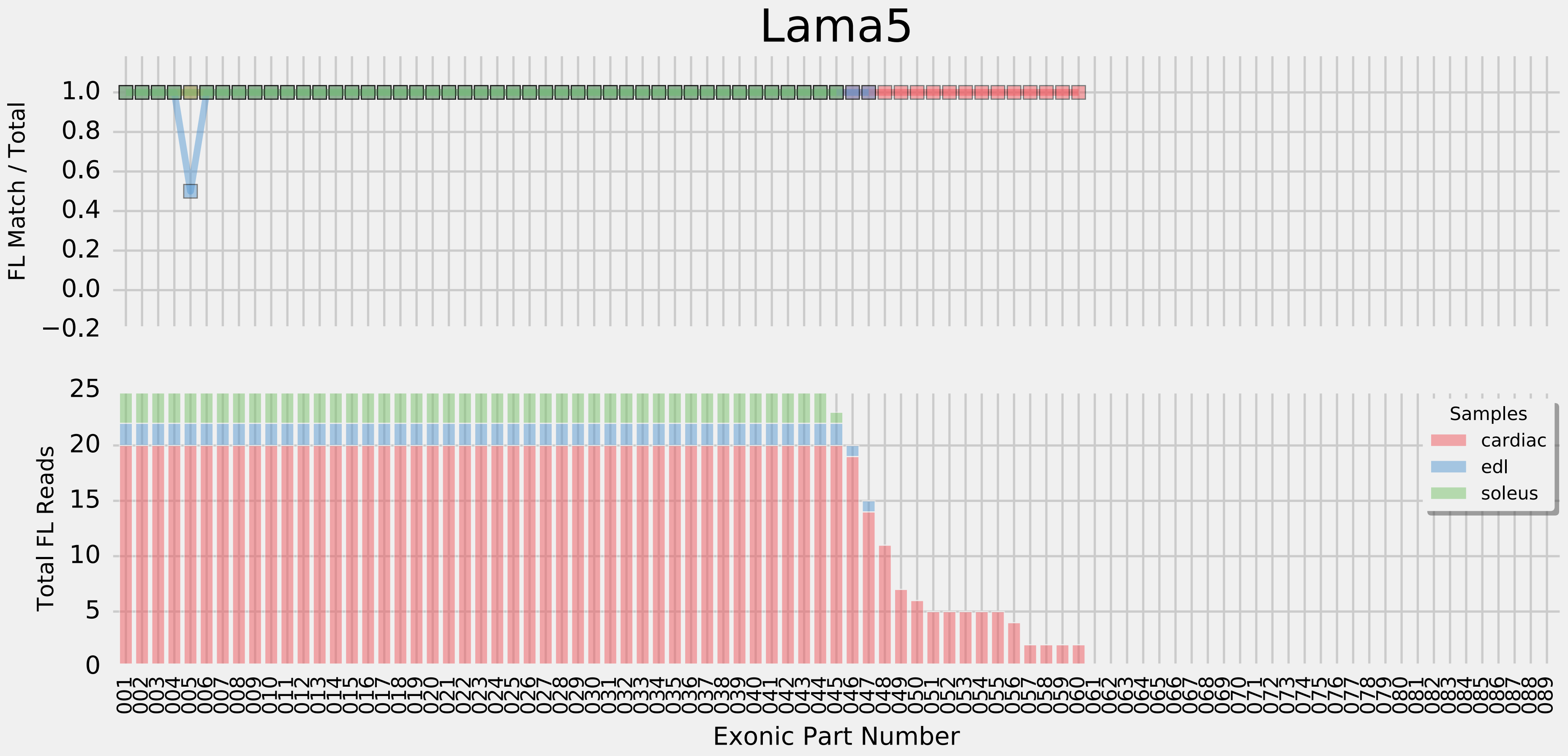

Lama5
FL Match / Total
1.0
0.8
0.6
0.4
0.2
0.0
−0.2
Total FL Reads
25
20
15
10
5
0
Samples
cardiac
edl
soleus
Exonic Part Number

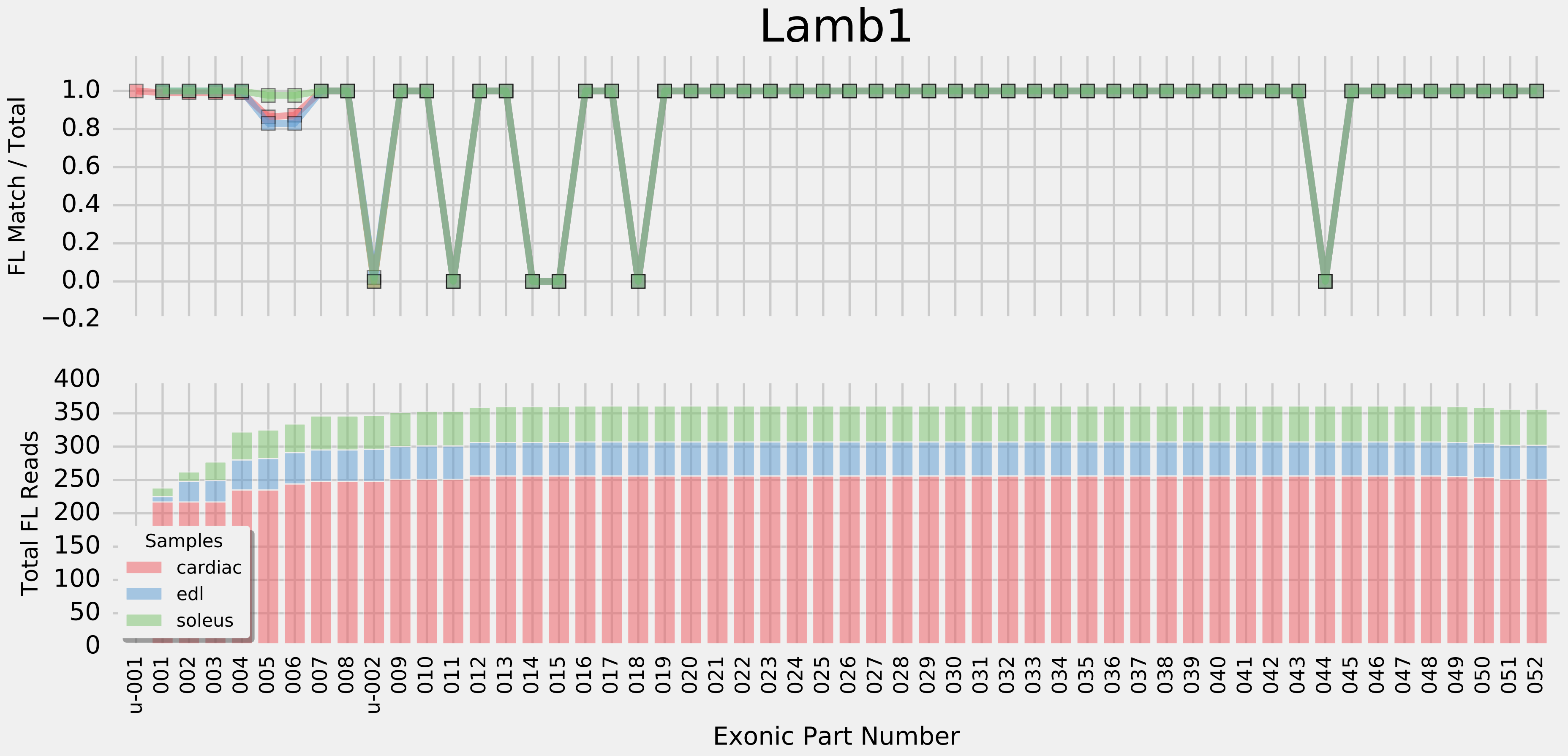

Lamb1
FL Match / Total
Total FL Reads
Samples
cardiac
edl
soleus
Exonic Part Number

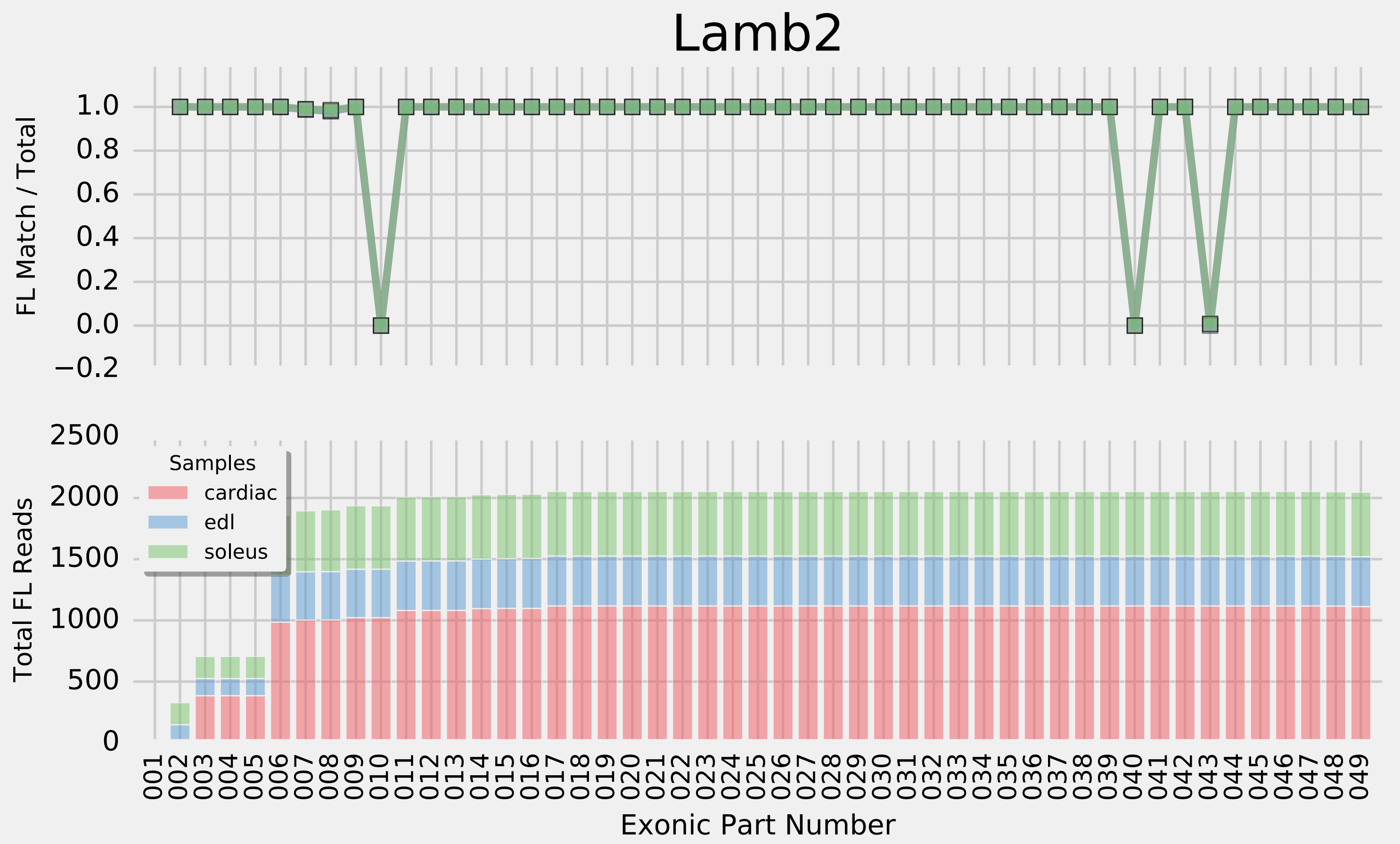

Lamb2
FL Match / Total
1.0
0.8
0.6
0.4
0.2
0.0
−0.2
Total FL Reads
2500
2000
1500
1000
500
0
Samples
cardiac
edl
soleus
001 002 003 004 005 006 007 008 009 010 011 012 013 014 015 016 017 018 019 020 021 022 023 024 025 026 027 028 029 030 031 032 033 034 035 036 037 038 039 040 041 042 043 044 045 046 047 048 049
Exonic Part Number

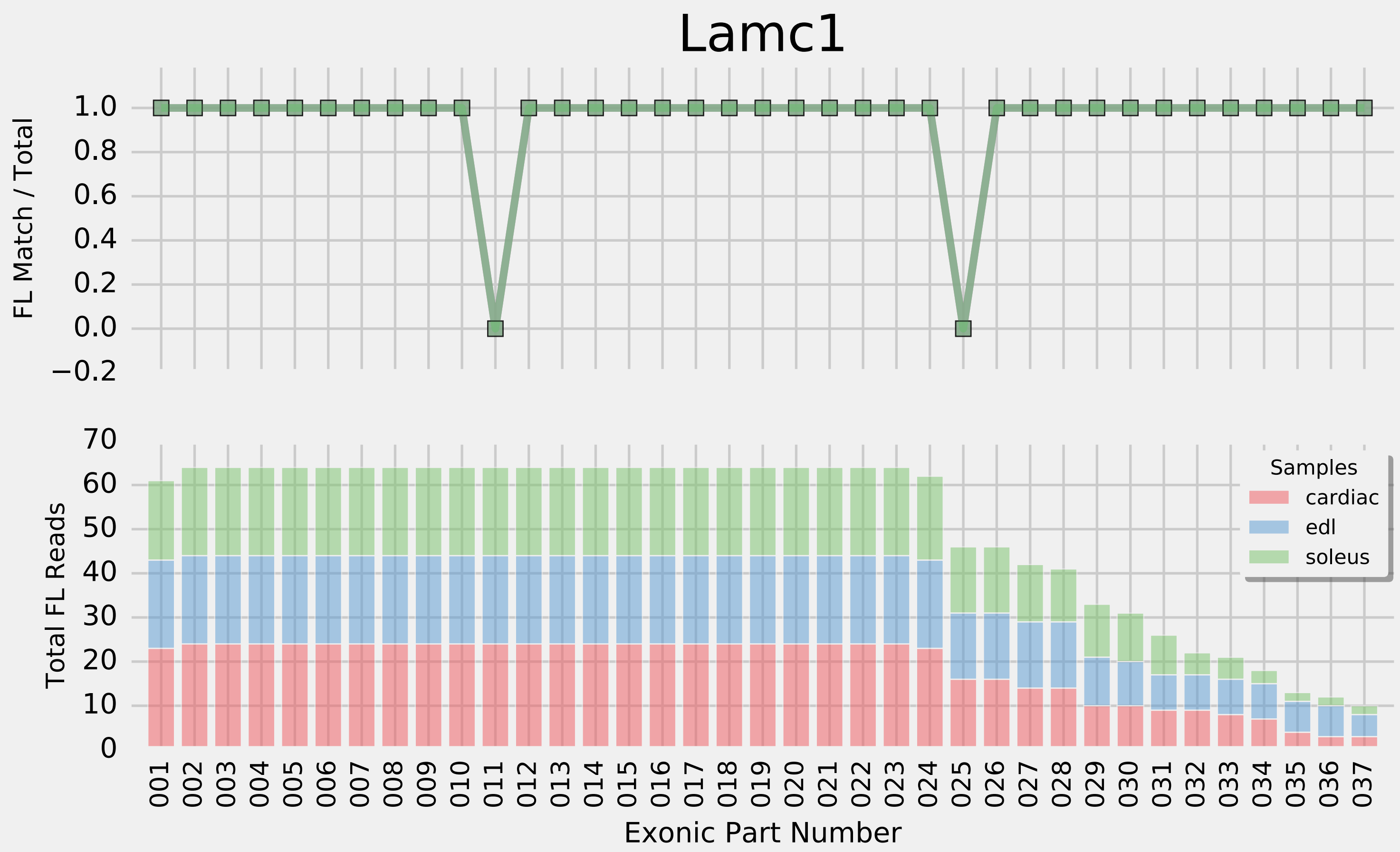

Lamc1
FL Match / Total
1.0
0.8
0.6
0.4
0.2
0.0
−0.2
Total FL Reads
70
60
50
40
30
20
10
0
Samples
cardiac
edl
soleus
001 002 003 004 005 006 007 008 009 010 011 012 013 014 015 016 017 018 019 020 021 022 023 024 025 026 027 028 029 030 031 032 033 034 035 036 037
Exonic Part Number

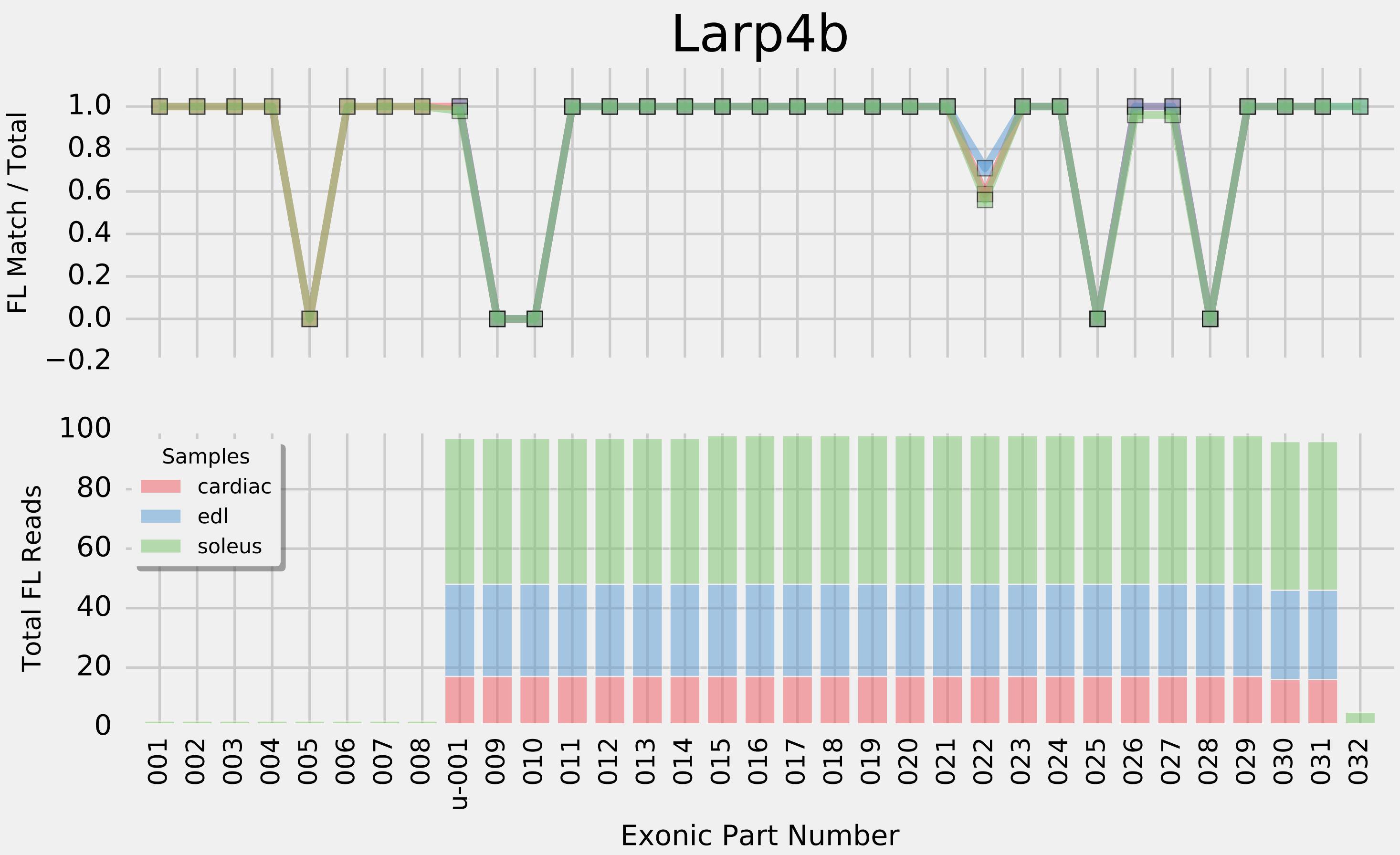

Larp4b
FL Match / Total
1.0
0.8
0.6
0.4
0.2
0.0
−0.2
100
80
60
40
20
0
Total FL Reads
Samples
cardiac
edl
soleus
001
002
003
004
005
006
007
008
u-001
009
010
011
012
013
014
015
016
017
018
019
020
021
022
023
024
025
026
027
028
029
030
031
032
Exonic Part Number

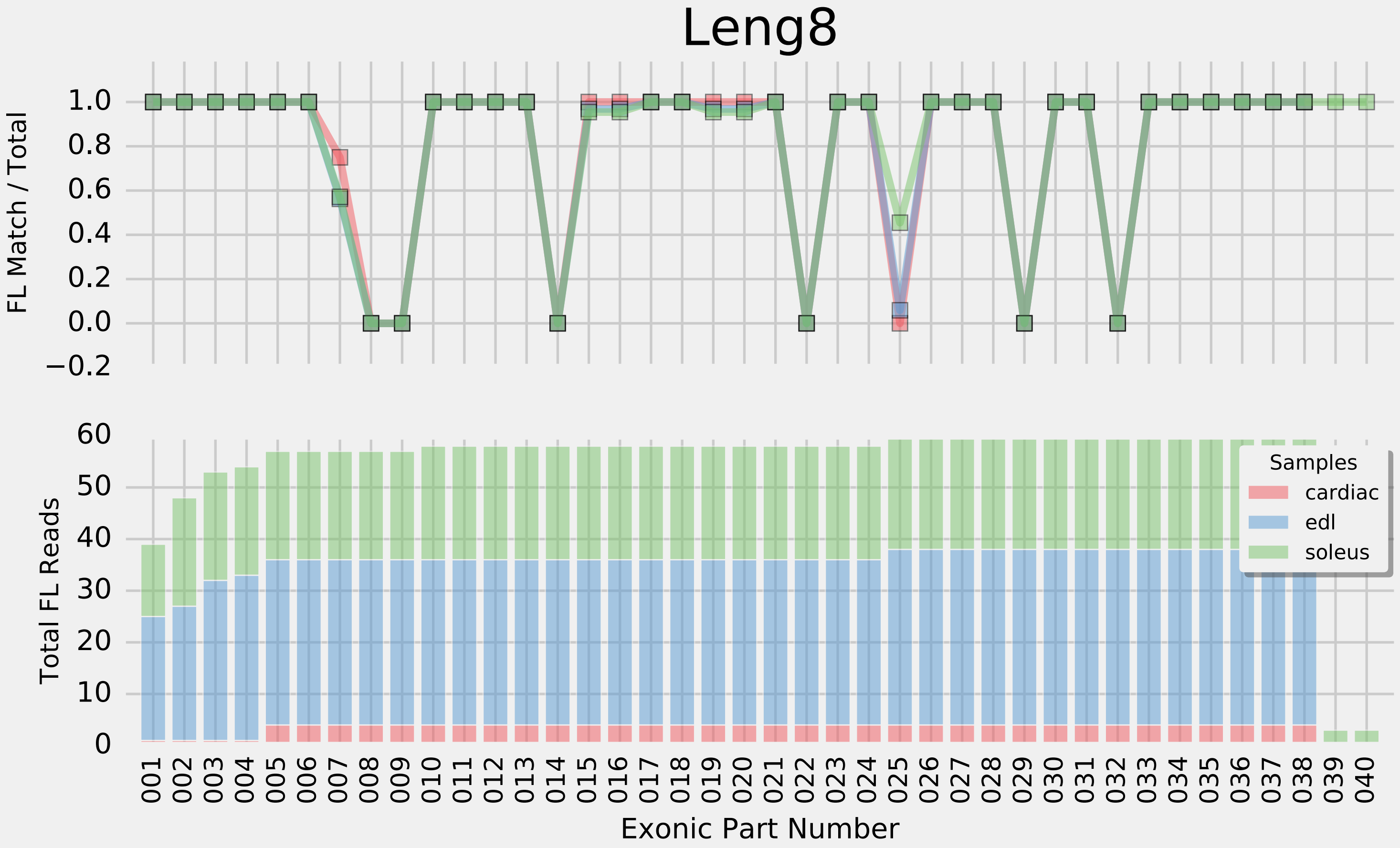
Leng8
FL Match / Total
1.0
0.8
0.6
0.4
0.2
0.0
−0.2
Total FL Reads
60
50
40
30
20
10
0
Samples
cardiac
edl
soleus
001 002 003 004 005 006 007 008 009 010 011 012 013 014 015 016 017 018 019 020 021 022 023 024 025 026 027 028 029 030 031 032 033 034 035 036 037 038 039 040
Exonic Part Number

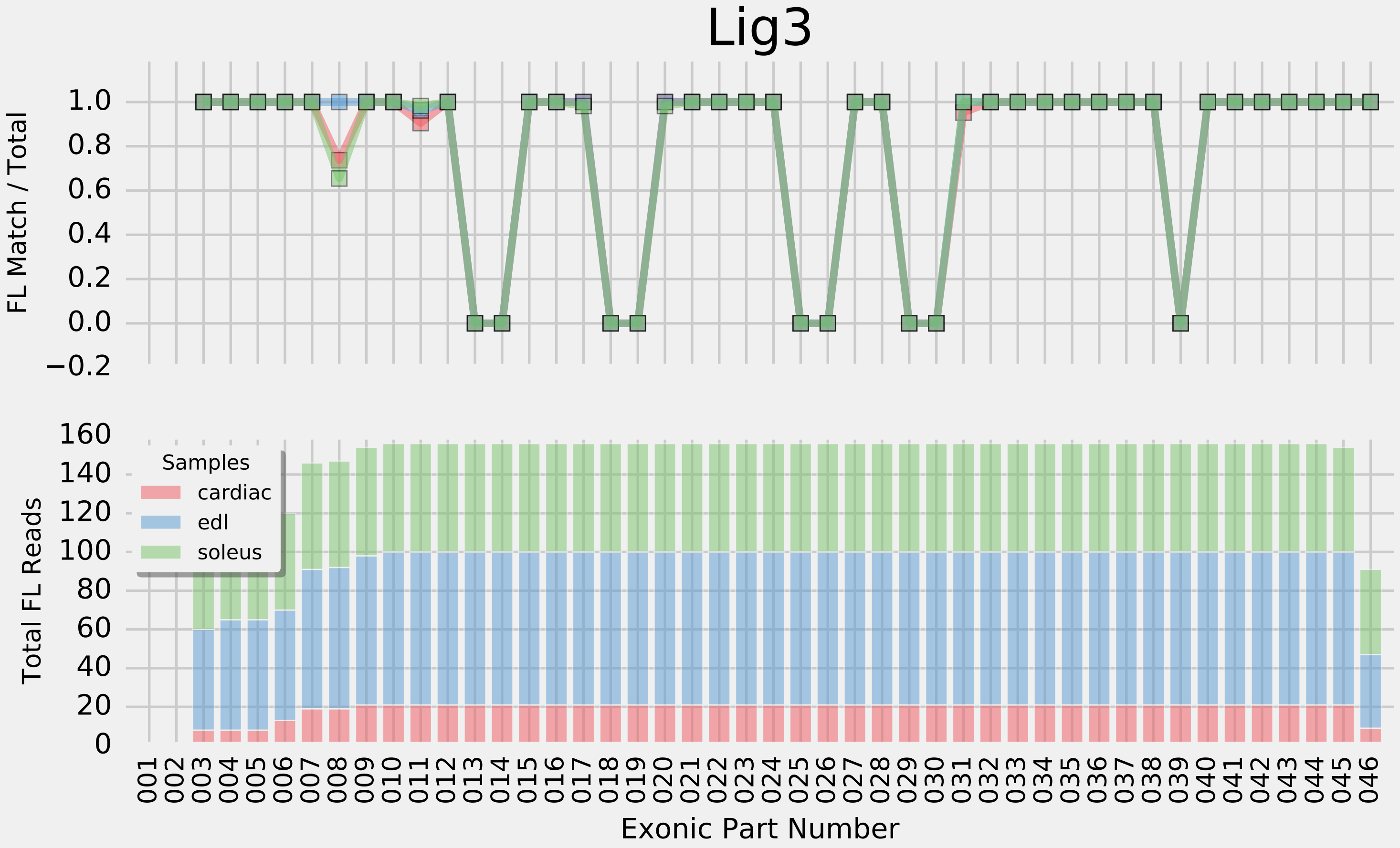

Lig3
FL Match / Total
1.0
0.8
0.6
0.4
0.2
0.0
−0.2
Total FL Reads
160
140
120
100
80
60
40
20
0
Samples
cardiac
edl
soleus
001 002 003 004 005 006 007 008 009 010 011 012 013 014 015 016 017 018 019 020 021 022 023 024 025 026 027 028 029 030 031 032 033 034 035 036 037 038 039 040 041 042 043 044 045 046
Exonic Part Number

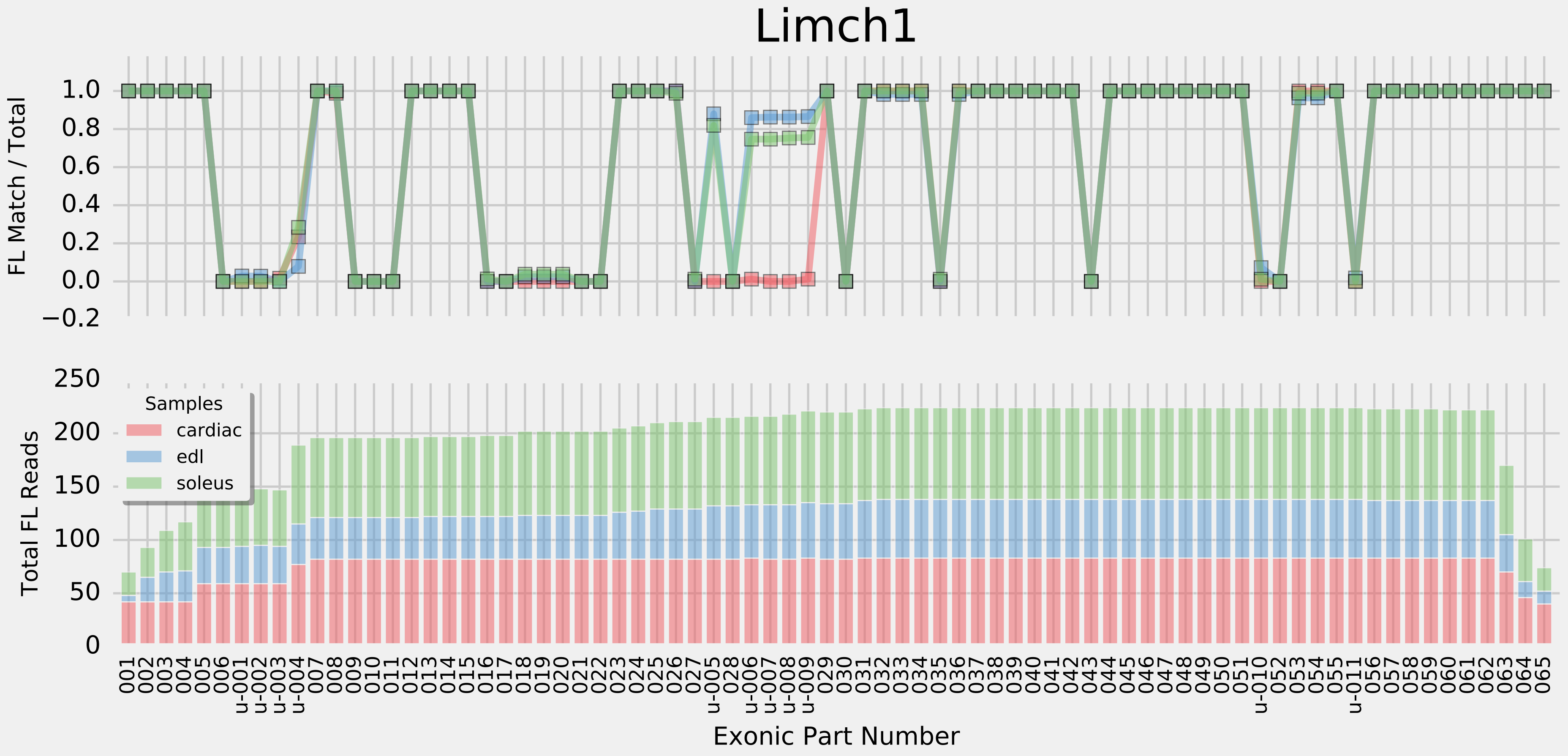

Limch1
FL Match / Total
1.0
0.8
0.6
0.4
0.2
0.0
−0.2
250
200
150
100
50
0
Total FL Reads
Samples
cardiac
edl
soleus
001 002 003 004 005 006 u-001 u-002 u-003 u-004 007 008 009 010 011 012 013 014 015 016 017 018 019 020 021 022 023 024 025 026 027 u-005 028 u-006 u-007 u-008 u-009 029 030 031 032 033 034 035 036 037 038 039 040 041 042 043 044 045 046 047 048 049 050 051 u-010 052 053 054 055 u-011 056 057 058 059 060 061 062 063 064 065
Exonic Part Number

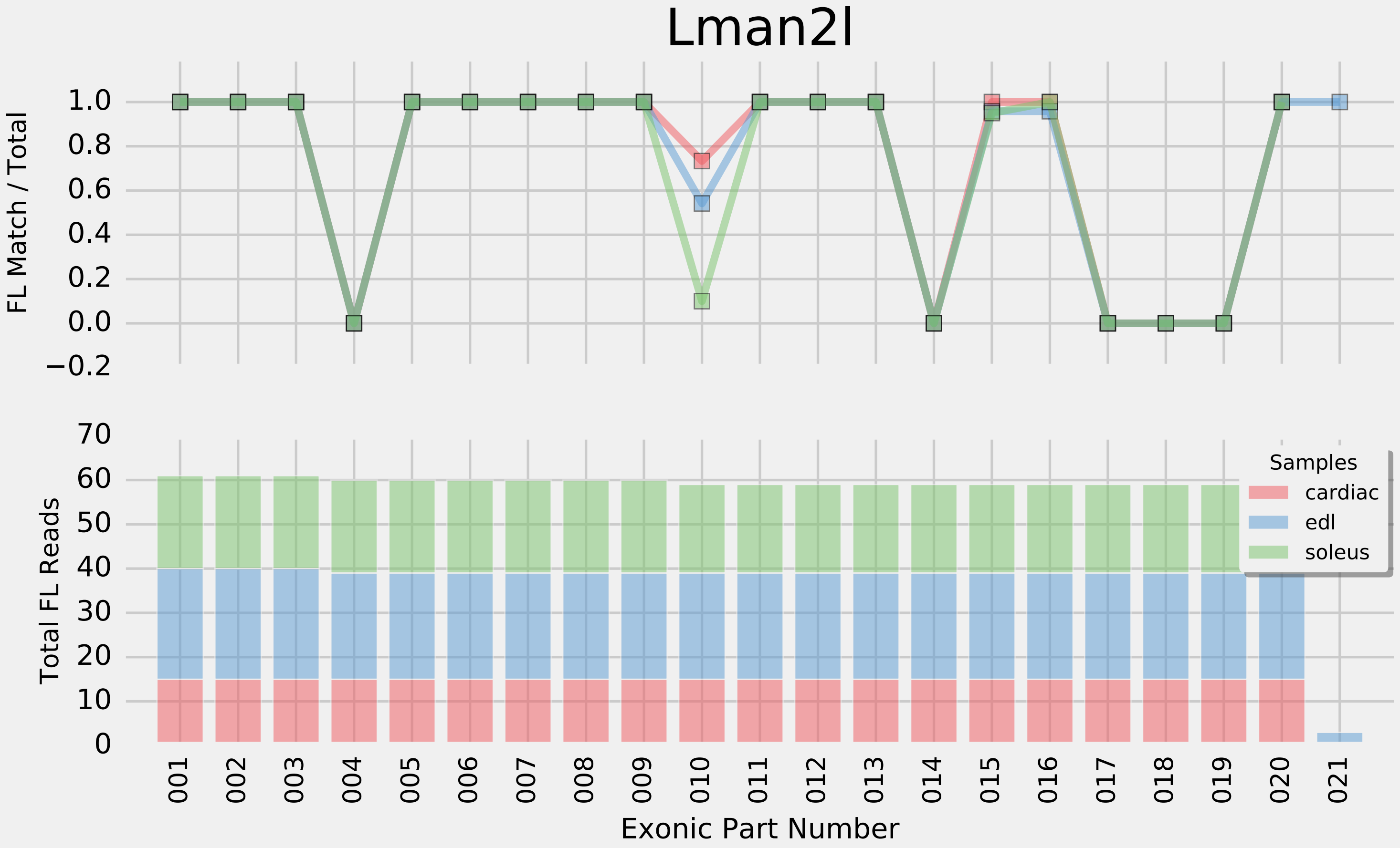

Lman2l
FL Match / Total
1.0
0.8
0.6
0.4
0.2
0.0
−0.2
Total FL Reads
70
60
50
40
30
20
10
0
Samples
cardiac
edl
soleus
001
002
003
004
005
006
007
008
009
010
011
012
013
014
015
016
017
018
019
020
021
Exonic Part Number

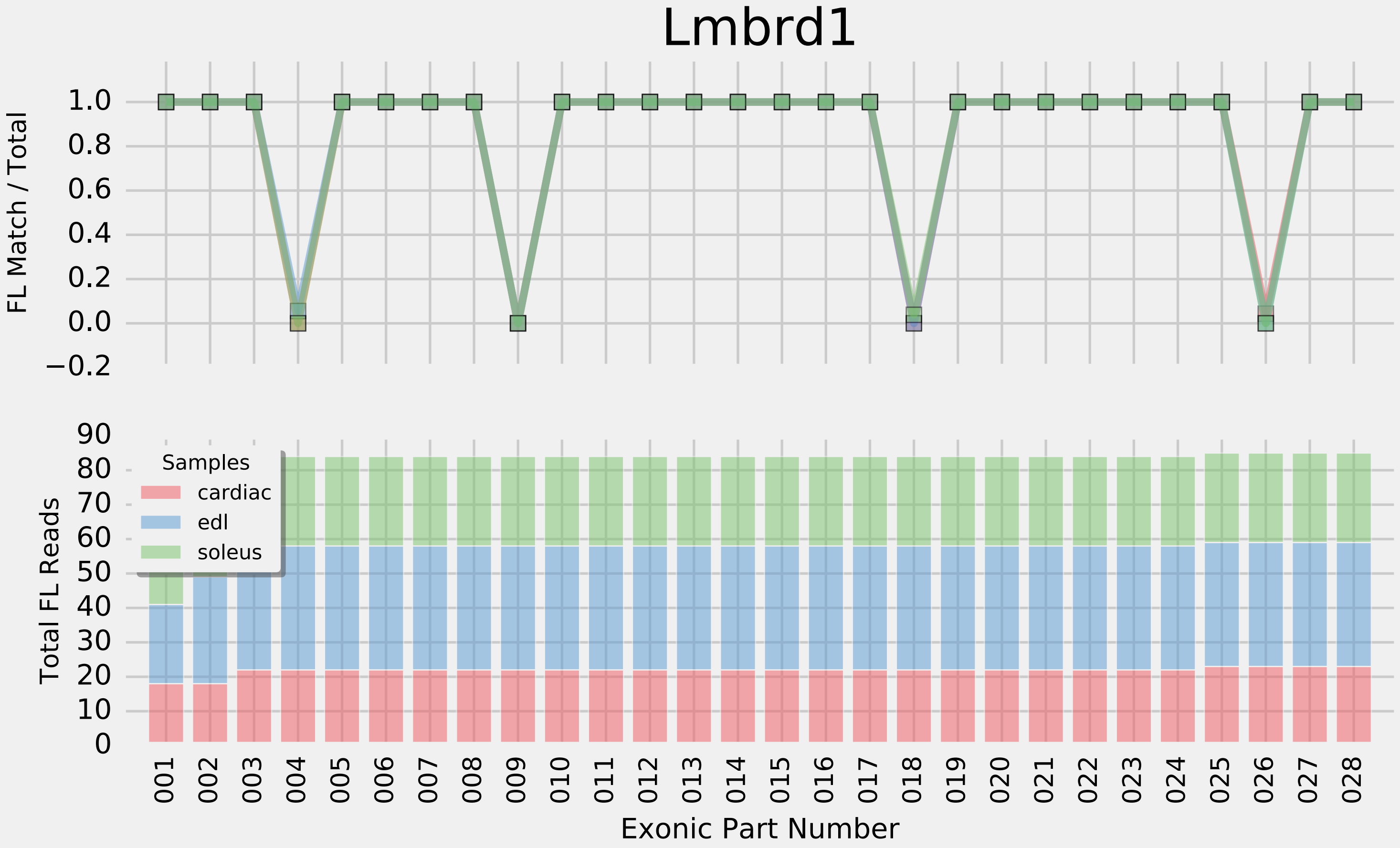

Lmbrd1
FL Match / Total
1.0
0.8
0.6
0.4
0.2
0.0
−0.2
Total FL Reads
90
80
70
60
50
40
30
20
10
0
Samples
cardiac
edl
soleus
001 002 003 004 005 006 007 008 009 010 011 012 013 014 015 016 017 018 019 020 021 022 023 024 025 026 027 028
Exonic Part Number

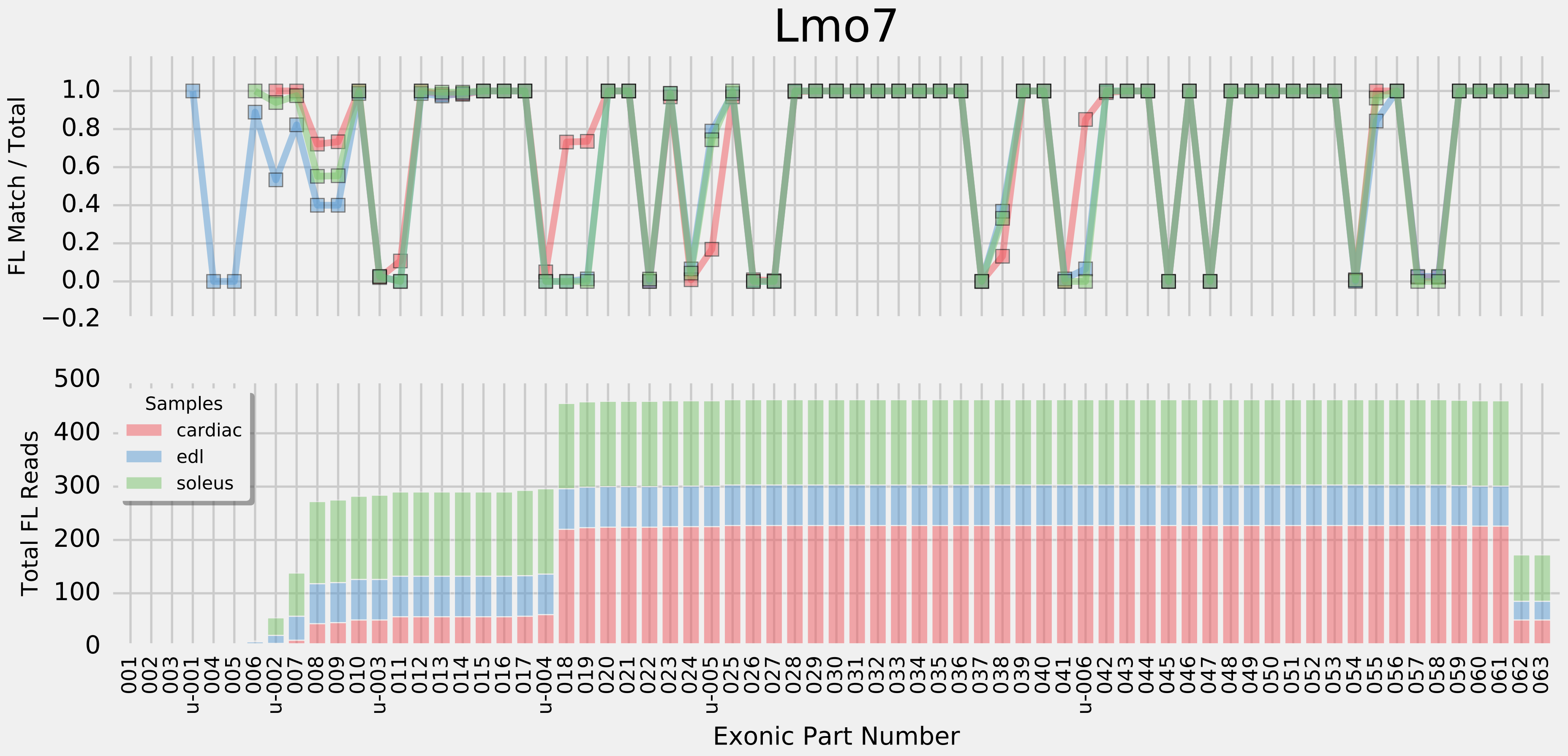

Lmo7
FL Match / Total
1.0
0.8
0.6
0.4
0.2
0.0
−0.2
Total FL Reads
500
400
300
200
100
0
Samples
cardiac
edl
soleus
001 002 003 u-001 004 005 006 u-002 007 008 009 010 u-003 011 012 013 014 015 016 017 u-004 018 019 020 021 022 023 024 u-005 025 026 027 028 029 030 031 032 033 034 035 036 037 038 039 040 041 u-006 042 043 044 045 046 047 048 049 050 051 052 053 054 055 056 057 058 059 060 061 062 063
Exonic Part Number

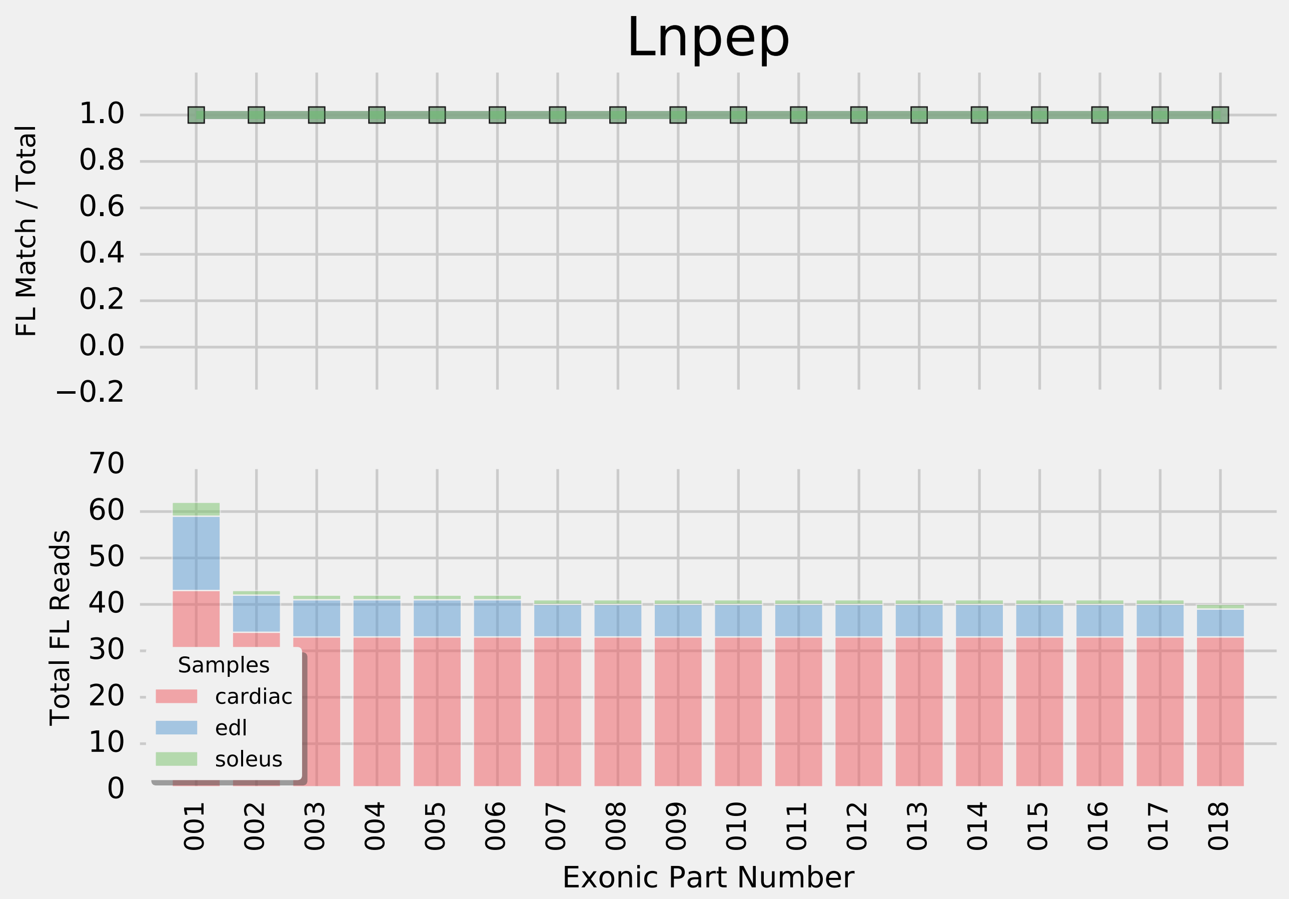
Lnpep
FL Match / Total
1.0
0.8
0.6
0.4
0.2
0.0
−0.2
Total FL Reads
70
60
50
40
30
20
10
0
Samples
cardiac
edl
soleus
001
002
003
004
005
006
007
008
009
010
011
012
013
014
015
016
017
018
Exonic Part Number

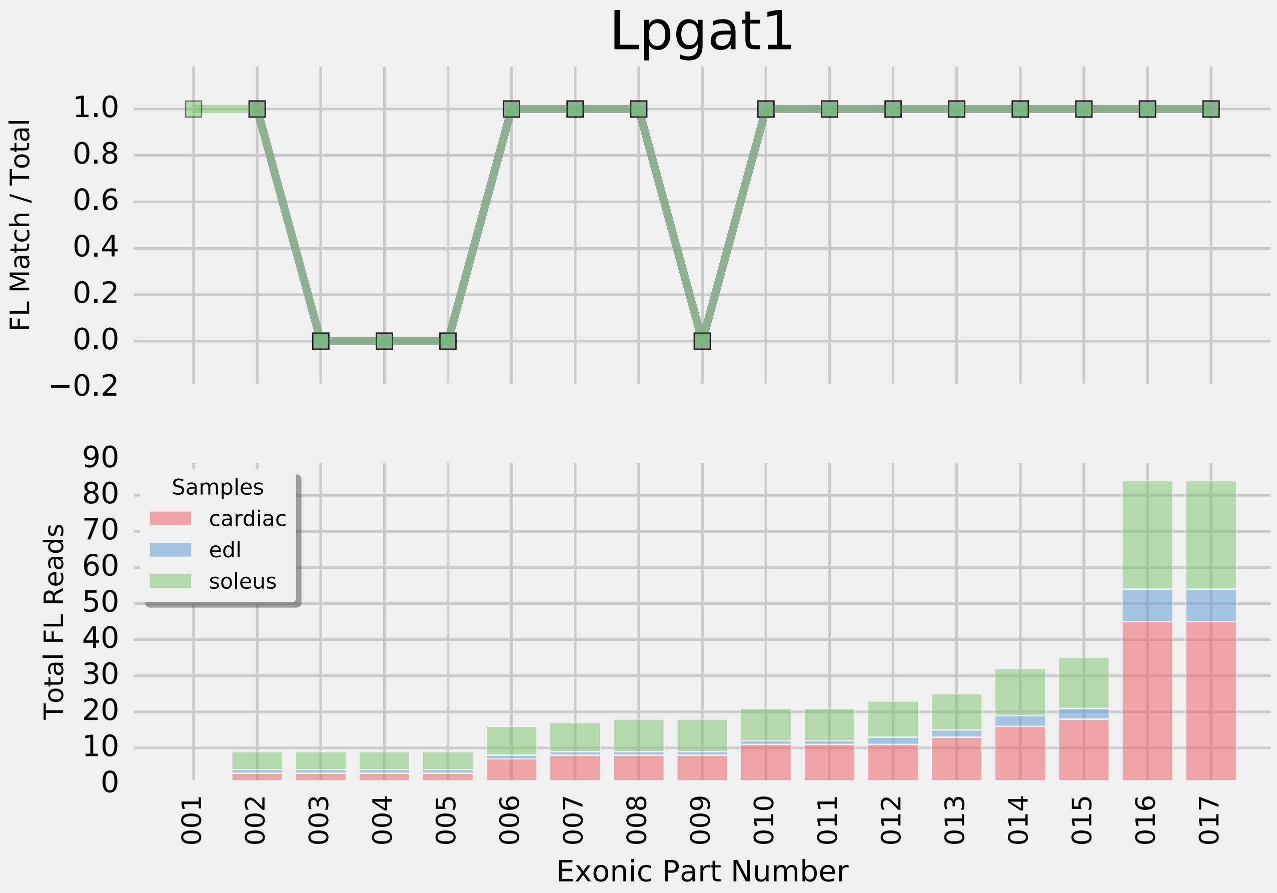
Lpgat1
FL Match / Total
1.0
0.8
0.6
0.4
0.2
0.0
−0.2
Total FL Reads
90
80
70
60
50
40
30
20
10
0
Samples
cardiac
edl
soleus
001
002
003
004
005
006
007
008
009
010
011
012
013
014
015
016
017
Exonic Part Number

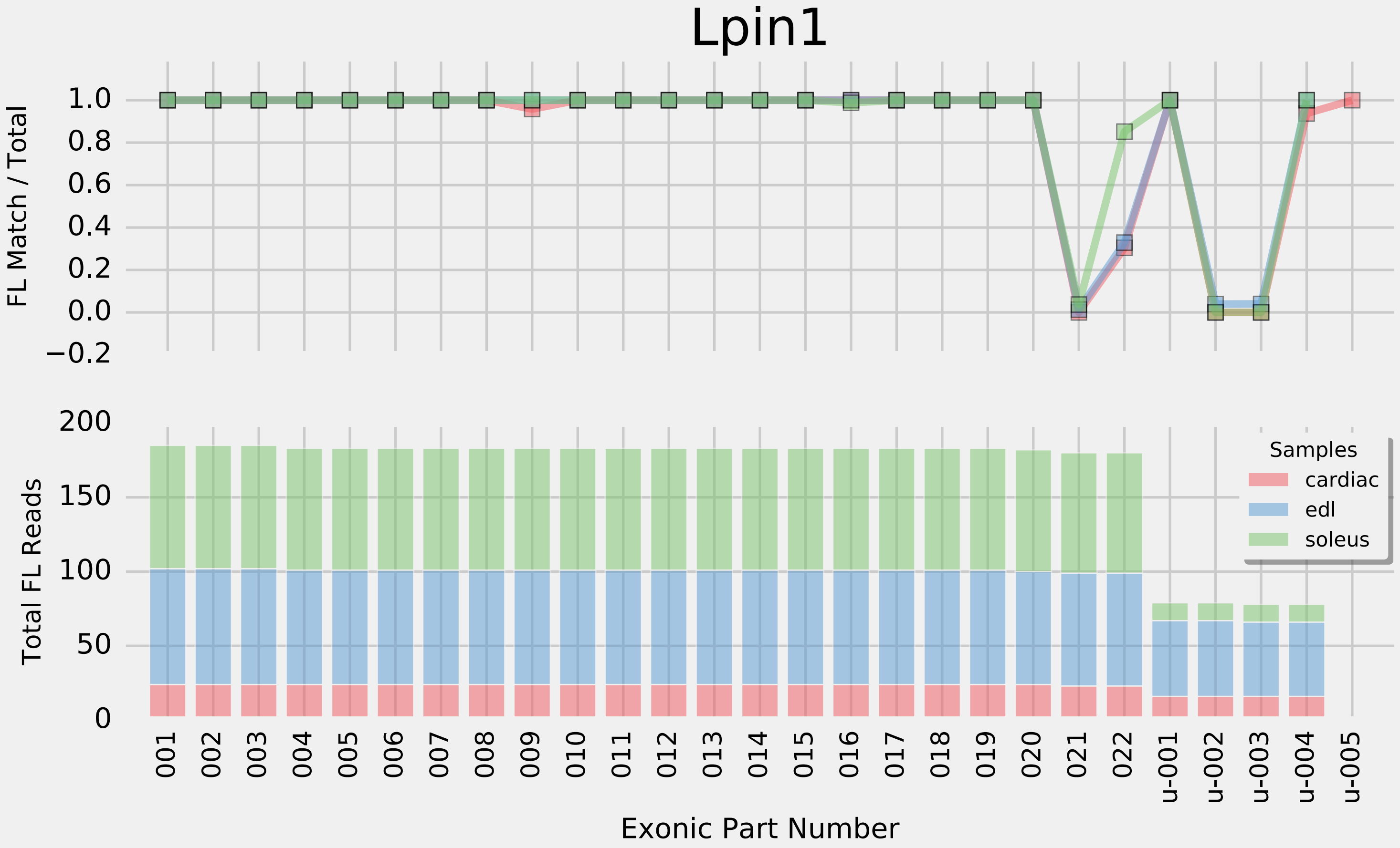

Lpin1
FL Match / Total
1.0
0.8
0.6
0.4
0.2
0.0
−0.2
Total FL Reads
200
150
100
50
0
Samples
cardiac
edl
soleus
001
002
003
004
005
006
007
008
009
010
011
012
013
014
015
016
017
018
019
020
021
022
u-001
u-002
u-003
u-004
u-005
Exonic Part Number

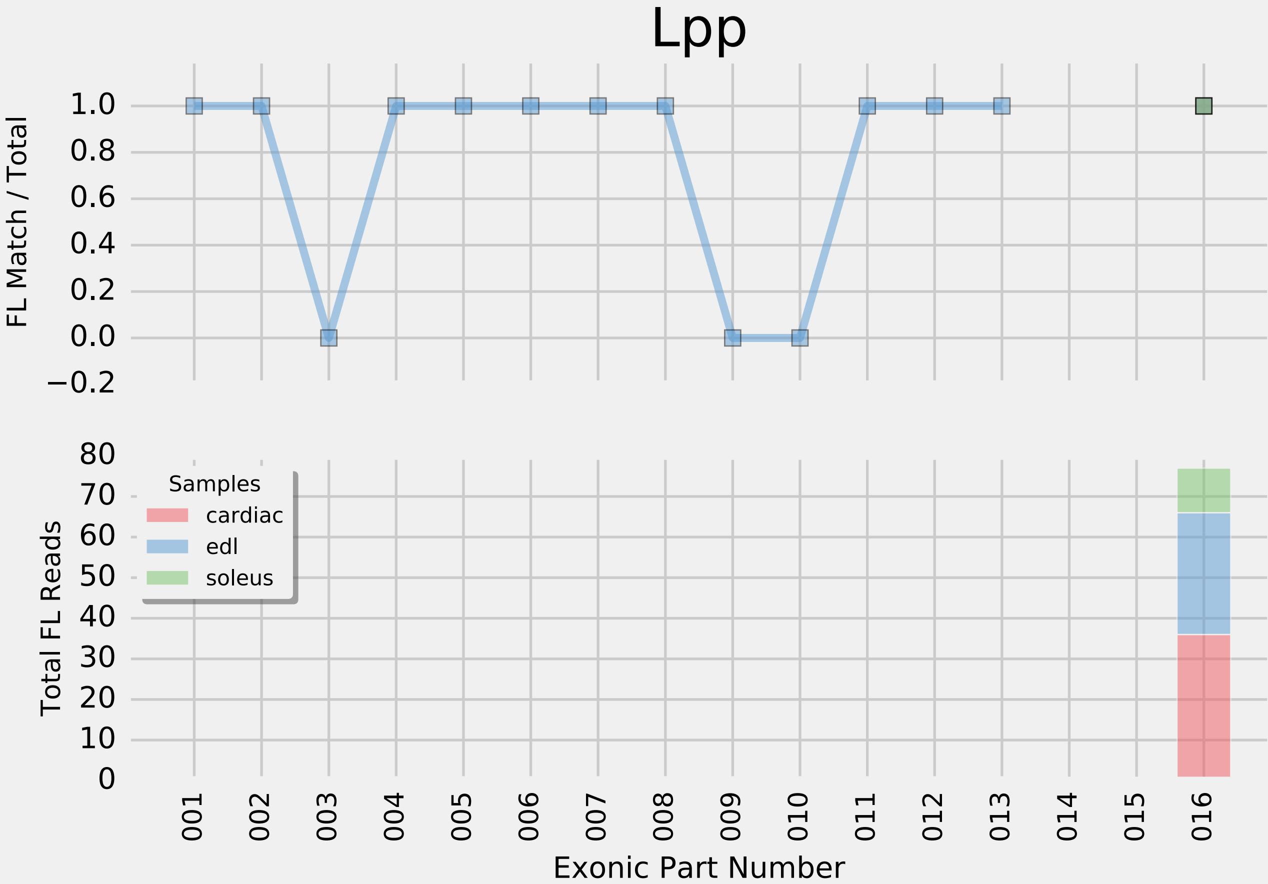

Lpp
FL Match / Total
1.0
0.8
0.6
0.4
0.2
0.0
−0.2
Total FL Reads
80
70
60
50
40
30
20
10
0
Samples
cardiac
edl
soleus
001
002
003
004
005
006
007
008
009
010
011
012
013
014
015
016
Exonic Part Number

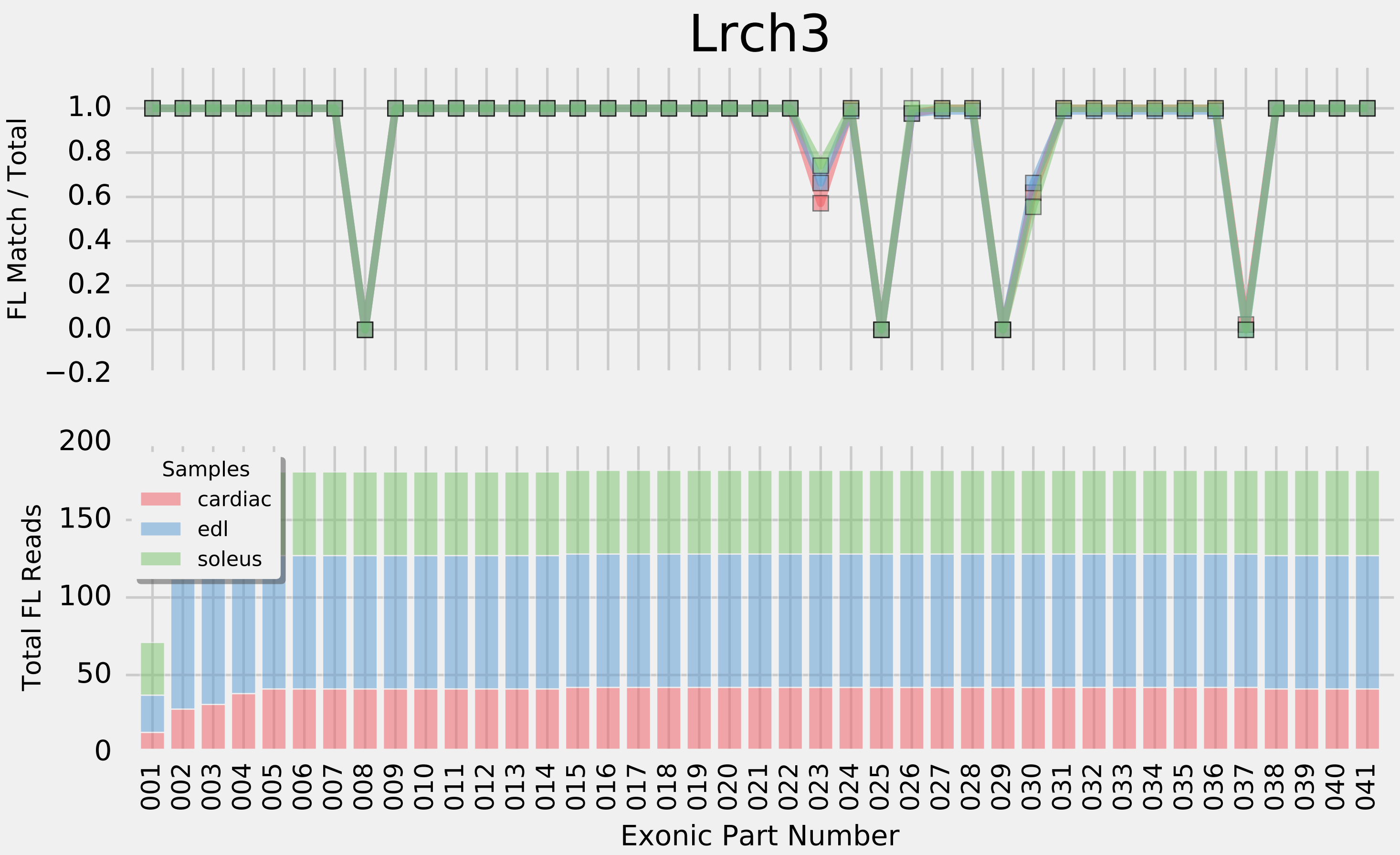

Lrch3
FL Match / Total
1.0
0.8
0.6
0.4
0.2
0.0
−0.2
Total FL Reads
200
150
100
50
0
Samples
cardiac
edl
soleus
001
002
003
004
005
006
007
008
009
010
011
012
013
014
015
016
017
018
019
020
021
022
023
024
025
026
027
028
029
030
031
032
033
034
035
036
037
038
039
040
041
Exonic Part Number

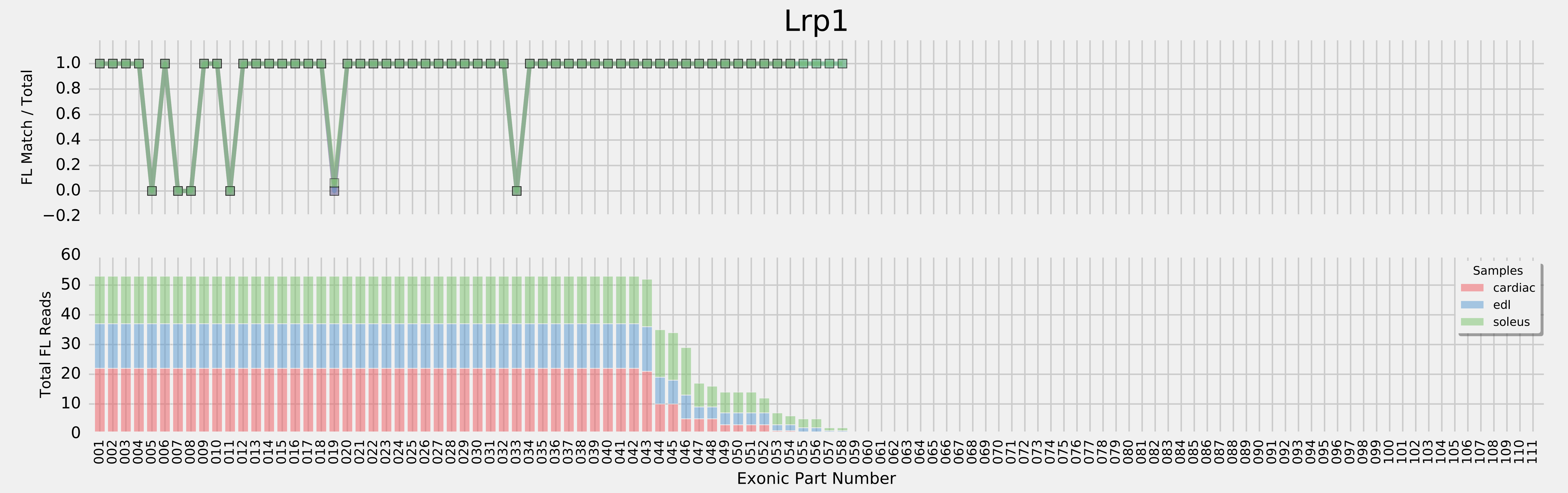

Lrp1
FL Match / Total
Total FL Reads
Exonic Part Number
Samples
cardiac
edl
soleus

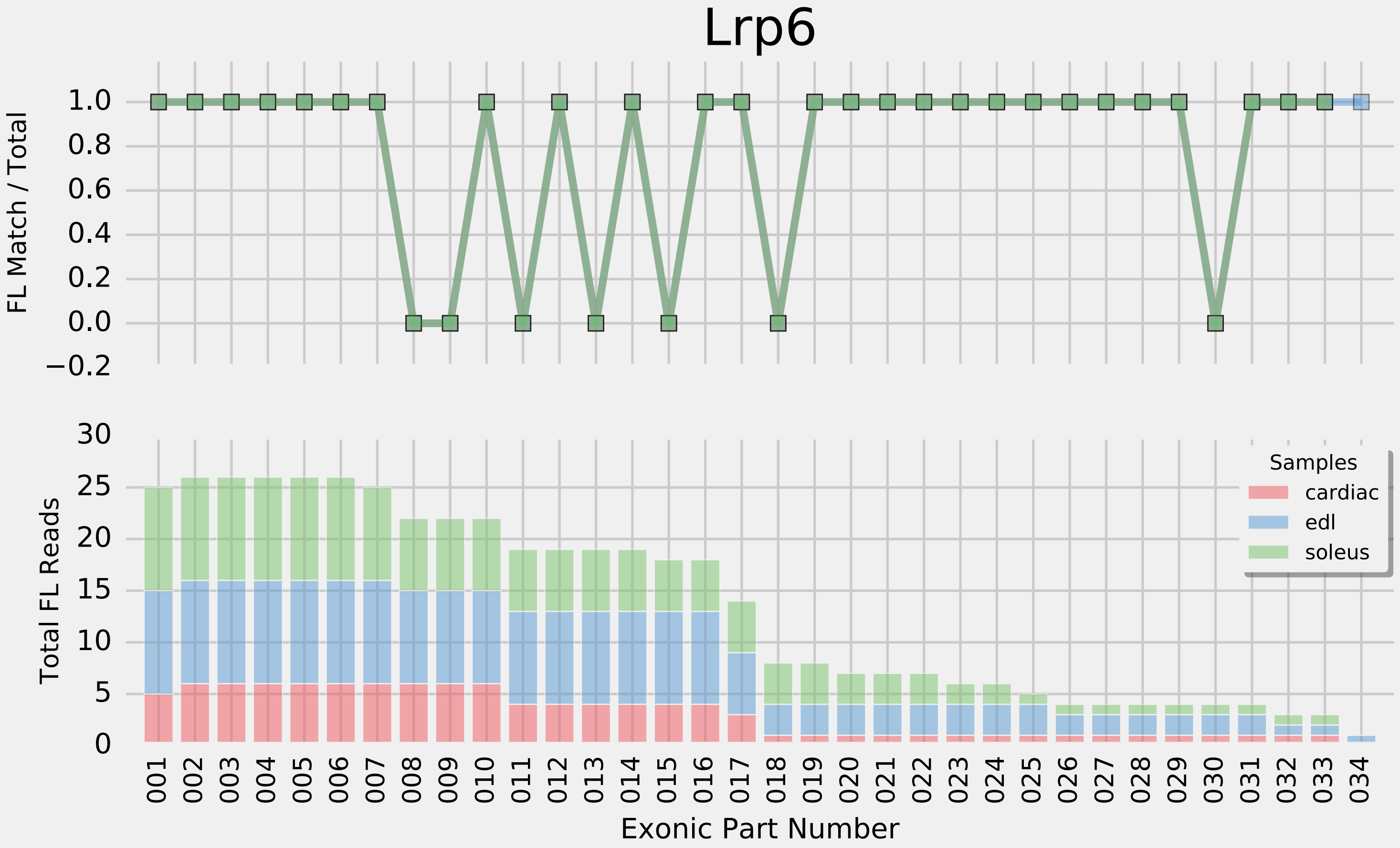

Lrp6
FL Match / Total
1.0
0.8
0.6
0.4
0.2
0.0
−0.2
Total FL Reads
30
25
20
15
10
5
0
Samples
cardiac
edl
soleus
001
002
003
004
005
006
007
008
009
010
011
012
013
014
015
016
017
018
019
020
021
022
023
024
025
026
027
028
029
030
031
032
033
034
Exonic Part Number

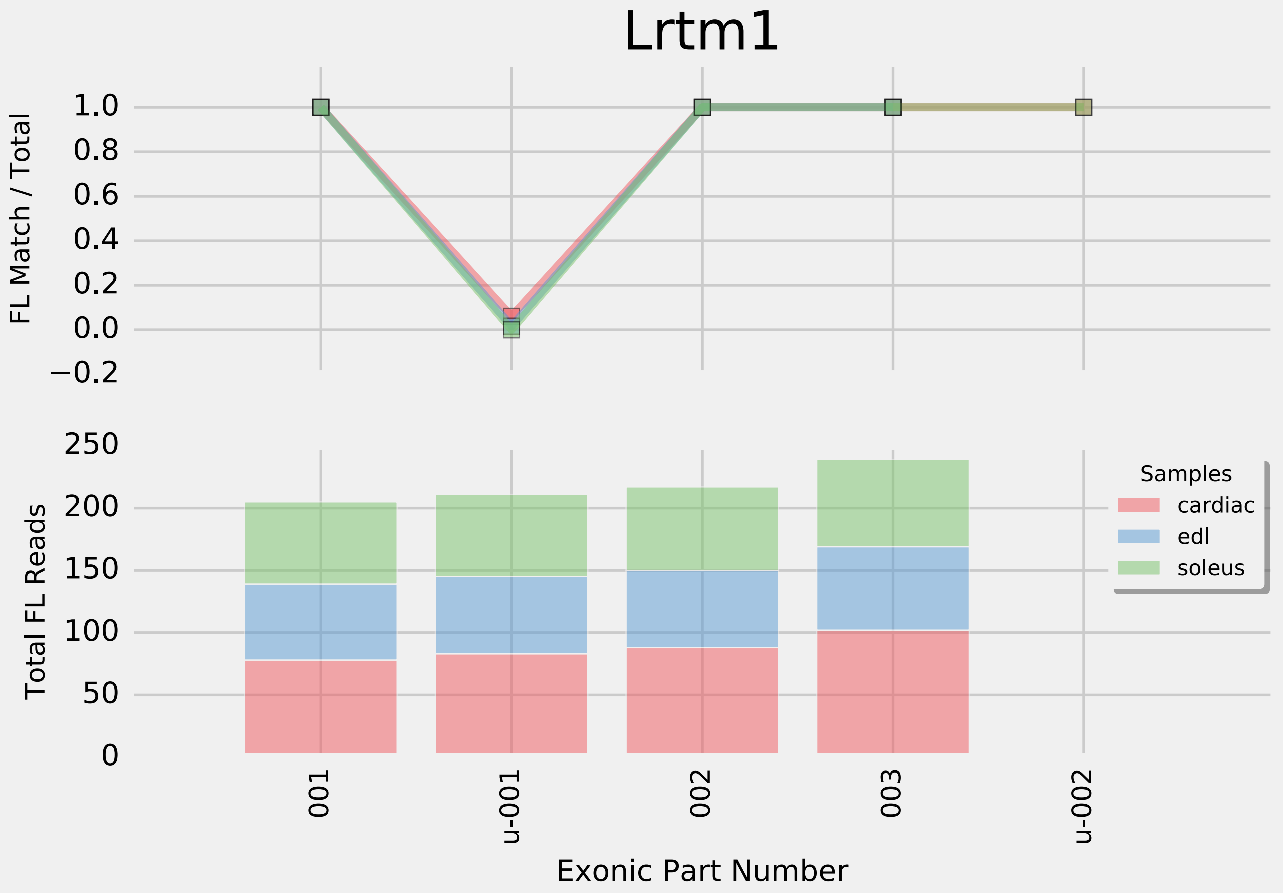

Lrtm1
FL Match / Total
1.0
0.8
0.6
0.4
0.2
0.0
−0.2
Total FL Reads
250
200
150
100
50
0
Samples
cardiac
edl
soleus
001
u-001
002
003
u-002
Exonic Part Number

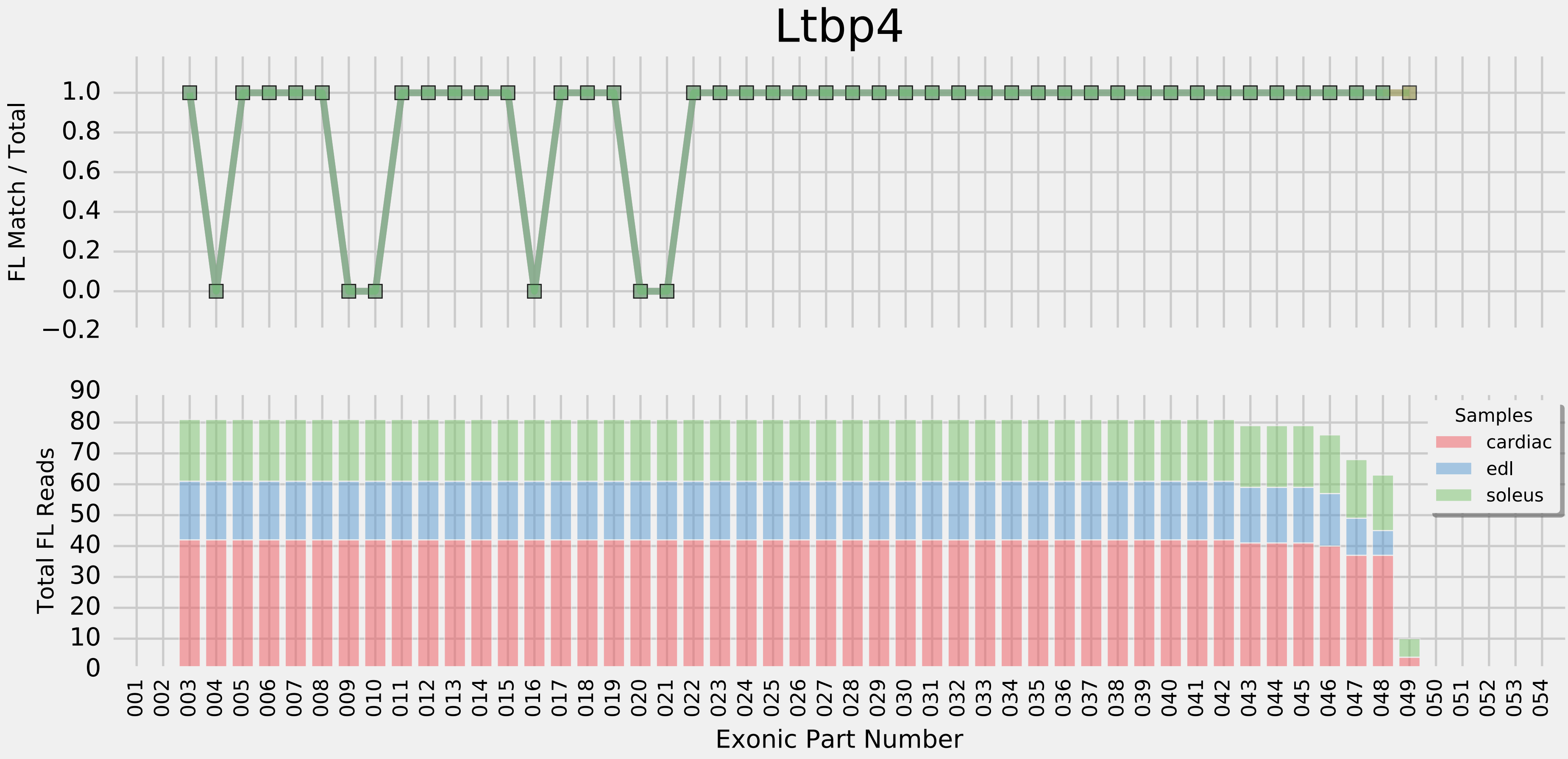

Ltbp4
FL Match / Total
Total FL Reads
Exonic Part Number
Samples
cardiac
edl
soleus

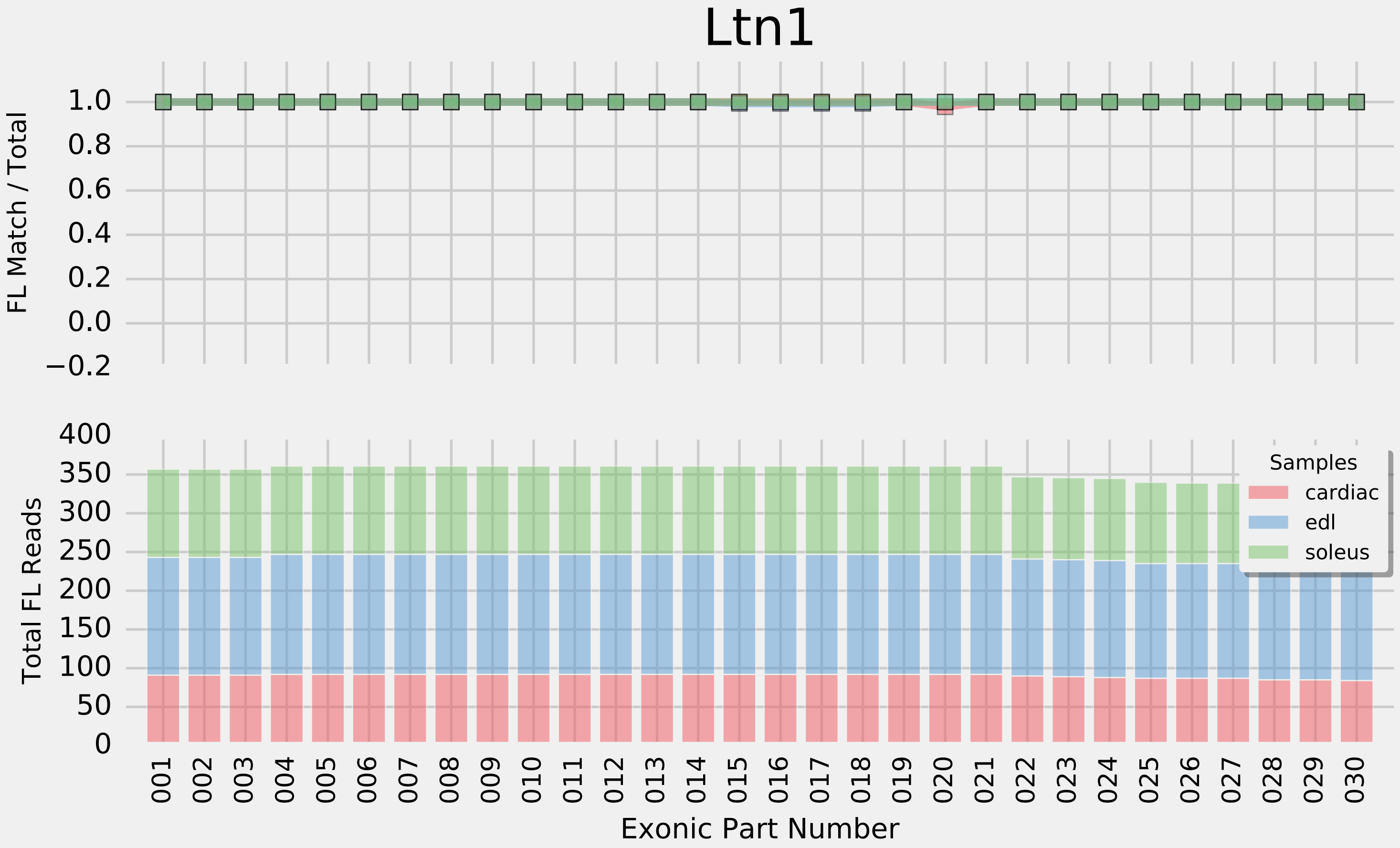

Ltn1
FL Match / Total
1.0
0.8
0.6
0.4
0.2
0.0
−0.2
Total FL Reads
400
350
300
250
200
150
100
50
0
001 002 003 004 005 006 007 008 009 010 011 012 013 014 015 016 017 018 019 020 021 022 023 024 025 026 027 028 029 030
Exonic Part Number
Samples
cardiac
edl
soleus

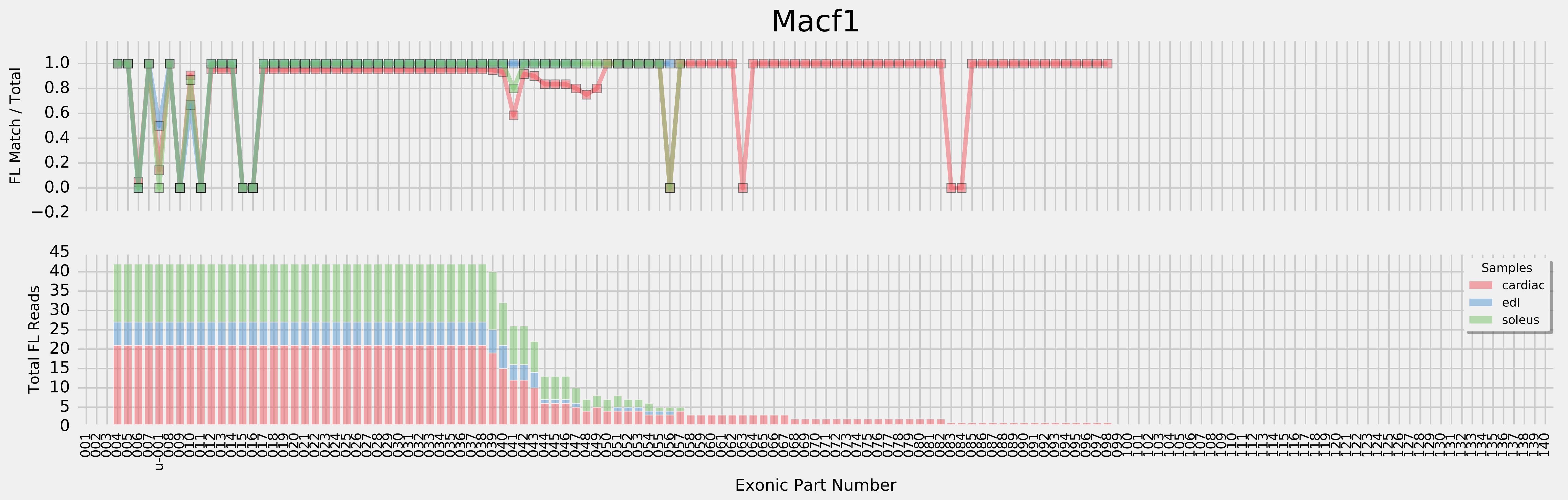

Macf1
FL Match / Total
Total FL Reads
Exonic Part Number
Samples
cardiac
edl
soleus

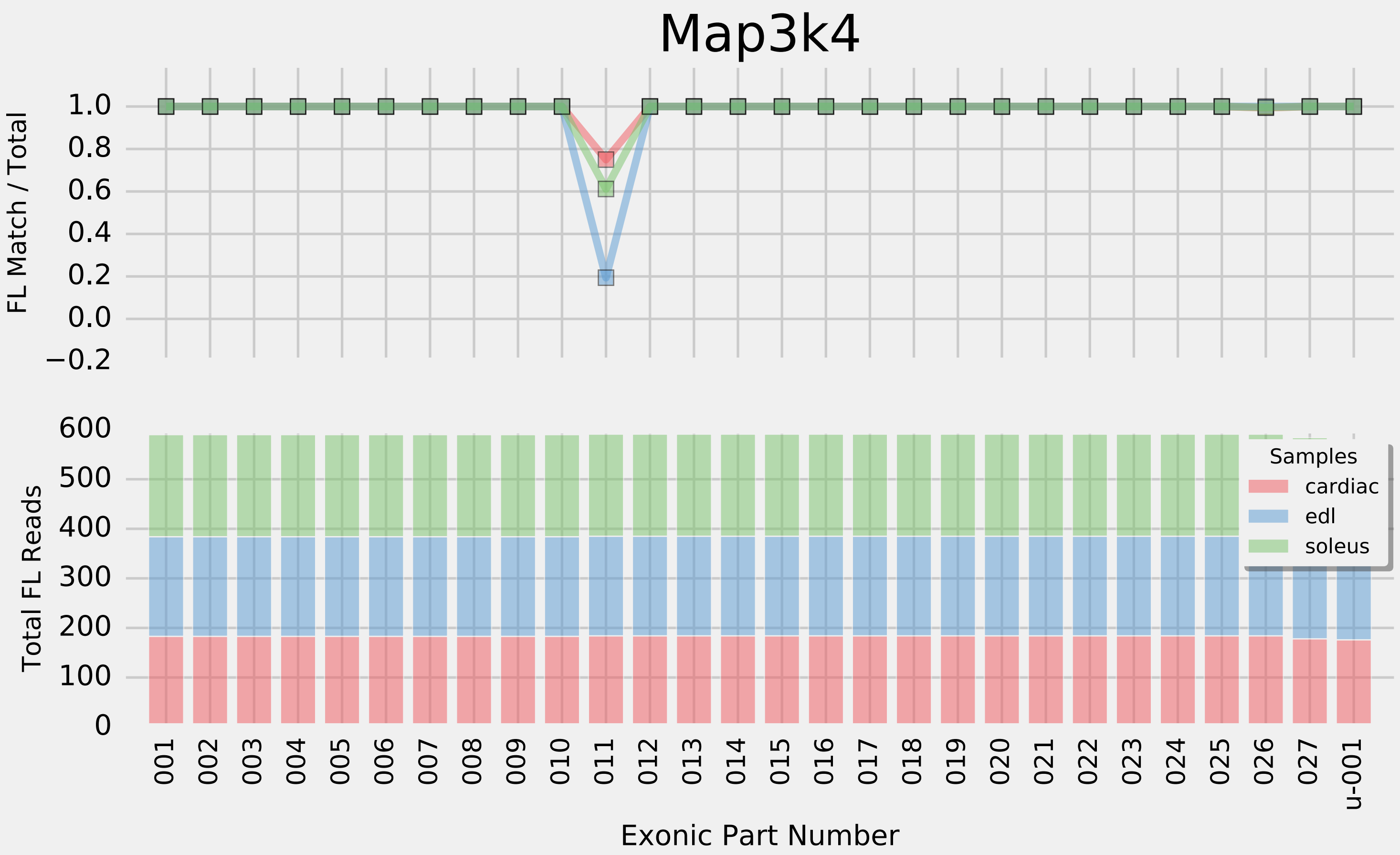

Map3k4
FL Match / Total
1.0
0.8
0.6
0.4
0.2
0.0
−0.2
600
500
400
300
200
100
0
Total FL Reads
Samples
cardiac
edl
soleus
001
002
003
004
005
006
007
008
009
010
011
012
013
014
015
016
017
018
019
020
021
022
023
024
025
026
027
u-001
Exonic Part Number

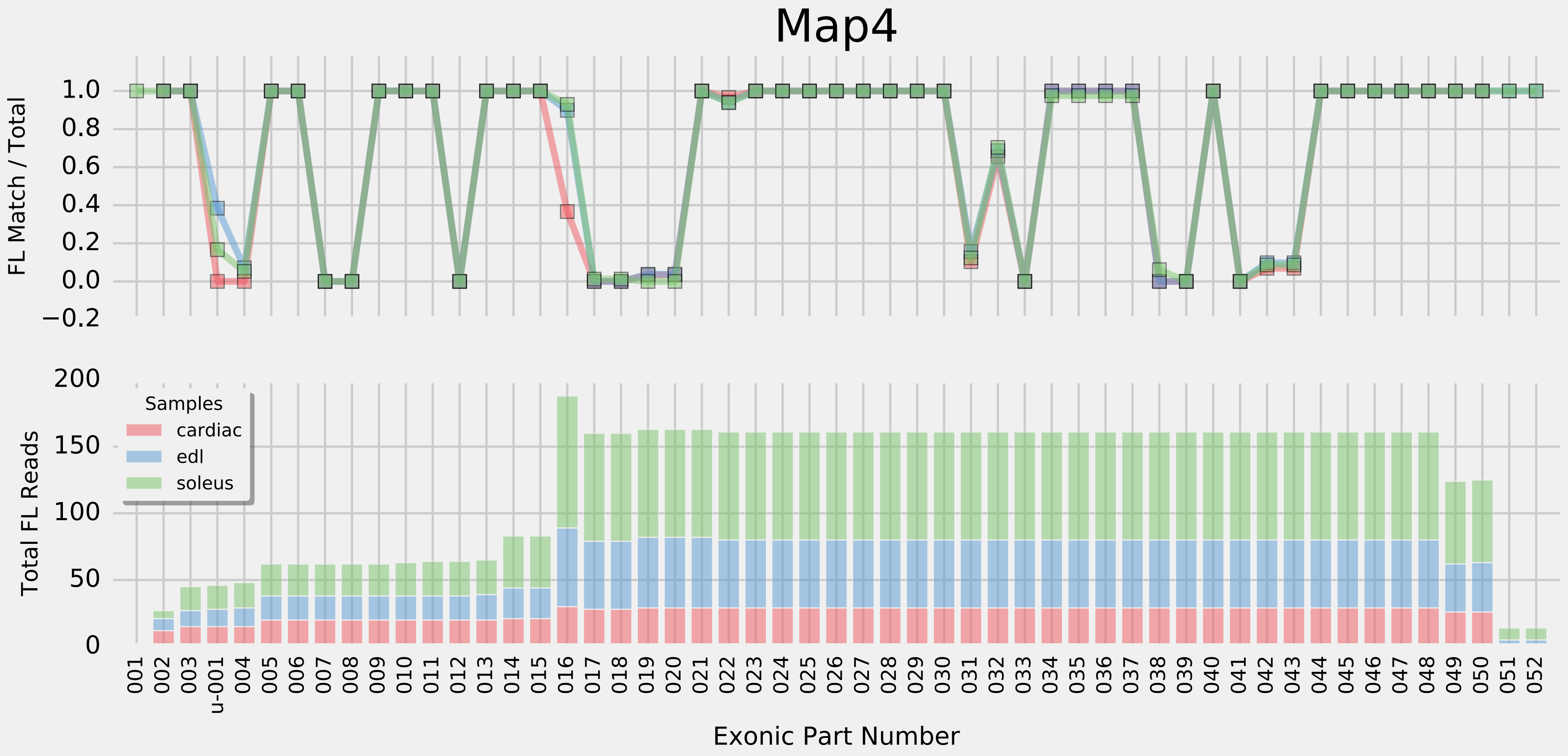

Map4
FL Match / Total
1.0
0.8
0.6
0.4
0.2
0.0
−0.2
Total FL Reads
200
150
100
50
0
Samples
cardiac
edl
soleus
001
002
003
u-001
004
005
006
007
008
009
010
011
012
013
014
015
016
017
018
019
020
021
022
023
024
025
026
027
028
029
030
031
032
033
034
035
036
037
038
039
040
041
042
043
044
045
046
047
048
049
050
051
052
Exonic Part Number

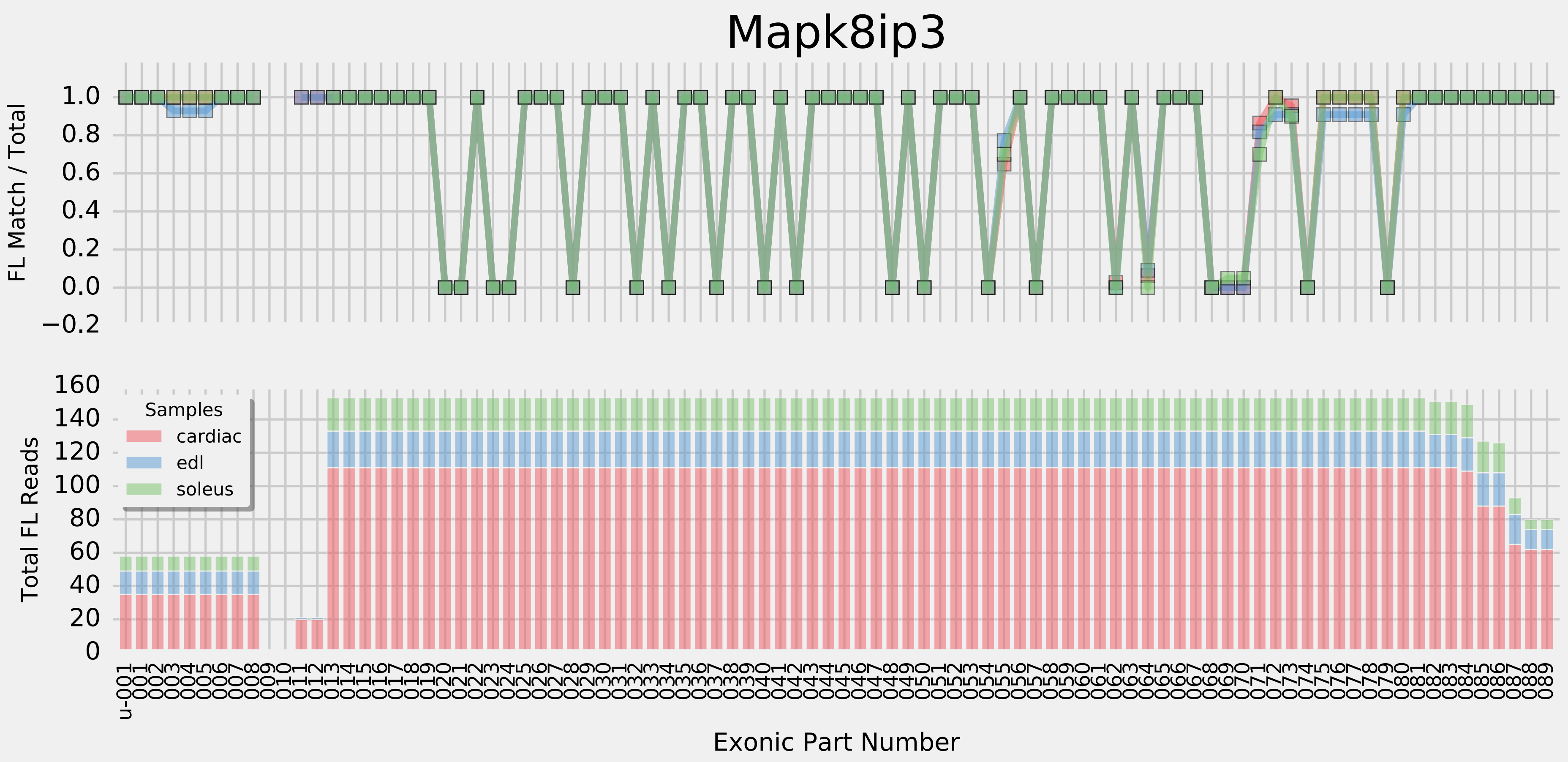

Mapk8ip3
FL Match / Total
Total FL Reads
Samples
cardiac
edl
soleus
Exonic Part Number

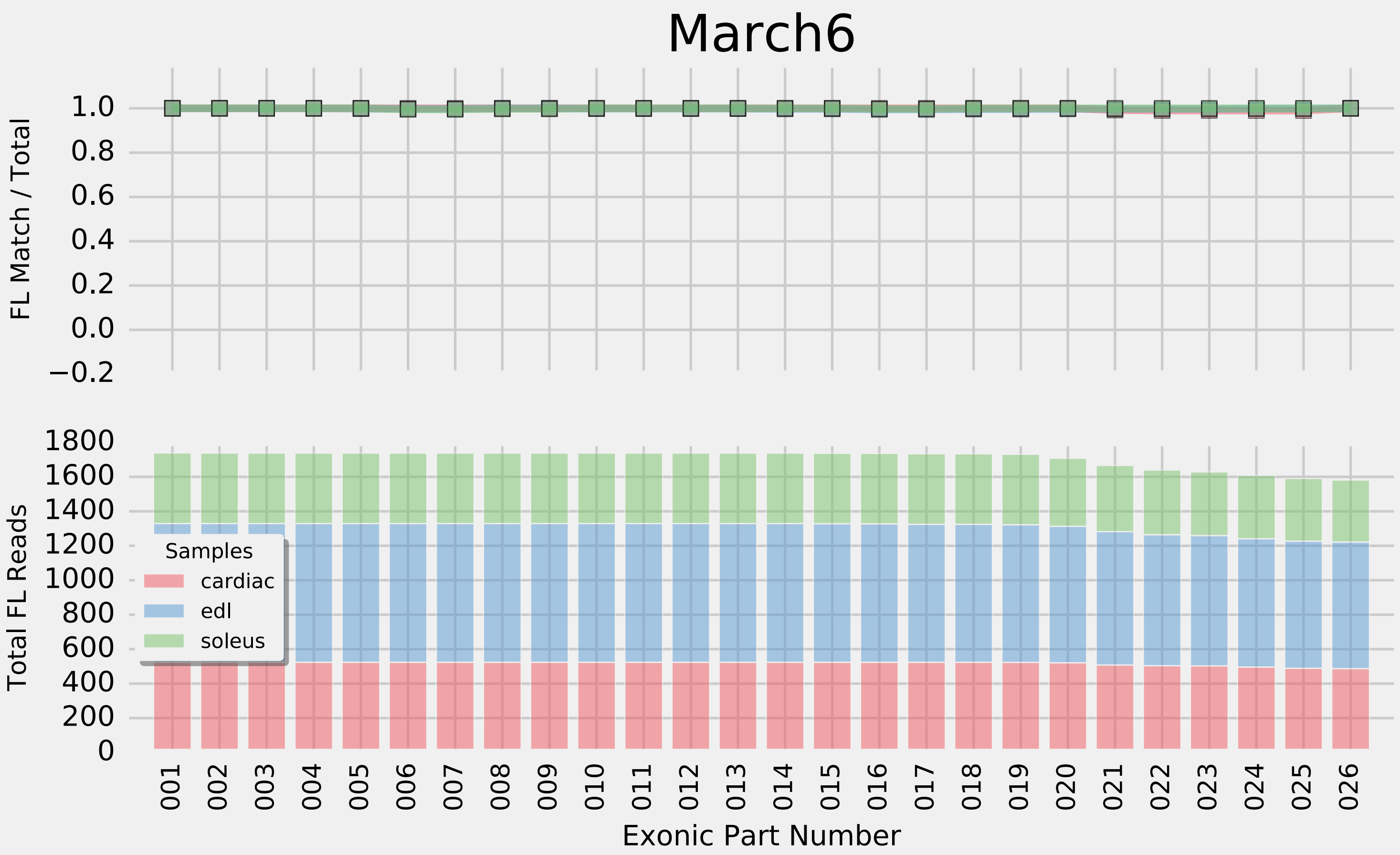

March6
FL Match / Total
1.0
0.8
0.6
0.4
0.2
0.0
−0.2
Total FL Reads
1800
1600
1400
1200
1000
800
600
400
200
0
Samples
cardiac
edl
soleus
001
002
003
004
005
006
007
008
009
010
011
012
013
014
015
016
017
018
019
020
021
022
023
024
025
026
Exonic Part Number

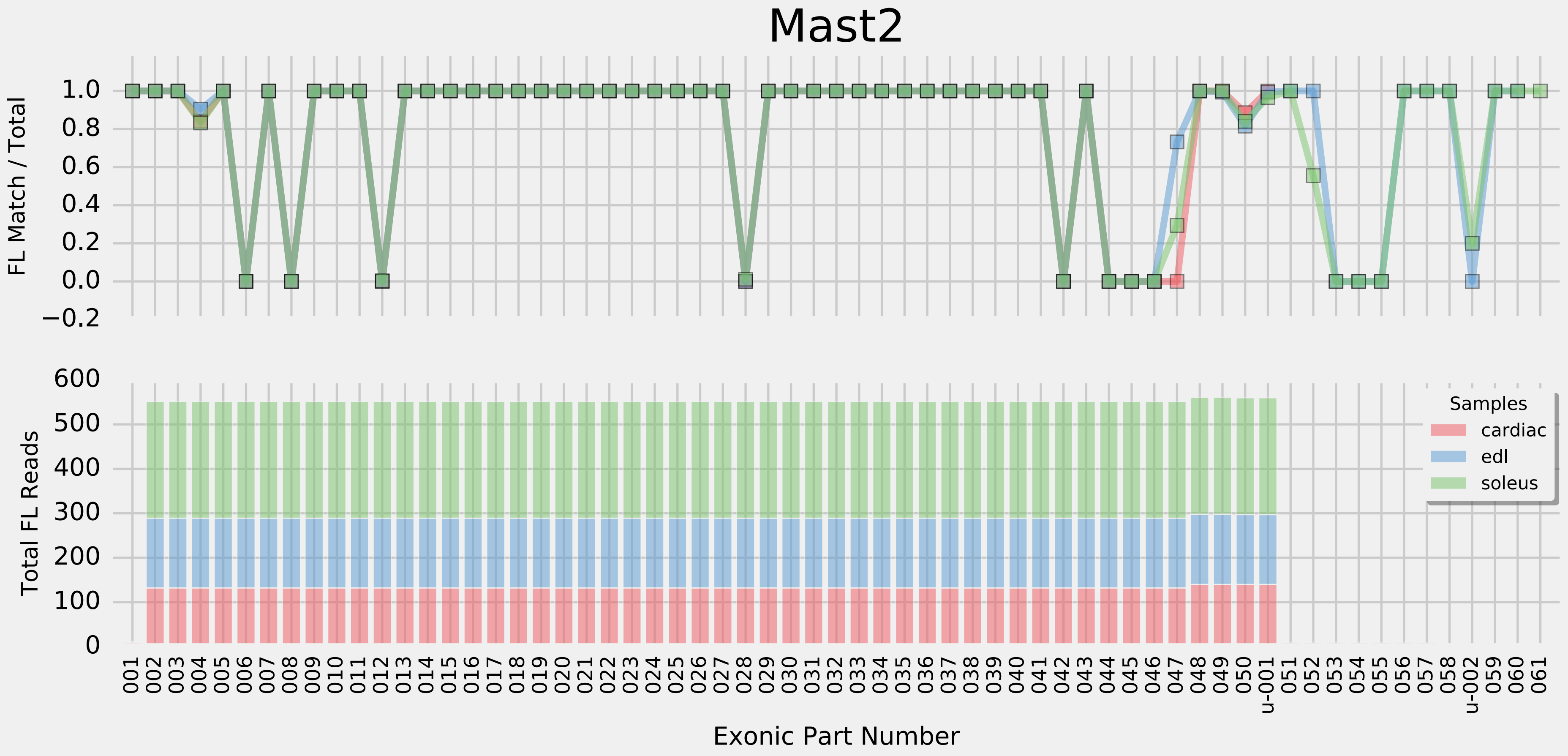
Mast2
FL Match / Total
1.0
0.8
0.6
0.4
0.2
0.0
−0.2
Total FL Reads
600
500
400
300
200
100
0
Samples
cardiac
edl
soleus
001 002 003 004 005 006 007 008 009 010 011 012 013 014 015 016 017 018 019 020 021 022 023 024 025 026 027 028 029 030 031 032 033 034 035 036 037 038 039 040 041 042 043 044 045 046 047 048 049 050 u-001 051 052 053 054 055 056 057 058 u-002 059 060 061
Exonic Part Number

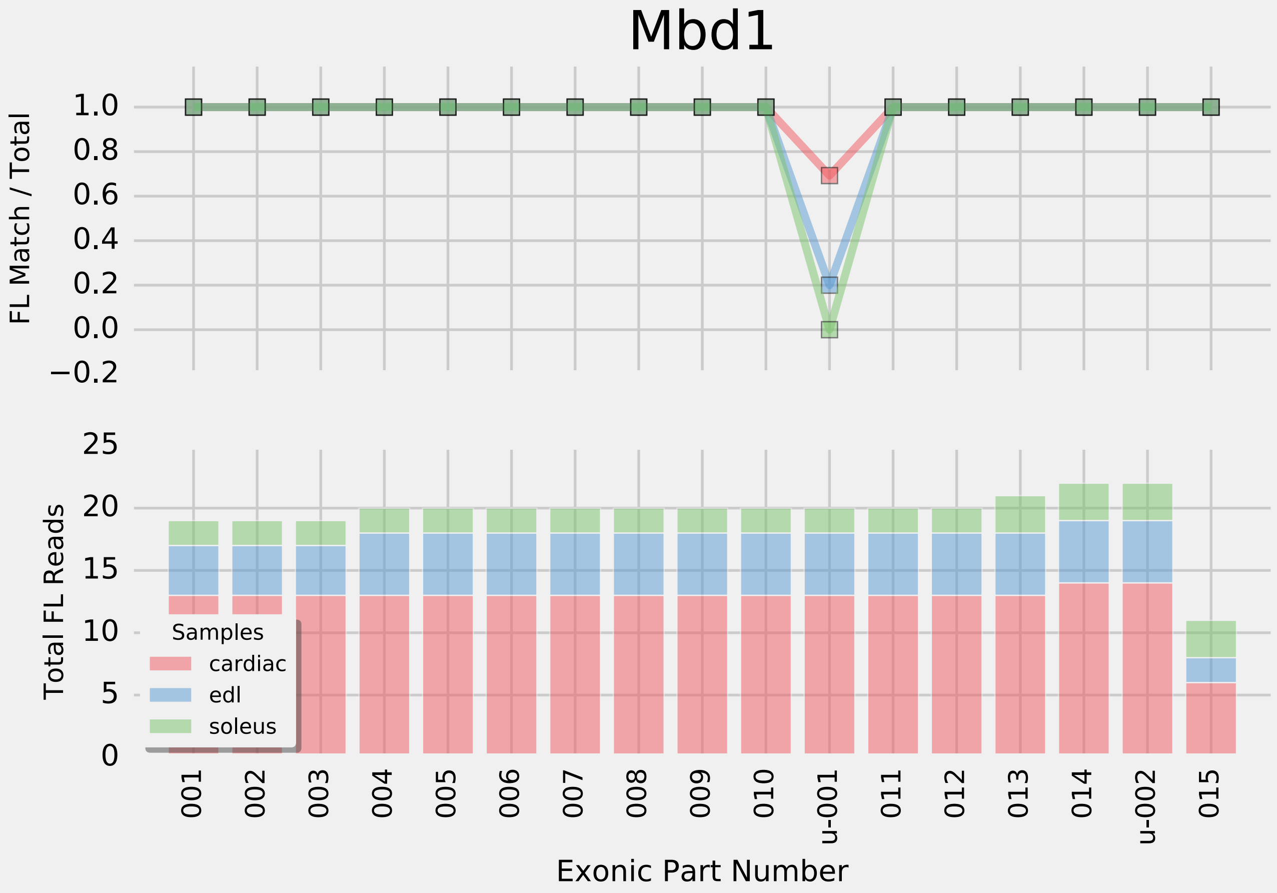
Mbd1
FL Match / Total
1.0
0.8
0.6
0.4
0.2
0.0
−0.2
Total FL Reads
25
20
15
10
5
0
Samples
cardiac
edl
soleus
001
002
003
004
005
006
007
008
009
010
u-001
011
012
013
014
u-002
015
Exonic Part Number

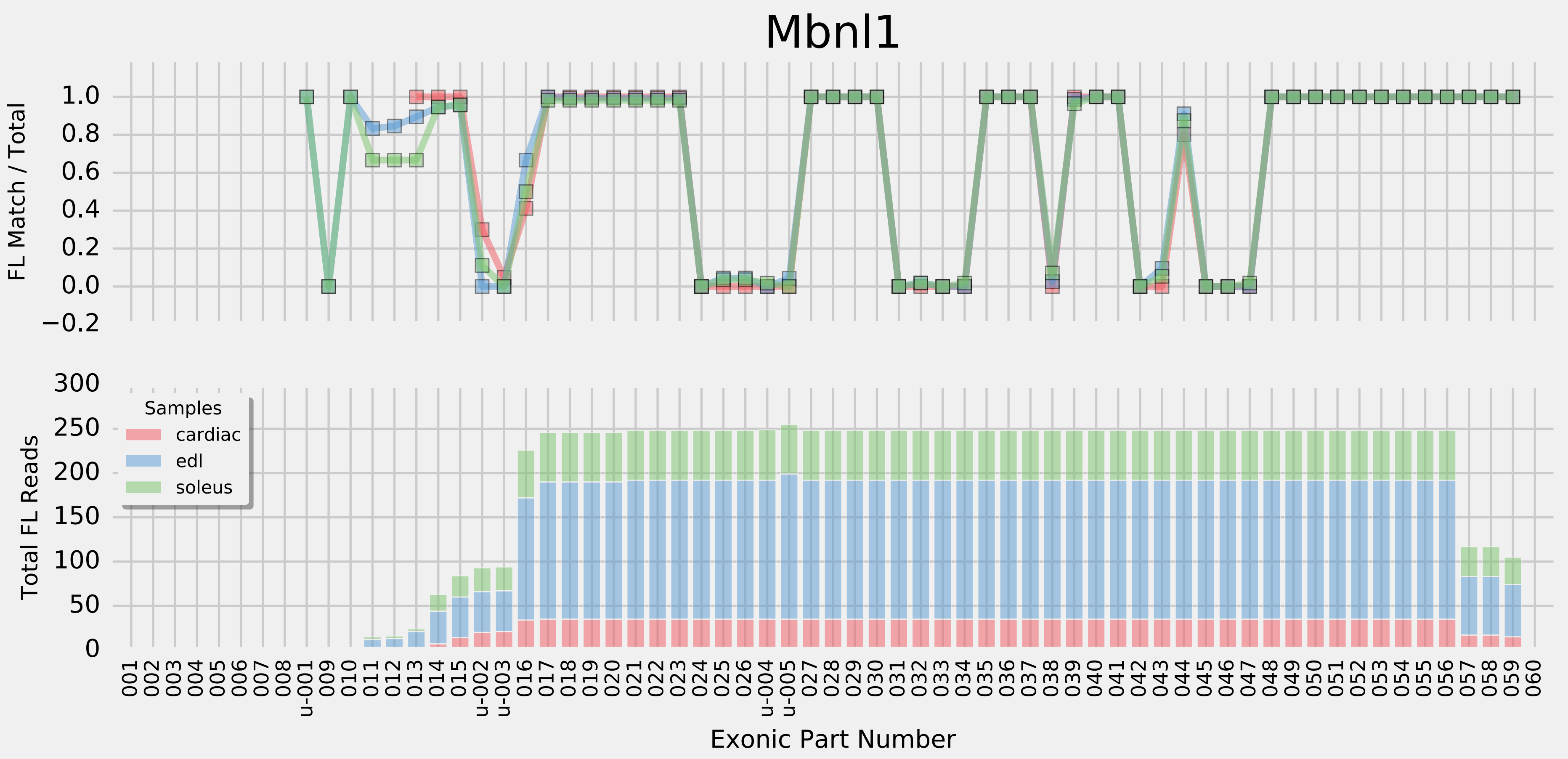

Mbnl1
FL Match / Total
Total FL Reads
Samples
cardiac
edl
soleus
Exonic Part Number

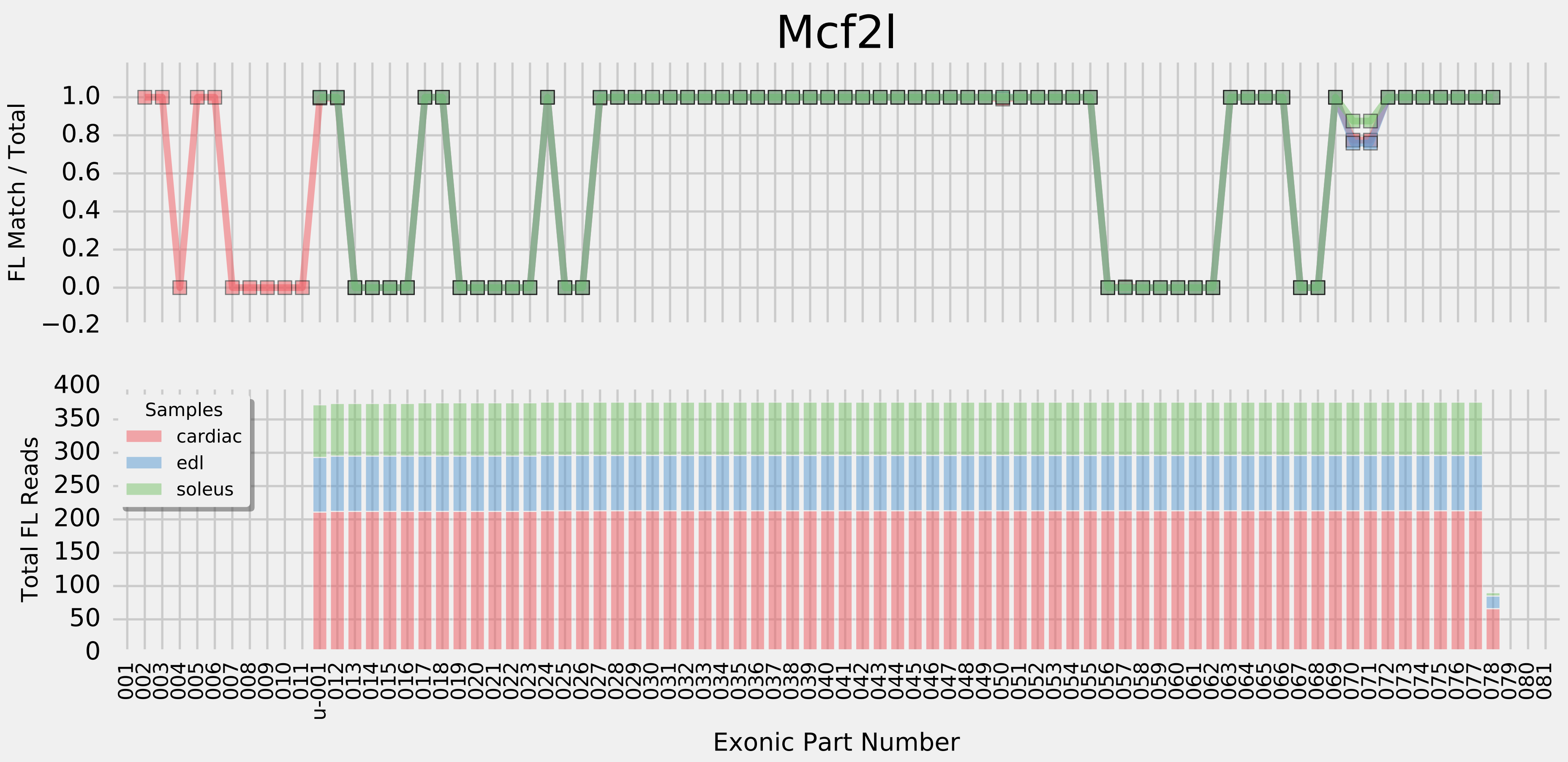

Mcf2l
FL Match / Total
Total FL Reads
Samples
cardiac
edl
soleus
Exonic Part Number

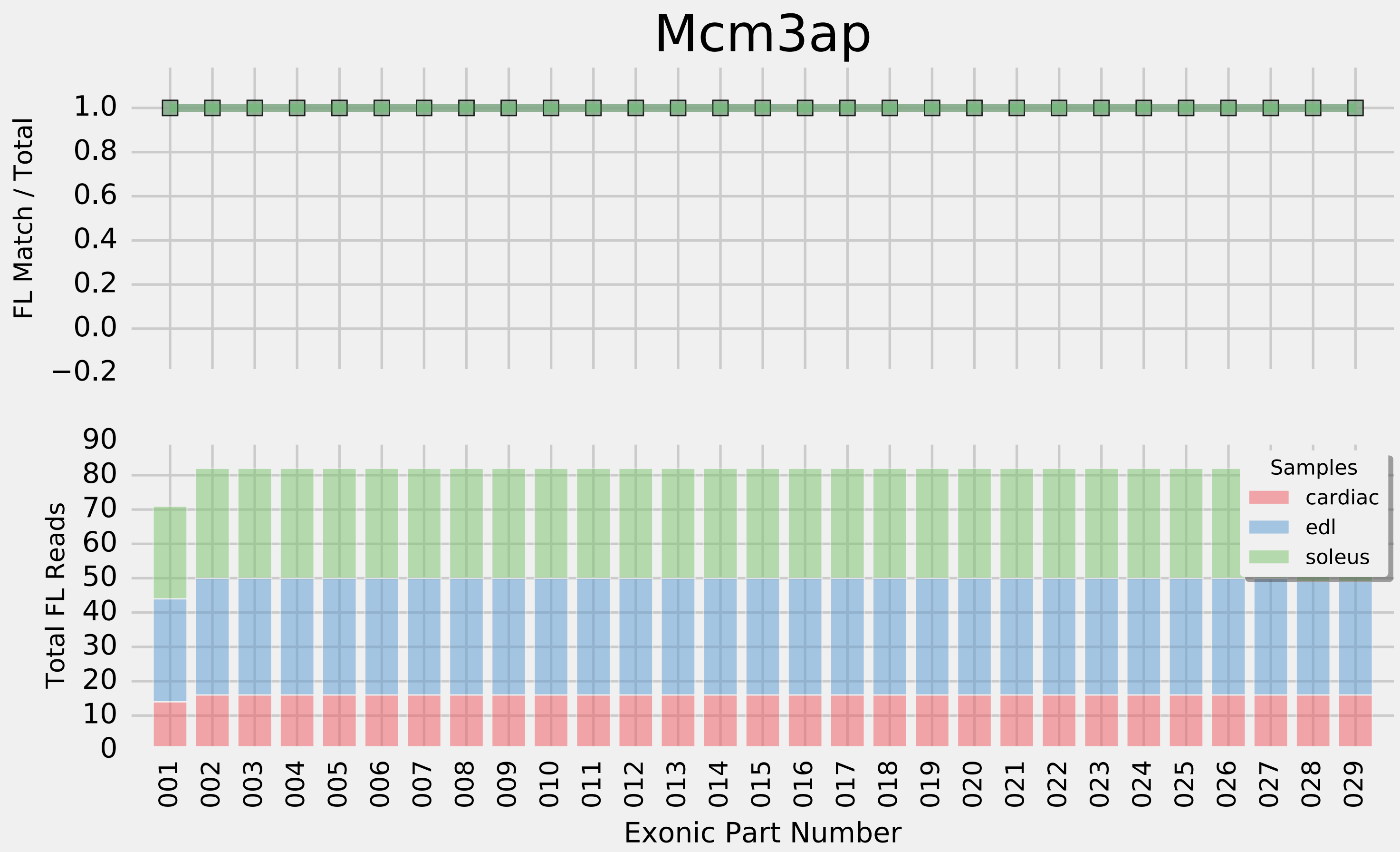

Mcm3ap
FL Match / Total
1.0
0.8
0.6
0.4
0.2
0.0
−0.2
Total FL Reads
90
80
70
60
50
40
30
20
10
0
Samples
cardiac
edl
soleus
001
002
003
004
005
006
007
008
009
010
011
012
013
014
015
016
017
018
019
020
021
022
023
024
025
026
027
028
029
Exonic Part Number

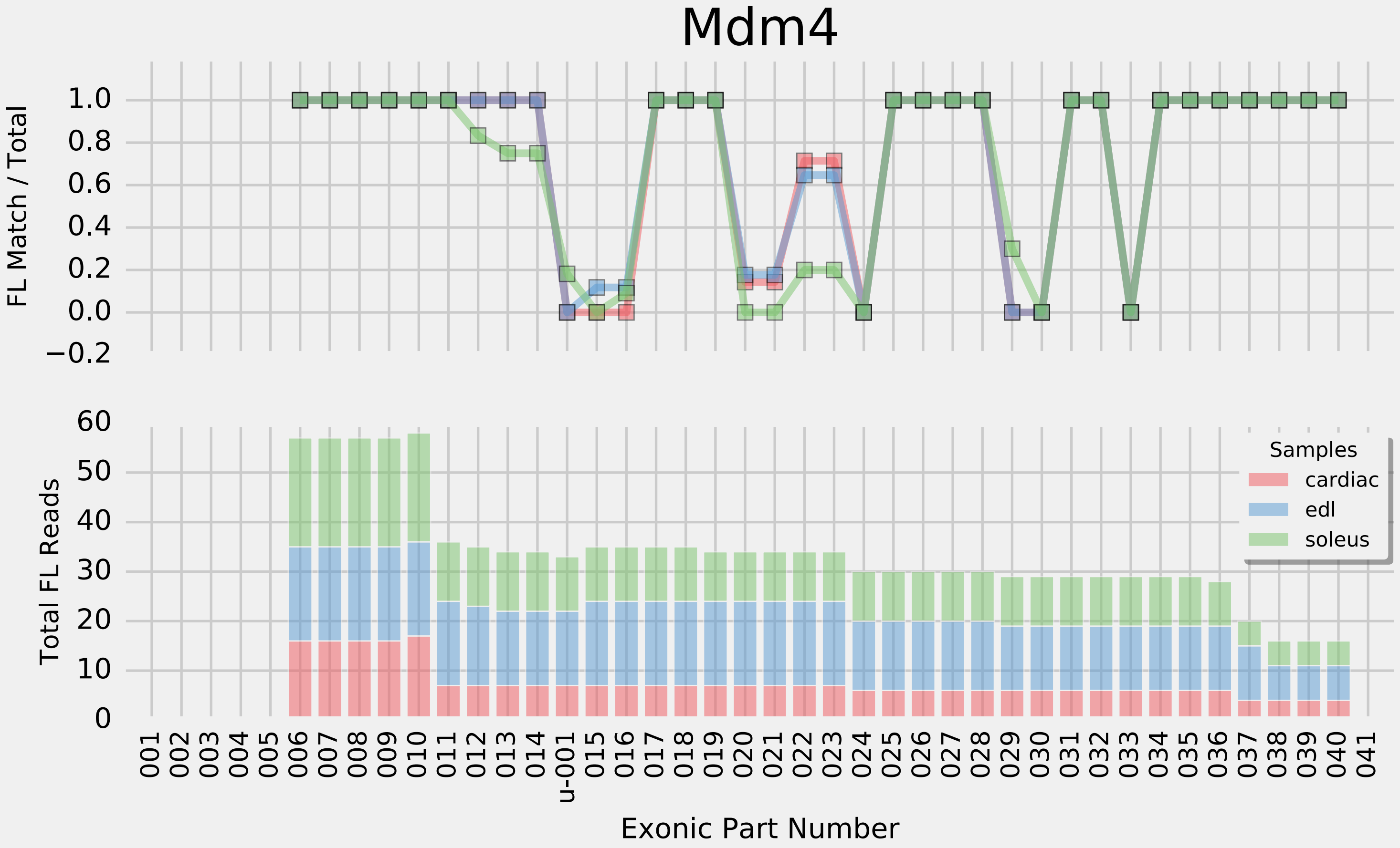
Mdm4
FL Match / Total
1.0
0.8
0.6
0.4
0.2
0.0
−0.2
Total FL Reads
60
50
40
30
20
10
0
Samples
cardiac
edl
soleus
001 002 003 004 005 006 007 008 009 010 011 012 013 014 u-001 015 016 017 018 019 020 021 022 023 024 025 026 027 028 029 030 031 032 033 034 035 036 037 038 039 040 041
Exonic Part Number

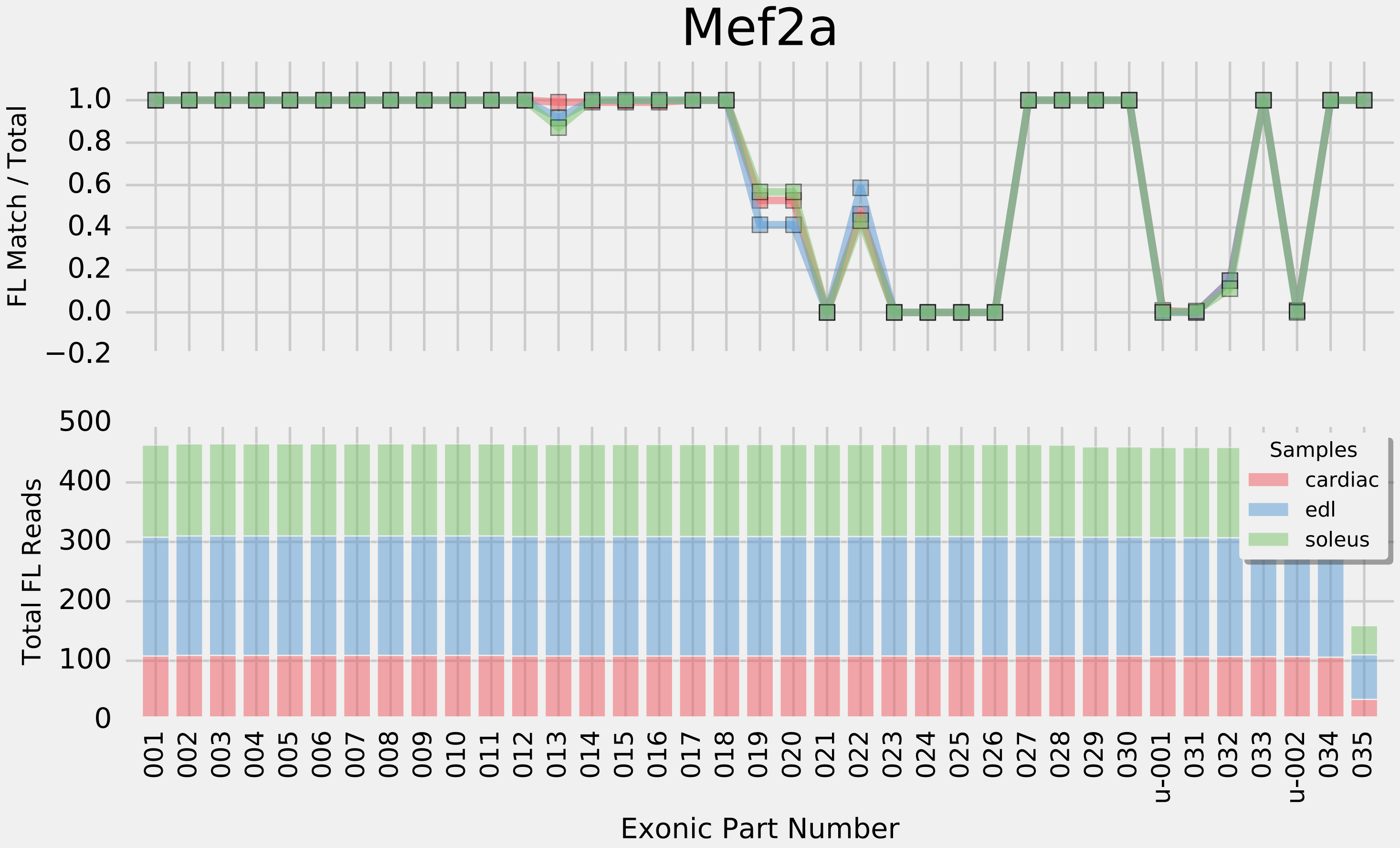

Mef2a
FL Match / Total
1.0
0.8
0.6
0.4
0.2
0.0
−0.2
Total FL Reads
500
400
300
200
100
0
Samples
cardiac
edl
soleus
001 002 003 004 005 006 007 008 009 010 011 012 013 014 015 016 017 018 019 020 021 022 023 024 025 026 027 028 029 030 u-001 031 032 033 u-002 034 035
Exonic Part Number

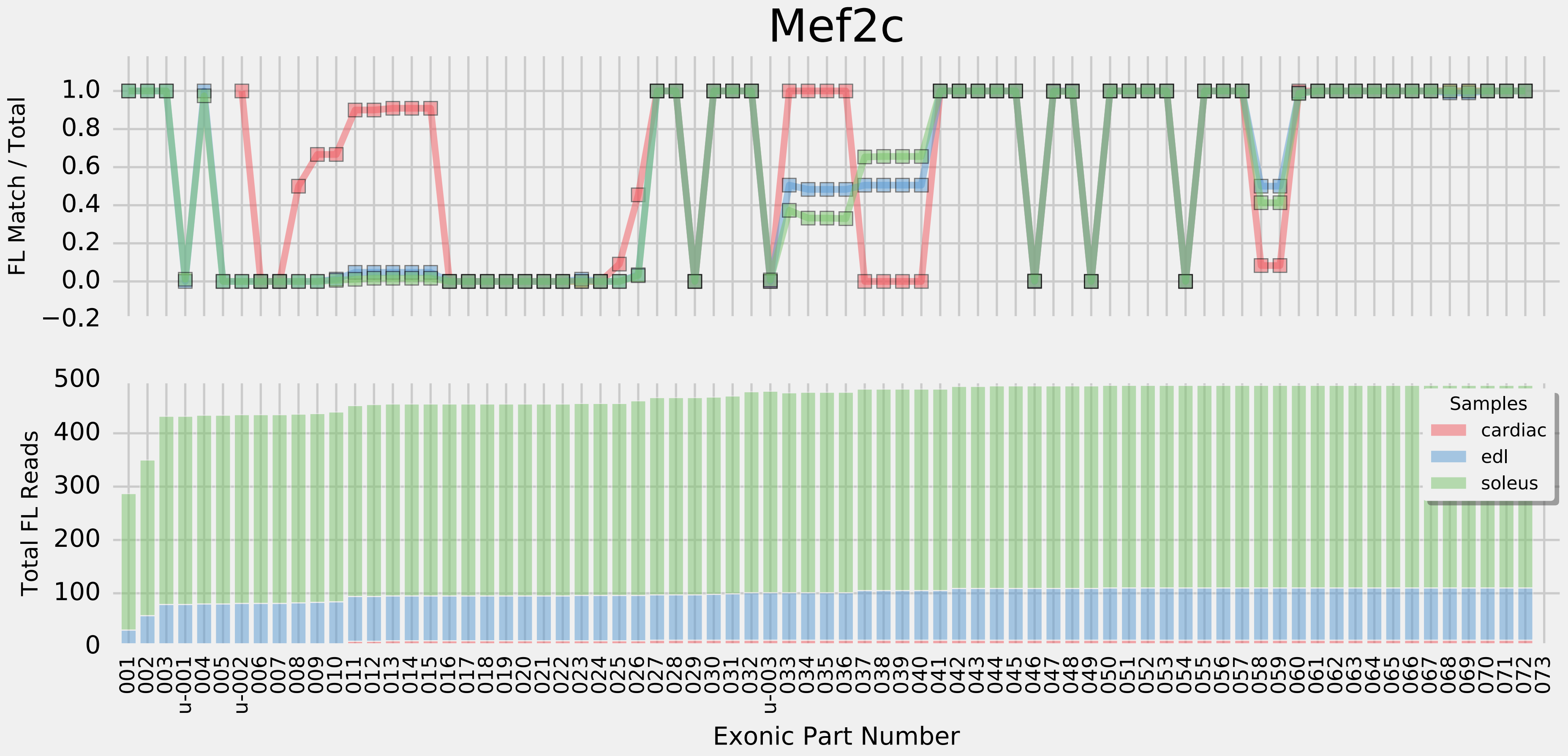

Mef2c
FL Match / Total
1.0
0.8
0.6
0.4
0.2
0.0
−0.2
Total FL Reads
500
400
300
200
100
0
Samples
cardiac
edl
soleus
001
002
003
u-001
004
005
u-002
006
007
008
009
010
011
012
013
014
015
016
017
018
019
020
021
022
023
024
025
026
027
028
029
030
031
032
u-003
033
034
035
036
037
038
039
040
041
042
043
044
045
046
047
048
049
050
051
052
053
054
055
056
057
058
059
060
061
062
063
064
065
066
067
068
069
070
071
072
073
Exonic Part Number

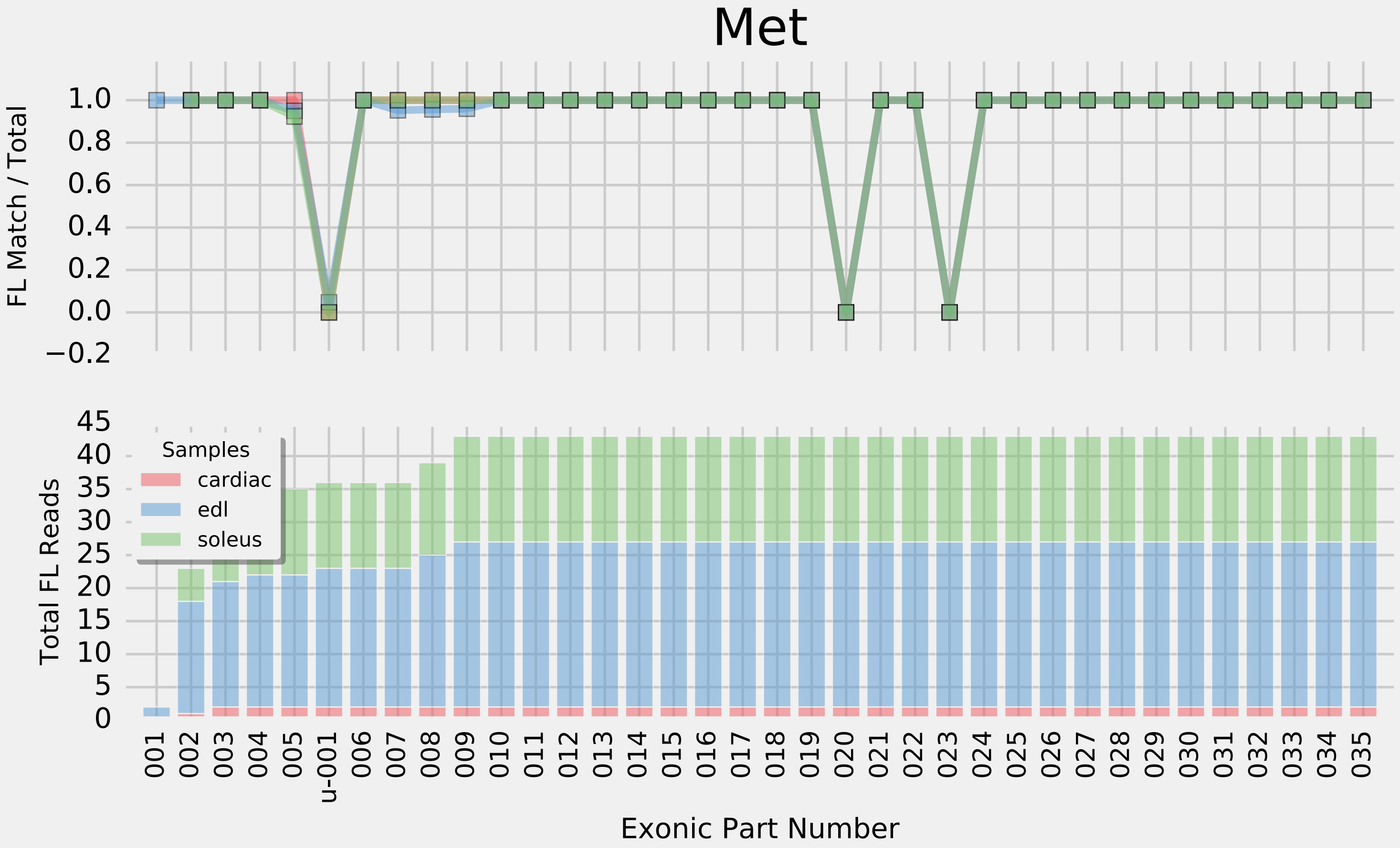

Met
FL Match / Total
1.0
0.8
0.6
0.4
0.2
0.0
−0.2
Total FL Reads
45
40
35
30
25
20
15
10
5
0
Samples
cardiac
edl
soleus
001 002 003 004 005 u-001 006 007 008 009 010 011 012 013 014 015 016 017 018 019 020 021 022 023 024 025 026 027 028 029 030 031 032 033 034 035
Exonic Part Number

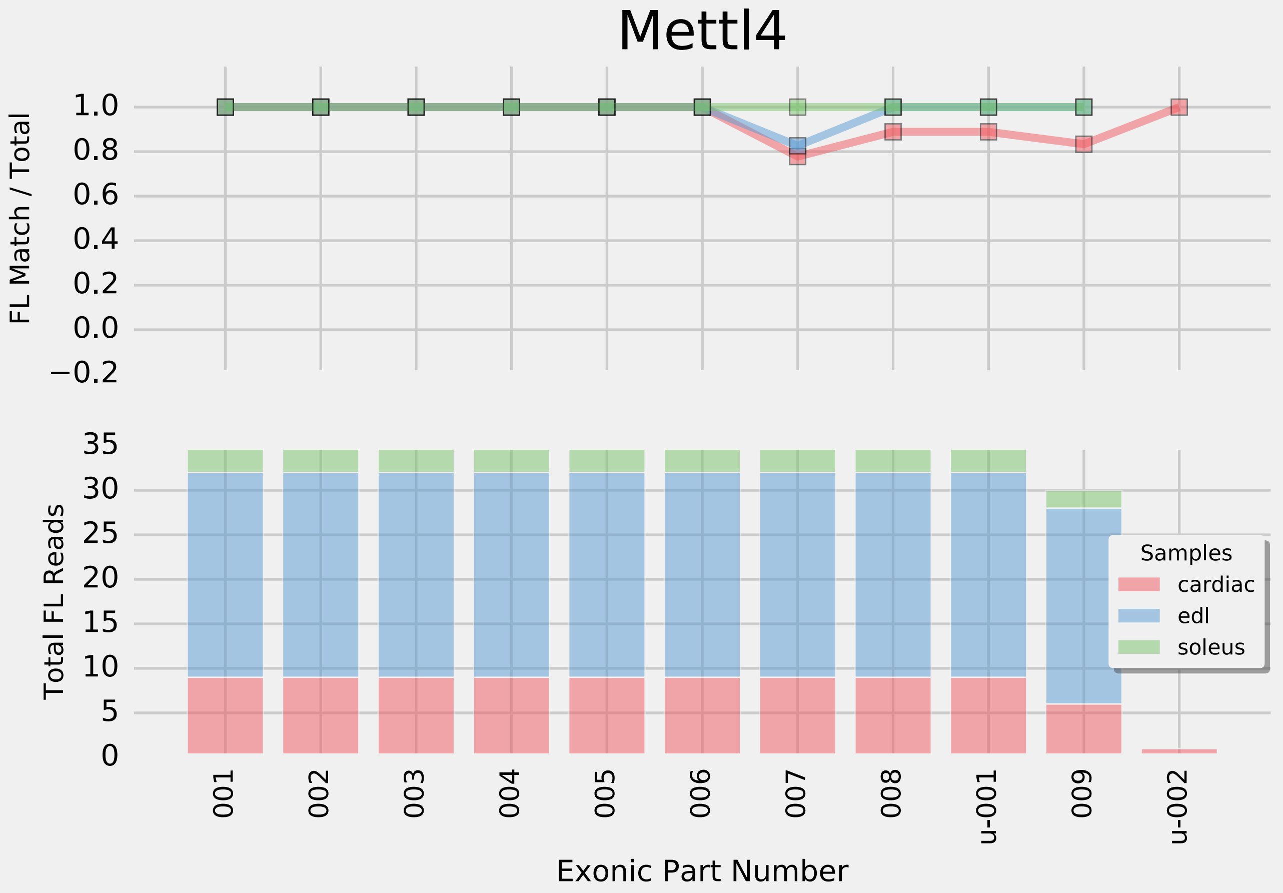

Mettl4
FL Match / Total
1.0
0.8
0.6
0.4
0.2
0.0
−0.2
Total FL Reads
35
30
25
20
15
10
5
0
Samples
cardiac
edl
soleus
001
002
003
004
005
006
007
008
u-001
009
u-002
Exonic Part Number

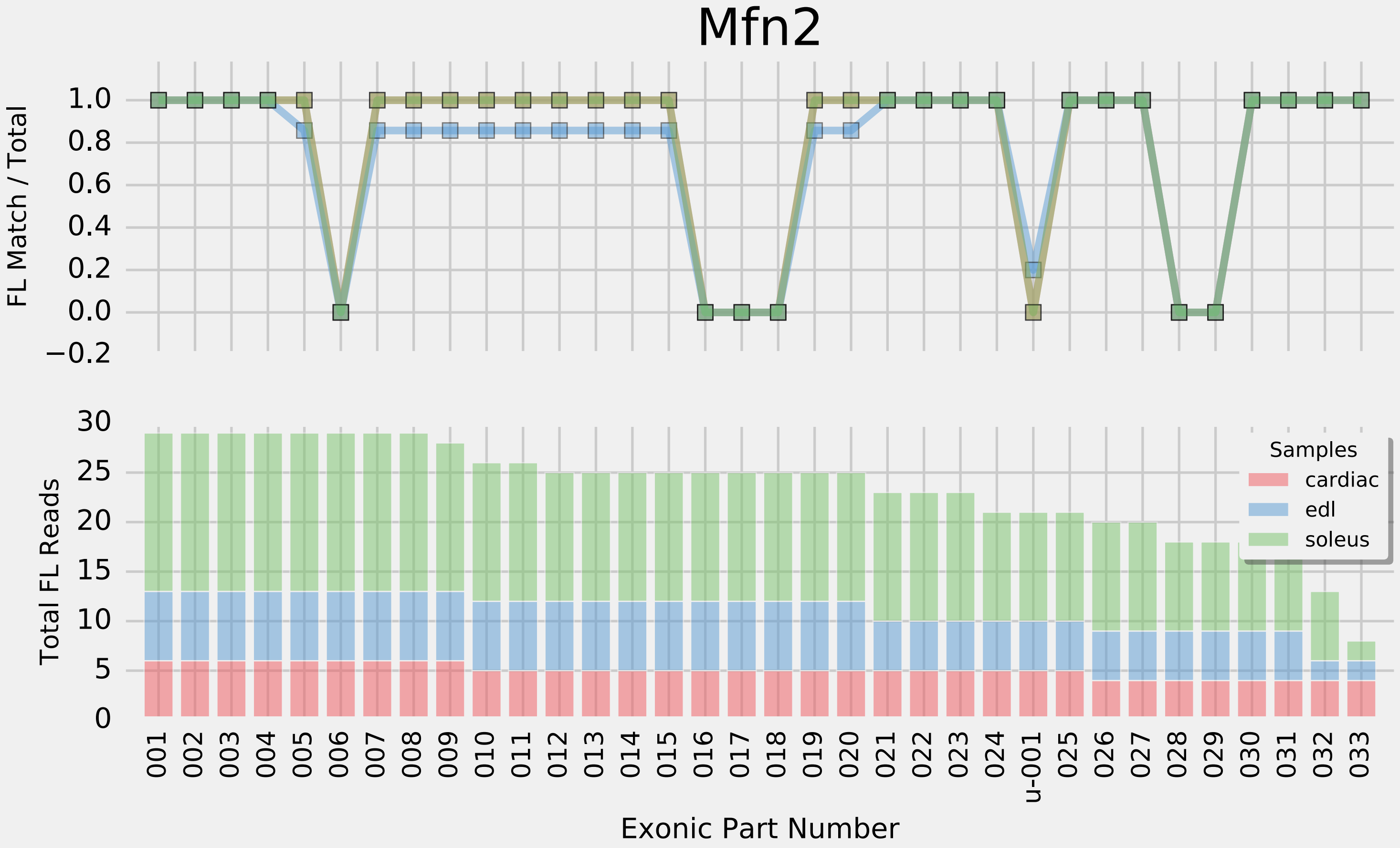

Mfn2
FL Match / Total
1.0
0.8
0.6
0.4
0.2
0.0
−0.2
Total FL Reads
30
25
20
15
10
5
0
Samples
cardiac
edl
soleus
001 002 003 004 005 006 007 008 009 010 011 012 013 014 015 016 017 018 019 020 021 022 023 024 u-001 025 026 027 028 029 030 031 032 033
Exonic Part Number

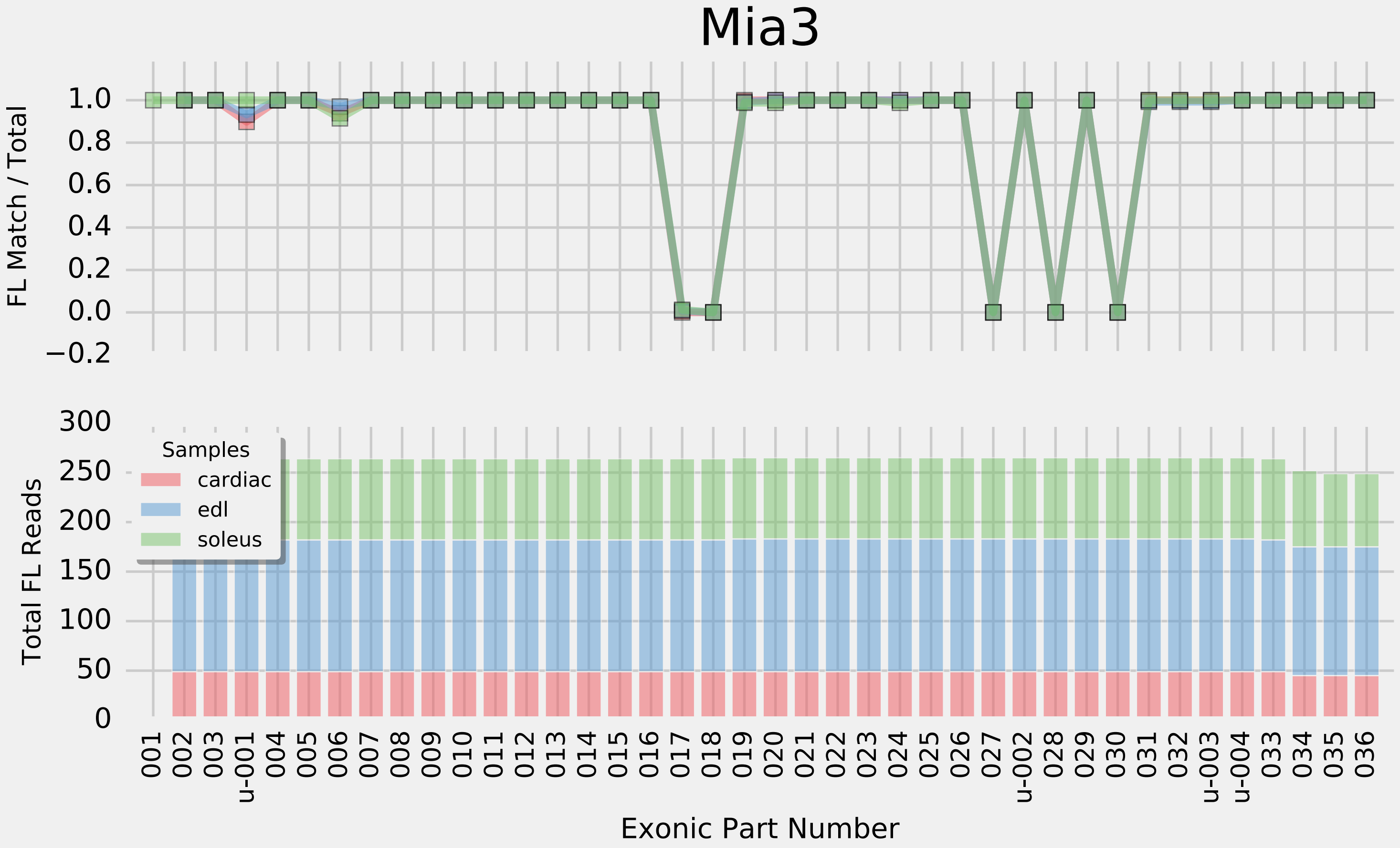

Mia3
FL Match / Total
1.0
0.8
0.6
0.4
0.2
0.0
−0.2
Total FL Reads
300
250
200
150
100
50
0
Samples
cardiac
edl
soleus
001
002
003
u-001
004
005
006
007
008
009
010
011
012
013
014
015
016
017
018
019
020
021
022
023
024
025
026
027
u-002
028
029
030
031
032
u-003
u-004
033
034
035
036
Exonic Part Number

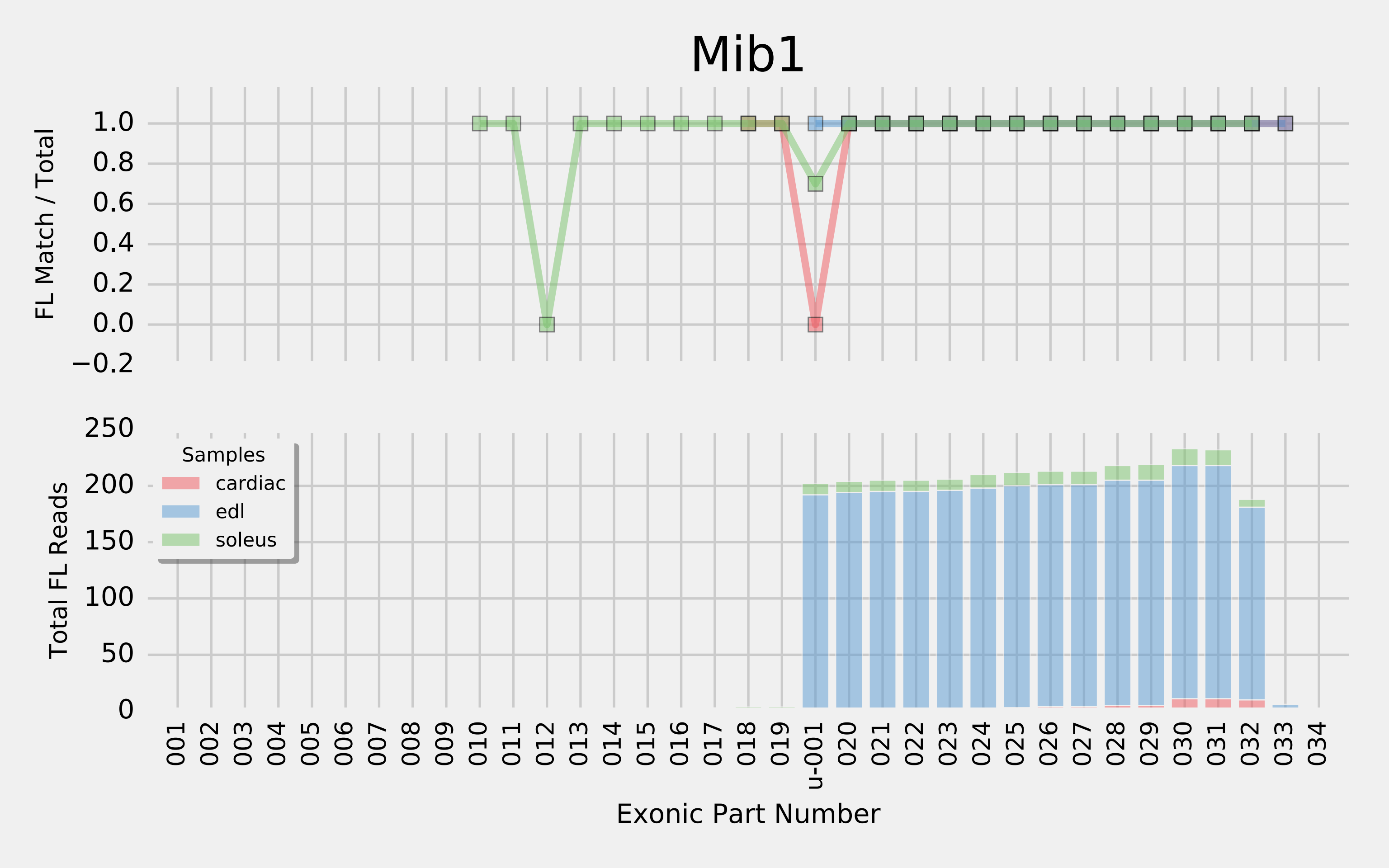

Mib1
FL Match / Total
1.0
0.8
0.6
0.4
0.2
0.0
−0.2
Total FL Reads
250
200
150
100
50
0
Samples
cardiac
edl
soleus
001
002
003
004
005
006
007
008
009
010
011
012
013
014
015
016
017
018
019
u-001
020
021
022
023
024
025
026
027
028
029
030
031
032
033
034
Exonic Part Number

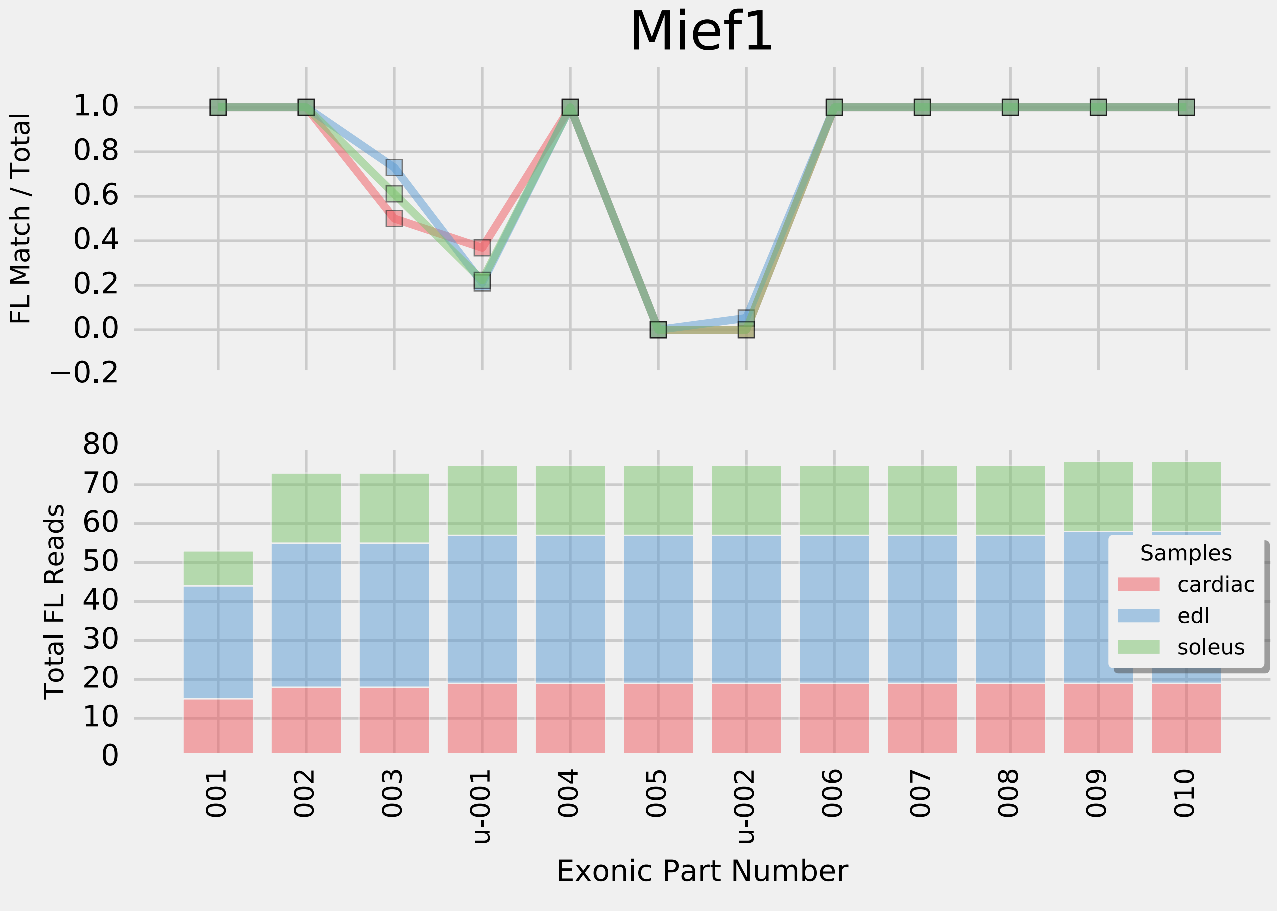

Mief1
FL Match / Total
1.0
0.8
0.6
0.4
0.2
0.0
−0.2
Total FL Reads
80
70
60
50
40
30
20
10
0
Samples
cardiac
edl
soleus
001
002
003
u-001
004
005
u-002
006
007
008
009
010
Exonic Part Number

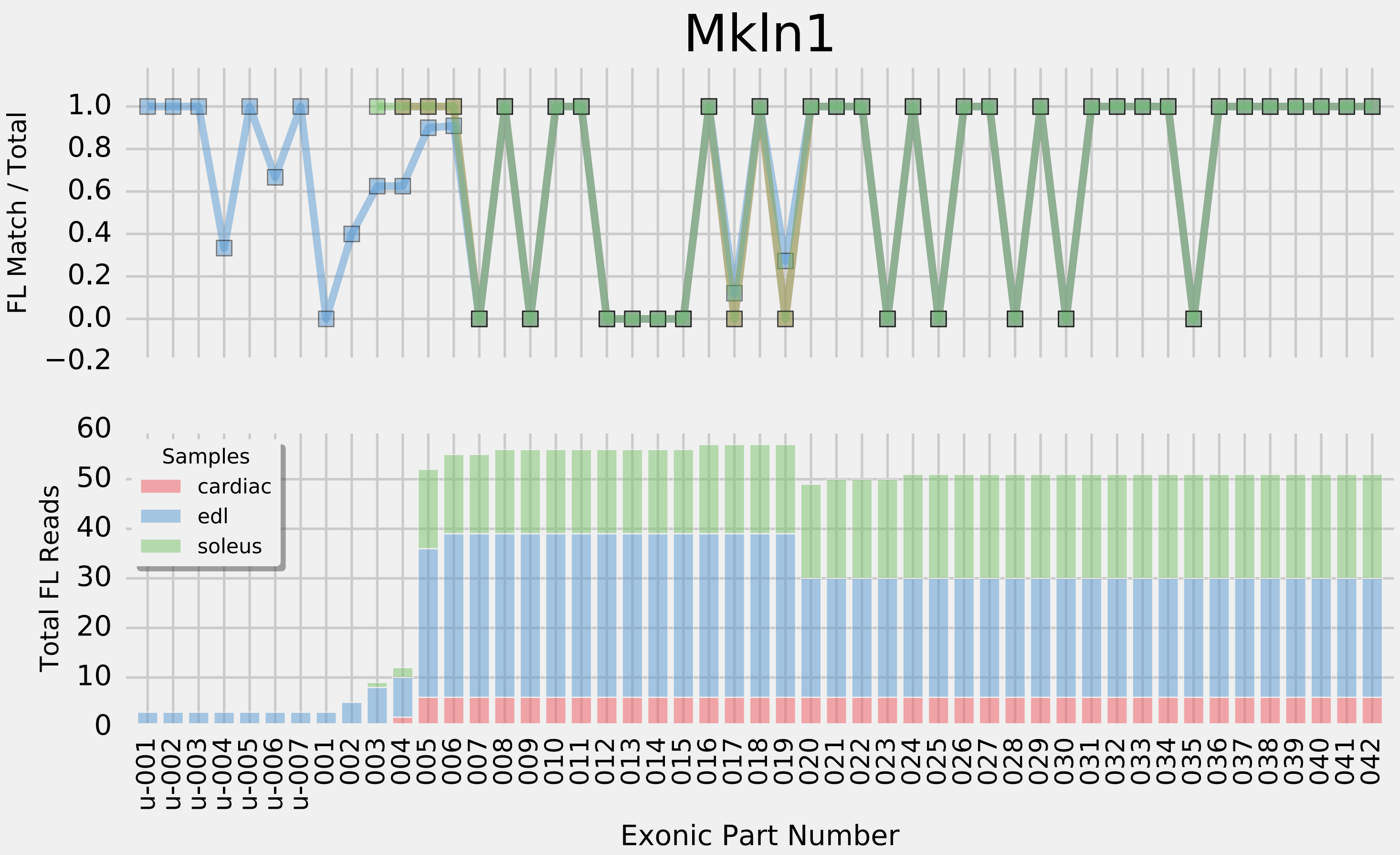

Mkln1
FL Match / Total
1.0
0.8
0.6
0.4
0.2
0.0
−0.2
60
50
40
30
20
10
0
Total FL Reads
Samples
cardiac
edl
soleus
u-001 u-002 u-003 u-004 u-005 u-006 u-007 001 002 003 004 005 006 007 008 009 010 011 012 013 014 015 016 017 018 019 020 021 022 023 024 025 026 027 028 029 030 031 032 033 034 035 036 037 038 039 040 041 042
Exonic Part Number

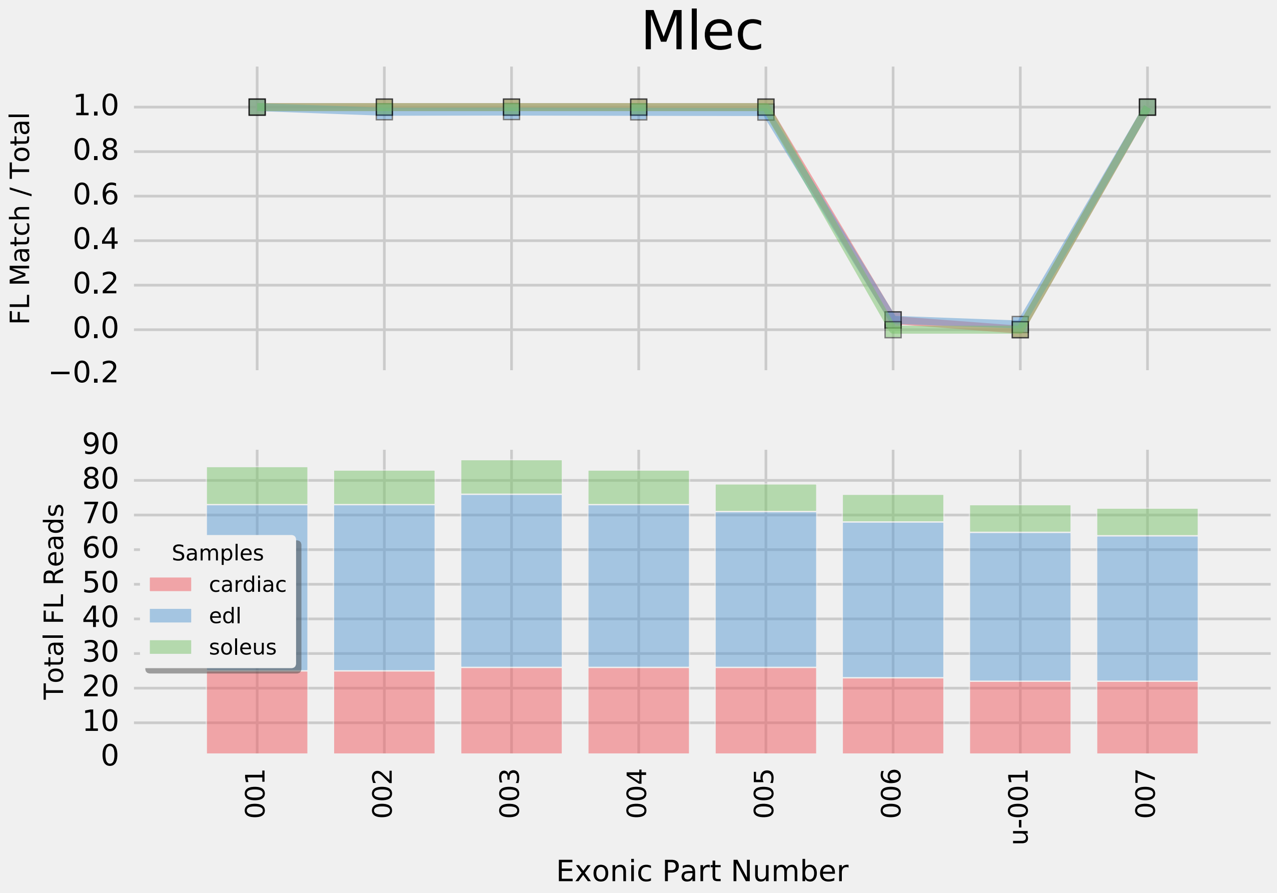

Mlec
FL Match / Total
1.0
0.8
0.6
0.4
0.2
0.0
−0.2
Total FL Reads
90
80
70
60
50
40
30
20
10
0
Samples
cardiac
edl
soleus
001
002
003
004
005
006
u-001
007
Exonic Part Number

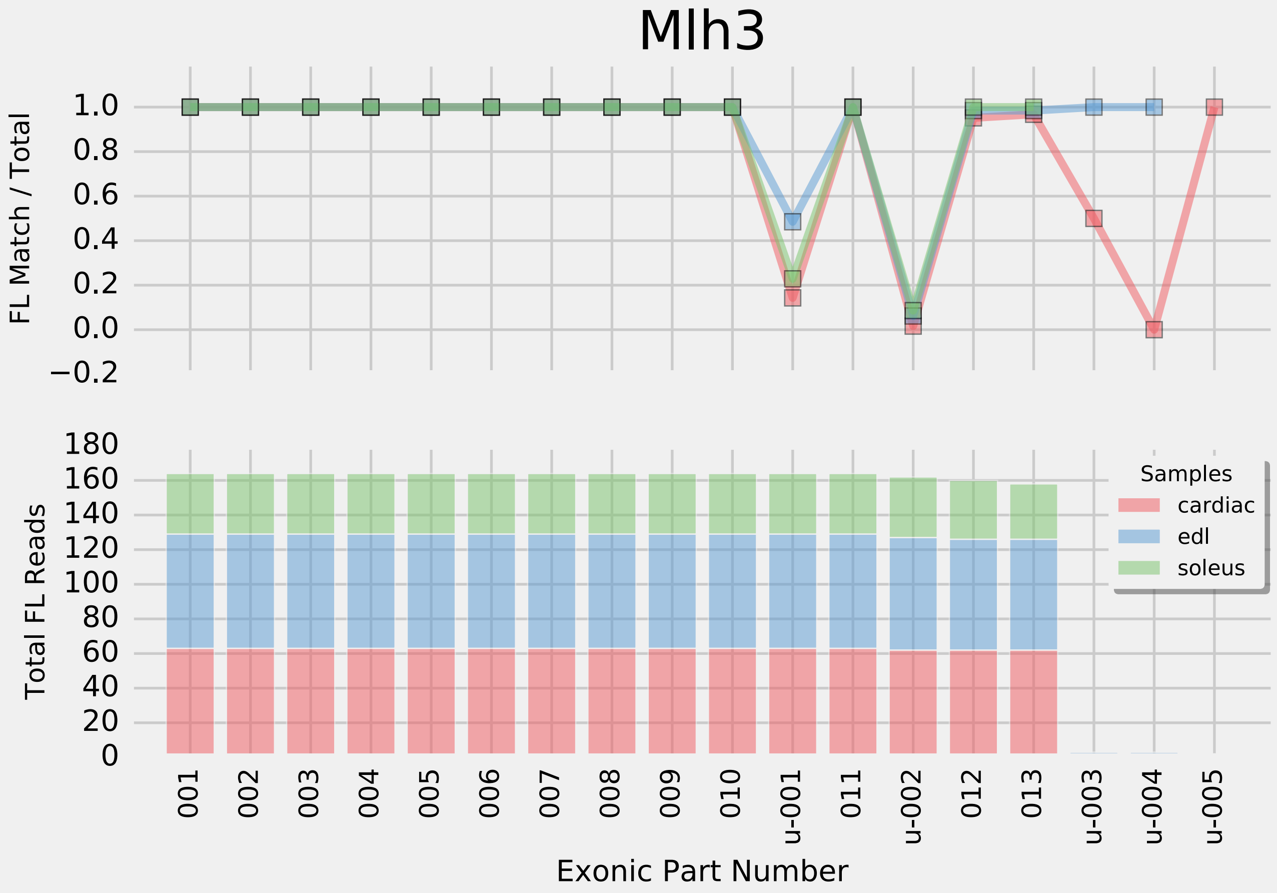
Mlh3
FL Match / Total
1.0
0.8
0.6
0.4
0.2
0.0
−0.2
Total FL Reads
180
160
140
120
100
80
60
40
20
0
Samples
cardiac
edl
soleus
001
002
003
004
005
006
007
008
009
010
u-001
011
u-002
012
013
u-003
u-004
u-005
Exonic Part Number

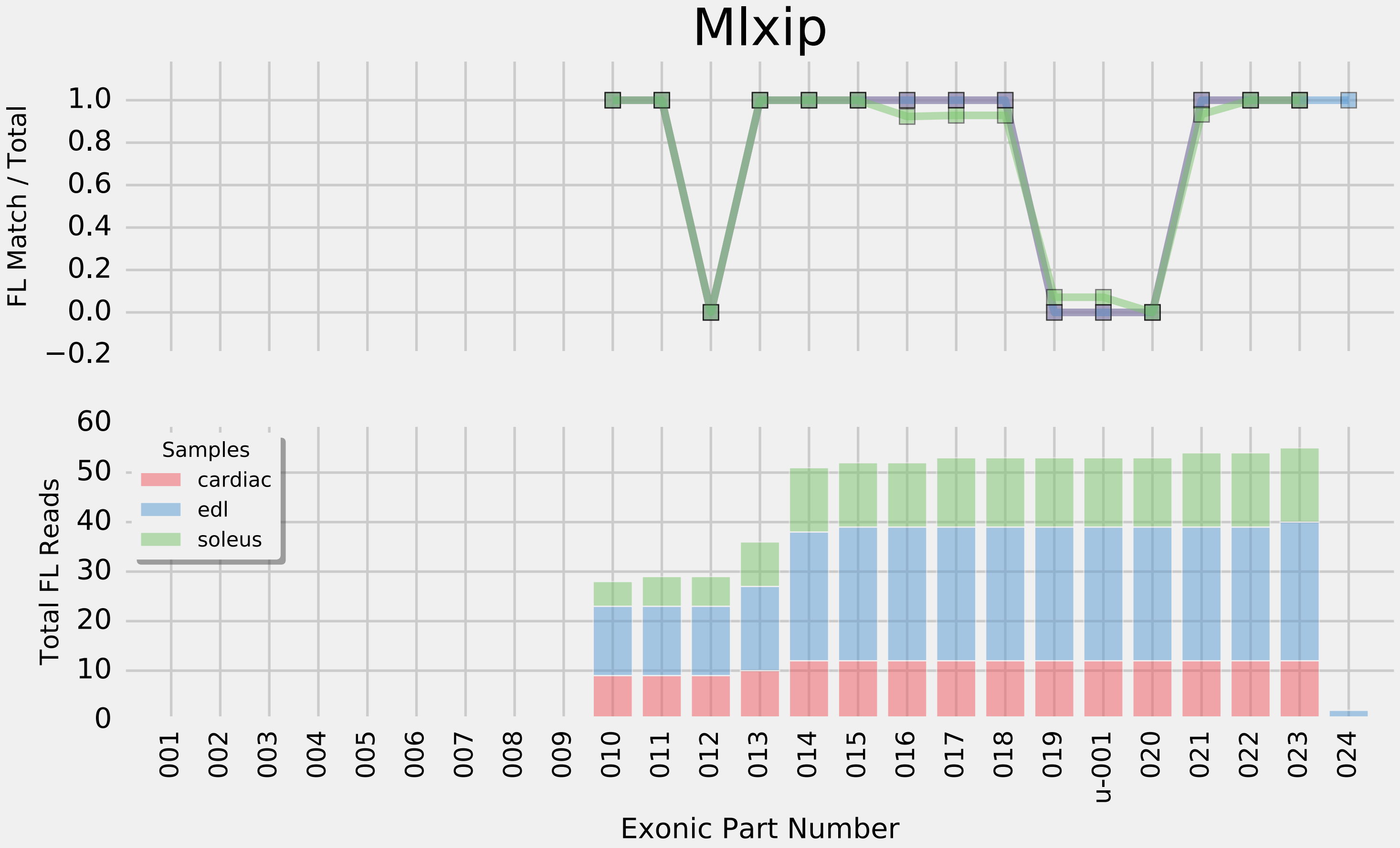

Mlxip
FL Match / Total
1.0
0.8
0.6
0.4
0.2
0.0
−0.2
Total FL Reads
60
50
40
30
20
10
0
Samples
cardiac
edl
soleus
001
002
003
004
005
006
007
008
009
010
011
012
013
014
015
016
017
018
019
u-001
020
021
022
023
024
Exonic Part Number

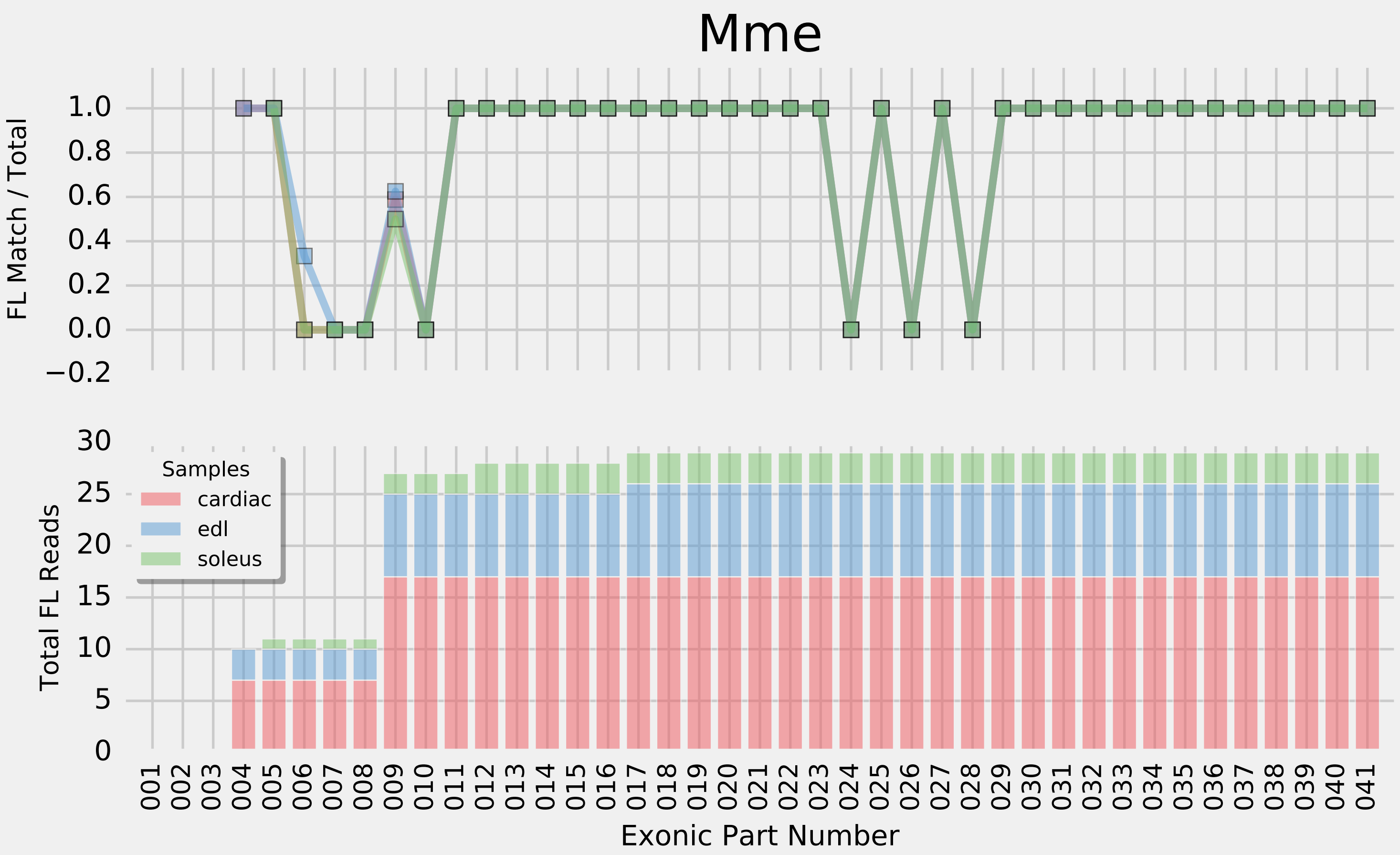

Mme
FL Match / Total
1.0
0.8
0.6
0.4
0.2
0.0
−0.2
Total FL Reads
30
25
20
15
10
5
0
Samples
cardiac
edl
soleus
001 002 003 004 005 006 007 008 009 010 011 012 013 014 015 016 017 018 019 020 021 022 023 024 025 026 027 028 029 030 031 032 033 034 035 036 037 038 039 040 041
Exonic Part Number

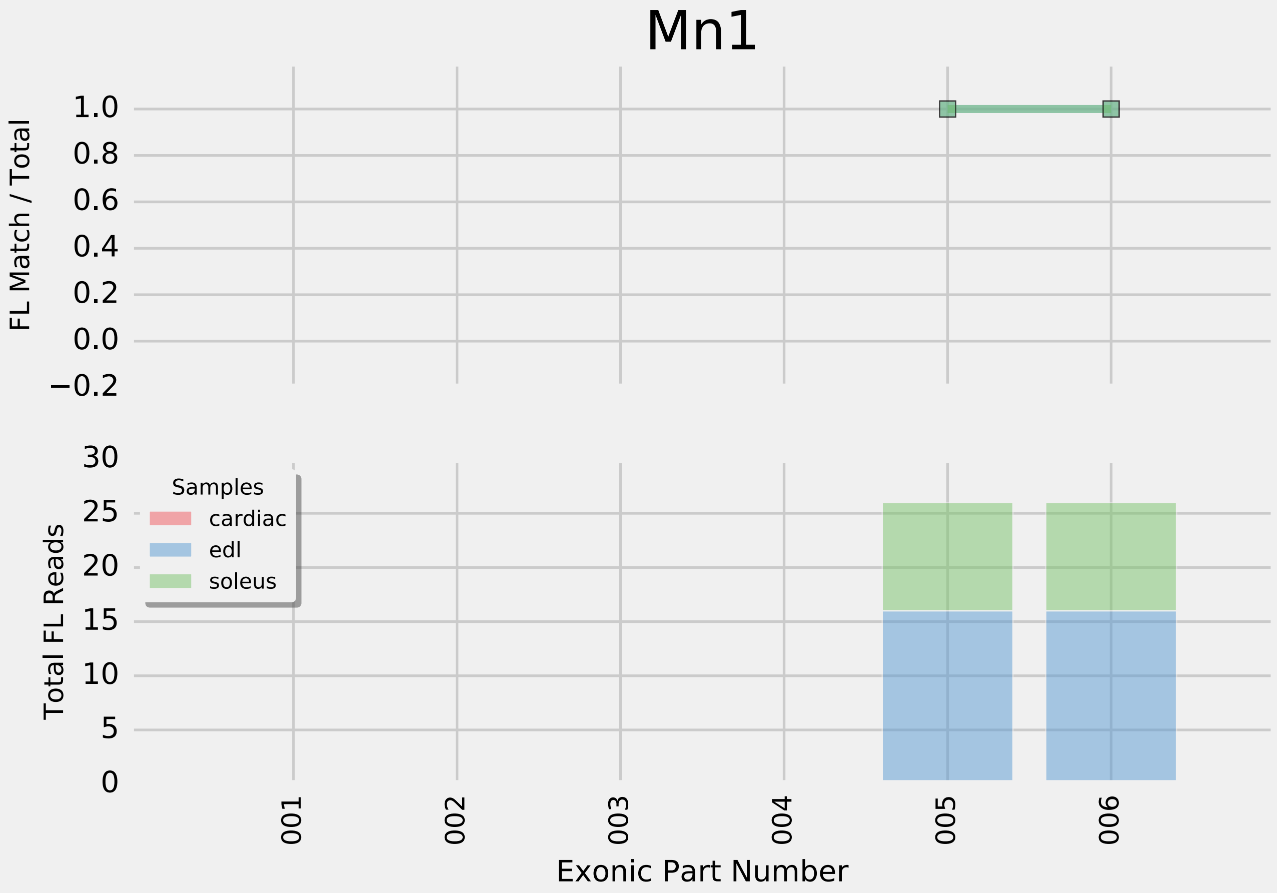
Mn1
FL Match / Total
1.0
0.8
0.6
0.4
0.2
0.0
−0.2
Total FL Reads
30
25
20
15
10
5
0
Samples
cardiac
edl
soleus
001
002
003
004
005
006
Exonic Part Number

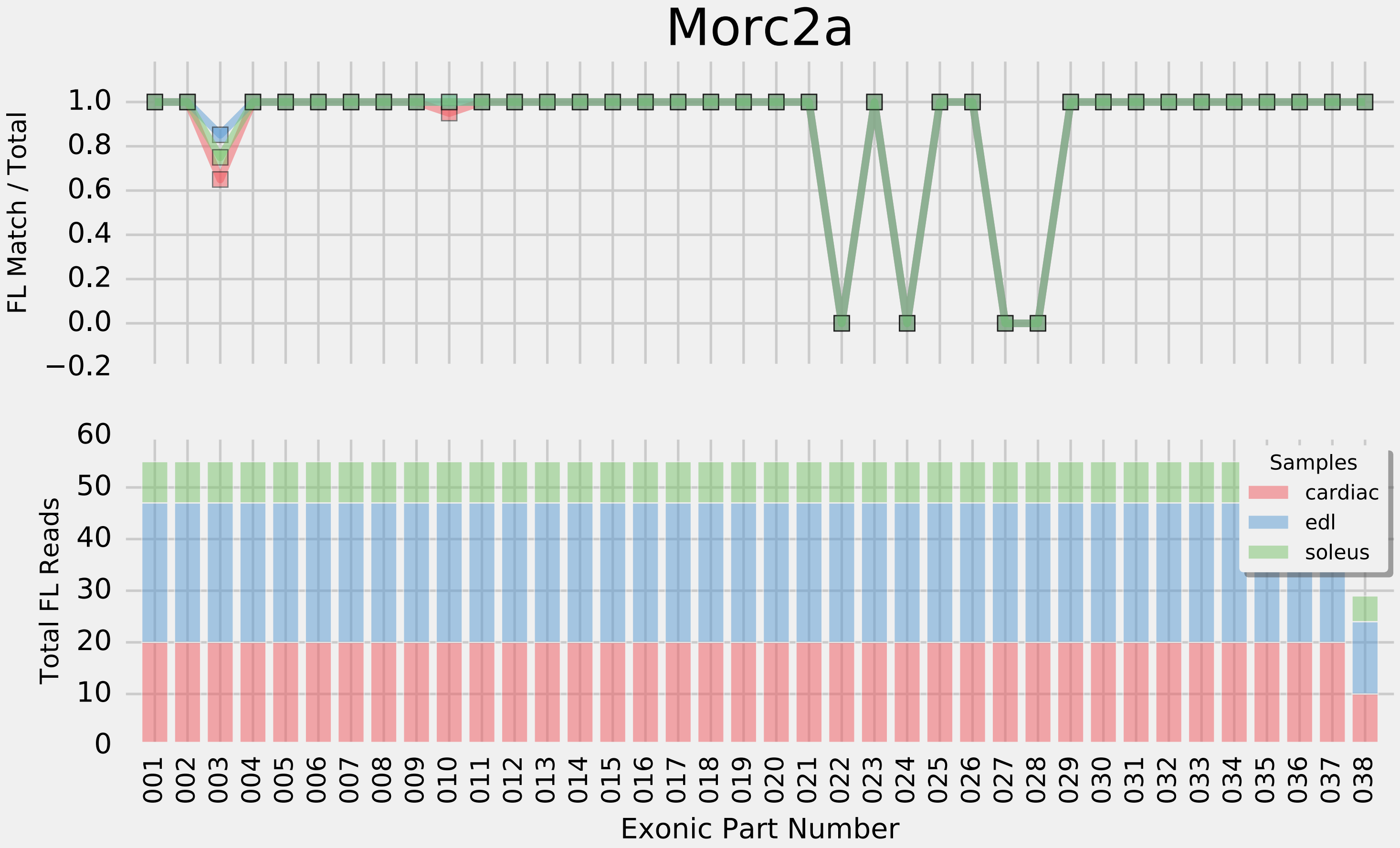

Morc2a
FL Match / Total
1.0
0.8
0.6
0.4
0.2
0.0
−0.2
Total FL Reads
60
50
40
30
20
10
0
Samples
cardiac
edl
soleus
001 002 003 004 005 006 007 008 009 010 011 012 013 014 015 016 017 018 019 020 021 022 023 024 025 026 027 028 029 030 031 032 033 034 035 036 037 038
Exonic Part Number

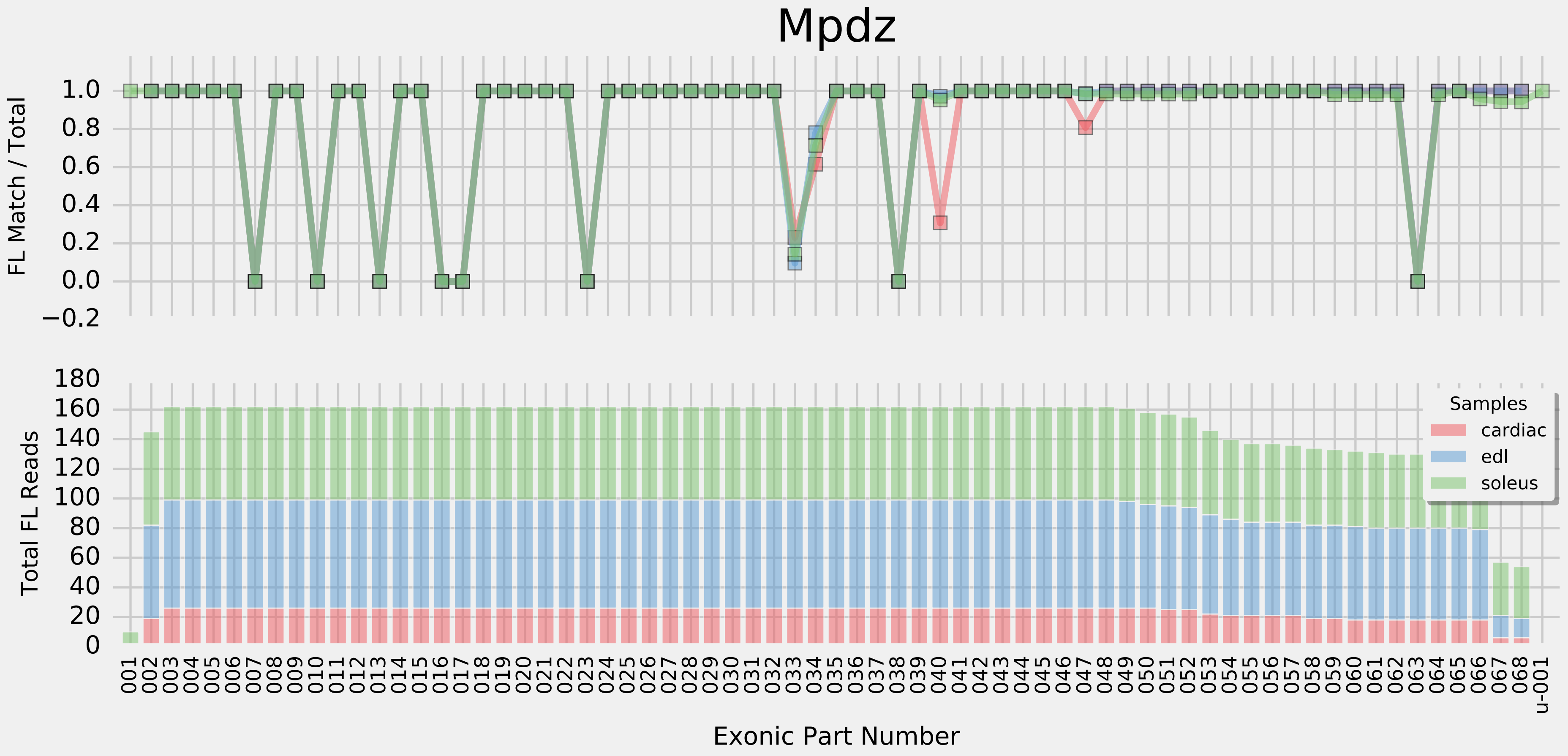

Mpdz
FL Match / Total
Total FL Reads
Samples
cardiac
edl
soleus
Exonic Part Number

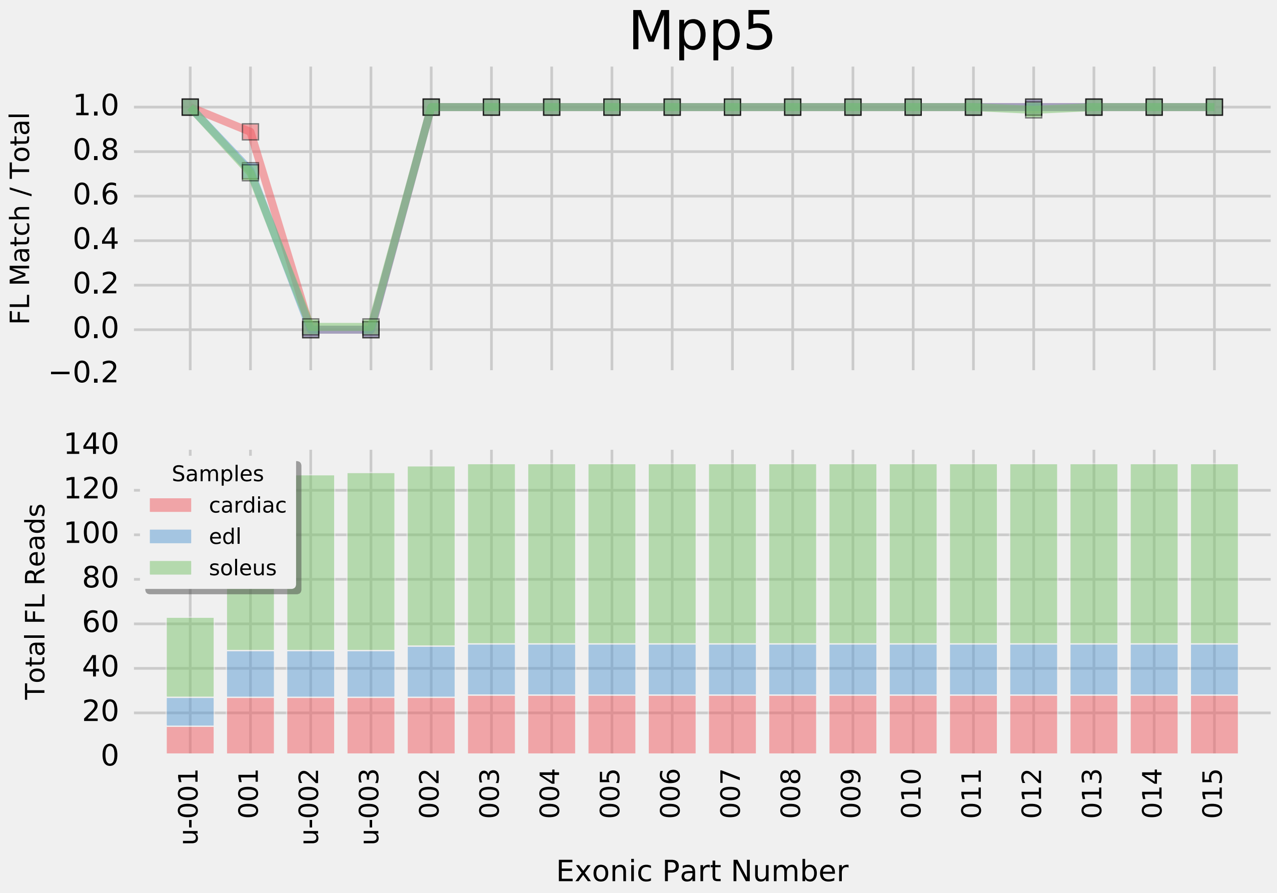

Mpp5
FL Match / Total
1.0
0.8
0.6
0.4
0.2
0.0
−0.2
Total FL Reads
140
120
100
80
60
40
20
0
Samples
cardiac
edl
soleus
u-001
001
u-002
u-003
002
003
004
005
006
007
008
009
010
011
012
013
014
015
Exonic Part Number

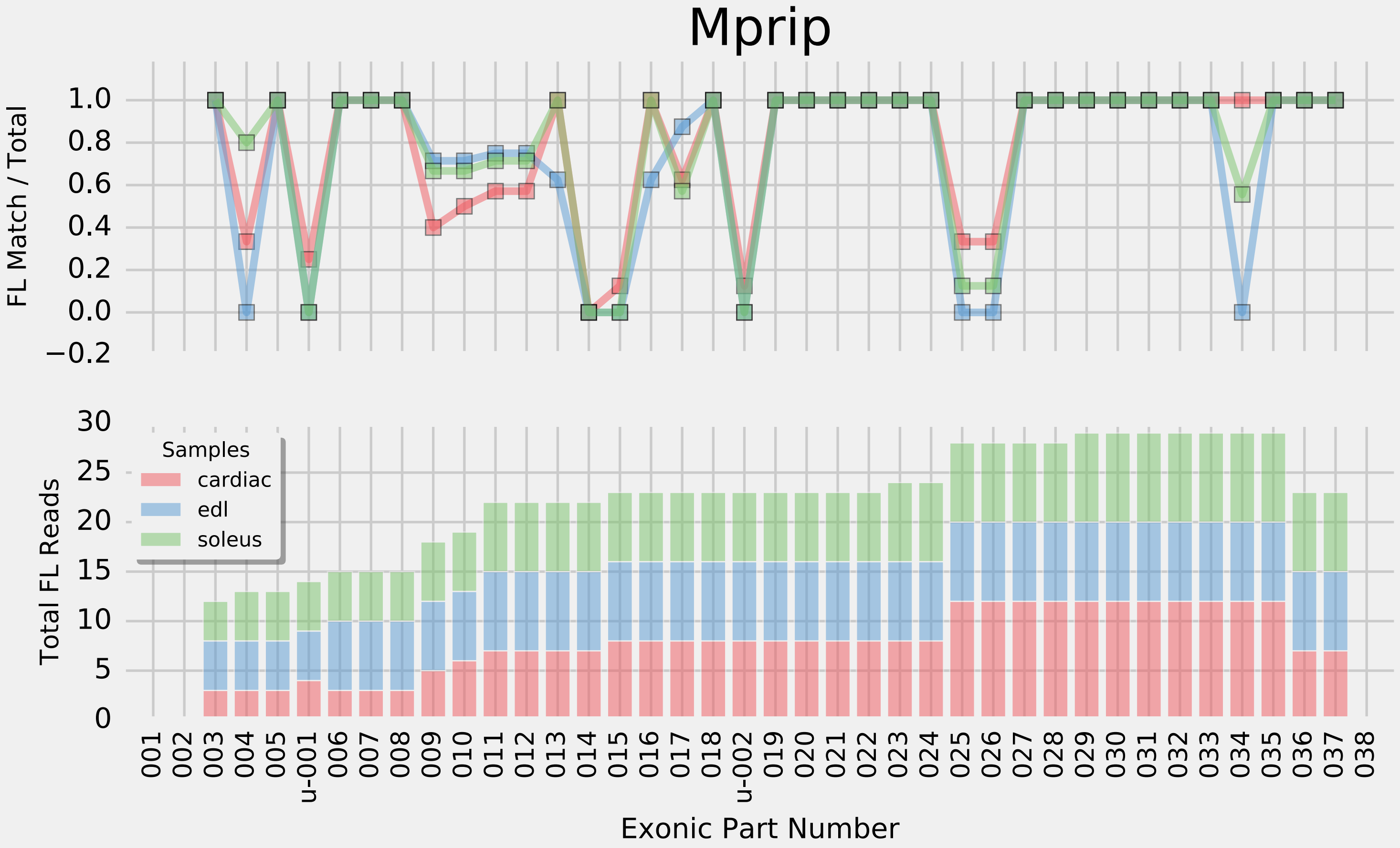
Mprip
FL Match / Total
1.0
0.8
0.6
0.4
0.2
0.0
−0.2
Total FL Reads
30
25
20
15
10
5
0
Samples
cardiac
edl
soleus
001
002
003
004
005
u-001
006
007
008
009
010
011
012
013
014
015
016
017
018
u-002
019
020
021
022
023
024
025
026
027
028
029
030
031
032
033
034
035
036
037
038
Exonic Part Number

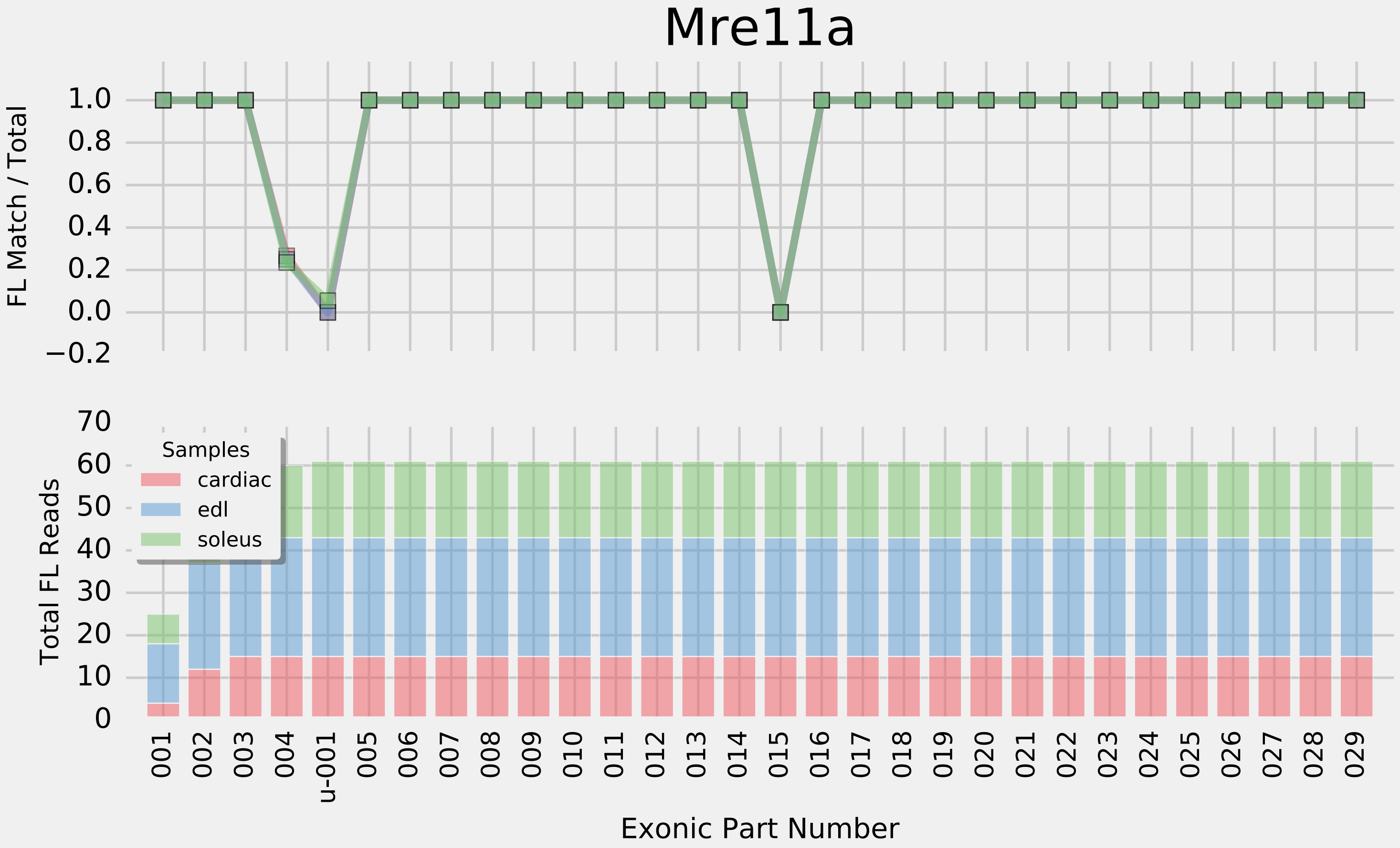

Mre11a
FL Match / Total
1.0
0.8
0.6
0.4
0.2
0.0
−0.2
70
60
50
40
30
20
10
0
Total FL Reads
Samples
cardiac
edl
soleus
001
002
003
004
u-001
005
006
007
008
009
010
011
012
013
014
015
016
017
018
019
020
021
022
023
024
025
026
027
028
029
Exonic Part Number

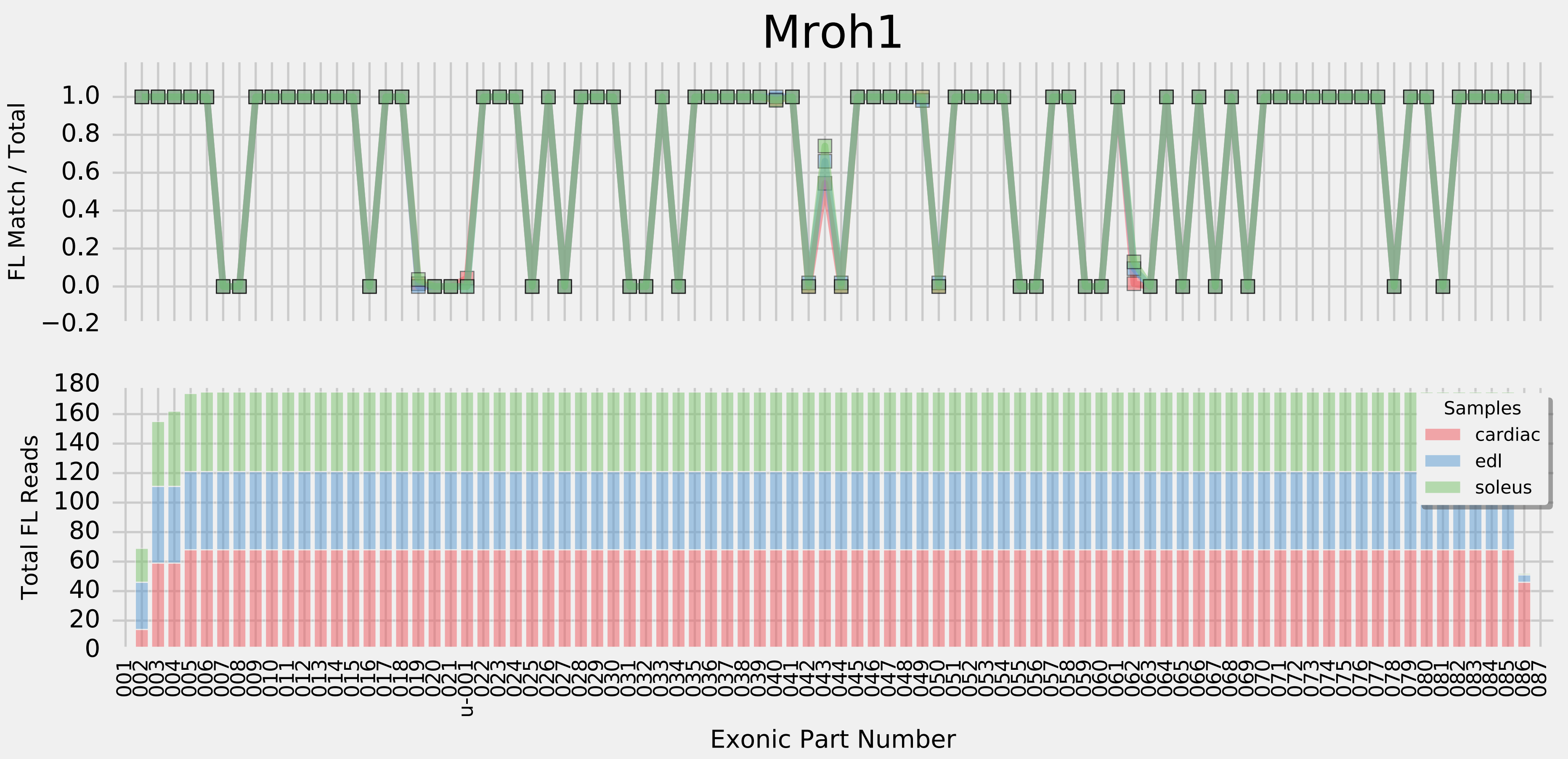

Mroh1
FL Match / Total
Total FL Reads
Samples
cardiac
edl
soleus
Exonic Part Number

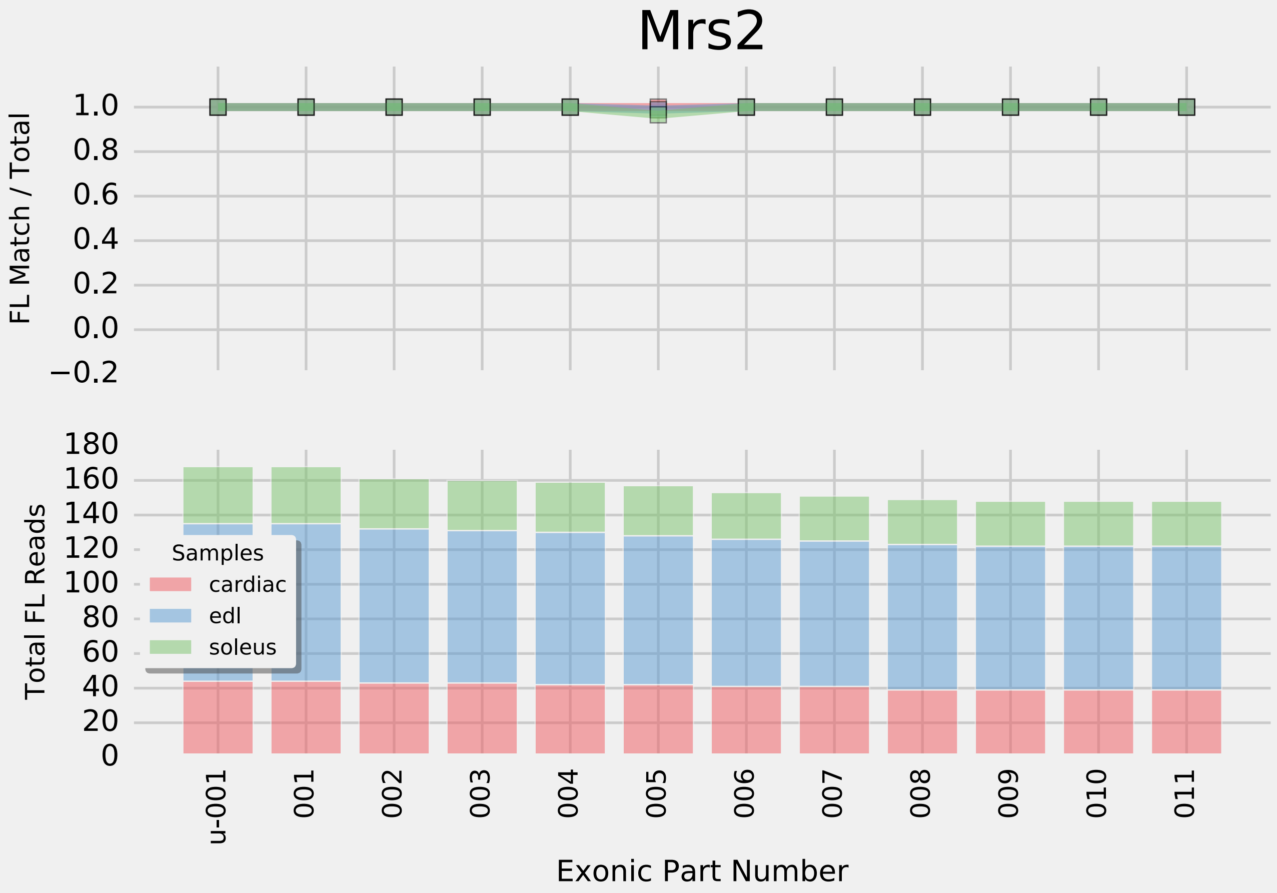

Mrs2
FL Match / Total
1.0
0.8
0.6
0.4
0.2
0.0
−0.2
Total FL Reads
180
160
140
120
100
80
60
40
20
0
Samples
cardiac
edl
soleus
u-001
001
002
003
004
005
006
007
008
009
010
011
Exonic Part Number

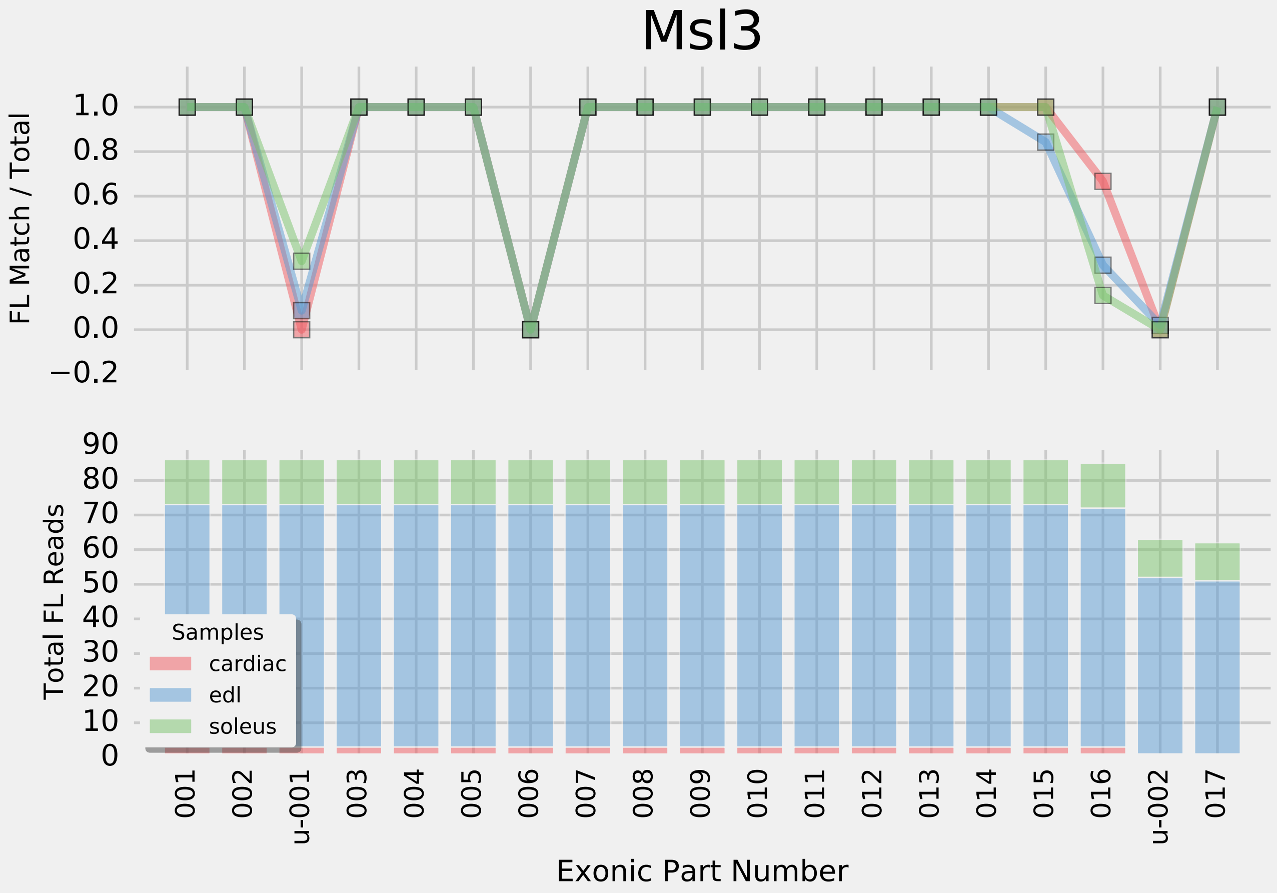
Msl3
FL Match / Total
1.0
0.8
0.6
0.4
0.2
0.0
−0.2
Total FL Reads
90
80
70
60
50
40
30
20
10
0
Samples
cardiac
edl
soleus
001
002
u-001
003
004
005
006
007
008
009
010
011
012
013
014
015
016
u-002
017
Exonic Part Number

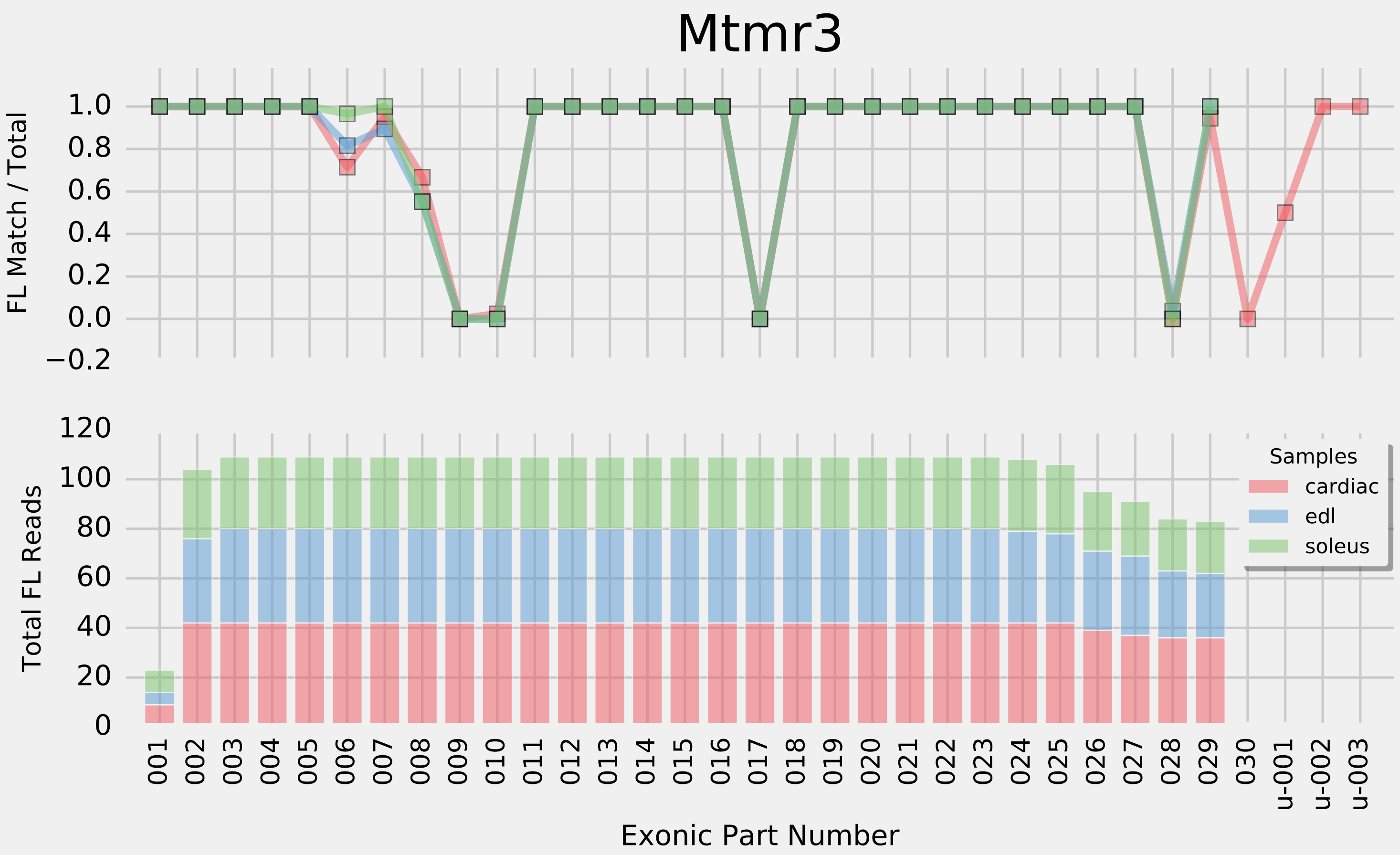
Mtmr3
FL Match / Total
1.0
0.8
0.6
0.4
0.2
0.0
−0.2
Total FL Reads
120
100
80
60
40
20
0
Samples
cardiac
edl
soleus
001 002 003 004 005 006 007 008 009 010 011 012 013 014 015 016 017 018 019 020 021 022 023 024 025 026 027 028 029 030 u-001 u-002 u-003
Exonic Part Number

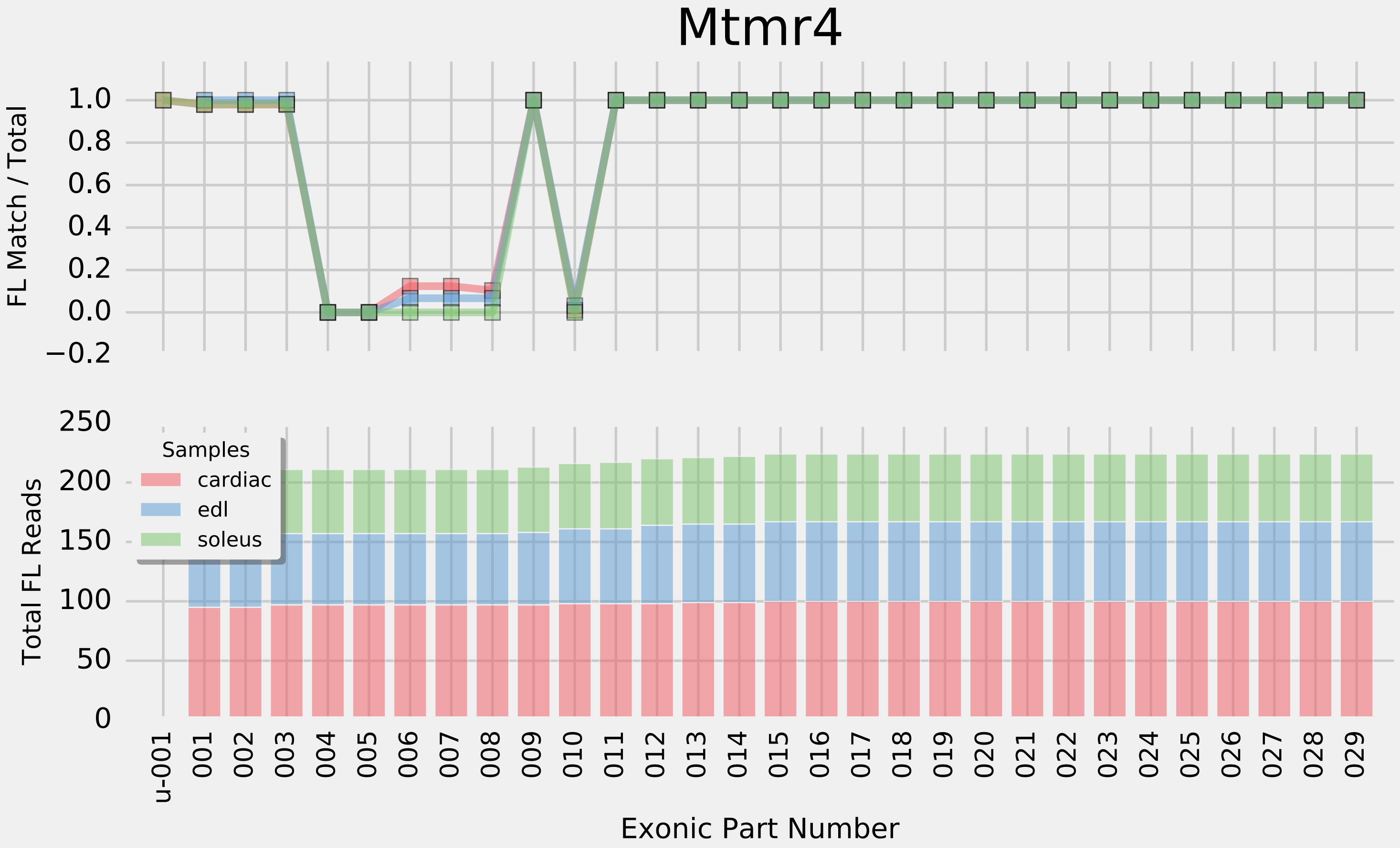

Mtmr4
FL Match / Total
1.0
0.8
0.6
0.4
0.2
0.0
−0.2
Total FL Reads
250
200
150
100
50
0
Samples
cardiac
edl
soleus
u-001
001
002
003
004
005
006
007
008
009
010
011
012
013
014
015
016
017
018
019
020
021
022
023
024
025
026
027
028
029
Exonic Part Number

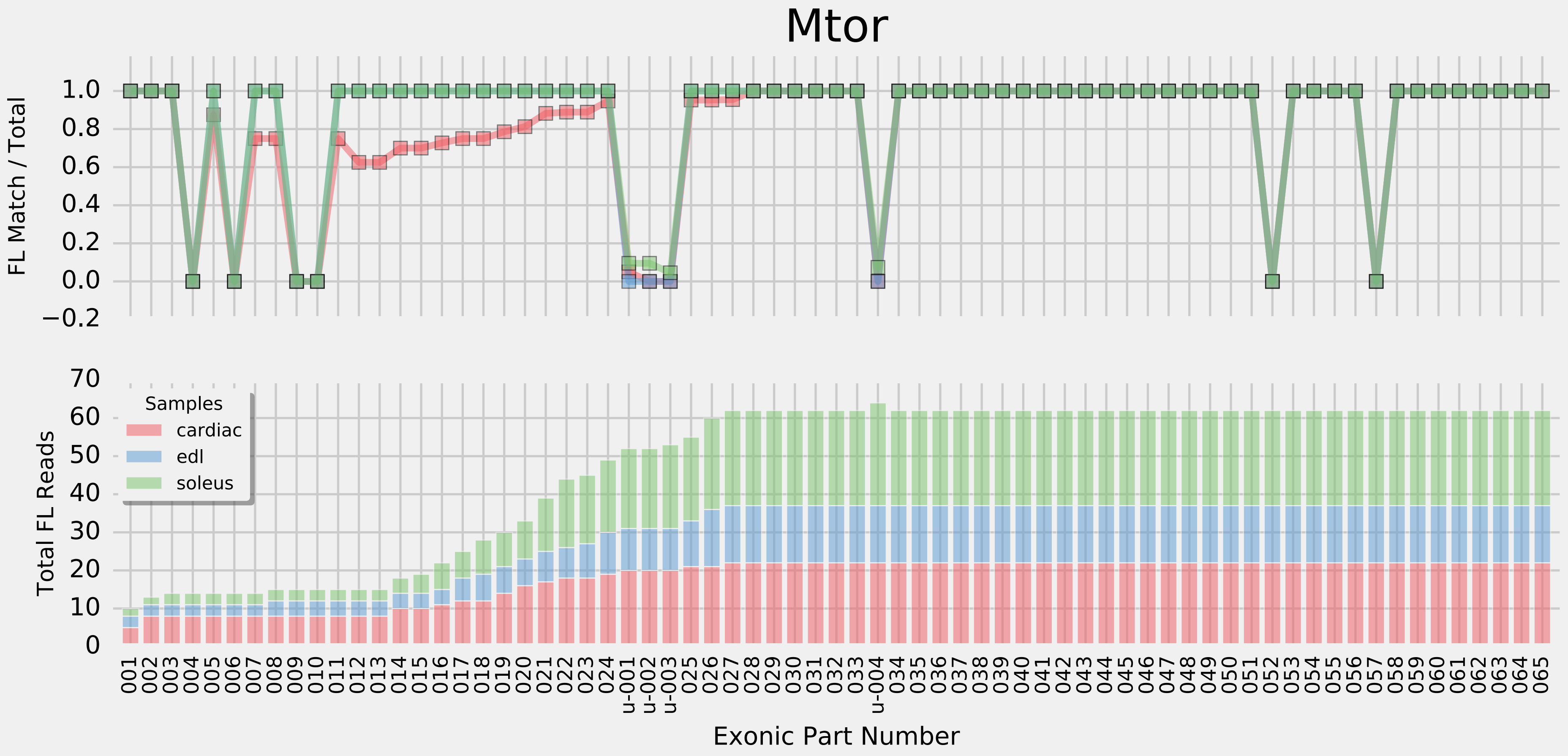

Mtor
FL Match / Total
1.0
0.8
0.6
0.4
0.2
0.0
−0.2
Total FL Reads
70
60
50
40
30
20
10
0
Samples
cardiac
edl
soleus
001 002 003 004 005 006 007 008 009 010 011 012 013 014 015 016 017 018 019 020 021 022 023 024 u-001 u-002 u-003 025 026 027 028 029 030 031 032 033 u-004 034 035 036 037 038 039 040 041 042 043 044 045 046 047 048 049 050 051 052 053 054 055 056 057 058 059 060 061 062 063 064 065
Exonic Part Number

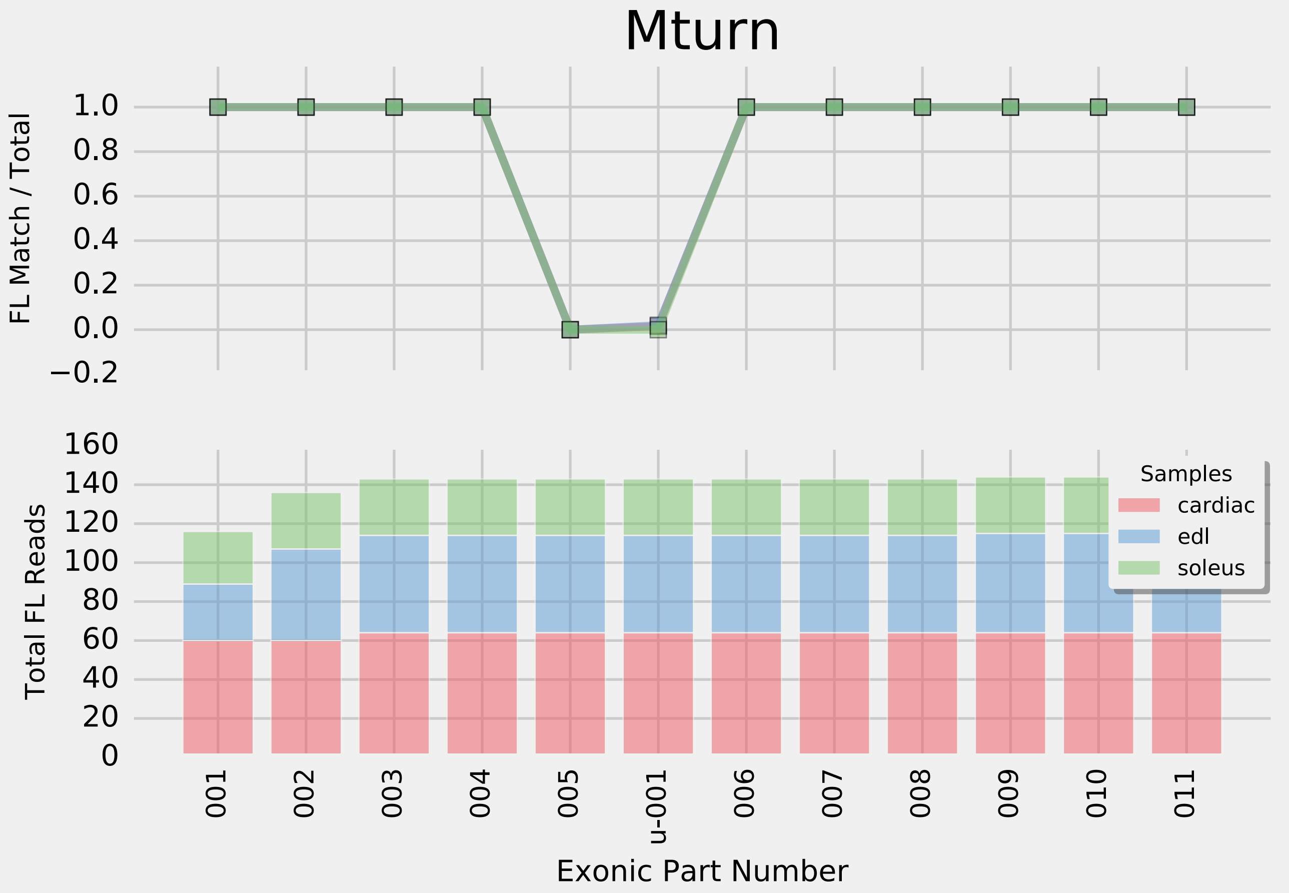

Mturn
FL Match / Total
1.0
0.8
0.6
0.4
0.2
0.0
−0.2
Total FL Reads
160
140
120
100
80
60
40
20
0
Samples
cardiac
edl
soleus
001
002
003
004
005
u-001
006
007
008
009
010
011
Exonic Part Number

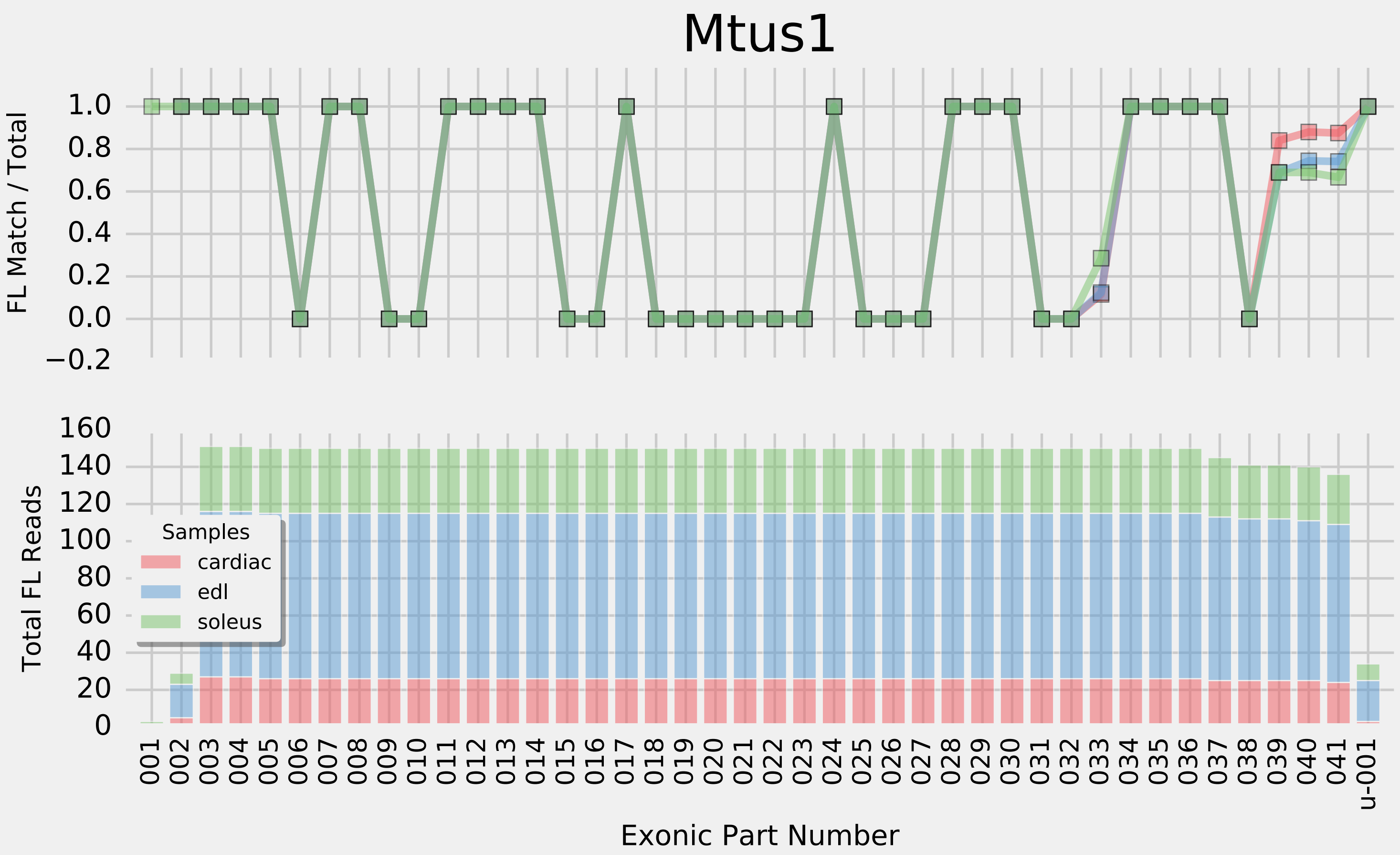

Mtus1
FL Match / Total
1.0
0.8
0.6
0.4
0.2
0.0
−0.2
Total FL Reads
160
140
120
100
80
60
40
20
0
Samples
cardiac
edl
soleus
001 002 003 004 005 006 007 008 009 010 011 012 013 014 015 016 017 018 019 020 021 022 023 024 025 026 027 028 029 030 031 032 033 034 035 036 037 038 039 040 041 u-001
Exonic Part Number

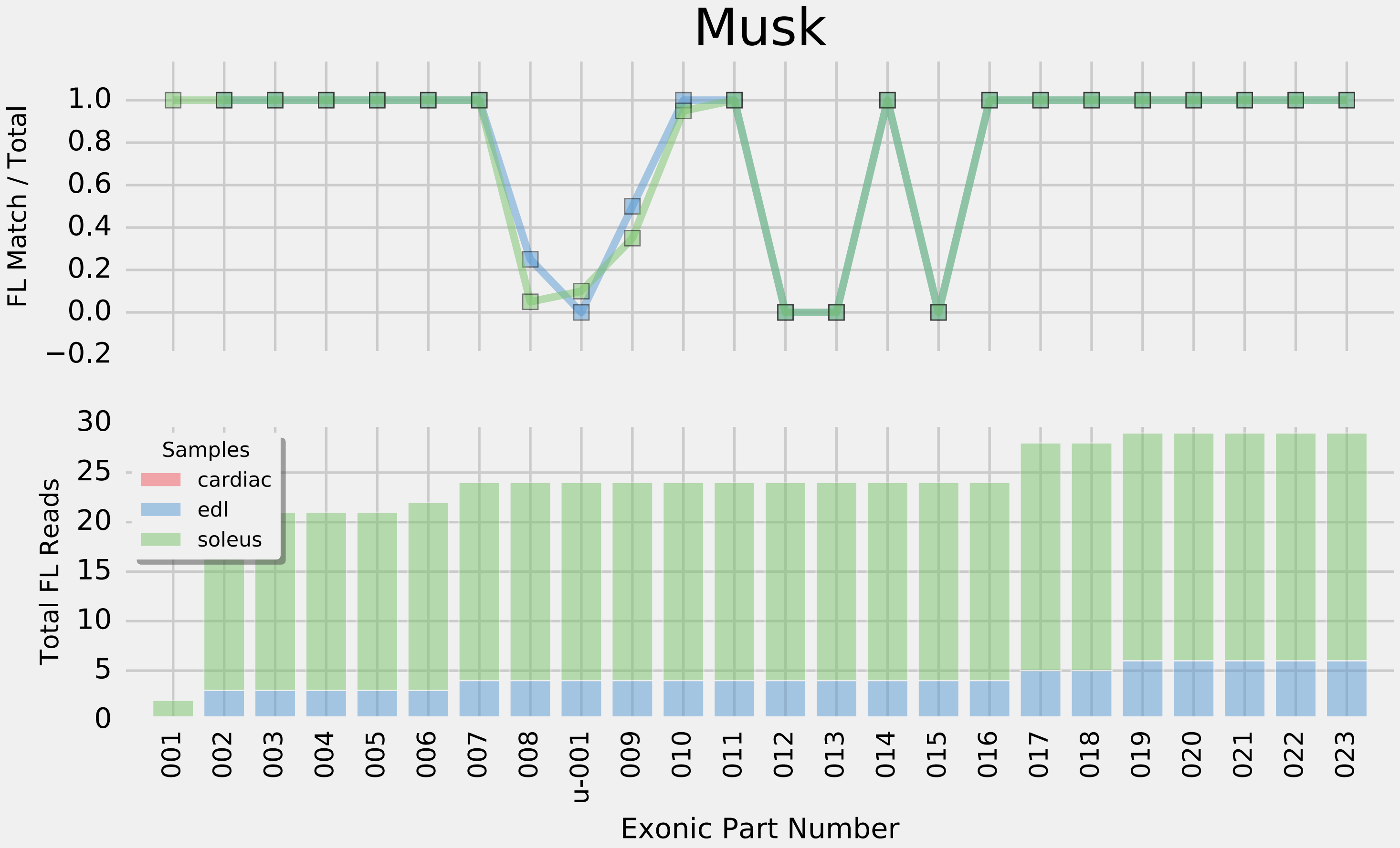
Musk
FL Match / Total
1.0
0.8
0.6
0.4
0.2
0.0
−0.2
Total FL Reads
30
25
20
15
10
5
0
Samples
cardiac
edl
soleus
001
002
003
004
005
006
007
008
u-001
009
010
011
012
013
014
015
016
017
018
019
020
021
022
023
Exonic Part Number

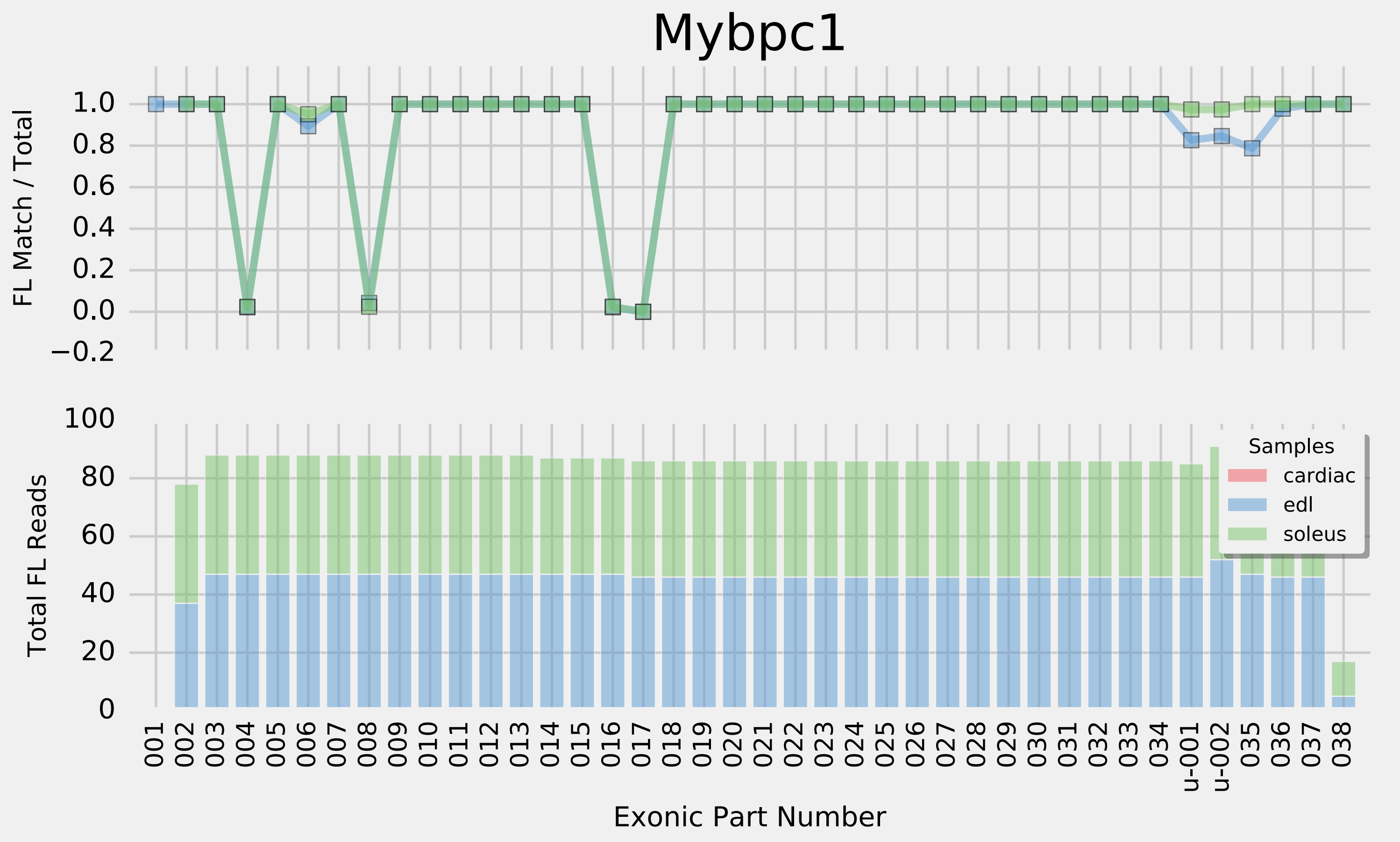

Mybpc1
FL Match / Total
1.0
0.8
0.6
0.4
0.2
0.0
−0.2
Total FL Reads
100
80
60
40
20
0
Samples
cardiac
edl
soleus
001
002
003
004
005
006
007
008
009
010
011
012
013
014
015
016
017
018
019
020
021
022
023
024
025
026
027
028
029
030
031
032
033
034
u-001
u-002
035
036
037
038
Exonic Part Number

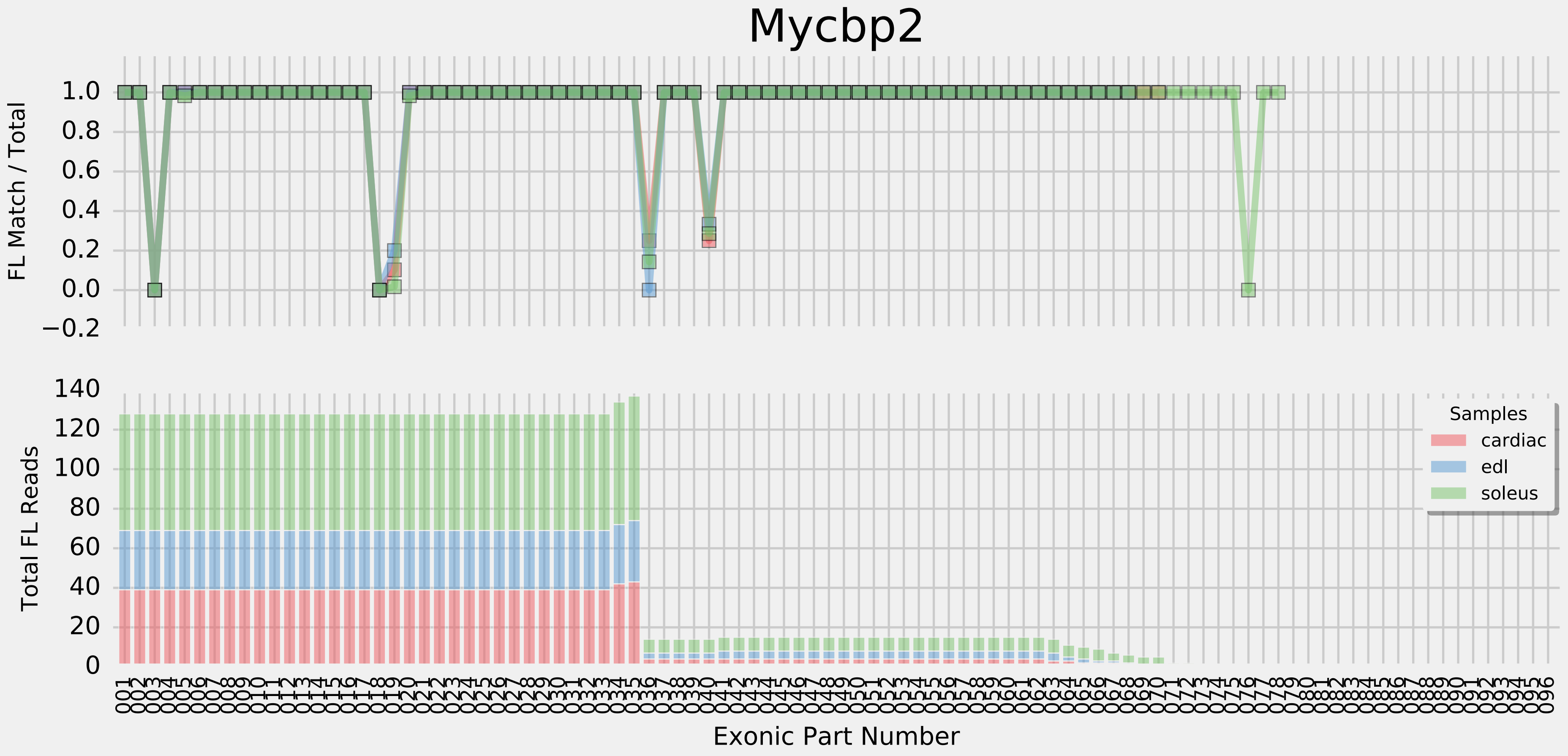
Mycbp2
FL Match / Total
Total FL Reads
Exonic Part Number
Samples
cardiac
edl
soleus

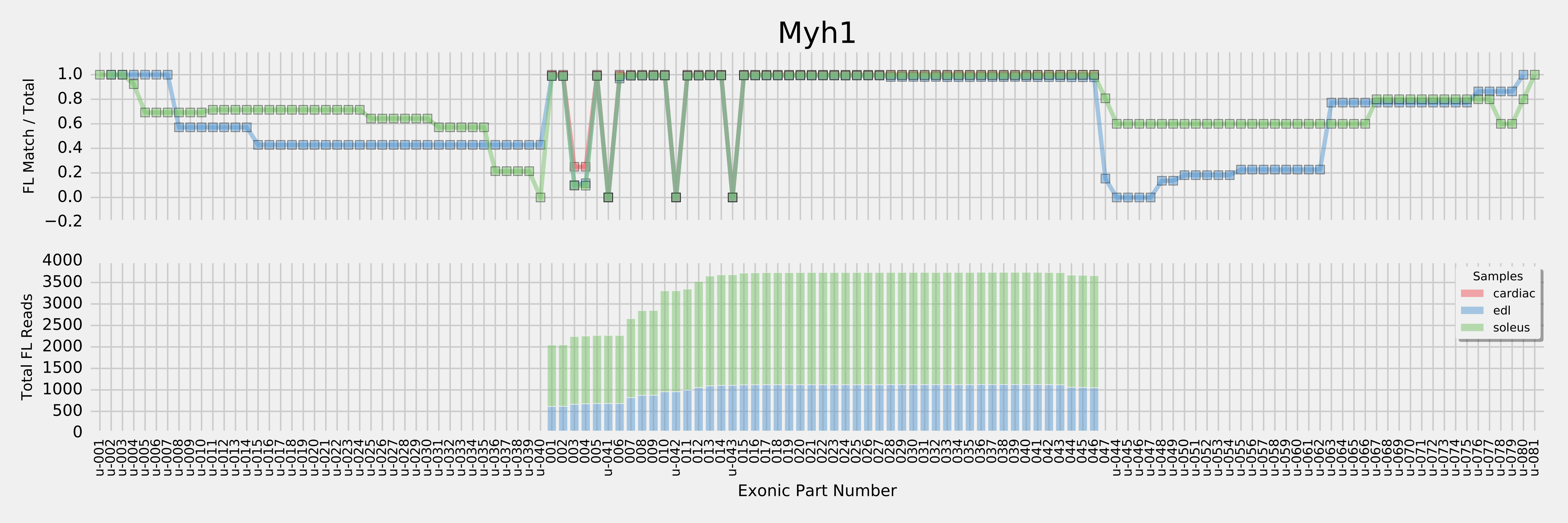

Myh1
FL Match / Total
Total FL Reads
Samples
cardiac
edl
soleus
Exonic Part Number

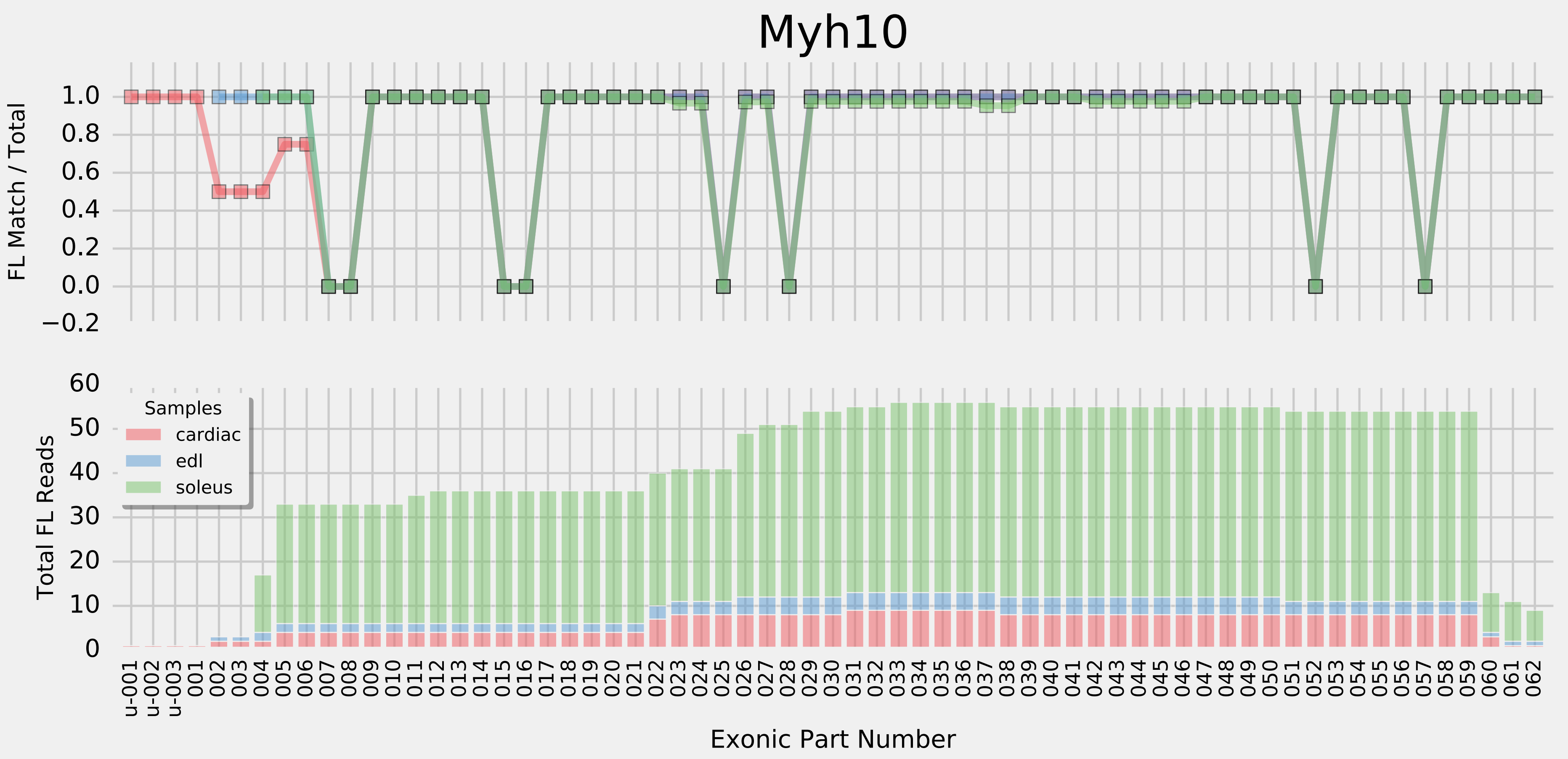

Myh10
FL Match / Total
1.0
0.8
0.6
0.4
0.2
0.0
−0.2
Total FL Reads
60
50
40
30
20
10
0
Samples
cardiac
edl
soleus
u-001
u-002
u-003
001
002
003
004
005
006
007
008
009
010
011
012
013
014
015
016
017
018
019
020
021
022
023
024
025
026
027
028
029
030
031
032
033
034
035
036
037
038
039
040
041
042
043
044
045
046
047
048
049
050
051
052
053
054
055
056
057
058
059
060
061
062
Exonic Part Number

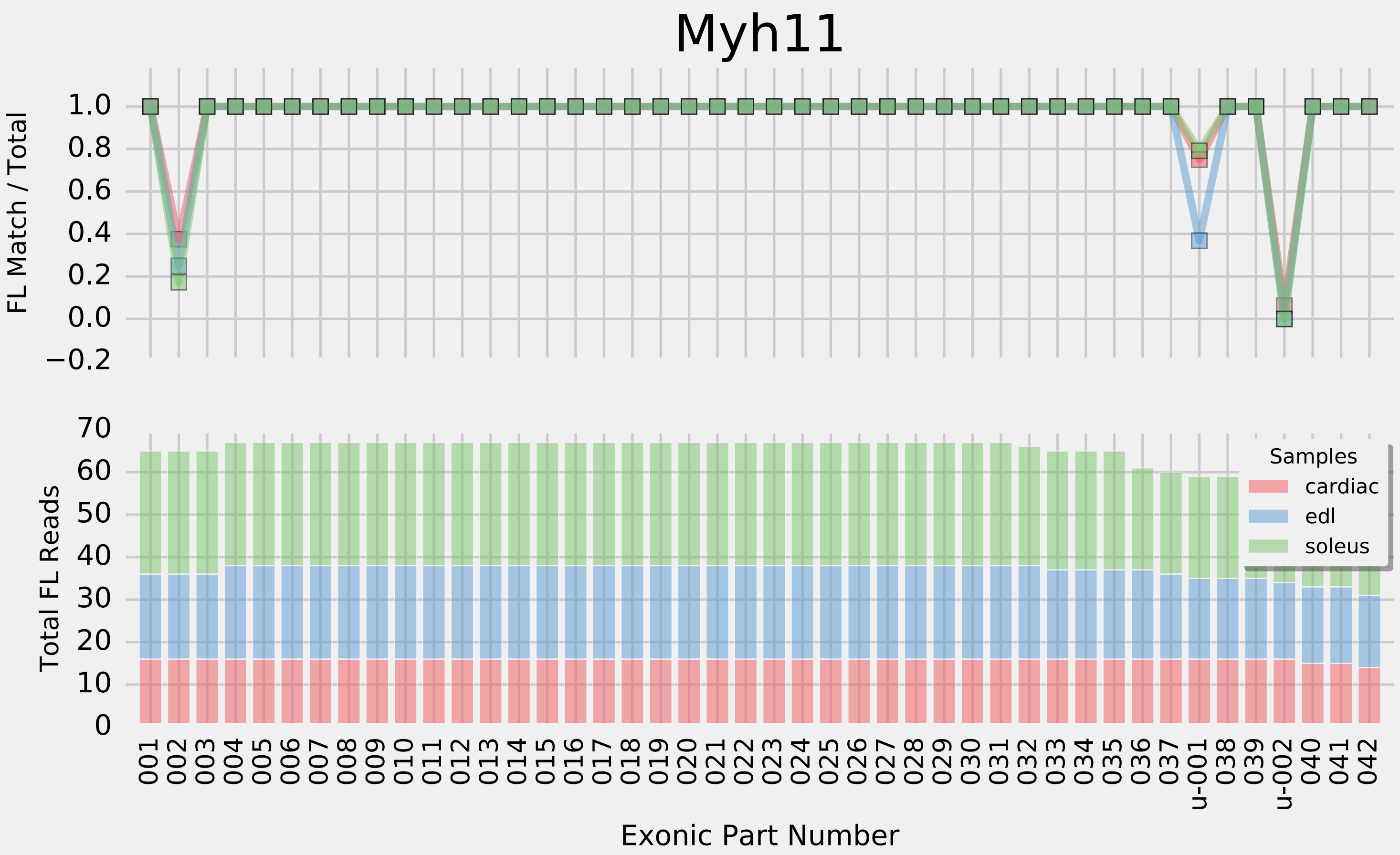
Myh11
FL Match / Total
Total FL Reads
Samples
cardiac
edl
soleus
Exonic Part Number
001 002 003 004 005 006 007 008 009 010 011 012 013 014 015 016 017 018 019 020 021 022 023 024 025 026 027 028 029 030 031 032 033 034 035 036 037 u-001 038 039 u-002 040 041 042

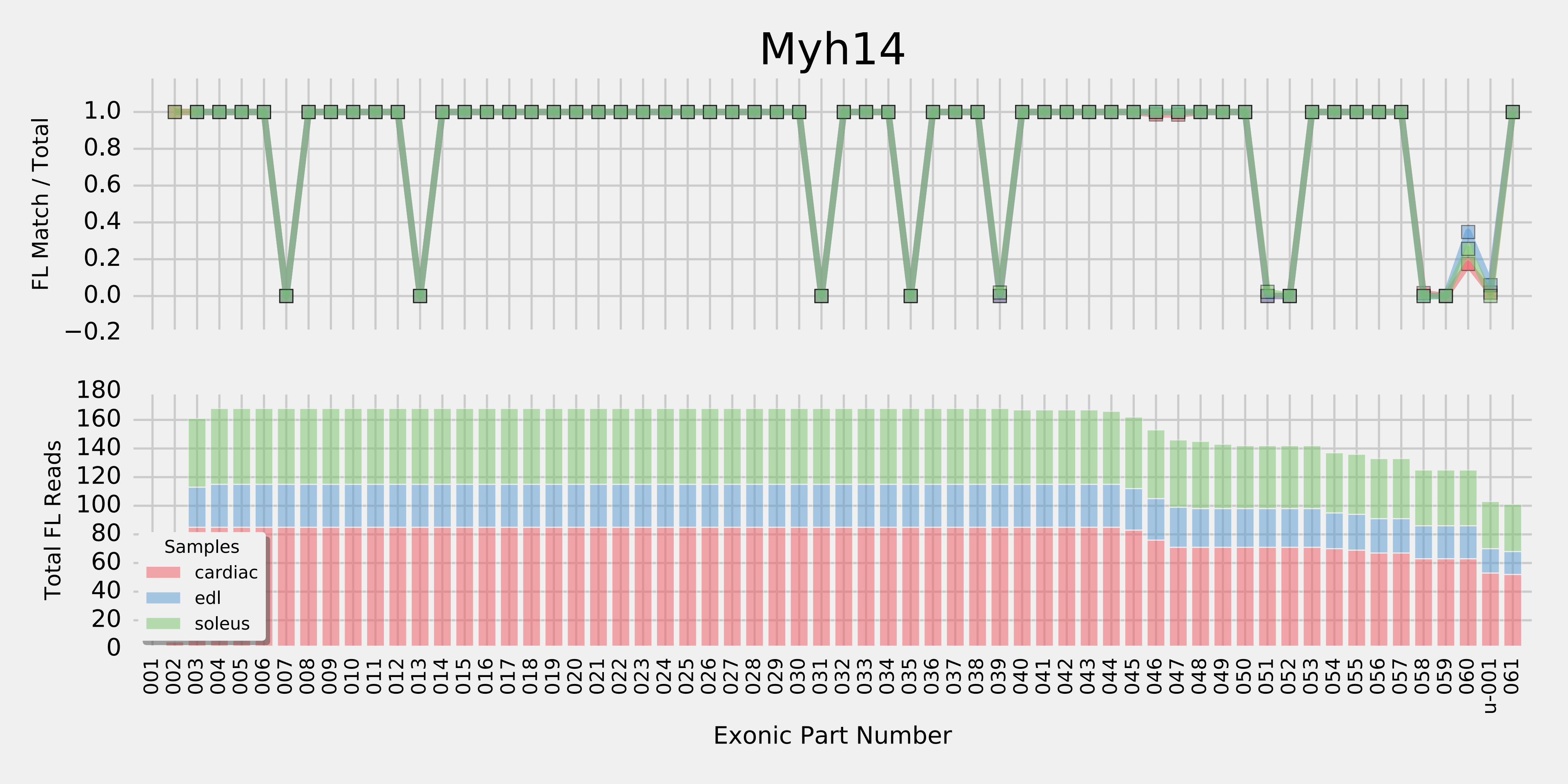

Myh14
FL Match / Total
Total FL Reads
Samples
cardiac
edl
soleus
Exonic Part Number

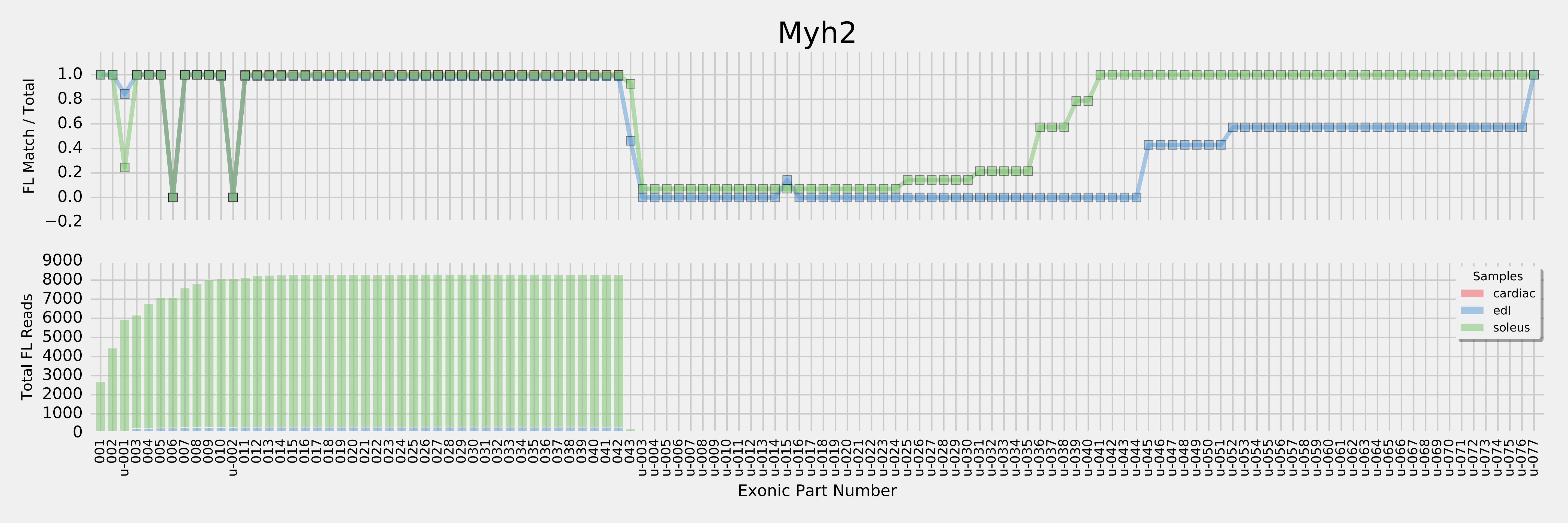

Myh2
FL Match / Total
Total FL Reads
Exonic Part Number
Samples
cardiac
edl
soleus

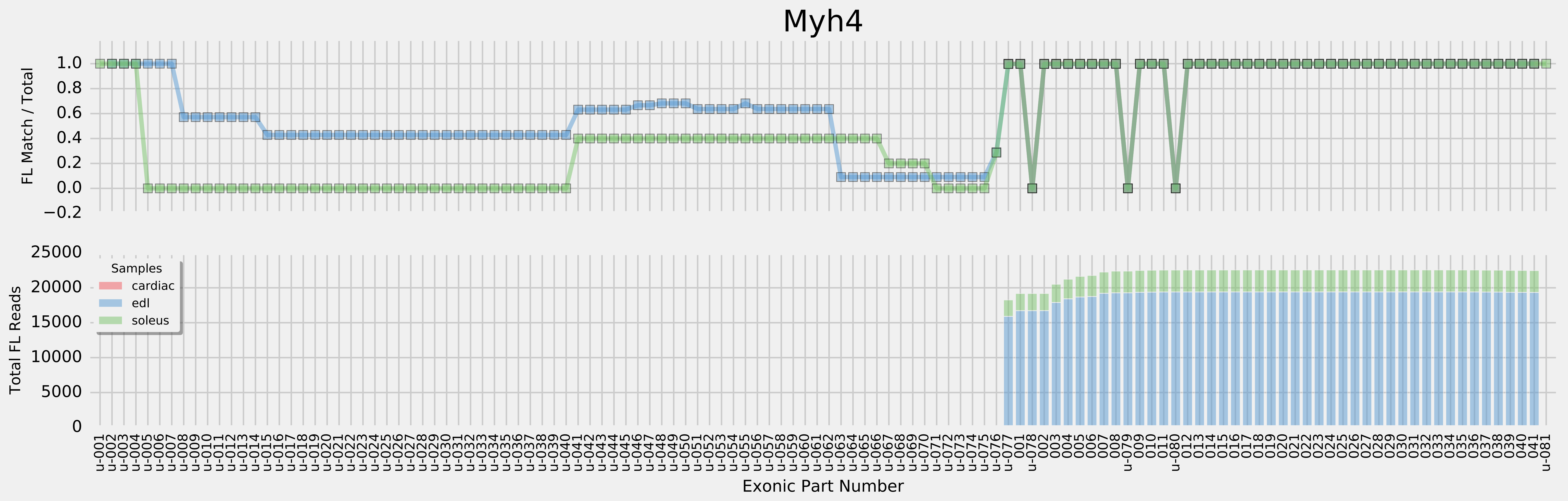

Myh4
FL Match / Total
Total FL Reads
Samples
cardiac
edl
soleus
Exonic Part Number

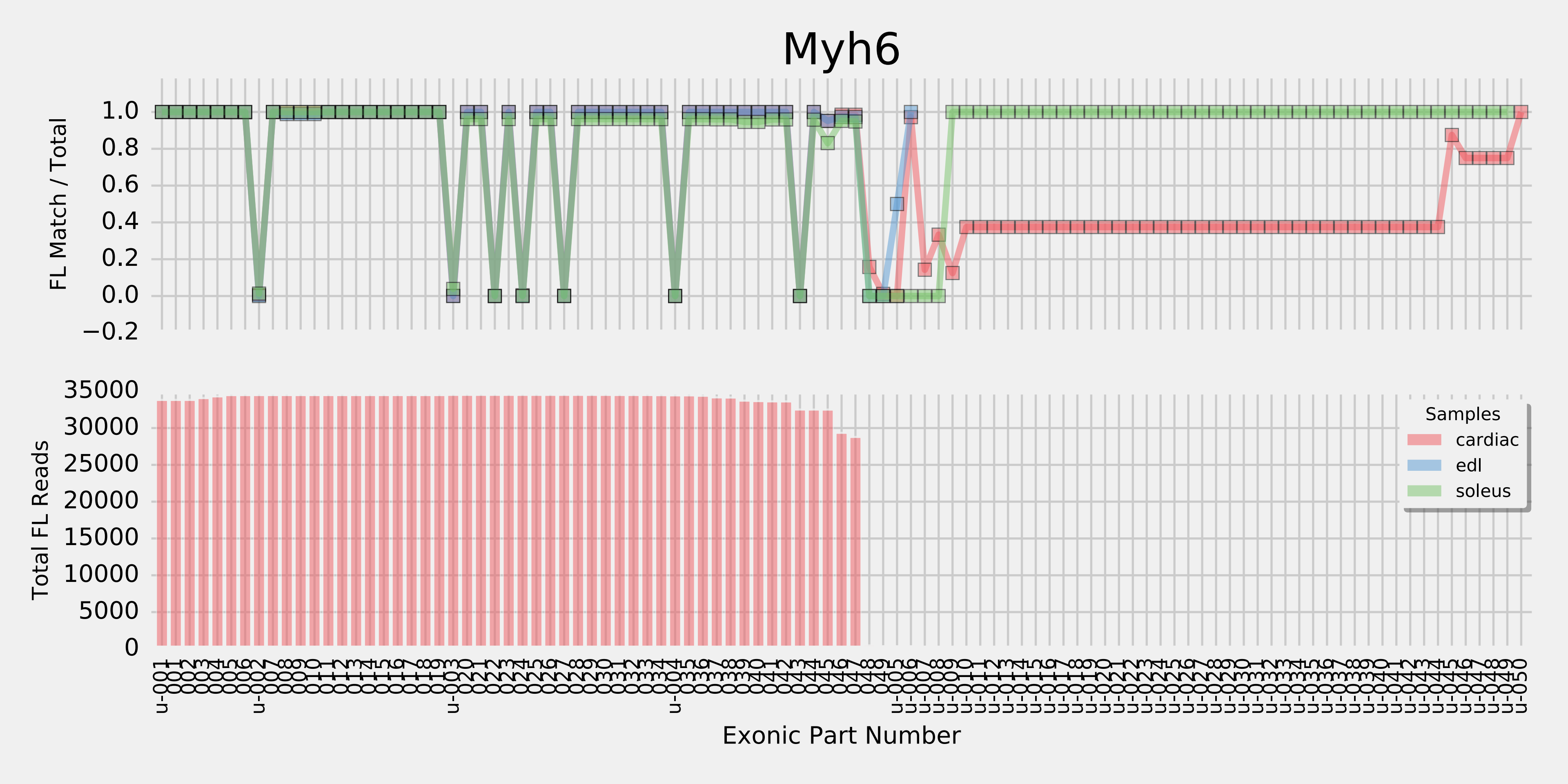

Myh6
FL Match / Total
Total FL Reads
Exonic Part Number
Samples
cardiac
edl
soleus

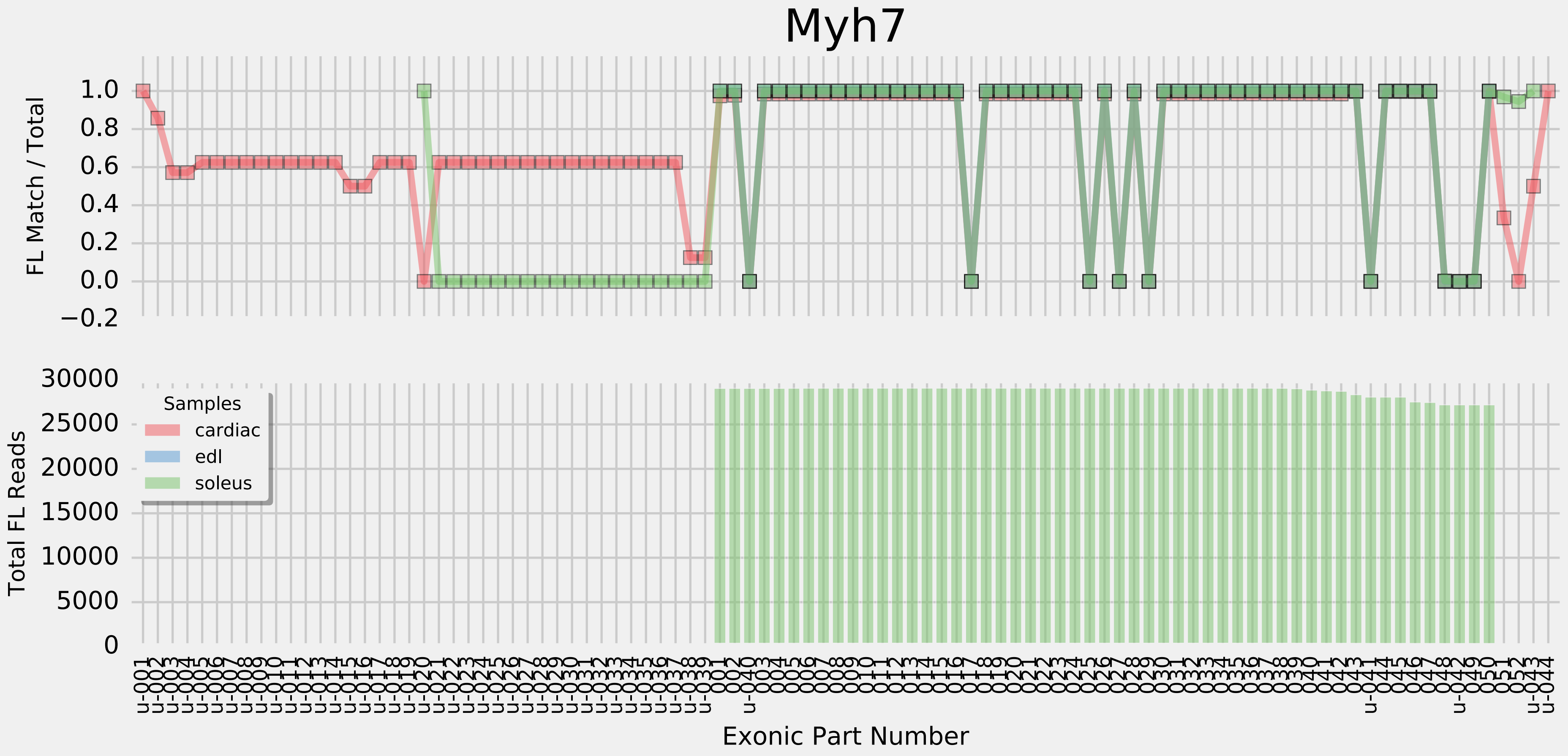
Myh7
FL Match / Total
1.0
0.8
0.6
0.4
0.2
0.0
−0.2
Total FL Reads
30000
25000
20000
15000
10000
5000
0
Samples
cardiac
edl
soleus
Exonic Part Number

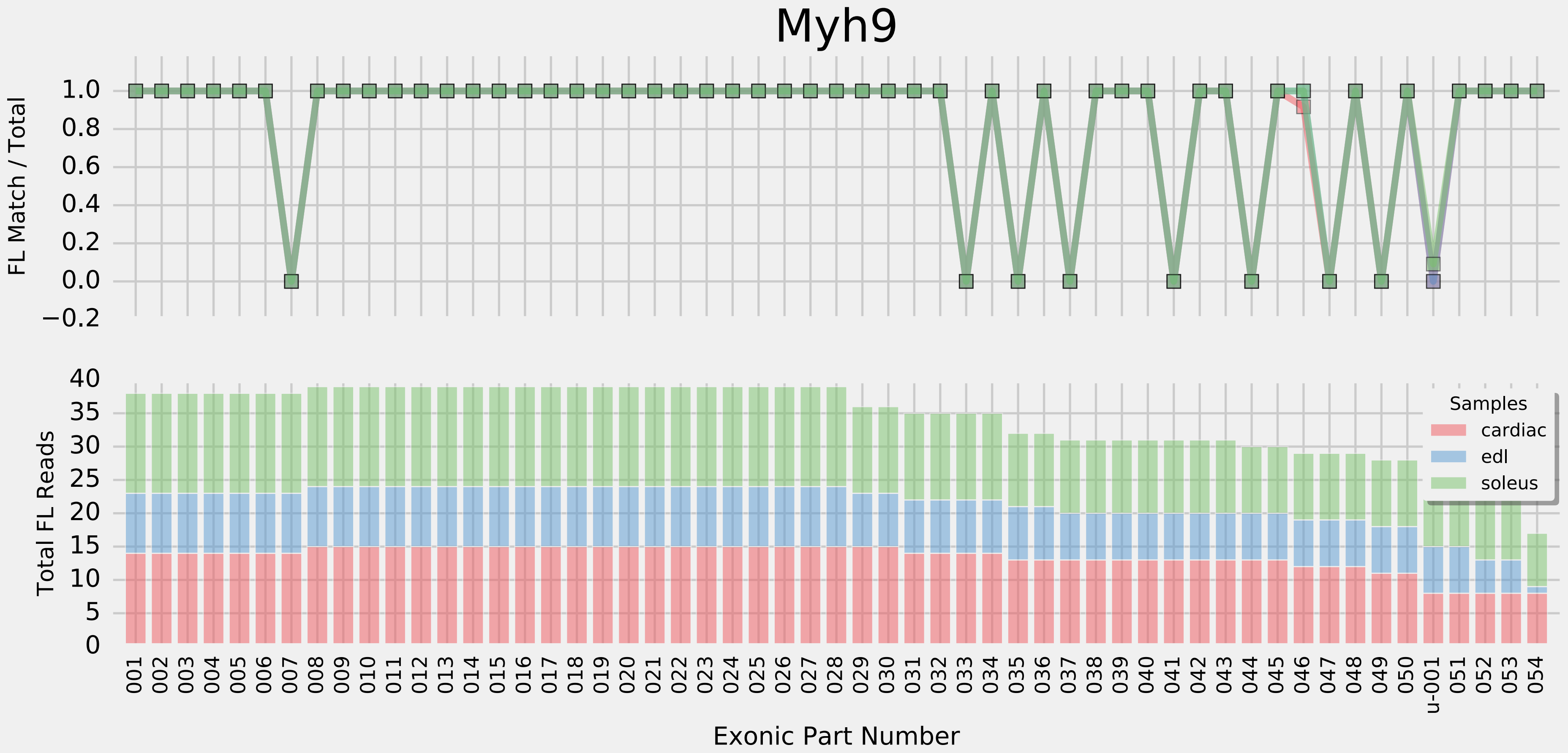

Myh9
FL Match / Total
1.0
0.8
0.6
0.4
0.2
0.0
−0.2
Total FL Reads
40
35
30
25
20
15
10
5
0
Samples
cardiac
edl
soleus
001 002 003 004 005 006 007 008 009 010 011 012 013 014 015 016 017 018 019 020 021 022 023 024 025 026 027 028 029 030 031 032 033 034 035 036 037 038 039 040 041 042 043 044 045 046 047 048 049 050 u-001 051 052 053 054
Exonic Part Number

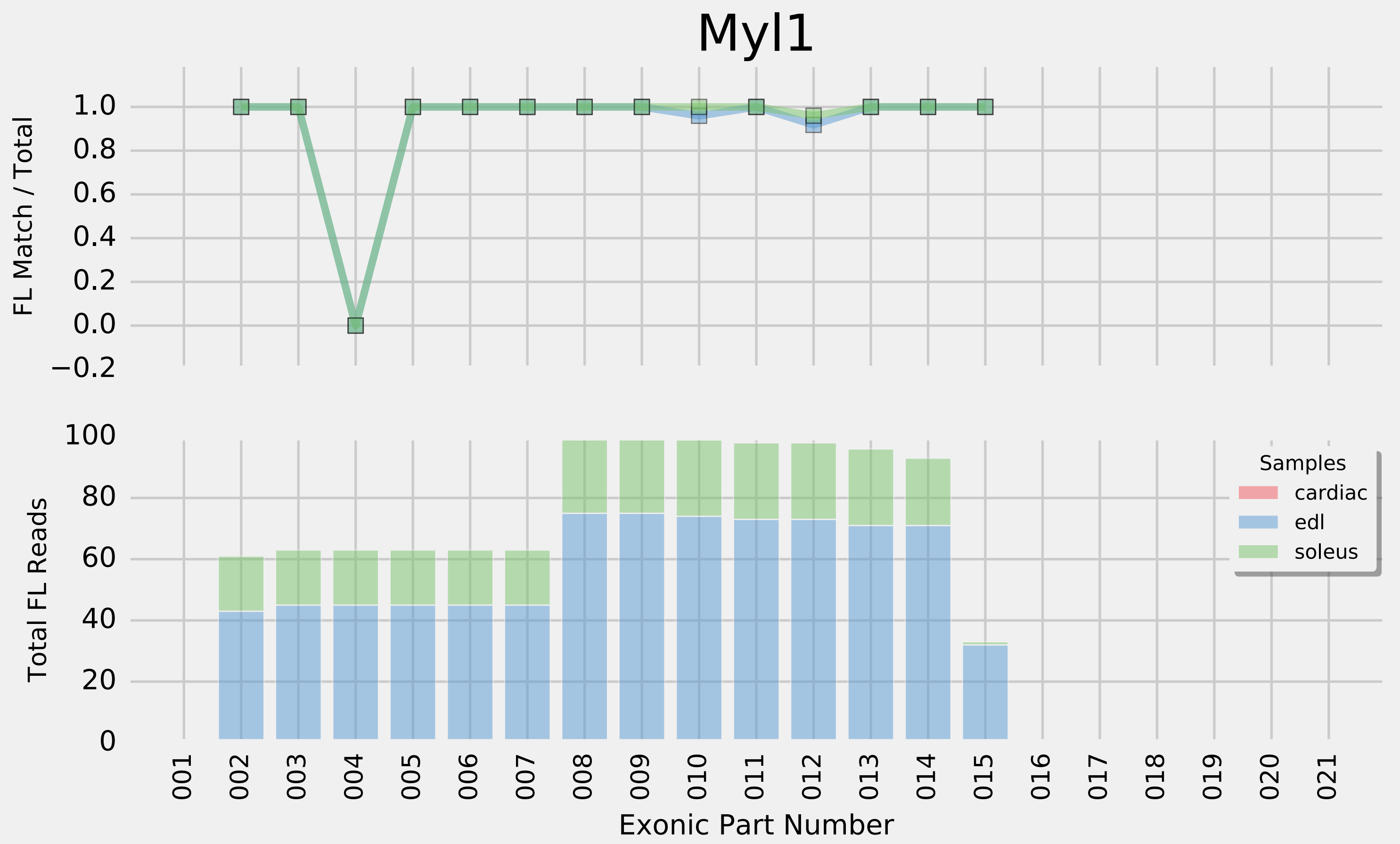

Myl1
FL Match / Total
1.0
0.8
0.6
0.4
0.2
0.0
−0.2
Total FL Reads
100
80
60
40
20
0
Samples
cardiac
edl
soleus
001
002
003
004
005
006
007
008
009
010
011
012
013
014
015
016
017
018
019
020
021
Exonic Part Number

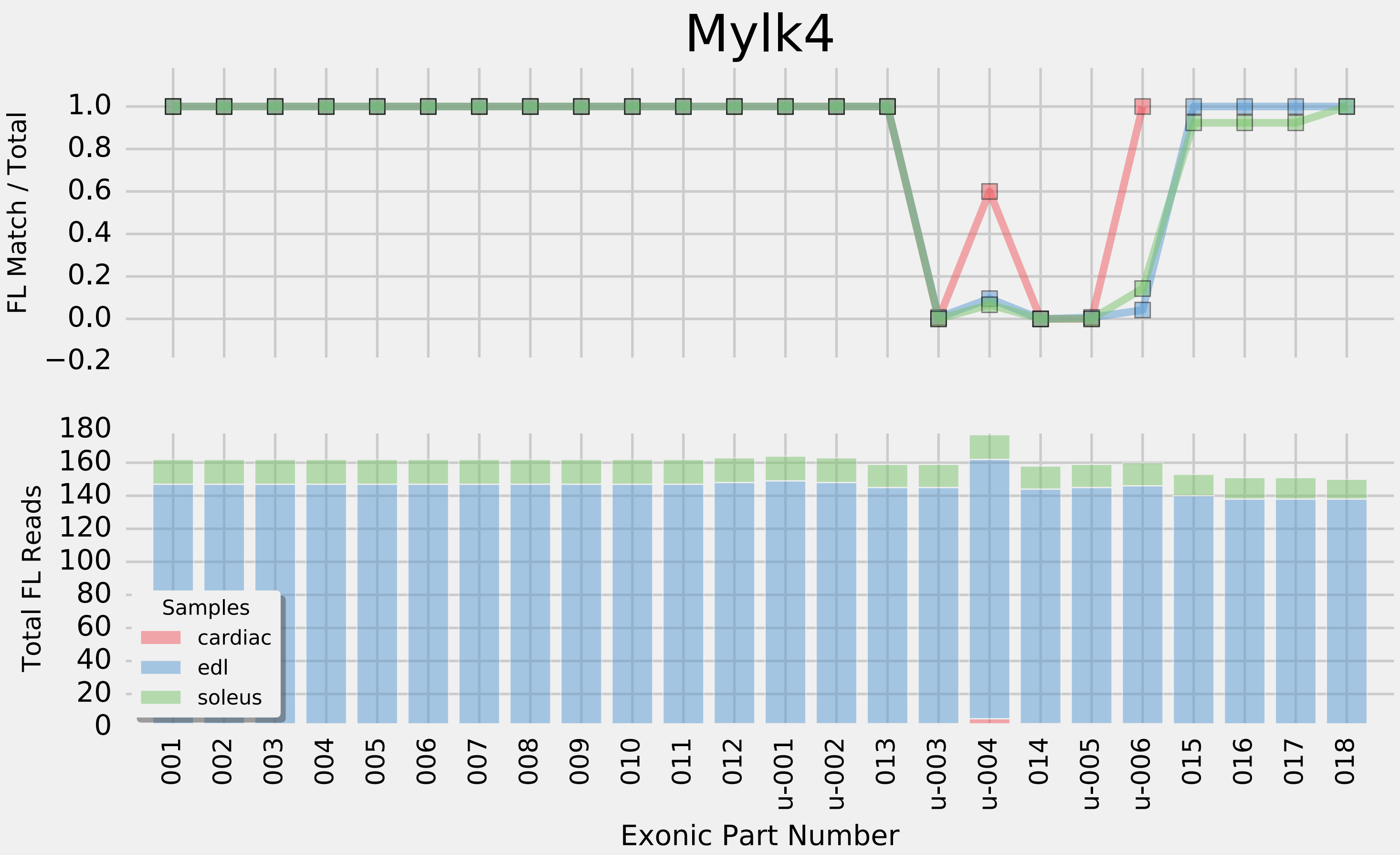

Mylk4
FL Match / Total
1.0
0.8
0.6
0.4
0.2
0.0
−0.2
Total FL Reads
180
160
140
120
100
80
60
40
20
0
Samples
cardiac
edl
soleus
001
002
003
004
005
006
007
008
009
010
011
012
u-001
u-002
013
u-003
u-004
014
u-005
u-006
015
016
017
018
Exonic Part Number

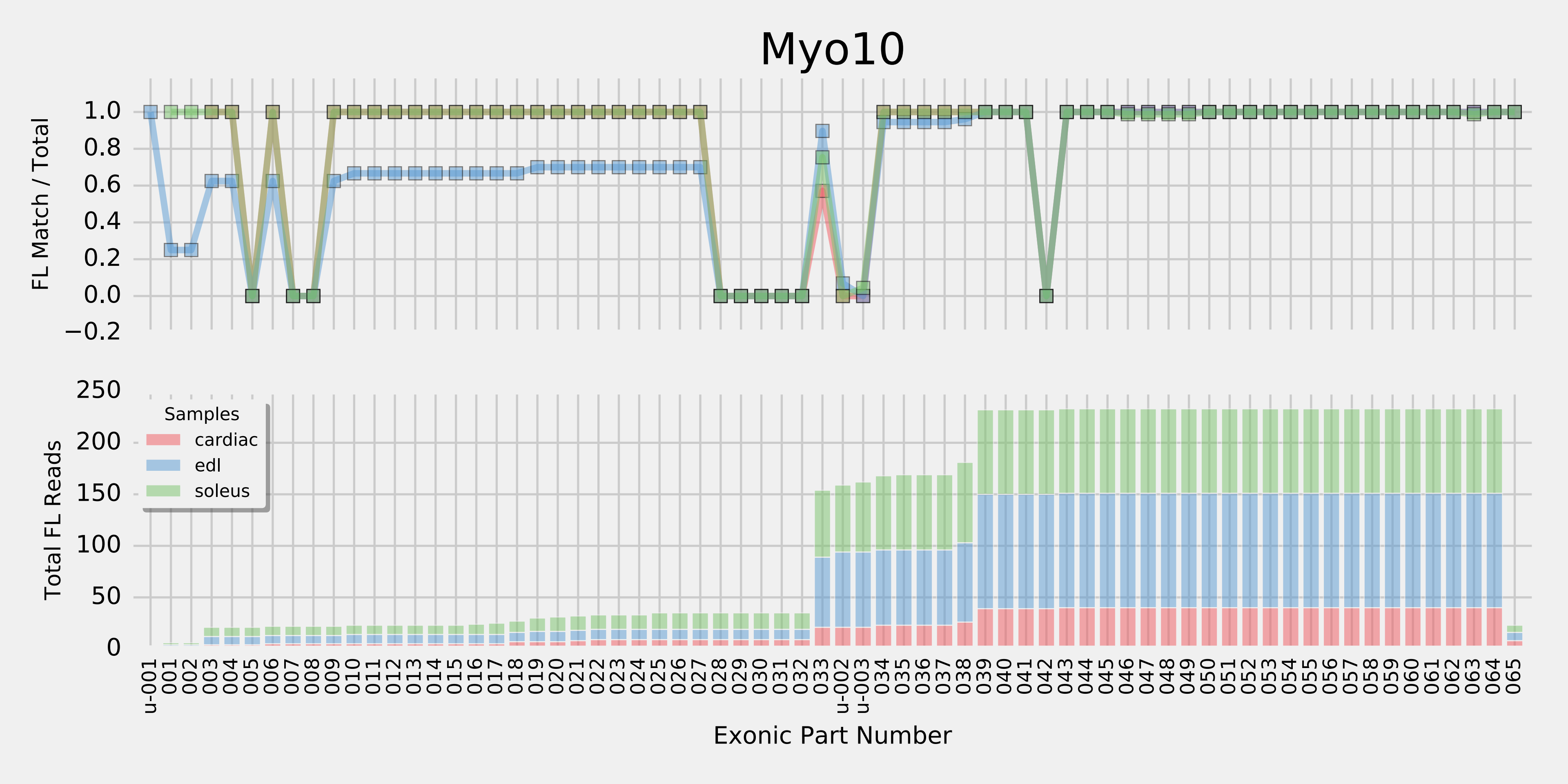

Myo10
FL Match / Total
Total FL Reads
Samples
cardiac
edl
soleus
Exonic Part Number

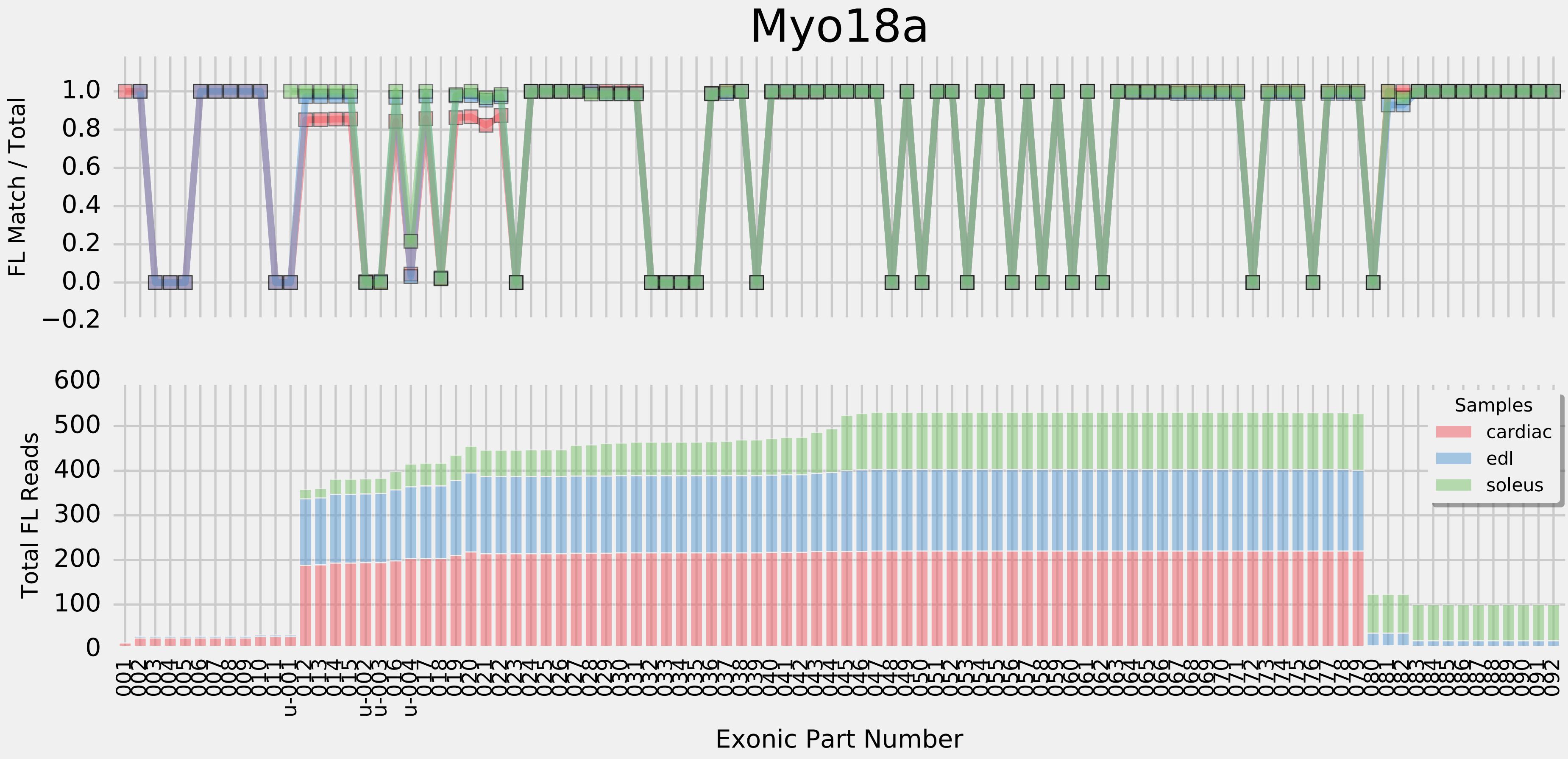
Myo18a
FL Match / Total
Total FL Reads
Samples
cardiac
edl
soleus
Exonic Part Number

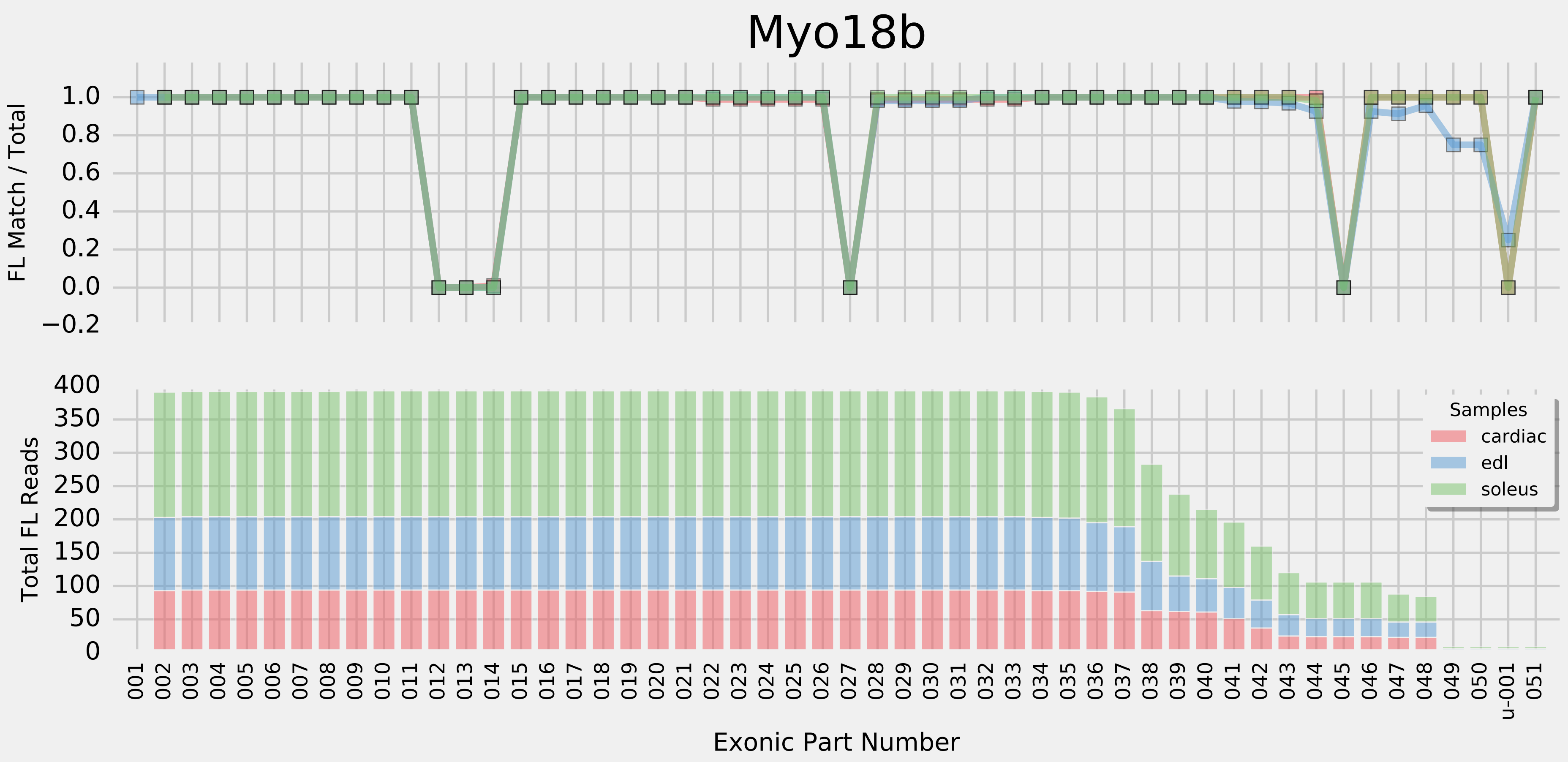

Myo18b
FL Match / Total
Total FL Reads
Samples
cardiac
edl
soleus
Exonic Part Number
001 002 003 004 005 006 007 008 009 010 011 012 013 014 015 016 017 018 019 020 021 022 023 024 025 026 027 028 029 030 031 032 033 034 035 036 037 038 039 040 041 042 043 044 045 046 047 048 049 050 u-001 051

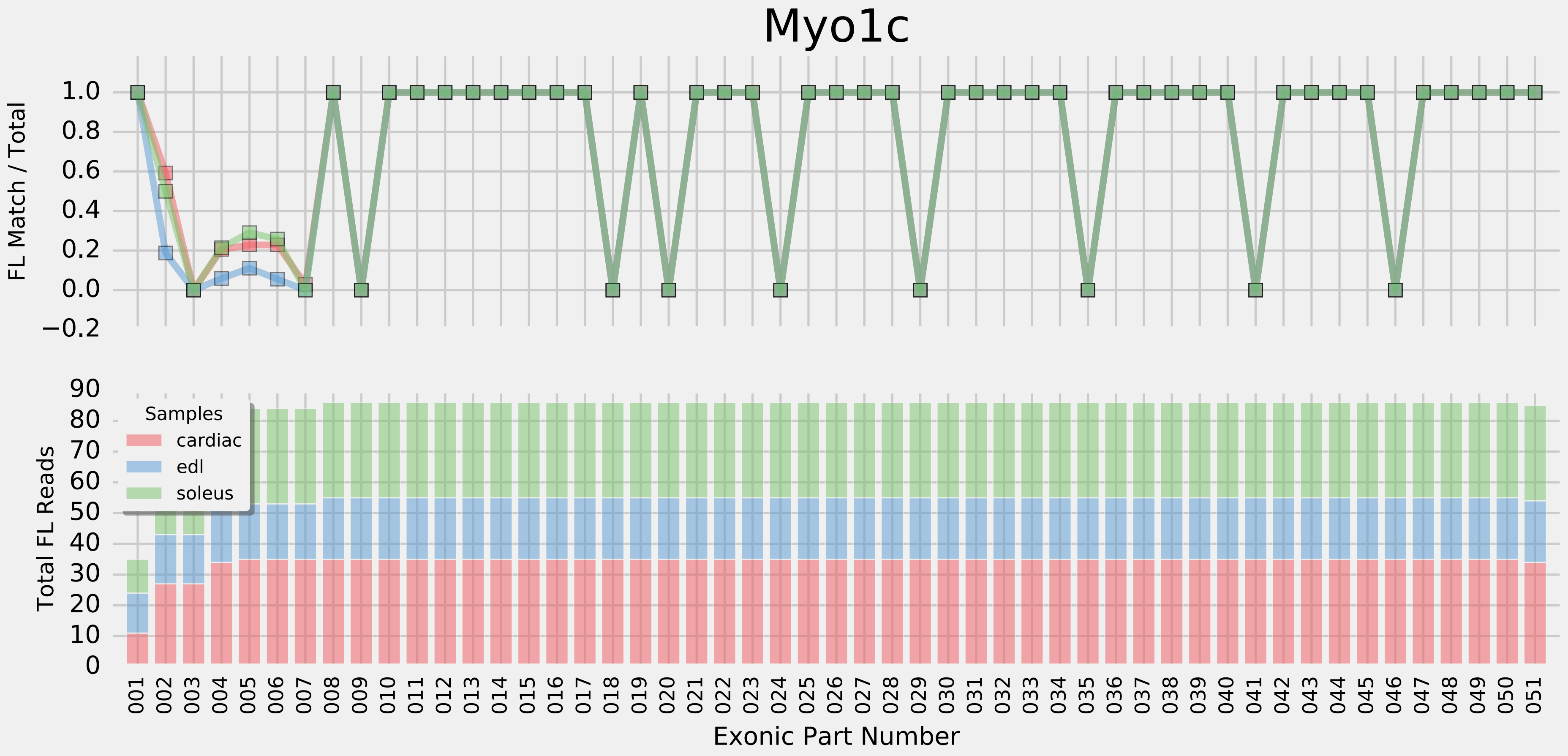

Myo1c
FL Match / Total
1.0
0.8
0.6
0.4
0.2
0.0
−0.2
Total FL Reads
90
80
70
60
50
40
30
20
10
0
Samples
cardiac
edl
soleus
001 002 003 004 005 006 007 008 009 010 011 012 013 014 015 016 017 018 019 020 021 022 023 024 025 026 027 028 029 030 031 032 033 034 035 036 037 038 039 040 041 042 043 044 045 046 047 048 049 050 051
Exonic Part Number

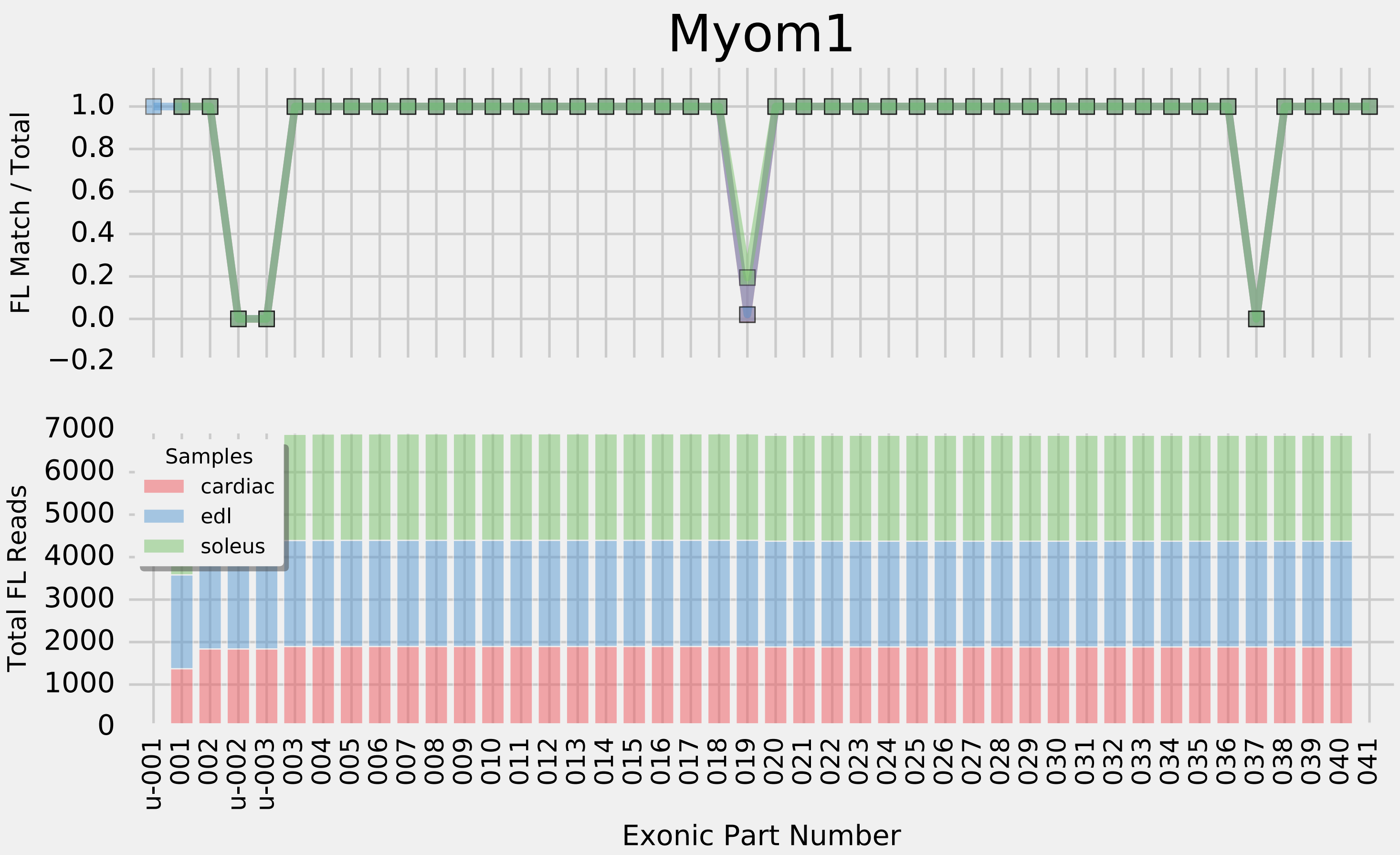
Myom1
FL Match / Total
1.0
0.8
0.6
0.4
0.2
0.0
−0.2
Total FL Reads
7000
6000
5000
4000
3000
2000
1000
0
Samples
cardiac
edl
soleus
u-001
001
002
u-002
u-003
003
004
005
006
007
008
009
010
011
012
013
014
015
016
017
018
019
020
021
022
023
024
025
026
027
028
029
030
031
032
033
034
035
036
037
038
039
040
041
Exonic Part Number

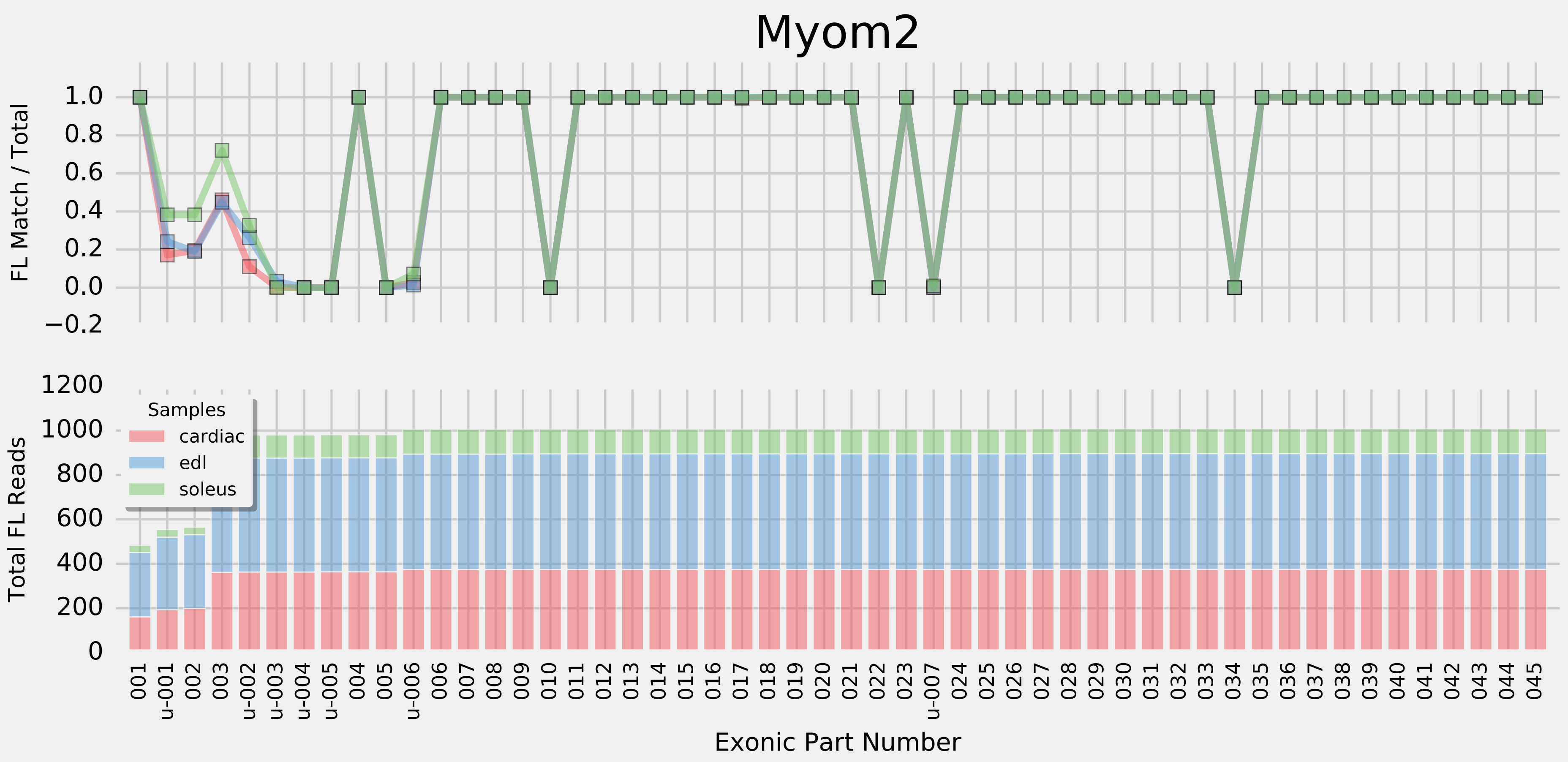

Myom2
FL Match / Total
1.0
0.8
0.6
0.4
0.2
0.0
−0.2
Total FL Reads
1200
1000
800
600
400
200
0
Samples
cardiac
edl
soleus
001 u-001 002 003 u-002 u-003 u-004 u-005 004 005 u-006 006 007 008 009 010 011 012 013 014 015 016 017 018 019 020 021 022 023 u-007 024 025 026 027 028 029 030 031 032 033 034 035 036 037 038 039 040 041 042 043 044 045
Exonic Part Number

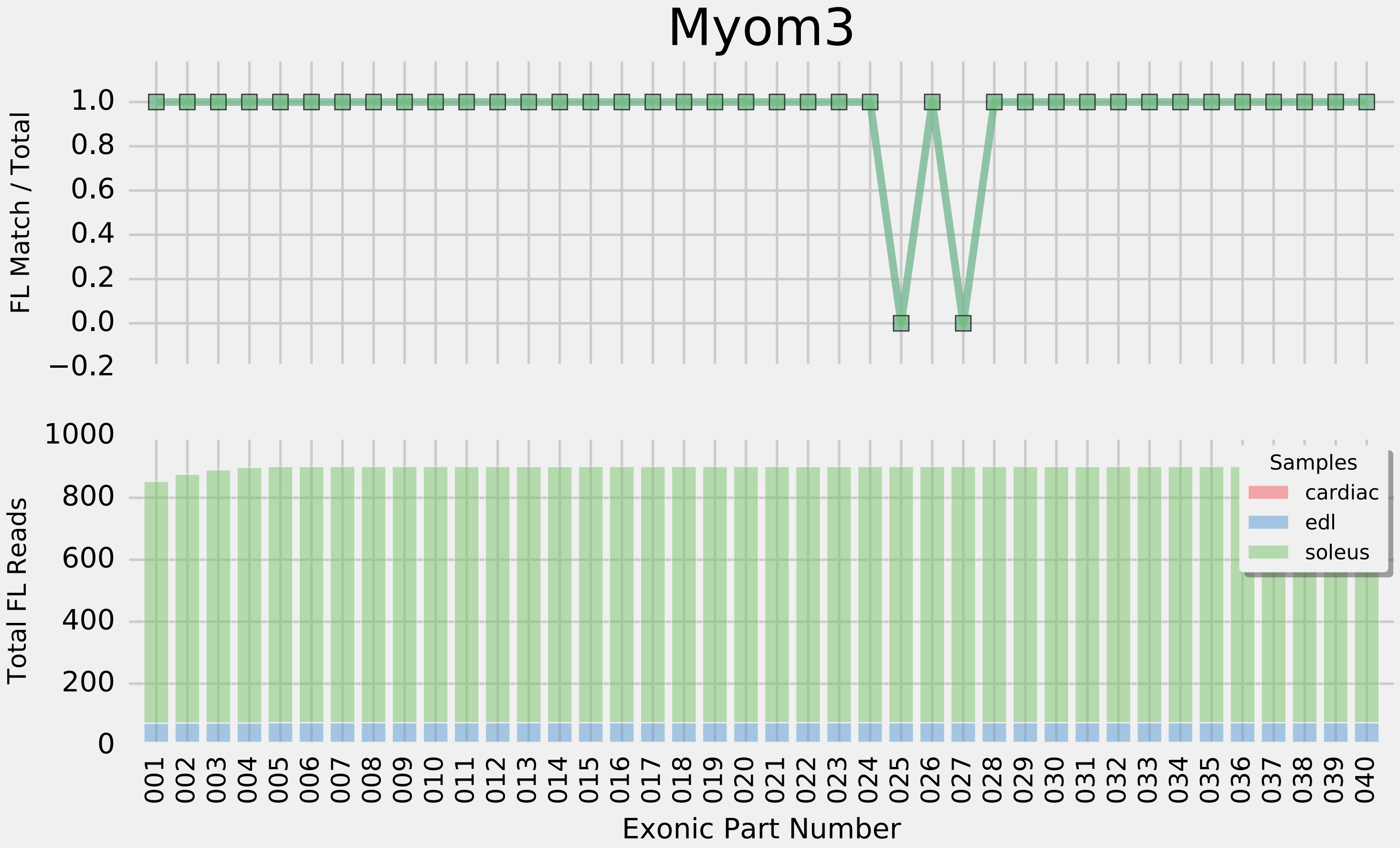

Myom3
FL Match / Total
1.0
0.8
0.6
0.4
0.2
0.0
−0.2
Total FL Reads
1000
800
600
400
200
0
Samples
cardiac
edl
soleus
001 002 003 004 005 006 007 008 009 010 011 012 013 014 015 016 017 018 019 020 021 022 023 024 025 026 027 028 029 030 031 032 033 034 035 036 037 038 039 040
Exonic Part Number

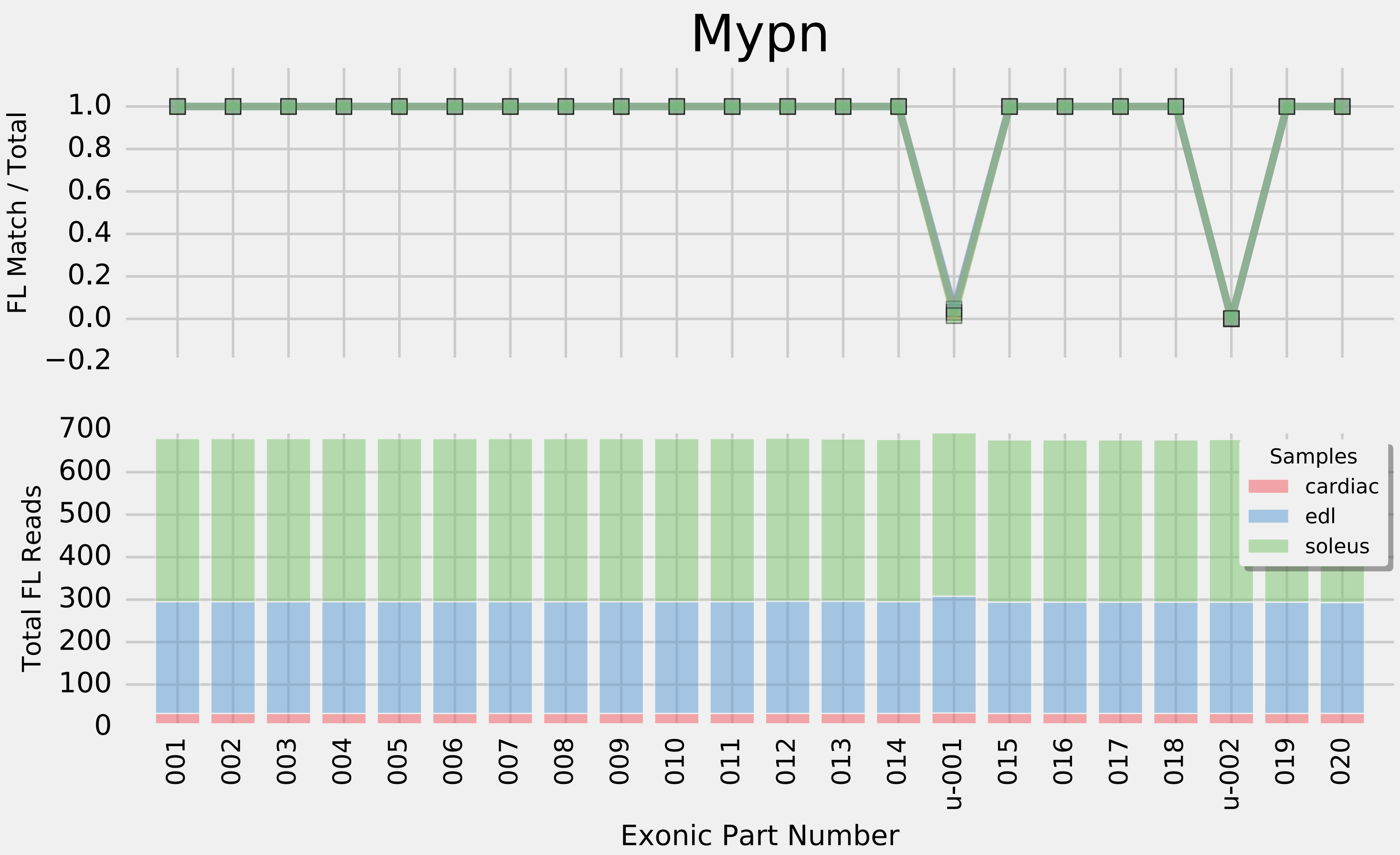
Mypn
FL Match / Total
1.0
0.8
0.6
0.4
0.2
0.0
−0.2
Total FL Reads
700
600
500
400
300
200
100
0
Samples
cardiac
edl
soleus
001
002
003
004
005
006
007
008
009
010
011
012
013
014
u-001
015
016
017
018
u-002
019
020
Exonic Part Number

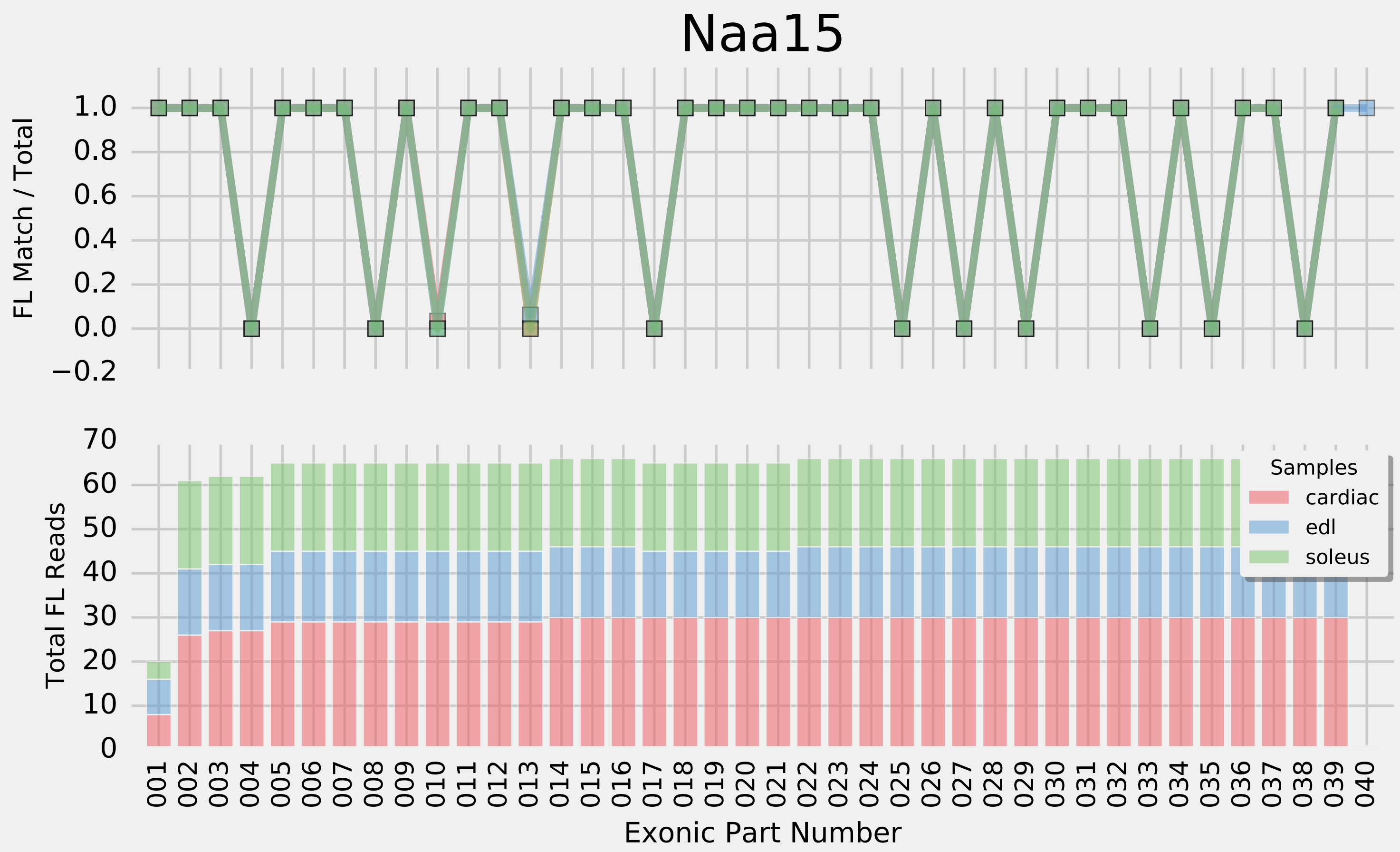

Naa15
FL Match / Total
1.0
0.8
0.6
0.4
0.2
0.0
−0.2
Total FL Reads
70
60
50
40
30
20
10
0
Samples
cardiac
edl
soleus
001 002 003 004 005 006 007 008 009 010 011 012 013 014 015 016 017 018 019 020 021 022 023 024 025 026 027 028 029 030 031 032 033 034 035 036 037 038 039 040
Exonic Part Number

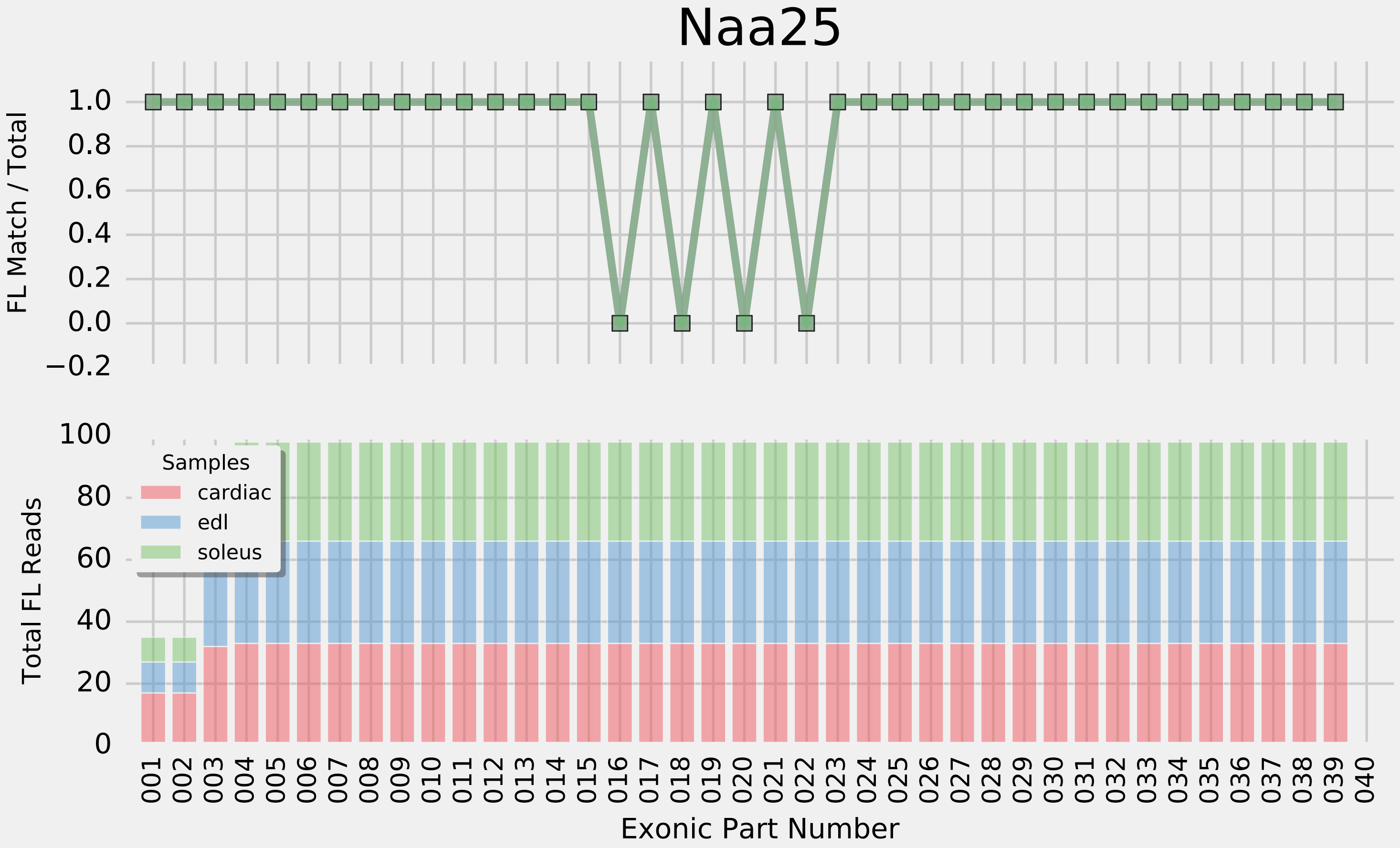
Naa25
FL Match / Total
1.0
0.8
0.6
0.4
0.2
0.0
−0.2
Total FL Reads
100
80
60
40
20
0
Samples
cardiac
edl
soleus
001 002 003 004 005 006 007 008 009 010 011 012 013 014 015 016 017 018 019 020 021 022 023 024 025 026 027 028 029 030 031 032 033 034 035 036 037 038 039 040
Exonic Part Number

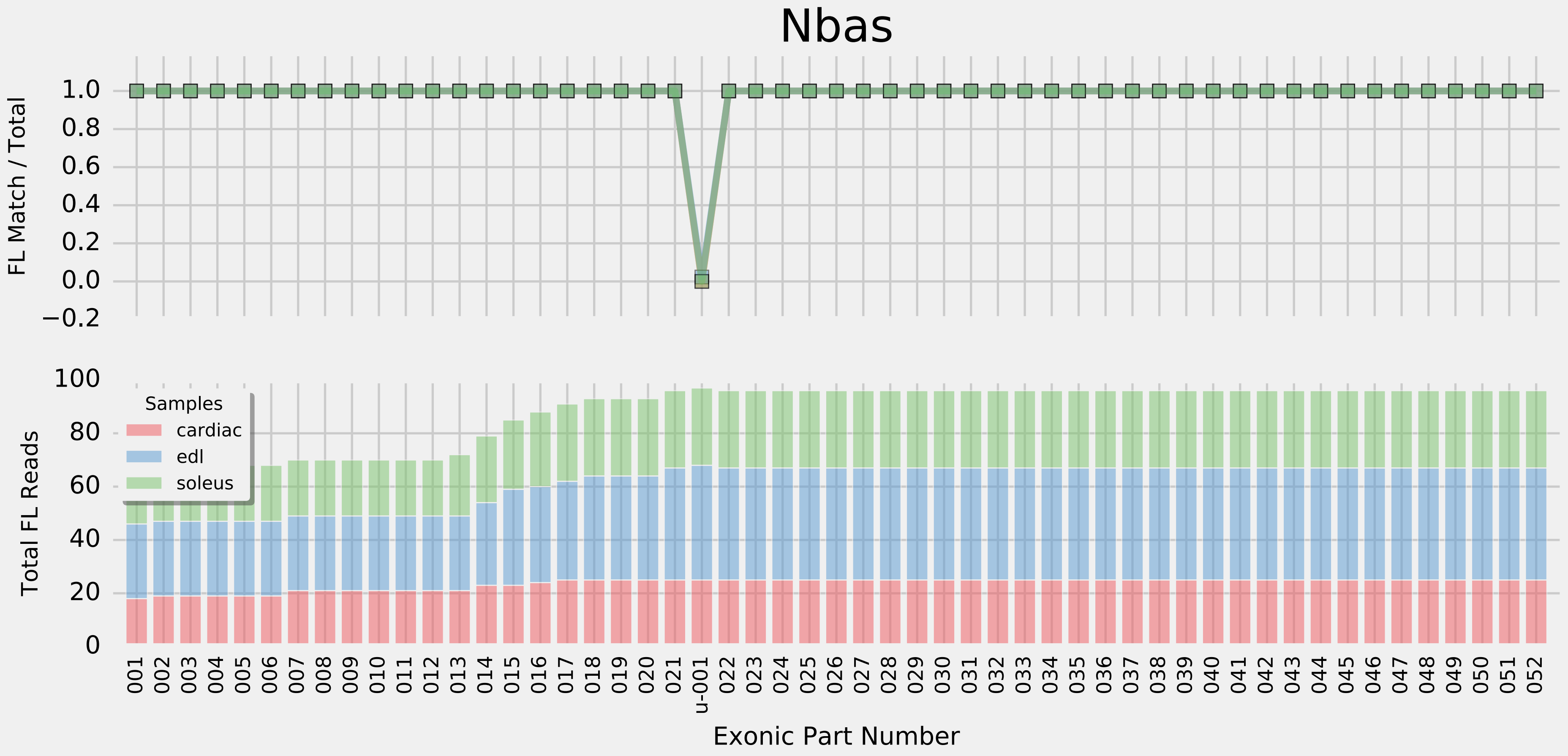

Nbas
FL Match / Total
1.0
0.8
0.6
0.4
0.2
0.0
−0.2
Total FL Reads
100
80
60
40
20
0
Samples
cardiac
edl
soleus
001 002 003 004 005 006 007 008 009 010 011 012 013 014 015 016 017 018 019 020 021 u-001 022 023 024 025 026 027 028 029 030 031 032 033 034 035 036 037 038 039 040 041 042 043 044 045 046 047 048 049 050 051 052
Exonic Part Number

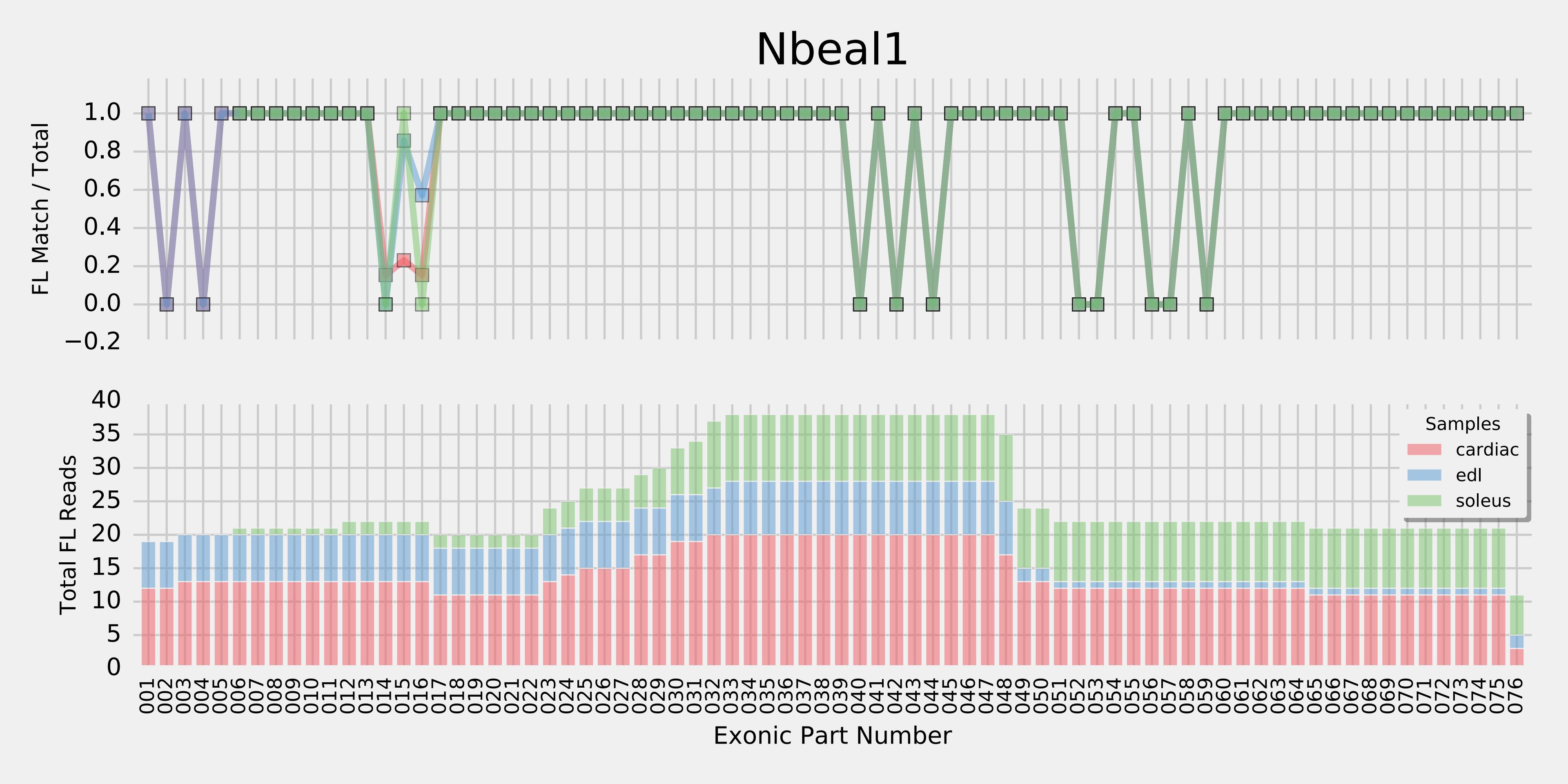

Nbeal1
FL Match / Total
Total FL Reads
Exonic Part Number
Samples
cardiac
edl
soleus

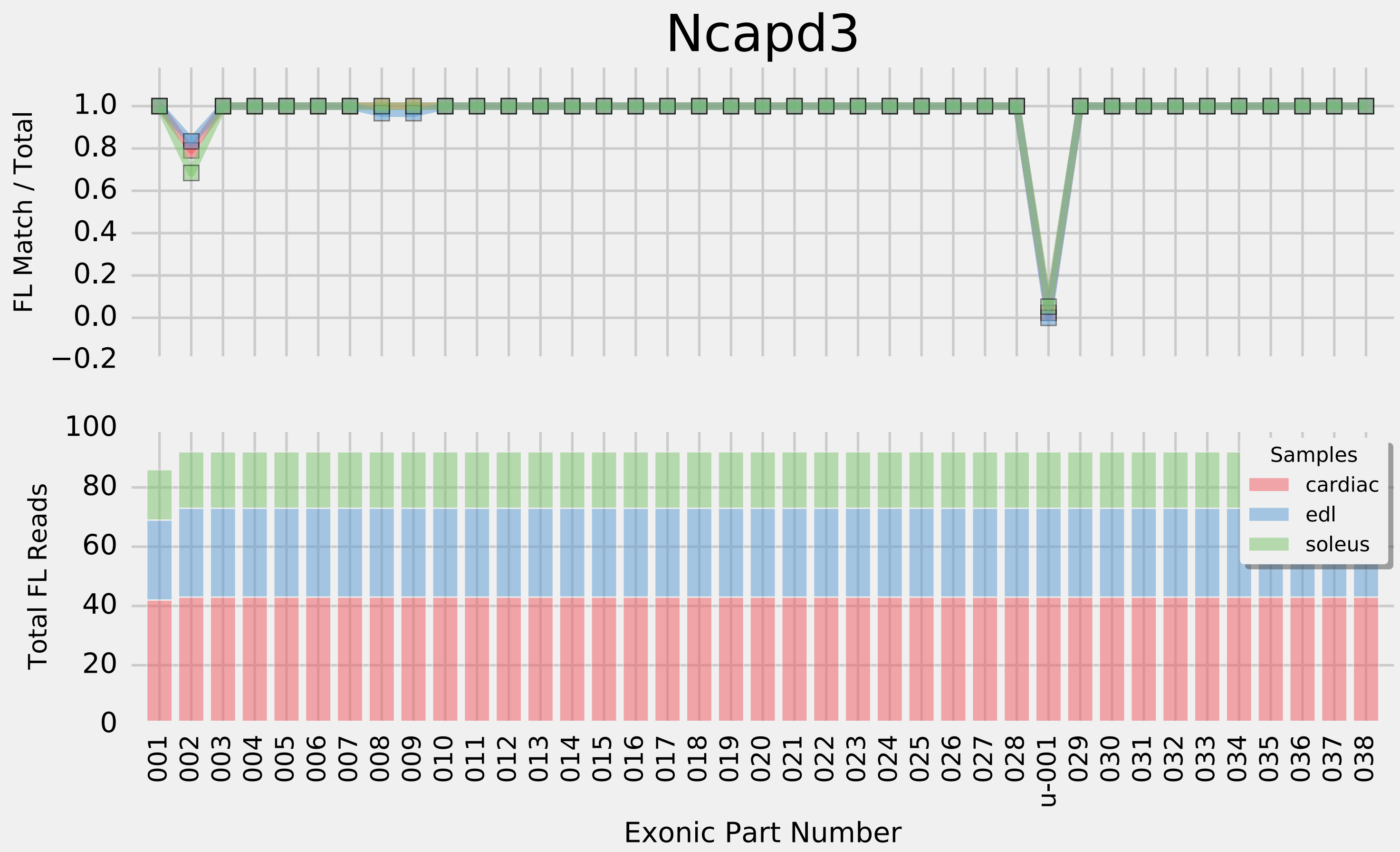

Ncapd3
FL Match / Total
1.0
0.8
0.6
0.4
0.2
0.0
−0.2
Total FL Reads
100
80
60
40
20
0
Samples
cardiac
edl
soleus
001 002 003 004 005 006 007 008 009 010 011 012 013 014 015 016 017 018 019 020 021 022 023 024 025 026 027 028 u-001 029 030 031 032 033 034 035 036 037 038
Exonic Part Number

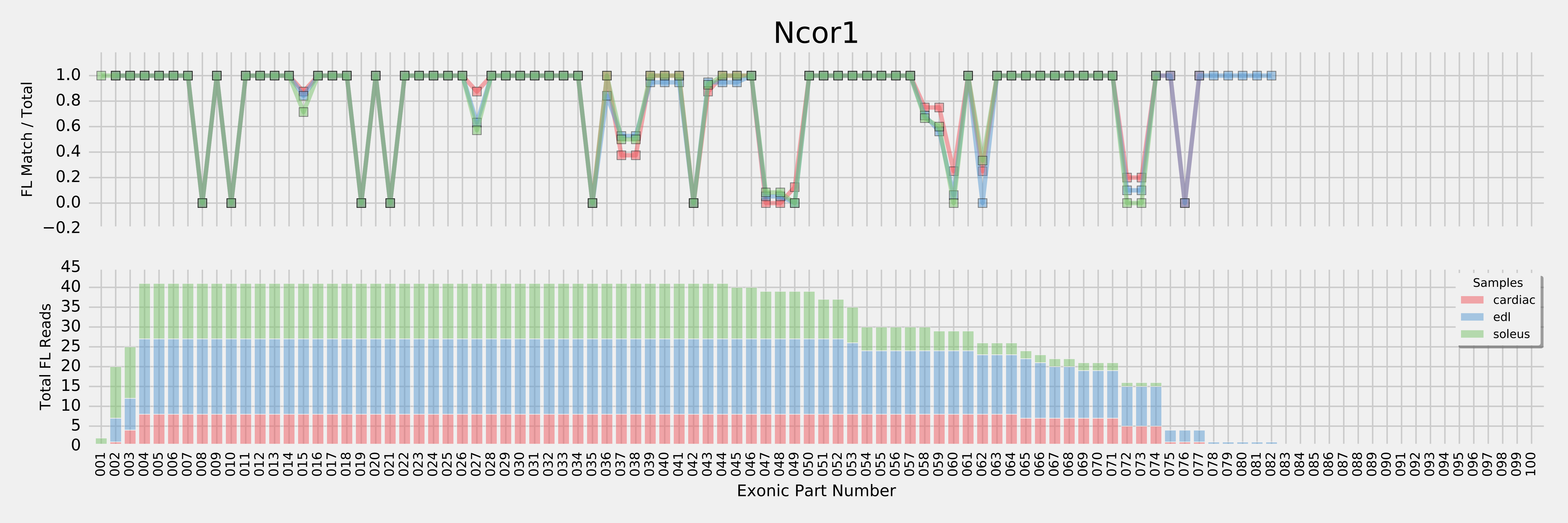

Ncor1
FL Match / Total
Total FL Reads
Exonic Part Number
Samples
cardiac
edl
soleus

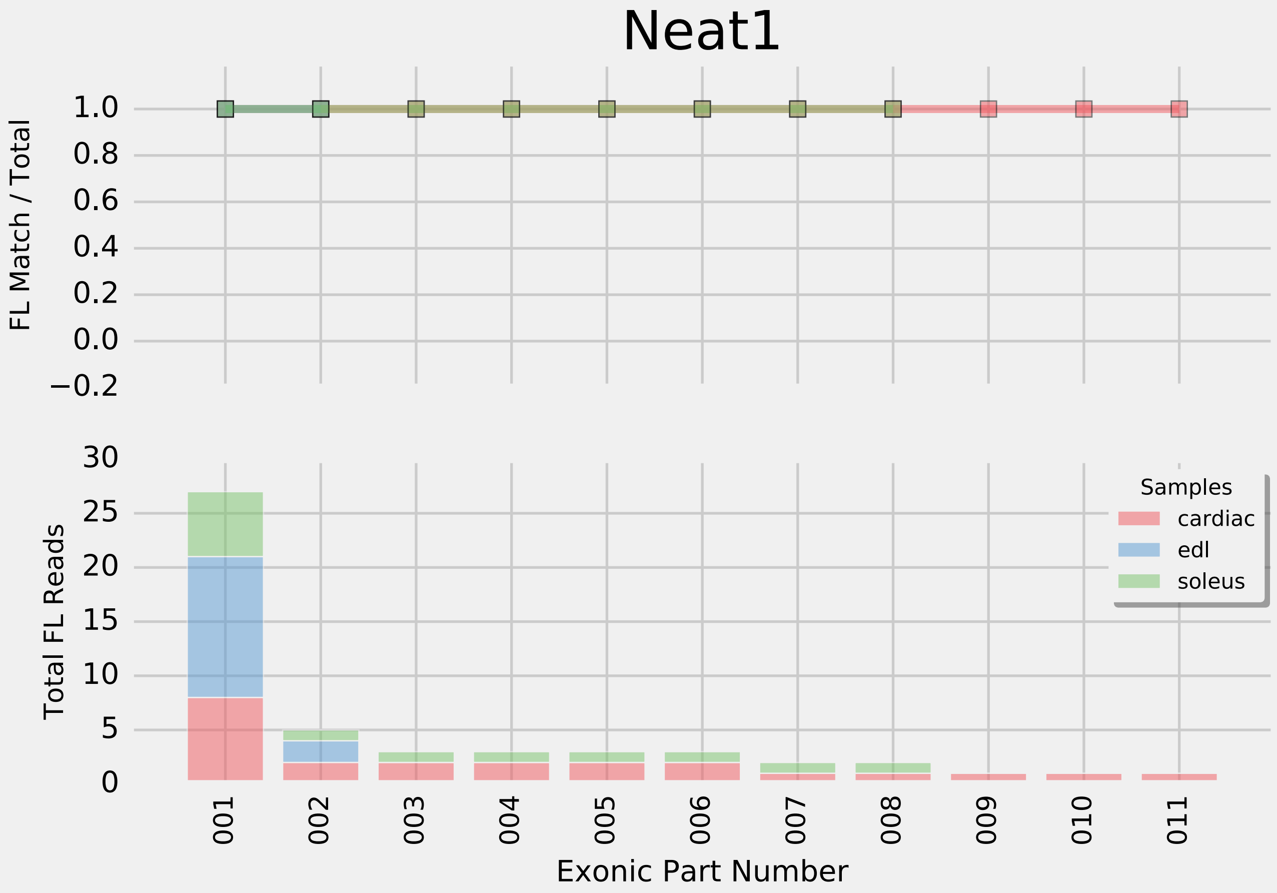
Neat1
FL Match / Total
1.0
0.8
0.6
0.4
0.2
0.0
−0.2
Total FL Reads
30
25
20
15
10
5
0
Samples
cardiac
edl
soleus
001
002
003
004
005
006
007
008
009
010
011
Exonic Part Number

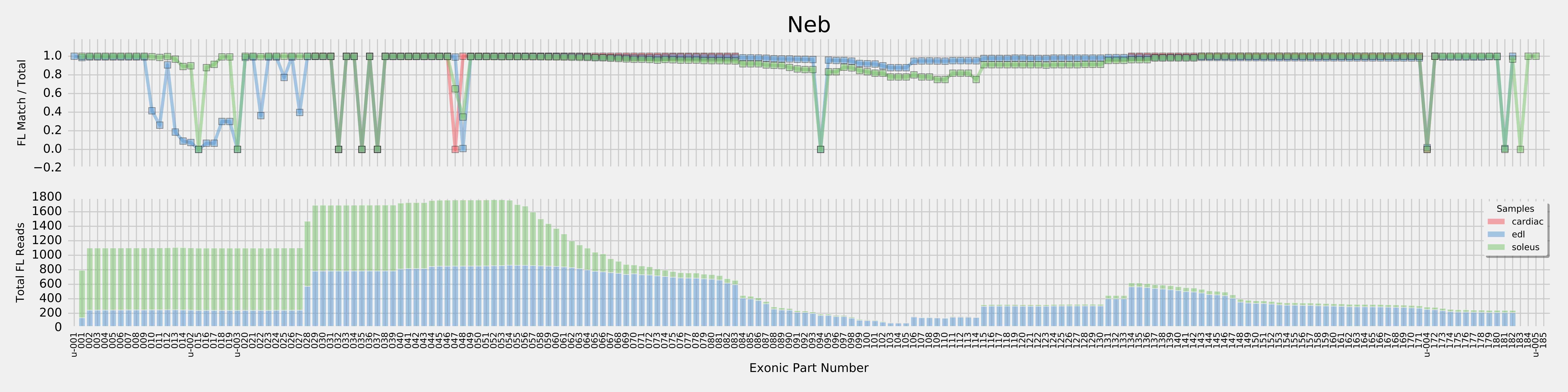

Neb
FL Match / Total
Total FL Reads
Exonic Part Number
Samples
cardiac
edl
soleus

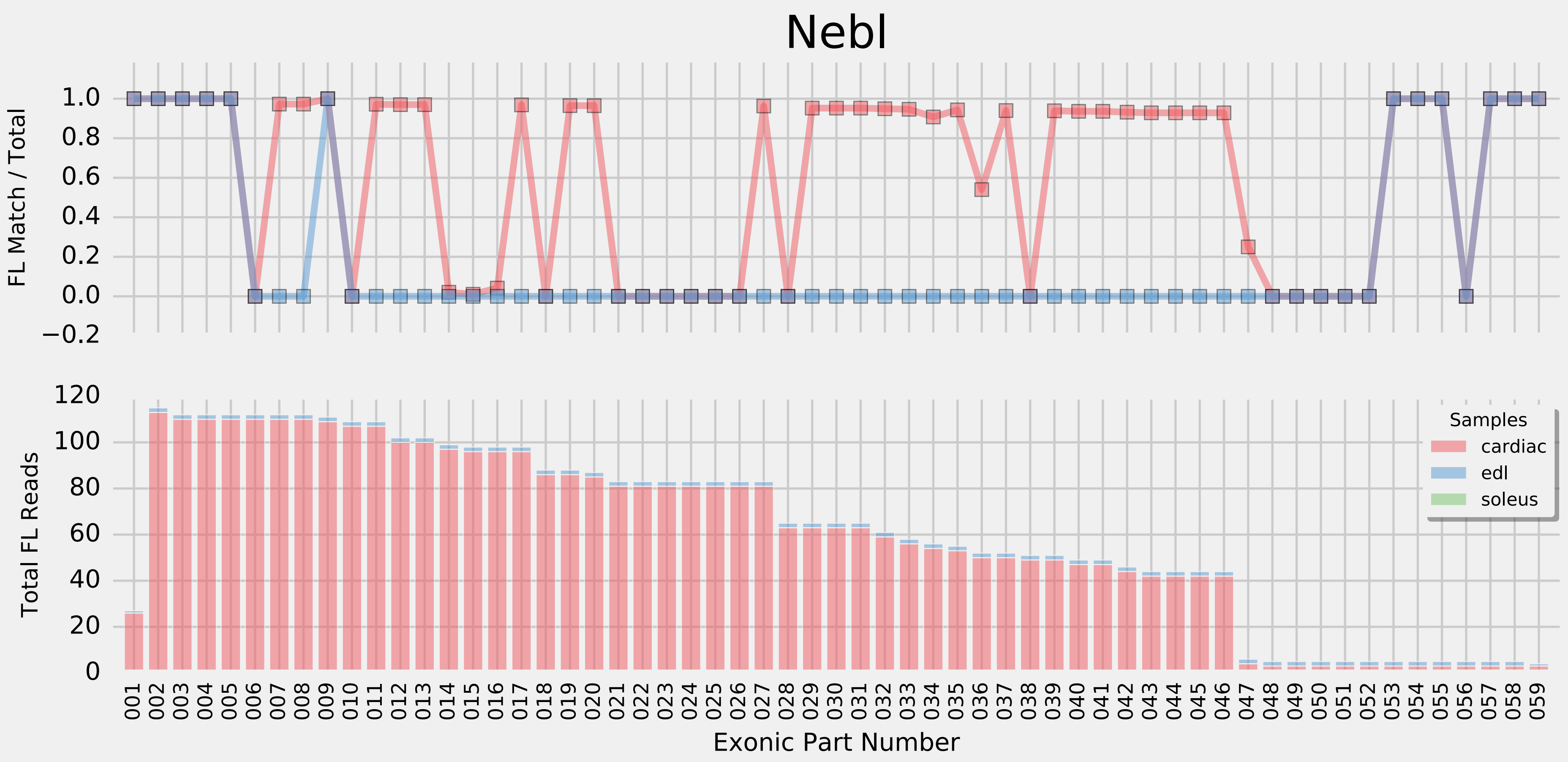
Nebl
FL Match / Total
1.0
0.8
0.6
0.4
0.2
0.0
−0.2
Total FL Reads
120
100
80
60
40
20
0
Samples
cardiac
edl
soleus
001 002 003 004 005 006 007 008 009 010 011 012 013 014 015 016 017 018 019 020 021 022 023 024 025 026 027 028 029 030 031 032 033 034 035 036 037 038 039 040 041 042 043 044 045 046 047 048 049 050 051 052 053 054 055 056 057 058 059
Exonic Part Number

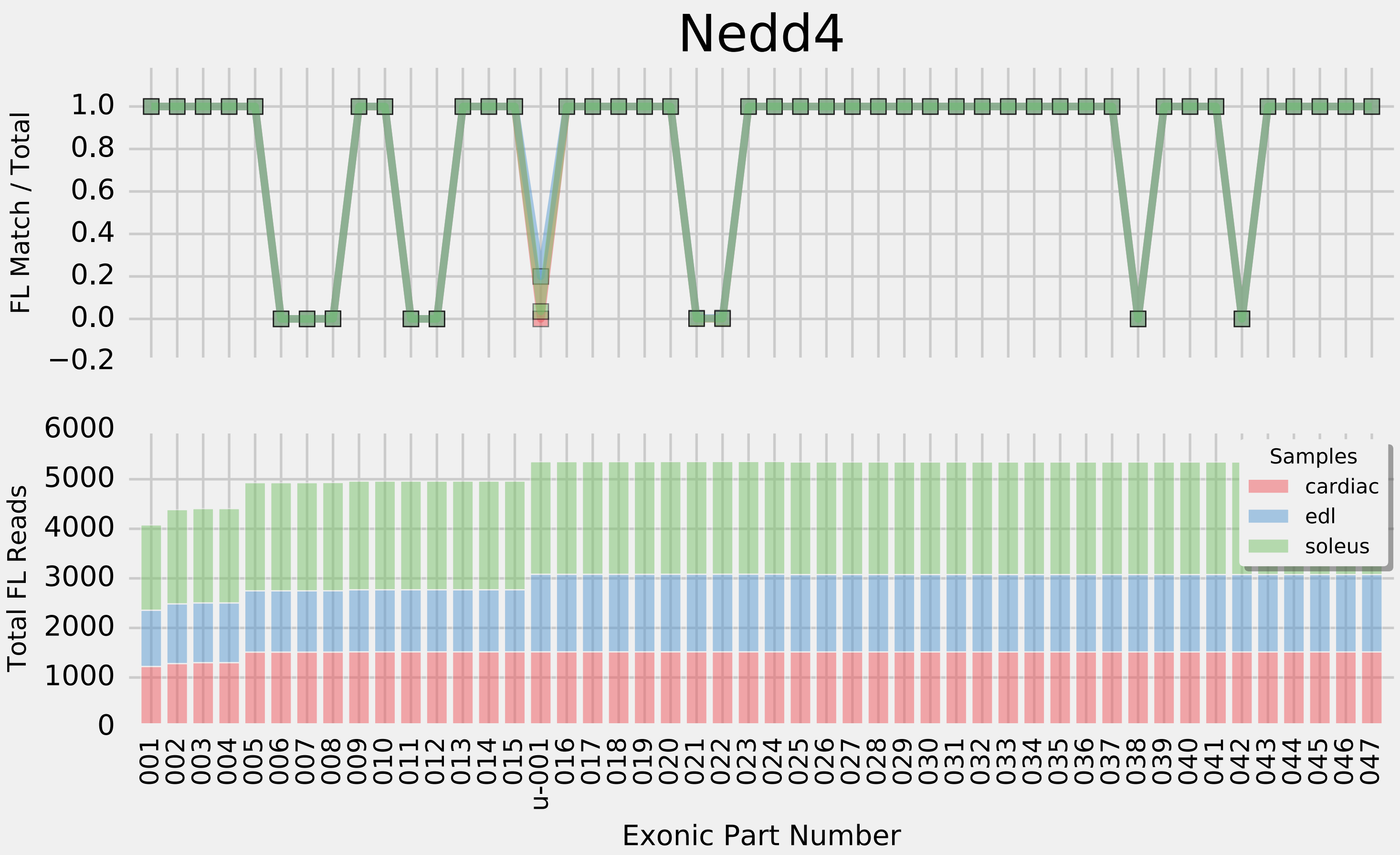

Nedd4
FL Match / Total
1.0
0.8
0.6
0.4
0.2
0.0
−0.2
Total FL Reads
6000
5000
4000
3000
2000
1000
0
Samples
cardiac
edl
soleus
001 002 003 004 005 006 007 008 009 010 011 012 013 014 015 u-001 016 017 018 019 020 021 022 023 024 025 026 027 028 029 030 031 032 033 034 035 036 037 038 039 040 041 042 043 044 045 046 047
Exonic Part Number

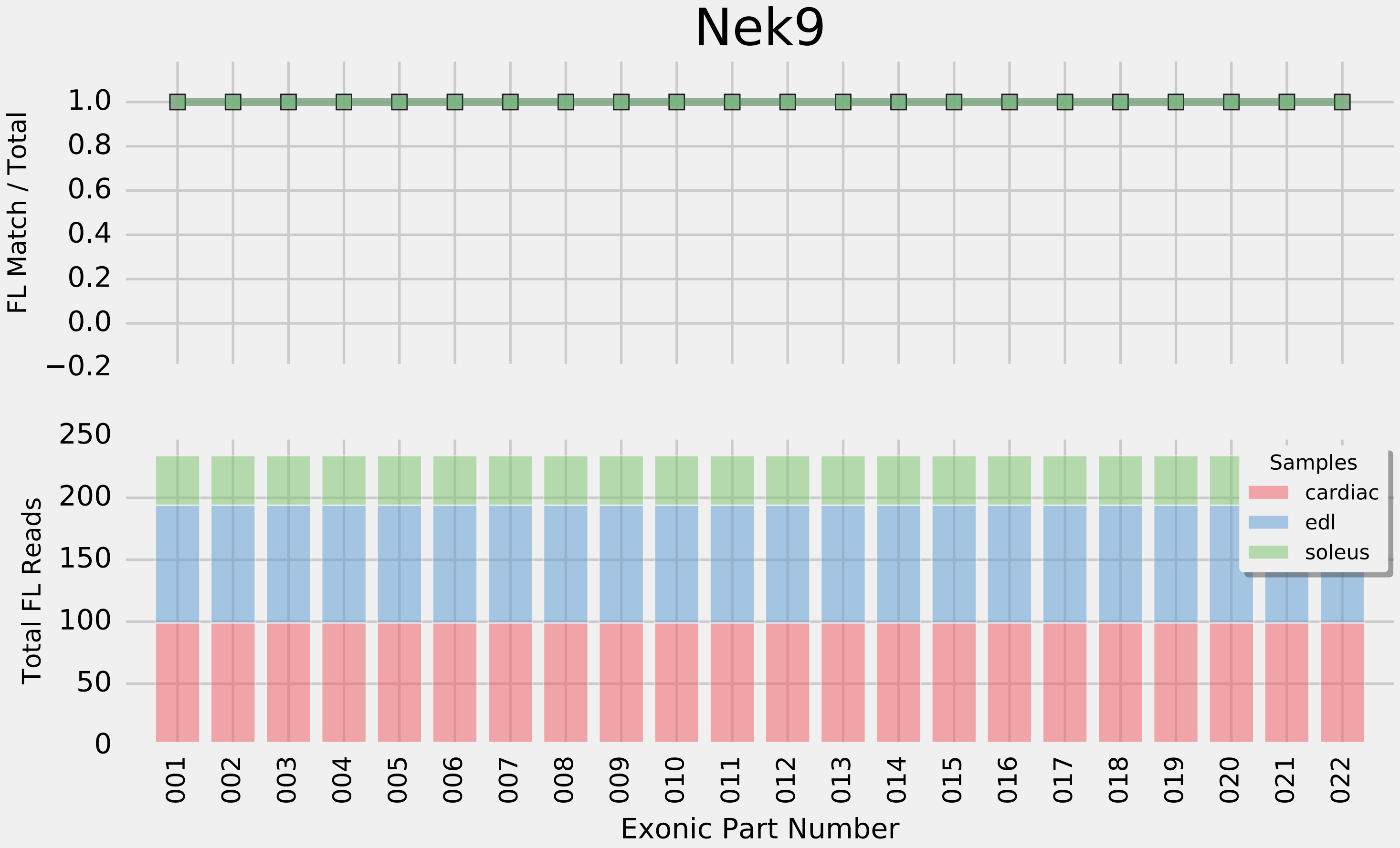

Nek9
FL Match / Total
1.0
0.8
0.6
0.4
0.2
0.0
−0.2
Total FL Reads
250
200
150
100
50
0
Samples
cardiac
edl
soleus
001
002
003
004
005
006
007
008
009
010
011
012
013
014
015
016
017
018
019
020
021
022
Exonic Part Number

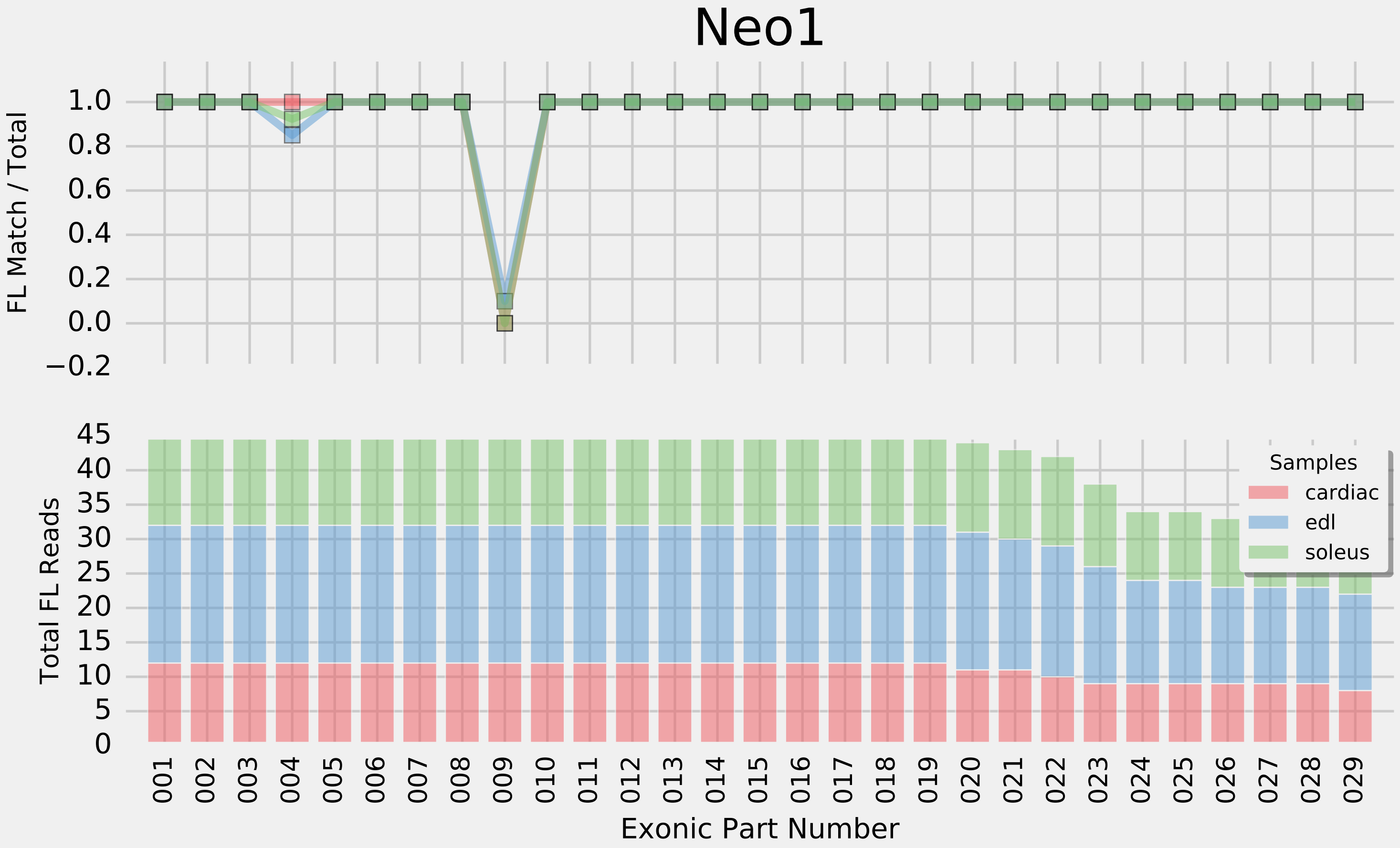

Neo1
FL Match / Total
1.0
0.8
0.6
0.4
0.2
0.0
−0.2
Total FL Reads
45
40
35
30
25
20
15
10
5
0
001
002
003
004
005
006
007
008
009
010
011
012
013
014
015
016
017
018
019
020
021
022
023
024
025
026
027
028
029
Exonic Part Number
Samples
cardiac
edl
soleus

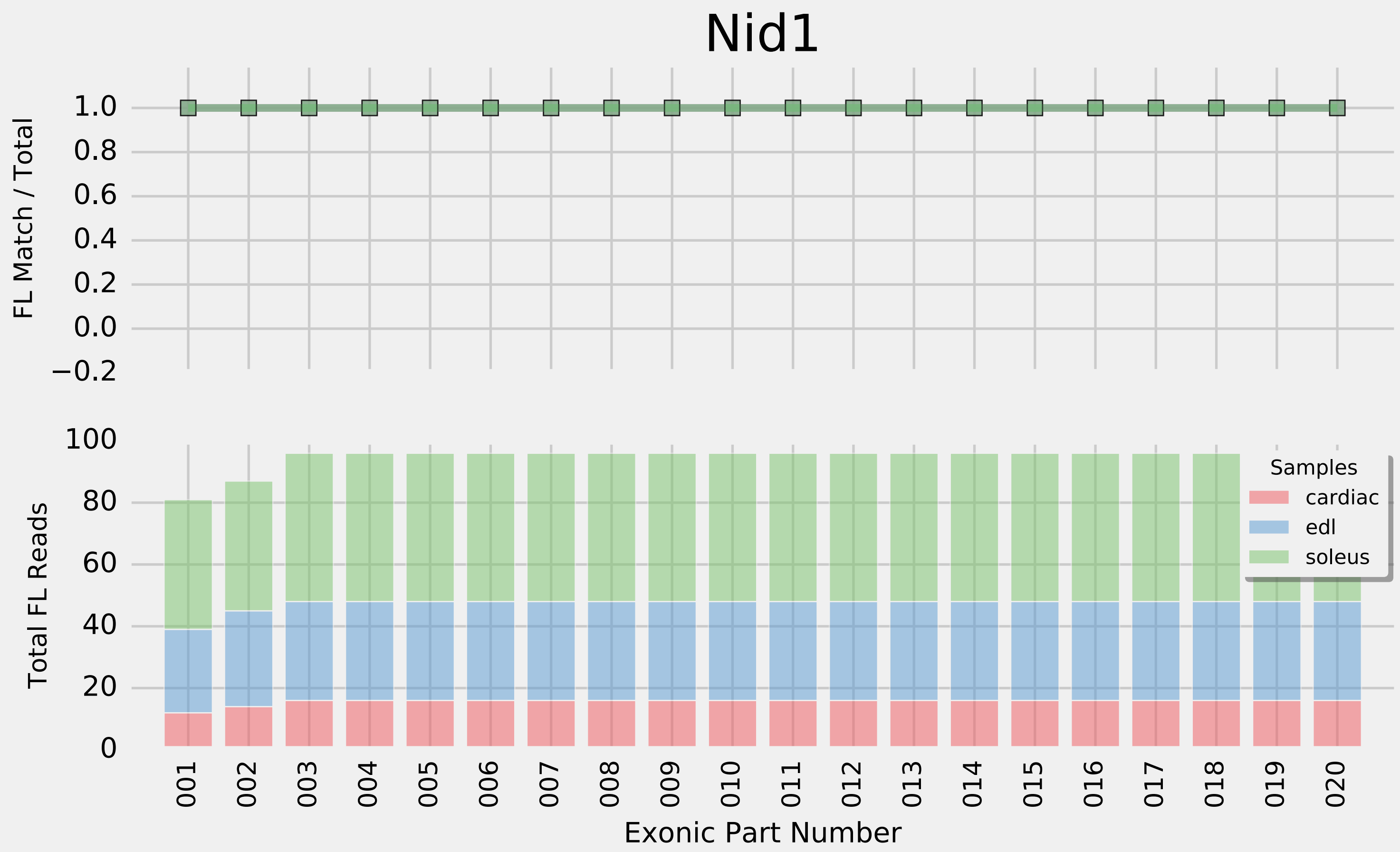
Nid1
FL Match / Total
1.0
0.8
0.6
0.4
0.2
0.0
−0.2
Total FL Reads
100
80
60
40
20
0
Samples
cardiac
edl
soleus
001
002
003
004
005
006
007
008
009
010
011
012
013
014
015
016
017
018
019
020
Exonic Part Number

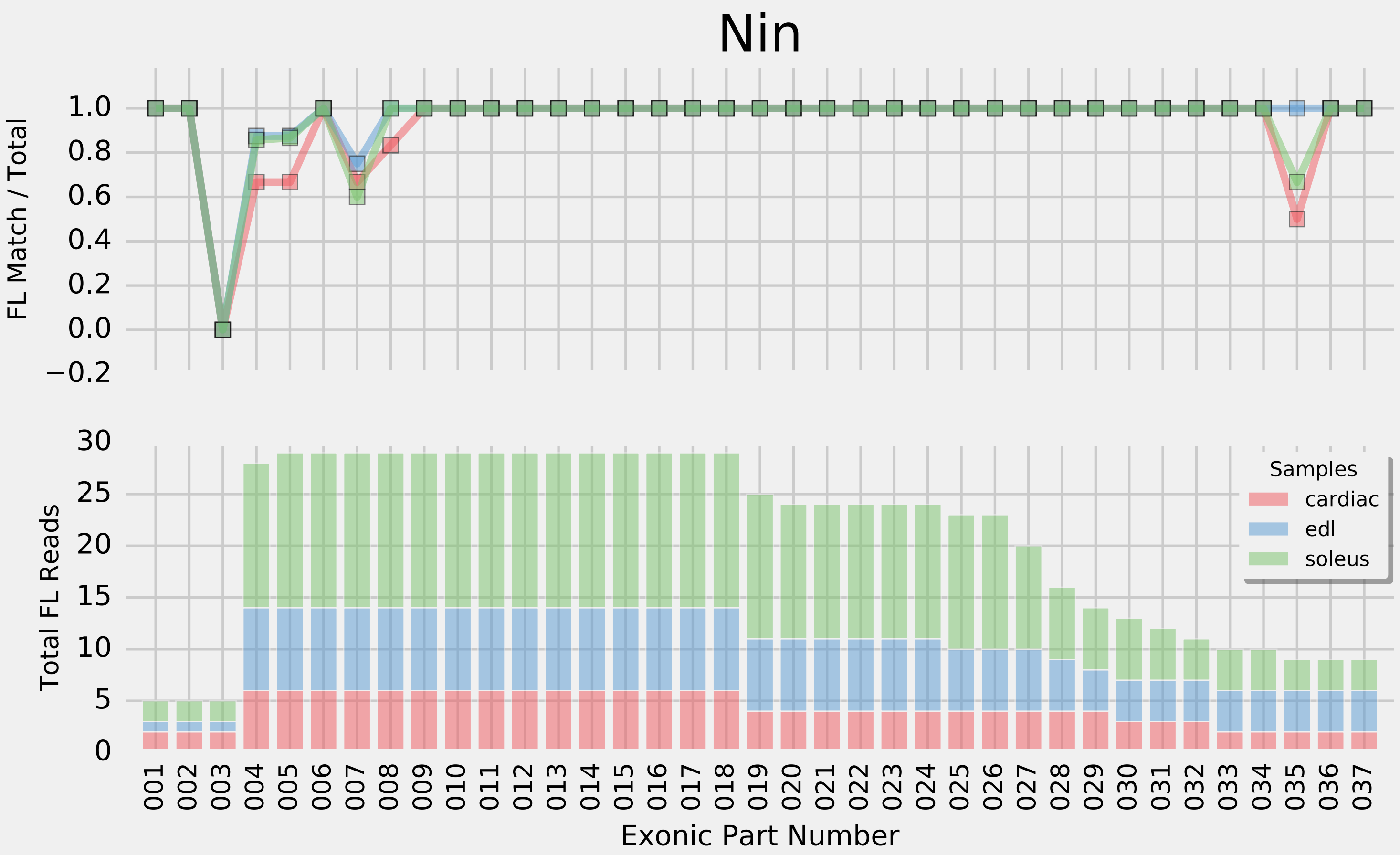

Nin
FL Match / Total
1.0
0.8
0.6
0.4
0.2
0.0
−0.2
Total FL Reads
30
25
20
15
10
5
0
Samples
cardiac
edl
soleus
001 002 003 004 005 006 007 008 009 010 011 012 013 014 015 016 017 018 019 020 021 022 023 024 025 026 027 028 029 030 031 032 033 034 035 036 037
Exonic Part Number

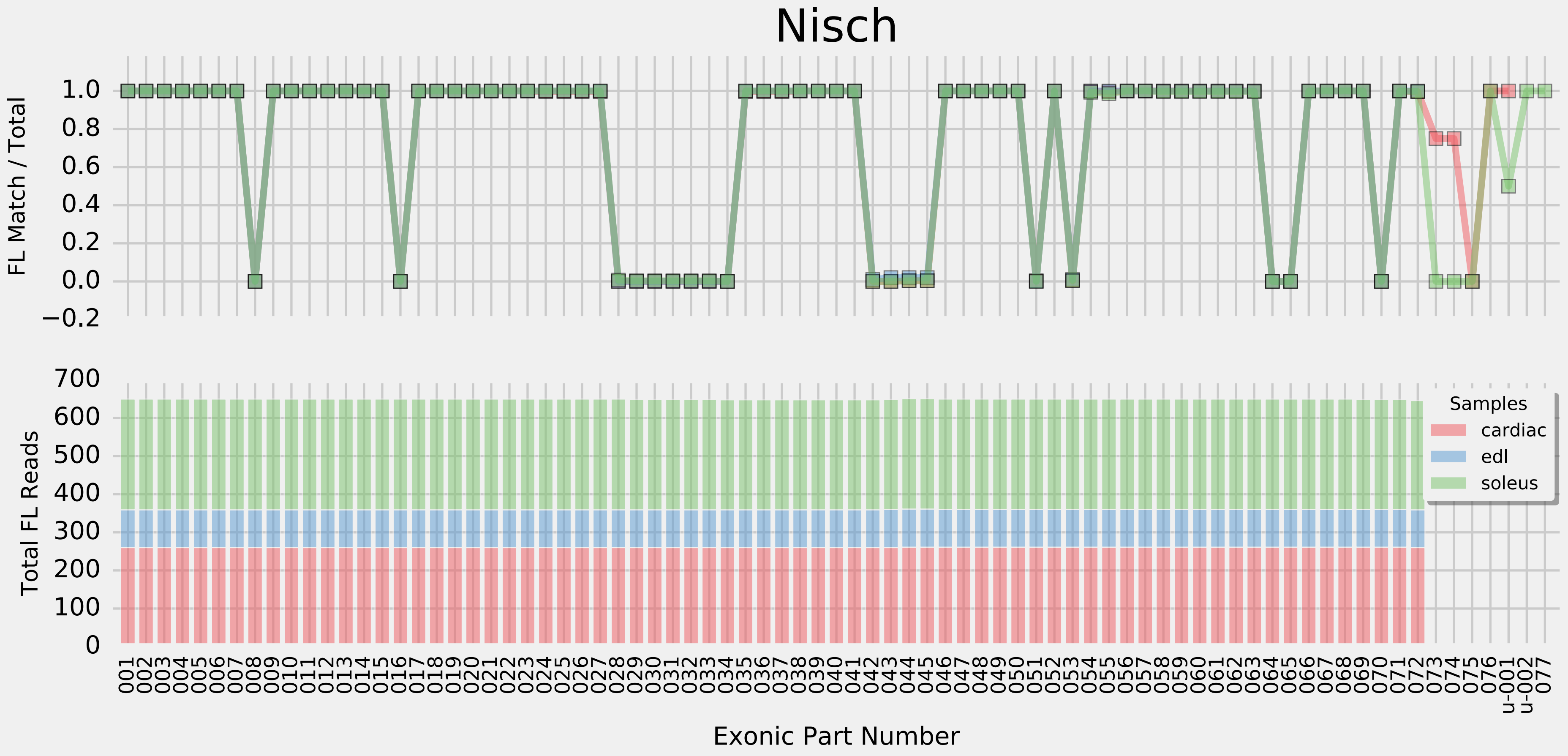
Nisch
FL Match / Total
1.0
0.8
0.6
0.4
0.2
0.0
−0.2
Total FL Reads
700
600
500
400
300
200
100
0
Samples
cardiac
edl
soleus
001 002 003 004 005 006 007 008 009 010 011 012 013 014 015 016 017 018 019 020 021 022 023 024 025 026 027 028 029 030 031 032 033 034 035 036 037 038 039 040 041 042 043 044 045 046 047 048 049 050 051 052 053 054 055 056 057 058 059 060 061 062 063 064 065 066 067 068 069 070 071 072 073 074 075 076 u-001 u-002 077
Exonic Part Number

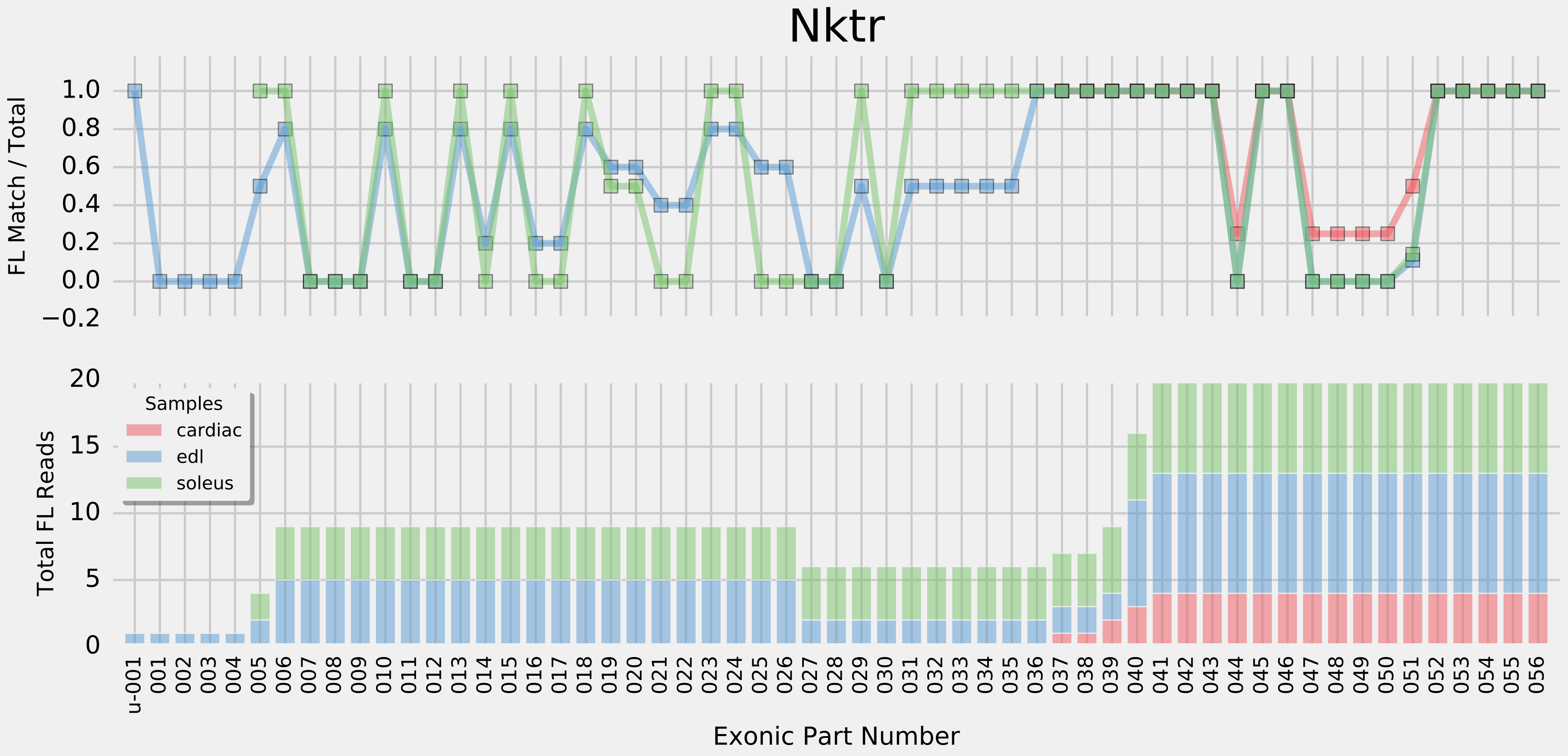
Nktr
FL Match / Total
1.0
0.8
0.6
0.4
0.2
0.0
−0.2
Total FL Reads
20
15
10
5
0
Samples
cardiac
edl
soleus
u-001
001
002
003
004
005
006
007
008
009
010
011
012
013
014
015
016
017
018
019
020
021
022
023
024
025
026
027
028
029
030
031
032
033
034
035
036
037
038
039
040
041
042
043
044
045
046
047
048
049
050
051
052
053
054
055
056
Exonic Part Number

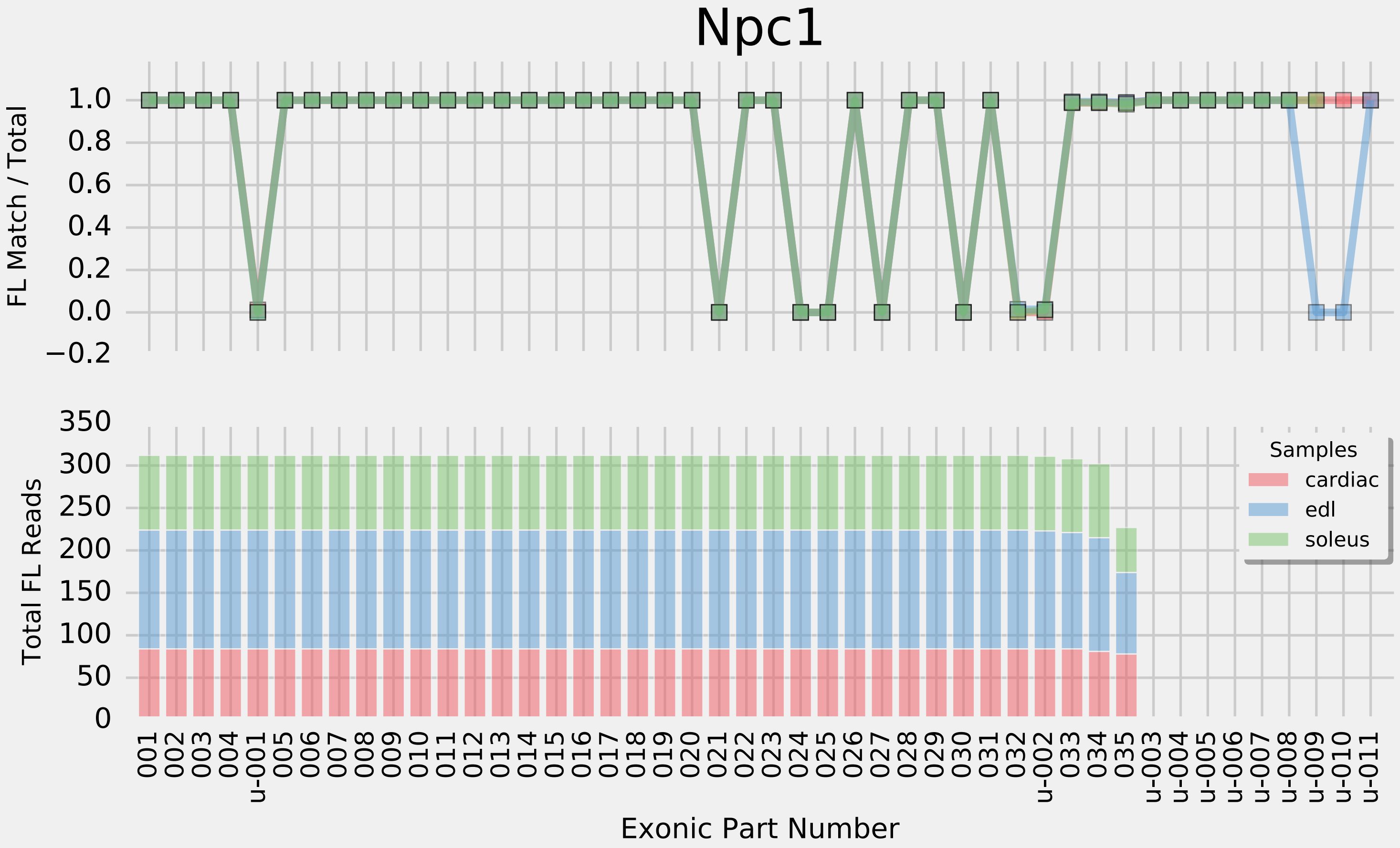
Npc1
FL Match / Total
1.0
0.8
0.6
0.4
0.2
0.0
−0.2
350
300
250
200
150
100
50
0
Total FL Reads
Samples
cardiac
edl
soleus
001
002
003
004
u-001
005
006
007
008
009
010
011
012
013
014
015
016
017
018
019
020
021
022
023
024
025
026
027
028
029
030
031
032
u-002
033
034
035
u-003
u-004
u-005
u-006
u-007
u-008
u-009
u-010
u-011
Exonic Part Number

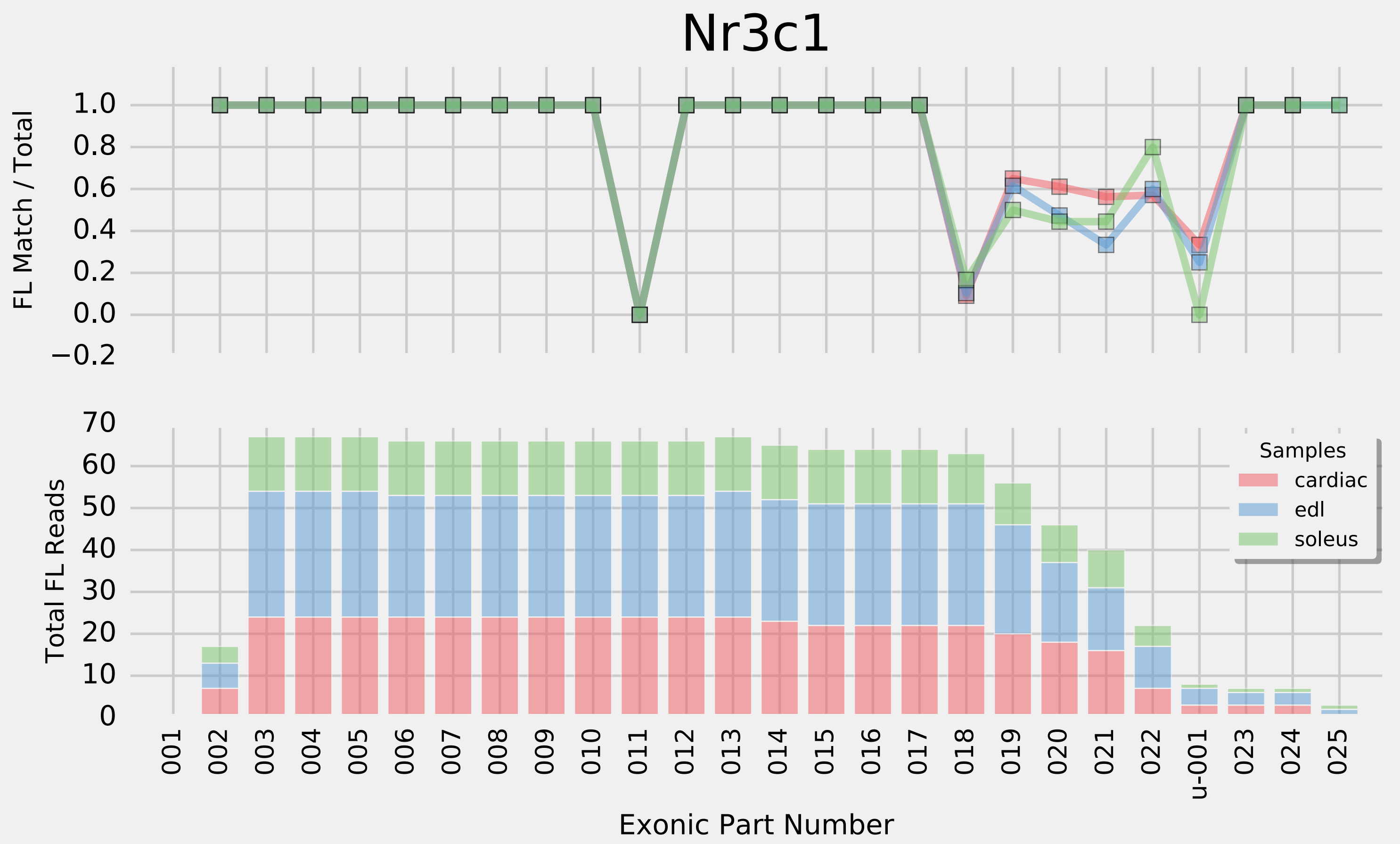
Nr3c1
FL Match / Total
1.0
0.8
0.6
0.4
0.2
0.0
−0.2
Total FL Reads
70
60
50
40
30
20
10
0
Samples
cardiac
edl
soleus
001
002
003
004
005
006
007
008
009
010
011
012
013
014
015
016
017
018
019
020
021
022
u-001
023
024
025
Exonic Part Number

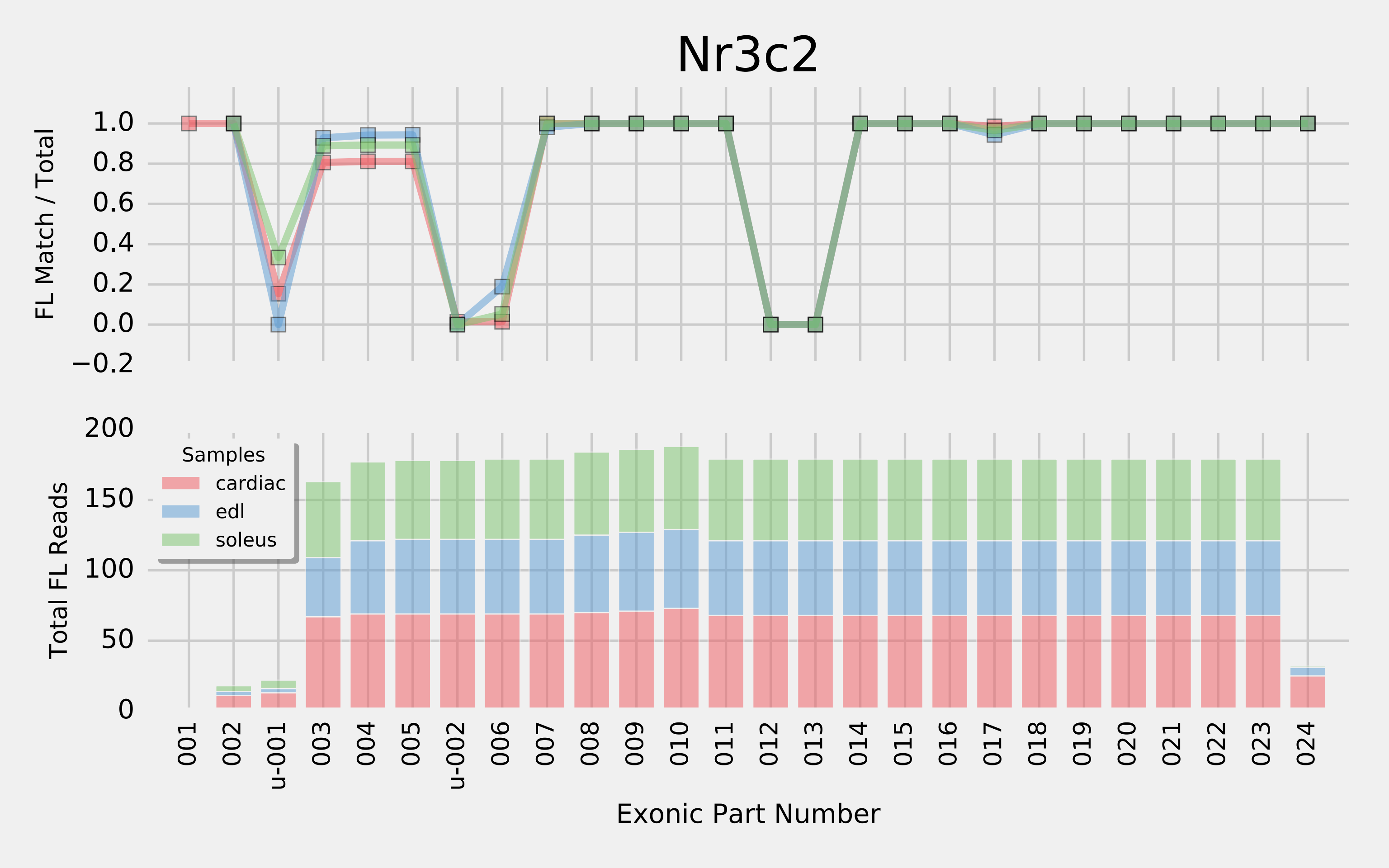

Nr3c2
FL Match / Total
1.0
0.8
0.6
0.4
0.2
0.0
−0.2
Total FL Reads
200
150
100
50
0
Samples
cardiac
edl
soleus
001
002
u-001
003
004
005
u-002
006
007
008
009
010
011
012
013
014
015
016
017
018
019
020
021
022
023
024
Exonic Part Number

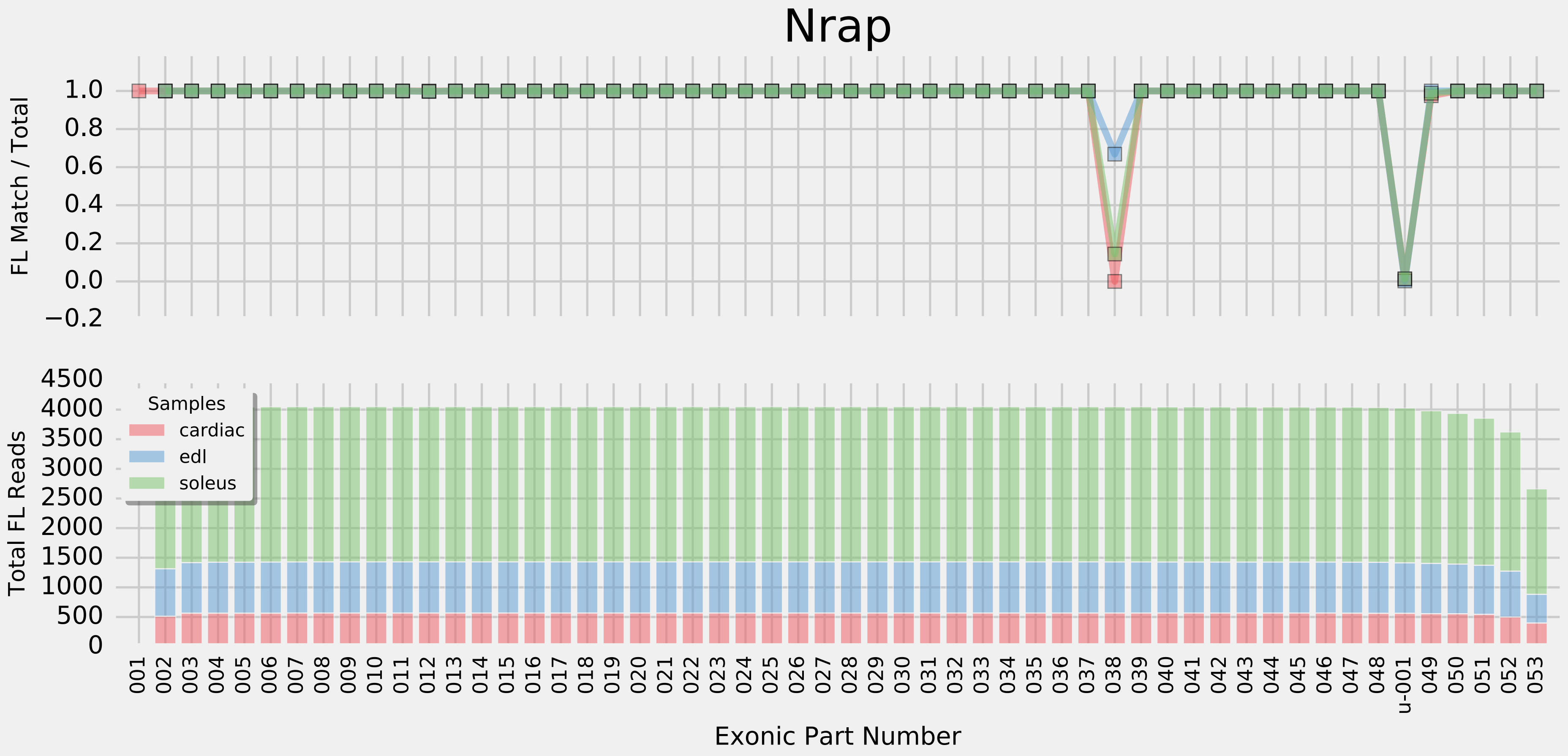

Nrap
FL Match / Total
1.0
0.8
0.6
0.4
0.2
0.0
−0.2
Total FL Reads
4500
4000
3500
3000
2500
2000
1500
1000
500
0
Samples
cardiac
edl
soleus
001 002 003 004 005 006 007 008 009 010 011 012 013 014 015 016 017 018 019 020 021 022 023 024 025 026 027 028 029 030 031 032 033 034 035 036 037 038 039 040 041 042 043 044 045 046 047 048 u-001 049 050 051 052 053
Exonic Part Number

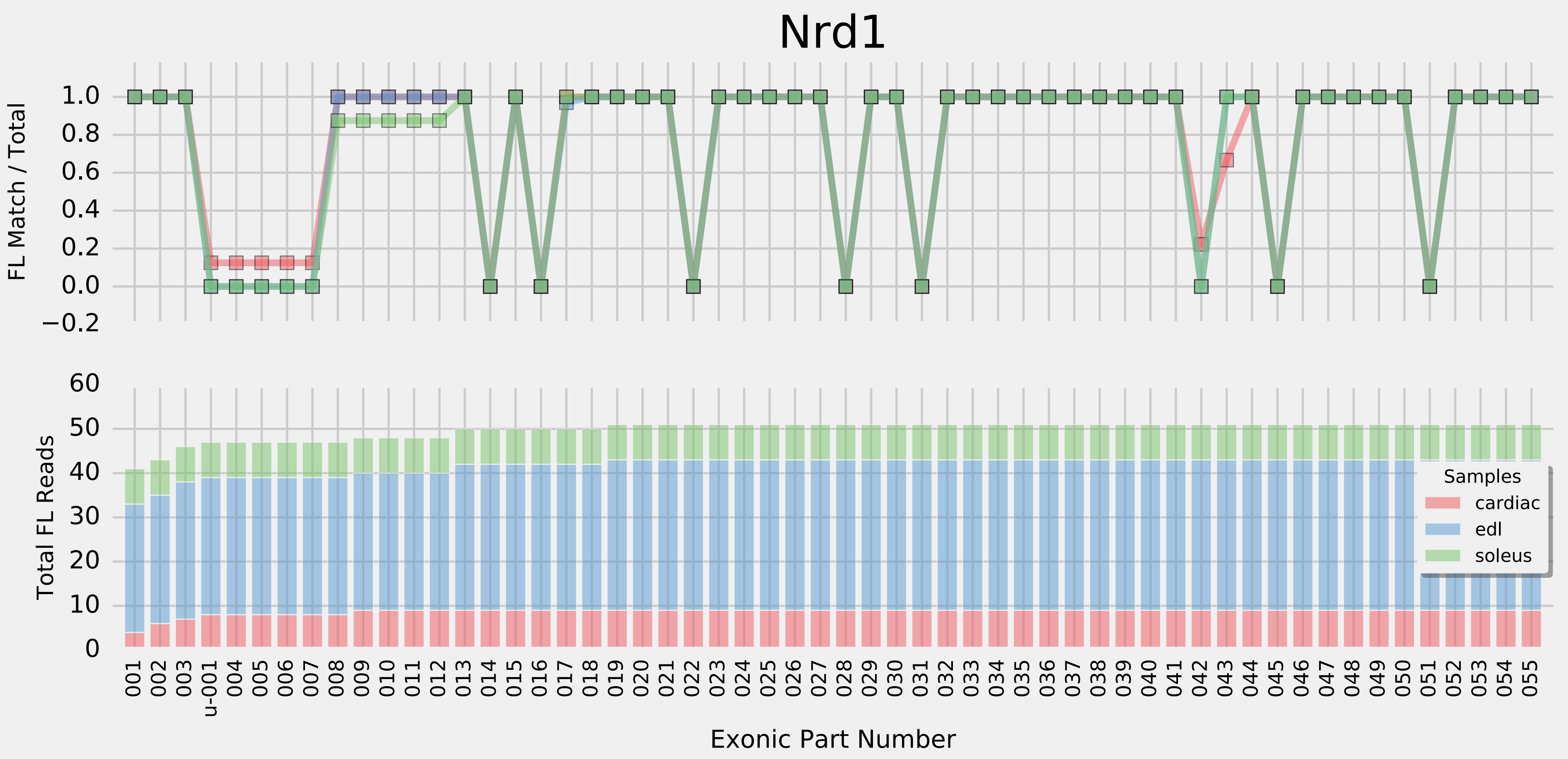

Nrd1
FL Match / Total
Total FL Reads
Exonic Part Number
Samples
cardiac
edl
soleus
001
002
003
u-001
004
005
006
007
008
009
010
011
012
013
014
015
016
017
018
019
020
021
022
023
024
025
026
027
028
029
030
031
032
033
034
035
036
037
038
039
040
041
042
043
044
045
046
047
048
049
050
051
052
053
054
055

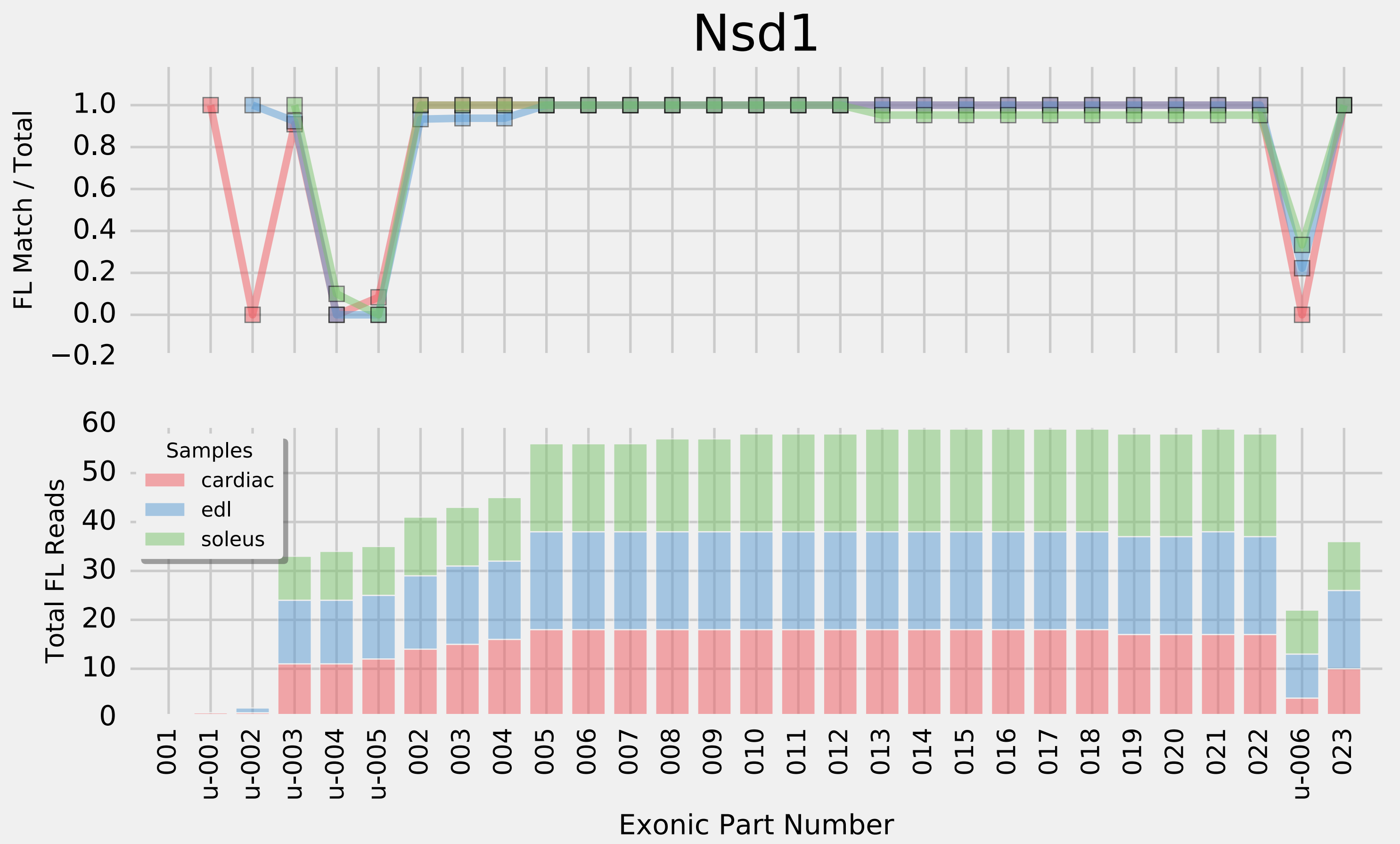

Nsd1
FL Match / Total
1.0
0.8
0.6
0.4
0.2
0.0
−0.2
60
50
40
30
20
10
0
Samples
cardiac
edl
soleus
Total FL Reads
001
u-001
u-002
u-003
u-004
u-005
002
003
004
005
006
007
008
009
010
011
012
013
014
015
016
017
018
019
020
021
022
u-006
023
Exonic Part Number

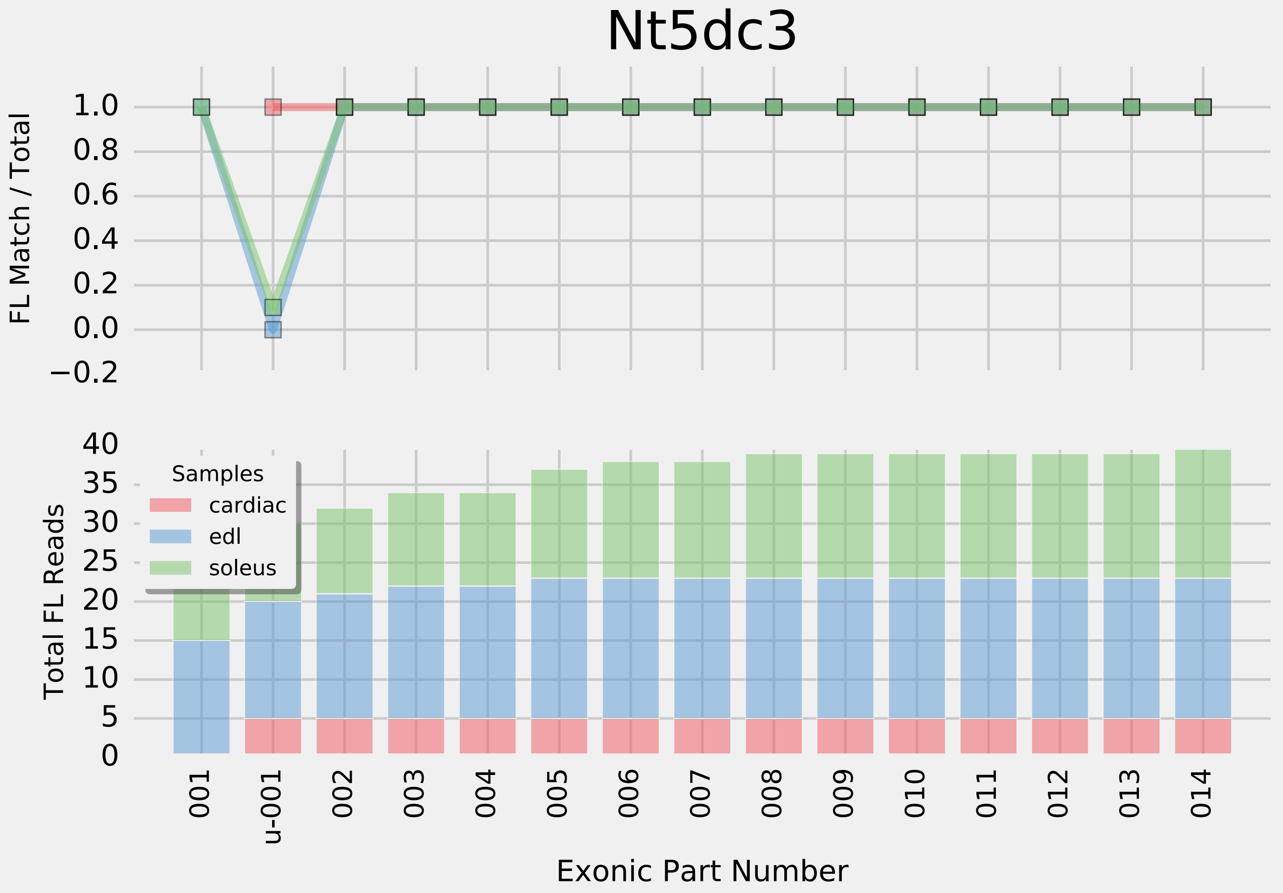

Nt5dc3
FL Match / Total
1.0
0.8
0.6
0.4
0.2
0.0
−0.2
Total FL Reads
40
35
30
25
20
15
10
5
0
Samples
cardiac
edl
soleus
001
u-001
002
003
004
005
006
007
008
009
010
011
012
013
014
Exonic Part Number

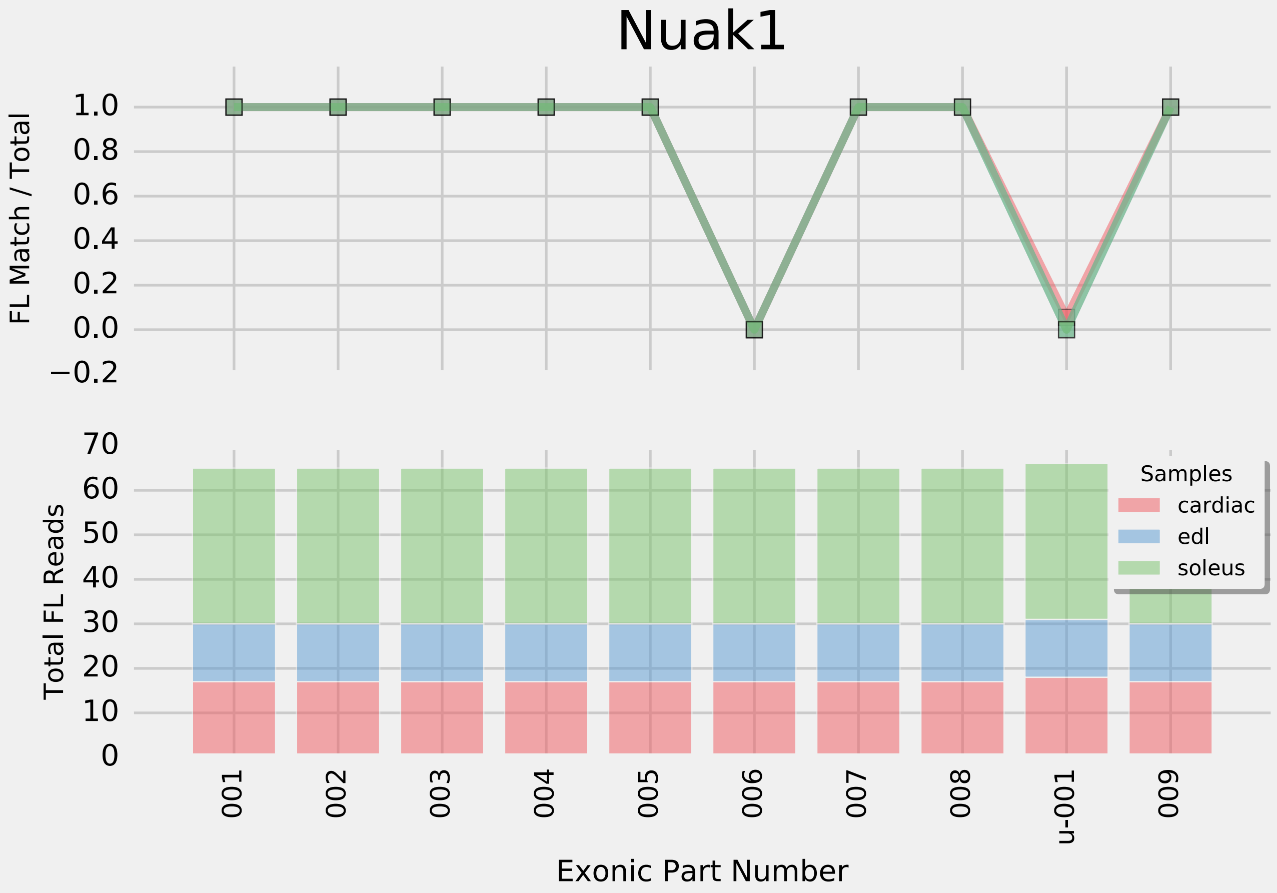

Nuak1
FL Match / Total
1.0
0.8
0.6
0.4
0.2
0.0
−0.2
Total FL Reads
70
60
50
40
30
20
10
0
Samples
cardiac
edl
soleus
001
002
003
004
005
006
007
008
u-001
009
Exonic Part Number

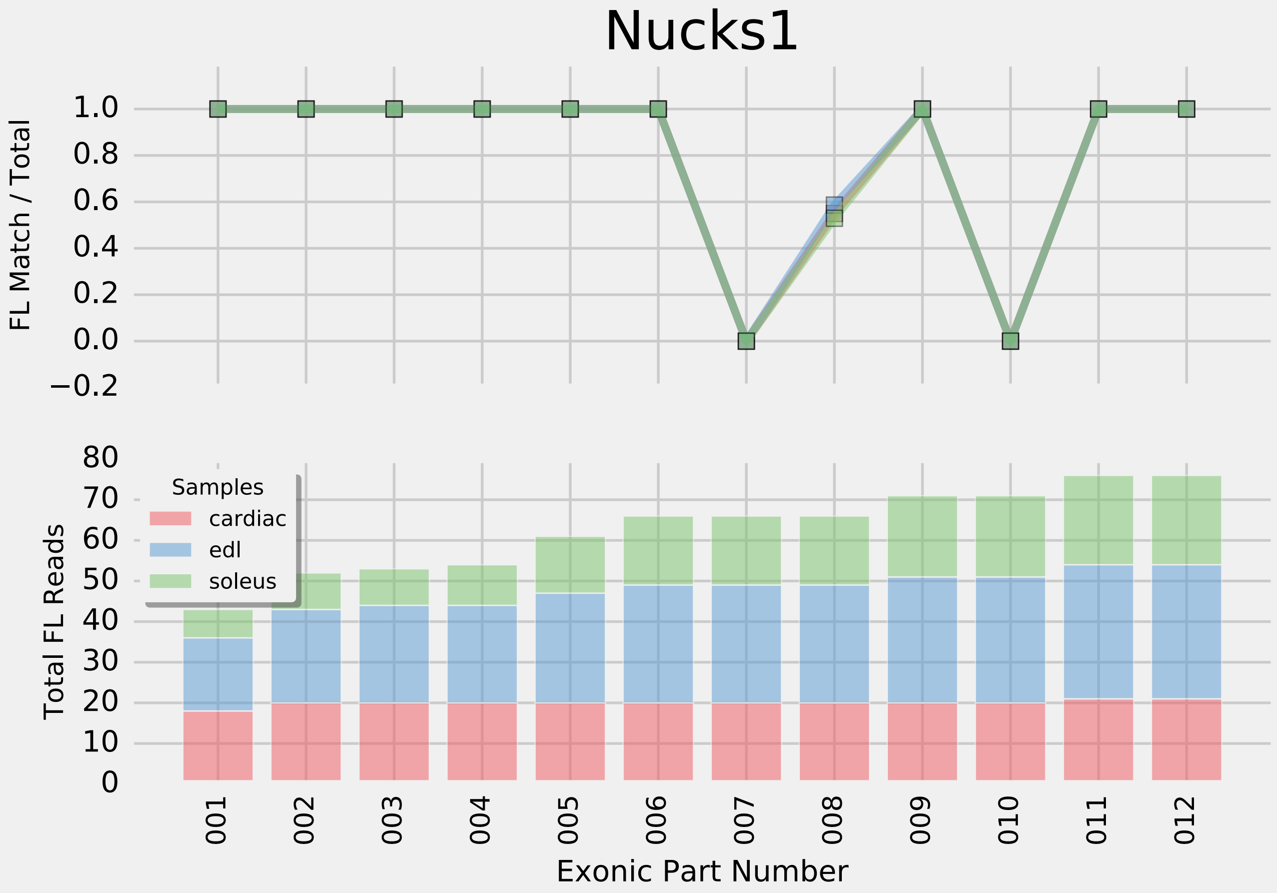

Nucks1
FL Match / Total
1.0
0.8
0.6
0.4
0.2
0.0
−0.2
Total FL Reads
80
70
60
50
40
30
20
10
0
Samples
cardiac
edl
soleus
001
002
003
004
005
006
007
008
009
010
011
012
Exonic Part Number

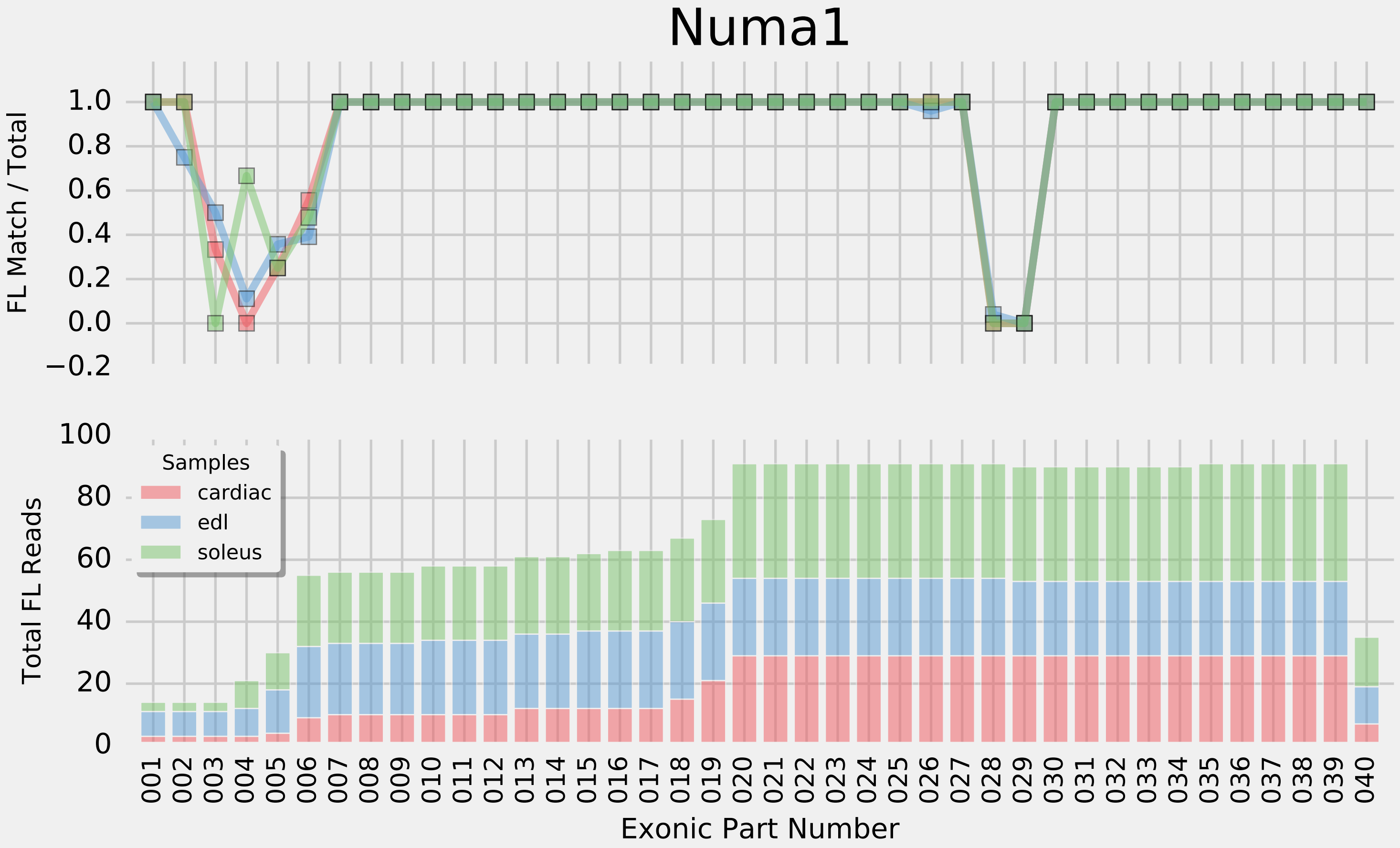

Numa1
FL Match / Total
1.0
0.8
0.6
0.4
0.2
0.0
−0.2
Total FL Reads
100
80
60
40
20
0
Samples
cardiac
edl
soleus
001 002 003 004 005 006 007 008 009 010 011 012 013 014 015 016 017 018 019 020 021 022 023 024 025 026 027 028 029 030 031 032 033 034 035 036 037 038 039 040
Exonic Part Number

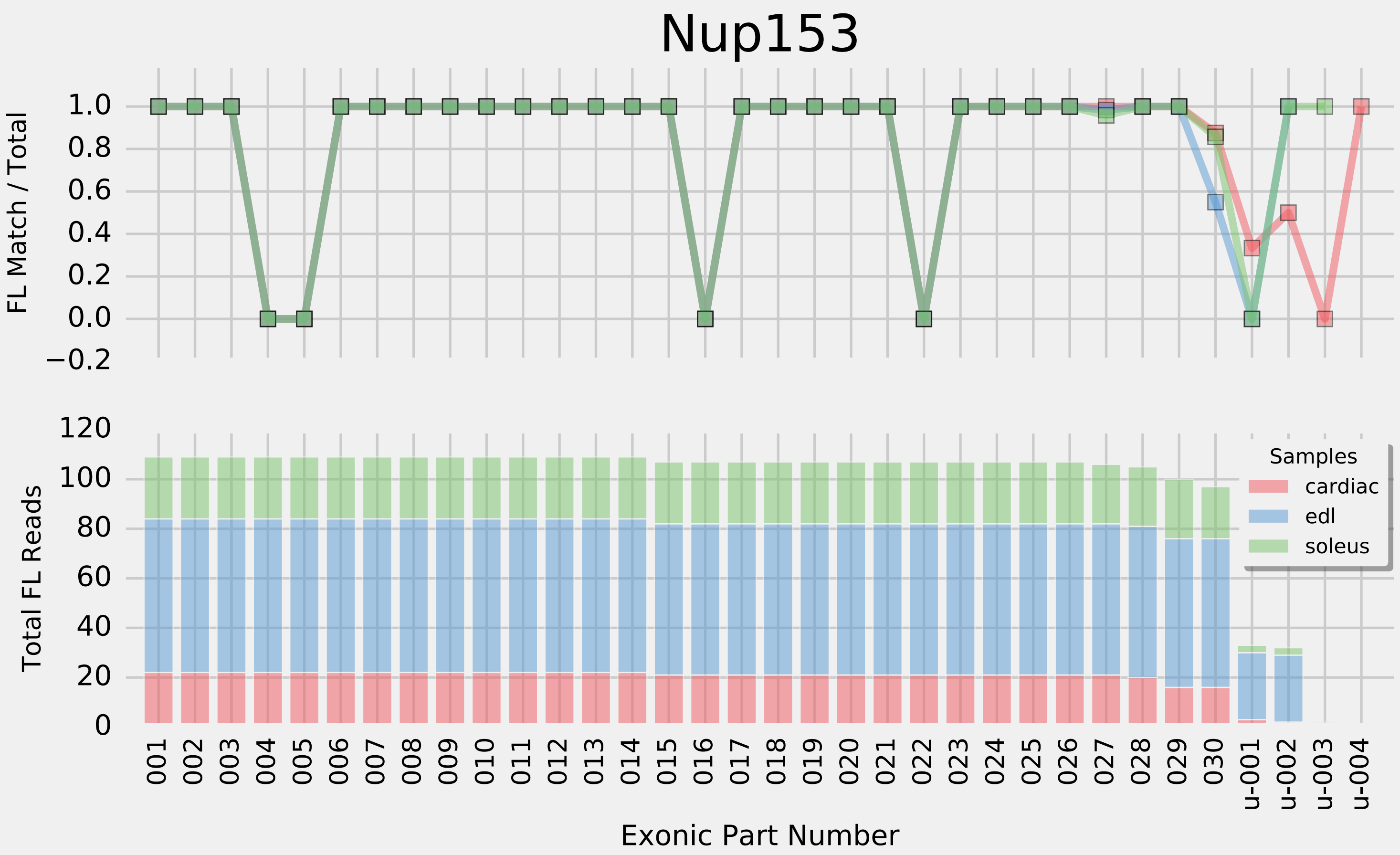

Nup153
FL Match / Total
1.0
0.8
0.6
0.4
0.2
0.0
−0.2
Total FL Reads
120
100
80
60
40
20
0
Samples
cardiac
edl
soleus
001 002 003 004 005 006 007 008 009 010 011 012 013 014 015 016 017 018 019 020 021 022 023 024 025 026 027 028 029 030 u-001 u-002 u-003 u-004
Exonic Part Number

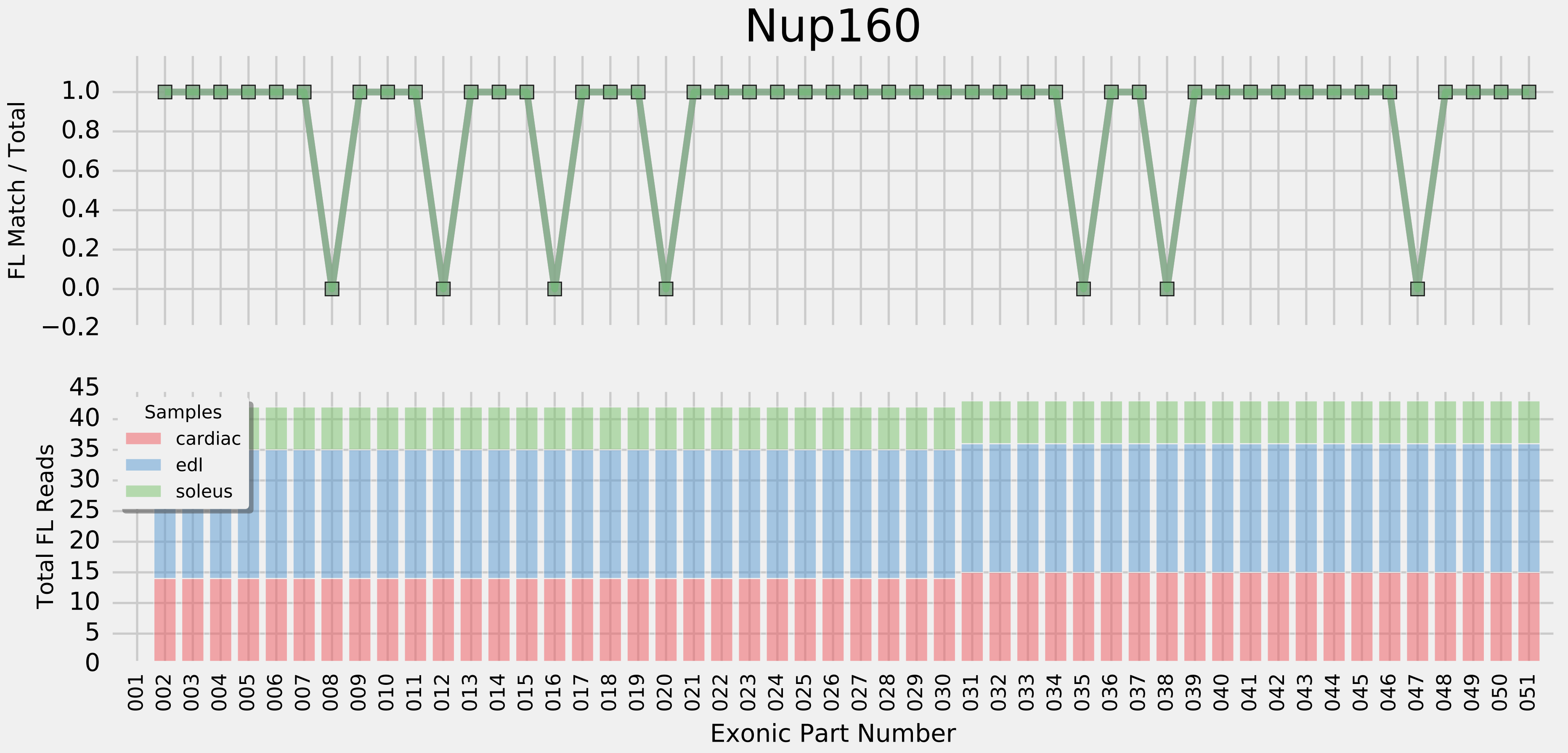

Nup160
FL Match / Total
1.0
0.8
0.6
0.4
0.2
0.0
−0.2
Total FL Reads
45
40
35
30
25
20
15
10
5
0
Samples
cardiac
edl
soleus
001 002 003 004 005 006 007 008 009 010 011 012 013 014 015 016 017 018 019 020 021 022 023 024 025 026 027 028 029 030 031 032 033 034 035 036 037 038 039 040 041 042 043 044 045 046 047 048 049 050 051
Exonic Part Number

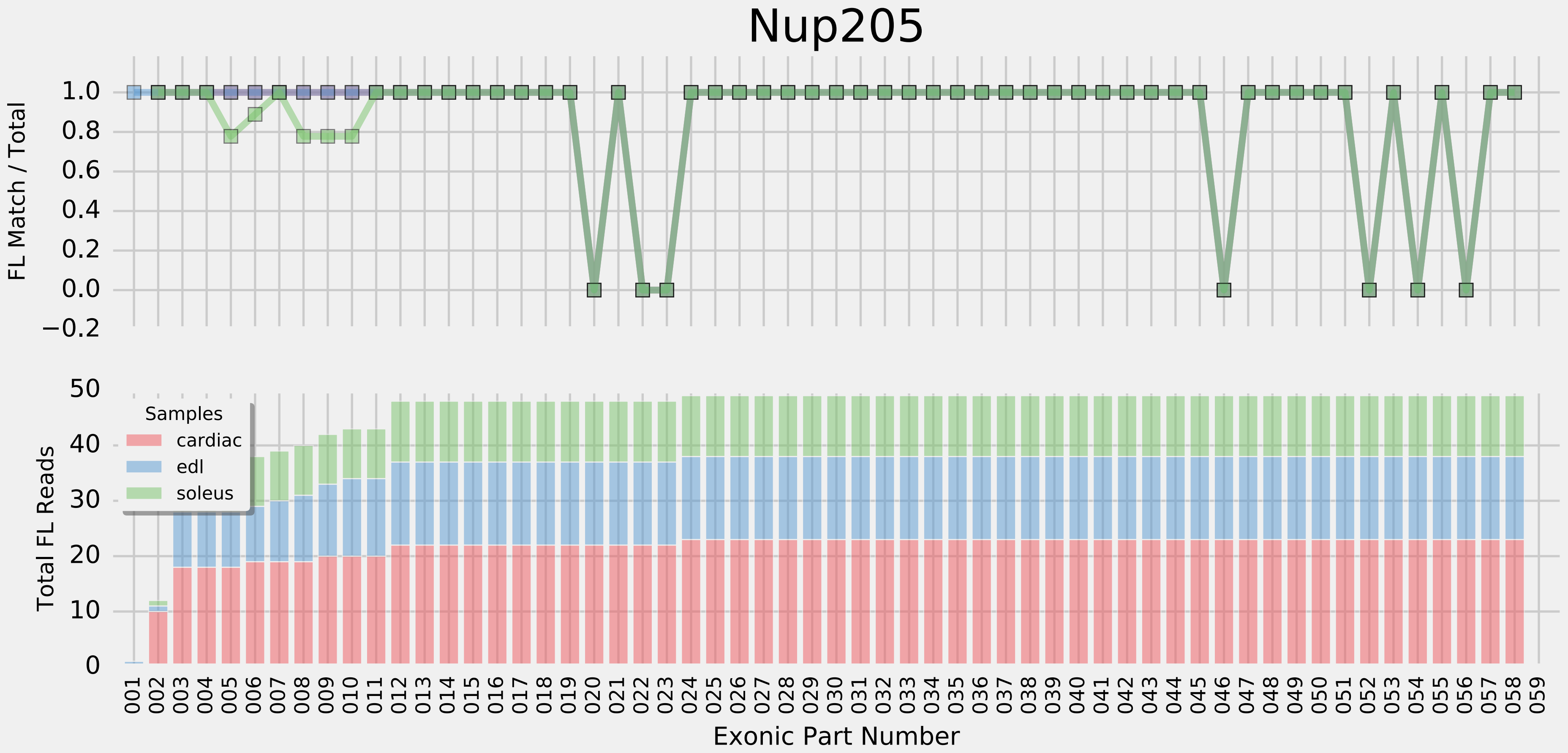

Nup205
FL Match / Total
Total FL Reads
Samples
cardiac
edl
soleus
Exonic Part Number

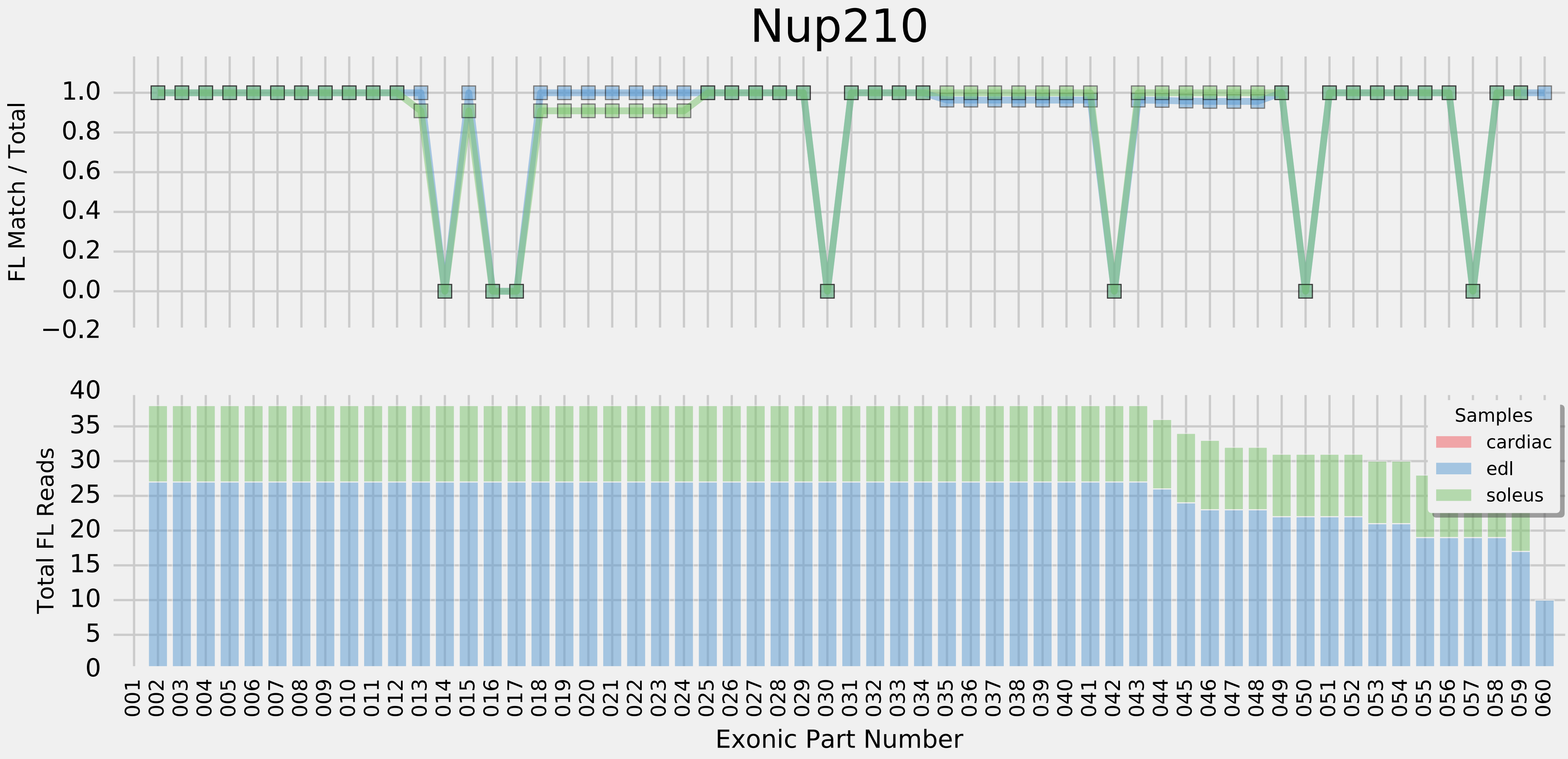

Nup210
FL Match / Total
Total FL Reads
Exonic Part Number
Samples
cardiac
edl
soleus

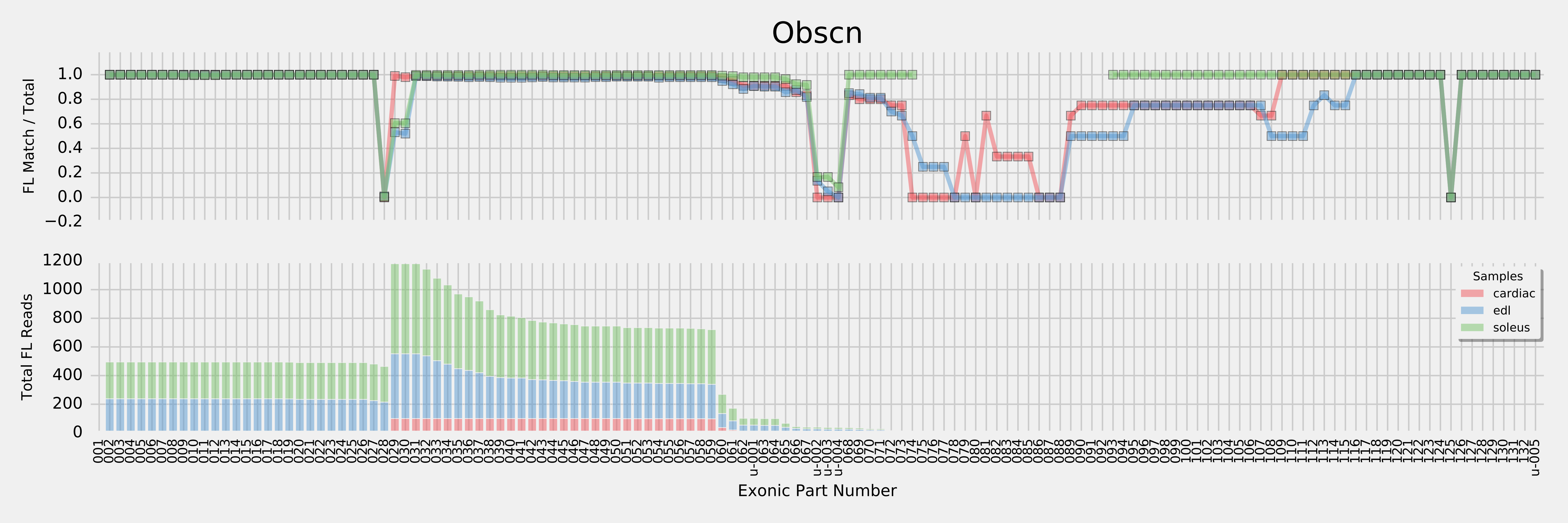

Obscn
FL Match / Total
Total FL Reads
Exonic Part Number
Samples
cardiac
edl
soleus

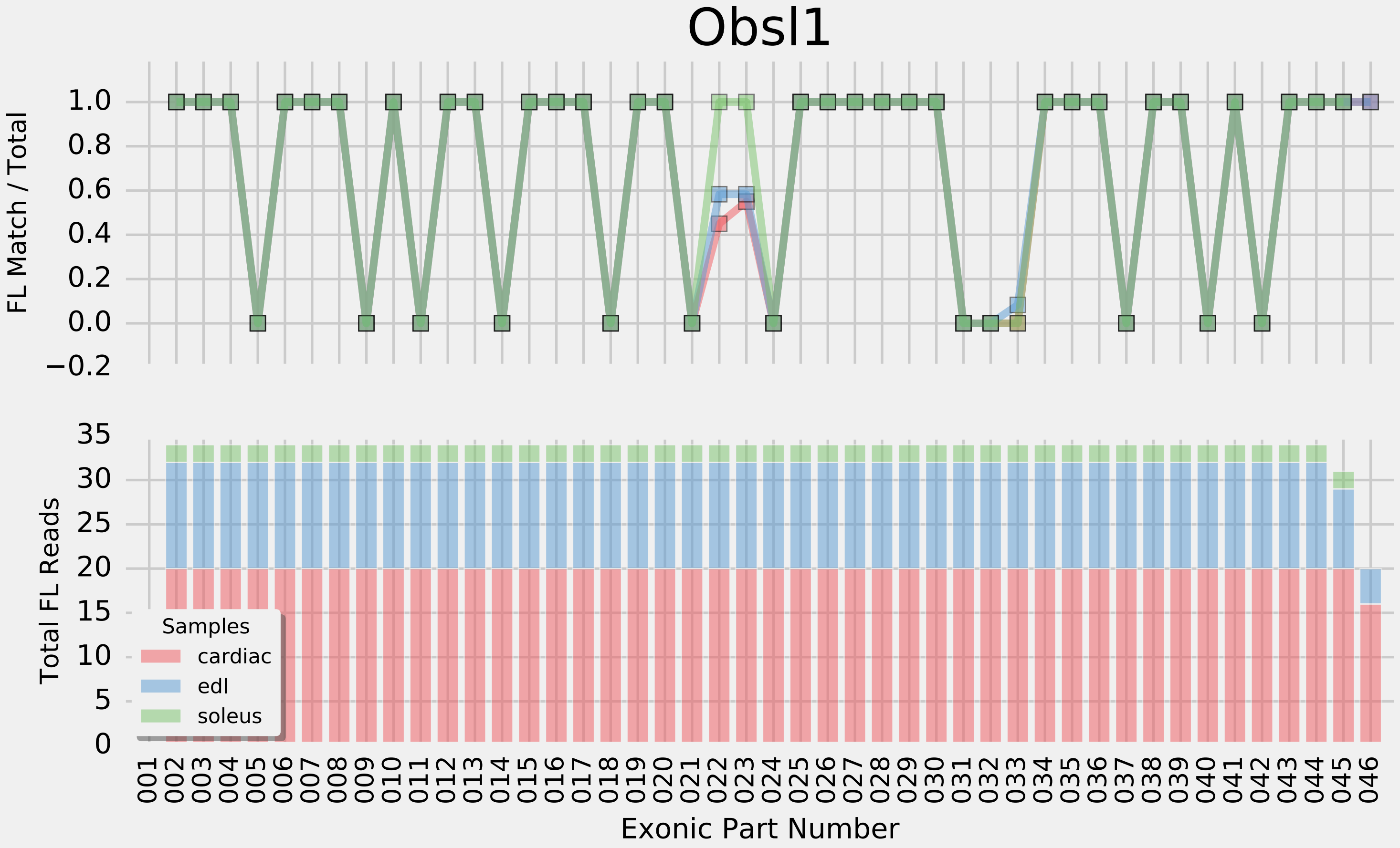

Obsl1
FL Match / Total
1.0
0.8
0.6
0.4
0.2
0.0
−0.2
Total FL Reads
35
30
25
20
15
10
5
0
Samples
cardiac
edl
soleus
001 002 003 004 005 006 007 008 009 010 011 012 013 014 015 016 017 018 019 020 021 022 023 024 025 026 027 028 029 030 031 032 033 034 035 036 037 038 039 040 041 042 043 044 045 046
Exonic Part Number

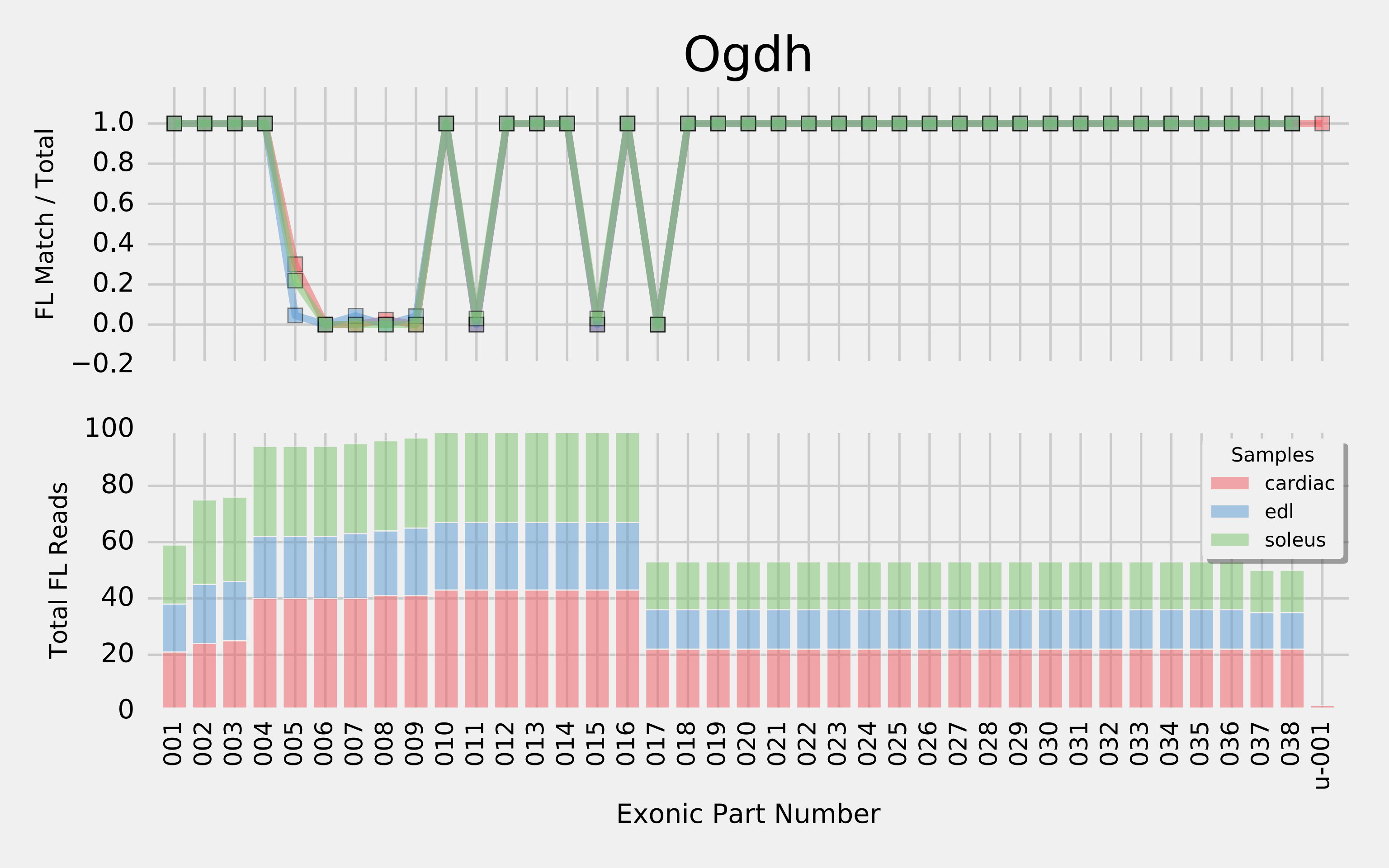

Ogdh
FL Match / Total
1.0
0.8
0.6
0.4
0.2
0.0
−0.2
Total FL Reads
100
80
60
40
20
0
Samples
cardiac
edl
soleus
001 002 003 004 005 006 007 008 009 010 011 012 013 014 015 016 017 018 019 020 021 022 023 024 025 026 027 028 029 030 031 032 033 034 035 036 037 038 u-001
Exonic Part Number

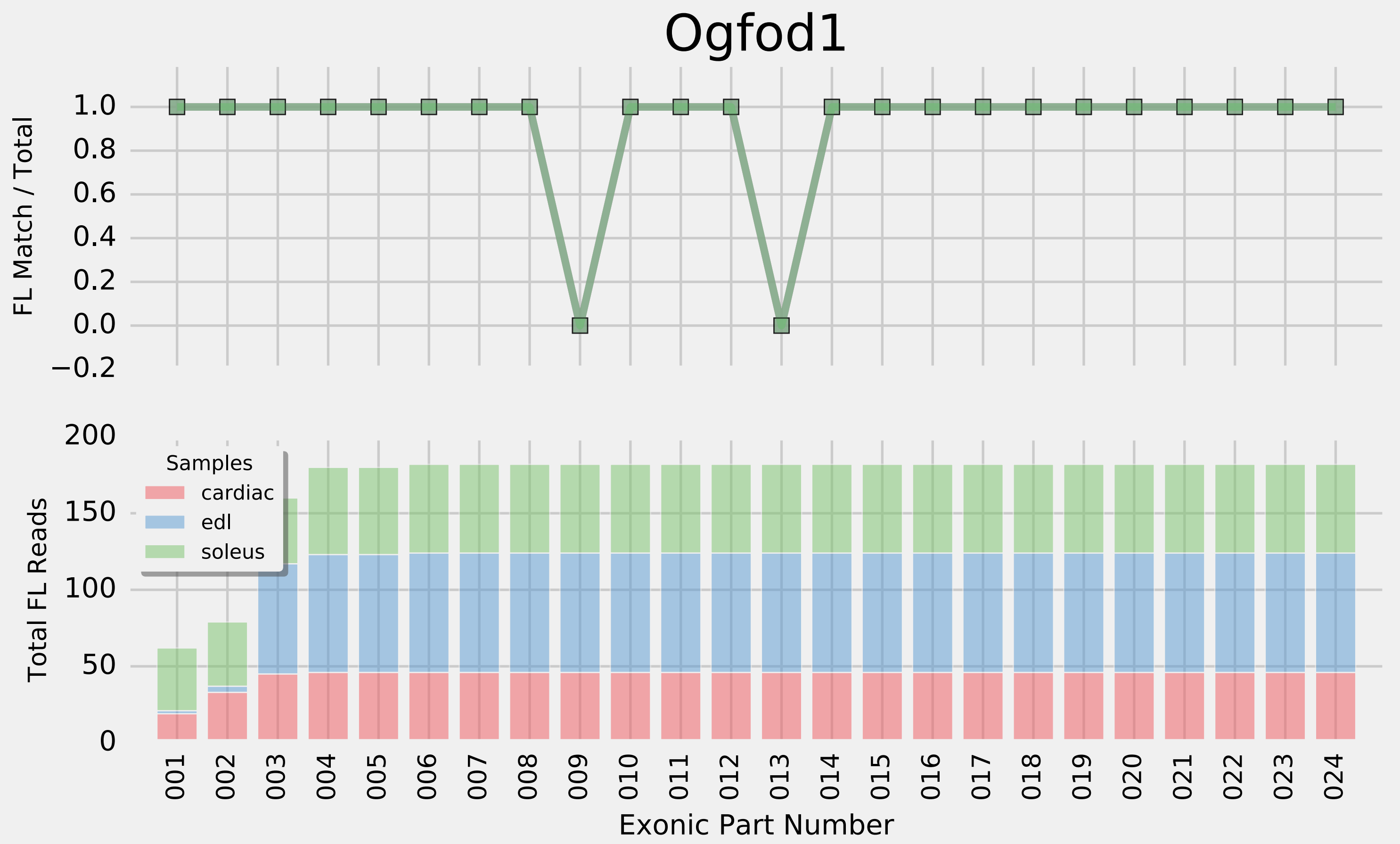

Ogfod1
FL Match / Total
1.0
0.8
0.6
0.4
0.2
0.0
−0.2
200
150
100
50
0
Total FL Reads
Samples
cardiac
edl
soleus
001
002
003
004
005
006
007
008
009
010
011
012
013
014
015
016
017
018
019
020
021
022
023
024
Exonic Part Number

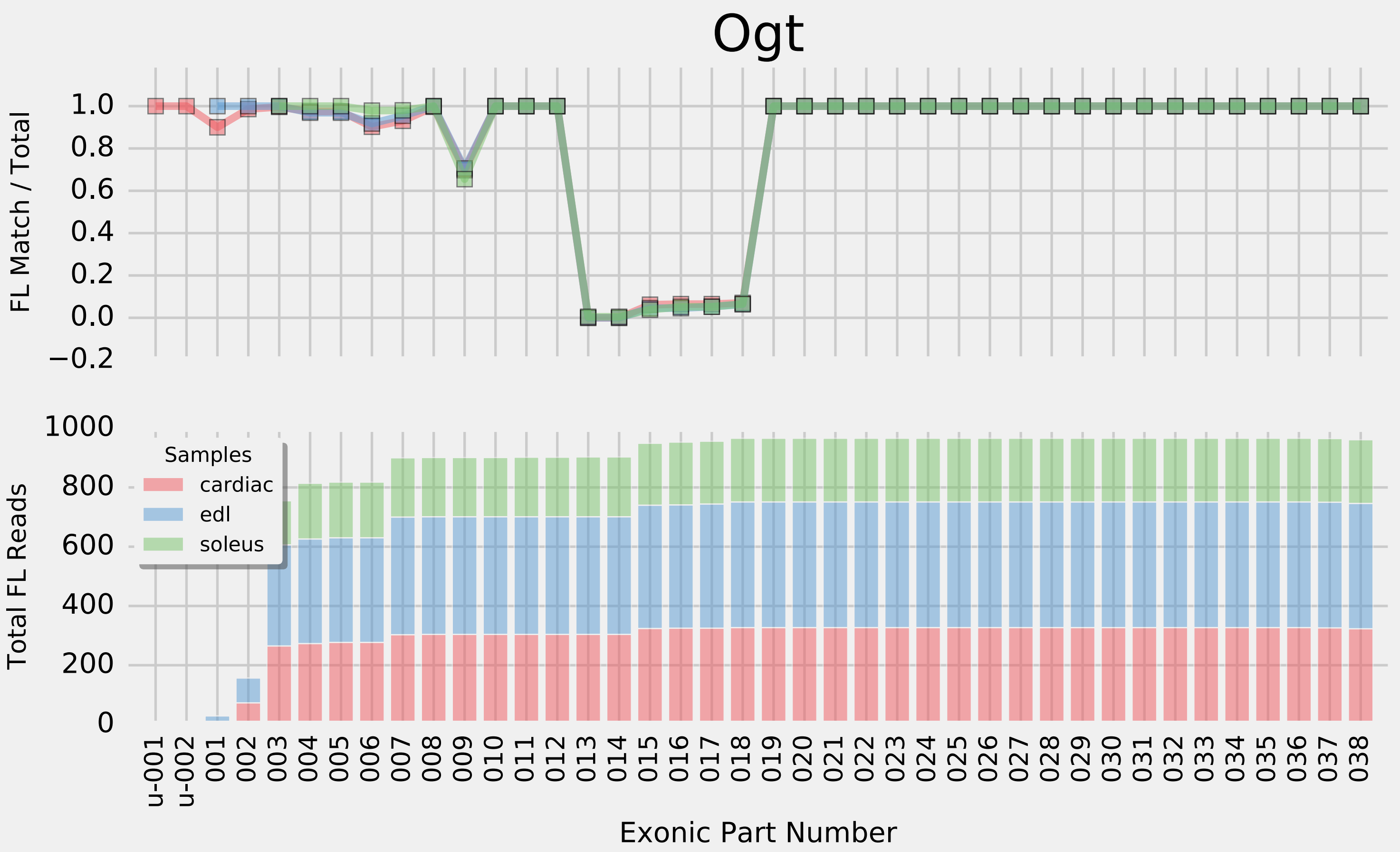

Ogt
FL Match / Total
1.0
0.8
0.6
0.4
0.2
0.0
−0.2
Total FL Reads
1000
800
600
400
200
0
Samples
cardiac
edl
soleus
u-001
u-002
001
002
003
004
005
006
007
008
009
010
011
012
013
014
015
016
017
018
019
020
021
022
023
024
025
026
027
028
029
030
031
032
033
034
035
036
037
038
Exonic Part Number

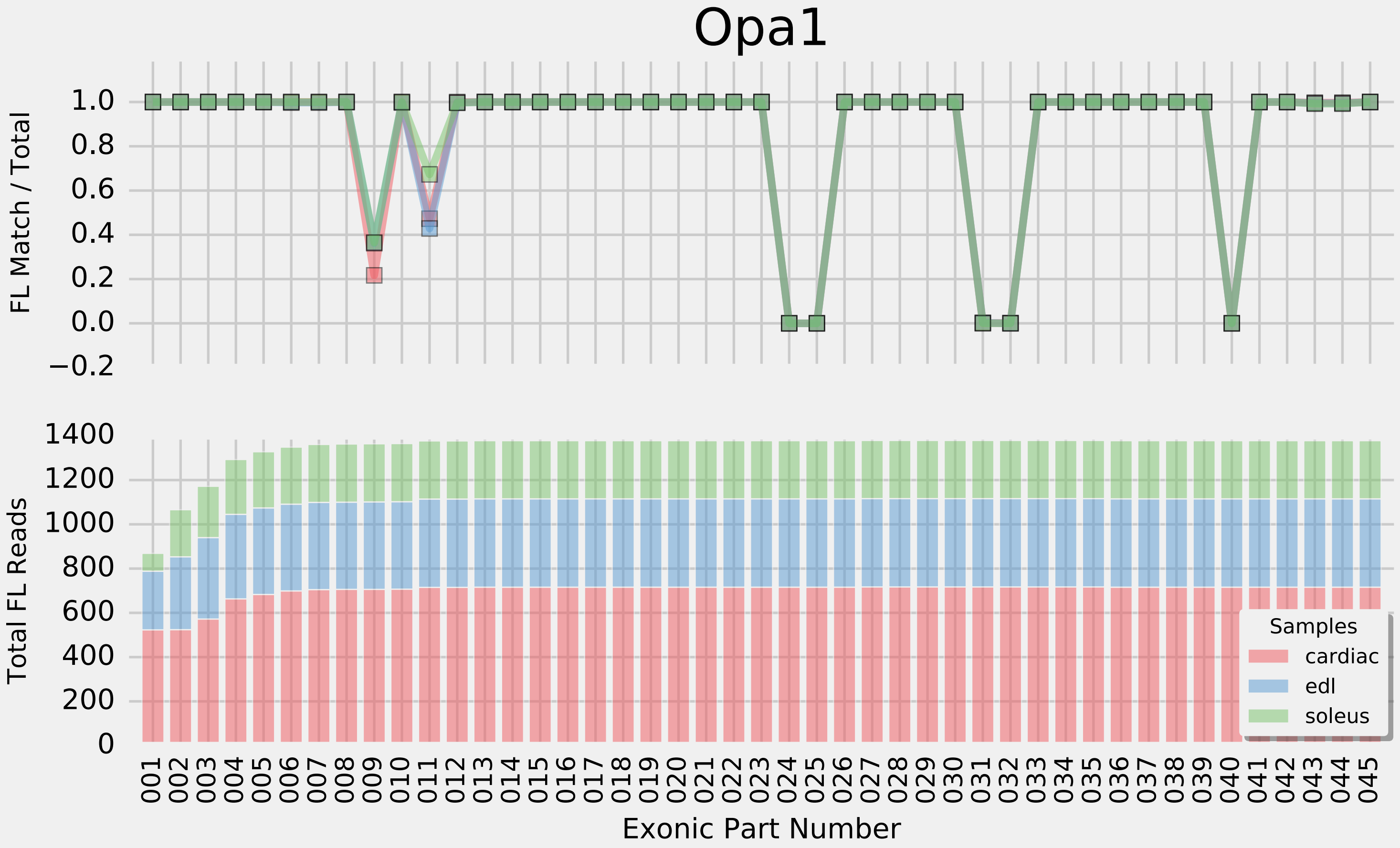
Opa1
FL Match / Total
1.0
0.8
0.6
0.4
0.2
0.0
−0.2
Total FL Reads
1400
1200
1000
800
600
400
200
0
Samples
cardiac
edl
soleus
001 002 003 004 005 006 007 008 009 010 011 012 013 014 015 016 017 018 019 020 021 022 023 024 025 026 027 028 029 030 031 032 033 034 035 036 037 038 039 040 041 042 043 044 045
Exonic Part Number

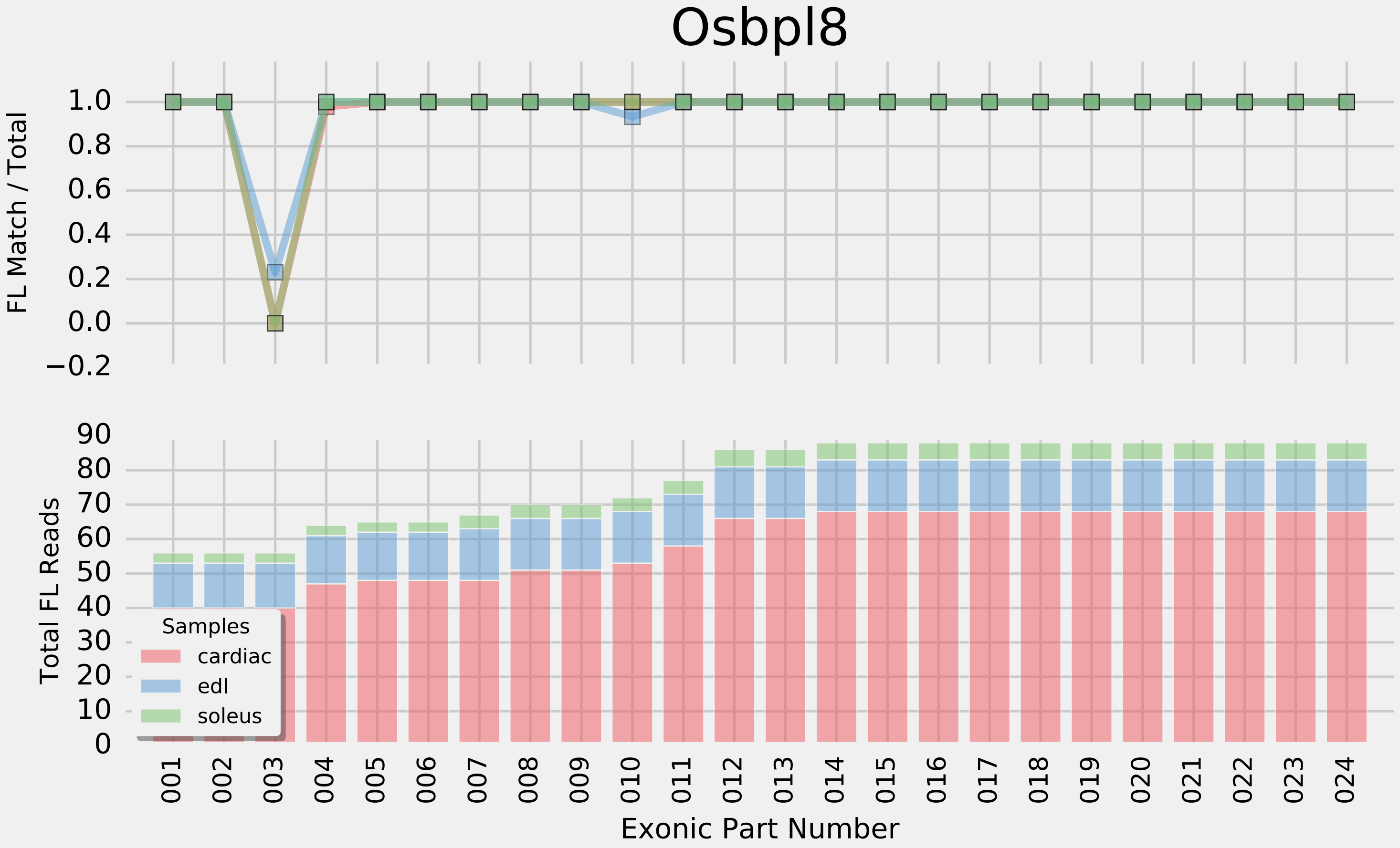

Osbpl8
FL Match / Total
1.0
0.8
0.6
0.4
0.2
0.0
−0.2
Total FL Reads
90
80
70
60
50
40
30
20
10
0
Samples
cardiac
edl
soleus
001
002
003
004
005
006
007
008
009
010
011
012
013
014
015
016
017
018
019
020
021
022
023
024
Exonic Part Number

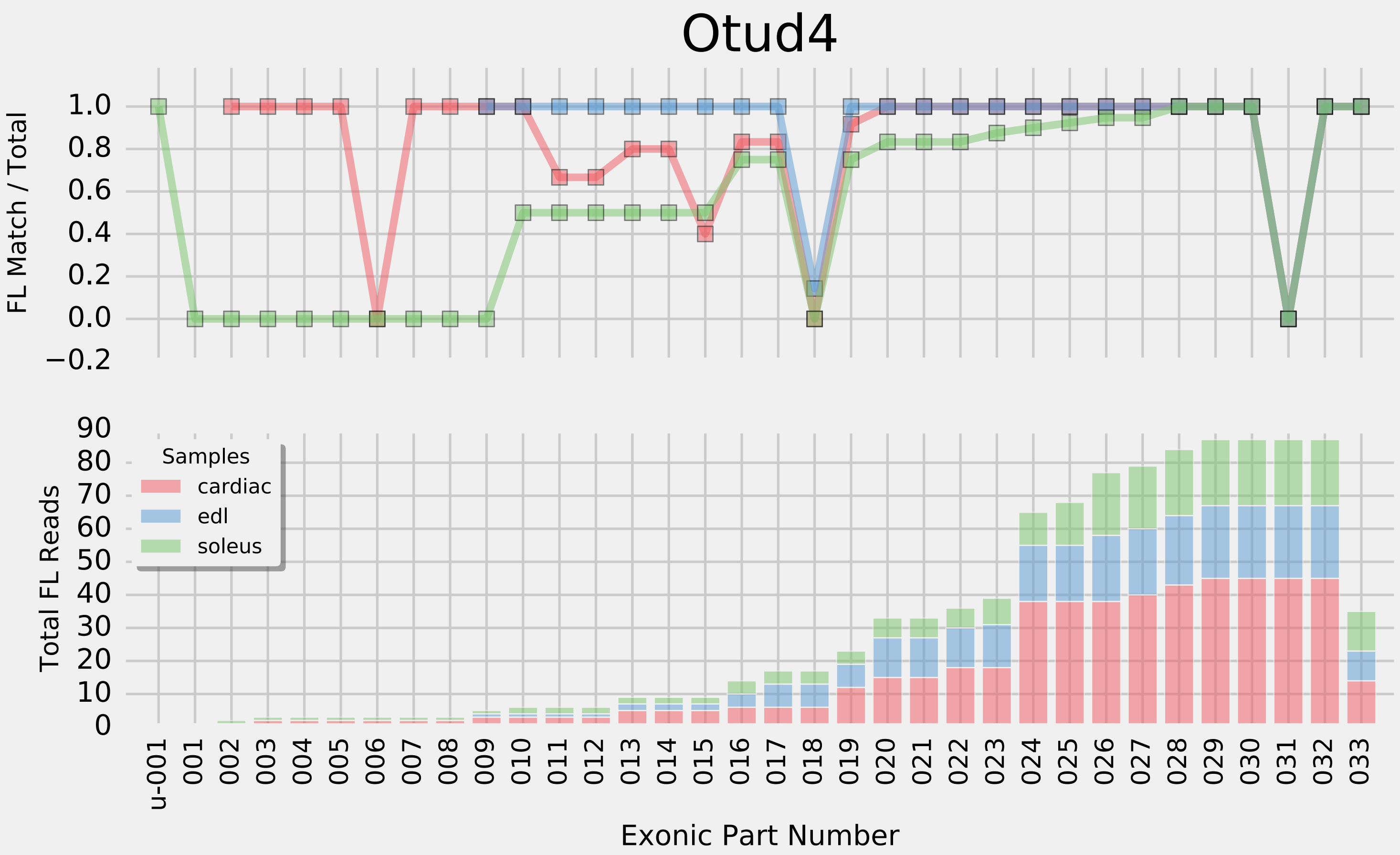

Otud4
FL Match / Total
1.0
0.8
0.6
0.4
0.2
0.0
−0.2
Total FL Reads
90
80
70
60
50
40
30
20
10
0
Samples
cardiac
edl
soleus
u-001
001
002
003
004
005
006
007
008
009
010
011
012
013
014
015
016
017
018
019
020
021
022
023
024
025
026
027
028
029
030
031
032
033
Exonic Part Number

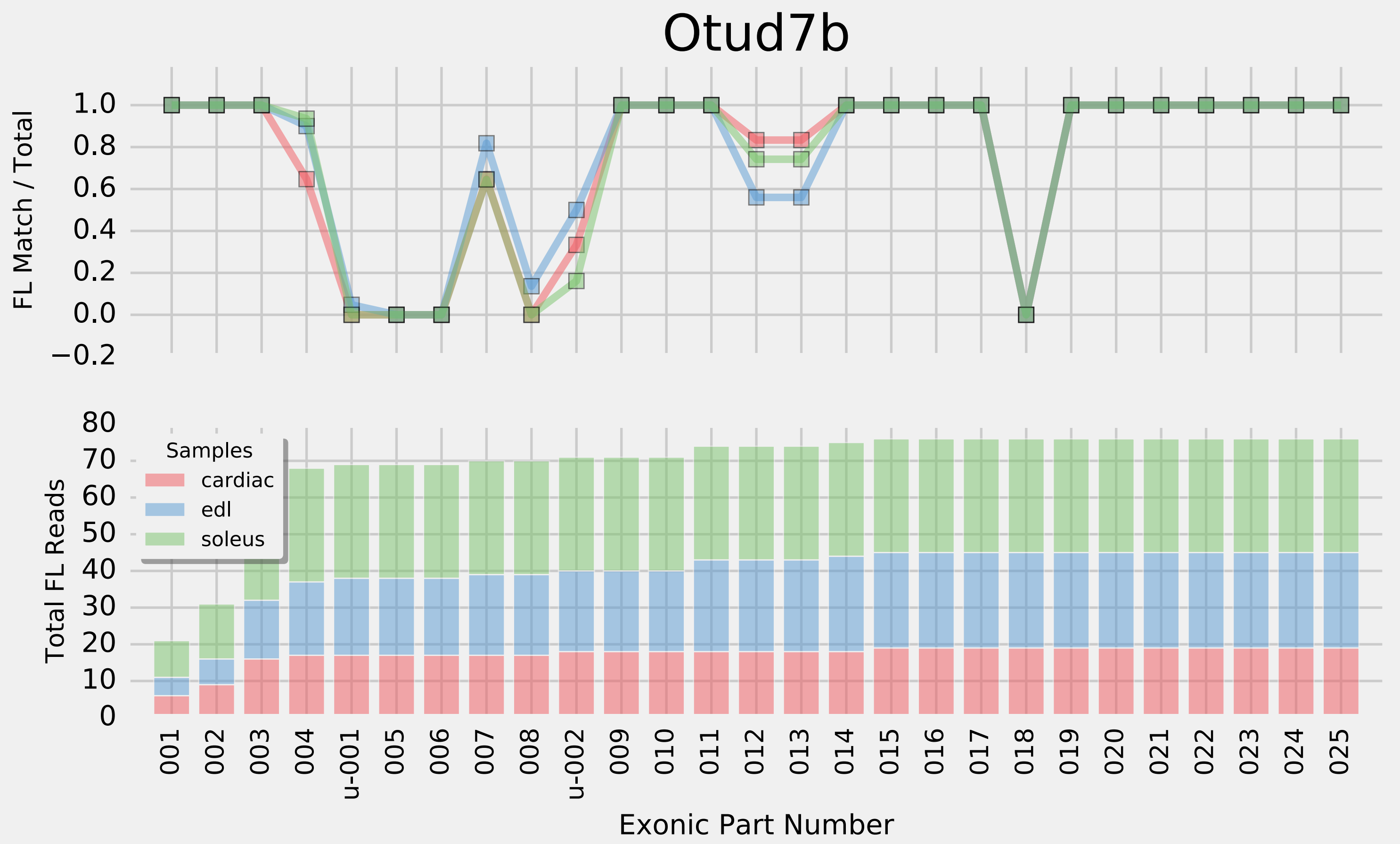
Otud7b
FL Match / Total
1.0
0.8
0.6
0.4
0.2
0.0
−0.2
Total FL Reads
80
70
60
50
40
30
20
10
0
Samples
cardiac
edl
soleus
001
002
003
004
u-001
005
006
007
008
u-002
009
010
011
012
013
014
015
016
017
018
019
020
021
022
023
024
025
Exonic Part Number

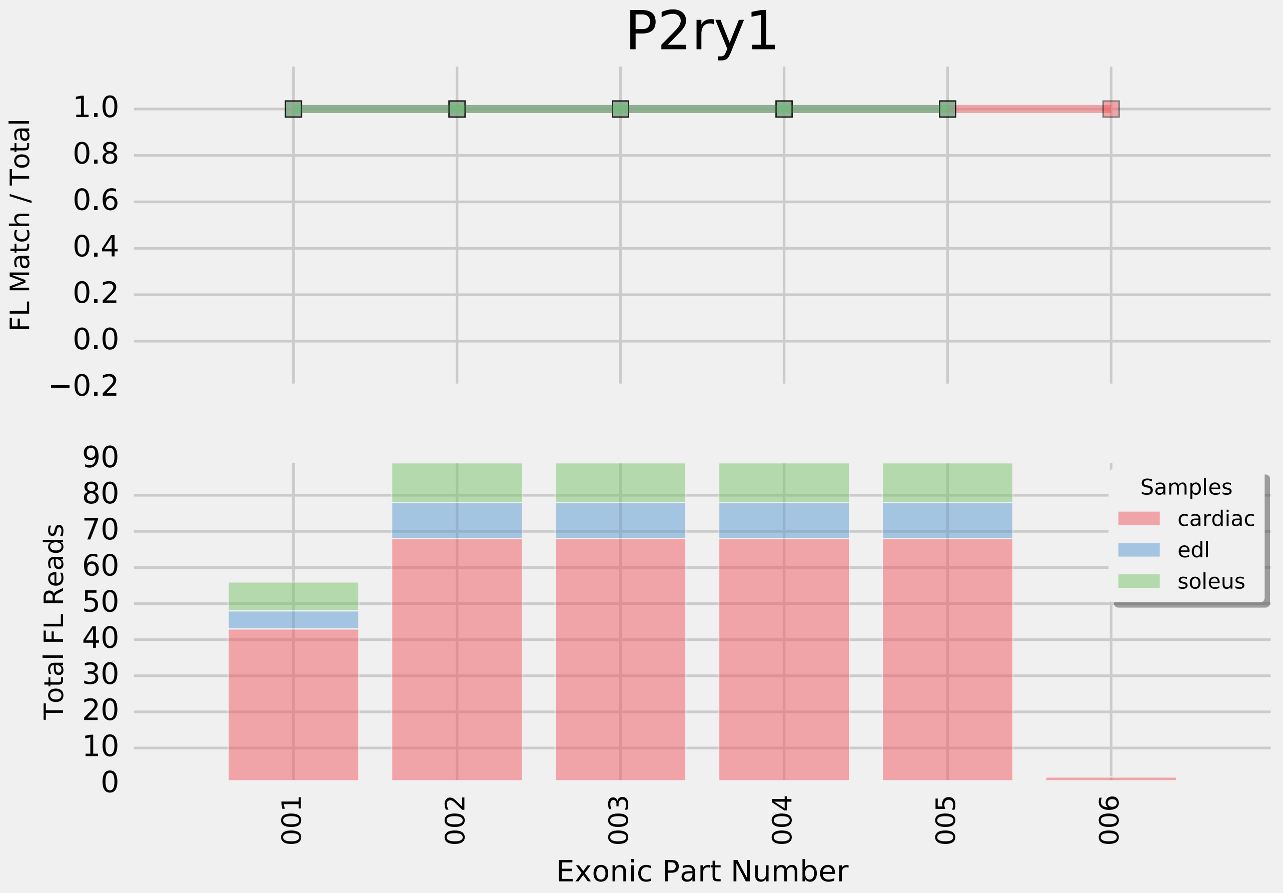

P2ry1
FL Match / Total
1.0
0.8
0.6
0.4
0.2
0.0
−0.2
Total FL Reads
90
80
70
60
50
40
30
20
10
0
Samples
cardiac
edl
soleus
001
002
003
004
005
006
Exonic Part Number

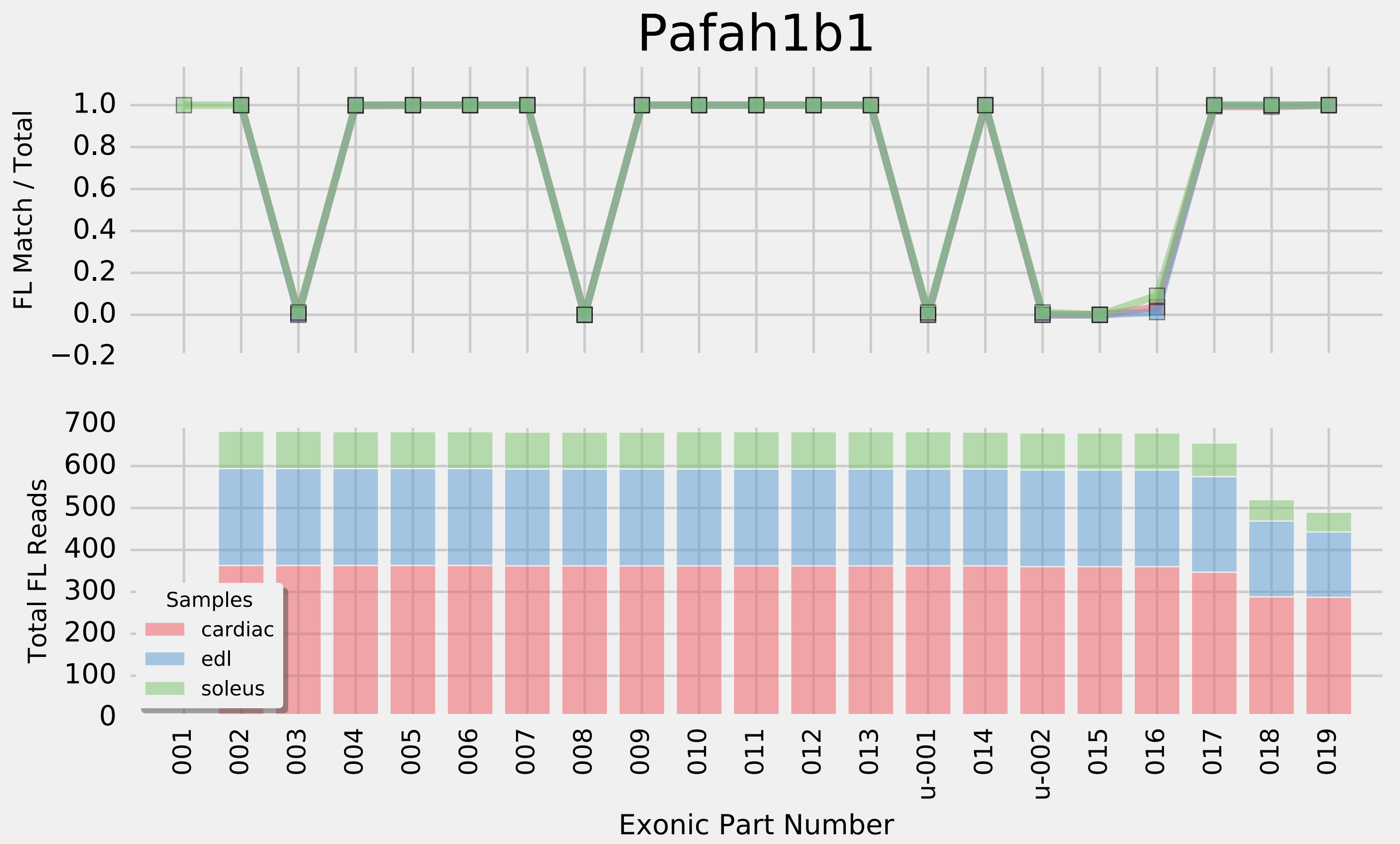

Pafah1b1
FL Match / Total
1.0
0.8
0.6
0.4
0.2
0.0
−0.2
Total FL Reads
700
600
500
400
300
200
100
0
Samples
cardiac
edl
soleus
001
002
003
004
005
006
007
008
009
010
011
012
013
u-001
014
u-002
015
016
017
018
019
Exonic Part Number

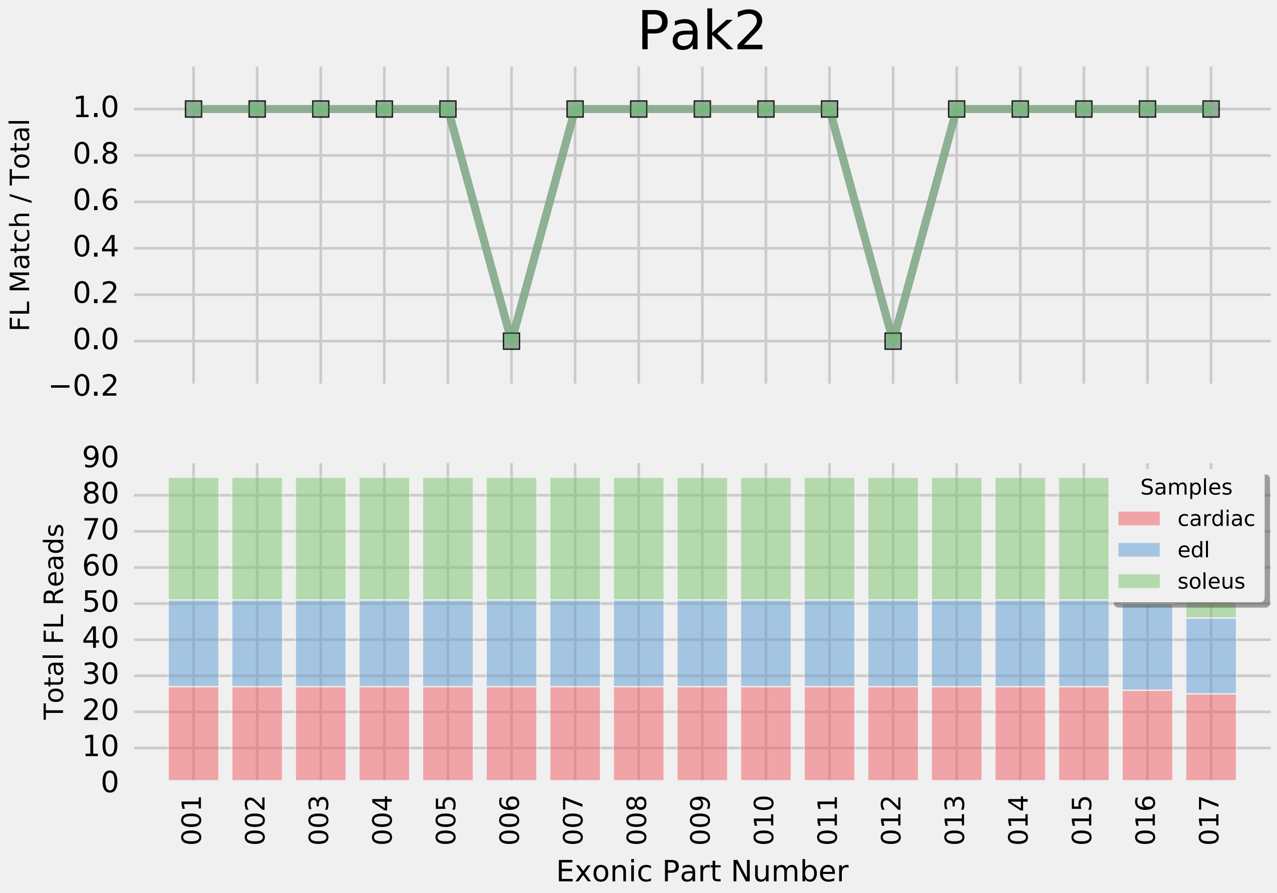

Pak2
FL Match / Total
1.0
0.8
0.6
0.4
0.2
0.0
−0.2
Total FL Reads
90
80
70
60
50
40
30
20
10
0
Samples
cardiac
edl
soleus
001
002
003
004
005
006
007
008
009
010
011
012
013
014
015
016
017
Exonic Part Number

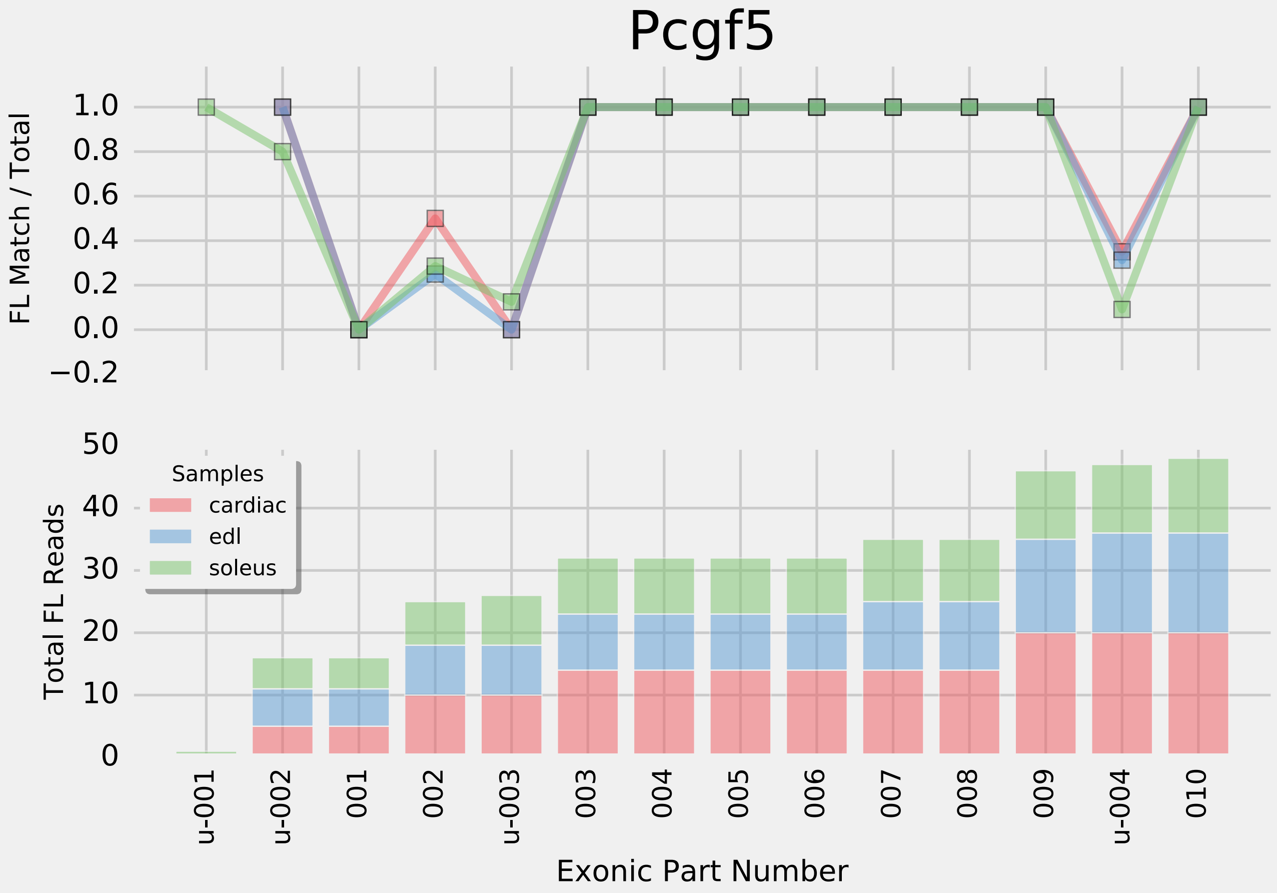
Pcgf5
FL Match / Total
1.0
0.8
0.6
0.4
0.2
0.0
−0.2
Total FL Reads
50
40
30
20
10
0
Samples
cardiac
edl
soleus
u-001
u-002
001
002
u-003
003
004
005
006
007
008
009
u-004
010
Exonic Part Number

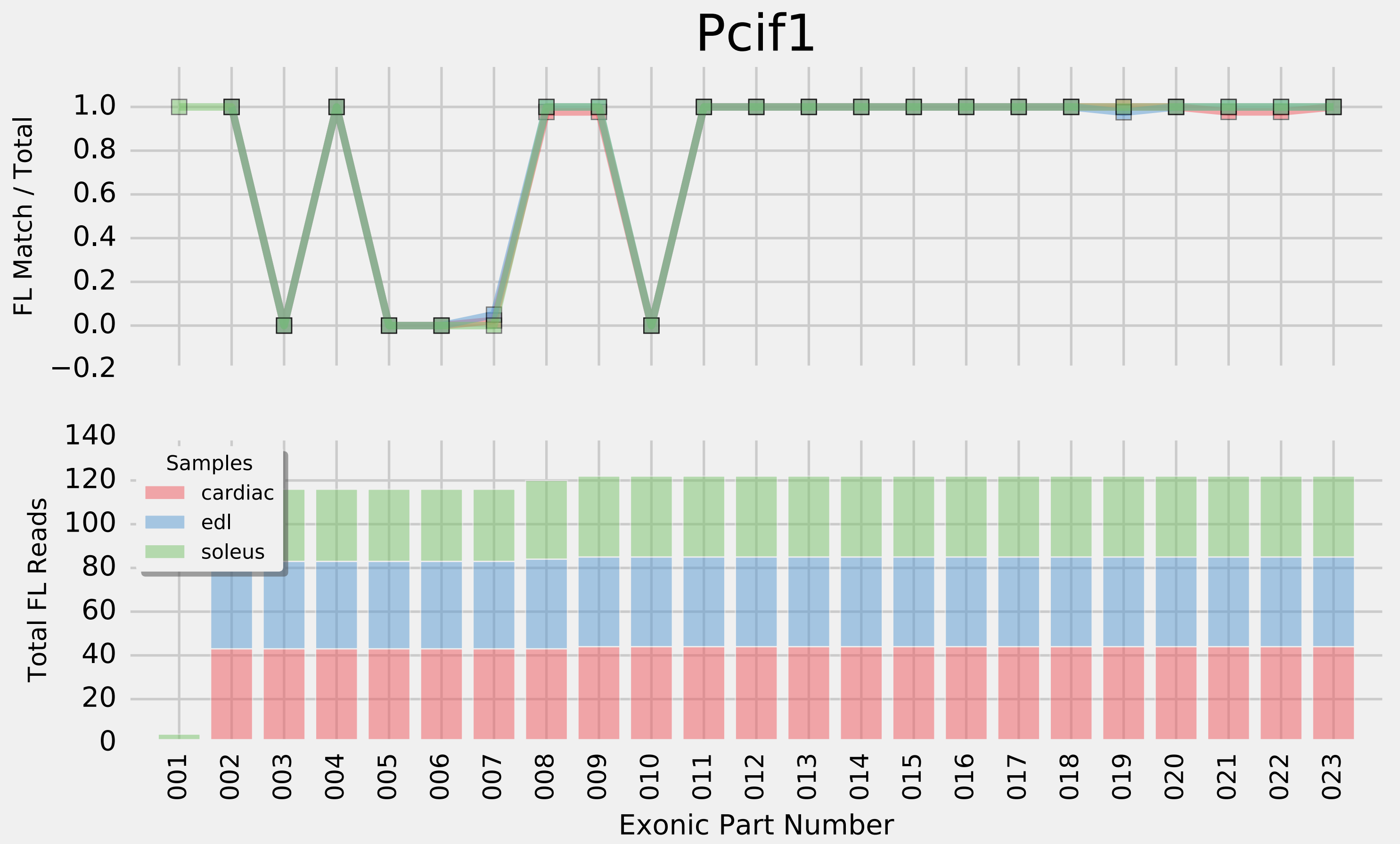
Pcif1
FL Match / Total
1.0
0.8
0.6
0.4
0.2
0.0
−0.2
Total FL Reads
140
120
100
80
60
40
20
0
Samples
cardiac
edl
soleus
001
002
003
004
005
006
007
008
009
010
011
012
013
014
015
016
017
018
019
020
021
022
023
Exonic Part Number

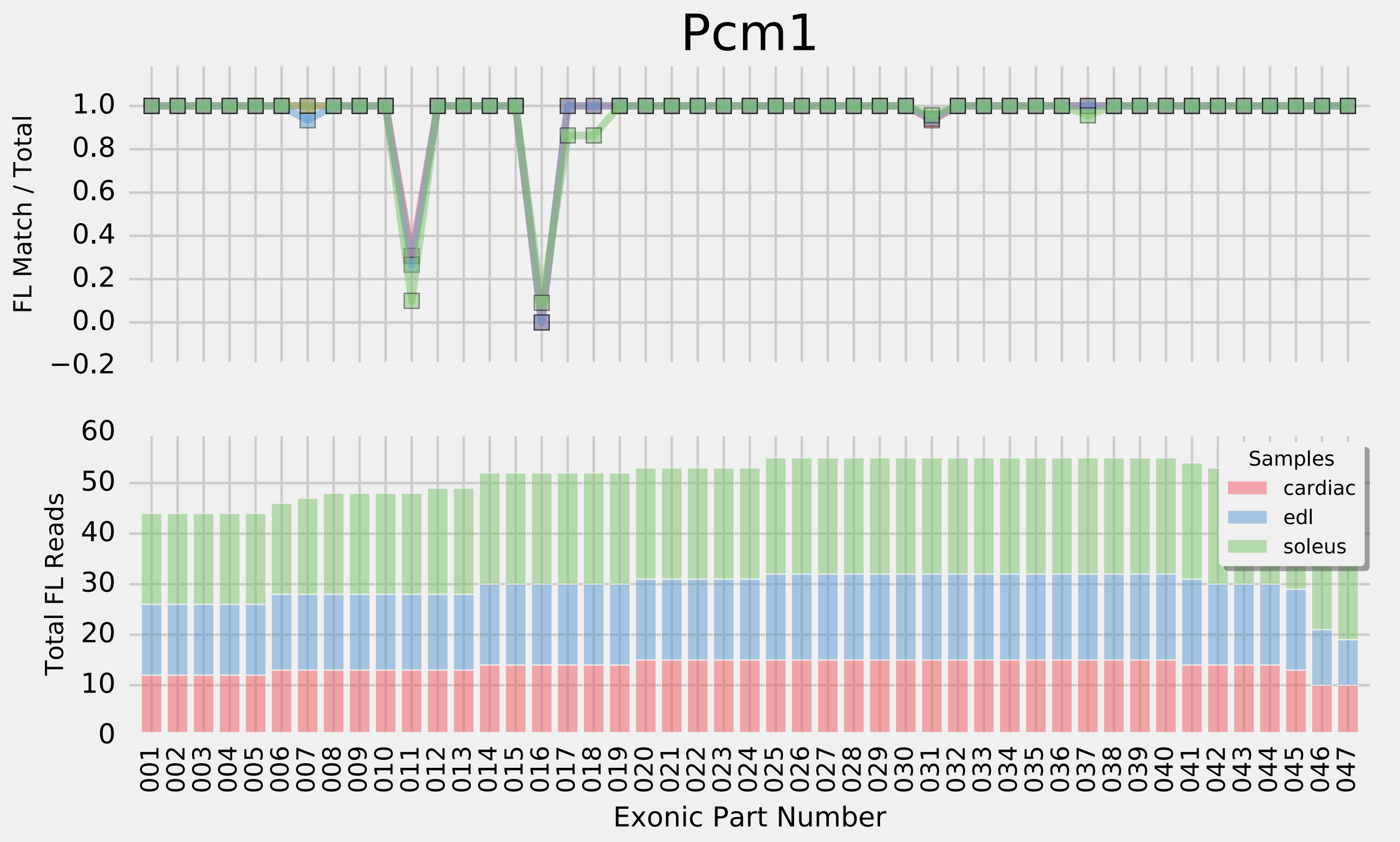

Pcm1
FL Match / Total
1.0
0.8
0.6
0.4
0.2
0.0
−0.2
Total FL Reads
60
50
40
30
20
10
0
Samples
cardiac
edl
soleus
001 002 003 004 005 006 007 008 009 010 011 012 013 014 015 016 017 018 019 020 021 022 023 024 025 026 027 028 029 030 031 032 033 034 035 036 037 038 039 040 041 042 043 044 045 046 047
Exonic Part Number

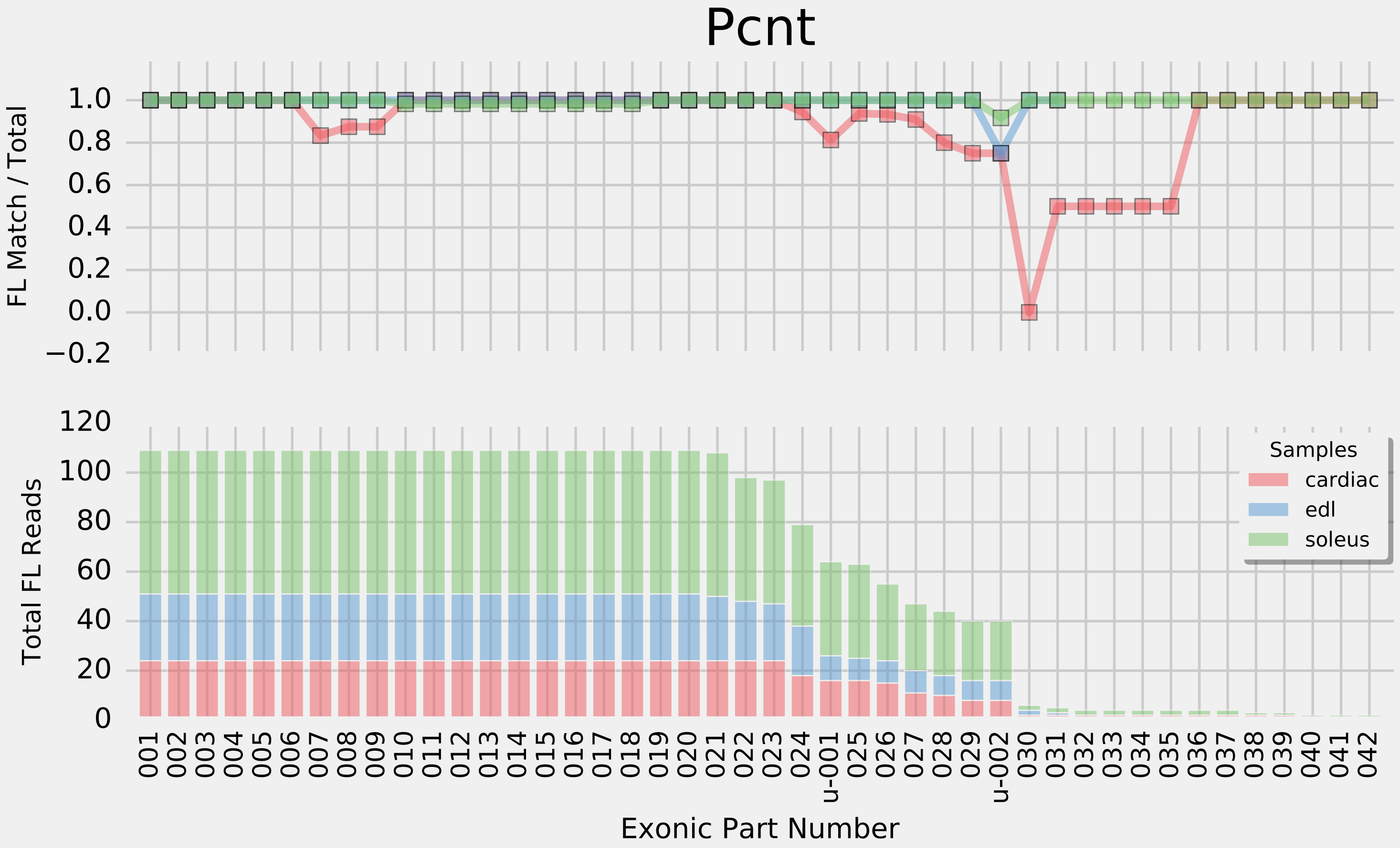

Pcnt
FL Match / Total
1.0
0.8
0.6
0.4
0.2
0.0
−0.2
Total FL Reads
120
100
80
60
40
20
0
Samples
cardiac
edl
soleus
001
002
003
004
005
006
007
008
009
010
011
012
013
014
015
016
017
018
019
020
021
022
023
024
u-001
025
026
027
028
029
u-002
030
031
032
033
034
035
036
037
038
039
040
041
042
Exonic Part Number

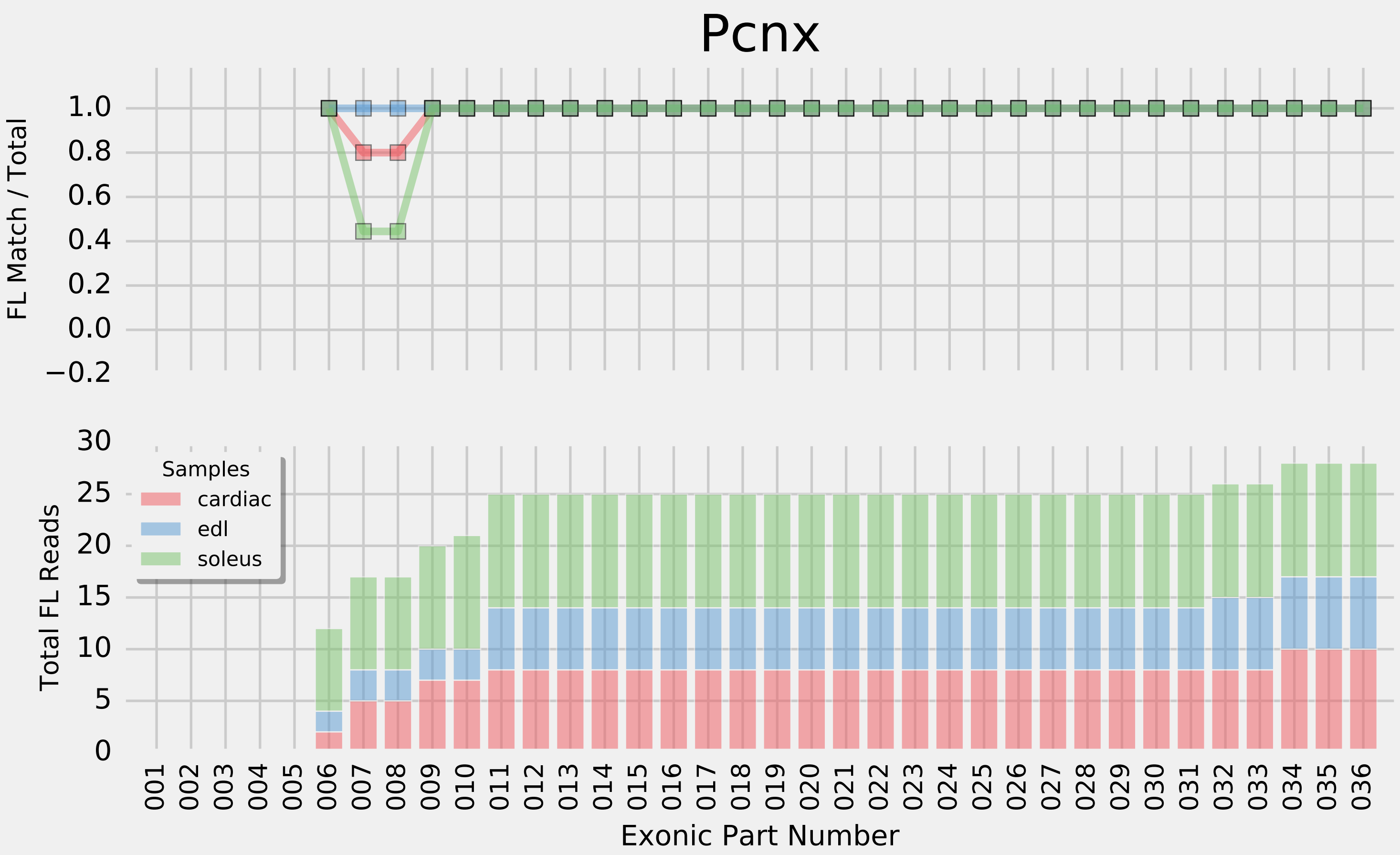
Pcnx
FL Match / Total
1.0
0.8
0.6
0.4
0.2
0.0
−0.2
Total FL Reads
30
25
20
15
10
5
0
Samples
cardiac
edl
soleus
001 002 003 004 005 006 007 008 009 010 011 012 013 014 015 016 017 018 019 020 021 022 023 024 025 026 027 028 029 030 031 032 033 034 035 036
Exonic Part Number

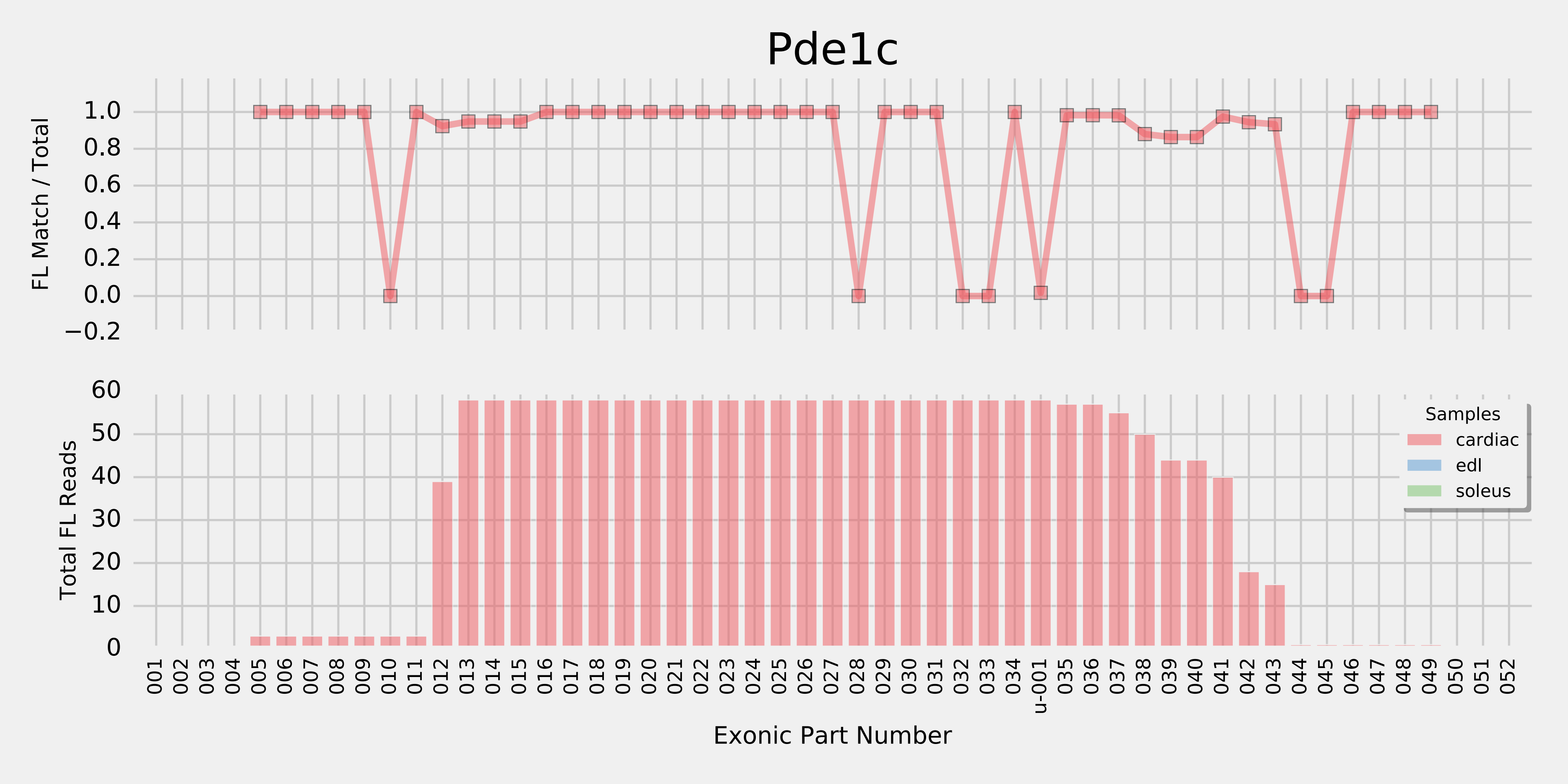

Pde1c
FL Match / Total
Total FL Reads
Exonic Part Number
Samples
cardiac
edl
soleus

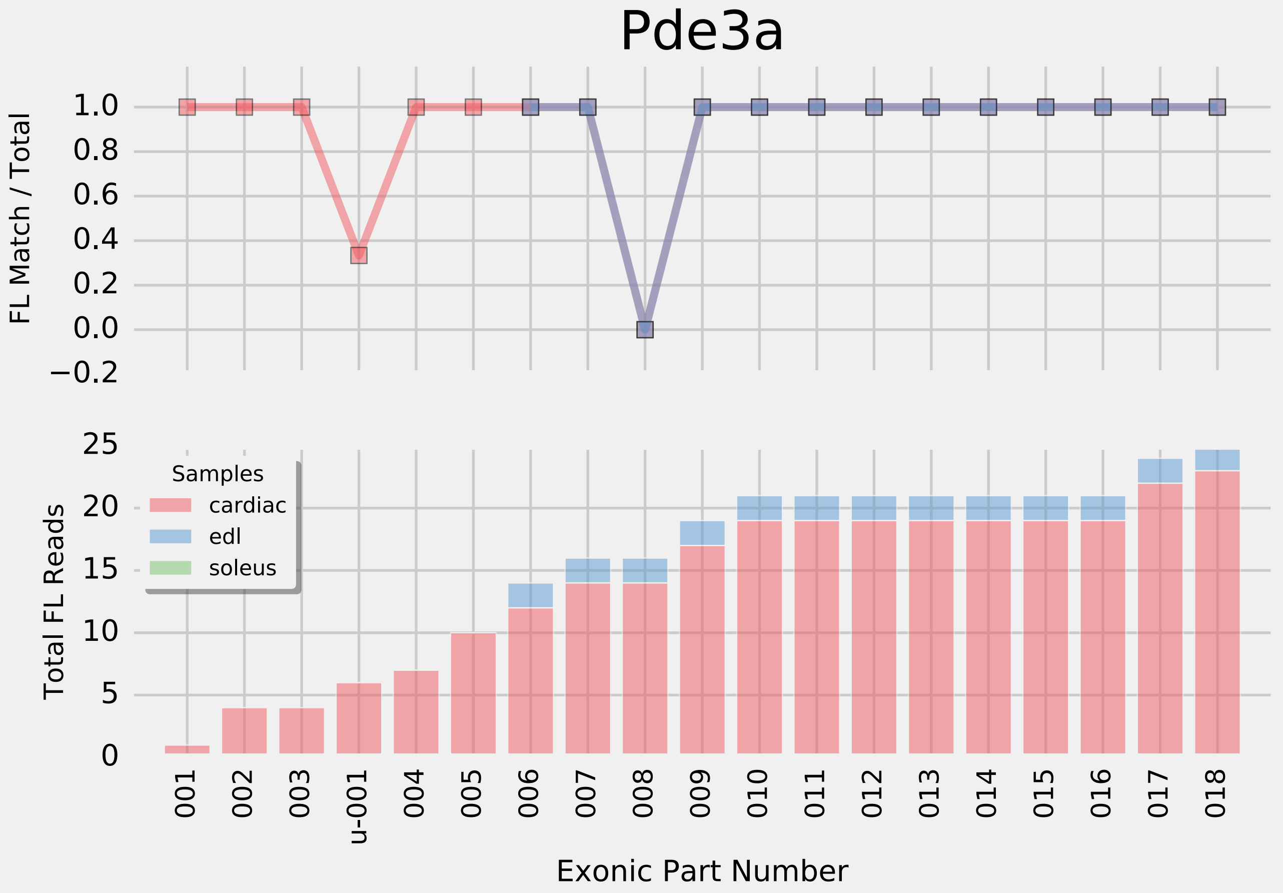

Pde3a
FL Match / Total
1.0
0.8
0.6
0.4
0.2
0.0
−0.2
Total FL Reads
25
20
15
10
5
0
Samples
cardiac
edl
soleus
001
002
003
u-001
004
005
006
007
008
009
010
011
012
013
014
015
016
017
018
Exonic Part Number

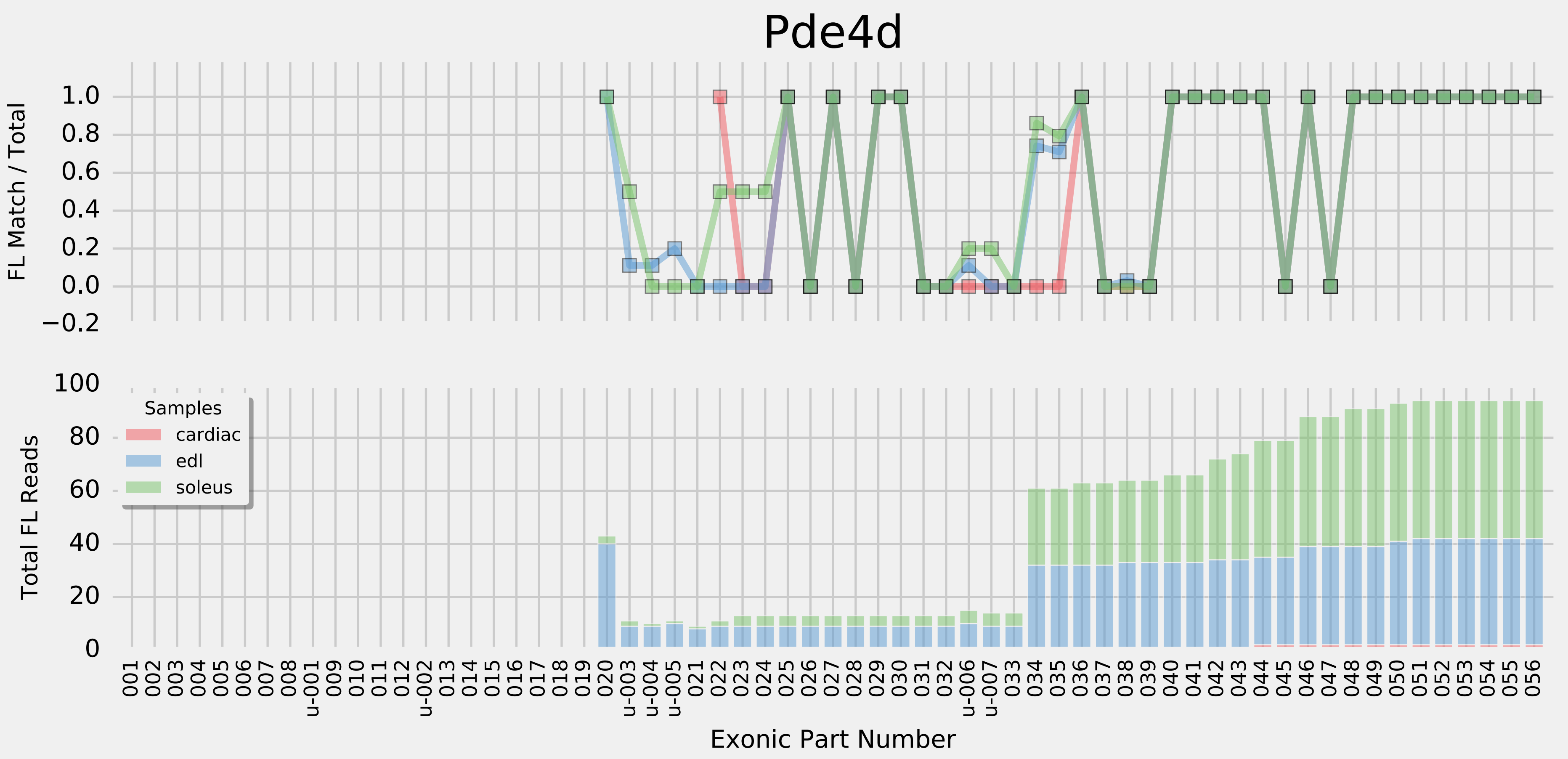

Pde4d
FL Match / Total
1.0
0.8
0.6
0.4
0.2
0.0
−0.2
100
80
60
40
20
0
Total FL Reads
Samples
cardiac
edl
soleus
001
002
003
004
005
006
007
008
u-001
009
010
011
012
u-002
013
014
015
016
017
018
019
020
u-003
u-004
u-005
021
022
023
024
025
026
027
028
029
030
031
032
u-006
u-007
033
034
035
036
037
038
039
040
041
042
043
044
045
046
047
048
049
050
051
052
053
054
055
056
Exonic Part Number

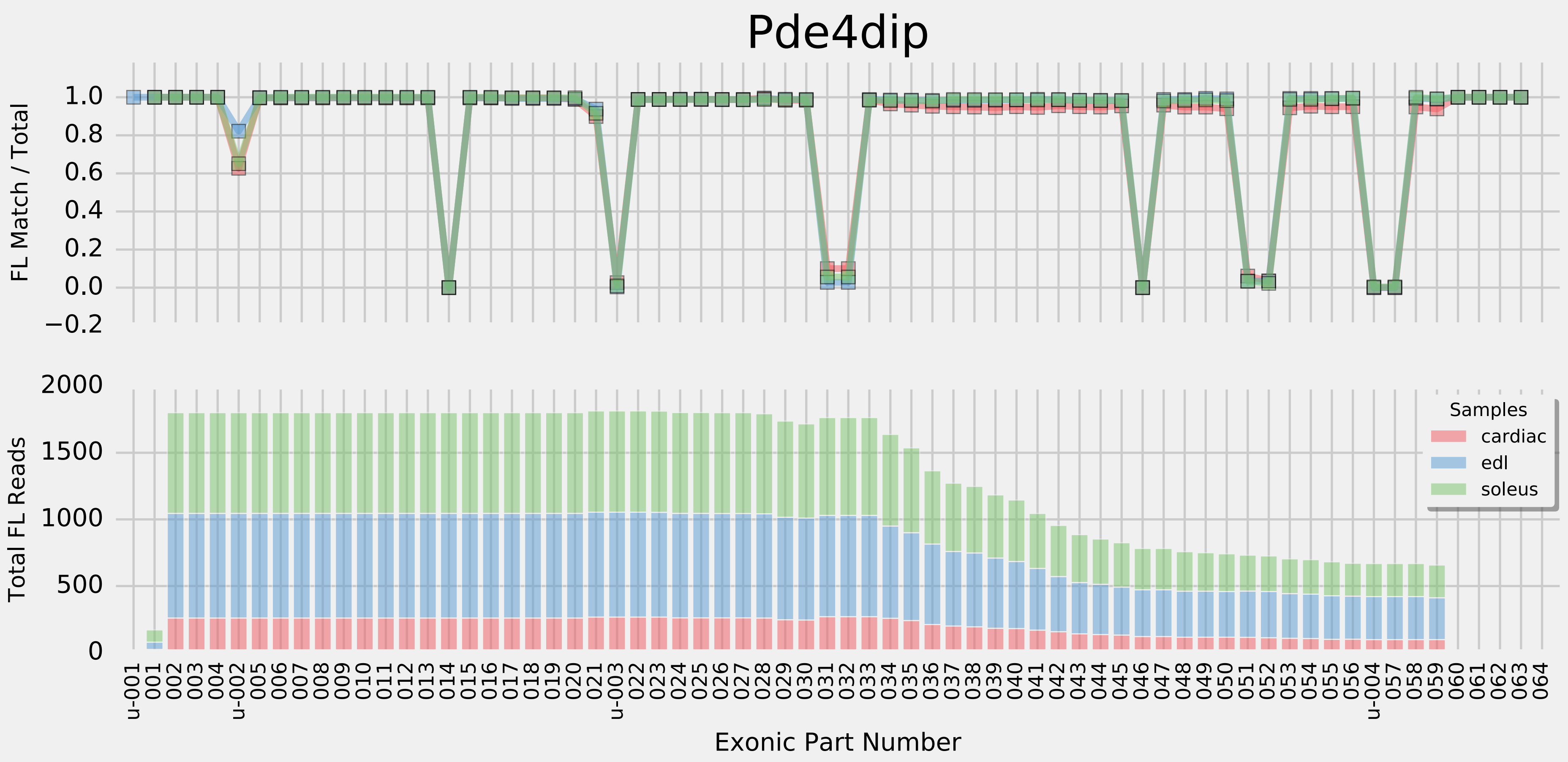

Pde4dip
FL Match / Total
1.0
0.8
0.6
0.4
0.2
0.0
−0.2
Total FL Reads
2000
1500
1000
500
0
Samples
cardiac
edl
soleus
u-001
001
002
003
004
u-002
005
006
007
008
009
010
011
012
013
014
015
016
017
018
019
020
021
u-003
022
023
024
025
026
027
028
029
030
031
032
033
034
035
036
037
038
039
040
041
042
043
044
045
046
047
048
049
050
051
052
053
054
055
056
u-004
057
058
059
060
061
062
063
064
Exonic Part Number

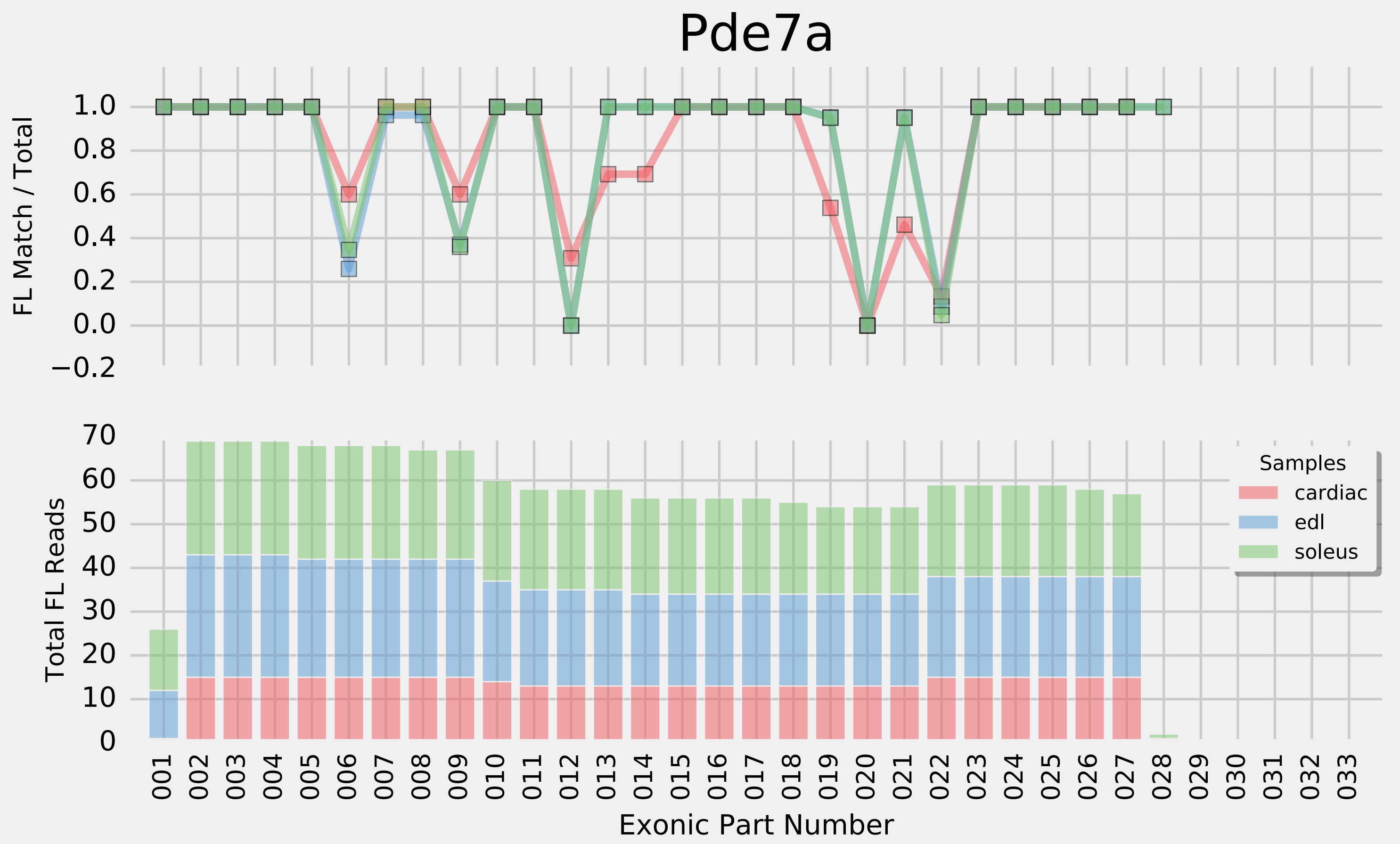

Pde7a
FL Match / Total
1.0
0.8
0.6
0.4
0.2
0.0
−0.2
Total FL Reads
70
60
50
40
30
20
10
0
Samples
cardiac
edl
soleus
001 002 003 004 005 006 007 008 009 010 011 012 013 014 015 016 017 018 019 020 021 022 023 024 025 026 027 028 029 030 031 032 033
Exonic Part Number

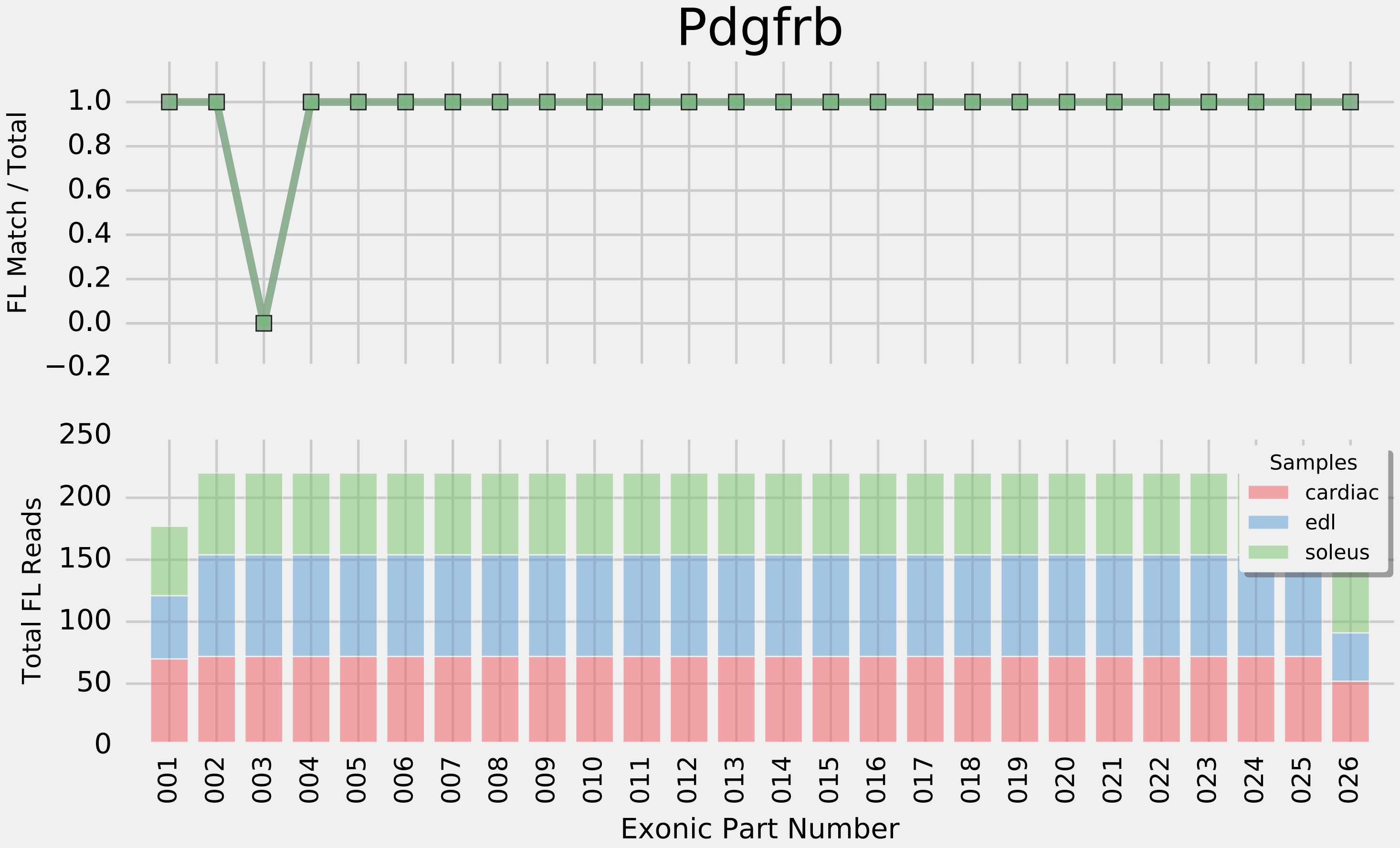

Pdgfrb
FL Match / Total
1.0
0.8
0.6
0.4
0.2
0.0
−0.2
Total FL Reads
250
200
150
100
50
0
Samples
cardiac
edl
soleus
001
002
003
004
005
006
007
008
009
010
011
012
013
014
015
016
017
018
019
020
021
022
023
024
025
026
Exonic Part Number

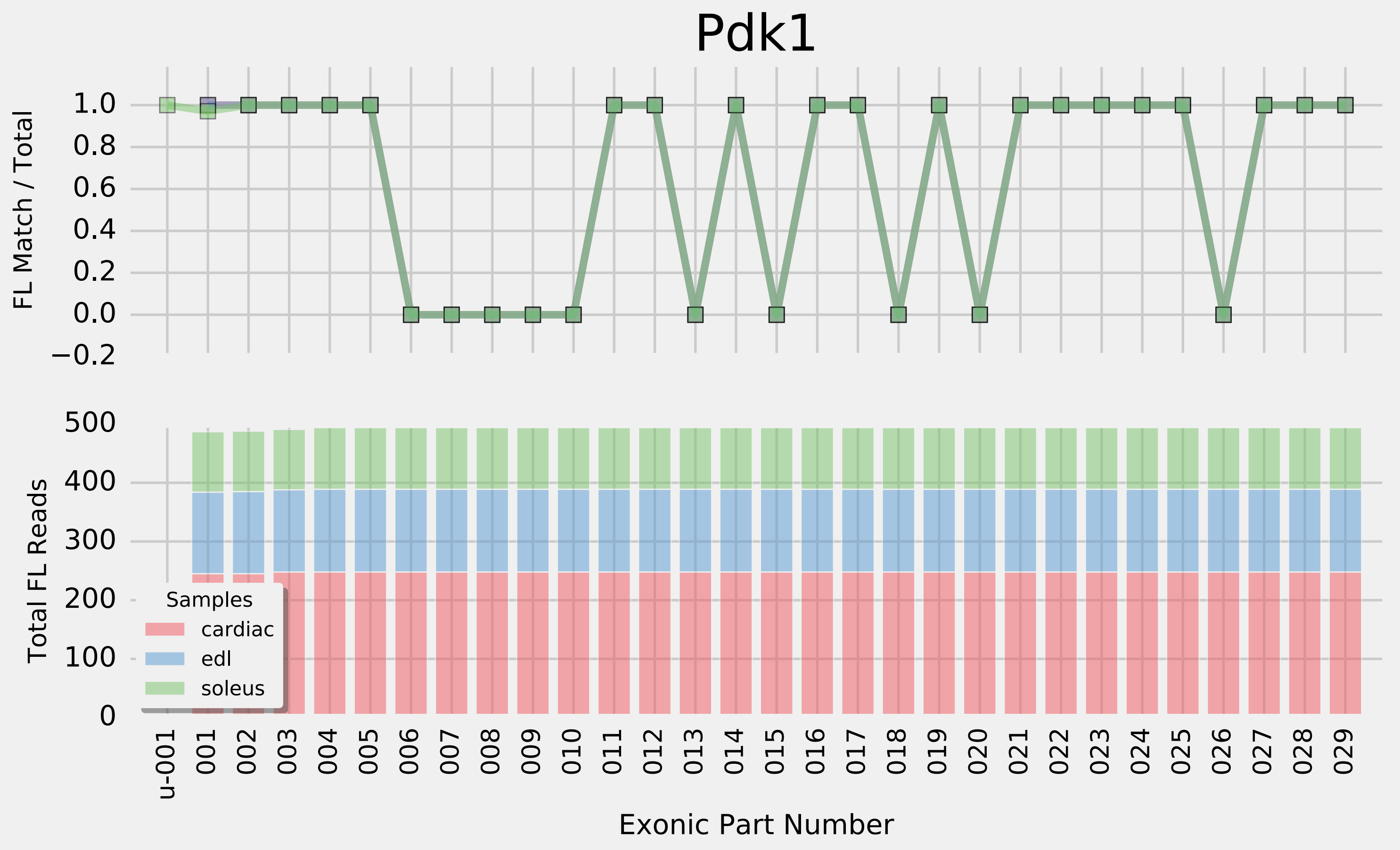
Pdk1
FL Match / Total
1.0
0.8
0.6
0.4
0.2
0.0
−0.2
Total FL Reads
500
400
300
200
100
0
Samples
cardiac
edl
soleus
u-001
001
002
003
004
005
006
007
008
009
010
011
012
013
014
015
016
017
018
019
020
021
022
023
024
025
026
027
028
029
Exonic Part Number

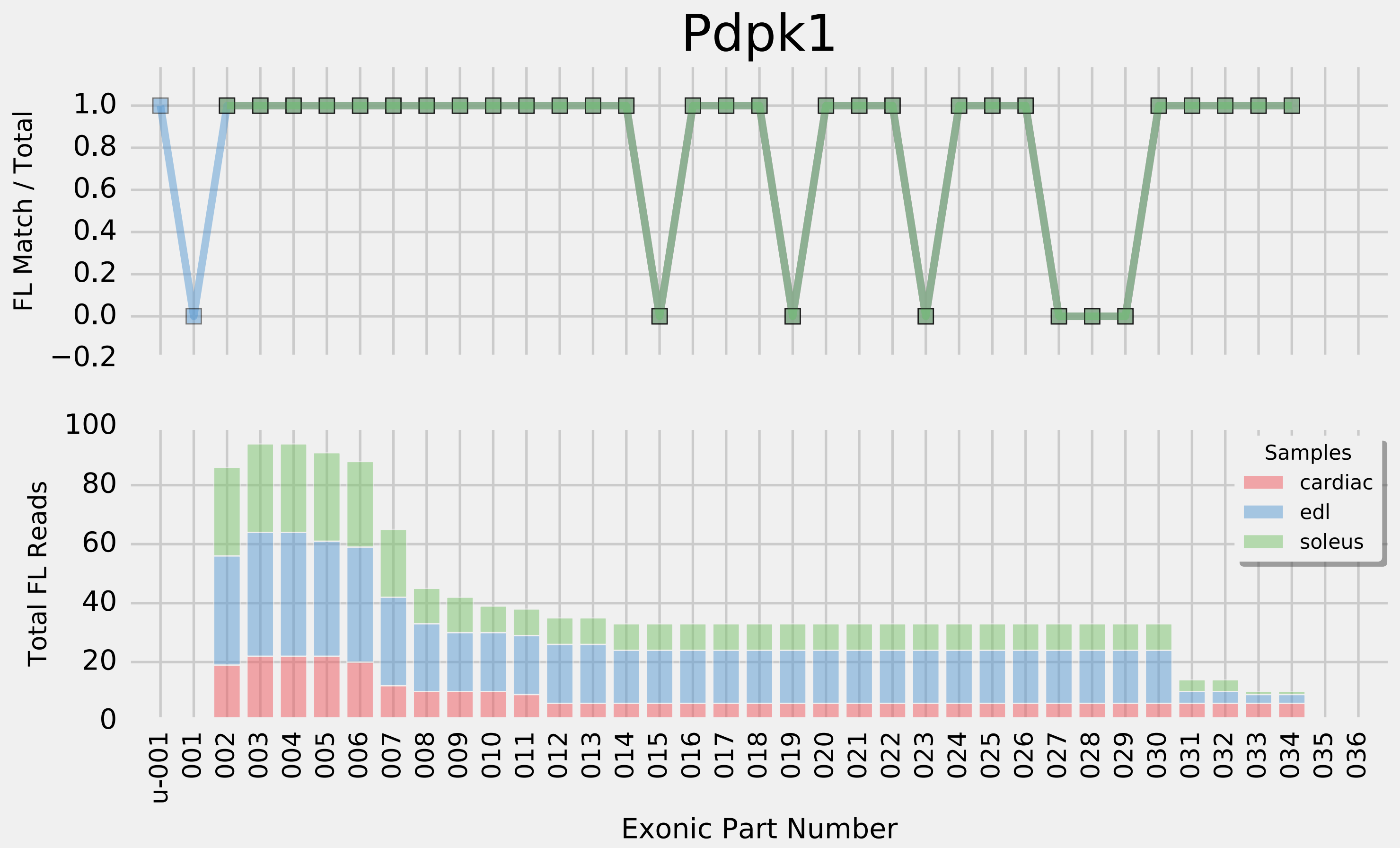

Pdpk1
FL Match / Total
1.0
0.8
0.6
0.4
0.2
0.0
−0.2
100
80
60
40
20
0
Total FL Reads
Samples
cardiac
edl
soleus
u-001
001
002
003
004
005
006
007
008
009
010
011
012
013
014
015
016
017
018
019
020
021
022
023
024
025
026
027
028
029
030
031
032
033
034
035
036
Exonic Part Number

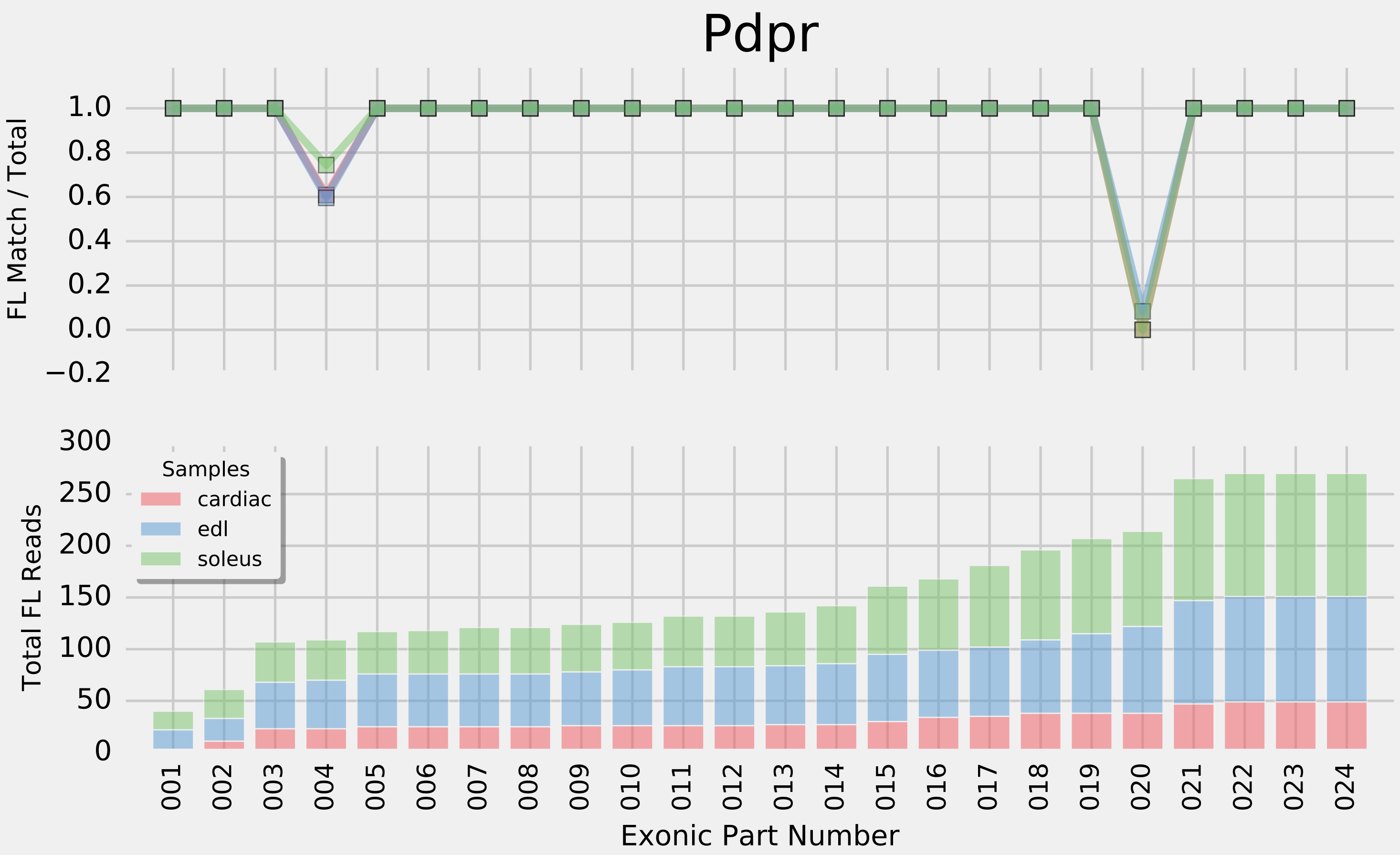

Pdpr
FL Match / Total
1.0
0.8
0.6
0.4
0.2
0.0
−0.2
Total FL Reads
300
250
200
150
100
50
0
Samples
cardiac
edl
soleus
001
002
003
004
005
006
007
008
009
010
011
012
013
014
015
016
017
018
019
020
021
022
023
024
Exonic Part Number

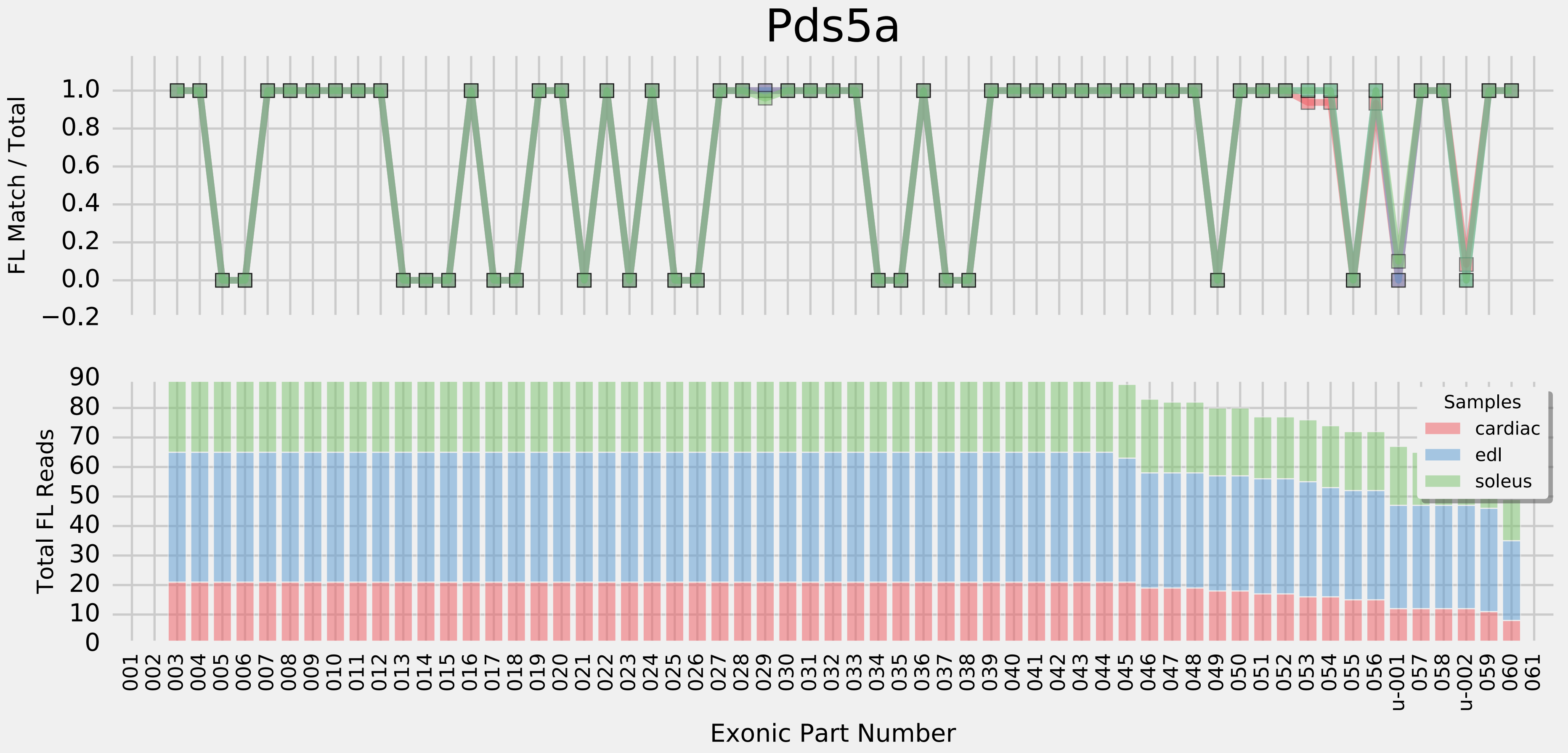

Pds5a
FL Match / Total
Total FL Reads
Exonic Part Number
Samples
cardiac
edl
soleus
001 002 003 004 005 006 007 008 009 010 011 012 013 014 015 016 017 018 019 020 021 022 023 024 025 026 027 028 029 030 031 032 033 034 035 036 037 038 039 040 041 042 043 044 045 046 047 048 049 050 051 052 053 054 055 056 u-001 057 058 u-002 059 060 061

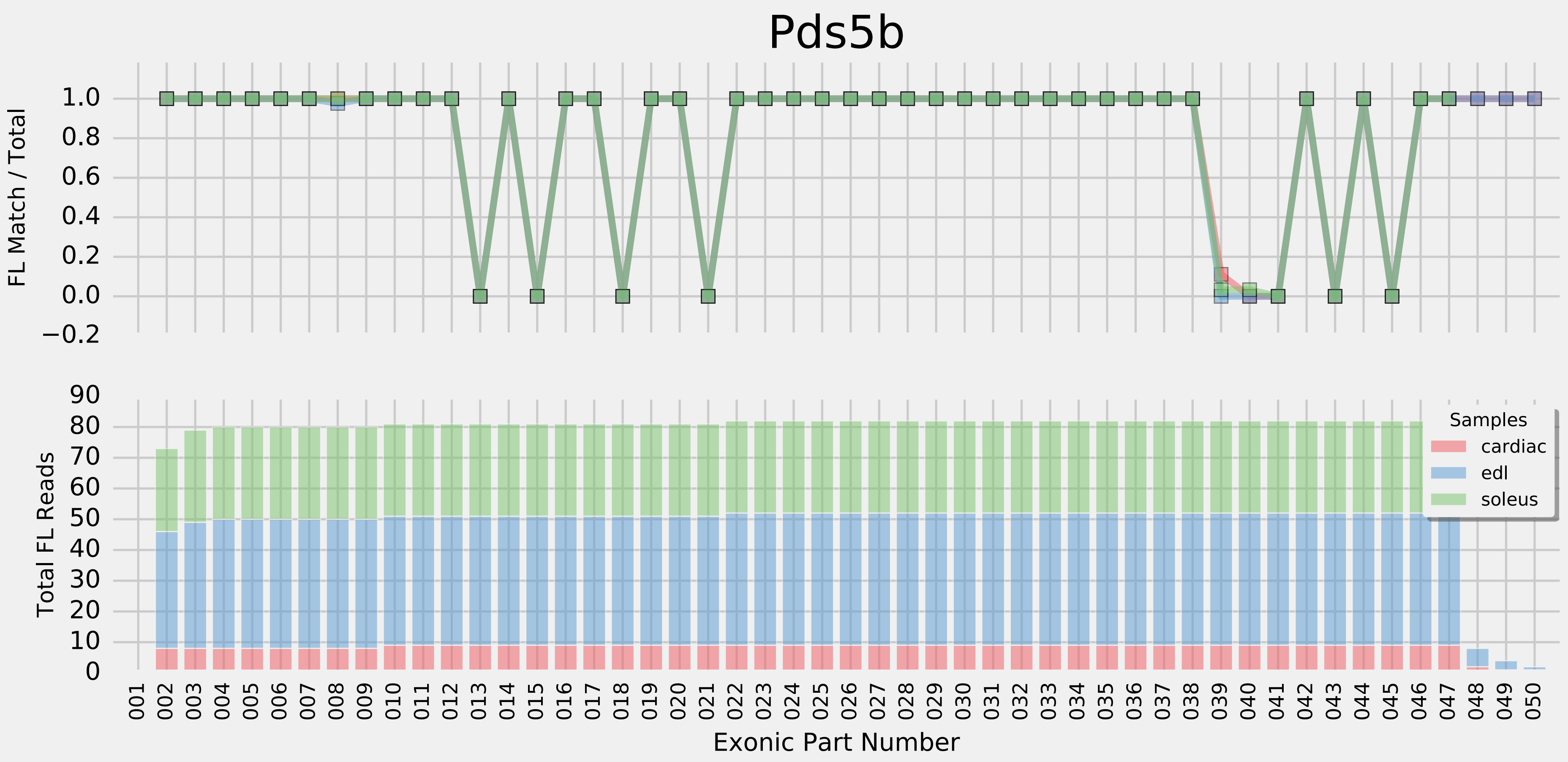

Pds5b
FL Match / Total
1.0
0.8
0.6
0.4
0.2
0.0
−0.2
Total FL Reads
90
80
70
60
50
40
30
20
10
0
Samples
cardiac
edl
soleus
001
002
003
004
005
006
007
008
009
010
011
012
013
014
015
016
017
018
019
020
021
022
023
024
025
026
027
028
029
030
031
032
033
034
035
036
037
038
039
040
041
042
043
044
045
046
047
048
049
050
Exonic Part Number

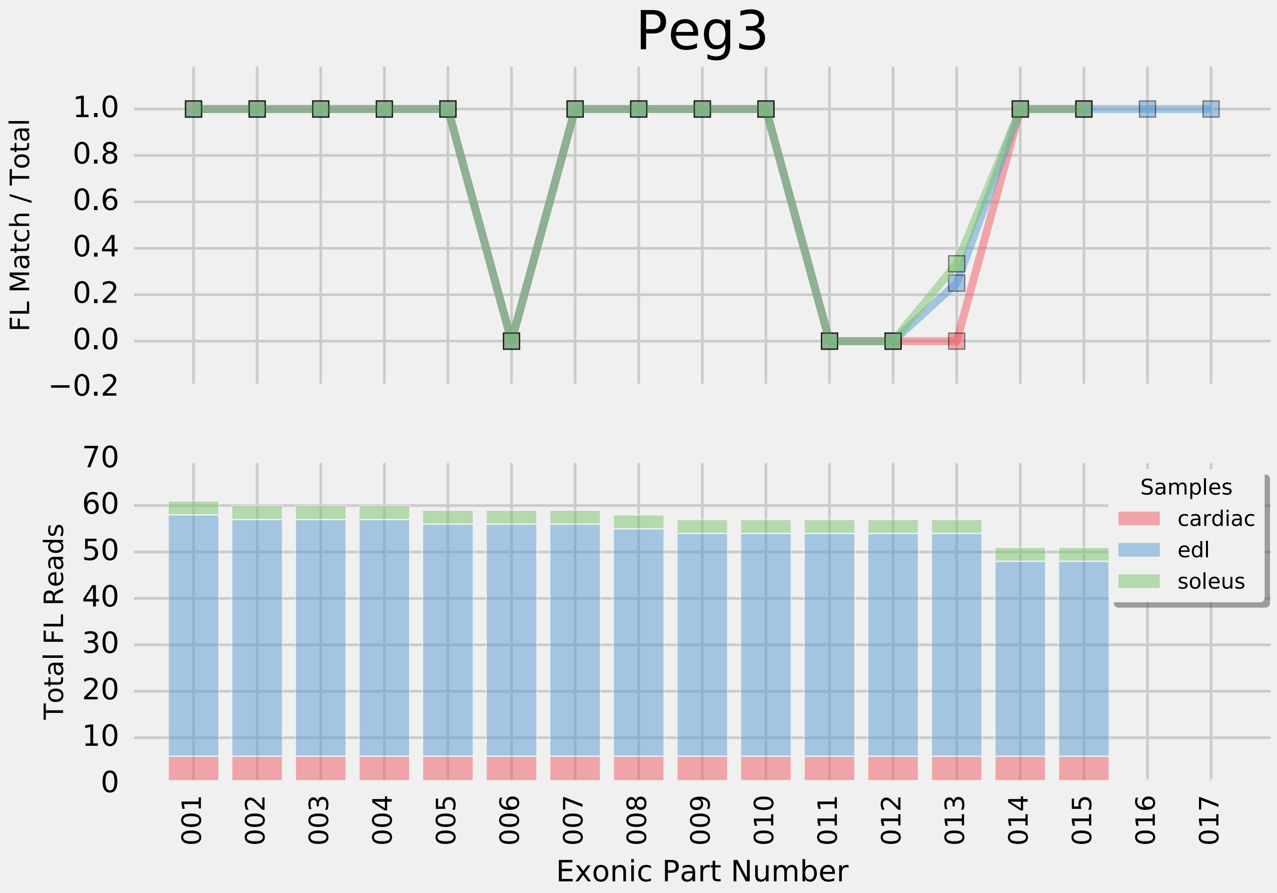

Peg3
FL Match / Total
1.0
0.8
0.6
0.4
0.2
0.0
−0.2
Total FL Reads
70
60
50
40
30
20
10
0
001
002
003
004
005
006
007
008
009
010
011
012
013
014
015
016
017
Exonic Part Number
Samples
cardiac
edl
soleus

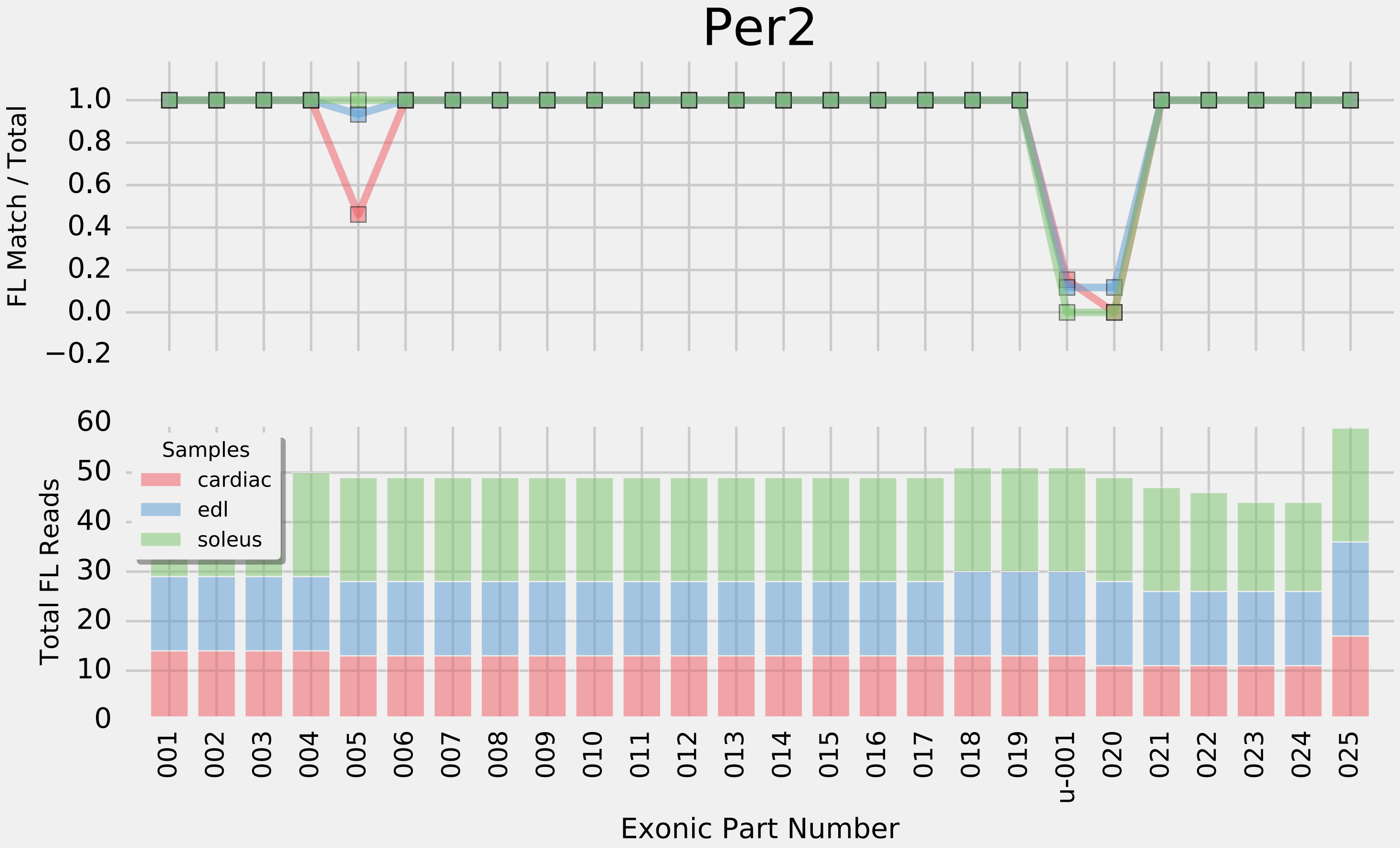

Per2
FL Match / Total
1.0
0.8
0.6
0.4
0.2
0.0
−0.2
Total FL Reads
60
50
40
30
20
10
0
Samples
cardiac
edl
soleus
001
002
003
004
005
006
007
008
009
010
011
012
013
014
015
016
017
018
019
u-001
020
021
022
023
024
025
Exonic Part Number

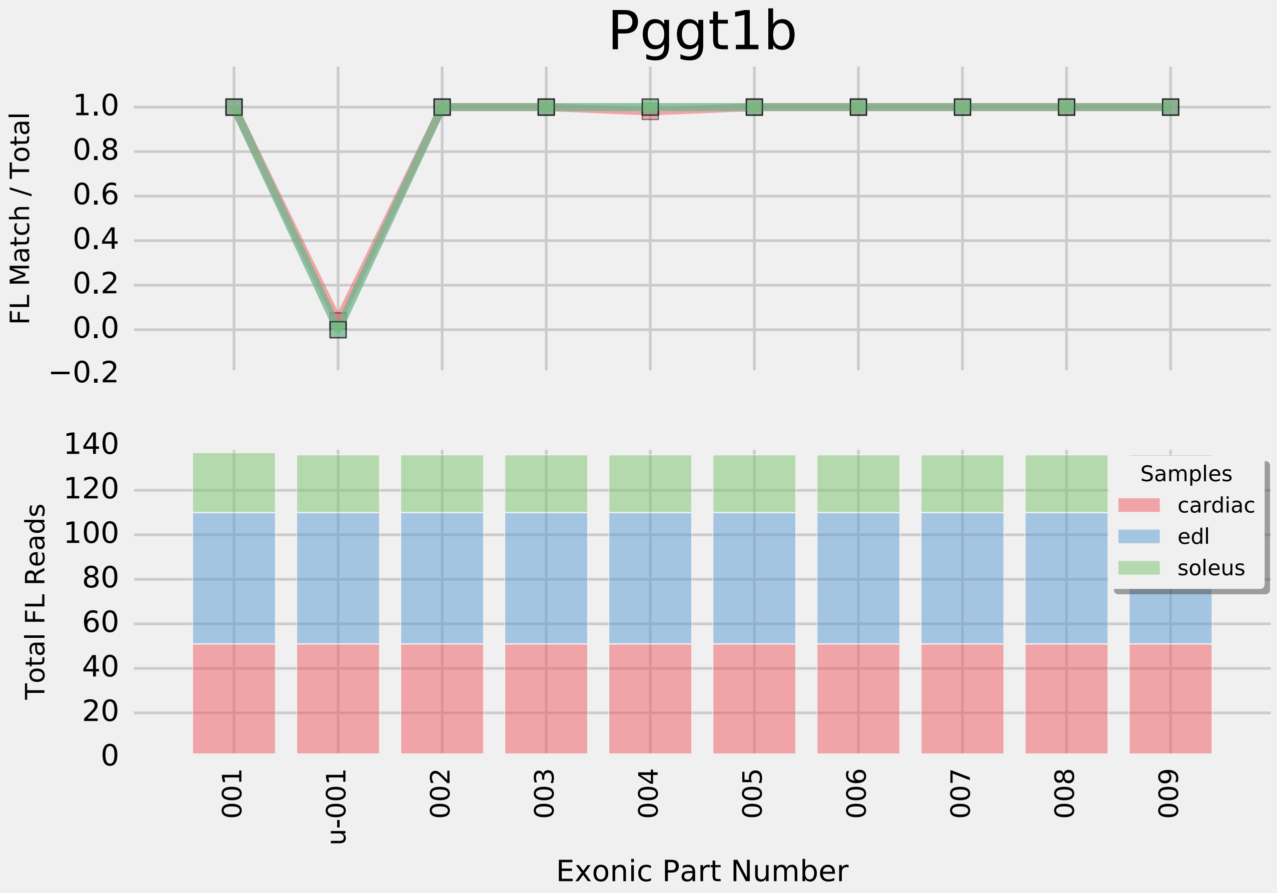

Pggt1b
FL Match / Total
1.0
0.8
0.6
0.4
0.2
0.0
−0.2
Total FL Reads
140
120
100
80
60
40
20
0
Samples
cardiac
edl
soleus
001
u-001
002
003
004
005
006
007
008
009
Exonic Part Number

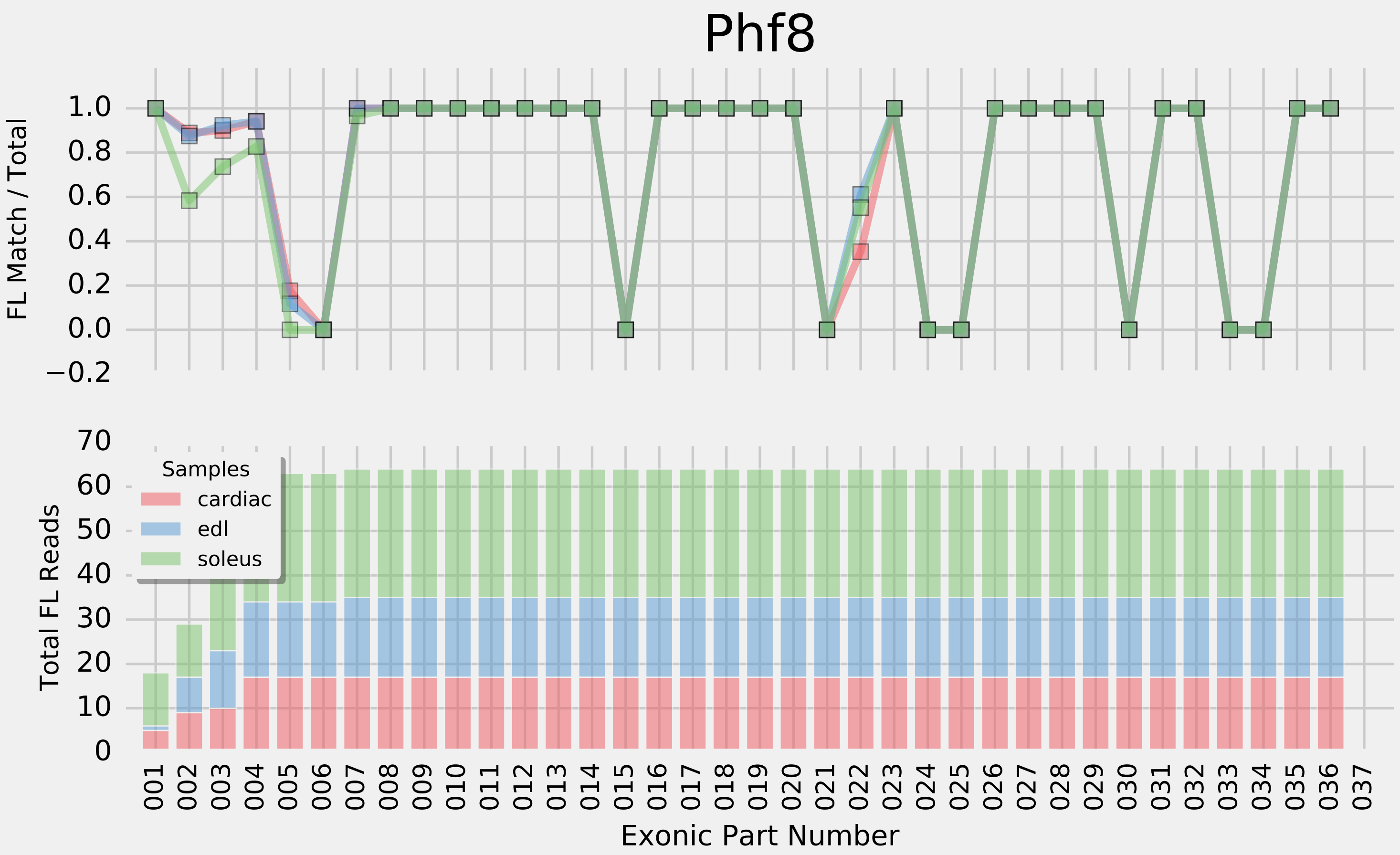

Phf8
FL Match / Total
1.0
0.8
0.6
0.4
0.2
0.0
−0.2
Total FL Reads
70
60
50
40
30
20
10
0
Samples
cardiac
edl
soleus
001
002
003
004
005
006
007
008
009
010
011
012
013
014
015
016
017
018
019
020
021
022
023
024
025
026
027
028
029
030
031
032
033
034
035
036
037
Exonic Part Number

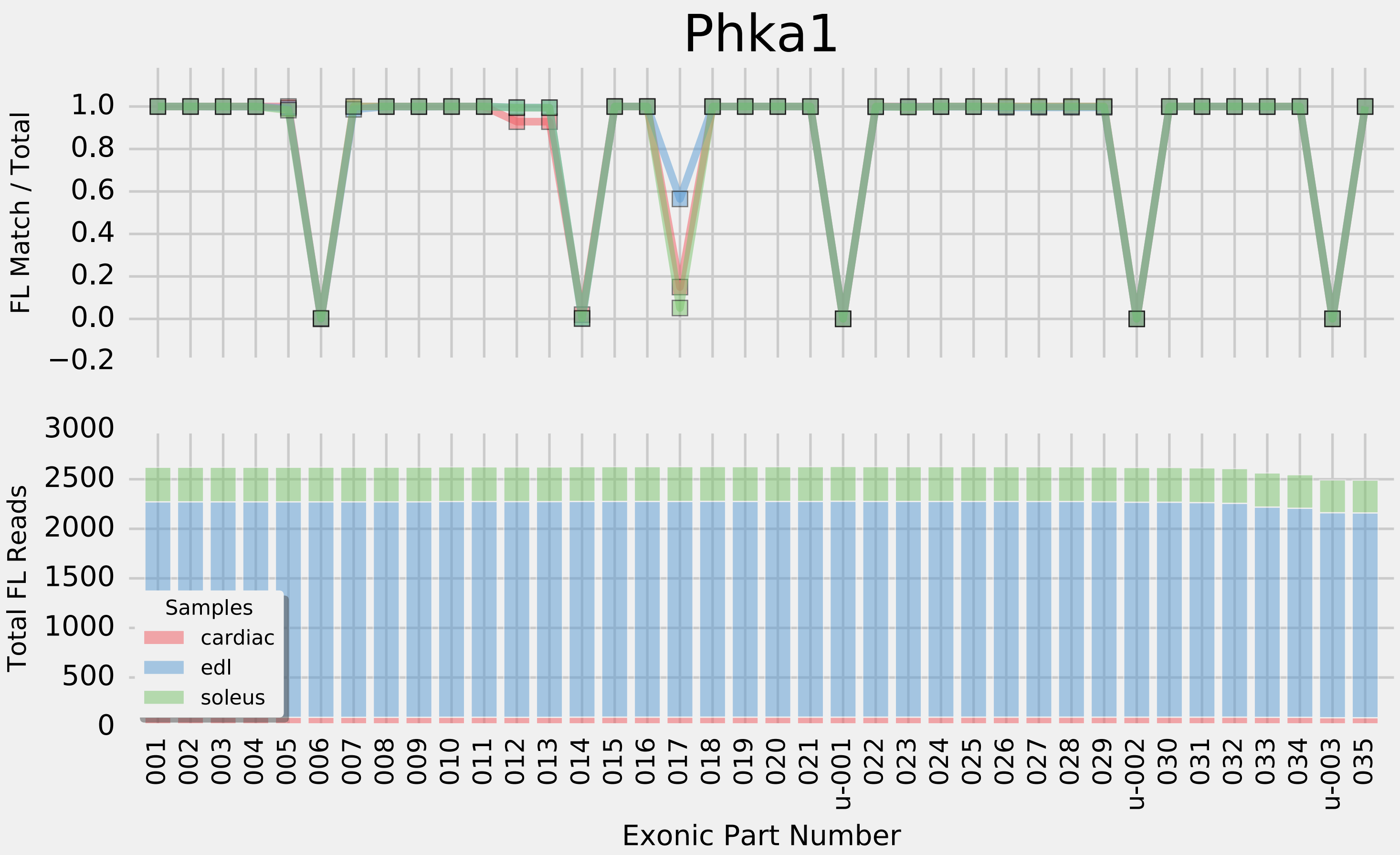

Phka1
FL Match / Total
1.0
0.8
0.6
0.4
0.2
0.0
−0.2
Total FL Reads
3000
2500
2000
1500
1000
500
0
Samples
cardiac
edl
soleus
001 002 003 004 005 006 007 008 009 010 011 012 013 014 015 016 017 018 019 020 021 u-001 022 023 024 025 026 027 028 029 u-002 030 031 032 033 034 u-003 035
Exonic Part Number

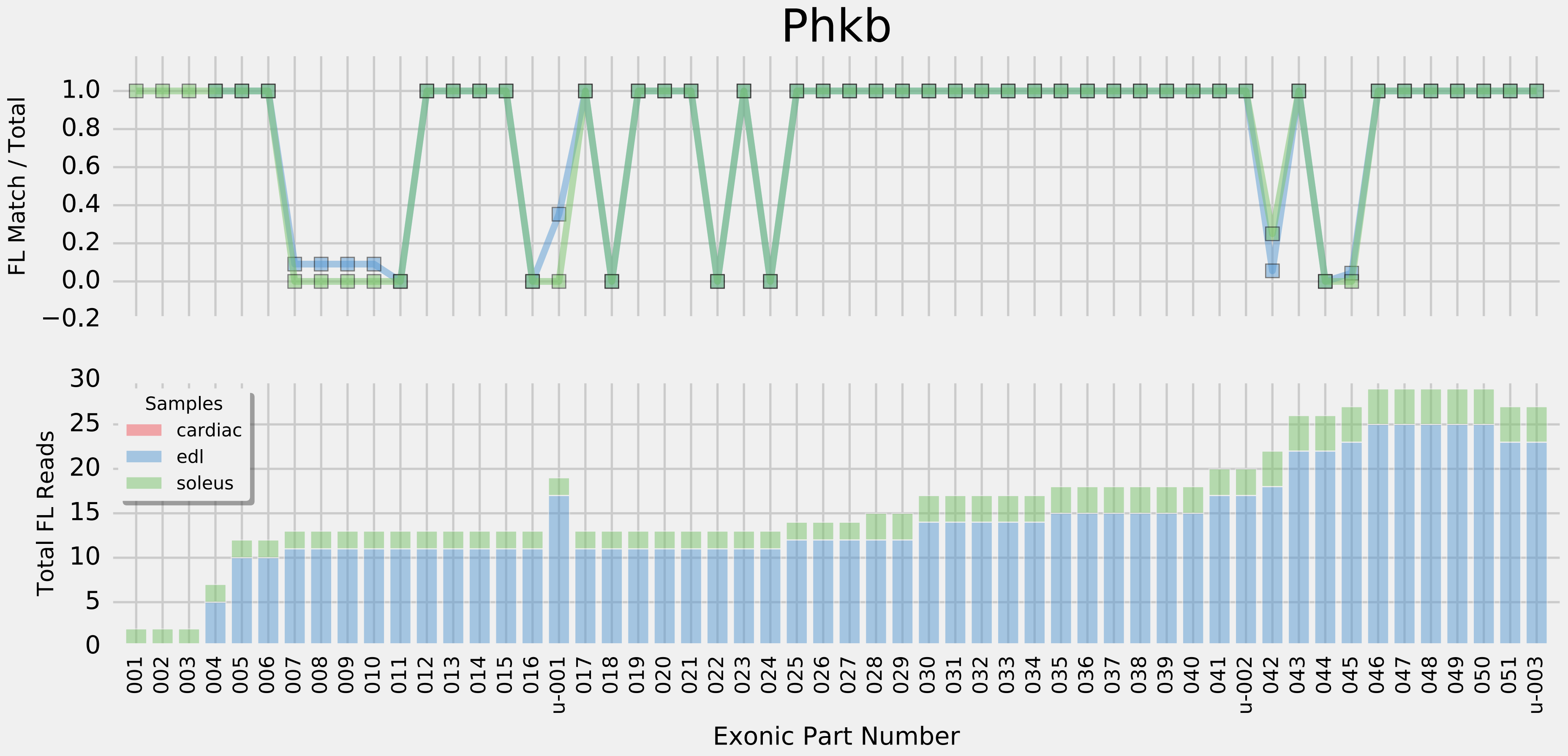

Phkb
FL Match / Total
1.0
0.8
0.6
0.4
0.2
0.0
−0.2
Total FL Reads
30
25
20
15
10
5
0
Samples
cardiac
edl
soleus
001 002 003 004 005 006 007 008 009 010 011 012 013 014 015 016 u-001 017 018 019 020 021 022 023 024 025 026 027 028 029 030 031 032 033 034 035 036 037 038 039 040 041 u-002 042 043 044 045 046 047 048 049 050 051 u-003
Exonic Part Number

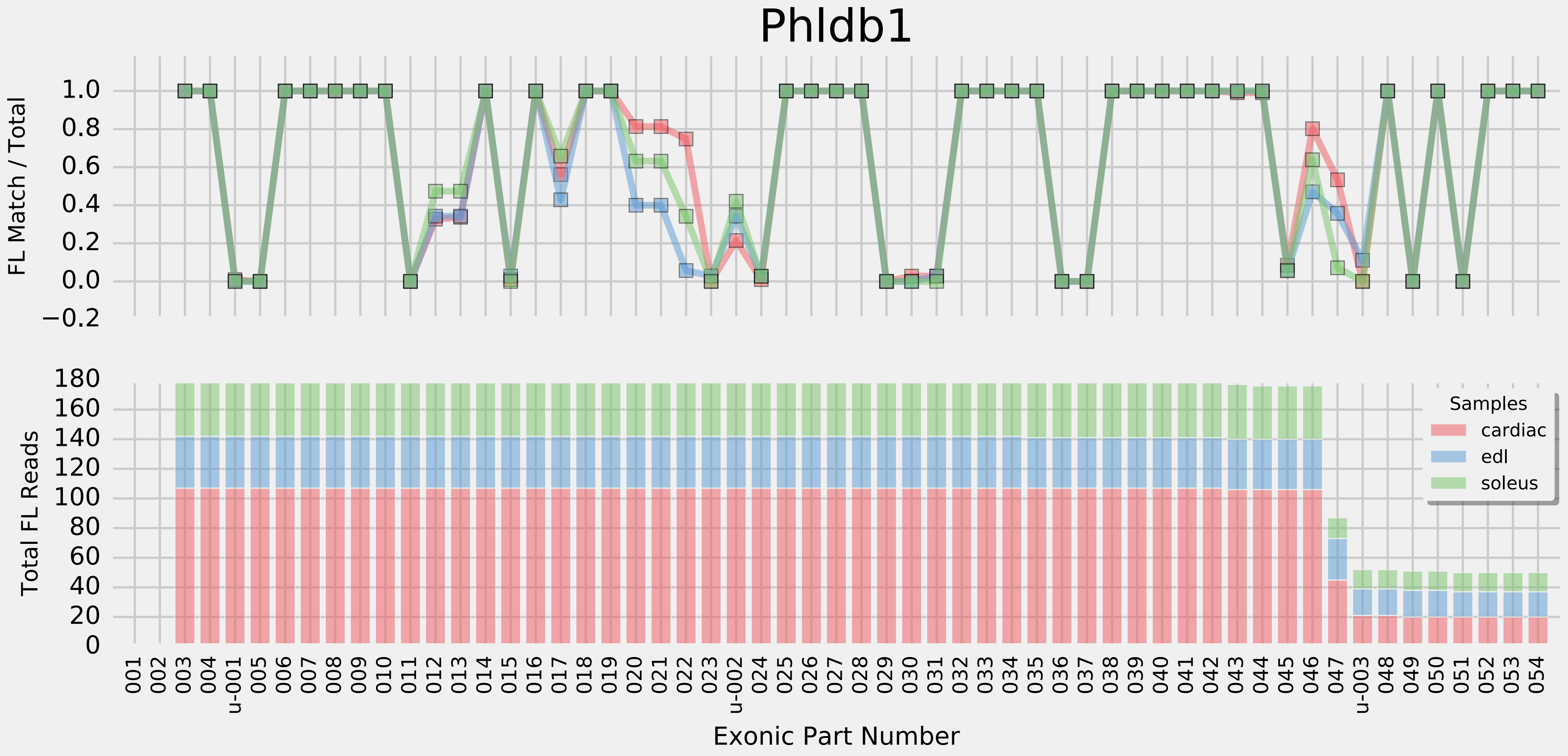

Phldb1
FL Match / Total
Total FL Reads
Samples
cardiac
edl
soleus
Exonic Part Number
001 002 003 004 u-001 005 006 007 008 009 010 011 012 013 014 015 016 017 018 019 020 021 022 023 u-002 024 025 026 027 028 029 030 031 032 033 034 035 036 037 038 039 040 041 042 043 044 045 046 047 u-003 048 049 050 051 052 053 054

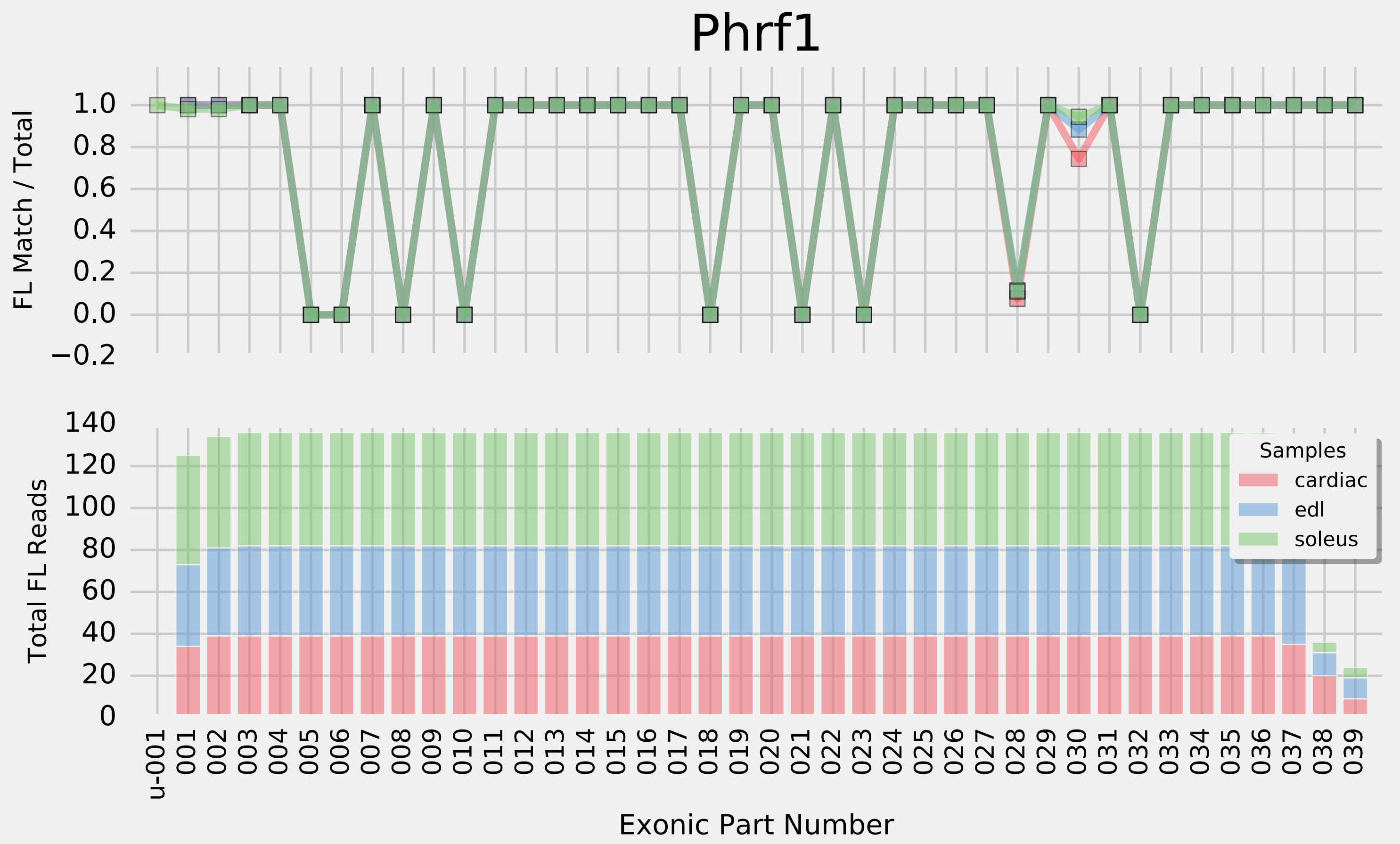

Phrf1
FL Match / Total
1.0
0.8
0.6
0.4
0.2
0.0
−0.2
Total FL Reads
140
120
100
80
60
40
20
0
Samples
cardiac
edl
soleus
u-001
001
002
003
004
005
006
007
008
009
010
011
012
013
014
015
016
017
018
019
020
021
022
023
024
025
026
027
028
029
030
031
032
033
034
035
036
037
038
039
Exonic Part Number

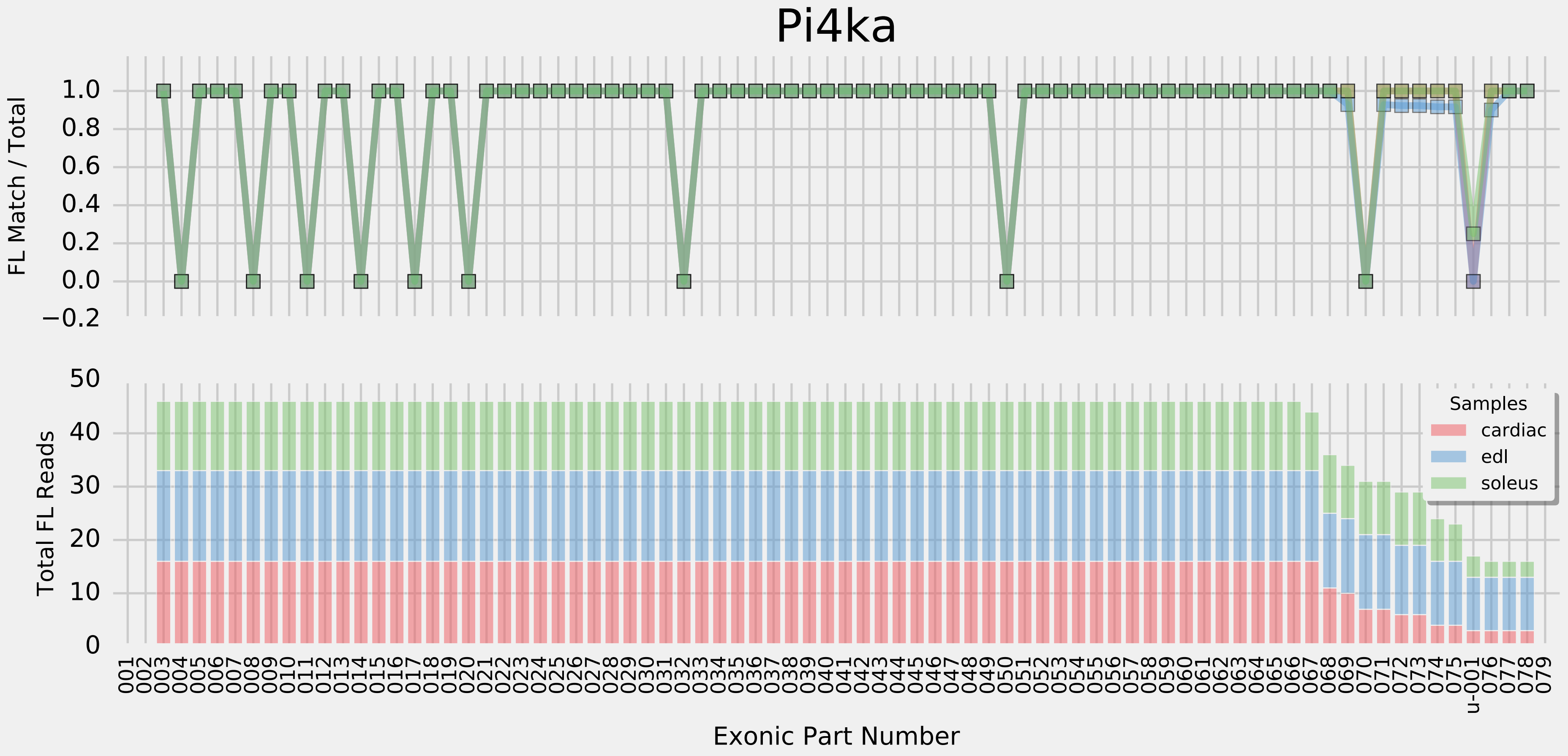

Pi4ka
FL Match / Total
1.0
0.8
0.6
0.4
0.2
0.0
−0.2
Total FL Reads
50
40
30
20
10
0
Samples
cardiac
edl
soleus
001 002 003 004 005 006 007 008 009 010 011 012 013 014 015 016 017 018 019 020 021 022 023 024 025 026 027 028 029 030 031 032 033 034 035 036 037 038 039 040 041 042 043 044 045 046 047 048 049 050 051 052 053 054 055 056 057 058 059 060 061 062 063 064 065 066 067 068 069 070 071 072 073 074 075 u-001 076 077 078 079
Exonic Part Number

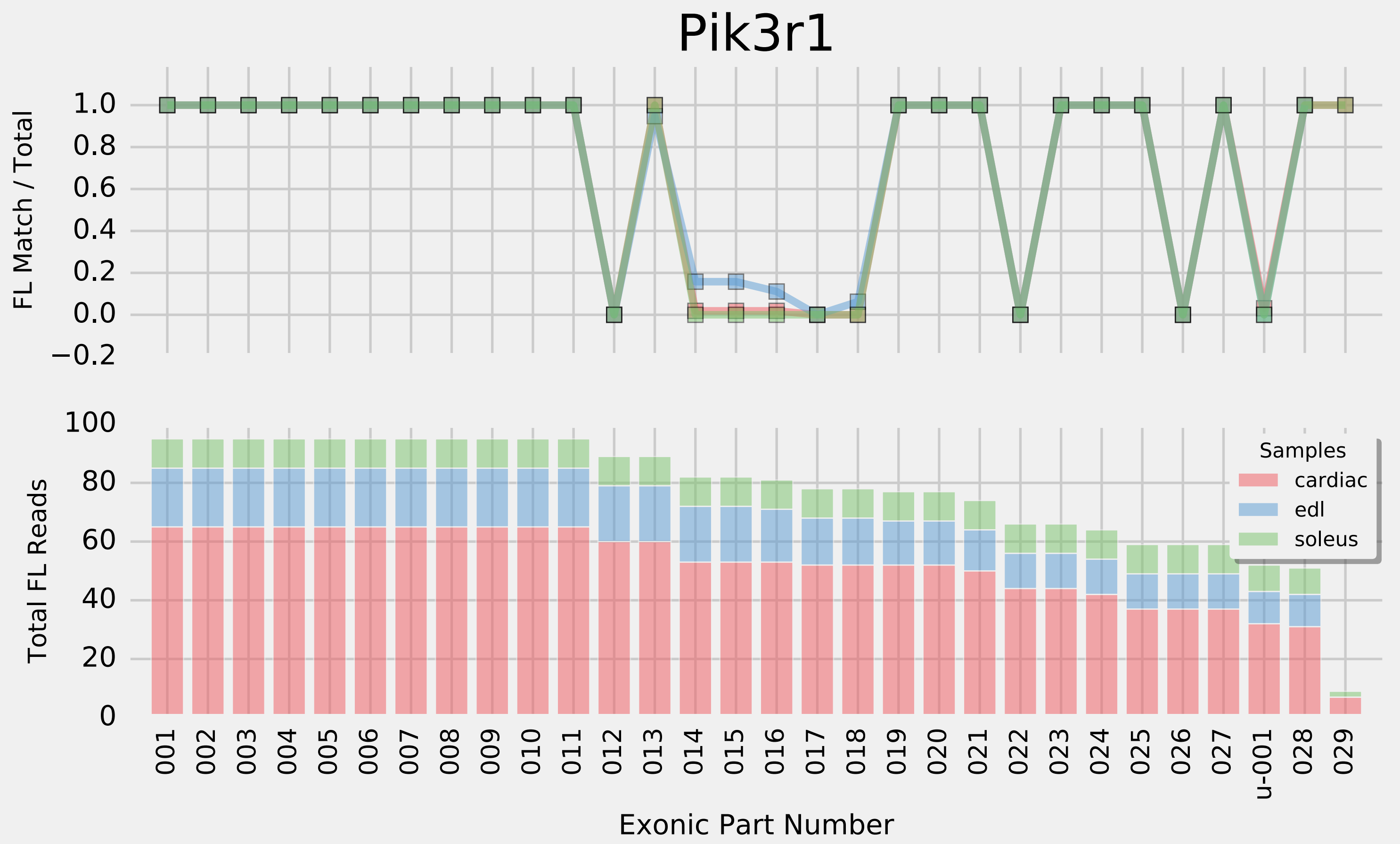

Pik3r1
FL Match / Total
1.0
0.8
0.6
0.4
0.2
0.0
−0.2
Total FL Reads
100
80
60
40
20
0
Samples
cardiac
edl
soleus
001
002
003
004
005
006
007
008
009
010
011
012
013
014
015
016
017
018
019
020
021
022
023
024
025
026
027
u-001
028
029
Exonic Part Number

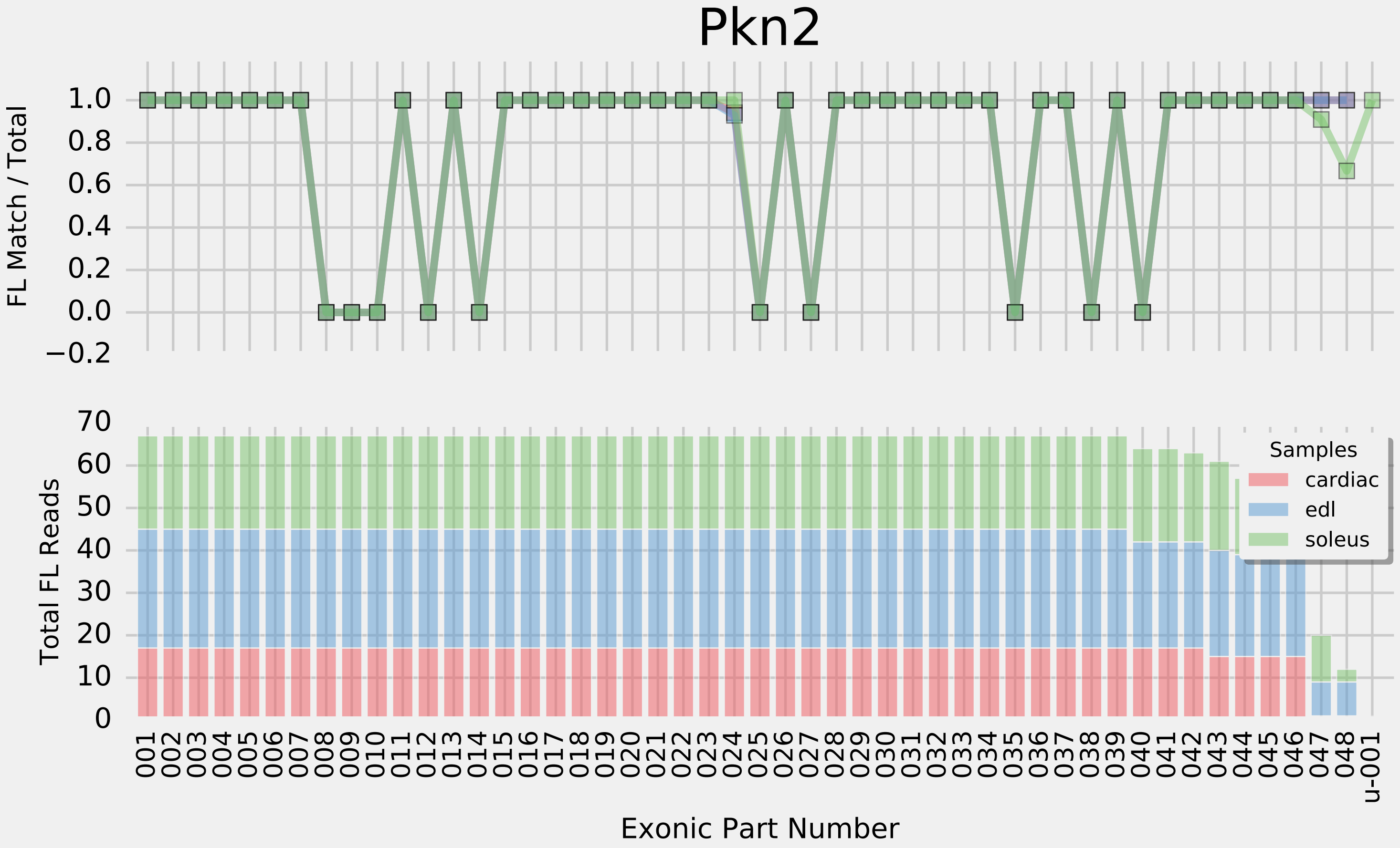

Pkn2
FL Match / Total
1.0
0.8
0.6
0.4
0.2
0.0
−0.2
Total FL Reads
70
60
50
40
30
20
10
0
Samples
cardiac
edl
soleus
001 002 003 004 005 006 007 008 009 010 011 012 013 014 015 016 017 018 019 020 021 022 023 024 025 026 027 028 029 030 031 032 033 034 035 036 037 038 039 040 041 042 043 044 045 046 047 048 u-001
Exonic Part Number

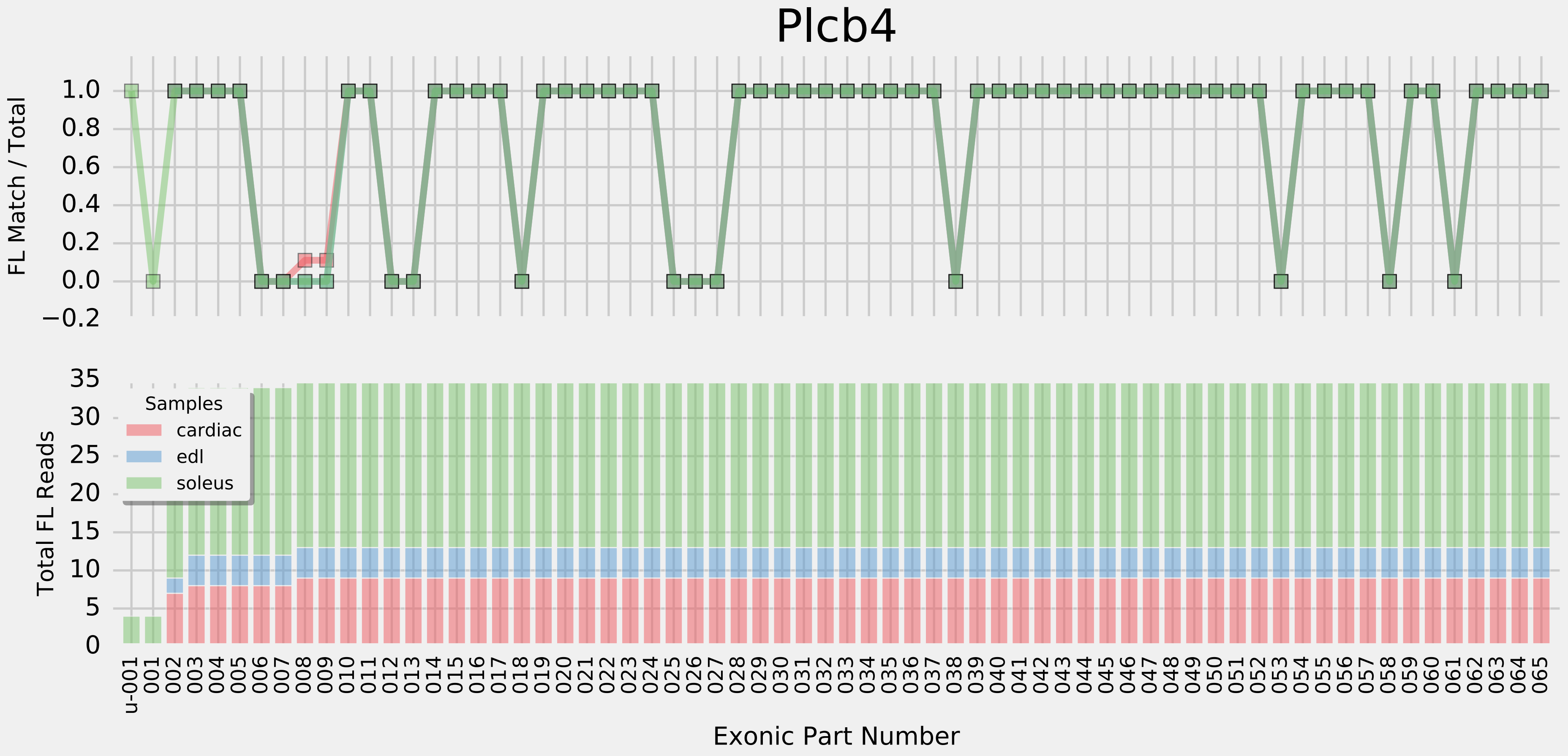

Plcb4
FL Match / Total
1.0
0.8
0.6
0.4
0.2
0.0
−0.2
Total FL Reads
35
30
25
20
15
10
5
0
Samples
cardiac
edl
soleus
u-001 001 002 003 004 005 006 007 008 009 010 011 012 013 014 015 016 017 018 019 020 021 022 023 024 025 026 027 028 029 030 031 032 033 034 035 036 037 038 039 040 041 042 043 044 045 046 047 048 049 050 051 052 053 054 055 056 057 058 059 060 061 062 063 064 065
Exonic Part Number

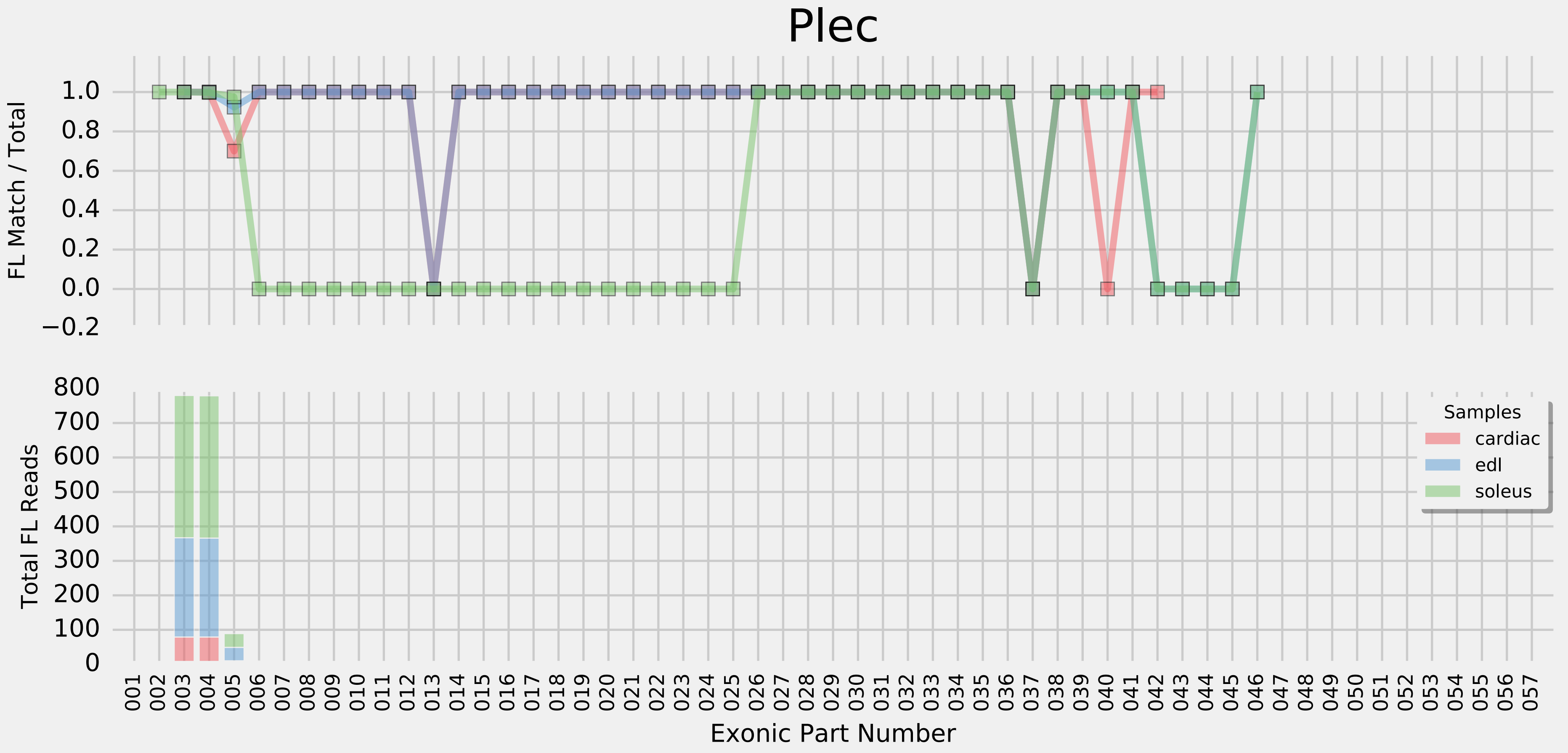

Plec
FL Match / Total
1.0
0.8
0.6
0.4
0.2
0.0
−0.2
Total FL Reads
800
700
600
500
400
300
200
100
0
Samples
cardiac
edl
soleus
001 002 003 004 005 006 007 008 009 010 011 012 013 014 015 016 017 018 019 020 021 022 023 024 025 026 027 028 029 030 031 032 033 034 035 036 037 038 039 040 041 042 043 044 045 046 047 048 049 050 051 052 053 054 055 056 057
Exonic Part Number

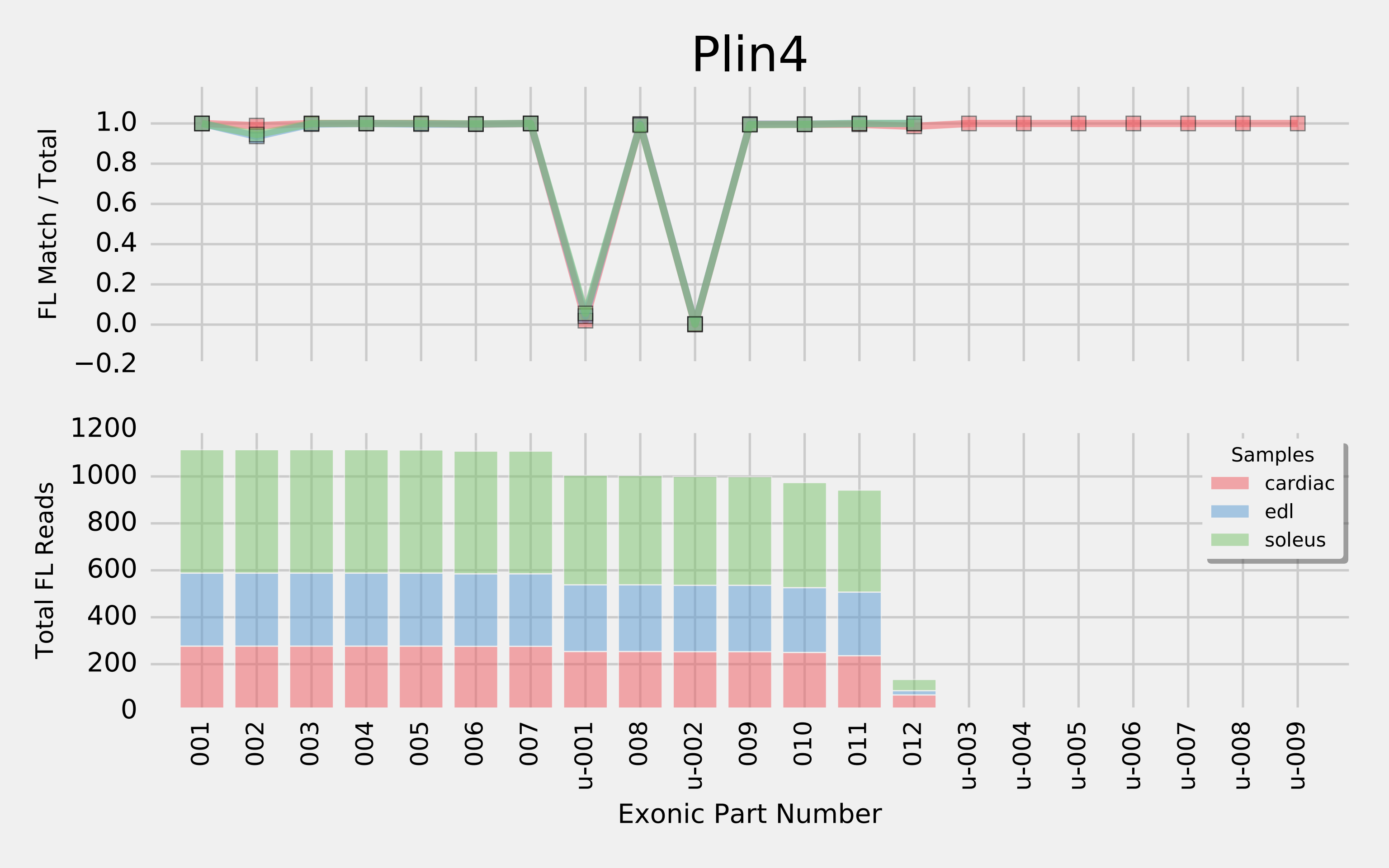

Plin4
FL Match / Total
1.0
0.8
0.6
0.4
0.2
0.0
−0.2
Total FL Reads
1200
1000
800
600
400
200
0
Samples
cardiac
edl
soleus
001
002
003
004
005
006
007
u-001
008
u-002
009
010
011
012
u-003
u-004
u-005
u-006
u-007
u-008
u-009
Exonic Part Number

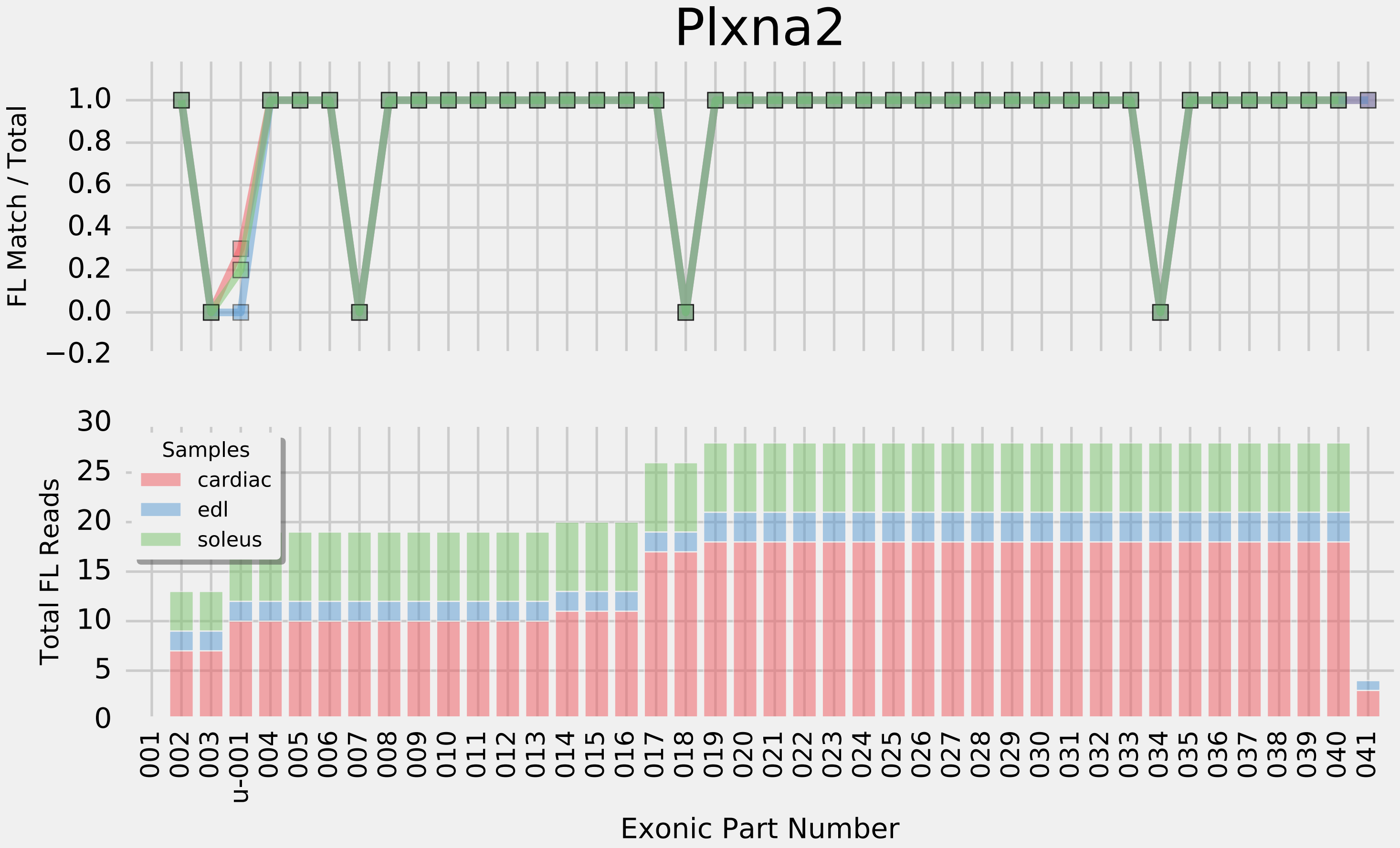
Plxna2
FL Match / Total
1.0
0.8
0.6
0.4
0.2
0.0
−0.2
Total FL Reads
30
25
20
15
10
5
0
Samples
cardiac
edl
soleus
001
002
003
u-001
004
005
006
007
008
009
010
011
012
013
014
015
016
017
018
019
020
021
022
023
024
025
026
027
028
029
030
031
032
033
034
035
036
037
038
039
040
041
Exonic Part Number

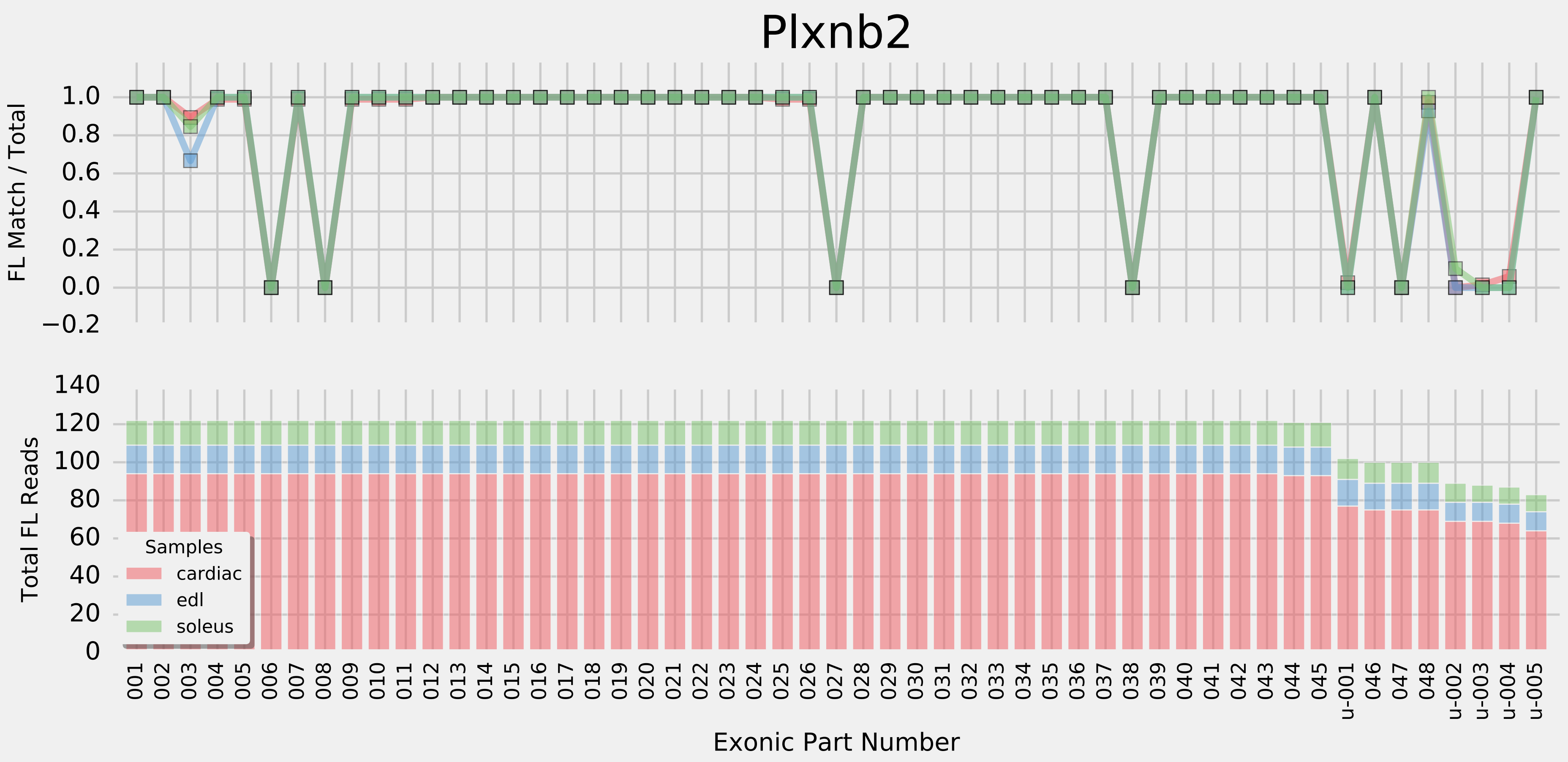

Plxnb2
FL Match / Total
1.0
0.8
0.6
0.4
0.2
0.0
−0.2
140
120
100
80
60
40
20
0
Total FL Reads
Samples
cardiac
edl
soleus
001 002 003 004 005 006 007 008 009 010 011 012 013 014 015 016 017 018 019 020 021 022 023 024 025 026 027 028 029 030 031 032 033 034 035 036 037 038 039 040 041 042 043 044 045 u-001 046 047 048 u-002 u-003 u-004 u-005
Exonic Part Number

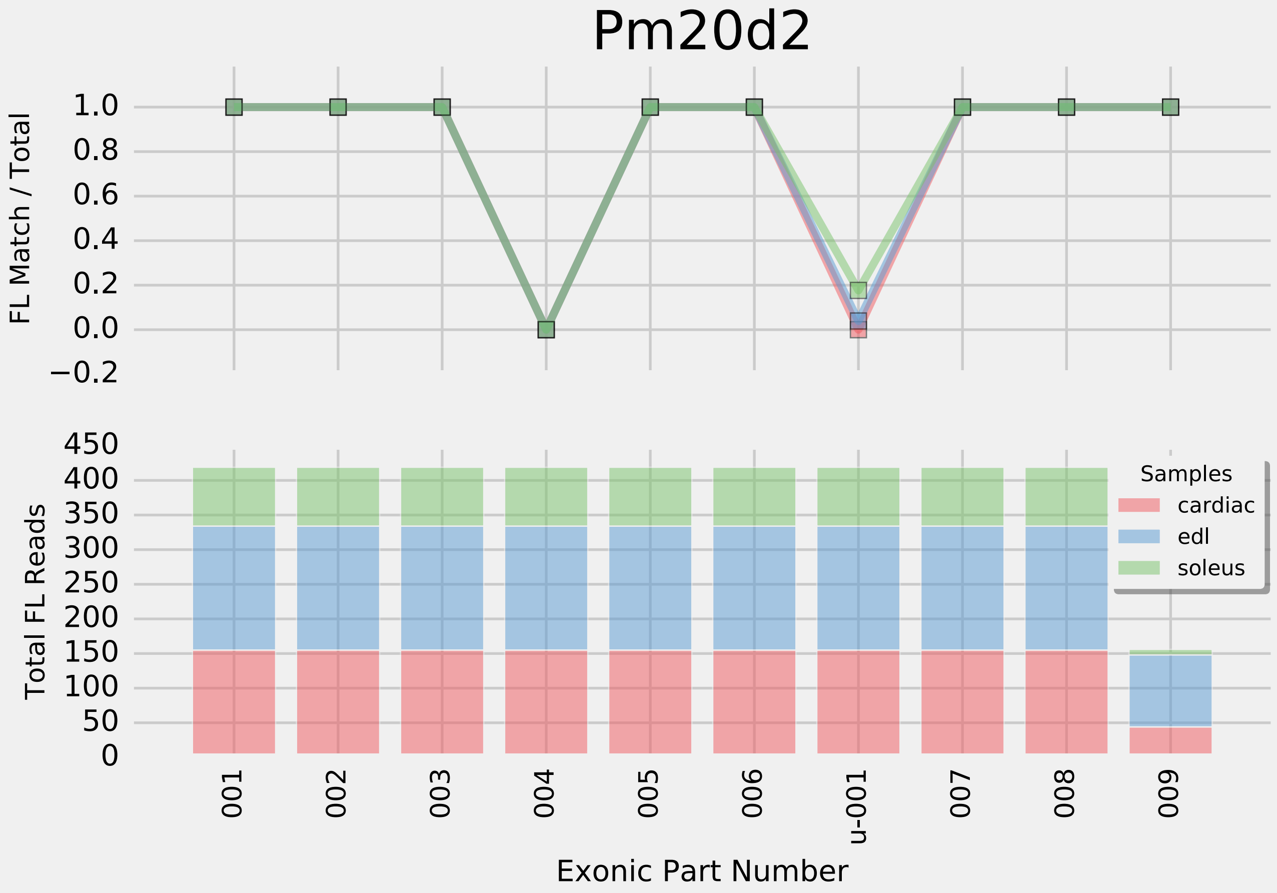

Pm20d2
FL Match / Total
1.0
0.8
0.6
0.4
0.2
0.0
−0.2
Total FL Reads
450
400
350
300
250
200
150
100
50
0
Samples
cardiac
edl
soleus
001
002
003
004
005
006
u-001
007
008
009
Exonic Part Number

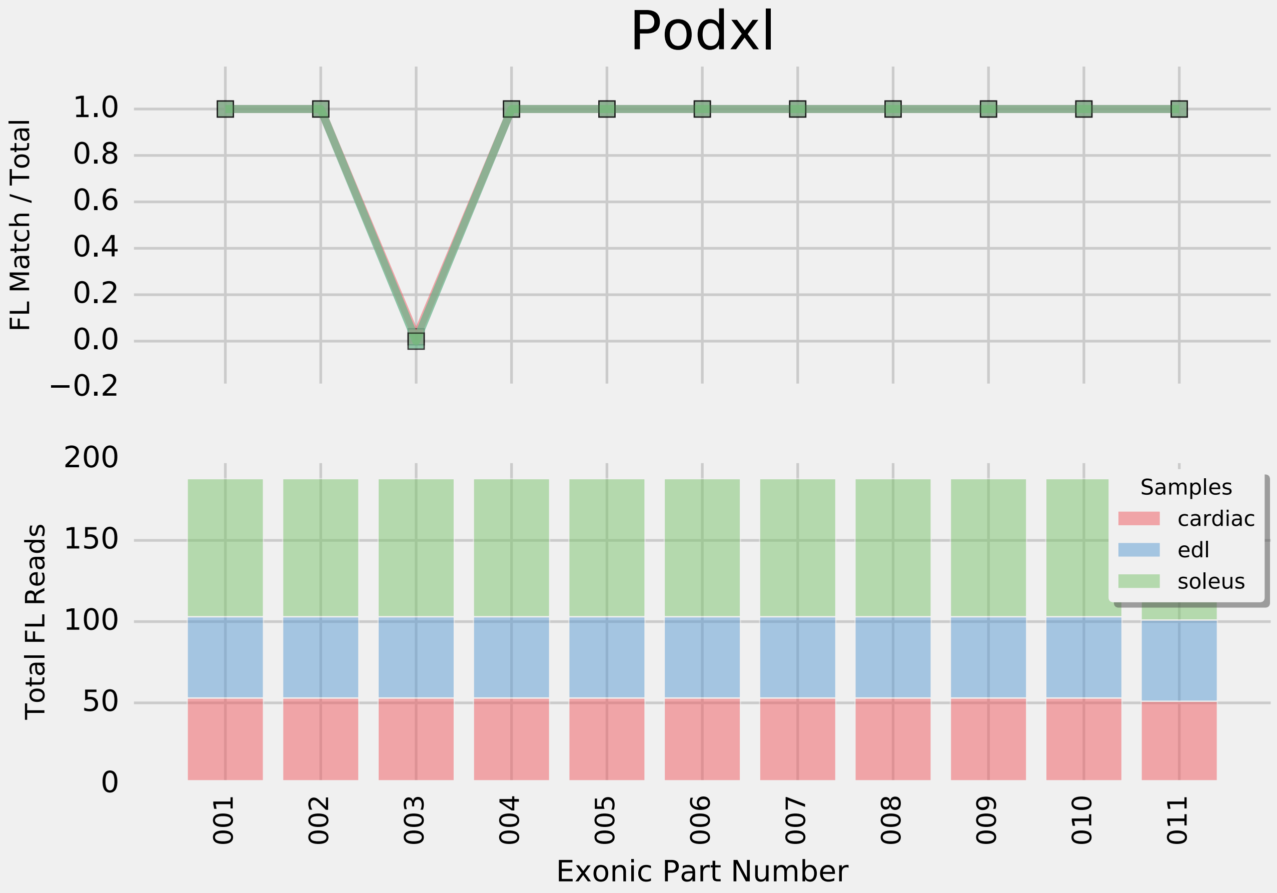

Podxl
FL Match / Total
1.0
0.8
0.6
0.4
0.2
0.0
−0.2
Total FL Reads
200
150
100
50
0
Samples
cardiac
edl
soleus
001
002
003
004
005
006
007
008
009
010
011
Exonic Part Number

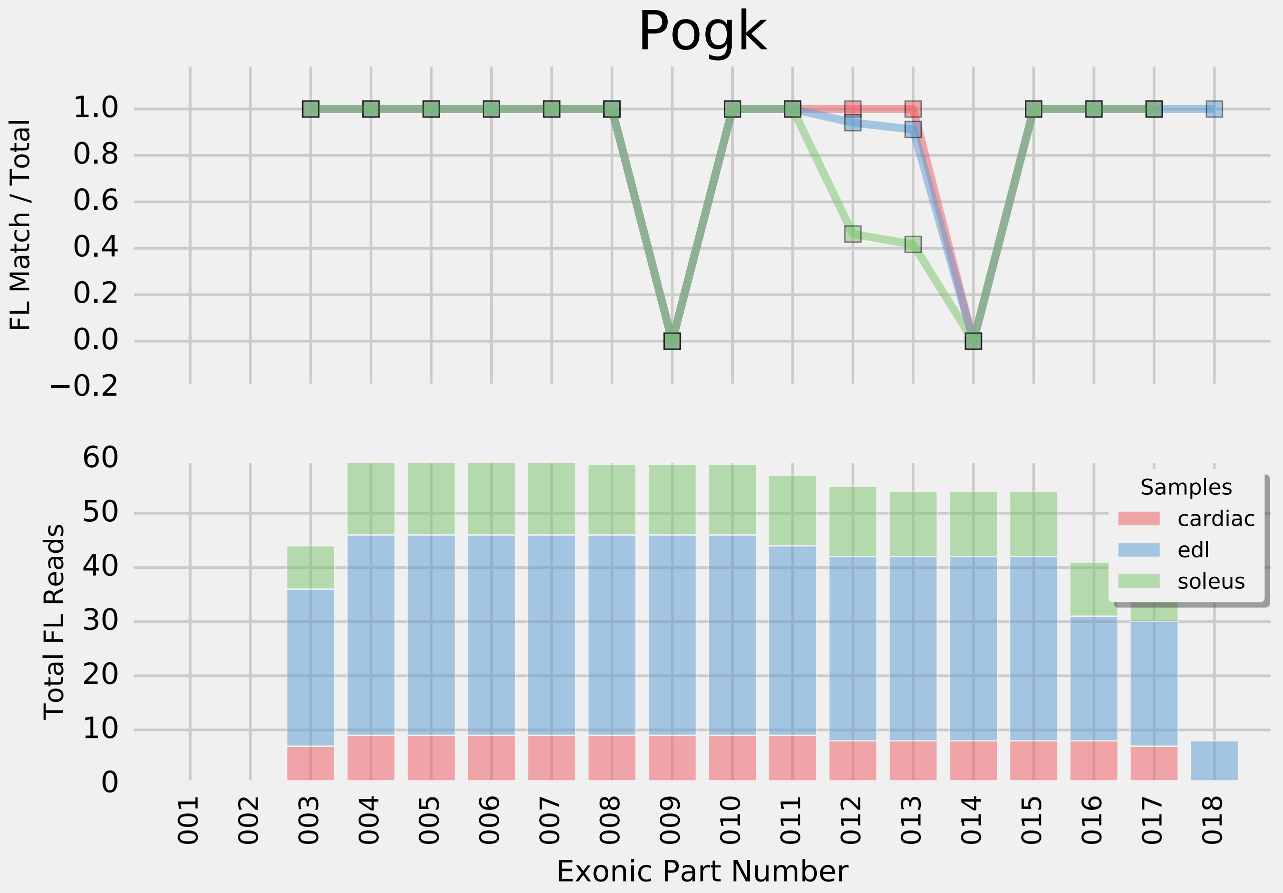

Pogk
FL Match / Total
1.0
0.8
0.6
0.4
0.2
0.0
−0.2
Total FL Reads
60
50
40
30
20
10
0
Samples
cardiac
edl
soleus
001
002
003
004
005
006
007
008
009
010
011
012
013
014
015
016
017
018
Exonic Part Number

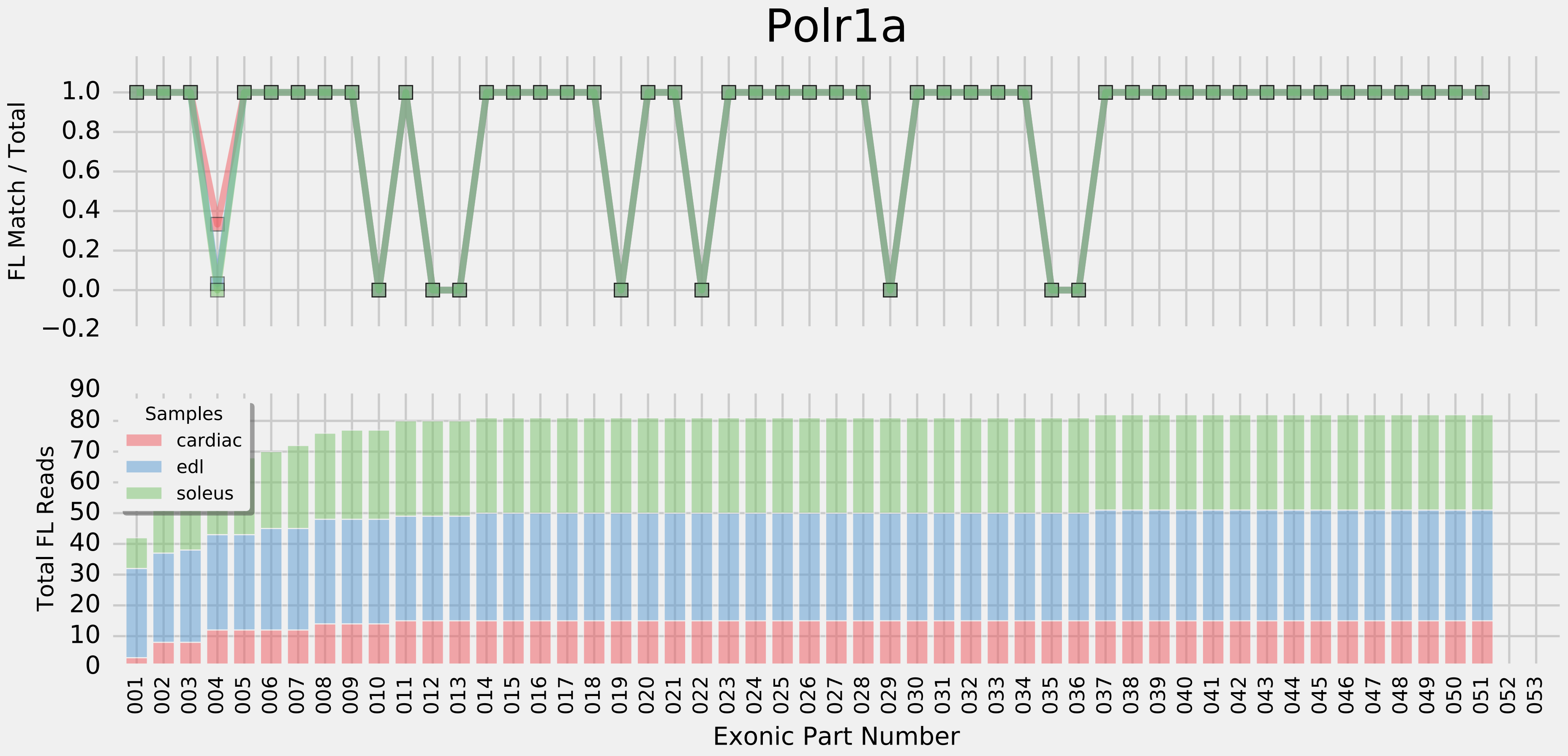

Polr1a
FL Match / Total
1.0
0.8
0.6
0.4
0.2
0.0
−0.2
Total FL Reads
90
80
70
60
50
40
30
20
10
0
Samples
cardiac
edl
soleus
001 002 003 004 005 006 007 008 009 010 011 012 013 014 015 016 017 018 019 020 021 022 023 024 025 026 027 028 029 030 031 032 033 034 035 036 037 038 039 040 041 042 043 044 045 046 047 048 049 050 051 052 053
Exonic Part Number

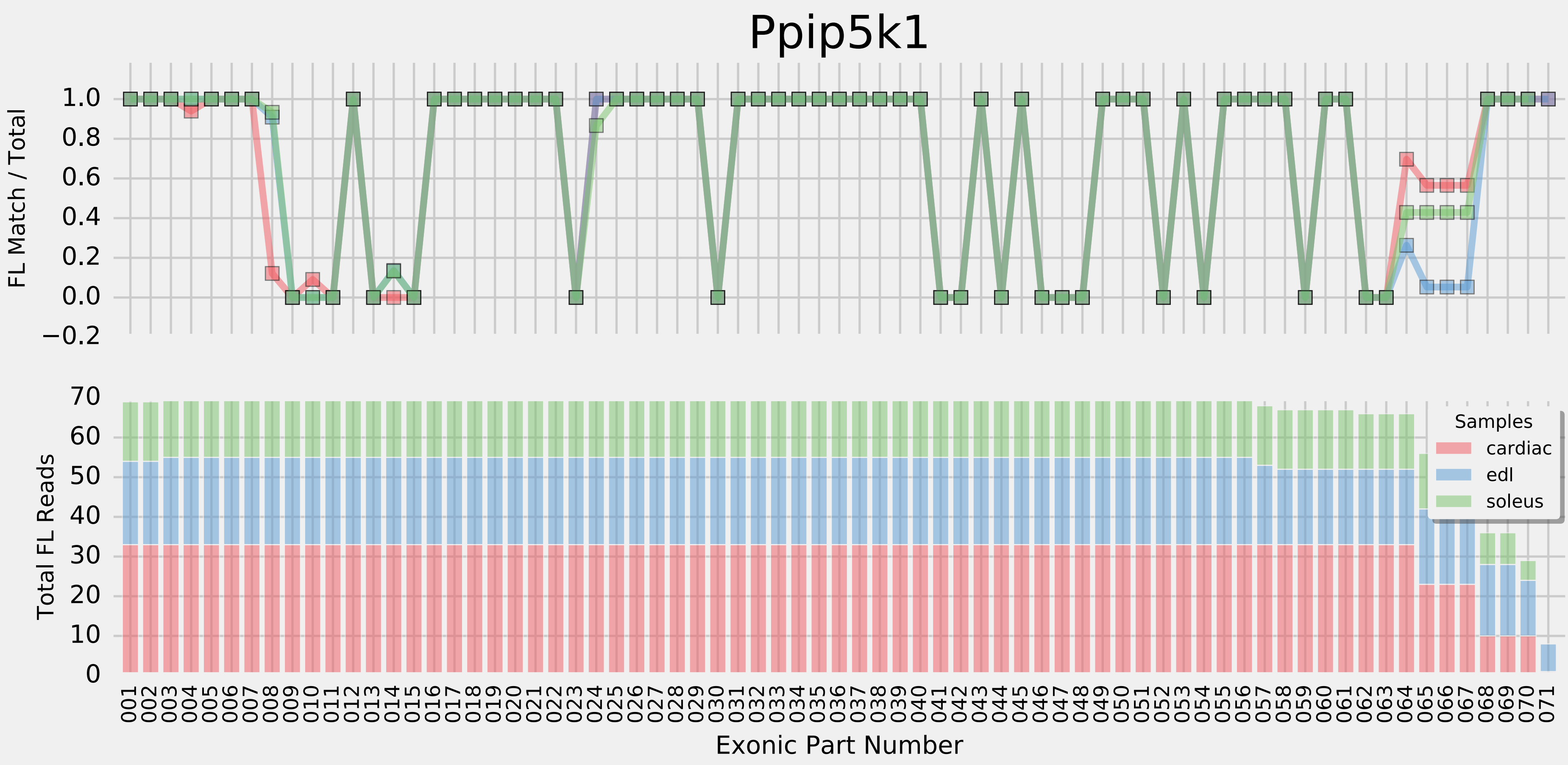

Ppip5k1
FL Match / Total
Total FL Reads
Samples
cardiac
edl
soleus
Exonic Part Number

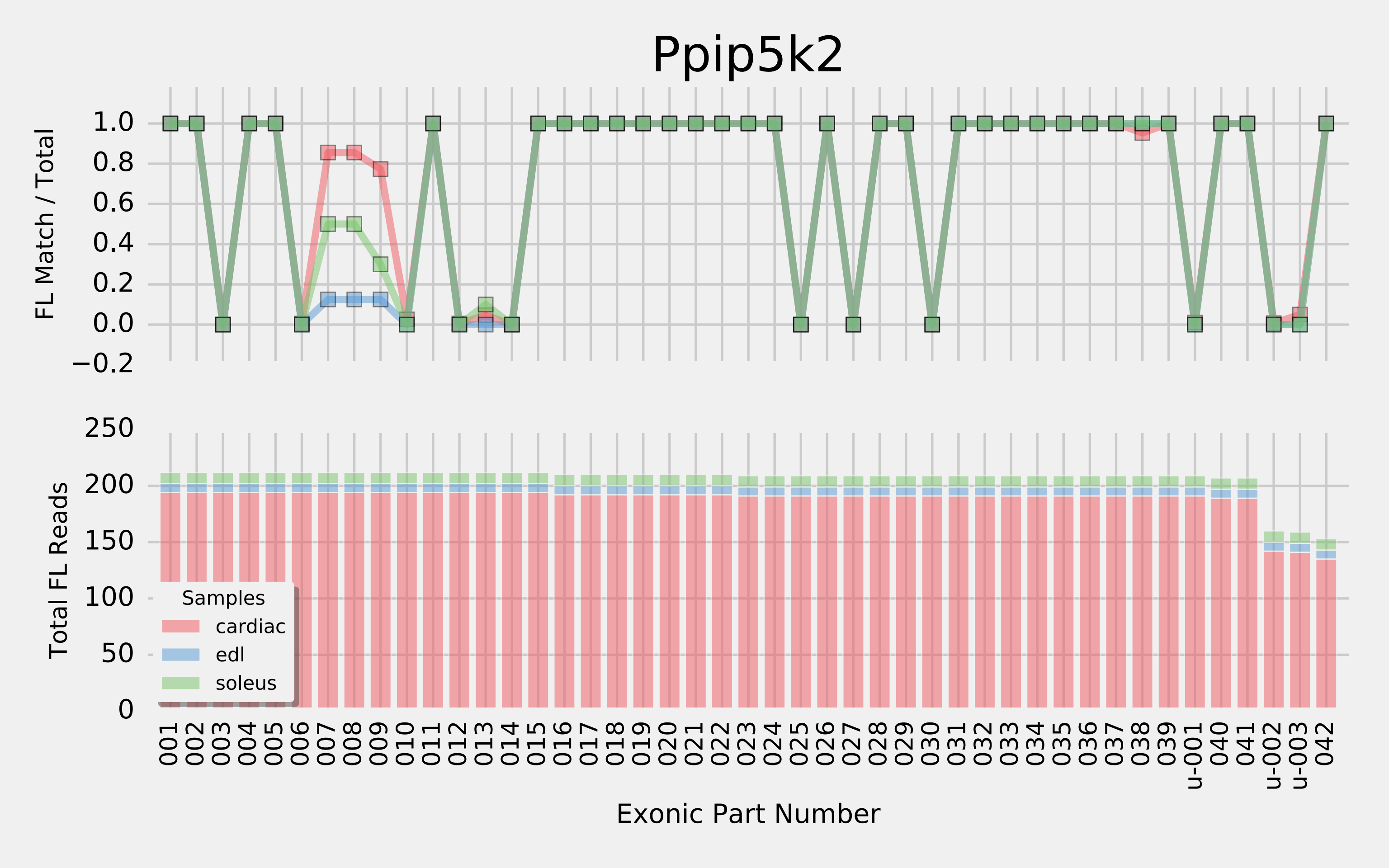

Ppip5k2
FL Match / Total
1.0
0.8
0.6
0.4
0.2
0.0
−0.2
Total FL Reads
250
200
150
100
50
0
Samples
cardiac
edl
soleus
001 002 003 004 005 006 007 008 009 010 011 012 013 014 015 016 017 018 019 020 021 022 023 024 025 026 027 028 029 030 031 032 033 034 035 036 037 038 039 u-001 040 041 u-002 u-003 042
Exonic Part Number

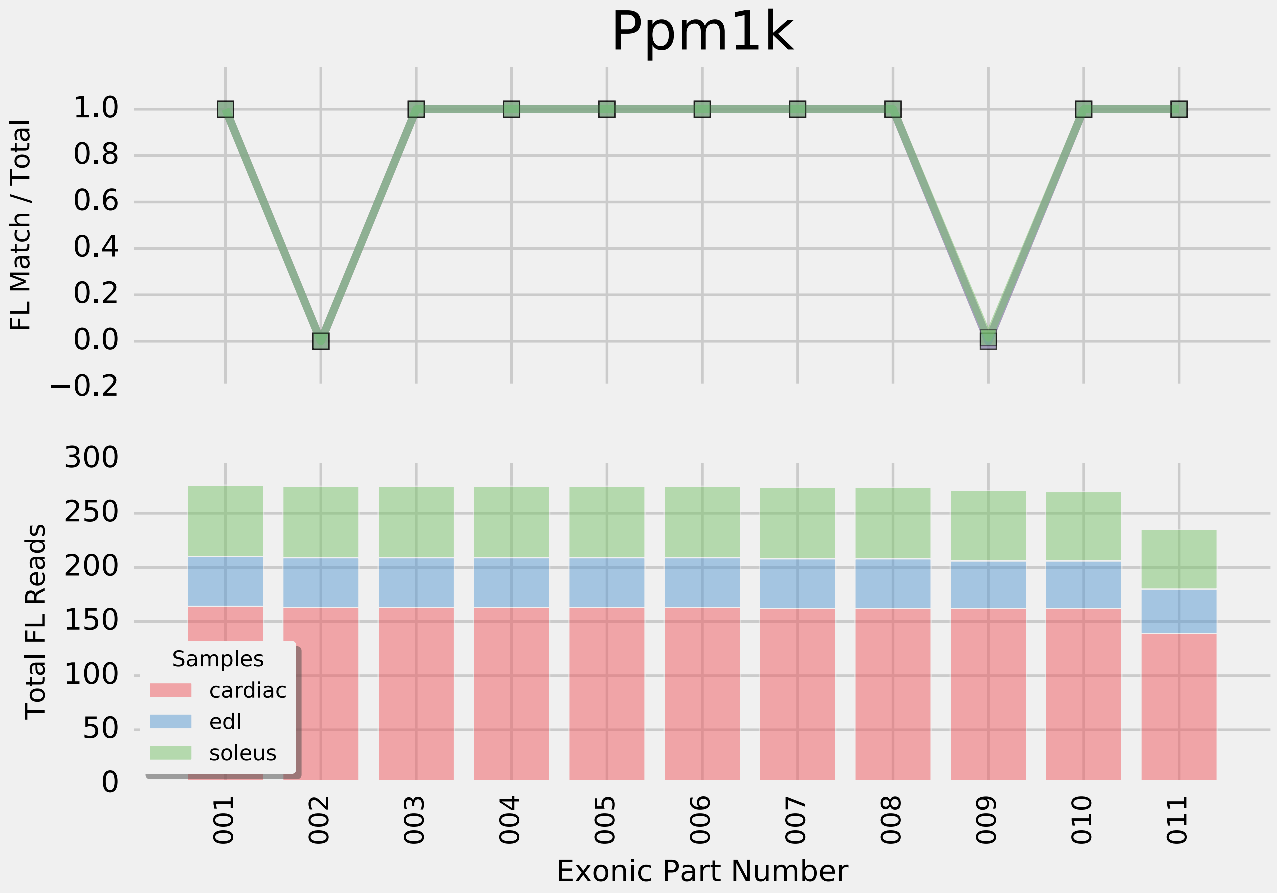

Ppm1k
FL Match / Total
1.0
0.8
0.6
0.4
0.2
0.0
−0.2
Total FL Reads
300
250
200
150
100
50
0
Samples
cardiac
edl
soleus
001
002
003
004
005
006
007
008
009
010
011
Exonic Part Number

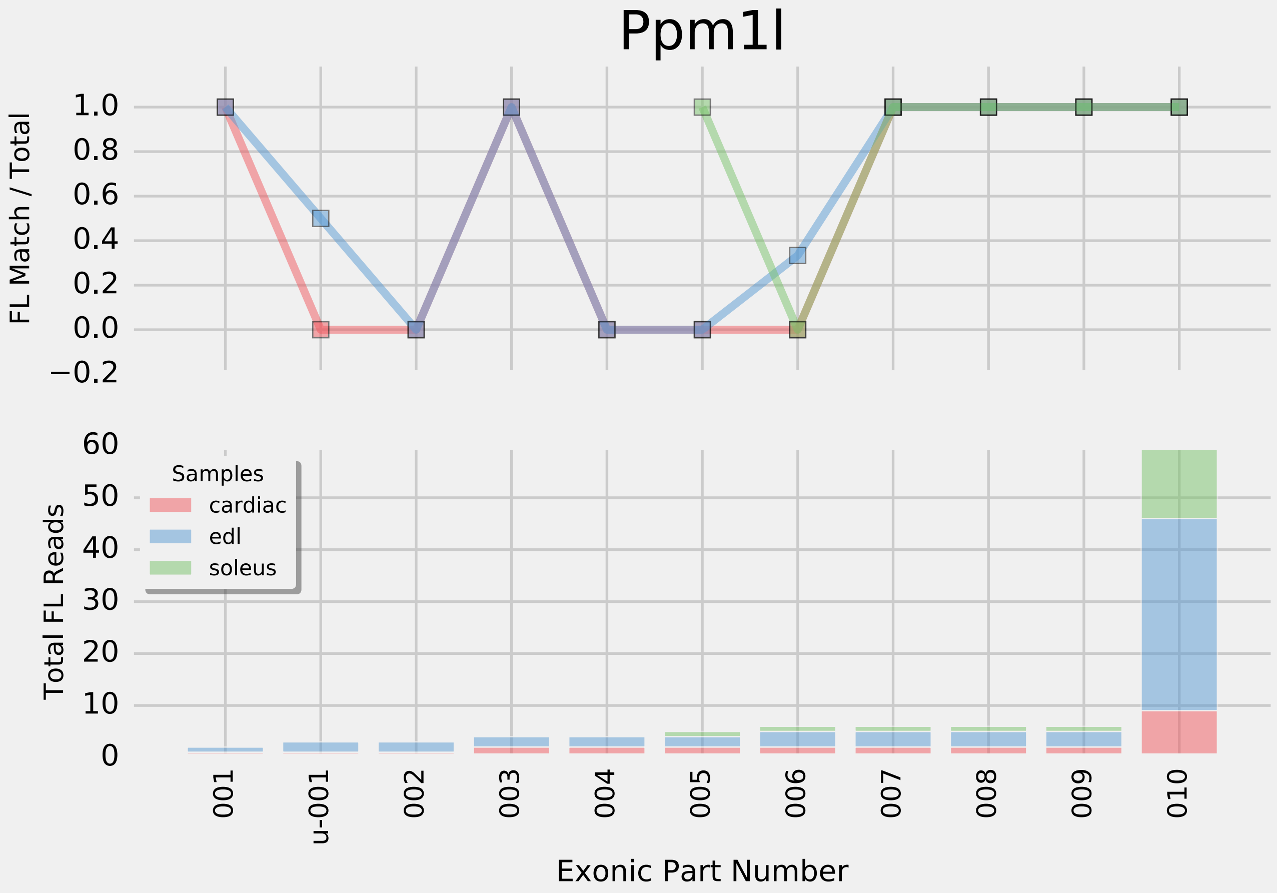

Ppm1l
FL Match / Total
1.0
0.8
0.6
0.4
0.2
0.0
−0.2
Total FL Reads
60
50
40
30
20
10
0
Samples
cardiac
edl
soleus
001
u-001
002
003
004
005
006
007
008
009
010
Exonic Part Number

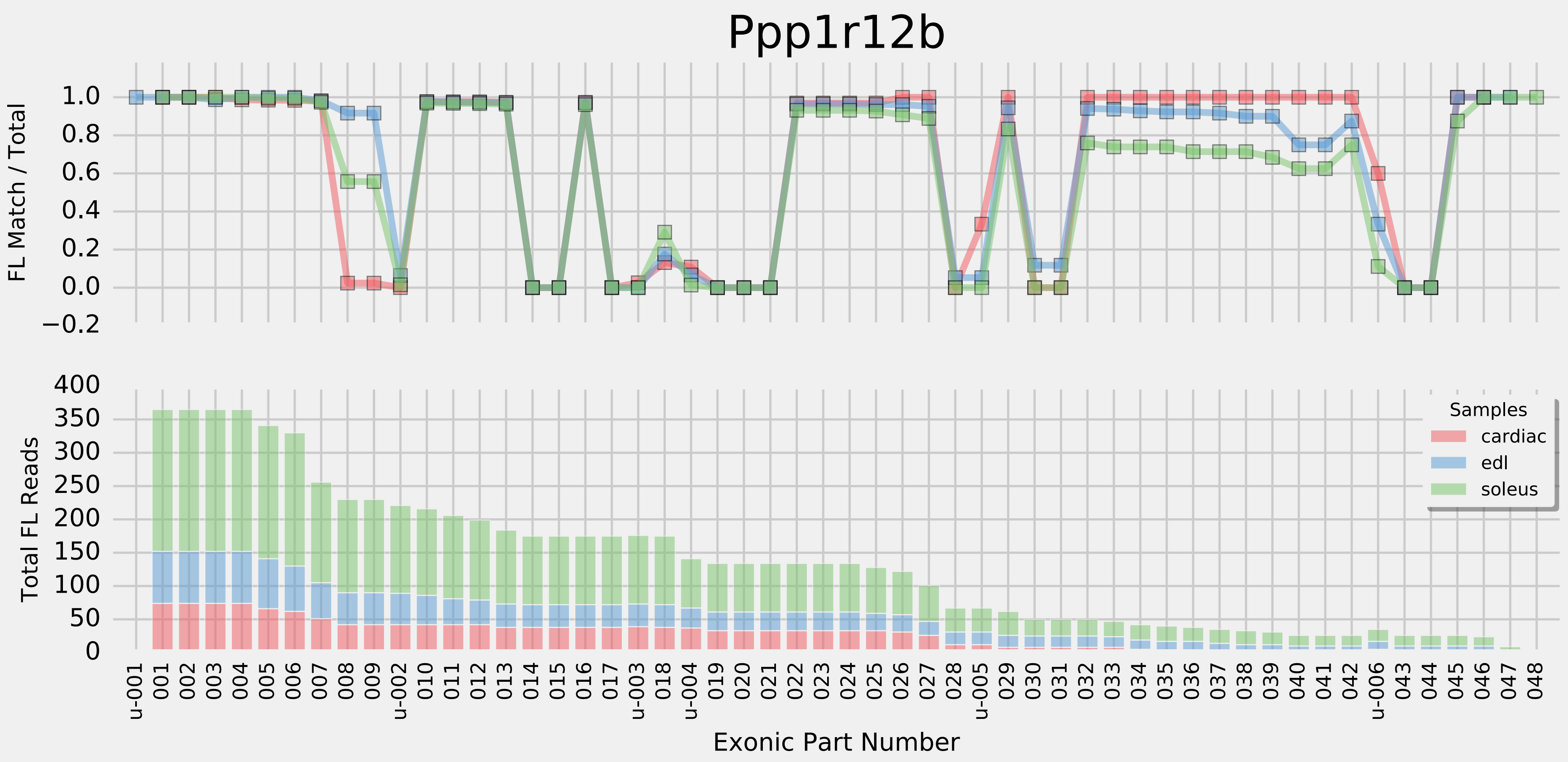

Ppp1r12b
FL Match / Total
1.0
0.8
0.6
0.4
0.2
0.0
−0.2
Total FL Reads
400
350
300
250
200
150
100
50
0
Samples
cardiac
edl
soleus
u-001 001 002 003 004 005 006 007 008 009 u-002 010 011 012 013 014 015 016 017 u-003 018 u-004 019 020 021 022 023 024 025 026 027 028 u-005 029 030 031 032 033 034 035 036 037 038 039 040 041 042 u-006 043 044 045 046 047 048
Exonic Part Number

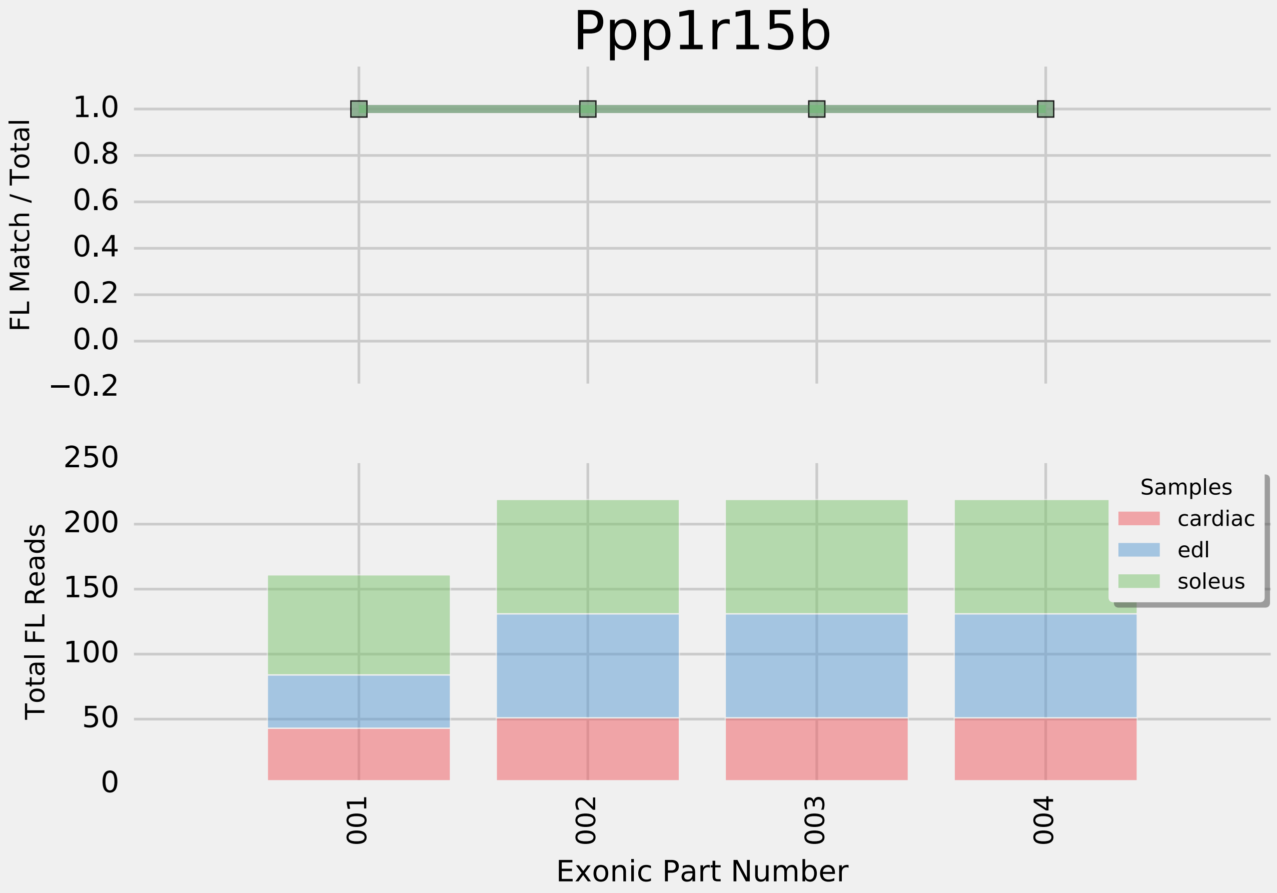

Ppp1r15b
FL Match / Total
1.0
0.8
0.6
0.4
0.2
0.0
−0.2
Total FL Reads
250
200
150
100
50
0
Samples
cardiac
edl
soleus
001
002
003
004
Exonic Part Number

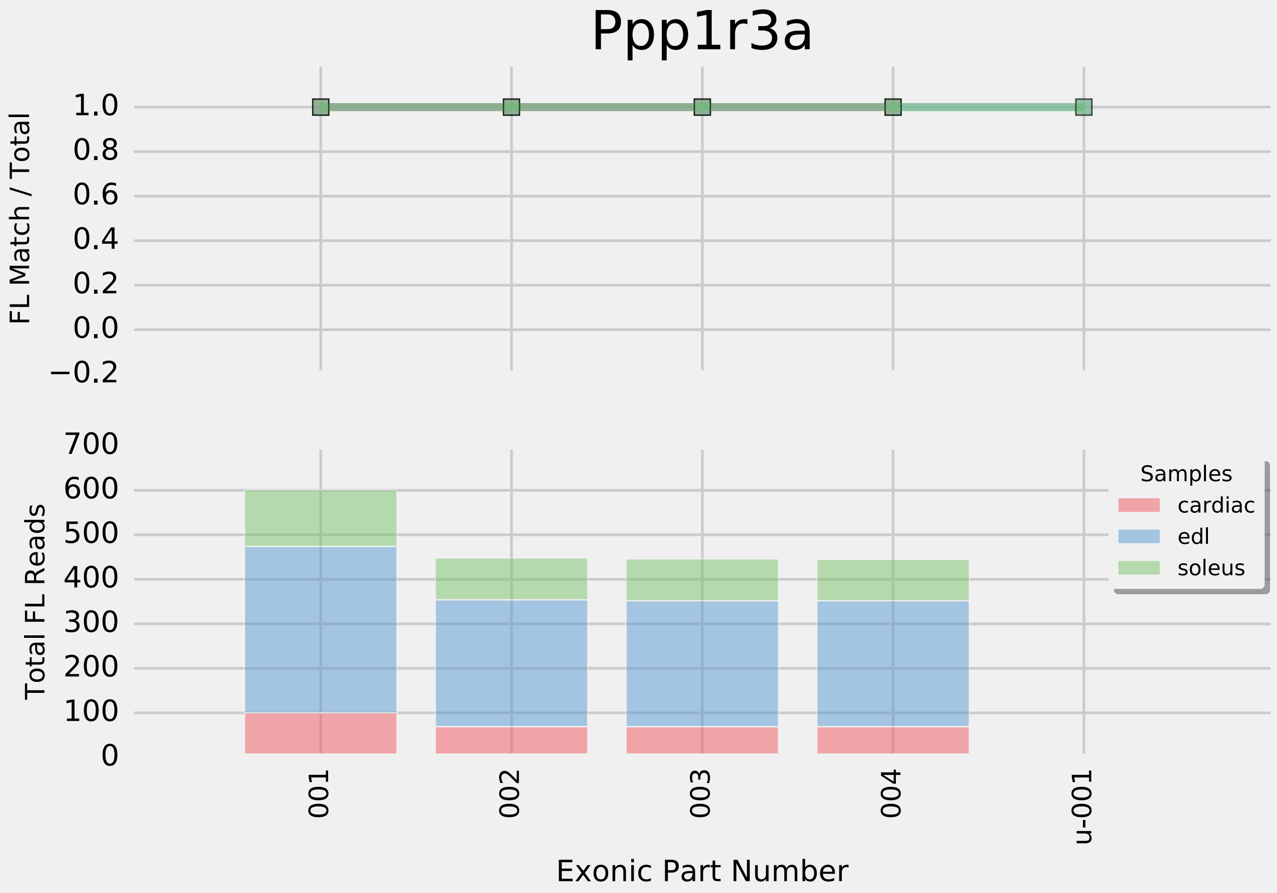
Ppp1r3a
FL Match / Total
1.0
0.8
0.6
0.4
0.2
0.0
−0.2
Total FL Reads
700
600
500
400
300
200
100
0
Samples
cardiac
edl
soleus
001
002
003
004
u-001
Exonic Part Number

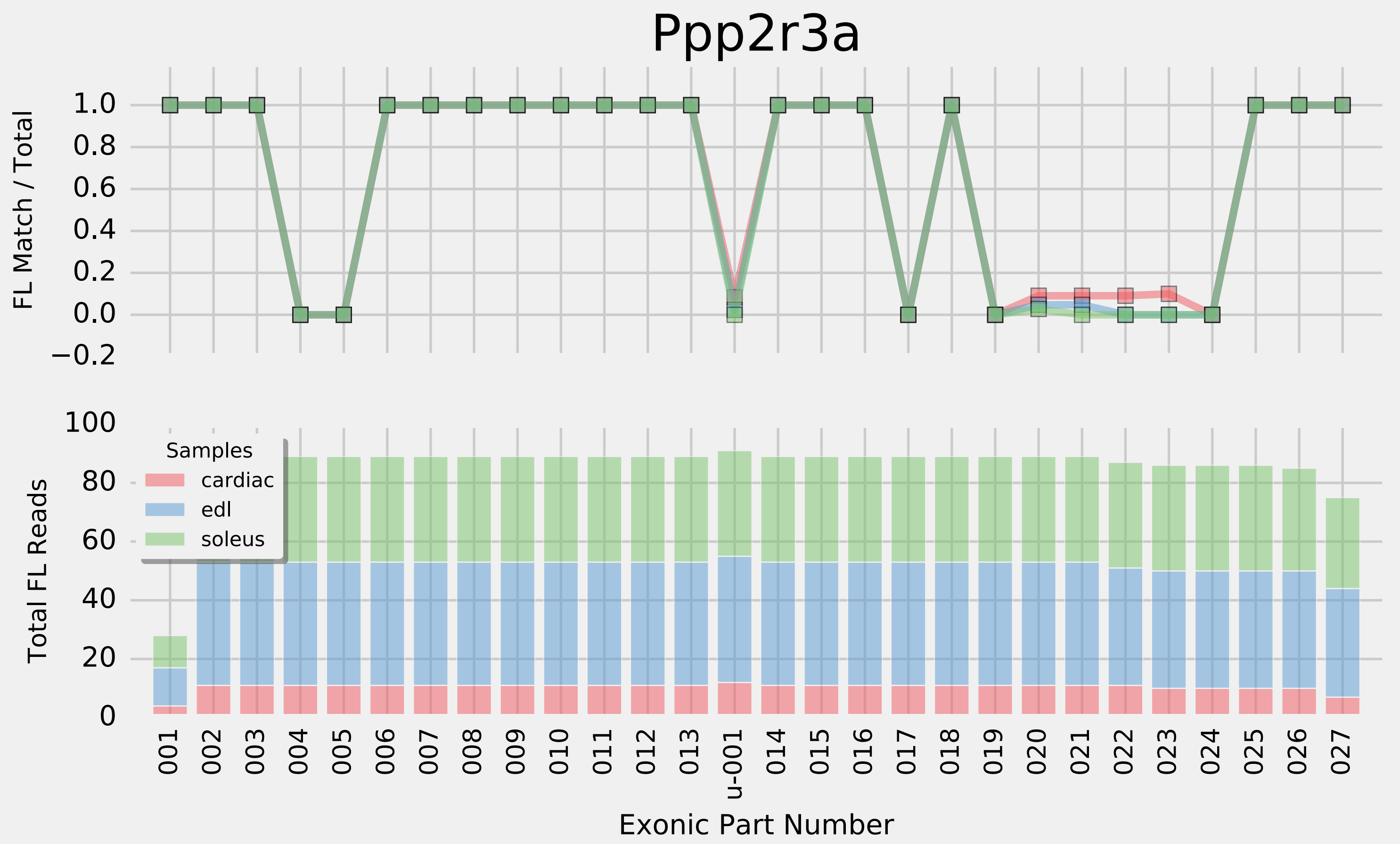

Ppp2r3a
FL Match / Total
1.0
0.8
0.6
0.4
0.2
0.0
−0.2
Total FL Reads
100
80
60
40
20
0
Samples
cardiac
edl
soleus
001
002
003
004
005
006
007
008
009
010
011
012
013
u-001
014
015
016
017
018
019
020
021
022
023
024
025
026
027
Exonic Part Number

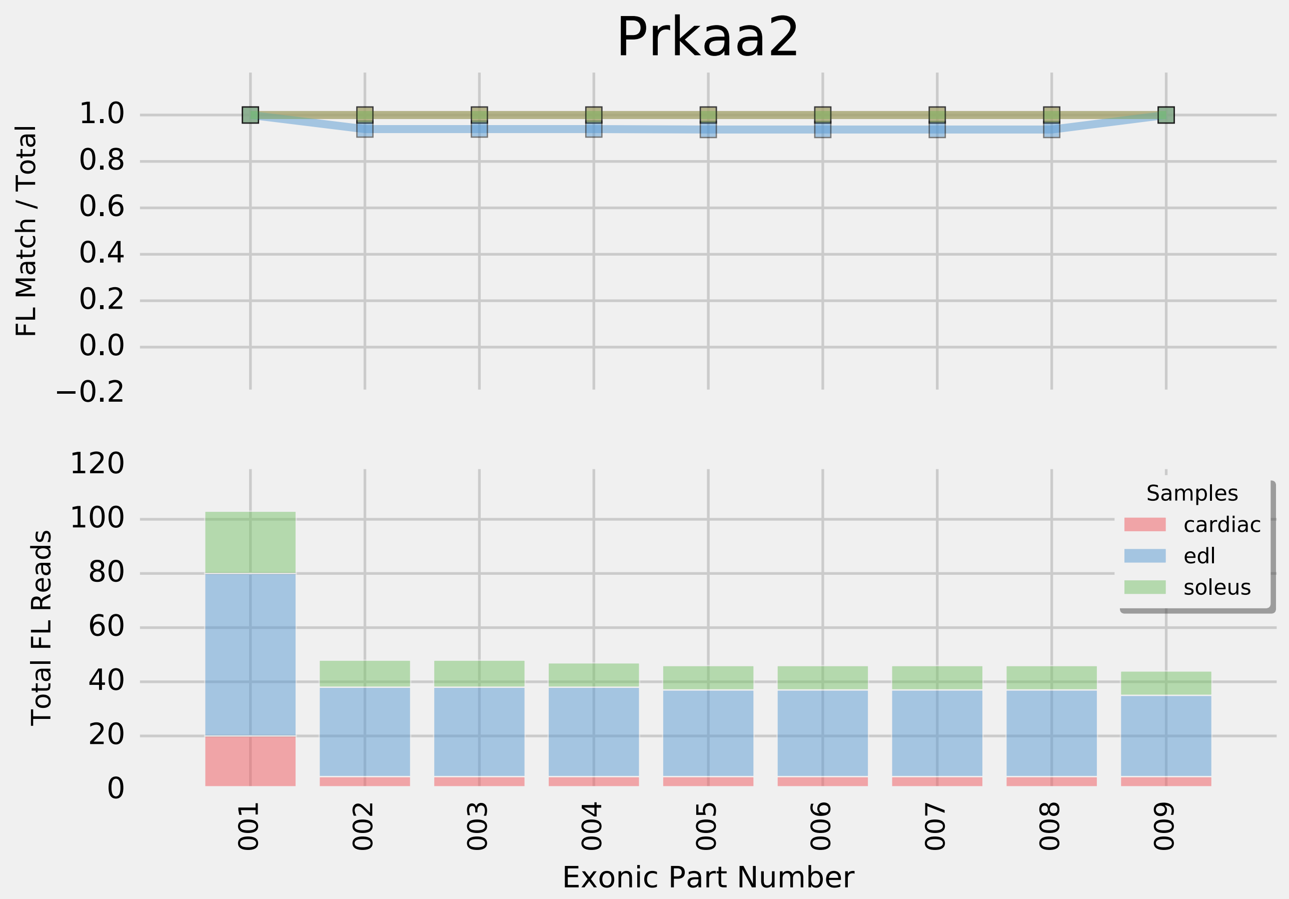

Prrkaa2
FL Match / Total
1.0
0.8
0.6
0.4
0.2
0.0
−0.2
Total FL Reads
120
100
80
60
40
20
0
Samples
cardiac
edl
soleus
001
002
003
004
005
006
007
008
009
Exonic Part Number

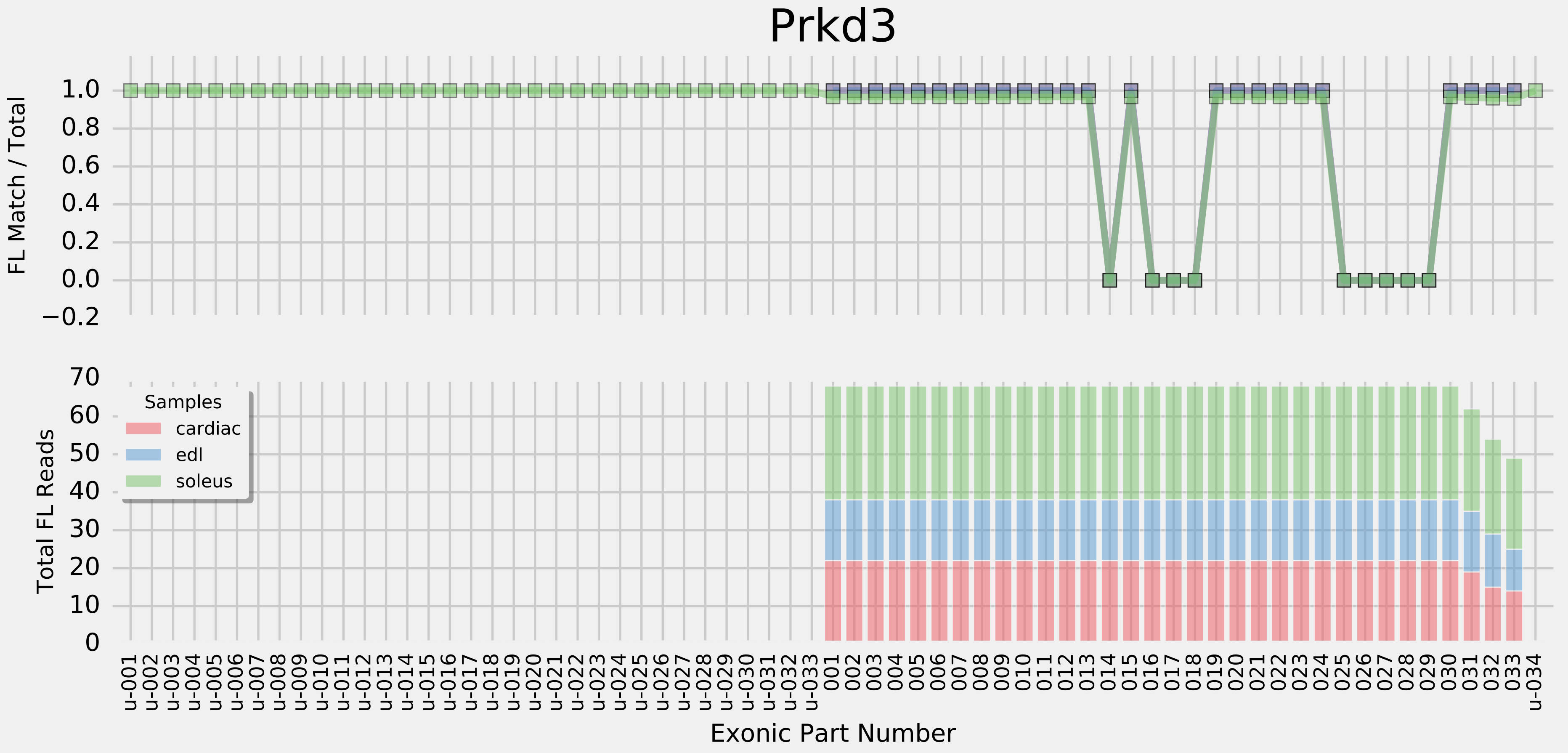

Prkd3
FL Match / Total
1.0
0.8
0.6
0.4
0.2
0.0
−0.2
Total FL Reads
70
60
50
40
30
20
10
0
Samples
cardiac
edl
soleus
u-001 u-002 u-003 u-004 u-005 u-006 u-007 u-008 u-009 u-010 u-011 u-012 u-013 u-014 u-015 u-016 u-017 u-018 u-019 u-020 u-021 u-022 u-023 u-024 u-025 u-026 u-027 u-028 u-029 u-030 u-031 u-032 u-033 001 002 003 004 005 006 007 008 009 010 011 012 013 014 015 016 017 018 019 020 021 022 023 024 025 026 027 028 029 030 031 032 033 u-034
Exonic Part Number

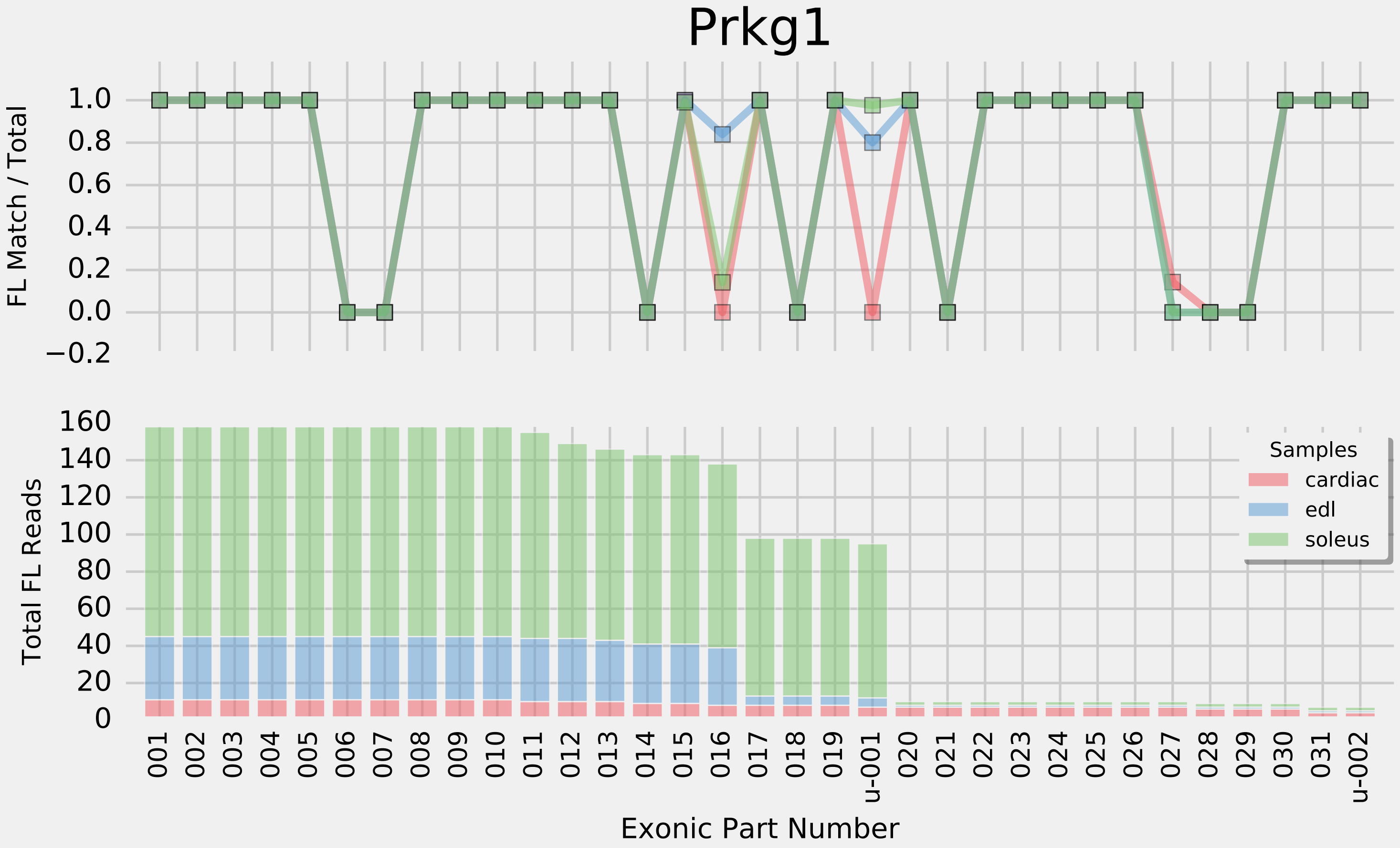

Prkg1
FL Match / Total
1.0
0.8
0.6
0.4
0.2
0.0
−0.2
Total FL Reads
160
140
120
100
80
60
40
20
0
Samples
cardiac
edl
soleus
001
002
003
004
005
006
007
008
009
010
011
012
013
014
015
016
017
018
019
u-001
020
021
022
023
024
025
026
027
028
029
030
031
u-002
Exonic Part Number

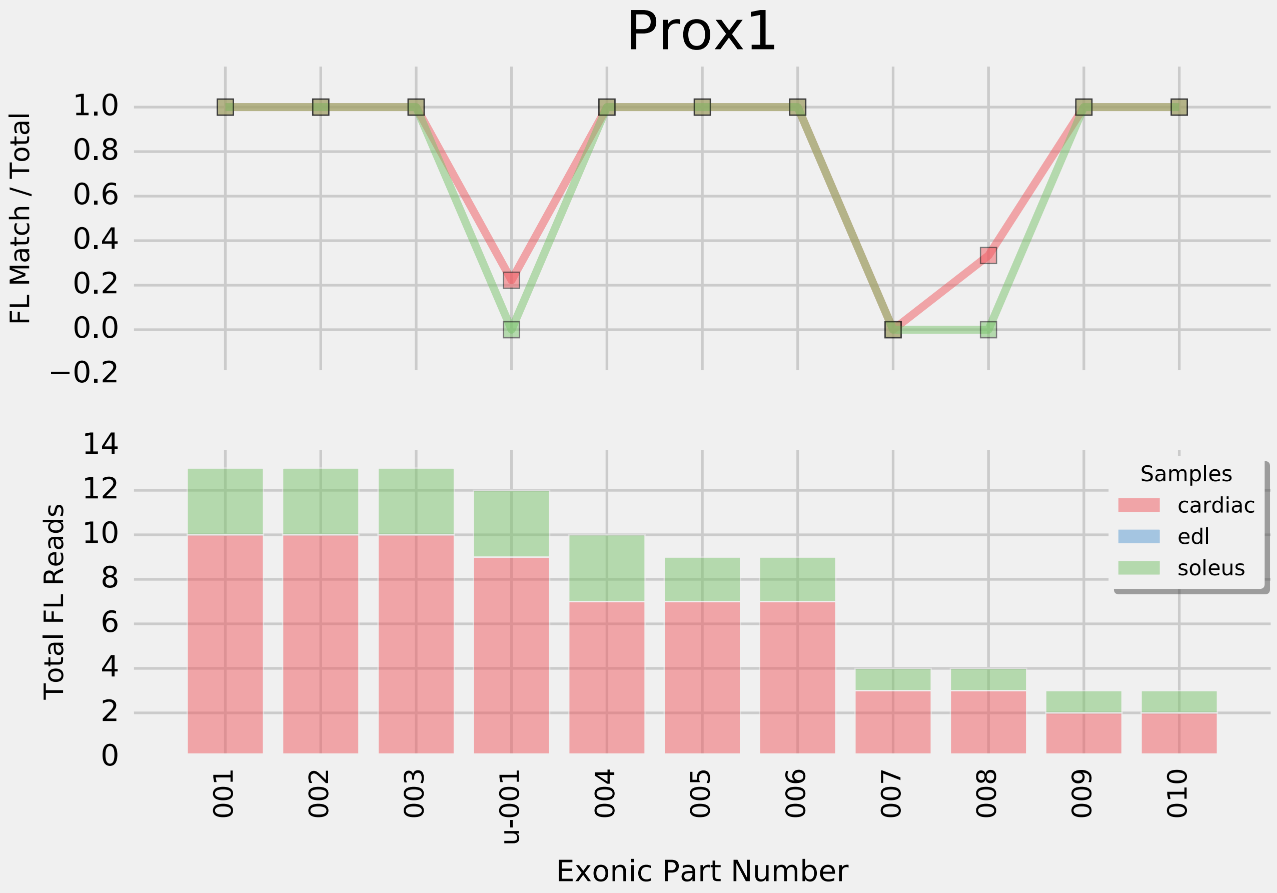

Prox1
FL Match / Total
1.0
0.8
0.6
0.4
0.2
0.0
−0.2
Total FL Reads
14
12
10
8
6
4
2
0
Samples
cardiac
edl
soleus
001
002
003
u-001
004
005
006
007
008
009
010
Exonic Part Number

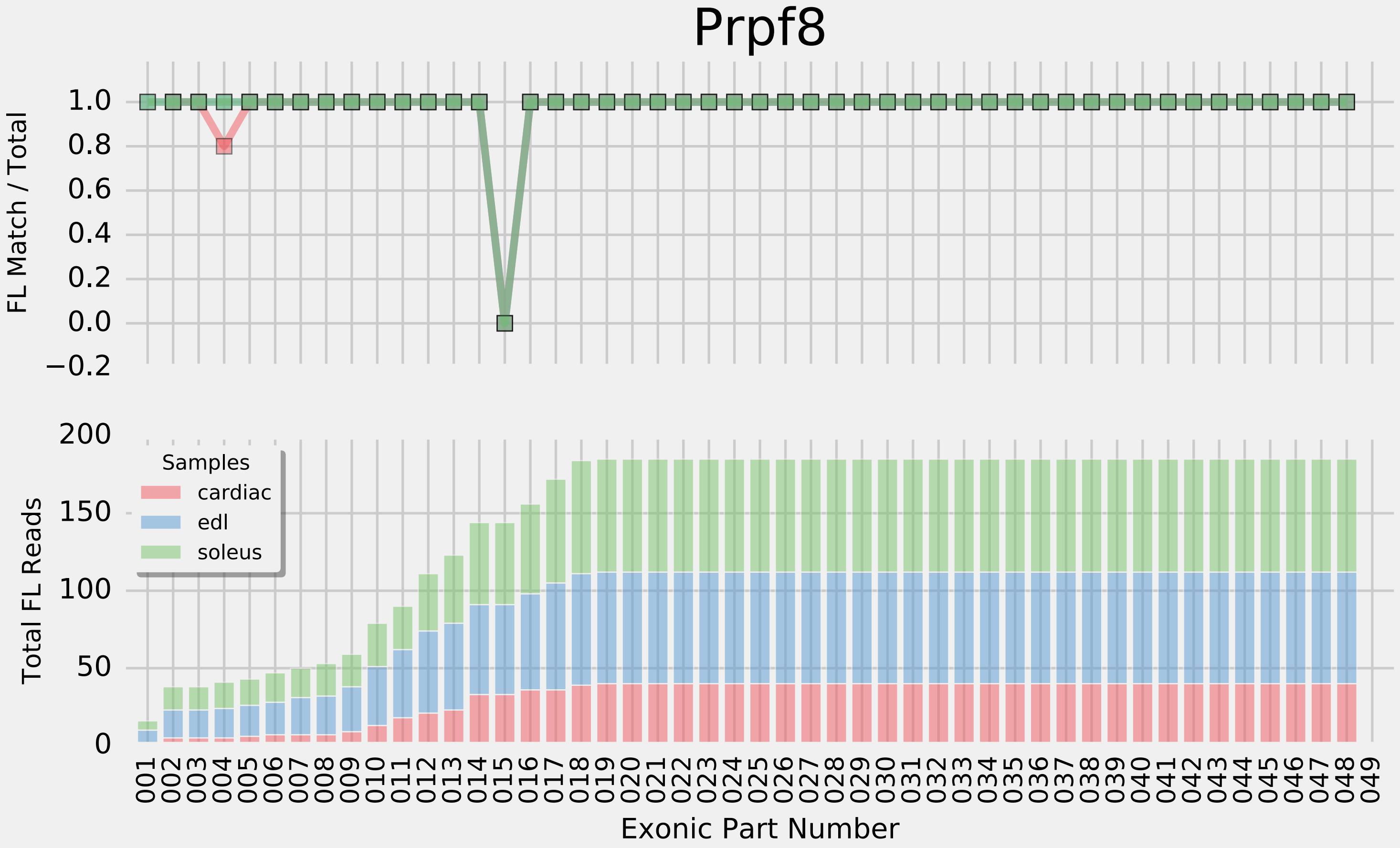
Prpf8
FL Match / Total
1.0
0.8
0.6
0.4
0.2
0.0
−0.2
Total FL Reads
200
150
100
50
0
Samples
cardiac
edl
soleus
001 002 003 004 005 006 007 008 009 010 011 012 013 014 015 016 017 018 019 020 021 022 023 024 025 026 027 028 029 030 031 032 033 034 035 036 037 038 039 040 041 042 043 044 045 046 047 048 049
Exonic Part Number

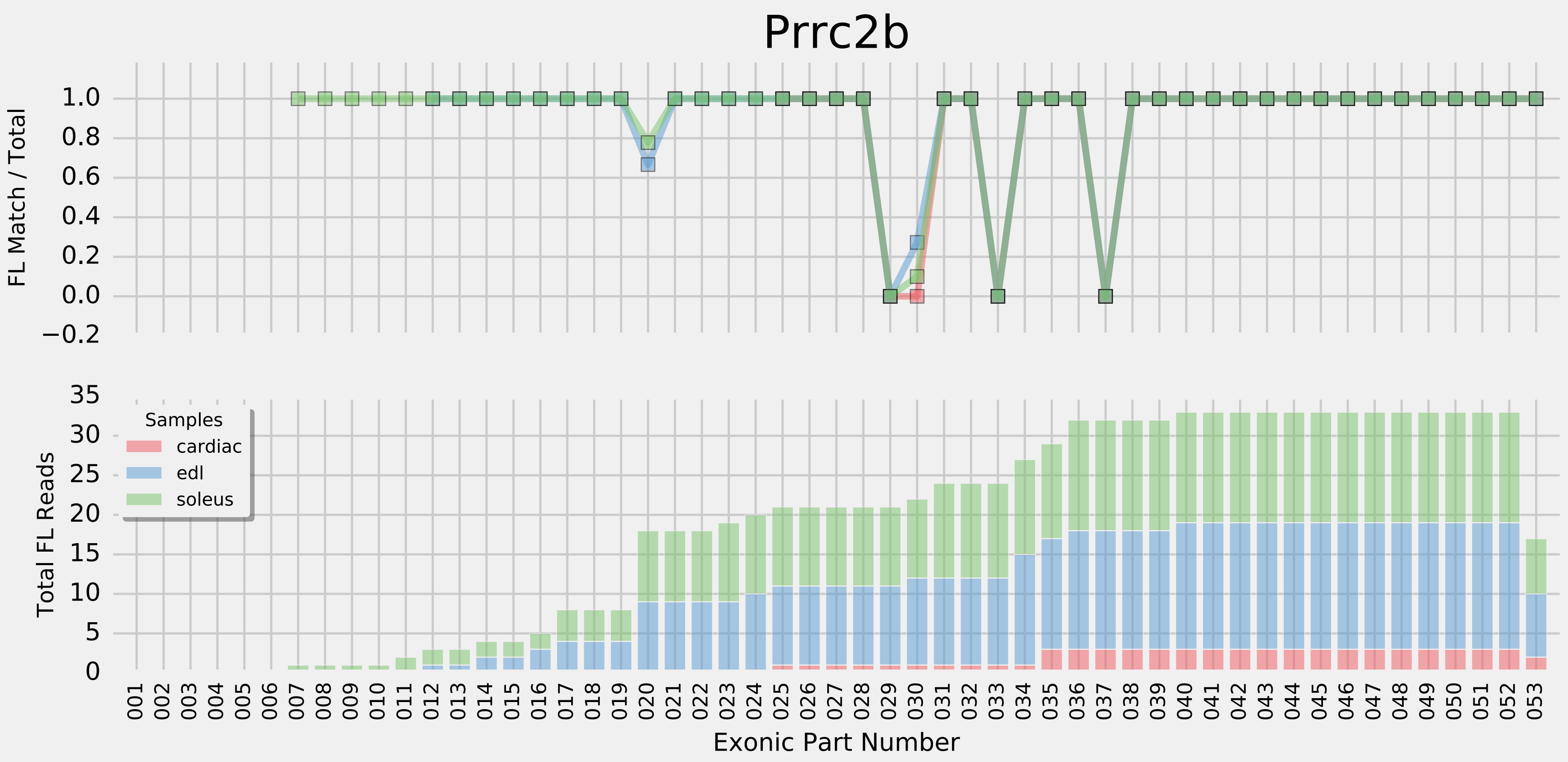

Prrc2b
FL Match / Total
1.0
0.8
0.6
0.4
0.2
0.0
−0.2
Samples
cardiac
edl
soleus
Total FL Reads
35
30
25
20
15
10
5
0
001 002 003 004 005 006 007 008 009 010 011 012 013 014 015 016 017 018 019 020 021 022 023 024 025 026 027 028 029 030 031 032 033 034 035 036 037 038 039 040 041 042 043 044 045 046 047 048 049 050 051 052 053
Exonic Part Number

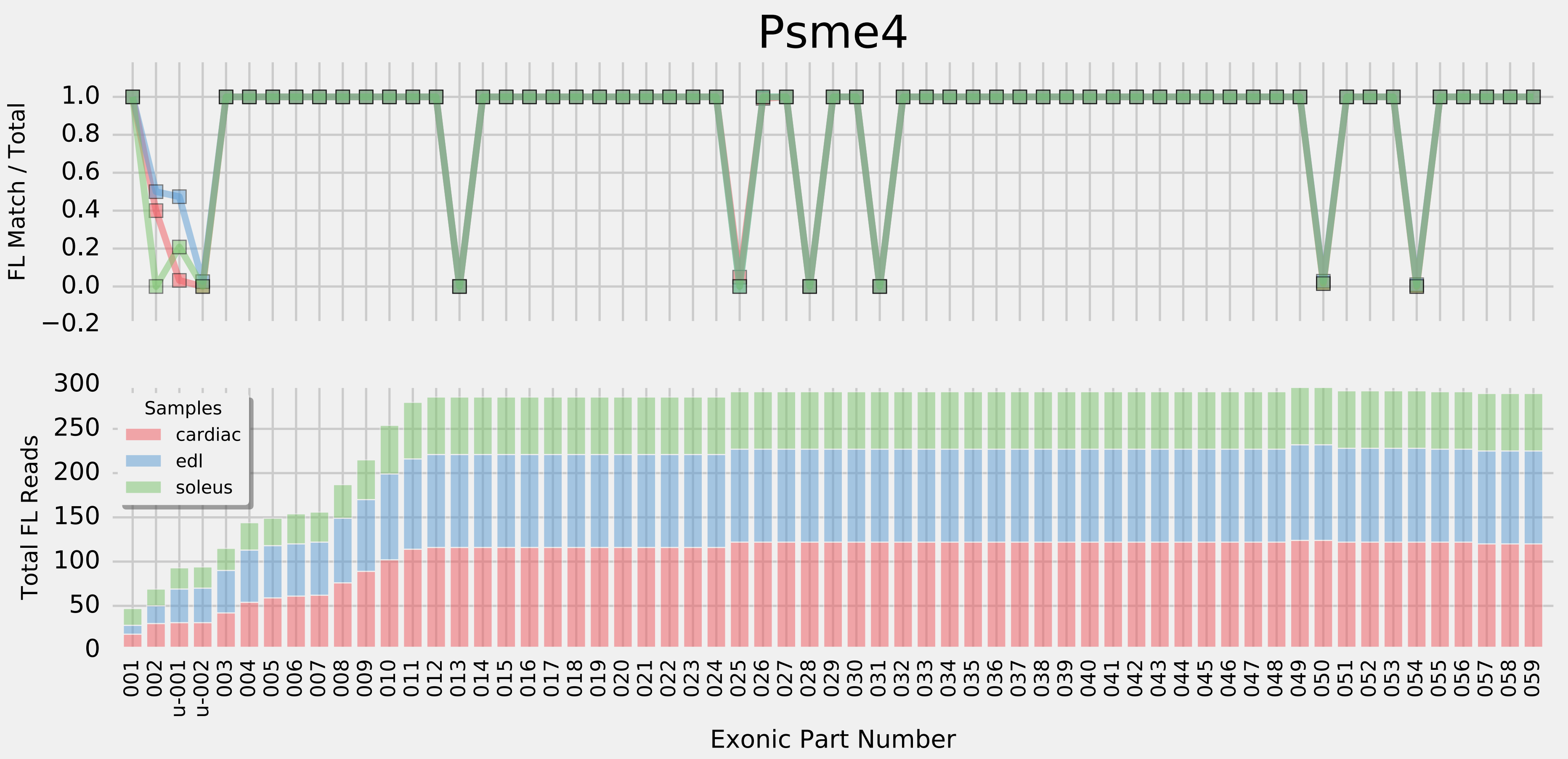

Psme4
FL Match / Total
1.0
0.8
0.6
0.4
0.2
0.0
−0.2
Total FL Reads
300
250
200
150
100
50
0
Samples
cardiac
edl
soleus
001
002
u-001
u-002
003
004
005
006
007
008
009
010
011
012
013
014
015
016
017
018
019
020
021
022
023
024
025
026
027
028
029
030
031
032
033
034
035
036
037
038
039
040
041
042
043
044
045
046
047
048
049
050
051
052
053
054
055
056
057
058
059
Exonic Part Number

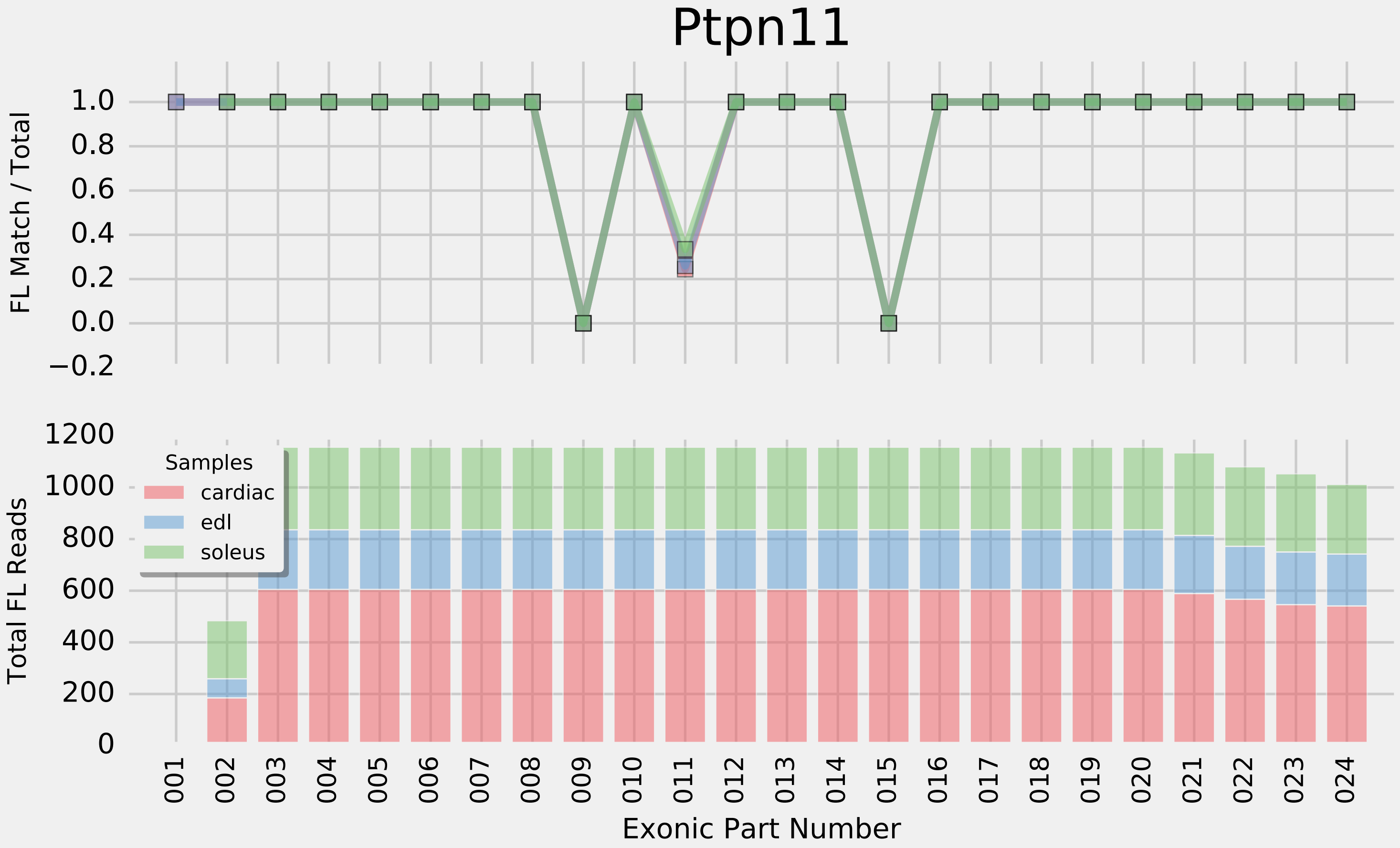

Ptpn11
FL Match / Total
1.0
0.8
0.6
0.4
0.2
0.0
−0.2
Total FL Reads
1200
1000
800
600
400
200
0
Samples
cardiac
edl
soleus
001
002
003
004
005
006
007
008
009
010
011
012
013
014
015
016
017
018
019
020
021
022
023
024
Exonic Part Number

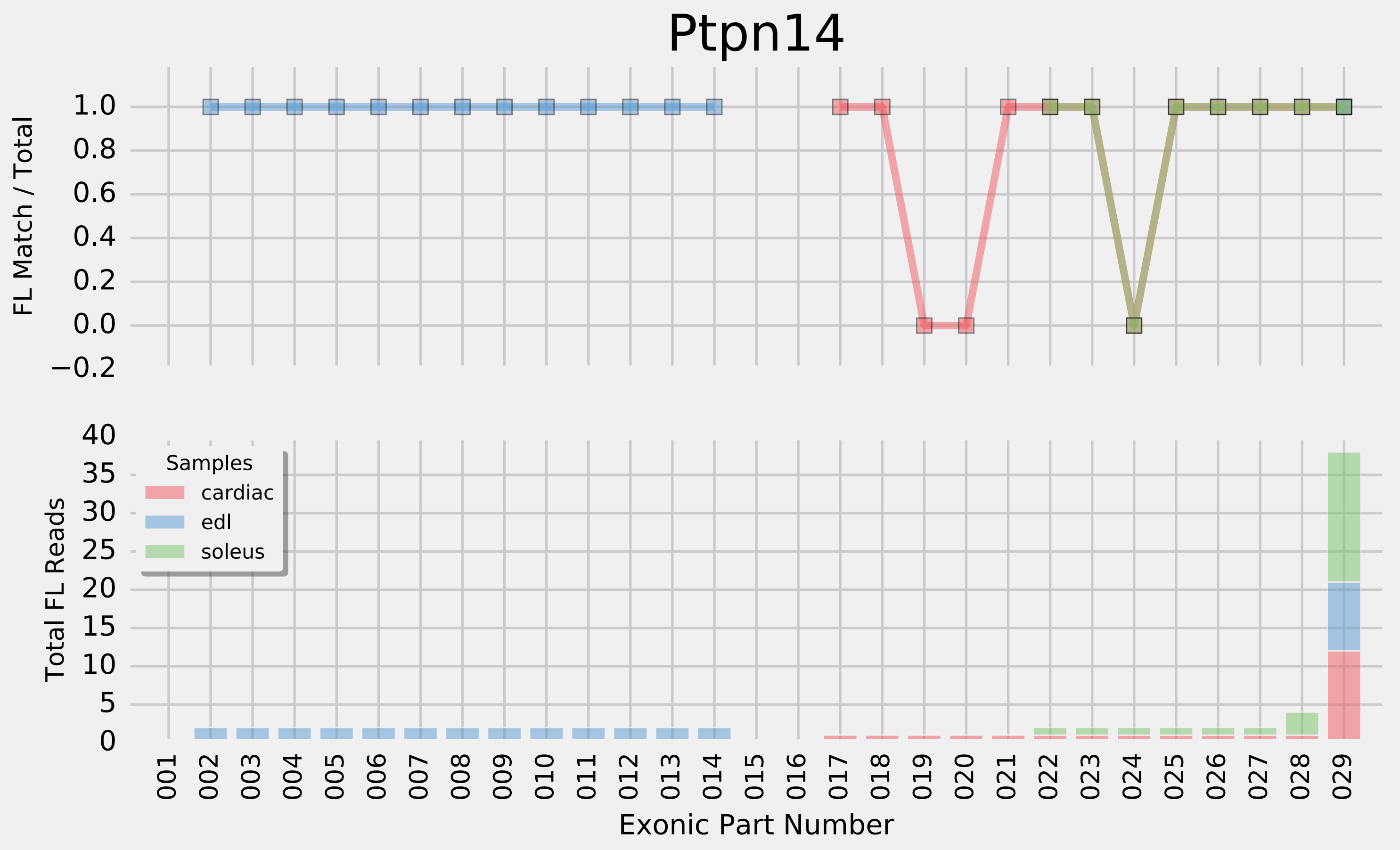

Ptpn14
FL Match / Total
1.0
0.8
0.6
0.4
0.2
0.0
−0.2
Total FL Reads
40
35
30
25
20
15
10
5
0
Samples
cardiac
edl
soleus
001
002
003
004
005
006
007
008
009
010
011
012
013
014
015
016
017
018
019
020
021
022
023
024
025
026
027
028
029
Exonic Part Number

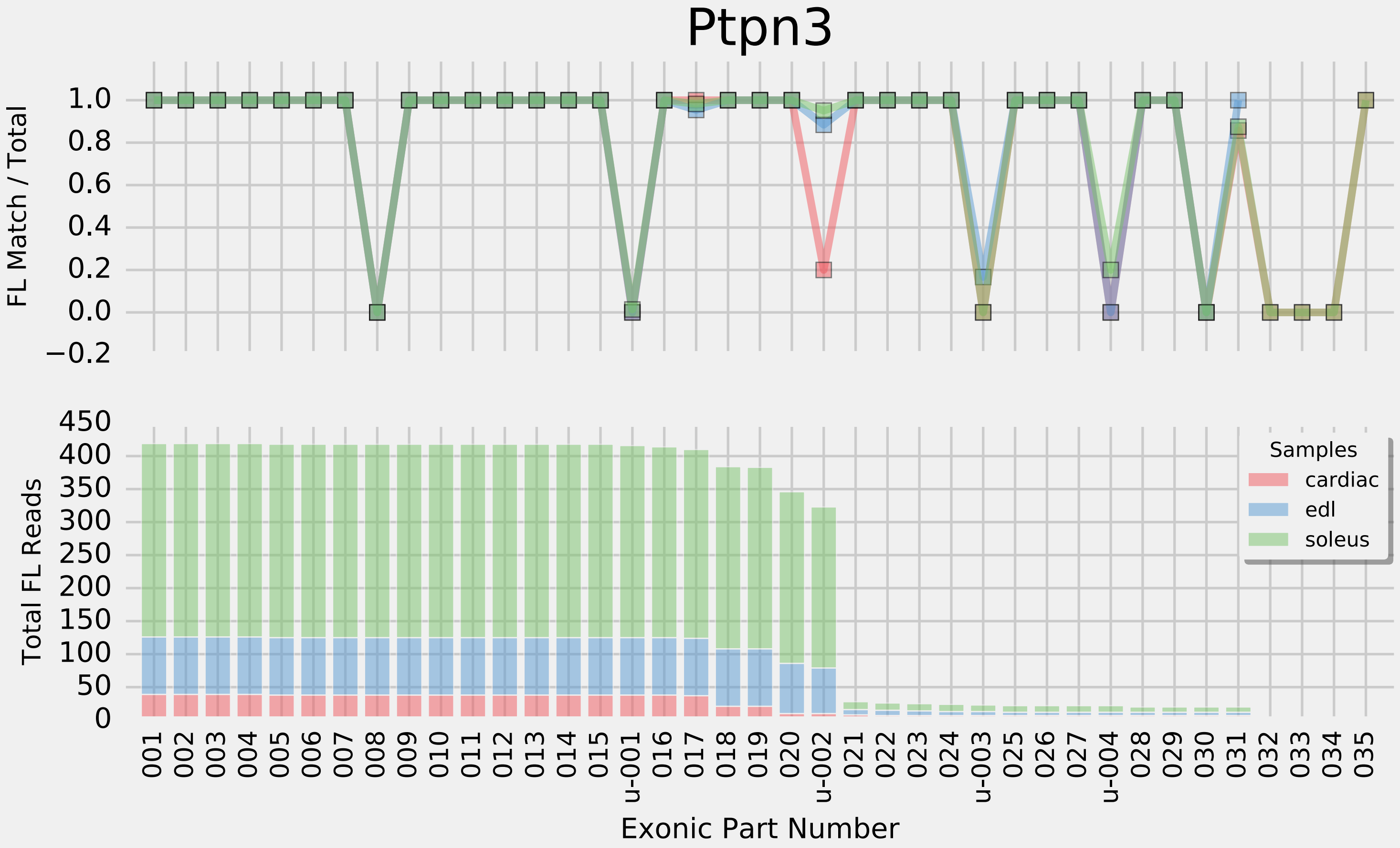

Ptpn3
FL Match / Total
1.0
0.8
0.6
0.4
0.2
0.0
−0.2
Total FL Reads
450
400
350
300
250
200
150
100
50
0
Samples
cardiac
edl
soleus
001 002 003 004 005 006 007 008 009 010 011 012 013 014 015 u-001 016 017 018 019 020 u-002 021 022 023 024 u-003 025 026 027 u-004 028 029 030 031 032 033 034 035
Exonic Part Number

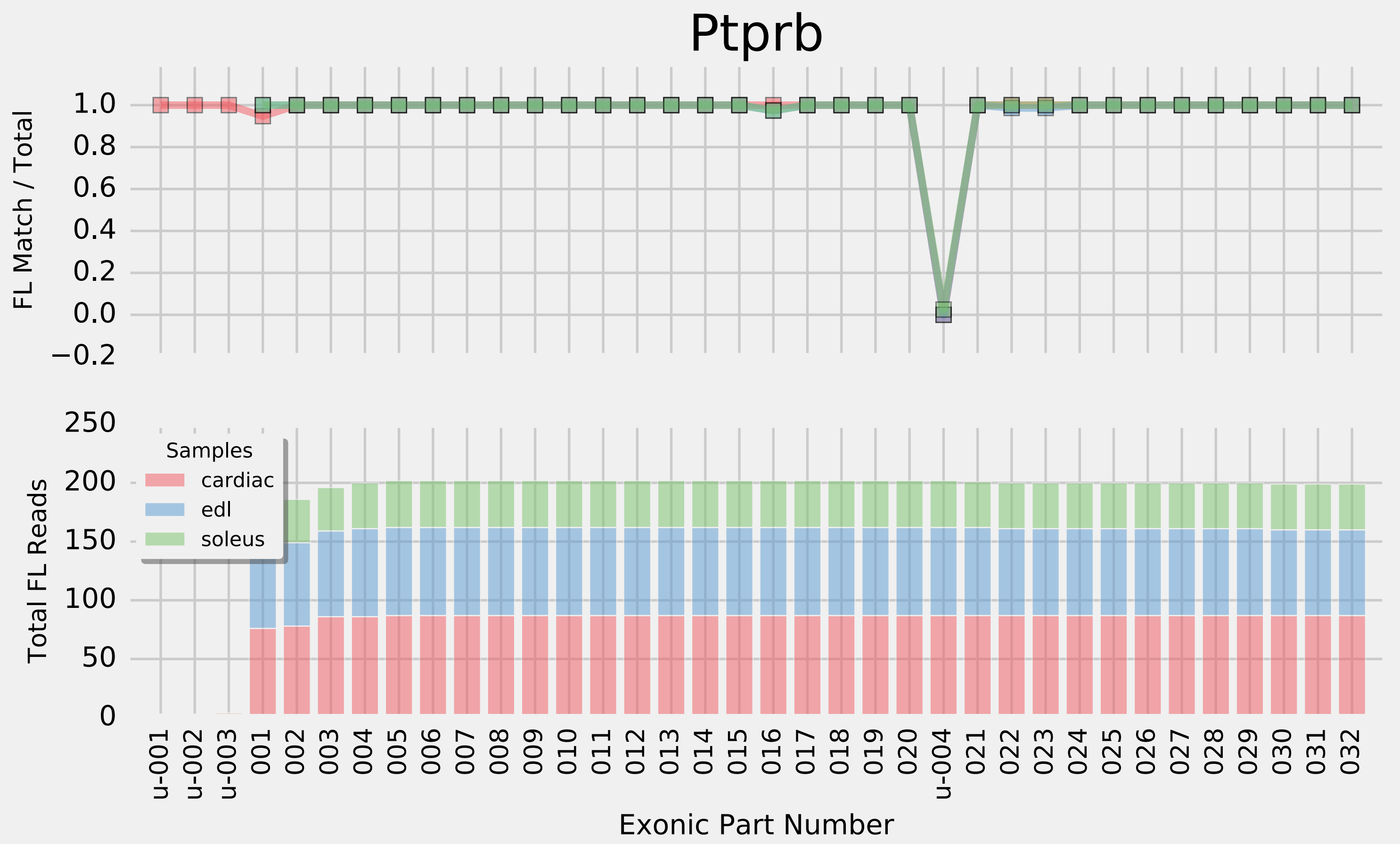

Ptprb
FL Match / Total
1.0
0.8
0.6
0.4
0.2
0.0
−0.2
Total FL Reads
250
200
150
100
50
0
Samples
cardiac
edl
soleus
u-001
u-002
u-003
001
002
003
004
005
006
007
008
009
010
011
012
013
014
015
016
017
018
019
020
u-004
021
022
023
024
025
026
027
028
029
030
031
032
Exonic Part Number

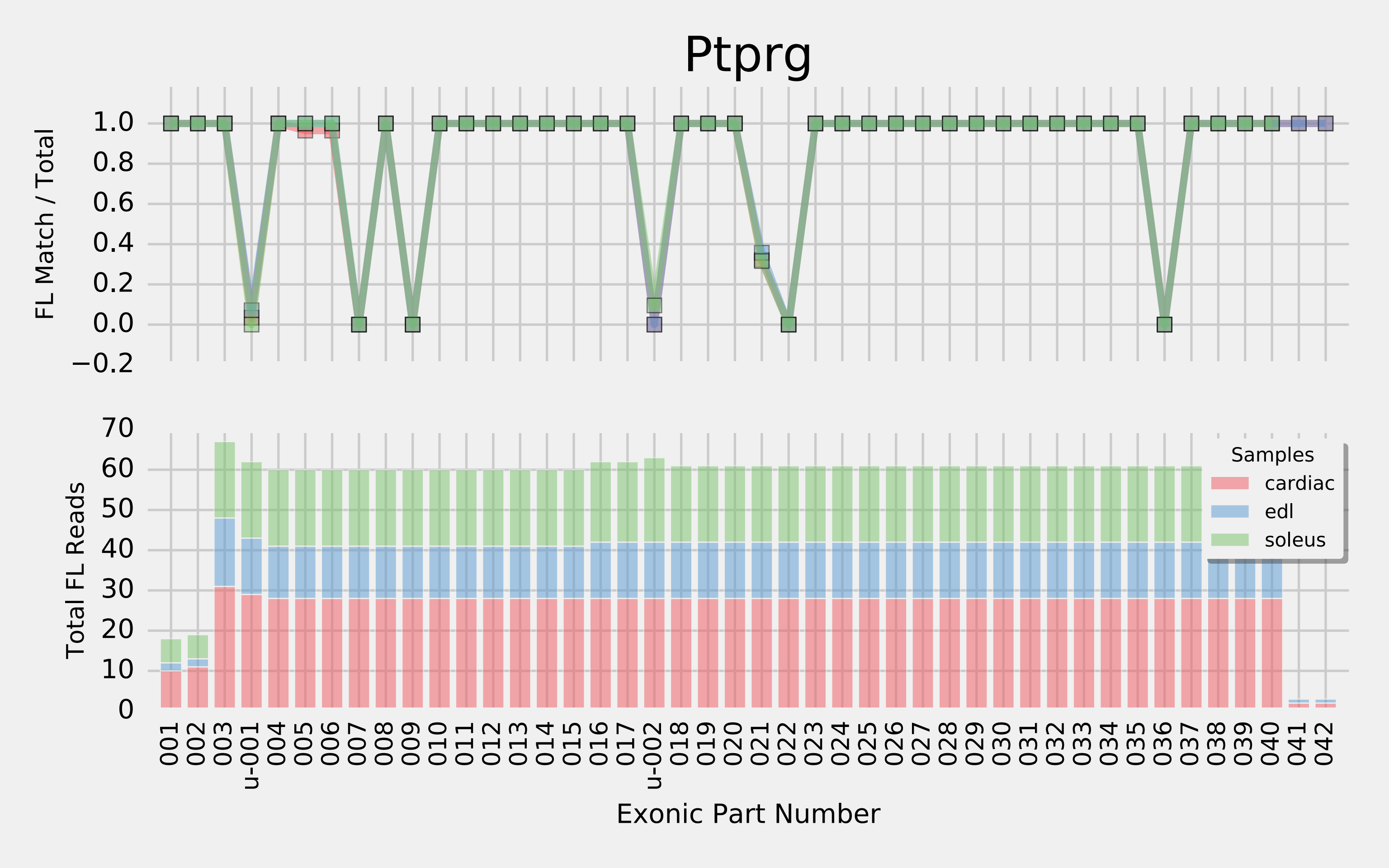
Ptprg
FL Match / Total
1.0
0.8
0.6
0.4
0.2
0.0
−0.2
Total FL Reads
70
60
50
40
30
20
10
0
Samples
cardiac
edl
soleus
001
002
003
u-001
004
005
006
007
008
009
010
011
012
013
014
015
016
017
u-002
018
019
020
021
022
023
024
025
026
027
028
029
030
031
032
033
034
035
036
037
038
039
040
041
042
Exonic Part Number

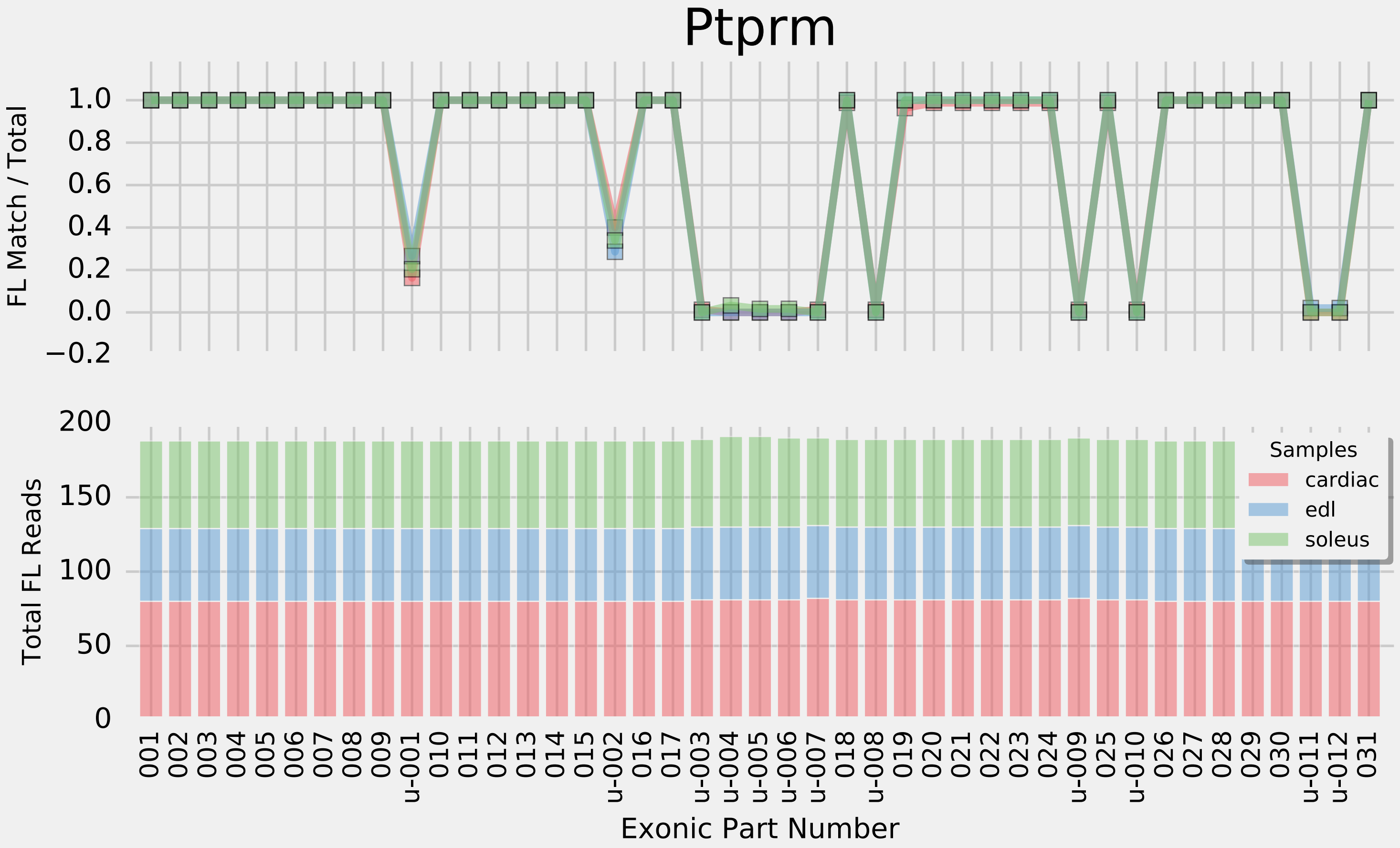

Ptprm
FL Match / Total
1.0
0.8
0.6
0.4
0.2
0.0
−0.2
Total FL Reads
200
150
100
50
0
Samples
cardiac
edl
soleus
001
002
003
004
005
006
007
008
009
u-001
010
011
012
013
014
015
u-002
016
017
u-003
u-004
u-005
u-006
u-007
018
u-008
019
020
021
022
023
024
u-009
025
u-010
026
027
028
029
030
u-011
u-012
031
Exonic Part Number

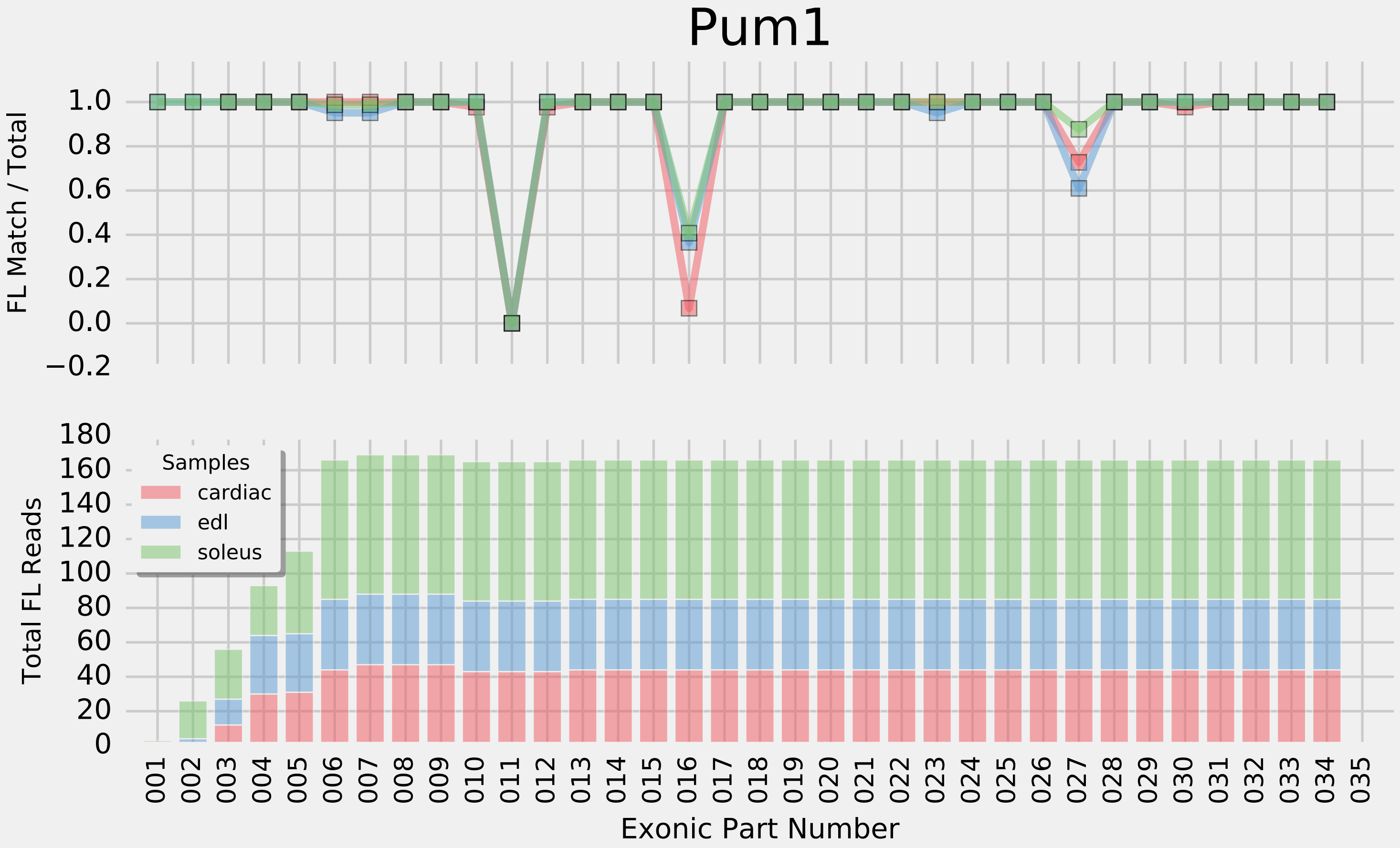

Pum1
FL Match / Total
1.0
0.8
0.6
0.4
0.2
0.0
−0.2
Total FL Reads
180
160
140
120
100
80
60
40
20
0
Samples
cardiac
edl
soleus
001
002
003
004
005
006
007
008
009
010
011
012
013
014
015
016
017
018
019
020
021
022
023
024
025
026
027
028
029
030
031
032
033
034
035
Exonic Part Number

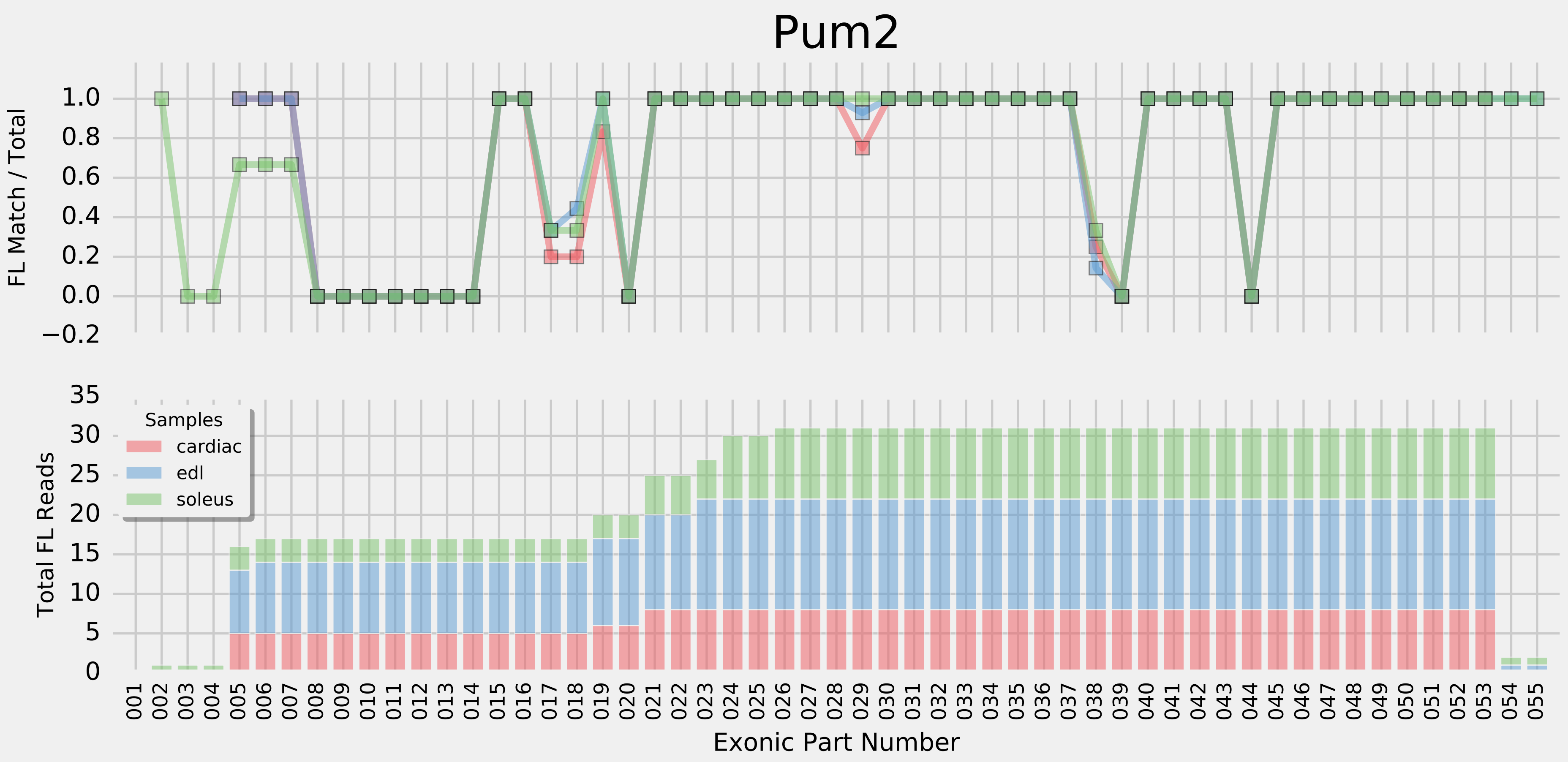

Pum2
FL Match / Total
1.0
0.8
0.6
0.4
0.2
0.0
−0.2
Total FL Reads
35
30
25
20
15
10
5
0
Samples
cardiac
edl
soleus
001 002 003 004 005 006 007 008 009 010 011 012 013 014 015 016 017 018 019 020 021 022 023 024 025 026 027 028 029 030 031 032 033 034 035 036 037 038 039 040 041 042 043 044 045 046 047 048 049 050 051 052 053 054 055
Exonic Part Number

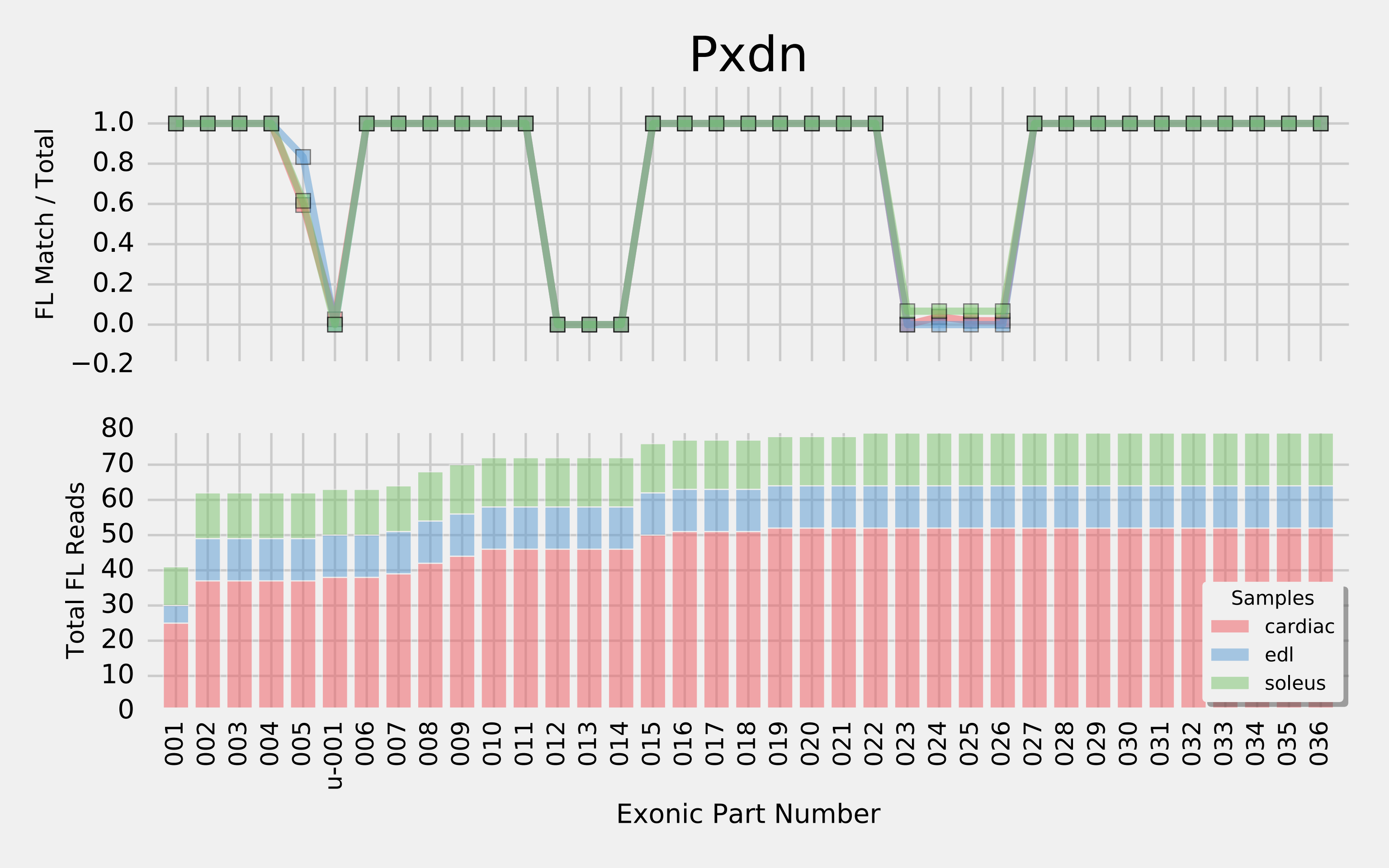

Pxdn
FL Match / Total
1.0
0.8
0.6
0.4
0.2
0.0
−0.2
Total FL Reads
80
70
60
50
40
30
20
10
0
Samples
cardiac
edl
soleus
001
002
003
004
005
u-001
006
007
008
009
010
011
012
013
014
015
016
017
018
019
020
021
022
023
024
025
026
027
028
029
030
031
032
033
034
035
036
Exonic Part Number

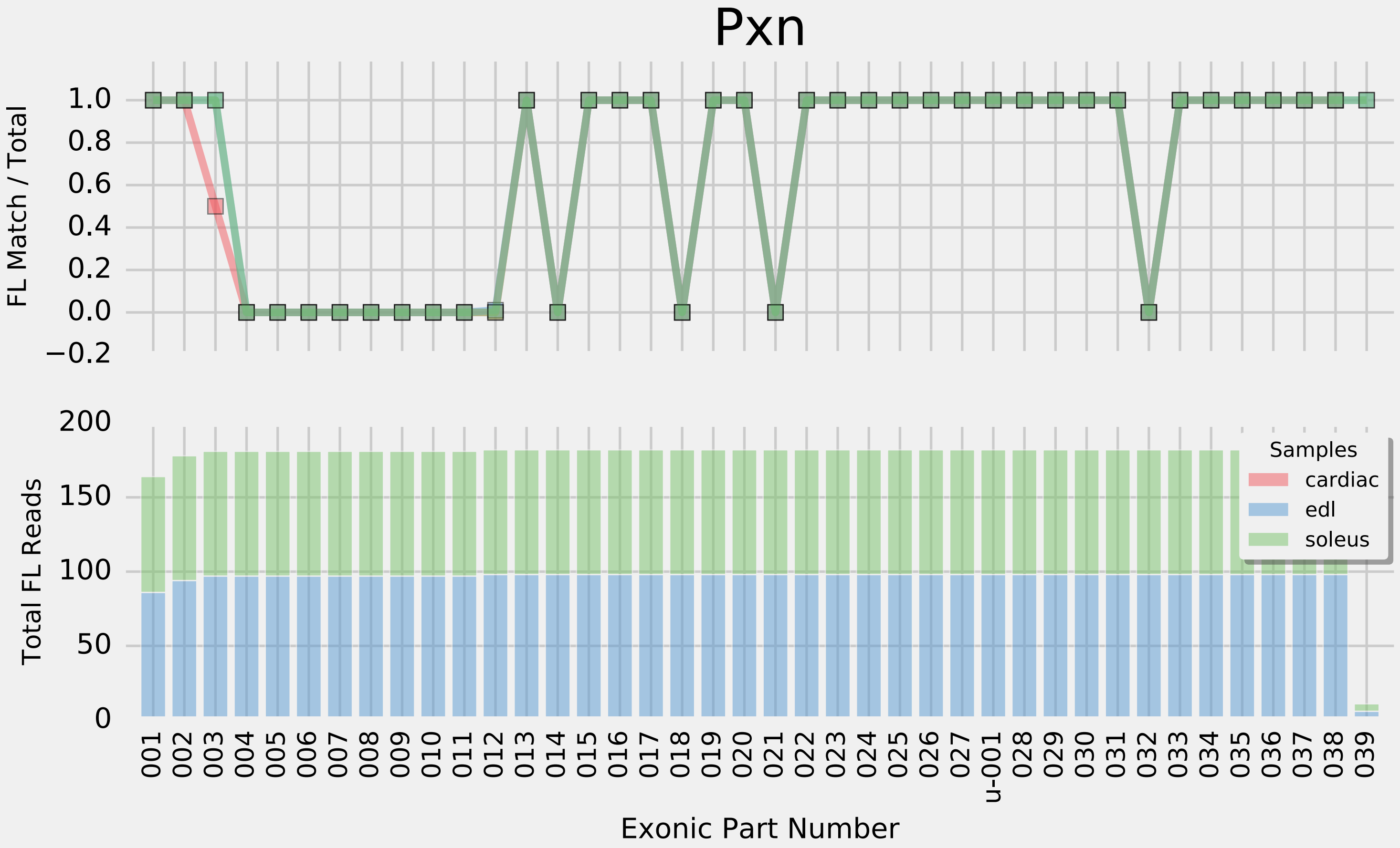

Pxn
FL Match / Total
1.0
0.8
0.6
0.4
0.2
0.0
−0.2
Total FL Reads
200
150
100
50
0
Samples
cardiac
edl
soleus
001 002 003 004 005 006 007 008 009 010 011 012 013 014 015 016 017 018 019 020 021 022 023 024 025 026 027 u-001 028 029 030 031 032 033 034 035 036 037 038 039
Exonic Part Number

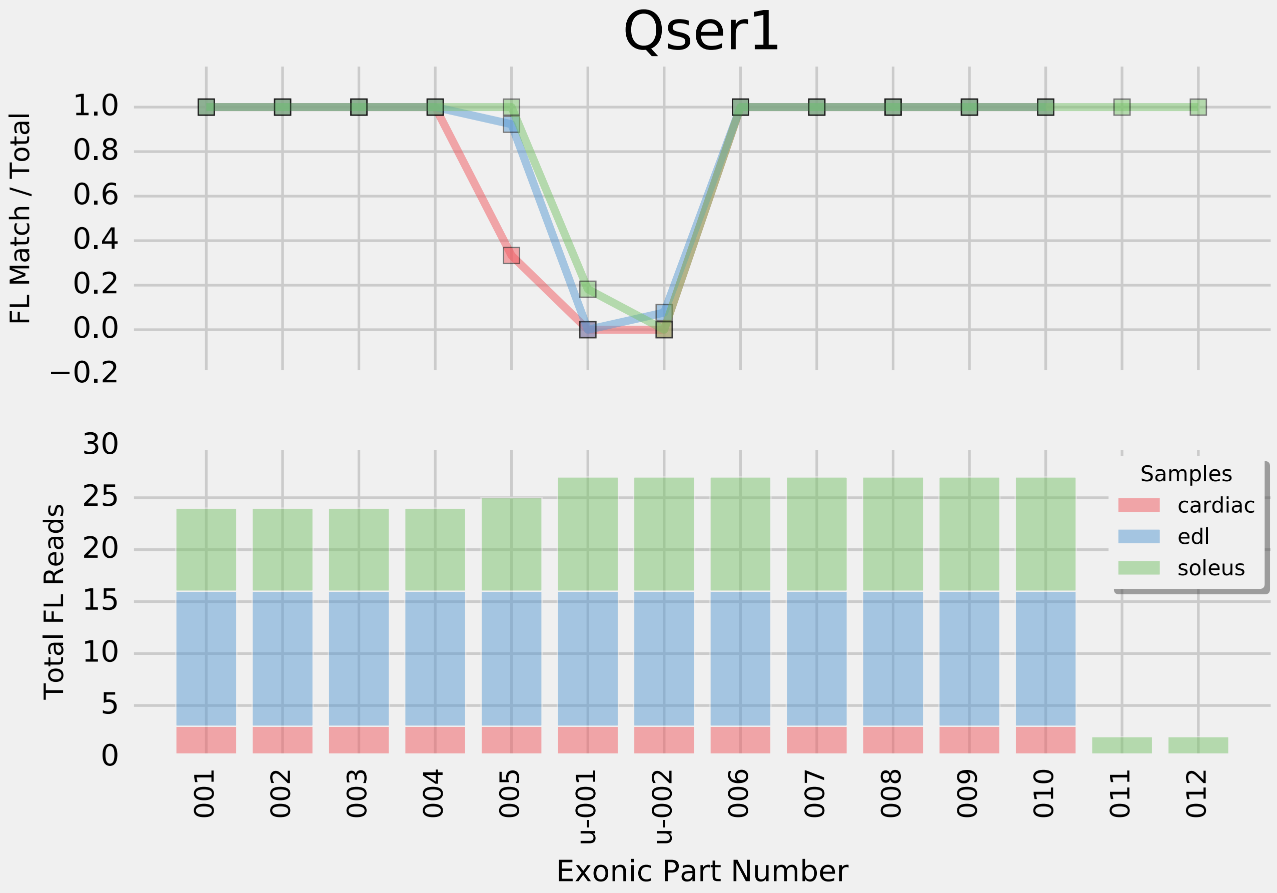

Qser1
FL Match / Total
1.0
0.8
0.6
0.4
0.2
0.0
−0.2
Total FL Reads
30
25
20
15
10
5
0
Samples
cardiac
edl
soleus
001
002
003
004
005
u-001
u-002
006
007
008
009
010
011
012
Exonic Part Number

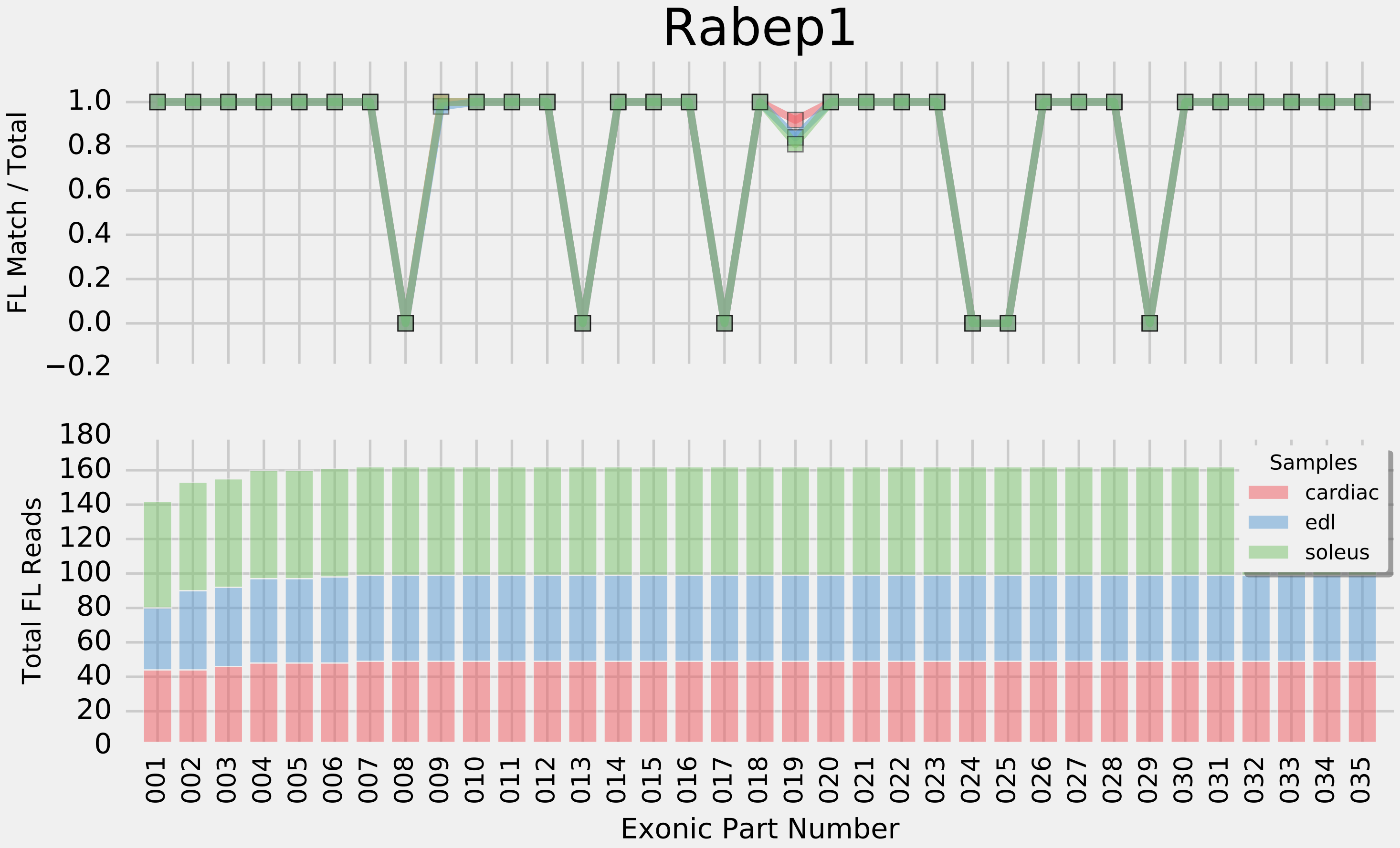

Rabep1
FL Match / Total
1.0
0.8
0.6
0.4
0.2
0.0
−0.2
Total FL Reads
180
160
140
120
100
80
60
40
20
0
Samples
cardiac
edl
soleus
001
002
003
004
005
006
007
008
009
010
011
012
013
014
015
016
017
018
019
020
021
022
023
024
025
026
027
028
029
030
031
032
033
034
035
Exonic Part Number

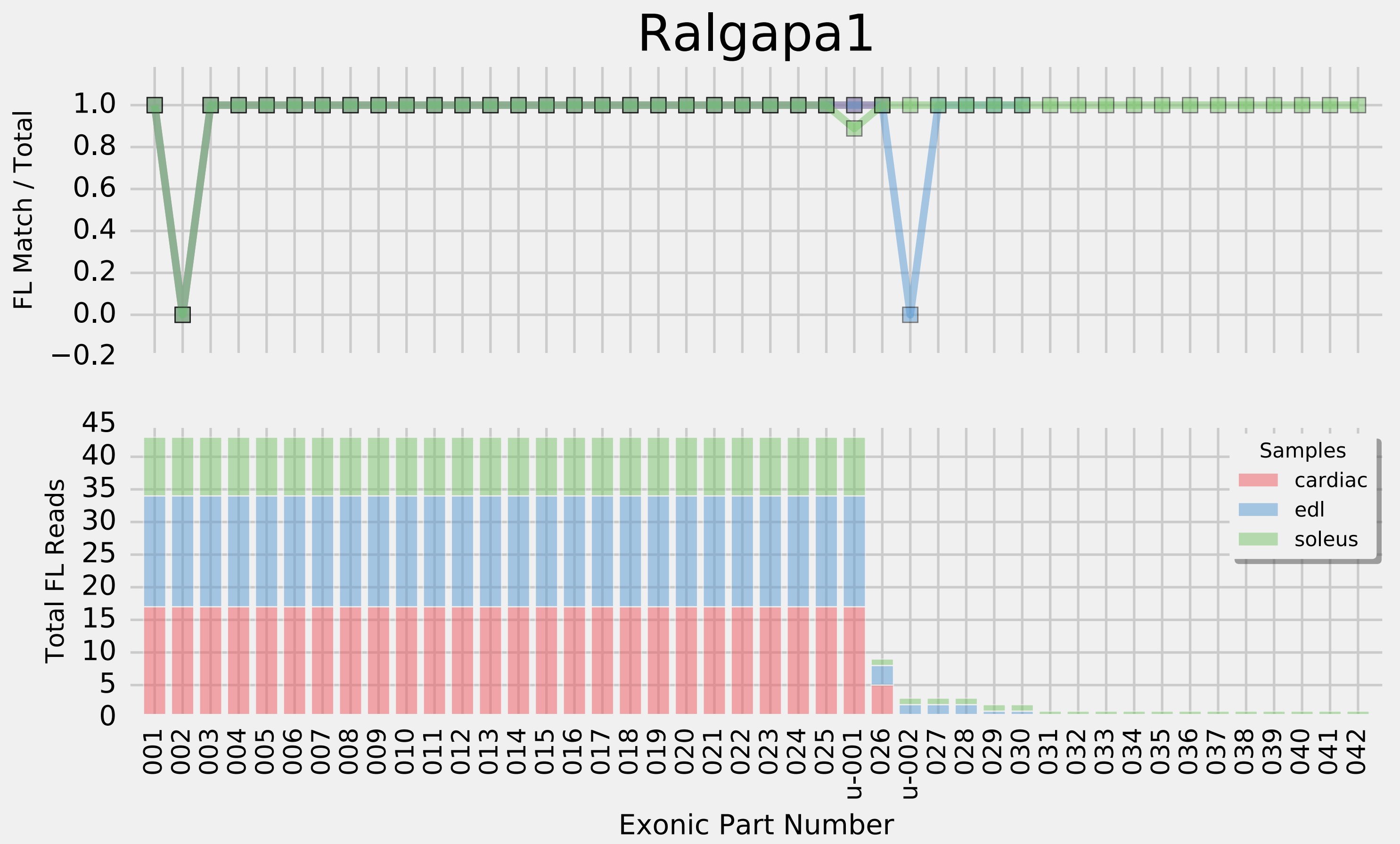
Ralgapa1
FL Match / Total
1.0
0.8
0.6
0.4
0.2
0.0
−0.2
Total FL Reads
45
40
35
30
25
20
15
10
5
0
Samples
cardiac
edl
soleus
001
002
003
004
005
006
007
008
009
010
011
012
013
014
015
016
017
018
019
020
021
022
023
024
025
u-001
026
u-002
027
028
029
030
031
032
033
034
035
036
037
038
039
040
041
042
Exonic Part Number

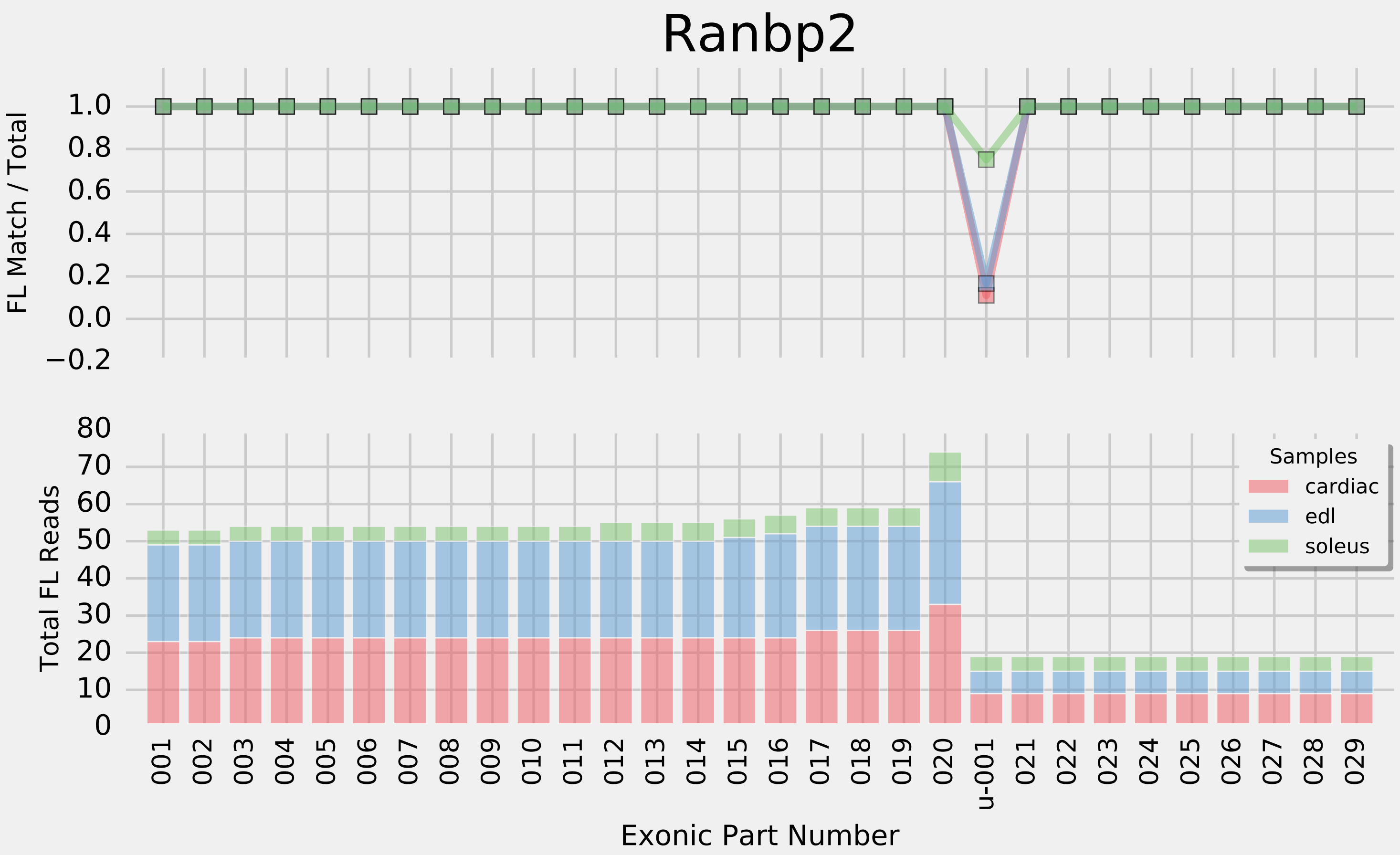
Ranbp2
FL Match / Total
1.0
0.8
0.6
0.4
0.2
0.0
−0.2
Total FL Reads
80
70
60
50
40
30
20
10
0
Samples
cardiac
edl
soleus
001
002
003
004
005
006
007
008
009
010
011
012
013
014
015
016
017
018
019
020
u-001
021
022
023
024
025
026
027
028
029
Exonic Part Number

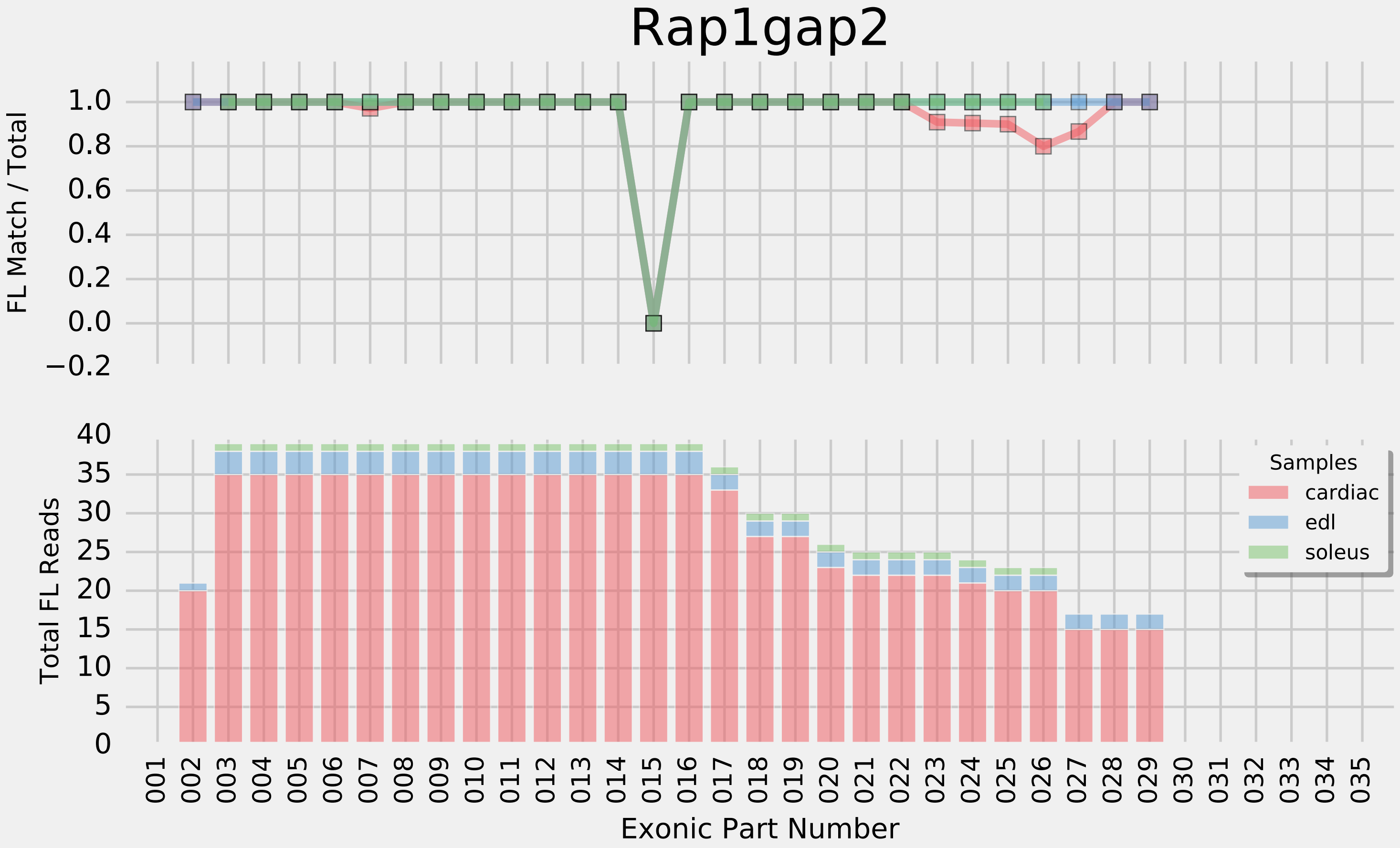

Rap1gap2
FL Match / Total
1.0
0.8
0.6
0.4
0.2
0.0
−0.2
Total FL Reads
40
35
30
25
20
15
10
5
0
Samples
cardiac
edl
soleus
001
002
003
004
005
006
007
008
009
010
011
012
013
014
015
016
017
018
019
020
021
022
023
024
025
026
027
028
029
030
031
032
033
034
035
Exonic Part Number

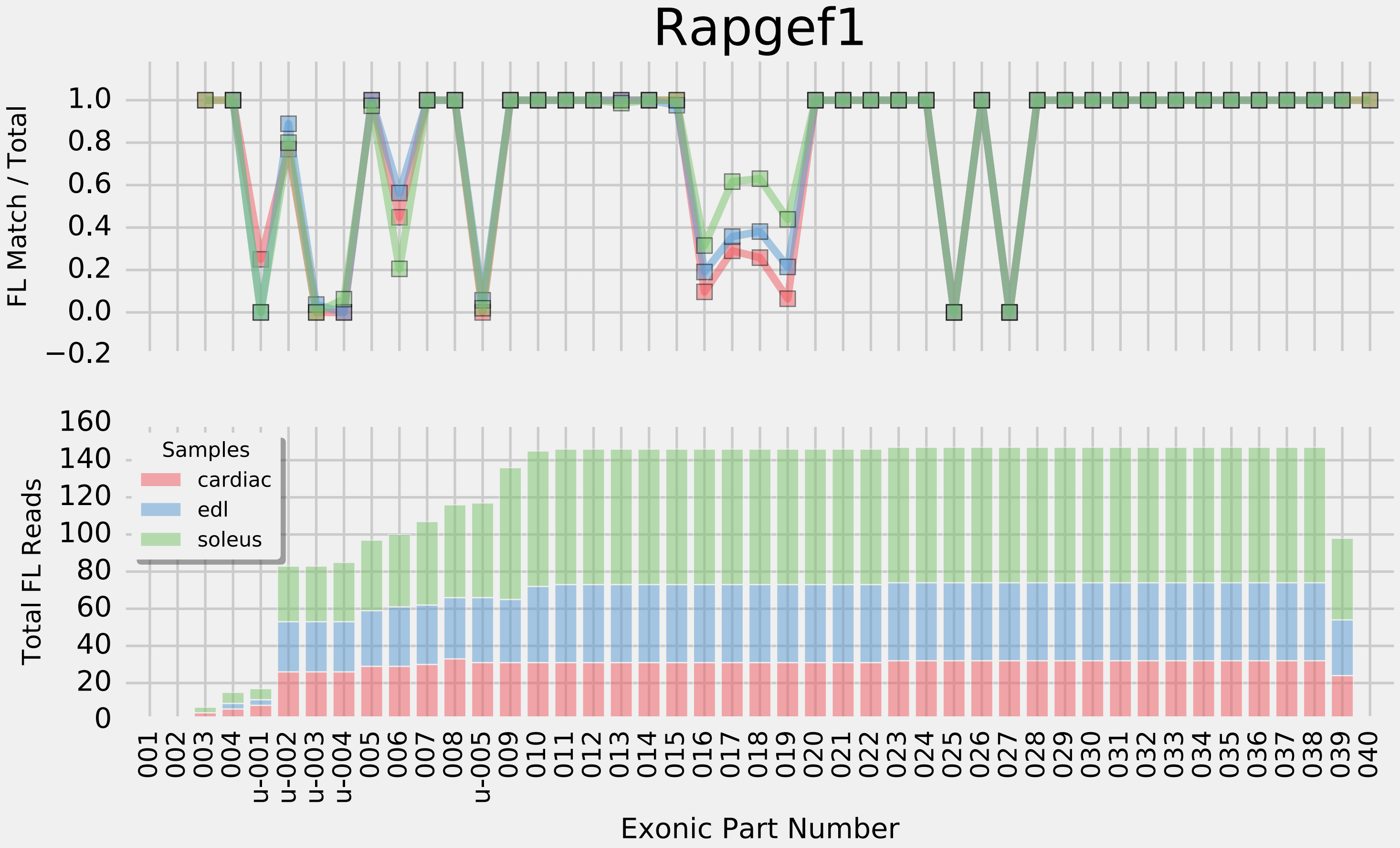

Rapgef1
FL Match / Total
1.0
0.8
0.6
0.4
0.2
0.0
−0.2
Total FL Reads
160
140
120
100
80
60
40
20
0
Samples
cardiac
edl
soleus
001
002
003
004
u-001
u-002
u-003
u-004
005
006
007
008
u-005
009
010
011
012
013
014
015
016
017
018
019
020
021
022
023
024
025
026
027
028
029
030
031
032
033
034
035
036
037
038
039
040
Exonic Part Number

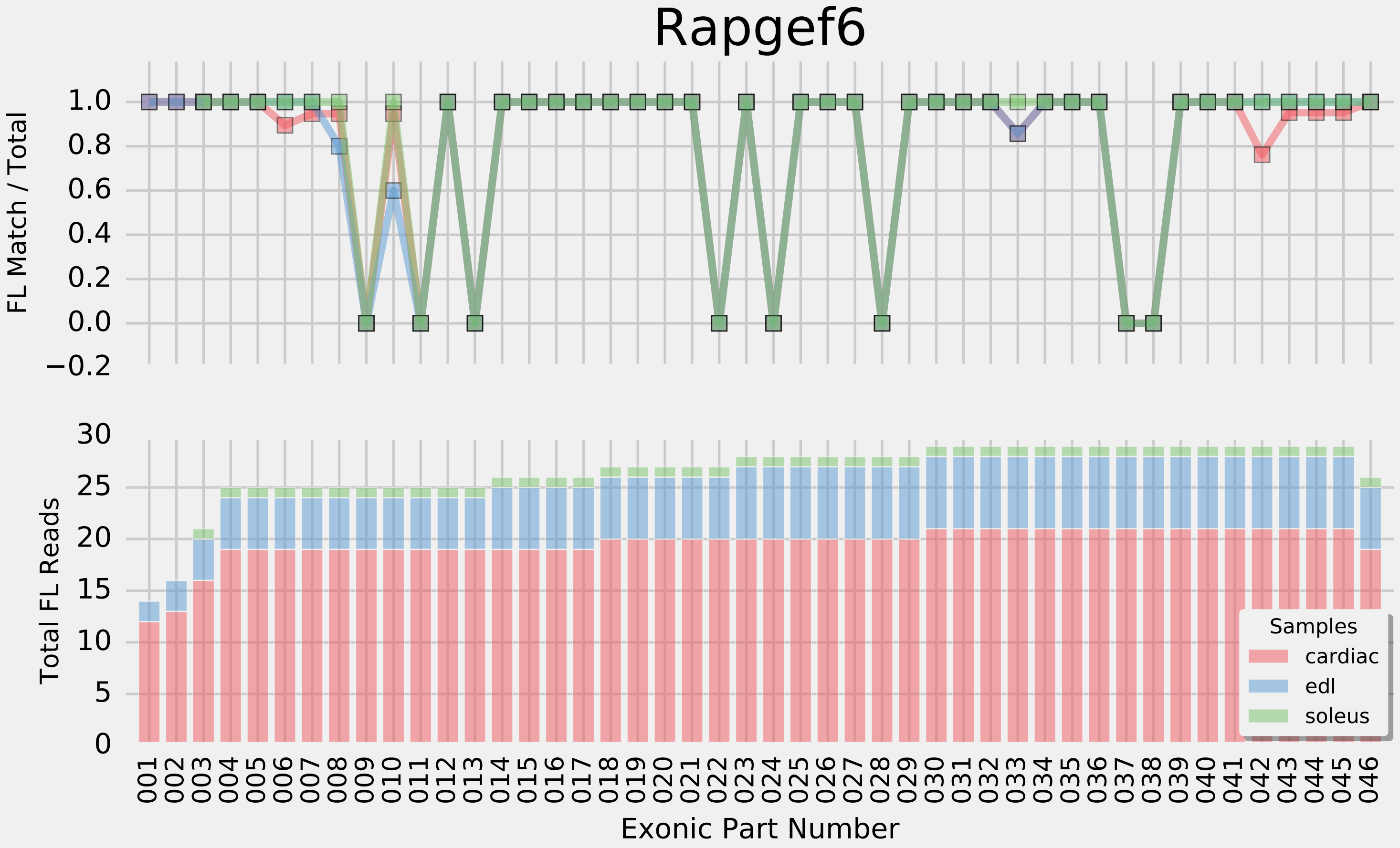
Rapgef6
FL Match / Total
1.0
0.8
0.6
0.4
0.2
0.0
−0.2
Total FL Reads
30
25
20
15
10
5
0
001 002 003 004 005 006 007 008 009 010 011 012 013 014 015 016 017 018 019 020 021 022 023 024 025 026 027 028 029 030 031 032 033 034 035 036 037 038 039 040 041 042 043 044 045 046
Exonic Part Number
Samples
cardiac
edl
soleus

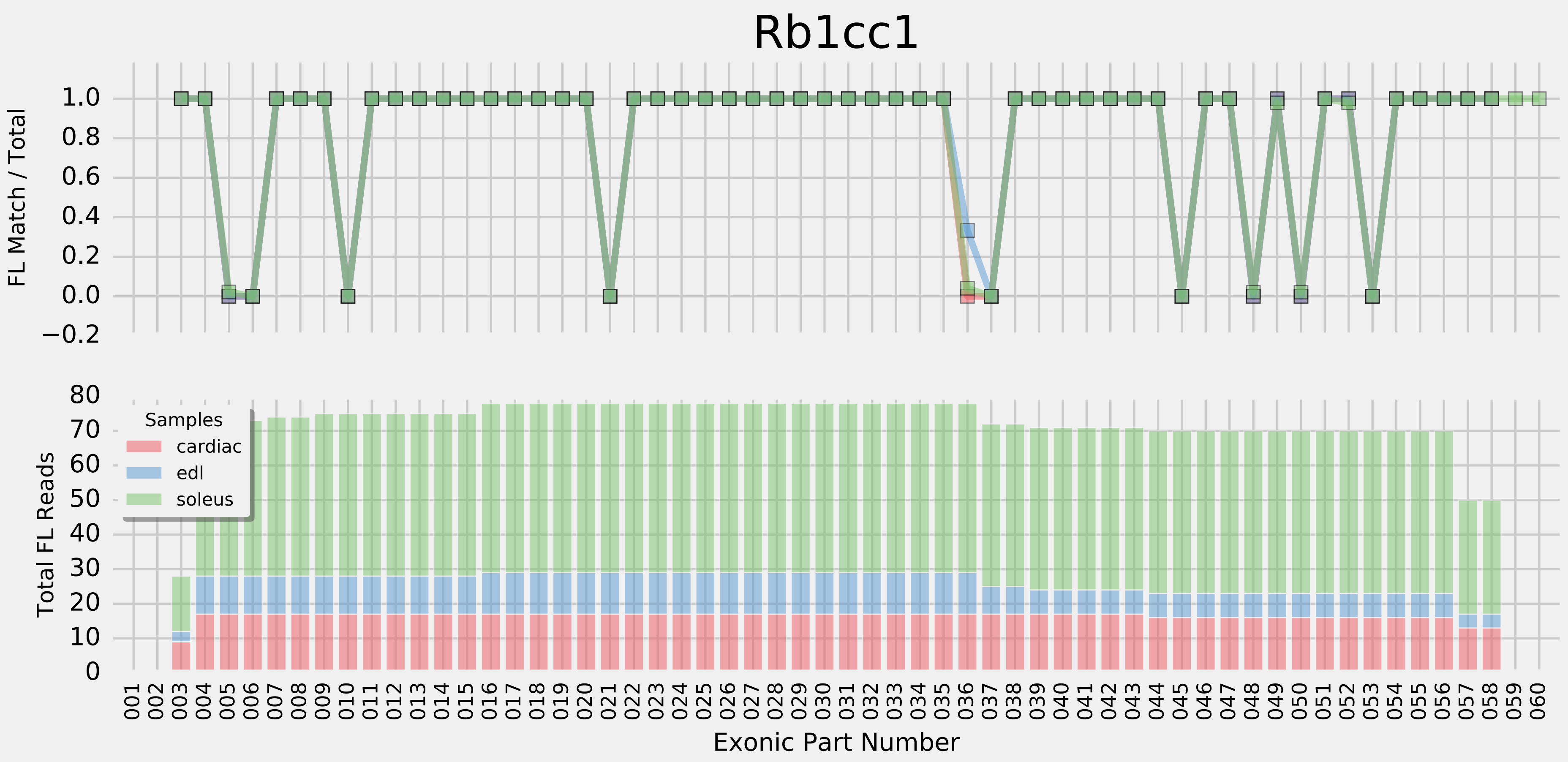

Rb1cc1
FL Match / Total
Total FL Reads
Samples
cardiac
edl
soleus
Exonic Part Number

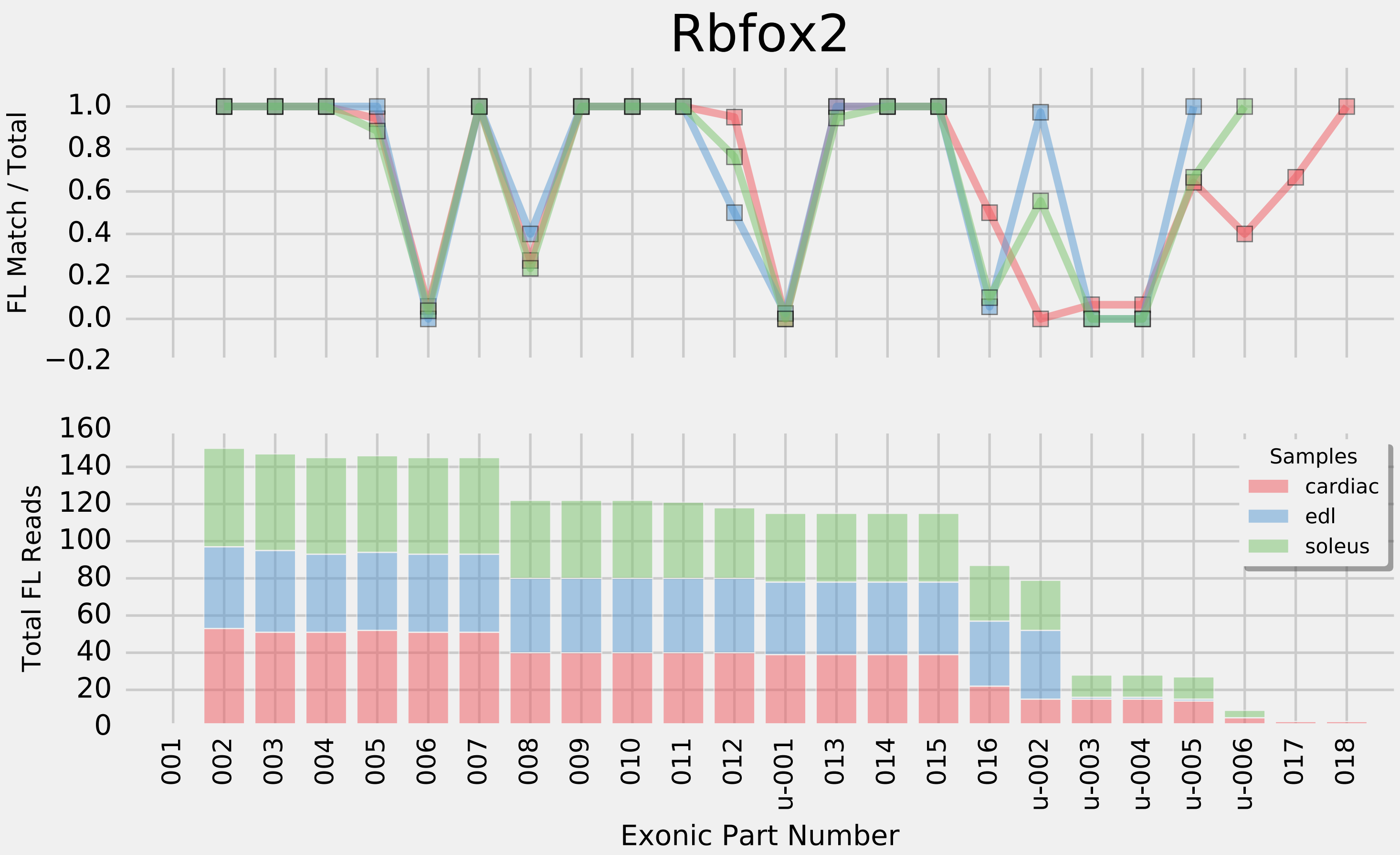

Rbfox2
FL Match / Total
1.0
0.8
0.6
0.4
0.2
0.0
−0.2
Total FL Reads
160
140
120
100
80
60
40
20
0
Samples
cardiac
edl
soleus
001
002
003
004
005
006
007
008
009
010
011
012
u-001
013
014
015
016
u-002
u-003
u-004
u-005
u-006
017
018
Exonic Part Number

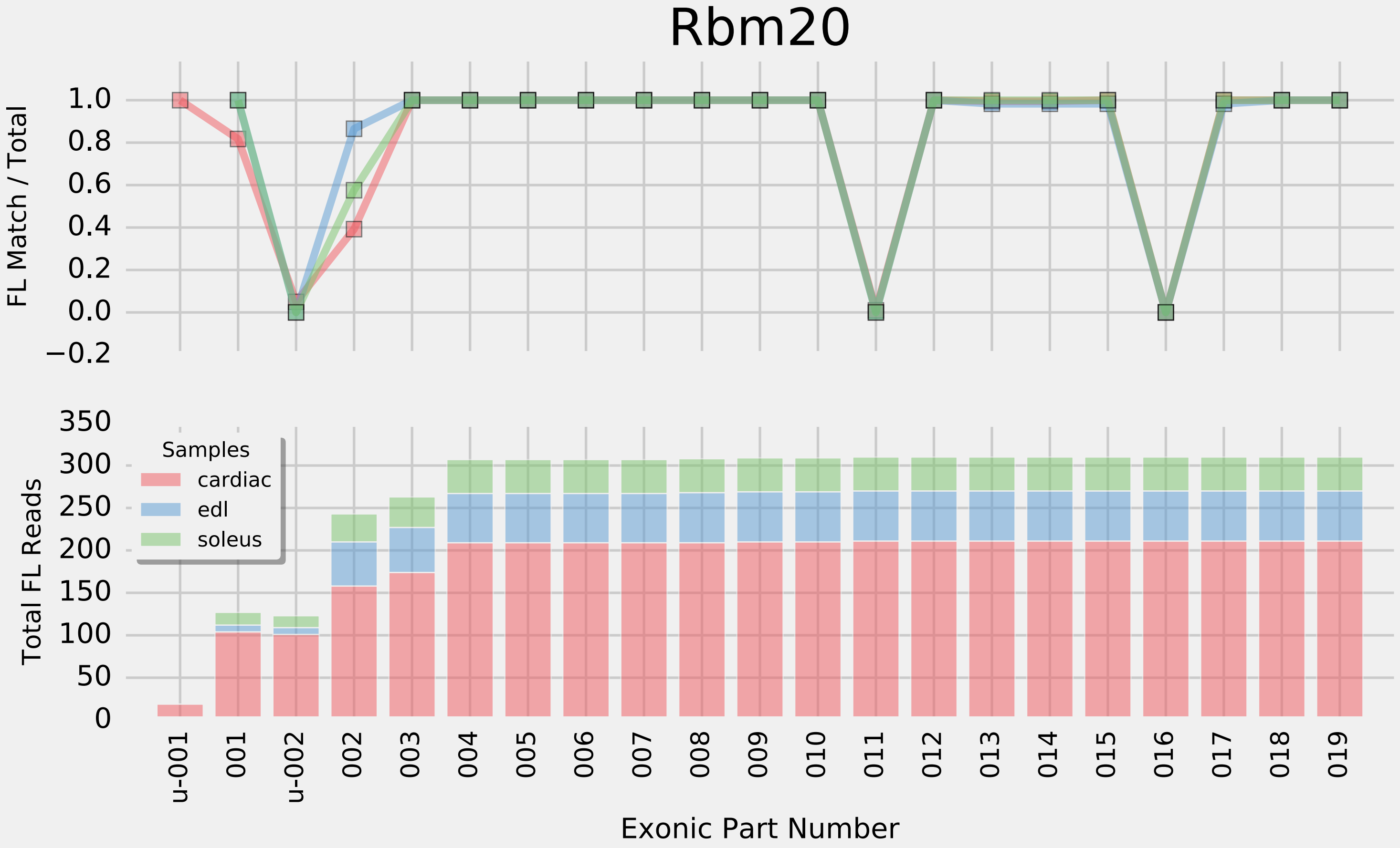
Rbm20
FL Match / Total
1.0
0.8
0.6
0.4
0.2
0.0
−0.2
350
300
250
200
150
100
50
0
Total FL Reads
Samples
cardiac
edl
soleus
u-001
001
u-002
002
003
004
005
006
007
008
009
010
011
012
013
014
015
016
017
018
019
Exonic Part Number

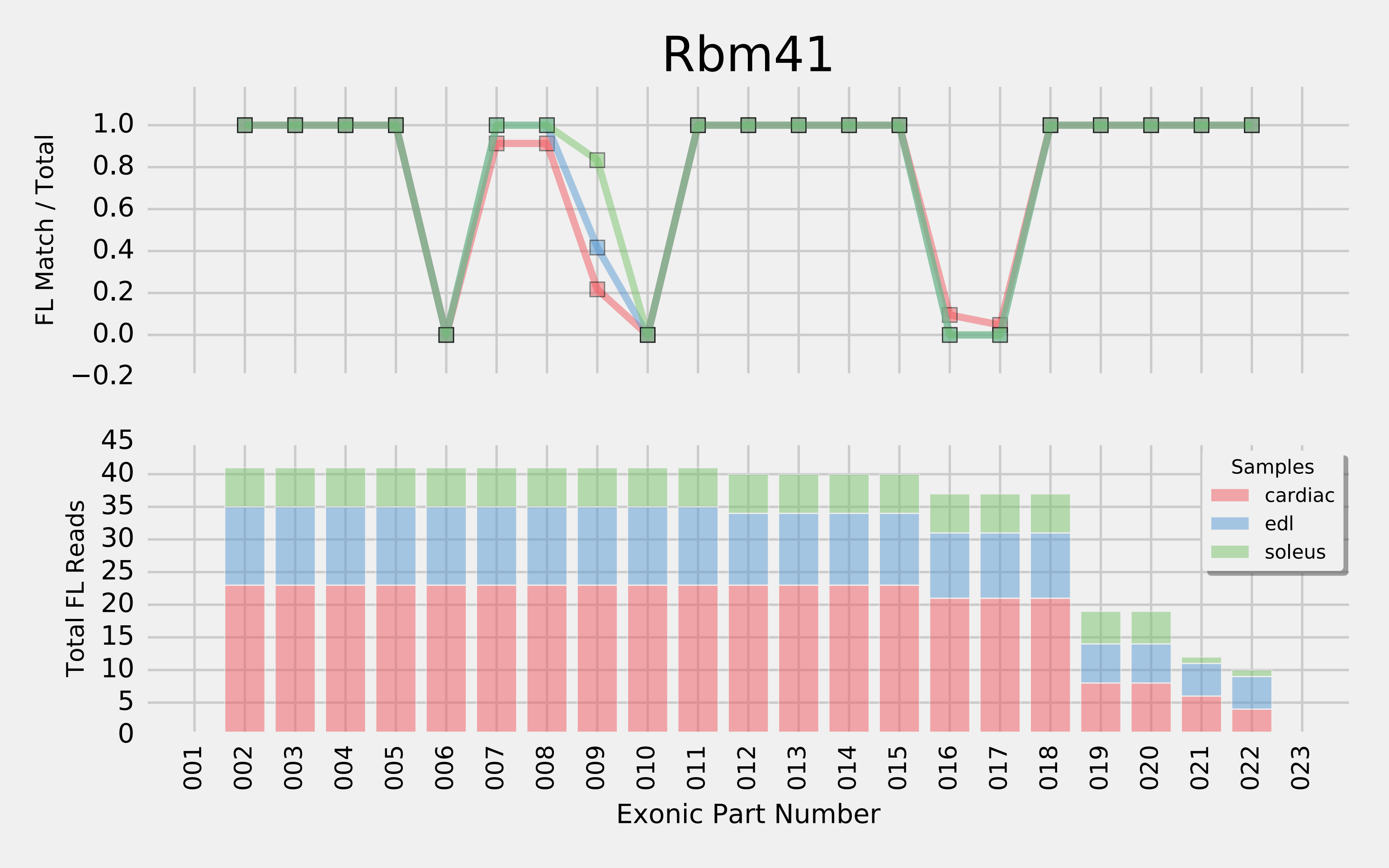

Rbm41
FL Match / Total
1.0
0.8
0.6
0.4
0.2
0.0
−0.2
Total FL Reads
45
40
35
30
25
20
15
10
5
0
Samples
cardiac
edl
soleus
001
002
003
004
005
006
007
008
009
010
011
012
013
014
015
016
017
018
019
020
021
022
023
Exonic Part Number

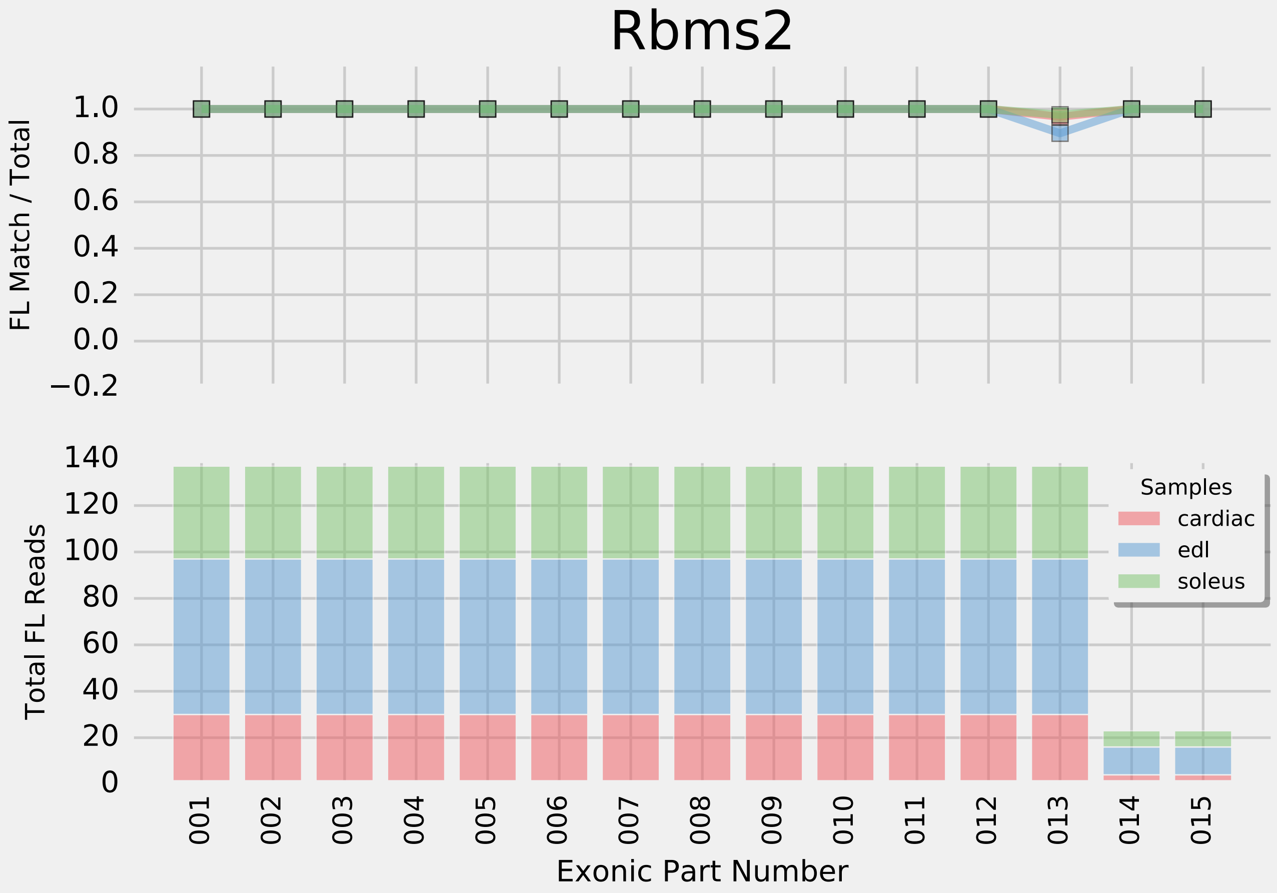

Rbms2
FL Match / Total
1.0
0.8
0.6
0.4
0.2
0.0
−0.2
Total FL Reads
140
120
100
80
60
40
20
0
Samples
cardiac
edl
soleus
001
002
003
004
005
006
007
008
009
010
011
012
013
014
015
Exonic Part Number

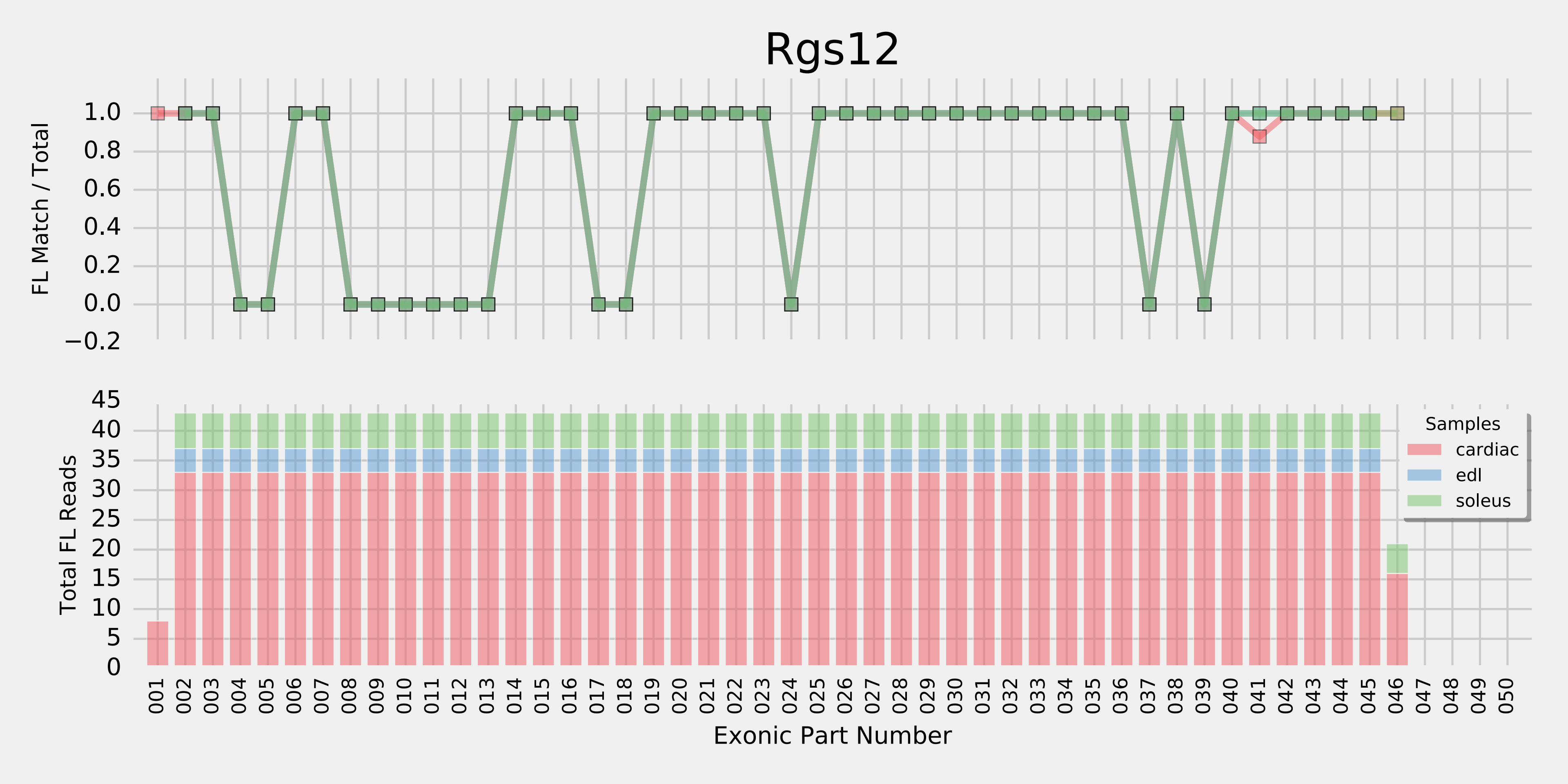
Rgs12
FL Match / Total
Total FL Reads
Exonic Part Number
Samples
cardiac
edl
soleus

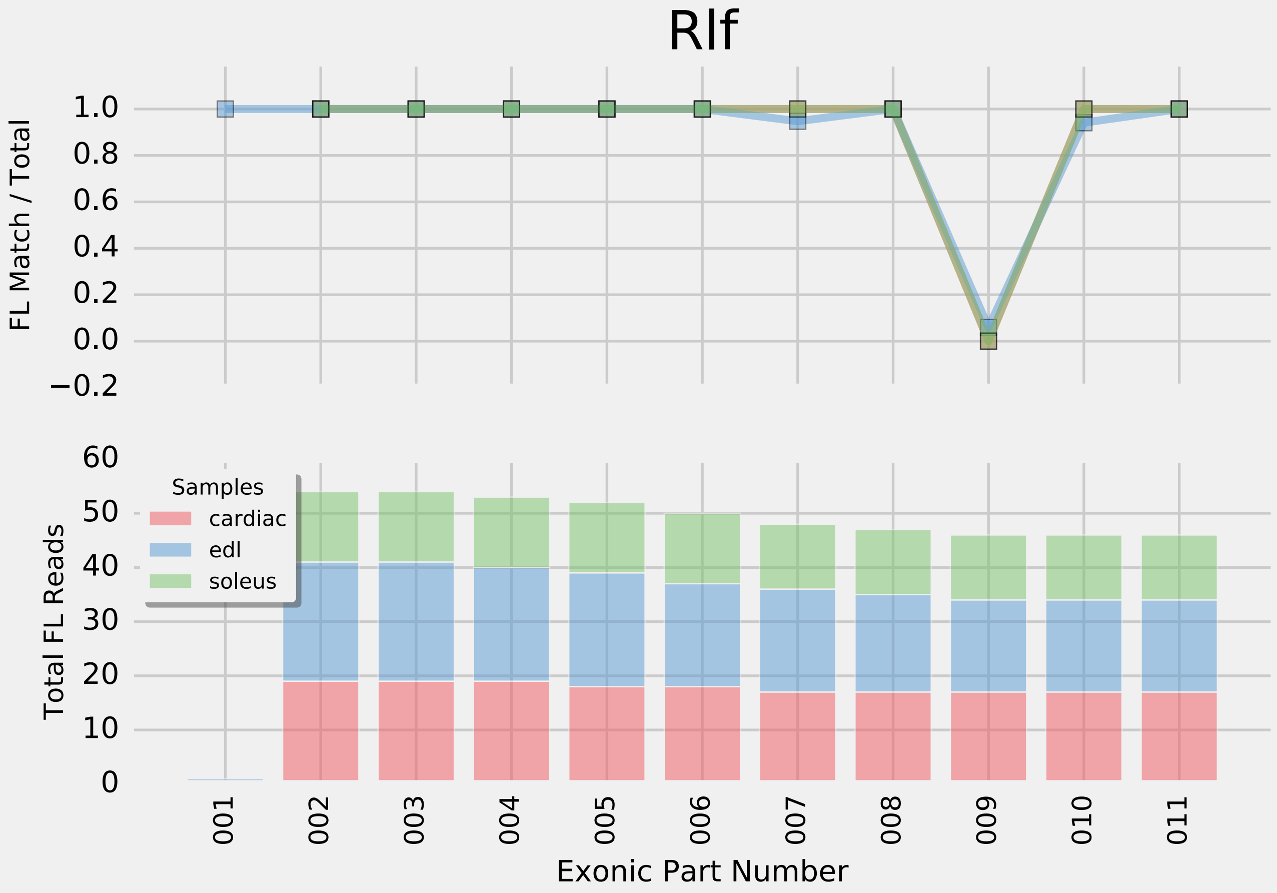
Rlf
FL Match / Total
1.0
0.8
0.6
0.4
0.2
0.0
−0.2
Total FL Reads
60
50
40
30
20
10
0
Samples
cardiac
edl
soleus
001
002
003
004
005
006
007
008
009
010
011
Exonic Part Number

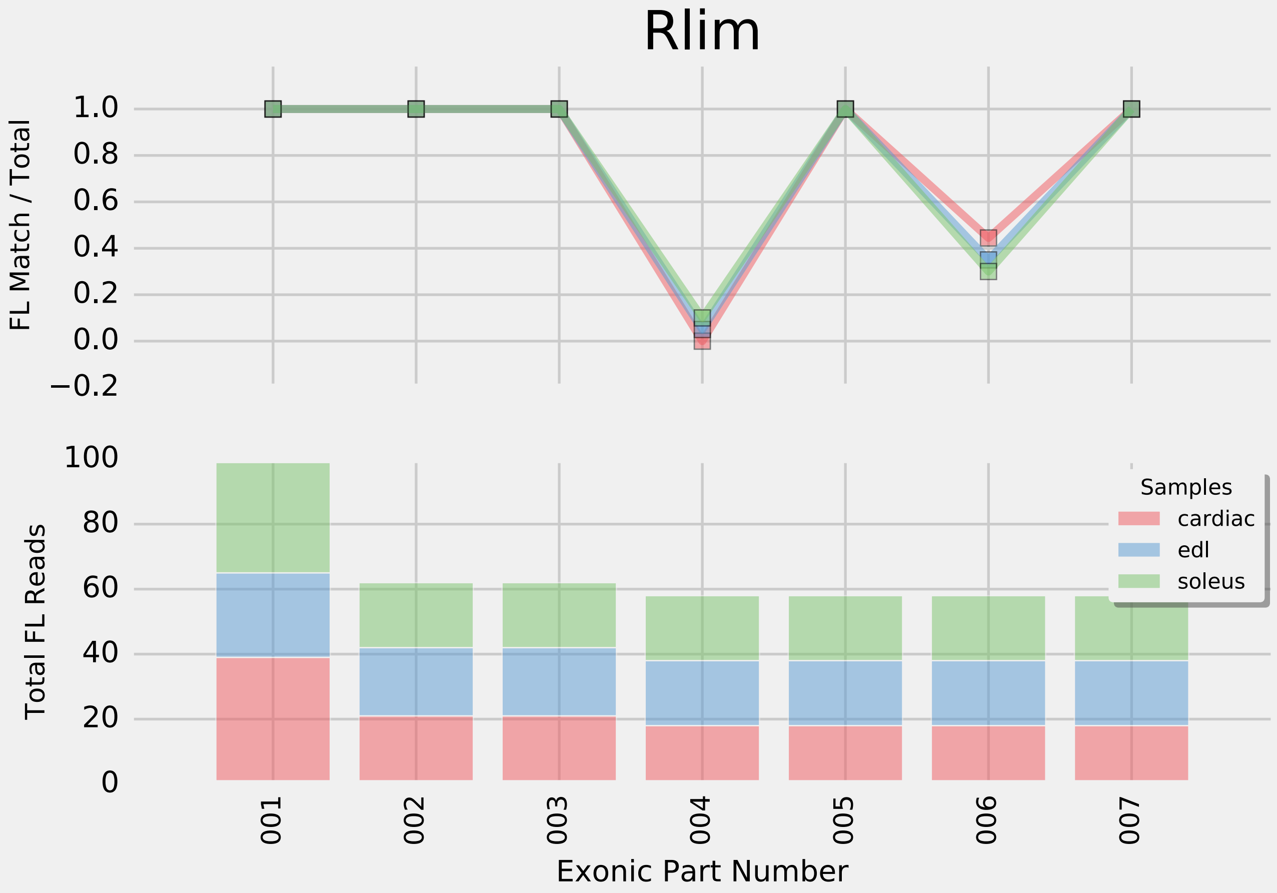
Rlim
FL Match / Total
1.0
0.8
0.6
0.4
0.2
0.0
−0.2
Total FL Reads
100
80
60
40
20
0
Samples
cardiac
edl
soleus
001
002
003
004
005
006
007
Exonic Part Number

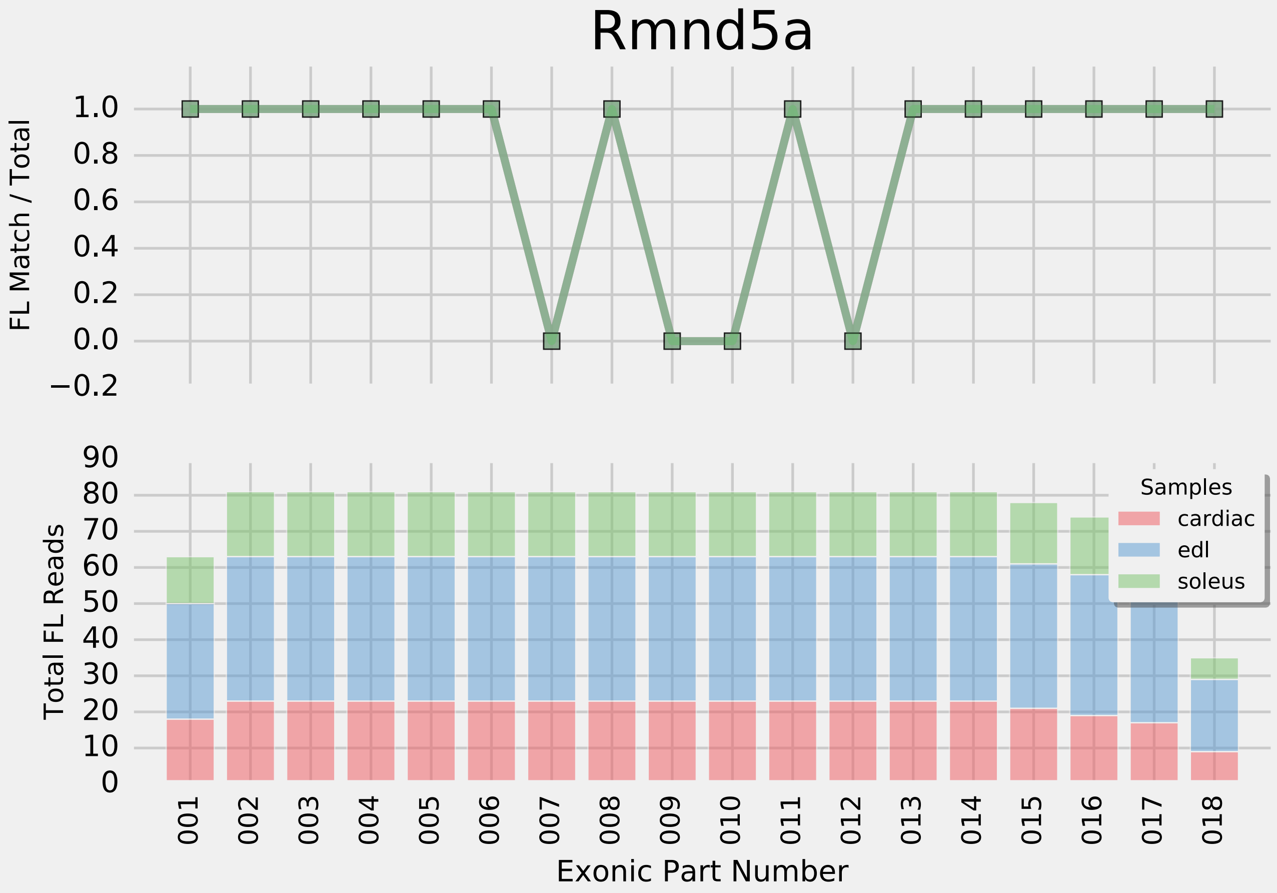
Rmnd5a
FL Match / Total
1.0
0.8
0.6
0.4
0.2
0.0
−0.2
Total FL Reads
90
80
70
60
50
40
30
20
10
0
Samples
cardiac
edl
soleus
001
002
003
004
005
006
007
008
009
010
011
012
013
014
015
016
017
018
Exonic Part Number

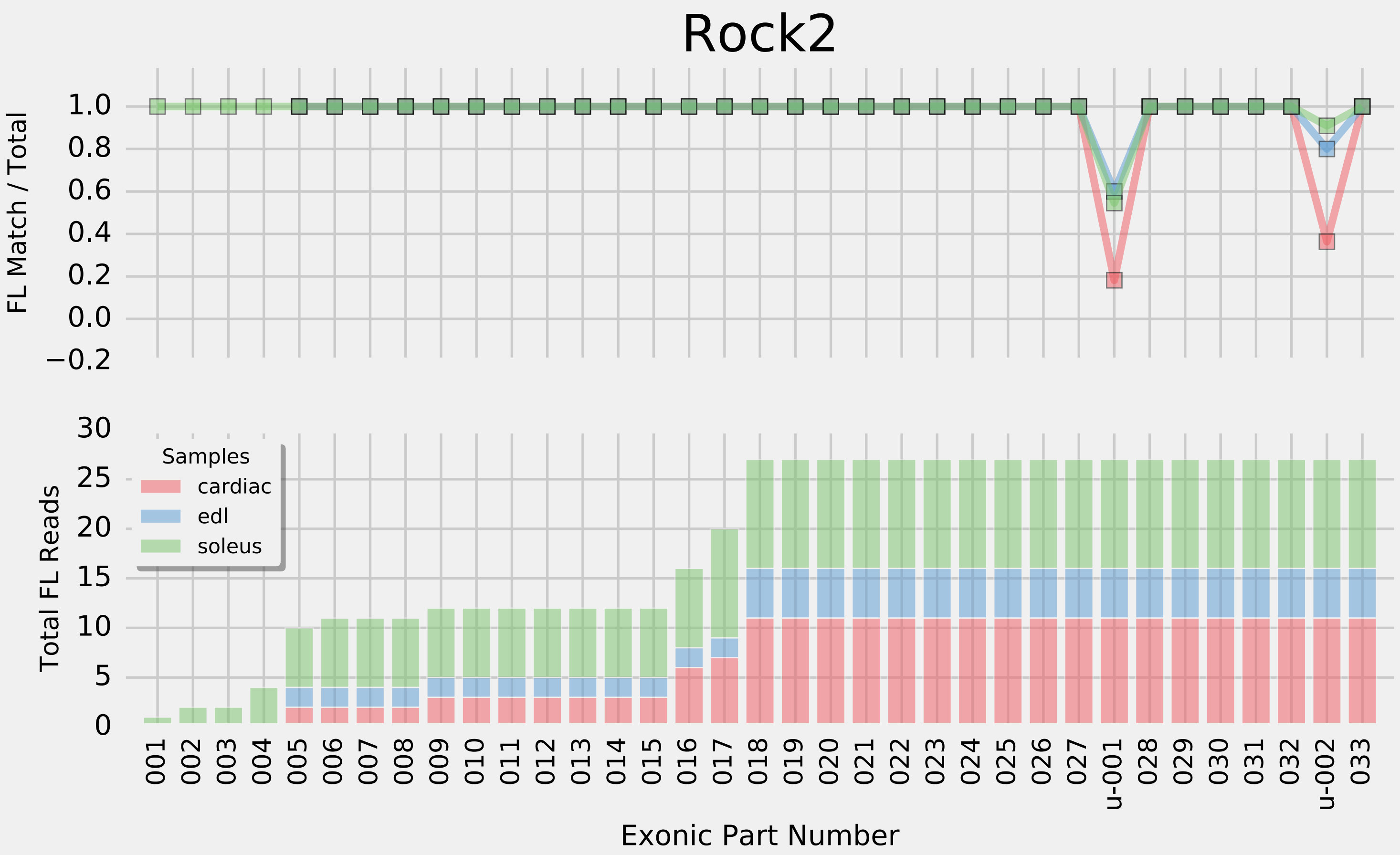

Rock2
FL Match / Total
1.0
0.8
0.6
0.4
0.2
0.0
−0.2
Total FL Reads
30
25
20
15
10
5
0
Samples
cardiac
edl
soleus
001
002
003
004
005
006
007
008
009
010
011
012
013
014
015
016
017
018
019
020
021
022
023
024
025
026
027
u-001
028
029
030
031
032
u-002
033
Exonic Part Number

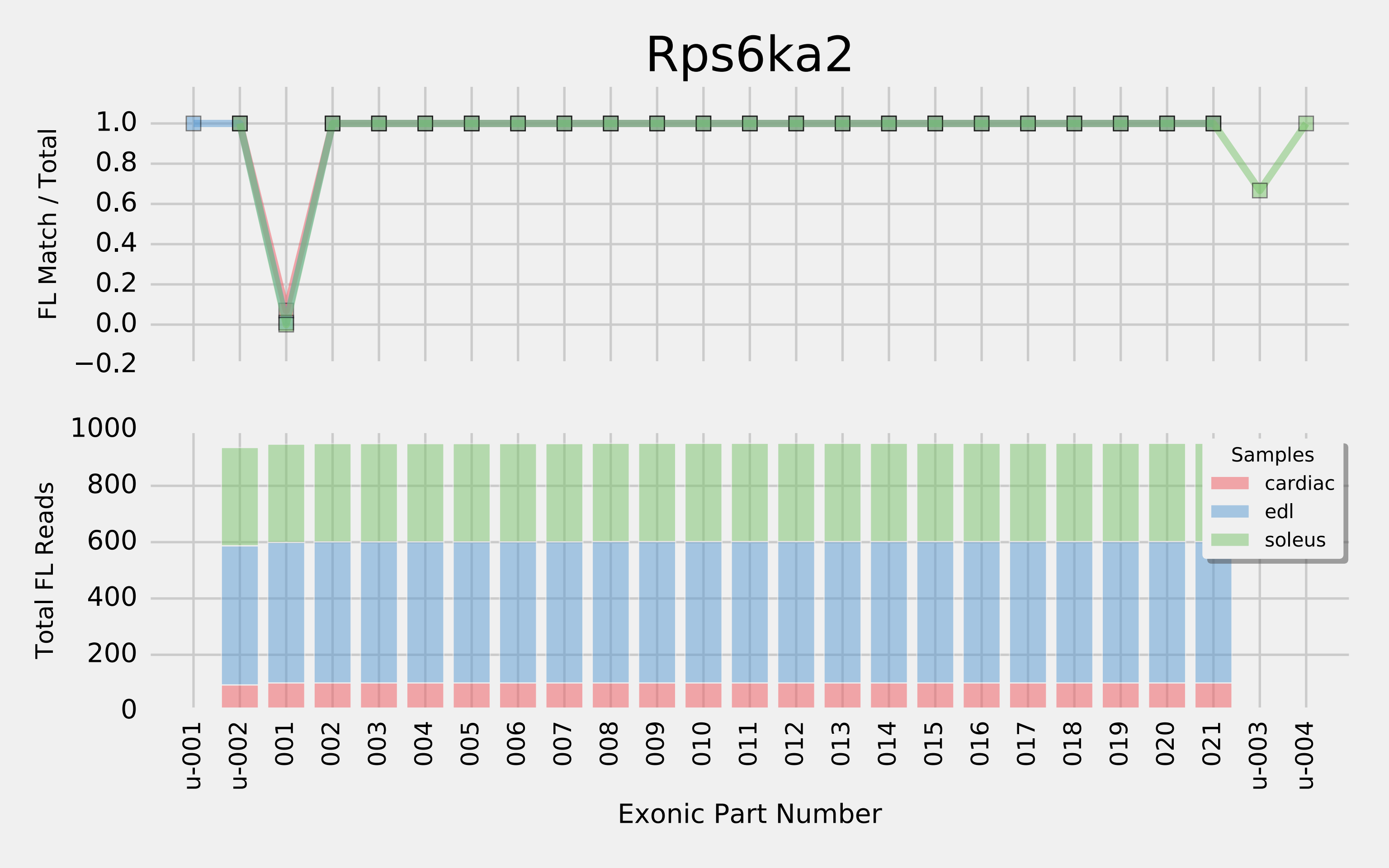

Rps6ka2
FL Match / Total
1.0
0.8
0.6
0.4
0.2
0.0
−0.2
Total FL Reads
1000
800
600
400
200
0
Samples
cardiac
edl
soleus
u-001
u-002
001
002
003
004
005
006
007
008
009
010
011
012
013
014
015
016
017
018
019
020
021
u-003
u-004
Exonic Part Number

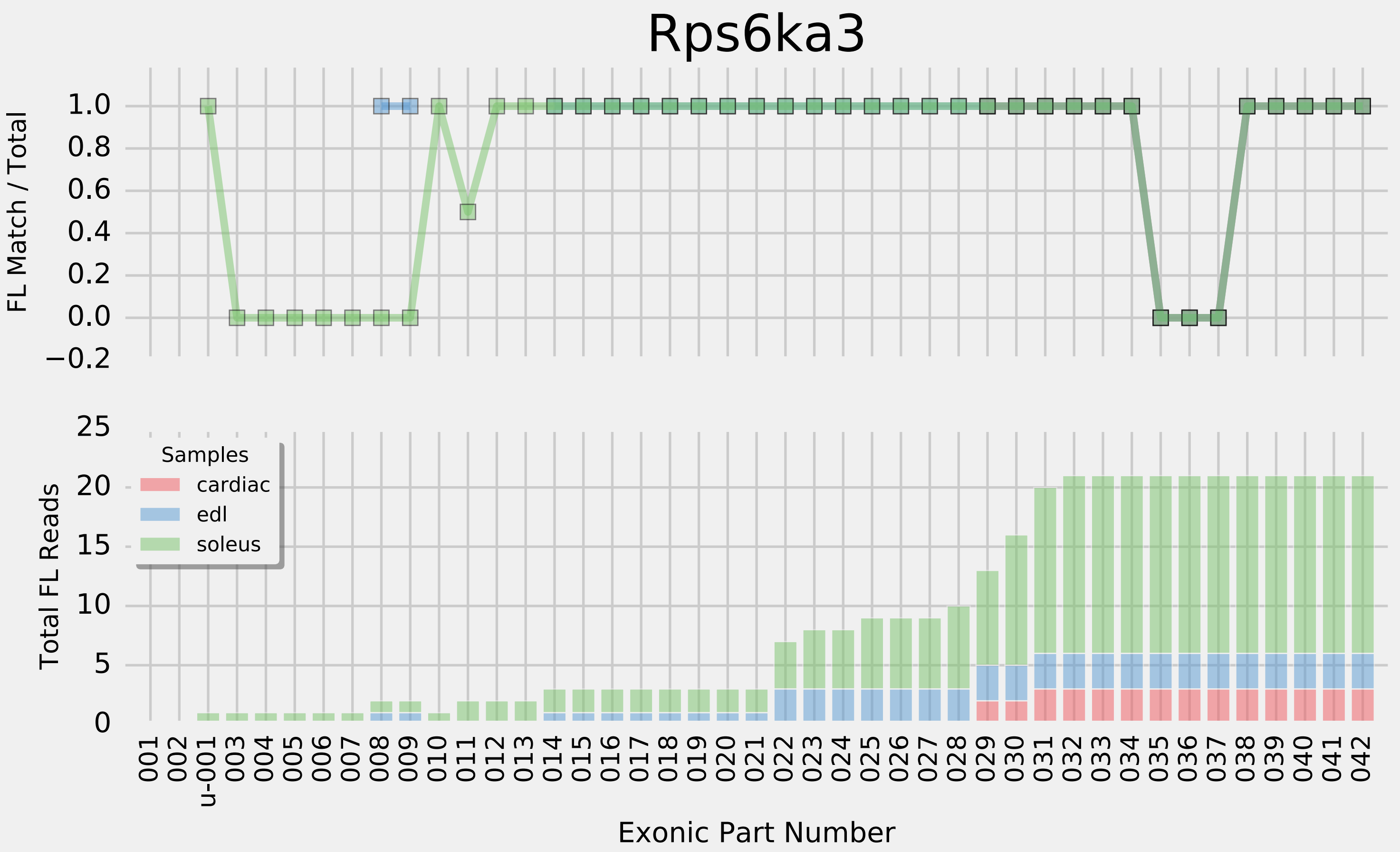
Rps6ka3
FL Match / Total
Total FL Reads
Samples
cardiac
edl
soleus
Exonic Part Number
001
002
u-001
003
004
005
006
007
008
009
010
011
012
013
014
015
016
017
018
019
020
021
022
023
024
025
026
027
028
029
030
031
032
033
034
035
036
037
038
039
040
041
042

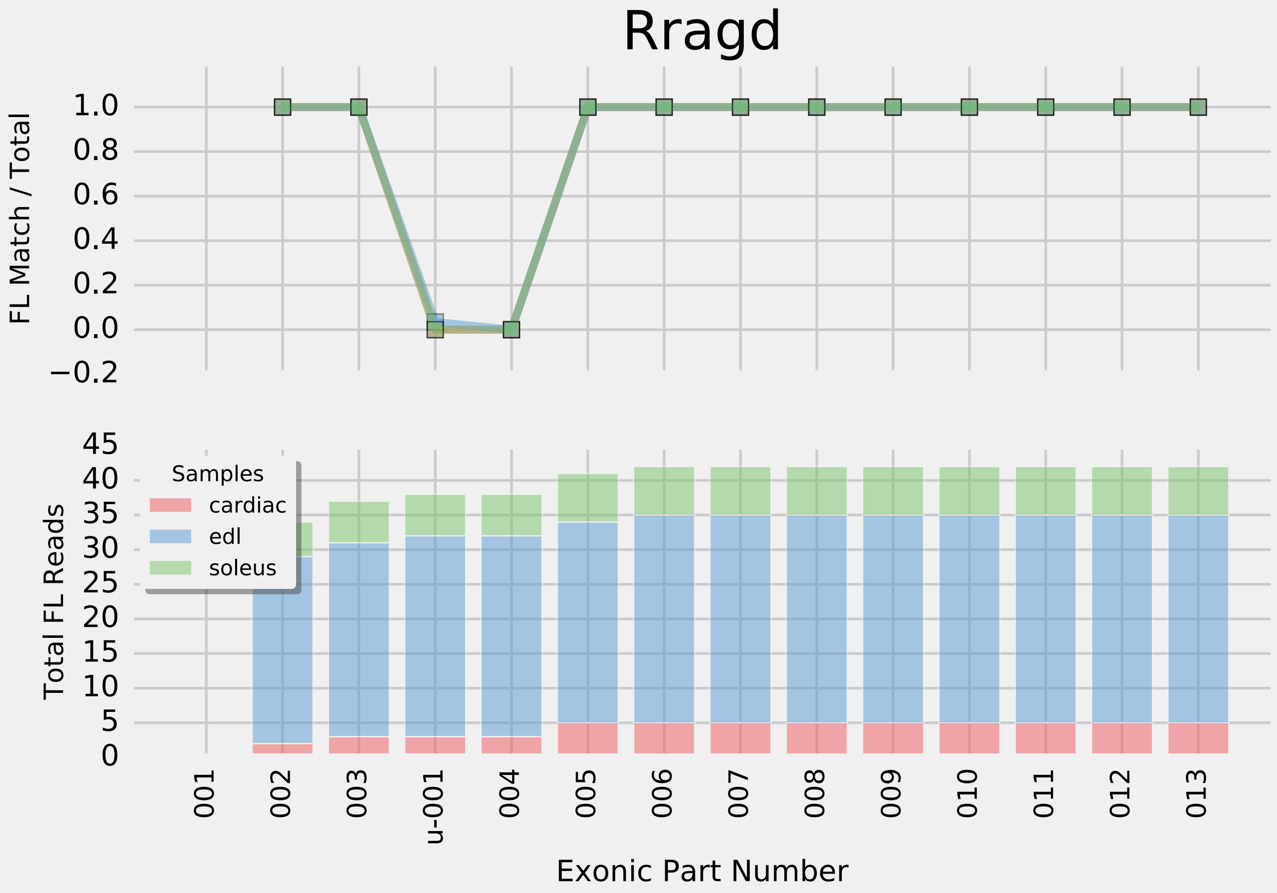

Rragd
FL Match / Total
1.0
0.8
0.6
0.4
0.2
0.0
−0.2
Total FL Reads
45
40
35
30
25
20
15
10
5
0
Samples
cardiac
edl
soleus
001
002
003
u-001
004
005
006
007
008
009
010
011
012
013
Exonic Part Number

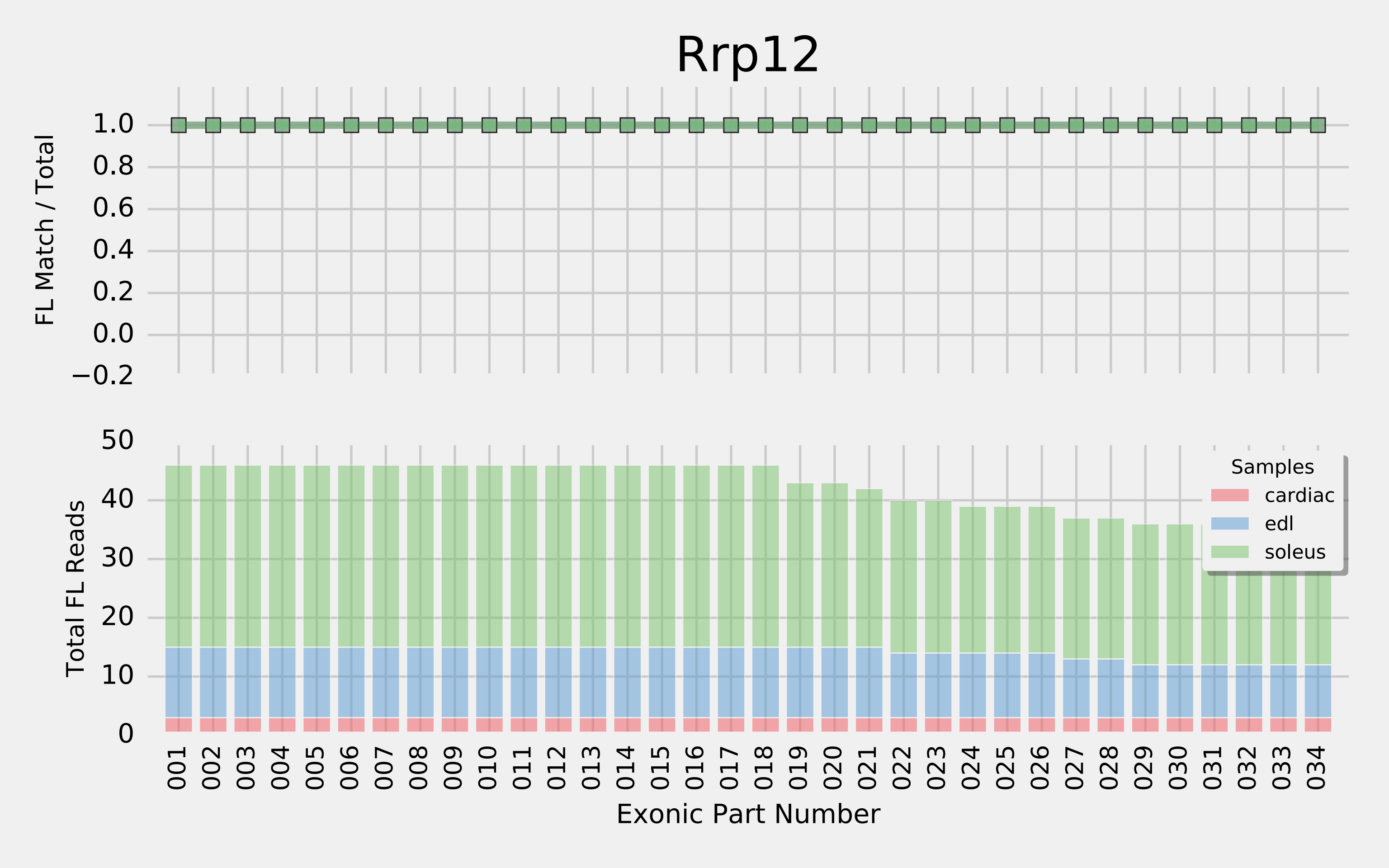

Rrp12
FL Match / Total
1.0
0.8
0.6
0.4
0.2
0.0
−0.2
Total FL Reads
50
40
30
20
10
0
Samples
cardiac
edl
soleus
001
002
003
004
005
006
007
008
009
010
011
012
013
014
015
016
017
018
019
020
021
022
023
024
025
026
027
028
029
030
031
032
033
034
Exonic Part Number

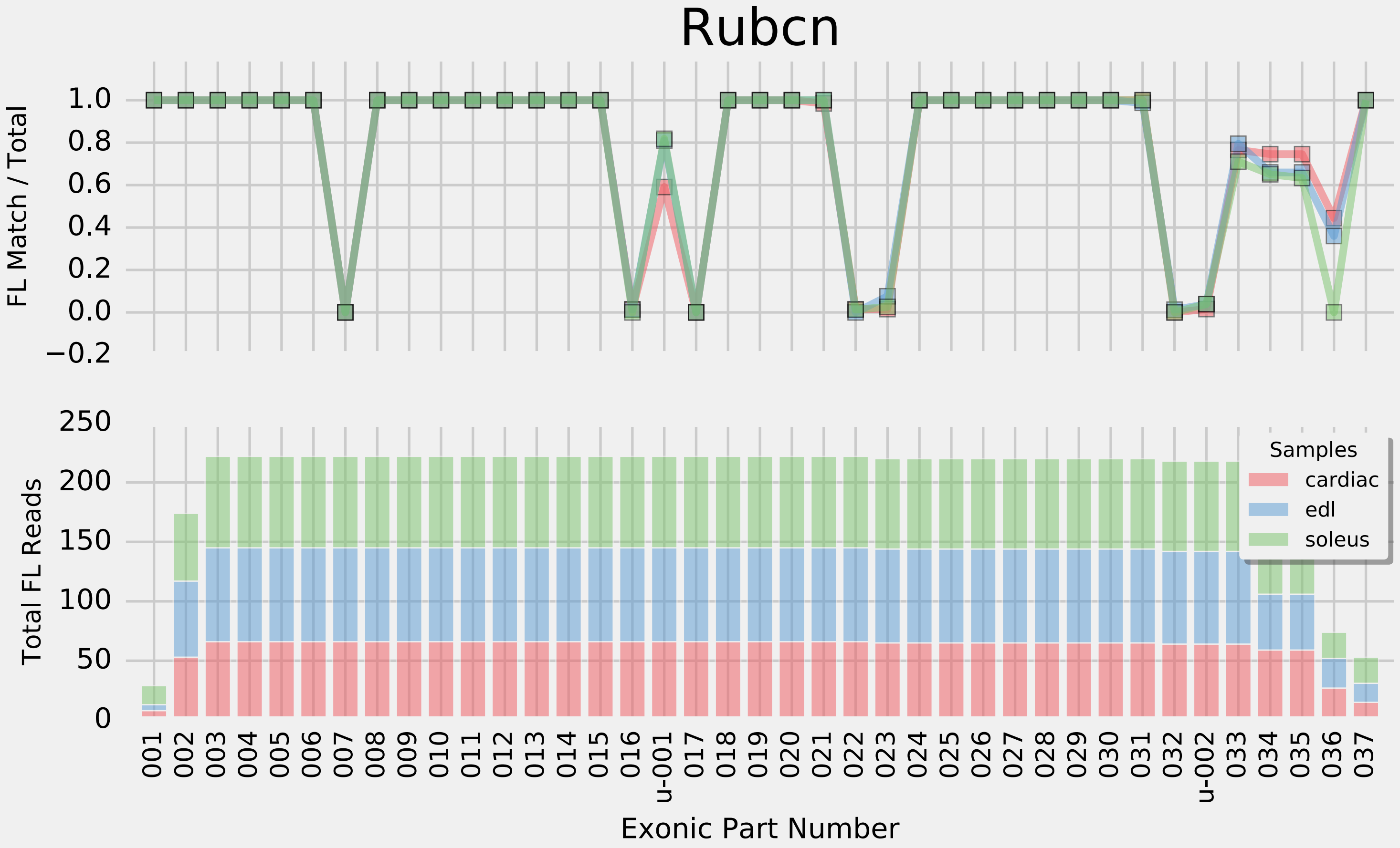

Rubcn
FL Match / Total
1.0
0.8
0.6
0.4
0.2
0.0
−0.2
Total FL Reads
250
200
150
100
50
0
Samples
cardiac
edl
soleus
001
002
003
004
005
006
007
008
009
010
011
012
013
014
015
016
u-001
017
018
019
020
021
022
023
024
025
026
027
028
029
030
031
032
u-002
033
034
035
036
037
Exonic Part Number

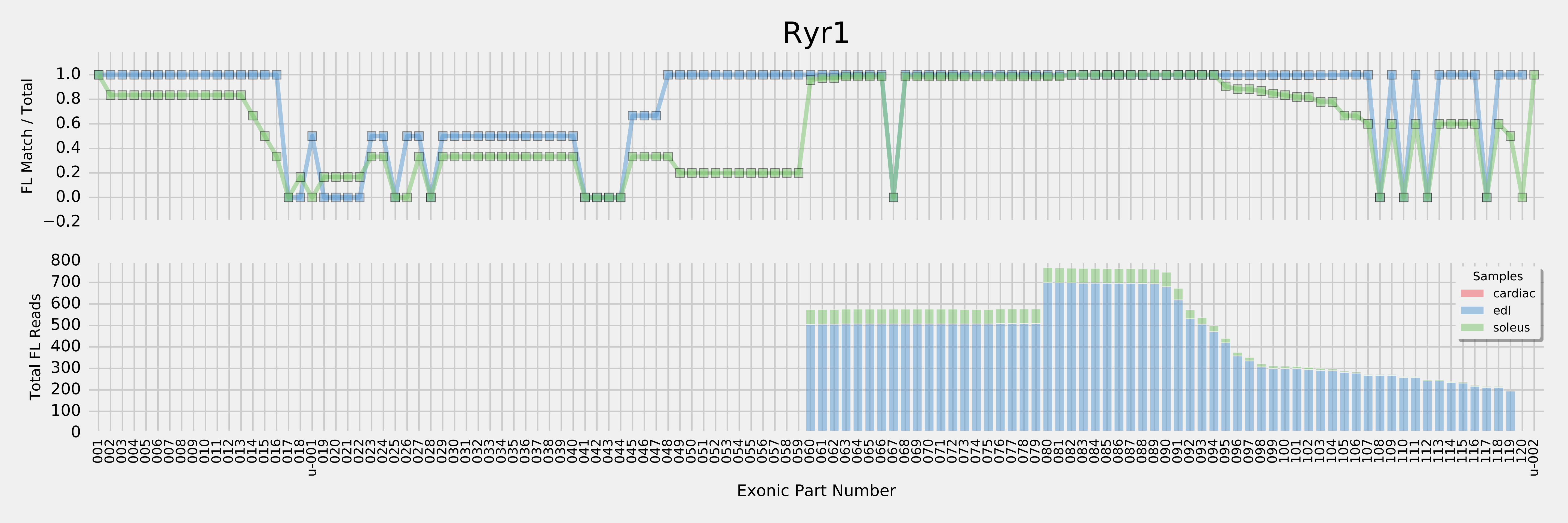

Ryr1
FL Match / Total
Total FL Reads
Exonic Part Number
Samples
cardiac
edl
soleus

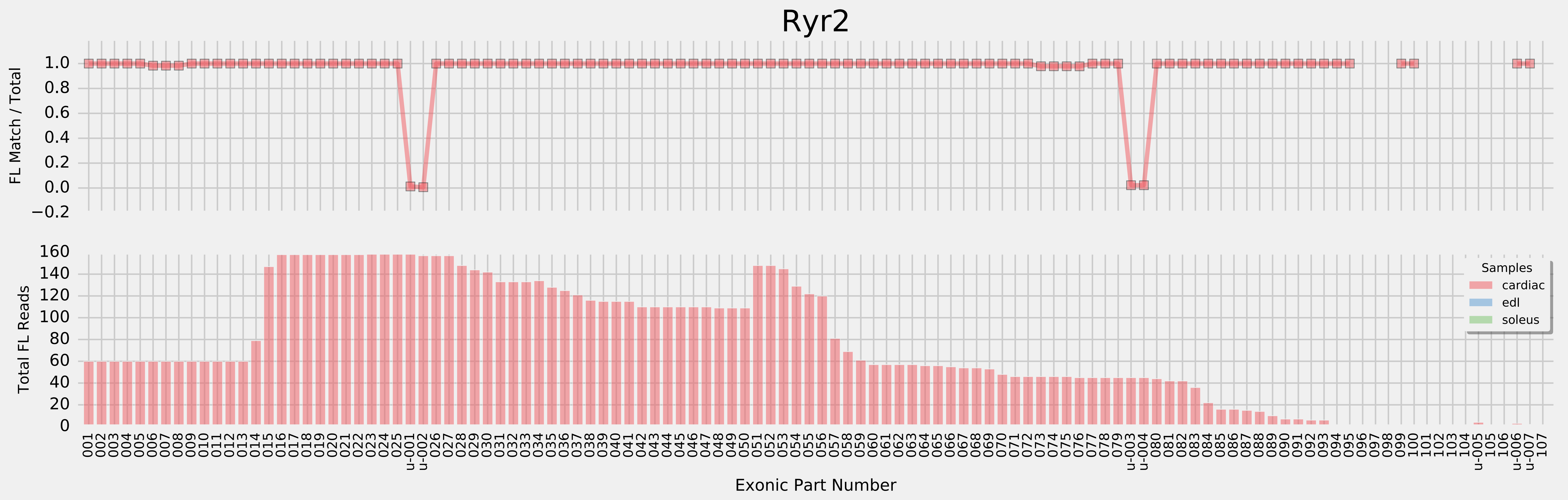

Ryr2
FL Match / Total
Total FL Reads
Exonic Part Number
Samples
cardiac
edl
soleus

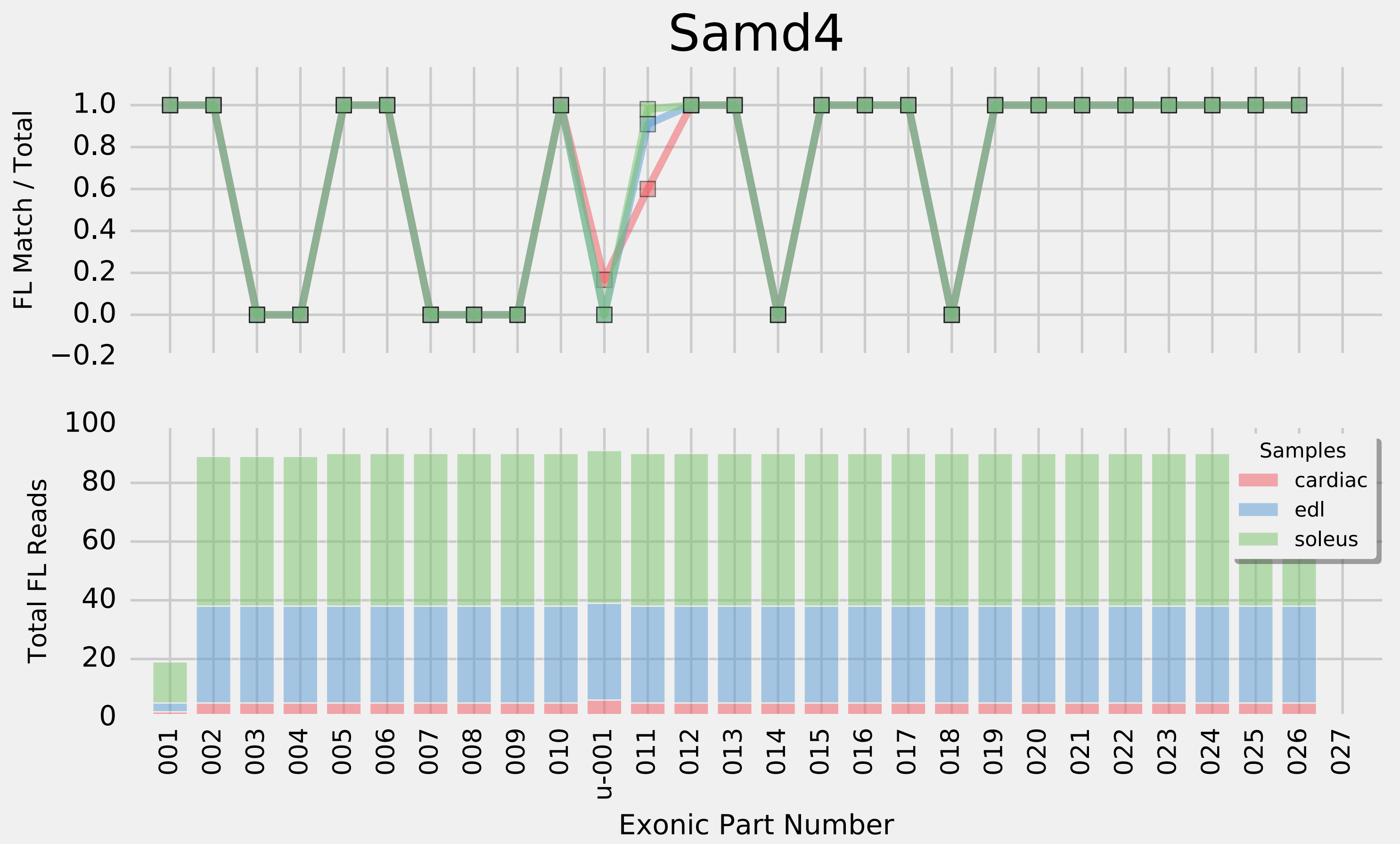
Samd4
FL Match / Total
1.0
0.8
0.6
0.4
0.2
0.0
−0.2
Total FL Reads
100
80
60
40
20
0
Samples
cardiac
edl
soleus
001
002
003
004
005
006
007
008
009
010
u-001
011
012
013
014
015
016
017
018
019
020
021
022
023
024
025
026
027
Exonic Part Number

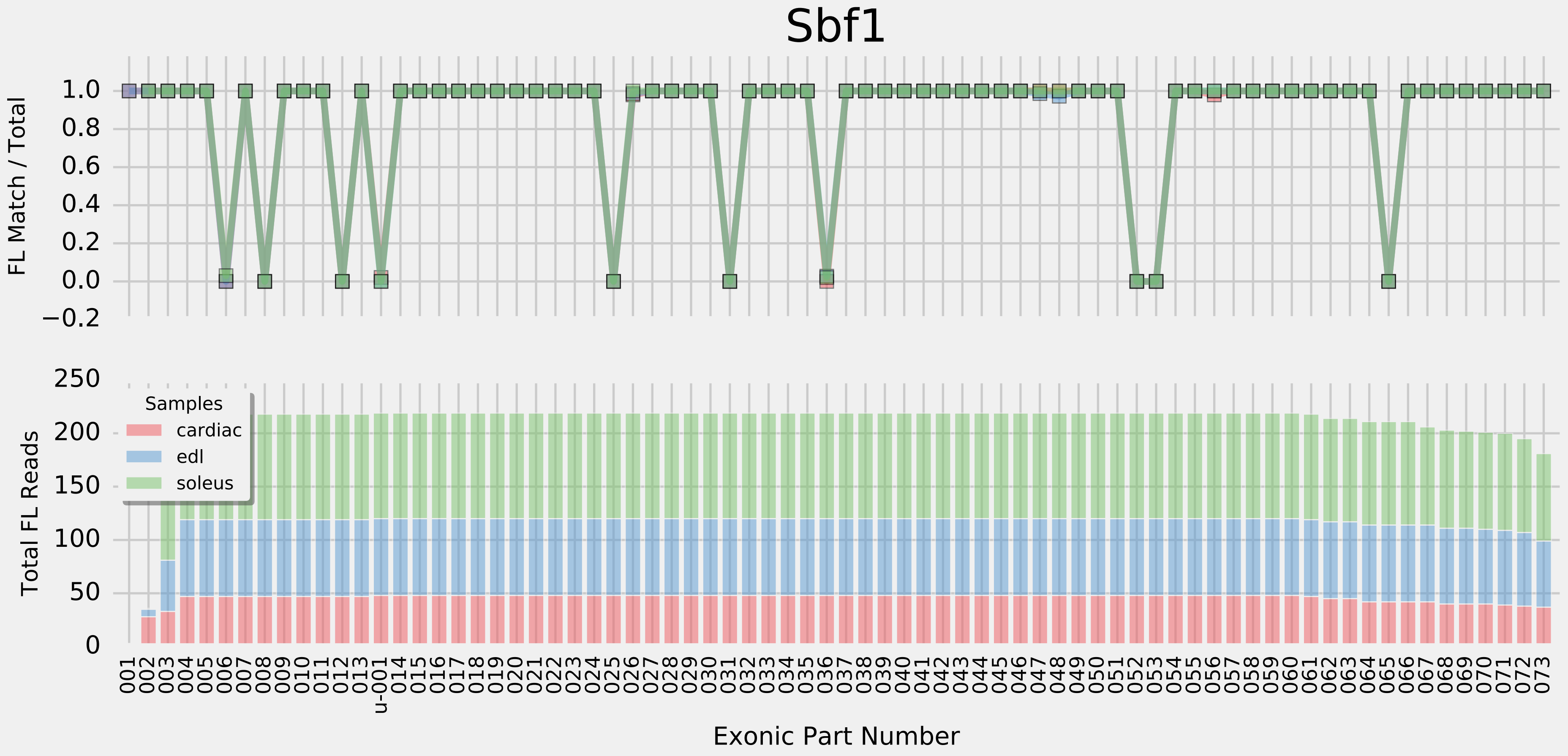

Sbf1
FL Match / Total
Total FL Reads
Samples
cardiac
edl
soleus
Exonic Part Number

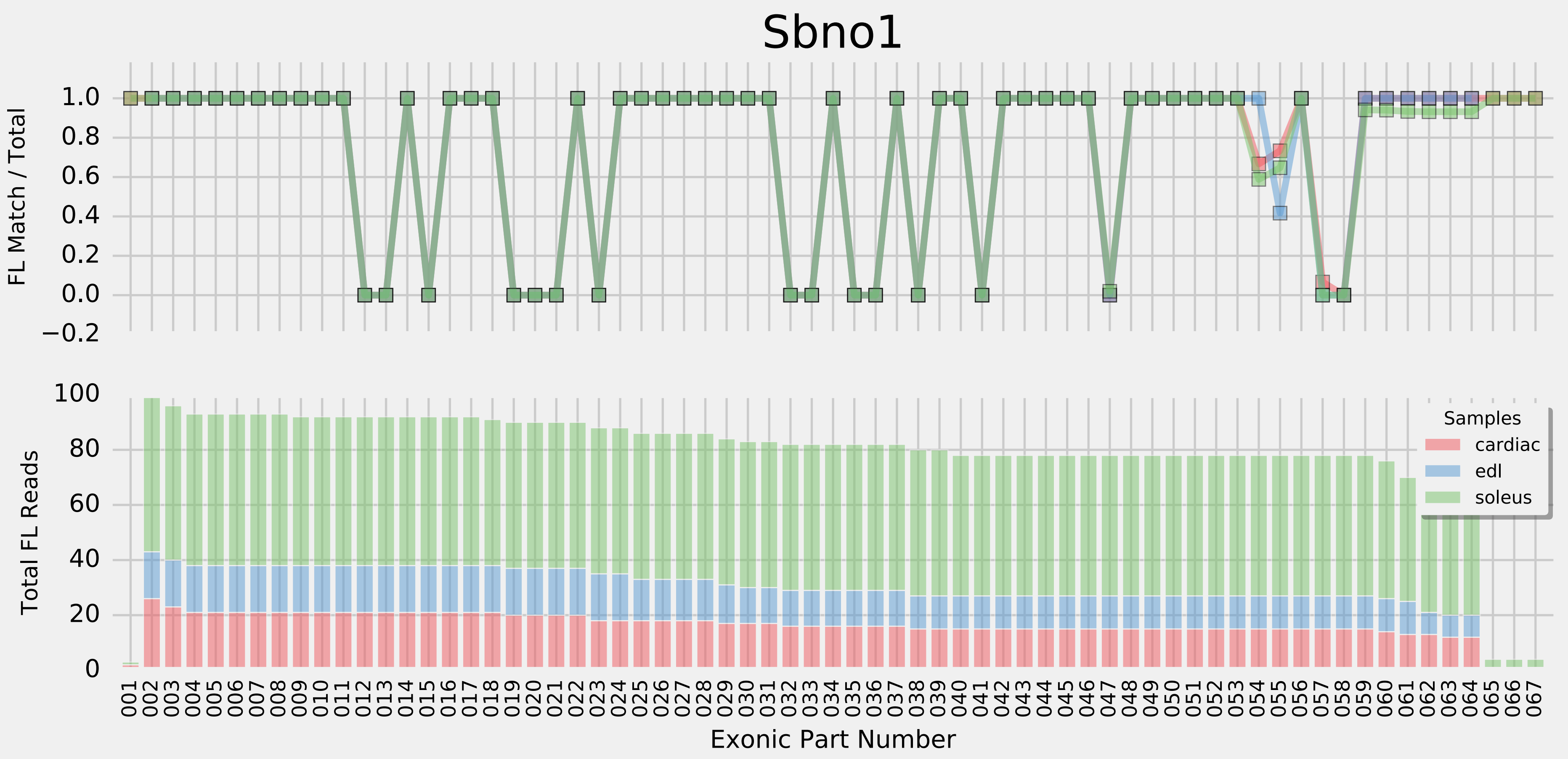

Sbno1
FL Match / Total
1.0
0.8
0.6
0.4
0.2
0.0
−0.2
Total FL Reads
100
80
60
40
20
0
Samples
cardiac
edl
soleus
001 002 003 004 005 006 007 008 009 010 011 012 013 014 015 016 017 018 019 020 021 022 023 024 025 026 027 028 029 030 031 032 033 034 035 036 037 038 039 040 041 042 043 044 045 046 047 048 049 050 051 052 053 054 055 056 057 058 059 060 061 062 063 064 065 066 067
Exonic Part Number

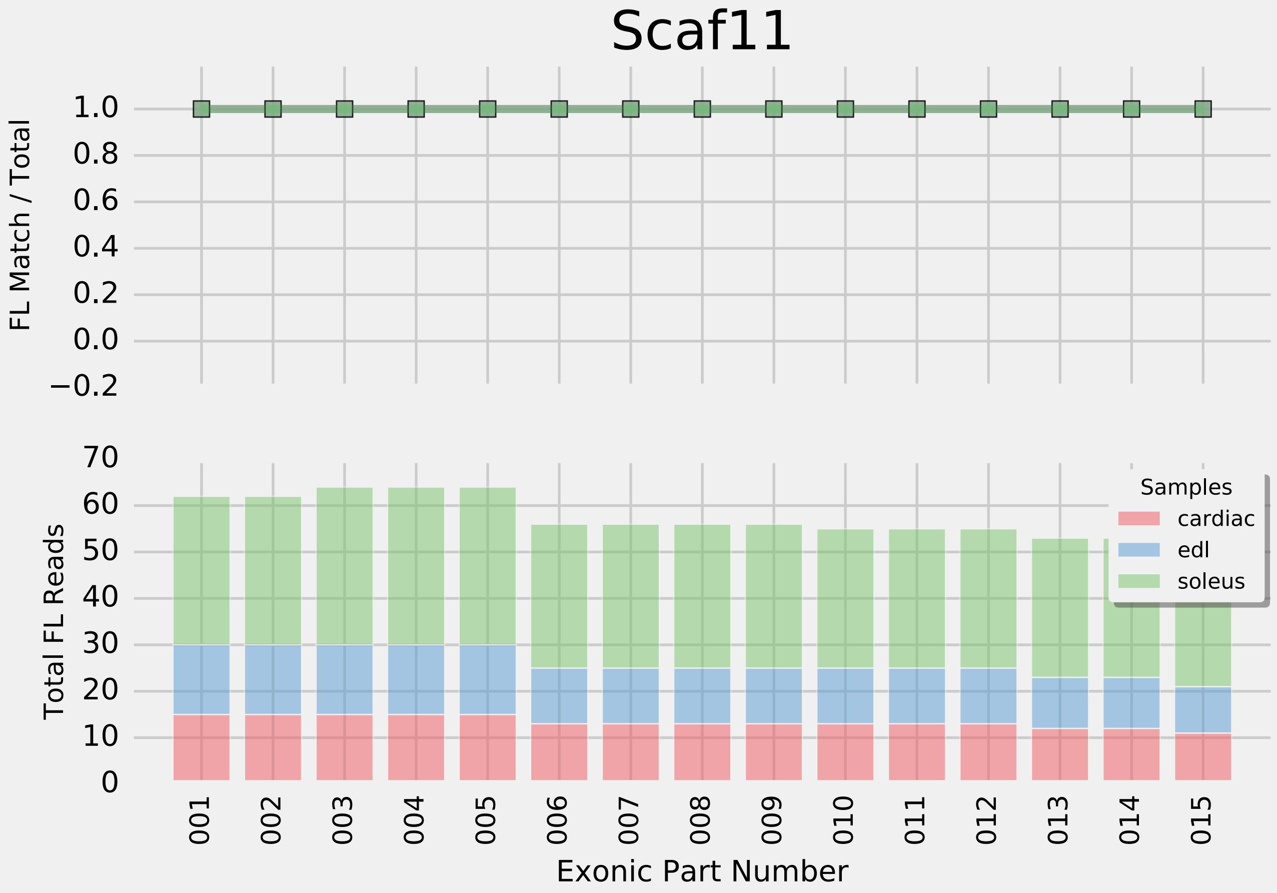

Scaf11
FL Match / Total
1.0
0.8
0.6
0.4
0.2
0.0
−0.2
Total FL Reads
70
60
50
40
30
20
10
0
Samples
cardiac
edl
soleus
001
002
003
004
005
006
007
008
009
010
011
012
013
014
015
Exonic Part Number

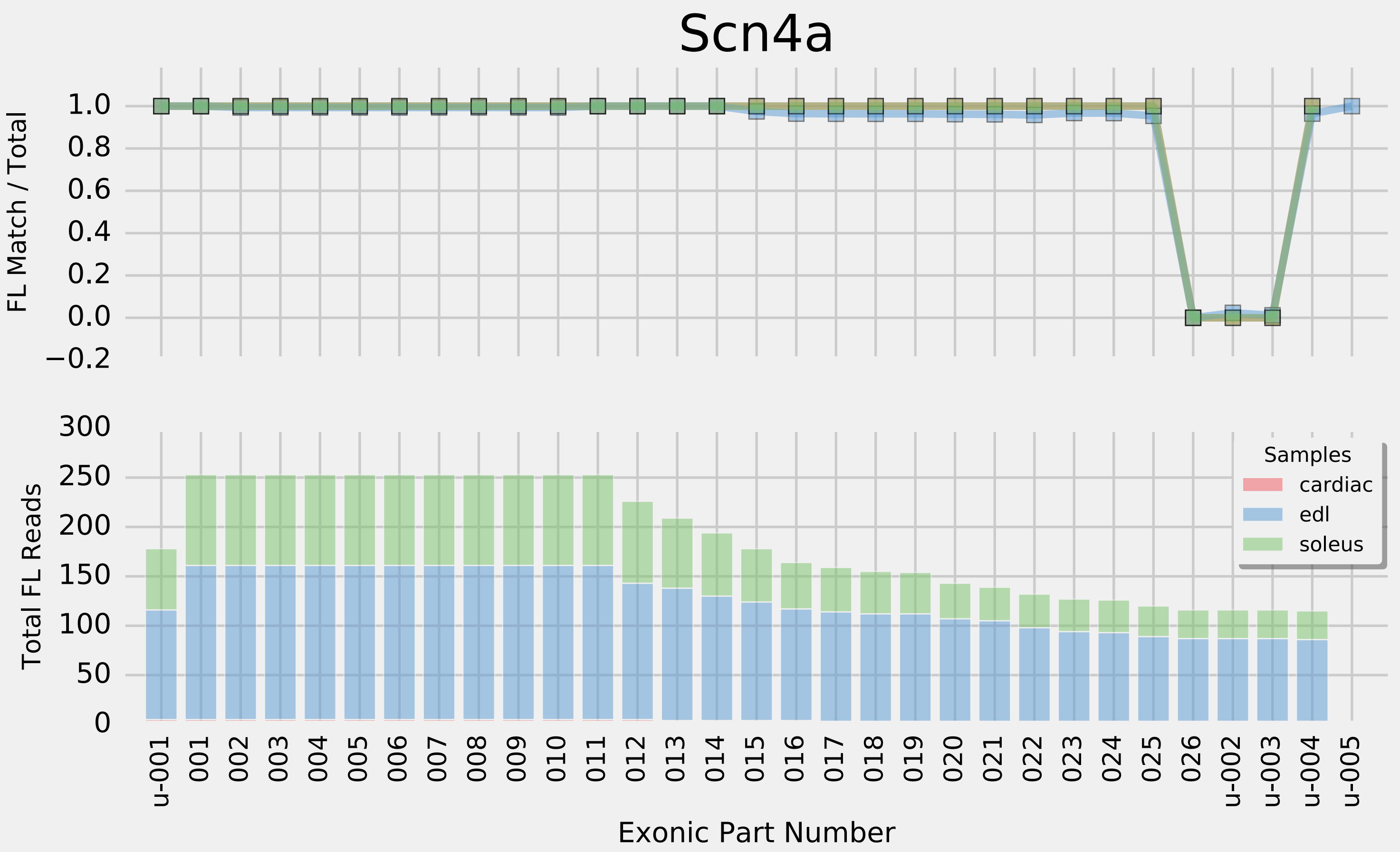

Scn4a
FL Match / Total
1.0
0.8
0.6
0.4
0.2
0.0
−0.2
Total FL Reads
300
250
200
150
100
50
0
Samples
cardiac
edl
soleus
u-001
001
002
003
004
005
006
007
008
009
010
011
012
013
014
015
016
017
018
019
020
021
022
023
024
025
026
u-002
u-003
u-004
u-005
Exonic Part Number

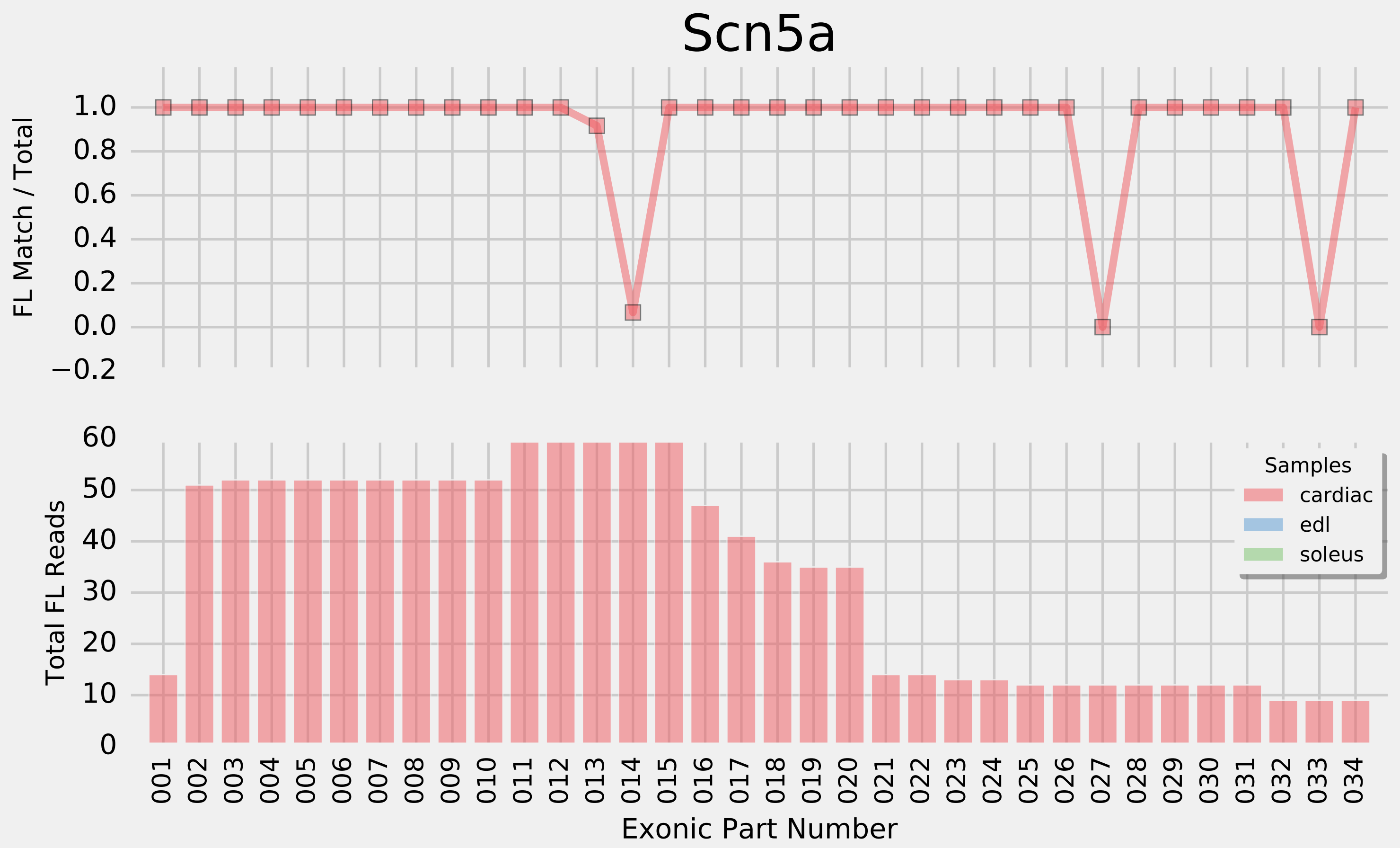

Scn5a
FL Match / Total
1.0
0.8
0.6
0.4
0.2
0.0
−0.2
Total FL Reads
60
50
40
30
20
10
0
Samples
cardiac
edl
soleus
001
002
003
004
005
006
007
008
009
010
011
012
013
014
015
016
017
018
019
020
021
022
023
024
025
026
027
028
029
030
031
032
033
034
Exonic Part Number

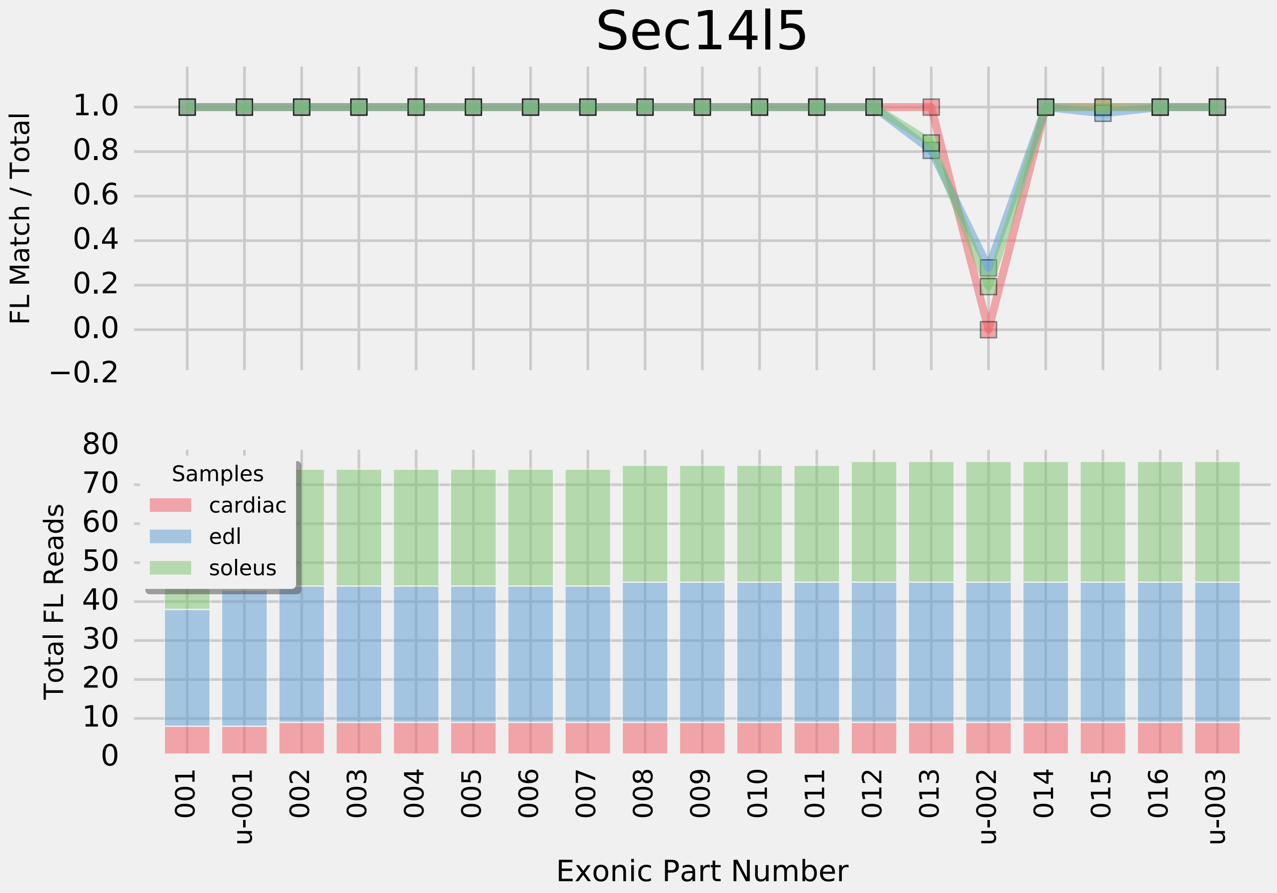

Sec14l5
FL Match / Total
1.0
0.8
0.6
0.4
0.2
0.0
−0.2
Total FL Reads
80
70
60
50
40
30
20
10
0
Samples
cardiac
edl
soleus
001
u-001
002
003
004
005
006
007
008
009
010
011
012
013
u-002
014
015
016
u-003
Exonic Part Number

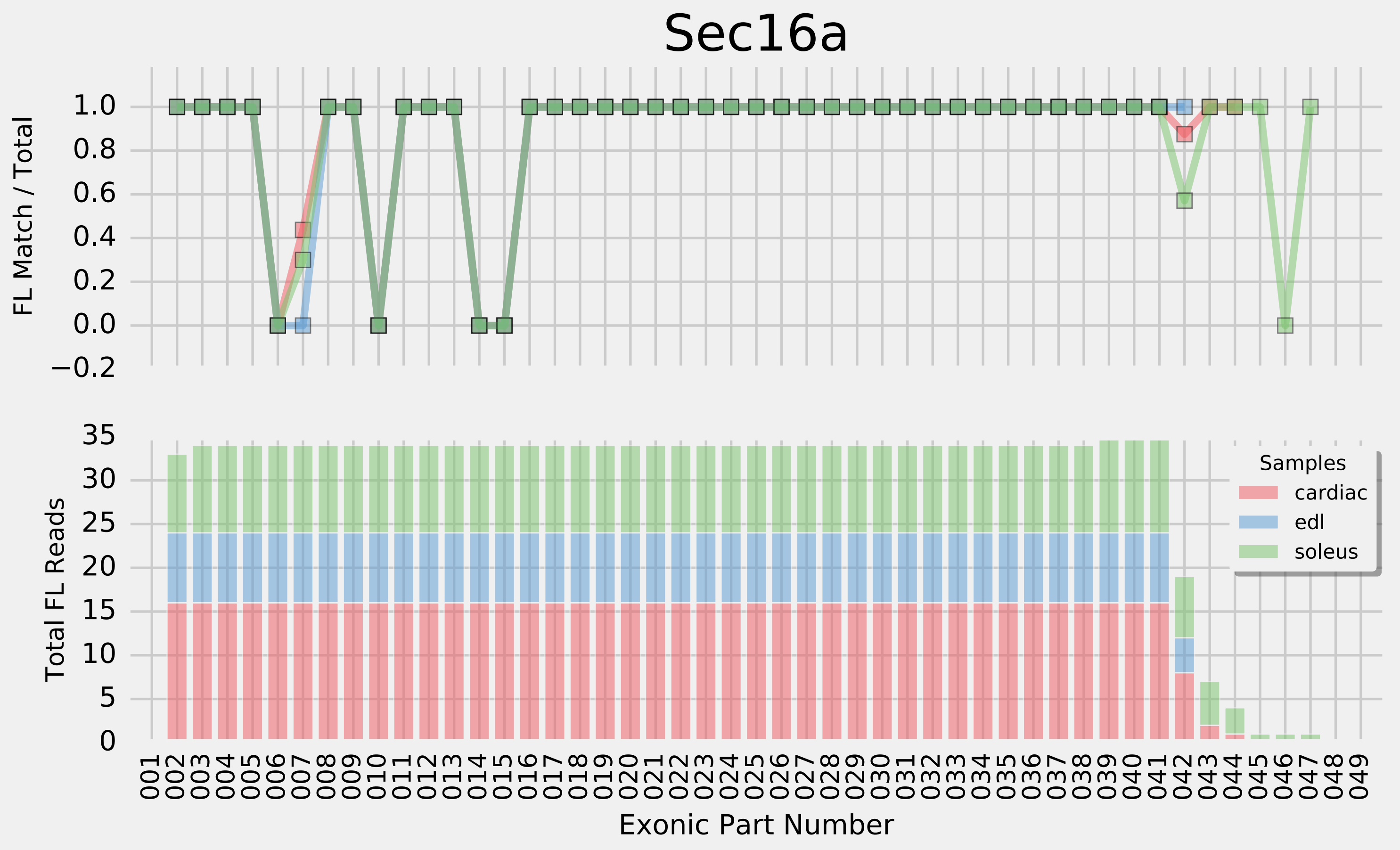

Sec16a
FL Match / Total
Total FL Reads
Exonic Part Number
Samples
cardiac
edl
soleus

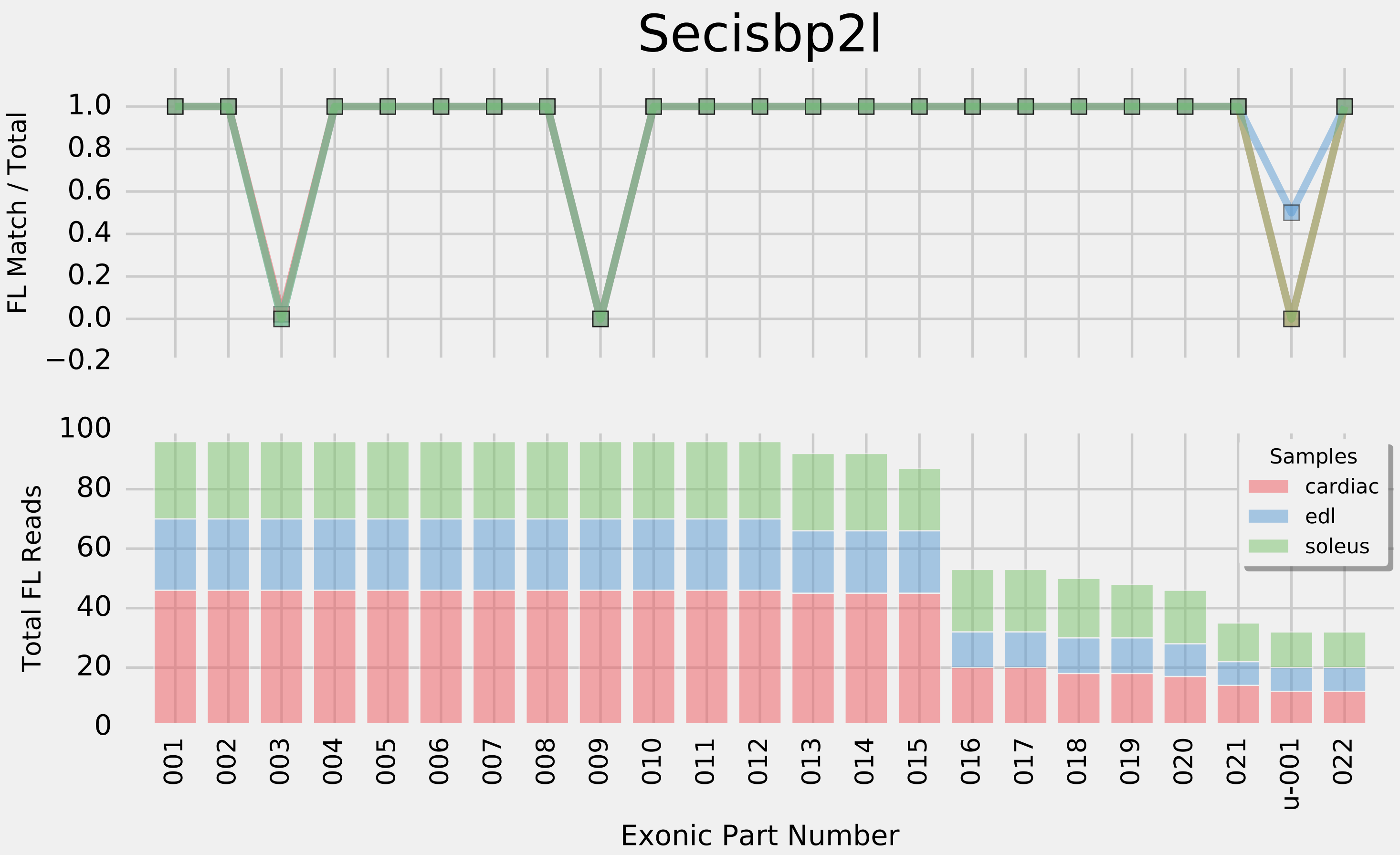

Secisbp2l
FL Match / Total
1.0
0.8
0.6
0.4
0.2
0.0
−0.2
100
80
60
40
20
0
Total FL Reads
Samples
cardiac
edl
soleus
001
002
003
004
005
006
007
008
009
010
011
012
013
014
015
016
017
018
019
020
021
u-001
022
Exonic Part Number

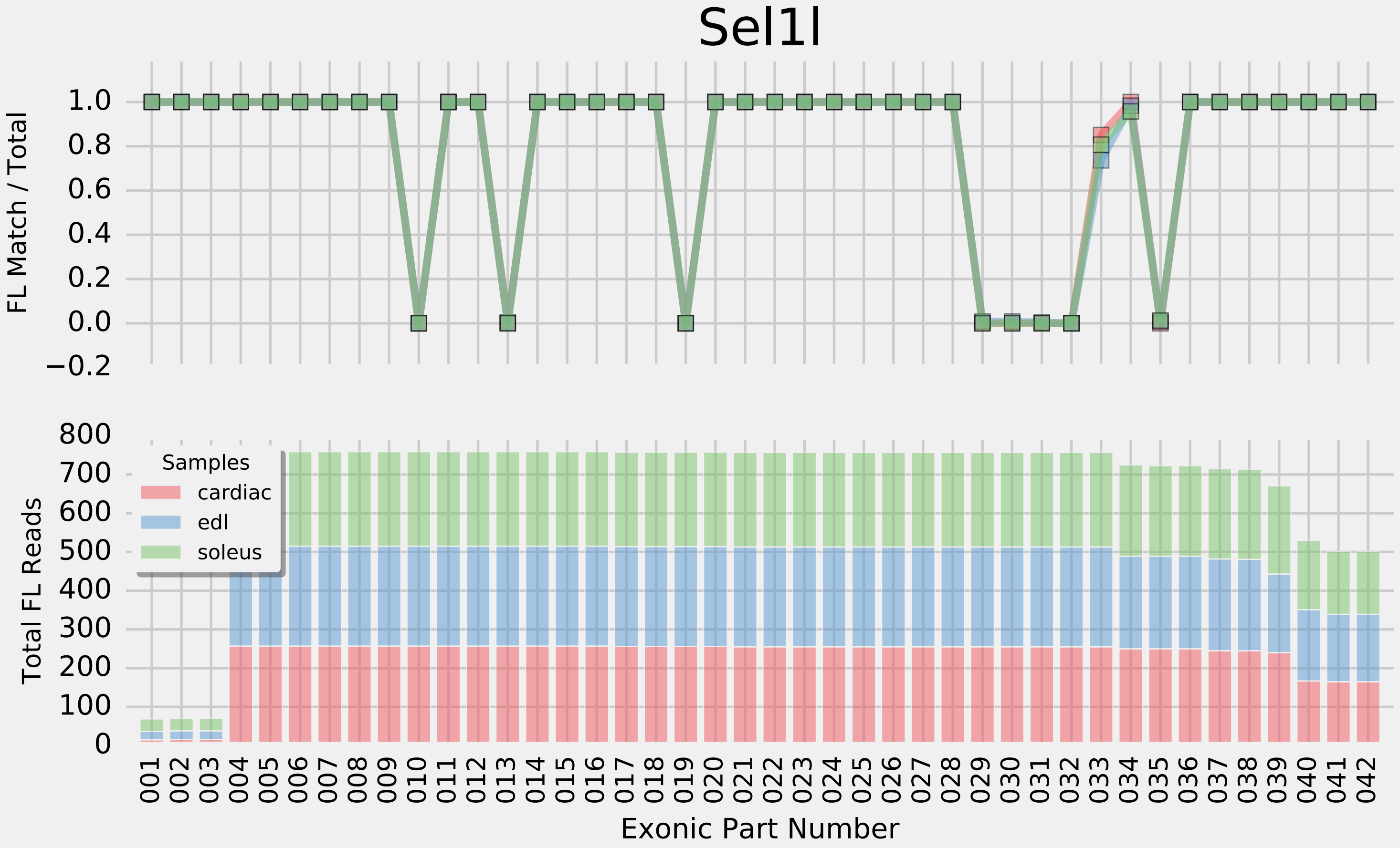

Sel1l
FL Match / Total
1.0
0.8
0.6
0.4
0.2
0.0
−0.2
Total FL Reads
800
700
600
500
400
300
200
100
0
Samples
cardiac
edl
soleus
001 002 003 004 005 006 007 008 009 010 011 012 013 014 015 016 017 018 019 020 021 022 023 024 025 026 027 028 029 030 031 032 033 034 035 036 037 038 039 040 041 042
Exonic Part Number

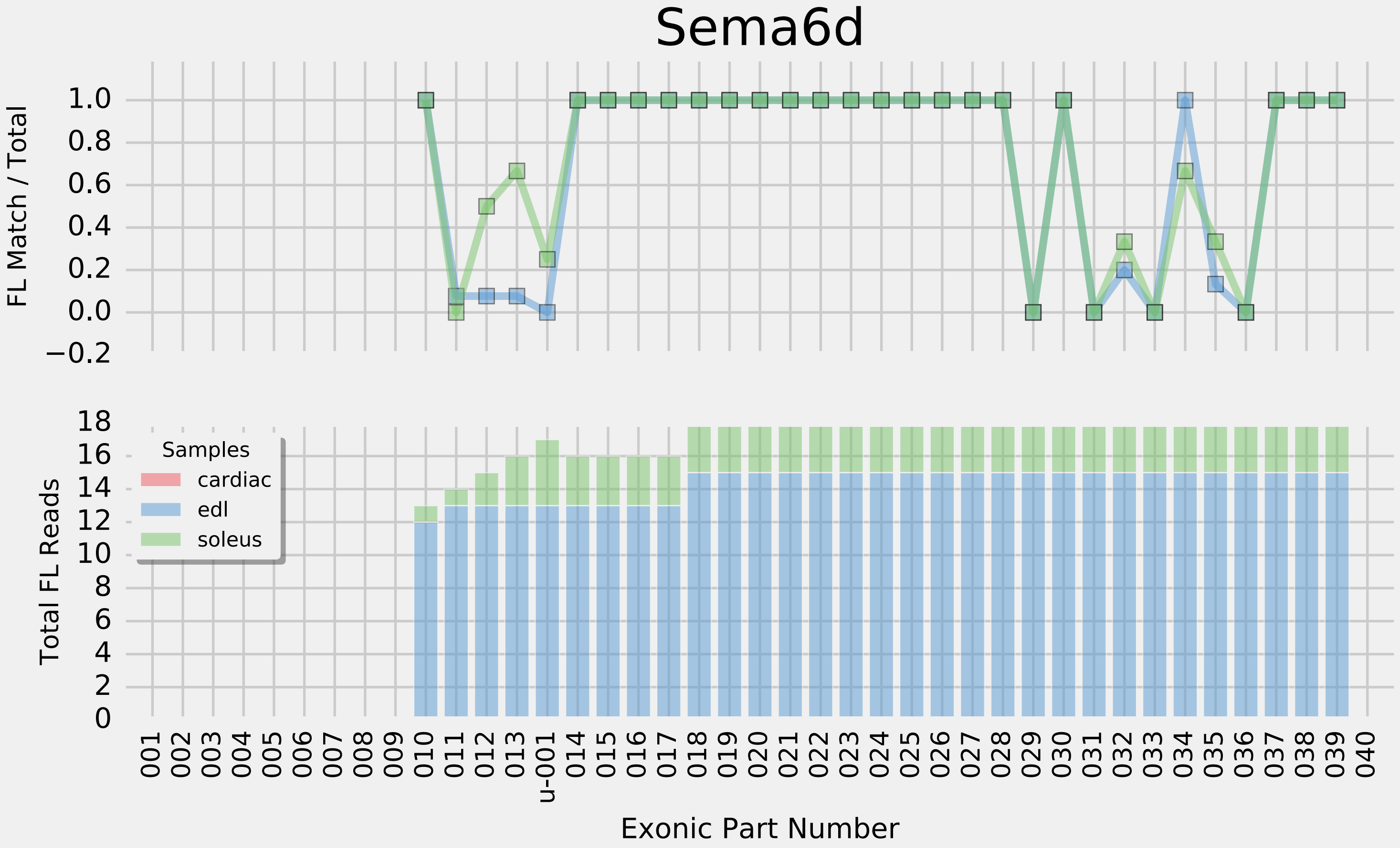

Sema6d
FL Match / Total
1.0
0.8
0.6
0.4
0.2
0.0
−0.2
Total FL Reads
18
16
14
12
10
8
6
4
2
0
Samples
cardiac
edl
soleus
001
002
003
004
005
006
007
008
009
010
011
012
013
u-001
014
015
016
017
018
019
020
021
022
023
024
025
026
027
028
029
030
031
032
033
034
035
036
037
038
039
040
Exonic Part Number

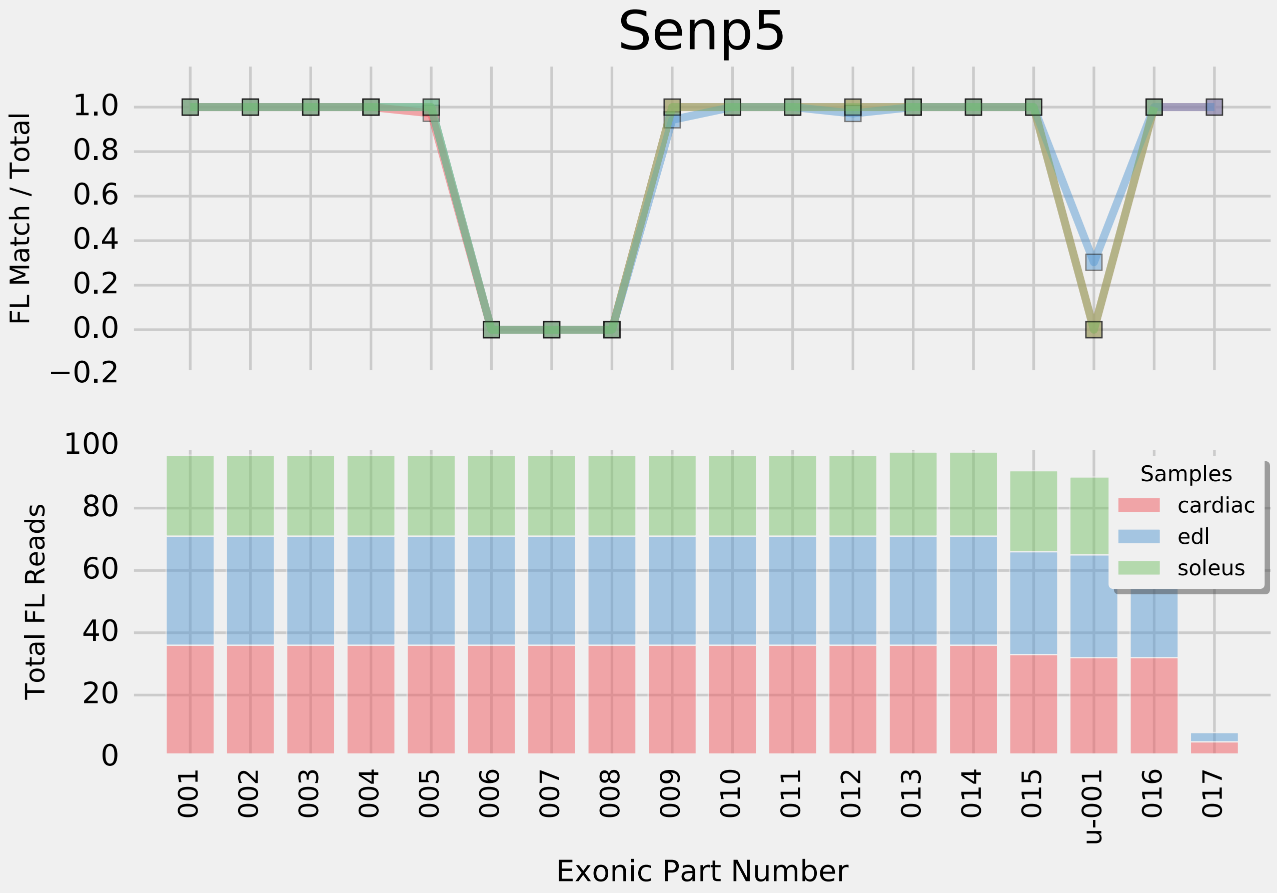
Senp5
FL Match / Total
1.0
0.8
0.6
0.4
0.2
0.0
−0.2
Total FL Reads
100
80
60
40
20
0
Samples
cardiac
edl
soleus
001
002
003
004
005
006
007
008
009
010
011
012
013
014
015
u-001
016
017
Exonic Part Number

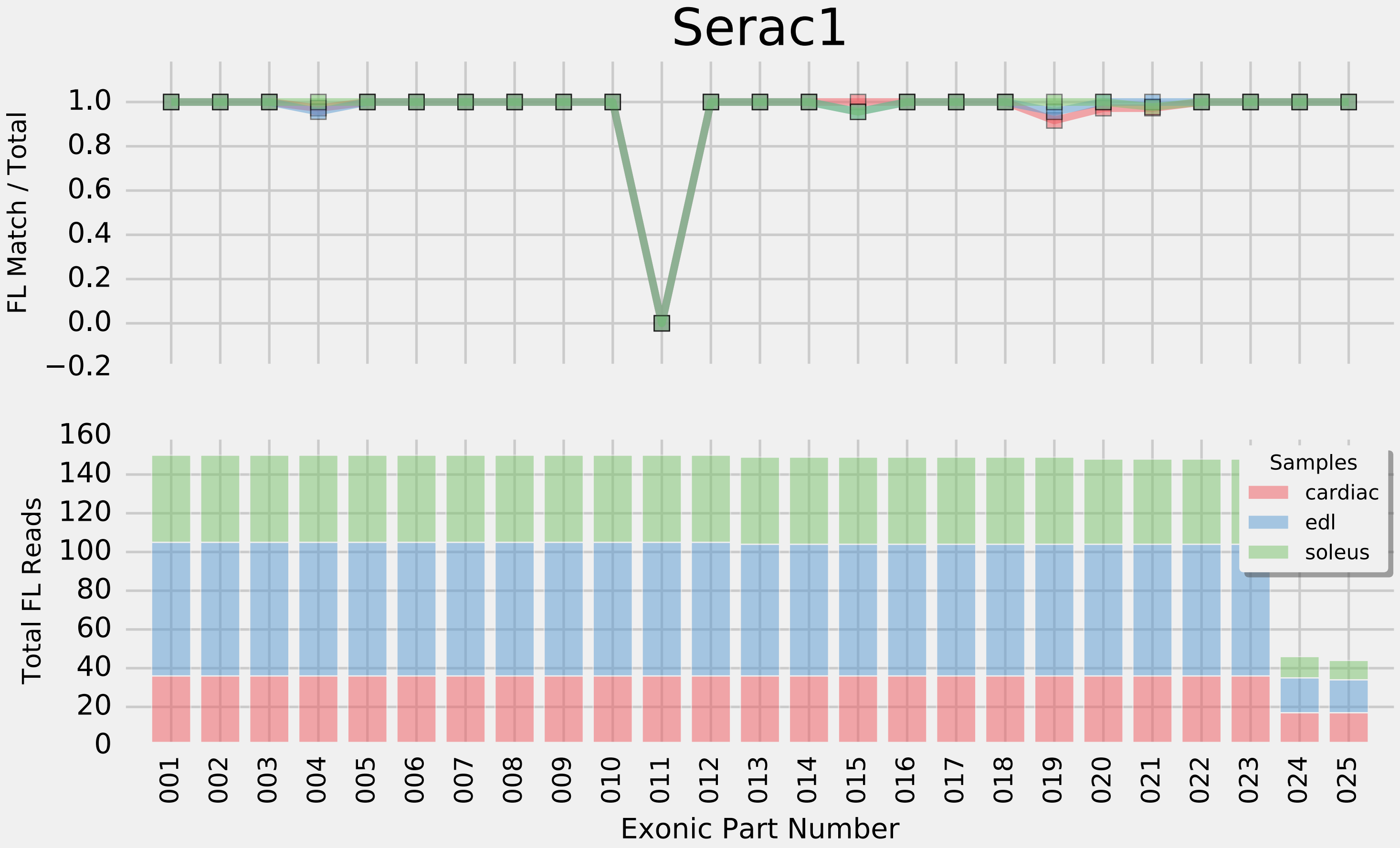

Serac1
FL Match / Total
1.0
0.8
0.6
0.4
0.2
0.0
−0.2
Total FL Reads
160
140
120
100
80
60
40
20
0
Samples
cardiac
edl
soleus
001
002
003
004
005
006
007
008
009
010
011
012
013
014
015
016
017
018
019
020
021
022
023
024
025
Exonic Part Number

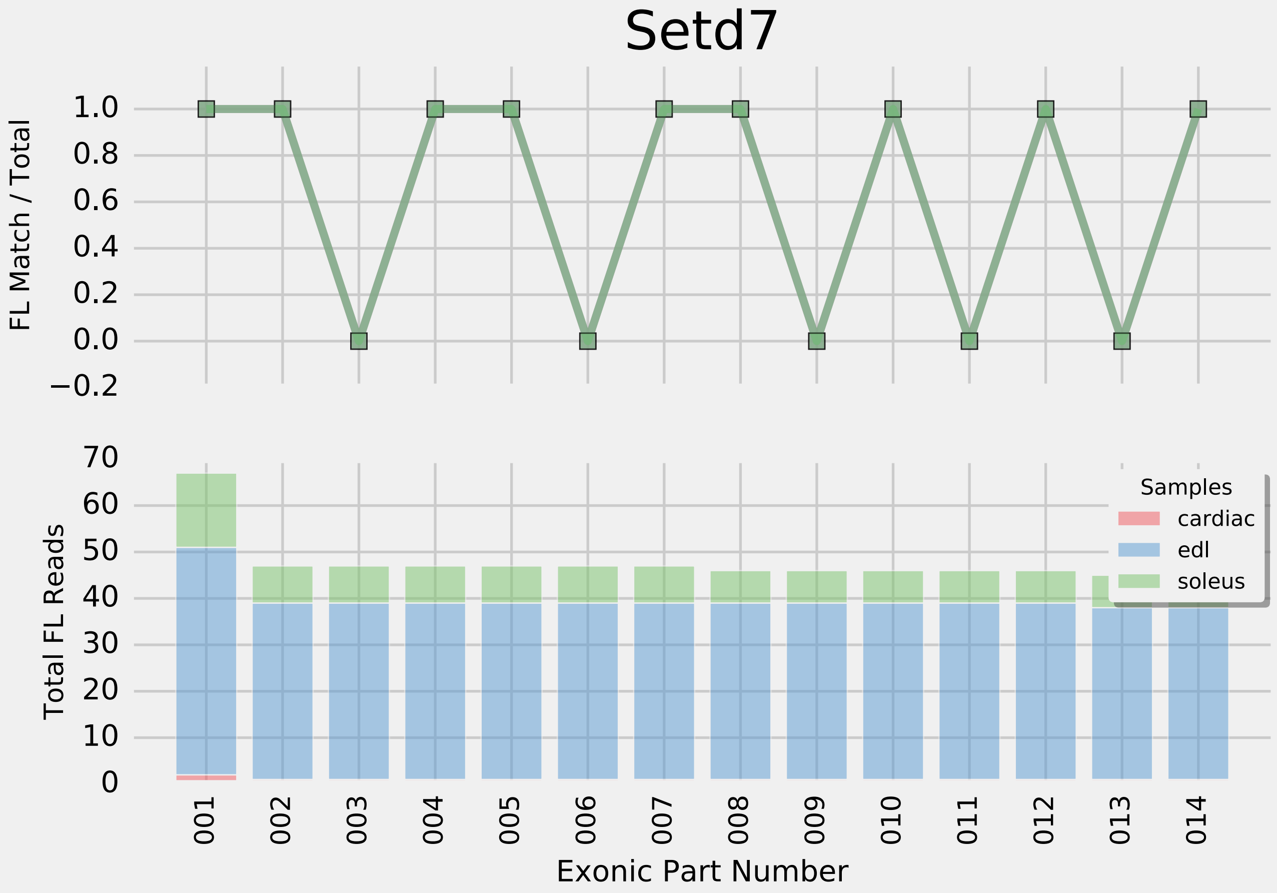
Setd7
FL Match / Total
1.0
0.8
0.6
0.4
0.2
0.0
−0.2
Total FL Reads
70
60
50
40
30
20
10
0
Samples
cardiac
edl
soleus
001
002
003
004
005
006
007
008
009
010
011
012
013
014
Exonic Part Number

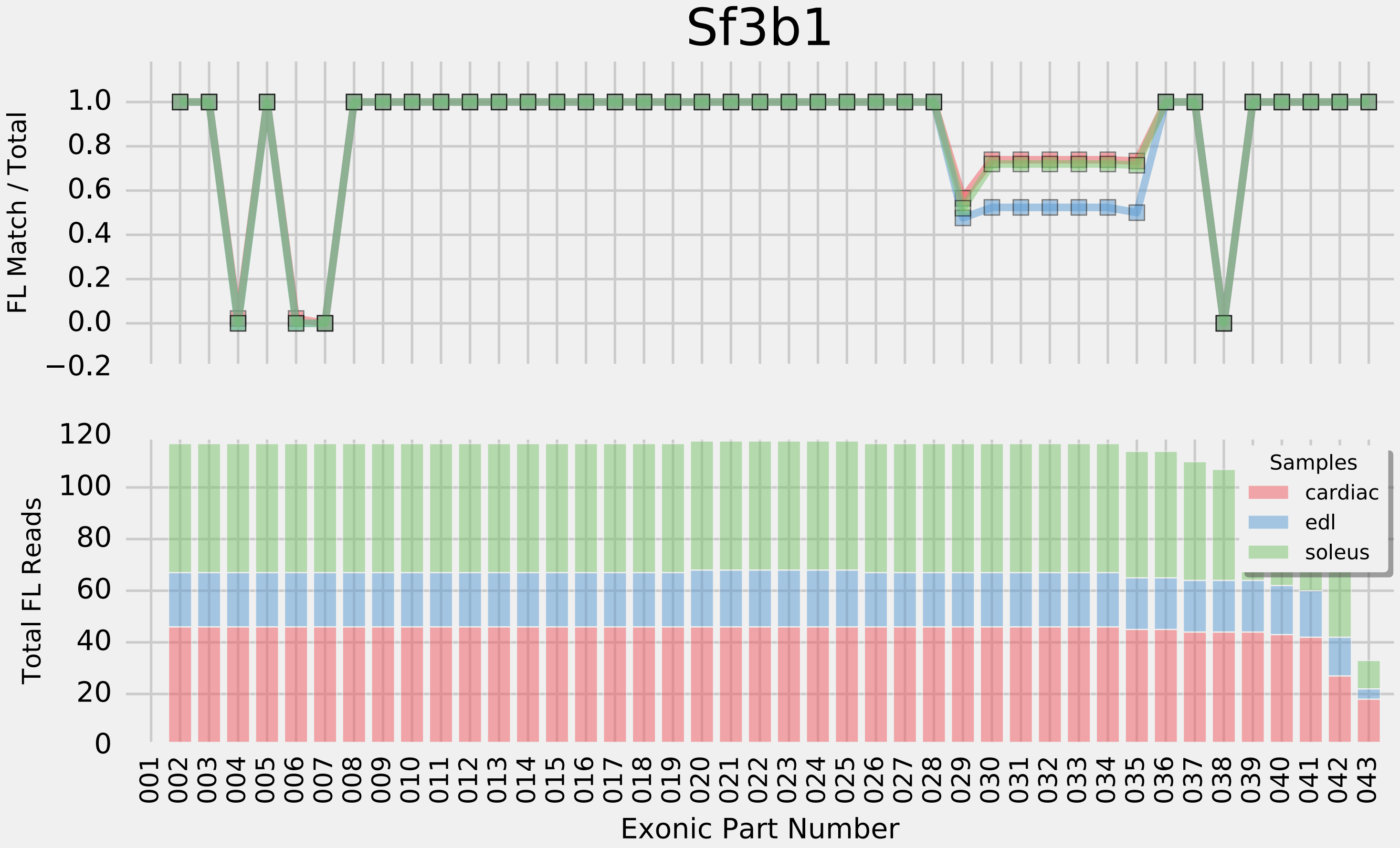
Sf3b1
FL Match / Total
1.0
0.8
0.6
0.4
0.2
0.0
−0.2
Total FL Reads
120
100
80
60
40
20
0
Samples
cardiac
edl
soleus
001 002 003 004 005 006 007 008 009 010 011 012 013 014 015 016 017 018 019 020 021 022 023 024 025 026 027 028 029 030 031 032 033 034 035 036 037 038 039 040 041 042 043
Exonic Part Number

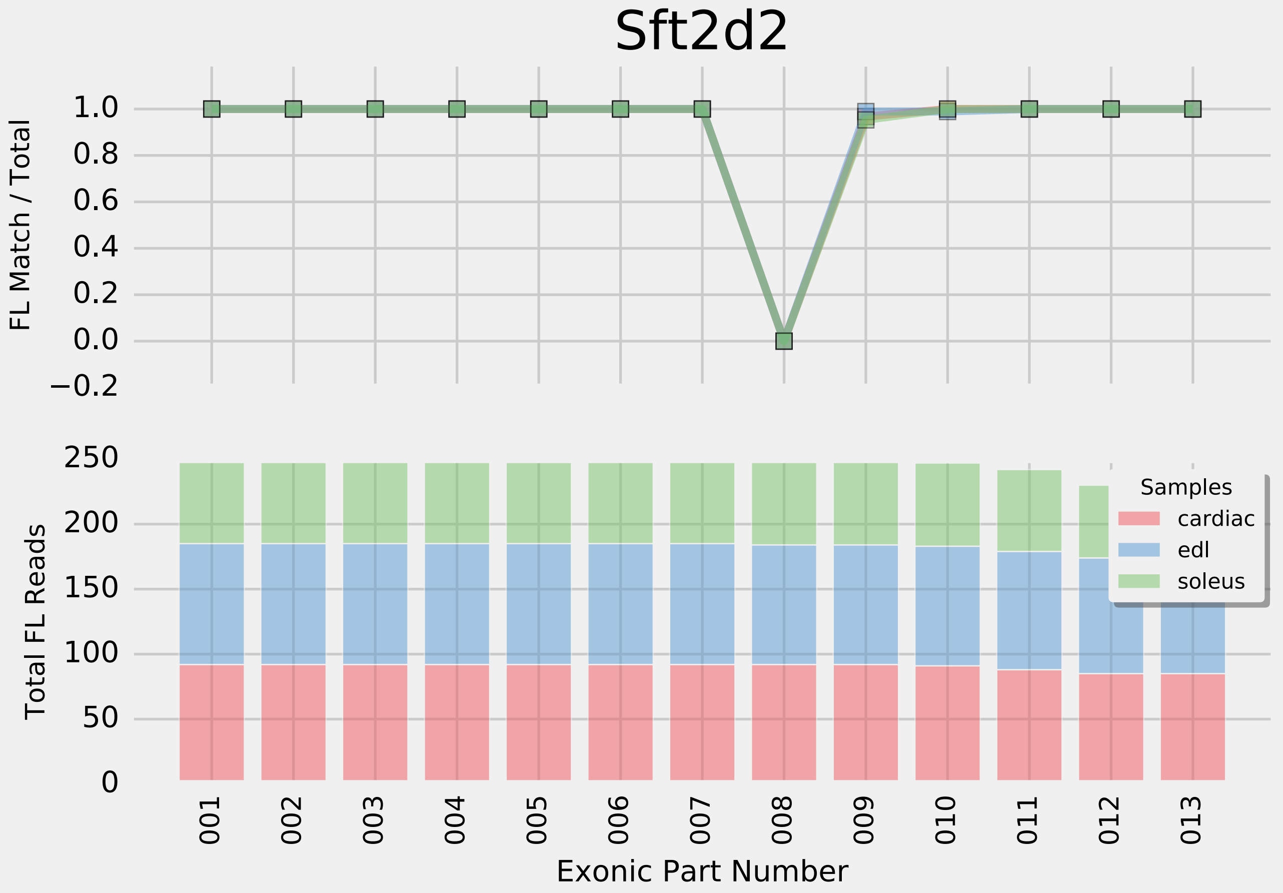
Sft2d2
FL Match / Total
1.0
0.8
0.6
0.4
0.2
0.0
−0.2
Total FL Reads
250
200
150
100
50
0
Samples
cardiac
edl
soleus
001
002
003
004
005
006
007
008
009
010
011
012
013
Exonic Part Number

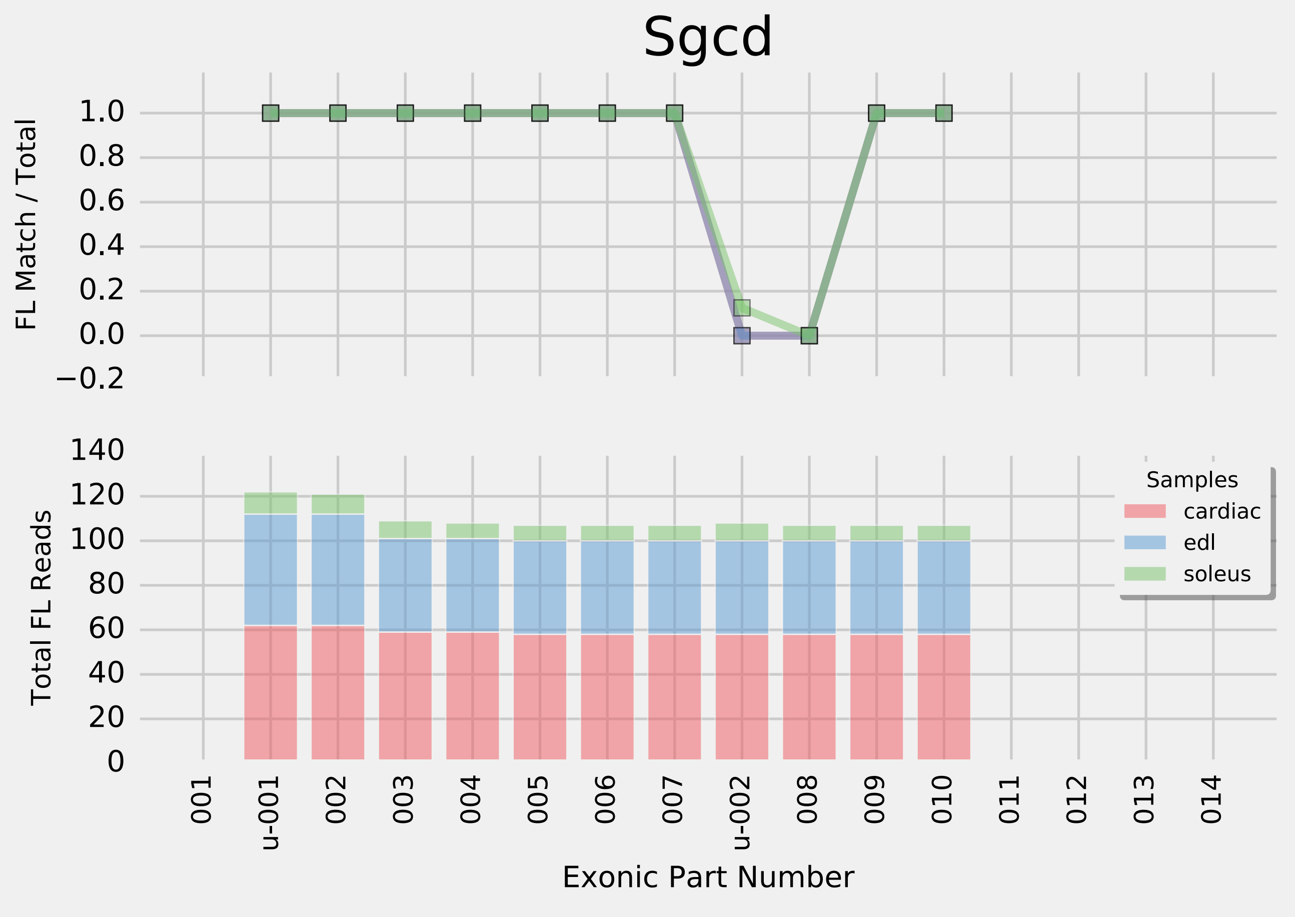
Sgcd
FL Match / Total
1.0
0.8
0.6
0.4
0.2
0.0
−0.2
Total FL Reads
140
120
100
80
60
40
20
0
Samples
cardiac
edl
soleus
001
u-001
002
003
004
005
006
007
u-002
008
009
010
011
012
013
014
Exonic Part Number

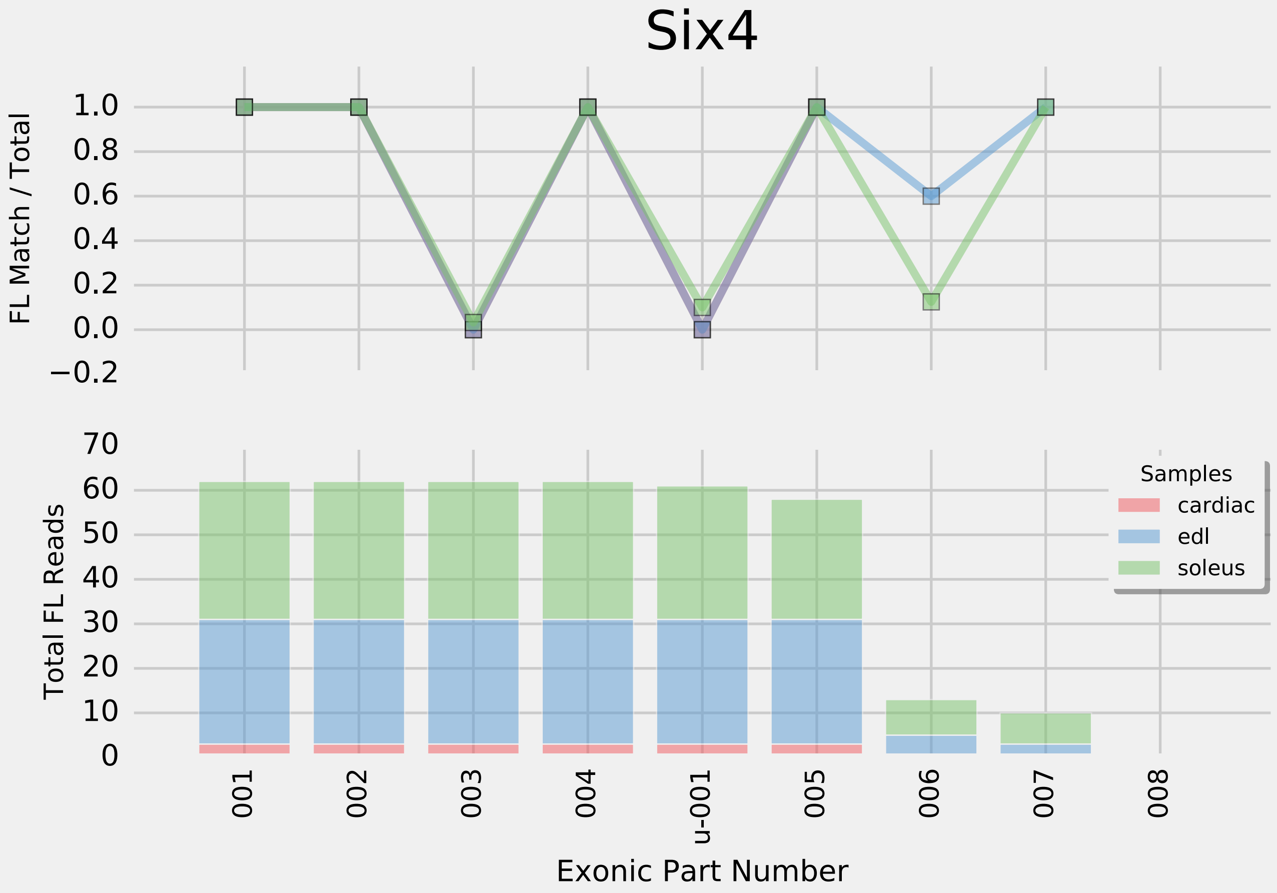

Six4
FL Match / Total
1.0
0.8
0.6
0.4
0.2
0.0
−0.2
Total FL Reads
70
60
50
40
30
20
10
0
Samples
cardiac
edl
soleus
001
002
003
004
u-001
005
006
007
008
Exonic Part Number

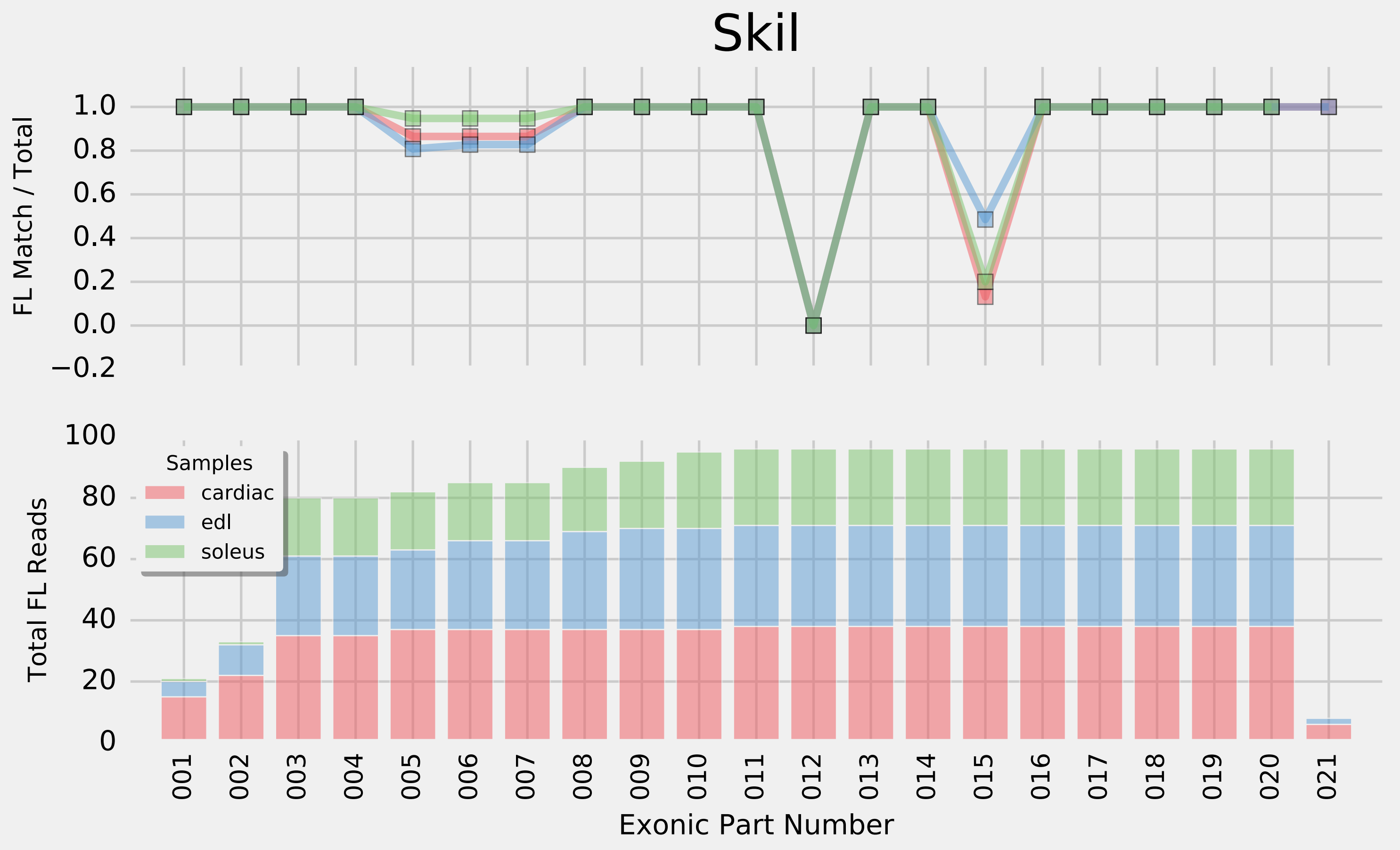

Skil
FL Match / Total
1.0
0.8
0.6
0.4
0.2
0.0
−0.2
Total FL Reads
100
80
60
40
20
0
Samples
cardiac
edl
soleus
001
002
003
004
005
006
007
008
009
010
011
012
013
014
015
016
017
018
019
020
021
Exonic Part Number

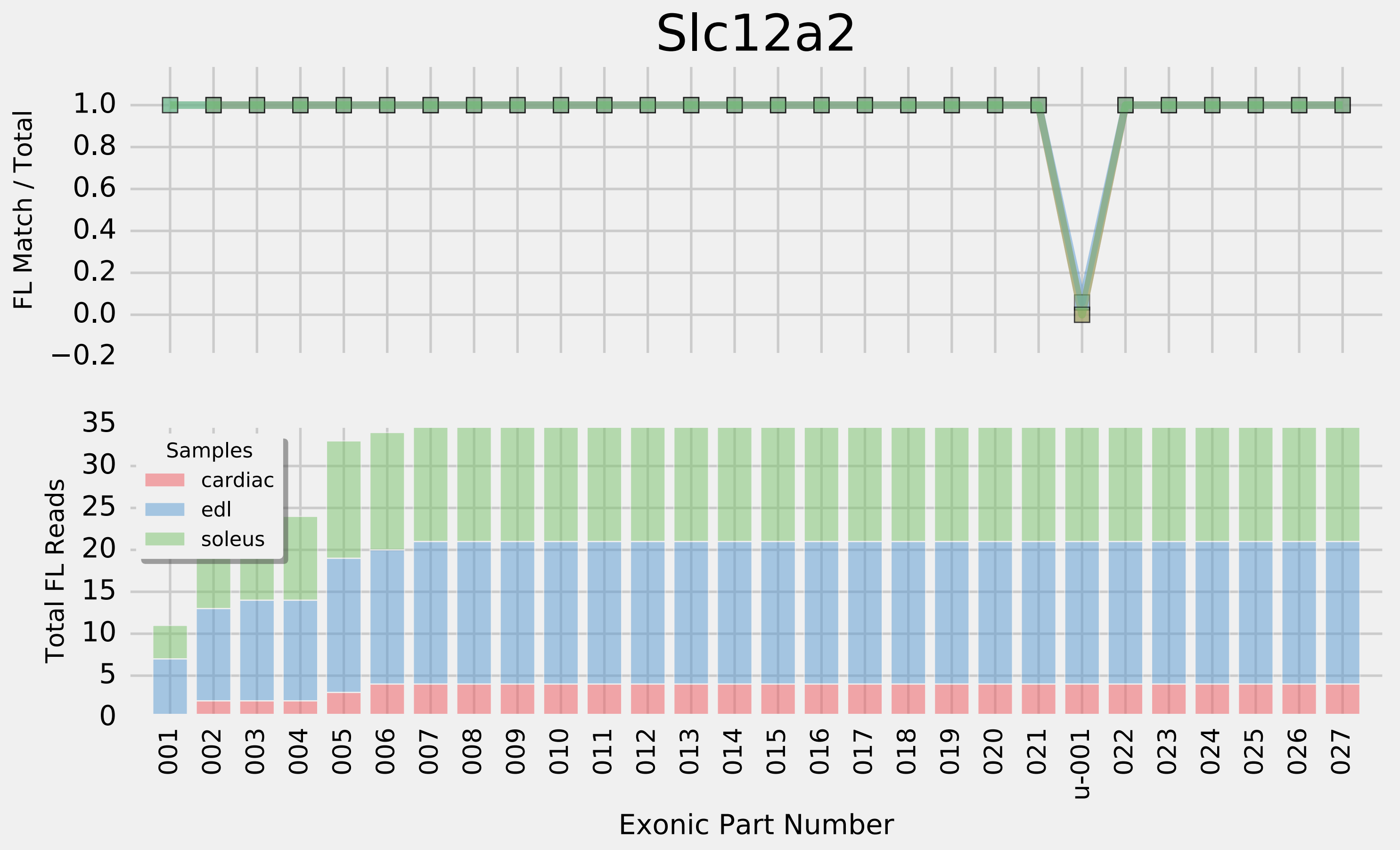

Slc12a2
FL Match / Total
1.0
0.8
0.6
0.4
0.2
0.0
−0.2
Total FL Reads
35
30
25
20
15
10
5
0
Samples
cardiac
edl
soleus
001
002
003
004
005
006
007
008
009
010
011
012
013
014
015
016
017
018
019
020
021
u-001
022
023
024
025
026
027
Exonic Part Number

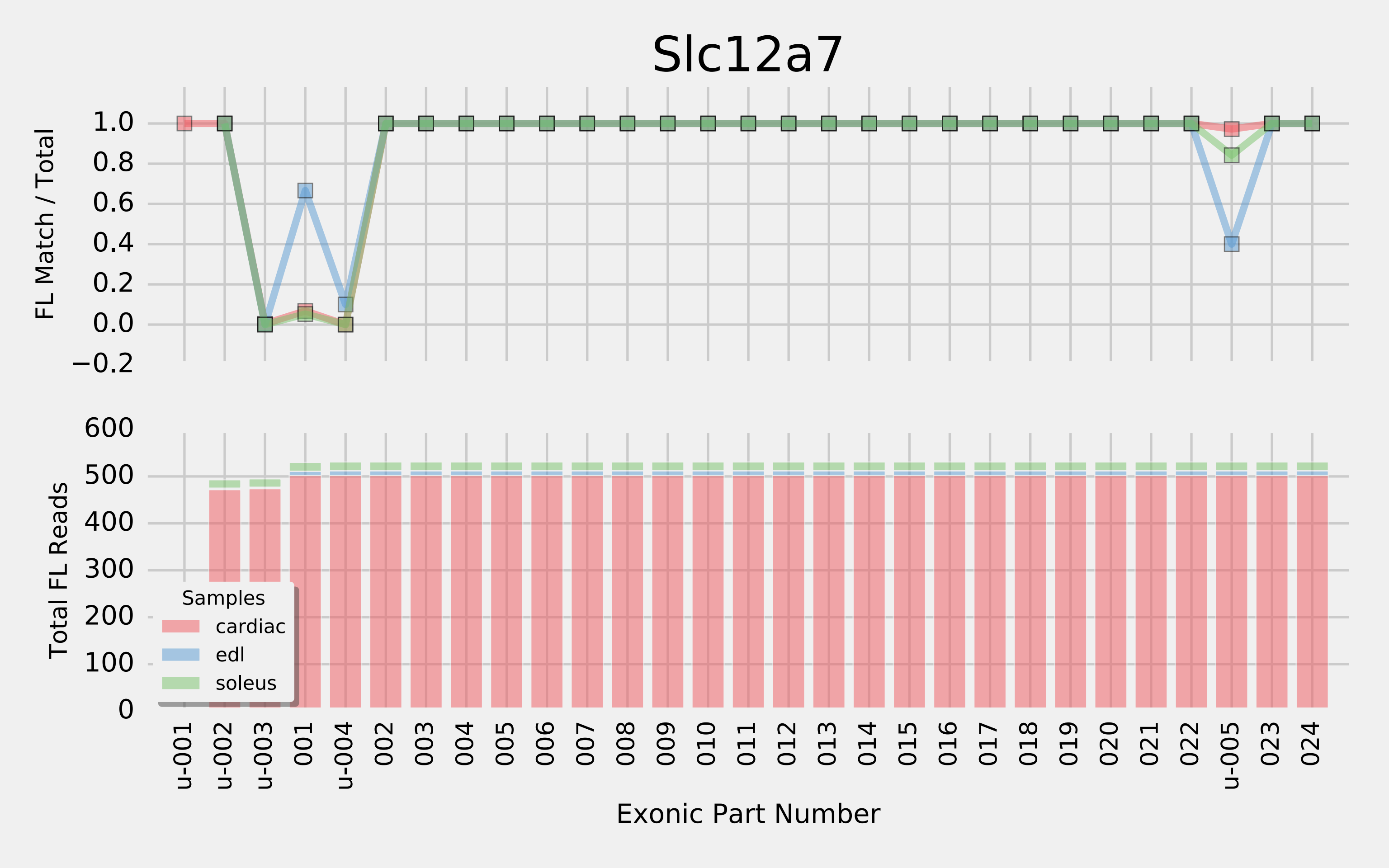

Slc12a7
FL Match / Total
1.0
0.8
0.6
0.4
0.2
0.0
−0.2
Total FL Reads
600
500
400
300
200
100
0
Samples
cardiac
edl
soleus
u-001
u-002
u-003
001
u-004
002
003
004
005
006
007
008
009
010
011
012
013
014
015
016
017
018
019
020
021
022
u-005
023
024
Exonic Part Number

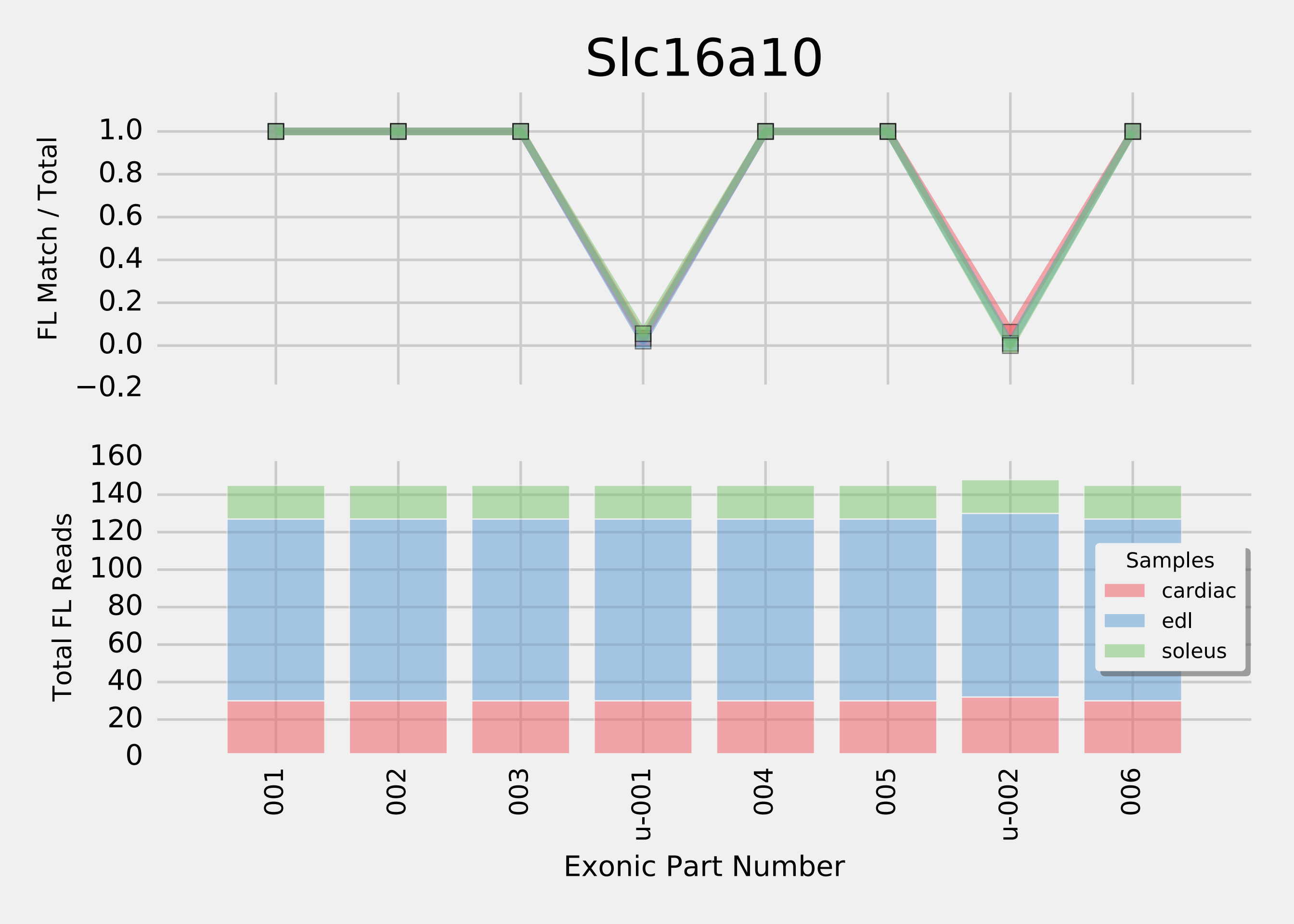
Slc16a10
FL Match / Total
1.0
0.8
0.6
0.4
0.2
0.0
−0.2
Total FL Reads
160
140
120
100
80
60
40
20
0
001
002
003
u-001
004
005
u-002
006
Exonic Part Number
Samples
cardiac
edl
soleus

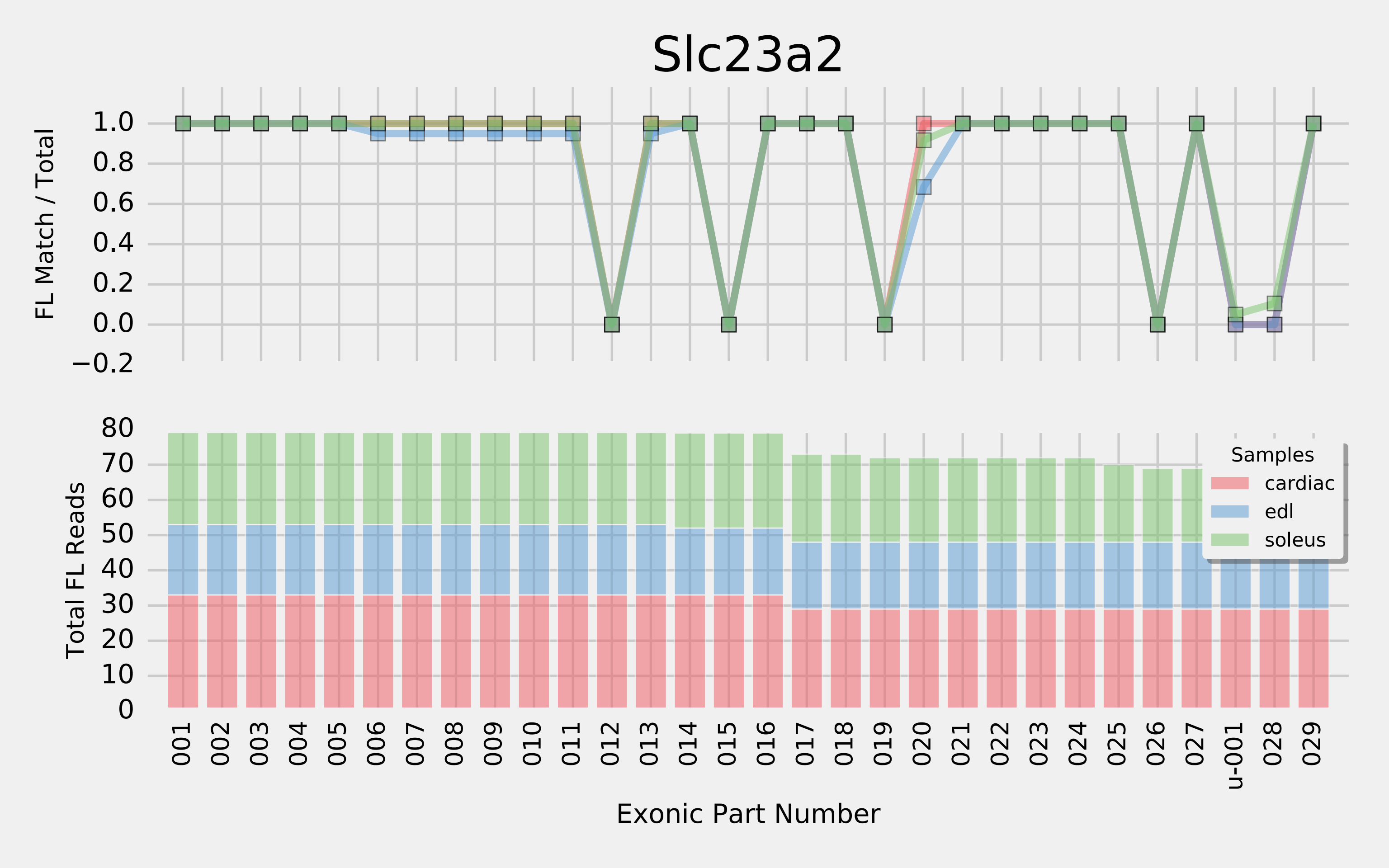
Slc23a2
FL Match / Total
1.0
0.8
0.6
0.4
0.2
0.0
−0.2
Total FL Reads
80
70
60
50
40
30
20
10
0
Samples
cardiac
edl
soleus
001
002
003
004
005
006
007
008
009
010
011
012
013
014
015
016
017
018
019
020
021
022
023
024
025
026
027
u-001
028
029
Exonic Part Number

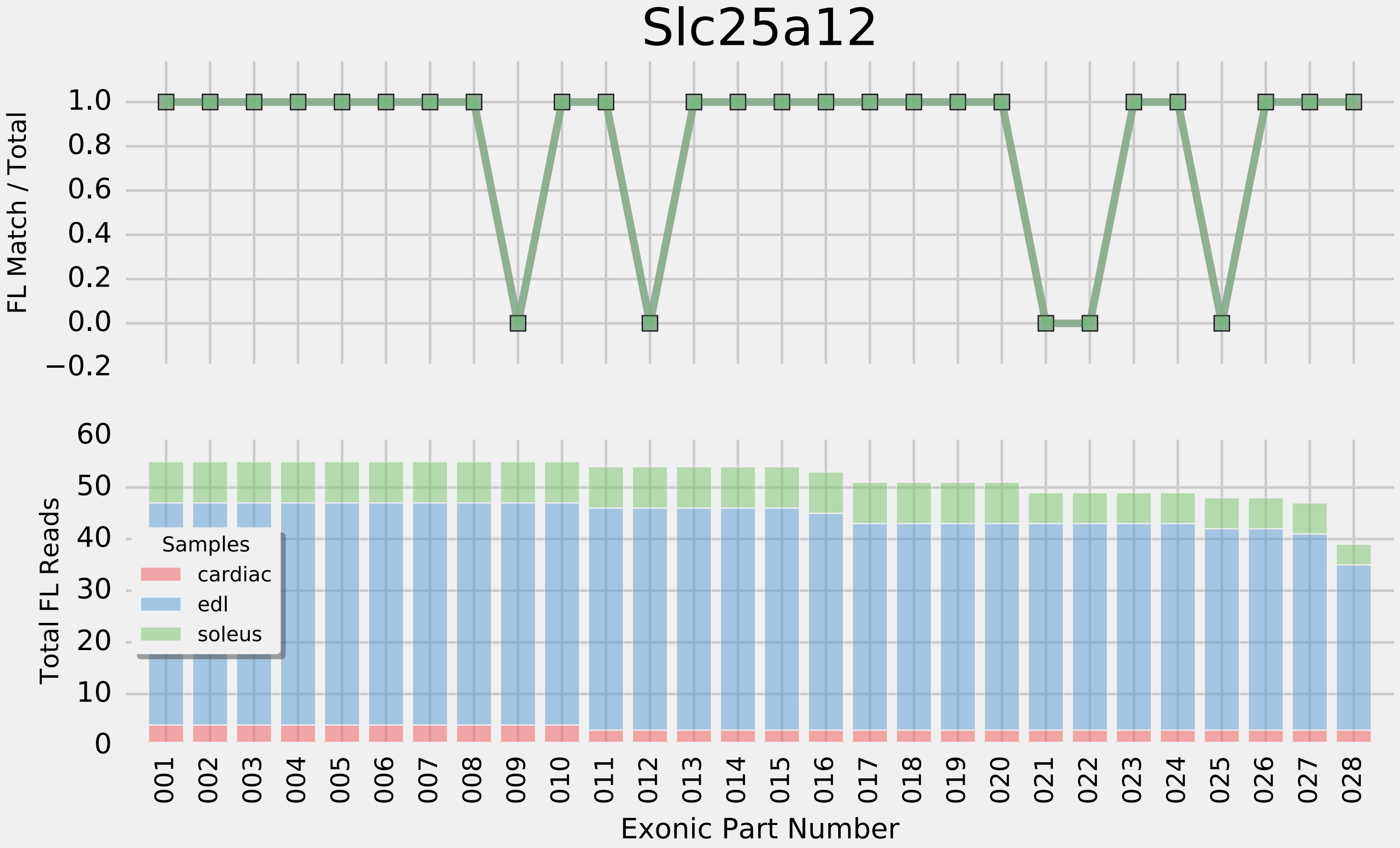

Slc25a12
FL Match / Total
1.0
0.8
0.6
0.4
0.2
0.0
−0.2
Total FL Reads
60
50
40
30
20
10
0
Samples
cardiac
edl
soleus
001
002
003
004
005
006
007
008
009
010
011
012
013
014
015
016
017
018
019
020
021
022
023
024
025
026
027
028
Exonic Part Number

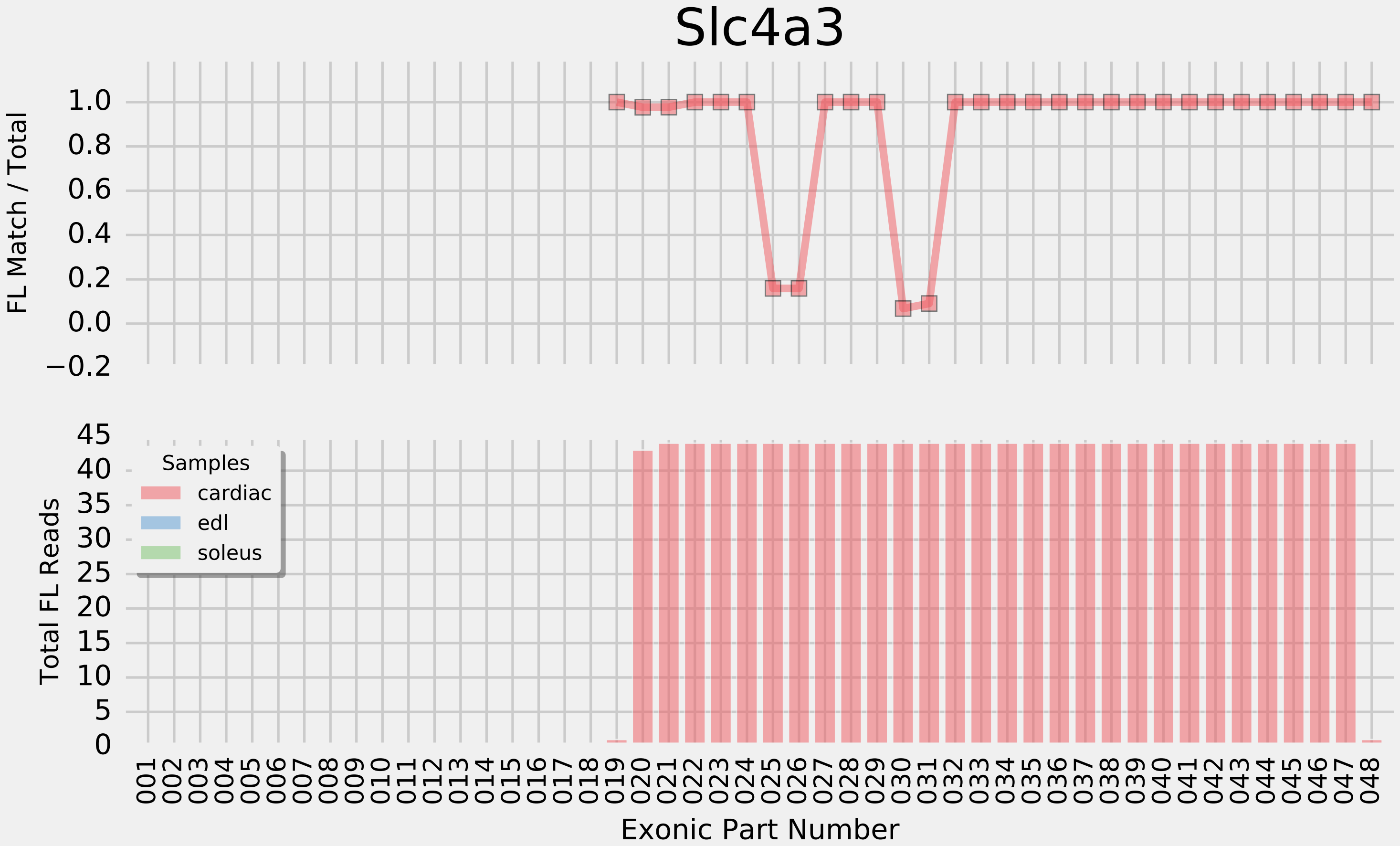

Slc4a3
FL Match / Total
1.0
0.8
0.6
0.4
0.2
0.0
−0.2
Total FL Reads
45
40
35
30
25
20
15
10
5
0
Samples
cardiac
edl
soleus
001 002 003 004 005 006 007 008 009 010 011 012 013 014 015 016 017 018 019 020 021 022 023 024 025 026 027 028 029 030 031 032 033 034 035 036 037 038 039 040 041 042 043 044 045 046 047 048
Exonic Part Number

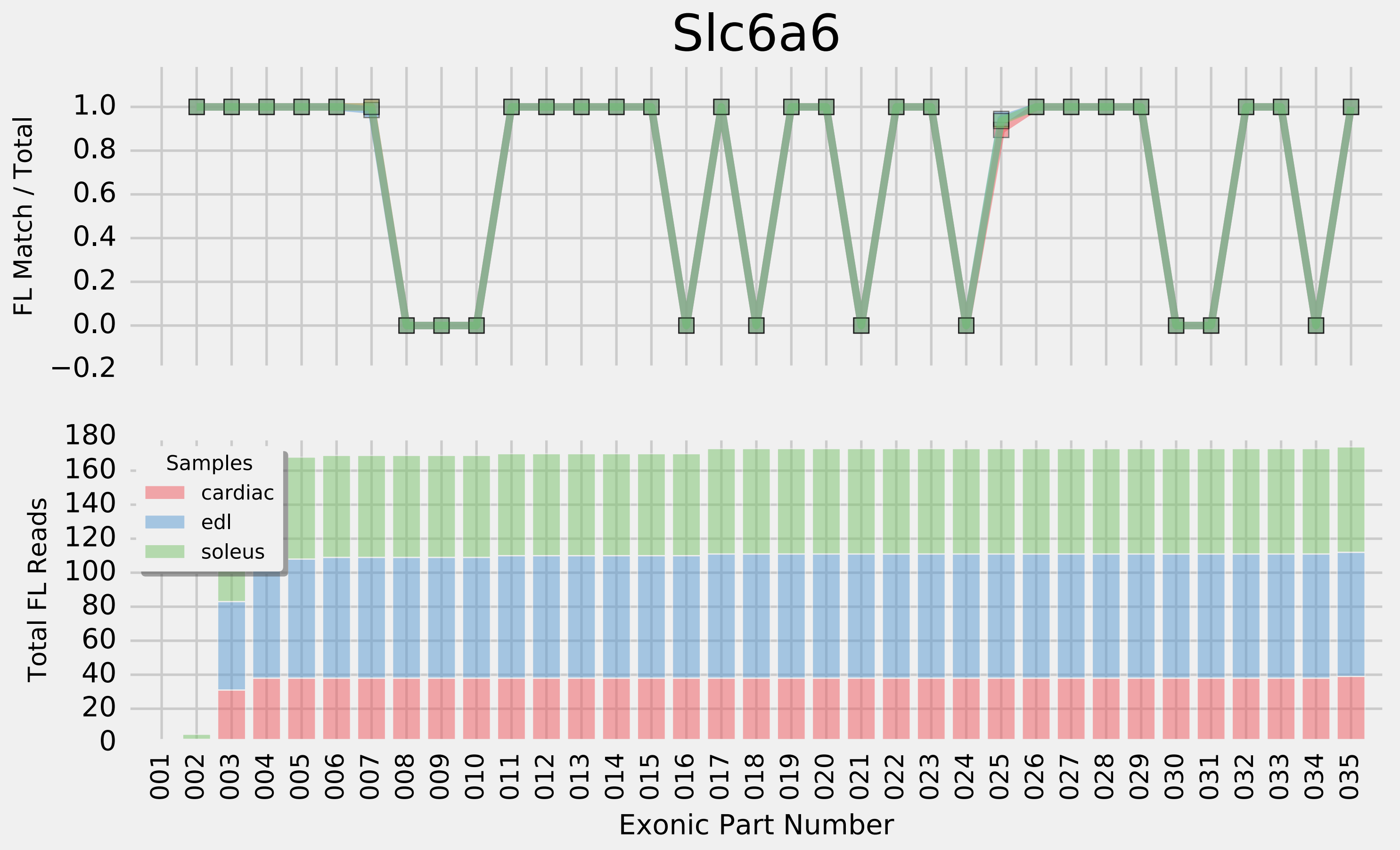
Slc6a6
FL Match / Total
1.0
0.8
0.6
0.4
0.2
0.0
−0.2
Total FL Reads
180
160
140
120
100
80
60
40
20
0
Samples
cardiac
edl
soleus
001
002
003
004
005
006
007
008
009
010
011
012
013
014
015
016
017
018
019
020
021
022
023
024
025
026
027
028
029
030
031
032
033
034
035
Exonic Part Number

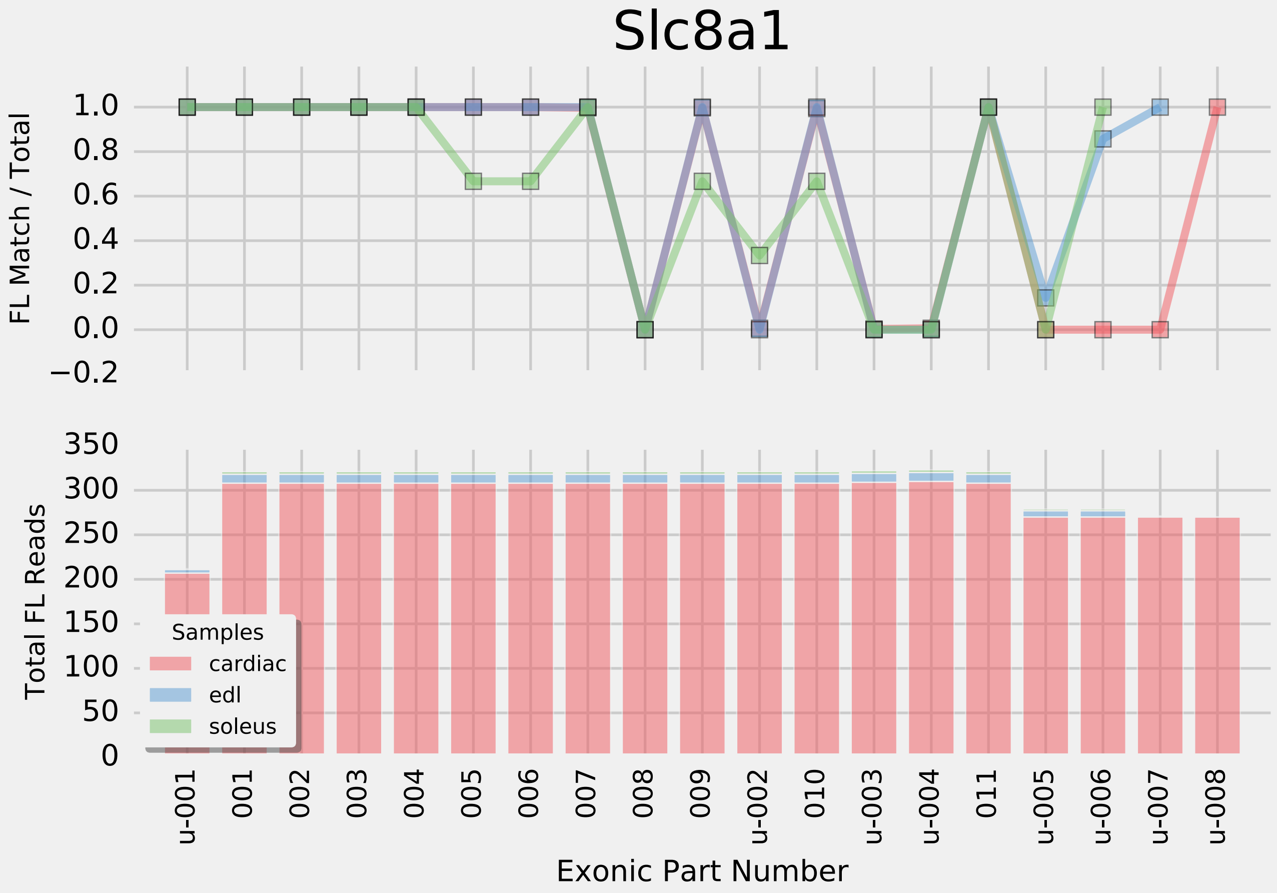
Slc8a1
FL Match / Total
1.0
0.8
0.6
0.4
0.2
0.0
−0.2
Total FL Reads
350
300
250
200
150
100
50
0
Samples
cardiac
edl
soleus
u-001
001
002
003
004
005
006
007
008
009
u-002
010
u-003
u-004
011
u-005
u-006
u-007
u-008
Exonic Part Number

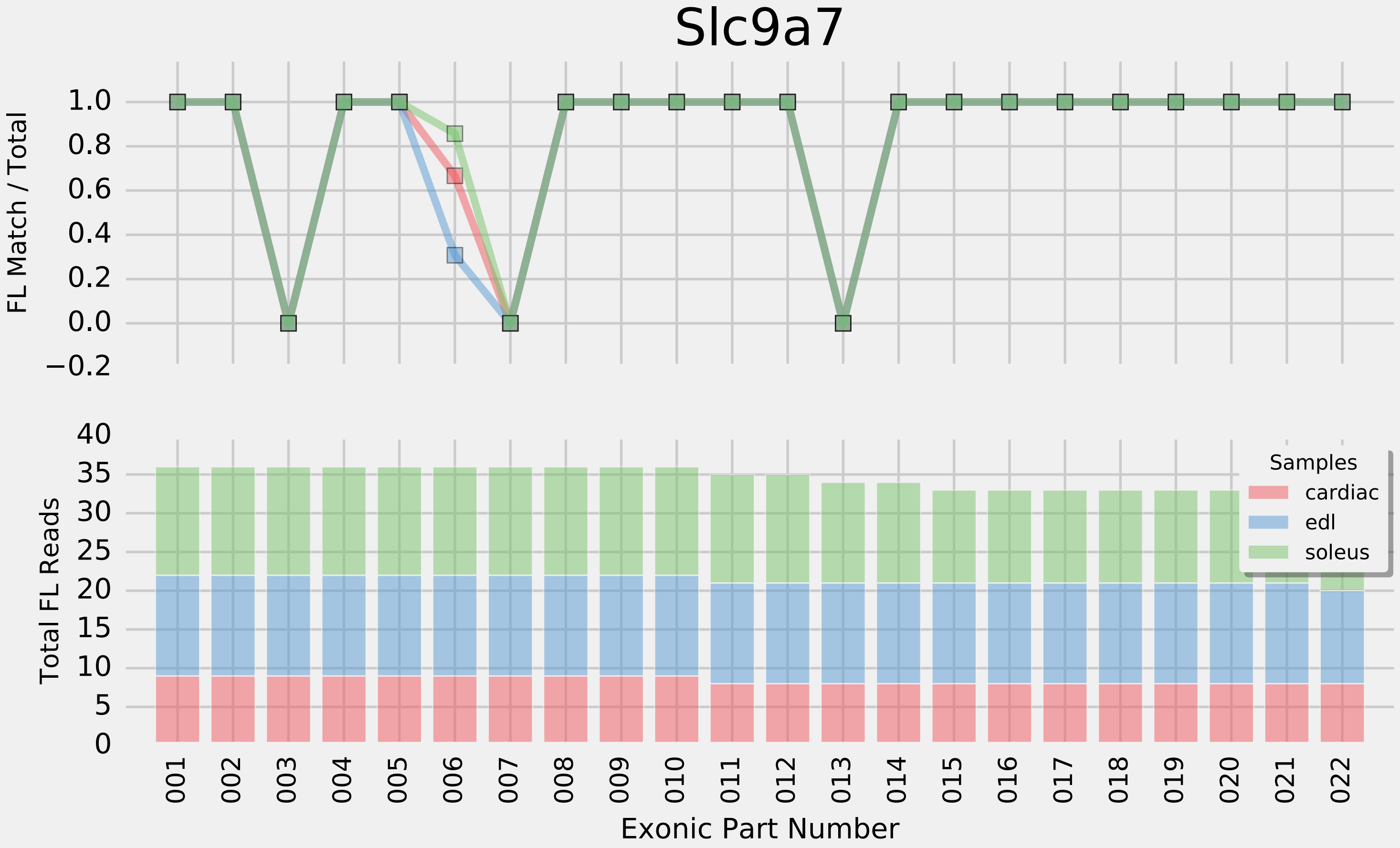

Slc9a7
FL Match / Total
1.0
0.8
0.6
0.4
0.2
0.0
−0.2
Total FL Reads
40
35
30
25
20
15
10
5
0
Samples
cardiac
edl
soleus
001
002
003
004
005
006
007
008
009
010
011
012
013
014
015
016
017
018
019
020
021
022
Exonic Part Number

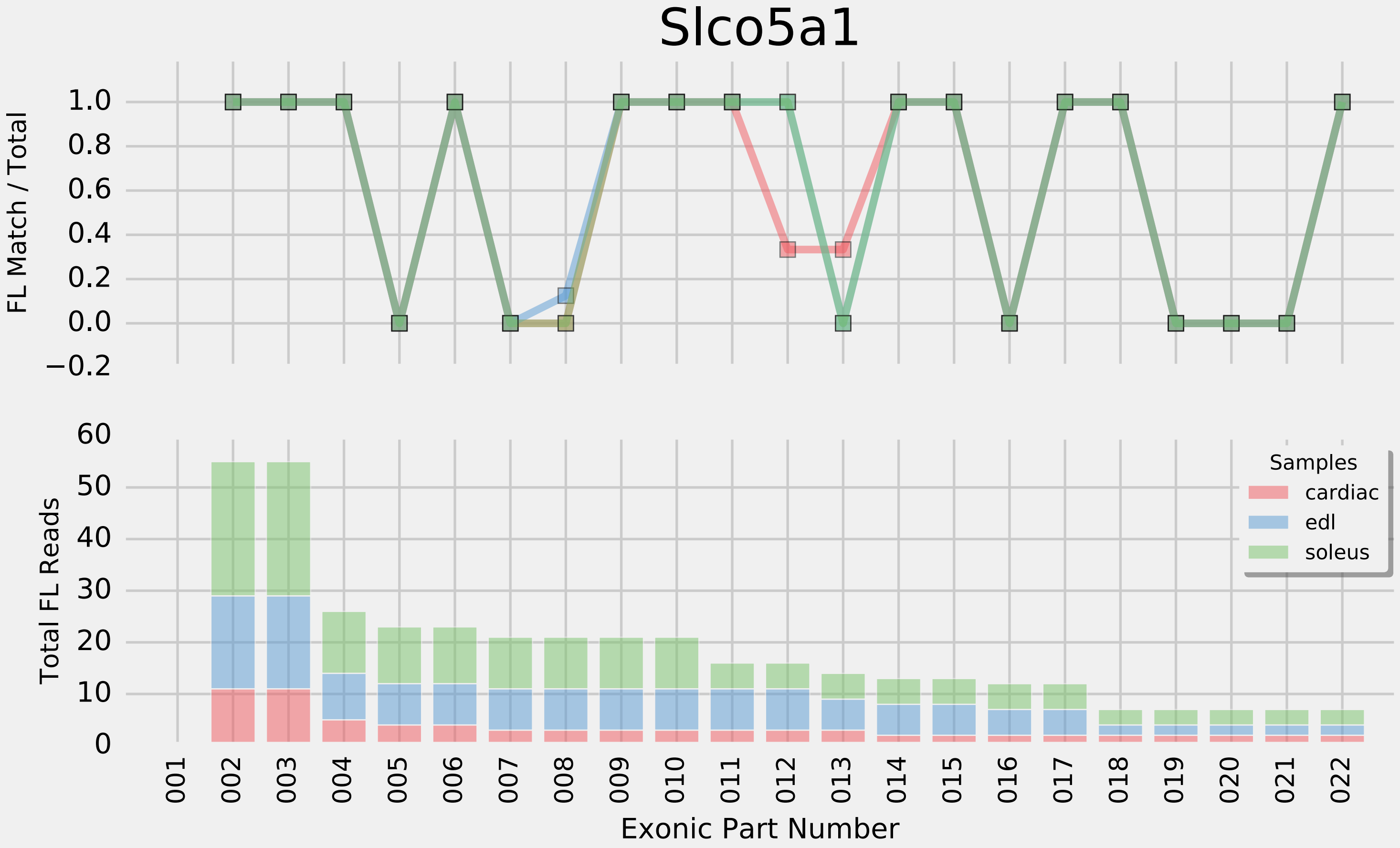

Slco5a1
FL Match / Total
1.0
0.8
0.6
0.4
0.2
0.0
−0.2
Total FL Reads
60
50
40
30
20
10
0
Samples
cardiac
edl
soleus
001
002
003
004
005
006
007
008
009
010
011
012
013
014
015
016
017
018
019
020
021
022
Exonic Part Number

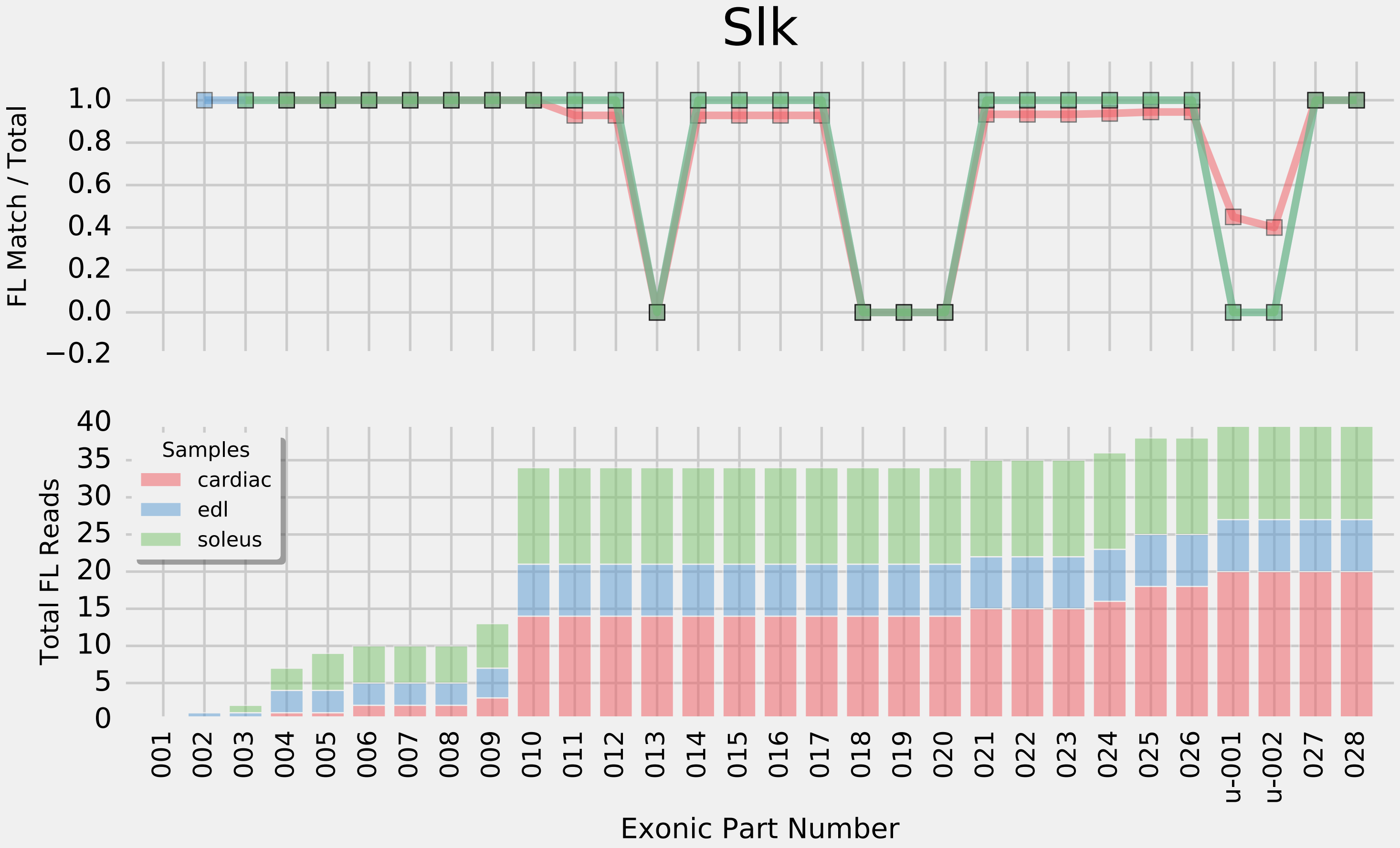
Slk
FL Match / Total
1.0
0.8
0.6
0.4
0.2
0.0
−0.2
Total FL Reads
40
35
30
25
20
15
10
5
0
Samples
cardiac
edl
soleus
001
002
003
004
005
006
007
008
009
010
011
012
013
014
015
016
017
018
019
020
021
022
023
024
025
026
u-001
u-002
027
028
Exonic Part Number

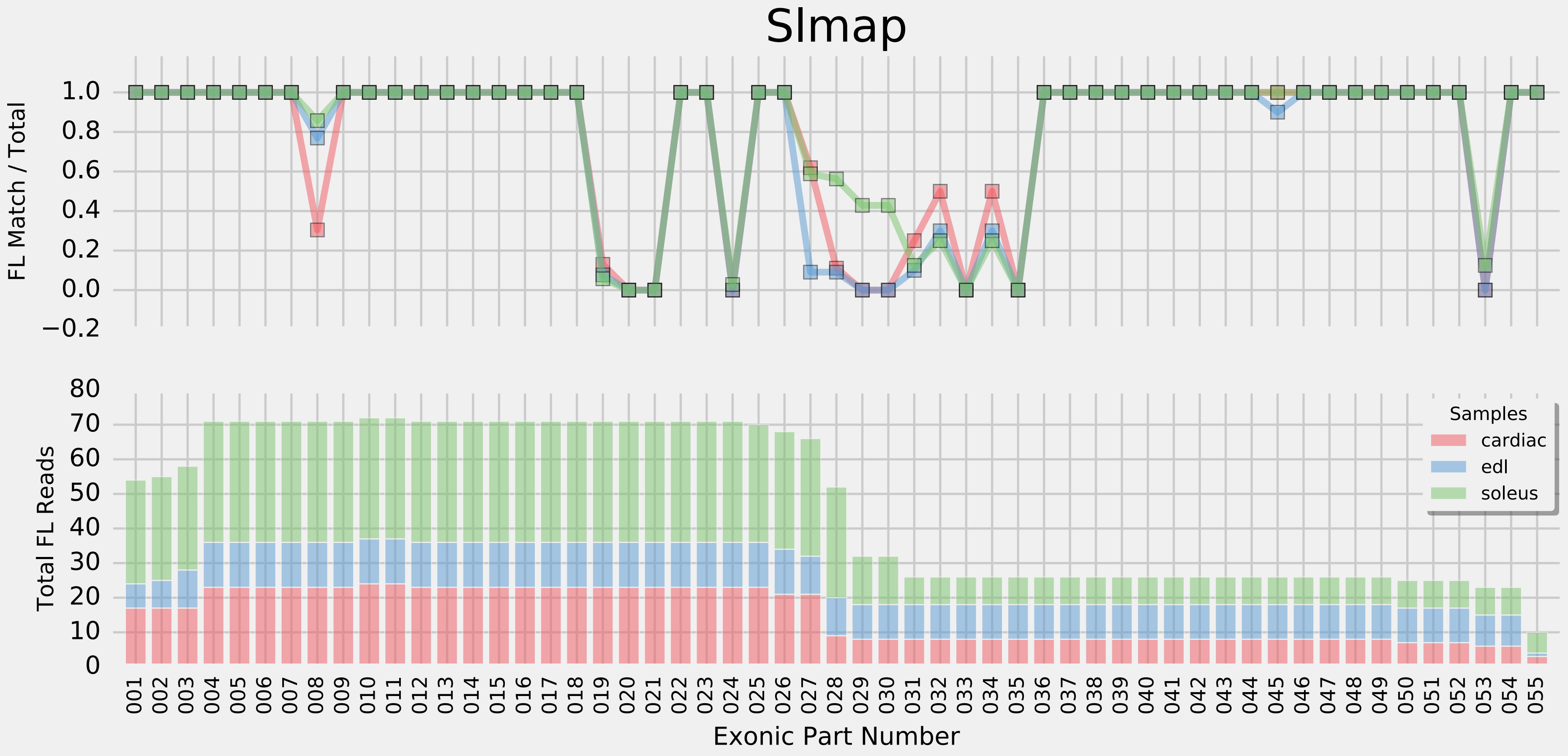

Slmap
FL Match / Total
1.0
0.8
0.6
0.4
0.2
0.0
−0.2
Total FL Reads
80
70
60
50
40
30
20
10
0
Samples
cardiac
edl
soleus
001 002 003 004 005 006 007 008 009 010 011 012 013 014 015 016 017 018 019 020 021 022 023 024 025 026 027 028 029 030 031 032 033 034 035 036 037 038 039 040 041 042 043 044 045 046 047 048 049 050 051 052 053 054 055
Exonic Part Number

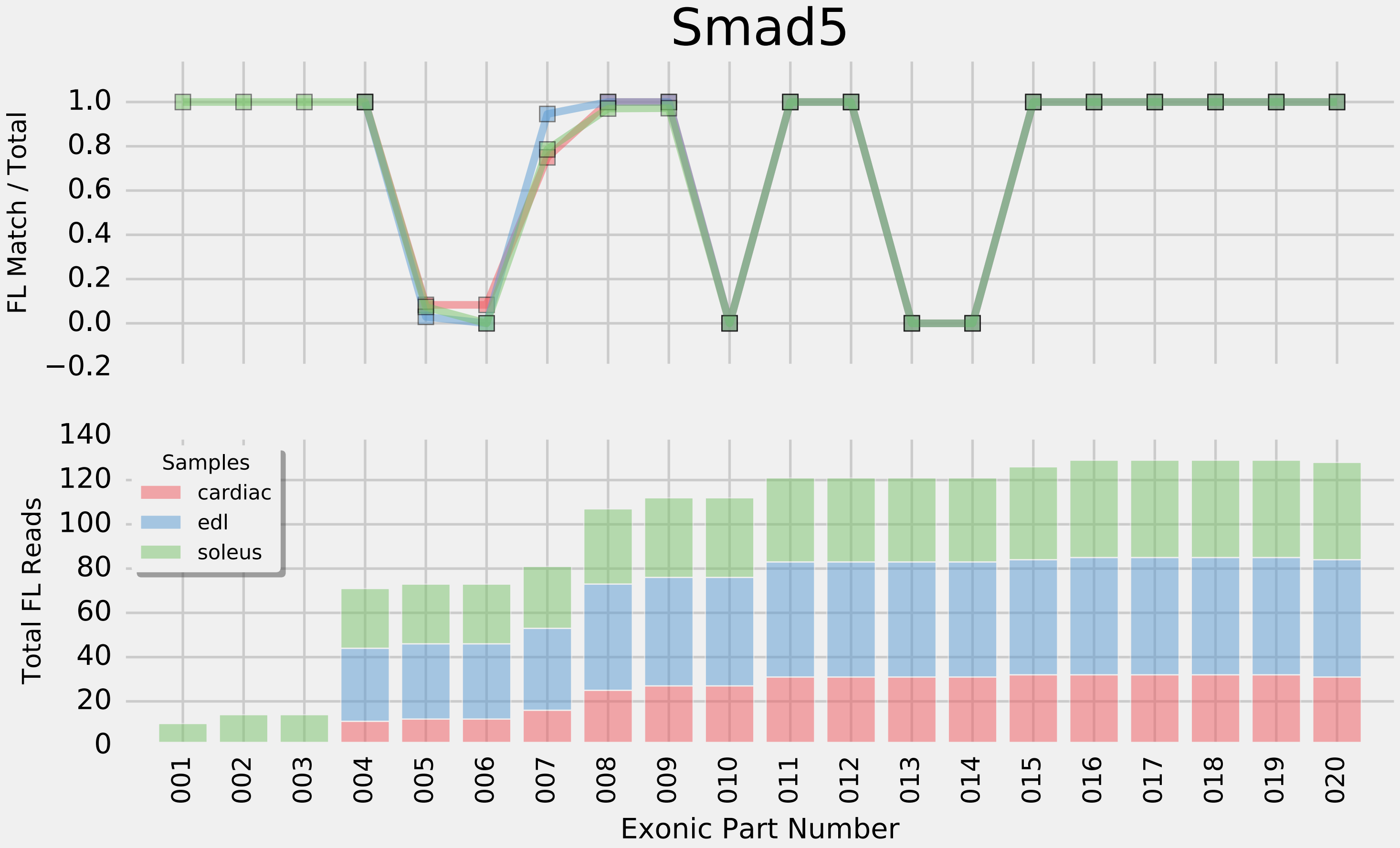

Smad5
FL Match / Total
1.0
0.8
0.6
0.4
0.2
0.0
−0.2
Total FL Reads
140
120
100
80
60
40
20
0
Samples
cardiac
edl
soleus
001
002
003
004
005
006
007
008
009
010
011
012
013
014
015
016
017
018
019
020
Exonic Part Number

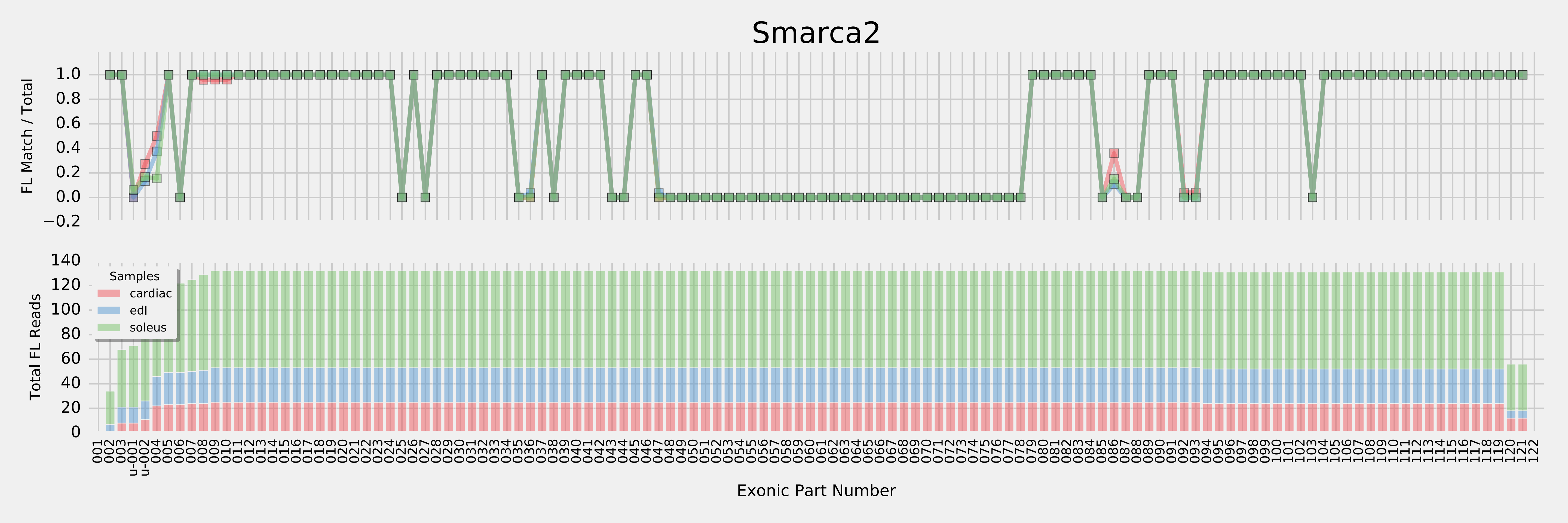

Smarca2
FL Match / Total
Total FL Reads
Samples
cardiac
edl
soleus
Exonic Part Number

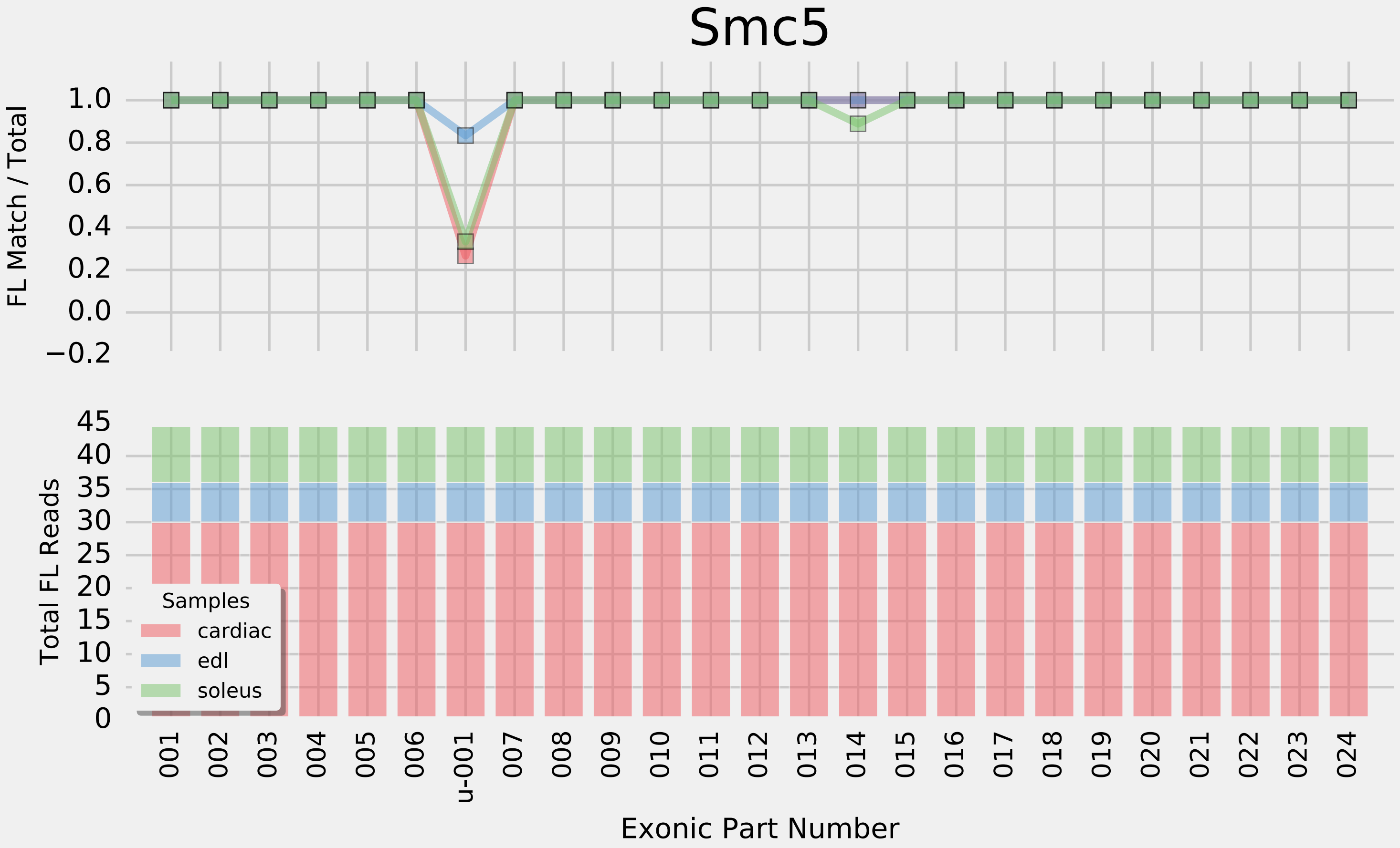

Smc5
FL Match / Total
1.0
0.8
0.6
0.4
0.2
0.0
−0.2
Total FL Reads
45
40
35
30
25
20
15
10
5
0
Samples
cardiac
edl
soleus
001
002
003
004
005
006
u-001
007
008
009
010
011
012
013
014
015
016
017
018
019
020
021
022
023
024
Exonic Part Number

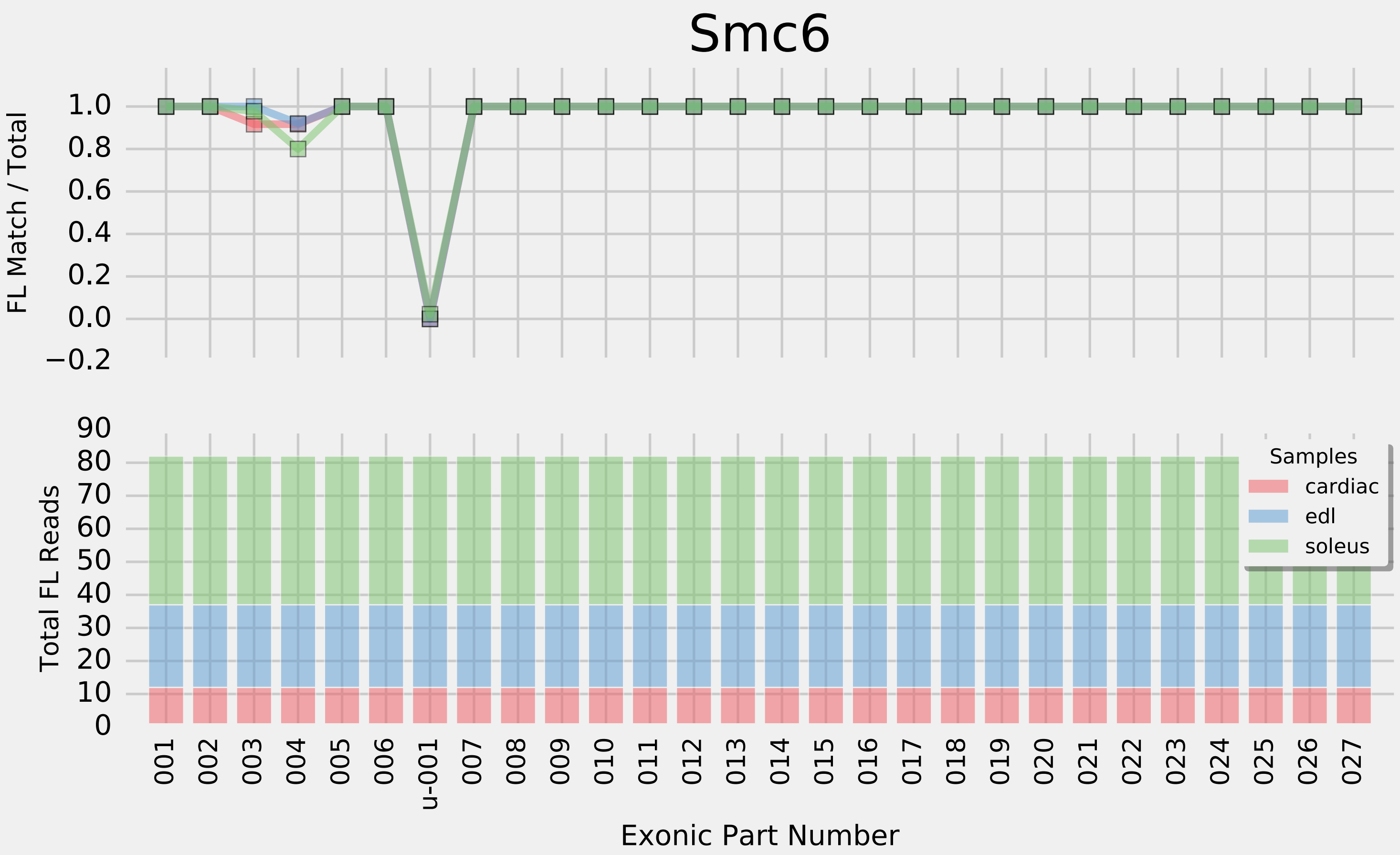

Smc6
FL Match / Total
1.0
0.8
0.6
0.4
0.2
0.0
−0.2
Total FL Reads
90
80
70
60
50
40
30
20
10
0
Samples
cardiac
edl
soleus
001
002
003
004
005
006
u-001
007
008
009
010
011
012
013
014
015
016
017
018
019
020
021
022
023
024
025
026
027
Exonic Part Number

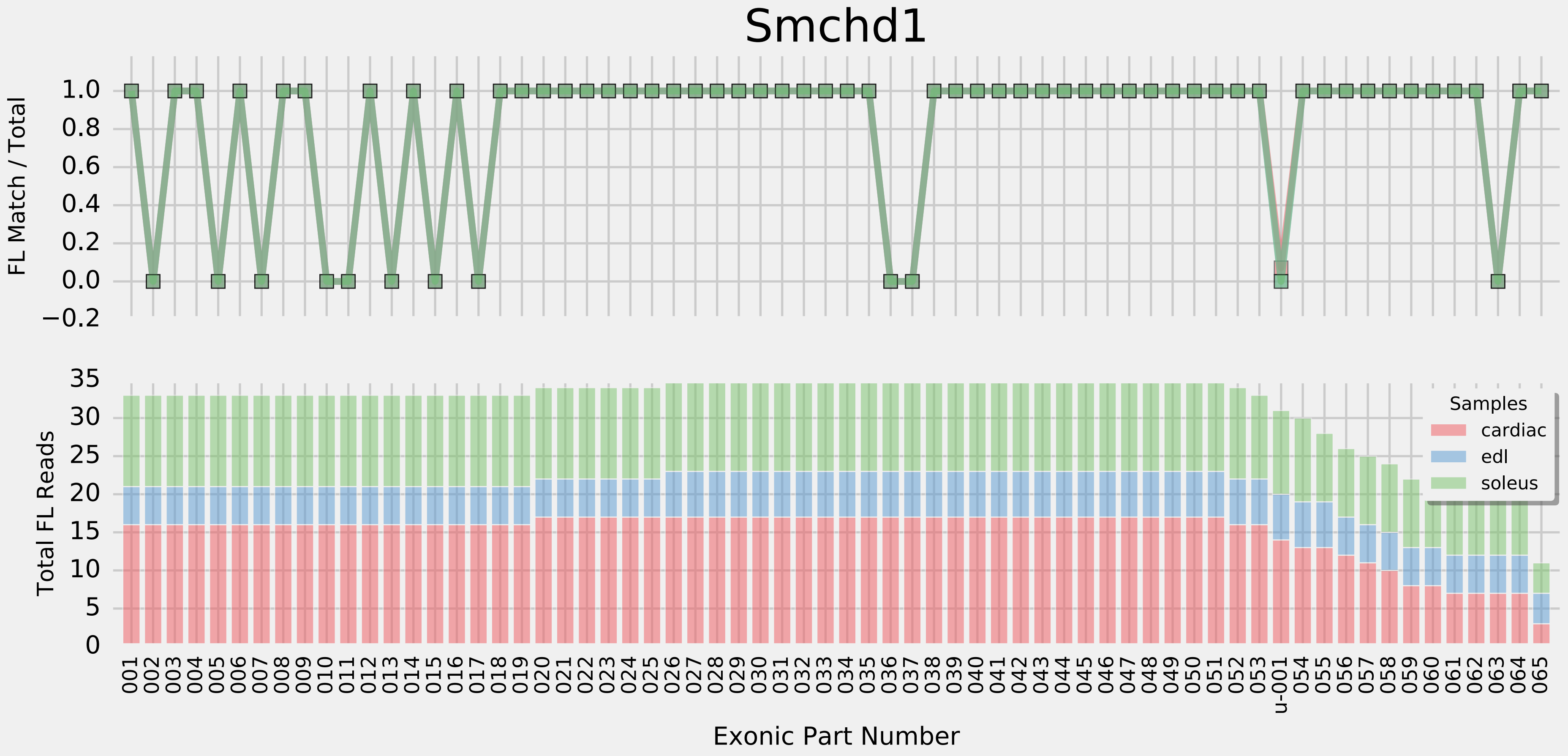

Smchd1
FL Match / Total
Total FL Reads
Exonic Part Number
Samples
cardiac
edl
soleus
001 002 003 004 005 006 007 008 009 010 011 012 013 014 015 016 017 018 019 020 021 022 023 024 025 026 027 028 029 030 031 032 033 034 035 036 037 038 039 040 041 042 043 044 045 046 047 048 049 050 051 052 053 u-001 054 055 056 057 058 059 060 061 062 063 064 065

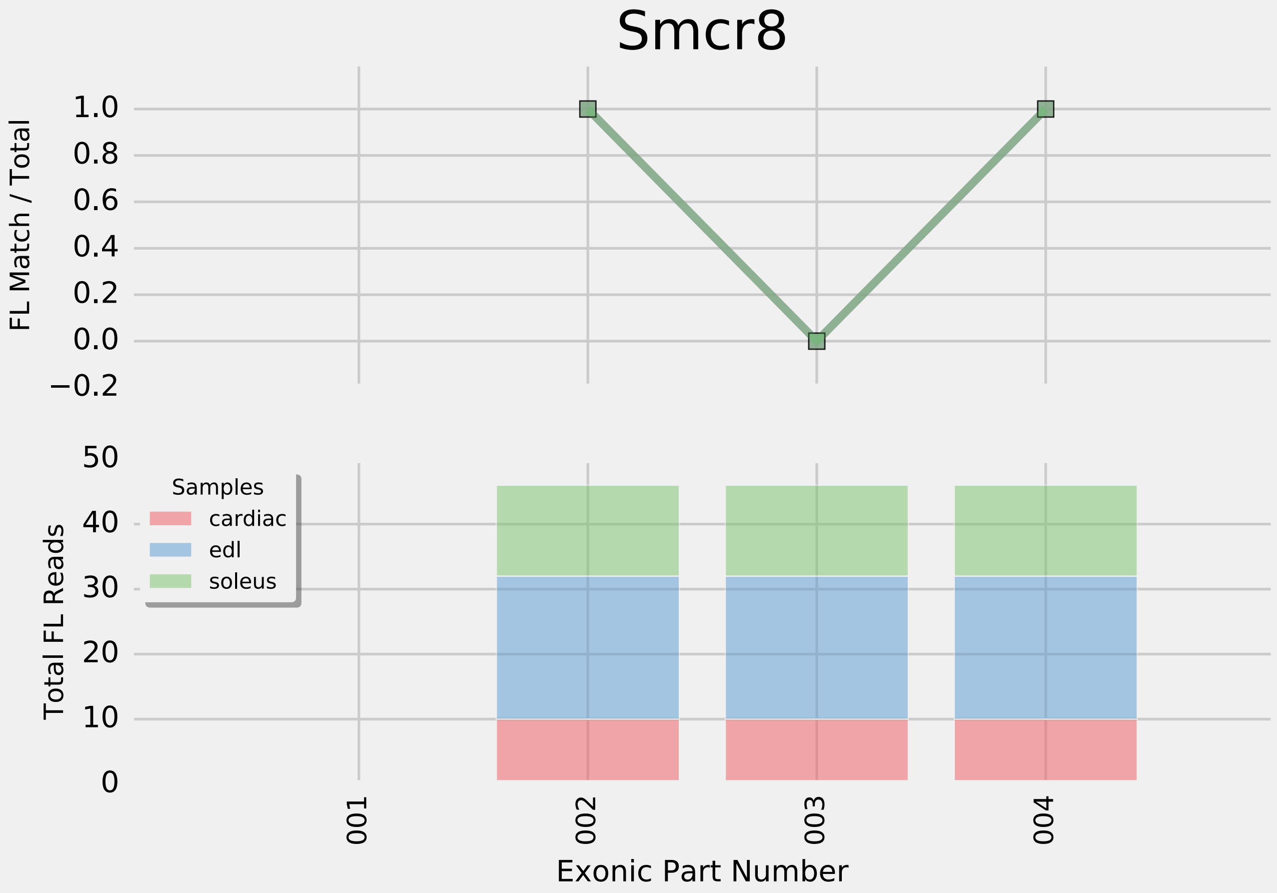
Smcr8
FL Match / Total
1.0
0.8
0.6
0.4
0.2
0.0
−0.2
50
40
30
20
10
0
Total FL Reads
Samples
cardiac
edl
soleus
001
002
003
004
Exonic Part Number

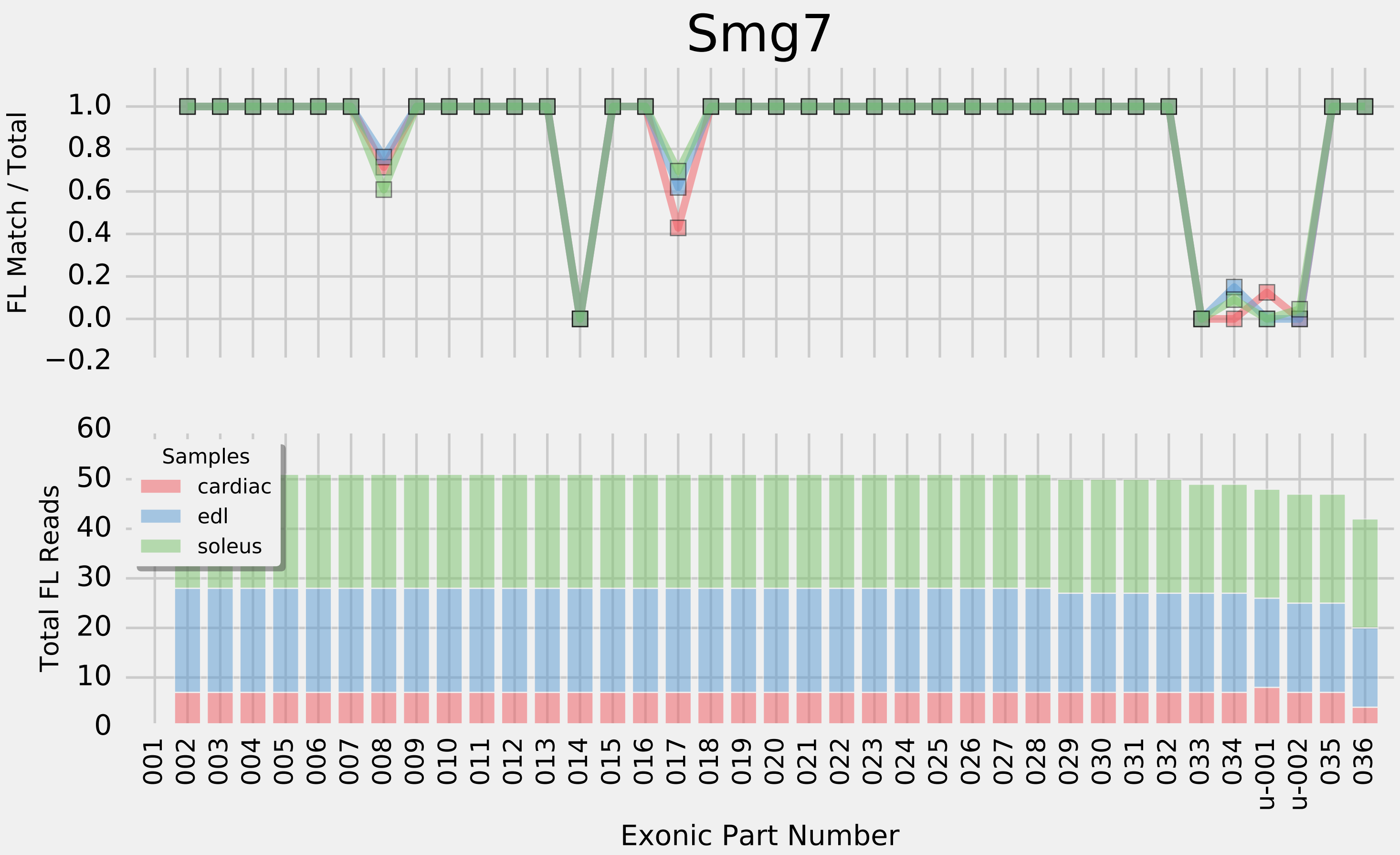

Smg7
FL Match / Total
1.0
0.8
0.6
0.4
0.2
0.0
−0.2
Total FL Reads
60
50
40
30
20
10
0
Samples
cardiac
edl
soleus
001
002
003
004
005
006
007
008
009
010
011
012
013
014
015
016
017
018
019
020
021
022
023
024
025
026
027
028
029
030
031
032
033
034
u-001
u-002
035
036
Exonic Part Number

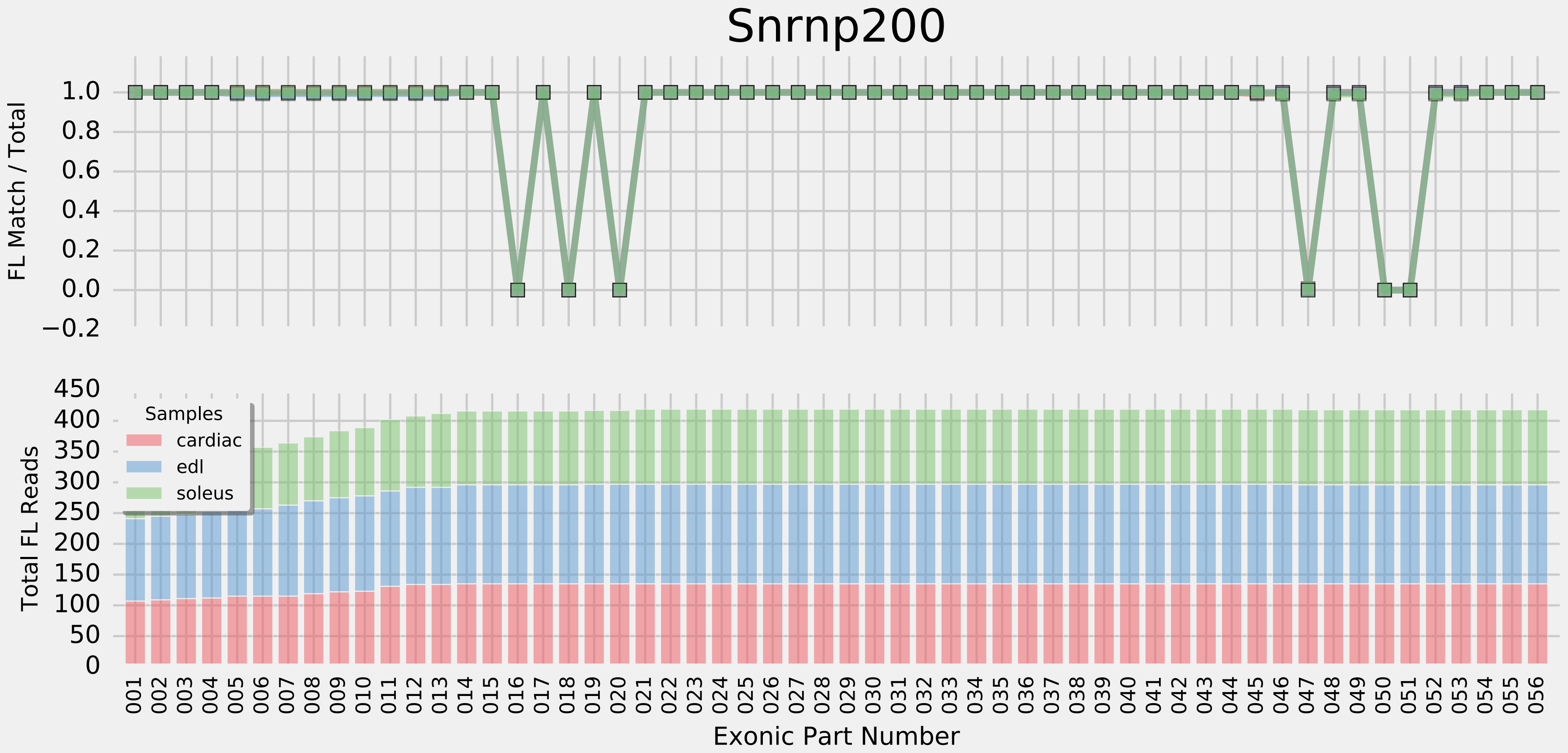

Snrnp200
FL Match / Total
1.0
0.8
0.6
0.4
0.2
0.0
−0.2
Total FL Reads
450
400
350
300
250
200
150
100
50
0
Samples
cardiac
edl
soleus
001 002 003 004 005 006 007 008 009 010 011 012 013 014 015 016 017 018 019 020 021 022 023 024 025 026 027 028 029 030 031 032 033 034 035 036 037 038 039 040 041 042 043 044 045 046 047 048 049 050 051 052 053 054 055 056
Exonic Part Number

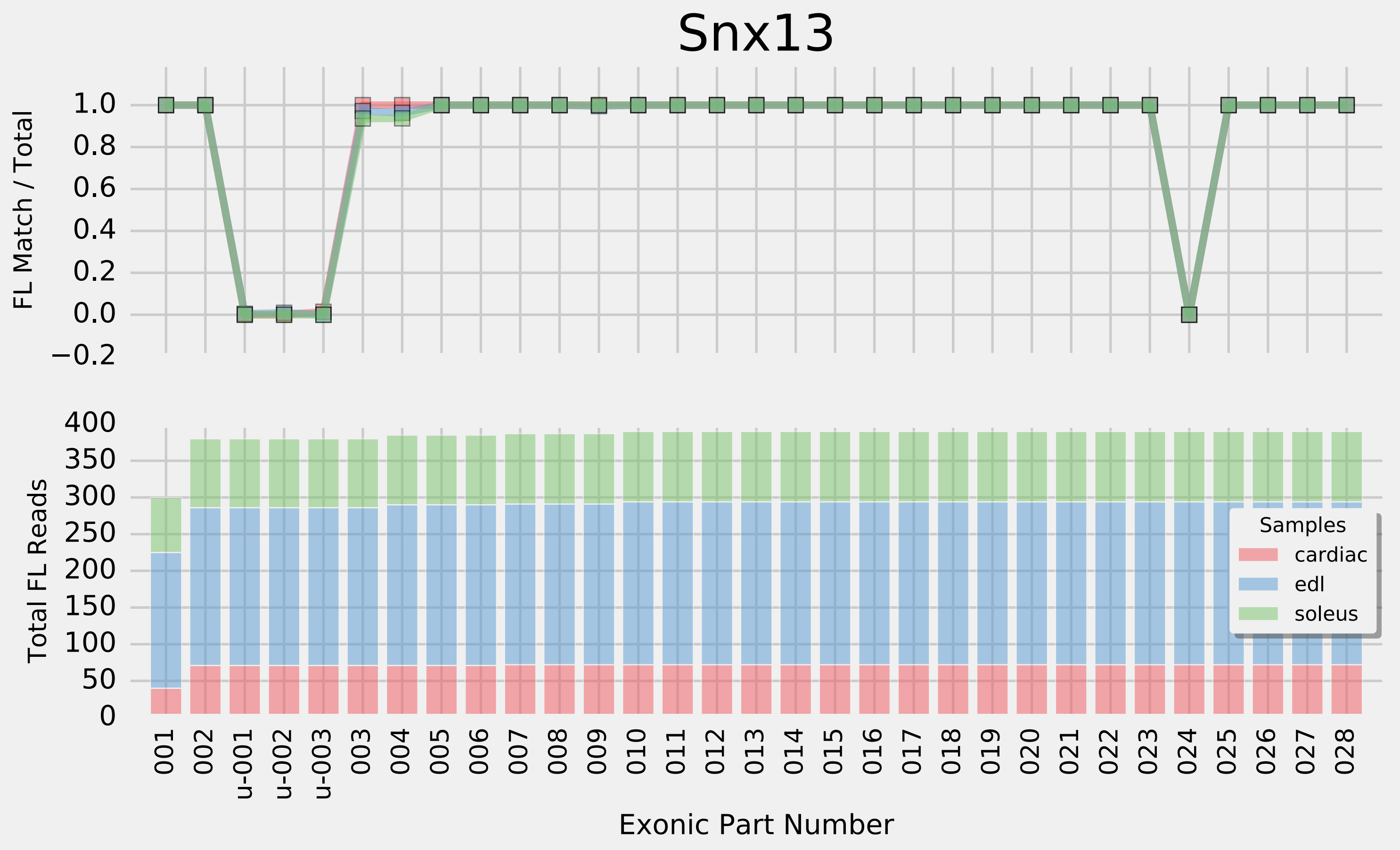

Snx13
FL Match / Total
1.0
0.8
0.6
0.4
0.2
0.0
−0.2
Total FL Reads
400
350
300
250
200
150
100
50
0
Samples
cardiac
edl
soleus
001
002
u-001
u-002
u-003
003
004
005
006
007
008
009
010
011
012
013
014
015
016
017
018
019
020
021
022
023
024
025
026
027
028
Exonic Part Number

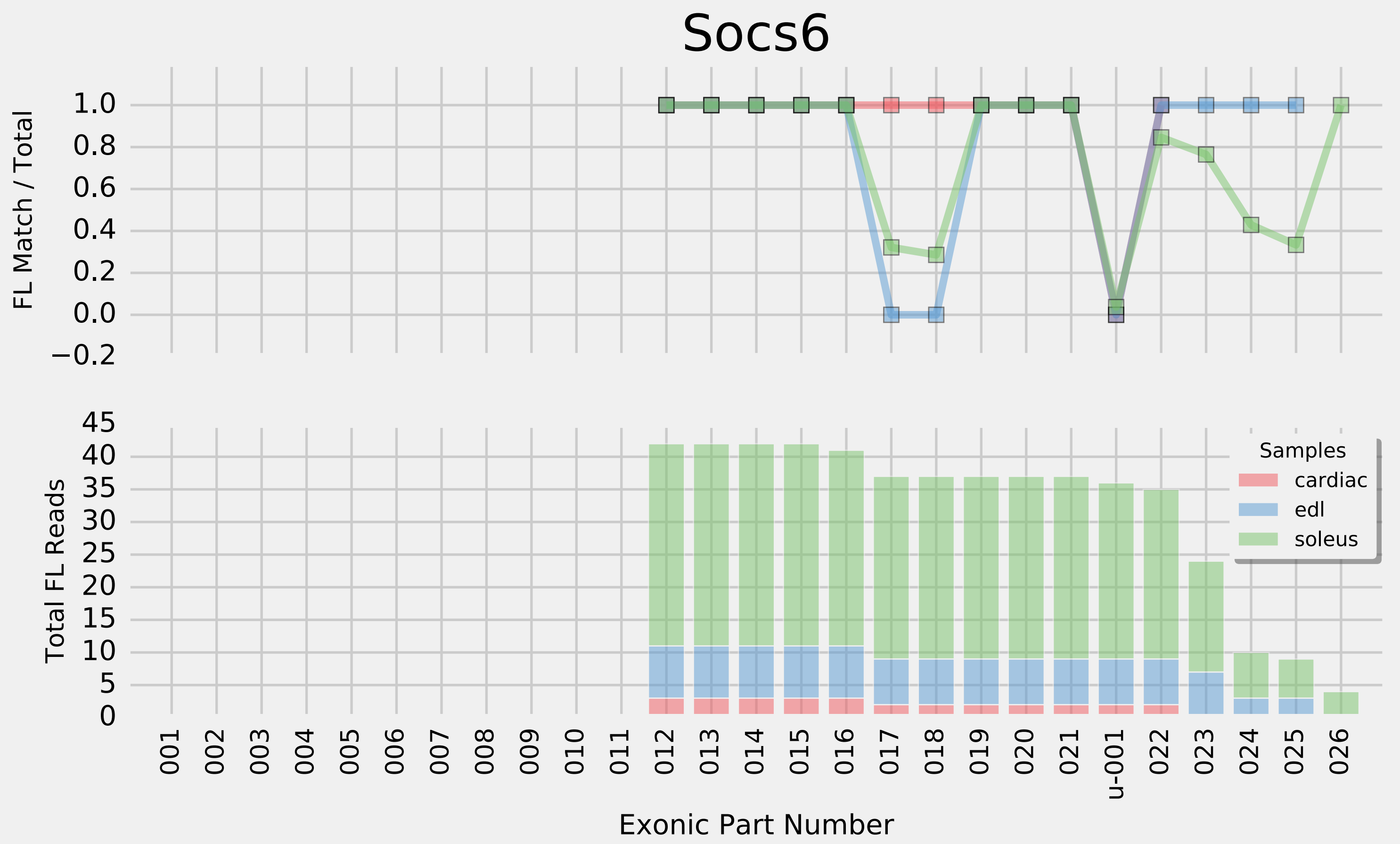

Socs6
FL Match / Total
1.0
0.8
0.6
0.4
0.2
0.0
−0.2
Total FL Reads
45
40
35
30
25
20
15
10
5
0
Samples
cardiac
edl
soleus
001
002
003
004
005
006
007
008
009
010
011
012
013
014
015
016
017
018
019
020
021
u-001
022
023
024
025
026
Exonic Part Number

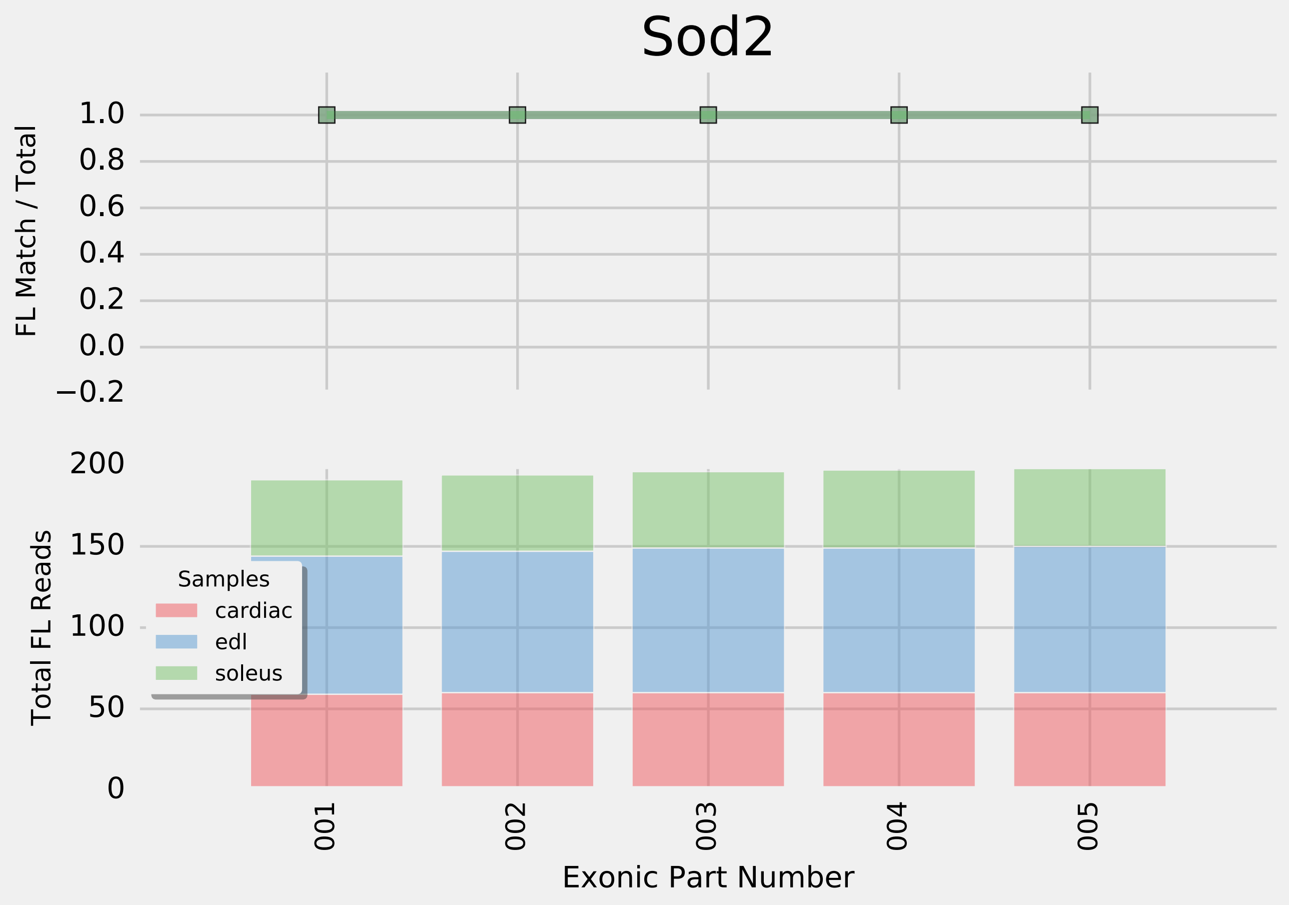
Sod2
FL Match / Total
1.0
0.8
0.6
0.4
0.2
0.0
−0.2
Total FL Reads
200
150
100
50
0
Samples
cardiac
edl
soleus
001
002
003
004
005
Exonic Part Number

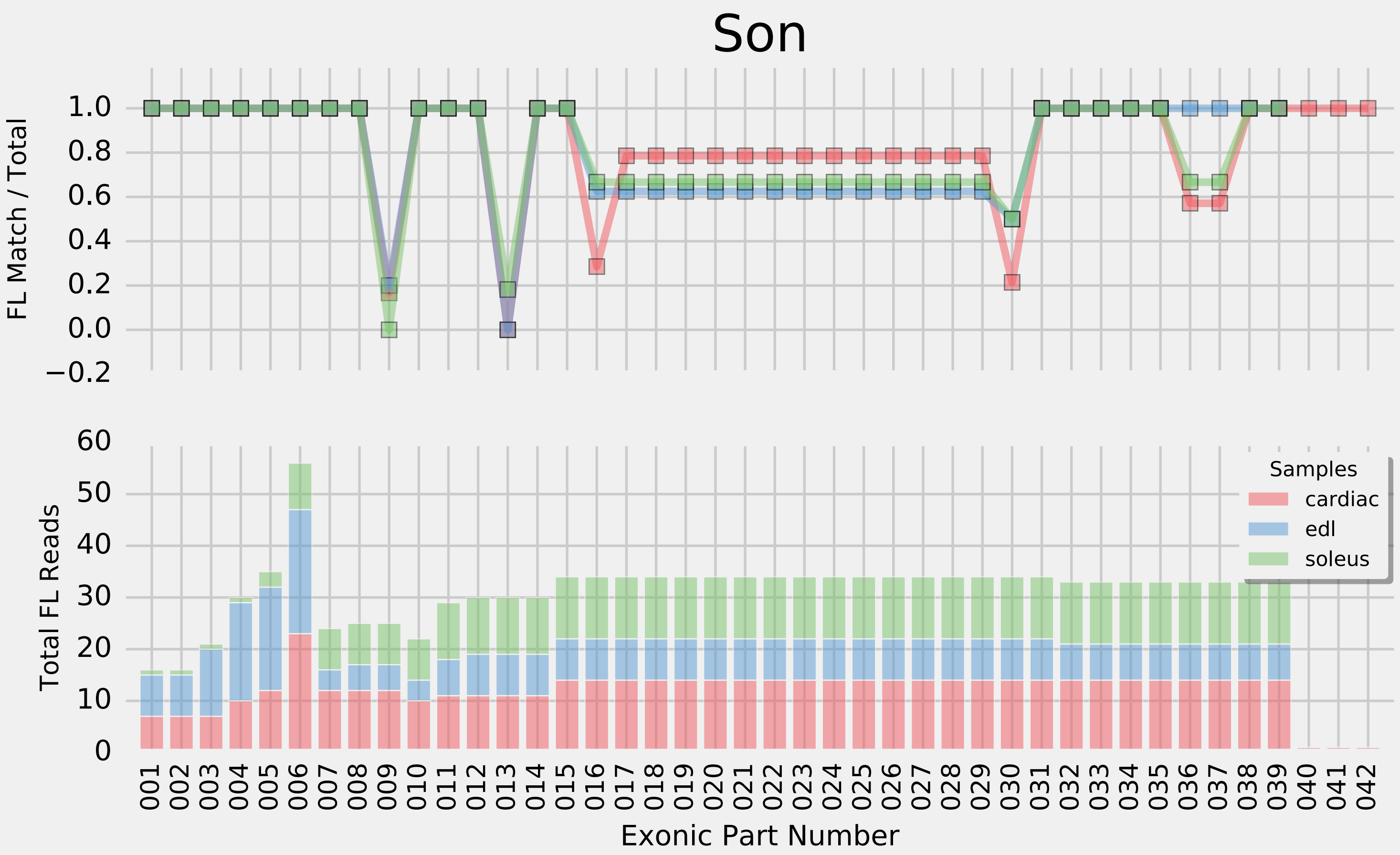

Son
FL Match / Total
1.0
0.8
0.6
0.4
0.2
0.0
−0.2
Total FL Reads
60
50
40
30
20
10
0
Samples
cardiac
edl
soleus
001 002 003 004 005 006 007 008 009 010 011 012 013 014 015 016 017 018 019 020 021 022 023 024 025 026 027 028 029 030 031 032 033 034 035 036 037 038 039 040 041 042
Exonic Part Number

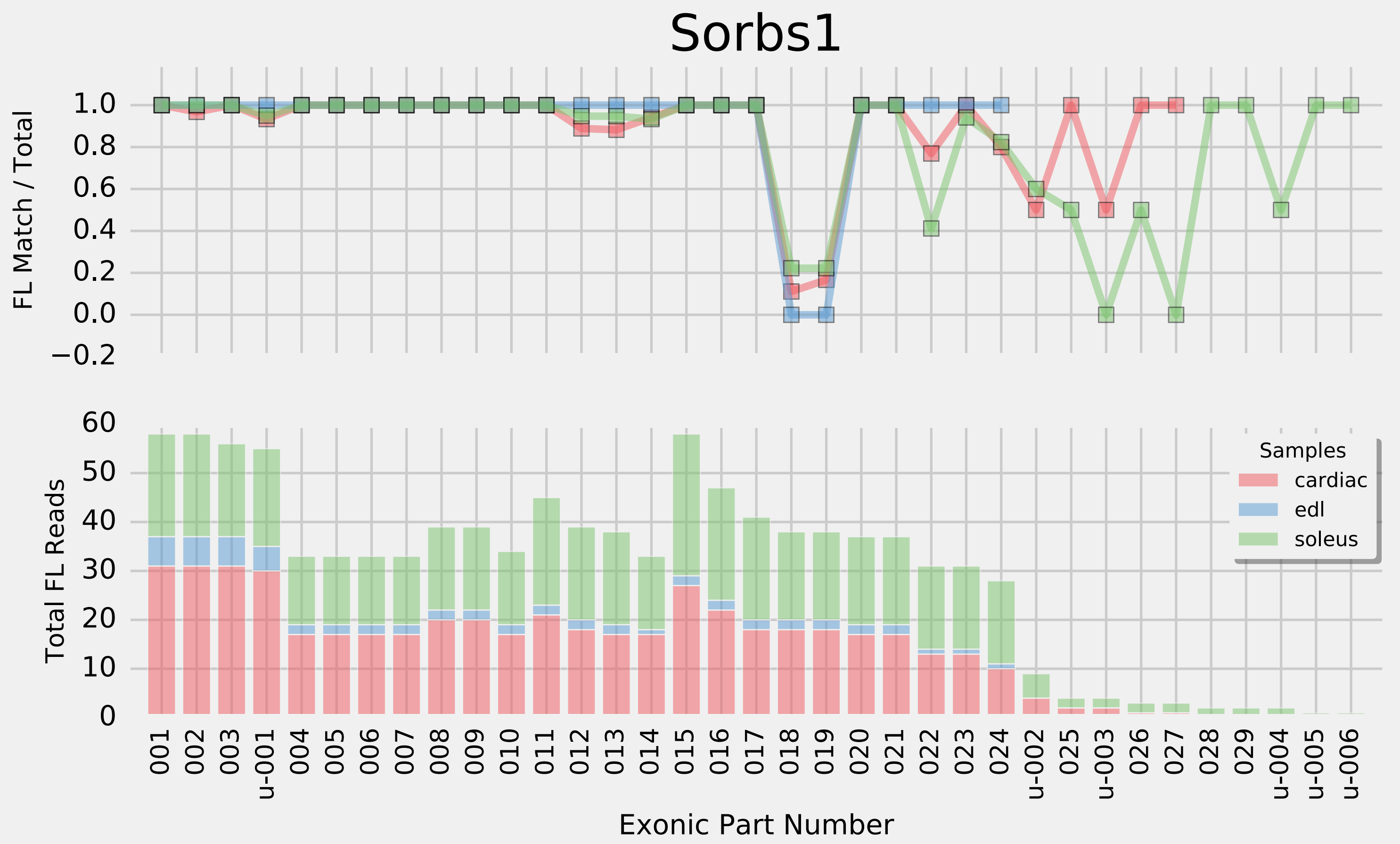

Sorbs1
FL Match / Total
1.0
0.8
0.6
0.4
0.2
0.0
−0.2
60
50
40
30
20
10
0
Total FL Reads
Samples
cardiac
edl
soleus
001 002 003 u-001 004 005 006 007 008 009 010 011 012 013 014 015 016 017 018 019 020 021 022 023 024 u-002 025 u-003 026 027 028 029 u-004 u-005 u-006
Exonic Part Number

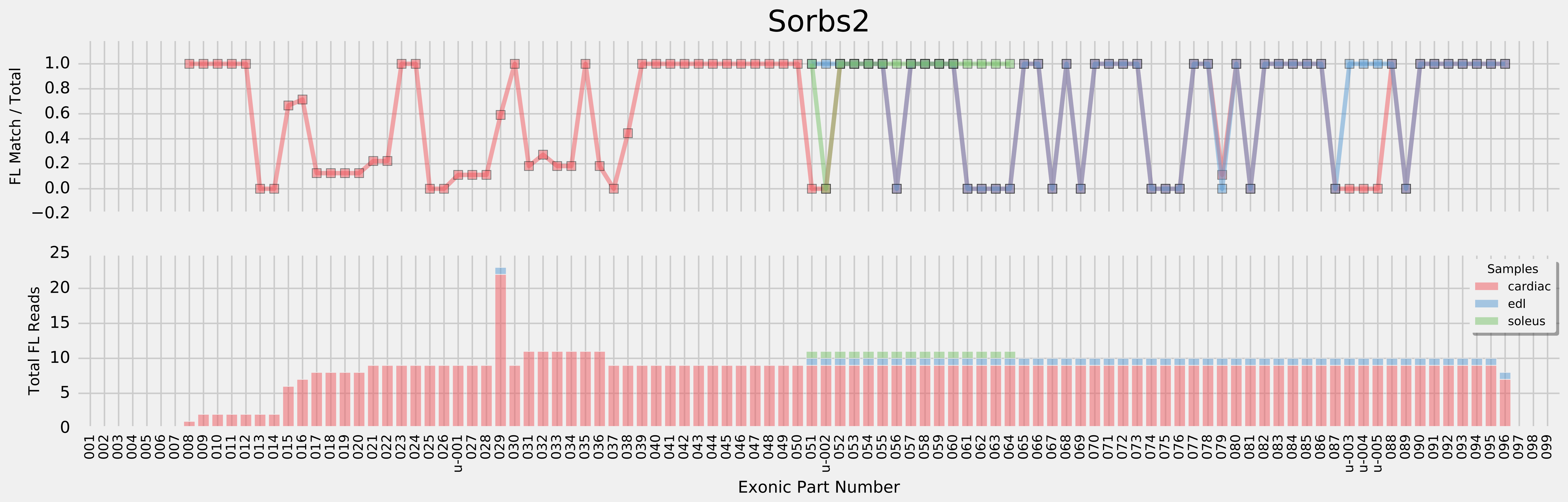
Sorbs2
FL Match / Total
Total FL Reads
Exonic Part Number
Samples
cardiac
edl
soleus

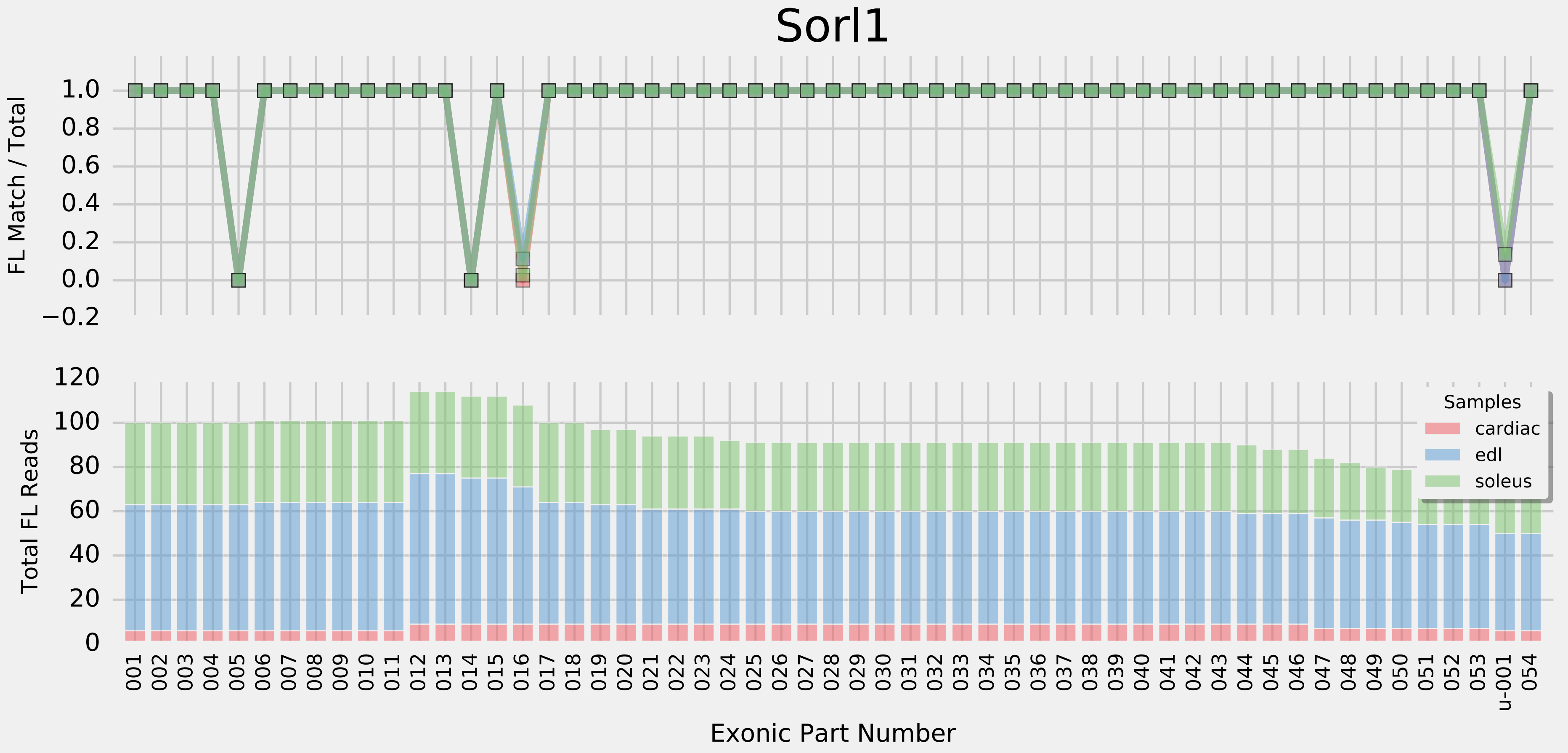

Sorl1
FL Match / Total
1.0
0.8
0.6
0.4
0.2
0.0
−0.2
Total FL Reads
120
100
80
60
40
20
0
001 002 003 004 005 006 007 008 009 010 011 012 013 014 015 016 017 018 019 020 021 022 023 024 025 026 027 028 029 030 031 032 033 034 035 036 037 038 039 040 041 042 043 044 045 046 047 048 049 050 051 052 053 u-001 054
Exonic Part Number
Samples
cardiac
edl
soleus

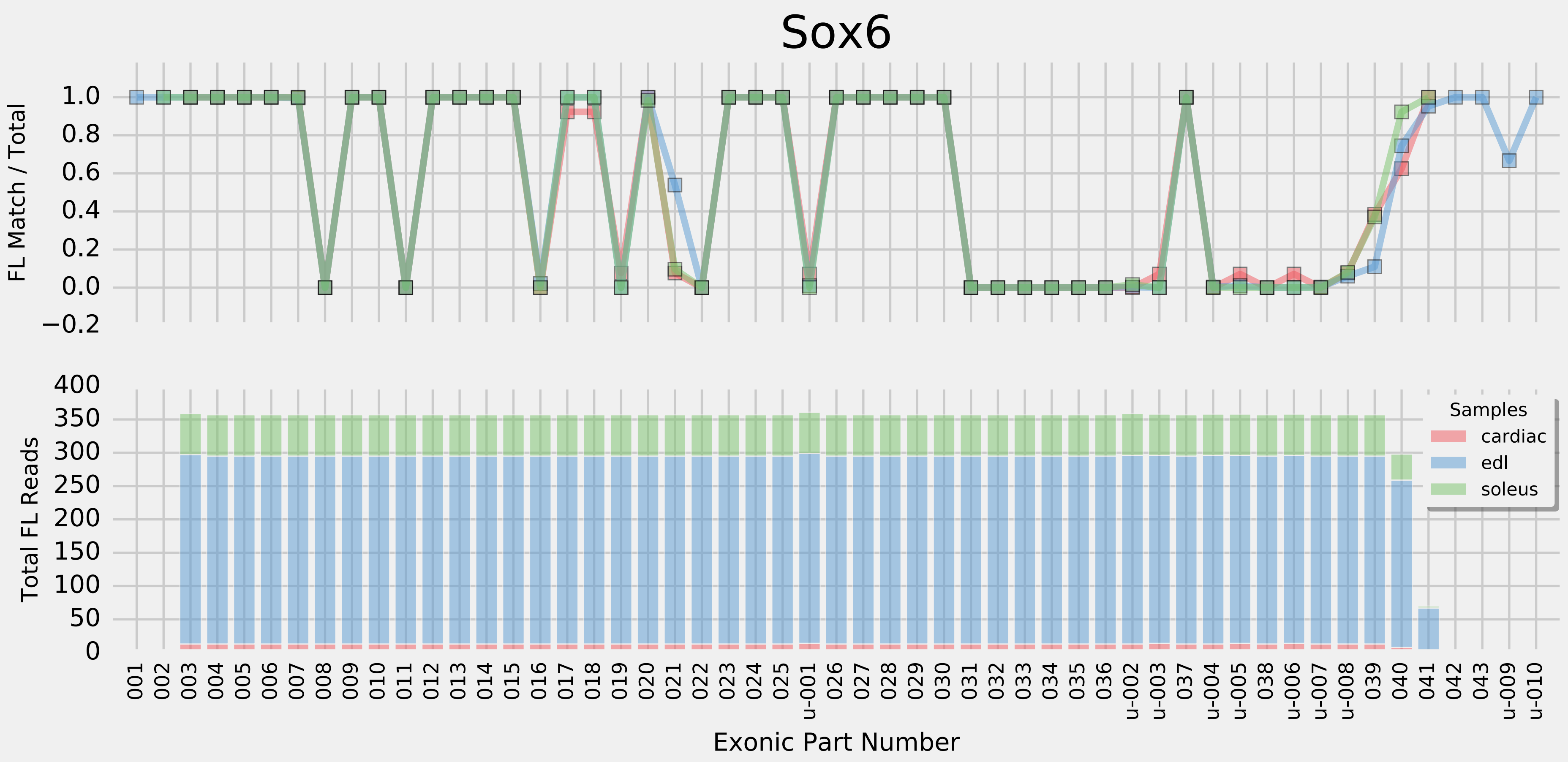

Sox6
FL Match / Total
1.0
0.8
0.6
0.4
0.2
0.0
−0.2
Total FL Reads
400
350
300
250
200
150
100
50
0
Samples
cardiac
edl
soleus
001 002 003 004 005 006 007 008 009 010 011 012 013 014 015 016 017 018 019 020 021 022 023 024 025 u-001 026 027 028 029 030 031 032 033 034 035 036 u-002 u-003 037 u-004 u-005 038 u-006 u-007 u-008 039 040 041 042 043 u-009 u-010
Exonic Part Number

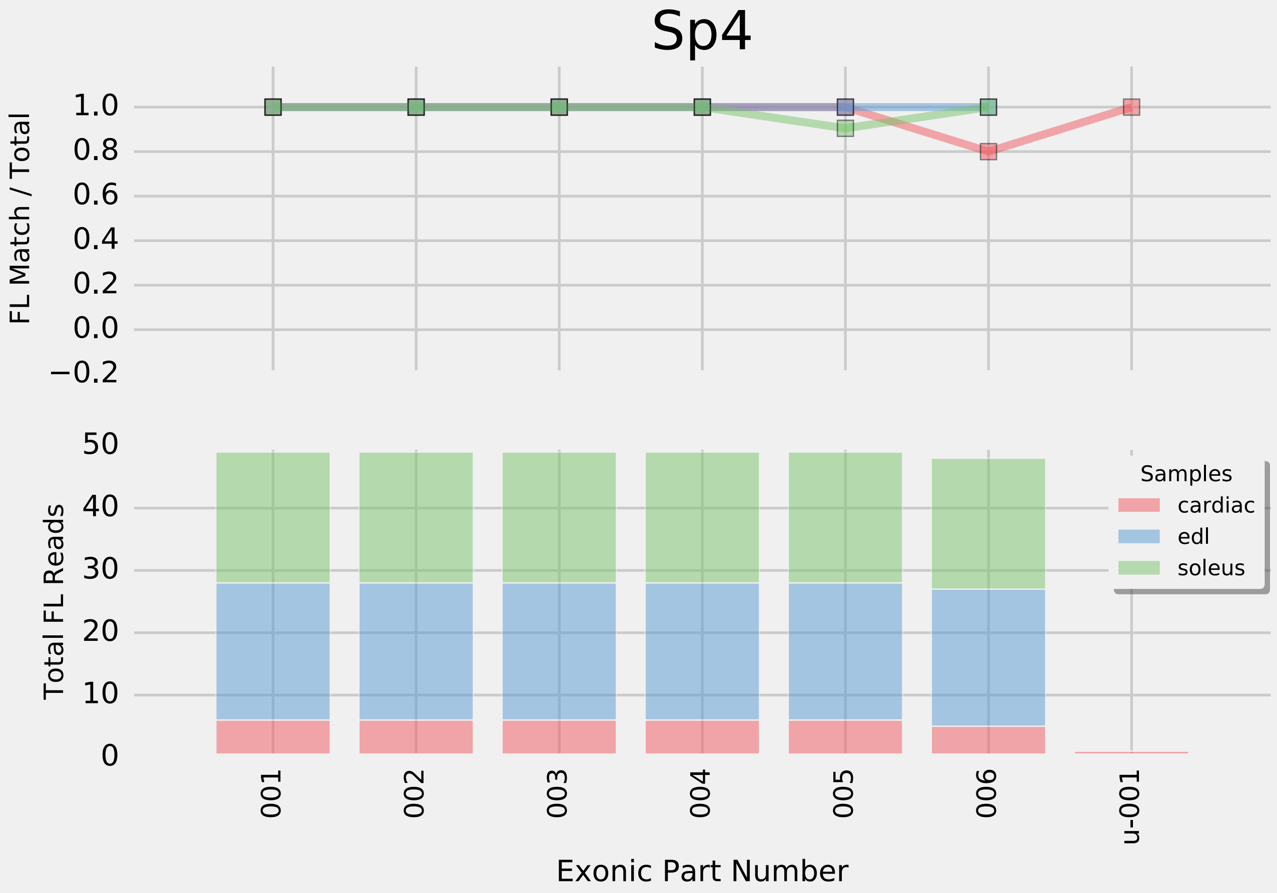
Sp4
FL Match / Total
1.0
0.8
0.6
0.4
0.2
0.0
−0.2
Total FL Reads
50
40
30
20
10
0
Samples
cardiac
edl
soleus
001
002
003
004
005
006
u-001
Exonic Part Number

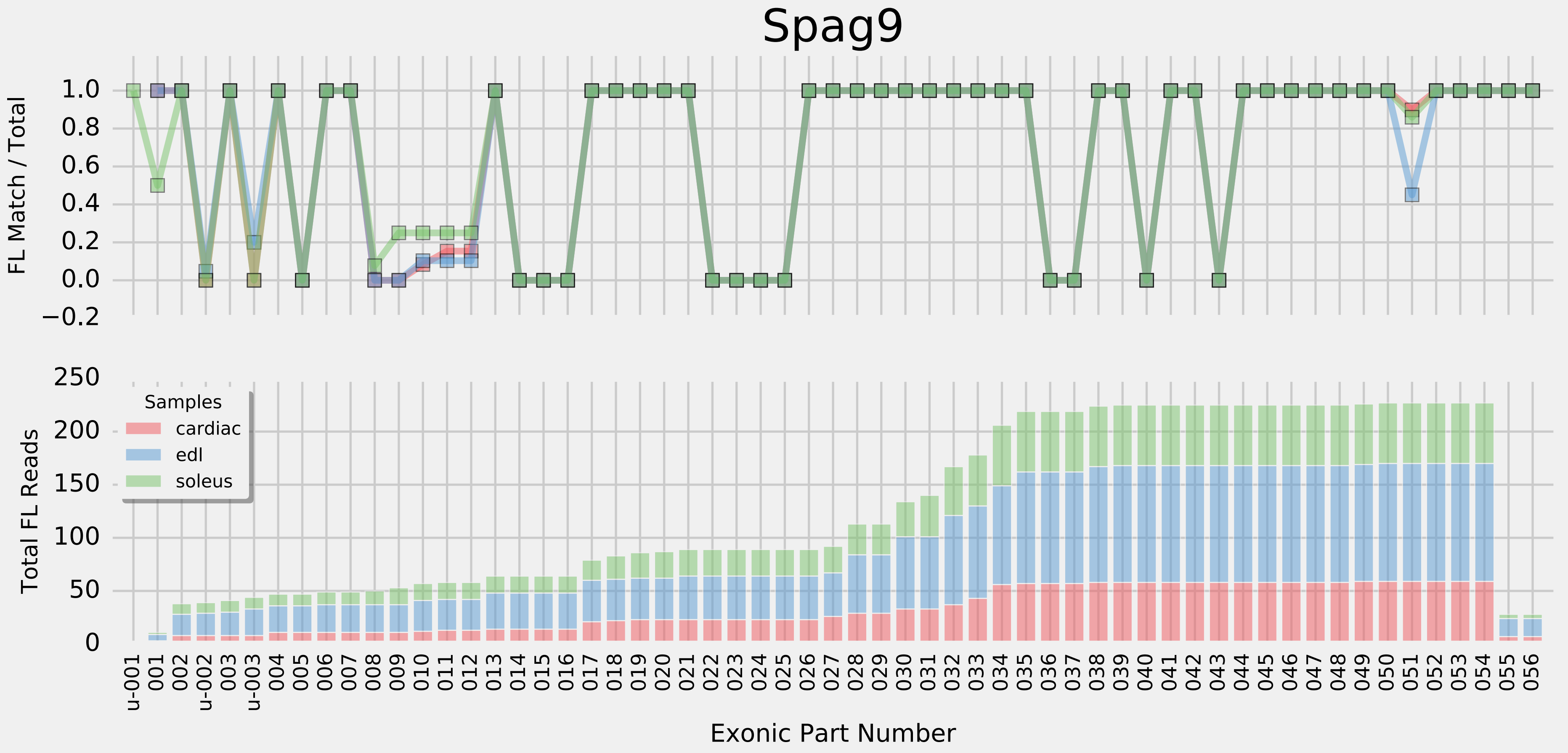

Spag9
FL Match / Total
1.0
0.8
0.6
0.4
0.2
0.0
−0.2
Total FL Reads
250
200
150
100
50
0
Samples
cardiac
edl
soleus
u-001 001 002 u-002 003 u-003 004 005 006 007 008 009 010 011 012 013 014 015 016 017 018 019 020 021 022 023 024 025 026 027 028 029 030 031 032 033 034 035 036 037 038 039 040 041 042 043 044 045 046 047 048 049 050 051 052 053 054 055 056
Exonic Part Number

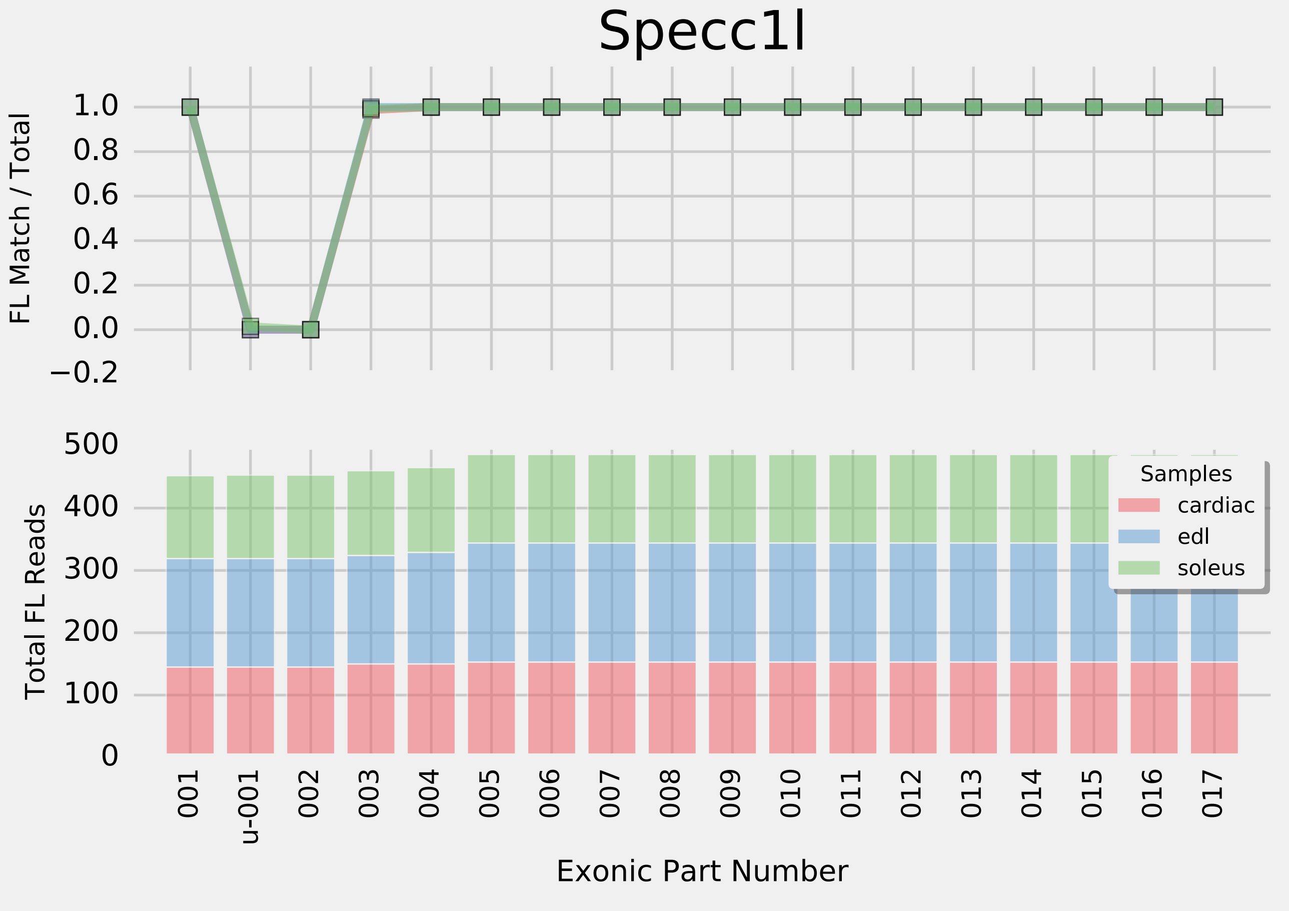
Specc1l
FL Match / Total
1.0
0.8
0.6
0.4
0.2
0.0
−0.2
Total FL Reads
500
400
300
200
100
0
Samples
cardiac
edl
soleus
001
u-001
002
003
004
005
006
007
008
009
010
011
012
013
014
015
016
017
Exonic Part Number

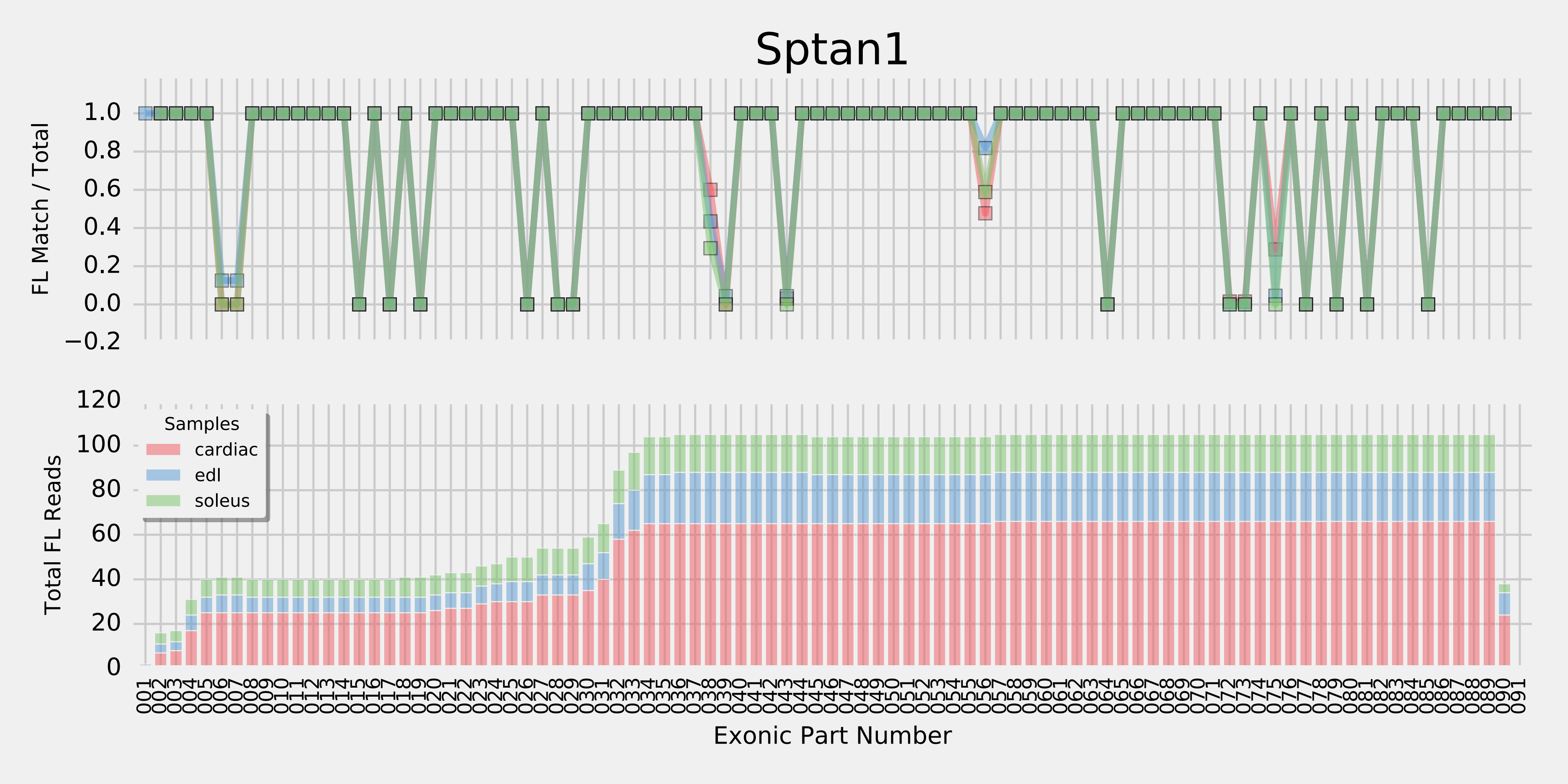

Sptan1
FL Match / Total
Total FL Reads
Samples
cardiac
edl
soleus
Exonic Part Number

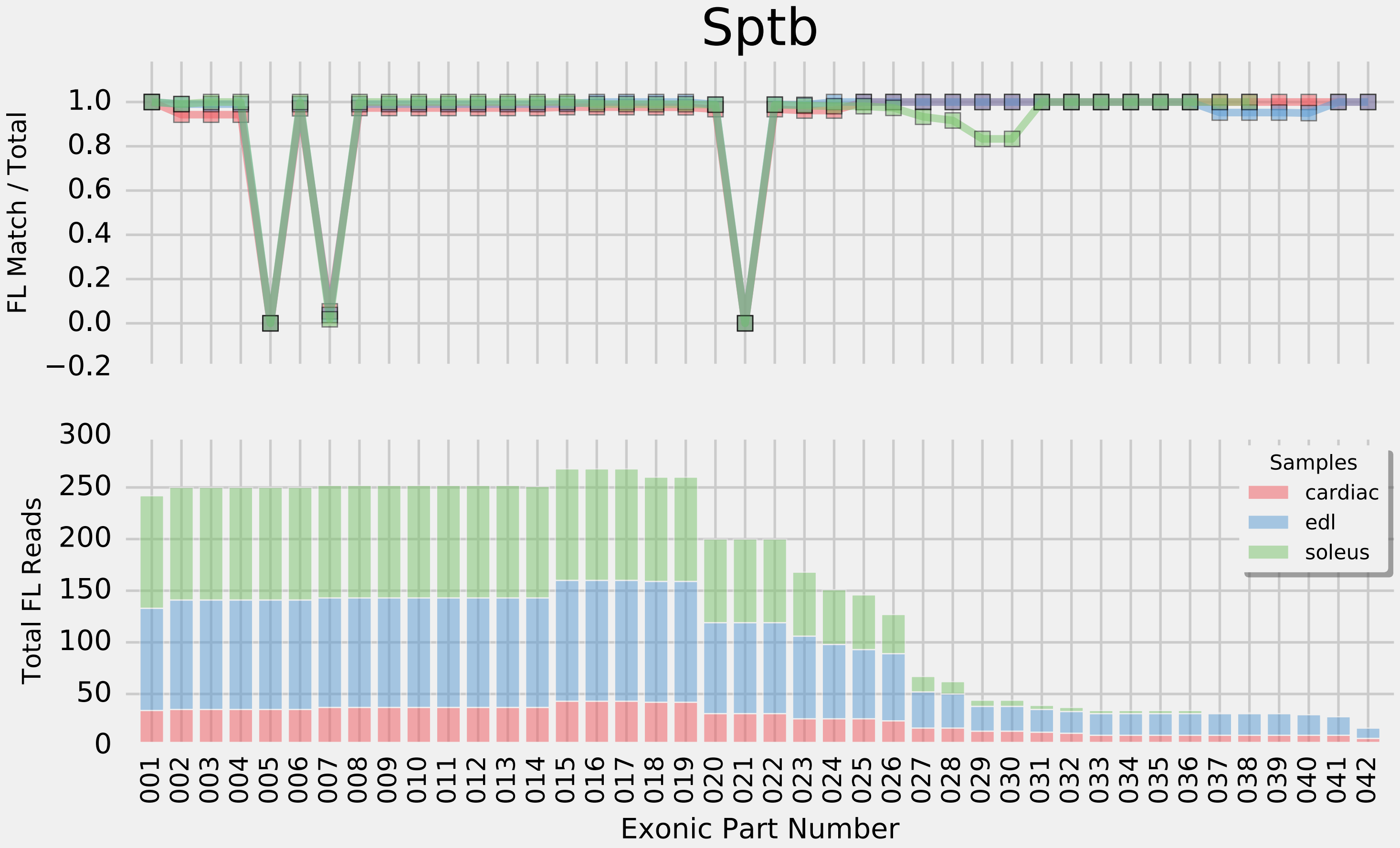

Sptb
FL Match / Total
1.0
0.8
0.6
0.4
0.2
0.0
−0.2
Total FL Reads
300
250
200
150
100
50
0
Samples
cardiac
edl
soleus
001
002
003
004
005
006
007
008
009
010
011
012
013
014
015
016
017
018
019
020
021
022
023
024
025
026
027
028
029
030
031
032
033
034
035
036
037
038
039
040
041
042
Exonic Part Number

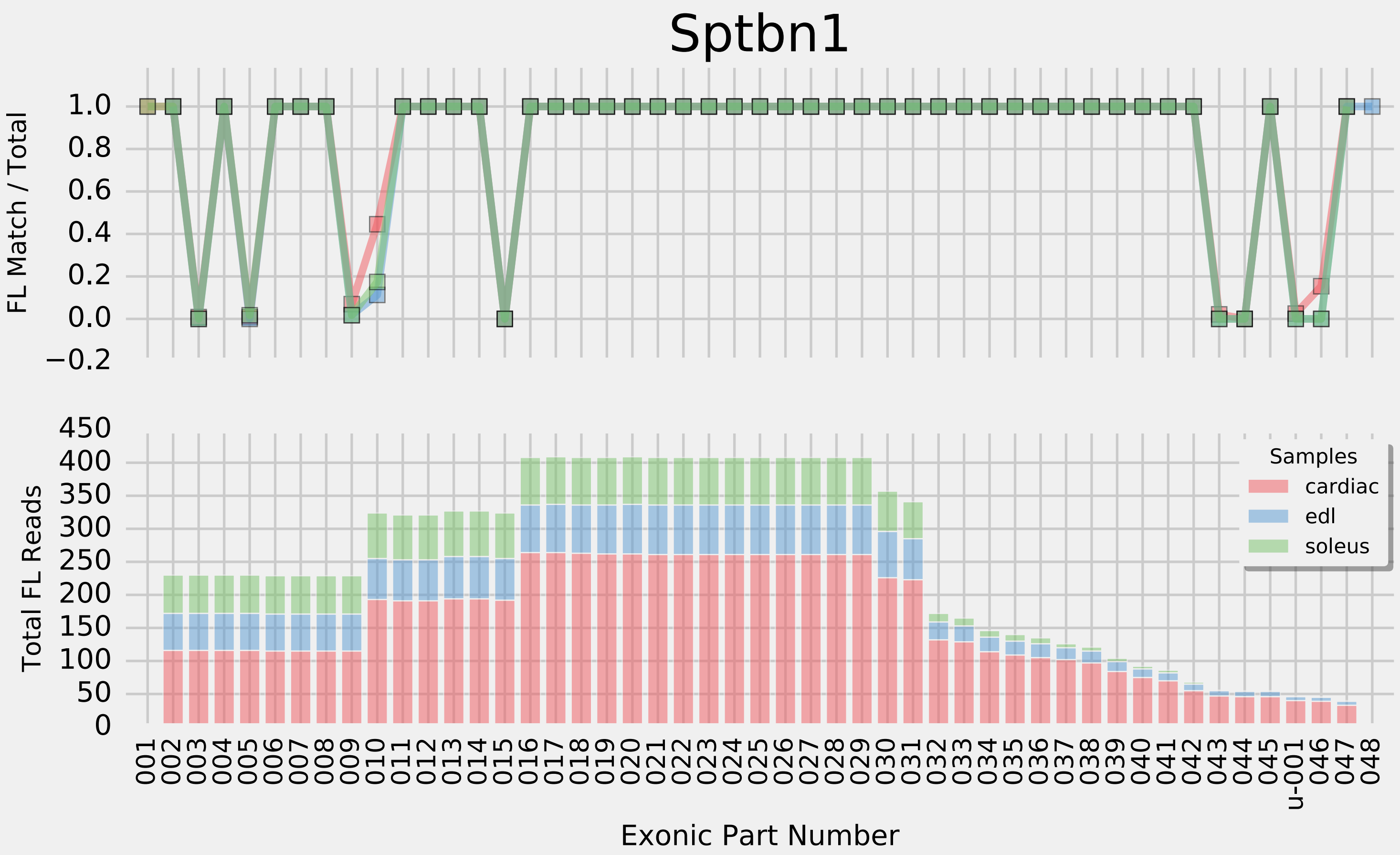
Sptbn1
FL Match / Total
1.0
0.8
0.6
0.4
0.2
0.0
−0.2
Total FL Reads
450
400
350
300
250
200
150
100
50
0
Samples
cardiac
edl
soleus
001 002 003 004 005 006 007 008 009 010 011 012 013 014 015 016 017 018 019 020 021 022 023 024 025 026 027 028 029 030 031 032 033 034 035 036 037 038 039 040 041 042 043 044 045 u-001 046 047 048
Exonic Part Number

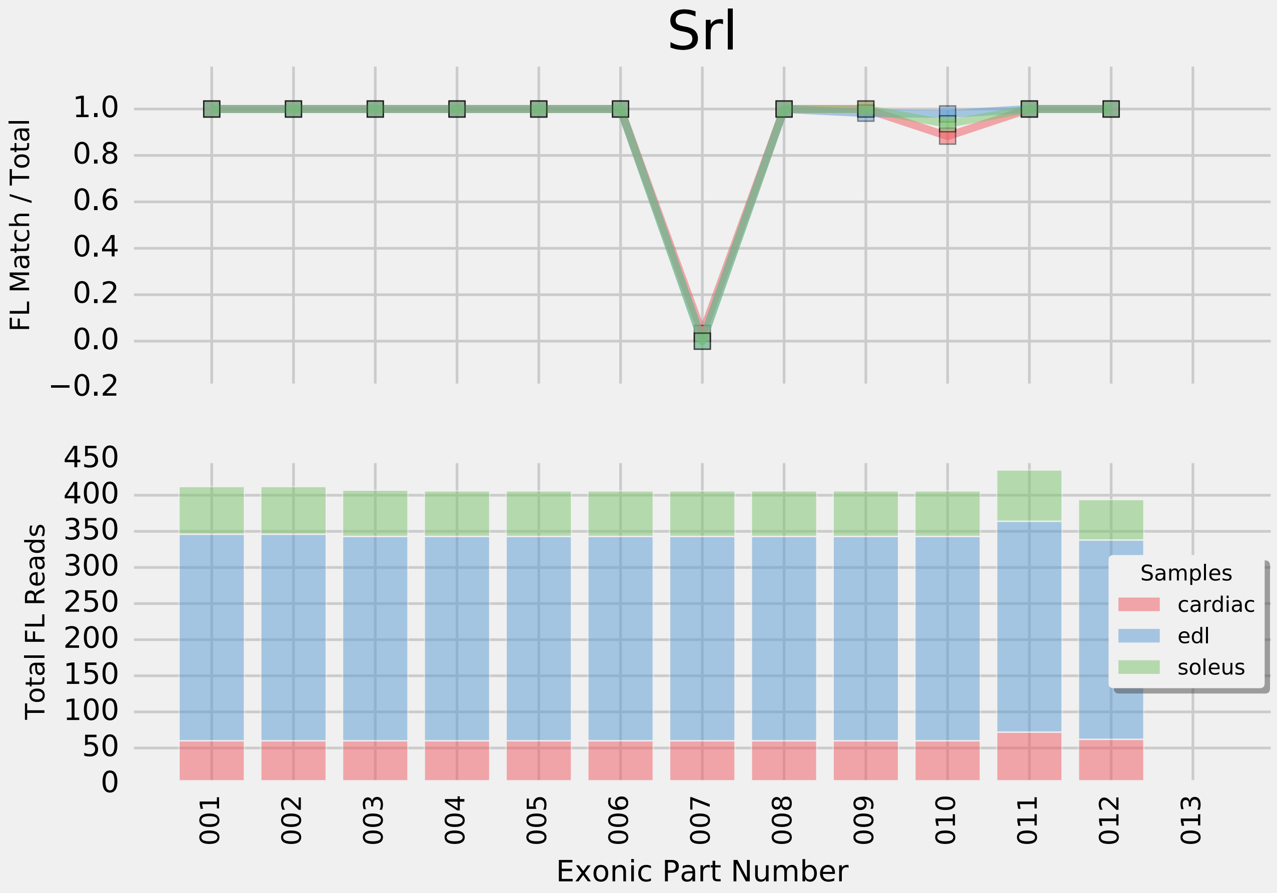

Srl
FL Match / Total
1.0
0.8
0.6
0.4
0.2
0.0
−0.2
Total FL Reads
450
400
350
300
250
200
150
100
50
0
Samples
cardiac
edl
soleus
001
002
003
004
005
006
007
008
009
010
011
012
013
Exonic Part Number

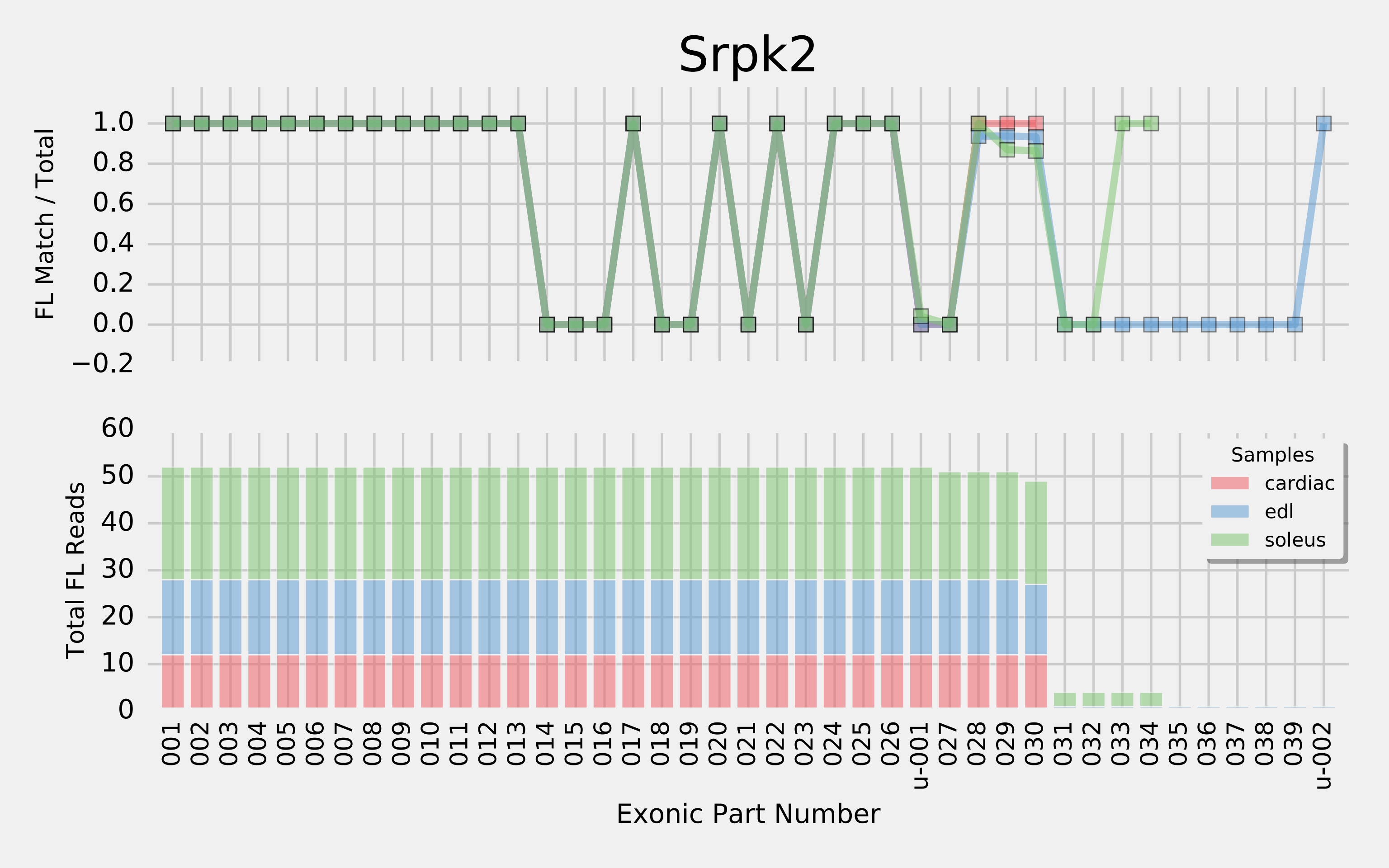

Srpk2
FL Match / Total
1.0
0.8
0.6
0.4
0.2
0.0
−0.2
Total FL Reads
60
50
40
30
20
10
0
Samples
cardiac
edl
soleus
001 002 003 004 005 006 007 008 009 010 011 012 013 014 015 016 017 018 019 020 021 022 023 024 025 026 u-001 027 028 029 030 031 032 033 034 035 036 037 038 039 u-002
Exonic Part Number

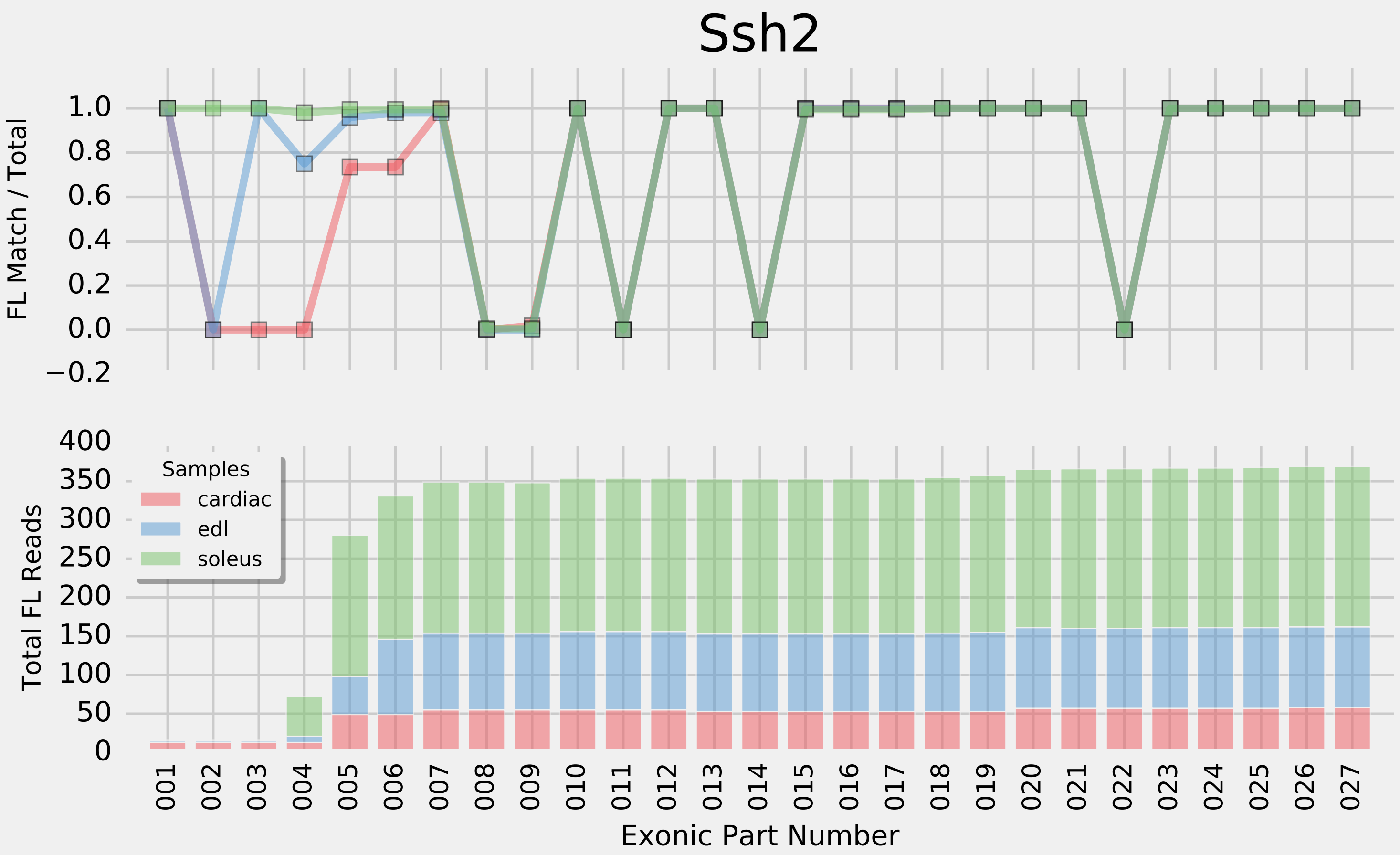
Ssh2
FL Match / Total
1.0
0.8
0.6
0.4
0.2
0.0
−0.2
Total FL Reads
400
350
300
250
200
150
100
50
0
Samples
cardiac
edl
soleus
001 002 003 004 005 006 007 008 009 010 011 012 013 014 015 016 017 018 019 020 021 022 023 024 025 026 027
Exonic Part Number

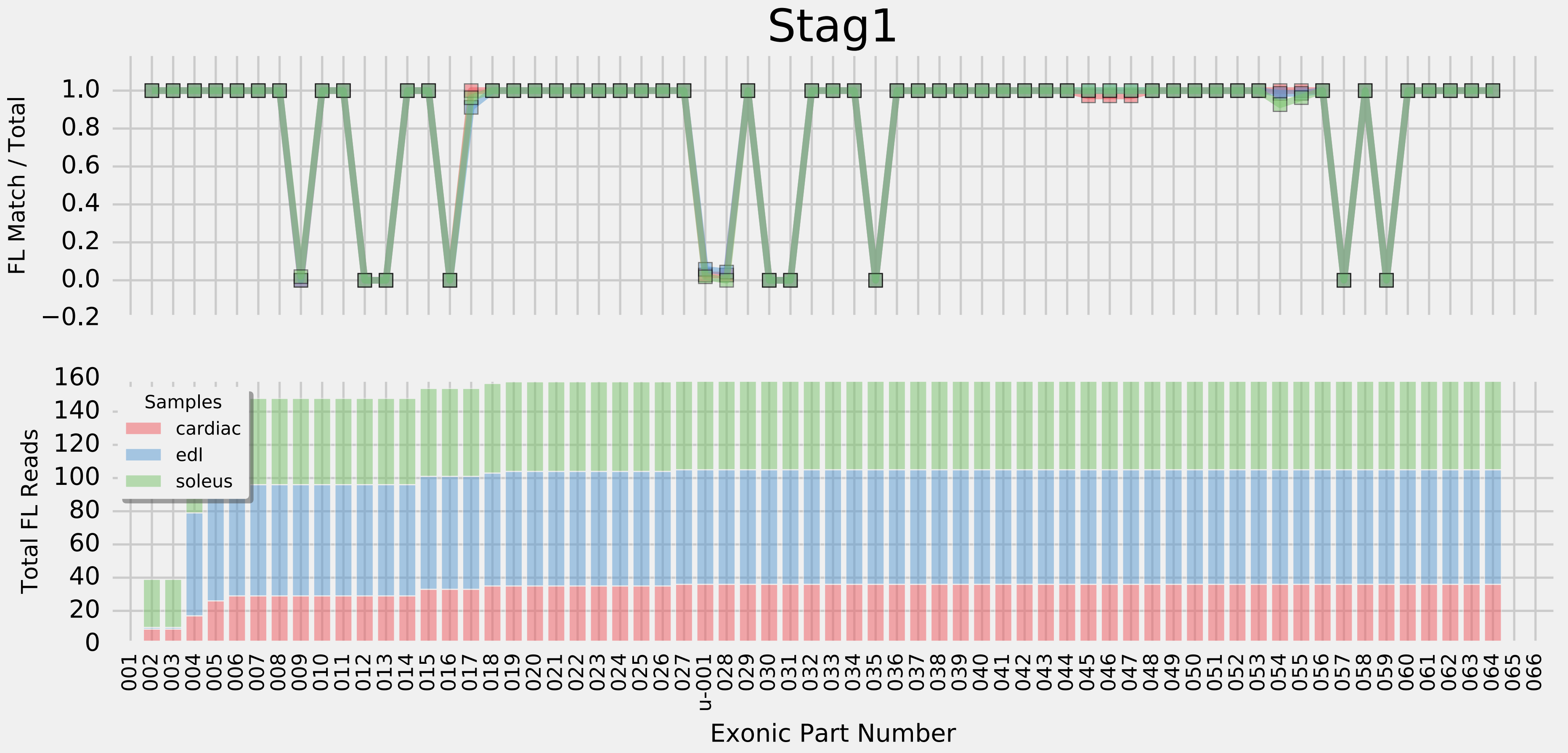

Stag1
FL Match / Total
1.0
0.8
0.6
0.4
0.2
0.0
−0.2
Total FL Reads
160
140
120
100
80
60
40
20
0
Samples
cardiac
edl
soleus
001 002 003 004 005 006 007 008 009 010 011 012 013 014 015 016 017 018 019 020 021 022 023 024 025 026 027 u-001 028 029 030 031 032 033 034 035 036 037 038 039 040 041 042 043 044 045 046 047 048 049 050 051 052 053 054 055 056 057 058 059 060 061 062 063 064 065 066
Exonic Part Number

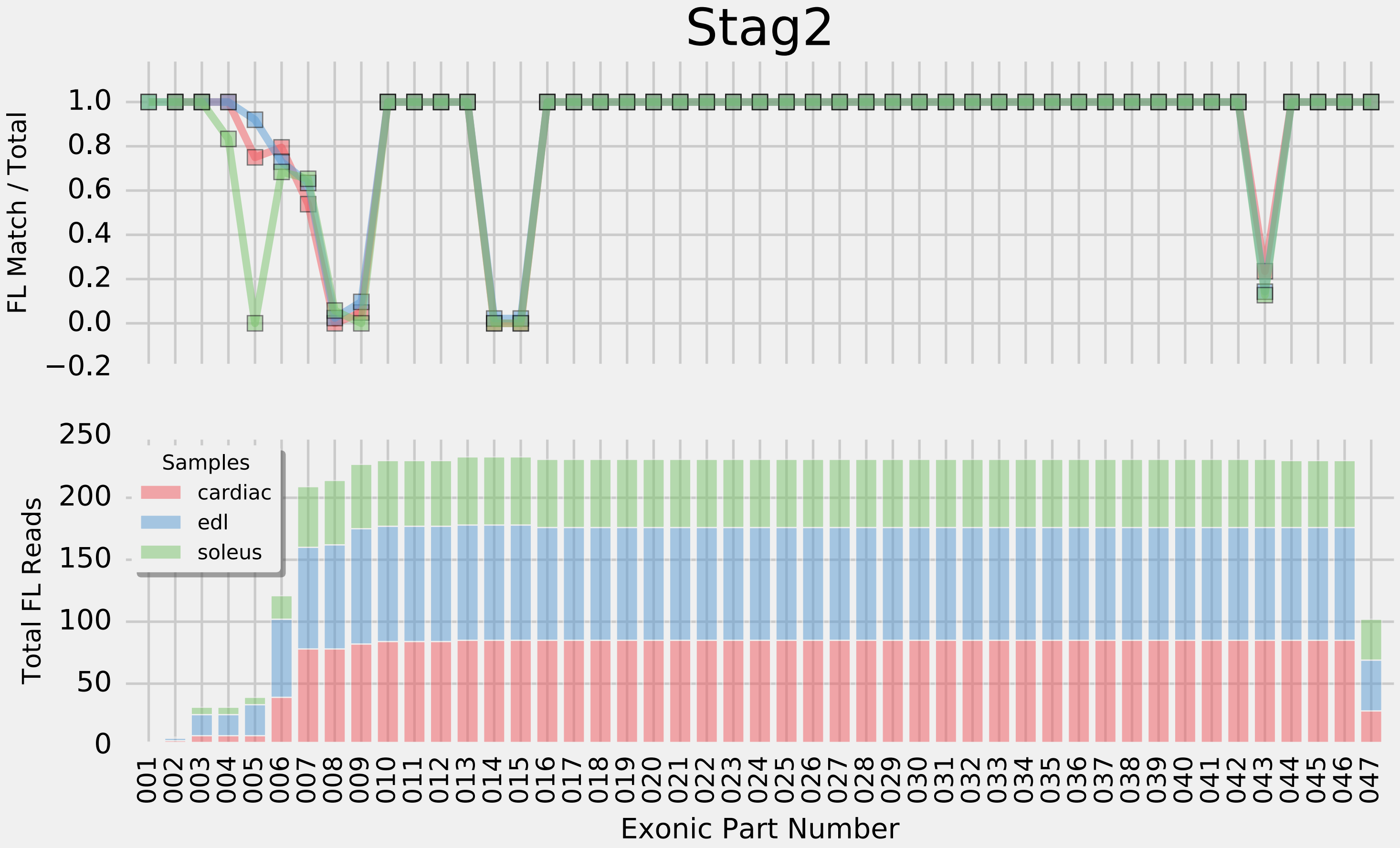

Stag2
FL Match / Total
1.0
0.8
0.6
0.4
0.2
0.0
−0.2
Total FL Reads
250
200
150
100
50
0
Samples
cardiac
edl
soleus
001 002 003 004 005 006 007 008 009 010 011 012 013 014 015 016 017 018 019 020 021 022 023 024 025 026 027 028 029 030 031 032 033 034 035 036 037 038 039 040 041 042 043 044 045 046 047
Exonic Part Number

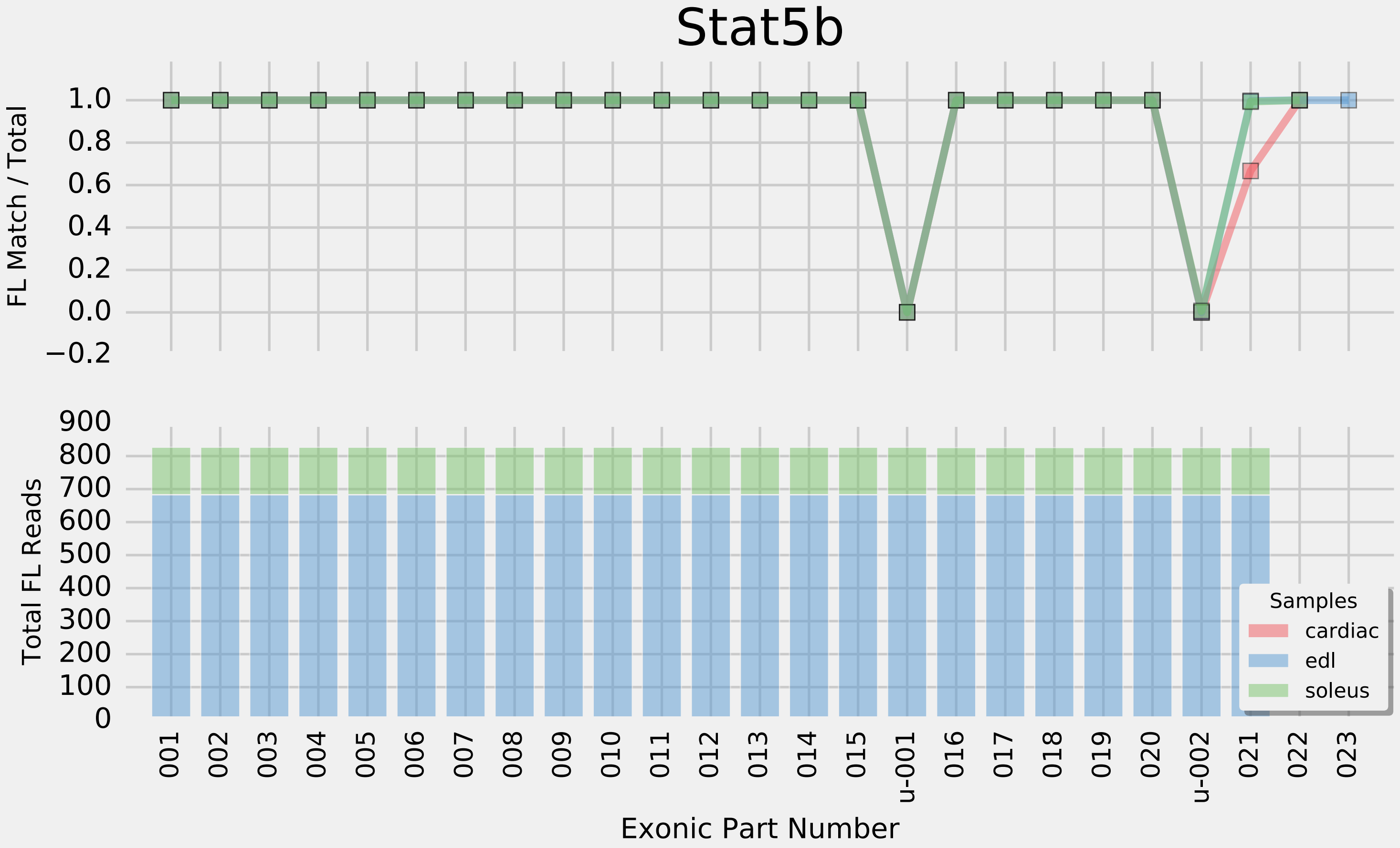

Stat5b
FL Match / Total
1.0
0.8
0.6
0.4
0.2
0.0
−0.2
900
800
700
600
500
400
300
200
100
0
Total FL Reads
Samples
cardiac
edl
soleus
001
002
003
004
005
006
007
008
009
010
011
012
013
014
015
u-001
016
017
018
019
020
u-002
021
022
023
Exonic Part Number

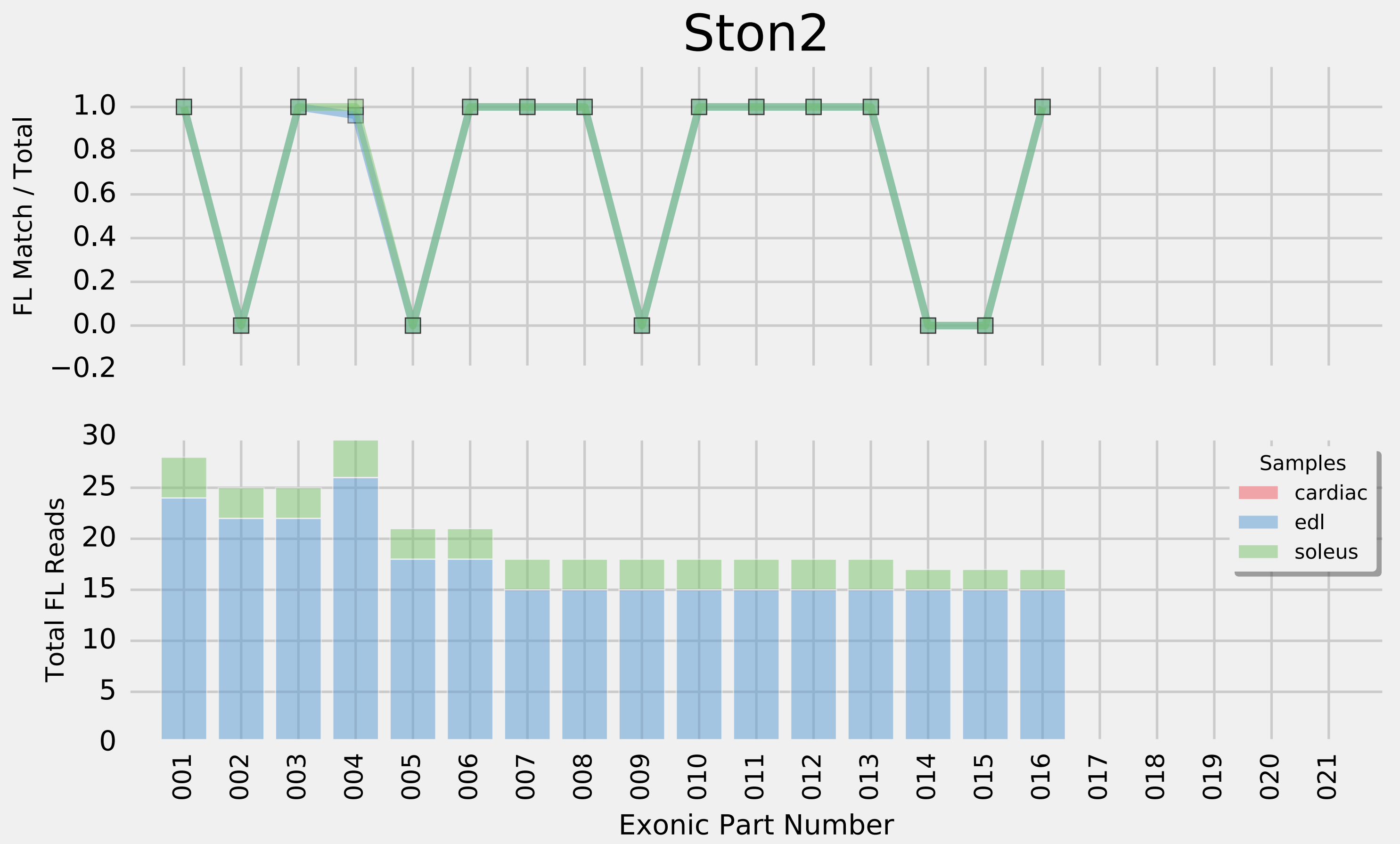

Ston2
FL Match / Total
1.0
0.8
0.6
0.4
0.2
0.0
−0.2
Total FL Reads
30
25
20
15
10
5
0
Samples
cardiac
edl
soleus
001
002
003
004
005
006
007
008
009
010
011
012
013
014
015
016
017
018
019
020
021
Exonic Part Number

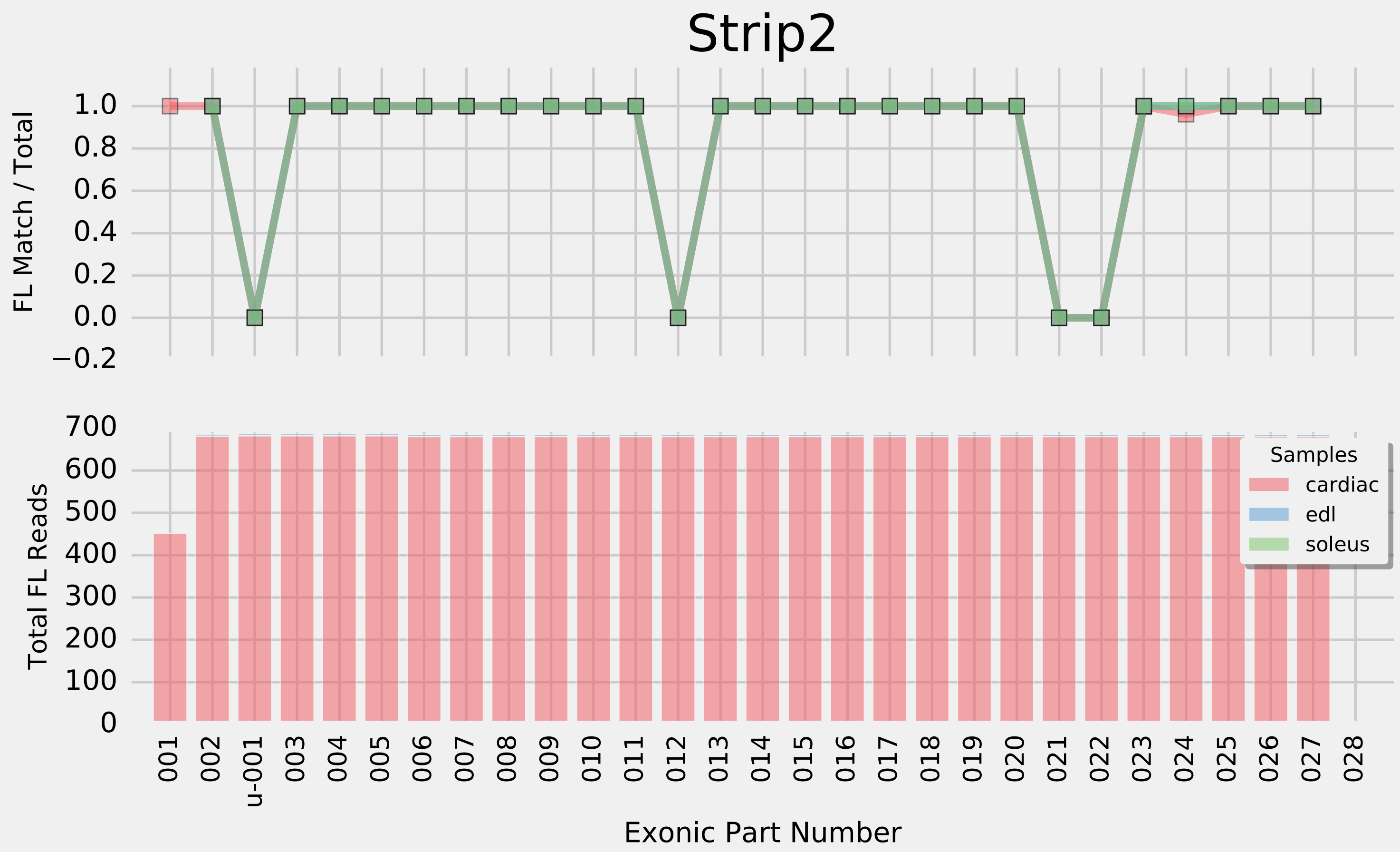

Strip2
FL Match / Total
1.0
0.8
0.6
0.4
0.2
0.0
−0.2
Total FL Reads
700
600
500
400
300
200
100
0
Samples
cardiac
edl
soleus
001
002
u-001
003
004
005
006
007
008
009
010
011
012
013
014
015
016
017
018
019
020
021
022
023
024
025
026
027
028
Exonic Part Number

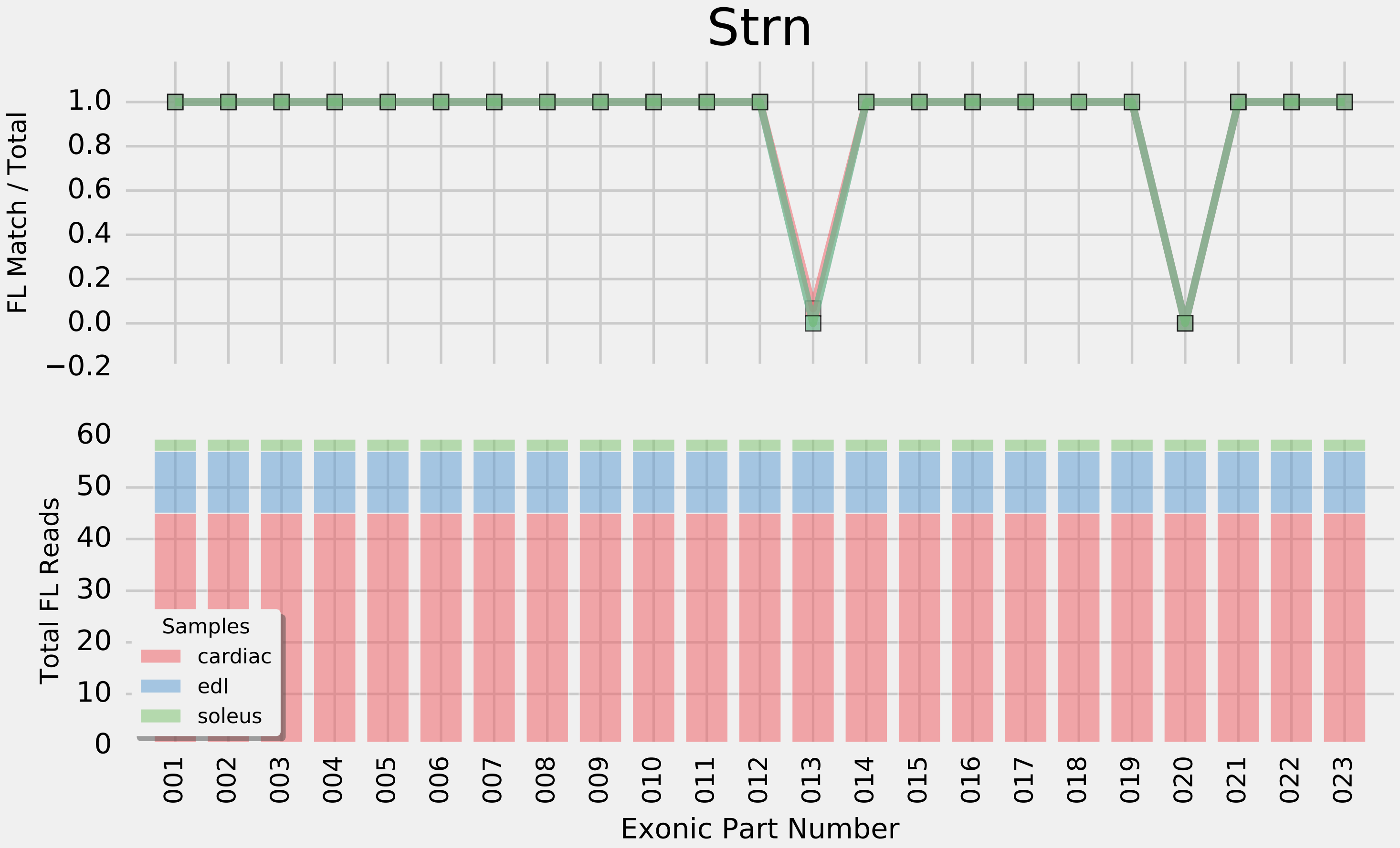

Strn
FL Match / Total
1.0
0.8
0.6
0.4
0.2
0.0
−0.2
Total FL Reads
60
50
40
30
20
10
0
Samples
cardiac
edl
soleus
001
002
003
004
005
006
007
008
009
010
011
012
013
014
015
016
017
018
019
020
021
022
023
Exonic Part Number

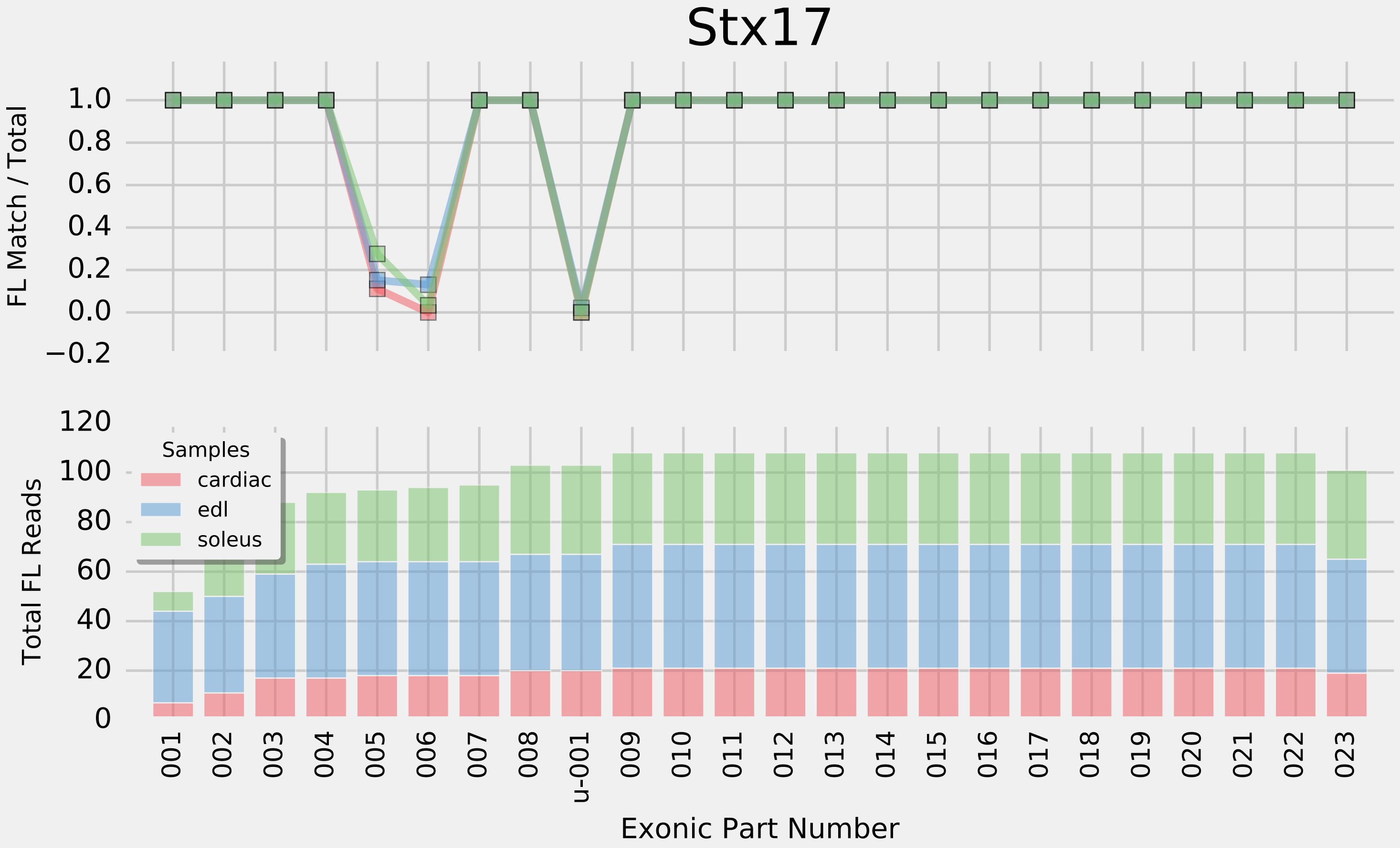

Stx17
FL Match / Total
1.0
0.8
0.6
0.4
0.2
0.0
−0.2
Total FL Reads
120
100
80
60
40
20
0
Samples
cardiac
edl
soleus
001
002
003
004
005
006
007
008
u-001
009
010
011
012
013
014
015
016
017
018
019
020
021
022
023
Exonic Part Number

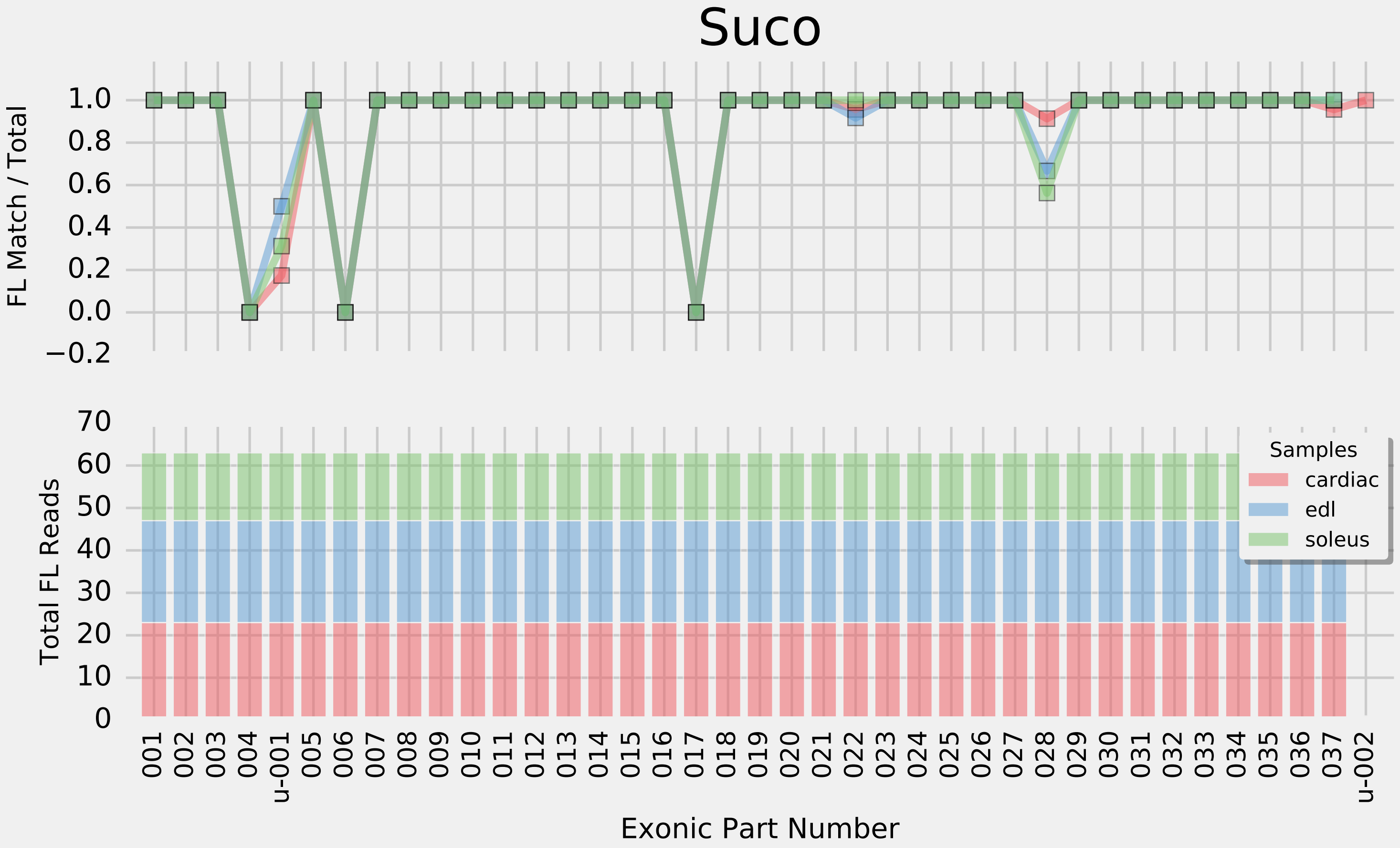

Suco
FL Match / Total
1.0
0.8
0.6
0.4
0.2
0.0
−0.2
Total FL Reads
70
60
50
40
30
20
10
0
Samples
cardiac
edl
soleus
001 002 003 004 u-001 005 006 007 008 009 010 011 012 013 014 015 016 017 018 019 020 021 022 023 024 025 026 027 028 029 030 031 032 033 034 035 036 037 u-002
Exonic Part Number

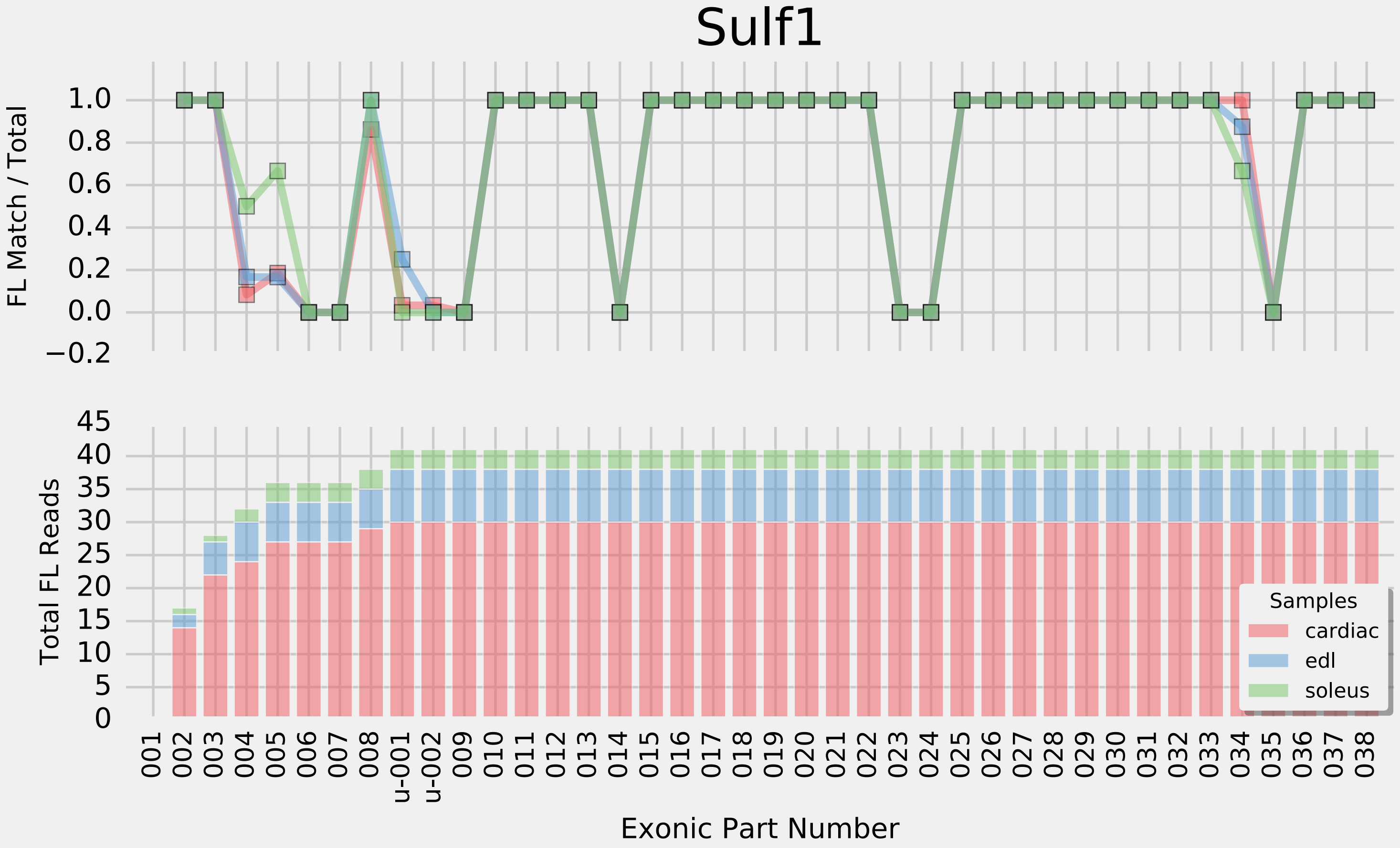

Sulf1
FL Match / Total
1.0
0.8
0.6
0.4
0.2
0.0
−0.2
Total FL Reads
45
40
35
30
25
20
15
10
5
0
Samples
cardiac
edl
soleus
001
002
003
004
005
006
007
008
u-001
u-002
009
010
011
012
013
014
015
016
017
018
019
020
021
022
023
024
025
026
027
028
029
030
031
032
033
034
035
036
037
038
Exonic Part Number

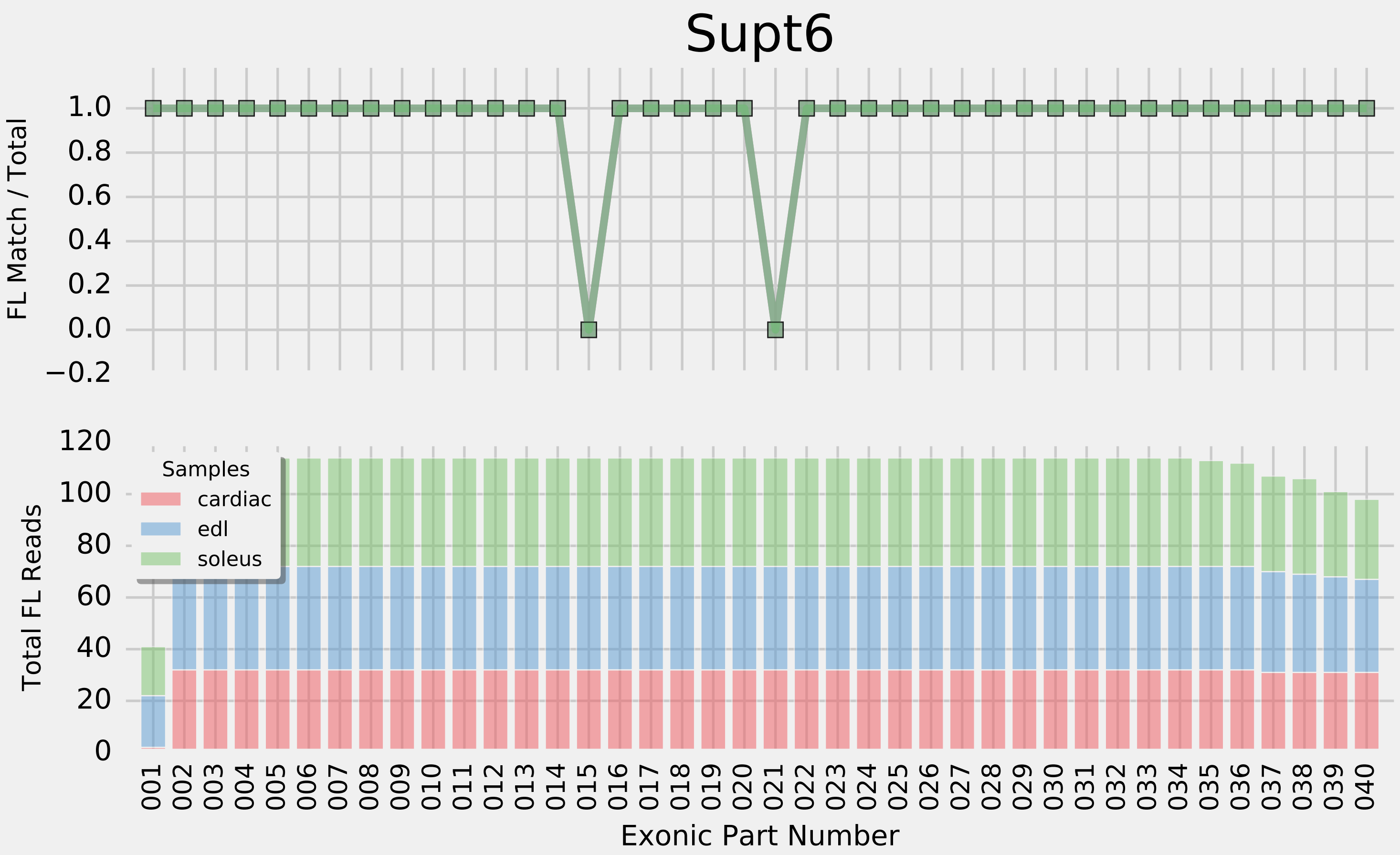

Supt6
FL Match / Total
1.0
0.8
0.6
0.4
0.2
0.0
−0.2
Total FL Reads
120
100
80
60
40
20
0
Samples
cardiac
edl
soleus
001
002
003
004
005
006
007
008
009
010
011
012
013
014
015
016
017
018
019
020
021
022
023
024
025
026
027
028
029
030
031
032
033
034
035
036
037
038
039
040
Exonic Part Number

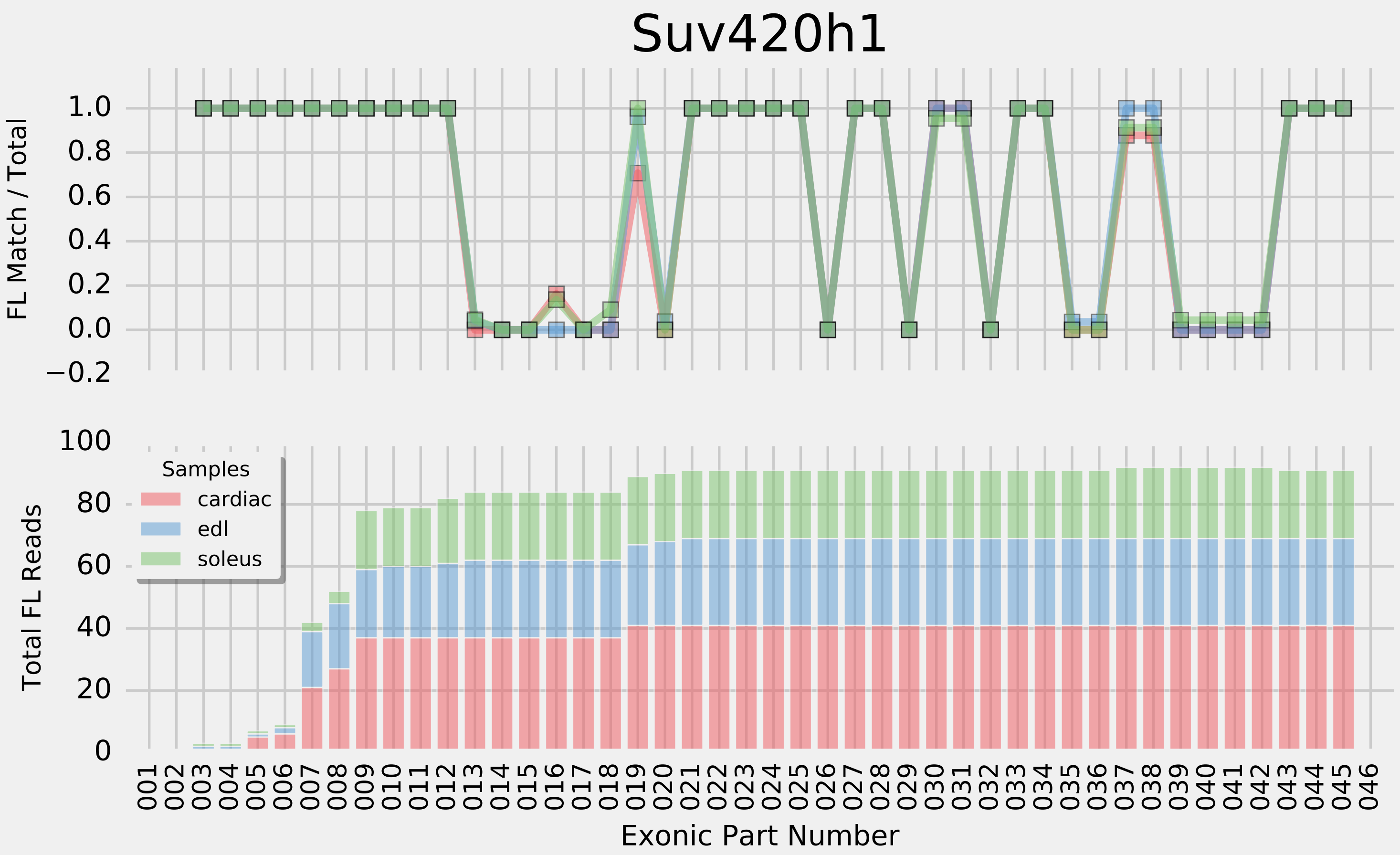

Suv420h1
FL Match / Total
1.0
0.8
0.6
0.4
0.2
0.0
−0.2
Total FL Reads
100
80
60
40
20
0
Samples
cardiac
edl
soleus
001
002
003
004
005
006
007
008
009
010
011
012
013
014
015
016
017
018
019
020
021
022
023
024
025
026
027
028
029
030
031
032
033
034
035
036
037
038
039
040
041
042
043
044
045
046
Exonic Part Number

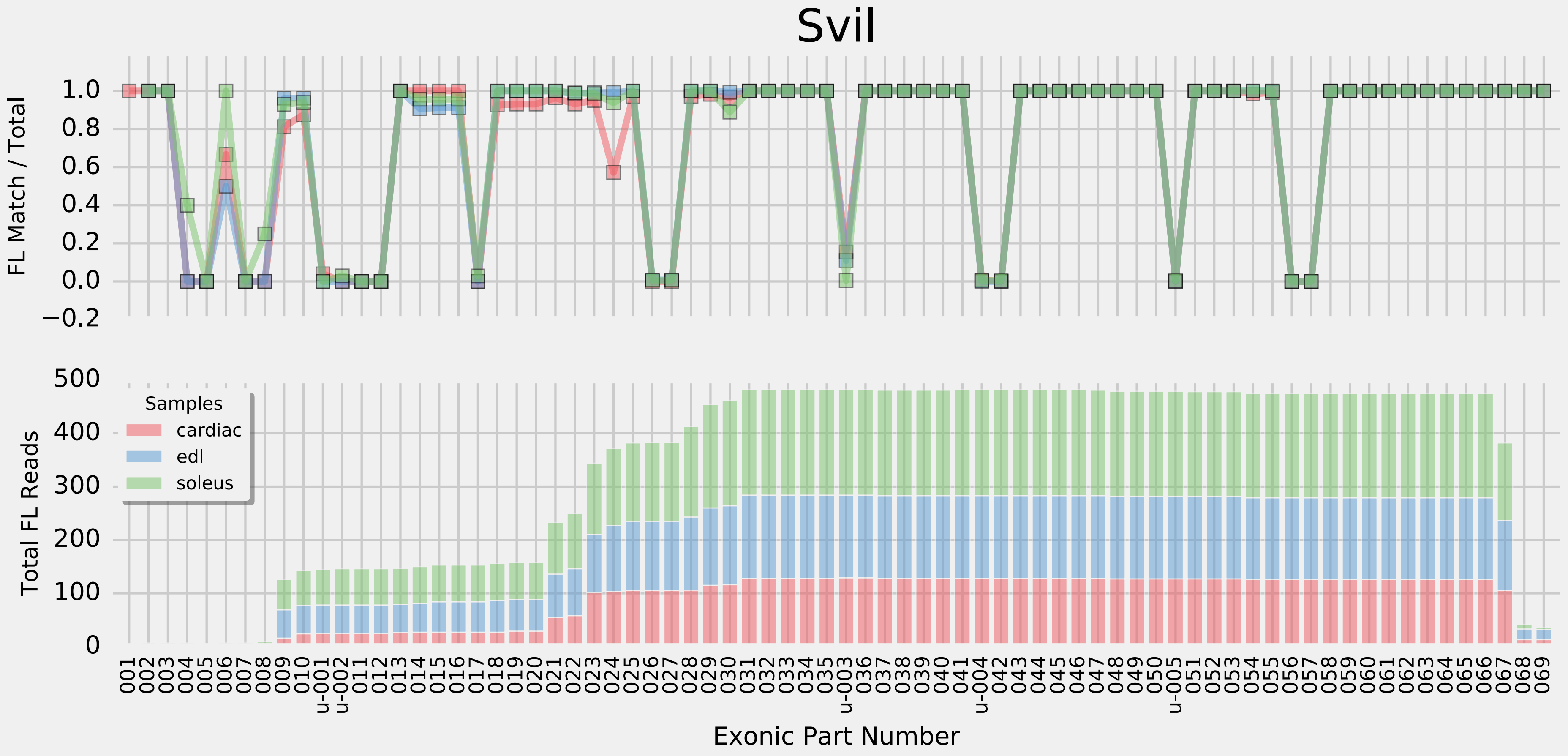

Svil
FL Match / Total
1.0
0.8
0.6
0.4
0.2
0.0
−0.2
Total FL Reads
500
400
300
200
100
0
Samples
cardiac
edl
soleus
001 002 003 004 005 006 007 008 009 010 u-001 u-002 011 012 013 014 015 016 017 018 019 020 021 022 023 024 025 026 027 028 029 030 031 032 033 034 035 u-003 036 037 038 039 040 041 u-004 042 043 044 045 046 047 048 049 050 u-005 051 052 053 054 055 056 057 058 059 060 061 062 063 064 065 066 067 068 069
Exonic Part Number

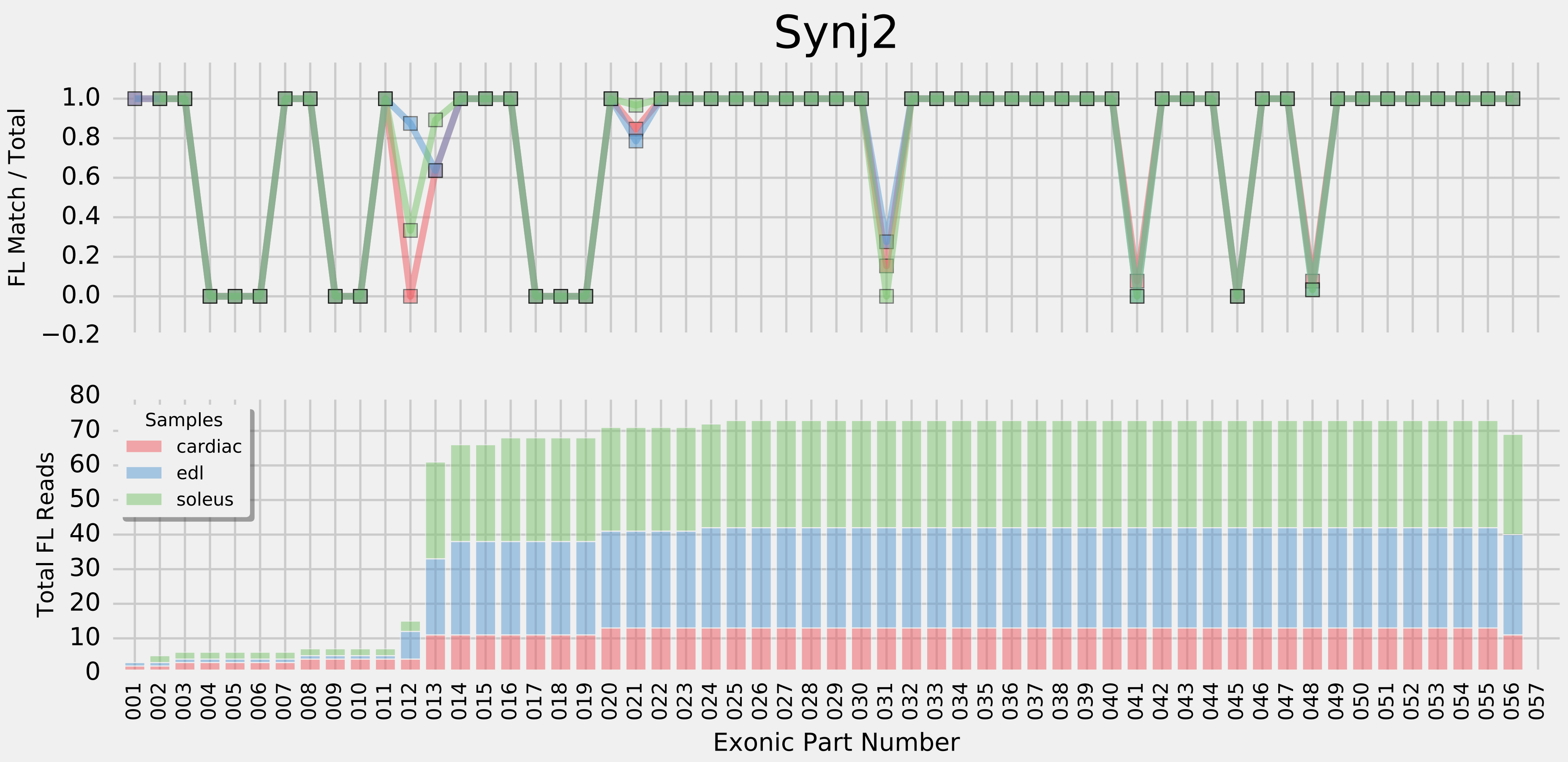
Synj2
FL Match / Total
Total FL Reads
Samples
cardiac
edl
soleus
Exonic Part Number

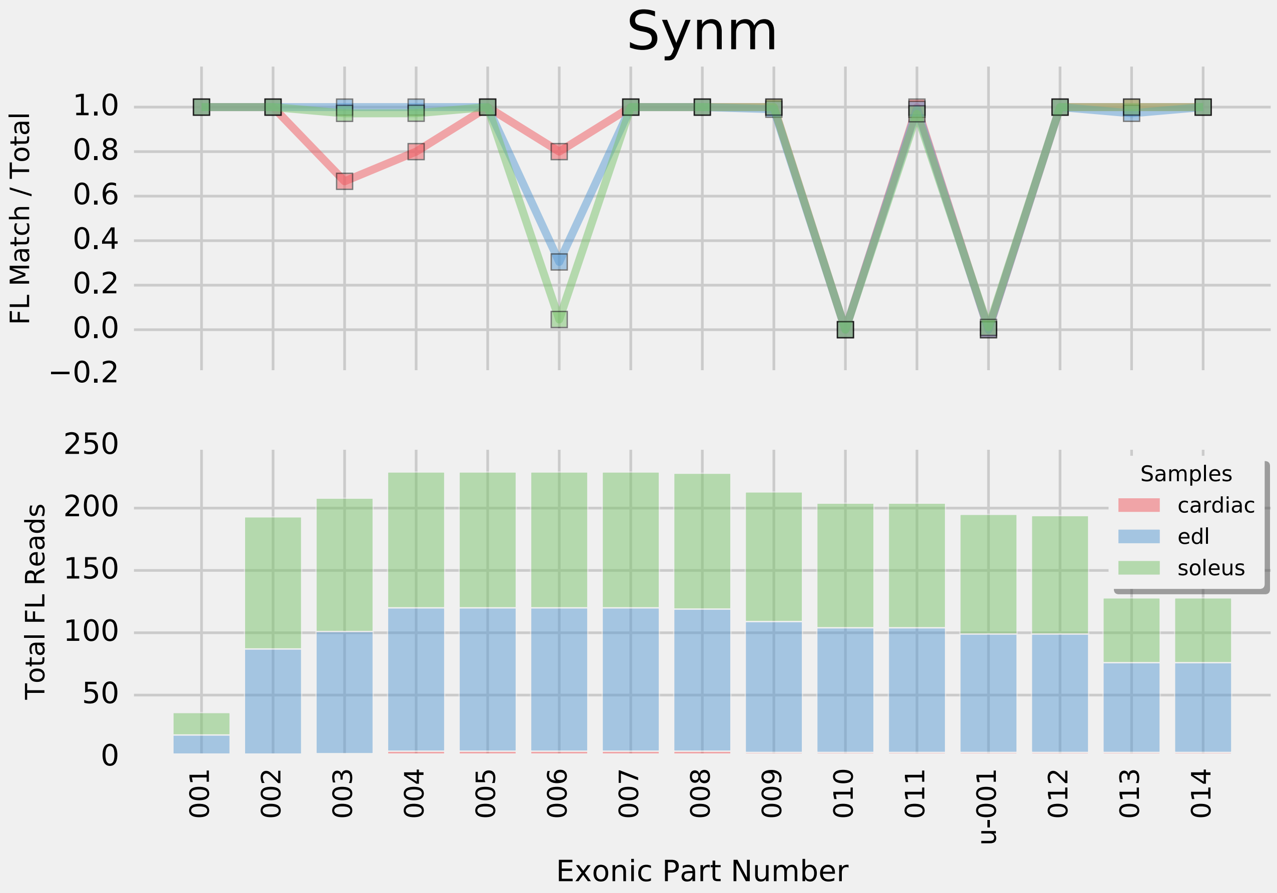

Synm
FL Match / Total
1.0
0.8
0.6
0.4
0.2
0.0
−0.2
250
200
150
100
50
0
Total FL Reads
Samples
cardiac
edl
soleus
001
002
003
004
005
006
007
008
009
010
011
u-001
012
013
014
Exonic Part Number

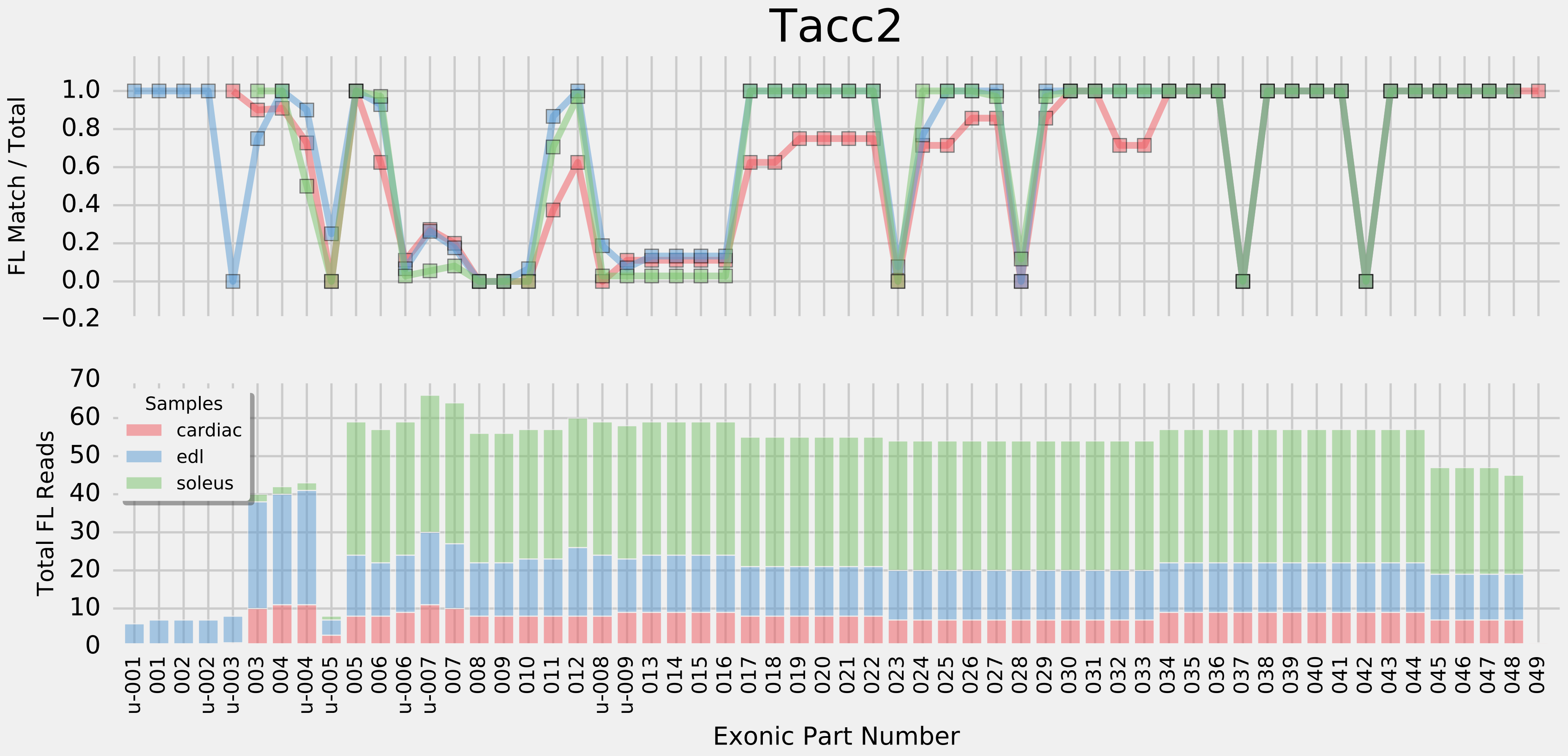

Tacc2
FL Match / Total
1.0
0.8
0.6
0.4
0.2
0.0
−0.2
Total FL Reads
70
60
50
40
30
20
10
0
Samples
cardiac
edl
soleus
u-001 001 002 u-002 u-003 003 004 u-004 u-005 005 006 u-006 u-007 007 008 009 010 011 012 u-008 u-009 013 014 015 016 017 018 019 020 021 022 023 024 025 026 027 028 029 030 031 032 033 034 035 036 037 038 039 040 041 042 043 044 045 046 047 048 049
Exonic Part Number

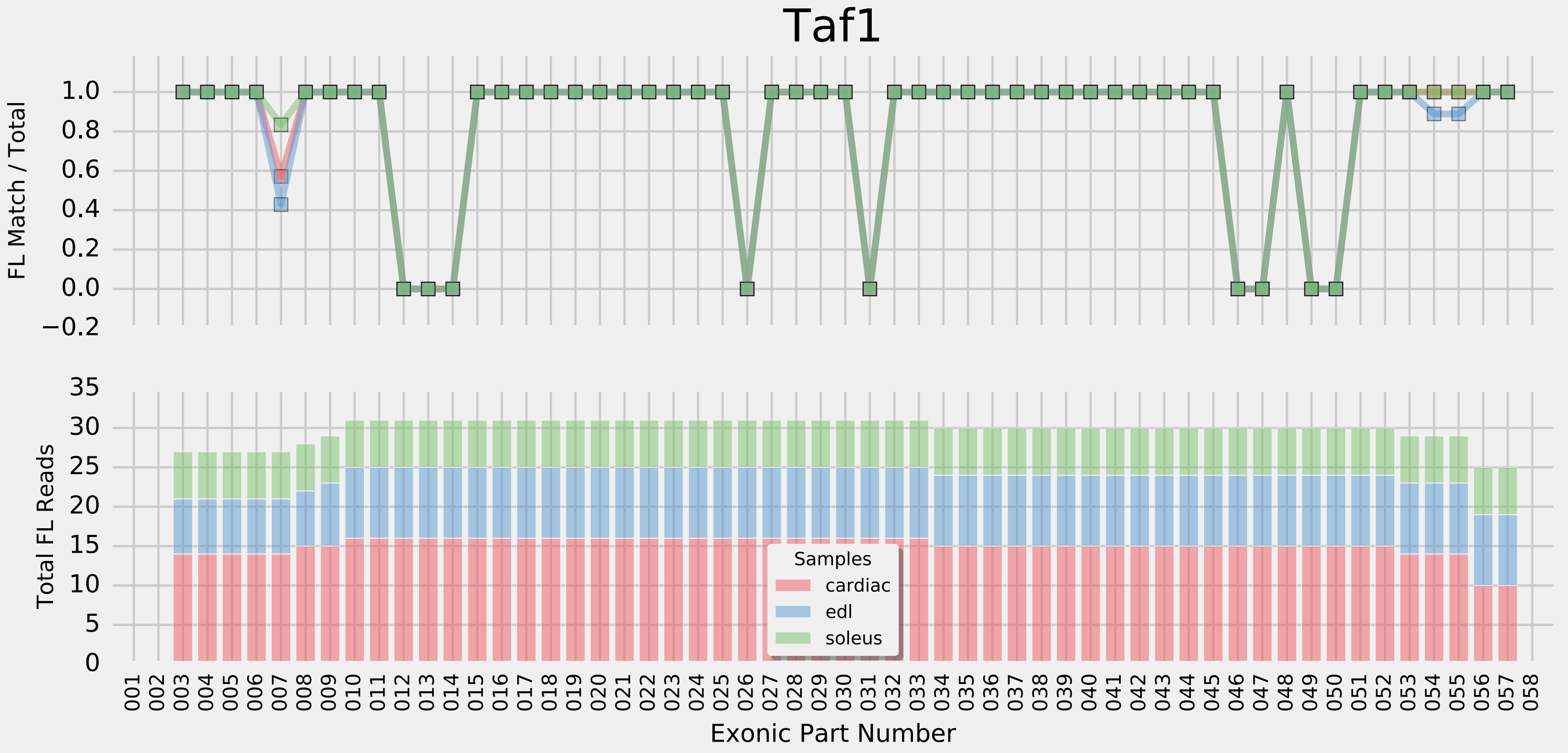

Taf1
FL Match / Total
1.0
0.8
0.6
0.4
0.2
0.0
−0.2
Total FL Reads
35
30
25
20
15
10
5
0
Samples
cardiac
edl
soleus
001 002 003 004 005 006 007 008 009 010 011 012 013 014 015 016 017 018 019 020 021 022 023 024 025 026 027 028 029 030 031 032 033 034 035 036 037 038 039 040 041 042 043 044 045 046 047 048 049 050 051 052 053 054 055 056 057 058
Exonic Part Number

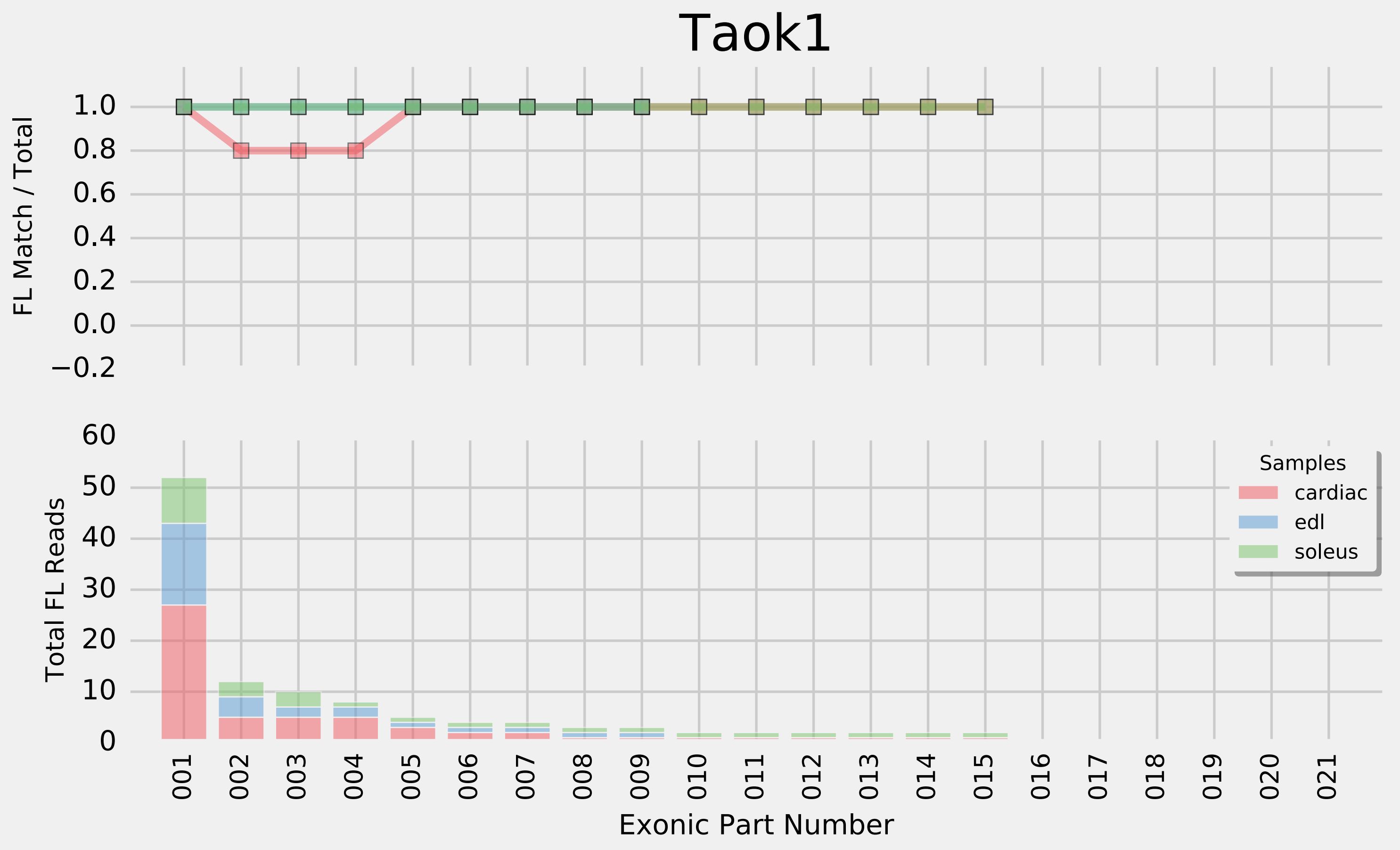

Taok1
FL Match / Total
1.0
0.8
0.6
0.4
0.2
0.0
−0.2
Total FL Reads
60
50
40
30
20
10
0
Samples
cardiac
edl
soleus
001
002
003
004
005
006
007
008
009
010
011
012
013
014
015
016
017
018
019
020
021
Exonic Part Number

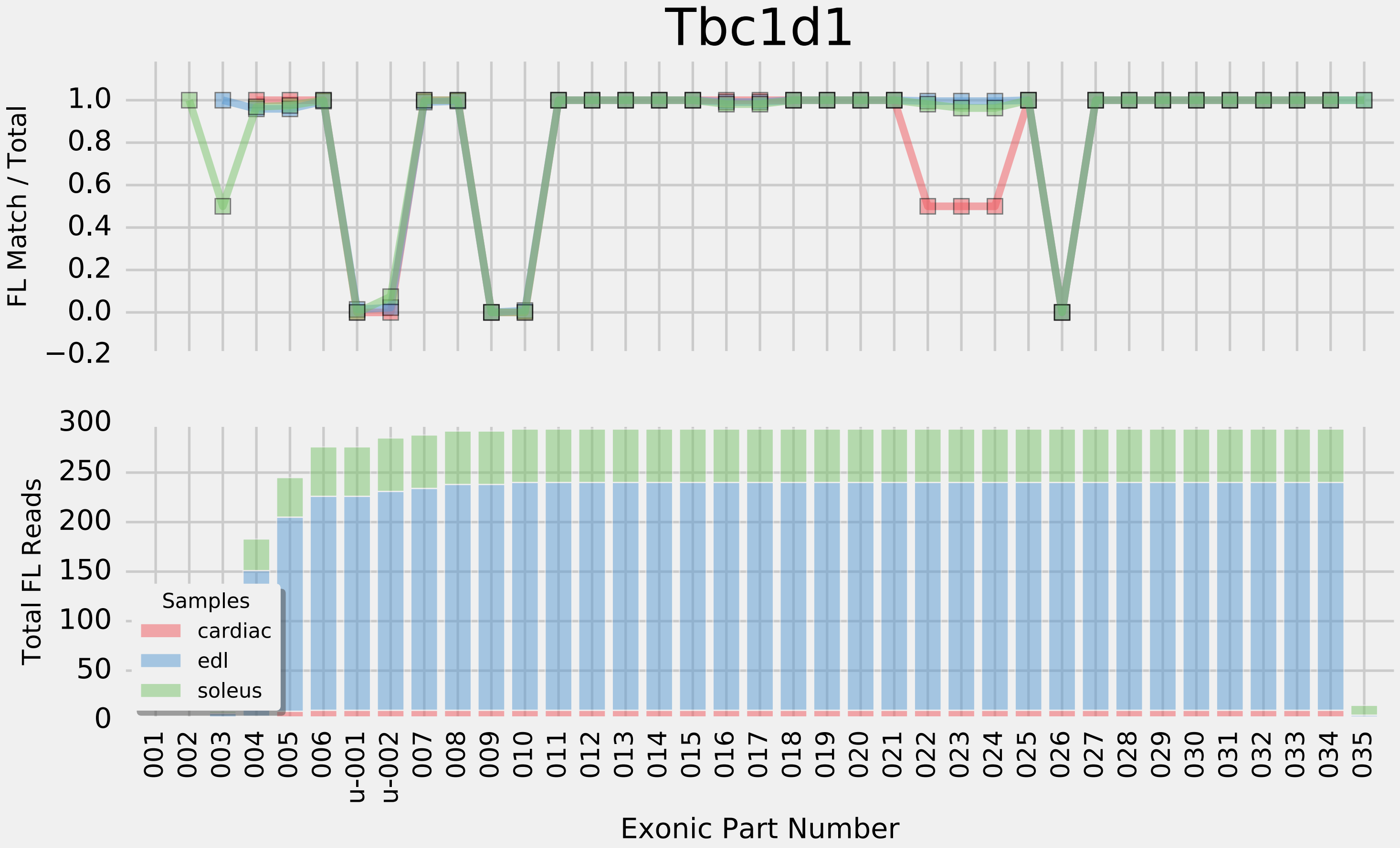

Tbc1d1
FL Match / Total
1.0
0.8
0.6
0.4
0.2
0.0
−0.2
Total FL Reads
300
250
200
150
100
50
0
Samples
cardiac
edl
soleus
001
002
003
004
005
006
u-001
u-002
007
008
009
010
011
012
013
014
015
016
017
018
019
020
021
022
023
024
025
026
027
028
029
030
031
032
033
034
035
Exonic Part Number

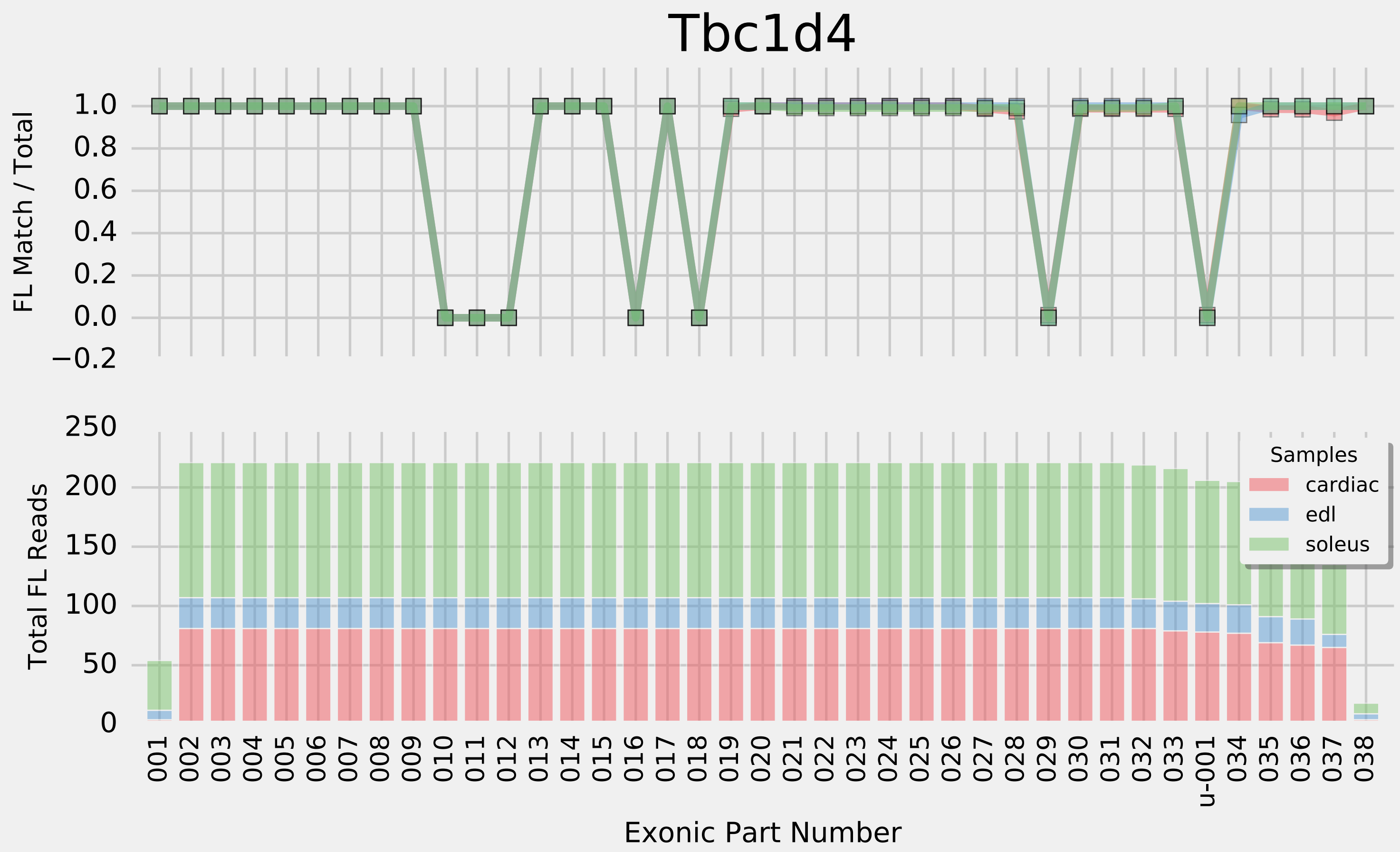
Tbc1d4
FL Match / Total
1.0
0.8
0.6
0.4
0.2
0.0
−0.2
250
200
150
100
50
0
Total FL Reads
Samples
cardiac
edl
soleus
001
002
003
004
005
006
007
008
009
010
011
012
013
014
015
016
017
018
019
020
021
022
023
024
025
026
027
028
029
030
031
032
033
u-001
034
035
036
037
038
Exonic Part Number

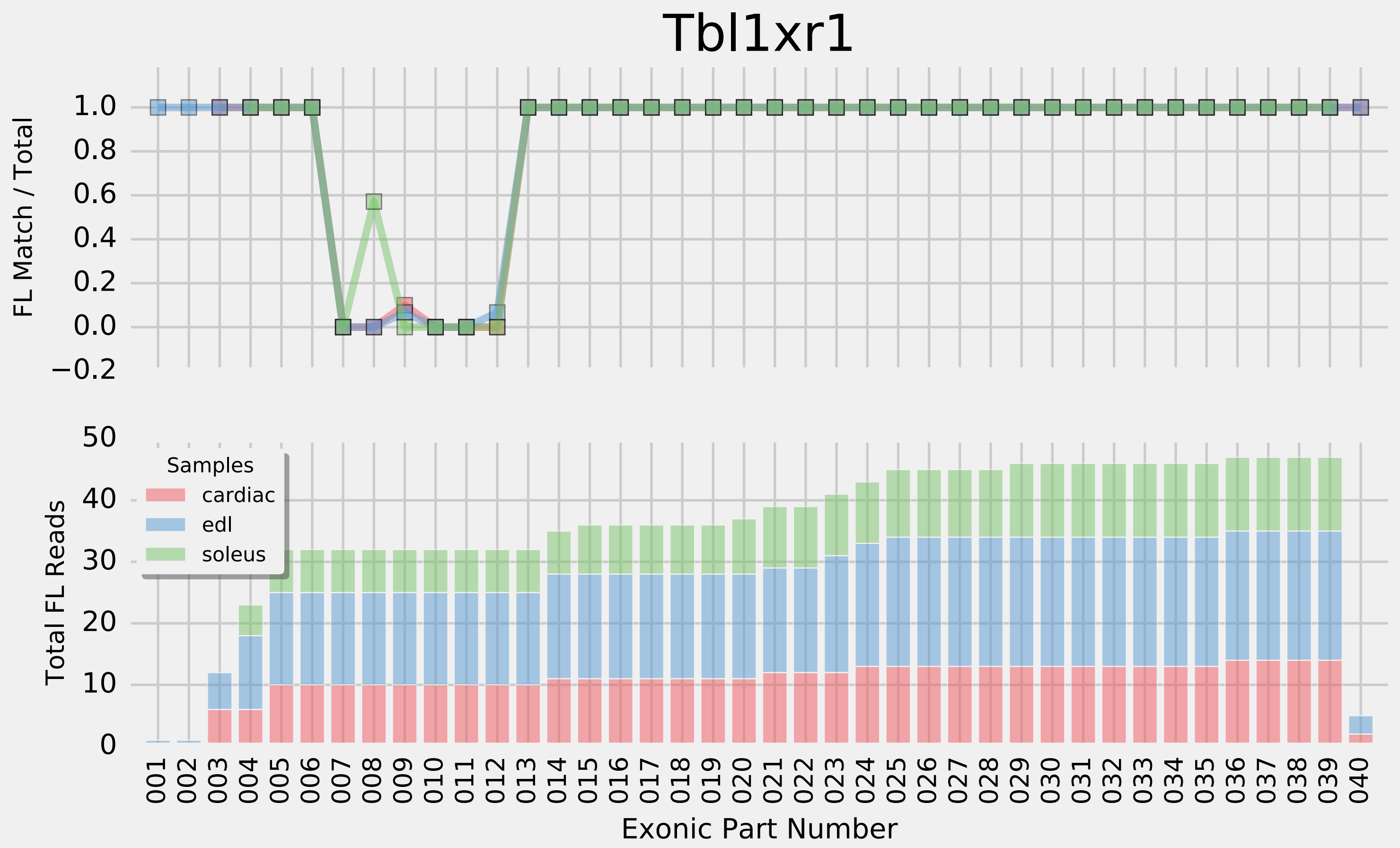

Tbl1xr1
FL Match / Total
1.0
0.8
0.6
0.4
0.2
0.0
−0.2
Total FL Reads
50
40
30
20
10
0
Samples
cardiac
edl
soleus
001
002
003
004
005
006
007
008
009
010
011
012
013
014
015
016
017
018
019
020
021
022
023
024
025
026
027
028
029
030
031
032
033
034
035
036
037
038
039
040
Exonic Part Number

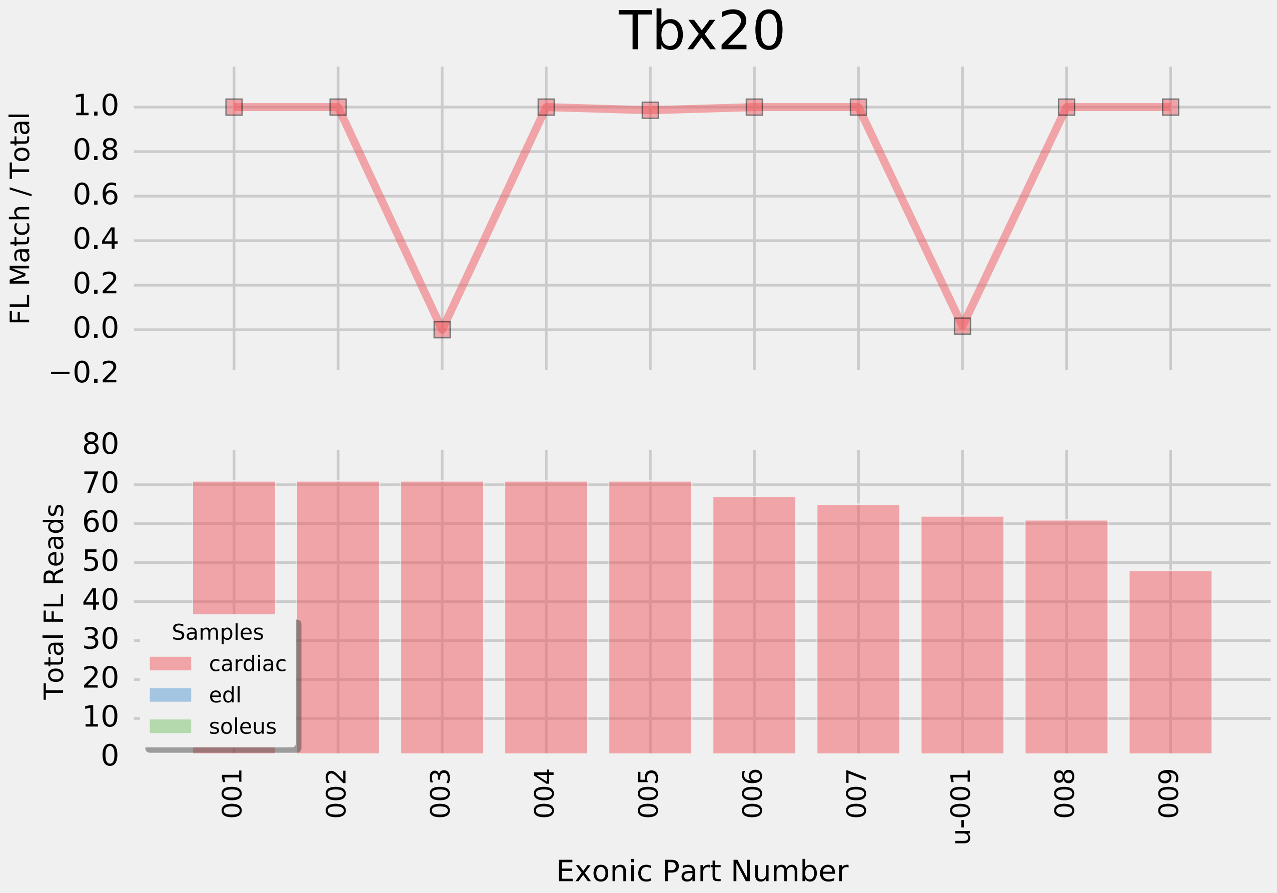

Tbx20
FL Match / Total
1.0
0.8
0.6
0.4
0.2
0.0
−0.2
Total FL Reads
80
70
60
50
40
30
20
10
0
Samples
cardiac
edl
soleus
001
002
003
004
005
006
007
u-001
008
009
Exonic Part Number

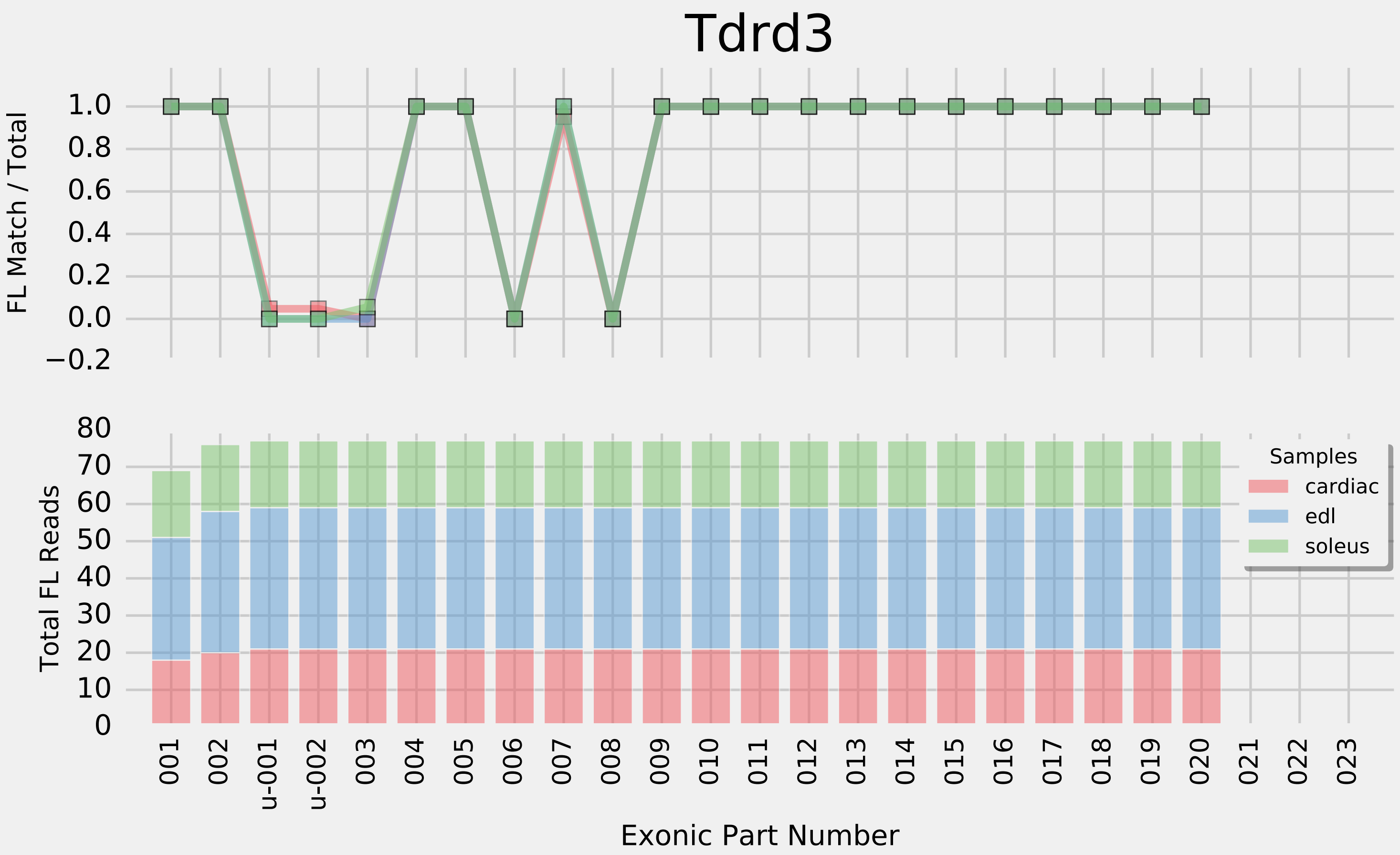

Tdrd3
FL Match / Total
1.0
0.8
0.6
0.4
0.2
0.0
−0.2
Total FL Reads
80
70
60
50
40
30
20
10
0
Samples
cardiac
edl
soleus
001
002
u-001
u-002
003
004
005
006
007
008
009
010
011
012
013
014
015
016
017
018
019
020
021
022
023
Exonic Part Number

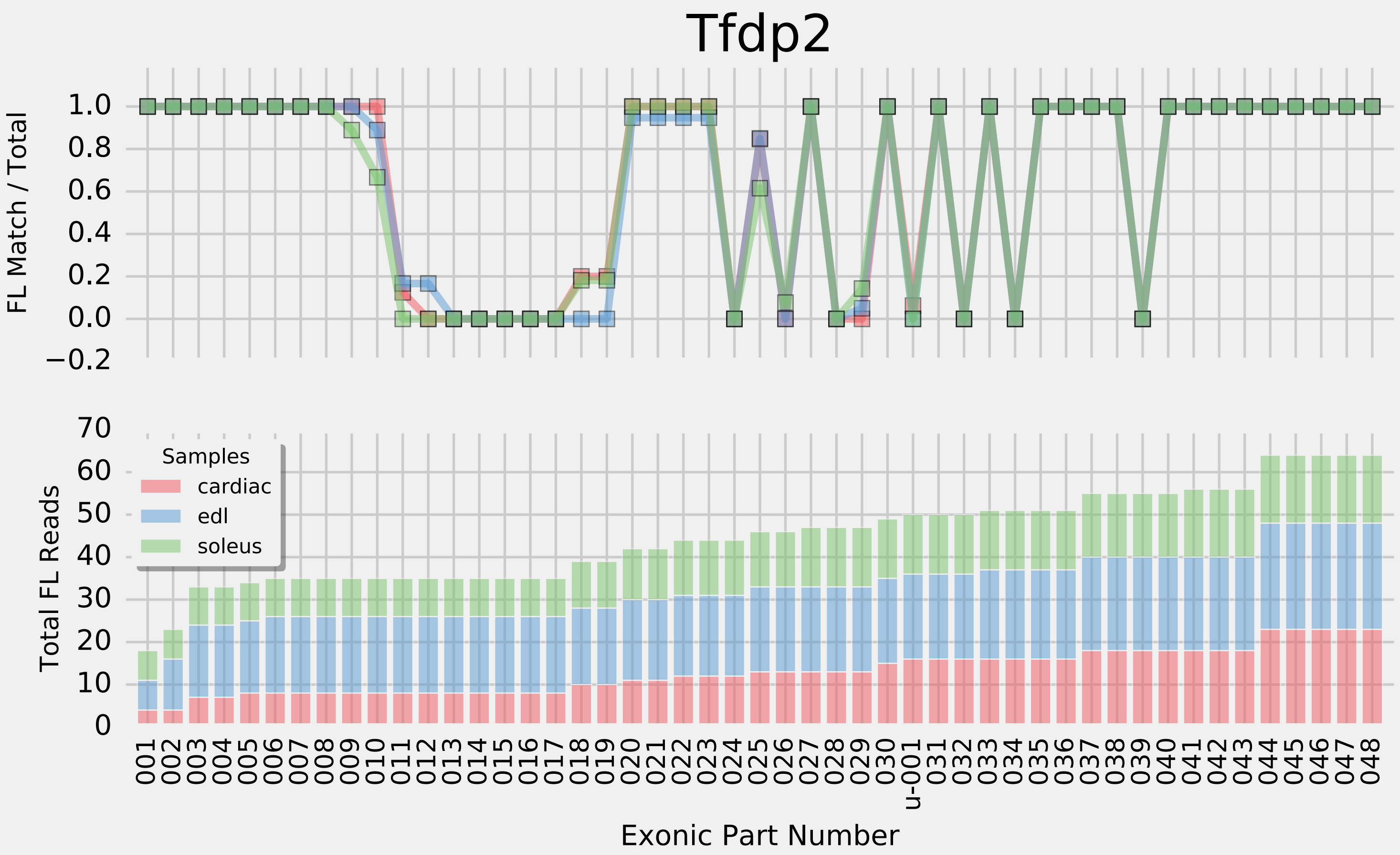

Tfdp2
FL Match / Total
Total FL Reads
Samples
cardiac
edl
soleus
Exonic Part Number
001
002
003
004
005
006
007
008
009
010
011
012
013
014
015
016
017
018
019
020
021
022
023
024
025
026
027
028
029
030
u-001
031
032
033
034
035
036
037
038
039
040
041
042
043
044
045
046
047
048

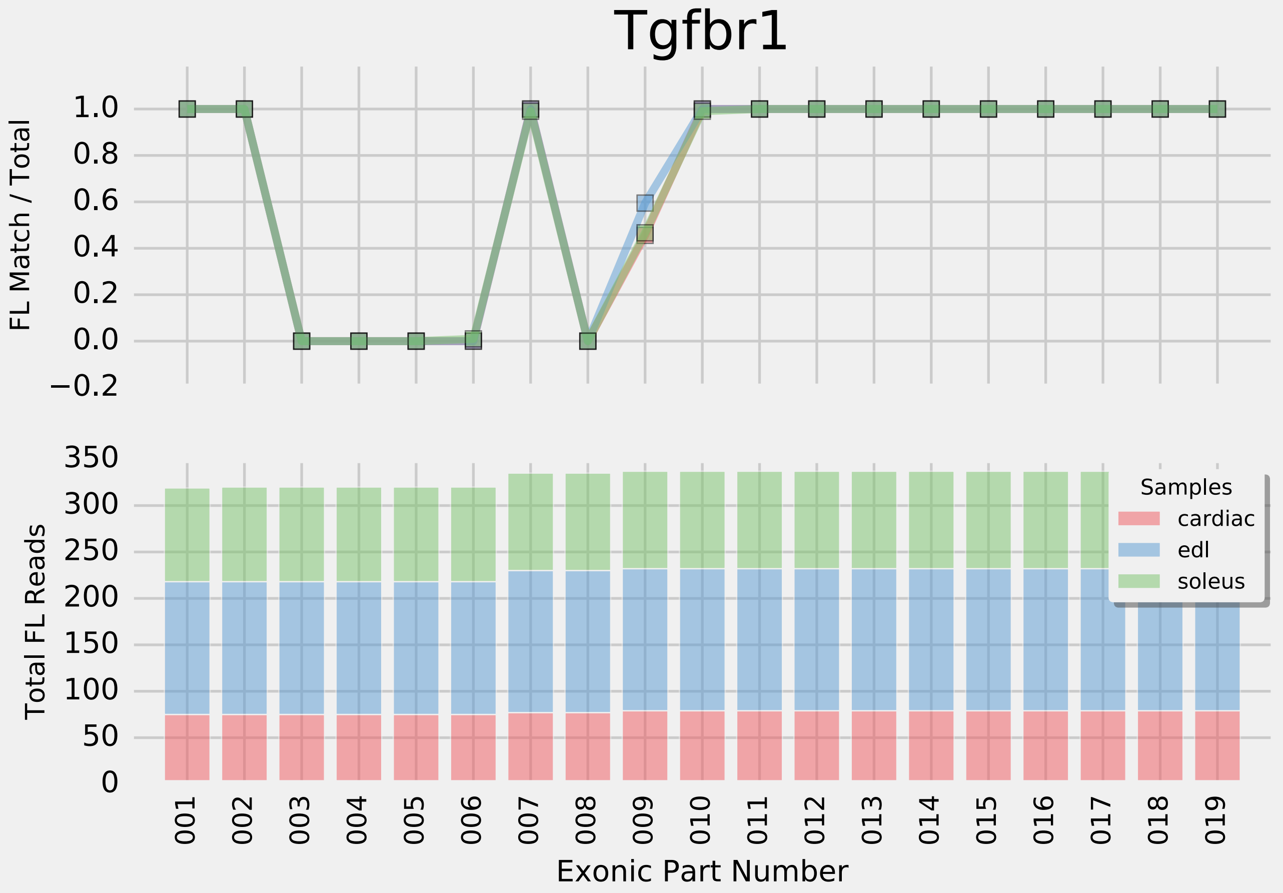

Tgfbr1
FL Match / Total
1.0
0.8
0.6
0.4
0.2
0.0
−0.2
Total FL Reads
350
300
250
200
150
100
50
0
Samples
cardiac
edl
soleus
001
002
003
004
005
006
007
008
009
010
011
012
013
014
015
016
017
018
019
Exonic Part Number

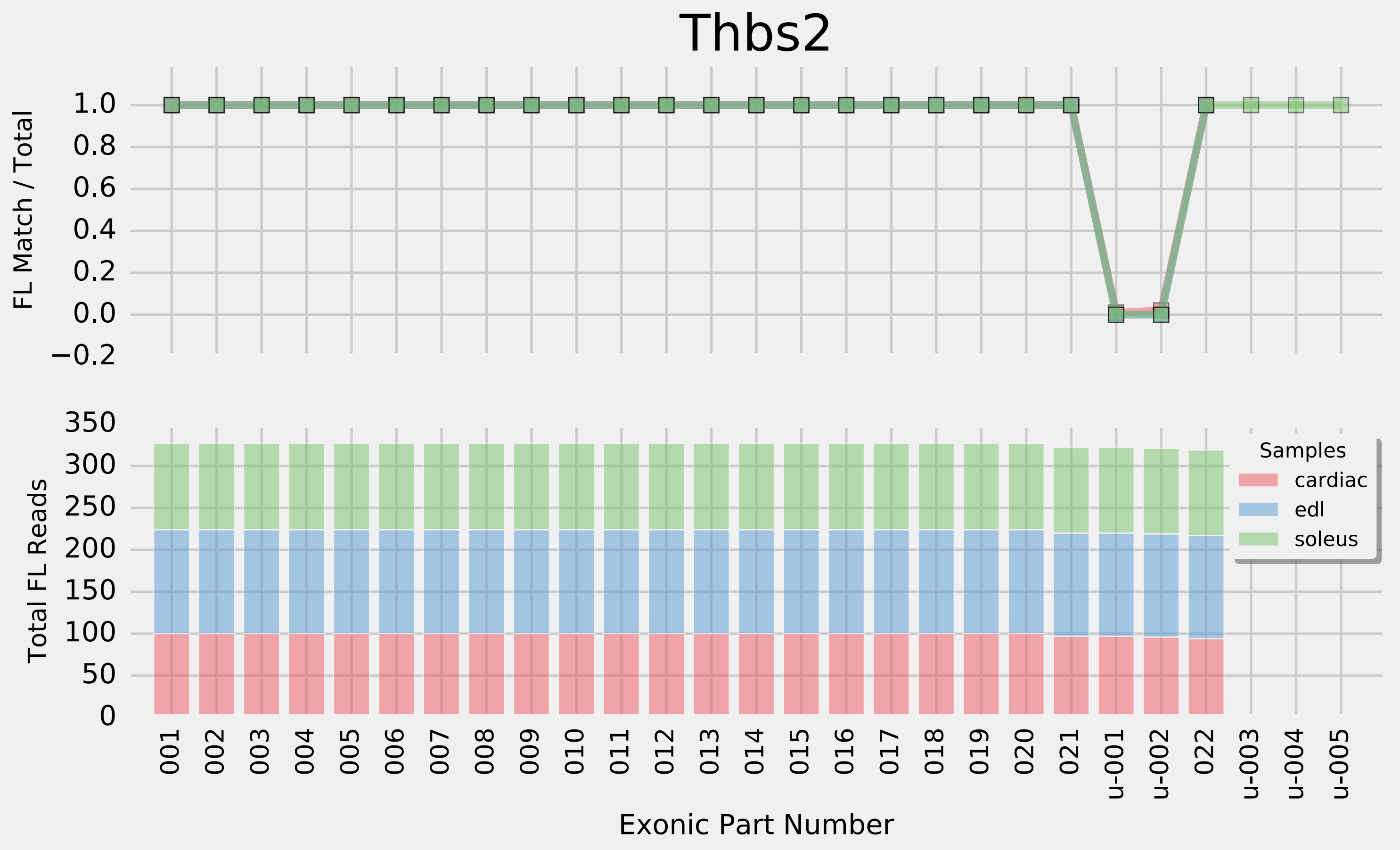

Thbs2
FL Match / Total
1.0
0.8
0.6
0.4
0.2
0.0
−0.2
Total FL Reads
350
300
250
200
150
100
50
0
Samples
cardiac
edl
soleus
001
002
003
004
005
006
007
008
009
010
011
012
013
014
015
016
017
018
019
020
021
u-001
u-002
022
u-003
u-004
u-005
Exonic Part Number

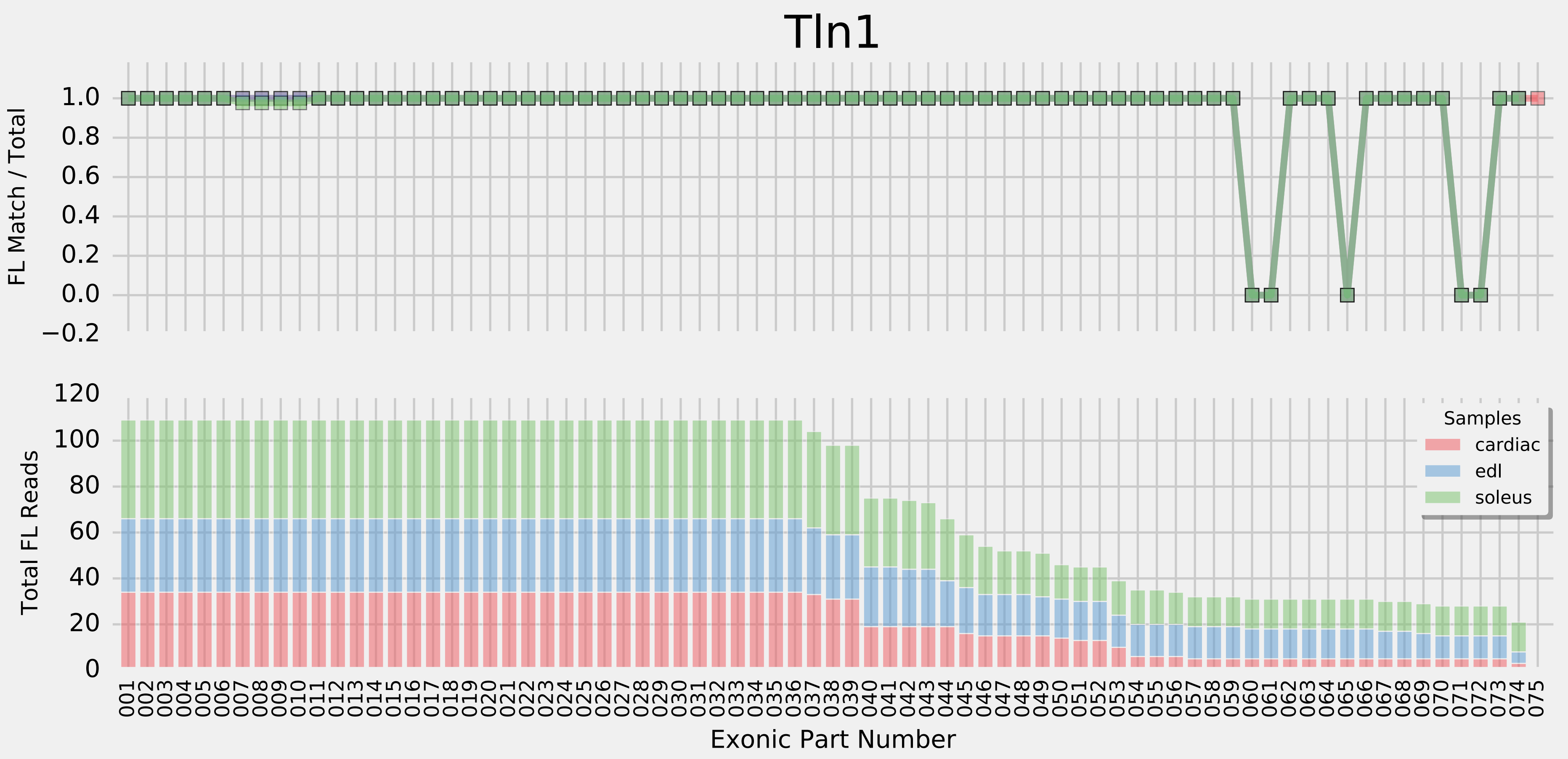

Tln1
FL Match / Total
1.0
0.8
0.6
0.4
0.2
0.0
−0.2
Total FL Reads
120
100
80
60
40
20
0
Samples
cardiac
edl
soleus
Exonic Part Number

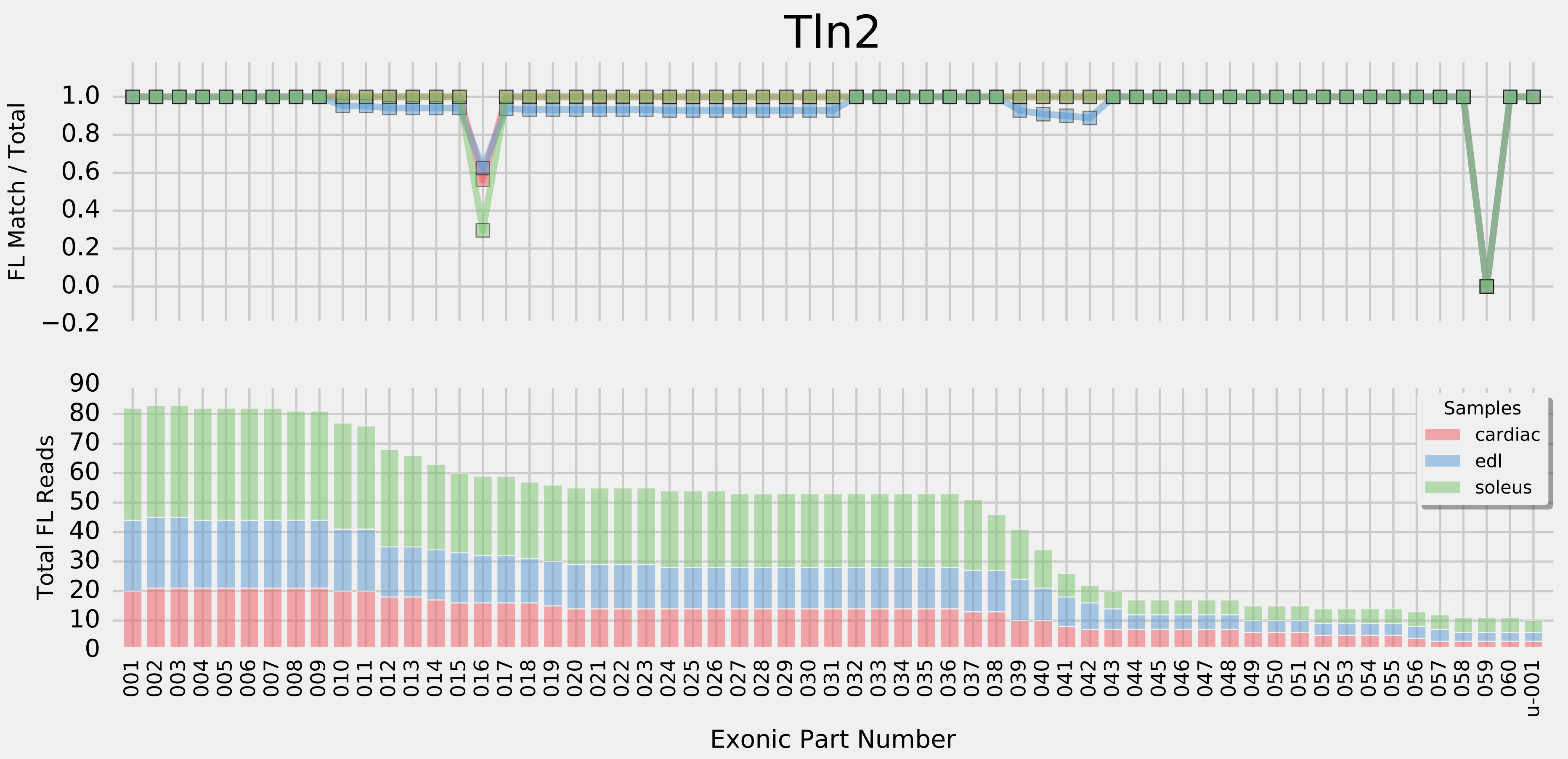

Tln2
FL Match / Total
Total FL Reads
Samples
cardiac
edl
soleus
Exonic Part Number

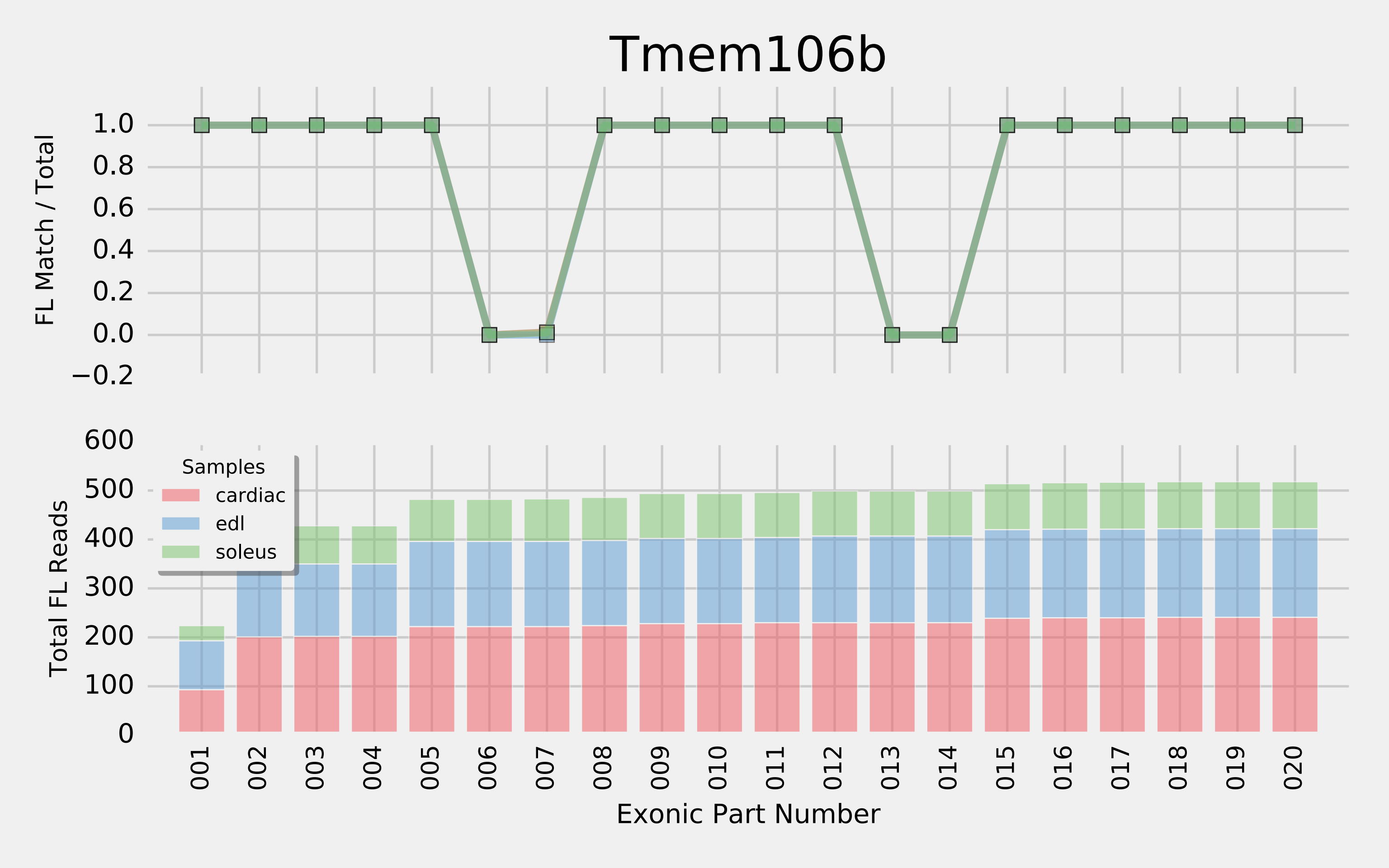

Tmem106b
FL Match / Total
1.0
0.8
0.6
0.4
0.2
0.0
−0.2
Total FL Reads
600
500
400
300
200
100
0
Samples
cardiac
edl
soleus
001
002
003
004
005
006
007
008
009
010
011
012
013
014
015
016
017
018
019
020
Exonic Part Number

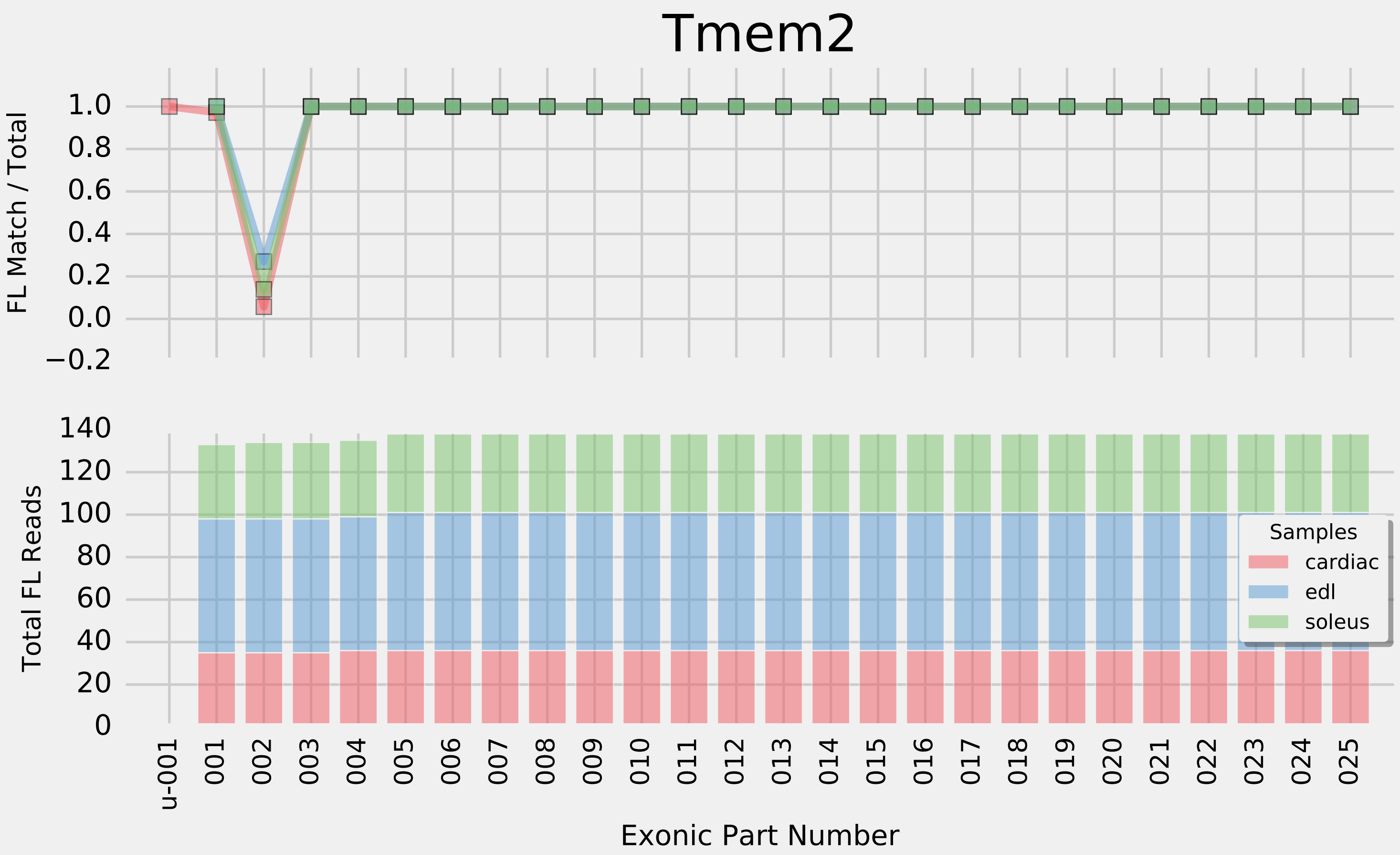

Tmem2
FL Match / Total
1.0
0.8
0.6
0.4
0.2
0.0
−0.2
Total FL Reads
140
120
100
80
60
40
20
0
Samples
cardiac
edl
soleus
u-001
001
002
003
004
005
006
007
008
009
010
011
012
013
014
015
016
017
018
019
020
021
022
023
024
025
Exonic Part Number

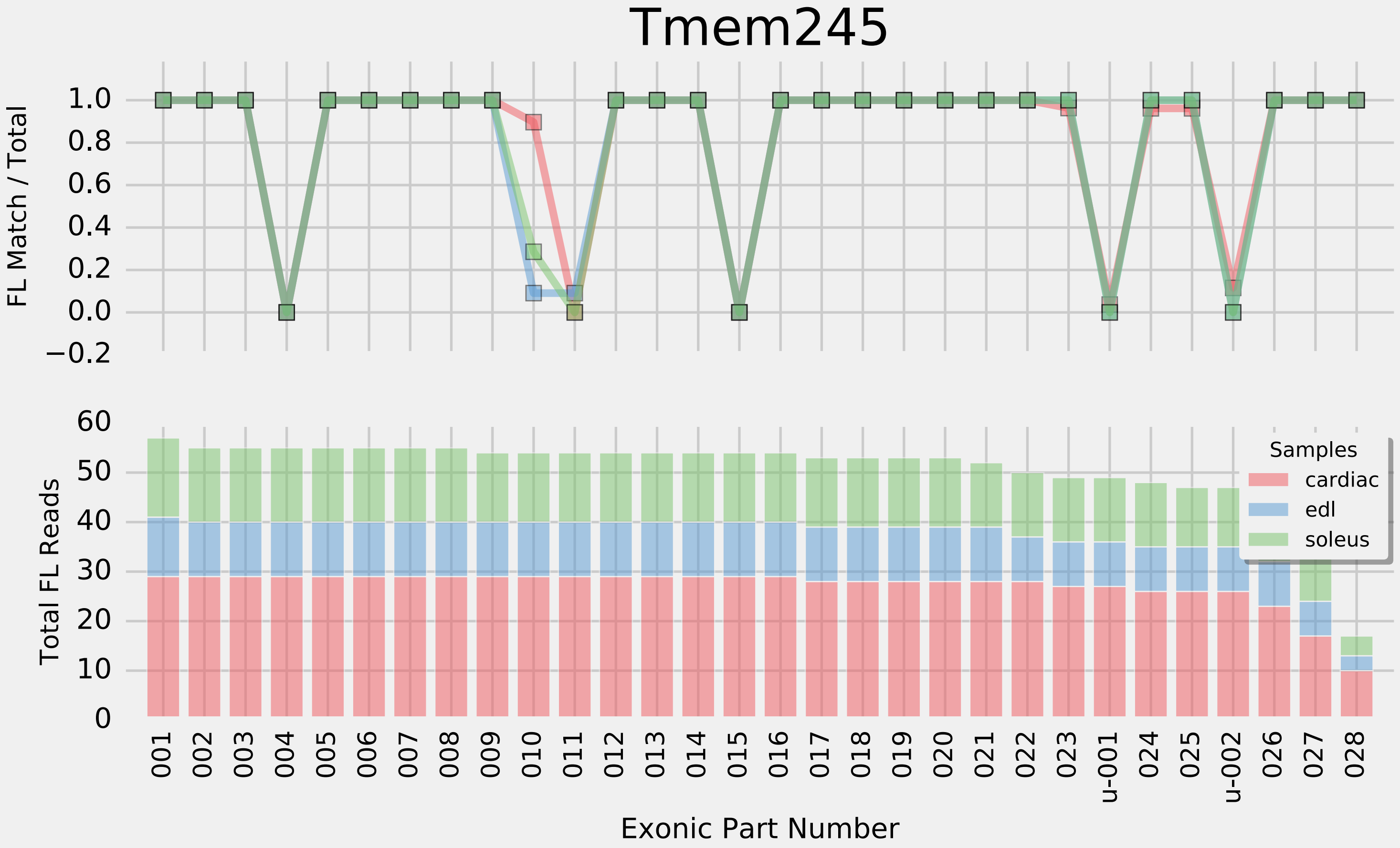

Tmem245
FL Match / Total
1.0
0.8
0.6
0.4
0.2
0.0
−0.2
60
50
40
30
20
10
0
Total FL Reads
Samples
cardiac
edl
soleus
001
002
003
004
005
006
007
008
009
010
011
012
013
014
015
016
017
018
019
020
021
022
023
u-001
024
025
u-002
026
027
028
Exonic Part Number

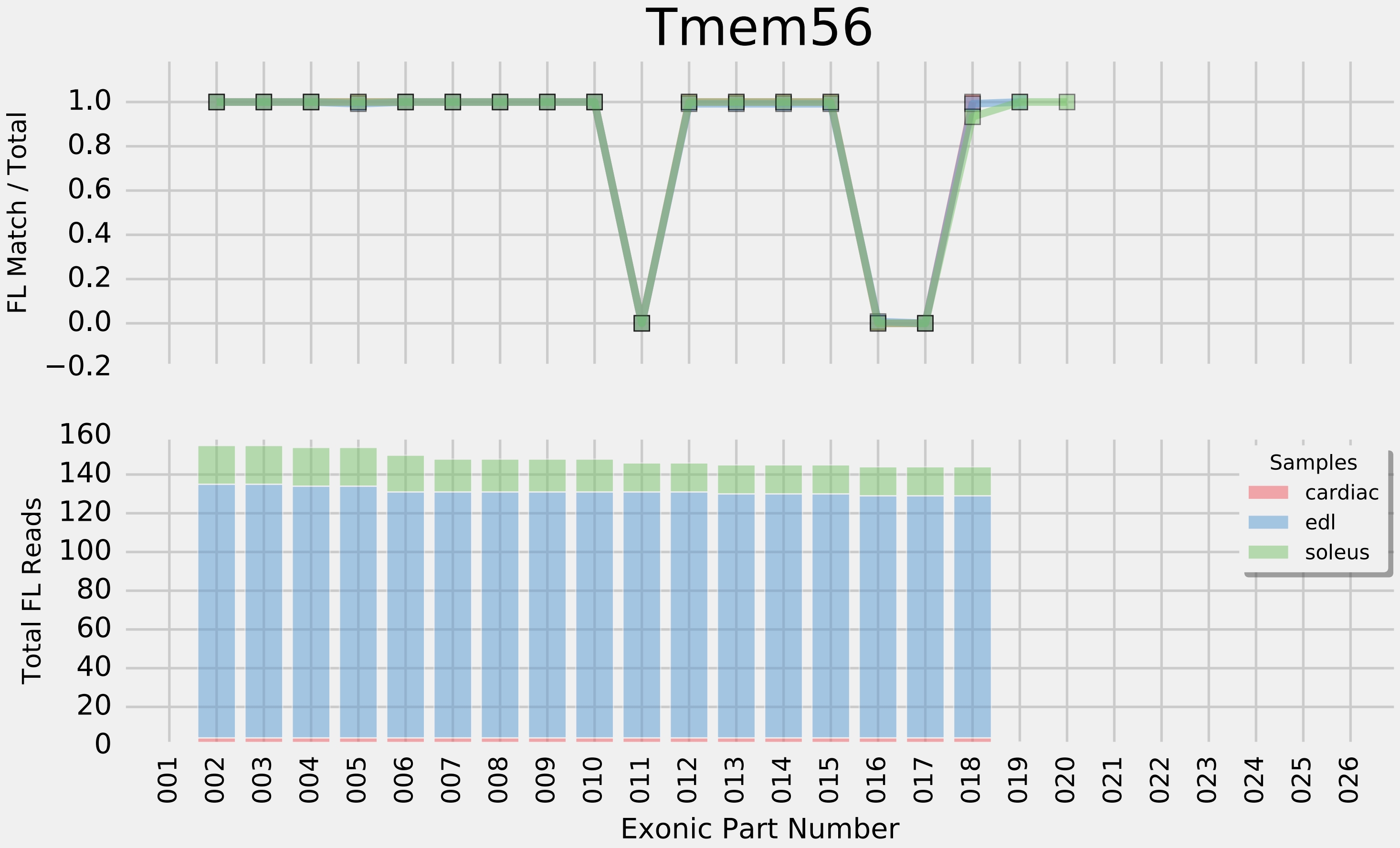

Tmem56
FL Match / Total
1.0
0.8
0.6
0.4
0.2
0.0
−0.2
Total FL Reads
160
140
120
100
80
60
40
20
0
Samples
cardiac
edl
soleus
001
002
003
004
005
006
007
008
009
010
011
012
013
014
015
016
017
018
019
020
021
022
023
024
025
026
Exonic Part Number

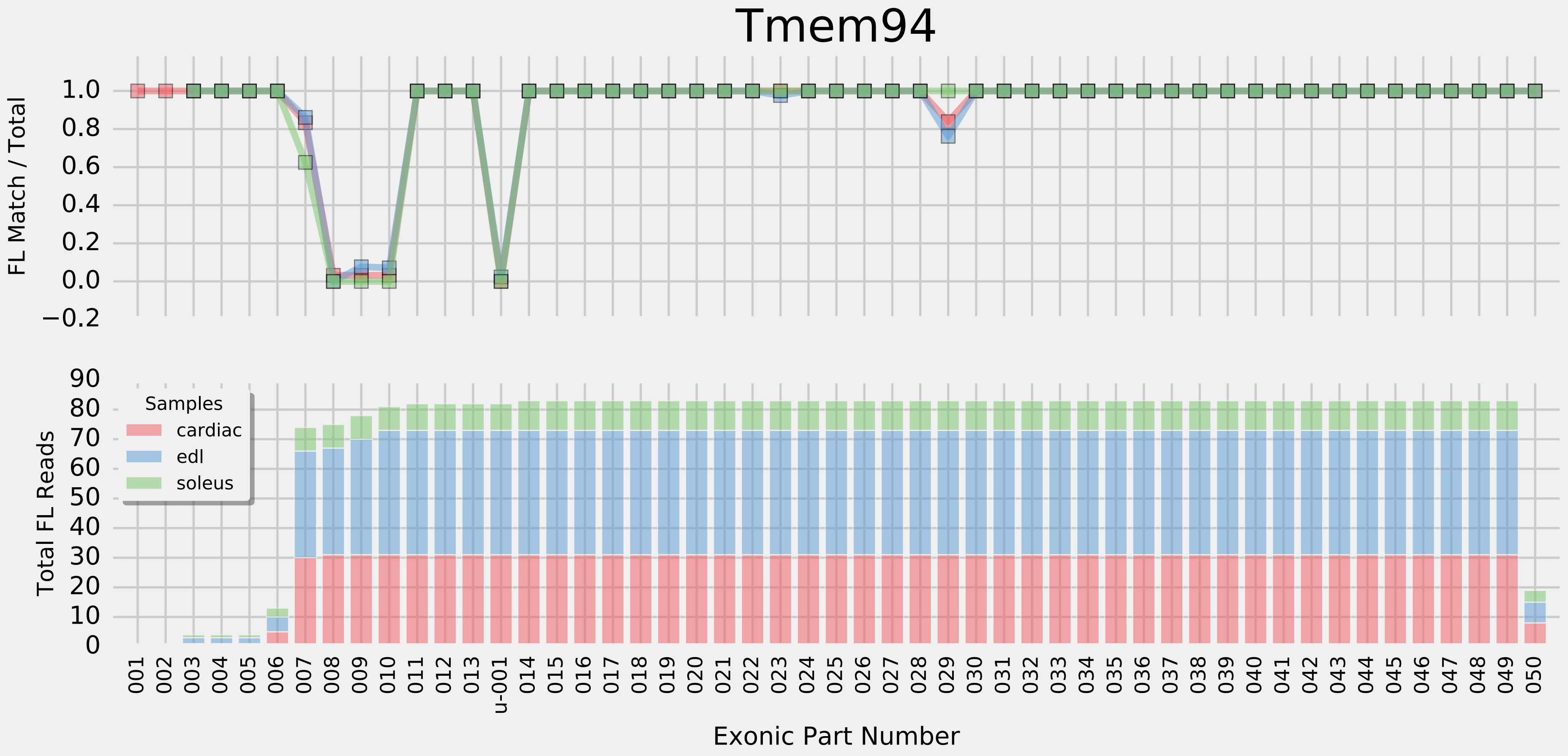

Tmem94
FL Match / Total
1.0
0.8
0.6
0.4
0.2
0.0
−0.2
Total FL Reads
90
80
70
60
50
40
30
20
10
0
Samples
cardiac
edl
soleus
001 002 003 004 005 006 007 008 009 010 011 012 013 u-001 014 015 016 017 018 019 020 021 022 023 024 025 026 027 028 029 030 031 032 033 034 035 036 037 038 039 040 041 042 043 044 045 046 047 048 049 050
Exonic Part Number

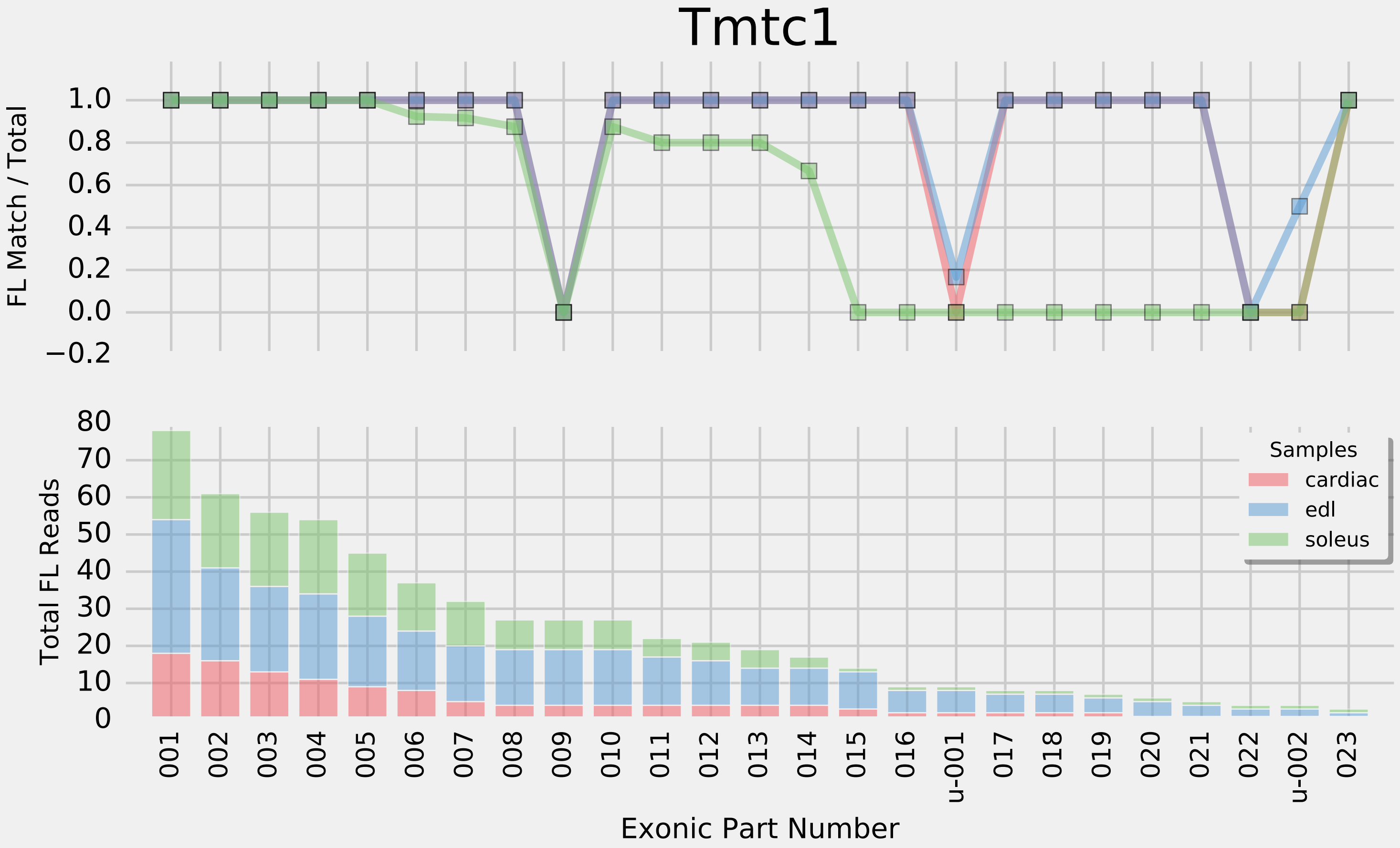

Tmtc1
FL Match / Total
1.0
0.8
0.6
0.4
0.2
0.0
−0.2
Total FL Reads
80
70
60
50
40
30
20
10
0
Samples
cardiac
edl
soleus
001
002
003
004
005
006
007
008
009
010
011
012
013
014
015
016
u-001
017
018
019
020
021
022
u-002
023
Exonic Part Number

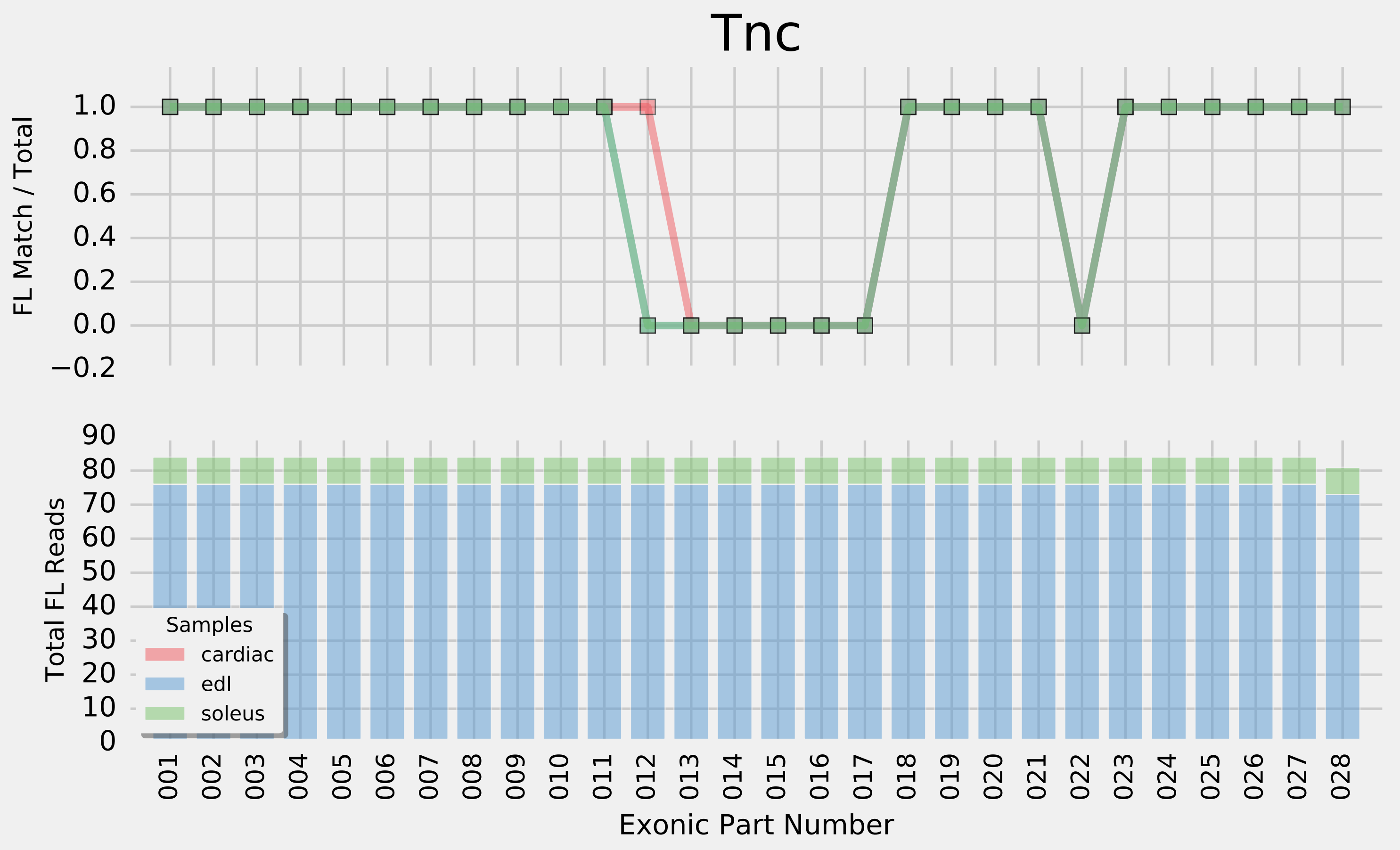
Tnc
FL Match / Total
1.0
0.8
0.6
0.4
0.2
0.0
−0.2
Total FL Reads
90
80
70
60
50
40
30
20
10
0
Samples
cardiac
edl
soleus
001
002
003
004
005
006
007
008
009
010
011
012
013
014
015
016
017
018
019
020
021
022
023
024
025
026
027
028
Exonic Part Number

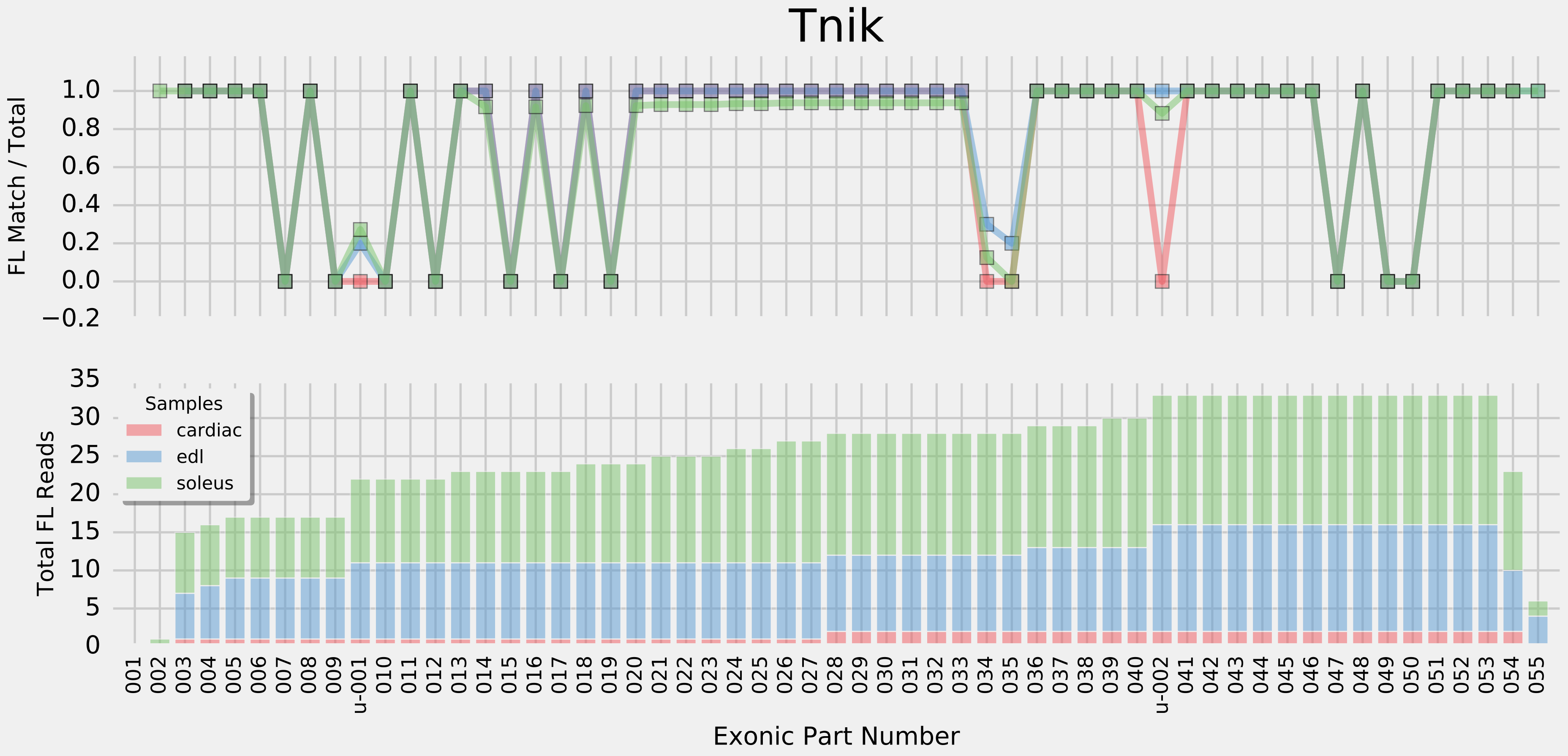

Tnik
FL Match / Total
Total FL Reads
Samples
cardiac
edl
soleus
Exonic Part Number

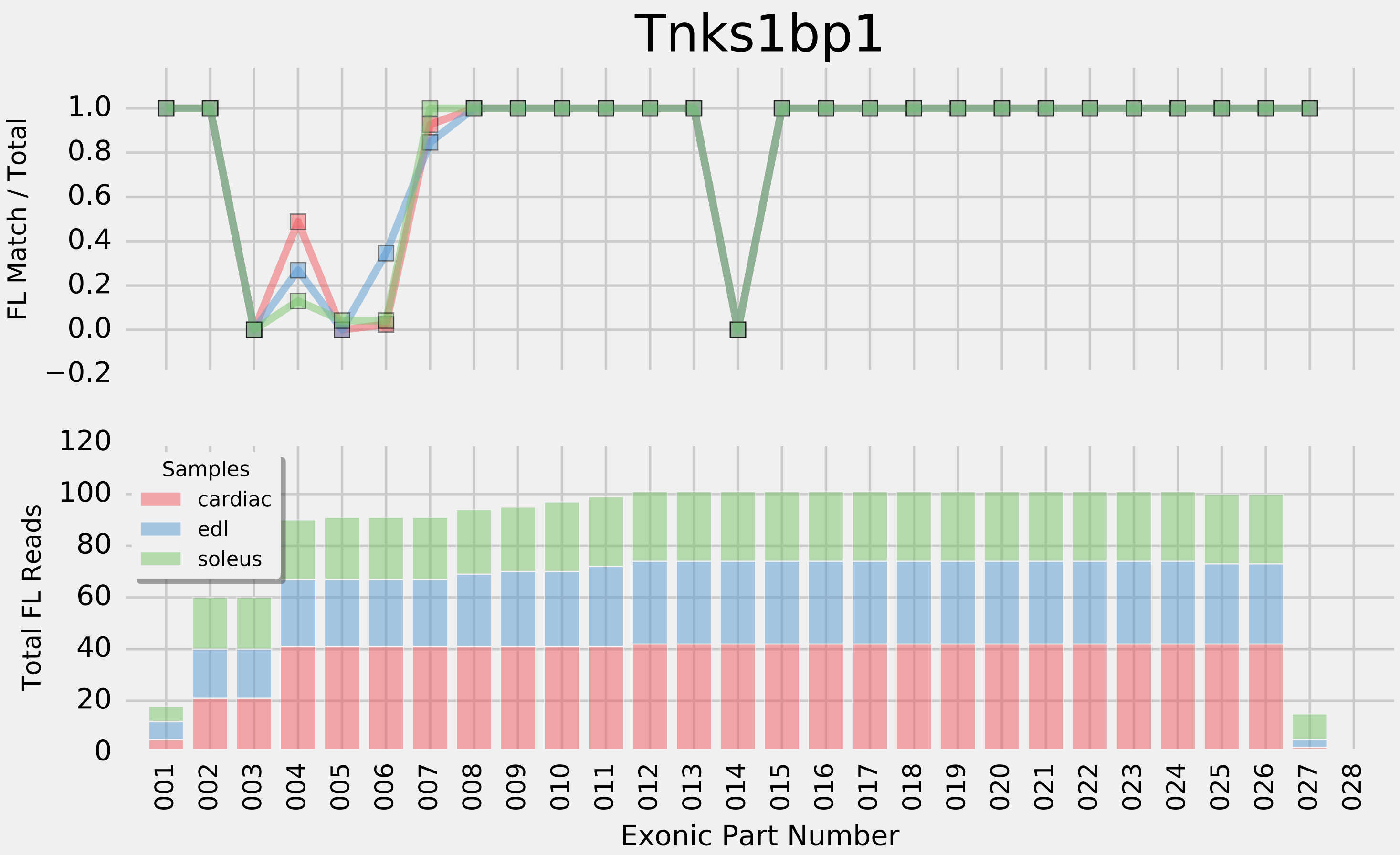

Tnks1bp1
FL Match / Total
1.0
0.8
0.6
0.4
0.2
0.0
−0.2
Total FL Reads
120
100
80
60
40
20
0
Samples
cardiac
edl
soleus
001
002
003
004
005
006
007
008
009
010
011
012
013
014
015
016
017
018
019
020
021
022
023
024
025
026
027
028
Exonic Part Number

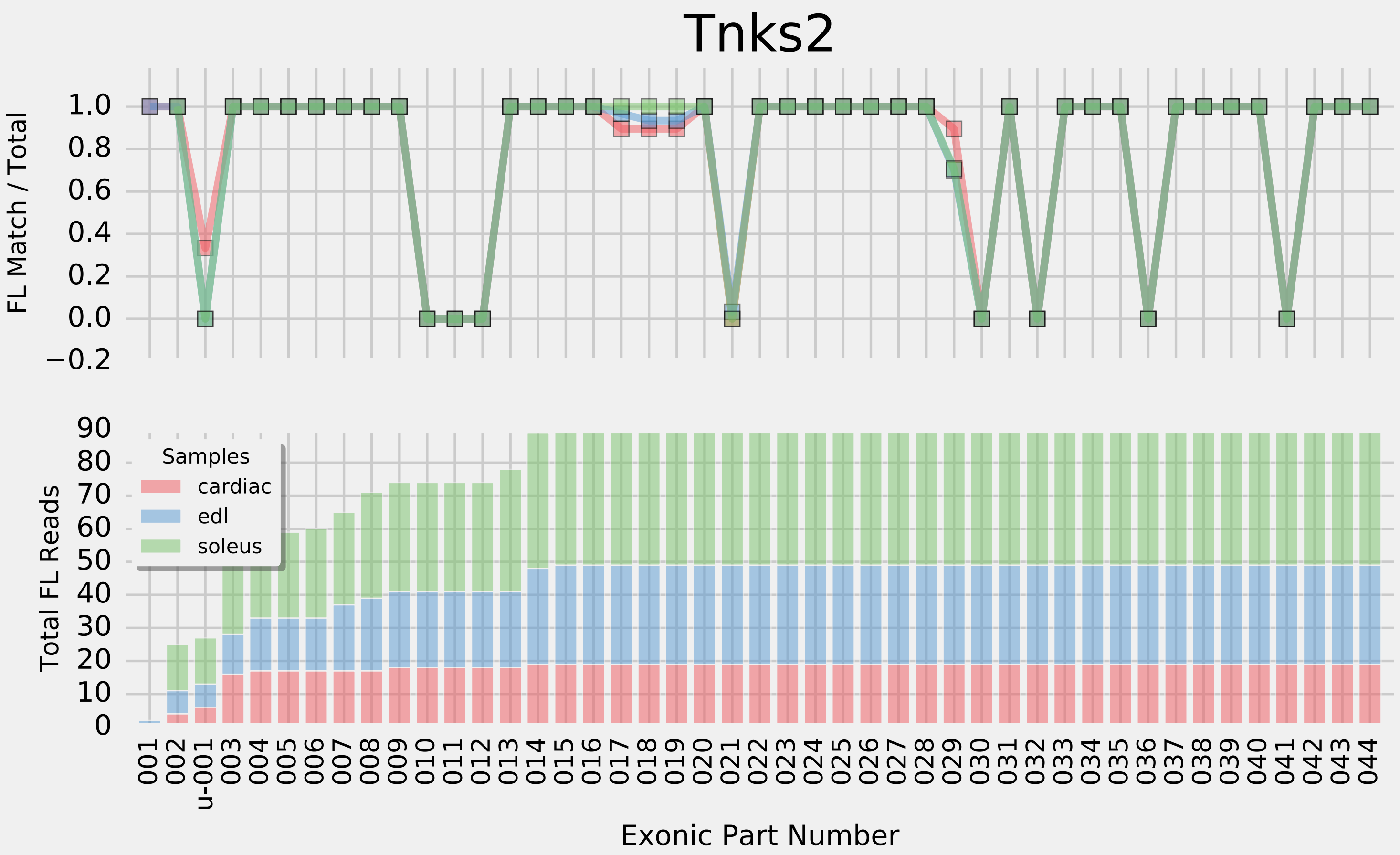
Tnks2
FL Match / Total
1.0
0.8
0.6
0.4
0.2
0.0
−0.2
Total FL Reads
90
80
70
60
50
40
30
20
10
0
Samples
cardiac
edl
soleus
001
002
u-001
003
004
005
006
007
008
009
010
011
012
013
014
015
016
017
018
019
020
021
022
023
024
025
026
027
028
029
030
031
032
033
034
035
036
037
038
039
040
041
042
043
044
Exonic Part Number

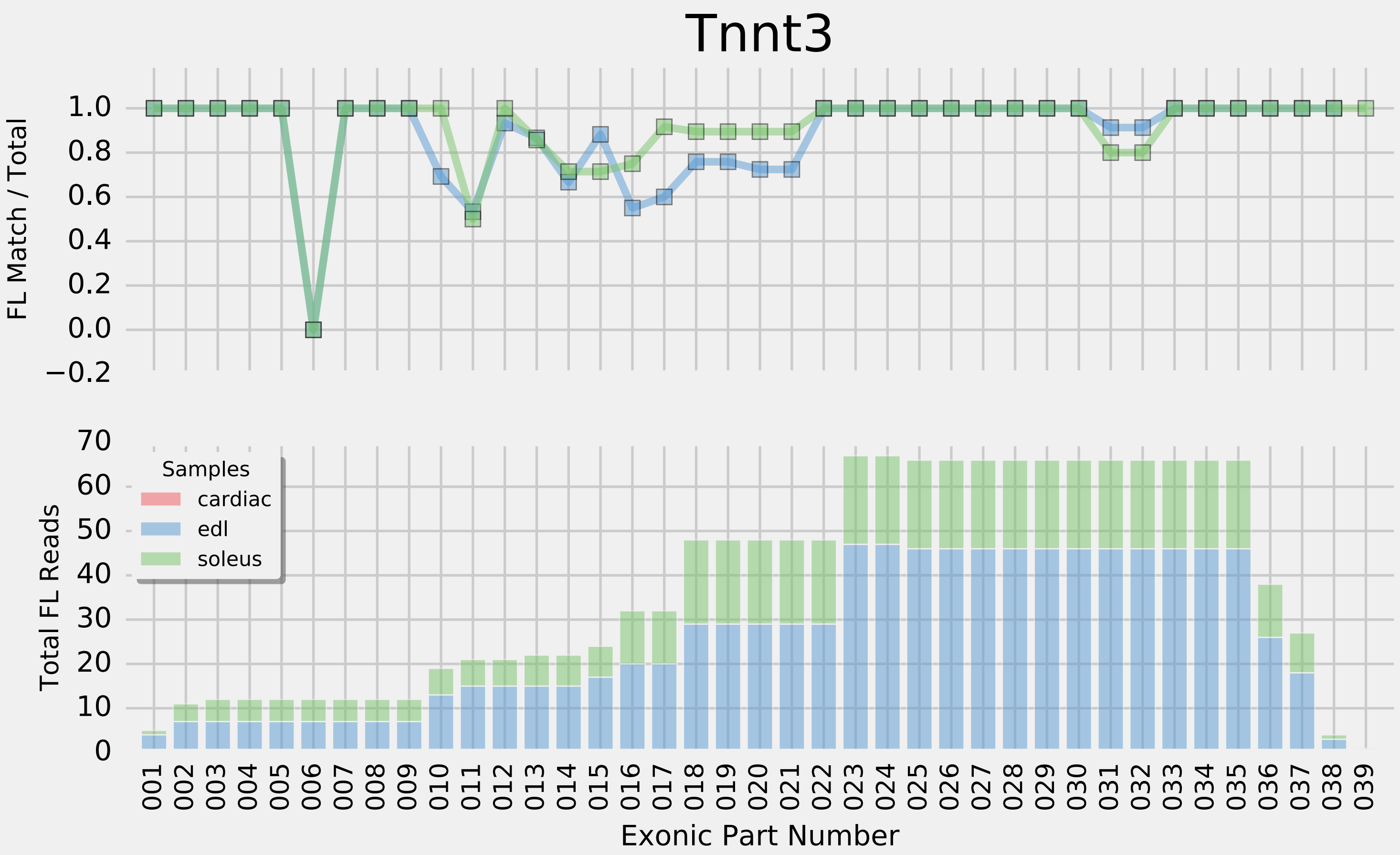

Tnnt3
FL Match / Total
1.0
0.8
0.6
0.4
0.2
0.0
−0.2
Total FL Reads
70
60
50
40
30
20
10
0
Samples
cardiac
edl
soleus
001
002
003
004
005
006
007
008
009
010
011
012
013
014
015
016
017
018
019
020
021
022
023
024
025
026
027
028
029
030
031
032
033
034
035
036
037
038
039
Exonic Part Number

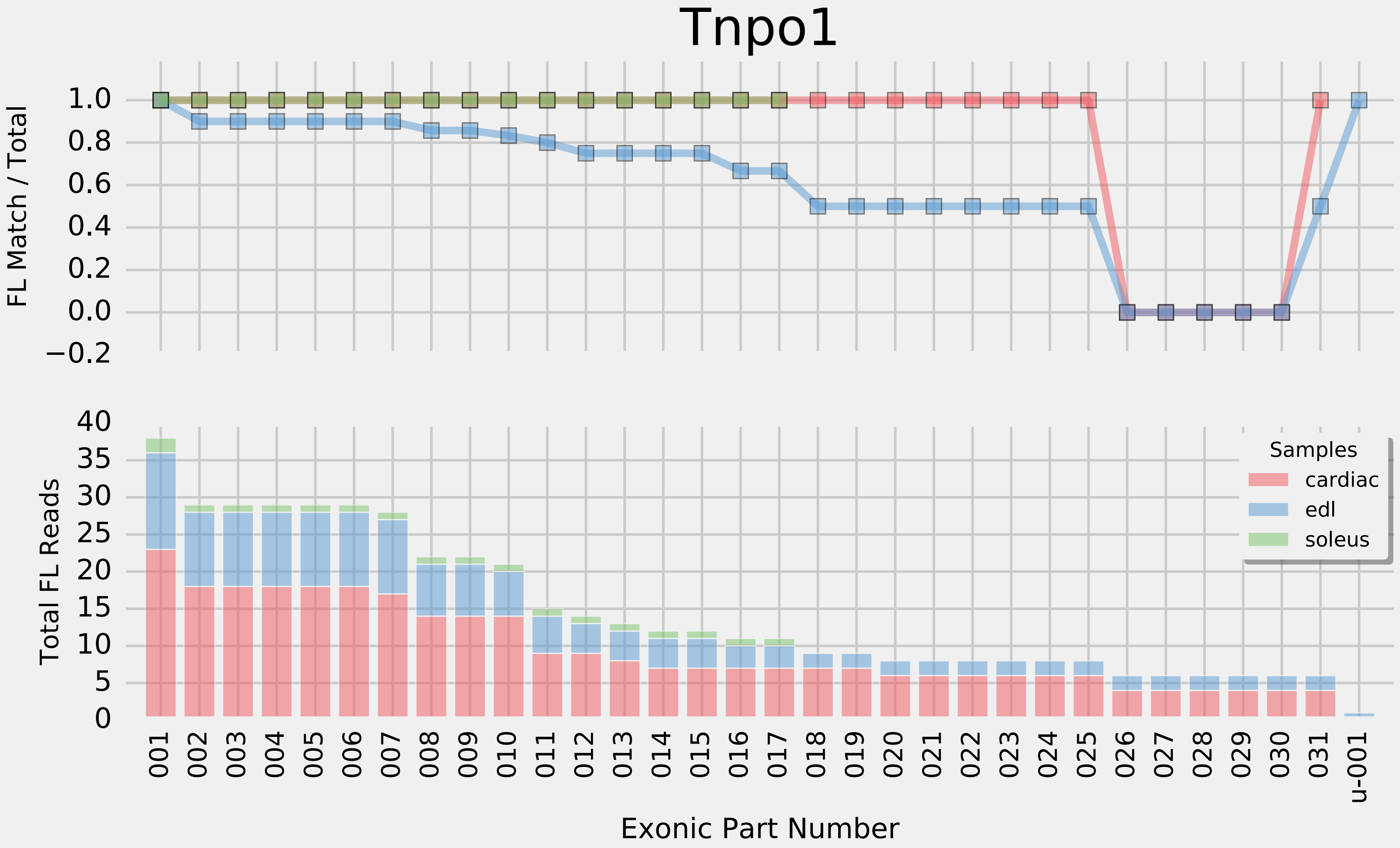

Tnpo1
FL Match / Total
1.0
0.8
0.6
0.4
0.2
0.0
−0.2
Total FL Reads
40
35
30
25
20
15
10
5
0
Samples
cardiac
edl
soleus
001
002
003
004
005
006
007
008
009
010
011
012
013
014
015
016
017
018
019
020
021
022
023
024
025
026
027
028
029
030
031
u-001
Exonic Part Number

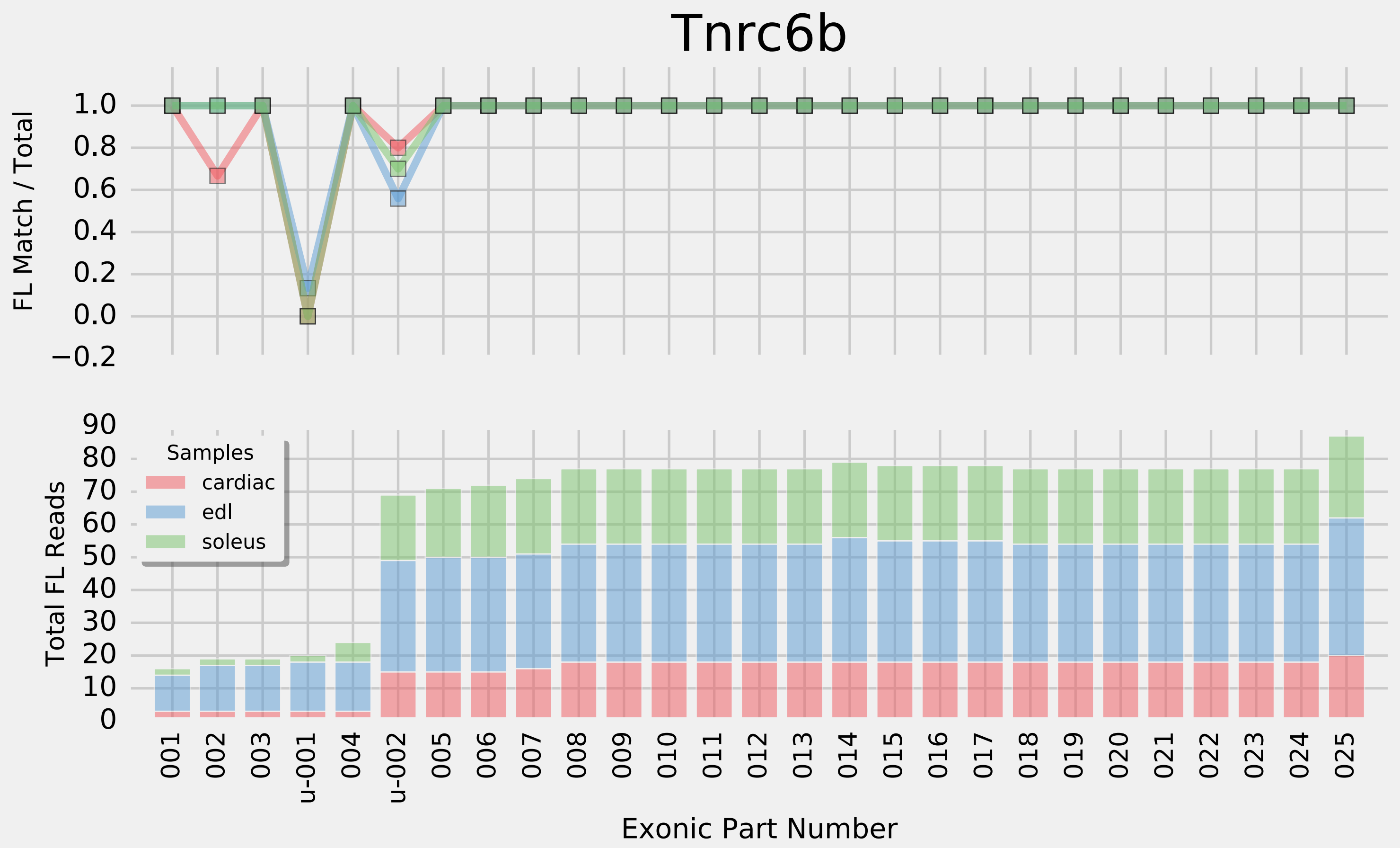

Tnrc6b
FL Match / Total
1.0
0.8
0.6
0.4
0.2
0.0
−0.2
Total FL Reads
90
80
70
60
50
40
30
20
10
0
Samples
cardiac
edl
soleus
001
002
003
u-001
004
u-002
005
006
007
008
009
010
011
012
013
014
015
016
017
018
019
020
021
022
023
024
025
Exonic Part Number

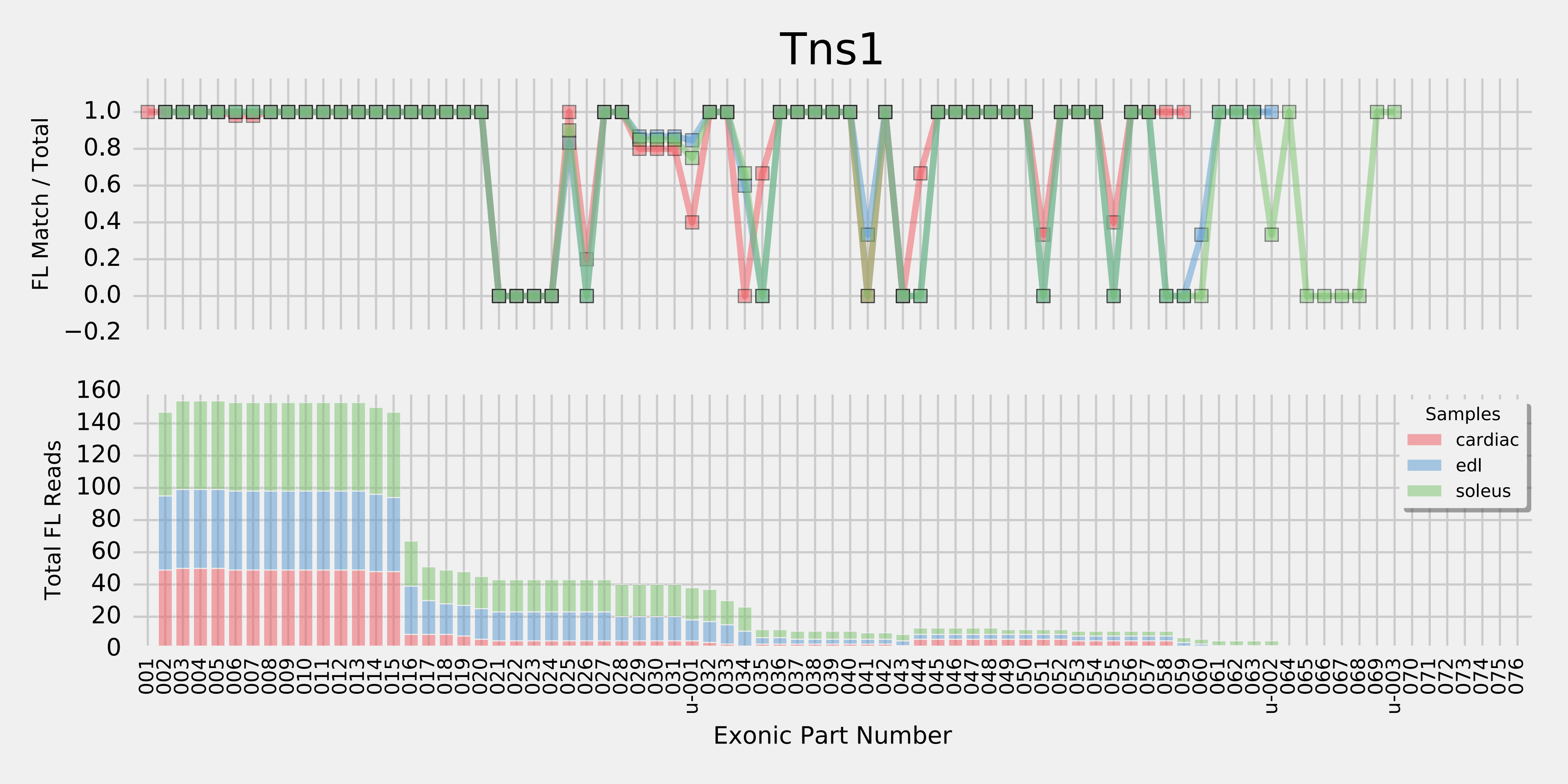

Tns1
FL Match / Total
1.0
0.8
0.6
0.4
0.2
0.0
−0.2
160
140
120
100
80
60
40
20
0
Total FL Reads
Samples
cardiac
edl
soleus
001 002 003 004 005 006 007 008 009 010 011 012 013 014 015 016 017 018 019 020 021 022 023 024 025 026 027 028 029 030 031 u-001 032 033 034 035 036 037 038 039 040 041 042 043 044 045 046 047 048 049 050 051 052 053 054 055 056 057 058 059 060 061 062 063 u-002 064 065 066 067 068 069 u-003 070 071 072 073 074 075 076
Exonic Part Number

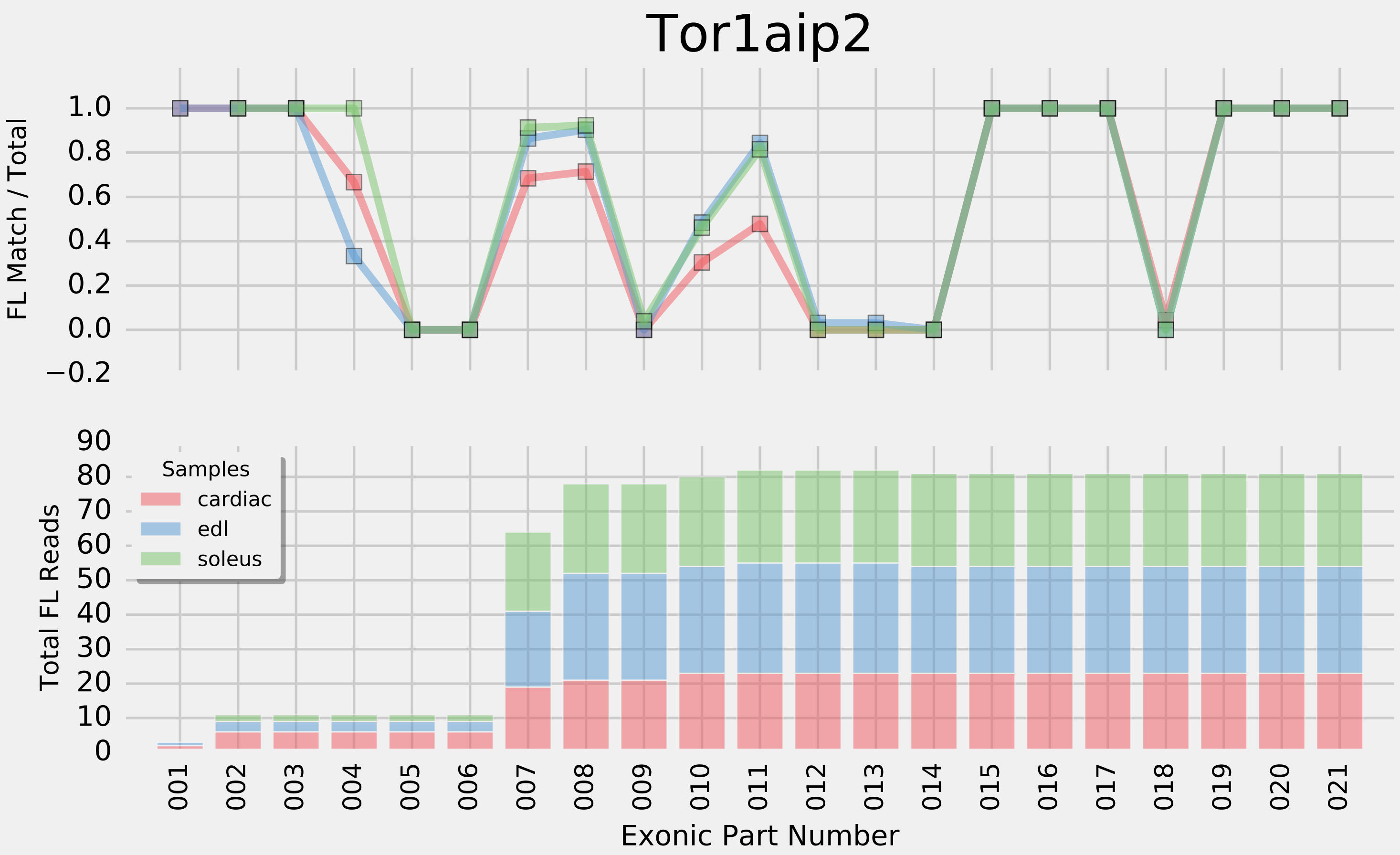

Tor1aip2
FL Match / Total
1.0
0.8
0.6
0.4
0.2
0.0
−0.2
Total FL Reads
90
80
70
60
50
40
30
20
10
0
Samples
cardiac
edl
soleus
001
002
003
004
005
006
007
008
009
010
011
012
013
014
015
016
017
018
019
020
021
Exonic Part Number

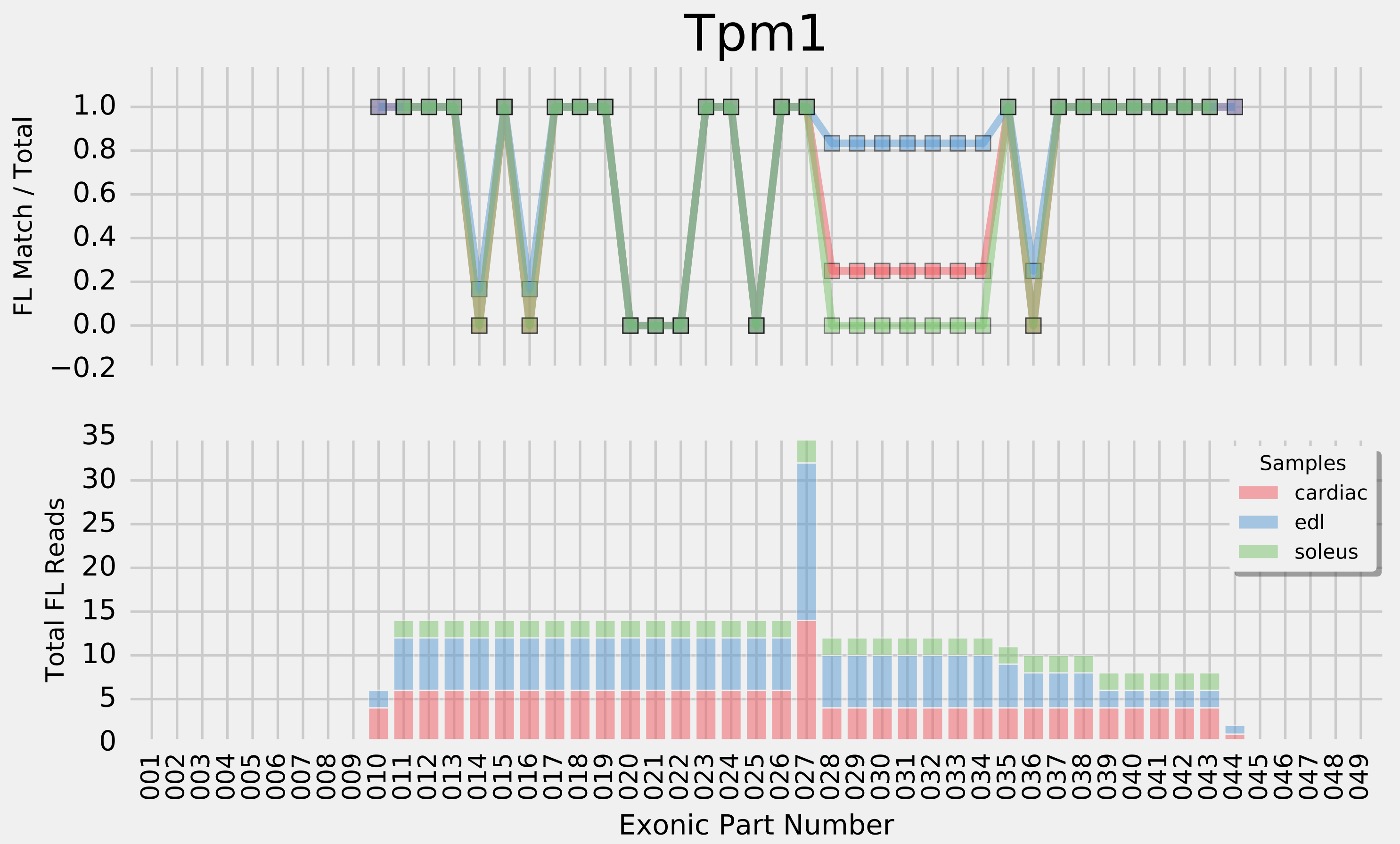

Tpm1
FL Match / Total
Total FL Reads
Exonic Part Number
Samples
cardiac
edl
soleus

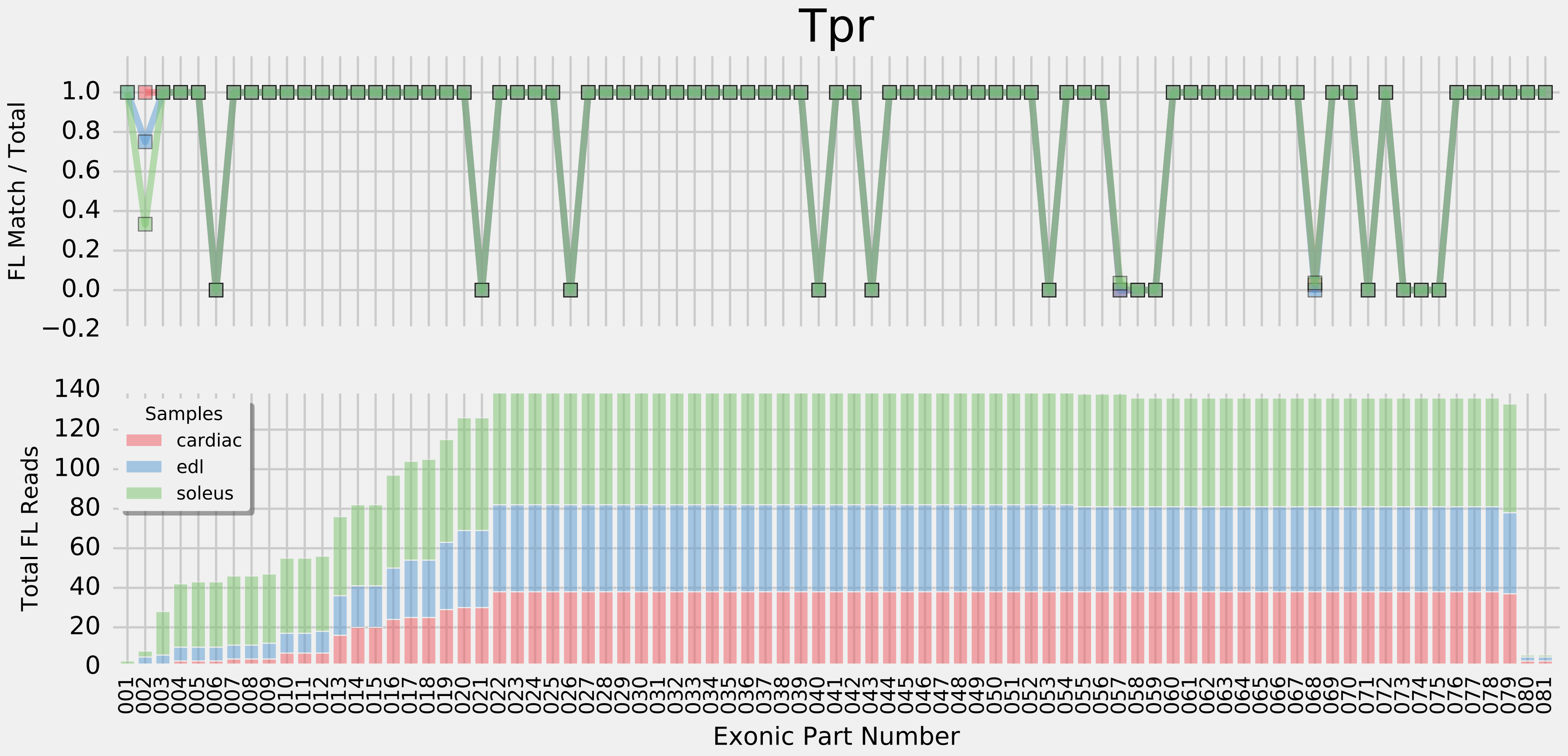
Tpr
FL Match / Total
1.0
0.8
0.6
0.4
0.2
0.0
−0.2
Total FL Reads
140
120
100
80
60
40
20
0
Samples
cardiac
edl
soleus
Exonic Part Number
001 002 003 004 005 006 007 008 009 010 011 012 013 014 015 016 017 018 019 020 021 022 023 024 025 026 027 028 029 030 031 032 033 034 035 036 037 038 039 040 041 042 043 044 045 046 047 048 049 050 051 052 053 054 055 056 057 058 059 060 061 062 063 064 065 066 067 068 069 070 071 072 073 074 075 076 077 078 079 080 081

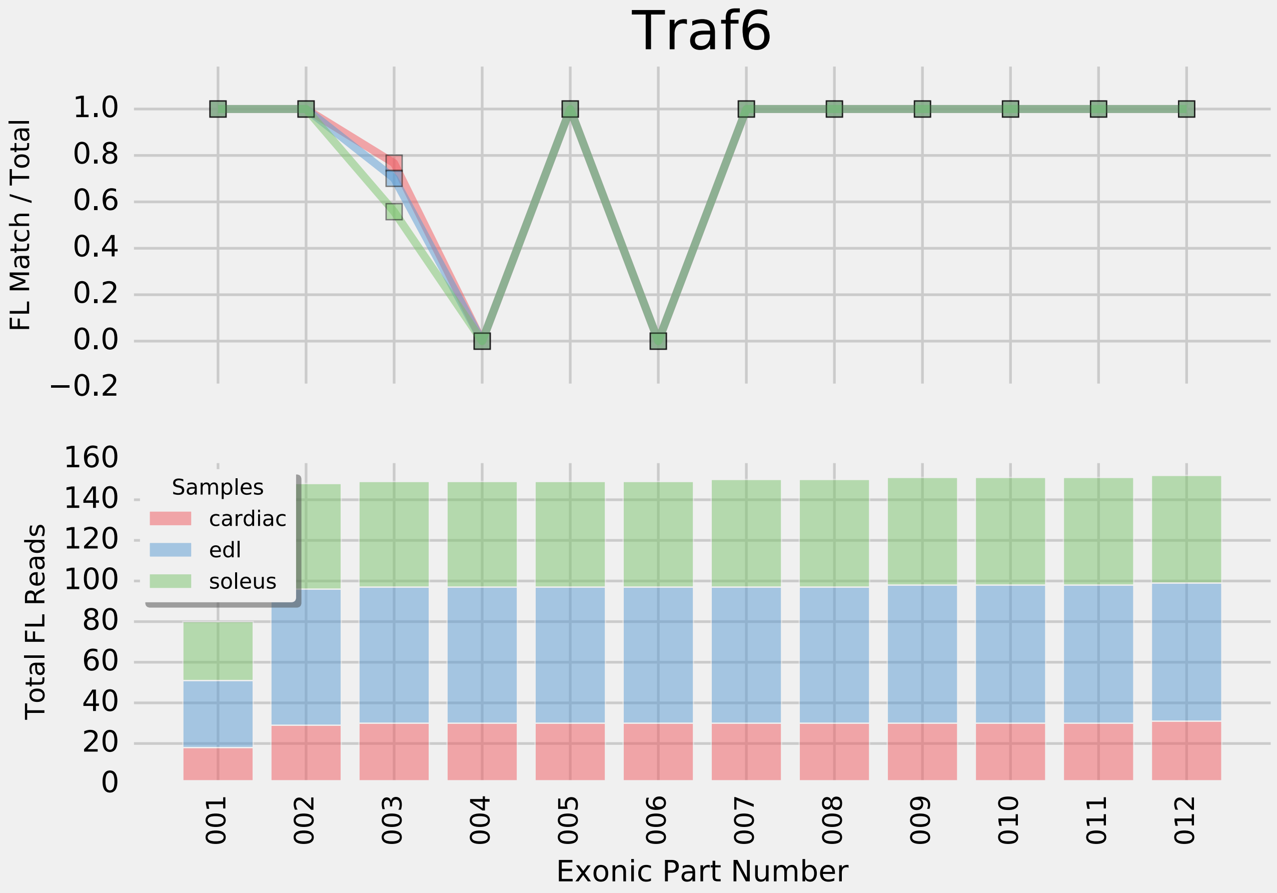

Traf6
FL Match / Total
1.0
0.8
0.6
0.4
0.2
0.0
−0.2
Total FL Reads
160
140
120
100
80
60
40
20
0
Samples
cardiac
edl
soleus
001
002
003
004
005
006
007
008
009
010
011
012
Exonic Part Number

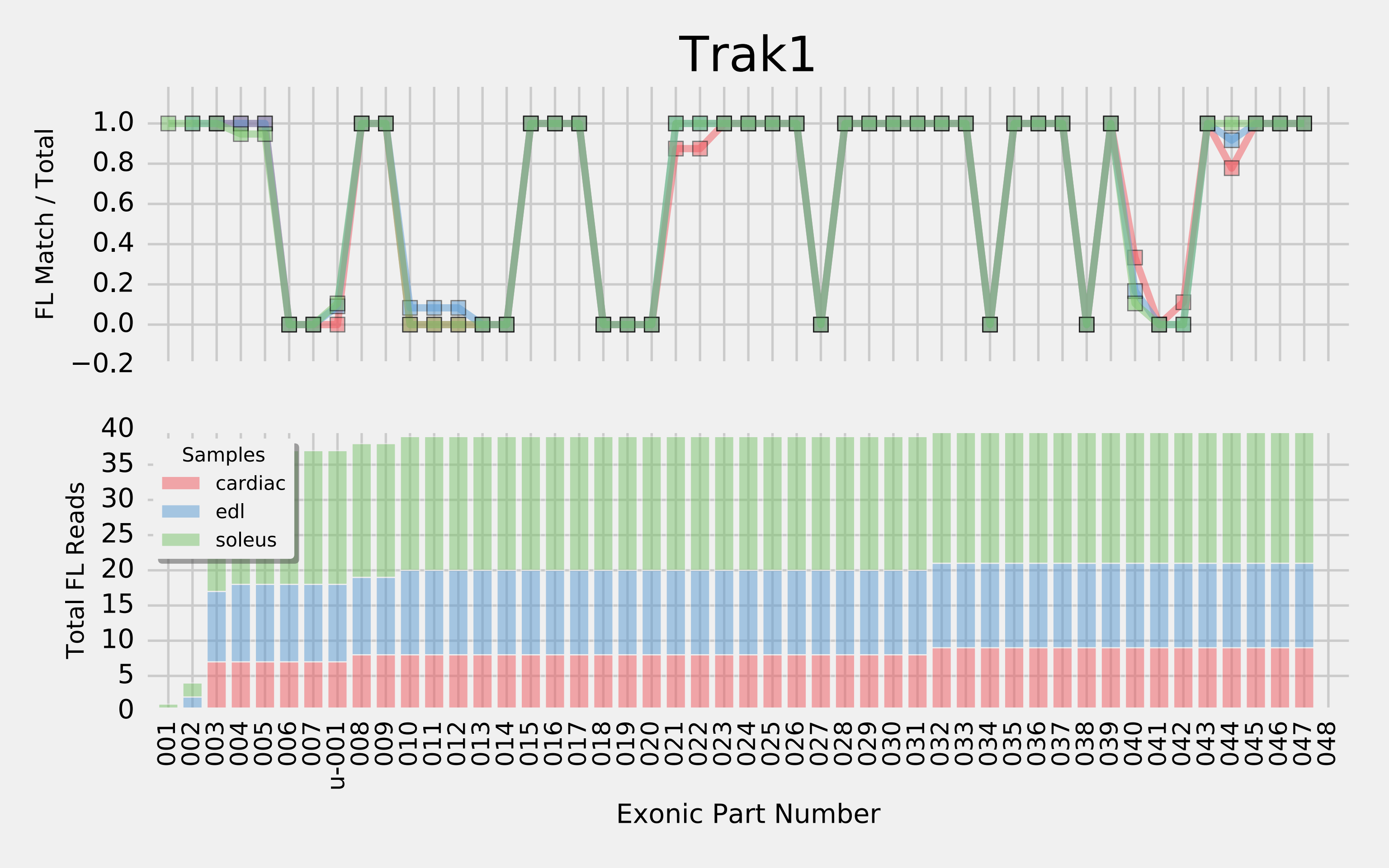

Trak1
FL Match / Total
1.0
0.8
0.6
0.4
0.2
0.0
−0.2
Total FL Reads
40
35
30
25
20
15
10
5
0
Samples
cardiac
edl
soleus
001
002
003
004
005
006
007
u-001
008
009
010
011
012
013
014
015
016
017
018
019
020
021
022
023
024
025
026
027
028
029
030
031
032
033
034
035
036
037
038
039
040
041
042
043
044
045
046
047
048
Exonic Part Number

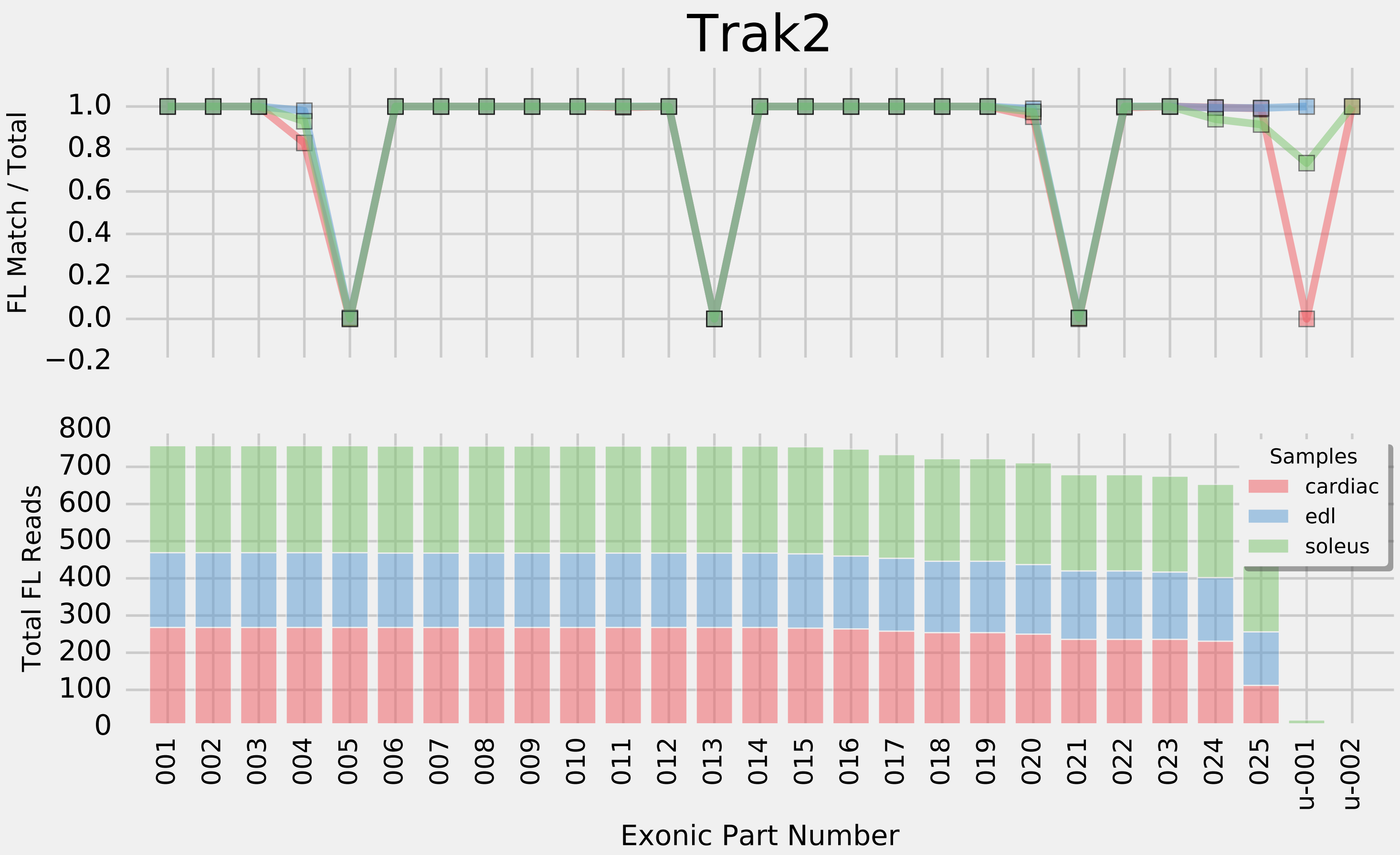

Trak2
FL Match / Total
1.0
0.8
0.6
0.4
0.2
0.0
−0.2
Total FL Reads
800
700
600
500
400
300
200
100
0
Samples
cardiac
edl
soleus
001
002
003
004
005
006
007
008
009
010
011
012
013
014
015
016
017
018
019
020
021
022
023
024
025
u-001
u-002
Exonic Part Number

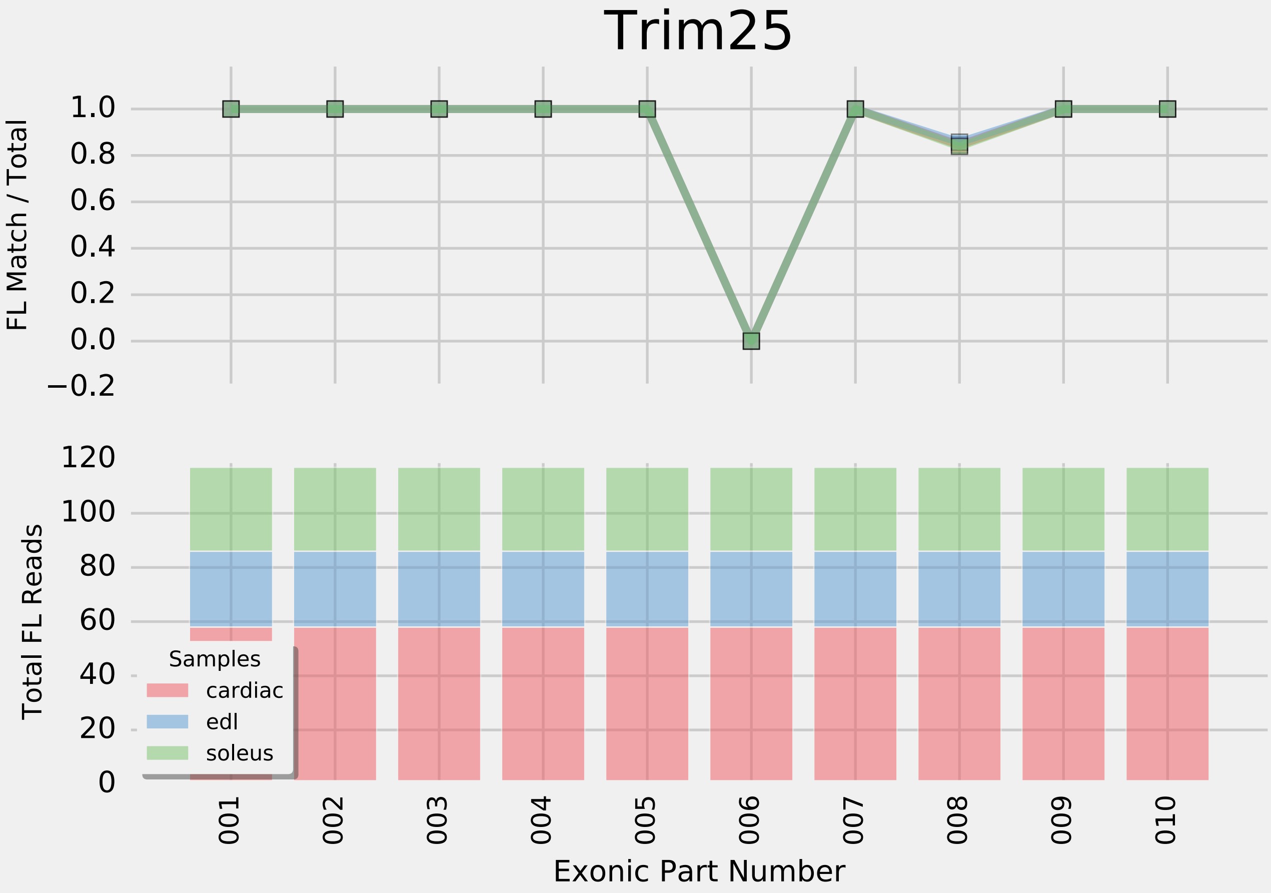

Trim25
FL Match / Total
1.0
0.8
0.6
0.4
0.2
0.0
−0.2
Total FL Reads
120
100
80
60
40
20
0
Samples
cardiac
edl
soleus
001
002
003
004
005
006
007
008
009
010
Exonic Part Number

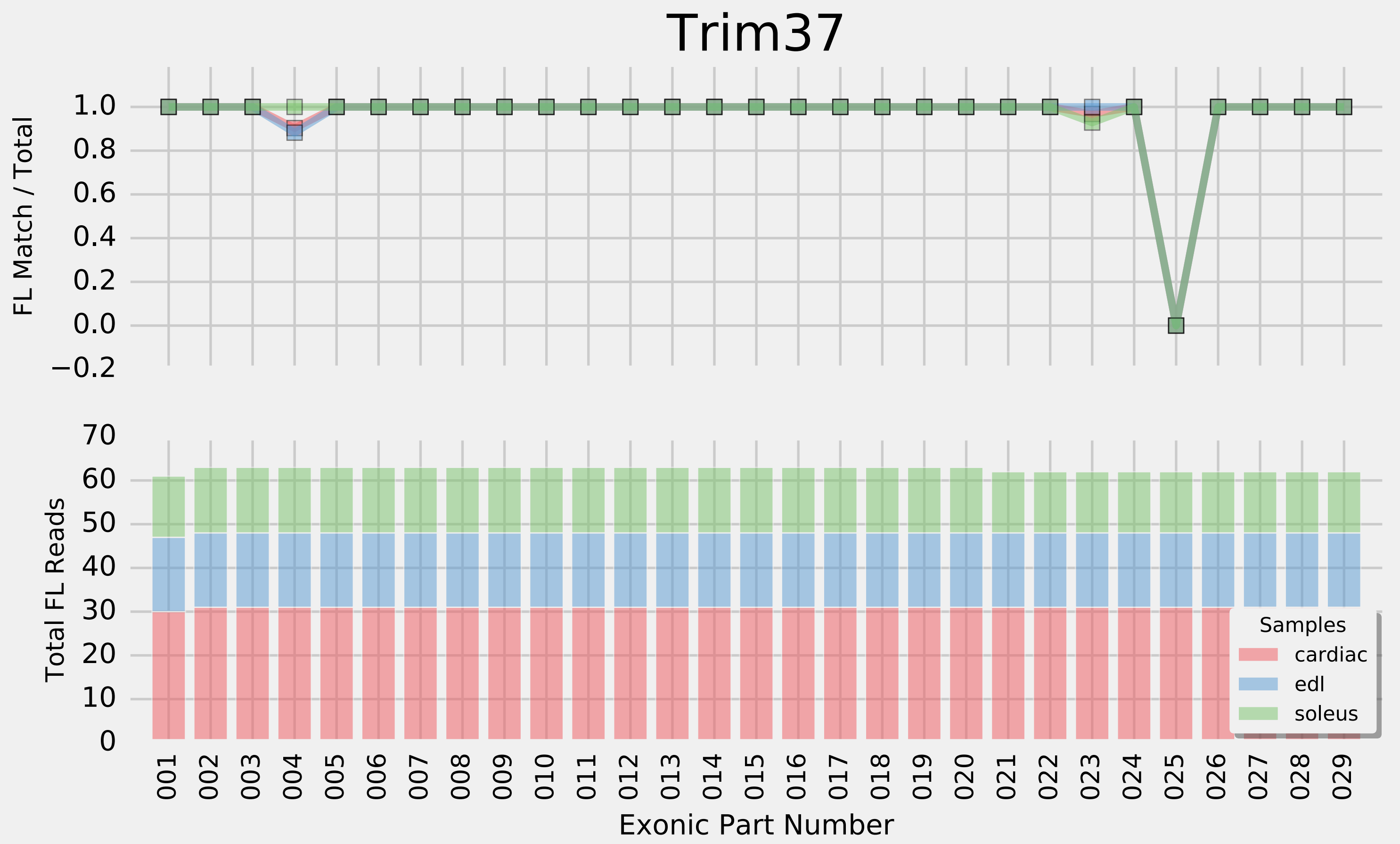

Trim37
FL Match / Total
1.0
0.8
0.6
0.4
0.2
0.0
−0.2
Total FL Reads
70
60
50
40
30
20
10
0
001
002
003
004
005
006
007
008
009
010
011
012
013
014
015
016
017
018
019
020
021
022
023
024
025
026
027
028
029
Exonic Part Number
Samples
cardiac
edl
soleus

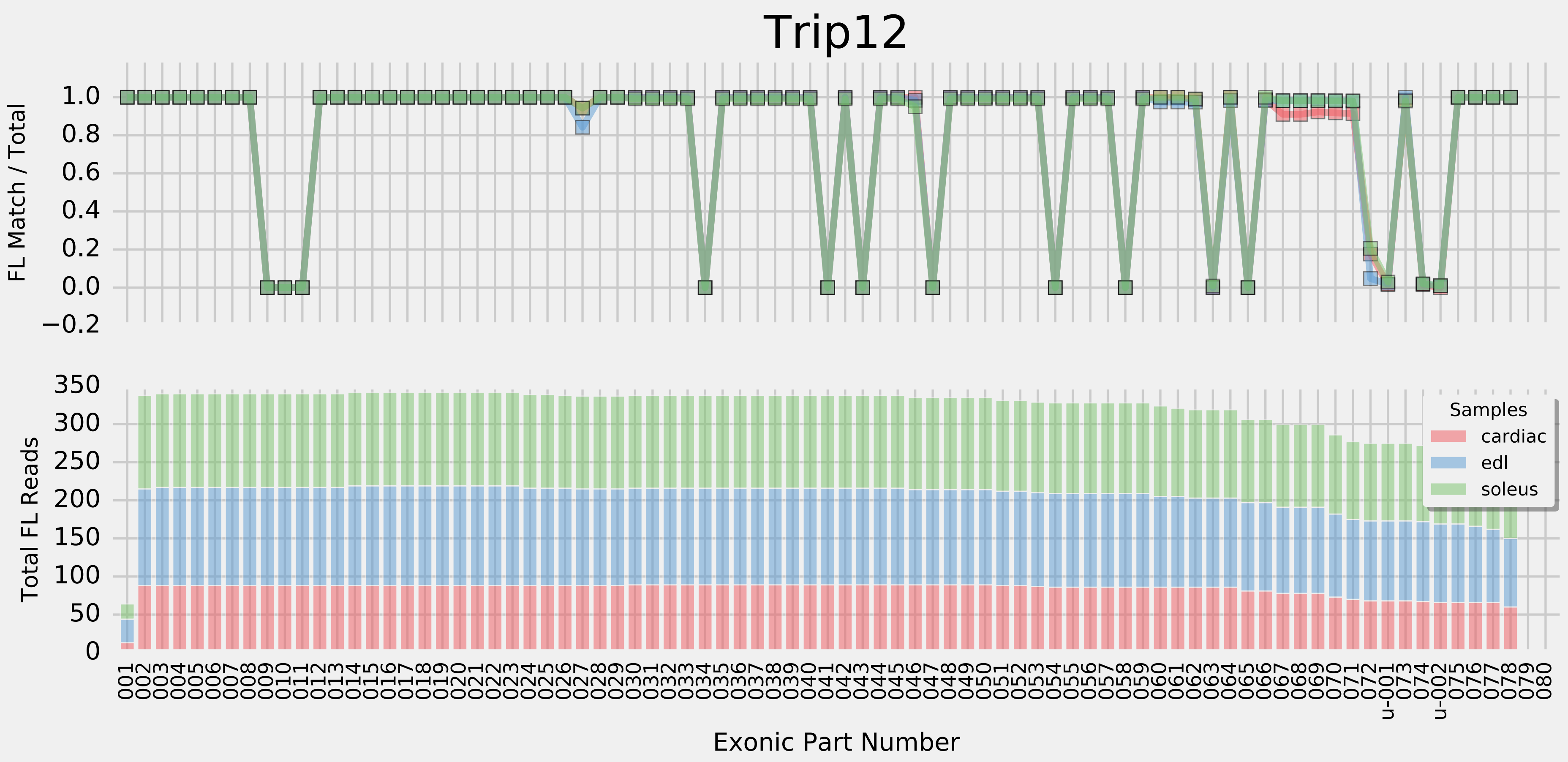

Trip12
FL Match / Total
Total FL Reads
Exonic Part Number
Samples
cardiac
edl
soleus

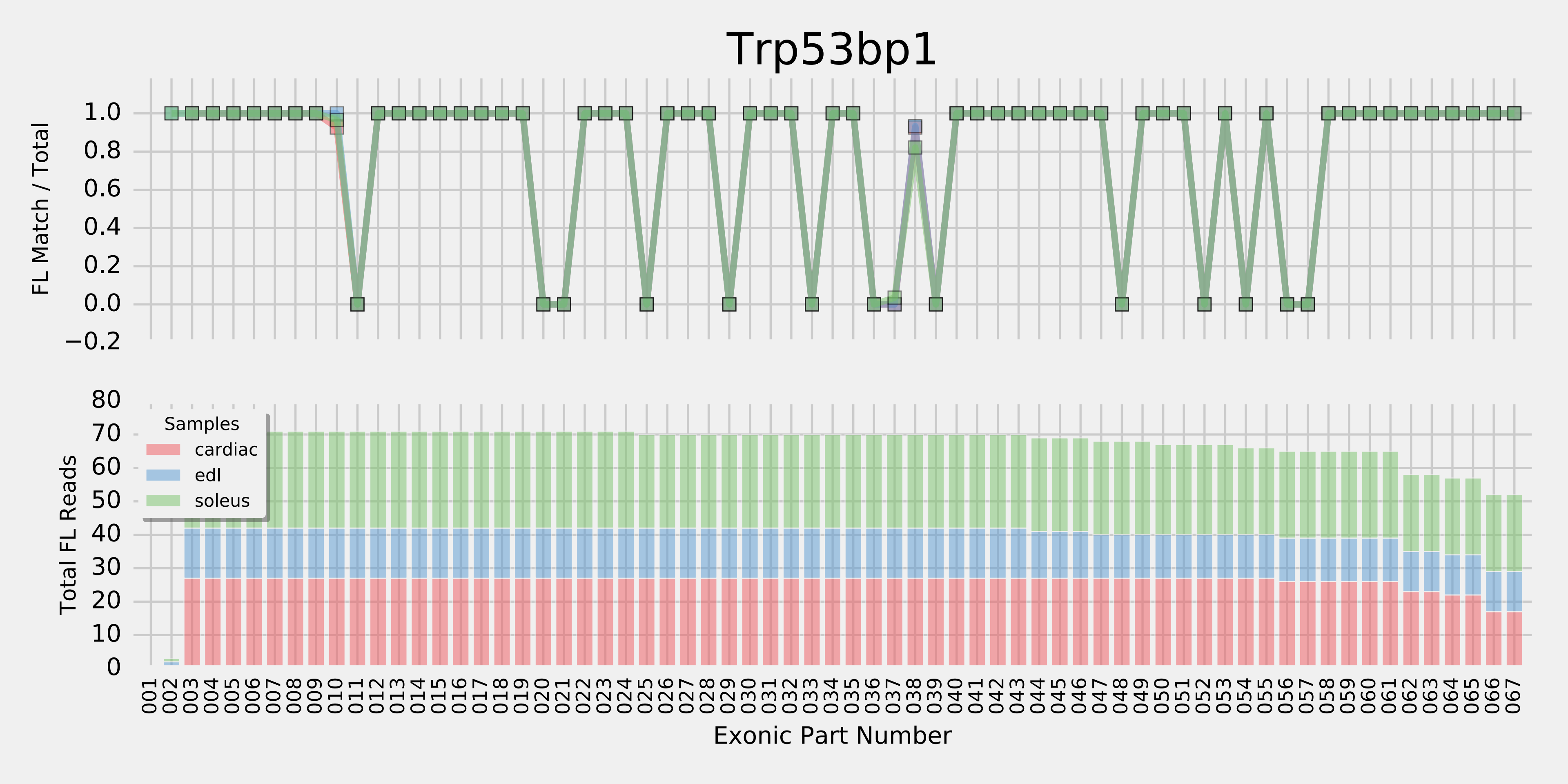

Trp53bp1
FL Match / Total
1.0
0.8
0.6
0.4
0.2
0.0
−0.2
Total FL Reads
80
70
60
50
40
30
20
10
0
Samples
cardiac
edl
soleus
001 002 003 004 005 006 007 008 009 010 011 012 013 014 015 016 017 018 019 020 021 022 023 024 025 026 027 028 029 030 031 032 033 034 035 036 037 038 039 040 041 042 043 044 045 046 047 048 049 050 051 052 053 054 055 056 057 058 059 060 061 062 063 064 065 066 067
Exonic Part Number

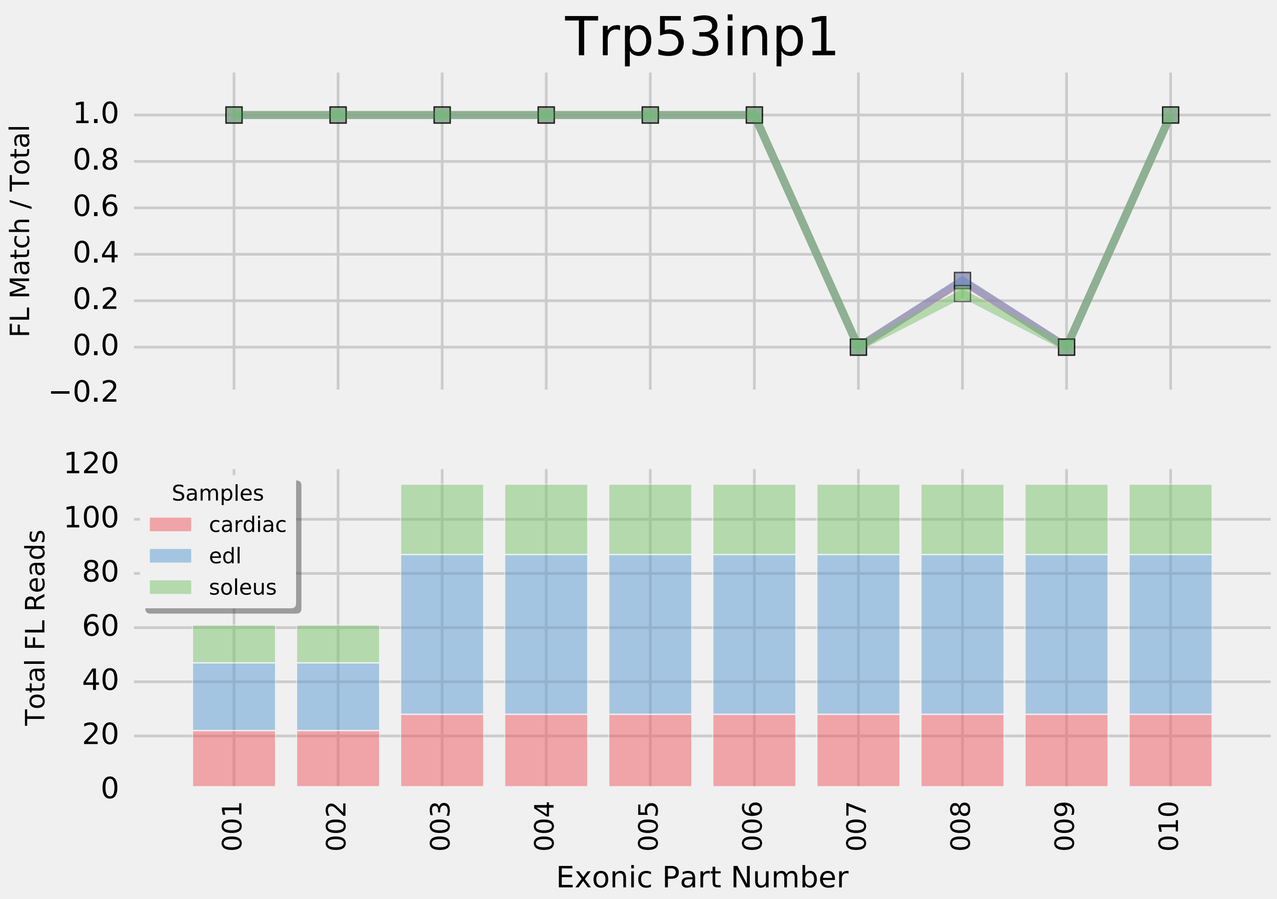
Trp53inp1
FL Match / Total
1.0
0.8
0.6
0.4
0.2
0.0
−0.2
Total FL Reads
120
100
80
60
40
20
0
Samples
cardiac
edl
soleus
001
002
003
004
005
006
007
008
009
010
Exonic Part Number

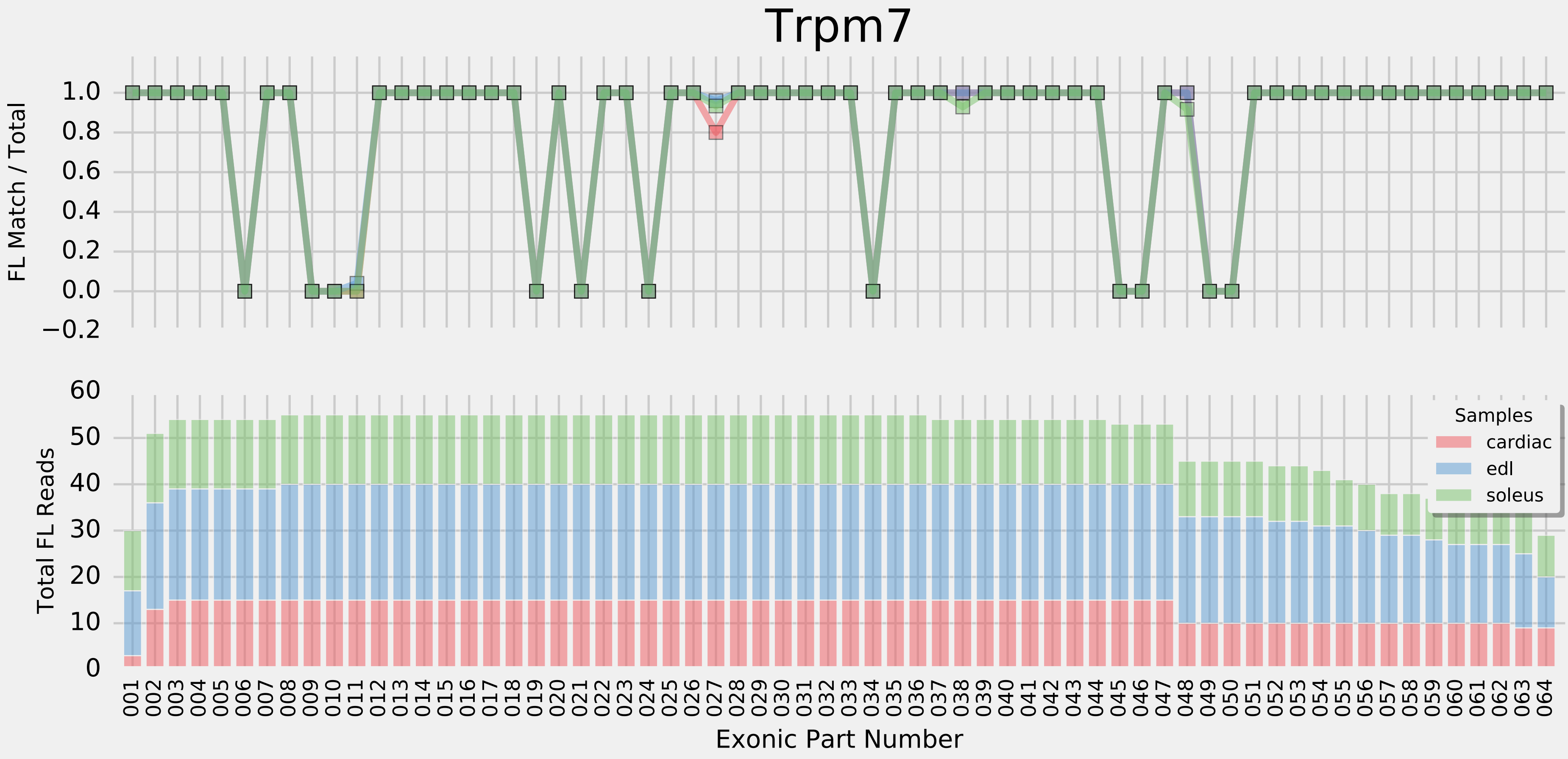

Trpm7
FL Match / Total
1.0
0.8
0.6
0.4
0.2
0.0
−0.2
Total FL Reads
60
50
40
30
20
10
0
Samples
cardiac
edl
soleus
001 002 003 004 005 006 007 008 009 010 011 012 013 014 015 016 017 018 019 020 021 022 023 024 025 026 027 028 029 030 031 032 033 034 035 036 037 038 039 040 041 042 043 044 045 046 047 048 049 050 051 052 053 054 055 056 057 058 059 060 061 062 063 064
Exonic Part Number

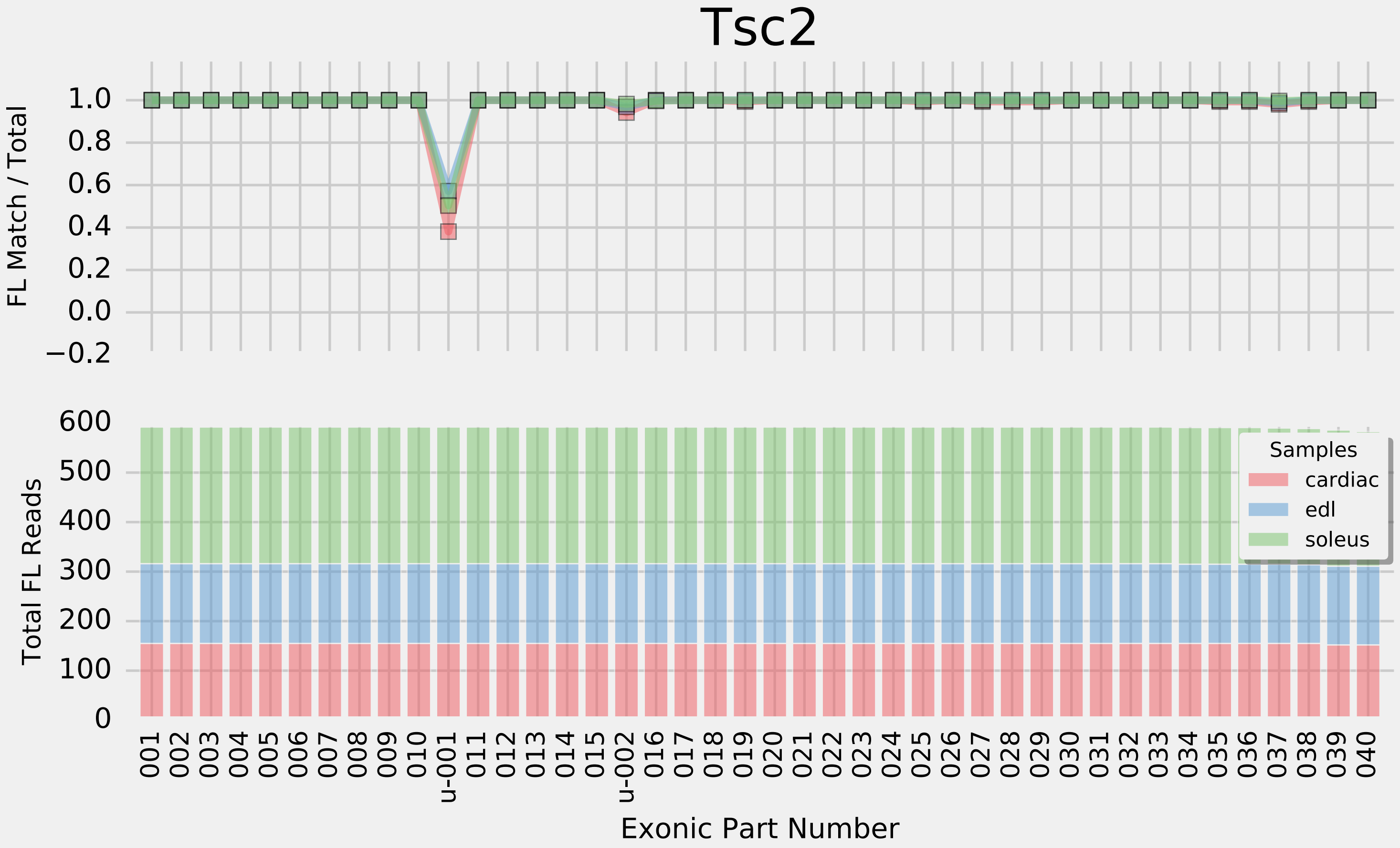

Tsc2
FL Match / Total
1.0
0.8
0.6
0.4
0.2
0.0
−0.2
Total FL Reads
600
500
400
300
200
100
0
Samples
cardiac
edl
soleus
001 002 003 004 005 006 007 008 009 010 u-001 011 012 013 014 015 u-002 016 017 018 019 020 021 022 023 024 025 026 027 028 029 030 031 032 033 034 035 036 037 038 039 040
Exonic Part Number

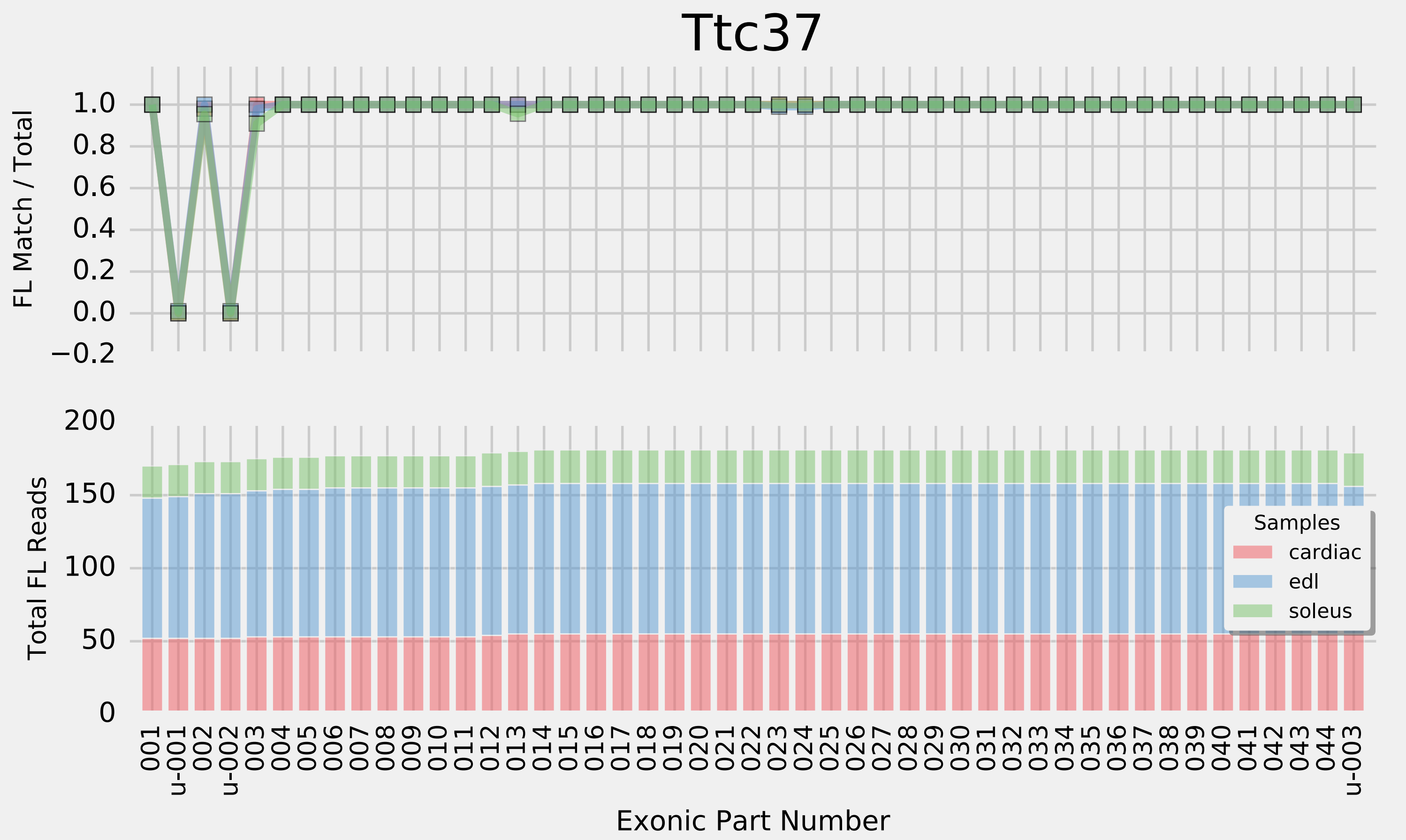

Ttc37
FL Match / Total
1.0
0.8
0.6
0.4
0.2
0.0
−0.2
Total FL Reads
200
150
100
50
0
Samples
cardiac
edl
soleus
001
u-001
002
u-002
003
004
005
006
007
008
009
010
011
012
013
014
015
016
017
018
019
020
021
022
023
024
025
026
027
028
029
030
031
032
033
034
035
036
037
038
039
040
041
042
043
044
u-003
Exonic Part Number

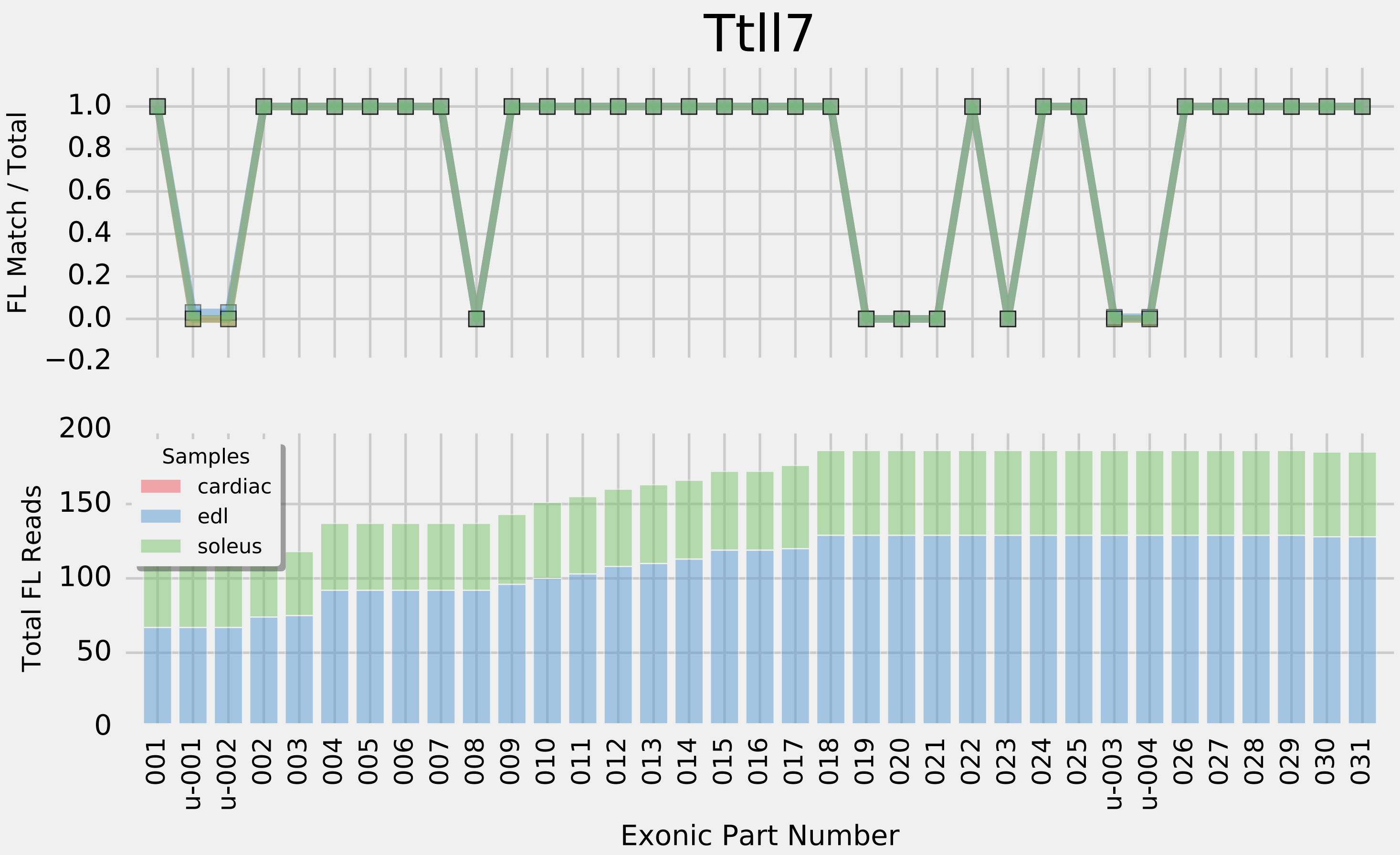

Ttll7
FL Match / Total
1.0
0.8
0.6
0.4
0.2
0.0
−0.2
Total FL Reads
200
150
100
50
0
Samples
cardiac
edl
soleus
001
u-001
u-002
002
003
004
005
006
007
008
009
010
011
012
013
014
015
016
017
018
019
020
021
022
023
024
025
u-003
u-004
026
027
028
029
030
031
Exonic Part Number

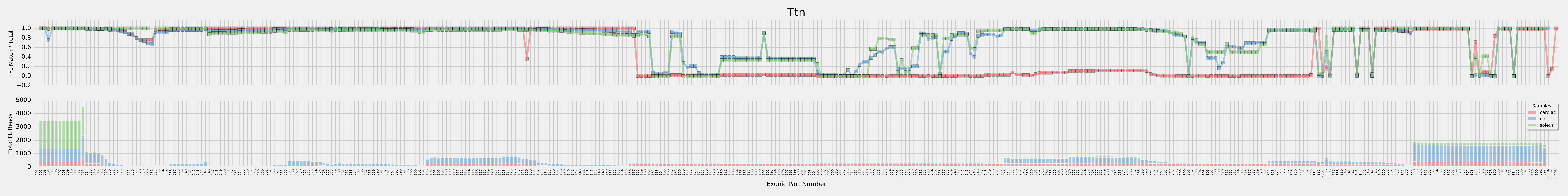

Ttn
FL Match / Total
Total FL Reads
Exonic Part Number
Samples
cardiac
edl
soleus

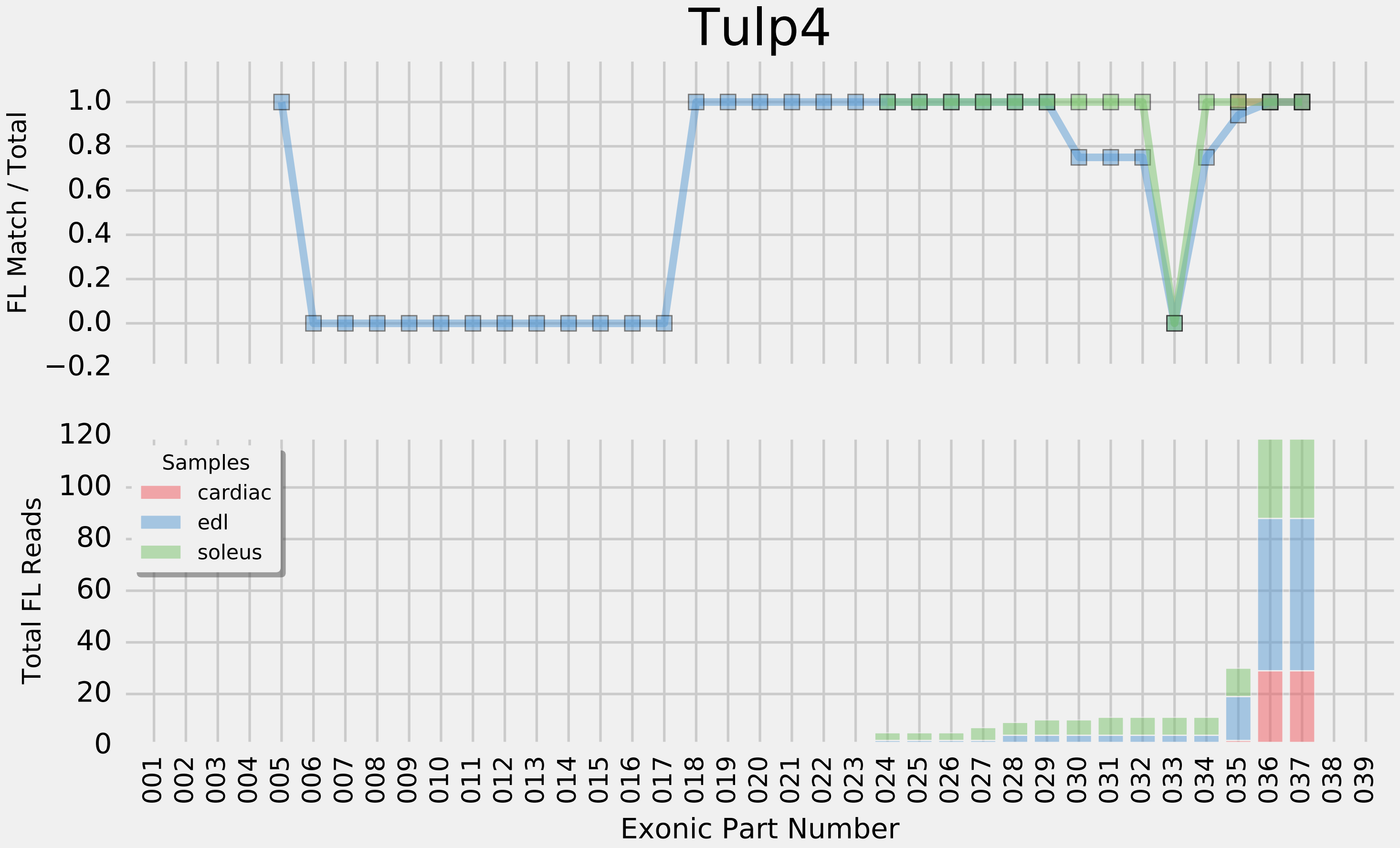

Tulp4
FL Match / Total
1.0
0.8
0.6
0.4
0.2
0.0
−0.2
Total FL Reads
120
100
80
60
40
20
0
Samples
cardiac
edl
soleus
001 002 003 004 005 006 007 008 009 010 011 012 013 014 015 016 017 018 019 020 021 022 023 024 025 026 027 028 029 030 031 032 033 034 035 036 037 038 039
Exonic Part Number

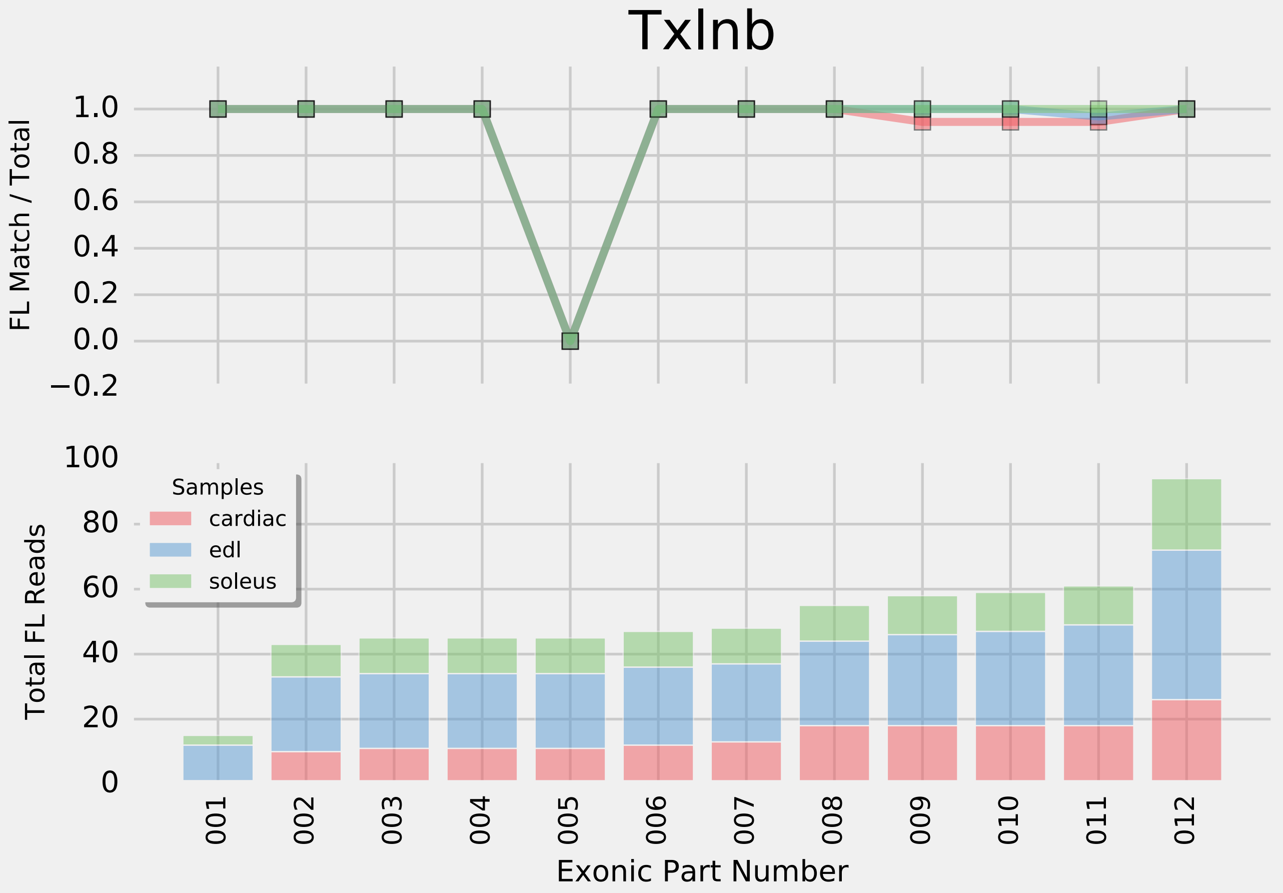
Txlnb
FL Match / Total
1.0
0.8
0.6
0.4
0.2
0.0
−0.2
Total FL Reads
100
80
60
40
20
0
Samples
cardiac
edl
soleus
001
002
003
004
005
006
007
008
009
010
011
012
Exonic Part Number

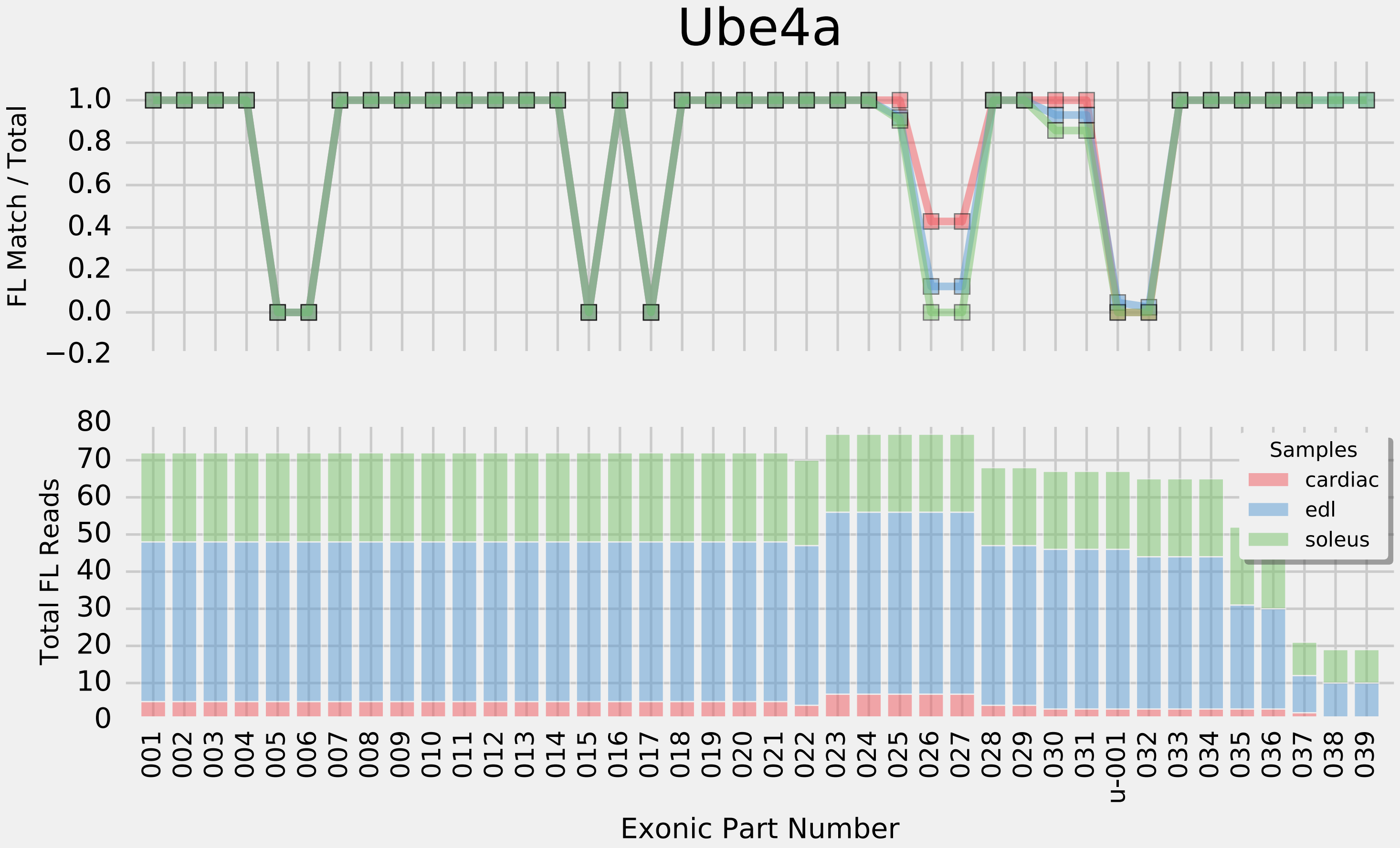
Ube4a
FL Match / Total
1.0
0.8
0.6
0.4
0.2
0.0
−0.2
Total FL Reads
80
70
60
50
40
30
20
10
0
Samples
cardiac
edl
soleus
001
002
003
004
005
006
007
008
009
010
011
012
013
014
015
016
017
018
019
020
021
022
023
024
025
026
027
028
029
030
031
u-001
032
033
034
035
036
037
038
039
Exonic Part Number

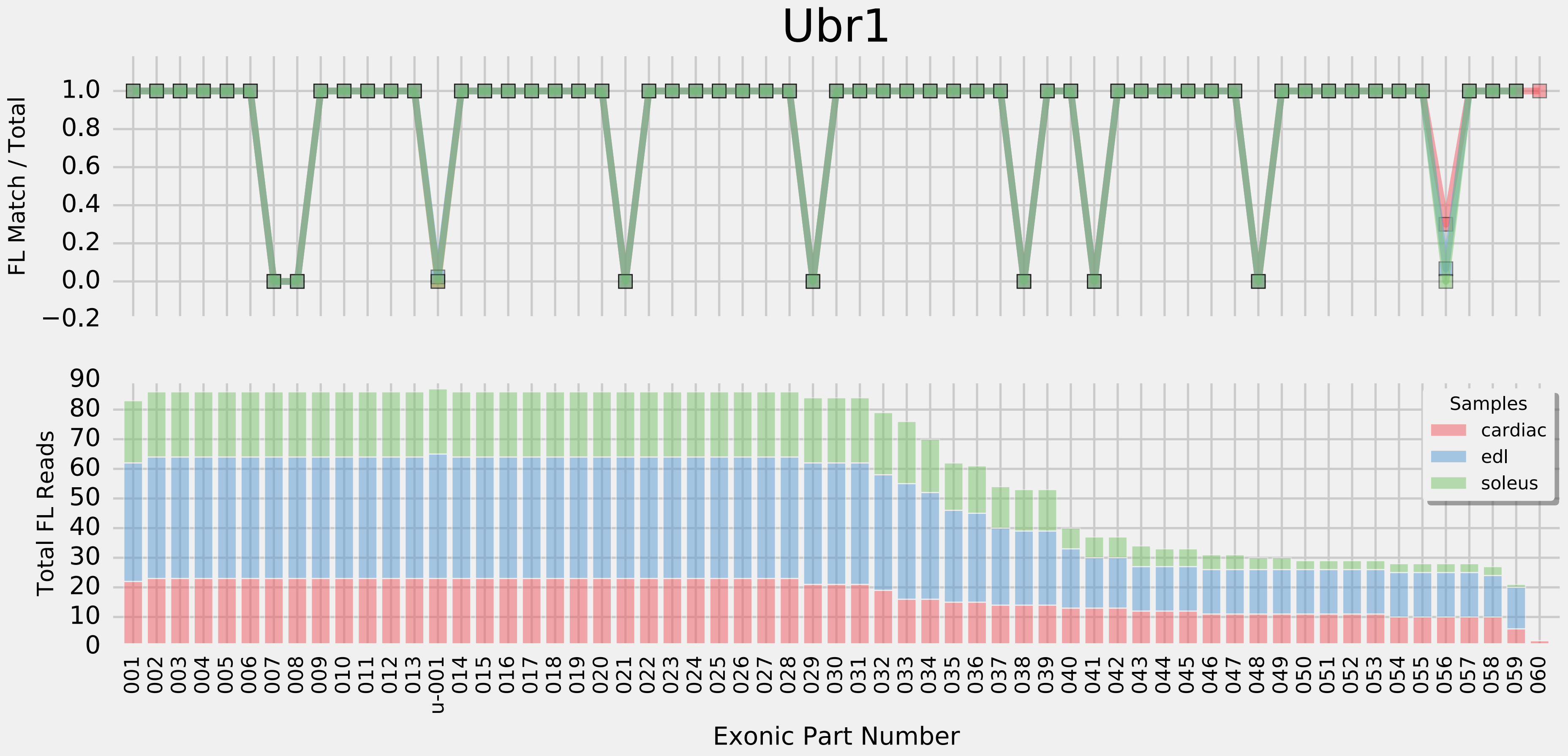

Ubr1
FL Match / Total
1.0
0.8
0.6
0.4
0.2
0.0
−0.2
Total FL Reads
90
80
70
60
50
40
30
20
10
0
Samples
cardiac
edl
soleus
001 002 003 004 005 006 007 008 009 010 011 012 013 u-001 014 015 016 017 018 019 020 021 022 023 024 025 026 027 028 029 030 031 032 033 034 035 036 037 038 039 040 041 042 043 044 045 046 047 048 049 050 051 052 053 054 055 056 057 058 059 060
Exonic Part Number

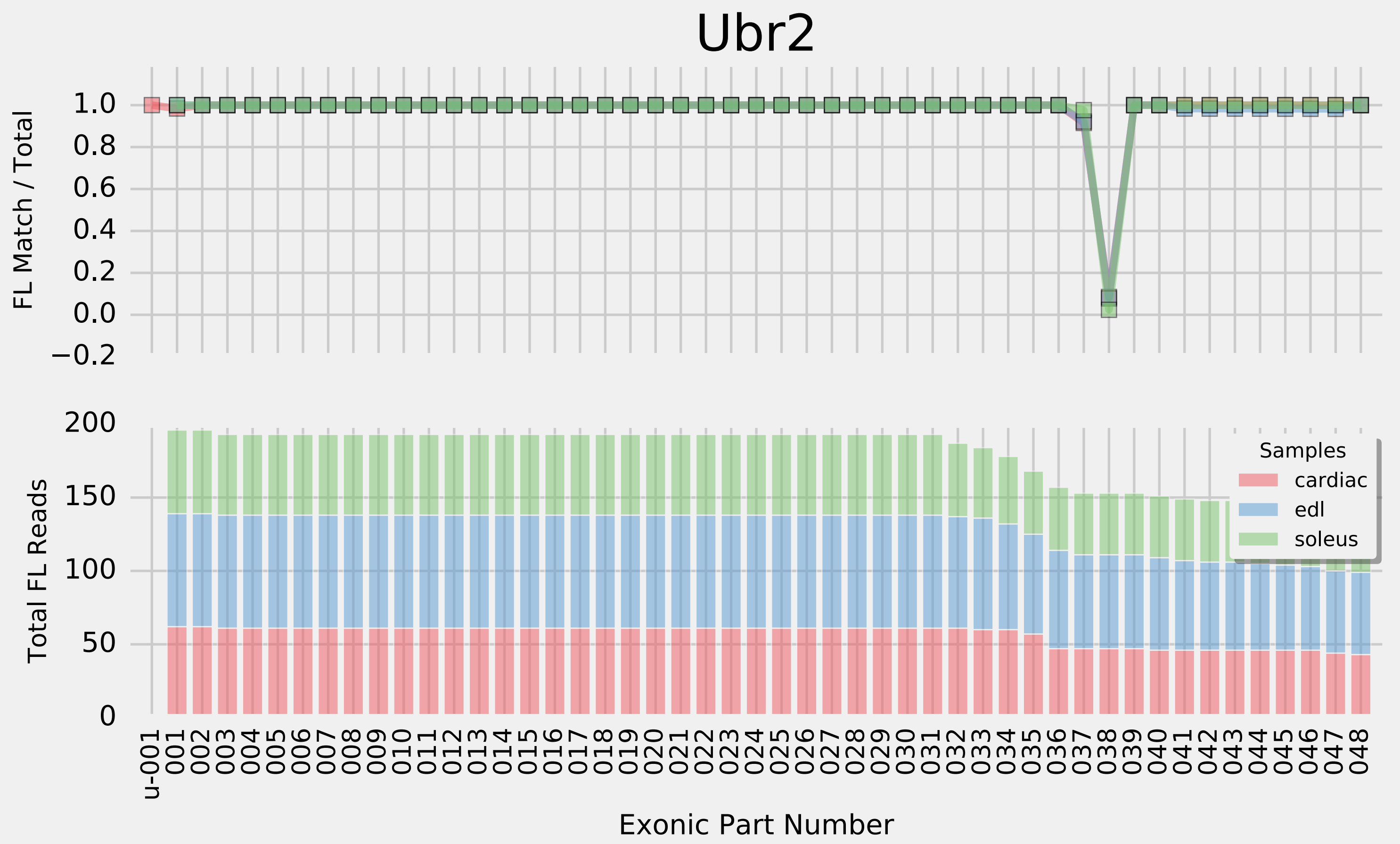

Ubr2
FL Match / Total
1.0
0.8
0.6
0.4
0.2
0.0
−0.2
Total FL Reads
200
150
100
50
0
Samples
cardiac
edl
soleus
u-001
001
002
003
004
005
006
007
008
009
010
011
012
013
014
015
016
017
018
019
020
021
022
023
024
025
026
027
028
029
030
031
032
033
034
035
036
037
038
039
040
041
042
043
044
045
046
047
048
Exonic Part Number

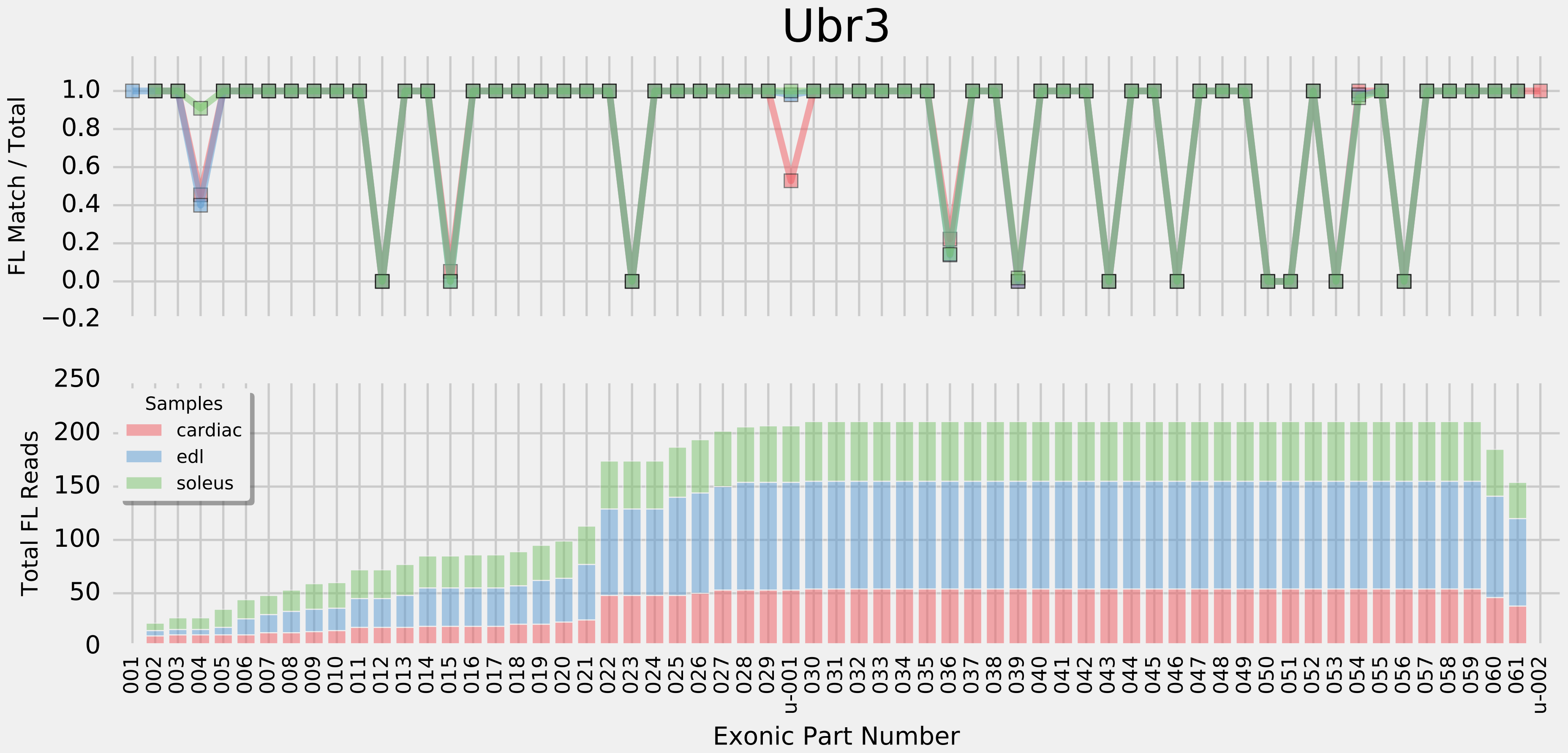

Ubr3
FL Match / Total
1.0
0.8
0.6
0.4
0.2
0.0
−0.2
250
200
150
100
50
0
Total FL Reads
Samples
cardiac
edl
soleus
001 002 003 004 005 006 007 008 009 010 011 012 013 014 015 016 017 018 019 020 021 022 023 024 025 026 027 028 029 u-001 030 031 032 033 034 035 036 037 038 039 040 041 042 043 044 045 046 047 048 049 050 051 052 053 054 055 056 057 058 059 060 061 u-002
Exonic Part Number

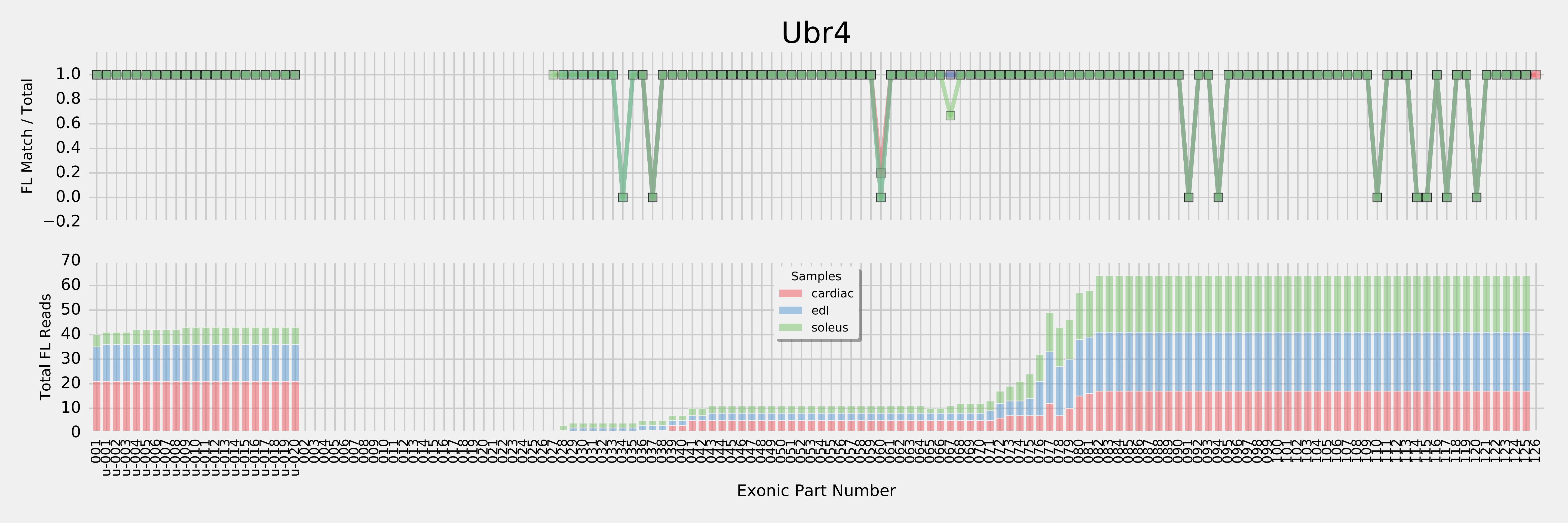
Ubr4
FL Match / Total
Total FL Reads
Samples
cardiac
edl
soleus
Exonic Part Number

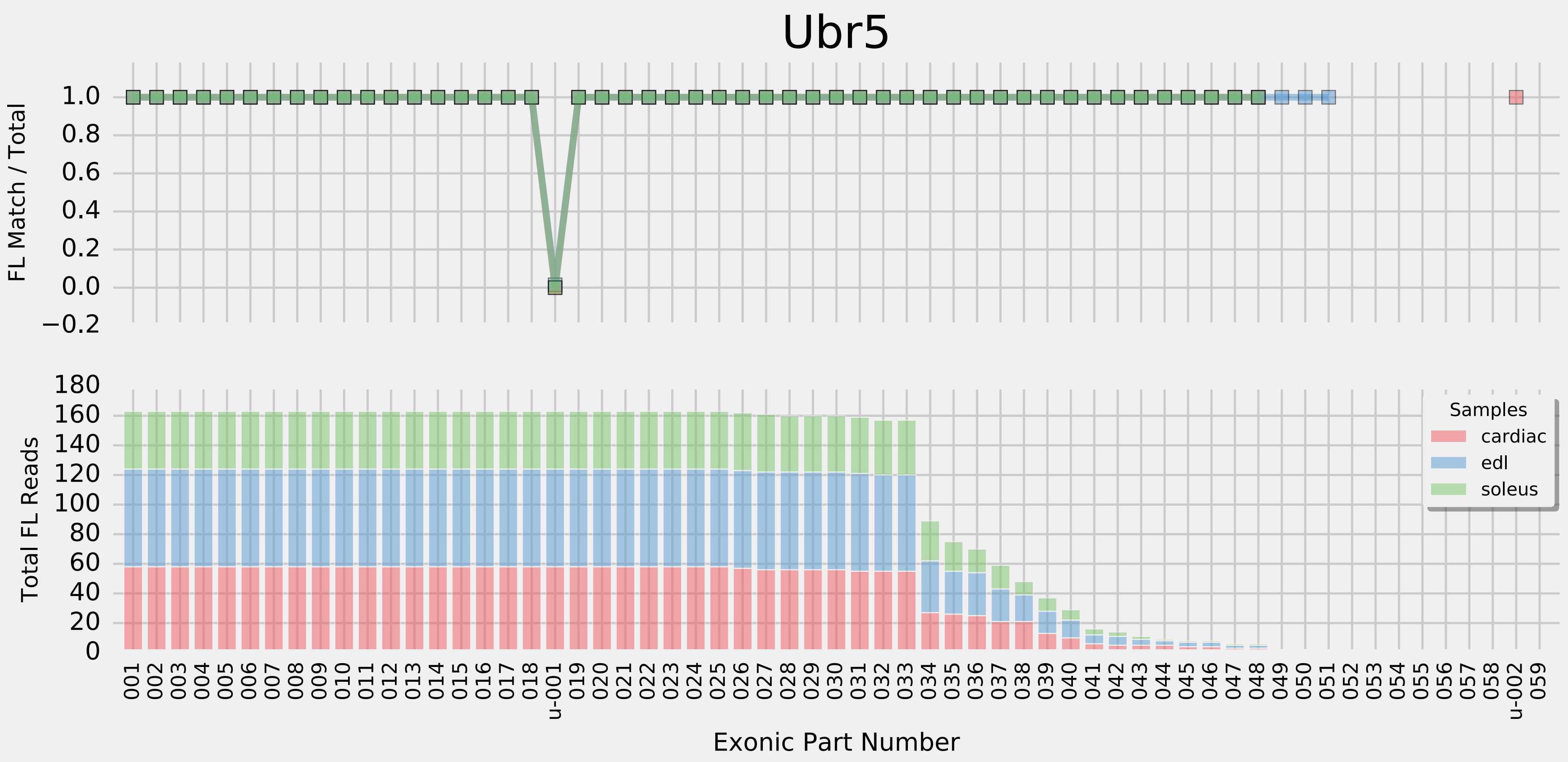
Ubr5
FL Match / Total
1.0
0.8
0.6
0.4
0.2
0.0
−0.2
Total FL Reads
180
160
140
120
100
80
60
40
20
0
Samples
cardiac
edl
soleus
001 002 003 004 005 006 007 008 009 010 011 012 013 014 015 016 017 018 u-001 019 020 021 022 023 024 025 026 027 028 029 030 031 032 033 034 035 036 037 038 039 040 041 042 043 044 045 046 047 048 049 050 051 052 053 054 055 056 057 058 u-002 059
Exonic Part Number

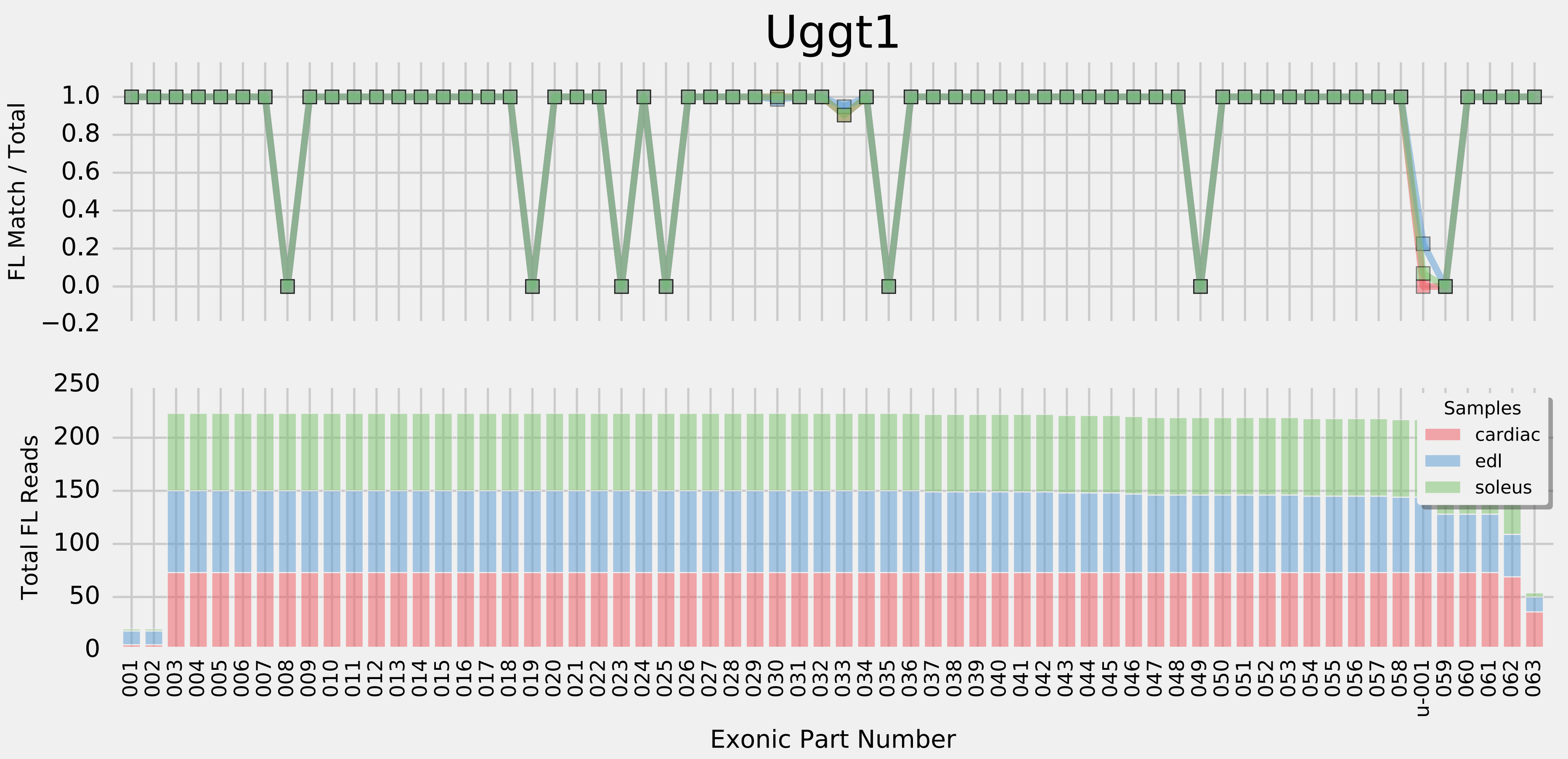
Uggt1
FL Match / Total
Total FL Reads
Exonic Part Number
Samples
cardiac
edl
soleus
001
002
003
004
005
006
007
008
009
010
011
012
013
014
015
016
017
018
019
020
021
022
023
024
025
026
027
028
029
030
031
032
033
034
035
036
037
038
039
040
041
042
043
044
045
046
047
048
049
050
051
052
053
054
055
056
057
058
u-001
059
060
061
062
063

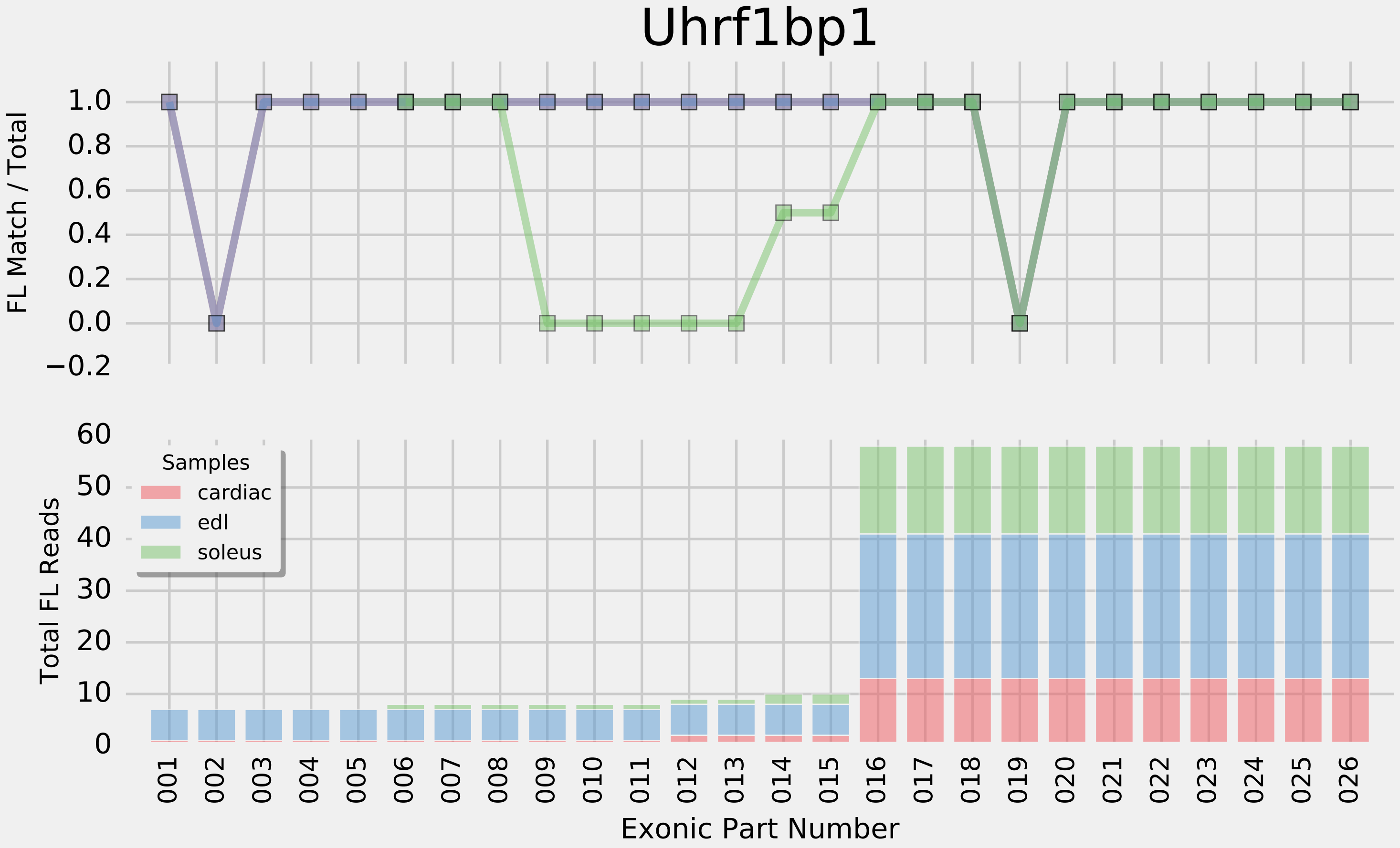

Uhrf1bp1
FL Match / Total
1.0
0.8
0.6
0.4
0.2
0.0
−0.2
Total FL Reads
60
50
40
30
20
10
0
Samples
cardiac
edl
soleus
001
002
003
004
005
006
007
008
009
010
011
012
013
014
015
016
017
018
019
020
021
022
023
024
025
026
Exonic Part Number

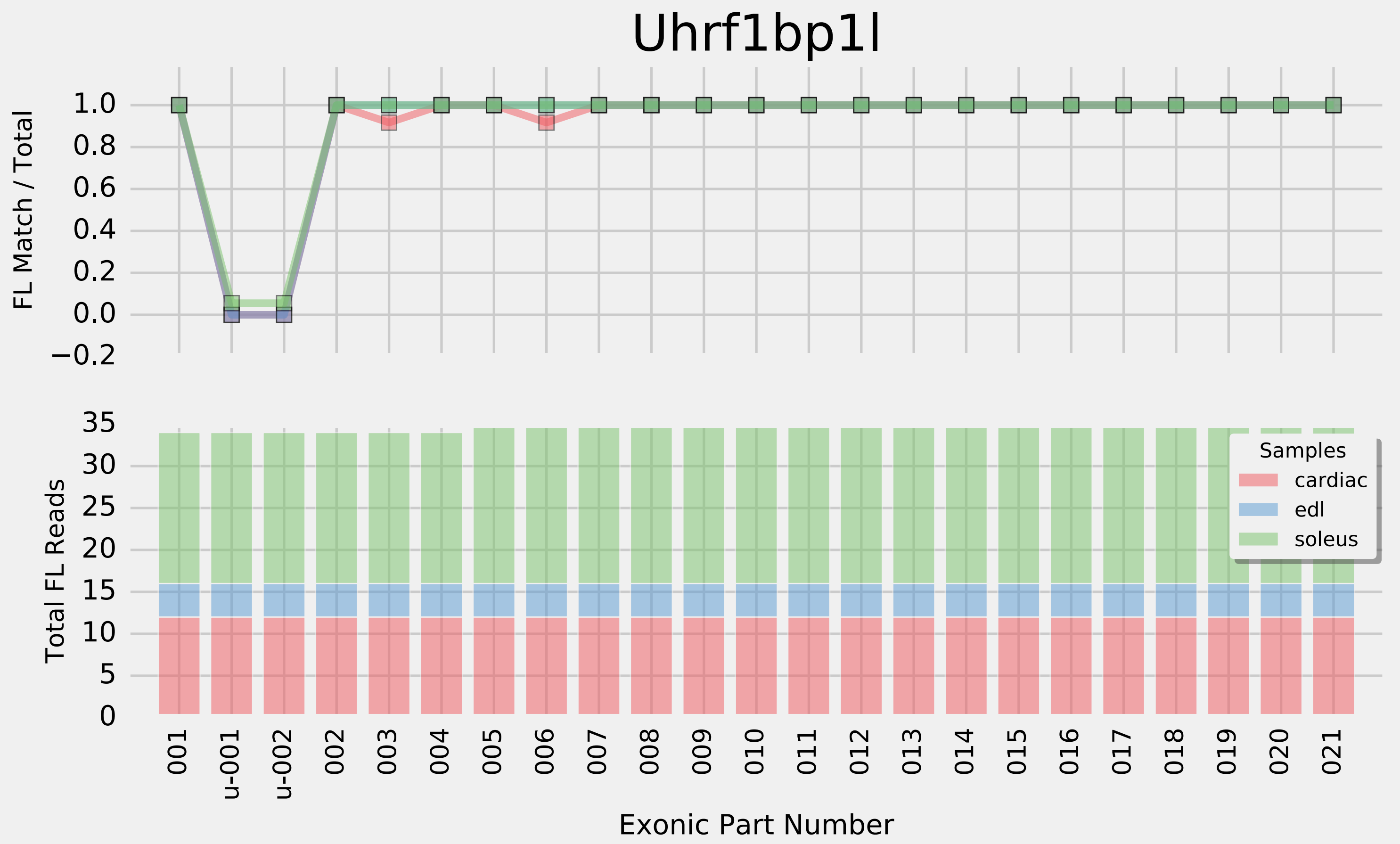

Uhrf1bp1l
FL Match / Total
1.0
0.8
0.6
0.4
0.2
0.0
−0.2
Total FL Reads
35
30
25
20
15
10
5
0
Samples
cardiac
edl
soleus
001
u-001
u-002
002
003
004
005
006
007
008
009
010
011
012
013
014
015
016
017
018
019
020
021
Exonic Part Number

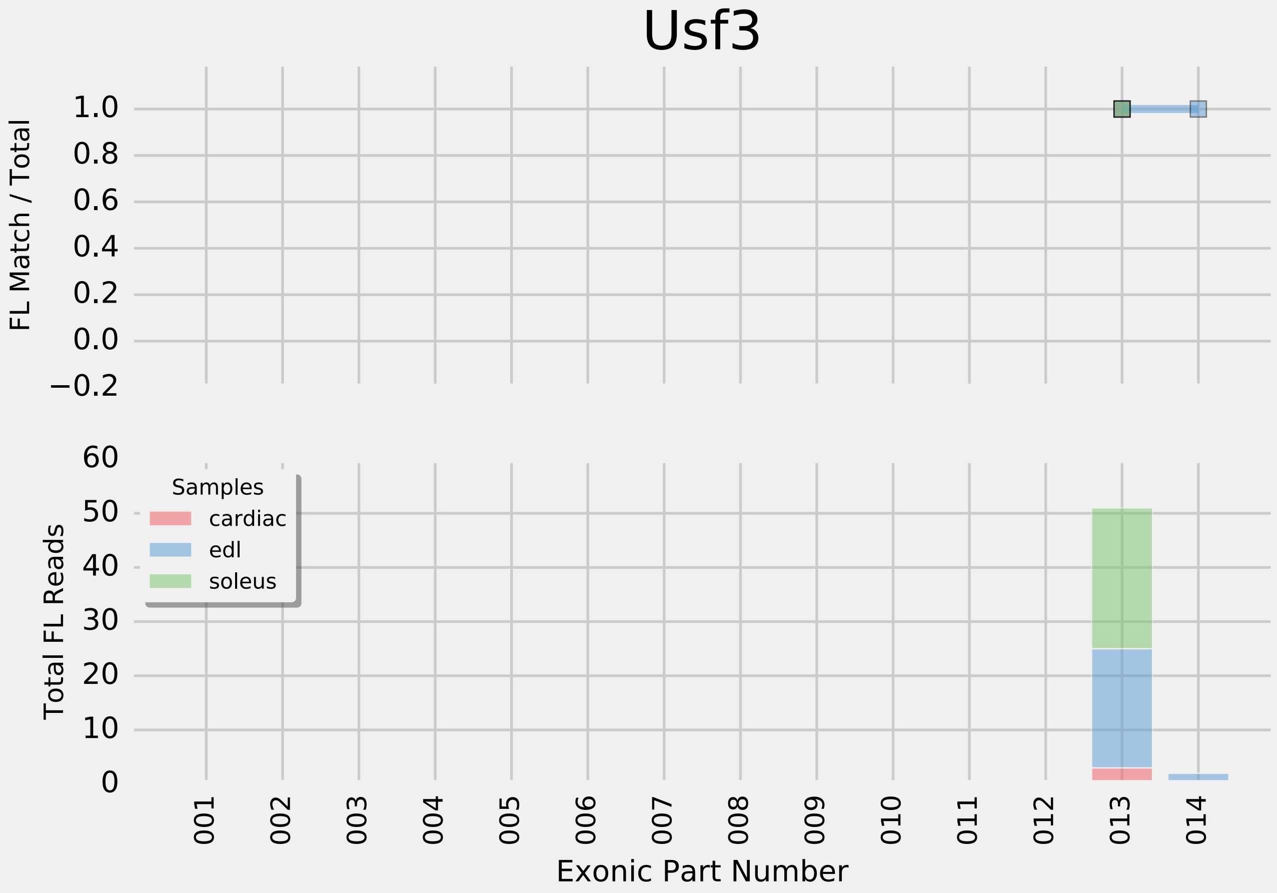

Usf3
FL Match / Total
1.0
0.8
0.6
0.4
0.2
0.0
−0.2
Total FL Reads
60
50
40
30
20
10
0
Samples
cardiac
edl
soleus
001
002
003
004
005
006
007
008
009
010
011
012
013
014
Exonic Part Number

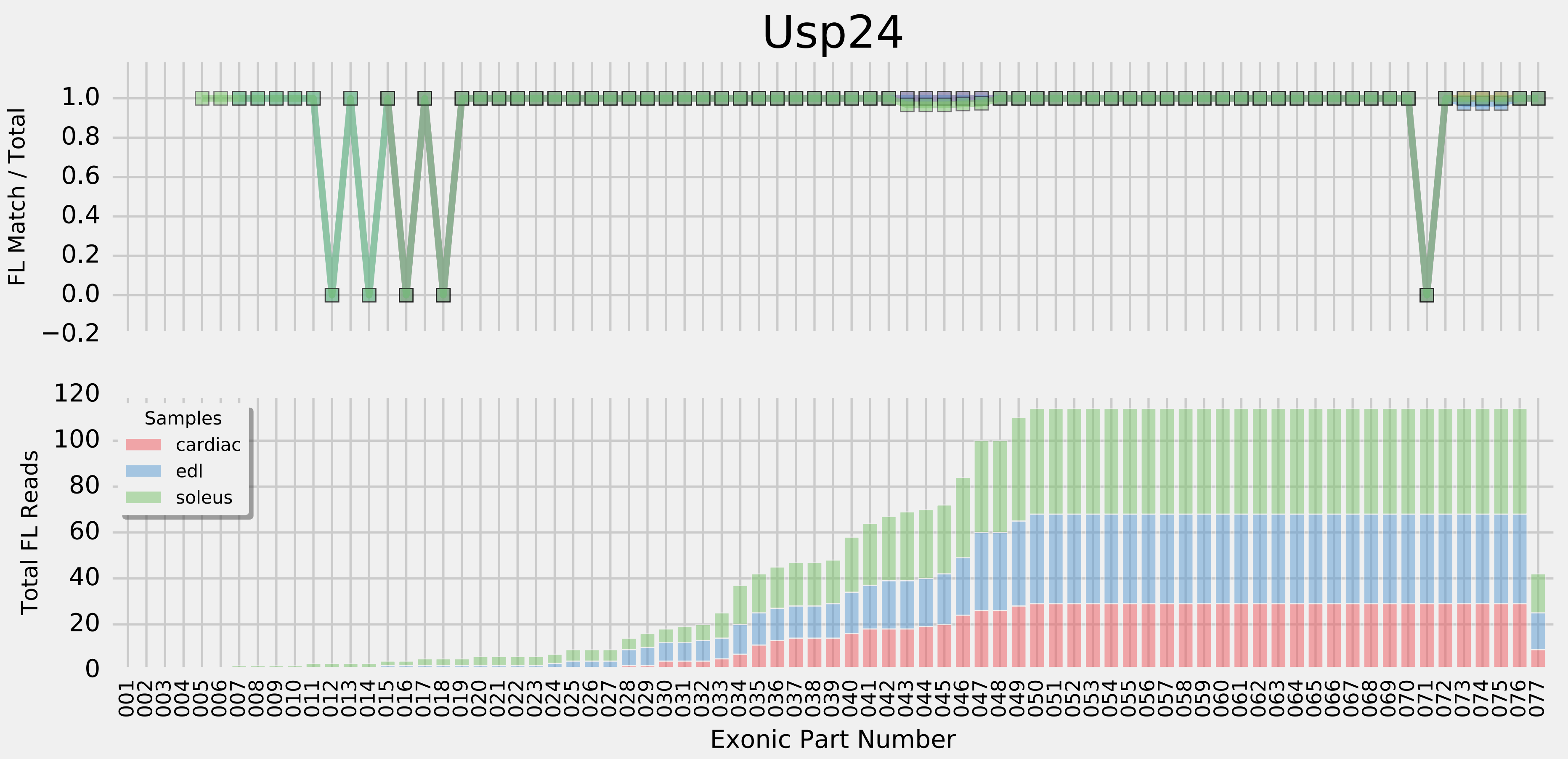

Usp24
FL Match / Total
1.0
0.8
0.6
0.4
0.2
0.0
−0.2
Total FL Reads
120
100
80
60
40
20
0
Samples
cardiac
edl
soleus
001 002 003 004 005 006 007 008 009 010 011 012 013 014 015 016 017 018 019 020 021 022 023 024 025 026 027 028 029 030 031 032 033 034 035 036 037 038 039 040 041 042 043 044 045 046 047 048 049 050 051 052 053 054 055 056 057 058 059 060 061 062 063 064 065 066 067 068 069 070 071 072 073 074 075 076 077
Exonic Part Number

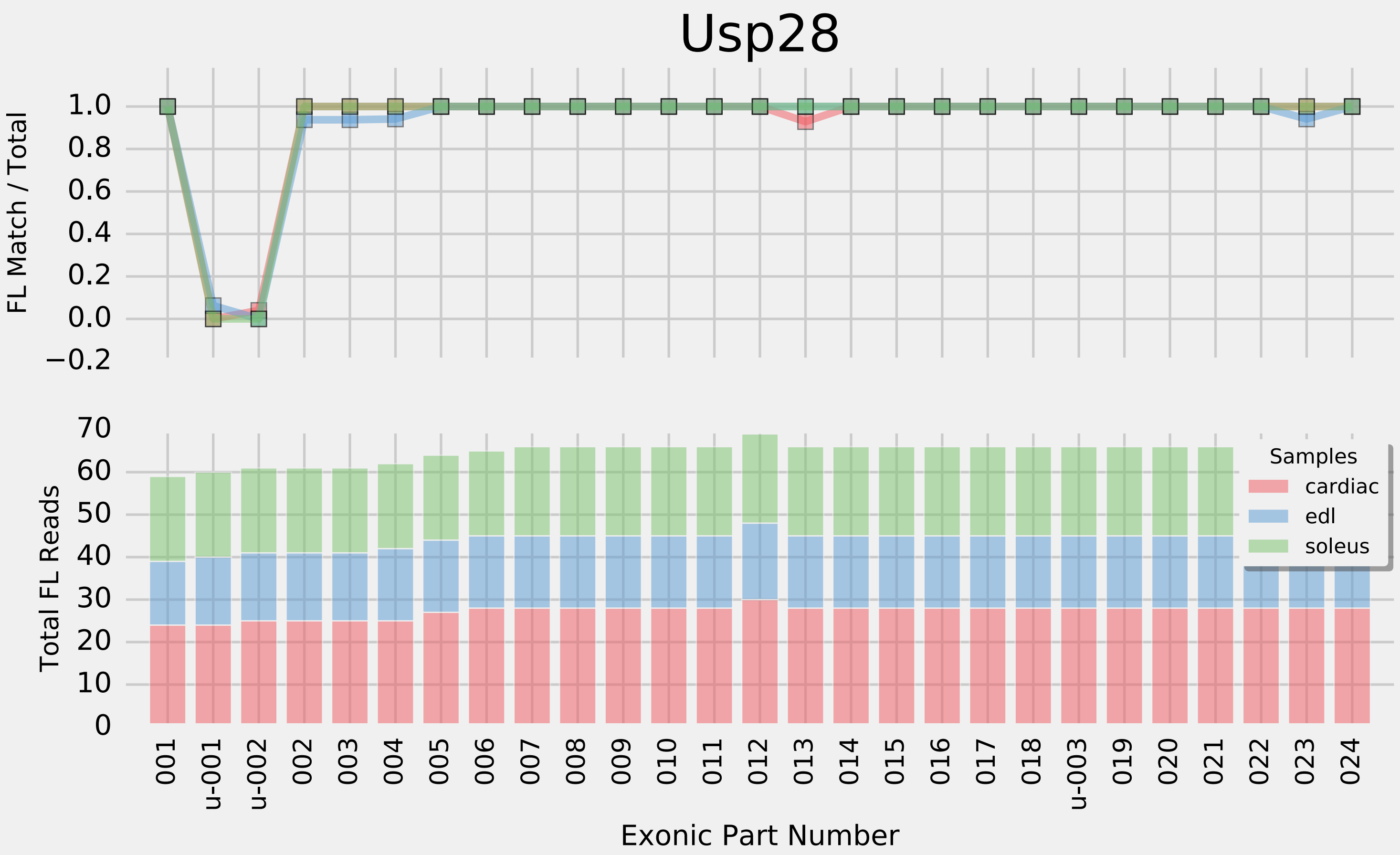

Usp28
FL Match / Total
1.0
0.8
0.6
0.4
0.2
0.0
−0.2
Total FL Reads
70
60
50
40
30
20
10
0
Samples
cardiac
edl
soleus
001
u-001
u-002
002
003
004
005
006
007
008
009
010
011
012
013
014
015
016
017
018
u-003
019
020
021
022
023
024
Exonic Part Number

Usp34

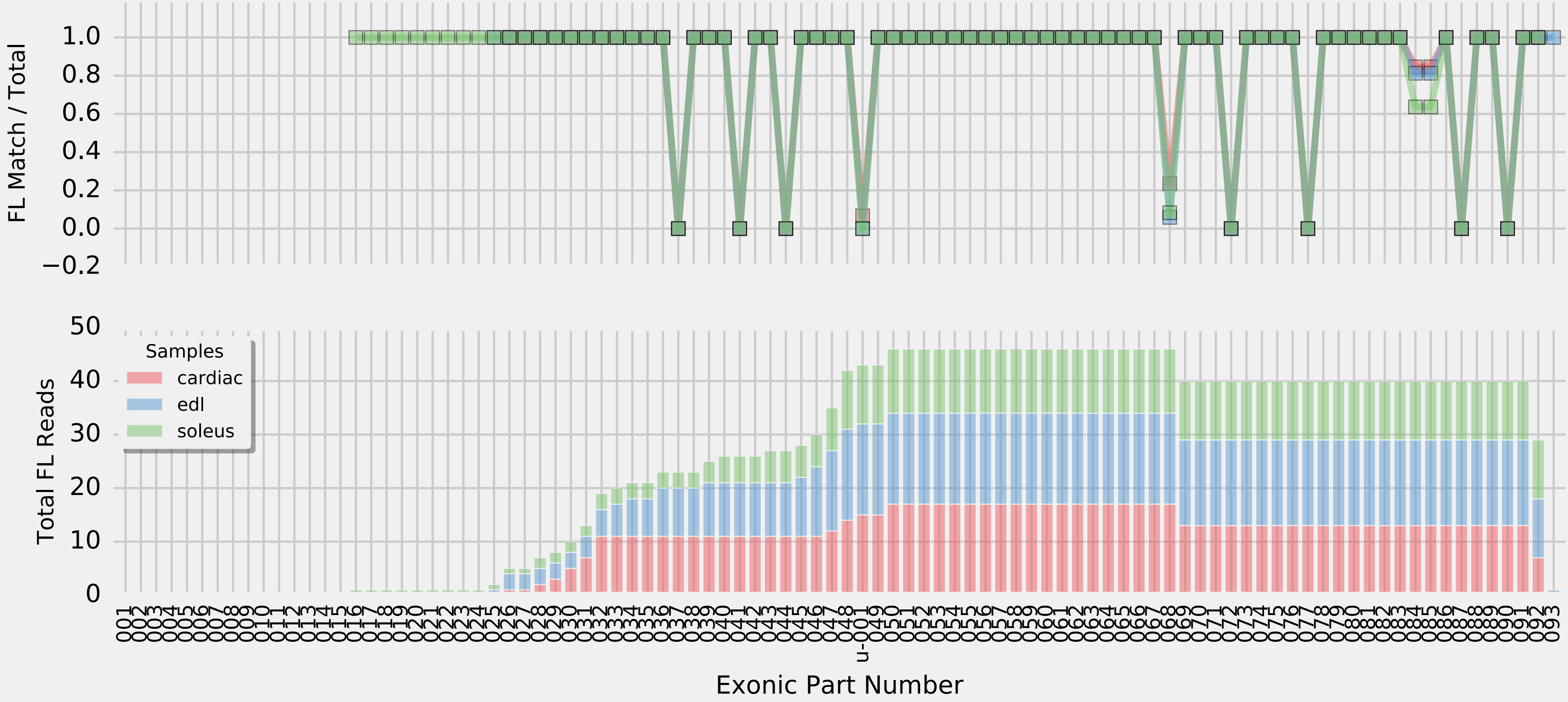

FL Match / Total
1.0
0.8
0.6
0.4
0.2
0.0
−0.2
Samples
cardiac
edl
soleus
Total FL Reads
50
40
30
20
10
0
Exonic Part Number

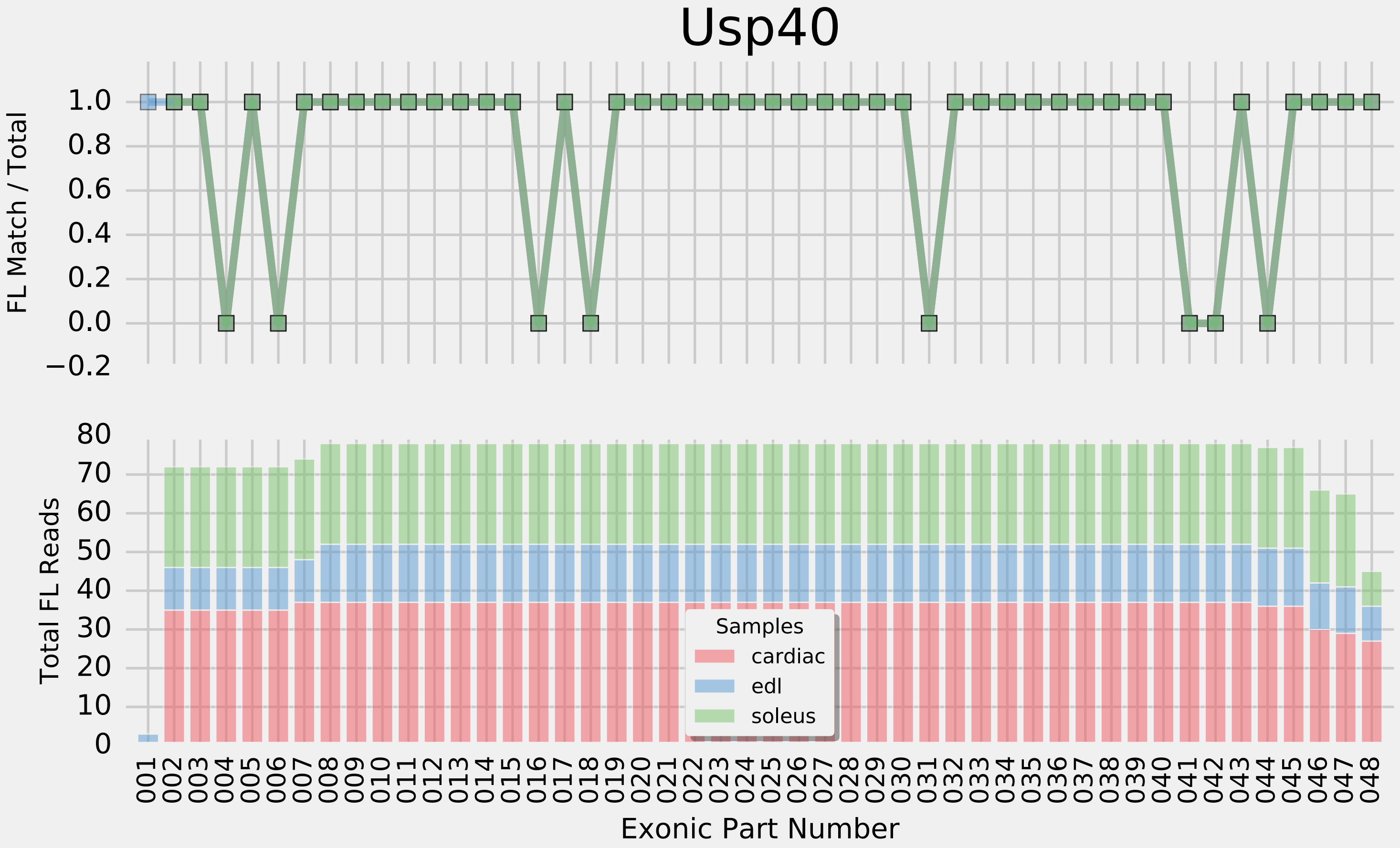

Usp40
FL Match / Total
1.0
0.8
0.6
0.4
0.2
0.0
−0.2
Total FL Reads
80
70
60
50
40
30
20
10
0
Samples
cardiac
edl
soleus
001 002 003 004 005 006 007 008 009 010 011 012 013 014 015 016 017 018 019 020 021 022 023 024 025 026 027 028 029 030 031 032 033 034 035 036 037 038 039 040 041 042 043 044 045 046 047 048
Exonic Part Number

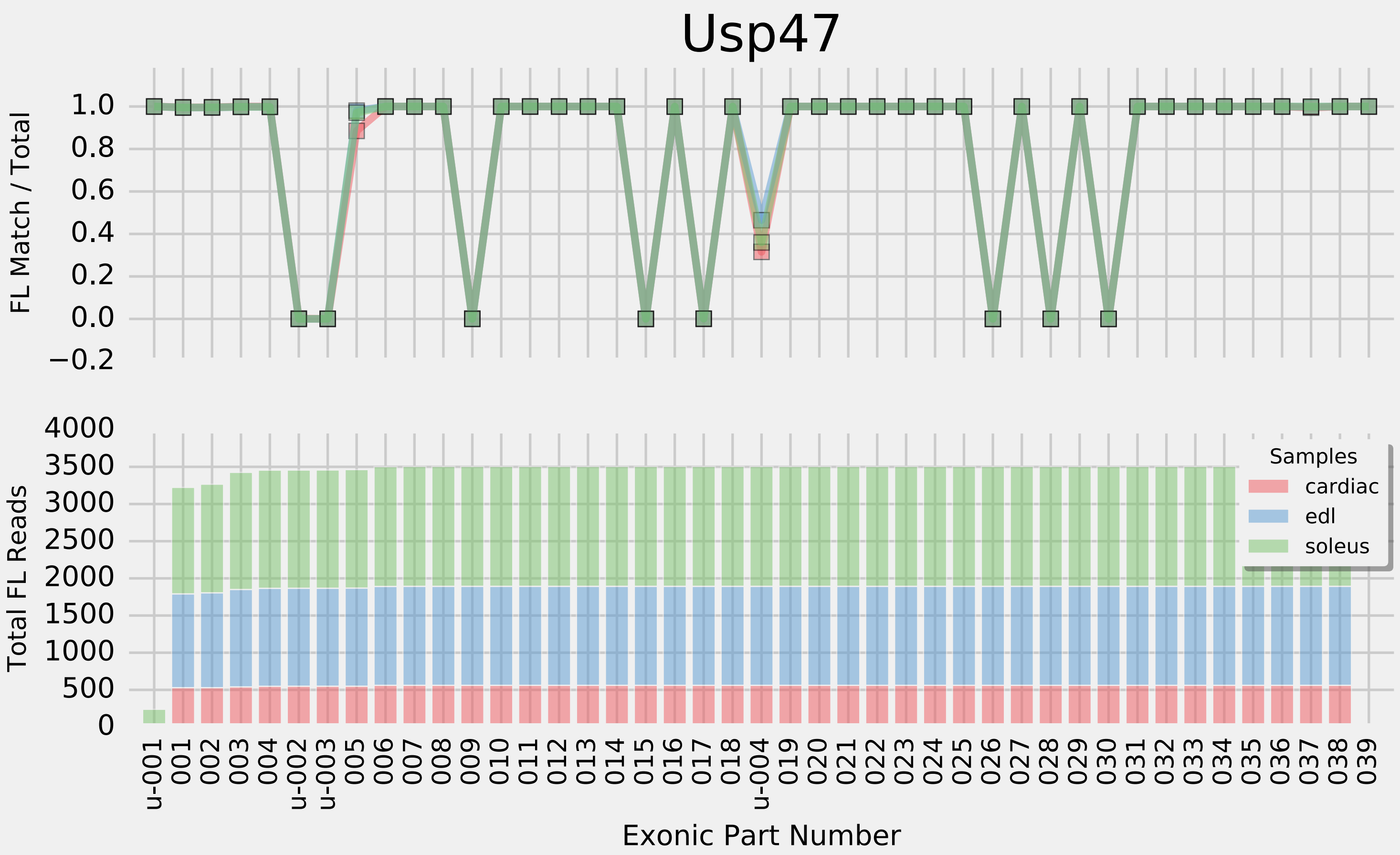

Usp47
FL Match / Total
1.0
0.8
0.6
0.4
0.2
0.0
−0.2
4000
3500
3000
2500
2000
1500
1000
500
0
Total FL Reads
Samples
cardiac
edl
soleus
u-001 001 002 003 004 u-002 u-003 005 006 007 008 009 010 011 012 013 014 015 016 017 018 u-004 019 020 021 022 023 024 025 026 027 028 029 030 031 032 033 034 035 036 037 038 039
Exonic Part Number

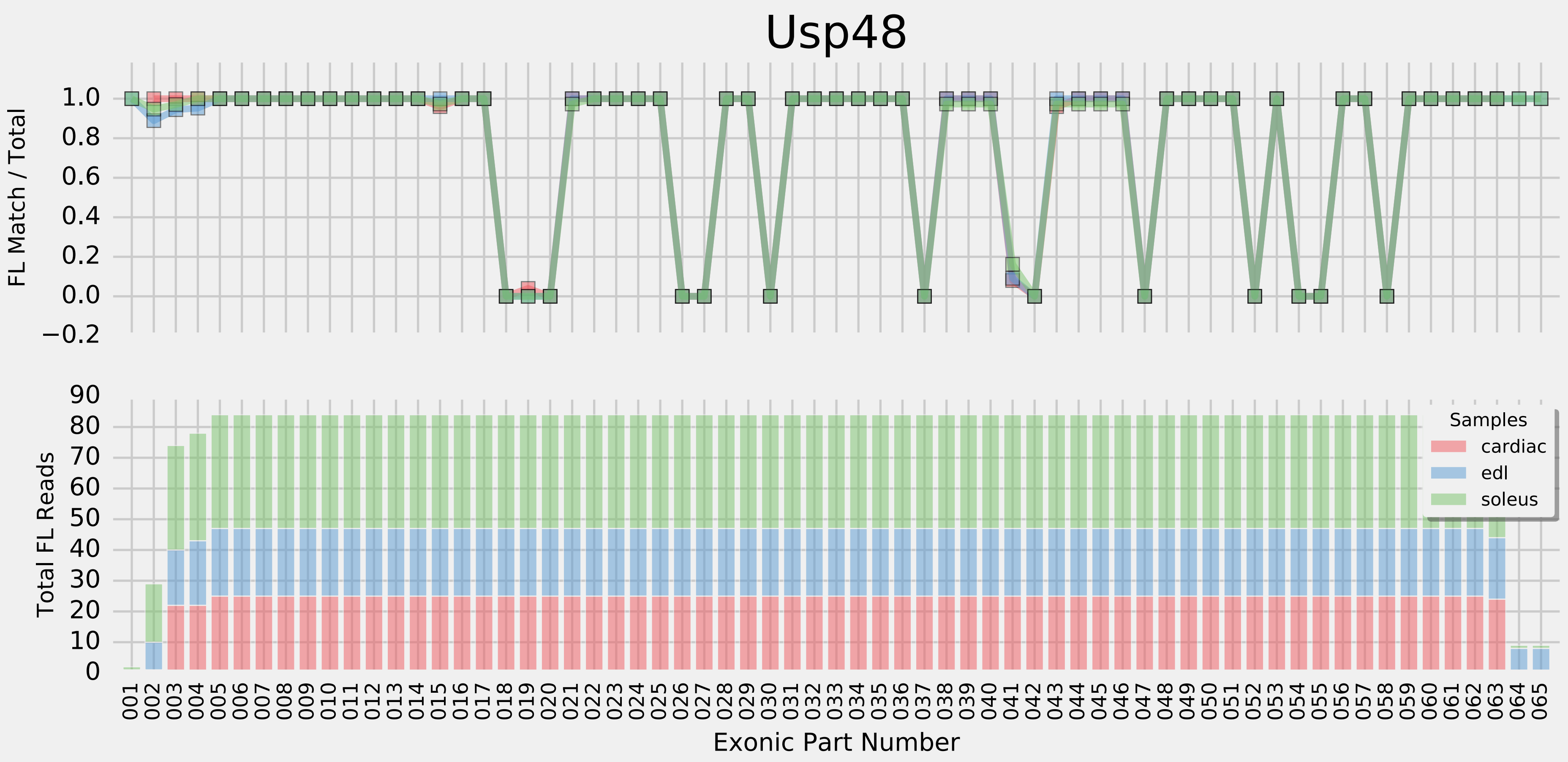
Usp48
FL Match / Total
Total FL Reads
Exonic Part Number
Samples
cardiac
edl
soleus

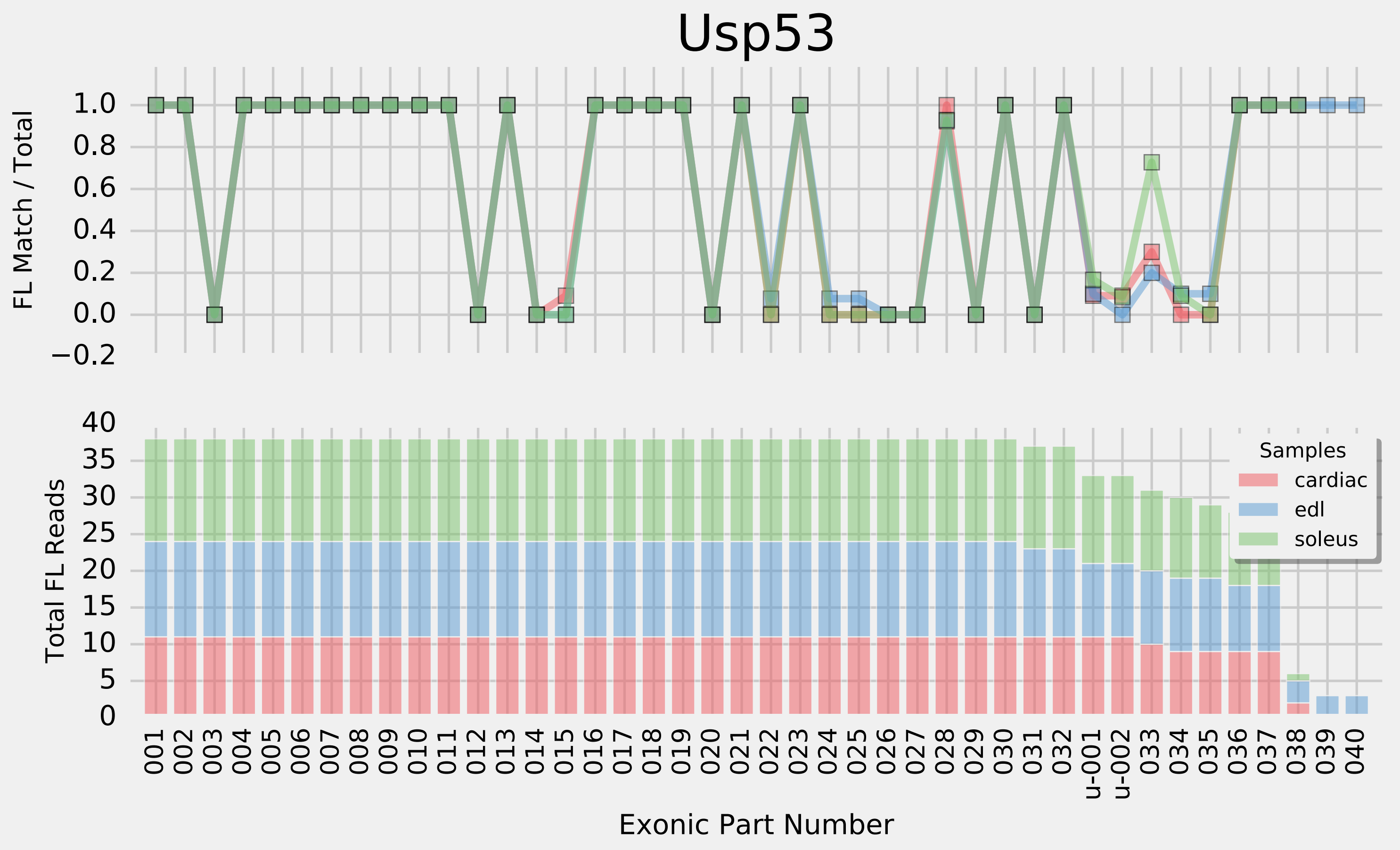

Usp53
FL Match / Total
1.0
0.8
0.6
0.4
0.2
0.0
−0.2
Total FL Reads
40
35
30
25
20
15
10
5
0
Samples
cardiac
edl
soleus
001 002 003 004 005 006 007 008 009 010 011 012 013 014 015 016 017 018 019 020 021 022 023 024 025 026 027 028 029 030 031 032 u-001 u-002 033 034 035 036 037 038 039 040
Exonic Part Number

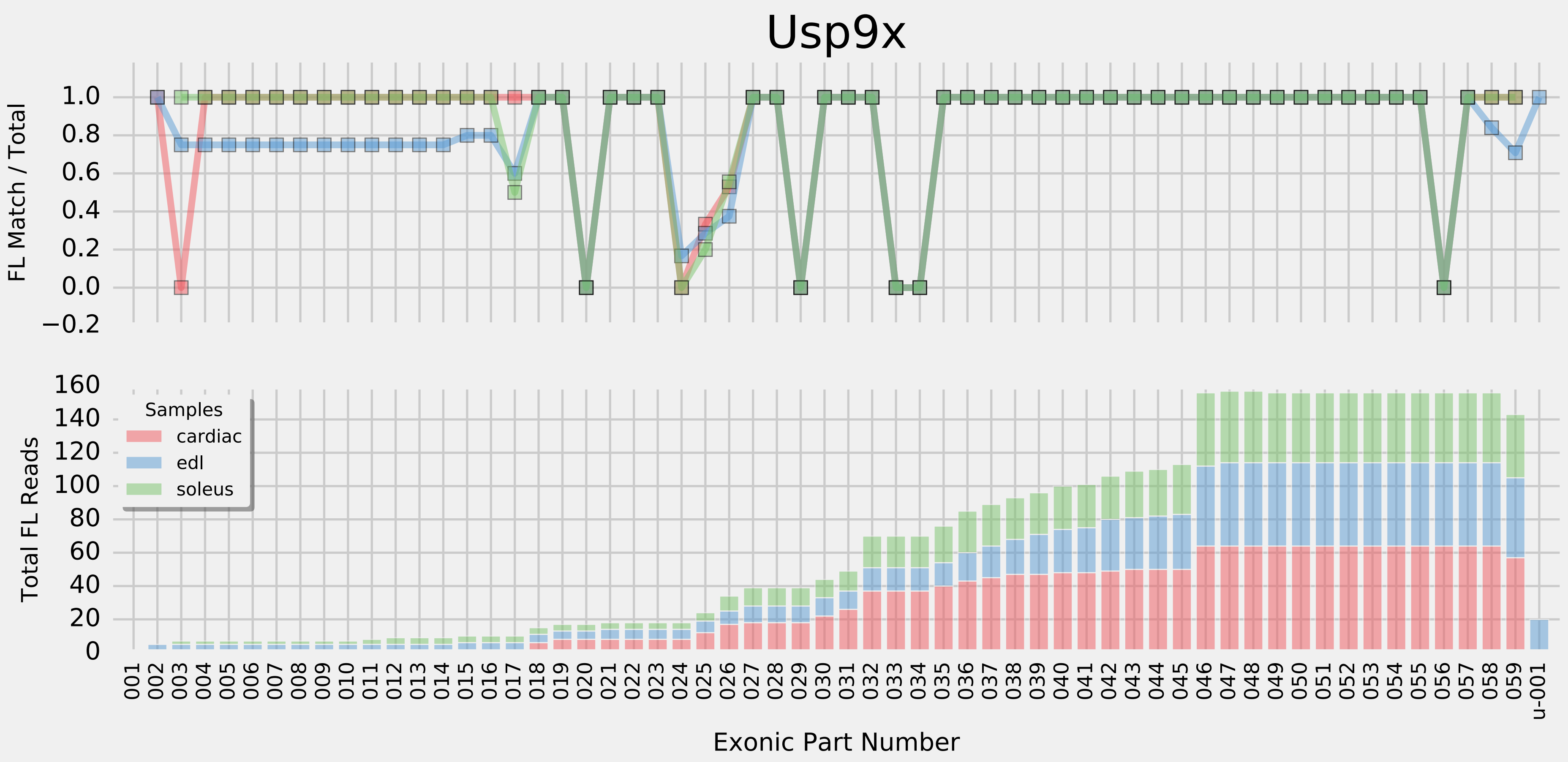
Usp9x
FL Match / Total
Total FL Reads
Samples
cardiac
edl
soleus
Exonic Part Number

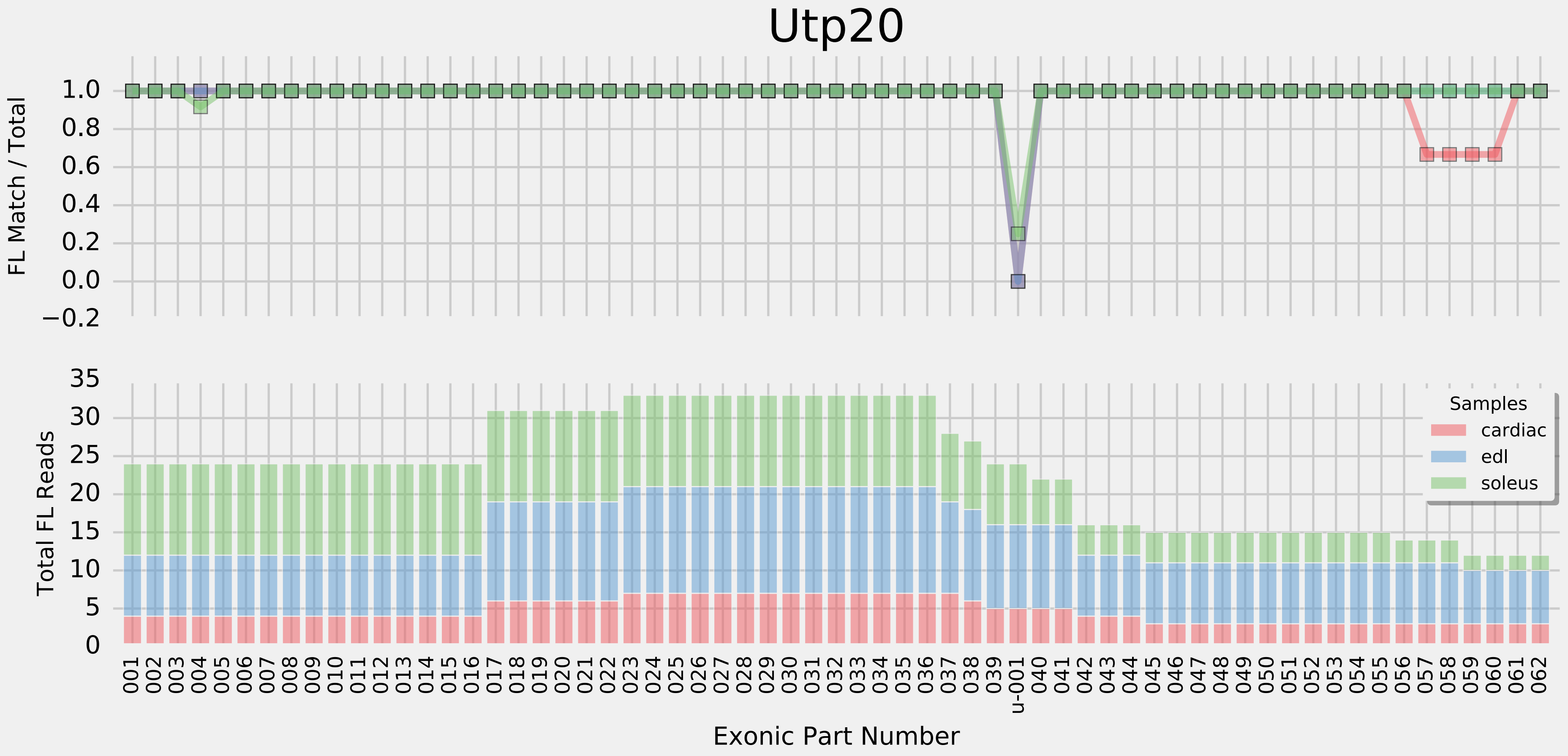

Utp20
FL Match / Total
1.0
0.8
0.6
0.4
0.2
0.0
−0.2
Total FL Reads
35
30
25
20
15
10
5
0
Samples
cardiac
edl
soleus
001 002 003 004 005 006 007 008 009 010 011 012 013 014 015 016 017 018 019 020 021 022 023 024 025 026 027 028 029 030 031 032 033 034 035 036 037 038 039 u-001 040 041 042 043 044 045 046 047 048 049 050 051 052 053 054 055 056 057 058 059 060 061 062
Exonic Part Number

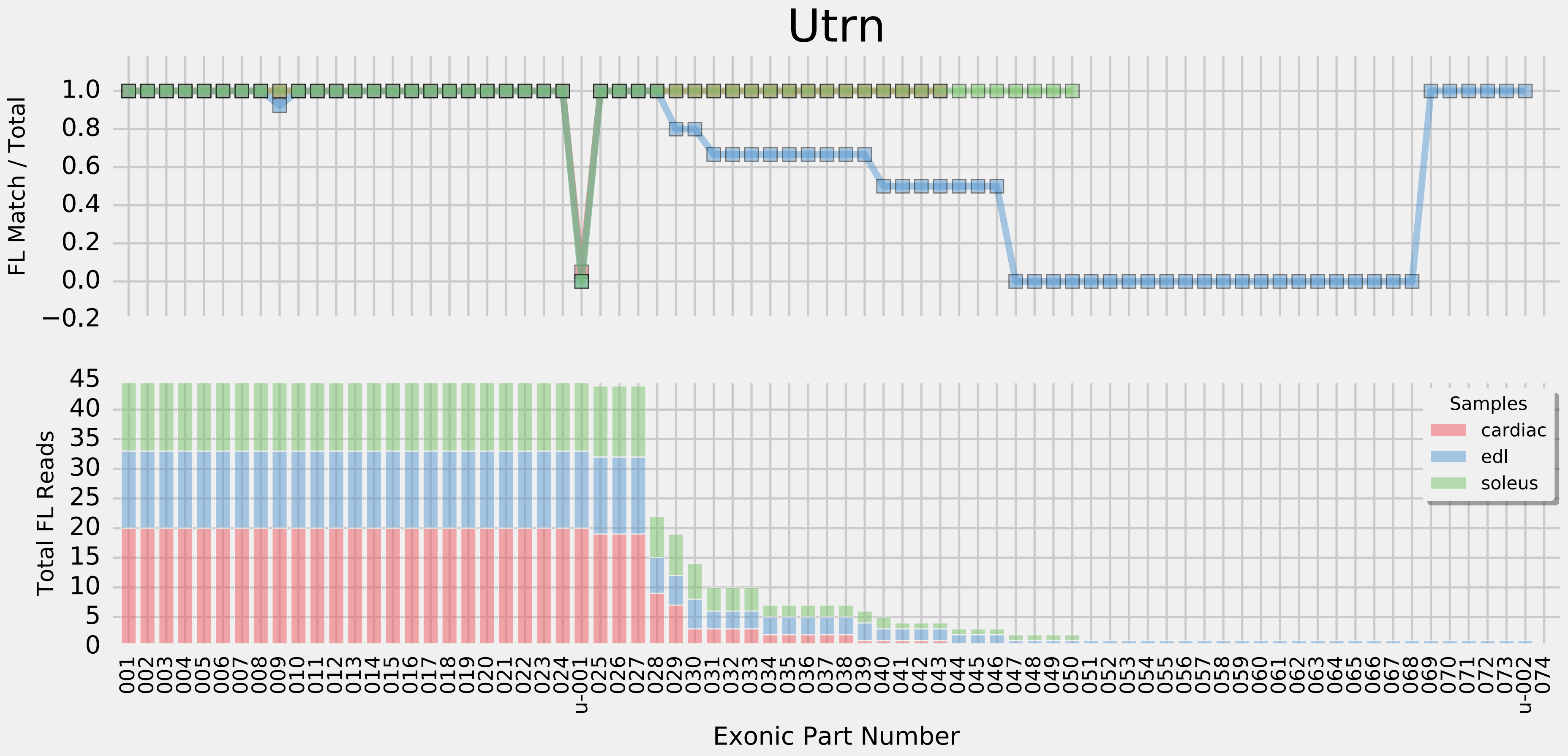

Utrn
FL Match / Total
1.0
0.8
0.6
0.4
0.2
0.0
−0.2
Total FL Reads
45
40
35
30
25
20
15
10
5
0
Samples
cardiac
edl
soleus
Exonic Part Number
001 002 003 004 005 006 007 008 009 010 011 012 013 014 015 016 017 018 019 020 021 022 023 024 u-001 025 026 027 028 029 030 031 032 033 034 035 036 037 038 039 040 041 042 043 044 045 046 047 048 049 050 051 052 053 054 055 056 057 058 059 060 061 062 063 064 065 066 067 068 069 070 071 072 073 u-002 074

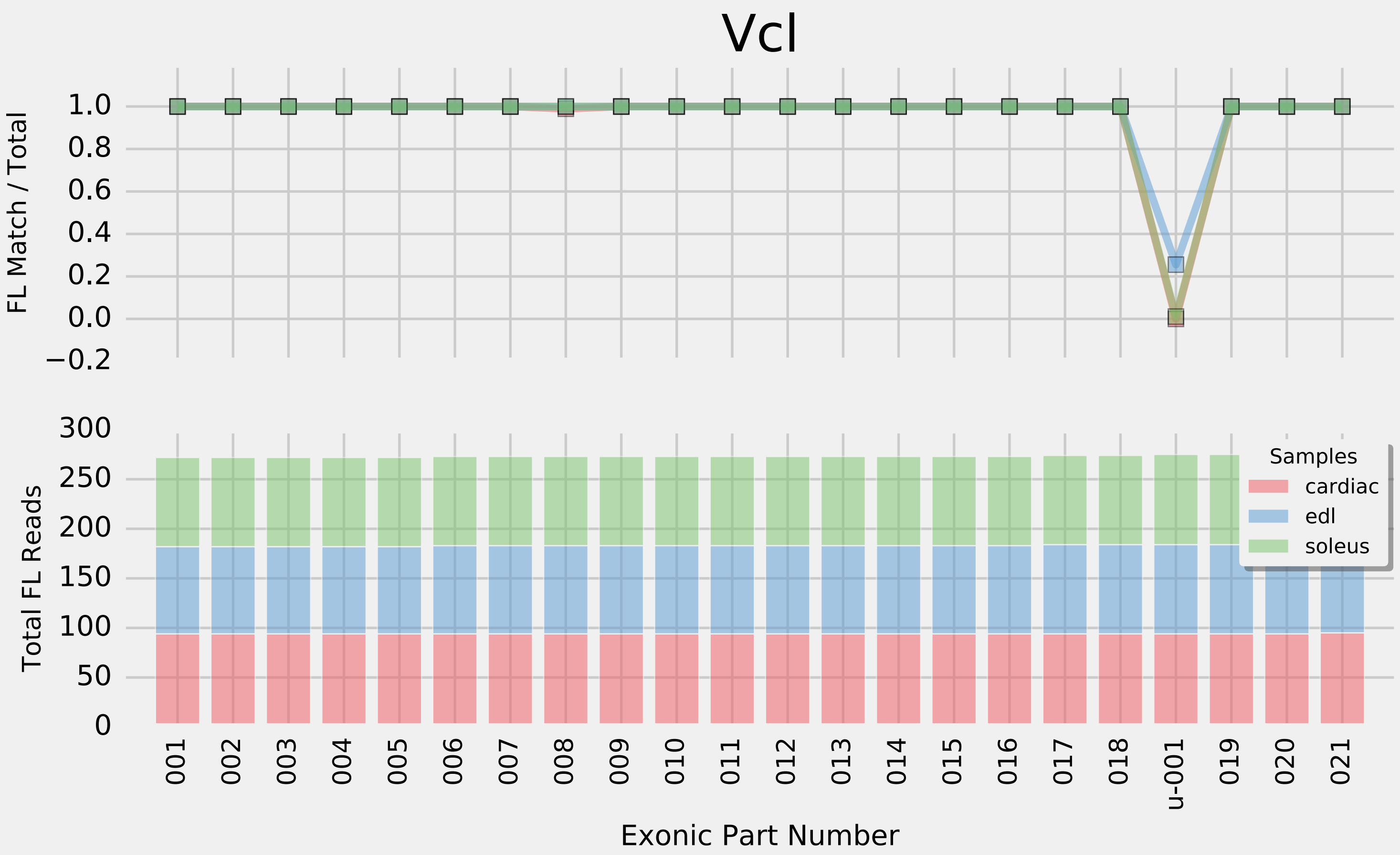

Vcl
FL Match / Total
1.0
0.8
0.6
0.4
0.2
0.0
−0.2
Total FL Reads
300
250
200
150
100
50
0
Samples
cardiac
edl
soleus
001
002
003
004
005
006
007
008
009
010
011
012
013
014
015
016
017
018
u-001
019
020
021
Exonic Part Number

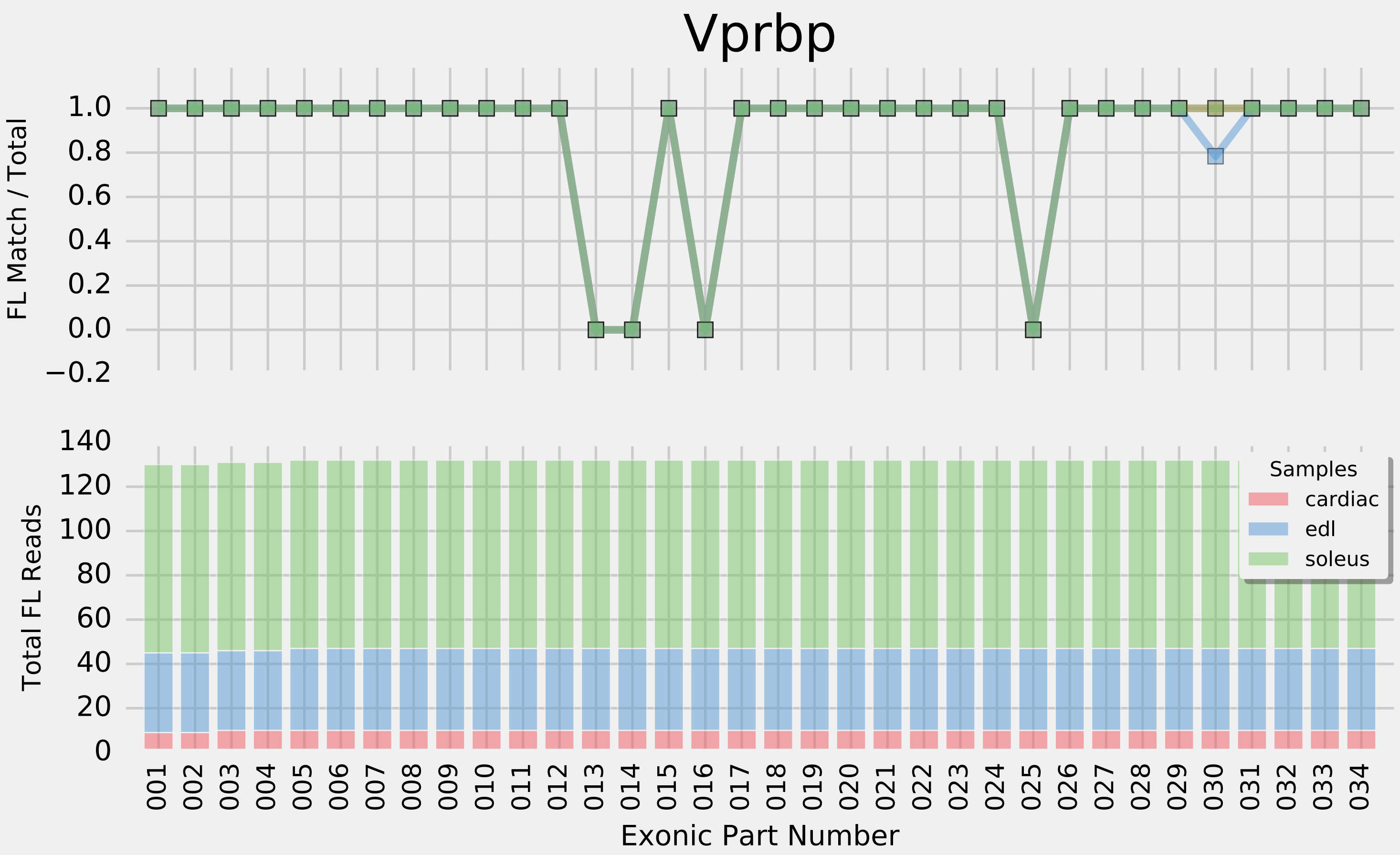

Vprbp
FL Match / Total
1.0
0.8
0.6
0.4
0.2
0.0
−0.2
Total FL Reads
140
120
100
80
60
40
20
0
Samples
cardiac
edl
soleus
001 002 003 004 005 006 007 008 009 010 011 012 013 014 015 016 017 018 019 020 021 022 023 024 025 026 027 028 029 030 031 032 033 034
Exonic Part Number

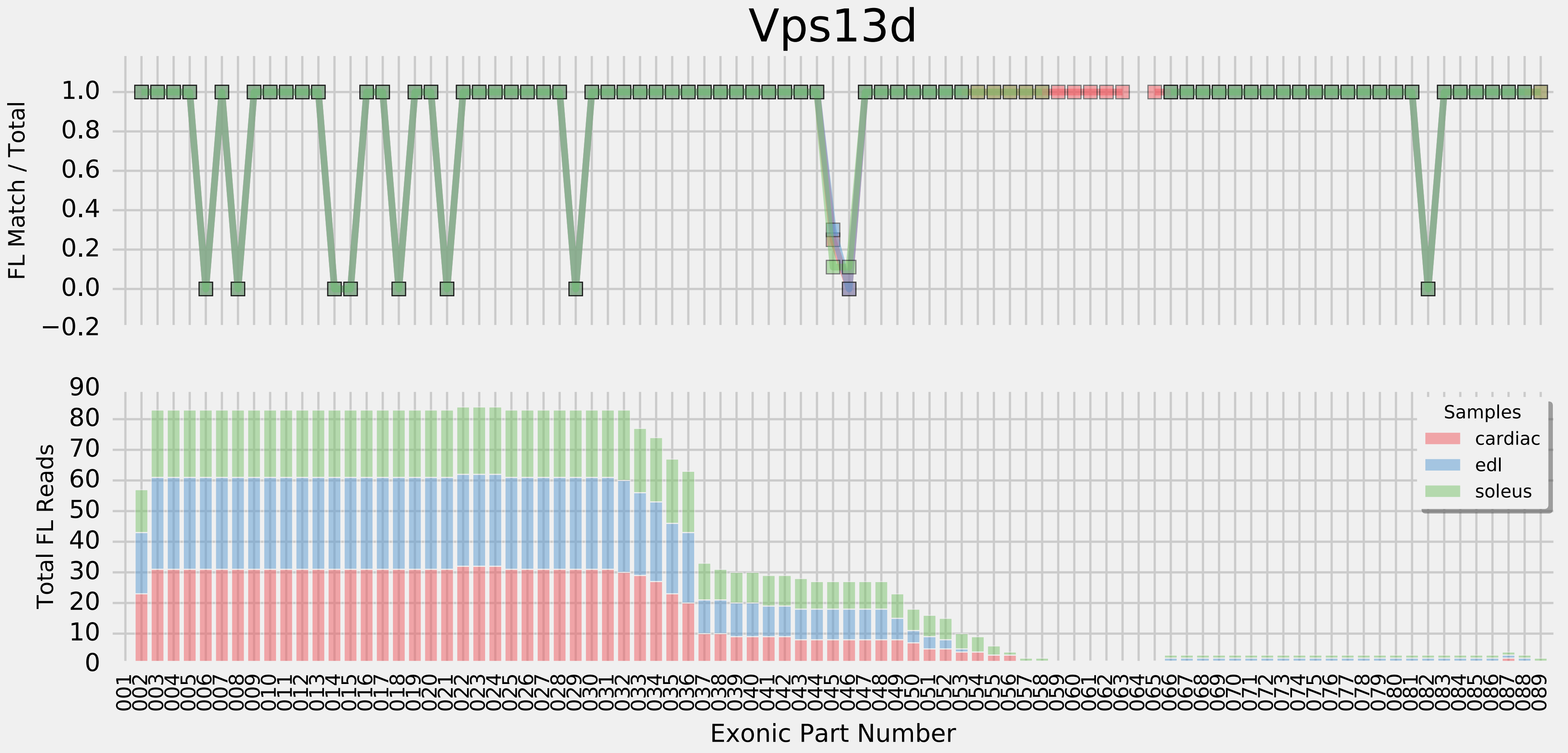

Vps13d
FL Match / Total
Total FL Reads
Exonic Part Number
Samples
cardiac
edl
soleus

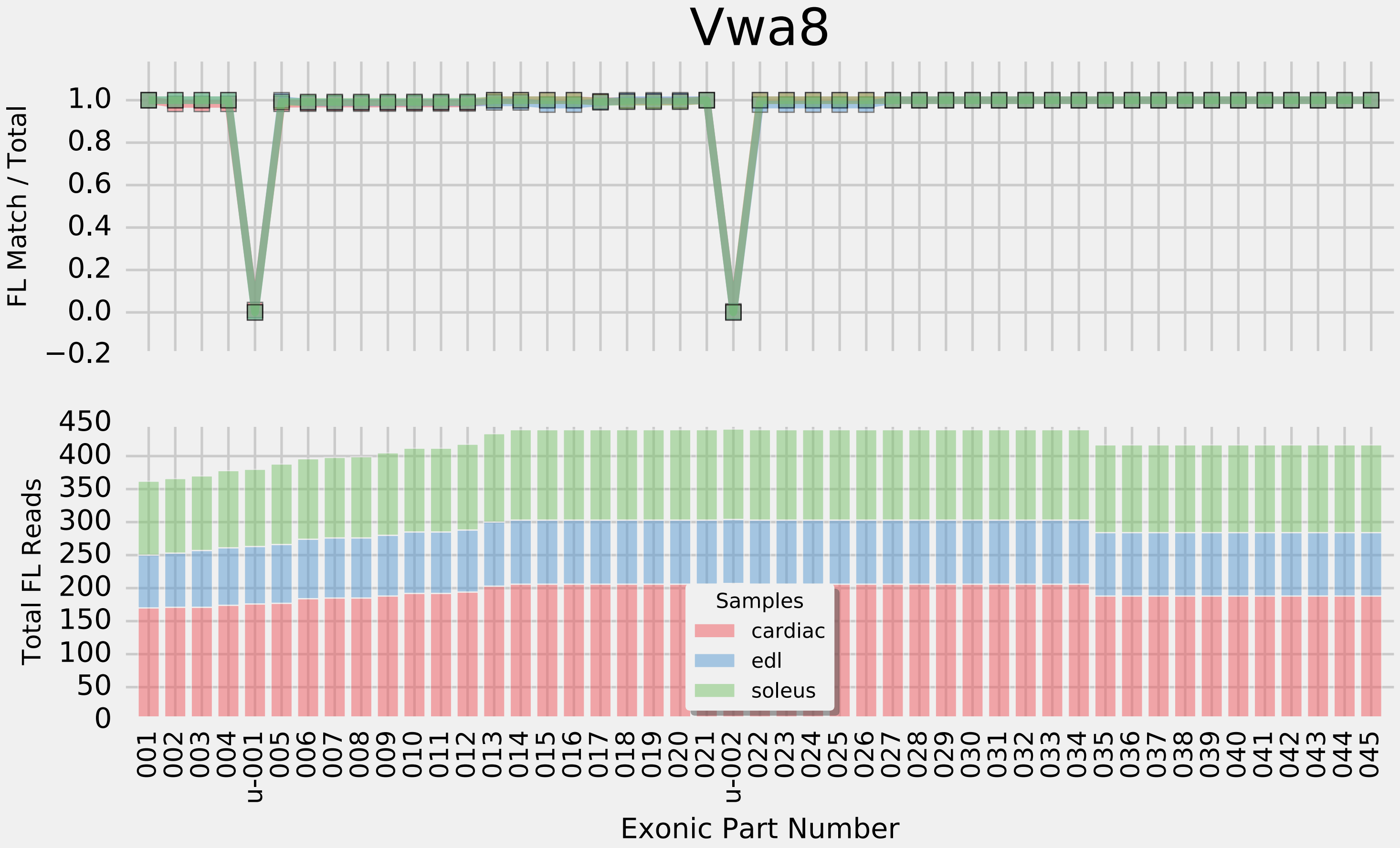

Vwa8
FL Match / Total
1.0
0.8
0.6
0.4
0.2
0.0
−0.2
Total FL Reads
450
400
350
300
250
200
150
100
50
0
Samples
cardiac
edl
soleus
001 002 003 004 u-001 005 006 007 008 009 010 011 012 013 014 015 016 017 018 019 020 021 u-002 022 023 024 025 026 027 028 029 030 031 032 033 034 035 036 037 038 039 040 041 042 043 044 045
Exonic Part Number

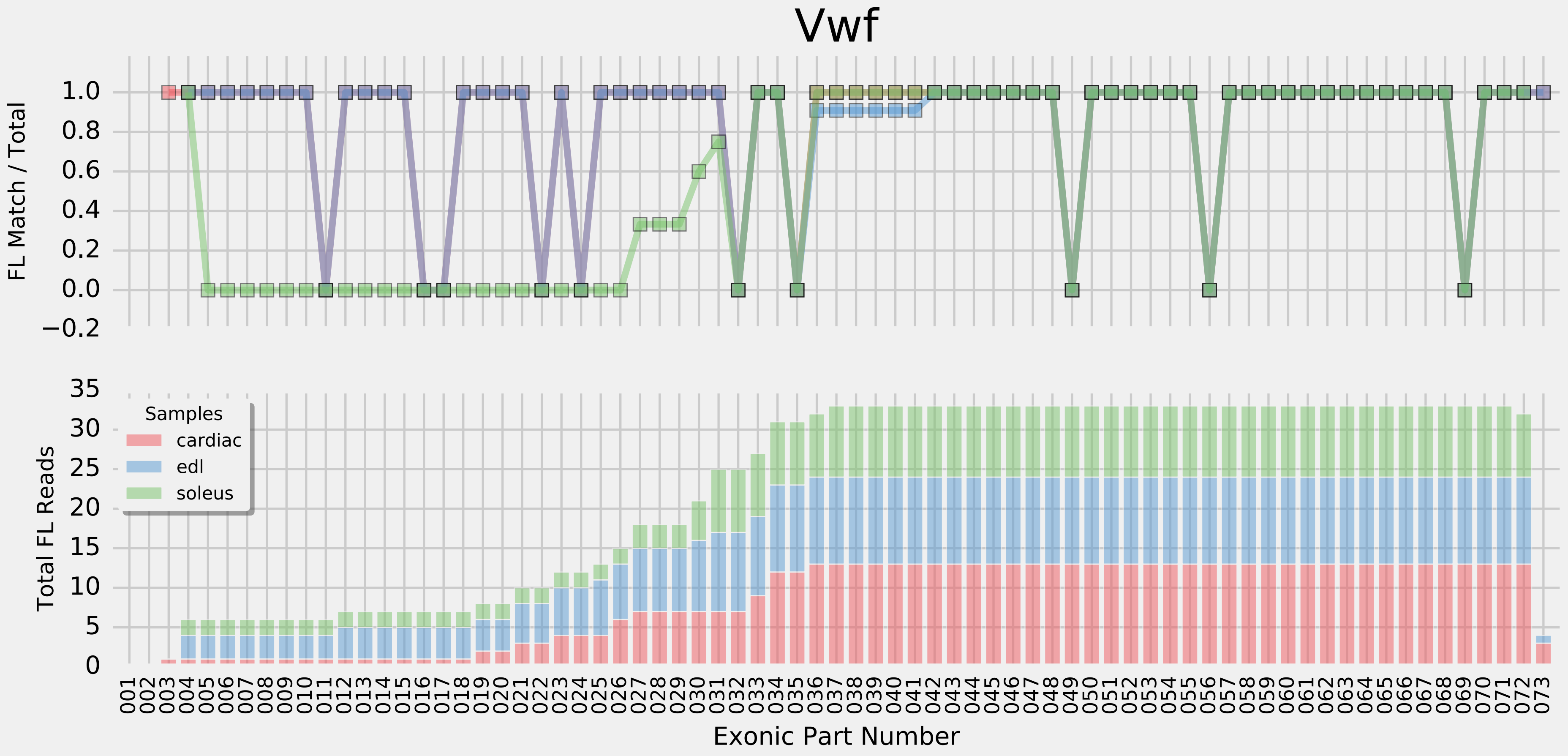

Vwf
FL Match / Total
1.0
0.8
0.6
0.4
0.2
0.0
−0.2
Total FL Reads
35
30
25
20
15
10
5
0
Samples
cardiac
edl
soleus
001 002 003 004 005 006 007 008 009 010 011 012 013 014 015 016 017 018 019 020 021 022 023 024 025 026 027 028 029 030 031 032 033 034 035 036 037 038 039 040 041 042 043 044 045 046 047 048 049 050 051 052 053 054 055 056 057 058 059 060 061 062 063 064 065 066 067 068 069 070 071 072 073
Exonic Part Number

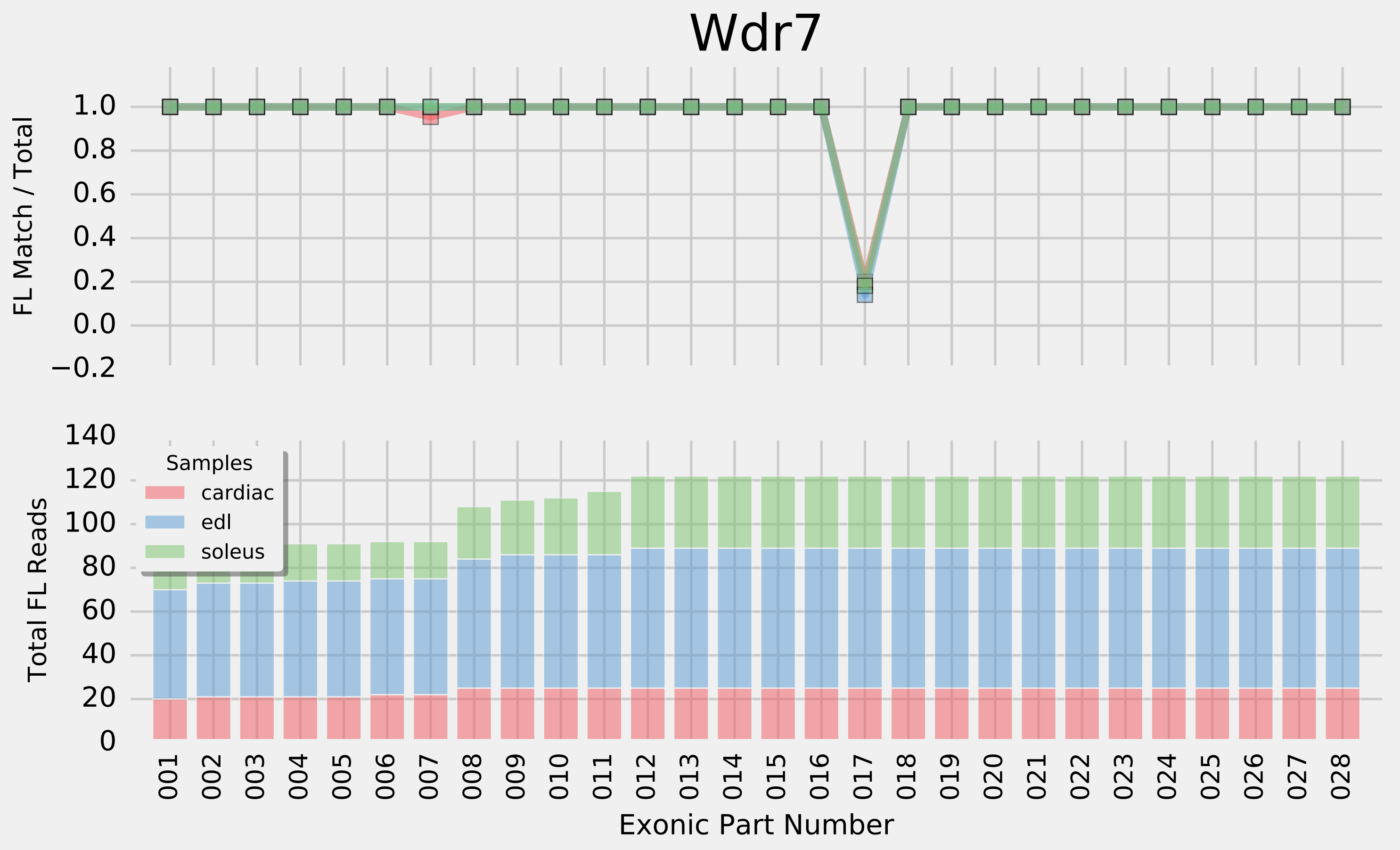

Wdr7
FL Match / Total
1.0
0.8
0.6
0.4
0.2
0.0
−0.2
Total FL Reads
140
120
100
80
60
40
20
0
Samples
cardiac
edl
soleus
001 002 003 004 005 006 007 008 009 010 011 012 013 014 015 016 017 018 019 020 021 022 023 024 025 026 027 028
Exonic Part Number

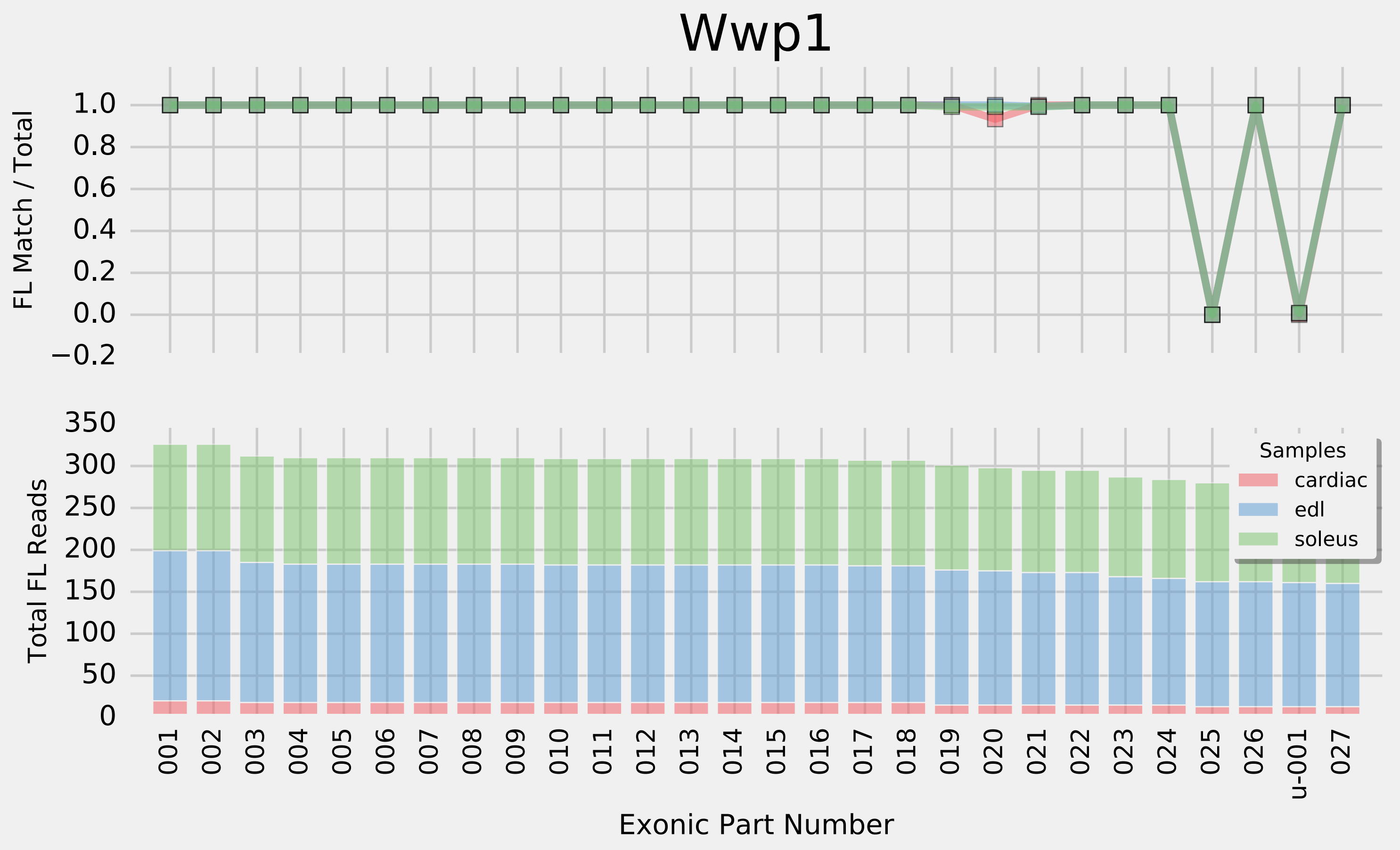

Wwp1
FL Match / Total
1.0
0.8
0.6
0.4
0.2
0.0
−0.2
Total FL Reads
350
300
250
200
150
100
50
0
Samples
cardiac
edl
soleus
001
002
003
004
005
006
007
008
009
010
011
012
013
014
015
016
017
018
019
020
021
022
023
024
025
026
u-001
027
Exonic Part Number

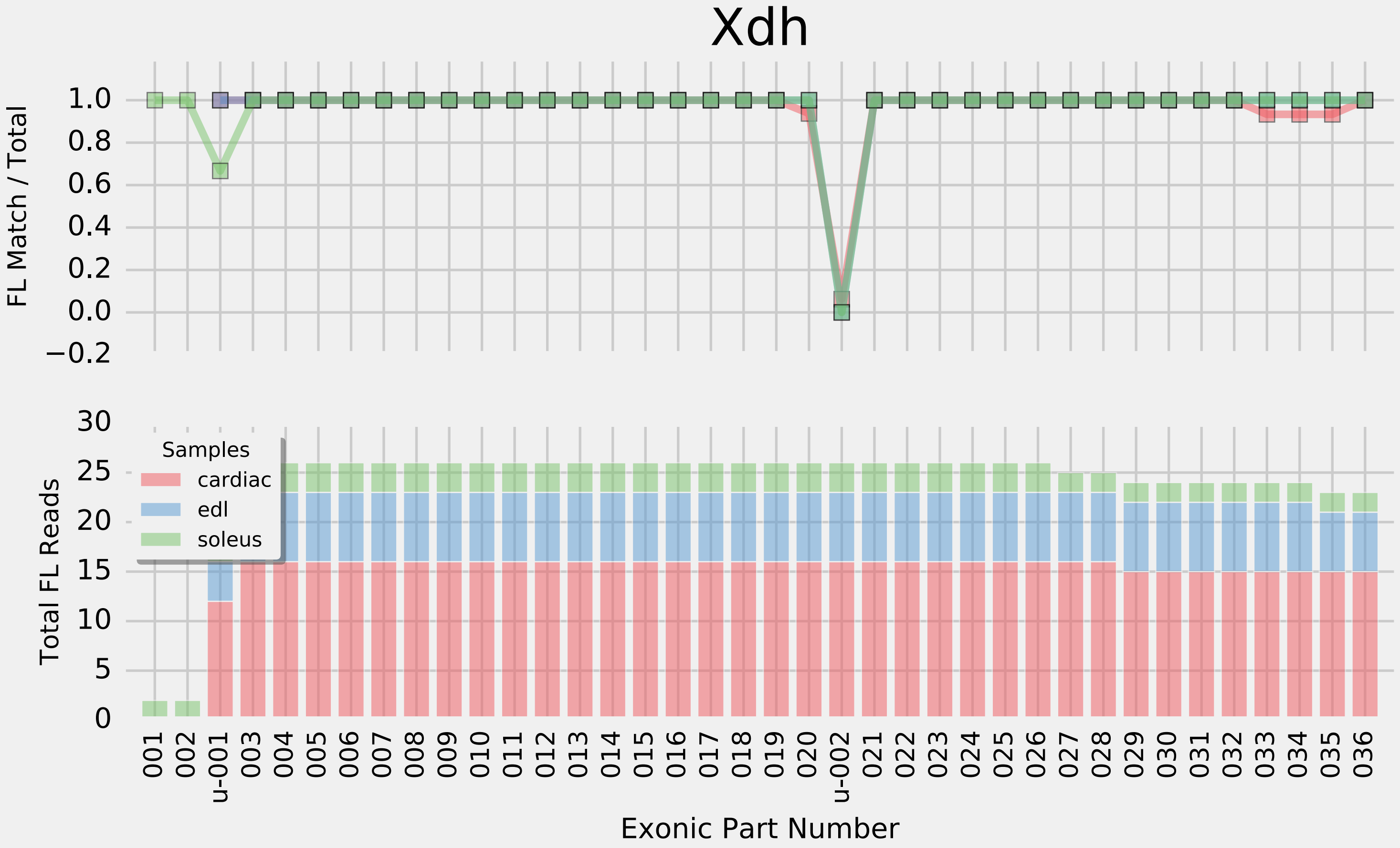

Xdh
FL Match / Total
1.0
0.8
0.6
0.4
0.2
0.0
−0.2
Total FL Reads
30
25
20
15
10
5
0
Samples
cardiac
edl
soleus
001
002
u-001
003
004
005
006
007
008
009
010
011
012
013
014
015
016
017
018
019
020
u-002
021
022
023
024
025
026
027
028
029
030
031
032
033
034
035
036
Exonic Part Number

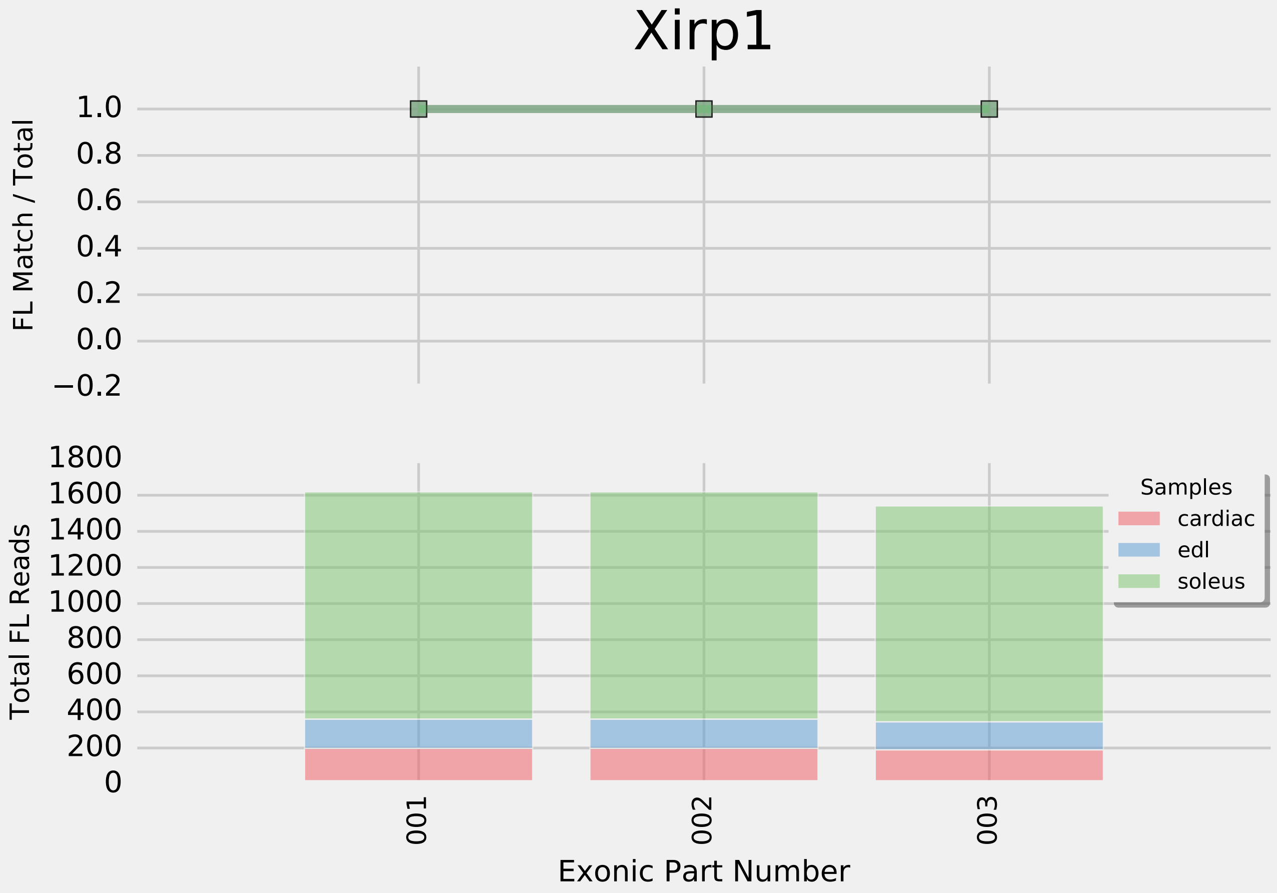
Xirp1
FL Match / Total
1.0
0.8
0.6
0.4
0.2
0.0
−0.2
Total FL Reads
1800
1600
1400
1200
1000
800
600
400
200
0
Samples
cardiac
edl
soleus
001
002
003
Exonic Part Number

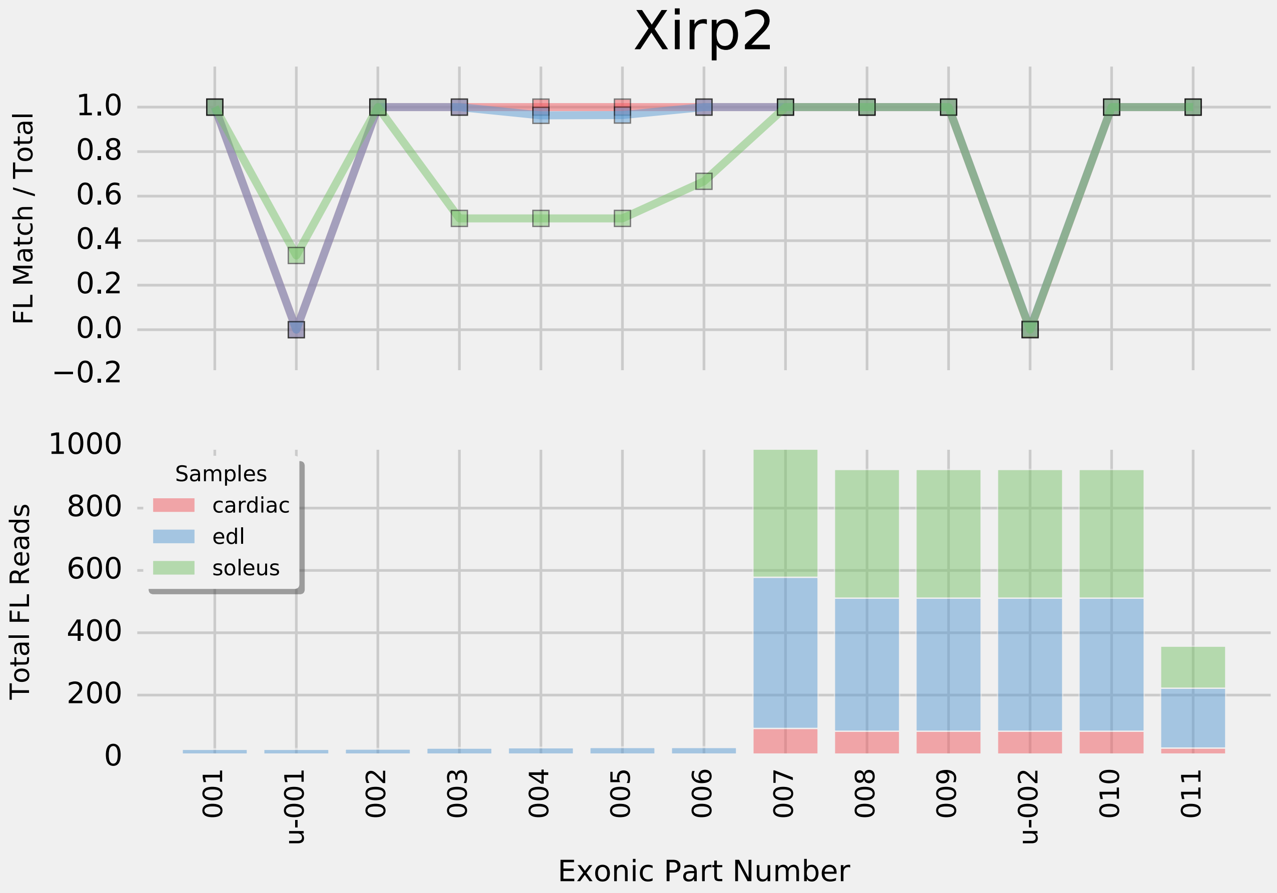

Xirp2
FL Match / Total
1.0
0.8
0.6
0.4
0.2
0.0
−0.2
Total FL Reads
1000
800
600
400
200
0
Samples
cardiac
edl
soleus
001
u-001
002
003
004
005
006
007
008
009
u-002
010
011
Exonic Part Number

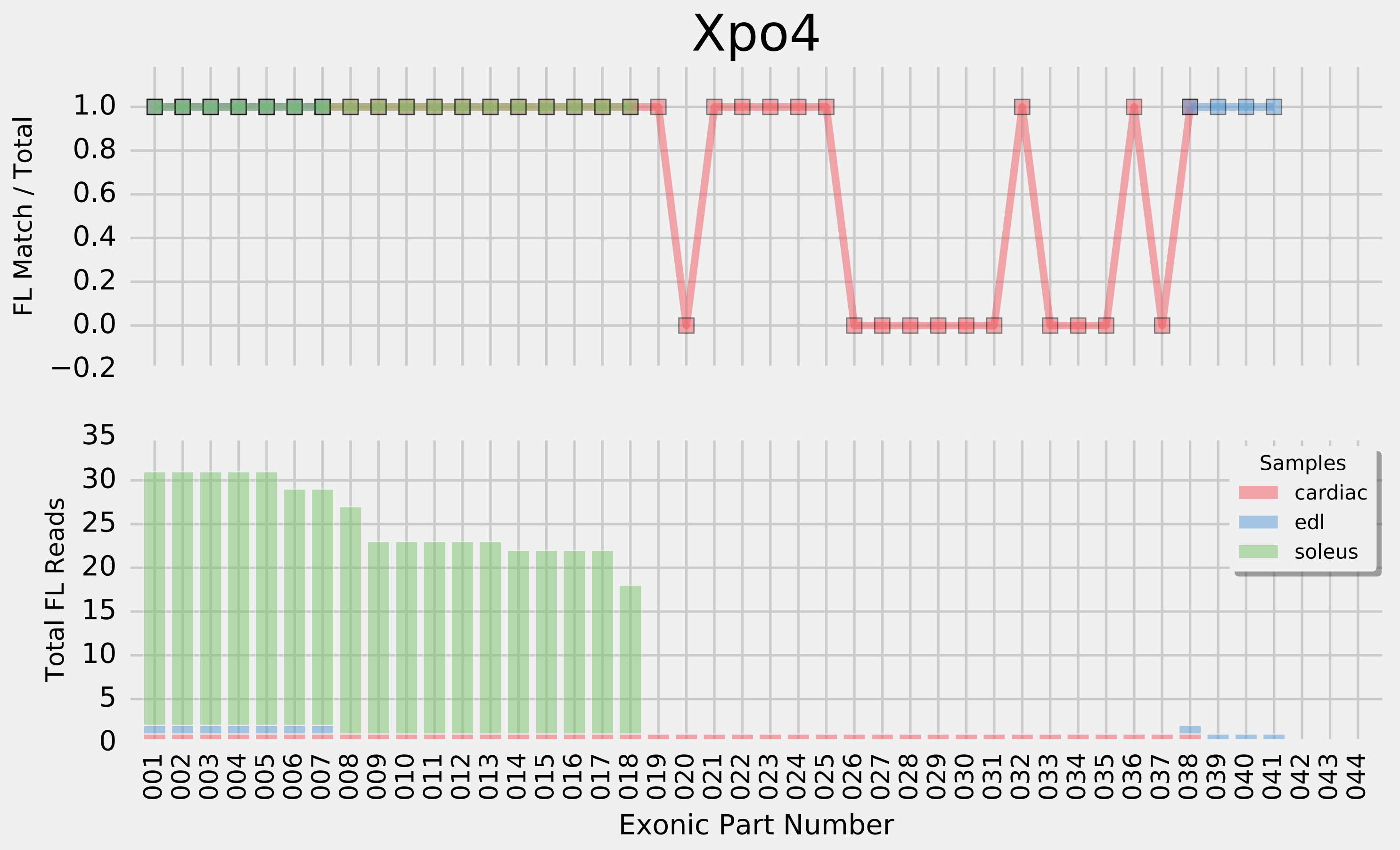

Xpo4
FL Match / Total
Total FL Reads
Exonic Part Number
Samples
cardiac
edl
soleus

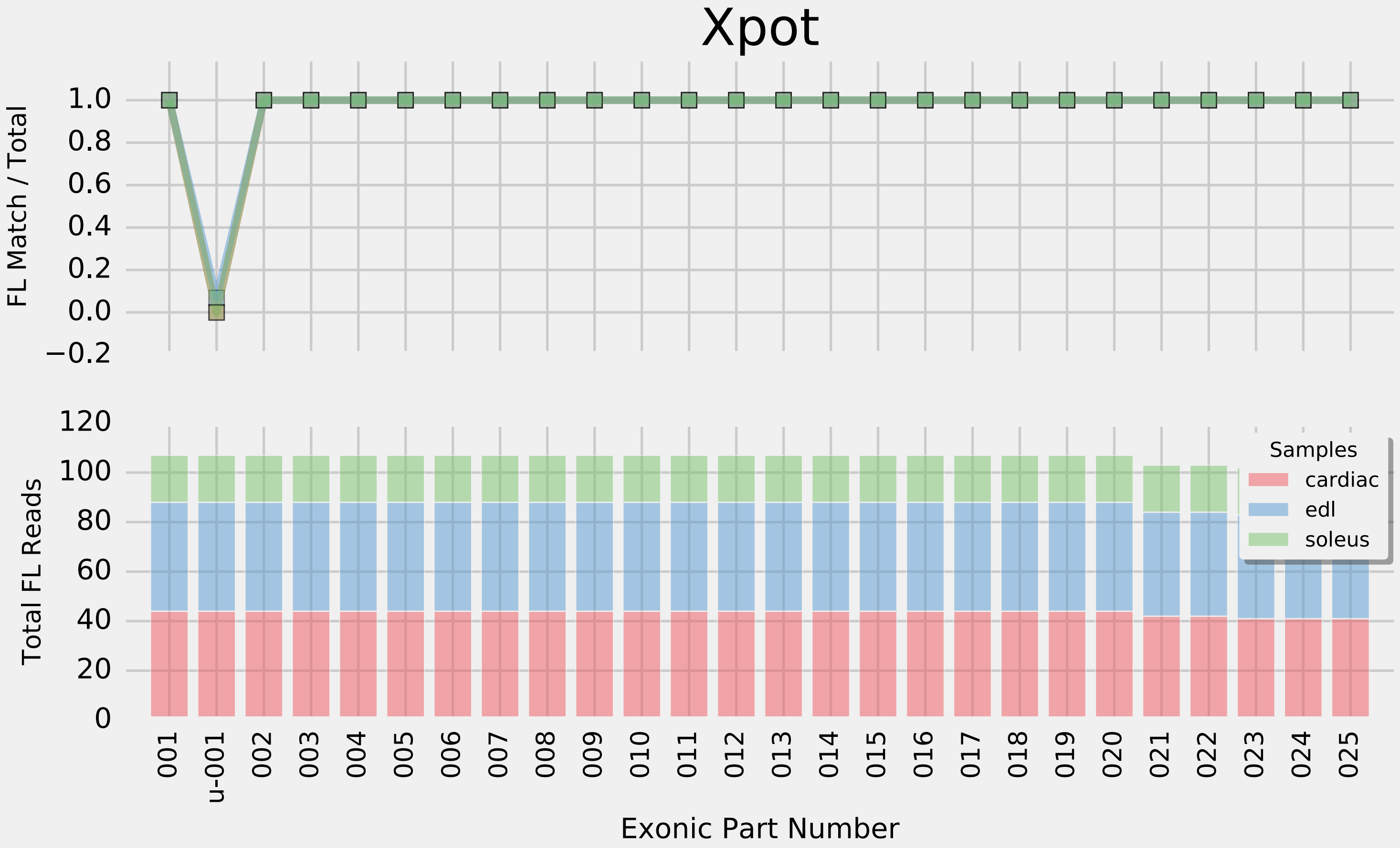

Xpot
FL Match / Total
1.0
0.8
0.6
0.4
0.2
0.0
−0.2
Total FL Reads
120
100
80
60
40
20
0
Samples
cardiac
edl
soleus
001
u-001
002
003
004
005
006
007
008
009
010
011
012
013
014
015
016
017
018
019
020
021
022
023
024
025
Exonic Part Number

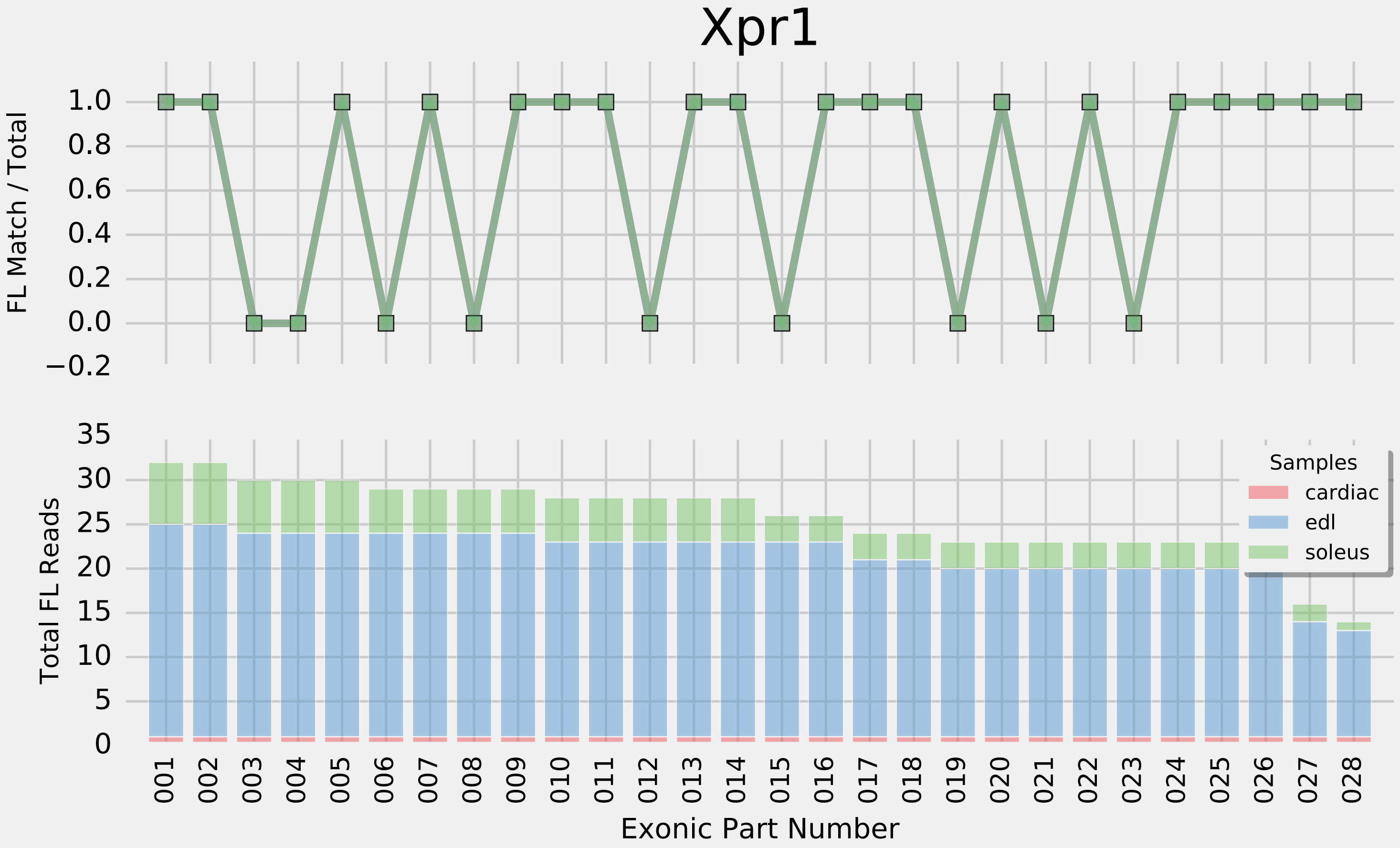

Xpr1
FL Match / Total
1.0
0.8
0.6
0.4
0.2
0.0
−0.2
Total FL Reads
35
30
25
20
15
10
5
0
Samples
cardiac
edl
soleus
001
002
003
004
005
006
007
008
009
010
011
012
013
014
015
016
017
018
019
020
021
022
023
024
025
026
027
028
Exonic Part Number

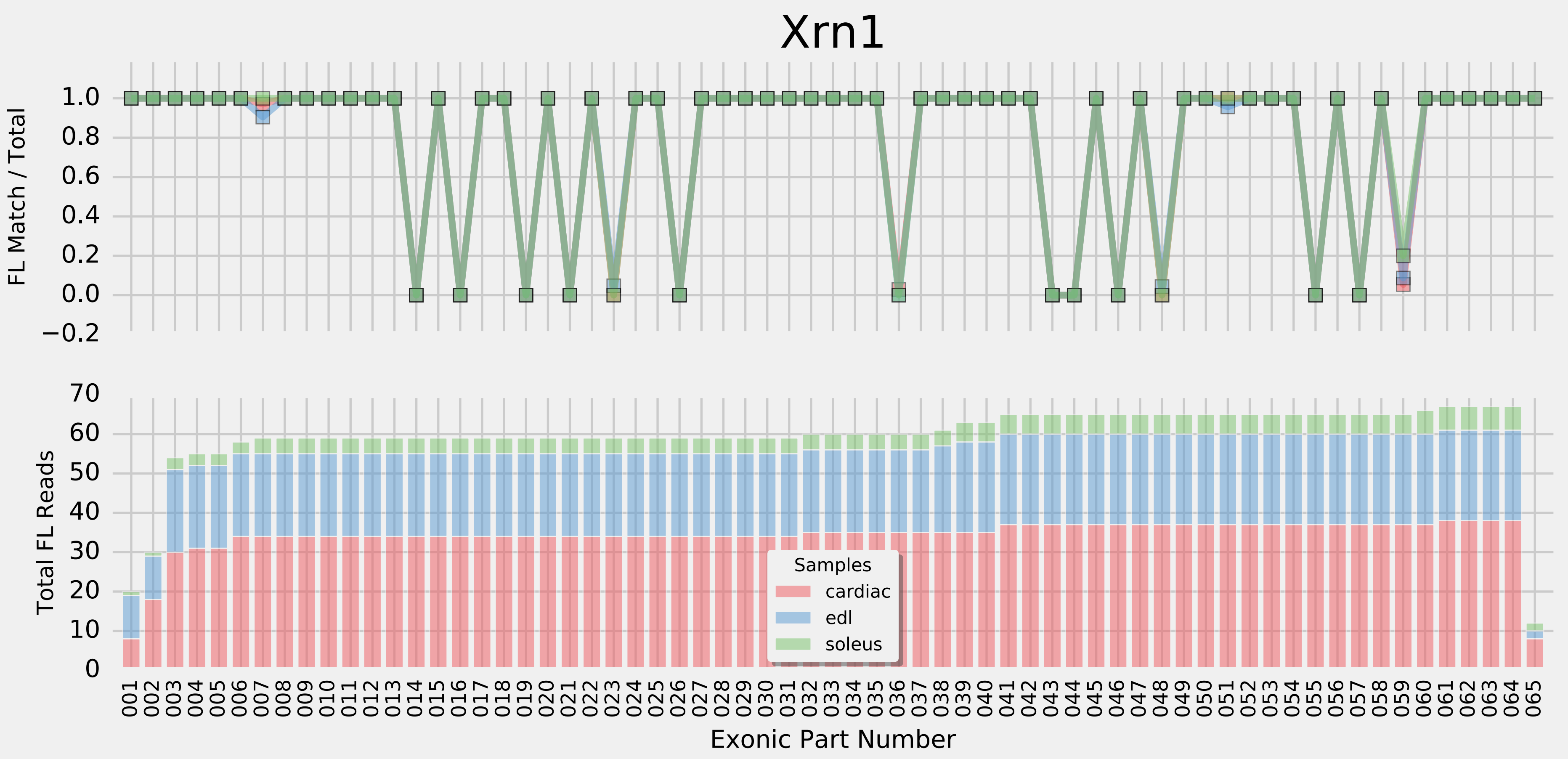

Xrn1
FL Match / Total
Total FL Reads
Samples
cardiac
edl
soleus
Exonic Part Number

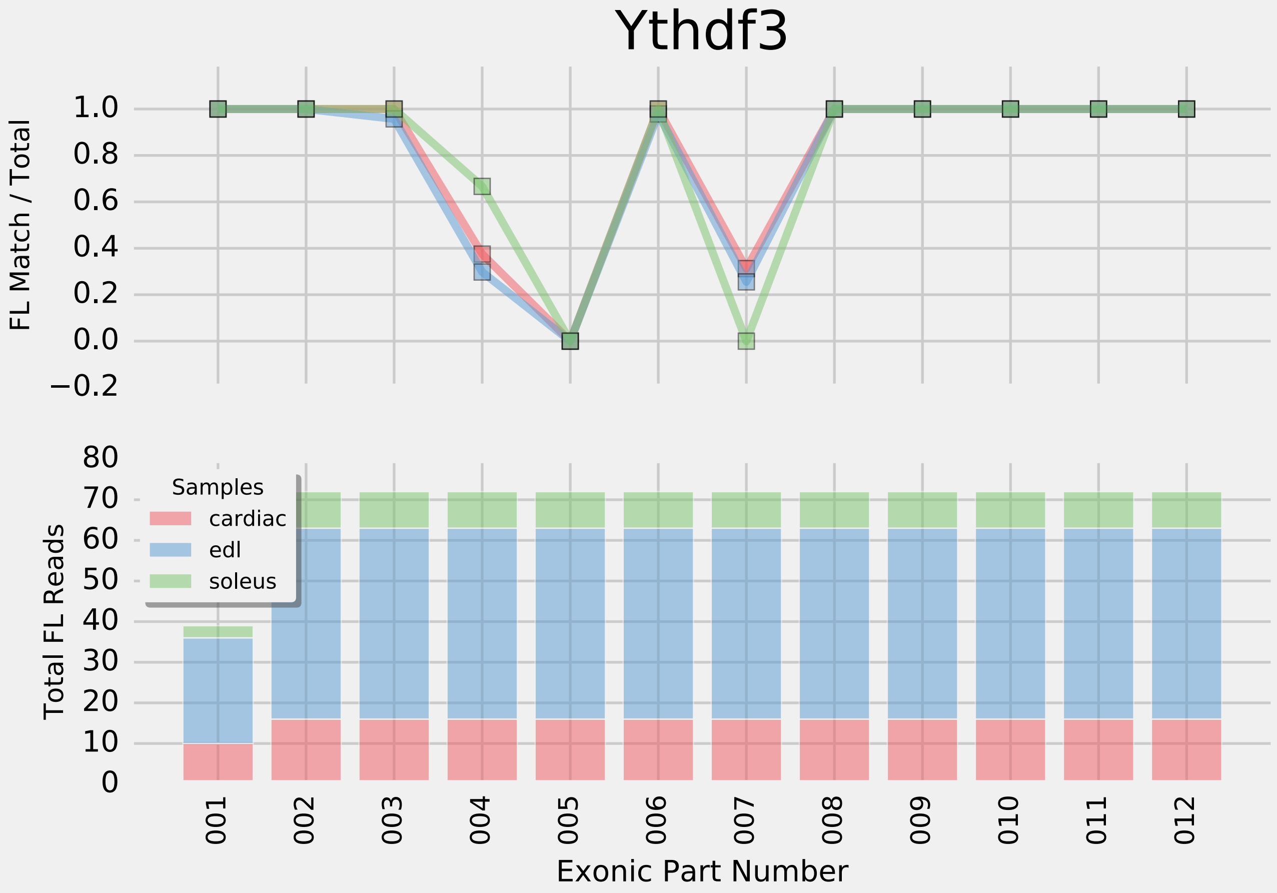

Ythdf3
FL Match / Total
1.0
0.8
0.6
0.4
0.2
0.0
−0.2
80
70
60
50
40
30
20
10
0
Total FL Reads
Samples
cardiac
edl
soleus
001
002
003
004
005
006
007
008
009
010
011
012
Exonic Part Number

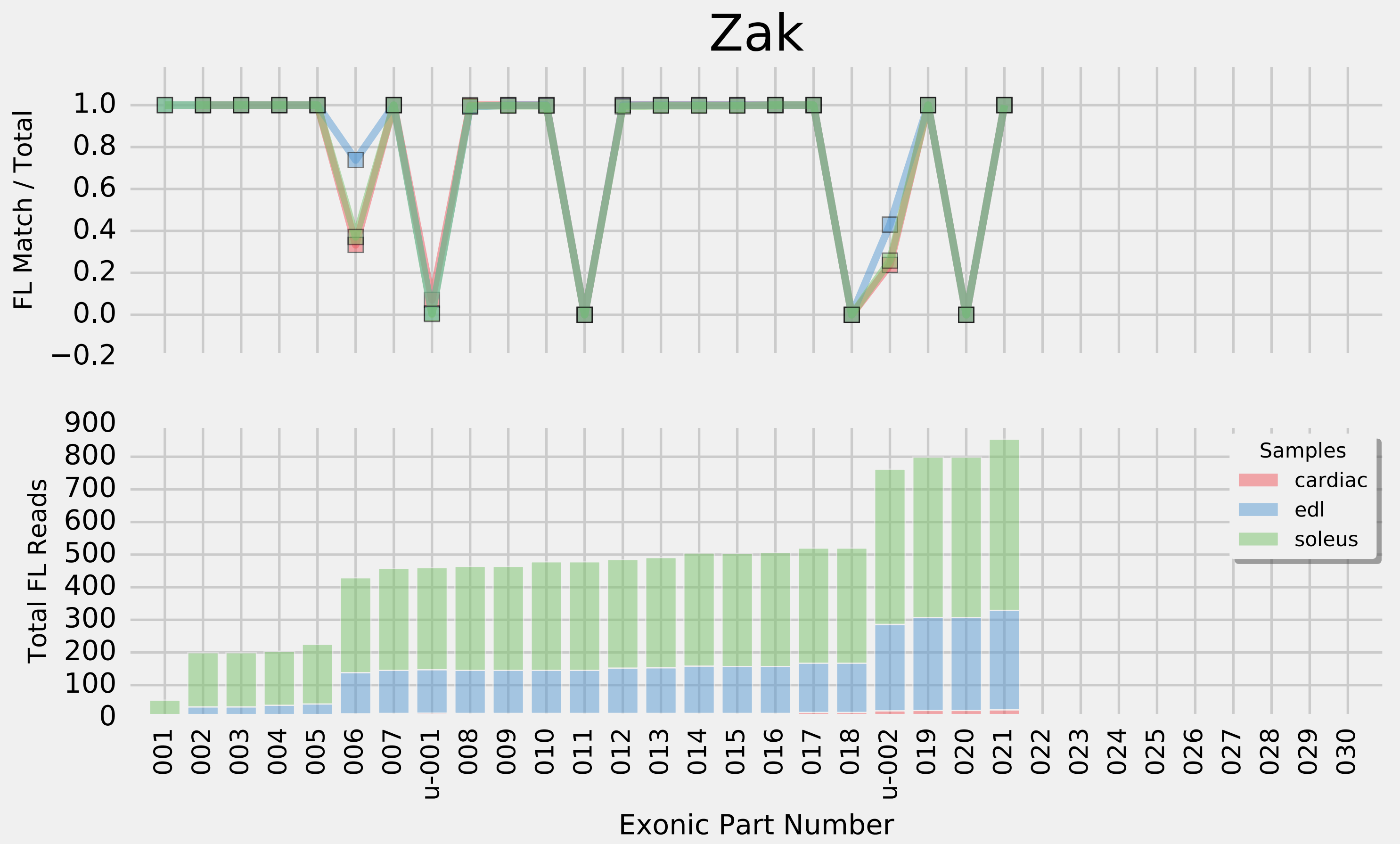
Zak
FL Match / Total
1.0
0.8
0.6
0.4
0.2
0.0
−0.2
Total FL Reads
900
800
700
600
500
400
300
200
100
0
Samples
cardiac
edl
soleus
001
002
003
004
005
006
007
u-001
008
009
010
011
012
013
014
015
016
017
018
u-002
019
020
021
022
023
024
025
026
027
028
029
030
Exonic Part Number

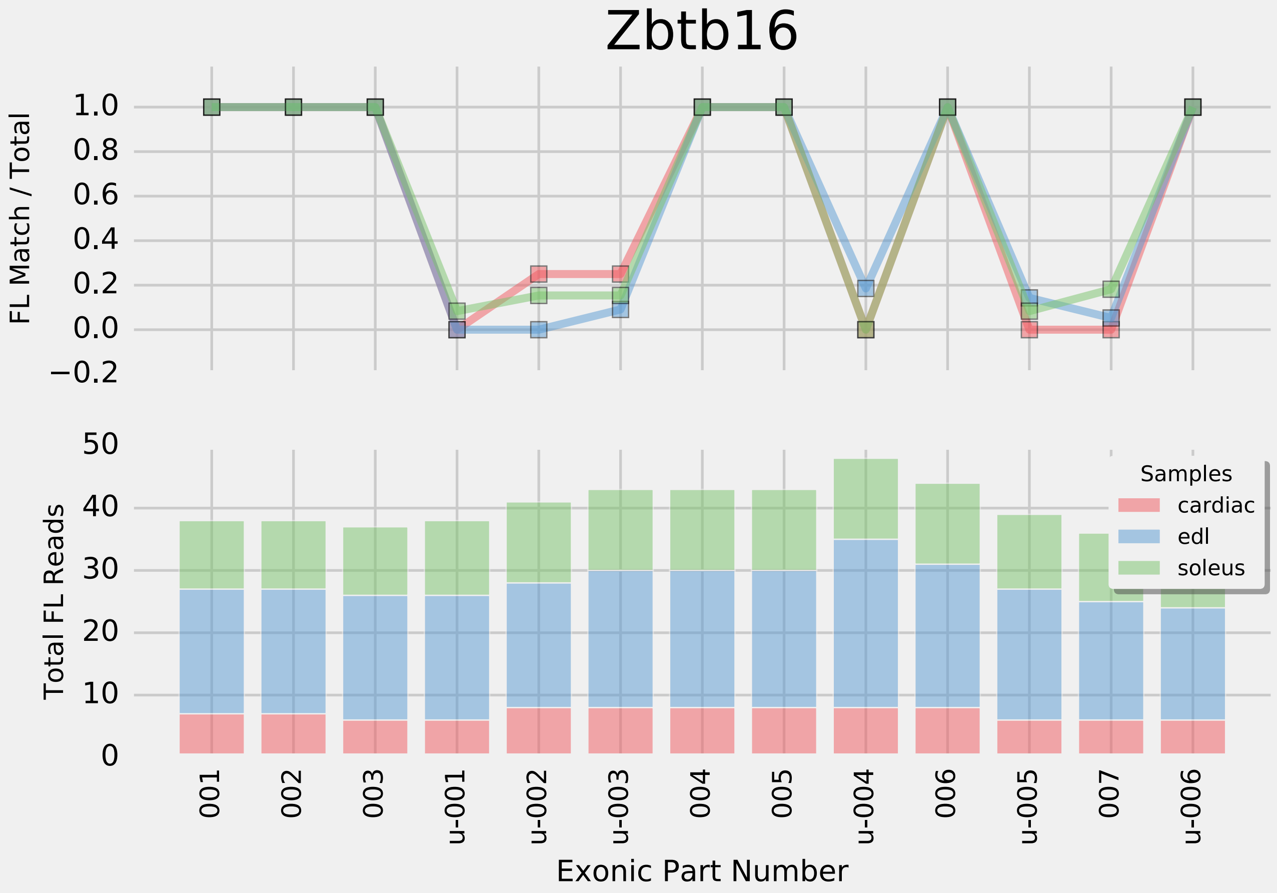
Zbtb16
FL Match / Total
1.0
0.8
0.6
0.4
0.2
0.0
−0.2
Total FL Reads
50
40
30
20
10
0
Samples
cardiac
edl
soleus
001
002
003
u-001
u-002
u-003
004
005
u-004
006
u-005
007
u-006
Exonic Part Number

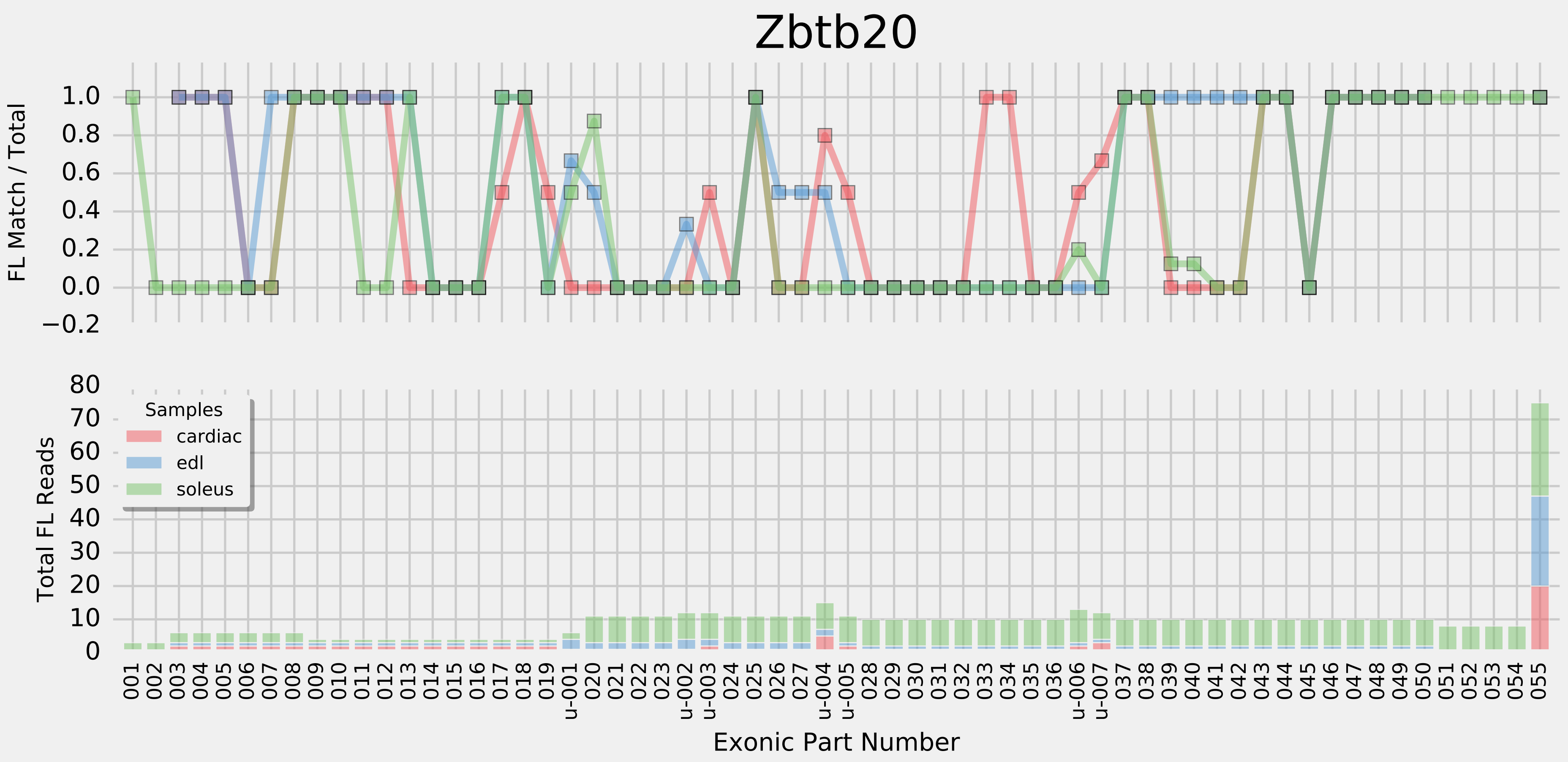

Zbtb20
FL Match / Total
1.0
0.8
0.6
0.4
0.2
0.0
−0.2
Total FL Reads
80
70
60
50
40
30
20
10
0
Samples
cardiac
edl
soleus
001 002 003 004 005 006 007 008 009 010 011 012 013 014 015 016 017 018 019 u-001 020 021 022 023 u-002 u-003 024 025 026 027 u-004 u-005 028 029 030 031 032 033 034 035 036 u-006 u-007 037 038 039 040 041 042 043 044 045 046 047 048 049 050 051 052 053 054 055
Exonic Part Number

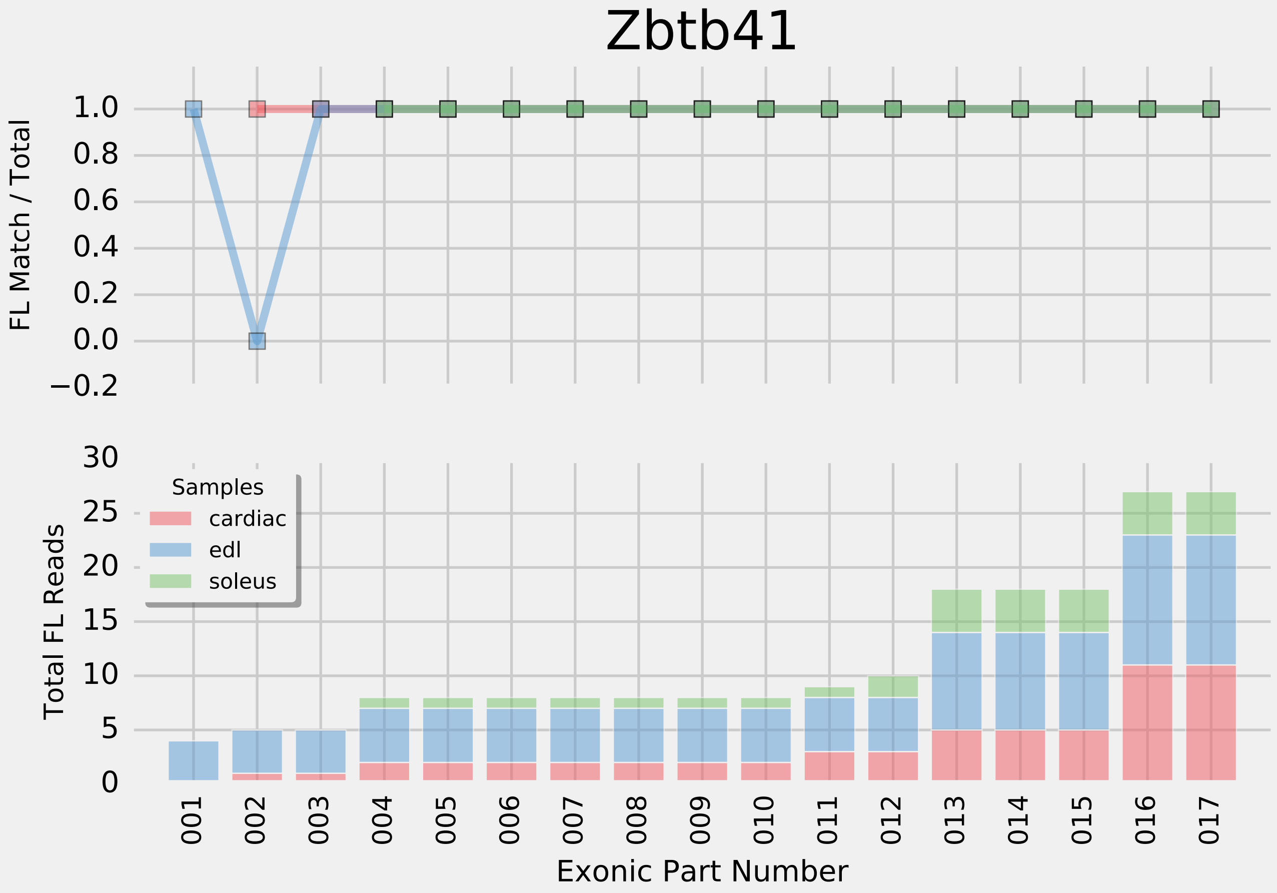

Zbtb41
FL Match / Total
1.0
0.8
0.6
0.4
0.2
0.0
−0.2
Samples
cardiac
edl
soleus
Total FL Reads
30
25
20
15
10
5
0
001
002
003
004
005
006
007
008
009
010
011
012
013
014
015
016
017
Exonic Part Number

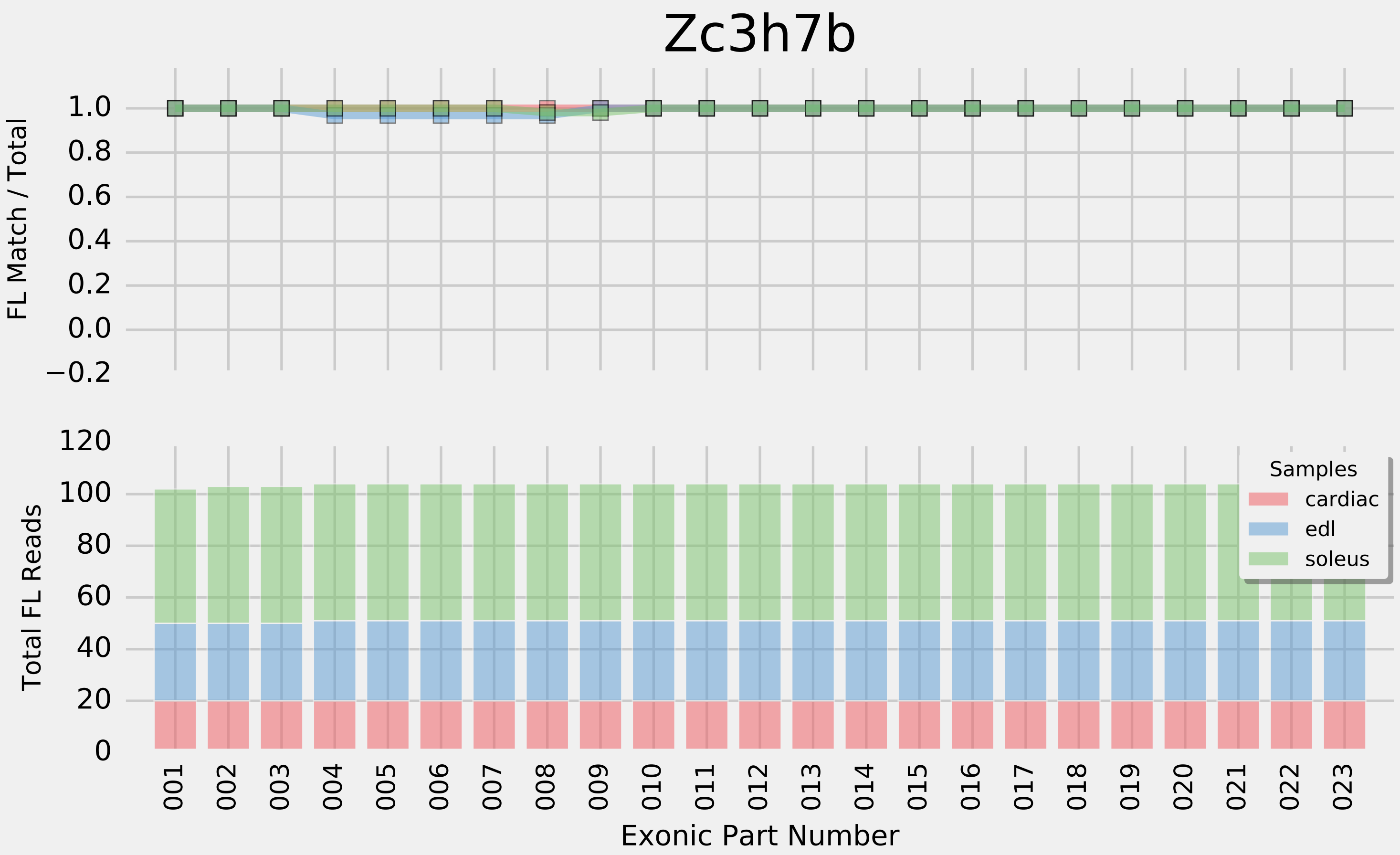

Zc3h7b
FL Match / Total
1.0
0.8
0.6
0.4
0.2
0.0
−0.2
Total FL Reads
120
100
80
60
40
20
0
Samples
cardiac
edl
soleus
001
002
003
004
005
006
007
008
009
010
011
012
013
014
015
016
017
018
019
020
021
022
023
Exonic Part Number

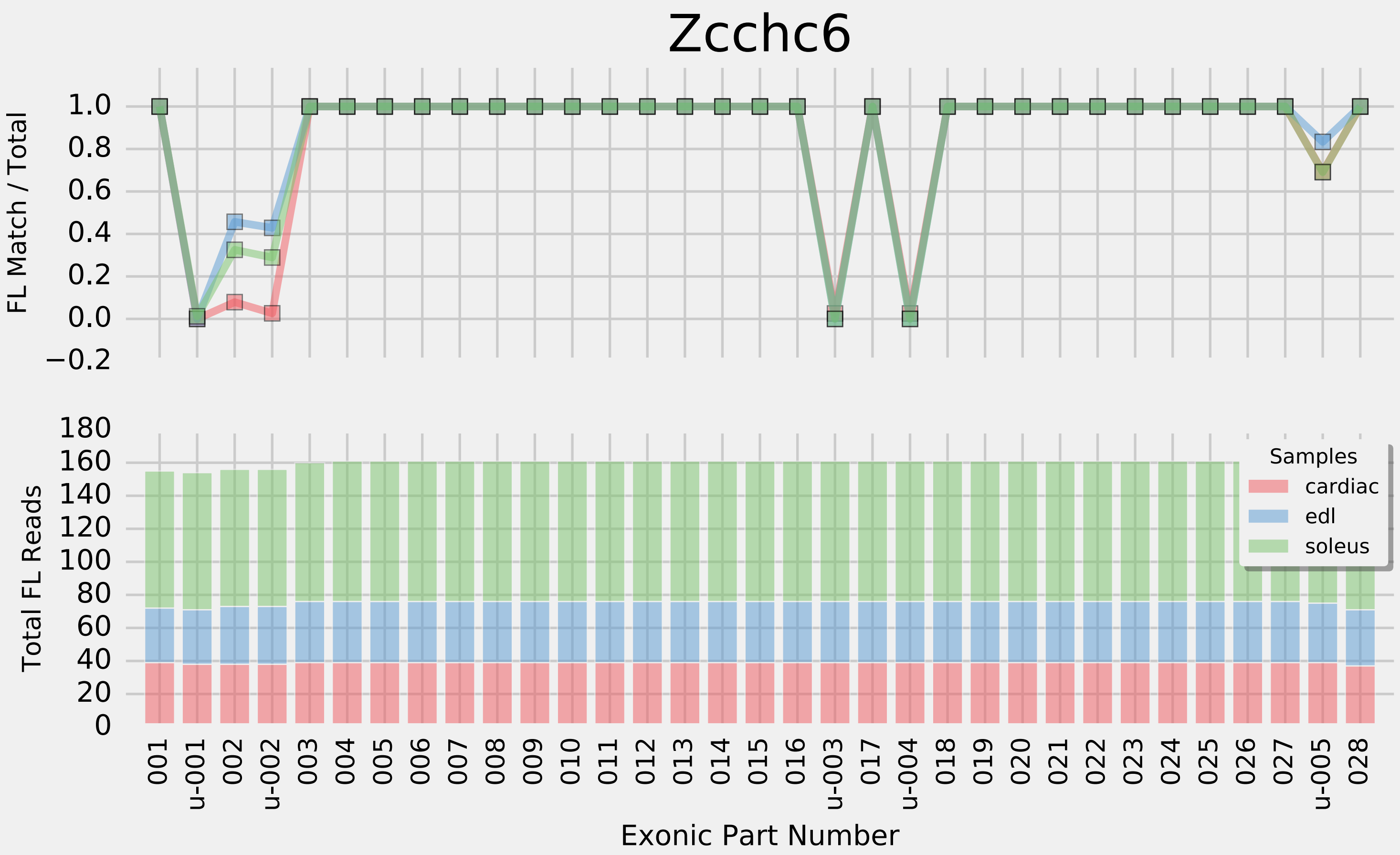
Zcchc6
FL Match / Total
1.0
0.8
0.6
0.4
0.2
0.0
−0.2
Total FL Reads
180
160
140
120
100
80
60
40
20
0
Samples
cardiac
edl
soleus
001
u-001
002
u-002
003
004
005
006
007
008
009
010
011
012
013
014
015
016
u-003
017
u-004
018
019
020
021
022
023
024
025
026
027
u-005
028
Exonic Part Number

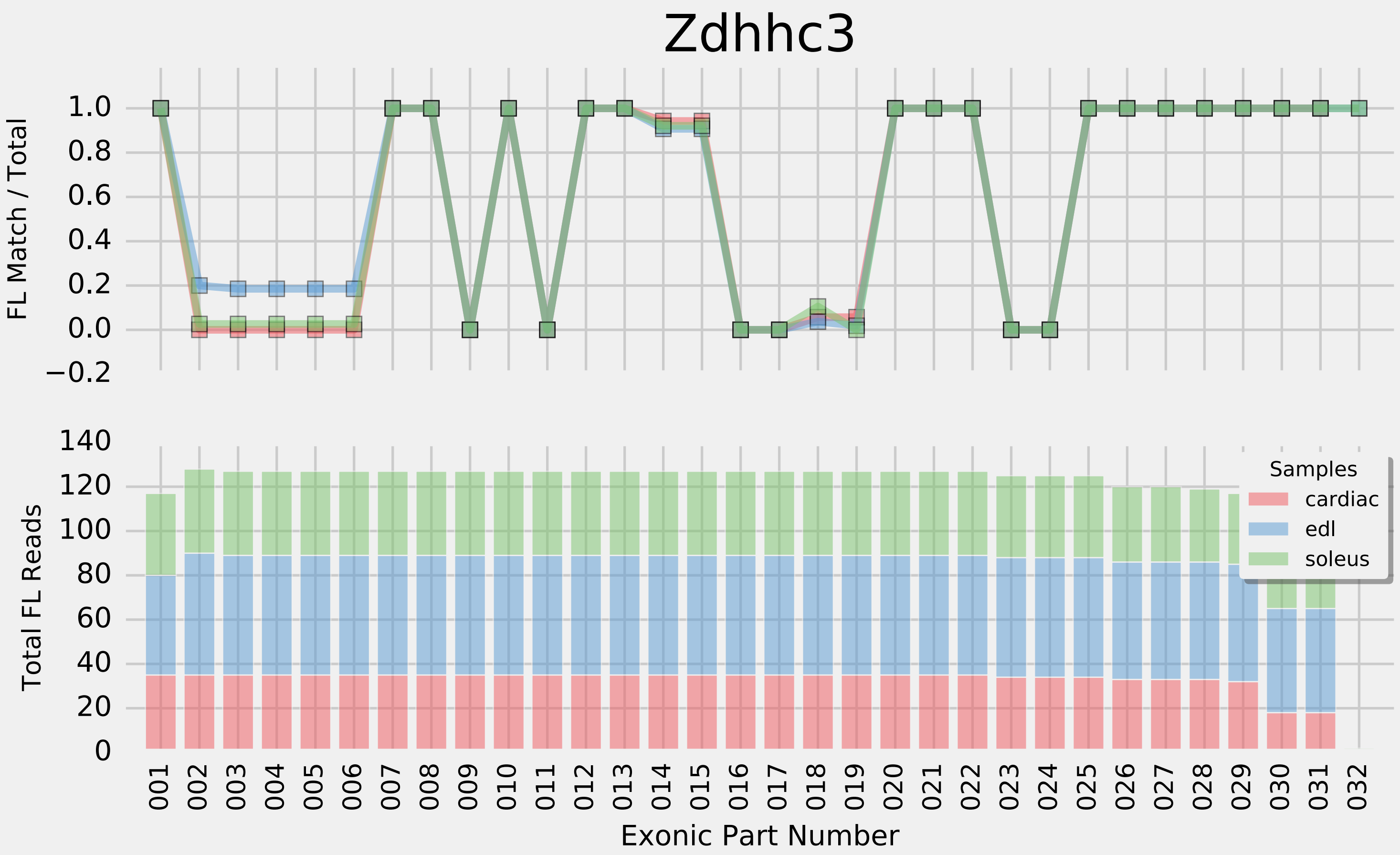

Zdhhc3
FL Match / Total
1.0
0.8
0.6
0.4
0.2
0.0
−0.2
Total FL Reads
140
120
100
80
60
40
20
0
Samples
cardiac
edl
soleus
001
002
003
004
005
006
007
008
009
010
011
012
013
014
015
016
017
018
019
020
021
022
023
024
025
026
027
028
029
030
031
032
Exonic Part Number

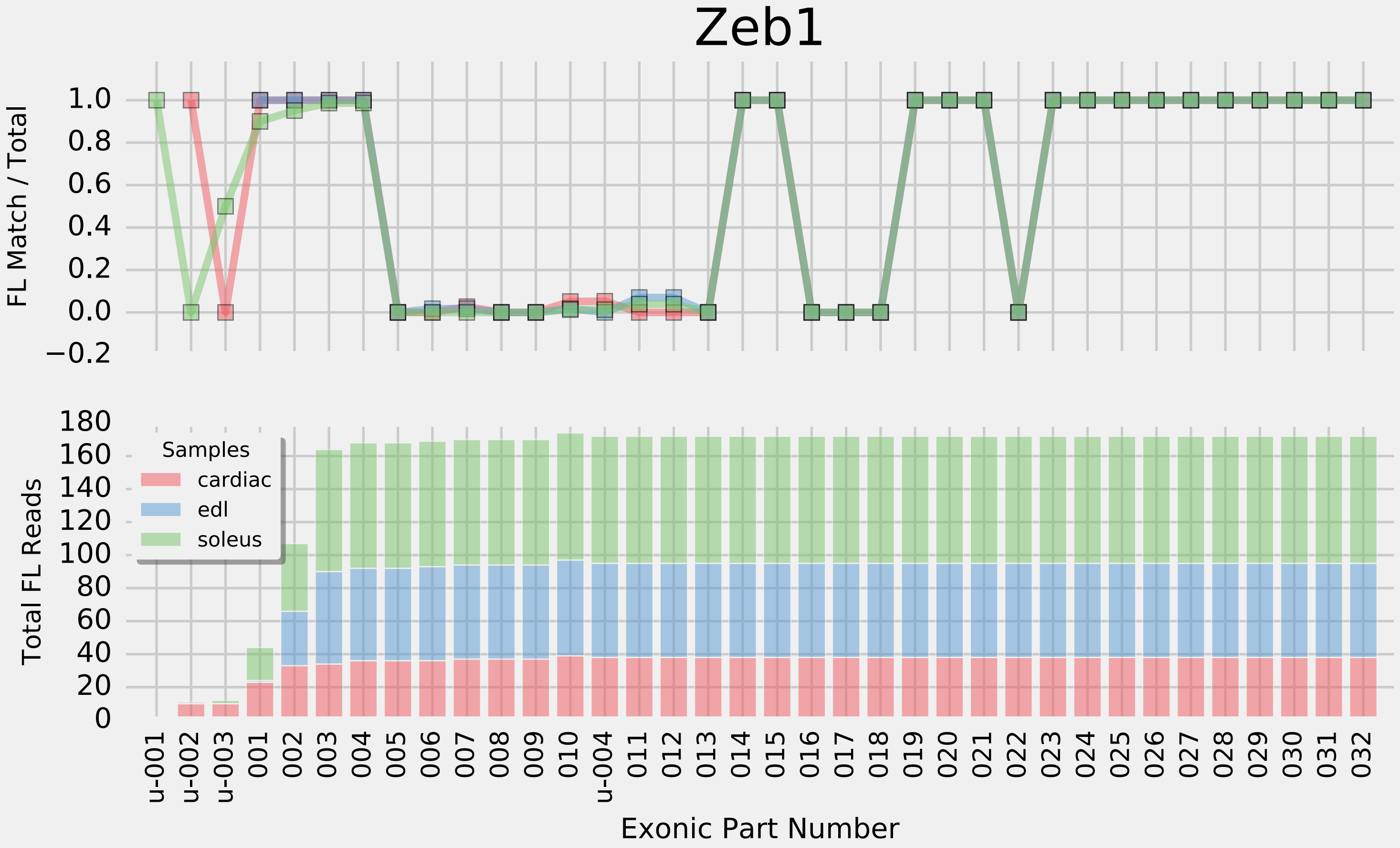

Zeb1
FL Match / Total
1.0
0.8
0.6
0.4
0.2
0.0
−0.2
Total FL Reads
180
160
140
120
100
80
60
40
20
0
Samples
cardiac
edl
soleus
u-001
u-002
u-003
001
002
003
004
005
006
007
008
009
010
u-004
011
012
013
014
015
016
017
018
019
020
021
022
023
024
025
026
027
028
029
030
031
032
Exonic Part Number

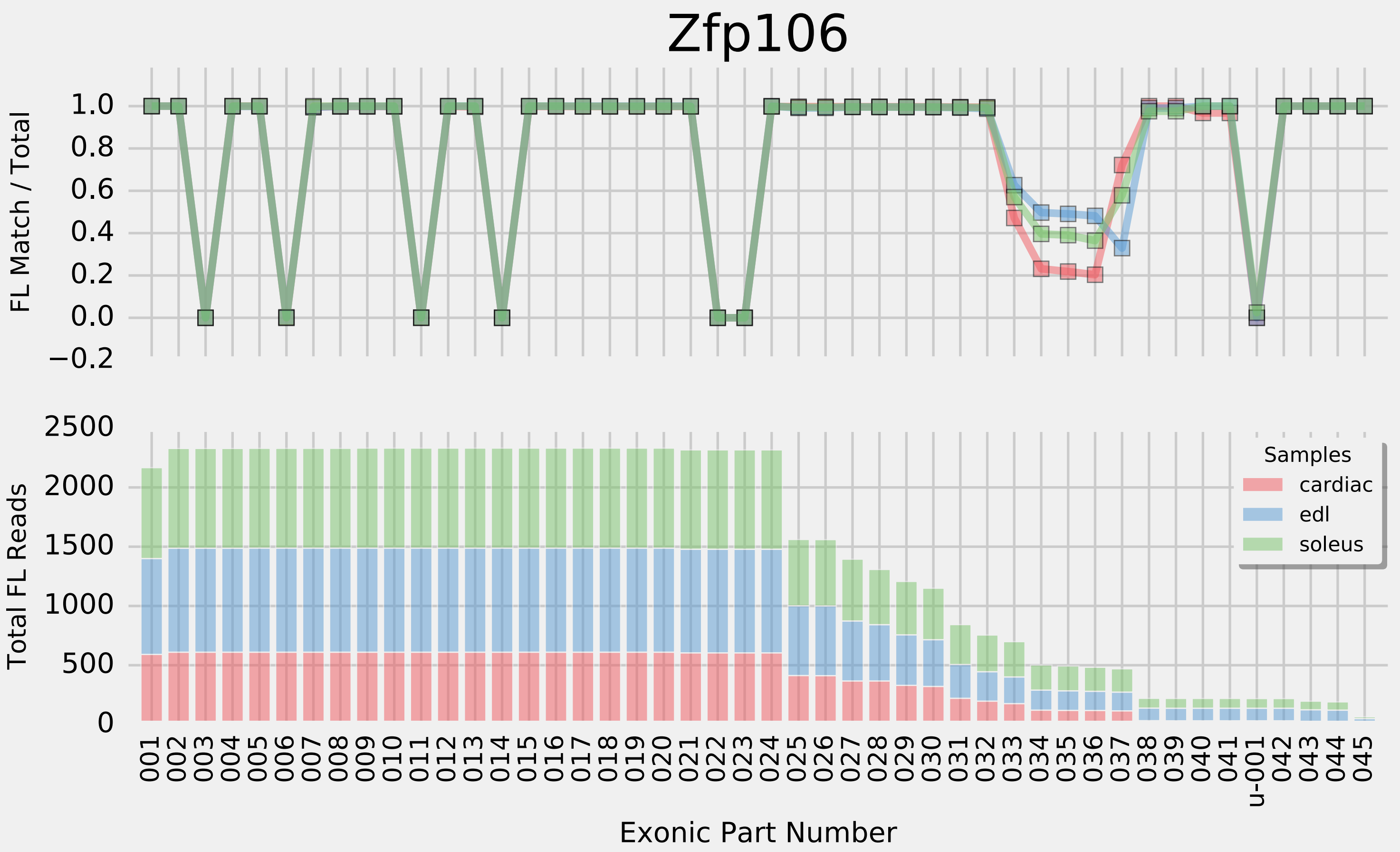
Zfp106
FL Match / Total
1.0
0.8
0.6
0.4
0.2
0.0
−0.2
Total FL Reads
2500
2000
1500
1000
500
0
Samples
cardiac
edl
soleus
001 002 003 004 005 006 007 008 009 010 011 012 013 014 015 016 017 018 019 020 021 022 023 024 025 026 027 028 029 030 031 032 033 034 035 036 037 038 039 040 041 u-001 042 043 044 045
Exonic Part Number

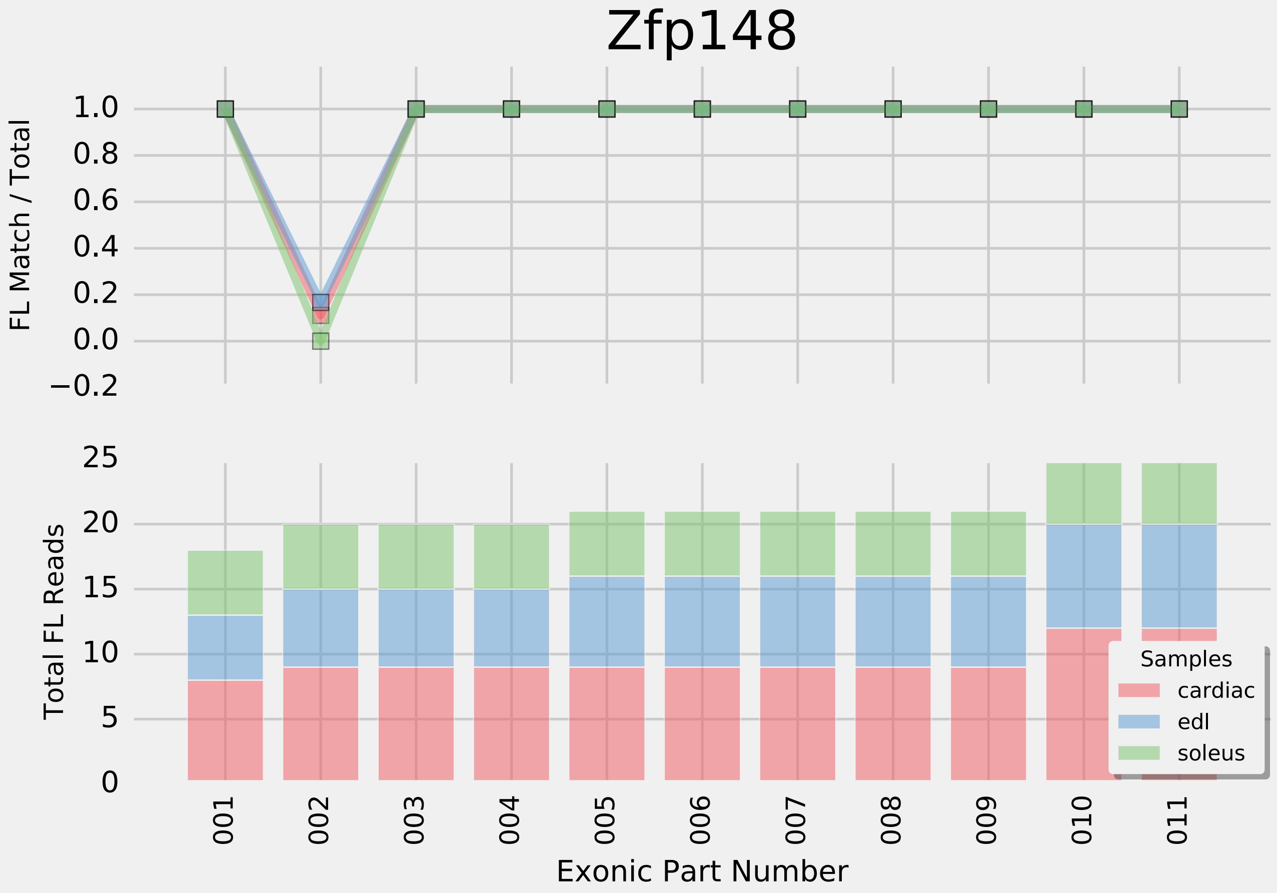

Zfp148
FL Match / Total
1.0
0.8
0.6
0.4
0.2
0.0
−0.2
Total FL Reads
25
20
15
10
5
0
Samples
cardiac
edl
soleus
001
002
003
004
005
006
007
008
009
010
011
Exonic Part Number

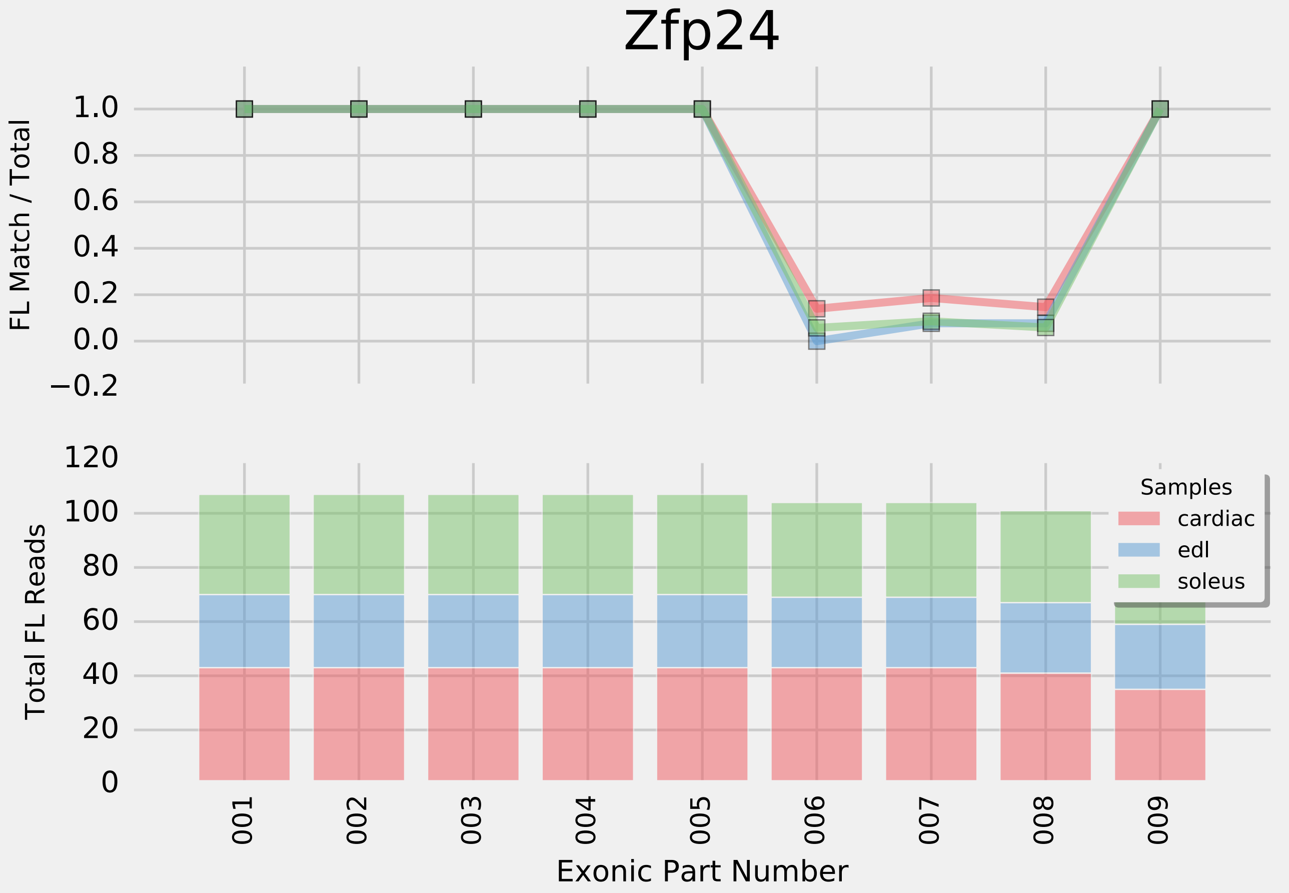

Zfp24
FL Match / Total
1.0
0.8
0.6
0.4
0.2
0.0
−0.2
Total FL Reads
120
100
80
60
40
20
0
Samples
cardiac
edl
soleus
001
002
003
004
005
006
007
008
009
Exonic Part Number

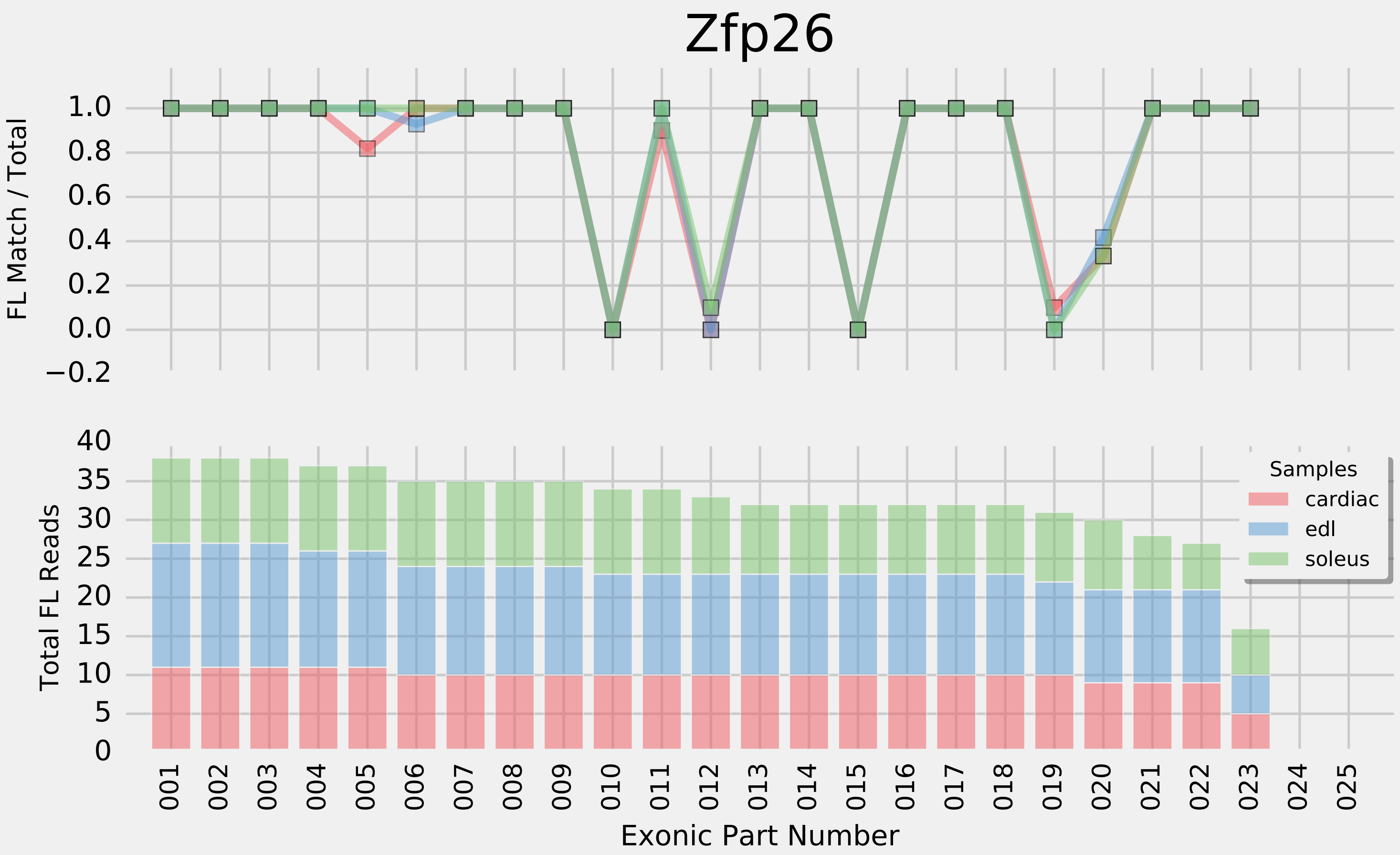

Zfp26
FL Match / Total
1.0
0.8
0.6
0.4
0.2
0.0
−0.2
Total FL Reads
40
35
30
25
20
15
10
5
0
Samples
cardiac
edl
soleus
001
002
003
004
005
006
007
008
009
010
011
012
013
014
015
016
017
018
019
020
021
022
023
024
025
Exonic Part Number

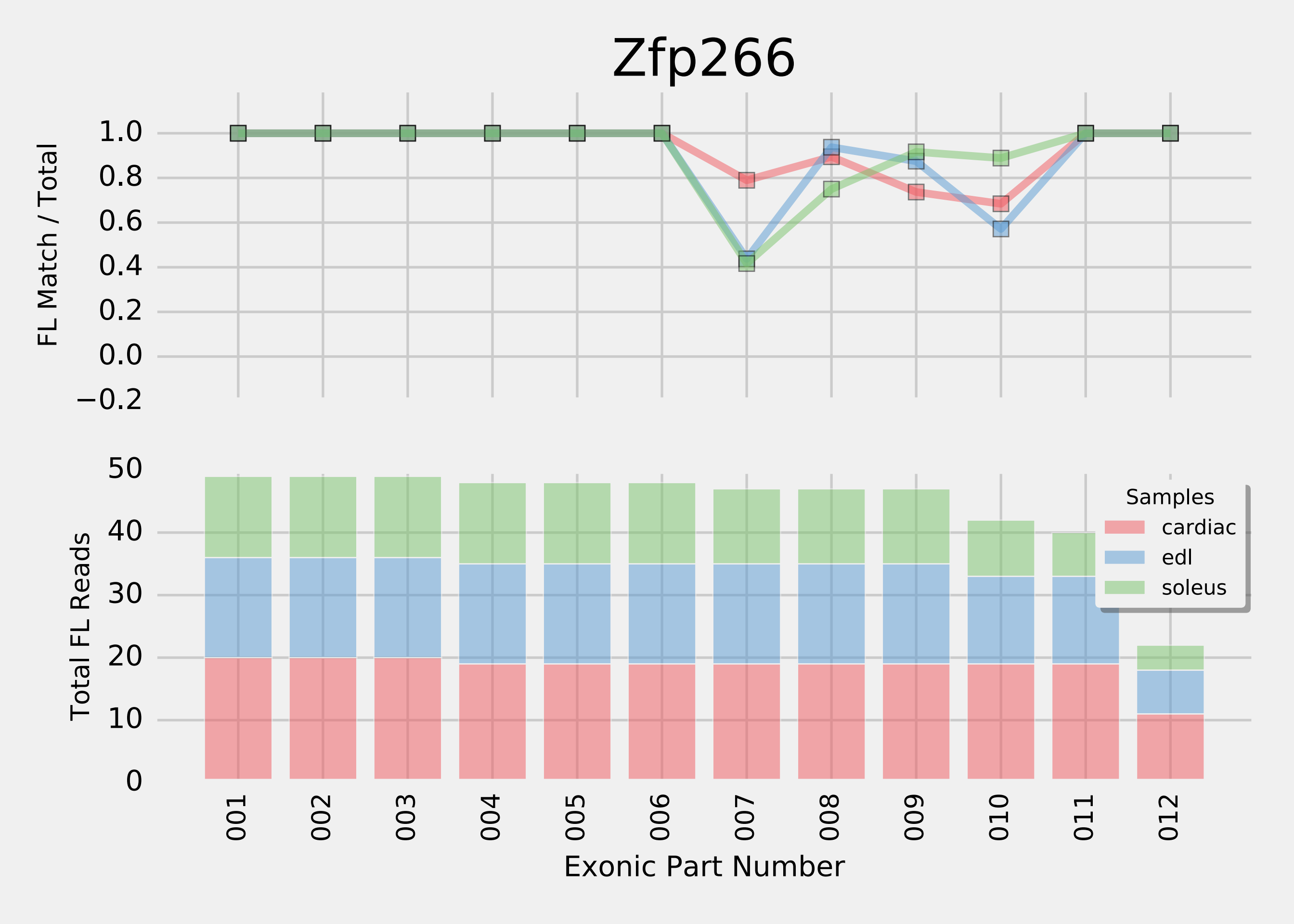

Zfp266
FL Match / Total
1.0
0.8
0.6
0.4
0.2
0.0
−0.2
Total FL Reads
50
40
30
20
10
0
Samples
cardiac
edl
soleus
001
002
003
004
005
006
007
008
009
010
011
012
Exonic Part Number

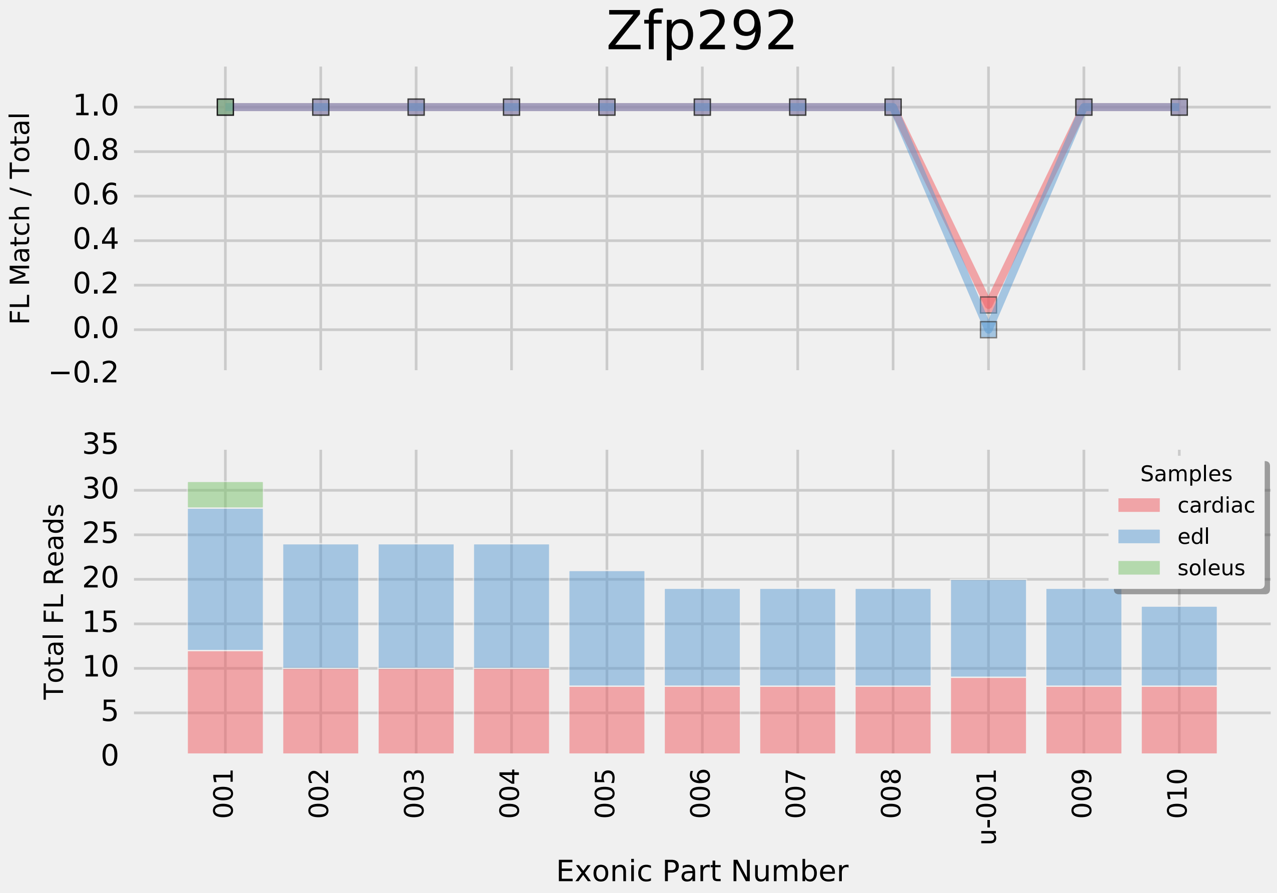

Zfp292
FL Match / Total
1.0
0.8
0.6
0.4
0.2
0.0
−0.2
Total FL Reads
35
30
25
20
15
10
5
0
Samples
cardiac
edl
soleus
001
002
003
004
005
006
007
008
u-001
009
010
Exonic Part Number

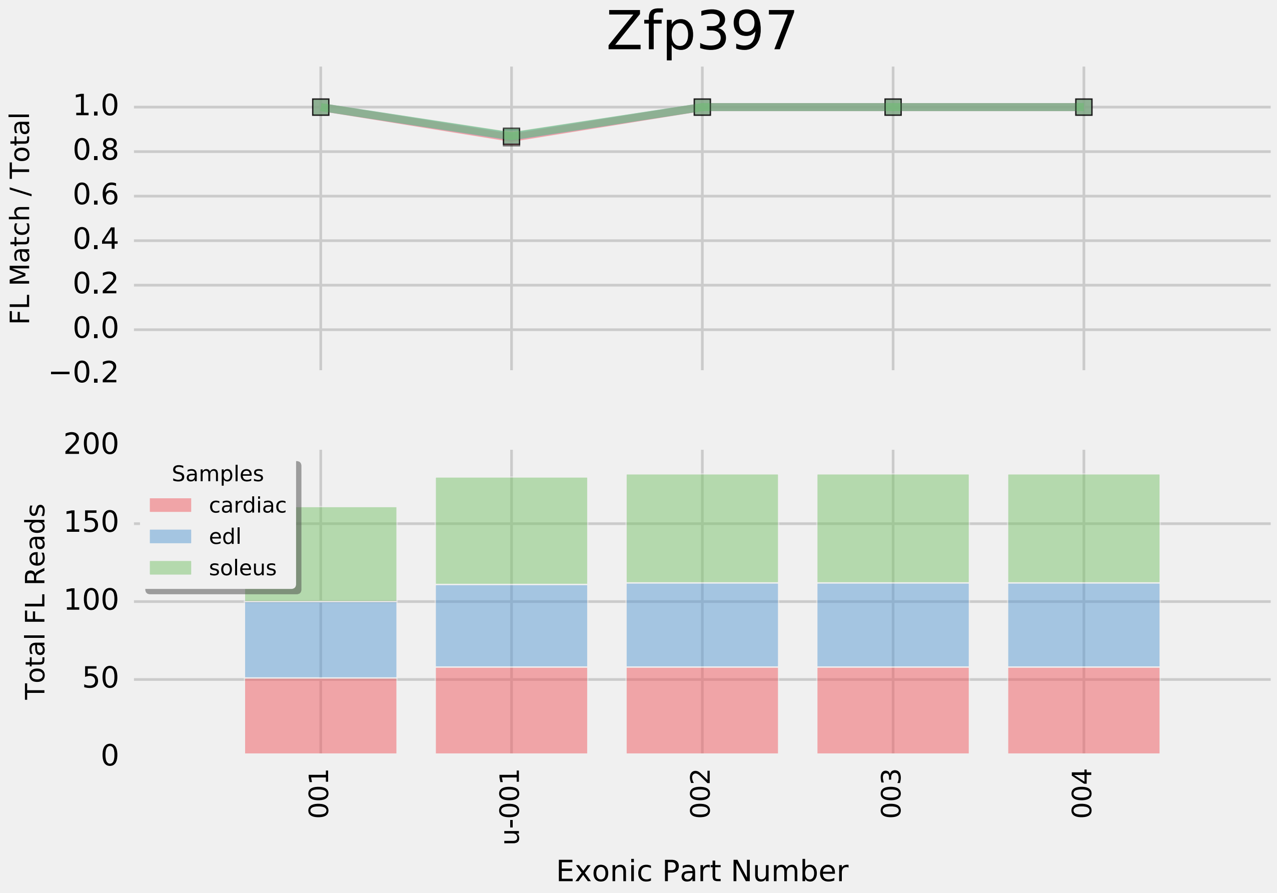
Zfp397
FL Match / Total
1.0
0.8
0.6
0.4
0.2
0.0
−0.2
Total FL Reads
200
150
100
50
0
Samples
cardiac
edl
soleus
001
u-001
002
003
004
Exonic Part Number

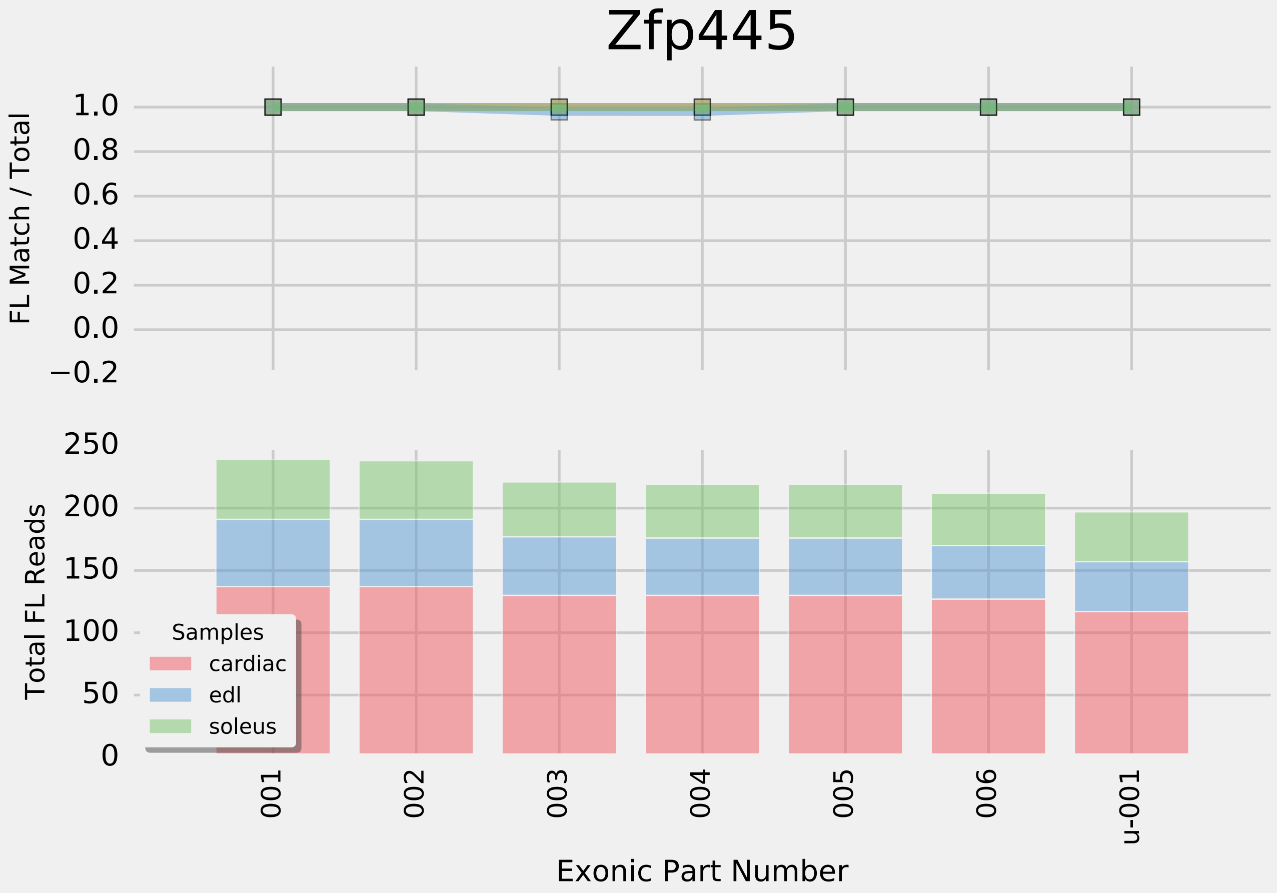
Zfp445
FL Match / Total
1.0
0.8
0.6
0.4
0.2
0.0
−0.2
Total FL Reads
250
200
150
100
50
0
Samples
cardiac
edl
soleus
001
002
003
004
005
006
u-001
Exonic Part Number

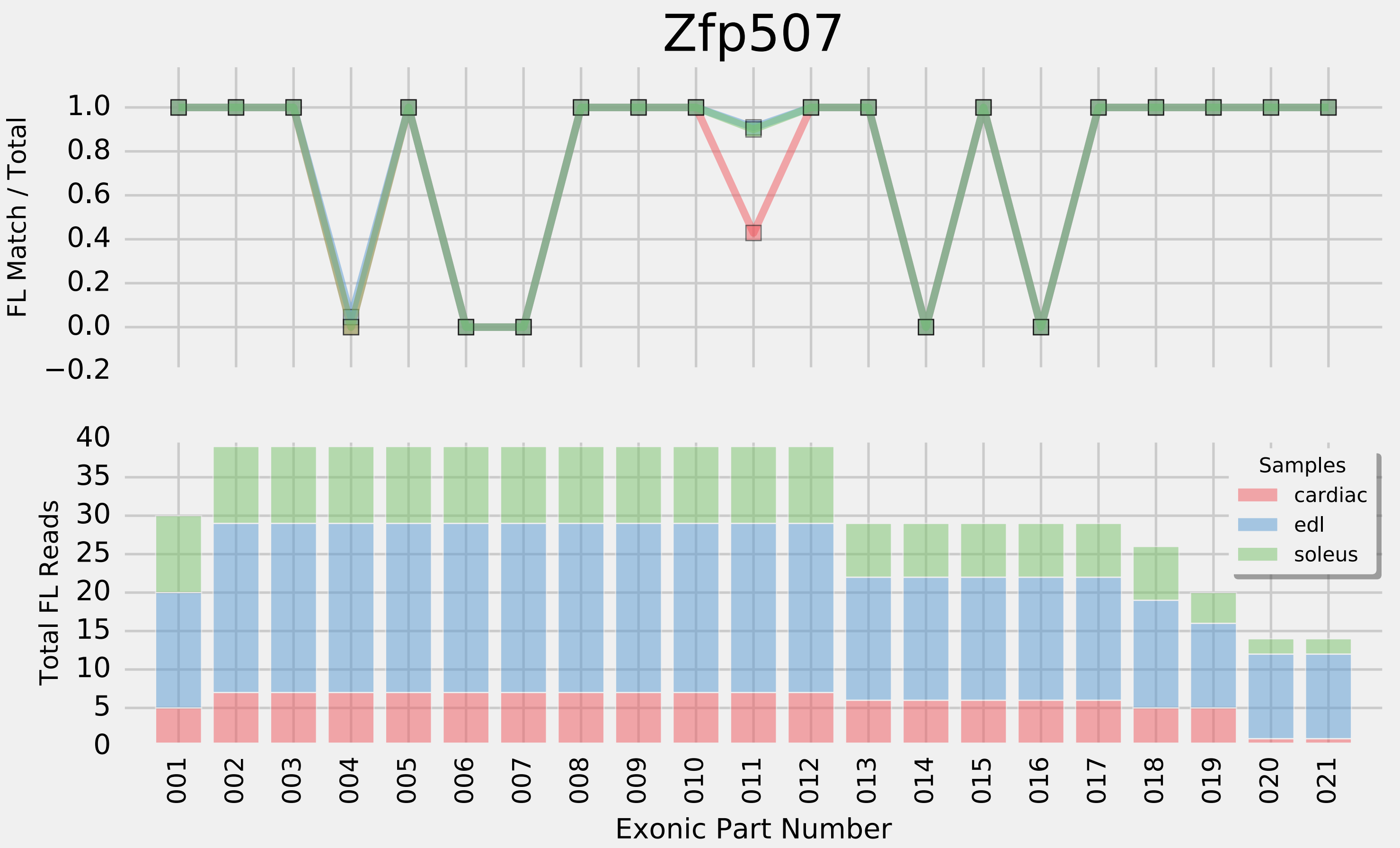

Zfp507
FL Match / Total
1.0
0.8
0.6
0.4
0.2
0.0
−0.2
Total FL Reads
40
35
30
25
20
15
10
5
0
Samples
cardiac
edl
soleus
001
002
003
004
005
006
007
008
009
010
011
012
013
014
015
016
017
018
019
020
021
Exonic Part Number

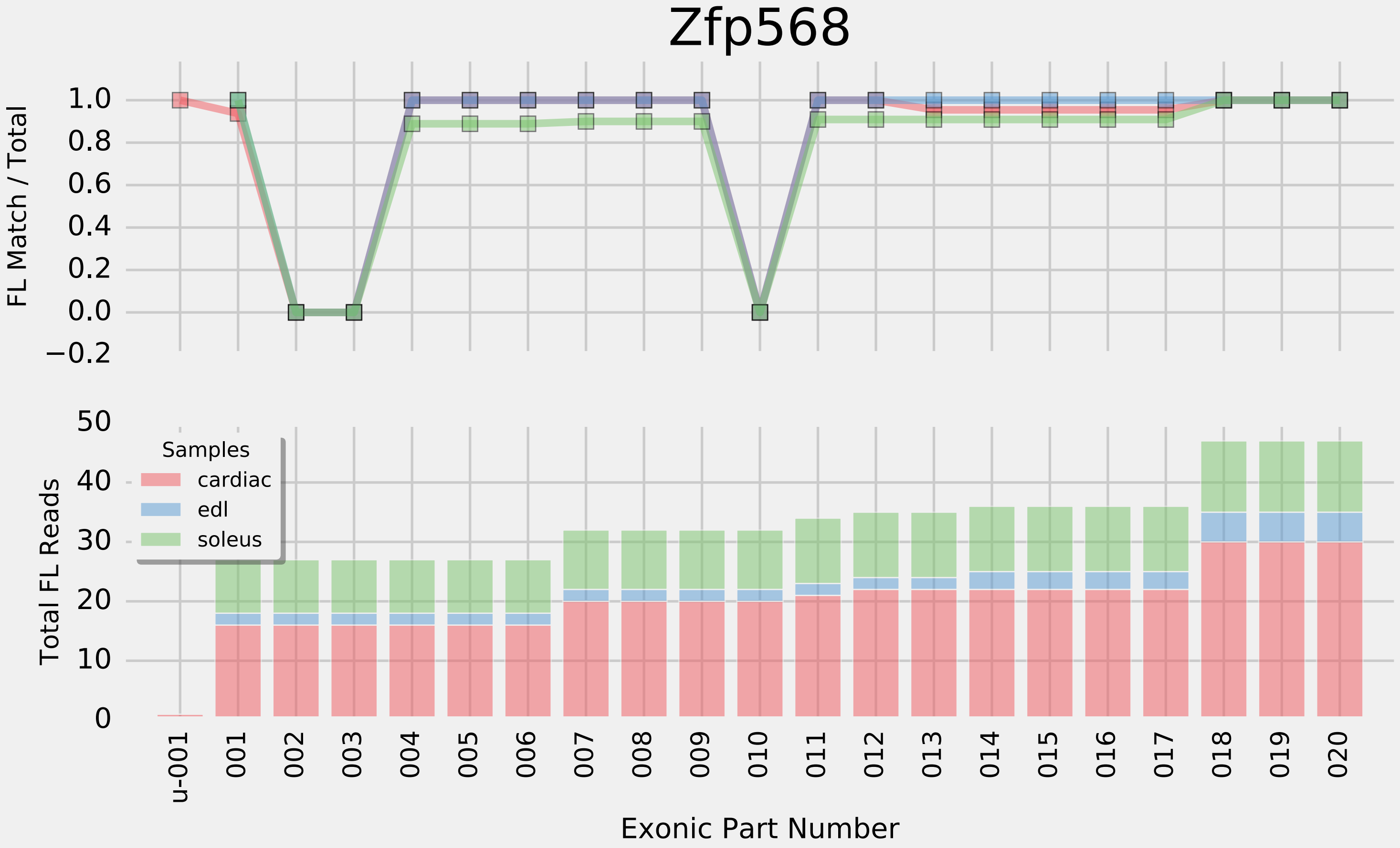

Zfp568
FL Match / Total
1.0
0.8
0.6
0.4
0.2
0.0
−0.2
Total FL Reads
50
40
30
20
10
0
Samples
cardiac
edl
soleus
u-001
001
002
003
004
005
006
007
008
009
010
011
012
013
014
015
016
017
018
019
020
Exonic Part Number

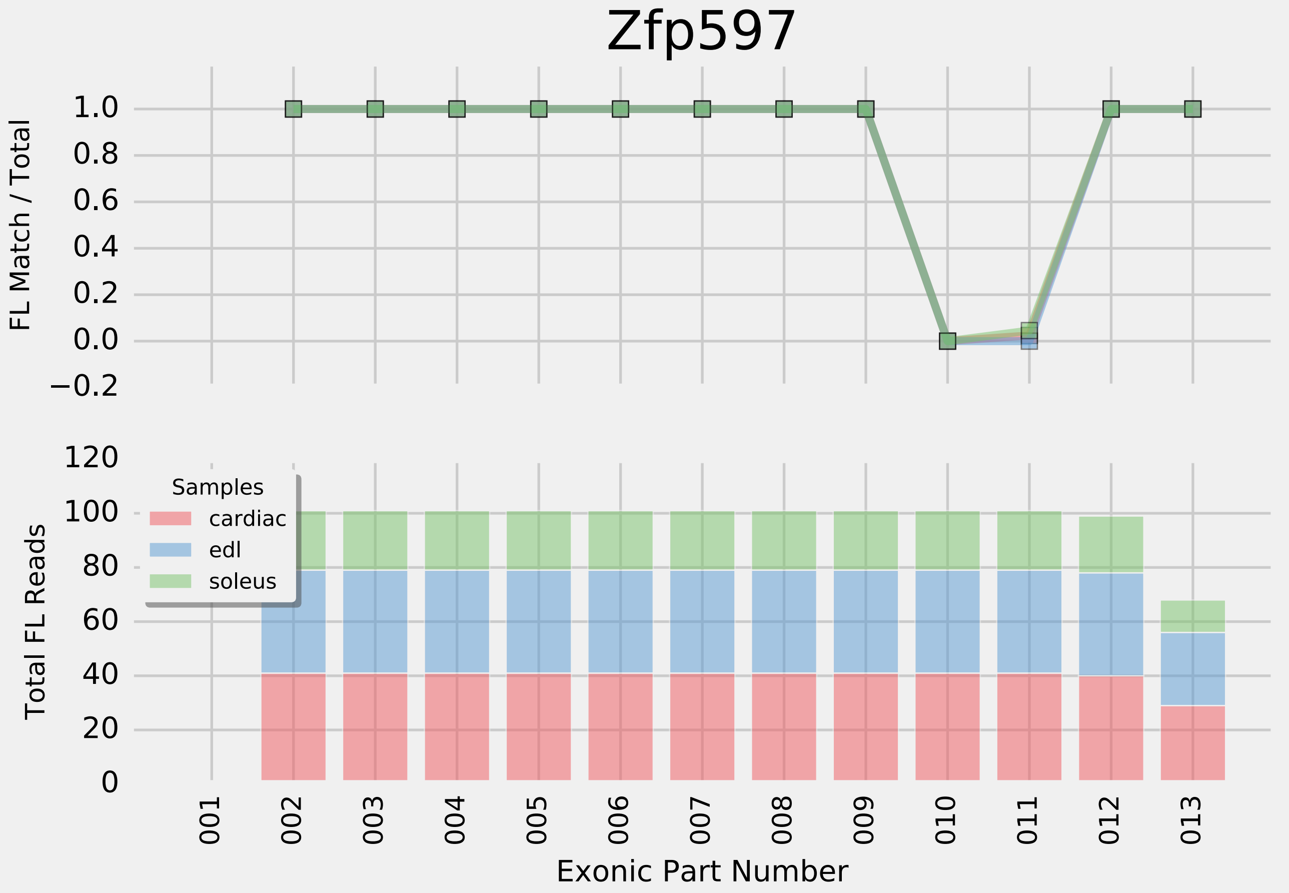
Zfp597
FL Match / Total
1.0
0.8
0.6
0.4
0.2
0.0
−0.2
Total FL Reads
120
100
80
60
40
20
0
Samples
cardiac
edl
soleus
001
002
003
004
005
006
007
008
009
010
011
012
013
Exonic Part Number

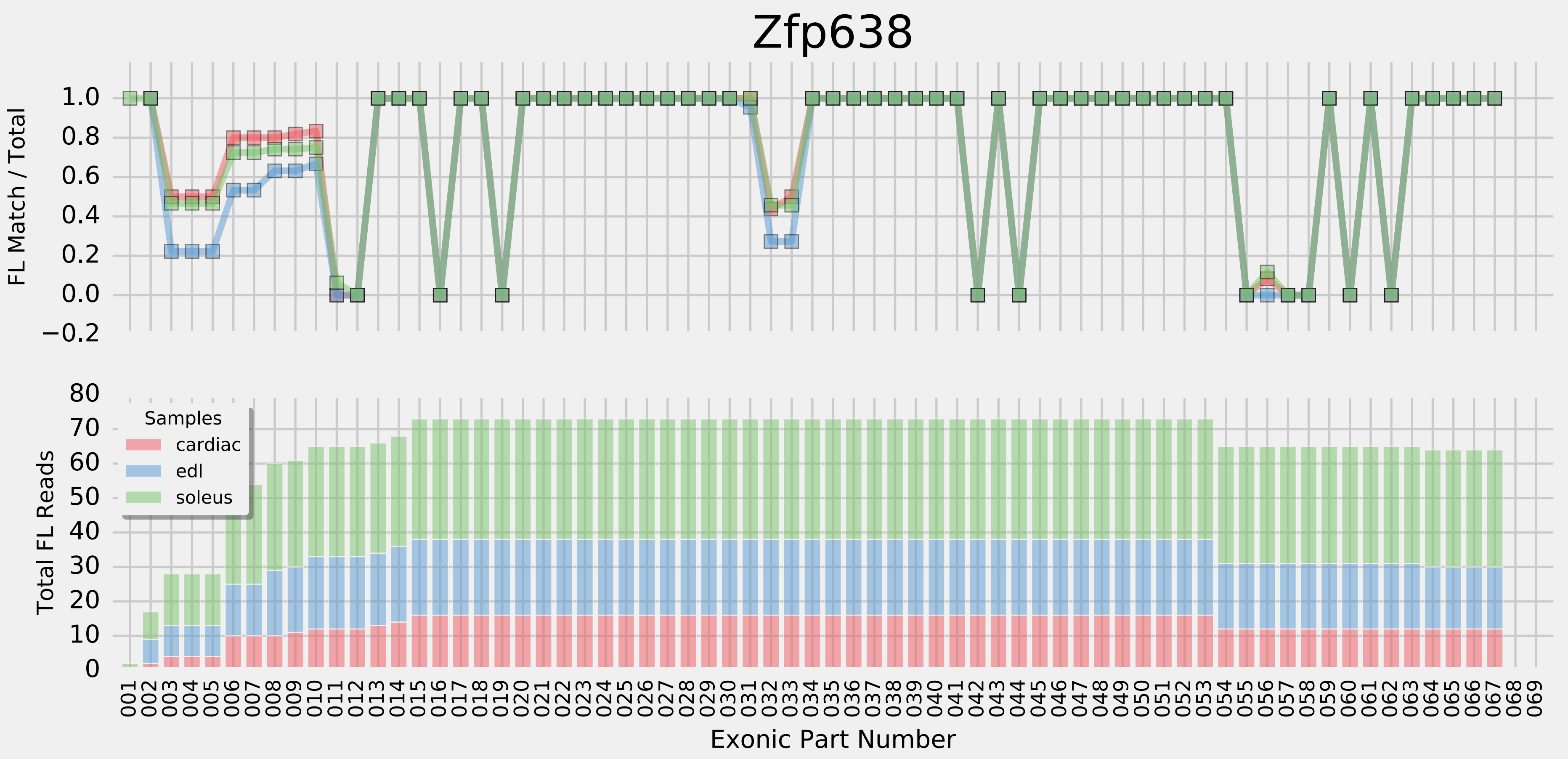

Zfp638
FL Match / Total
Total FL Reads
Samples
cardiac
edl
soleus
Exonic Part Number

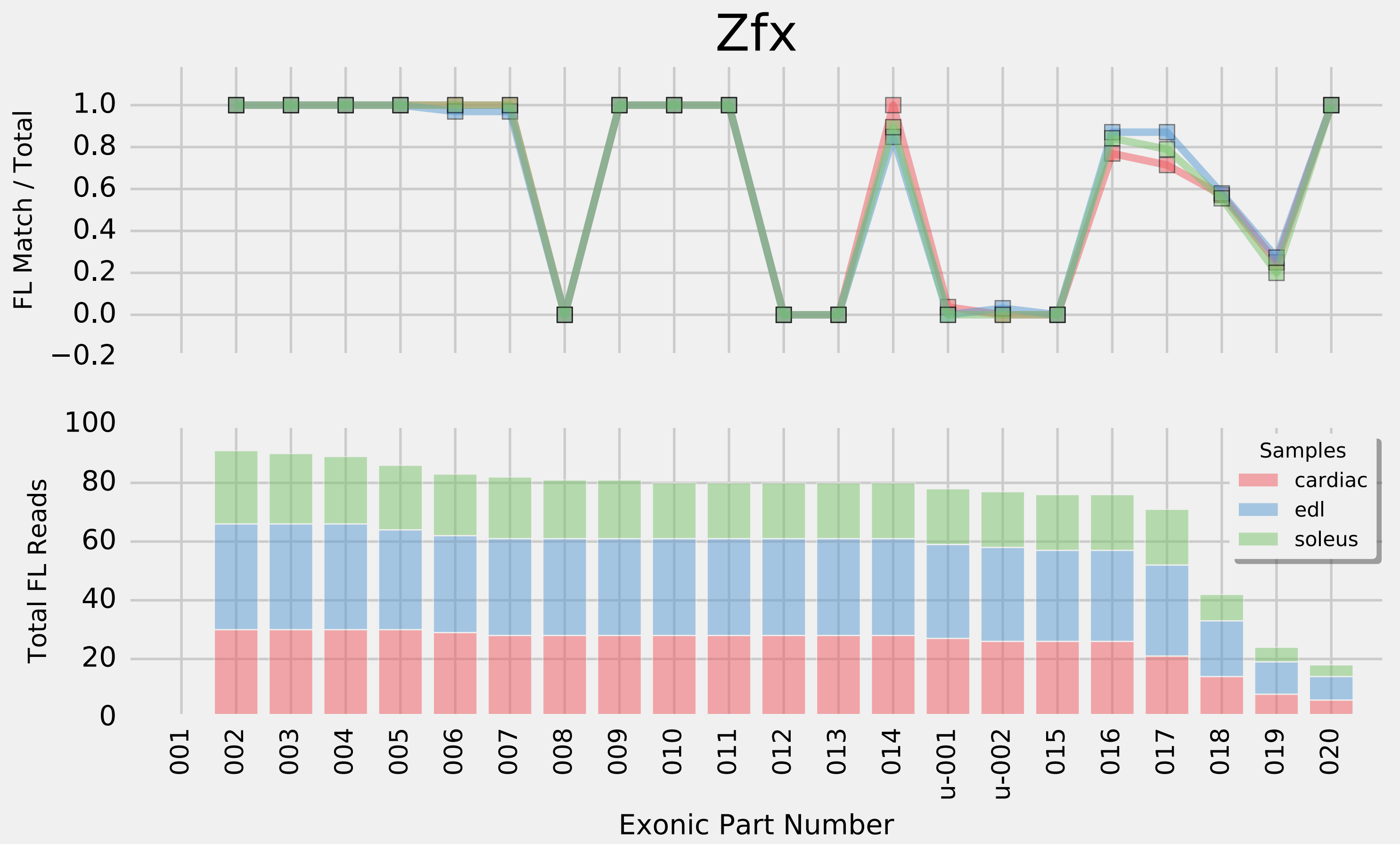
Zfx
FL Match / Total
1.0
0.8
0.6
0.4
0.2
0.0
−0.2
Total FL Reads
100
80
60
40
20
0
Samples
cardiac
edl
soleus
001
002
003
004
005
006
007
008
009
010
011
012
013
014
u-001
u-002
015
016
017
018
019
020
Exonic Part Number

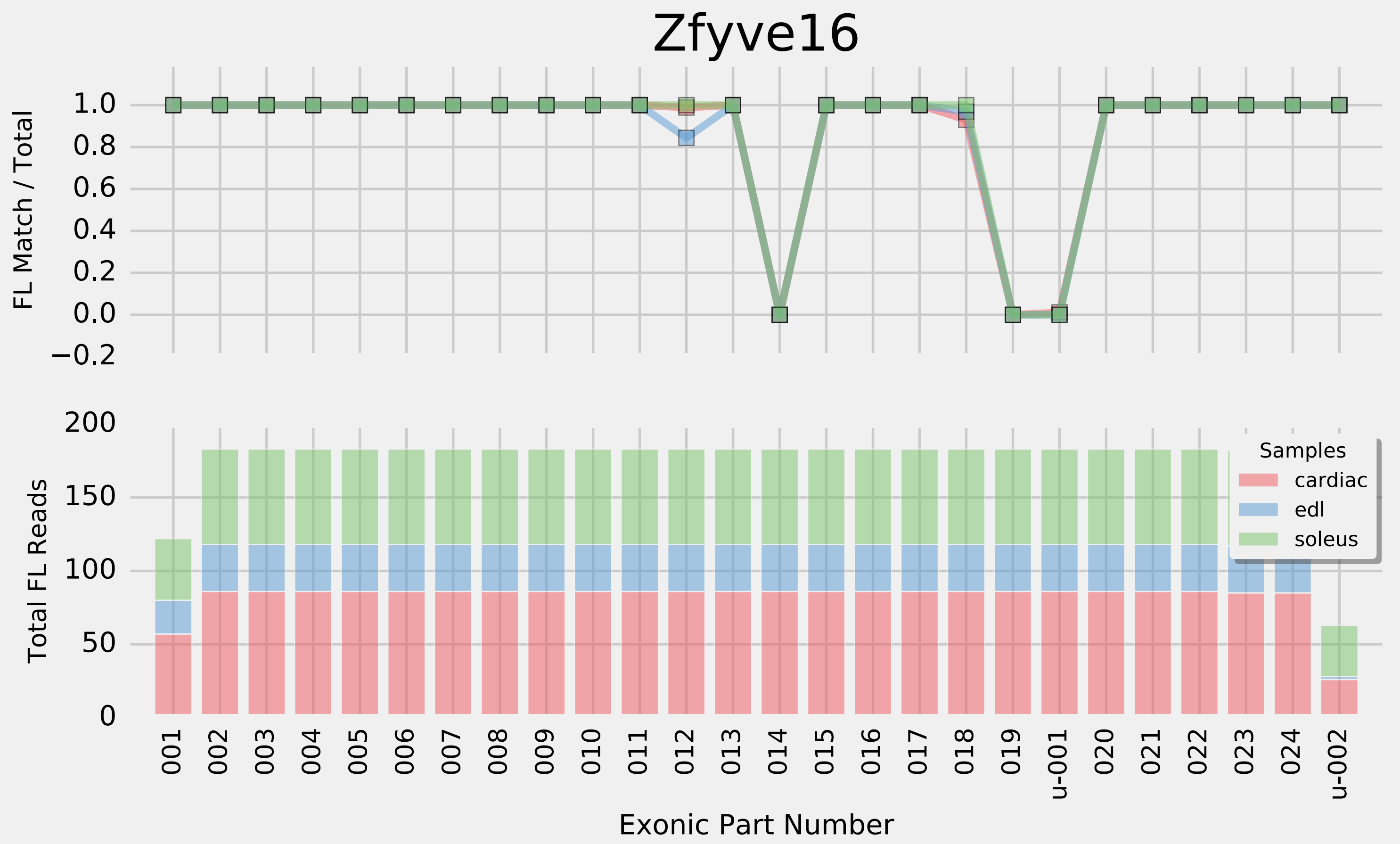

Zfyve16
FL Match / Total
1.0
0.8
0.6
0.4
0.2
0.0
−0.2
200
150
100
50
0
Total FL Reads
Samples
cardiac
edl
soleus
001
002
003
004
005
006
007
008
009
010
011
012
013
014
015
016
017
018
019
u-001
020
021
022
023
024
u-002
Exonic Part Number

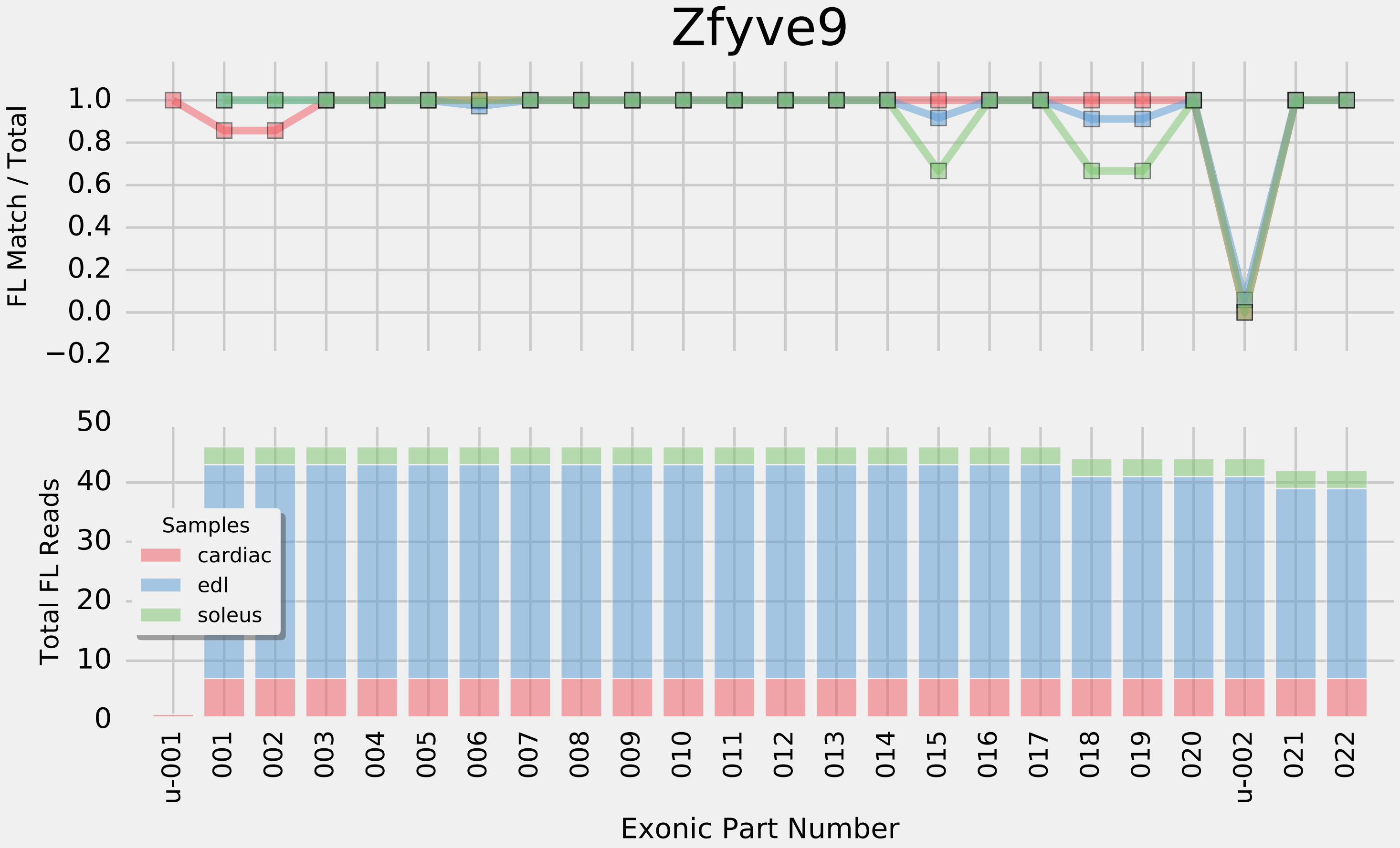

Zfyve9
FL Match / Total
1.0
0.8
0.6
0.4
0.2
0.0
−0.2
50
40
30
20
10
0
Total FL Reads
Samples
cardiac
edl
soleus
u-001
001
002
003
004
005
006
007
008
009
010
011
012
013
014
015
016
017
018
019
020
u-002
021
022
Exonic Part Number

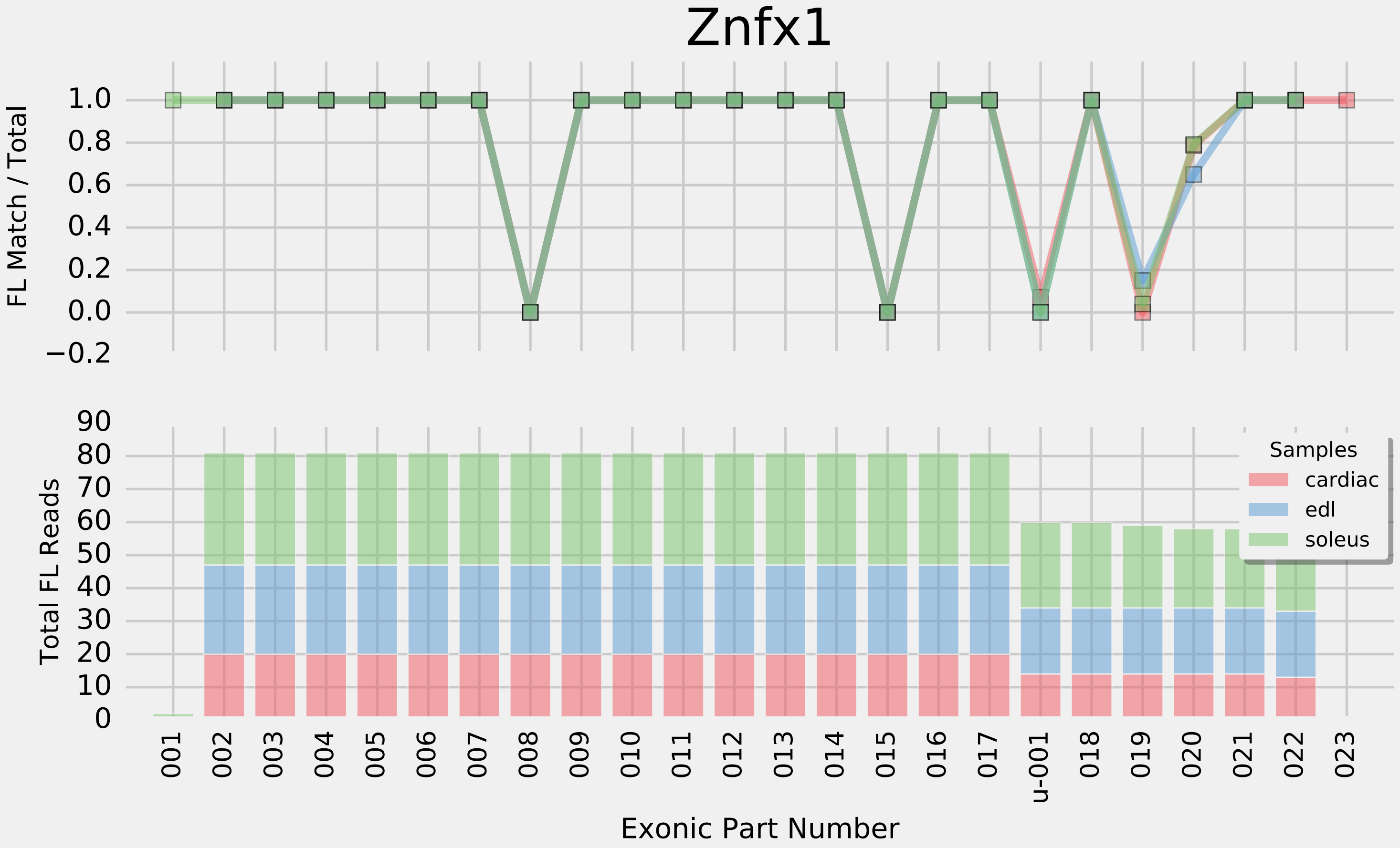

Znfx1
FL Match / Total
1.0
0.8
0.6
0.4
0.2
0.0
−0.2
Total FL Reads
90
80
70
60
50
40
30
20
10
0
Samples
cardiac
edl
soleus
001
002
003
004
005
006
007
008
009
010
011
012
013
014
015
016
017
u-001
018
019
020
021
022
023
Exonic Part Number

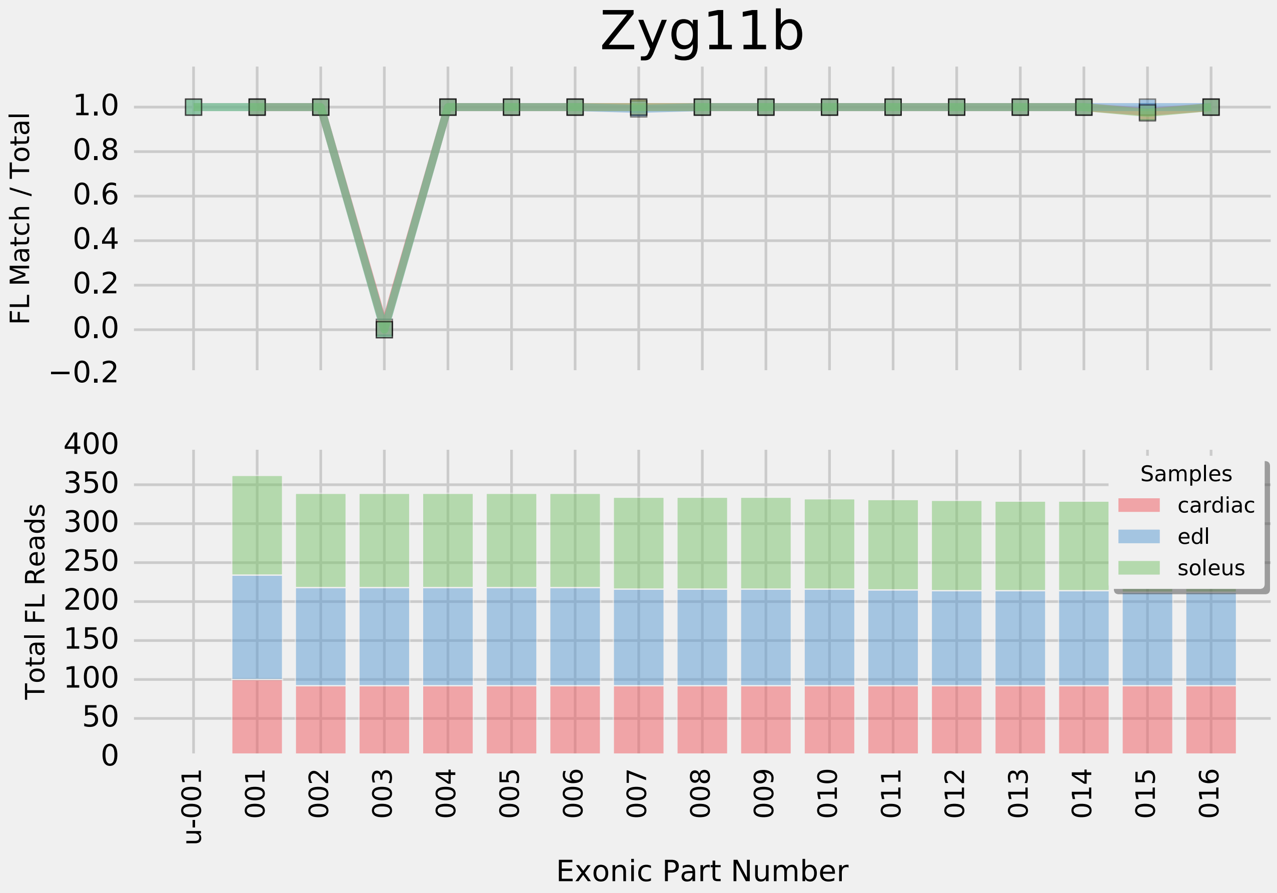
Zyg11b
FL Match / Total
1.0
0.8
0.6
0.4
0.2
0.0
−0.2
Total FL Reads
400
350
300
250
200
150
100
50
0
Samples
cardiac
edl
soleus
u-001
001
002
003
004
005
006
007
008
009
010
011
012
013
014
015
016
Exonic Part Number

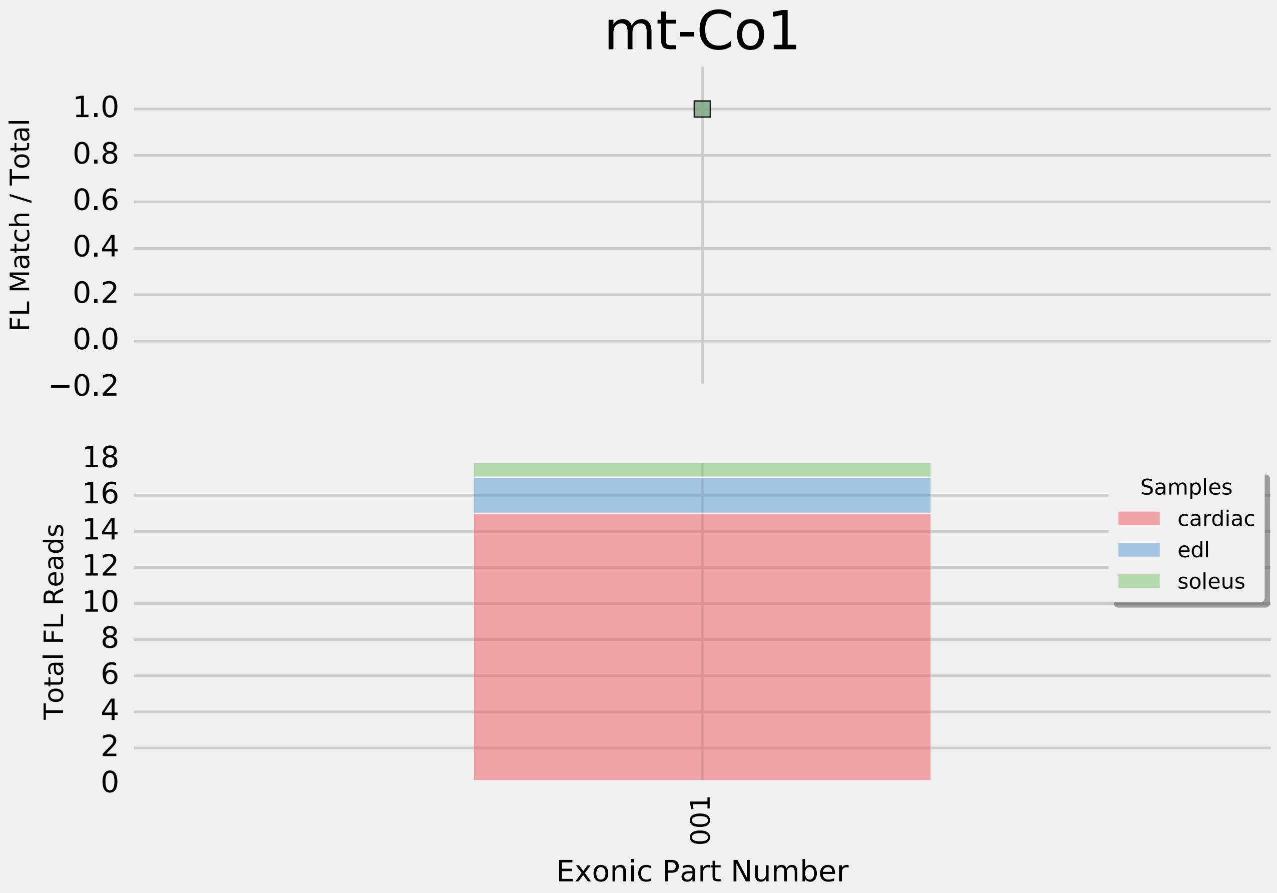
mt-Co1
FL Match / Total
1.0
0.8
0.6
0.4
0.2
0.0
−0.2
Total FL Reads
18
16
14
12
10
8
6
4
2
0
Samples
cardiac
edl
soleus
001
Exonic Part Number
