# Supplementary material for: A long-read RNA-seq approach to identify novel transcripts of very large genes
Source: Genome Res. 2020 Jun;30(6):885–97. doi: 10.1101/gr.259903.119 (PMC7370890; doi:10.1101/gr.259903.119)
Supplement: Supplemental Material [file supp_gr.259903.119_Supplemental_Code.zip › isoseq_manuscript_resources-master/s3_exPhaser_analysis_pipeline/exPhaser_implementation.pdf]

ExPhaser

Program for Determining phasing of isoform defining exons

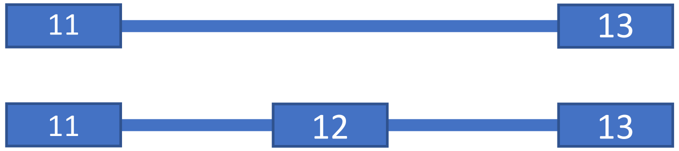

Brian Uapinyoying

Hoffman lab

8/6/2019

Introduction

- PacBio's long-read isoform sequencing data spans multiple exons (10kb+)
- This allows for phasing of exons when analyzing RNA-Seq data
- But some transcripts are too long > 10 kb (e.g. Ttn at 106 kb)
- We successfully used an exon-based approach (ExCOVator) and internal priming to determine differential usage between tissues in ultra long transcripts
- However, without phasing data we cannot determine the splicing pattern of these differentially used exons within individual transcripts from each tissue

Why is phasing of exons important?

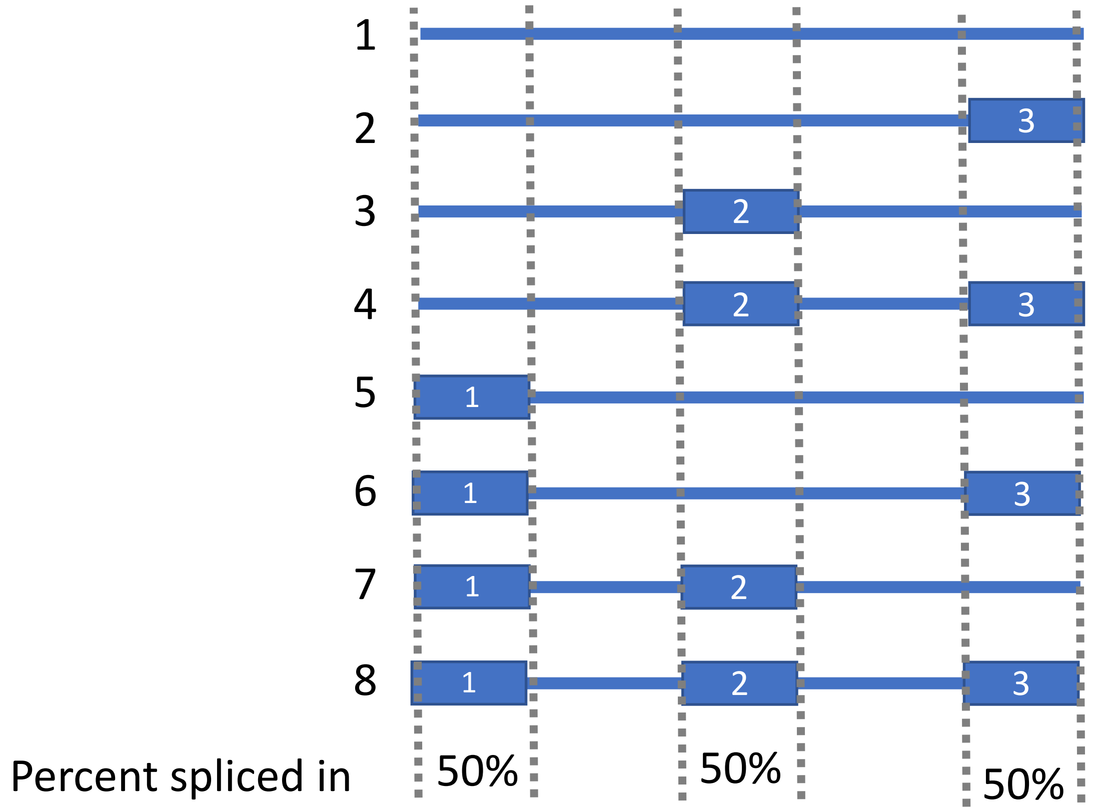

- Without phasing data, all 3 exons (independently) look like they are similarly expressed when they could be part of different transcript isoforms

General Implementation

- Use the python library HT-Seq
  - https://www.ncbi.nlm.nih.gov/pubmed/25260700
- Select key exons / isoform defining cassette exons
  - input into script as bed file (genomic coordinates)
- Loop through each IsoSeq read from sample bam files
  - Determine if the read is long enough to cover all key input exons
    - Filter out reads that are too short
  - Check if exons in the read match any of the key input exons & note splice pattern
  - Tally up all the reads for each splice pattern and output as data table/file

What are all the possible splicing patterns with a given number of exons?

- Formula = $2^{(\#exons)}$
- For 3 exons, 2^3 = 8 total splicing possibilities or 8 rows of information
- Each row (i) can be seen as binary representation of [row number – 1]

Number of rows = $2^{(\#exons)}$

(row# – 1)

Row #	Binary
0	000
1	001
2	010
3	011
4	100
5	101
6	110
7	111

Split into Binary table

Exon 1	Exon 2	Exon 3
0	0	0
0	0	1
0	1	0
0	1	1
1	0	0
1	0	1
1	1	0
1	1	1

Convert to Boolean values
(Exon Spliced in? True/False)

Pattern	Exon 1	Exon 2	Exon 3
1	False	False	False
2	False	False	True
3	False	True	False
4	False	True	True
5	True	False	False
6	True	False	True
7	True	True	False
8	True	True	True

Not all possible patterns will exist due to constitutively expressed exons

Hypothetical Example: NRAP Soleus

Likely only a subset of the total possibilities will be seen in the data, the rest may be artifacts

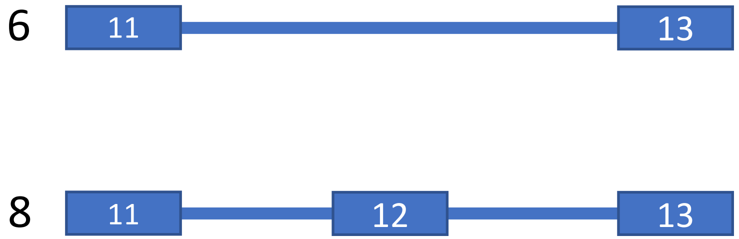

Pattern	Exon 11	Exon 12	Exon 13	FL_count	%
1	False	False	False	2	0
2	False	False	True	5	0
3	False	True	False	0	0
4	False	True	True	1	0
5	True	False	False	0	0
6	True	False	True	1500	30
7	True	True	False	0	
8	True	True	True	3500	70

Technically, we also do not have to limit ourselves to adjacent (neighboring) exons as long as the reads are long enough to contain all exons

- If we know which exons are variably spliced (output from my exCOVator script), we can strategically select these key exons for phase analysis
- Then determine presence/absence of input exons relative to each other within each read
- This same approach can be done for transcript annotations!
- However, the longer the distance (interval range) between the first and last key exon, the fewer reads will contain all key exons

Pattern	Exon 9	Exon 17	Exon 47
1	False	False	False
2	False	False	True
3	False	True	False
4	False	True	True
5	True	False	False
6	True	False	True
7	True	True	False
8	True	True	True

Only reads and transcript annotations that **contain** the bed/exon interval range are selected for determining and quantifying splicing patterns

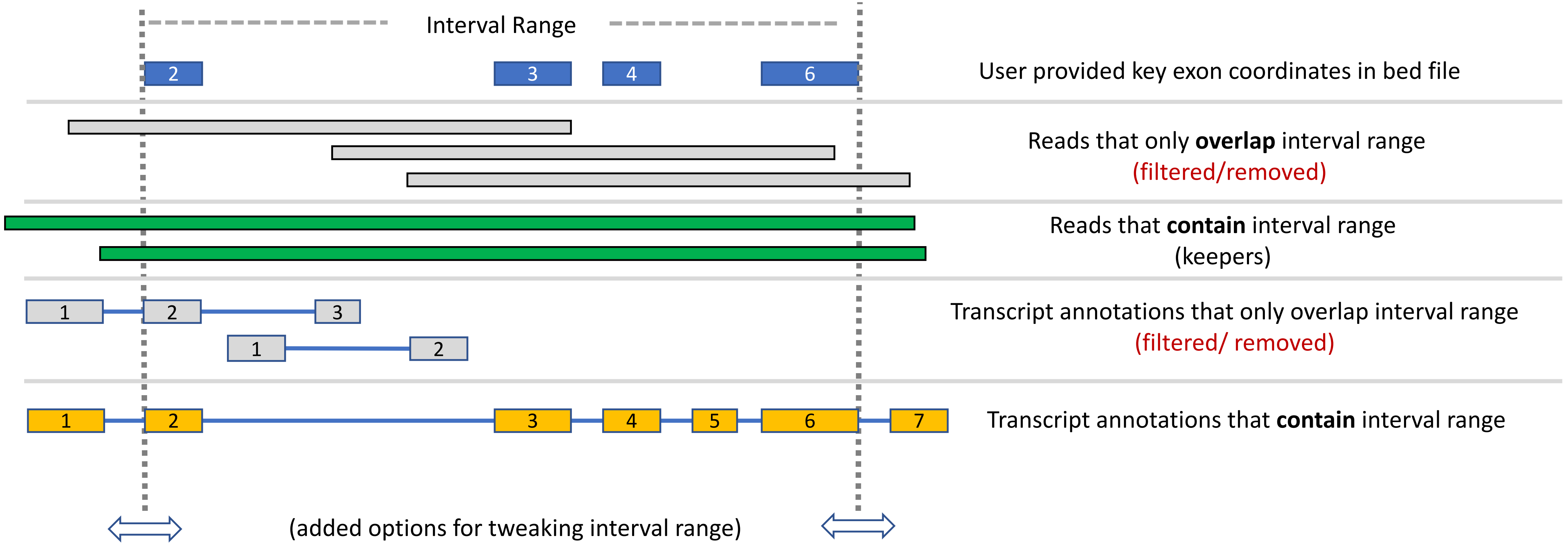

- Interval range is calculated using the start site of the lowest coordinate exon and the end site of the highest coordinate exon
- Therefore, the farther the first and last exons are the fewer reads will contain the interval range
- Reads that overlap vs contain the interval range is output to a data file for the user

We can also use this same approach to determine which pattern is associated with which annotated transcripts

Isoform	Exon 9	Exon 17	Exon 47	Annotation
1	False	False	False	?
2	False	False	True	NM_XX3
3	False	True	False	?
4	False	True	True	?
5	True	False	False	?
6	True	False	True	NM_XX2
7	True	True	False	?
8	True	True	True	NM_XX1, NM_XX4

Most patterns won't be annotated or exist

Some patterns may match multiple transcript annotations

Finally, we can combine all three pieces of data (pattern, annotation, read count) and filter out patterns with no reads in any sample and with no annotation found

All patterns

Isoform	Exon 9	Exon 17	Exon 47	Annotation	Cardiac Read count	EDL Read count	Soleus Read count
1	False	False	False	?	0	0	0
2	False	False	True	NM_XX3	100	700	300
3	False	True	False	?	2	0	0
4	False	True	True	?	0	250	0
5	True	False	False	?	0	0	0
6	True	False	True	NM_XX2	30	0	900
7	True	True	False	?	0	0	0
8	True	True	True	NM_XX1, NM_XX4	500	0	40

Selected patterns

Isoform	Exon 9	Exon 17	Exon 47	Annotation	Cardiac Read count	EDL Read count	Soleus Read count	
2	False	False	True	NM_XX3	100	700	300	
3	False	True	False	?	2	0	0	Artifact or very rare transcript?
4	False	True	True	?	0	250	0	Potential novel isoforms
6	True	False	True	NM_XX2	30	0	900	
8	True	True	True	NM_XX1, NM_XX4	500	0	40	Not enough data to distinguish the two isoforms

Pilot Analysis: Nrap transcript expression between Cardiac, EDL and Soleus using Gencode mm10 annotations

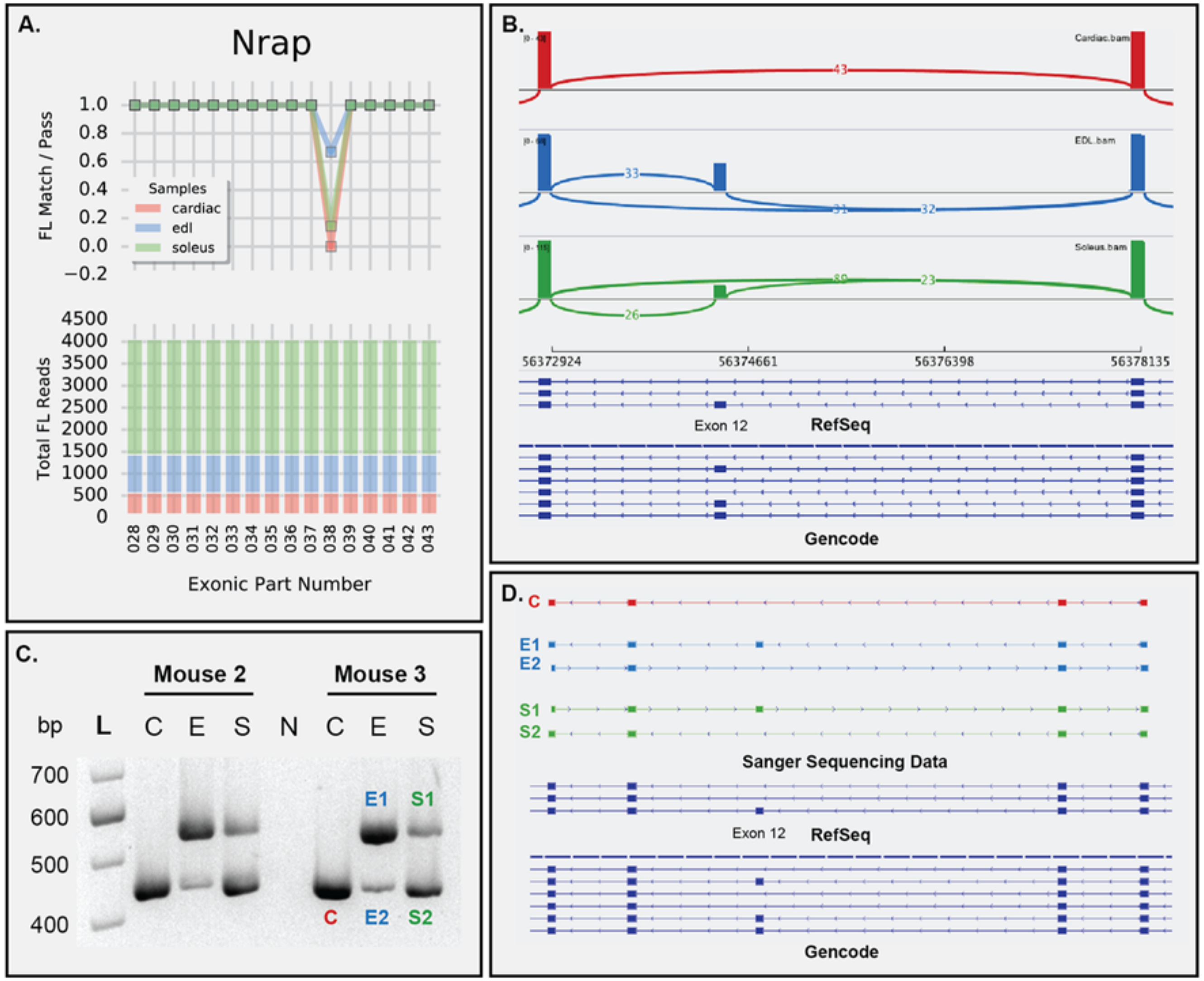

- Exon 12 was differentially spliced between tissues
- Are there other exons we didn't initially detect?

Difficult to tell based on eyeballing reads (IGV)

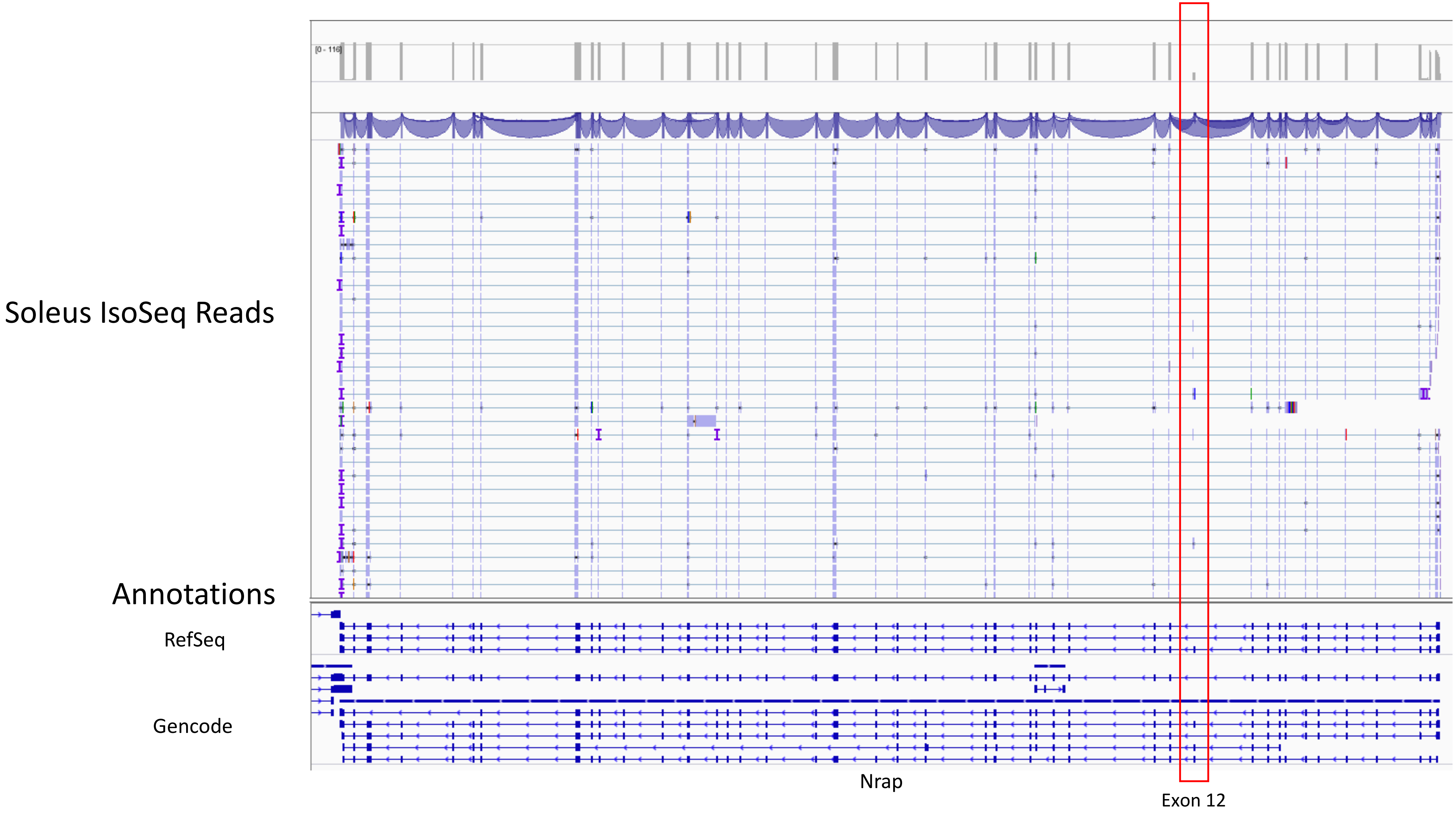

Example Analysis: Nrap transcript expression between Cardiac, EDL and Soleus using Gencode mm10 annotations

- Exons 2, 12, 37, 38, 39 and 40 are cassette/isoform defining exons (6 total)
  - Omitting ENSMUST00000169099.7 which is short and has been labed as ‘non-sense mediated decay’

- Added a 6 constitutively expressed adjacent/neighboring exons as anchors for analysis, exons 41, 36, 13, 11, 3, 1
  - Not required for phasing to work, but adds some context and helps validate findings

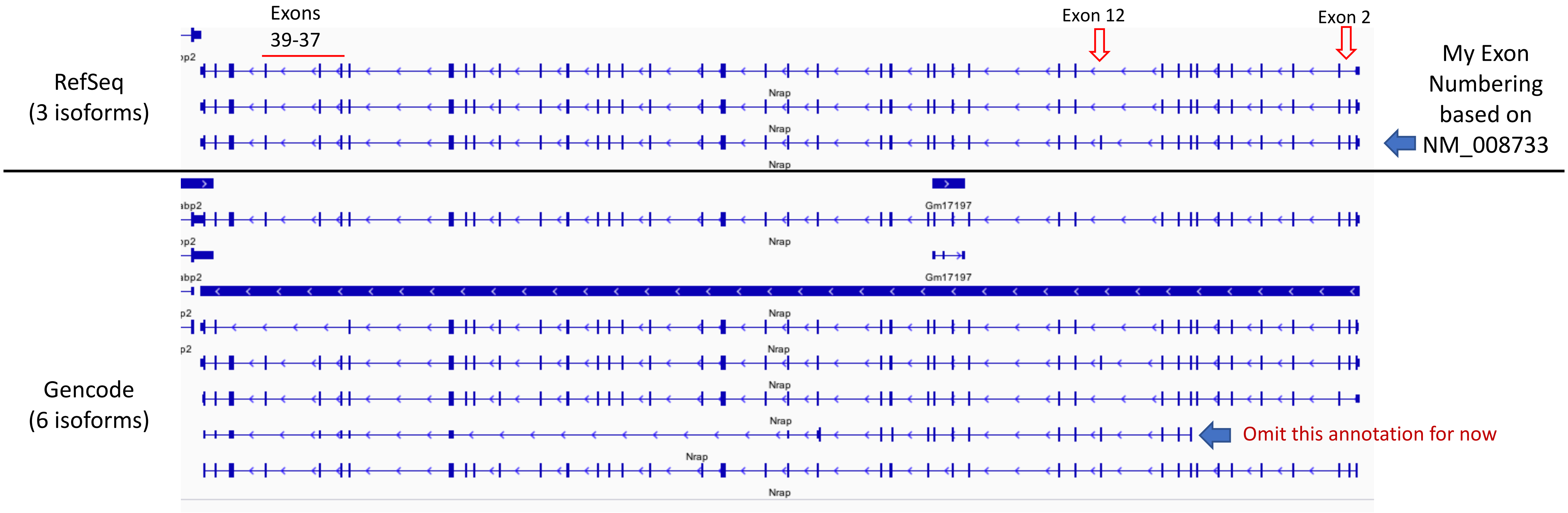

Input bed file (genomic coordinate file separated by tabs) for Nrap

Chrom	Start	End	Name	Score	Strand
chr19	56320884	56321016	exon41	0	-
chr19	56321722	56322034	exon40	0	-
chr19	56323887	56323995	exon39	0	-
chr19	56327194	56327299	exon38	0	-
chr19	56328500	56328599	exon37	0	-
chr19	56328995	56329100	exon36	0	-
chr19	56372806	56372914	exon13	0	-
chr19	56374363	56374468	exon12	0	-
chr19	56378054	56378171	exon11	0	-
chr19	56388748	56388836	exon3	0	-
chr19	56389389	56389484	exon2	0	-
chr19	56389805	56390038	exon1	0	-

Red exon names are isoform defining exons

- Original coordinates were extracted from UCSC Table Browser (RefSeq mm10 for NRAP)
- Modified name column to make it easier for me to remember the exons
- Input to script also requires sample bam files and gencode annotation (gtf) file selected for the target gene
  - cardiac.bam, edl.bam, soleus.bam, nrap.gtf, nrap_exons.bed

Basic statistics on reads that contained interval range (keepers used in phasing analysis) vs total reads overlapping interval range (all reads)

	Reads that Contain Interval range		Reads that overlap interval range		Percent contained / overlap	
**Sample**	**Cluster count**	**Full-length count**	**Cluster count**	**Full-length count**	**Cluster count**	**Full-length count**
cardiac	12	**399**	43	**567**	27.9%	**70.4%**
edl	12	**483**	68	**863**	17.6%	**56.0%**
soleus	32	**1771**	116	**2622**	27.6%	**67.5%**

- **Cluster count** = Pacbio's cluster/consensus read that is output from its ICE algorithm. Basically grouping multiple full-length reads by similarity and using it to increase sequence quality by forming in a single unique polished read

- **Full-length count** = number of full-length reads extracted from information in the cluster read (more accurate)

Results for Nrap show that we are able to determine transcript level information from phasing of key exons in the annotation and read data

Exon 41	Exon 40c	Exon 39c	Exon 38c	Exon 37c	Exon 36	Exon 13	Exon 12c	Exon 11	Exon 3	Exon 2c	Exon 1	transcript_ids	Cardiac flCount	Cardiac pct	Edl flCount	Edl pct	Soleus flCount	Soleus pct
TRUE	FALSE	FALSE	FALSE	FALSE	TRUE	TRUE	FALSE	TRUE	TRUE	TRUE	TRUE	ENSMUST00000167239.7	0	0%	0	0%	0	0%
TRUE	TRUE	TRUE	TRUE	TRUE	FALSE	TRUE	FALSE	TRUE	TRUE	TRUE	TRUE	?	0	0%	0	0%	4	0%
TRUE	TRUE	TRUE	TRUE	TRUE	TRUE	TRUE	FALSE	TRUE	TRUE	FALSE	TRUE	ENSMUST00000095947.10	14	**4%**	0	0%	1	0%
TRUE	TRUE	TRUE	TRUE	TRUE	TRUE	TRUE	FALSE	TRUE	TRUE	TRUE	TRUE	ENSMUST00000040711.14	385	**96%**	153	**32%**	1553	**88%**
TRUE	TRUE	TRUE	TRUE	TRUE	TRUE	TRUE	TRUE	TRUE	TRUE	FALSE	TRUE	?	0	0%	0	0%	1	0%
TRUE	TRUE	TRUE	TRUE	TRUE	TRUE	TRUE	TRUE	TRUE	TRUE	TRUE	TRUE	ENSMUST00000073536.12, ENSMUST00000166203.1	0	0%	330	**68%**	212	**12%**
												**total**	**399**		**483**		**1771**	

- ENSMUST00000073536.12, and ENSMUST00000166203.1 are almost identical except for lengths of 3’ and 5’ UTR
  - Indistinguishable with current Interval range
- Some reads in cardiac for ENSMUST00000095947.10 wasn’t seen before
  - Has low coverage (14 reads). Could be rare transcript.
- Transcript level analysis shows similar ratios of differential expression as exon 12 RT-PCR
- The two unannotated matches (?) were checked in IGV to be from artifact reads

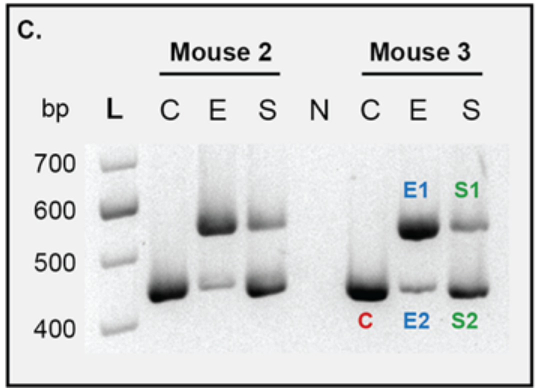

RT-PCR

With Exon 12

Without Exon 12
